# Genome survey and high-resolution genetic map provide valuable genetic resources for *Fenneropenaeus chinensis*

**DOI:** 10.1038/s41598-021-87237-4

**Published:** 2021-04-06

**Authors:** Xianhong Meng, Qiang Fu, Sheng Luan, Kun Luo, Juan Sui, Jie Kong

**Affiliations:** 1 Key Laboratory for Sustainable Utilization of Marine Fisheries Resources of Ministry of Agriculture and Rural Affairs, Yellow Sea Fisheries Research Institute, Chinese Academy of Fishery Sciences, Qingdao, 266071 China; 2 Laboratory for Marine Fisheries Science and Food Production Processes, Qingdao National Laboratory for Marine Science and Technology , Qingdao, 266071 China

**Keywords:** Next-generation sequencing, Genetic linkage study

## Abstract

*Fenneropenaeus chinensis* is one of the most important aquaculture species in China. Research on its genomic and genetic structure not only helps us comprehend the genetic basis of complex economic traits, but also offers theoretical guidance in selective breeding. In the present study, a genome survey sequencing was performed to generate a rough reference genome utilized for groping preliminary genome characteristics and facilitate linkage and quantitative trait locus (QTL) mapping. Linkage mapping was conducted using a reduced-representation sequencing method 2b-RAD. In total, 36,762 SNPs were genotyped from 273 progenies in a mapping family, and a high-resolution linkage map was constructed. The consensus map contained 12,884 markers and spanned 5257.81 cM with an average marker interval of 0.41 cM, which was the first high-resolution genetic map in *F. chinensis* to our knowledge. QTL mapping and association analysis were carried out in 29 characters including body size, sex and disease resistance. 87 significant QTLs were detected in several traits and they were also evaluated by association analysis. Results of this study provide us valuable suggestions in genetic improvement and breeding of new varieties and also lay a basic foundation for further application of cloning of economic genes in selective breeding program and marker-assisted selection.

## Introduction

Chinese shrimp, *Fenneropenaeus chinensis*, is one of the most valuable mariculture species in China. It is mainly distributed along the coast of Bo Hai and Yellow Sea in China and the west coast of Korean peninsula. The booming development of penaeid shrimp industry ever since the late 1980s makes it a pivotal aquaculture specie that support both commercial fisheries and mariculture and once account for about 70% of shrimp production during its 1991 peak in China. However, the industry had not been spared in the outbreak of communicable disease (especially white spot syndrome virus, WSSV) that significantly affect shrimp aquaculture worldwide since 1993. Worse still, other issues such as depression of germplasm resources and deterioration of farming environment also arise. Chinese researchers have been fully aware of the importance of genetic and genomic studies, especially the genetic basis of traits of economic importance for the purpose of breeding improved varieties of high-yield or stress-resistance, which was also the critical point to solve the bottleneck of prawn industry and to maintain their sustainable development.

Genetic parameter assessment on phenotypic level could lead to a better understanding to the question that how much the economic traits are controlled by genetic factors. Quantitative genetics has also been applied in analysis and improvement of genetic basis and germplasm resources in prawn, such as *Penaeus monodon*^1^, *Penaeus japonicas*^2^ and *Litopenaeus vannamei*^3^. A mass of selective breeding techniques and comprehensive genetic parameters evaluation of economic important traits had also been well conducted in *Fenneropenaeus chinensis*^4^. With the application of modern molecular biology, molecular tools supply more direct and effective approaches to elucidation genetics of prawns, which has been a crucial task in their breeding studies for the purpose of genetic improvement. As the most widely applied molecular tool, the high-resolution genetic linkage maps are not only greatly useful in quantitative trait locus (QTL) mapping, marker-assisted selection (MAS) and hereditary basis of various kinds of biological phenomenon, but can be utilized in genomic applications such as genome assembly and comparative genomics research^5–7^.

The key prerequisite for a high-resolution linkage map is exploitation of markers that are good representation and widespread in genome [most appropriate, single nucleotide polymorphisms (SNPs)]. Unfortunately, abundant development of SNPs in non-model species used to be subject to the cost and genotyping technique. However, it became entirely available benefitted from the burgeoning high-throughput sequencing, especially with the development of Genotyping-By-Sequencing (GBS) represented by RAD (restriction site associated DNA)^8^ and several more efficient reduced-representation sequencing methods^9,10^. For example, 2b-RAD method developed by Wang et al.^10^ hold great promise for marker development in organisms lacking extensive genomic resources, owing to its streamlined protocol, even and tunable genome coverage and flexibility. Taking advantage of similar techniques, high-resolution genetic maps (with marker density < 1 cM) in aquatic species with poor molecular basis have been reported in *Chlamys farreri*^11^, *Apostichopus japonicas*^12^, *Larimichthys crocea*^13^, *Ietalurus punetaus*^14^, *Cyprinus carpio*^15^ etc.

Genomic research in penaeid prawns remains insufficient, as the relatively large genome and high proportion of repetitive sequences imply significant challenges for these species. Until just recently, the whole genome of *Litopenaeus vannamei* was published and provided the first glimpse into penaeid genome^16^. In *F. chinensis*, we have made many efforts in its genetic studies focusing on growth^4,17^, immunity^18,19^, effects of inbreeding^20^ and feed efficiency^21^. Remarkable progress have also been achieved by transcriptome sequencing for identification of disease-resistant genes and polymorphisms in these genes^22,23^. However, the constructed linkage maps of *F. chinensis* that would certainly be a valuable tool for the purpose of genetic basis dissection as well as genome assembly^24^, showed low resolution^25^ thus limiting their further applications. A basic genome survey and high-resolution linkage map became an urgent need.

In the present study, genome survey sequencing was conducted for the first time in *F. chinensis* to describe preliminary genome characteristics. Taking advantage of the breeding resource continuously selected in the last 13 years, we built a mapping population by strict mating of male and female of distinct phenotypic differences according to the mating design with a method of artificial insemination. 2b-RAD method was then employed to develop representative SNPs that evenly covered the whole genome in all progenies. On that basis, we established the first high-resolution genetic map of *F. chinensis*. QTL mapping was conducted to detect markers related to main phenotypic traits. Association analysis was also performed to verify the mapping results simultaneously. Construction of this genetic map and QTL mapping would help us understand the genetic basis of economic importance traits, and provide us with essential suggestion in genetic improvement of *F. chinensis*. It also lays a solid foundation for screening and cloning genes of economic importance in selective breeding program and further application of marker-assisted selection.

## Results

### Genome survey sequencing of *F. chinensis*

**Figure 1:**
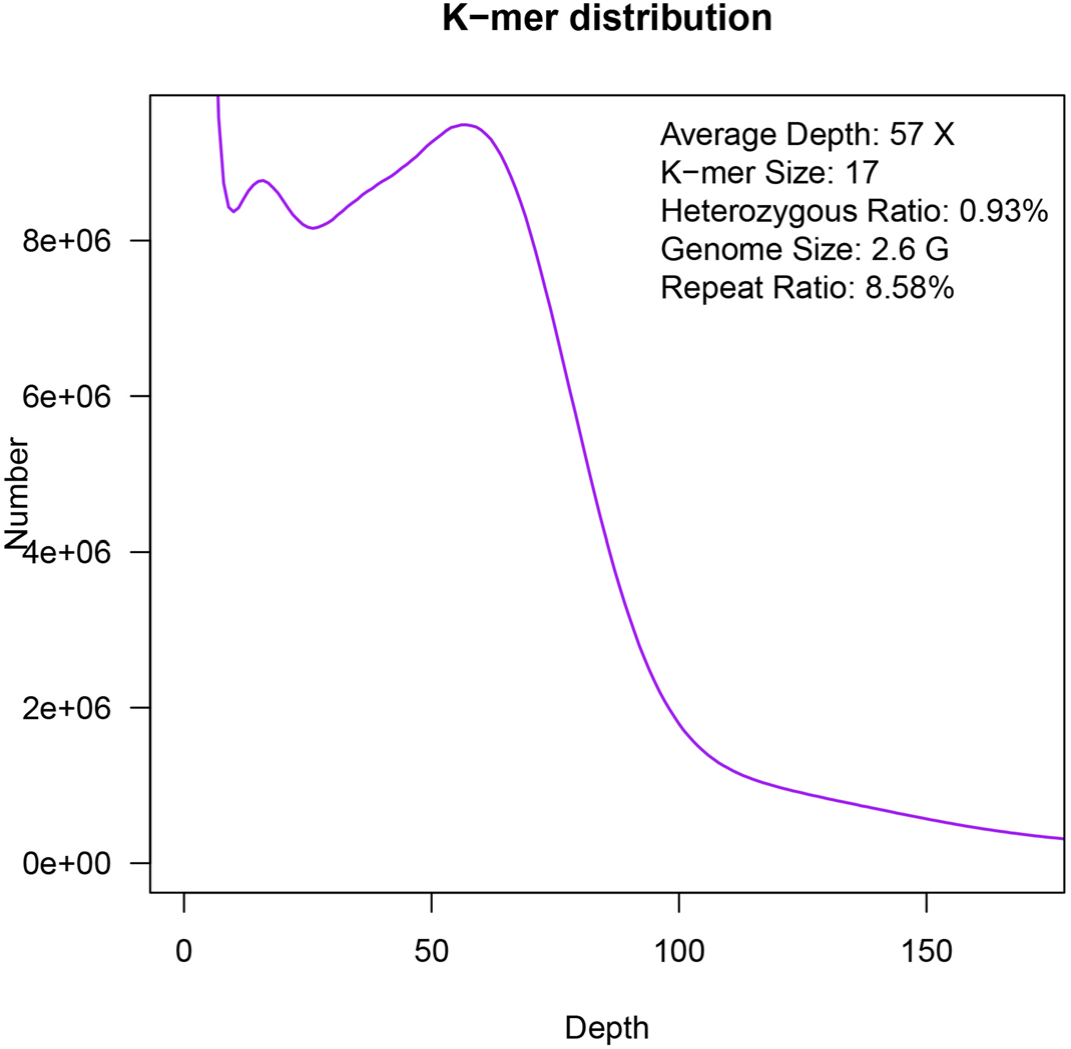
17-mer distribution of sequencing reads. 17-mer sequences were extracted from filtered database of pair-end library.

The pair-end DNA library with 300–400 bp insert size was sequenced for genome survey analysis. Illumina X Ten platform output a total of 108.84 Gb data, of which 91.4% were retained after quality filtering. 99.48 Gb high-quality data covered approximately 37-fold genome size of *F. chinensis*, with the estimated genome size of 2.66G inferred from *K*-mer analysis. A curve of 17-mers frequencies were calculated and constructed in Fig. 1. The major peak of *K*-mer depth was 57×. According to this result, we speculated that the heterozygosity of *F. chinensis* genome was relatively high (0.93%). The calculated GC content was 37.58%.

Taking advantage of all these sequencing data, de novo assembly of the *F. chinensis* genome generated scaffolds that covered 2,083,205,357 bp, corresponding to 78% of genome coverage. Soap *denovo* produced a large number of short contigs with the mean length of 221 bp and an N50 size of 247 bp (Table 1), which might be owing to the difficulty in highly repetitive genome assembly. A total of 6,665,022 scaffolds with an N50 size of 503 bp were further produced, of which 339,106 were longer than 1 Kb and the longest scaffold reached 100 Kb. The genome was not well assembled owing to its complexity and the highly repetitive nature, which essentially forbade its assembly only by short-read whole genome sequencing or other classical tools.

**Table 1:** Statistics of genome assembly for *F. chinensis.*

	Contig	Scaffold
Number	9,087,278	6,665,022
Total length (bp)	2,058,923,430	2,083,205,357
Longest (bp)	100,693	100,693
Shortest (bp)	100	100
> 1 Kb	103,693	339,106
Mean length (bp)	221	304
N50 (bp)	247	503

### Data processing of 2b-RAD sequencing and marker genotyping

The mapping family consists of 2 parents and 273 progenies. Based on these libraries, a database of 3,787,890,709 reads was output from HiSeq-2500 Sequencing System. Sequencing of paternal and maternal libraries produced 16.5 and 15.5 million reads respectively, in which the proportion of high-quality reads reached 85.75% on average. Sequencing also produced 8.94–21.31 million reads per progeny, with an average of 82.11% high-quality reads remained. 345,635 representative reference sites were generated by clustering parental reads, including 286,345 parent-shared and 59,290 parent-specific unique tags after strict filter of low-quality sites. These data formed the high-quality reference sites for the subsequent genotyping. Sequence depth in two parents were 32× and 26× respectively, higher than that in progenies, which ranged from 10.2× to 31.5× with an average of 19.8× . Both parents and progenies were sequenced to a sufficient depth that were well above the request of high accuracy genotyping^26^.

In total, 36,762 polymorphic SNPs and 8414 dominant markers were identified from RAD-typing program. In co-dominant SNPs, the proportions of 1:2:1-type (hk × hk), 1:1female-type (nn × np) and 1:1 male-type (lm × ll) were 29.5%, 35.5% and 35% respectively. Dominant markers of 3:1 segregation pattern in progenies were not included in subsequent analysis due to their inability to construct sex-specific maps. Polymorphic markers that could not be genotyped in at least 80% of the progenies were discarded (3.2%). An average of 55.1% polymorphic markers, which were not strictly conformed to the mendelian inheritance, were also abandoned. As a final result, 14,436 SNPs and 3768 dominant markers conformed to the expected Mendelian ratios (P ≥ 0.05) and proceeded with further linkage analysis. For all these markers, segregation ratio test of parental genotypes (1.049:1) and the rate of homozygotes (45.91%) showed no significant bias to any parent, or obvious bias to homozygote/heterozygote.

### High-resolution linkage mapping

**Figure 2:**
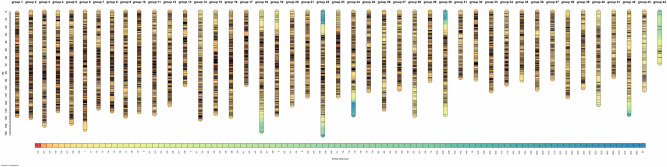
The high-resolution consensus map of *F. chinensis* which contain 12,884 markers in 44 linkage groups.

A total of 18,204 markers met the standard for pseudo-testcross strategy of linkage map construction. At the LOD threshold of 5.0, these markers were grouped into 44 linkage groups, corresponding to the haploid chromosome number of *F. chinensis*. The male map contained 6509 markers and spanned 3711.41 cM, and the length of each linkage group ranged from 48.42 to 125.97 cM. While the female map contained 7138 markers and spanned 5069.74 cM, and the length of each linkage group ranged from 60.06 to 153.79 cM. The average marker interval of male and female maps were 0.58 cM and 0.72 cM respectively (Table 2). 1463 hk × hk shared markers were the same between the male and female maps. The recombination rate between the female and male maps ranged from 0.84 to 1.59 among all linkage groups and was 1.29 in average. Shared markers were also used as anchor markers to merge two sex-specific maps in Joinmap program. The consensus map contained 12,884 markers and spanned 5257.81 cM with an average marker interval of 0.41 cM (Fig. 2). The length of each linkage group ranged from 67.62 to 162.25 cM, and the marker number varied from 28 to 440 across linkage groups (Table 3).

**Table 2:** Summary of sex-specific and consensus genetic maps.

	Male map	Female map	Consensus map
SNP number	6509	7138	12,884
Linkage group number	44	44	44
Average number of markers in each group	147	162	292
Average marker interval (cM)	0.58	0.72	0.41
Average group length (cM)	84.35	115.22	119.49
Total length (cM)	3711.41	5069.74	5257.81

**Table 3:** Summary of consensus genetic map in *F. chinensis.*

Linkage group	Number of markers	Average marker interval (cM)	Length (cM)	Female/male recombination rate
1	440	0.31	136.65	1.39
2	433	0.32	139.33	1.24
3	404	0.37	149.42	1.10
4	401	0.33	130.29	1.52
5	442	0.33	146.55	1.20
6	453	0.34	154.81	1.14
7	383	0.33	126.88	1.30
8	393	0.33	130.59	1.42
9	379	0.36	137.40	1.35
10	364	0.36	131.66	1.15
11	382	0.35	134.58	1.08
12	327	0.38	122.39	1.40
13	258	0.37	96.18	1.47
14	333	0.43	141.20	1.43
15	376	0.36	134.06	1.20
16	388	0.36	137.87	1.35
17	331	0.29	95.68	1.45
18	318	0.5	157.10	1.26
19	312	0.44	135.43	1.18
20	277	0.44	122.78	1.37
21	299	0.37	111.01	1.53
22	303	0.54	162.25	1.24
23	314	0.35	111.03	1.23
24	255	0.53	135.80	1.37
25	303	0.34	104.06	1.18
26	271	0.47	128.17	1.38
27	268	0.38	102.03	1.42
28	249	0.55	136.48	0.84
29	216	0.42	90.60	1.49
30	264	0.51	135.07	1.44
31	191	0.46	86.61	1.59
32	223	0.4	88.69	1.11
33	288	0.36	104.07	1.41
34	252	0.38	95.36	1.40
35	230	0.39	90.02	1.34
36	236	0.43	102.06	1.32
37	203	0.44	88.40	1.29
38	225	0.5	111.82	1.39
39	197	0.51	100.08	1.15
40	206	0.6	122.23	1.01
41	164	0.52	85.23	1.22
42	186	0.73	135.02	1.46
43	119	0.88	103.25	1.04
44	28	2.42	67.62	1.14
All	12,884	0.41	5257.81	1.29

Marker distribution and intermarker distance were evaluated in consensus map. On linkage group level, numbers of markers in each group ranged from 119 to 440 except the smallest LG44 that only contained 28 SNPs. Marker interval analysis showed that 92.5% of the consensus map were covered by markers with interval distances of less-than 1 cM. 98.2% of markers in consensus map showed an interval of less-than 2 cM. Only 24 marker intervals were longer than 5 cM. This indicated that the distribution of markers in this map were even whether among or within linkage groups. 908 markers (7.05% of all markers on the map) showed a marker interval of zero which means they do not have any recombination. These clustered markers were referred as bin signatures.

The estimated genome length was 5275.85 cM (Ge1) and 5299.59 cM (Ge2). Taking the average of these two estimated value using different methods, the final expected genome length was 52,787.72 cM. Genome coverage of the consensus map was nearly complete and reached 99.43%. With an estimated genome size of 2.66 Gb, the ratio of physical and genetic distances was 503.05 Kb/cM.

### QTL mapping and association analysis

The target traits in the present study included overall length (T1), body weight (T2), body length (T3), length, width and height of carapace (T4–T6), rostrum length (T7), number of spinule on rostrum (T8), telson length (T9), gender (T10), dimensions of uromeres from 1 to 6 (T11–T28) and antiviral property (T29). The phenotypic data of all quantitative traits followed the normal distribution. Results of Pearson’s correlation analysis between 29 characters were shown in supplementary Figure S1. High correlation coefficients were observed in weight-related traits (0.895–0.940 between overall length, body length and body weight).

Chromosome-wide critical threshold values for QTL detection were measured by the LOD significance threshold (α < 0.05), which was estimated by 1000 times’ permutation test. In consensus map, 86 significant QTLs of 27 quantitative traits were detected in 29 linkage groups (Table 4), according to the respective chromosome-wide critical threshold values estimated by permutation tests. Association analysis revealed a similar distribution pattern across all linkage groups as in QTL mapping analysis, which means that the peak of a QTL region also had a significant signal of association analysis in most cases. The proportion of phenotypic variation explained by each QTL ranged from 5.2 to 24.3%.

**Table 4:** QTLs for 5 representative traits.

Traits	QTL	LG	Position/cM	Markers	LOD	R^2^ (%)
T1	T-1.1	2	29.32	m1266–m1029	4.56	7.4
T1	T-1.2	4	31.27–32.16	f3096–f2243	3.59	5.9
T1	T-1.3	6	117.62	f55	7.29	11.6
T1	T-1.4	18	105.76	m701	3.57	5.9
T1	T-1.5	18	135.53	df1127	6.04	9.7
T1	T-1.6	33	27.09	m1835	3.73	6.1
T2	T-2.1	18	138.53	df1127	15.79	23.4
T2	T-2.2	21	14.09–14.97	f5951–f6124	6.23	10
T2	T-2.3	21	54.71	f2170	5.8	9.3
T2	T-2.4	31	26.66	m1504	4.27	6.9
T2	T-2.5	33	72.07–72.27	m4916-dm431	4.41	7.2
T2	T-2.6	43	99.05	f5245	5.57	9
T3	T-3.1	6	103.87	m2349	6.2	9.9
T3	T-3.2	8	44.69–45.01	f5281–f1329	7.59	12
T3	T-3.3	10	3.45	f3448	7.74	12.2
T3	T-3.4	15	32.83–33.14	h1054–f1100	6.04	9.7
T3	T-3.5	18	127.59	f768	4.89	7.9
T3	T-3.6	43	99.05	f5245	16.51	24.3
T10	T-10.1	6	121.34	f1635	84.45	75.9
T29	T-29.1	2	10.15	dm728	8.17	12.9
T29	T-29.2	6	117.34–117.78	f4812–f2012	15.82	23.4
T29	T-29.3	7	87.71	f2179	6.89	11
T29	T-29.4	8	44.69	f5281	9.26	14.5
T29	T-29.5	13	96.18	m4472	15.81	23.4
T29	T-29.6	15	2.68	f5337	10.6	16.4
T29	T-29.7	22	132.07	df570	6.82	10.9

*T1* overall length, *T2* body weight, *T3* body length, *T10 *sex, *T29* antiviral properties.

**Figure 3:**
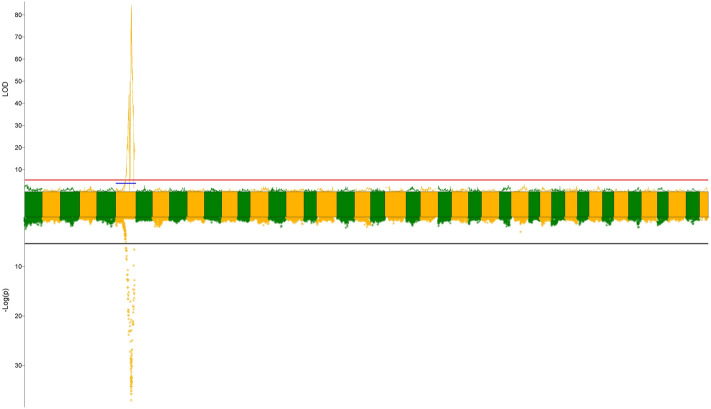
QTL mapping and association analysis of sex in *F. chinensis*. The top half of figure indicated the LOD of QTL analysis, the red and blue lines indicate the genome-wide and chromosome-wide significance thresholds, respectively. The bottom half of figure indicated –Log(*P*) of association analysis, the black line indicated the threshold modified by bonferroni correction.

While for sex, a highly significant QTL was fine mapped at 121.34 cM on LG6 (Fig. 3). The nearest marker named f1635 with the highest LOD score (84.5) explained 75.9% of variation in sex. Association analysis also revealed a same set of sex-related markers with high statistical significance and all of them fell into the narrow QTL region identified by the QTL mapping analysis. We selected 47 sex-related markers with statistical significance and 39 of them could be anchored to assembled contigs/scaffolds of survey sequencing. 6 sequences could be successfully aligned to the existing genome of *Penaeus vannamei* and 3 genes were annotated (Supplementary Table S1).

**Figure 4:**
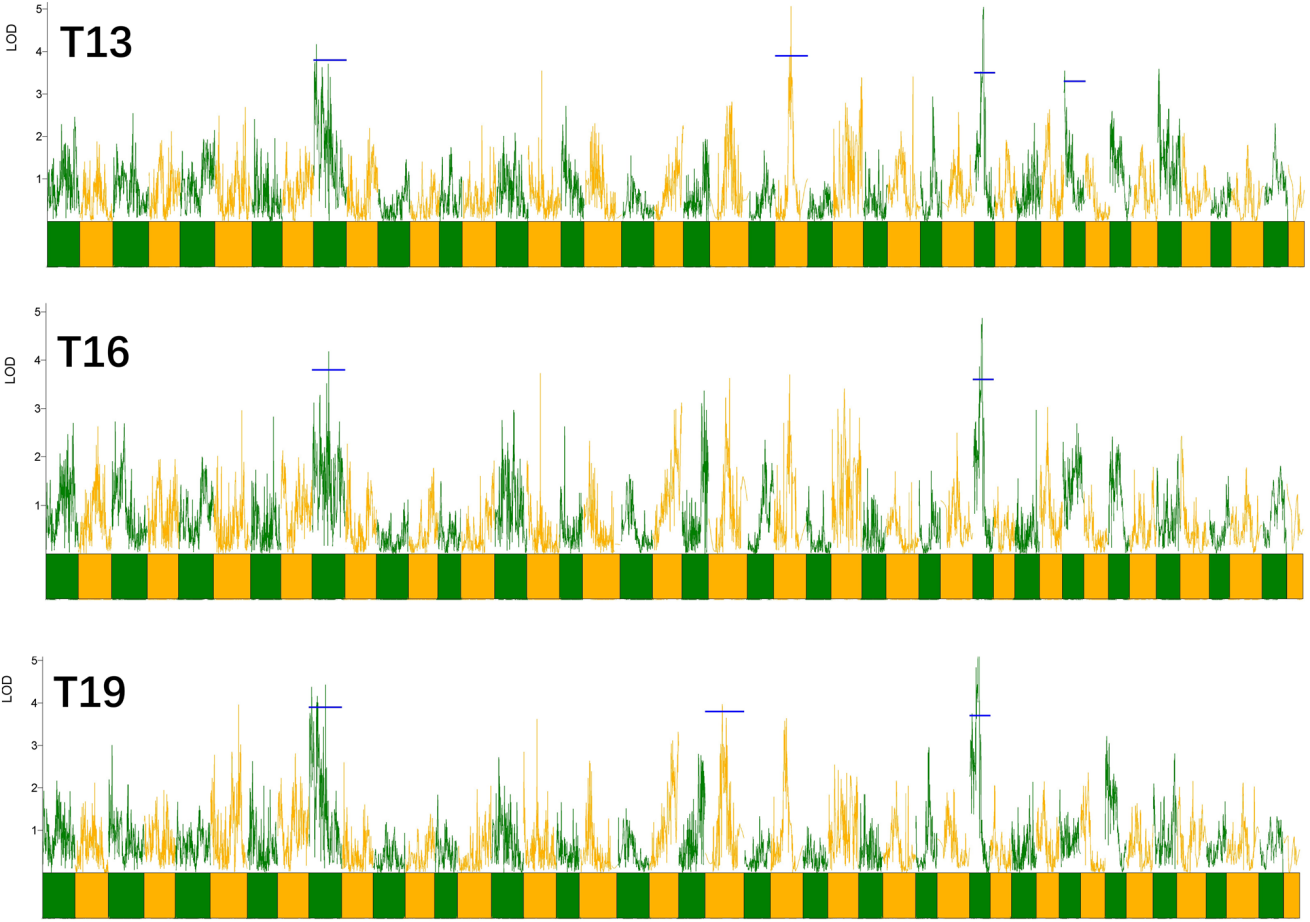
A scan of LOD profiles in a group of analogous traits. T13, T16 and T19 represents the height of 1st, 2nd and 3rd uromere, respectively.

The records of phenotypic traits were relatively detailed in the present study, thus a scan of LOD profiles in analogous traits were conducted. Some traits exhibited quite similar LOD distributions. As shown in Fig. 4, LOD profiles among the height of 1st, 2nd and 3rd uromere were exactly similar.

### Integration of the linkage map and genomic scaffolds

**Figure 5:**
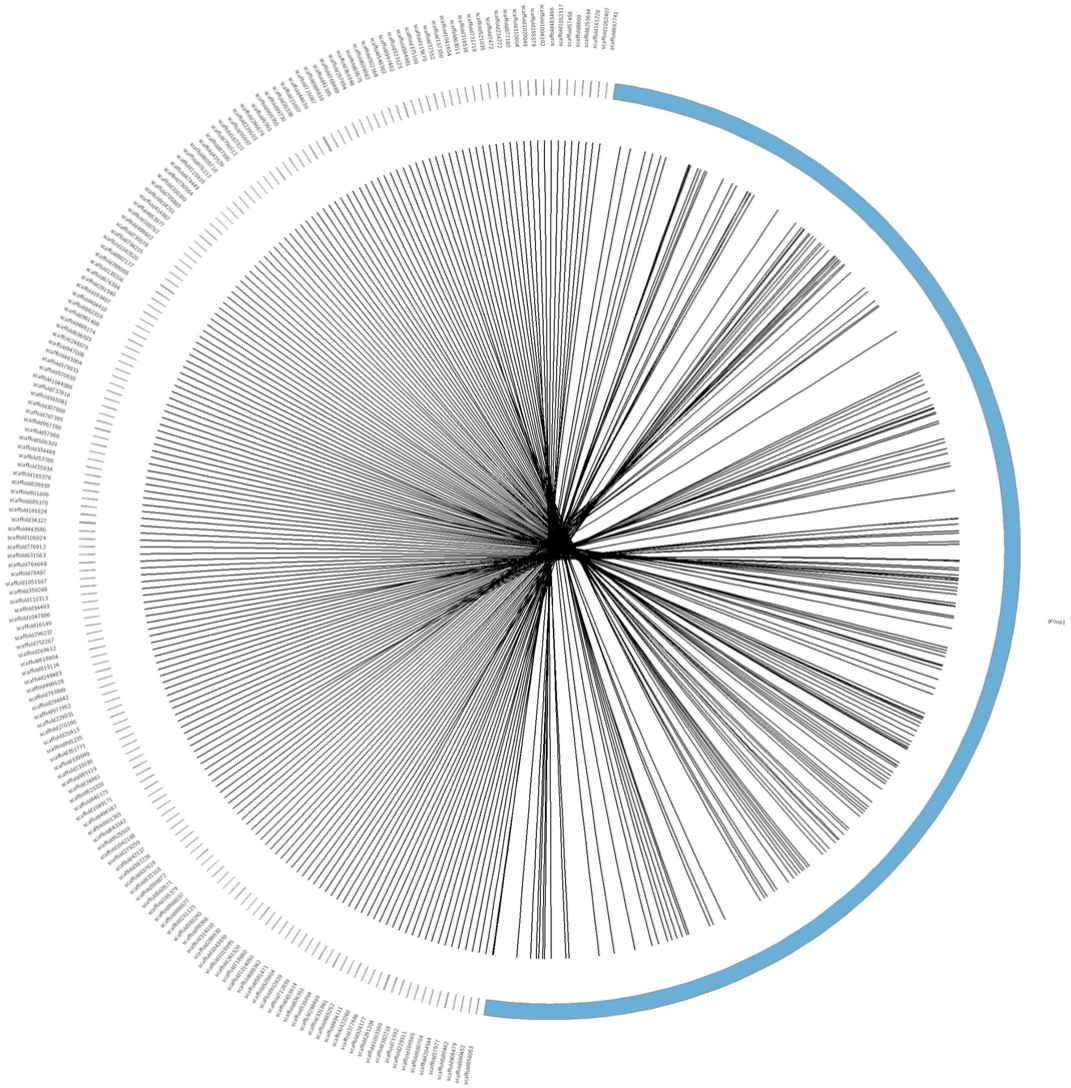
Circos demonstration of the integration of LG1 and genomic scaffolds.

Of the 12,884 markers in the consensus map, 9615 (74.63%) could be anchored to 8937 scaffolds. These scaffolds could serve as ‘long’ surrogates for 2b-RAD tags to enhance their utility in unifying genetic resources^11^. Combined with these resources, the high-resolution genetic map could also serve as an important tool for directing genome assembly by orienting genomic scaffolds. To show this potential, we integrated linkage group 1 with genomic scaffolds. Collinear analysis showed that 313 genomic sequences could be mapped to LG1 and had significant genomic synteny. An example of the integration of LG1 and 185 long scaffolds was shown in Fig. 5.

## Discussion

“Huanghai No. 2”, our research object, is a very significant cultivated variety of Chinese shrimp. It was established by crossing several domesticated and wild germplasm resources, and had been continuously selected for growth, survival and resistance to white spot syndrome virus (WSSV) over 13 generations by means of pedigree method. Strict mating of male and female parents were controlled according to high selection index based on estimated breeding values and inbreeding coefficient (< 1%). Previous studies have shown that the body weight had significantly increased by 34.31% compared with wild population^17^ and the realized genetic gain was 3.72% per generation. The estimates of heritability in different traits were low to moderate in magnitude (0.00–0.36) in different assessment^4^. Construction of genetic map and QTL mapping would provide us with essential suggestions in further genetic improvement of the breeding population.

Penaeid is one of the most important aquatic economic species around the world. In spite of species abundance and extensive cultivation, seldom efforts have been devoted to decoding their genomes. Until just recently, whole-genome sequence of *Litopenaeus vannamei* has been reported^16^, which provided the first glimpse into penaeid genome. The genome revealed relatively large (~ 2.6G with 44 linkage groups), extremely high level of heterozygosity and a remarkably high percentage of repetitive sequences (~ 79.37%)^16,24^. The complexity of penaeid shrimp genome thus meant a great challenge in sequencing and assembly. Thereby it could hardly been accepted as a conventional tool owing to the expenditure at least in the near future.

Until the acquisition of full genome sequence variation becomes possible for other shrimps, molecular markers and high-resolution linkage maps have been crucial for genetic analysis on species diversity, genetic elucidation of important traits, speciation and evolution. Thus far, linkage maps have been constructed in many penaeid species^27,28^ including *F. chinensis*^25^. However, these maps were mostly built with hundreds of markers and the resolution was generally low (mostly10–20 cM), thereby limiting their use in fine-scale QTL mapping and many other applications. Genome survey and high-resolution linkage map in *F. chinensis*, which were cost-effective in organisms lacking extensive genomic resources and were exceptionally essential bases in elucidation of economically important traits, are urgently needed.

Genome survey performed by single DNA library on Illumina X Ten platform produced a large number of short scaffolds, with N50 only reached 0.5 kb. The characteristic *K*-mer curve highlighted the complexity of *F. chinensis* genome, which might be the major reason of difficulty in assembly procedure. A heterozygosity of 0.93% was observed, which was lower than that of oyster^29^, but higher than most other species^15,30,31^. Generally, genome characteristics of *F. chinensis* were quite similar to that of *L. vannamei* except the ratio of repetitive sequences^24^, which might be caused by its special characteristics. Although this “genome” was probably sufficient to serve as mediums for short-read tags to identify genes or associate with public genomic resources, assembly of this genome was essentially uncompleted. Again, the results confirmed that the highly repetitive nature of aqua-genomes forbade their assembly only with short-read sequencing or other classical tools, and different approaches should be taken into account^32^.

Although genomic studies has already benefitted from the burgeoning high throughput sequencing, large-scale linkage mapping in marine animals with large and complex genome still call for a cost-effective sequencing plan that reasonably balances the sequencing cost and genotyping accuracy. 2b-RAD technology was adopted in this research since it featured even and tunable genome coverage to provide reliable and flexible SNPs^10^. The technique has been successfully applied to mapping studies in marine animals lacking genomic resources^11,12^. In the first large-scale development of SNPs implemented in *F. chinensis*, a strategy of standard BsaXI libraries instead of a subset of BsaXI sites deriving from reduced representation libraries were chosen, although it might require greater investment. In order to insure the genotyping accuracy, the amount of sequencing required was references to the suggested depth (20× and 15× for parents and progenies through simulation analysis, genotyping accuracy > 96%)^26^ and the total number of restriction sites predicted according to genome size and GC content.

Sequencing of parental and progenies’ BsaXI sites produced 16.0 and 13.8 million reads in average and more than 85% of reads remained after quality filtering. This demonstrated that sequencing experiments were successful and markers we developed were fairly reliable. In total, 36,762 high-qualified representative SNPs and 8414 dominant markers were genotyped. These data provided valuable resources for further genetic studies. The insufficient length of RAD tags (typically 35–100 bp depend on different methods) was a major concern that might limit their application in other genetic or genomic aspects. Database obtained from genome survey, however, provided us a feasible solution. Although the assembled scaffold N50 only reached 0.5 kb, they could already serve as mediums for 2b-RAD tags to identify genes surrounding them or associate with public genomic resources.

In the process of quality filtering, a large number of markers were discarded owing to their inconformity of Mendelian separation ratio. The deviation of molecular markers were very common in marine animals^12,33–36^ including Penaeus^24,27,28^. However, the proportion of distorted markers in the present study were astonishing, for it reached up to 54.9% and 55.2% in co-dominant and dominant markers. Terrible segregation distortion is usually caused by genomic structural variation^37^. But it was not the likely reason for this problem here, for the parents of mapping family were derived from a stable population and segregation ratio test of parental genotypes as well as the rate of homozygotes were normal. The early lethality and artificial selection during family construction process came to our attention. As a rule of thumb, only about half of hatched larvae survived after Nauplius, Zoea and Mysis stage. Artificial selection was then conducted for 3 times in different Post-larvae stages (2000 in P5, 1000 in P12 and 500 in P19) on the basis of selection criterion of better fitness. In this way, the sample of mapping individuals were chosen from 10,000 initial Nauplius, and the elimination rate reached up to 95%. That was probably the main reason of the novel segregation distortion as high genetic load were reported in marine animals^38,39^. Selection effects incorporation with high genetic load might act on certain alleles/genotypes and lead to segregation distortion.

We focused on this interesting phenomenon and these distorted loci in another study. Although their exact genomic or genetic positions could not be displayed on the basis of the imperfect genome assembly or genetic map in this research (addition of deviated markers significantly disturbed normal marker order of genetic map) for now, characteristic analysis of genotypes detected high linkage disequilibrium among these markers which tended to cluster. It was supposed that the authentically selected loci were involved in our deviated markers whereas more deviated markers were possibly neutral loci caused by hitch-hiking effect rather than being selected themselves. Gene and functional studies based on all deviated markers indicated imprecise and redundant biological activities and signaling pathways^40^. To further identify the authentically selected loci, comparison among different families and mapping methods might be effective. And this is the way we have been working on.

Low-resolution linkage maps in penaeid^25,27,28^, although extensively reported, were powerless in fine-scale QTL mapping or other genetic applications. The high-resolution linkage map we built contained 12,884 markers with a marker interval of 0.41 cM. This was the highest marker density in maps constructed for *F. chinensis* to our knowledge. The marker density was also higher compared to other linkage maps in aquaculture species taking advantage of next-generation sequencing technology^15,24,41^. Moreover, the distribution of markers is relatively even in our map. Marker interval analysis revealed that 98.2% of markers in consensus map showed an interval of less-than 2 cM. Only 7% of mapped markers showed a marker interval of zero. They were clustered together as bin signatures but the orientation was unknown. This high-quality map could not only facilitate the discovery of quantitative trait genes but provide a valuable reference in genome assembly and scaffolding.

In sex-specific maps, female map was 36.6% longer than male map, whereas the number of markers in female map was only 9.7% larger than that in male map. The female map therefore was observed higher recombination rates in almost all linkage groups except LG28. This phenomenon has been reported in other penaeid such as *Litopenaeus vannamei*^42^ and *Penaeus monodon*^27^, which indicated that recombination rate may be different in male and female. However, same recombination rate in both sexes^43^ or confused results^42,44^ were also reported in other studies. The diverse results may be due to different types or unreliable density of markers. If this is the case, results concluded from our research taking use of large amount of SNPs were relatively credible. Although there were still no clear explanation, different recombination may be closely related to genomic sequences, chromosome structure and chromosomal assignment^36^.

Body size and disease-resistance traits are of particular interest to breeders due to their high commercial significance in prawn aquaculture. QTL mapping represents an efficient approach to identify genetic loci underlying these traits in genetic breeding, which had been successfully applied in aqua-species such as fish^6,45^, shrimp^24,46^ and scallops^11^. In our research, phenotypic traits were measured as specific as possible and conducted in QTL analysis. Scan of LOD profiles for analogous traits showed that some traits exhibited quite similar LOD distributions. This illustrated the pleiotropic of the multi-gene regulation and indicated that similar genes might control these traits. For example, QTLs detected for different uromere sizes shared 2 identical loci and 1 locus that within 5 cM. QTL results were also supported by the association analysis, a complementary approach to evaluate QTL mapping. Markers located at the confidence intervals of QTLs constituted a valuable marker set for further evaluation of their utility in marker-assisted selection.

Sex determination mechanism in Penaeus remained unknown as most crustacean animals do not evolve sex chromosomes. Whereas, highly correlated regions involved in sex determination were reported in fishes and crustacean^11,47,48^, which might point the way toward possible gene or chromosomal region. In this study, a narrow chromosome region highly related to sex determination was detected near 121.34 cM on linkage group 6 by QTL mapping. Association analysis also identified a set of related markers in the same region. These results provided us valuable resources in the further gender studies in *F. chinensis*. Unfortunately, only 3 sex-related markers were annotated as a result of the lack of annotation information. These genes may only play an important role in biological activities such as cell cycle, reverse transcriptase and metabolism of steroids. Marker f4641 were tightly linked with D-beta-hydroxybutyrate dehydrogenase, a group of isozymes that catalyze activation and inactivation of estrogen and androgens in human. But we do not have direct evidence of their connection with sex-determining. The verification of sex-determining gene still needs further study.

## Conclusions

We conducted a genome survey for *F. chinensis*. It identified preliminary genome characteristics and provided valuable genomic resources in this important commercial shrimp. 2b-RAD method was employed for genome-wide development of SNPs. A high-resolution linkage map was also constructed with a marker density that has, to our knowledge, never been achieved in this specie. Several growth-related QTLs and one putative sex-determination region were detected, which verified the value of genetic foundation we completed preliminarily in this research. These genomic and genetic resources would without doubt provide valuable tools for genetic breeding studies and genomic research in penaeid shrimps.

## Materials and methods

### Genome survey sequencing of *F. chinensis*

An adult shrimp from the breeding population was randomly selected and applied to genome survey sequencing. Muscle tissue was dissected for genomic DNA extraction. A paired-end DNA library with insert sizes of 300–400 bp was constructed following the standard Illumina preparation procedure (TruSeq DNA LT Sample Prep kit). The purified DNA library was then sequenced on the Illumina HiSeq X Ten platform.

NGSQC tool kit^49^ was employed to remove low-quality reads or bases which may result from sequence errors. *K*-mer analysis was conducted with the help of jellyfish (v 2.2.4)^50^. The empirical formula of estimated genome size was G = *K*_num/*K*_depth, where *K*_num is the total number of *K*-mers, and *K*_depth is the average frequency. The filtered clean reads was assembled using SOAP denovo V2.04 to constructed contigs^51^. The framework of assembly was based on *de Bruijn* graph structure with the parameter –K 55.

### Construction of the mapping family

The parent shrimps were derived from the core breeding population of Huanghai No. 2. It was now kept in the Marine Genetic Breeding Center of Chinese Academy of Fishery Sciences (Qingdao, China), where also our studies were carried out. The brood stocks had been promoting sexual maturity for over 2 months since January before spawning. The parents of full-sib families were of known pedigree and chosen according to our breeding project. Mating of male and female parents were completely controlled by artificial insemination.

A standardized procedure for family construction was used in larvae rearing and cultivation of juvenile shrimp^4^. Hatched larvae went through three metamorphosis stages [Nauplius (N), Zoea (Z), Mysis (M)], and left about half of them survived to Post-larvae stage (P) in about 3 weeks. When the mean body length of each family reached one centimeter, a sample of 300 post-larvae was transferred into larger tanks (3 m^3^) for subsequent cultivation. When the mean body weight of adult shrimps reached about 9 g, one family with largest phenotypic variance (indicate relatively high genetic diversity) was singled out as the mapping population.

273 progenies with no obvious trauma were remained for linkage mapping study. Before that, 28 phenotypic characters mentioned above were measured. Shrimps were then transferred to an isolated workshop to test the antiviral properties (measured by survival time after infection of white spot syndrome virus), using an equivalent and single oral administration method (patent number ZL201210107377.8). All shrimps died after antiviral test. The muscle of each sample was dissected, fixed with 95% alcohol and stored at −80 ° in less than 2 h after death.

### 2b-RAD Library construction and sequencing

All progenies and two parents of the mapping family were gathered for further high throughput sequencing. The strategy chosen in this study was a simple and flexible method known as 2b-RAD. Genomic DNA was extracted using TIANamp Marine Animals DNA Kit (TianGen, BeiJing). We followed the protocol of Wang et al.^10^ to construct standard 2b-RAD libraries by adopting the Type IIB restriction enzymes BsaXI and original adaptors without any selective base in the terminal 3-bp positions. Therefore, the libraries of 33 bp tags contained almost all recognition sites of BsaXI in *F. chinensis* genome, which can develop markers that evenly covered the whole genome as much as possible. Each library contained an individual-specific barcode incorporated during the library preparation to facilitate the pooling of all samples. All libraries were then pooled together as planed and applied to an Illumina Hiseq2500-V2 for single-end sequencing (1 × 50 bp). In consideration of the de novo genotyping strategy and requirement of establishing high quality reference, libraries of two parents were given extra sequencing depth (1.5-fold higher than progenies).

### Sequence data processing and genotyping

Raw reads were first filtered to remove adaptors, terminal 3-bp ligation sites, reads with no restriction sites or containing ambiguous base calls (N), reads of inferior quality and mitochondrial origins. The remaining high-quality reads could be applied to subsequent genotyping. As there was no published complete genome sequences of *F. chinensis* until now, we employed the de novo genotyping strategy using the RAD-typing program v1.0^11^. Briefly, pre-processed reads of two parents were combined together and assembled into exactly matched allele clusters and locus clusters that allowed certain mismatches. Collection of consensus sequences from all locus clusters comprised the original parent-shared representative reference sites. The rough references were further filtered by excluding sites that either with insufficient sequencing depth or with depth far above expected (most likely derived from repetitive genomic regions or false alignment) using an iML algorithm^52^. Pre-processed reads of all individuals were then mapped to those high-quality reference sites separately. The most likely genotype for a SNP was calculated by posterior probability and finally determined by a likelihood ratio test. Only one SNP within a tag was retained for linkage analysis. For dominant markers, RAD-typing program can determine the absence or presence of each site and prevent incorrect presence calls from sites with misaligned reads^26^. Only those dominant markers with genotyping accuracy > 95% were retained for linkage analysis. All genotyping process were conducted under optimized default parameters for marine animals^11^.

### Linkage map construction

Polymorphic markers that were heterozygous in at least one parent and could be genotyped in at least 80% of the progenies were considered as qualified segregating markers. For these markers, goodness of fit of the observed with the expected Mendelian ratio was assessed using the *x*^2^ test. Those markers conforming to the expected Mendelian ratios (*P* > 0.05) were retained for further linkage analysis. The linkage map was constructed using the Joinmap 4.1 software^53^. Pseudo-testcross strategy was adopt to construct sex-specific maps taking advantage of maternal and paternal datasets (mapping population CP). Linkage groups were determined according to a logarithm of odds (LOD) threshold of 5.0. The regression mapping algorithm was selected for map construction. Recombination rates were converted to map distances (centi Morgans, cM) based on the Kosambi mapping function. The ratio of female/male recombination rate in each linkage group was calculated by shared markers in sex-specific maps. Maternal and paternal specific maps were integrated into a consensus map with the help of shared markers using MergeMap software^54^.

### QTL mapping and association analysis of phenotypic traits

The target traits in the present study included sex, antiviral property and 27 phenotypic quantitative traits. Distributions of quantitative traits including antiviral property were first checked if they followed normal distributions using the univariate procedure of MATLAB software. Traits that were significantly different between male and female were corrected for sex by centering the trait mean in each sex.

QTL analysis was carried out using the interval mapping method in MapQTL program. Chromosome-wide critical threshold values for QTL detection were measured by the LOD significance threshold (α < 0.05), which was estimated by 1000 times’ permutation test. Critical threshold values for genome-wide were approximated using Piepho’s method^55^. Interval mapping method was conducted by regression analysis and maximum likelihood method based on two interval markers. In each linkage group, possibility of a QTL was scanned every 1 cM, and a LOD score that exceeds the threshold value indicated a potential QTL. The percentage of phenotypic variation explained by each QTL (R^2^) could also be obtained. The peak of LOD score in each confidence interval was considered to be the most probable position and represented by the nearest marker.

All markers on consensus map was simultaneously tested between genotypes and phenotypes by association study, which was performed as a complement approach to QTL mapping using a Plink software package^56^. The significance of threshold was set *P* < 0.05. Genotypic effect of each loci was modified by bonferroni correction and showed by –Log (*P*).

## Supplementary Information

Supplementary Information

## Data Availability

The detailed genotyping information of codominant SNPs in mapping family were shown in Supplementary Table S2.
