# Supplementary material for: Genome survey and high-resolution genetic map provide valuable genetic resources for Fenneropenaeus chinensis
Source: Sci Rep. 2021 Apr 6;11:7533. doi: 10.1038/s41598-021-87237-4 (PMC8024304; doi:10.1038/s41598-021-87237-4)
Supplement: Supplementary file 1 — Supplementary Information [file 41598_2021_87237_MOESM1_ESM.pdf]

Title: Genome survey and high-resolution genetic map provide valuable genetic resources for *Fenneropenaeus chinensis*

Authors: Xianhong Meng[1,2,+], Qiang Fu[1,2,+], Sheng Luan[1,2], Kun Luo[1,2], Juan Sui[1,2], Jie Kong[1,2,*]

Affiliation: [1] Key Laboratory for Sustainable Utilization of Marine Fisheries Resources of Ministry of Agriculture and Rural Affairs, Yellow Sea Fisheries Research Institute, Chinese Academy of Fishery Sciences, Qingdao, 266071, China; [2] Laboratory for Marine Fisheries Science and Food Production Processes, Qingdao National Laboratory for Marine Science and Technology. Qingdao, 266071, China.

+ These authors contributed equally to the work.

*Correspondence and requests for materials should be addressed to J.K. (Email: kongjie@ysfri.ac.cn)

Supplementary Information

Supplementary Figure

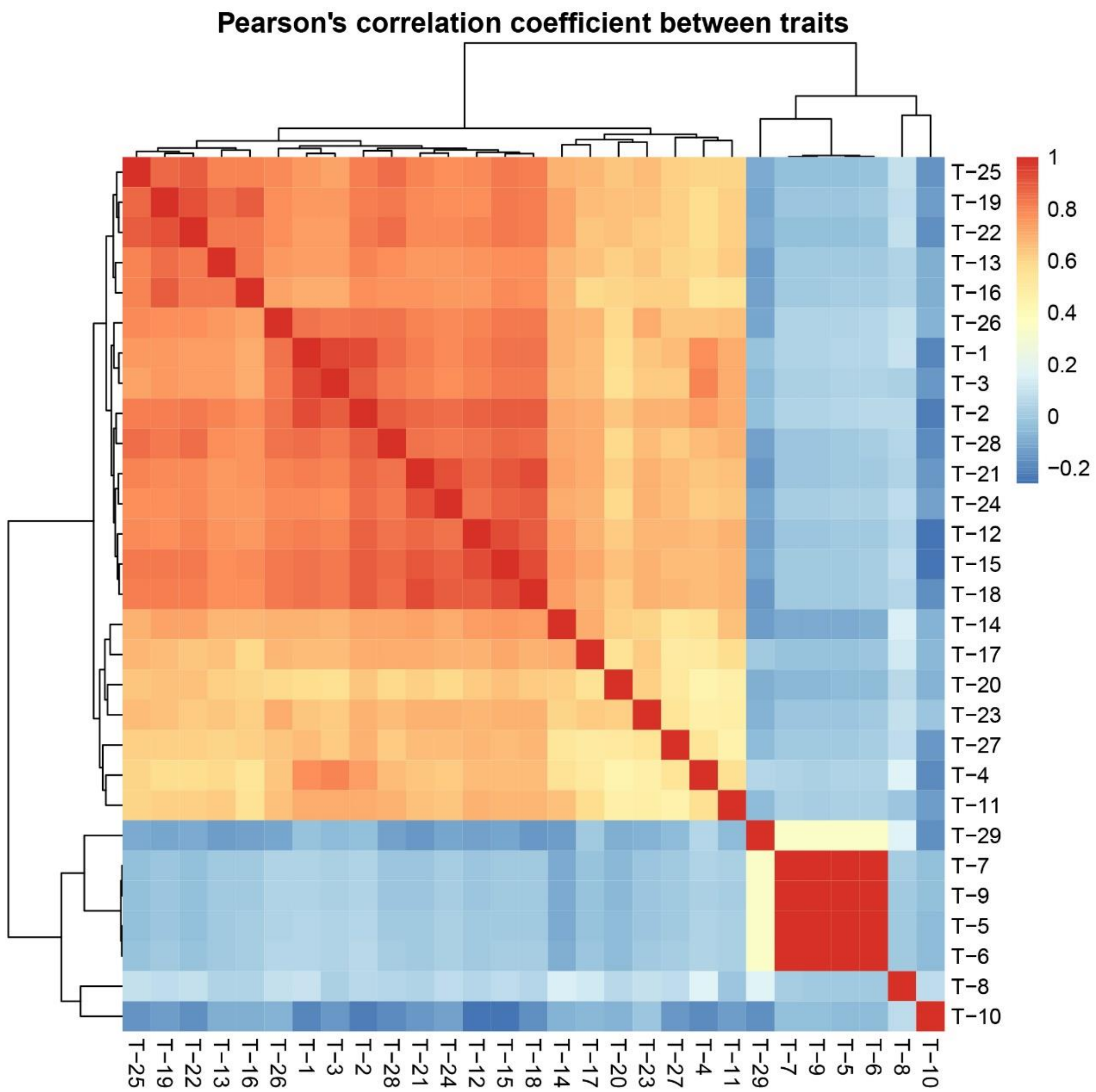

Supplementary Figure S1. heatmap of Pearson's correlation analysis between 29 characters

Supplementary Table S1 The annotation information of sex-related markers

Marker ID	Reference ID	Ref-Sequence	Survey ID	Gene	Accession Number
f3784	>ref-233036	GGGGAGAGGAGCTGGTGTTCAATGAGA	scaffold341789		
f1468	>ref-89758	ATTCCTACCACCCTCTCTCCATGCTCC	C54469922		
f4801	>ref-299024	CGCCACAGCACAAGGCCTCCTCCGCCT	scaffold173963		
f4812	>ref-299808	CACTCATCGACTCGGACTCCATTCCCT			
f2552	>ref-157690	GGACAAAGGAGAAAGCGTGAAGAAGGG	C53742805		
f55	>ref-3554	AGGGTGTCCACGCACCCTCCTCACCAG	scaffold1050818	putative RNA-directed DNA polymerase from mobile element jockey-like	ROT82827.1
f2012	>ref-124617	CCCGAACCTACTGTGGCTCCCAGACTG	C52655139		
f2988	>ref-186674	ATGGGAGGGAGGGACGGTGAGAGCTGG	scaffold939398		
f6097	>ref-381072	CATTGCAGGAGAAACAGTTTGCAGCAT	scaffold338215	uncharacterized protein LOC113828054	XP_027236792.1
f555	>ref-35075	TACTCTCCTACCCTTCCTCCCCCCATC			
f2281	>ref-140679	AAGAGAATTACTTCTCCTCCTTGTCCT	C58689652		
f5255	>ref-327492	ATCCCCAACACTATCACTCCAACACCA			
f612	>ref-38391	TGTGTGTCAACAGATGCTCCAACAAGC	scaffold122243		
f4317	>ref-267518	GGGAGAGGGAGAATGTGTAGGTGGGCG	scaffold994711		
f1662	>ref-102589	GGGTTAAGGAGGCAGAGTCACTGGTCA	scaffold179837		
m2210	>ref-131357	GTAAGTTGGAGGTATGGTAGCTTAGGT	scaffold509432		
f3588	>ref-221494	TAAGGAAGGAGAACAAGTGCATAAGGC	C50981811		
f4072	>ref-250842	ATCTCTCCCACACCCTCTCCTTCCGTC	scaffold1028943		
f5662	>ref-354956	CGCTGCAGCACATGATCTCCAGCTTAT	scaffold444790		
f4461	>ref-276653	CGATTGTCCACGCCCACTCCTACGGCG			
f3513	>ref-216571	AGCTCAGCAACCCCTCCTCCTTGCTTA	scaffold80204		
h673	>ref-168672	CCTCTTTTAACCCTGCCTCCATCTCCT			
f3831	>ref-236063	GGAGGGCCCACCTGGGCTCCCCACGGG	scaffold150247		
f1934	>ref-120170	CACTGGCGGAGGCTCTGTGTCTGTTTC	C56762042		

f5905	>ref-369992	TCCACCACCACTTTCTCTCCGTTATCC			
f4641	>ref-288215	CACCCAAACACGCCAACTCCCGCATTC	C46989668	putative D-beta-hydroxybutyrate dehydrogenase, mitochondrial-like isoform X2	ROT82587.1
f5398	>ref-337367	GAGGGAAGGAGGGATCGTAAGAATGCG	C47548549		
f5044	>ref-314802	TCCTTTACTACCCTACCTCCCTCCGTC	C42278840		
f2123	>ref-131672	TATGGATGGAGAGATAGTTTTAGGTAA	scaffold651175		
m34	>ref-1925	CCCCCTAATACTCCCCCTCCATCCTGC			
m5426	>ref-320201	TCTTTCCTAACCTCGGCTCCTTCTTTC			
h332	>ref-83358	GGAGGCAGGAGGTACGGTGAAATCATG	C57887271		
f5405	>ref-337974	AACACCGAAACCAGATCTCCTGCGTCC	scaffold272925		
f4855	>ref-302644	CACTATCCCACCTGCTCTCCCTCCTTC	scaffold404113		
f3664	>ref-225820	CAGCCACGGAGAGGGAGTGGCCGCACG	C56627369		
f1635	>ref-100426	AAGGCGTGGAGTATAGGTGGTGGAGGA	scaffold323590		
f3787	>ref-233163	ATACCACATACAATGCCTCCTCCCTCA	scaffold405429		
f1975	>ref-122707	GATACTCGTACGTACGCTCCTTTTAGA	scaffold447370		
f5532	>ref-346162	ATCAACACTACTATCGCTCCTCCATCA	scaffold953913		
f847	>ref-51535	ATCTCCGGGAGGGCGTGTTGAAGGCCG	scaffold416384	Nek3 protein	ROT73799.1
f963	>ref-58351	TGGAGGAGGAGCACGGGTTCGCTTGTT	scaffold571918		
f6156	>ref-384445	AGTTTATTGACTGATTCTCCGTCTGTT	C41597631		
f4808	>ref-299732	TGAAACTCTACGGACGCTCCCGCCATC	scaffold68697		
f1406	>ref-85686	TCCCTCTCTACCCAGCCTCCGCCCCTG	scaffold937967		
f438	>ref-27928	TACTATCACACTATCTCTCCATTCTAA	scaffold1040560	uncharacterized protein LOC113811618	XP_027219204.1
f4820	>ref-300562	ACTATGTGTACTTGTTCTCCTGCGTTG	scaffold696622	uncharacterized LOC119585131, mRNA	XM_037933765.1
m2179	>ref-129380	AGAAGGAGGAGGCGTAGTAGGTGGATT	scaffold886981		

Supplementary Table S2 The detailed genotyping information of codominant SNPs in mapping family

Reference_Id	Reference_sequence	SNP_Loci	Marker Type	Geno-type	Number	Geno-type	Number	Geno-type	Number
>ref-14	AACTCACGCACTCTCTCTCCTAAATCT	2	<hkxhk>	hh	90	hk	164	kk	0
>ref-14	AACTCACGCACTCTCTCTCCTAAATCT	21	<lmxll>	ll	130	lm	127		
>ref-14	AACTCACGCACTCTCTCTCCTAAATCT	26	<lmxll>	ll	130	lm	127		
>ref-16	AATTGAATAACACGCGCTCCGTCTAAC	15	<nnxnp>	nn	108	np	130		
>ref-28	TCGCAGGGAACGGATGCTCCAAGCCCC	2	<lmxll>	ll	152	lm	115		
>ref-28	TCGCAGGGAACGGATGCTCCAAGCCCC	23	<hkxhk>	hh	2	hk	268	kk	0
>ref-39	GTATGGGGCACGGTGCCTCCGTGTGCA	21	<lmxll>	ll	132	lm	131		
>ref-56	TGTTTGAGCACTGTCTCTCCTGCATGA	15	<nnxnp>	nn	116	np	122		
>ref-72	CCTCCTCCAACACCACCTCCGAAGCCG	6	<lmxll>	ll	6	lm	265		
>ref-72	CCTCCTCCAACACCACCTCCGAAGCCG	14	<hkxhk>	hh	93	hk	166	kk	0
>ref-72	CCTCCTCCAACACCACCTCCGAAGCCG	23	<lmxll>	ll	7	lm	259		
>ref-72	CCTCCTCCAACACCACCTCCGAAGCCG	24	<lmxll>	ll	7	lm	259		
>ref-72	CCTCCTCCAACACCACCTCCGAAGCCG	26	<hkxhk>	hh	93	hk	162	kk	0
>ref-72	CCTCCTCCAACACCACCTCCGAAGCCG	27	<hkxhk>	hh	0	hk	268	kk	5
>ref-106	CCCTTCCTTACTTCTACTCCGTCCCCA	27	<lmxll>	ll	113	lm	127		
>ref-128	ATCCAAACCACTCCCTCTCCCCCCTGA	2	<hkxhk>	hh	0	hk	97	kk	151
>ref-128	ATCCAAACCACTCCCTCTCCCCCCTGA	3	<lmxll>	ll	127	lm	128		
>ref-128	ATCCAAACCACTCCCTCTCCCCCCTGA	8	<hkxhk>	hh	43	hk	212	kk	0
>ref-128	ATCCAAACCACTCCCTCTCCCCCCTGA	24	<lmxll>	ll	259	lm	9		
>ref-128	ATCCAAACCACTCCCTCTCCCCCCTGA	26	<nnxnp>	nn	218	np	48		
>ref-128	ATCCAAACCACTCCCTCTCCCCCCTGA	27	<nnxnp>	nn	219	np	47		
>ref-130	TTTCCCCTTACTTTTCCTCCTTCCTTT	2	<nnxnp>	nn	232	np	1		
>ref-130	TTTCCCCTTACTTTTCCTCCTTCCTTT	4	<nnxnp>	nn	230	np	1		
>ref-150	TATCCATCGACCCTTCCTCCTCCATTT	9	<hkxhk>	hh	50	hk	145	kk	41
>ref-156	TGATACAACACCACCTCTCCAACCTCA	2	<hkxhk>	hh	80	hk	178	kk	0
>ref-156	TGATACAACACCACCTCTCCAACCTCA	12	<hkxhk>	hh	45	hk	206	kk	0
>ref-156	TGATACAACACCACCTCTCCAACCTCA	26	<hkxhk>	hh	79	hk	180	kk	0
>ref-157	CAGATAGACACAAAACCTCCATAAATA	9	<nnxnp>	nn	117	np	138		
>ref-160	CTAACAGTTACTTCAGCTCCCAAGGAC	4	<nnxnp>	nn	247	np	0		
>ref-180	CACACGTACACACACCCTCCCACCTTC	6	<nnxnp>	nn	257	np	2		
>ref-180	CACACGTACACACACCCTCCCACCTTC	14	<hkxhk>	hh	54	hk	160	kk	41
>ref-188	TTCATCATGACTCCTCCTCCCTCTTCT	9	<hkxhk>	hh	37	hk	149	kk	59
>ref-188	TTCATCATGACTCCTCCTCCCTCTTCT	23	<hkxhk>	hh	58	hk	147	kk	40
>ref-191	TTCCACTCTACTTCCCCTCCCTTCCCC	4	<nnxnp>	nn	270	np	0		
>ref-191	TTCCACTCTACTTCCCCTCCCTTCCCC	5	<lmxll>	ll	0	lm	251		

>ref-191	TTCCACTCTACTTCCCCTCCCTTCCCC	24	<lmxll>	ll	1	lm	270		
>ref-198	CCCTCCTCCACTCCCACTCCTCGCAAA	12	<lmxll>	ll	134	lm	118		
>ref-216	ATATTCATTACTCCAACTCCTTACTTC	13	<hkxhk>	hh	48	hk	122	kk	52
>ref-237	TCCTCCTTCACTTCATCTCCTTCTTCC	3	<nnxnp>	nn	268	np	0		
>ref-248	TGGATCACCACCATCACTCCGCCAACA	1	<nnxnp>	nn	116	np	148		
>ref-269	CCACATCCAACCAGACCTCCAGATCTG	21	<nnxnp>	nn	251	np	0		
>ref-278	TCATCATCAACACCCTCTCCACCACAC	27	<hkxhk>	hh	66	hk	141	kk	60
>ref-279	TCTGACATGACTATCTCTCCCTATACA	21	<lmxll>	ll	125	lm	121		
>ref-299	GCAGCTATTACCGCATCTCCGCTACTG	12	<nnxnp>	nn	125	np	137		
>ref-310	CCTGCAGGAACTCCGGCTCCGCCTCCT	16	<lmxll>	ll	146	lm	122		
>ref-357	AGAAACCCTACACCATCTCCACCGACA	22	<nnxnp>	nn	261	np	0		
>ref-373	CCTTCCCTTACCTCCCCTCCCATCTTC	21	<nnxnp>	nn	265	np	3		
>ref-373	CCTTCCCTTACCTCCCCTCCCATCTTC	22	<nnxnp>	nn	58	np	183		
>ref-374	ATATCTACCACTCCCTCTCCCTCTTAC	26	<nnxnp>	nn	107	np	127		
>ref-374	ATATCTACCACTCCCTCTCCCTCTTAC	27	<nnxnp>	nn	109	np	127		
>ref-393	CCCCCTCCTACTTCCCCTCCCCCTCCC	3	<hkxhk>	hh	0	hk	268	kk	0
>ref-393	CCCCCTCCTACTTCCCCTCCCCCTCCC	13	<hkxhk>	hh	0	hk	271	kk	0
>ref-393	CCCCCTCCTACTTCCCCTCCCCCTCCC	14	<lmxll>	ll	258	lm	11		
>ref-393	CCCCCTCCTACTTCCCCTCCCCCTCCC	15	<hkxhk>	hh	0	hk	268	kk	0
>ref-393	CCCCCTCCTACTTCCCCTCCCCCTCCC	21	<hkxhk>	hh	3	hk	268	kk	0
>ref-393	CCCCCTCCTACTTCCCCTCCCCCTCCC	27	<hkxhk>	hh	54	hk	213	kk	0
>ref-396	TCCTGTGTCACTCGGCCTCCAAAGTGA	13	<lmxll>	ll	120	lm	139		
>ref-409	CACAAGCATACACGAGCTCCCTCCTCC	26	<lmxll>	ll	135	lm	102		
>ref-452	ACACAGTCCACCCTCCCTCCGACCTGA	13	<lmxll>	ll	26	lm	233		
>ref-452	ACACAGTCCACCCTCCCTCCGACCTGA	14	<lmxll>	ll	25	lm	233		
>ref-483	CGAGTCCTTACTCATCCTCCTCCGGCC	8	<hkxhk>	hh	0	hk	262	kk	1
>ref-489	ATGAACCCCACTCCACCTCCCACCTCT	13	<lmxll>	ll	143	lm	124		
>ref-490	CTGACGAACACACGTCCTCCGTAGCAC	5	<lmxll>	ll	151	lm	109		
>ref-499	TTGAAGGGAACATCACCTCCTTTCTAG	16	<hkxhk>	hh	121	hk	135	kk	0
>ref-502	CCGCCCAGTACGGGGGCTCCCCCACCA	25	<hkxhk>	hh	1	hk	265	kk	4
>ref-515	TCTTCCCTCACCCTCCCTCCATCCCCT	1	<lmxll>	ll	230	lm	21		
>ref-515	TCTTCCCTCACCCTCCCTCCATCCCCT	9	<lmxll>	ll	268	lm	0		
>ref-515	TCTTCCCTCACCCTCCCTCCATCCCCT	21	<lmxll>	ll	14	lm	245		
>ref-515	TCTTCCCTCACCCTCCCTCCATCCCCT	24	<lmxll>	ll	268	lm	0		
>ref-517	AGCTACTTCACTTACACTCCTGCTATA	2	<hkxhk>	hh	4	hk	256	kk	5
>ref-517	AGCTACTTCACTTACACTCCTGCTATA	21	<hkxhk>	hh	4	hk	256	kk	5
>ref-519	GGGAGTGTGACCGCTACTCCCGTGGCA	1	<hkxhk>	hh	0	hk	256	kk	13
>ref-519	GGGAGTGTGACCGCTACTCCCGTGGCA	14	<hkxhk>	hh	75	hk	189	kk	0

>ref-519	GGGAGTGTGACCGCTACTCCCGTGGCA	22	<hkxhk>	hh	0	hk	272	kk	0
>ref-522	TGCATGAGAACCCCCCCTCCCCCCTCC	1	<hkxhk>	hh	0	hk	241	kk	7
>ref-522	TGCATGAGAACCCCCCCTCCCCCCTCC	8	<hkxhk>	hh	0	hk	242	kk	7
>ref-535	CCACGCCACACACGCACTCCCACACCC	6	<lmxll>	ll	127	lm	140		
>ref-540	GATCCAAGGACGGAGGCTCCTGCACAG	23	<hkxhk>	hh	3	hk	259	kk	0
>ref-540	GATCCAAGGACGGAGGCTCCTGCACAG	26	<hkxhk>	hh	3	hk	259	kk	0
>ref-583	CTGGCTTGAACCGCCGCTCCGAAAGGC	5	<nnxnp>	nn	111	np	138		
>ref-608	GATACTCACACCCATGCTCCCAGGAGT	9	<lmxll>	ll	130	lm	122		
>ref-609	GCACAATAAACGCCTCCTCCTTTGAAA	7	<lmxll>	ll	109	lm	117		
>ref-614	TTCGTCAGTACAACCTCTCCCATGTTC	14	<lmxll>	ll	142	lm	124		
>ref-631	TAAACACCTACTCTCTCTCCCTTCTCT	25	<nnxnp>	nn	130	np	117		
>ref-637	CCCCCCTTCACTTCTTCTCCCCTATTC	1	<nnxnp>	nn	220	np	1		
>ref-637	CCCCCCTTCACTTCTTCTCCCCTATTC	2	<nnxnp>	nn	225	np	0		
>ref-639	GCATCAATCACCAATTCTCCTTCCCCC	1	<nnxnp>	nn	147	np	103		
>ref-642	GAAGATATCACCATCTCTCCCTCTCAC	5	<nnxnp>	nn	267	np	0		
>ref-658	CAAGAGCACACAATGTCTCCAAATATA	13	<lmxll>	ll	124	lm	136		
>ref-668	ACAGCCCCAACACCTTCTCCTCTCCCT	3	<lmxll>	ll	233	lm	29		
>ref-668	ACAGCCCCAACACCTTCTCCTCTCCCT	16	<lmxll>	ll	202	lm	55		
>ref-670	CCTCCTGATACAACACCTCCTCCCCAA	4	<hkxhk>	hh	14	hk	246	kk	5
>ref-670	CCTCCTGATACAACACCTCCTCCCCAA	5	<hkxhk>	hh	6	hk	245	kk	12
>ref-670	CCTCCTGATACAACACCTCCTCCCCAA	7	<hkxhk>	hh	5	hk	246	kk	14
>ref-670	CCTCCTGATACAACACCTCCTCCCCAA	8	<hkxhk>	hh	13	hk	246	kk	5
>ref-670	CCTCCTGATACAACACCTCCTCCCCAA	9	<hkxhk>	hh	5	hk	247	kk	13
>ref-670	CCTCCTGATACAACACCTCCTCCCCAA	13	<hkxhk>	hh	12	hk	248	kk	5
>ref-670	CCTCCTGATACAACACCTCCTCCCCAA	21	<hkxhk>	hh	5	hk	247	kk	11
>ref-670	CCTCCTGATACAACACCTCCTCCCCAA	23	<hkxhk>	hh	5	hk	246	kk	14
>ref-670	CCTCCTGATACAACACCTCCTCCCCAA	24	<hkxhk>	hh	5	hk	245	kk	14
>ref-670	CCTCCTGATACAACACCTCCTCCCCAA	25	<hkxhk>	hh	5	hk	248	kk	14
>ref-681	ACCTCTATAACTGTCTCTCCCTTGAAC	21	<hkxhk>	hh	48	hk	149	kk	51
>ref-730	CATATTGACACTATCTCTCCTCAGTCT	22	<lmxll>	ll	135	lm	124		
>ref-754	CTCTCCCCCACTCTGTCTCCTCTATCC	3	<nnxnp>	nn	110	np	125		
>ref-757	ATACAATTCACATCCTCTCCGTTTATA	12	<lmxll>	ll	95	lm	128		
>ref-814	ATACCACCCACAGCCCCTCCTTTTTCC	6	<nnxnp>	nn	138	np	129		
>ref-828	CATTTCACCACCCTCCCTCCACCCCTC	5	<lmxll>	ll	120	lm	129		
>ref-837	TCGCCACCCACCTCCACTCCCTGAGAA	7	<nnxnp>	nn	131	np	106		
>ref-847	CCCCTTCCTACCTATACTCCTTCCCCT	8	<nnxnp>	nn	108	np	152		
>ref-847	CCCCTTCCTACCTATACTCCTTCCCCT	12	<nnxnp>	nn	103	np	154		
>ref-873	CCCATCTGTACTCCCCCTCCCCCTCTC	23	<nnxnp>	nn	120	np	110		

>ref-883	AATACGAAAACATTAACTCCTTCACCA	8	<lmxll>	ll	110	lm	144		
>ref-884	CCTCCTATCACCTCTTCTCCAATACCG	26	<nnxnp>	nn	130	np	134		
>ref-898	TTCCCCTCCACTGTCCCTCCCTCCCAT	13	<lmxll>	ll	220	lm	5		
>ref-898	TTCCCCTCCACTGTCCCTCCCTCCCAT	25	<lmxll>	ll	244	lm	0		
>ref-898	TTCCCCTCCACTGTCCCTCCCTCCCAT	26	<lmxll>	ll	232	lm	3		
>ref-899	CGACCGCAGACACGCCCTCCGCTCCAG	6	<lmxll>	ll	136	lm	137		
>ref-907	CAATCTCCTACCTTCCCTCCCCTATTT	8	<nnxnp>	nn	117	np	134		
>ref-918	GTCAACCCTACCACTCCTCCTCCTCCT	22	<lmxll>	ll	262	lm	0		
>ref-928	CTCGTACTCACTCAAGCTCCCTTCTAT	15	<hkxhk>	hh	64	hk	140	kk	55
>ref-958	TCTTTCGCCACACACTCTCCGTTGTAA	14	<lmxll>	ll	142	lm	124		
>ref-975	CTTTACCCCACTTTTCCTCCATTCTTC	1	<nnxnp>	nn	237	np	1		
>ref-984	CACACACAGACACTAACTCCCGACCAC	9	<lmxll>	ll	129	lm	140		
>ref-998	CTCCCCTAAACGACCTCTCCTGCTGAG	6	<nnxnp>	nn	122	np	109		
>ref-1004	CTCAAGCGTACCCCCTCTCCATTCCCC	3	<nnxnp>	nn	132	np	134		
>ref-1024	ATGTTTCCTACTCTTCCTCCCCTTCAC	1	<hkxhk>	hh	72	hk	190	kk	0
>ref-1024	ATGTTTCCTACTCTTCCTCCCCTTCAC	9	<lmxll>	ll	142	lm	125		
>ref-1024	ATGTTTCCTACTCTTCCTCCCCTTCAC	16	<hkxhk>	hh	72	hk	189	kk	0
>ref-1030	TTTTCTCTCACTCACTCTCCCCCGCCC	4	<nnxnp>	nn	134	np	127		
>ref-1030	TTTTCTCTCACTCACTCTCCCCCGCCC	15	<nnxnp>	nn	134	np	127		
>ref-1053	AAGACCCAGACCCACTCTCCCTGACTG	14	<nnxnp>	nn	113	np	154		
>ref-1076	TTCACATGCACCCACCCTCCAAGCCAC	26	<lmxll>	ll	136	lm	122		
>ref-1091	TGTACAATAACGAAGCCTCCATTTATA	1	<hkxhk>	hh	44	hk	126	kk	69
>ref-1091	TGTACAATAACGAAGCCTCCATTTATA	12	<hkxhk>	hh	70	hk	125	kk	44
>ref-1098	TACCAACCAACCACCTCTCCCTCTCTC	1	<lmxll>	ll	115	lm	149		
>ref-1115	TGAAGTGGCACGGGGCCTCCCTAAAGA	25	<nnxnp>	nn	251	np	0		
>ref-1115	TGAAGTGGCACGGGGCCTCCCTAAAGA	26	<nnxnp>	nn	251	np	0		
>ref-1120	ATGCAATTAACAGCCTCTCCCGTACAA	4	<lmxll>	ll	192	lm	69		
>ref-1120	ATGCAATTAACAGCCTCTCCCGTACAA	16	<lmxll>	ll	137	lm	133		
>ref-1120	ATGCAATTAACAGCCTCTCCCGTACAA	25	<lmxll>	ll	192	lm	69		
>ref-1121	CATAATATTACTTCTCCTCCTCCTCAT	24	<lmxll>	ll	121	lm	101		
>ref-1155	TCTCCCCTCACTTTCCCTCCCATTCGT	7	<nnxnp>	nn	120	np	142		
>ref-1165	ATGTACCACACAATCCCTCCAAAGATA	4	<hkxhk>	hh	0	hk	271	kk	0
>ref-1165	ATGTACCACACAATCCCTCCAAAGATA	15	<hkxhk>	hh	0	hk	271	kk	0
>ref-1168	TCCCTCCCTACCCTCCCTCCCCCTTTG	1	<nnxnp>	nn	11	np	244		
>ref-1168	TCCCTCCCTACCCTCCCTCCCCCTTTG	15	<lmxll>	ll	4	lm	253		
>ref-1168	TCCCTCCCTACCCTCCCTCCCCCTTTG	22	<lmxll>	ll	4	lm	253		
>ref-1168	TCCCTCCCTACCCTCCCTCCCCCTTTG	25	<nnxnp>	nn	10	np	244		
>ref-1187	AACTTTCTAACCGTGGCTCCACGTATC	3	<lmxll>	ll	130	lm	98		

>ref-1192	TCCCCTTACACCATATCTCCCTTATCC	4	<nnxnp>	nn	146	np	108		
>ref-1201	ACTACAGCTACAAGTCCTCCTCCTCCT	7	<lmxll>	ll	119	lm	138		
>ref-1207	AACACACGTACTTGAACTCCGCCATTA	7	<lmxll>	ll	128	lm	139		
>ref-1218	CTTCCTCTTACACCTGCTCCTTCCTCT	6	<lmxll>	ll	255	lm	0		
>ref-1218	CTTCCTCTTACACCTGCTCCTTCCTCT	16	<lmxll>	ll	218	lm	7		
>ref-1223	TTCCTCCCTACTCCCCCTCCCCCCTTA	9	<lmxll>	ll	193	lm	63		
>ref-1264	TATATATGTACACTTCCTCCTCTATCT	24	<lmxll>	ll	111	lm	120		
>ref-1264	TATATATGTACACTTCCTCCTCTATCT	26	<lmxll>	ll	109	lm	122		
>ref-1267	CCTCCTATTACACACCCTCCTTCTCCC	23	<nnxnp>	nn	141	np	114		
>ref-1285	ATGTATGACACGTTGACTCCGTAATCA	15	<nnxnp>	nn	135	np	131		
>ref-1307	CCCCCACACACATACCCTCCCTCCCTT	8	<hkxhk>	hh	2	hk	264	kk	0
>ref-1307	CCCCCACACACATACCCTCCCTCCCTT	21	<hkxhk>	hh	2	hk	264	kk	0
>ref-1321	TTCCACCACACCCACTCTCCGCCCACA	7	<lmxll>	ll	120	lm	128		
>ref-1328	AGAATATGCACTGACACTCCCACTCAT	6	<nnxnp>	nn	117	np	133		
>ref-1356	TCCTTATCAACTCCCTCTCCCCCCCTC	3	<nnxnp>	nn	52	np	180		
>ref-1356	TCCTTATCAACTCCCTCTCCCCCCCTC	14	<nnxnp>	nn	52	np	181		
>ref-1364	AATCTCCTTACCTCCCCTCCCTTTCTA	21	<hkxhk>	hh	74	hk	124	kk	40
>ref-1371	ATTGTTCATACAGATACTCCAACATCT	5	<lmxll>	ll	102	lm	144		
>ref-1404	CCCTCTCTGACCCTCCCTCCTATCACC	15	<lmxll>	ll	115	lm	121		
>ref-1405	TTTGTGCTCACCGAACCTCCCTTCTTT	6	<lmxll>	ll	143	lm	120		
>ref-1410	CTCCTGCCAACATCCCCTCCCCTTCCC	7	<nnxnp>	nn	163	np	88		
>ref-1410	CTCCTGCCAACATCCCCTCCCCTTCCC	22	<hkxhk>	hh	0	hk	200	kk	59
>ref-1418	TTTTCCCTTACTATCTCTCCATCTCTC	13	<nnxnp>	nn	72	np	152		
>ref-1418	TTTTCCCTTACTATCTCTCCATCTCTC	25	<nnxnp>	nn	77	np	150		
>ref-1425	TCCCCTTACACTGTGCCTCCACTGTGT	15	<nnxnp>	nn	266	np	0		
>ref-1430	GGCATTTGCACGGCTTCTCCTCTCCCT	2	<lmxll>	ll	136	lm	132		
>ref-1430	GGCATTTGCACGGCTTCTCCTCTCCCT	9	<lmxll>	ll	136	lm	132		
>ref-1472	CTCTCATTCACTTCCCCTCCTCTTTTT	13	<lmxll>	ll	140	lm	94		
>ref-1472	CTCTCATTCACTTCCCCTCCTCTTTTT	25	<nnxnp>	nn	251	np	1		
>ref-1520	TGTCCTCCCACTCCTCCTCCGCCTCCG	1	<lmxll>	ll	32	lm	224		
>ref-1520	TGTCCTCCCACTCCTCCTCCGCCTCCG	2	<lmxll>	ll	21	lm	227		
>ref-1520	TGTCCTCCCACTCCTCCTCCGCCTCCG	21	<nnxnp>	nn	215	np	25		
>ref-1520	TGTCCTCCCACTCCTCCTCCGCCTCCG	27	<nnxnp>	nn	224	np	17		
>ref-1526	GAATTATAAACCGAGGCTCCTCATGAT	27	<hkxhk>	hh	50	hk	141	kk	70
>ref-1530	AGTAGTATCACTACTGCTCCTGTTATG	27	<nnxnp>	nn	129	np	114		
>ref-1550	GGAGGCGTGACGTCATCTCCCTGACCT	3	<hkxhk>	hh	59	hk	151	kk	55
>ref-1561	ATGGTGATAACGCTGCCTCCACCACCC	1	<nnxnp>	nn	127	np	134		
>ref-1561	ATGGTGATAACGCTGCCTCCACCACCC	27	<lmxll>	ll	137	lm	126		

>ref-1562	CTGCCTTCCACGTTTACTCCCTCTTAT	12	<lmxll>	ll	126	lm	115		
>ref-1577	TCCGGTCTCACCCTTACTCCTGCCCCA	3	<lmxll>	ll	109	lm	149		
>ref-1578	CACATAAACACAGACTCTCCCTCTTTC	7	<nnxnp>	nn	128	np	138		
>ref-1580	CTCATCCCAACCATCCCTCCCTCTCCC	9	<hkxhk>	hh	76	hk	184	kk	0
>ref-1580	CTCATCCCAACCATCCCTCCCTCTCCC	12	<lmxll>	ll	137	lm	129		
>ref-1580	CTCATCCCAACCATCCCTCCCTCTCCC	13	<lmxll>	ll	126	lm	132		
>ref-1580	CTCATCCCAACCATCCCTCCCTCTCCC	21	<lmxll>	ll	143	lm	118		
>ref-1580	CTCATCCCAACCATCCCTCCCTCTCCC	24	<hkxhk>	hh	0	hk	182	kk	82
>ref-1585	CTTTGCTTCACACTCTCTCCATCCCTC	4	<hkxhk>	hh	0	hk	129	kk	124
>ref-1590	CACCCTTCAACTGCTCCTCCTGCTCAT	22	<nnxnp>	nn	134	np	134		
>ref-1599	ACGCCTTCCACTTGGACTCCCGATTGT	27	<hkxhk>	hh	0	hk	270	kk	1
>ref-1688	CGAGGTTTTACACACACTCCCTGGGAC	22	<hkxhk>	hh	0	hk	240	kk	21
>ref-1688	CGAGGTTTTACACACACTCCCTGGGAC	26	<hkxhk>	hh	33	hk	222	kk	0
>ref-1689	ATCATCACCACCTTCTCTCCCTCCTCC	22	<hkxhk>	hh	2	hk	196	kk	70
>ref-1693	TGATTAGAAACGTTTGCTCCAAGTGCC	27	<nnxnp>	nn	122	np	136		
>ref-1694	ATCGGCTGGACGGGTTCTCCTAACAGT	5	<lmxll>	ll	26	lm	216		
>ref-1694	ATCGGCTGGACGGGTTCTCCTAACAGT	9	<lmxll>	ll	52	lm	187		
>ref-1703	CTTCCCCACACTTATTCTCCCTCTCTC	2	<lmxll>	ll	158	lm	86		
>ref-1703	CTTCCCCACACTTATTCTCCCTCTCTC	9	<lmxll>	ll	158	lm	87		
>ref-1714	CCAGAATACACACAGCCTCCCCTGGTA	25	<lmxll>	ll	132	lm	140		
>ref-1747	CTTATTCCCACATTCCCTCCCTATCTC	2	<lmxll>	ll	113	lm	148		
>ref-1747	CTTATTCCCACATTCCCTCCCTATCTC	3	<lmxll>	ll	113	lm	148		
>ref-1781	TCACATCCCACCTTACCTCCTTCCCTT	25	<lmxll>	ll	134	lm	130		
>ref-1784	ATTGACAGGACATTCTCTCCCTCTCAG	15	<nnxnp>	nn	136	np	128		
>ref-1793	CAGCCTTCAACAGCGTCTCCTACCAAC	22	<lmxll>	ll	125	lm	144		
>ref-1794	ACTATTGACACGATTCCTCCTGCCACA	1	<hkxhk>	hh	50	hk	139	kk	67
>ref-1797	AATGCTCCCACATTCCCTCCCTCCACC	12	<nnxnp>	nn	266	np	0		
>ref-1797	AATGCTCCCACATTCCCTCCCTCCACC	21	<nnxnp>	nn	267	np	0		
>ref-1802	CATATATTTACTCTTTCTCCCATCTAT	2	<hkxhk>	hh	85	hk	142	kk	1
>ref-1802	CATATATTTACTCTTTCTCCCATCTAT	6	<hkxhk>	hh	9	hk	226	kk	12
>ref-1802	CATATATTTACTCTTTCTCCCATCTAT	16	<nnxnp>	nn	16	np	217		
>ref-1813	TACAGAGGCACCCCCGCTCCCCTCTCT	15	<hkxhk>	hh	63	hk	207	kk	0
>ref-1821	TGTTTATTCACATTTCCTCCCTCCCAA	24	<lmxll>	ll	1	lm	259		
>ref-1821	TGTTTATTCACATTTCCTCCCTCCCAA	27	<lmxll>	ll	1	lm	260		
>ref-1829	ATCTCACGCACACACACTCCTGAACTC	26	<nnxnp>	nn	153	np	102		
>ref-1845	TTTGTTCGCACAGATCCTCCTCCGCAG	8	<nnxnp>	nn	130	np	128		
>ref-1877	ATCCCTCCCACTCTCTCTCCCTTTGTA	7	<lmxll>	ll	60	lm	202		
>ref-1877	ATCCCTCCCACTCTCTCTCCCTTTGTA	15	<lmxll>	ll	59	lm	202		

>ref-1886	CAGACACTTACACTCACTCCCTCCCTT	22	<hkxhk>	hh	1	hk	201	kk	52
>ref-1894	ACAACAGCGACGAACCCTCCTCGTGCT	4	<nnxnp>	nn	264	np	0		
>ref-1894	ACAACAGCGACGAACCCTCCTCGTGCT	25	<nnxnp>	nn	263	np	0		
>ref-1900	TAGGACACCACTTACGCTCCGTAAGTG	14	<nnxnp>	nn	257	np	1		
>ref-1905	AATCAGCGCACGAGATCTCCTAAGCTA	16	<hkxhk>	hh	68	hk	147	kk	52
>ref-1910	GGAAAGATTACTGTCCCTCCTCCTTAT	22	<nnxnp>	nn	116	np	130		
>ref-1913	TTTCCTCTCACCCTTTCTCCTCTCTTT	1	<hkxhk>	hh	3	hk	238	kk	8
>ref-1913	TTTCCTCTCACCCTTTCTCCTCTCTTT	14	<hkxhk>	hh	3	hk	236	kk	8
>ref-1925	CCCCCTAATACTCCCCCTCCATCCTGC	4	<lmxll>	ll	137	lm	118		
>ref-1939	AGATATCAGACGCTTCCTCCCGAACAC	22	<nnxnp>	nn	122	np	125		
>ref-1940	TCTCCTCTCACTCACCCTCCTTCTCAT	2	<lmxll>	ll	259	lm	0		
>ref-1940	TCTCCTCTCACTCACCCTCCTTCTCAT	4	<lmxll>	ll	258	lm	0		
>ref-1940	TCTCCTCTCACTCACCCTCCTTCTCAT	5	<lmxll>	ll	259	lm	0		
>ref-1958	CCCTCCCCCACCCCGTCTCCCTCGCCA	1	<lmxll>	ll	243	lm	1		
>ref-1958	CCCTCCCCCACCCCGTCTCCCTCGCCA	9	<lmxll>	ll	249	lm	0		
>ref-1958	CCCTCCCCCACCCCGTCTCCCTCGCCA	14	<lmxll>	ll	237	lm	5		
>ref-1974	ATCGAAAACACAGTTACTCCTAAAGCA	27	<lmxll>	ll	138	lm	102		
>ref-1978	AAGGGTGAAACTGTCGCTCCTGATACA	5	<nnxnp>	nn	127	np	140		
>ref-1989	TTATTAAATACATTCTCTCCTTCTCTC	21	<nnxnp>	nn	125	np	111		
>ref-2007	AGCTCTCTCACCCTCACTCCTCATGCA	15	<nnxnp>	nn	133	np	131		
>ref-2030	TTTCCCTTTACGGACACTCCTCCTACG	13	<lmxll>	ll	102	lm	135		
>ref-2045	CATTCACTCACTCTTCCTCCGAGCTGC	21	<nnxnp>	nn	127	np	131		
>ref-2064	TCTCCCTTTACGTACTCTCCCTTTGCC	3	<hkxhk>	hh	0	hk	271	kk	2
>ref-2064	TCTCCCTTTACGTACTCTCCCTTTGCC	25	<hkxhk>	hh	0	hk	271	kk	2
>ref-2080	TCCCGTCTTACCCCATCTCCTCCTTCA	5	<nnxnp>	nn	110	np	148		
>ref-2095	TCCTTCTCTACGCTCTCTCCCTTCCTT	21	<nnxnp>	nn	128	np	137		
>ref-2103	ATGCGGATAACTCTATCTCCCTGTCTC	15	<lmxll>	ll	118	lm	137		
>ref-2107	GAGCTATTCACCCAACCTCCCCCACCC	6	<nnxnp>	nn	132	np	104		
>ref-2115	GTGTAAGTAACAATAACTCCTTTATGA	25	<nnxnp>	nn	251	np	0		
>ref-2119	AGTAATAACACGGCTTCTCCTTGGAGT	27	<lmxll>	ll	129	lm	123		
>ref-2120	TCTCGTTTTACCGCCTCTCCCCATTCT	22	<hkxhk>	hh	61	hk	137	kk	59
>ref-2133	ATATATAATACAGATGCTCCTTCTTCT	24	<nnxnp>	nn	116	np	128		
>ref-2165	CCTTTCTTCACTCCCCCTCCTCCATCC	9	<lmxll>	ll	35	lm	191		
>ref-2165	CCTTTCTTCACTCCCCCTCCTCCATCC	12	<lmxll>	ll	35	lm	191		
>ref-2175	TCAACATATACAGACACTCCATTTGTT	1	<lmxll>	ll	118	lm	124		
>ref-2184	TGCTGTCTGACTCTTCCTCCTCCTCCC	9	<lmxll>	ll	235	lm	0		
>ref-2184	TGCTGTCTGACTCTTCCTCCTCCTCCC	24	<lmxll>	ll	246	lm	0		
>ref-2260	ATGTACTCCACCCTTCCTCCTCTTTCT	4	<nnxnp>	nn	263	np	0		

>ref-2260	ATGTACTCCACCCTTCCTCCTCTTTCT	25	<nnxnp>	nn	259	np	2		
>ref-2263	GGAGTCCTTACACAATCTCCGTCTCTC	12	<lmxll>	ll	118	lm	144		
>ref-2273	CCCGGTAACACTGTCACTCCGCATAAC	21	<nnxnp>	nn	135	np	130		
>ref-2278	ATCCCTCCTACTCGATCTCCCTCCCCC	8	<nnxnp>	nn	121	np	125		
>ref-2278	ATCCCTCCTACTCGATCTCCCTCCCCC	14	<nnxnp>	nn	119	np	125		
>ref-2289	CCTACCTCTACCCCTCCTCCCTCATTC	1	<nnxnp>	nn	15	np	242		
>ref-2289	CCTACCTCTACCCCTCCTCCCTCATTC	4	<nnxnp>	nn	14	np	243		
>ref-2297	TTTGAACGCACACGAGCTCCTCCCTCG	7	<hkxhk>	hh	62	hk	132	kk	57
>ref-2360	GCCCACGTAACTGAACCTCCCCCCATC	26	<nnxnp>	nn	254	np	0		
>ref-2374	CACACCTATACAGACGCTCCTCCATTT	23	<hkxhk>	hh	73	hk	136	kk	53
>ref-2401	ATCTATCCTACAGTCGCTCCACACCAA	4	<nnxnp>	nn	115	np	143		
>ref-2408	ATAATCAGTACTTCCCCTCCGCCATCA	3	<hkxhk>	hh	64	hk	202	kk	0
>ref-2408	ATAATCAGTACTTCCCCTCCGCCATCA	27	<hkxhk>	hh	64	hk	201	kk	0
>ref-2412	CAGCAGTTCACTGAGTCTCCTTTGAAC	2	<hkxhk>	hh	0	hk	273	kk	0
>ref-2423	GAAAGTAACACGCTTGCTCCGCGACAC	25	<lmxll>	ll	109	lm	122		
>ref-2436	AACCCTGTCACTGCTTCTCCTCTGTCA	22	<hkxhk>	hh	46	hk	152	kk	66
>ref-2437	ACTGTCGTGACGACGGCTCCAAATATA	24	<hkxhk>	hh	34	hk	117	kk	78
>ref-2442	CCTACGCATACCCTGTCTCCATCACCC	27	<nnxnp>	nn	95	np	139		
>ref-2444	CGGTTTTGCACTCCCTCTCCCTCTCAT	6	<nnxnp>	nn	122	np	127		
>ref-2444	CGGTTTTGCACTCCCTCTCCCTCTCAT	12	<nnxnp>	nn	121	np	127		
>ref-2456	TAAGCATAAACAGTAACTCCTGATGAA	24	<lmxll>	ll	128	lm	131		
>ref-2471	GGGGTCTCCACCTTTCCTCCCCCTTTT	27	<nnxnp>	nn	132	np	132		
>ref-2478	TCTTGGTCCACCTCTCCTCCCCTCTTT	26	<lmxll>	ll	125	lm	135		
>ref-2484	CCCTCCCCCACCCCCTCTCCATGTGTG	25	<lmxll>	ll	13	lm	231		
>ref-2484	CCCTCCCCCACCCCCTCTCCATGTGTG	26	<lmxll>	ll	13	lm	231		
>ref-2488	CCTATAGTGACACTTCCTCCTTGAACA	1	<nnxnp>	nn	139	np	106		
>ref-2488	CCTATAGTGACACTTCCTCCTTGAACA	7	<lmxll>	ll	254	lm	11		
>ref-2493	TCTCAACATACAGGGACTCCACAGTGA	6	<hkxhk>	hh	1	hk	272	kk	0
>ref-2493	TCTCAACATACAGGGACTCCACAGTGA	9	<hkxhk>	hh	0	hk	273	kk	0
>ref-2493	TCTCAACATACAGGGACTCCACAGTGA	23	<hkxhk>	hh	1	hk	272	kk	0
>ref-2493	TCTCAACATACAGGGACTCCACAGTGA	26	<hkxhk>	hh	0	hk	273	kk	0
>ref-2502	TCTCTTCCCACTTACTCTCCCTCCTCC	5	<lmxll>	ll	40	lm	194		
>ref-2502	TCTCTTCCCACTTACTCTCCCTCCTCC	14	<lmxll>	ll	27	lm	201		
>ref-2515	CCCTCACATACACCCCCTCCCCCTTTC	2	<lmxll>	ll	3	lm	254		
>ref-2515	CCCTCACATACACCCCCTCCCCCTTTC	5	<lmxll>	ll	3	lm	253		
>ref-2564	TATTCCATTACGCGGACTCCGTCGCAC	8	<nnxnp>	nn	115	np	142		
>ref-2576	ACCAACCAAACAAGAACTCCATTTAAC	6	<lmxll>	ll	125	lm	131		
>ref-2620	GCGCTGAGCACCGACTCTCCTCTGACT	2	<nnxnp>	nn	0	np	273		

>ref-2620	GCGCTGAGCACCGACTCTCCTCTGACT	12	<nnxnp>	nn	0	np	273		
>ref-2620	GCGCTGAGCACCGACTCTCCTCTGACT	14	<nnxnp>	nn	134	np	137		
>ref-2626	AACCACACAACCACAACTCCATTCTCT	12	<lmxll>	ll	112	lm	148		
>ref-2632	ACCTTGCGTACAATTACTCCTGTACAA	9	<nnxnp>	nn	122	np	109		
>ref-2636	CCTCTTTTCACTTCTTCTCCCCTCTCC	2	<lmxll>	ll	61	lm	178		
>ref-2636	CCTCTTTTCACTTCTTCTCCCCTCTCC	15	<nnxnp>	nn	268	np	1		
>ref-2636	CCTCTTTTCACTTCTTCTCCCCTCTCC	24	<nnxnp>	nn	272	np	0		
>ref-2636	CCTCTTTTCACTTCTTCTCCCCTCTCC	26	<lmxll>	ll	61	lm	178		
>ref-2655	TCCCCATCCACCCTCCCTCCCCCTGTC	6	<lmxll>	ll	240	lm	1		
>ref-2655	TCCCCATCCACCCTCCCTCCCCCTGTC	25	<lmxll>	ll	223	lm	13		
>ref-2675	TCCCTTCCTACTCTCACTCCCTCTTCC	1	<nnxnp>	nn	255	np	1		
>ref-2675	TCCCTTCCTACTCTCACTCCCTCTTCC	9	<nnxnp>	nn	258	np	1		
>ref-2679	CTAACAATAACACAGCCTCCTCTGATT	1	<hkxhk>	hh	66	hk	138	kk	51
>ref-2697	CCATCACCCACCCTTCCTCCCTTCCCC	5	<lmxll>	ll	102	lm	137		
>ref-2697	CCATCACCCACCCTTCCTCCCTTCCCC	8	<hkxhk>	hh	77	hk	186	kk	1
>ref-2697	CCATCACCCACCCTTCCTCCCTTCCCC	24	<lmxll>	ll	103	lm	138		
>ref-2701	CGAACGTTCACACCGCCTCCCGCCACA	27	<nnxnp>	nn	138	np	132		
>ref-2708	CCTCTCTCCACCCCTTCTCCTACTCCC	7	<hkxhk>	hh	0	hk	273	kk	0
>ref-2708	CCTCTCTCCACCCCTTCTCCTACTCCC	12	<hkxhk>	hh	0	hk	273	kk	0
>ref-2708	CCTCTCTCCACCCCTTCTCCTACTCCC	21	<hkxhk>	hh	0	hk	193	kk	70
>ref-2725	CACTCTTACACACCTACTCCTTTCGCT	2	<lmxll>	ll	119	lm	118		
>ref-2725	CACTCTTACACACCTACTCCTTTCGCT	7	<lmxll>	ll	120	lm	117		
>ref-2728	TGGAACCAGACTGCCGCTCCCTCATTC	24	<nnxnp>	nn	130	np	127		
>ref-2733	ACTACTACTACTACACCTCCATACCAC	26	<hkxhk>	hh	45	hk	139	kk	54
>ref-2733	ACTACTACTACTACACCTCCATACCAC	27	<hkxhk>	hh	54	hk	139	kk	45
>ref-2742	CCTCTCTTTACTCTCTCTCCCTCAGTC	21	<lmxll>	ll	120	lm	140		
>ref-2821	ATCACGCCCACTTCTCCTCCTGTCTCC	9	<nnxnp>	nn	127	np	132		
>ref-2832	TCTCTTTCTACCACGTCTCCCCTTACG	15	<lmxll>	ll	119	lm	146		
>ref-2838	TCCCTTTTCACTCCGTCTCCTCATCCT	4	<nnxnp>	nn	10	np	256		
>ref-2838	TCCCTTTTCACTCCGTCTCCTCATCCT	15	<nnxnp>	nn	10	np	256		
>ref-2839	AAATCCATCACCACTACTCCATGCTGG	23	<nnxnp>	nn	220	np	41		
>ref-2839	AAATCCATCACCACTACTCCATGCTGG	26	<lmxll>	ll	212	lm	45		
>ref-2845	TCCATCCTCACCCCCGCTCCGTCTGCT	5	<nnxnp>	nn	136	np	133		
>ref-2850	ATTCCCCAAACTCTCCCTCCCTCCCCC	2	<nnxnp>	nn	184	np	58		
>ref-2856	GTGCCTCGAACCAGCGCTCCCTCCTAT	2	<nnxnp>	nn	175	np	69		
>ref-2882	CAACCCACGACAGCTACTCCTCCCCAC	22	<lmxll>	ll	149	lm	119		
>ref-2883	CTTCCTTACACCTCCCCTCCTCTCTCA	25	<nnxnp>	nn	66	np	198		
>ref-2886	AACCTCTCTACCCGGCCTCCCTGCCCA	14	<lmxll>	ll	112	lm	157		

>ref-2894	ACATACAACACAAAAACTCCGTCCGTG	27	<nnxnp>	nn	124	np	117		
>ref-2919	TTTCGGCTTACACTCTCTCCTCCTTAT	26	<hkxhk>	hh	37	hk	130	kk	71
>ref-2928	AGAAGGGACACCTTATCTCCGCACGGA	22	<lmxll>	ll	267	lm	0		
>ref-2950	TCCCCTCCGACCCCCTCTCCCTCTCTT	4	<nnxnp>	nn	257	np	0		
>ref-2950	TCCCCTCCGACCCCCTCTCCCTCTCTT	12	<nnxnp>	nn	249	np	4		
>ref-2989	GTTTATGTCACGCATCCTCCCTCTGCA	1	<nnxnp>	nn	269	np	0		
>ref-2989	GTTTATGTCACGCATCCTCCCTCTGCA	5	<lmxll>	ll	137	lm	122		
>ref-3028	ATTCTCTTCACCCCCCCTCCCTCCTCC	4	<lmxll>	ll	254	lm	0		
>ref-3028	ATTCTCTTCACCCCCCCTCCCTCCTCC	6	<lmxll>	ll	250	lm	0		
>ref-3029	GAAATAGATACTGACTCTCCATGGGTA	25	<lmxll>	ll	126	lm	115		
>ref-3030	CTCATATCAACCTTATCTCCCATCTTC	9	<hkxhk>	hh	56	hk	148	kk	45
>ref-3036	GACAAGAACACAAAACCTCCCTTCCCT	2	<hkxhk>	hh	0	hk	273	kk	0
>ref-3036	GACAAGAACACAAAACCTCCCTTCCCT	16	<lmxll>	ll	171	lm	89		
>ref-3037	CTTGACGTTACGGACTCTCCTTTACGT	23	<hkxhk>	hh	52	hk	122	kk	61
>ref-3111	CCACCCTCCACCCTCCCTCCACTCCTC	2	<nnxnp>	nn	230	np	23		
>ref-3111	CCACCCTCCACCCTCCCTCCACTCCTC	3	<nnxnp>	nn	197	np	23		
>ref-3126	CGACTCTTAACCTGTTCTCCGAAAAGA	5	<nnxnp>	nn	254	np	0		
>ref-3140	CCCCTGCCCACACTCACTCCTCTGGCC	25	<nnxnp>	nn	273	np	0		
>ref-3182	AATGAGGCGACGCTCGCTCCACATATA	6	<lmxll>	ll	126	lm	129		
>ref-3192	GATCAAGCCACTCTCTCTCCTGAAAAC	23	<nnxnp>	nn	171	np	83		
>ref-3192	GATCAAGCCACTCTCTCTCCTGAAAAC	26	<nnxnp>	nn	233	np	32		
>ref-3192	GATCAAGCCACTCTCTCTCCTGAAAAC	27	<lmxll>	ll	195	lm	63		
>ref-3196	CTCTCCCATACCCTCCCTCCCCGCATC	9	<lmxll>	ll	66	lm	196		
>ref-3196	CTCTCCCATACCCTCCCTCCCCGCATC	15	<nnxnp>	nn	233	np	36		
>ref-3196	CTCTCCCATACCCTCCCTCCCCGCATC	16	<nnxnp>	nn	272	np	1		
>ref-3196	CTCTCCCATACCCTCCCTCCCCGCATC	21	<hkxhk>	hh	59	hk	208	kk	0
>ref-3196	CTCTCCCATACCCTCCCTCCCCGCATC	24	<nnxnp>	nn	235	np	33		
>ref-3196	CTCTCCCATACCCTCCCTCCCCGCATC	26	<hkxhk>	hh	0	hk	273	kk	0
>ref-3202	AACCAGGTCACTATACCTCCGCCTACG	25	<lmxll>	ll	138	lm	128		
>ref-3222	GCTGCCCCAACTGTTCCTCCTACTACT	15	<hkxhk>	hh	3	hk	262	kk	2
>ref-3222	GCTGCCCCAACTGTTCCTCCTACTACT	25	<hkxhk>	hh	2	hk	262	kk	3
>ref-3232	ATCAACAGCACAAAGCCTCCCCCAGCG	1	<hkxhk>	hh	62	hk	144	kk	61
>ref-3260	TGCATAGGAACACAAGCTCCGCCTTCT	15	<hkxhk>	hh	0	hk	273	kk	0
>ref-3265	AGCACACACACCCTGCCTCCTTAGCCG	8	<nnxnp>	nn	135	np	121		
>ref-3274	TCTCACAGCACACCATCTCCCTGCGAT	14	<lmxll>	ll	137	lm	131		
>ref-3277	GTAGCGAGTACGTCCTCTCCTCCACGT	15	<hkxhk>	hh	69	hk	127	kk	47
>ref-3305	ACTACAACTACTACAACTCCCATTACA	1	<hkxhk>	hh	3	hk	250	kk	0
>ref-3305	ACTACAACTACTACAACTCCCATTACA	25	<hkxhk>	hh	3	hk	250	kk	0

>ref-3317	CACACTCTCACTCACTCTCCTCACGCT	25	<nnxnp>	nn	259	np	3		
>ref-3342	TCAAGTCTTACTGCCCCTCCTCCCCCT	9	<nnxnp>	nn	124	np	129		
>ref-3347	AAACGAAACACCAATCCTCCTCTTTCC	15	<hkxhk>	hh	49	hk	143	kk	68
>ref-3367	TTGCCAAGGACATGGACTCCCCCCACC	1	<hkxhk>	hh	0	hk	127	kk	134
>ref-3367	TTGCCAAGGACATGGACTCCCCCCACC	5	<hkxhk>	hh	0	hk	273	kk	0
>ref-3367	TTGCCAAGGACATGGACTCCCCCCACC	9	<nnxnp>	nn	269	np	3		
>ref-3367	TTGCCAAGGACATGGACTCCCCCCACC	15	<lmxll>	ll	217	lm	29		
>ref-3367	TTGCCAAGGACATGGACTCCCCCCACC	21	<hkxhk>	hh	0	hk	123	kk	135
>ref-3367	TTGCCAAGGACATGGACTCCCCCCACC	24	<hkxhk>	hh	0	hk	248	kk	21
>ref-3367	TTGCCAAGGACATGGACTCCCCCCACC	25	<hkxhk>	hh	19	hk	251	kk	0
>ref-3368	TCCTTCTCTACGCCTTCTCCTTCCCCC	27	<lmxll>	ll	133	lm	128		
>ref-3374	GTTTCTCATACGGCTTCTCCACTCCCC	5	<hkxhk>	hh	2	hk	270	kk	0
>ref-3374	GTTTCTCATACGGCTTCTCCACTCCCC	8	<hkxhk>	hh	0	hk	272	kk	0
>ref-3374	GTTTCTCATACGGCTTCTCCACTCCCC	21	<hkxhk>	hh	93	hk	172	kk	0
>ref-3391	CCTACTCCAACCCTACCTCCTTCCCCT	9	<nnxnp>	nn	224	np	8		
>ref-3413	GAGGACACCACCACCTCTCCCTATGTC	8	<hkxhk>	hh	0	hk	269	kk	2
>ref-3413	GAGGACACCACCACCTCTCCCTATGTC	9	<hkxhk>	hh	2	hk	269	kk	0
>ref-3413	GAGGACACCACCACCTCTCCCTATGTC	22	<hkxhk>	hh	0	hk	272	kk	1
>ref-3413	GAGGACACCACCACCTCTCCCTATGTC	23	<hkxhk>	hh	1	hk	272	kk	0
>ref-3454	GACAGAGGGACGTAGGCTCCTGGAGGA	14	<hkxhk>	hh	230	hk	32	kk	0
>ref-3454	GACAGAGGGACGTAGGCTCCTGGAGGA	25	<lmxll>	ll	258	lm	5		
>ref-3463	CTGCCTTCCACCCTTTCTCCTTGTCCT	24	<hkxhk>	hh	47	hk	146	kk	65
>ref-3466	TCTTCGTTCACTCGTTCTCCTCCACAA	21	<nnxnp>	nn	199	np	34		
>ref-3488	GGGAGAAACACAATCCCTCCGCAAACA	16	<nnxnp>	nn	112	np	131		
>ref-3498	GCATCCACAACATCAGCTCCAGTTGGA	13	<lmxll>	ll	109	lm	146		
>ref-3502	TGGTTGGCCACTTATACTCCCCAGAGT	24	<lmxll>	ll	127	lm	128		
>ref-3531	CGGCCGGTGACGTCAACTCCGCTAACT	2	<nnxnp>	nn	134	np	130		
>ref-3533	TGTCAGCAAACATACGCTCCAGCTAGT	24	<nnxnp>	nn	122	np	130		
>ref-3534	CTCTACTACACCCTCCCTCCCCCTTCC	3	<nnxnp>	nn	272	np	0		
>ref-3534	CTCTACTACACCCTCCCTCCCCCTTCC	5	<lmxll>	ll	124	lm	138		
>ref-3554	AGGGTGTCCACGCACCCTCCTCACCAG	8	<nnxnp>	nn	130	np	139		
>ref-3554	AGGGTGTCCACGCACCCTCCTCACCAG	26	<nnxnp>	nn	129	np	139		
>ref-3603	GTTACTCCAACTCATCCTCCTTACAAC	12	<hkxhk>	hh	60	hk	134	kk	38
>ref-3615	CGAGCGTTAACCTAACCTCCCACCTTT	6	<lmxll>	ll	125	lm	136		
>ref-3624	TCTTTTGCTACTGTCTCTCCTTCTCCC	3	<lmxll>	ll	145	lm	123		
>ref-3624	TCTTTTGCTACTGTCTCTCCTTCTCCC	9	<hkxhk>	hh	1	hk	270	kk	0
>ref-3624	TCTTTTGCTACTGTCTCTCCTTCTCCC	13	<hkxhk>	hh	0	hk	270	kk	1
>ref-3626	CCTTCCCCAACTTTCTCTCCTTAATCC	21	<nnxnp>	nn	94	np	142		

>ref-3641	CTGTTAAGCACTGTTGCTCCACAGTTT	15	<nnxnp>	nn	118	np	141		
>ref-3644	TTCCATCCAACAAGCCCTCCCTCTCCT	14	<nnxnp>	nn	135	np	127		
>ref-3649	GTTTTCTTCACTCTCCCTCCTTCCTTC	3	<hkxhk>	hh	0	hk	264	kk	9
>ref-3649	GTTTTCTTCACTCTCCCTCCTTCCTTC	15	<hkxhk>	hh	70	hk	178	kk	0
>ref-3649	GTTTTCTTCACTCTCCCTCCTTCCTTC	27	<hkxhk>	hh	70	hk	178	kk	0
>ref-3667	CACACACACACTTCACCTCCTTCACAA	5	<nnxnp>	nn	257	np	0		
>ref-3668	GAAGCGAAGACCTTTCCTCCTCTGCTA	13	<lmxll>	ll	7	lm	262		
>ref-3668	GAAGCGAAGACCTTTCCTCCTCTGCTA	24	<nnxnp>	nn	21	np	237		
>ref-3671	CACCCACGCACTTTACCTCCACAAGCT	1	<lmxll>	ll	112	lm	113		
>ref-3681	CTCTCTTCAACCTCCCCTCCATACCTT	4	<hkxhk>	hh	52	hk	125	kk	64
>ref-3685	AACTTGGCCACTGCATCTCCATTAGCA	27	<nnxnp>	nn	123	np	147		
>ref-3696	ACACGACCAACACGAGCTCCCAACCGA	2	<nnxnp>	nn	237	np	0		
>ref-3698	ACTTATCCAACAACTTCTCCAGTCACT	22	<hkxhk>	hh	0	hk	268	kk	0
>ref-3698	ACTTATCCAACAACTTCTCCAGTCACT	25	<hkxhk>	hh	5	hk	263	kk	0
>ref-3711	GATATCGCGACGCGATCTCCTCCCCGG	7	<hkxhk>	hh	44	hk	131	kk	74
>ref-3732	CCTCCGTTCACTCATTCTCCTTGTCTA	1	<hkxhk>	hh	0	hk	270	kk	1
>ref-3732	CCTCCGTTCACTCATTCTCCTTGTCTA	26	<hkxhk>	hh	1	hk	270	kk	0
>ref-3733	GTTCCTCCCACCTCTCCTCCCTGCATC	1	<lmxll>	ll	120	lm	128		
>ref-3761	CCCTTACCCACATCCCCTCCGCCCATT	1	<nnxnp>	nn	246	np	0		
>ref-3761	CCCTTACCCACATCCCCTCCGCCCATT	3	<nnxnp>	nn	249	np	0		
>ref-3772	TAATCACTAACTCAACCTCCCTTCACT	16	<hkxhk>	hh	5	hk	268	kk	0
>ref-3788	CACCCCCACACACTCCCTCCTCCTTCA	13	<lmxll>	ll	8	lm	264		
>ref-3788	CACCCCCACACACTCCCTCCTCCTTCA	25	<lmxll>	ll	8	lm	264		
>ref-3803	CCGCCGATCACTGTCTCTCCAAGCTAA	6	<nnxnp>	nn	271	np	0		
>ref-3832	AATTGGTGCACGTACCCTCCACCAAGT	16	<nnxnp>	nn	118	np	138		
>ref-3837	TCTCTTCCCACTCCATCTCCTCCTTGT	14	<hkxhk>	hh	62	hk	194	kk	0
>ref-3837	TCTCTTCCCACTCCATCTCCTCCTTGT	26	<hkxhk>	hh	60	hk	194	kk	0
>ref-3839	ACTGACTCAACTCACCCTCCCACTGAA	8	<nnxnp>	nn	107	np	113		
>ref-3841	AGCGTGCTGACTGTGTCTCCTCCTCTA	9	<lmxll>	ll	117	lm	153		
>ref-3847	ATCCCATACACTCTTCCTCCATCTCCT	1	<hkxhk>	hh	54	hk	142	kk	64
>ref-3855	AGGCGACCCACTTCCCCTCCATTCTTT	24	<nnxnp>	nn	129	np	131		
>ref-3923	TGTTGACCAACGAACTCTCCACATCTG	15	<lmxll>	ll	122	lm	128		
>ref-3930	CAGTGAATTACGGCTGCTCCTGTGTTT	4	<lmxll>	ll	4	lm	264		
>ref-3932	CACCCTGCCACCCCTTCTCCTTTCTTC	8	<lmxll>	ll	263	lm	0		
>ref-3933	CCTTTCTCCACTATCCCTCCCTCTCTT	3	<nnxnp>	nn	60	np	161		
>ref-3938	TTCTTCCCCACATCCCCTCCTTCCTTC	12	<hkxhk>	hh	234	hk	4	kk	0
>ref-3938	TTCTTCCCCACATCCCCTCCTTCCTTC	14	<lmxll>	ll	252	lm	0		
>ref-3961	TACCCTCATACCCCATCTCCCCCCTTC	24	<lmxll>	ll	138	lm	119		

>ref-3967	CTCCTGTCCACCTTTTCTCCTCTCCTC	1	<nnxnp>	nn	262	np	1		
>ref-3972	TAGAGATAGACTCGCCCTCCACCATAT	2	<nnxnp>	nn	136	np	130		
>ref-3972	TAGAGATAGACTCGCCCTCCACCATAT	25	<nnxnp>	nn	136	np	130		
>ref-3986	CCACCCCCCACCCTTTCTCCCTCCCAA	1	<nnxnp>	nn	84	np	169		
>ref-3986	CCACCCCCCACCCTTTCTCCCTCCCAA	26	<nnxnp>	nn	82	np	170		
>ref-4004	CCTCCCCCAACCCTACCTCCCCCTCAT	16	<nnxnp>	nn	129	np	117		
>ref-4005	CCTGCCTCTACCCTACCTCCCCCCTTC	21	<nnxnp>	nn	133	np	136		
>ref-4016	CCCCTTTCTACTTCTTCTCCCCTTCTC	2	<hkxhk>	hh	13	hk	237	kk	1
>ref-4016	CCCCTTTCTACTTCTTCTCCCCTTCTC	4	<nnxnp>	nn	180	np	76		
>ref-4052	TCCCTCCCTACCCACTCTCCGTCCCCA	21	<lmxll>	ll	185	lm	51		
>ref-4052	TCCCTCCCTACCCACTCTCCGTCCCCA	26	<lmxll>	ll	187	lm	50		
>ref-4071	CATCCCTCGACATCCTCTCCCTTGCTT	8	<lmxll>	ll	128	lm	140		
>ref-4090	ACTACCATAACTACAACTCCTTTAAAT	2	<hkxhk>	hh	25	hk	232	kk	0
>ref-4090	ACTACCATAACTACAACTCCTTTAAAT	14	<hkxhk>	hh	49	hk	191	kk	0
>ref-4090	ACTACCATAACTACAACTCCTTTAAAT	23	<hkxhk>	hh	0	hk	171	kk	66
>ref-4124	TGGCTCCTTACACCCCCTCCCCCCCAC	9	<lmxll>	ll	157	lm	81		
>ref-4124	TGGCTCCTTACACCCCCTCCCCCCCAC	12	<lmxll>	ll	156	lm	82		
>ref-4140	TAACCGCCAACAACAACTCCGGAGCCT	2	<hkxhk>	hh	60	hk	140	kk	60
>ref-4170	GGAGCCGATACGCCCTCTCCCTCGCCT	24	<nnxnp>	nn	137	np	130		
>ref-4212	TAACCGATTACCTGGTCTCCTGCTCCA	13	<nnxnp>	nn	129	np	136		
>ref-4214	ATTTTTATCACCTTCCCTCCTCCCTTA	21	<nnxnp>	nn	156	np	89		
>ref-4217	CTACCACCAACACTGCCTCCAACATTG	3	<hkxhk>	hh	2	hk	270	kk	0
>ref-4217	CTACCACCAACACTGCCTCCAACATTG	4	<lmxll>	ll	193	lm	63		
>ref-4217	CTACCACCAACACTGCCTCCAACATTG	6	<lmxll>	ll	105	lm	149		
>ref-4217	CTACCACCAACACTGCCTCCAACATTG	13	<hkxhk>	hh	2	hk	270	kk	0
>ref-4217	CTACCACCAACACTGCCTCCAACATTG	21	<hkxhk>	hh	76	hk	176	kk	0
>ref-4221	CACCACAACACGTCAACTCCTCCTCCA	25	<lmxll>	ll	237	lm	0		
>ref-4221	CACCACAACACGTCAACTCCTCCTCCA	27	<lmxll>	ll	228	lm	1		
>ref-4227	CCTCCTGGCACTGTATCTCCTTTATTT	4	<nnxnp>	nn	130	np	128		
>ref-4234	TTTCTCAACACCACAACTCCTTCCCTC	24	<nnxnp>	nn	131	np	119		
>ref-4259	ACCACATCAACTCGACCTCCTTATTTC	13	<lmxll>	ll	151	lm	114		
>ref-4263	CCGCTGAGCACGTGTCCTCCTTGGACG	12	<nnxnp>	nn	259	np	0		
>ref-4282	ATGCCTTTTACTGGTACTCCCTCCTTG	12	<hkxhk>	hh	58	hk	141	kk	50
>ref-4282	ATGCCTTTTACTGGTACTCCCTCCTTG	22	<hkxhk>	hh	60	hk	141	kk	50
>ref-4296	CCTACGCCTACGCCTACTCCGGCAAGT	6	<hkxhk>	hh	0	hk	272	kk	1
>ref-4304	TTAAGCCCTACGGTTGCTCCTCCCCCT	1	<lmxll>	ll	121	lm	134		
>ref-4336	ATAGCTGTAACACCTCCTCCAGCTGCT	2	<lmxll>	ll	115	lm	154		
>ref-4341	CTACCCCCCACGTCCCCTCCTTTCCTT	22	<nnxnp>	nn	107	np	145		

>ref-4357	TCACGTTGTACTCAGGCTCCTTCGCTG	25	<nnxnp>	nn	134	np	139		
>ref-4374	CACCCTTCCACCCTCTCTCCTCCCAAC	2	<nnxnp>	nn	96	np	144		
>ref-4374	CACCCTTCCACCCTCTCTCCTCCCAAC	26	<nnxnp>	nn	95	np	143		
>ref-4413	CACCCCTACACCCGTCCTCCCCTCCCA	16	<nnxnp>	nn	107	np	148		
>ref-4422	GTGTTTTTAACGCCCGCTCCAAAACCA	2	<lmxll>	ll	119	lm	131		
>ref-4422	GTGTTTTTAACGCCCGCTCCAAAACCA	16	<nnxnp>	nn	132	np	112		
>ref-4422	GTGTTTTTAACGCCCGCTCCAAAACCA	26	<lmxll>	ll	119	lm	125		
>ref-4431	GCCGTTTGGACGATCTCTCCCACCAAG	9	<lmxll>	ll	142	lm	119		
>ref-4440	CTTCCCCTTACTTCTCCTCCTCGCACA	23	<nnxnp>	nn	122	np	113		
>ref-4461	ATAGCATCAACACCCTCTCCTCCCCCC	27	<lmxll>	ll	141	lm	123		
>ref-4465	GCGGTAGTCACAGCTGCTCCGCTTGCA	12	<lmxll>	ll	138	lm	129		
>ref-4473	AAGATCTTGACGAGATCTCCCGGAATA	1	<hkxhk>	hh	60	hk	136	kk	46
>ref-4479	GCTCTCGTAACAGATCCTCCTCTACTG	8	<lmxll>	ll	119	lm	140		
>ref-4491	CCATCTTTTACTCTTCCTCCTCTTCAT	24	<hkxhk>	hh	0	hk	271	kk	1
>ref-4491	CCATCTTTTACTCTTCCTCCTCTTCAT	26	<hkxhk>	hh	1	hk	271	kk	0
>ref-4496	CCACCACTCACCCACCCTCCTTCGCTT	2	<hkxhk>	hh	3	hk	267	kk	0
>ref-4496	CCACCACTCACCCACCCTCCTTCGCTT	3	<hkxhk>	hh	2	hk	267	kk	0
>ref-4498	TGGACTTGAACTTTCGCTCCCAAGGGA	21	<nnxnp>	nn	105	np	159		
>ref-4501	ATTCAGGACACCTCAACTCCGTCGTCG	12	<nnxnp>	nn	260	np	0		
>ref-4550	ATATCAACAACCATGTCTCCAGCTCGG	2	<lmxll>	ll	141	lm	97		
>ref-4550	ATATCAACAACCATGTCTCCAGCTCGG	4	<lmxll>	ll	142	lm	97		
>ref-4567	TCCAGCTCCACCTTCCCTCCCACCTCC	2	<lmxll>	ll	143	lm	125		
>ref-4567	TCCAGCTCCACCTTCCCTCCCACCTCC	4	<lmxll>	ll	143	lm	125		
>ref-4571	CTCTCACGCACAAAAGCTCCCGCCCCT	12	<nnxnp>	nn	134	np	132		
>ref-4585	CAGAACATCACTCAGCCTCCCTCGCCT	14	<nnxnp>	nn	124	np	146		
>ref-4597	ACTTCTTTGACACGTGCTCCATTCCTC	9	<nnxnp>	nn	123	np	121		
>ref-4599	CACATATACACACCCTCTCCTCTCCTC	25	<lmxll>	ll	133	lm	136		
>ref-4605	CCCCTCCCCACGCTATCTCCCTTGCCA	15	<nnxnp>	nn	261	np	0		
>ref-4605	CCCCTCCCCACGCTATCTCCCTTGCCA	22	<nnxnp>	nn	265	np	0		
>ref-4605	CCCCTCCCCACGCTATCTCCCTTGCCA	23	<lmxll>	ll	113	lm	139		
>ref-4616	CTTCCCTCCACATCTCCTCCTCCACTC	1	<lmxll>	ll	105	lm	163		
>ref-4629	ATATATCACACATTTTCTCCCTCTCCT	13	<lmxll>	ll	143	lm	97		
>ref-4629	ATATATCACACATTTTCTCCCTCTCCT	15	<lmxll>	ll	143	lm	97		
>ref-4635	CAATTCTAGACTAAGCCTCCGGGCTAG	3	<nnxnp>	nn	114	np	136		
>ref-4647	TGTCTACCCACACTTCCTCCCTTCCTC	23	<lmxll>	ll	141	lm	126		
>ref-4675	CTCTCTCCCACTCCTGCTCCCCCTATC	1	<nnxnp>	nn	266	np	0		
>ref-4715	AAGTCCCCGACTCCTCCTCCTCCCCCA	5	<lmxll>	ll	127	lm	134		
>ref-4725	TATTTACCTACCTGTTCTCCACCTACT	21	<lmxll>	ll	130	lm	131		

>ref-4725	TATTTACCTACCTGTTCTCCACCTACT	26	<lmxll>	ll	129	lm	132		
>ref-4737	ATATAACGGACACGAACTCCGATATAG	6	<hkxhk>	hh	7	hk	263	kk	0
>ref-4737	ATATAACGGACACGAACTCCGATATAG	12	<hkxhk>	hh	13	hk	250	kk	0
>ref-4737	ATATAACGGACACGAACTCCGATATAG	13	<hkxhk>	hh	1	hk	272	kk	0
>ref-4745	TGTGGAGACACACGGCCTCCGGGCATA	13	<nnxnp>	nn	129	np	143		
>ref-4748	TCTCTCCCCACCACGCCTCCTCCATTC	8	<hkxhk>	hh	46	hk	119	kk	58
>ref-4791	AGCGATGAAACCTGAGCTCCTCGACCA	4	<nnxnp>	nn	130	np	130		
>ref-4818	CTTCAAACTACTCAAGCTCCGGGTGAA	2	<hkxhk>	hh	0	hk	214	kk	19
>ref-4818	CTTCAAACTACTCAAGCTCCGGGTGAA	3	<lmxll>	ll	128	lm	104		
>ref-4818	CTTCAAACTACTCAAGCTCCGGGTGAA	4	<lmxll>	ll	131	lm	104		
>ref-4818	CTTCAAACTACTCAAGCTCCGGGTGAA	7	<hkxhk>	hh	19	hk	214	kk	0
>ref-4832	ATCCCTCCAACACAATCTCCATCCCTC	3	<lmxll>	ll	4	lm	264		
>ref-4832	ATCCCTCCAACACAATCTCCATCCCTC	13	<nnxnp>	nn	181	np	84		
>ref-4832	ATCCCTCCAACACAATCTCCATCCCTC	15	<hkxhk>	hh	8	hk	262	kk	0
>ref-4832	ATCCCTCCAACACAATCTCCATCCCTC	24	<hkxhk>	hh	51	hk	209	kk	0
>ref-4843	CAAGAAGCAACGGGGCCTCCAGTCGCC	3	<nnxnp>	nn	248	np	2		
>ref-4869	TAACCAATCACAAGGTCTCCTTTATCT	26	<lmxll>	ll	138	lm	122		
>ref-4874	CTCTCTCTTACTCTTCCTCCGACGACA	21	<lmxll>	ll	141	lm	117		
>ref-4874	CTCTCTCTTACTCTTCCTCCGACGACA	22	<lmxll>	ll	140	lm	117		
>ref-4888	CGACATTATACGCAAACTCCCCCCCGC	9	<nnxnp>	nn	126	np	110		
>ref-4907	TCTTCTGTCACCCTTCCTCCTTGCCCT	21	<nnxnp>	nn	110	np	152		
>ref-4916	CACACACGTACACCTACTCCTACACGC	7	<lmxll>	ll	131	lm	140		
>ref-4916	CACACACGTACACCTACTCCTACACGC	26	<lmxll>	ll	129	lm	142		
>ref-4925	CTTCCTCCTACCTTCTCTCCCTCATCG	4	<nnxnp>	nn	254	np	0		
>ref-4925	CTTCCTCCTACCTTCTCTCCCTCATCG	21	<nnxnp>	nn	250	np	1		
>ref-4956	CTCATTCTTACTCCTCCTCCCTGCCTC	9	<nnxnp>	nn	267	np	0		
>ref-4956	CTCATTCTTACTCCTCCTCCCTGCCTC	23	<nnxnp>	nn	10	np	253		
>ref-4964	AAATGAAGGACATGGGCTCCGTCAGCT	21	<hkxhk>	hh	55	hk	150	kk	48
>ref-4982	CTCAGTCTAACGGCCTCTCCCTTCCCT	6	<hkxhk>	hh	3	hk	260	kk	0
>ref-4984	AGCATAAGCACGCCTTCTCCAGCCTTT	6	<hkxhk>	hh	58	hk	144	kk	64
>ref-4988	CACCTCTCCACCACCACTCCATACTCA	23	<lmxll>	ll	106	lm	146		
>ref-4996	TGTCGGGGCACAGCGTCTCCAGGTACA	22	<nnxnp>	nn	273	np	0		
>ref-5020	TCTTCTCTGACGTTCCCTCCTTCTCTC	15	<lmxll>	ll	60	lm	161		
>ref-5020	TCTTCTCTGACGTTCCCTCCTTCTCTC	16	<lmxll>	ll	59	lm	162		
>ref-5040	CCCTTTCCTACCGTCCCTCCCTACCCC	24	<lmxll>	ll	115	lm	133		
>ref-5051	GATATGAGCACGGCGGCTCCATAAACA	4	<lmxll>	ll	139	lm	102		
>ref-5052	AAATTCTTGACACTCTCTCCTCAGTAT	8	<nnxnp>	nn	119	np	126		
>ref-5079	CTCCTTCTAACCACTTCTCCTTTCCCT	4	<nnxnp>	nn	109	np	129		

>ref-5087	CTCCACCGCACCCCATCTCCATCACCA	3	<lmxll>	ll	117	lm	139		
>ref-5087	CTCCACCGCACCCCATCTCCATCACCA	4	<lmxll>	ll	112	lm	143		
>ref-5089	CAGCAAATGACACGCTCTCCATTCTTC	4	<nnxnp>	nn	10	np	259		
>ref-5119	CTTACCCCCACCCTACCTCCGAGGACT	23	<nnxnp>	nn	219	np	0		
>ref-5134	ACGCACACAACAGCGCCTCCACCCAAG	1	<nnxnp>	nn	140	np	131		
>ref-5134	ACGCACACAACAGCGCCTCCACCCAAG	16	<nnxnp>	nn	132	np	140		
>ref-5145	ACCTCCCTCACCCCCTCTCCCGCTGCC	22	<nnxnp>	nn	129	np	100		
>ref-5199	CACACATACACCCTCCCTCCTCTCTCC	5	<nnxnp>	nn	124	np	144		
>ref-5199	CACACATACACCCTCCCTCCTCTCTCC	23	<nnxnp>	nn	120	np	146		
>ref-5205	GGACTGGAAACAAAGCCTCCGCCGTAC	13	<nnxnp>	nn	122	np	145		
>ref-5210	ACTTTGTCGACGGGGCCTCCTTCTGCG	1	<nnxnp>	nn	263	np	0		
>ref-5210	ACTTTGTCGACGGGGCCTCCTTCTGCG	2	<nnxnp>	nn	263	np	0		
>ref-5239	ATGAGACTGACTTTGCCTCCAAAACTG	1	<nnxnp>	nn	155	np	109		
>ref-5239	ATGAGACTGACTTTGCCTCCAAAACTG	7	<hkxhk>	hh	0	hk	273	kk	0
>ref-5239	ATGAGACTGACTTTGCCTCCAAAACTG	9	<lmxll>	ll	172	lm	78		
>ref-5239	ATGAGACTGACTTTGCCTCCAAAACTG	22	<hkxhk>	hh	4	hk	267	kk	0
>ref-5279	CTCTCCTTTACTGCCCCTCCTCTCTCC	14	<hkxhk>	hh	120	hk	131	kk	1
>ref-5282	ACTCCCCTTACTCTCTCTCCCCTCCTT	7	<nnxnp>	nn	69	np	168		
>ref-5282	ACTCCCCTTACTCTCTCTCCCCTCCTT	15	<lmxll>	ll	16	lm	238		
>ref-5282	ACTCCCCTTACTCTCTCTCCCCTCCTT	26	<hkxhk>	hh	1	hk	268	kk	0
>ref-5319	GTAATCAACACTCCCTCTCCTCCGGAC	26	<hkxhk>	hh	24	hk	225	kk	0
>ref-5342	CCCTCTTGAACTCCATCTCCAACATGA	1	<lmxll>	ll	21	lm	240		
>ref-5342	CCCTCTTGAACTCCATCTCCAACATGA	8	<nnxnp>	nn	269	np	3		
>ref-5342	CCCTCTTGAACTCCATCTCCAACATGA	14	<hkxhk>	hh	129	hk	119	kk	0
>ref-5342	CCCTCTTGAACTCCATCTCCAACATGA	22	<nnxnp>	nn	238	np	21		
>ref-5342	CCCTCTTGAACTCCATCTCCAACATGA	25	<nnxnp>	nn	271	np	0		
>ref-5348	ACAGACTGAACCCCCTCTCCACCAAAC	23	<nnxnp>	nn	135	np	129		
>ref-5355	CCTCTTGCAACATAAACTCCACGTGTG	25	<lmxll>	ll	138	lm	111		
>ref-5359	TTCCCTTTCACTTTTTCTCCCTGCAAT	4	<hkxhk>	hh	0	hk	273	kk	0
>ref-5359	TTCCCTTTCACTTTTTCTCCCTGCAAT	26	<lmxll>	ll	159	lm	100		
>ref-5410	TCAGTCTAGACTTTAGCTCCAAAGAGT	9	<lmxll>	ll	118	lm	107		
>ref-5410	TCAGTCTAGACTTTAGCTCCAAAGAGT	21	<nnxnp>	nn	108	np	119		
>ref-5411	CTCTCCCCAACACACCCTCCTCTCCTC	2	<hkxhk>	hh	0	hk	257	kk	11
>ref-5411	CTCTCCCCAACACACCCTCCTCTCCTC	5	<hkxhk>	hh	0	hk	257	kk	11
>ref-5411	CTCTCCCCAACACACCCTCCTCTCCTC	25	<hkxhk>	hh	8	hk	258	kk	0
>ref-5423	TTCCTACTGACACCCCCTCCTTTCTAT	1	<lmxll>	ll	149	lm	111		
>ref-5423	TTCCTACTGACACCCCCTCCTTTCTAT	4	<hkxhk>	hh	2	hk	270	kk	1
>ref-5423	TTCCTACTGACACCCCCTCCTTTCTAT	16	<nnxnp>	nn	127	np	127		

>ref-5425	TCACCGTTCACCGCACCTCCCACACCA	6	<lmxll>	ll	131	lm	137		
>ref-5439	CCCTTCCCCACCTTCCCTCCCCACATA	25	<hkxhk>	hh	58	hk	161	kk	16
>ref-5455	ATATCCTTCACTCAATCTCCTCTTCCT	4	<nnxnp>	nn	144	np	122		
>ref-5455	ATATCCTTCACTCAATCTCCTCTTCCT	25	<hkxhk>	hh	0	hk	266	kk	4
>ref-5519	CATAGCGCTACGGCGGCTCCCTGTGAC	7	<hkxhk>	hh	49	hk	144	kk	72
>ref-5519	CATAGCGCTACGGCGGCTCCCTGTGAC	14	<hkxhk>	hh	72	hk	144	kk	49
>ref-5523	TTCATCATCACCTGCCCTCCCTCTCCT	14	<nnxnp>	nn	256	np	0		
>ref-5523	TTCATCATCACCTGCCCTCCCTCTCCT	27	<nnxnp>	nn	253	np	2		
>ref-5562	CATACCATAACTAACTCTCCAATCATA	16	<nnxnp>	nn	158	np	100		
>ref-5573	CTTCCTCTCACATCTGCTCCACTATAC	7	<nnxnp>	nn	114	np	136		
>ref-5573	CTTCCTCTCACATCTGCTCCACTATAC	12	<nnxnp>	nn	115	np	136		
>ref-5573	CTTCCTCTCACATCTGCTCCACTATAC	23	<nnxnp>	nn	46	np	211		
>ref-5573	CTTCCTCTCACATCTGCTCCACTATAC	27	<hkxhk>	hh	15	hk	252	kk	0
>ref-5632	CCTTCTCTTACTCTAACTCCCTTTCTT	23	<lmxll>	ll	259	lm	0		
>ref-5633	CCCCCCTCTACCCTCACTCCACCCCCT	1	<lmxll>	ll	158	lm	88		
>ref-5633	CCCCCCTCTACCCTCACTCCACCCCCT	7	<lmxll>	ll	161	lm	85		
>ref-5640	CCCCGCCCGACTCGGTCTCCTGGGGCT	16	<nnxnp>	nn	262	np	0		
>ref-5658	CGGCCCTTCACCTCCTCTCCTCTTCTT	3	<hkxhk>	hh	0	hk	272	kk	0
>ref-5658	CGGCCCTTCACCTCCTCTCCTCTTCTT	26	<hkxhk>	hh	0	hk	272	kk	0
>ref-5660	ACCTGCCTCACTCCTCCTCCTTTCCTT	9	<lmxll>	ll	146	lm	123		
>ref-5660	ACCTGCCTCACTCCTCCTCCTTTCCTT	15	<hkxhk>	hh	72	hk	197	kk	0
>ref-5662	ATCGTTCACACTCATCCTCCCTCAATC	3	<lmxll>	ll	140	lm	127		
>ref-5672	CTCCCTTAAACCCCCACTCCCTTGAAT	23	<nnxnp>	nn	262	np	0		
>ref-5673	TTAATGGCAACTACATCTCCATCTTTT	12	<nnxnp>	nn	4	np	225		
>ref-5673	TTAATGGCAACTACATCTCCATCTTTT	16	<nnxnp>	nn	4	np	225		
>ref-5675	TCTCTATGTACCCTTTCTCCTGTCGTG	23	<hkxhk>	hh	63	hk	139	kk	49
>ref-5683	TCAATTCCAACCCTTACTCCATTTTAC	8	<nnxnp>	nn	110	np	117		
>ref-5686	GTGACTTGAACCCAAACTCCCTCCAGG	21	<hkxhk>	hh	61	hk	136	kk	54
>ref-5690	GGACACCTTACTCGCCCTCCGTGACTT	22	<hkxhk>	hh	65	hk	132	kk	62
>ref-5698	CACCTCCACACTCCCCCTCCACCTACC	2	<hkxhk>	hh	11	hk	237	kk	0
>ref-5698	CACCTCCACACTCCCCCTCCACCTACC	21	<hkxhk>	hh	11	hk	237	kk	0
>ref-5714	GGTCGAGGGACTGCACCTCCATCCGCA	5	<hkxhk>	hh	0	hk	273	kk	0
>ref-5714	GGTCGAGGGACTGCACCTCCATCCGCA	8	<hkxhk>	hh	0	hk	273	kk	0
>ref-5714	GGTCGAGGGACTGCACCTCCATCCGCA	26	<hkxhk>	hh	2	hk	270	kk	0
>ref-5721	TCCCTTCTCACTCATTCTCCTCTCTCT	3	<hkxhk>	hh	31	hk	234	kk	0
>ref-5721	TCCCTTCTCACTCATTCTCCTCTCTCT	9	<hkxhk>	hh	80	hk	180	kk	0
>ref-5721	TCCCTTCTCACTCATTCTCCTCTCTCT	13	<lmxll>	ll	136	lm	135		
>ref-5721	TCCCTTCTCACTCATTCTCCTCTCTCT	25	<hkxhk>	hh	0	hk	270	kk	1

>ref-5725	GGCTCAGCGACAGGCACTCCGCCCGAT	3	<lmxll>	ll	234	lm	33		
>ref-5725	GGCTCAGCGACAGGCACTCCGCCCGAT	15	<lmxll>	ll	233	lm	34		
>ref-5725	GGCTCAGCGACAGGCACTCCGCCCGAT	21	<nnxnp>	nn	118	np	135		
>ref-5758	CTTCCTCTCACTATCTCTCCATCTTTC	4	<nnxnp>	nn	216	np	12		
>ref-5760	ACTGCGGCCACCGTATCTCCAGCTGTA	26	<lmxll>	ll	133	lm	140		
>ref-5764	CCTCCTCCCACTCCACCTCCTCTAAGT	5	<lmxll>	ll	116	lm	149		
>ref-5765	ATACAATTCACATTTTCTCCCTCCTGT	25	<lmxll>	ll	129	lm	129		
>ref-5782	CACATAGATACACCACCTCCCACTGCA	7	<nnxnp>	nn	139	np	129		
>ref-5794	CTCTTCCCCACCCCCACTCCAATCCTC	4	<lmxll>	ll	155	lm	111		
>ref-5794	CTCTTCCCCACCCCCACTCCAATCCTC	27	<nnxnp>	nn	149	np	114		
>ref-5828	TTCCTTTCCACCTTCTCTCCTTTATTC	7	<nnxnp>	nn	116	np	130		
>ref-5855	CTCCTACACACACAGCCTCCCCTCCCC	2	<hkxhk>	hh	2	hk	259	kk	6
>ref-5855	CTCCTACACACACAGCCTCCCCTCCCC	22	<hkxhk>	hh	2	hk	258	kk	8
>ref-5886	AAAGAGCTCACAACCACTCCATAATTG	9	<nnxnp>	nn	111	np	138		
>ref-5892	CTCCTTCTAACTCCCTCTCCCCTCCTC	24	<lmxll>	ll	266	lm	0		
>ref-5912	ACTTACCCTACTCTCCCTCCCTCTCCT	8	<nnxnp>	nn	131	np	136		
>ref-5924	AGAACATTCACTCTTGCTCCAGGCGTC	6	<nnxnp>	nn	124	np	138		
>ref-5939	CCAGAGGCAACGAGGCCTCCAGTTGCT	16	<nnxnp>	nn	6	np	262		
>ref-5939	CCAGAGGCAACGAGGCCTCCAGTTGCT	21	<nnxnp>	nn	142	np	128		
>ref-5946	AACAAACAAACACAGCCTCCCTCACAA	13	<nnxnp>	nn	111	np	145		
>ref-5967	TCCTGGAACACCATTTCTCCCTATAGT	27	<lmxll>	ll	120	lm	131		
>ref-5979	TGTGGGGTGACACTGACTCCCCGTGGC	4	<nnxnp>	nn	202	np	59		
>ref-5979	TGTGGGGTGACACTGACTCCCCGTGGC	12	<hkxhk>	hh	19	hk	244	kk	0
>ref-5979	TGTGGGGTGACACTGACTCCCCGTGGC	13	<lmxll>	ll	231	lm	35		
>ref-5996	TTCGAAGCCACTCCCCCTCCCCCTCCC	13	<lmxll>	ll	123	lm	133		
>ref-6009	CATCCTTCGACCCTTCCTCCTTCTCGG	3	<lmxll>	ll	115	lm	123		
>ref-6013	TATTCCAACACACACACTCCATTATCT	24	<nnxnp>	nn	123	np	131		
>ref-6024	TAAAATAAGACAATGGCTCCCTCTCTC	15	<nnxnp>	nn	127	np	128		
>ref-6029	TTCATCGTGACTTTATCTCCGCTTCTC	9	<lmxll>	ll	122	lm	116		
>ref-6042	CCCCCTATAACTCCCTCTCCCTTCCTT	14	<hkxhk>	hh	0	hk	271	kk	1
>ref-6042	CCCCCTATAACTCCCTCTCCCTTCCTT	16	<hkxhk>	hh	1	hk	271	kk	0
>ref-6061	AACGTTGCGACAGACCCTCCGTTCTGA	15	<lmxll>	ll	126	lm	144		
>ref-6140	CGACCCACGACCATCTCTCCTCACAAT	14	<hkxhk>	hh	0	hk	272	kk	1
>ref-6140	CGACCCACGACCATCTCTCCTCACAAT	27	<hkxhk>	hh	0	hk	272	kk	1
>ref-6156	TGCCTTTACACTCACTCTCCCTAATCC	8	<nnxnp>	nn	118	np	138		
>ref-6159	TCCTAAGTCACGCTTTCTCCCCTTGTC	4	<lmxll>	ll	126	lm	136		
>ref-6187	TCCCTCTCAACCCCTCCTCCCCTTCTT	3	<nnxnp>	nn	52	np	211		
>ref-6187	TCCCTCTCAACCCCTCCTCCCCTTCTT	5	<nnxnp>	nn	178	np	85		

>ref-6187	TCCCTCTCAACCCCTCCTCCCCTTCTT	9	<nnxnp>	nn	52	np	213		
>ref-6187	TCCCTCTCAACCCCTCCTCCCCTTCTT	27	<nnxnp>	nn	12	np	254		
>ref-6194	CCTTCCCCTACCATCTCTCCGTCTTTC	1	<hkxhk>	hh	2	hk	270	kk	0
>ref-6194	CCTTCCCCTACCATCTCTCCGTCTTTC	7	<hkxhk>	hh	2	hk	270	kk	0
>ref-6195	CTCTCTCTCACAACACCTCCGCCTCCG	1	<lmxll>	ll	133	lm	137		
>ref-6209	TTTCTTCCCACCTCATCTCCCTACCTG	3	<hkxhk>	hh	62	hk	143	kk	49
>ref-6209	TTTCTTCCCACCTCATCTCCCTACCTG	4	<hkxhk>	hh	49	hk	143	kk	62
>ref-6234	CAATGCCCCACCCCTTCTCCTCTGTCC	1	<lmxll>	ll	132	lm	137		
>ref-6284	ACACGAAGCACACATACTCCAACAACA	3	<hkxhk>	hh	56	hk	146	kk	64
>ref-6292	TCTTCTGAGACTCTCTCTCCATGATTG	13	<lmxll>	ll	112	lm	124		
>ref-6307	GGATTCCCAACCTAAGCTCCGCCCCCT	21	<lmxll>	ll	127	lm	131		
>ref-6307	GGATTCCCAACCTAAGCTCCGCCCCCT	25	<nnxnp>	nn	142	np	116		
>ref-6315	TTTGAAGACACCCCCTCTCCTTCCCCC	14	<lmxll>	ll	261	lm	0		
>ref-6360	TGTCCCTGAACCGCTTCTCCGTGGCAG	13	<lmxll>	ll	135	lm	135		
>ref-6360	TGTCCCTGAACCGCTTCTCCGTGGCAG	25	<lmxll>	ll	135	lm	135		
>ref-6366	CTTCATCTAACACCTTCTCCTTTAAAG	1	<lmxll>	ll	176	lm	74		
>ref-6366	CTTCATCTAACACCTTCTCCTTTAAAG	26	<lmxll>	ll	103	lm	142		
>ref-6371	TCGATAAATACCGAGCCTCCGTAACCT	26	<nnxnp>	nn	125	np	143		
>ref-6389	CTCTGCCCAACCCCTTCTCCTTCTCCT	22	<lmxll>	ll	118	lm	136		
>ref-6394	TGCTGTCACACTTTTTCTCCTCGTCCT	3	<lmxll>	ll	132	lm	133		
>ref-6401	GAGGGCAGCACCGTCCCTCCCCTGCTG	21	<hkxhk>	hh	0	hk	273	kk	0
>ref-6418	TTCCTATCTACAGGGACTCCTTTACCC	25	<hkxhk>	hh	49	hk	121	kk	58
>ref-6423	CGTATTAACACCTGCTCTCCATCGGGA	15	<lmxll>	ll	137	lm	131		
>ref-6434	GATTGGAAAACCGCTCCTCCACCGGGC	3	<lmxll>	ll	147	lm	102		
>ref-6438	TTTTCGTCCACTTCCTCTCCCCATTCT	21	<nnxnp>	nn	125	np	126		
>ref-6465	CACAATCACACACTCCCTCCCTCCACC	23	<nnxnp>	nn	122	np	143		
>ref-6523	TCATACCAGACACTCTCTCCTTTCTCT	22	<lmxll>	ll	136	lm	116		
>ref-6529	TCCCCCCTTACTCCTCCTCCTCGCGTT	1	<lmxll>	ll	134	lm	133		
>ref-6547	CTGATTCCCACCTTCCCTCCATCCCCC	4	<lmxll>	ll	253	lm	0		
>ref-6582	ATCCATCTCACTTATCCTCCATGTCTA	21	<nnxnp>	nn	122	np	143		
>ref-6597	AAATTACTTACCTGTTCTCCCAGCATA	26	<hkxhk>	hh	53	hk	132	kk	63
>ref-6641	CCTGCCATCACTTCCTCTCCGCTCATT	2	<nnxnp>	nn	134	np	135		
>ref-6647	CTCCCTGCCACCCTCACTCCACCTTTC	21	<nnxnp>	nn	129	np	139		
>ref-6671	CTTCCTCCCACCTCCTCTCCATATCTT	3	<nnxnp>	nn	230	np	2		
>ref-6706	TCCCTCCTCACTTCCTCTCCTAATCCC	7	<lmxll>	ll	24	lm	223		
>ref-6706	TCCCTCCTCACTTCCTCTCCTAATCCC	22	<lmxll>	ll	23	lm	222		
>ref-6729	AGCAGAAAGACCGCGGCTCCCCTGTCA	2	<nnxnp>	nn	132	np	136		
>ref-6733	TCCCCACCCACTCGCCCTCCCCGCCCA	1	<lmxll>	ll	67	lm	167		

>ref-6733	TCCCCACCCACTCGCCCTCCCCGCCCA	2	<lmxll>	ll	63	lm	168		
>ref-6734	TCCGGTGCCACACCCTCTCCTAAACCC	14	<lmxll>	ll	111	lm	145		
>ref-6735	AGAGCGCCAACTGTGACTCCATTTTCT	22	<nnxnp>	nn	265	np	0		
>ref-6739	CTGTCTACGACCTGCCCTCCTTACCTC	6	<nnxnp>	nn	125	np	144		
>ref-6739	CTGTCTACGACCTGCCCTCCTTACCTC	7	<nnxnp>	nn	119	np	144		
>ref-6773	GCTTTGACAACCTGTTCTCCTTCAGAT	3	<hkxhk>	hh	0	hk	253	kk	14
>ref-6773	GCTTTGACAACCTGTTCTCCTTCAGAT	25	<nnxnp>	nn	169	np	100		
>ref-6778	CTTTCCTCCACAACCTCTCCCTCCCCG	13	<lmxll>	ll	3	lm	260		
>ref-6778	CTTTCCTCCACAACCTCTCCCTCCCCG	15	<lmxll>	ll	3	lm	259		
>ref-6800	GGATCTTAAACTCGTTCTCCATTCCTT	21	<hkxhk>	hh	69	hk	129	kk	61
>ref-6822	GCTGAGCCGACTGGTTCTCCTTTTCAC	1	<lmxll>	ll	126	lm	115		
>ref-6822	GCTGAGCCGACTGGTTCTCCTTTTCAC	2	<lmxll>	ll	126	lm	115		
>ref-6827	TACCGCCTTACTCCATCTCCTCCTTCC	5	<nnxnp>	nn	119	np	152		
>ref-6833	TTTTTAAAGACCGGCTCTCCTCCTTTC	24	<nnxnp>	nn	115	np	119		
>ref-6852	CTATAGGGTACCCTCGCTCCTCTCCCC	15	<hkxhk>	hh	48	hk	135	kk	80
>ref-6855	TGTTCCTTTACATACGCTCCCTTTATA	6	<nnxnp>	nn	122	np	123		
>ref-6861	AGGAATAGGACTGCCACTCCTACGCAG	6	<lmxll>	ll	134	lm	119		
>ref-6866	CCTTGCCTCACCCTGCCTCCTCTTCCT	21	<nnxnp>	nn	124	np	149		
>ref-6868	GCTCCACCTACTCCACCTCCTGGTCTA	22	<nnxnp>	nn	67	np	188		
>ref-6907	ATTCCCTACACGCCCTCTCCCCTACCT	5	<hkxhk>	hh	64	hk	124	kk	45
>ref-6948	CCTACTAAAACAGATCCTCCCGGTGCA	23	<hkxhk>	hh	70	hk	122	kk	53
>ref-6949	ACTTCCCTCACAATTCCTCCCATCCTT	22	<hkxhk>	hh	57	hk	135	kk	68
>ref-6954	TATACGCATACTTGCTCTCCTCTTCTA	26	<hkxhk>	hh	79	hk	127	kk	61
>ref-6955	AGTCCCCAAACTCACCCTCCTTGTTTG	7	<lmxll>	ll	142	lm	128		
>ref-6966	CTCTGCGTTACTCTTCCTCCTACCCCA	21	<nnxnp>	nn	127	np	136		
>ref-6974	GTCTCCGGGACAGAGCCTCCTTGACCT	5	<lmxll>	ll	153	lm	116		
>ref-7001	AACCTAAATACCTTCCCTCCTGTAAAA	3	<lmxll>	ll	146	lm	97		
>ref-7007	CTCCCCAACACTCGTCCTCCTACCCTT	1	<lmxll>	ll	254	lm	1		
>ref-7019	ACCCCACTCACCGTCCCTCCATCCTCC	23	<lmxll>	ll	126	lm	136		
>ref-7024	ACGACCACTACGACCGCTCCAGCTACT	12	<hkxhk>	hh	11	hk	261	kk	0
>ref-7024	ACGACCACTACGACCGCTCCAGCTACT	24	<hkxhk>	hh	0	hk	273	kk	0
>ref-7024	ACGACCACTACGACCGCTCCAGCTACT	26	<hkxhk>	hh	3	hk	269	kk	0
>ref-7033	CGTAATAAAACTCGGGCTCCGTGAAGA	13	<lmxll>	ll	118	lm	136		
>ref-7053	TCTGTTCTTACCTTGCCTCCTCGCGTG	3	<hkxhk>	hh	0	hk	273	kk	0
>ref-7055	GACAAGTTGACTGTGTCTCCAAGTGAT	9	<nnxnp>	nn	122	np	136		
>ref-7055	GACAAGTTGACTGTGTCTCCAAGTGAT	24	<nnxnp>	nn	124	np	136		
>ref-7063	AGGAGGAACACACCCCCTCCACCGCCA	6	<lmxll>	ll	116	lm	150		
>ref-7065	GACTGACTGACTGACTCTCCCACGATG	9	<nnxnp>	nn	243	np	0		

>ref-7065	GACTGACTGACTGACTCTCCCACGATG	22	<nnxnp>	nn	110	np	119		
>ref-7085	CACCTCCCTACTCCCCCTCCATCCCCC	25	<nnxnp>	nn	121	np	135		
>ref-7102	CTTCATGGCACTTTACCTCCAGGCTTA	1	<nnxnp>	nn	272	np	0		
>ref-7102	CTTCATGGCACTTTACCTCCAGGCTTA	4	<hkxhk>	hh	1	hk	270	kk	0
>ref-7103	CATTCATGCACACCGCCTCCCTCTCCT	1	<lmxll>	ll	135	lm	136		
>ref-7131	TATCATTGCACTCCCCCTCCTCTCTAT	8	<lmxll>	ll	118	lm	136		
>ref-7185	CACCTACTCACCTGCTCTCCCTCCCCT	1	<nnxnp>	nn	120	np	139		
>ref-7200	CCATCATCAACGTCATCTCCGTCATCT	1	<hkxhk>	hh	0	hk	273	kk	0
>ref-7217	TCTACTTATACTTTCTCTCCCTCTCTC	22	<lmxll>	ll	135	lm	132		
>ref-7238	GGGAGGGGTACTGCCCCTCCCACTCAG	1	<hkxhk>	hh	0	hk	25	kk	241
>ref-7238	GGGAGGGGTACTGCCCCTCCCACTCAG	3	<lmxll>	ll	37	lm	219		
>ref-7238	GGGAGGGGTACTGCCCCTCCCACTCAG	23	<lmxll>	ll	6	lm	263		
>ref-7238	GGGAGGGGTACTGCCCCTCCCACTCAG	24	<hkxhk>	hh	0	hk	254	kk	13
>ref-7261	ATTAAAAATACTTCAGCTCCTTCCTCA	3	<lmxll>	ll	128	lm	105		
>ref-7261	ATTAAAAATACTTCAGCTCCTTCCTCA	21	<lmxll>	ll	129	lm	105		
>ref-7264	CTCCCTACTACCTTAACTCCTTCCCCT	26	<hkxhk>	hh	1	hk	271	kk	0
>ref-7276	TGGTGCAGAACTCCCACTCCATTTCTC	15	<hkxhk>	hh	14	hk	248	kk	0
>ref-7276	TGGTGCAGAACTCCCACTCCATTTCTC	21	<hkxhk>	hh	1	hk	269	kk	0
>ref-7300	CACTCCCCCACCTTCTCTCCCTCATCC	2	<hkxhk>	hh	2	hk	254	kk	4
>ref-7300	CACTCCCCCACCTTCTCTCCCTCATCC	23	<lmxll>	ll	182	lm	67		
>ref-7300	CACTCCCCCACCTTCTCTCCCTCATCC	26	<lmxll>	ll	183	lm	67		
>ref-7317	GGCCTCGATACCTGCGCTCCTTTTAAA	4	<nnxnp>	nn	56	np	199		
>ref-7317	GGCCTCGATACCTGCGCTCCTTTTAAA	14	<nnxnp>	nn	56	np	199		
>ref-7317	GGCCTCGATACCTGCGCTCCTTTTAAA	24	<hkxhk>	hh	0	hk	272	kk	1
>ref-7332	TAAATAACAACCCCTTCTCCGCGTCTG	15	<lmxll>	ll	88	lm	162		
>ref-7362	TACTGCCAAACGCTCTCTCCTCTATTC	27	<lmxll>	ll	119	lm	122		
>ref-7384	TAGATTCATACACATTCTCCCACACGC	12	<lmxll>	ll	141	lm	126		
>ref-7399	CTAACCTTTACTATCCCTCCTCTCCTC	25	<nnxnp>	nn	136	np	127		
>ref-7403	CTTTCTTATACATATCCTCCTTCTCAT	25	<lmxll>	ll	134	lm	120		
>ref-7437	TCACTTCTGACTGTAACTCCCTCTTCG	6	<lmxll>	ll	113	lm	118		
>ref-7438	ATCCATCTAACCCTTCCTCCCTTCCGA	8	<nnxnp>	nn	128	np	138		
>ref-7442	AGTCCTTAGACGTCAGCTCCCGAATAC	26	<lmxll>	ll	119	lm	135		
>ref-7455	CTTCCCTCCACCCTACCTCCTCCGCCC	9	<hkxhk>	hh	64	hk	123	kk	56
>ref-7465	CTTCATCGCACTCTCCCTCCCTATTCC	4	<nnxnp>	nn	136	np	129		
>ref-7480	CATGAATAAACGCATTCTCCATATAGA	12	<nnxnp>	nn	94	np	153		
>ref-7489	ACTACTACTACCCTCTCTCCCCTTCCT	1	<nnxnp>	nn	106	np	123		
>ref-7489	ACTACTACTACCCTCTCTCCCCTTCCT	4	<hkxhk>	hh	68	hk	128	kk	33
>ref-7504	CTCAATCACACTTCGACTCCTTACCTT	8	<nnxnp>	nn	121	np	144		

>ref-7534	ATATTTACTACAGGTTCTCCCCTAACA	25	<hkxhk>	hh	56	hk	137	kk	53
>ref-7558	GATTTTGTCACTGTCCCTCCTCTCTCT	22	<nnxnp>	nn	130	np	118		
>ref-7570	AGTATGTTTACTGCTGCTCCTGCTGAT	22	<lmxll>	ll	112	lm	142		
>ref-7570	AGTATGTTTACTGCTGCTCCTGCTGAT	26	<lmxll>	ll	137	lm	119		
>ref-7571	CGTAACCACACCCCAGCTCCGCCGACC	25	<lmxll>	ll	0	lm	262		
>ref-7576	CGTCGTTAAACTCCACCTCCGTTCTCT	5	<lmxll>	ll	134	lm	120		
>ref-7576	CGTCGTTAAACTCCACCTCCGTTCTCT	14	<nnxnp>	nn	119	np	139		
>ref-7584	CCCCCCAGCACACGGACTCCGGCGCGG	2	<hkxhk>	hh	71	hk	143	kk	49
>ref-7630	ACTGGGCTGACCTGACCTCCGCAACCT	8	<lmxll>	ll	99	lm	168		
>ref-7633	AGCACCTGAACCTAATCTCCCATTGTT	6	<lmxll>	ll	130	lm	128		
>ref-7653	TGACACACCACTCTCTCTCCAAACCCA	12	<nnxnp>	nn	95	np	145		
>ref-7666	ACCATCTACACTTTCCCTCCTCCTCCT	1	<hkxhk>	hh	0	hk	270	kk	1
>ref-7666	ACCATCTACACTTTCCCTCCTCCTCCT	4	<lmxll>	ll	134	lm	136		
>ref-7666	ACCATCTACACTTTCCCTCCTCCTCCT	22	<hkxhk>	hh	0	hk	262	kk	7
>ref-7666	ACCATCTACACTTTCCCTCCTCCTCCT	25	<hkxhk>	hh	7	hk	262	kk	0
>ref-7672	CTACCCAACACCCTCCCTCCCCTTATC	8	<nnxnp>	nn	141	np	78		
>ref-7682	AGGAGTGTCACAGACCCTCCTTCTGTA	1	<hkxhk>	hh	50	hk	155	kk	57
>ref-7706	ACAAACGAAACATCTCCTCCTGAATCC	1	<nnxnp>	nn	112	np	118		
>ref-7720	CTTCATTTAACCAACACTCCTTTAAGA	1	<nnxnp>	nn	172	np	82		
>ref-7741	ATGTAGCAGACTCCCGCTCCCAGCAGC	5	<hkxhk>	hh	131	hk	123	kk	0
>ref-7741	ATGTAGCAGACTCCCGCTCCCAGCAGC	14	<hkxhk>	hh	197	hk	59	kk	0
>ref-7741	ATGTAGCAGACTCCCGCTCCCAGCAGC	15	<hkxhk>	hh	0	hk	273	kk	0
>ref-7741	ATGTAGCAGACTCCCGCTCCCAGCAGC	21	<hkxhk>	hh	21	hk	240	kk	0
>ref-7741	ATGTAGCAGACTCCCGCTCCCAGCAGC	27	<hkxhk>	hh	132	hk	122	kk	0
>ref-7760	TTAAAATAGACATATGCTCCAGTATGT	21	<nnxnp>	nn	134	np	117		
>ref-7760	TTAAAATAGACATATGCTCCAGTATGT	23	<nnxnp>	nn	134	np	117		
>ref-7763	TCAAGTGCAACATTCCCTCCTTCGTCG	2	<hkxhk>	hh	4	hk	266	kk	0
>ref-7763	TCAAGTGCAACATTCCCTCCTTCGTCG	8	<hkxhk>	hh	0	hk	273	kk	0
>ref-7763	TCAAGTGCAACATTCCCTCCTTCGTCG	9	<hkxhk>	hh	30	hk	236	kk	0
>ref-7764	AGGTTTTACACAAACGCTCCCTCAGTT	16	<nnxnp>	nn	138	np	105		
>ref-7778	TTATCCTAGACCTTCCCTCCCCTTGTG	1	<hkxhk>	hh	2	hk	269	kk	0
>ref-7778	TTATCCTAGACCTTCCCTCCCCTTGTG	14	<hkxhk>	hh	2	hk	269	kk	0
>ref-7778	TTATCCTAGACCTTCCCTCCCCTTGTG	22	<hkxhk>	hh	99	hk	157	kk	0
>ref-7780	CCTCCTCAAACTACCACTCCAACTCTC	8	<nnxnp>	nn	259	np	0		
>ref-7782	CTGCCTCCCACCCTGCCTCCCTCATTT	15	<nnxnp>	nn	234	np	13		
>ref-7793	GTACATGCTACCAATACTCCATACCAG	1	<nnxnp>	nn	138	np	131		
>ref-7793	GTACATGCTACCAATACTCCATACCAG	14	<nnxnp>	nn	139	np	131		
>ref-7793	GTACATGCTACCAATACTCCATACCAG	23	<nnxnp>	nn	139	np	131		

>ref-7799	AAAAAGGACACGGATCCTCCTGGAGAG	6	<lmxll>	ll	122	lm	134		
>ref-7821	TCTTCCCCAACGTTCTCTCCTTCTCTC	21	<hkxhk>	hh	0	hk	271	kk	0
>ref-7821	TCTTCCCCAACGTTCTCTCCTTCTCTC	24	<nnxnp>	nn	9	np	263		
>ref-7821	TCTTCCCCAACGTTCTCTCCTTCTCTC	27	<nnxnp>	nn	80	np	182		
>ref-7823	CCAGCAATAACCTACCCTCCGCCACCT	13	<lmxll>	ll	115	lm	143		
>ref-7830	CTTCCTCTCACTTATGCTCCCTCCTTC	4	<hkxhk>	hh	53	hk	149	kk	52
>ref-7842	ATCTCCTCCACCCCCTCTCCCATCACC	13	<hkxhk>	hh	2	hk	269	kk	0
>ref-7843	ACTTCACCTACTCTTCCTCCTCAACCA	1	<lmxll>	ll	126	lm	139		
>ref-7843	ACTTCACCTACTCTTCCTCCTCAACCA	12	<nnxnp>	nn	123	np	137		
>ref-7854	AGCCACTCGACAGAAGCTCCACCAAAT	16	<lmxll>	ll	0	lm	272		
>ref-7858	CTCCATCTCACCATCACTCCCCCATCC	1	<lmxll>	ll	189	lm	73		
>ref-7858	CTCCATCTCACCATCACTCCCCCATCC	8	<lmxll>	ll	217	lm	34		
>ref-7858	CTCCATCTCACCATCACTCCCCCATCC	21	<lmxll>	ll	20	lm	246		
>ref-7858	CTCCATCTCACCATCACTCCCCCATCC	26	<lmxll>	ll	170	lm	96		
>ref-7858	CTCCATCTCACCATCACTCCCCCATCC	27	<lmxll>	ll	232	lm	32		
>ref-7871	CCCACAACCACACGTCCTCCCACAACC	9	<lmxll>	ll	220	lm	41		
>ref-7871	CCCACAACCACACGTCCTCCCACAACC	27	<hkxhk>	hh	63	hk	192	kk	0
>ref-7879	GGTCGTTTTACCACATCTCCGATCCAG	21	<nnxnp>	nn	116	np	144		
>ref-7881	CATCAATGCACAATCCCTCCTCTCTCC	4	<lmxll>	ll	105	lm	152		
>ref-7881	CATCAATGCACAATCCCTCCTCTCTCC	5	<lmxll>	ll	105	lm	152		
>ref-7899	ACCGACTGCACAGGTTCTCCTATATGT	4	<lmxll>	ll	140	lm	126		
>ref-7906	CACGTACTCACGCGCACTCCCCCTTTT	4	<lmxll>	ll	130	lm	136		
>ref-7906	CACGTACTCACGCGCACTCCCCCTTTT	27	<nnxnp>	nn	272	np	0		
>ref-7930	ACCTTCCCGACGTCCACTCCACACCGG	2	<nnxnp>	nn	154	np	112		
>ref-7945	CTCTTAGTCACCATCTCTCCCTCTATC	23	<lmxll>	ll	265	lm	0		
>ref-7948	GCCCCCTTCACCCTCACTCCCCCTTCC	15	<nnxnp>	nn	123	np	125		
>ref-7967	GTTAGTGTTACTTTTTCTCCCTCTTTC	13	<hkxhk>	hh	0	hk	271	kk	1
>ref-7975	AGCATCCGGACGCCGCCTCCTCACGCA	26	<lmxll>	ll	142	lm	131		
>ref-7978	CCTAACCCCACCATCTCTCCTCTCTCC	2	<hkxhk>	hh	9	hk	253	kk	0
>ref-7989	ACTGTCCCCACTTGCCCTCCATCGCCC	6	<lmxll>	ll	132	lm	131		
>ref-7990	CCAACCTCAACACACCCTCCGCTTACA	9	<hkxhk>	hh	53	hk	152	kk	44
>ref-7990	CCAACCTCAACACACCCTCCGCTTACA	12	<hkxhk>	hh	55	hk	152	kk	44
>ref-7995	GCAGGTTACACTCACGCTCCCTGACCT	1	<lmxll>	ll	121	lm	147		
>ref-7997	CCCATCCCAACCCCTCCTCCTTCGTCC	13	<lmxll>	ll	115	lm	143		
>ref-8056	TACACAAACACACAATCTCCCCTATTC	5	<hkxhk>	hh	57	hk	138	kk	49
>ref-8061	CGTCAGCATACCAGTACTCCTTCACAG	7	<hkxhk>	hh	13	hk	252	kk	0
>ref-8068	ATCTCTTACACTTTTTCTCCTTTCCCT	5	<nnxnp>	nn	160	np	85		
>ref-8068	ATCTCTTACACTTTTTCTCCTTTCCCT	25	<nnxnp>	nn	157	np	88		

>ref-8068	ATCTCTTACACTTTTTCTCCTTTCCCT	26	<lmxll>	ll	118	lm	132		
>ref-8075	CAATAGGCAACAGATCCTCCGCCGCGG	22	<hkxhk>	hh	50	hk	128	kk	76
>ref-8101	TCGTGTGATACCTTAGCTCCGGGGTAG	7	<lmxll>	ll	262	lm	0		
>ref-8124	CTCCCTGTAACATTGGCTCCAGTTTAG	1	<lmxll>	ll	22	lm	236		
>ref-8124	CTCCCTGTAACATTGGCTCCAGTTTAG	13	<hkxhk>	hh	0	hk	264	kk	4
>ref-8145	CCTGACCTGACCTAGCCTCCGCCGCCG	14	<lmxll>	ll	134	lm	134		
>ref-8153	CACTCTGCCACTCTCTCTCCAATGTCT	22	<nnxnp>	nn	266	np	0		
>ref-8157	GCCTAAAAAACATGAGCTCCAGATGAT	3	<hkxhk>	hh	33	hk	206	kk	0
>ref-8180	ATCCAGCCCACGATGACTCCTCCCTTC	7	<nnxnp>	nn	142	np	127		
>ref-8187	CGAATTAATACAGAATCTCCCCGGCTC	13	<lmxll>	ll	111	lm	110		
>ref-8192	CAGAGTTCGACTAGATCTCCGCTTACA	1	<nnxnp>	nn	136	np	128		
>ref-8192	CAGAGTTCGACTAGATCTCCGCTTACA	2	<nnxnp>	nn	136	np	128		
>ref-8215	GCAGCGTTGACGTCGCCTCCGTAATCA	24	<lmxll>	ll	106	lm	157		
>ref-8258	GTGACAACCACCCCCCCTCCTCCTCCT	3	<hkxhk>	hh	2	hk	257	kk	0
>ref-8258	GTGACAACCACCCCCCCTCCTCCTCCT	21	<hkxhk>	hh	0	hk	257	kk	2
>ref-8278	GTTTAATCCACCTTTTCTCCACCTCAA	9	<hkxhk>	hh	43	hk	206	kk	0
>ref-8278	GTTTAATCCACCTTTTCTCCACCTCAA	27	<hkxhk>	hh	2	hk	249	kk	0
>ref-8286	TGCCTCCCGACTCTTCCTCCCTCATTT	21	<nnxnp>	nn	139	np	116		
>ref-8321	CAATTATCTACGTACTCTCCTCCACTC	9	<nnxnp>	nn	115	np	127		
>ref-8324	CAAAGTGTGACTTTCTCTCCTTCCAAT	13	<nnxnp>	nn	103	np	117		
>ref-8341	GCAAGGACTACGTTAACTCCGGGGAAT	6	<nnxnp>	nn	123	np	128		
>ref-8341	GCAAGGACTACGTTAACTCCGGGGAAT	8	<nnxnp>	nn	123	np	128		
>ref-8343	ATCAGCGCCACCGCGTCTCCCCAGAAC	15	<lmxll>	ll	122	lm	126		
>ref-8343	ATCAGCGCCACCGCGTCTCCCCAGAAC	22	<nnxnp>	nn	159	np	82		
>ref-8343	ATCAGCGCCACCGCGTCTCCCCAGAAC	24	<nnxnp>	nn	157	np	84		
>ref-8364	CCTCCCTTCACTCGTTCTCCACCATCC	15	<lmxll>	ll	130	lm	133		
>ref-8368	ATAACGTATACGTAGACTCCCGGGCGA	1	<hkxhk>	hh	1	hk	267	kk	0
>ref-8368	ATAACGTATACGTAGACTCCCGGGCGA	5	<nnxnp>	nn	136	np	125		
>ref-8369	TGTTGAACTACCGAGTCTCCTGATTGT	22	<hkxhk>	hh	47	hk	225	kk	0
>ref-8370	TGTACCTTCACTACAACTCCTCCAAGA	21	<nnxnp>	nn	106	np	144		
>ref-8372	CGCCCAGGCACTGCCTCTCCCTATGTA	2	<nnxnp>	nn	132	np	134		
>ref-8372	CGCCCAGGCACTGCCTCTCCCTATGTA	3	<lmxll>	ll	135	lm	134		
>ref-8376	CAAAATGGAACTTTCCCTCCCTTTCAT	8	<hkxhk>	hh	50	hk	144	kk	55
>ref-8423	CTGTCTGCCACGGTGTCTCCCCCTTTT	12	<nnxnp>	nn	128	np	126		
>ref-8441	AGCACACTAACCATTCCTCCGTTGTCT	12	<lmxll>	ll	146	lm	124		
>ref-8450	TGATTATCCACACCATCTCCTGTGTTA	9	<hkxhk>	hh	54	hk	139	kk	55
>ref-8451	TACACGATAACACTTTCTCCTCACTTC	3	<lmxll>	ll	135	lm	132		
>ref-8465	AAGGAATAAACATCTGCTCCTGATATC	23	<lmxll>	ll	110	lm	135		

>ref-8465	AAGGAATAAACATCTGCTCCTGATATC	25	<lmxll>	ll	109	lm	136		
>ref-8481	TGCCCCATGACCTTTCCTCCCTCTCTC	9	<lmxll>	ll	264	lm	0		
>ref-8510	TACCCCAAAACCCTCTCTCCCCCACCA	9	<nnxnp>	nn	12	np	225		
>ref-8510	TACCCCAAAACCCTCTCTCCCCCACCA	27	<nnxnp>	nn	12	np	223		
>ref-8535	CATTCCTTCACCATCTCTCCTTTCCCC	8	<lmxll>	ll	248	lm	0		
>ref-8559	ATCACCCCCACCTCTTCTCCTCACCCC	1	<hkxhk>	hh	55	hk	191	kk	0
>ref-8559	ATCACCCCCACCTCTTCTCCTCACCCC	14	<hkxhk>	hh	58	hk	190	kk	0
>ref-8569	TCGTCCCTCACTCCTCCTCCCTCACTC	1	<nnxnp>	nn	256	np	11		
>ref-8569	TCGTCCCTCACTCCTCCTCCCTCACTC	2	<hkxhk>	hh	79	hk	177	kk	0
>ref-8569	TCGTCCCTCACTCCTCCTCCCTCACTC	3	<hkxhk>	hh	128	hk	140	kk	0
>ref-8569	TCGTCCCTCACTCCTCCTCCCTCACTC	16	<hkxhk>	hh	28	hk	236	kk	0
>ref-8579	CCACACACAACAACCTCTCCTATGAAC	14	<nnxnp>	nn	111	np	131		
>ref-8591	CATTCGGTTACAGTATCTCCCCGGTAA	13	<hkxhk>	hh	25	hk	232	kk	0
>ref-8591	CATTCGGTTACAGTATCTCCCCGGTAA	24	<hkxhk>	hh	0	hk	243	kk	21
>ref-8611	CCACCCTTCACCCCGCCTCCAACGCAC	24	<lmxll>	ll	125	lm	143		
>ref-8624	CAACCACCCACTCGCCCTCCCTCTCCA	2	<hkxhk>	hh	17	hk	243	kk	2
>ref-8624	CAACCACCCACTCGCCCTCCCTCTCCA	3	<hkxhk>	hh	17	hk	243	kk	2
>ref-8629	ACCGATGTGACTGCATCTCCTACTGAT	23	<nnxnp>	nn	126	np	114		
>ref-8661	TTACCAGACACCGGTGCTCCTCCTCCA	27	<hkxhk>	hh	61	hk	125	kk	61
>ref-8663	ACGTATCTGACCCGATCTCCTCTTGAA	23	<lmxll>	ll	115	lm	108		
>ref-8685	CACTTTCTCACAGCCTCTCCCTCTCTC	2	<nnxnp>	nn	101	np	139		
>ref-8685	CACTTTCTCACAGCCTCTCCCTCTCTC	12	<nnxnp>	nn	105	np	145		
>ref-8751	GCCCACATAACCCGCCCTCCTCCTCTC	14	<lmxll>	ll	130	lm	138		
>ref-8751	GCCCACATAACCCGCCCTCCTCCTCTC	15	<lmxll>	ll	130	lm	138		
>ref-8770	GGCACTCGCACGGCACCTCCTCCTTTA	26	<lmxll>	ll	116	lm	150		
>ref-8771	AAATAAAACACTCTATCTCCCTCCCTT	12	<lmxll>	ll	106	lm	141		
>ref-8790	CTGAGCTTAACTCAAGCTCCTTGCGAT	5	<nnxnp>	nn	132	np	134		
>ref-8794	GTATCCCCCACCCCTACTCCCTTTGTT	27	<nnxnp>	nn	116	np	135		
>ref-8803	CGGTCCGTCACCCTCTCTCCTCCCTCT	26	<lmxll>	ll	145	lm	127		
>ref-8814	ACAAGCAAGACGGAATCTCCAACAAGC	12	<nnxnp>	nn	125	np	141		
>ref-8839	ACGAAAGAGACGAGTCCTCCCATAACT	3	<lmxll>	ll	202	lm	56		
>ref-8839	ACGAAAGAGACGAGTCCTCCCATAACT	14	<lmxll>	ll	202	lm	56		
>ref-8846	CAAAACTAAACTCTCTCTCCCTACCTC	23	<hkxhk>	hh	2	hk	267	kk	1
>ref-8846	CAAAACTAAACTCTCTCTCCCTACCTC	27	<hkxhk>	hh	3	hk	266	kk	0
>ref-8852	ACAGAACACACGATATCTCCCTTCACA	8	<nnxnp>	nn	104	np	145		
>ref-8860	GGATACTAGACCTTGGCTCCCTTACTC	12	<nnxnp>	nn	121	np	144		
>ref-8861	TTGCTTTCCACAGGACCTCCGTCGCTC	2	<lmxll>	ll	0	lm	265		
>ref-8891	CCTAATCCAACTACAACTCCTAATCCA	5	<hkxhk>	hh	13	hk	256	kk	0

>ref-8891	CCTAATCCAACTACAACTCCTAATCCA	9	<hkxhk>	hh	0	hk	273	kk	0
>ref-8891	CCTAATCCAACTACAACTCCTAATCCA	12	<nnxnp>	nn	104	np	150		
>ref-8891	CCTAATCCAACTACAACTCCTAATCCA	23	<hkxhk>	hh	57	hk	199	kk	0
>ref-8893	CTCCGCAACACAATCTCTCCTTTTCTC	9	<nnxnp>	nn	133	np	133		
>ref-8897	AACTTAACTACTTGCCCTCCTCTACCA	16	<nnxnp>	nn	17	np	247		
>ref-8899	CCCCCTTCCACTGTCCCTCCTCTCCCC	12	<lmxll>	ll	165	lm	95		
>ref-8899	CCCCCTTCCACTGTCCCTCCTCTCCCC	13	<hkxhk>	hh	0	hk	242	kk	0
>ref-8899	CCCCCTTCCACTGTCCCTCCTCTCCCC	14	<nnxnp>	nn	270	np	0		
>ref-8899	CCCCCTTCCACTGTCCCTCCTCTCCCC	25	<lmxll>	ll	181	lm	73		
>ref-8899	CCCCCTTCCACTGTCCCTCCTCTCCCC	27	<hkxhk>	hh	3	hk	266	kk	0
>ref-8922	CCCTCCCCGACGCCATCTCCTGCCCCT	27	<hkxhk>	hh	0	hk	267	kk	1
>ref-8923	TCCCAAATGACCTGTCCTCCCGTATTA	4	<lmxll>	ll	120	lm	122		
>ref-8923	TCCCAAATGACCTGTCCTCCCGTATTA	5	<nnxnp>	nn	255	np	0		
>ref-8930	TCCCCTCACACGCCATCTCCCTCACAT	27	<hkxhk>	hh	0	hk	207	kk	56
>ref-8938	CAAAAACAAACAGAGCCTCCTCATCTC	15	<hkxhk>	hh	43	hk	131	kk	76
>ref-8941	GACAAACCTACACATCCTCCTACCCCC	7	<lmxll>	ll	122	lm	137		
>ref-8954	ACACTCAGAACAAGAACTCCTCATCCA	1	<lmxll>	ll	125	lm	142		
>ref-8956	TCAGTGGATACAGCAACTCCTCCTTGA	14	<nnxnp>	nn	141	np	105		
>ref-8975	CAGAAGCGCACGCACACTCCGACACAG	12	<nnxnp>	nn	148	np	121		
>ref-8976	GTTATTCCCACCTTCTCTCCTCTTATA	15	<lmxll>	ll	131	lm	95		
>ref-8976	GTTATTCCCACCTTCTCTCCTCTTATA	24	<lmxll>	ll	130	lm	95		
>ref-8991	TCTATGTATACATATACTCCTGGGTAT	3	<nnxnp>	nn	103	np	149		
>ref-9002	CCTCTCCGCACACCCCCTCCCCTTCCA	8	<hkxhk>	hh	49	hk	137	kk	76
>ref-9002	CCTCTCCGCACACCCCCTCCCCTTCCA	14	<hkxhk>	hh	49	hk	135	kk	78
>ref-9009	AGCACAAAAACAACAGCTCCATGATTT	22	<lmxll>	ll	131	lm	119		
>ref-9015	CCTTCCCACACACCCTCTCCATCTCTT	13	<nnxnp>	nn	129	np	140		
>ref-9040	TCCTTATTCACCTCCTCTCCGCCCGCT	22	<nnxnp>	nn	106	np	136		
>ref-9042	AGCCGGAGCACAGGTACTCCGTCATGA	3	<nnxnp>	nn	58	np	202		
>ref-9042	AGCCGGAGCACAGGTACTCCGTCATGA	8	<hkxhk>	hh	0	hk	267	kk	4
>ref-9057	CGCCCATTCACACCCACTCCTGCCCAC	21	<nnxnp>	nn	143	np	79		
>ref-9057	CGCCCATTCACACCCACTCCTGCCCAC	27	<nnxnp>	nn	145	np	77		
>ref-9061	GGACACAATACCCAACCTCCTCCCTCC	13	<nnxnp>	nn	138	np	117		
>ref-9064	CAGAGAACCACAGCTGCTCCTGTTTCC	15	<hkxhk>	hh	0	hk	226	kk	40
>ref-9072	ACTCTACGGACATGCACTCCGCGTTAT	21	<lmxll>	ll	32	lm	206		
>ref-9072	ACTCTACGGACATGCACTCCGCGTTAT	26	<lmxll>	ll	32	lm	206		
>ref-9097	CCTGCCTTCACCTCCTCTCCCCCCACC	25	<lmxll>	ll	73	lm	194		
>ref-9097	CCTGCCTTCACCTCCTCTCCCCCCACC	26	<lmxll>	ll	76	lm	192		
>ref-9122	CACACACACACAAATCCTCCAAAAATA	1	<lmxll>	ll	126	lm	111		

>ref-9161	TTATCTCCTACTCCCTCTCCCCCCTTA	26	<nnxnp>	nn	5	np	260		
>ref-9161	TTATCTCCTACTCCCTCTCCCCCCTTA	27	<nnxnp>	nn	4	np	260		
>ref-9178	CTGTGACCTACCCTGCCTCCTATGACC	1	<lmxll>	ll	261	lm	4		
>ref-9178	CTGTGACCTACCCTGCCTCCTATGACC	16	<hkxhk>	hh	0	hk	268	kk	3
>ref-9179	CCCAGACACACCCACACTCCGTAAGCT	3	<lmxll>	ll	112	lm	154		
>ref-9180	CCCGGGAGCACGTCAACTCCCGCCCAC	14	<lmxll>	ll	120	lm	141		
>ref-9184	CCATCTCCAACTTATCCTCCTCCACTT	24	<nnxnp>	nn	253	np	2		
>ref-9184	CCATCTCCAACTTATCCTCCTCCACTT	26	<nnxnp>	nn	259	np	0		
>ref-9186	TCCCCCATCACTCTACCTCCCTCTGTC	16	<lmxll>	ll	136	lm	135		
>ref-9188	TCCGCCCCGACTTTGACTCCAGCATGC	4	<hkxhk>	hh	0	hk	272	kk	0
>ref-9188	TCCGCCCCGACTTTGACTCCAGCATGC	6	<hkxhk>	hh	74	hk	187	kk	0
>ref-9188	TCCGCCCCGACTTTGACTCCAGCATGC	7	<hkxhk>	hh	74	hk	188	kk	0
>ref-9188	TCCGCCCCGACTTTGACTCCAGCATGC	8	<nnxnp>	nn	268	np	4		
>ref-9188	TCCGCCCCGACTTTGACTCCAGCATGC	22	<lmxll>	ll	61	lm	198		
>ref-9188	TCCGCCCCGACTTTGACTCCAGCATGC	26	<nnxnp>	nn	268	np	4		
>ref-9188	TCCGCCCCGACTTTGACTCCAGCATGC	27	<lmxll>	ll	32	lm	236		
>ref-9192	GAAGAGGAGACTTCCTCTCCCGTCGAG	4	<hkxhk>	hh	103	hk	137	kk	8
>ref-9194	TTTCTTCCCACCTTTCCTCCTCCCCTT	2	<lmxll>	ll	12	lm	260		
>ref-9194	TTTCTTCCCACCTTTCCTCCTCCCCTT	27	<lmxll>	ll	12	lm	260		
>ref-9203	AACAAAACGACAGCTACTCCTGCAACT	3	<nnxnp>	nn	144	np	124		
>ref-9211	ACTGTTTATACGATTGCTCCCTGTAGA	12	<nnxnp>	nn	263	np	0		
>ref-9227	CTCACCGCCACACCCTCTCCTCCCTAT	22	<hkxhk>	hh	0	hk	272	kk	0
>ref-9278	CCCTTCCTTACCCTTACTCCACCTCCC	23	<nnxnp>	nn	123	np	133		
>ref-9291	AGTATCAGAACCGAATCTCCAGCCATT	22	<lmxll>	ll	114	lm	123		
>ref-9306	ATAATACGAACCGATACTCCTCTAAAT	9	<hkxhk>	hh	51	hk	146	kk	54
>ref-9306	ATAATACGAACCGATACTCCTCTAAAT	22	<hkxhk>	hh	51	hk	146	kk	54
>ref-9316	TACCTGTTTACACAACCTCCAAGGTCT	5	<nnxnp>	nn	138	np	123		
>ref-9330	TCCCCCATTACCCATTCTCCGTCTCCC	6	<lmxll>	ll	85	lm	142		
>ref-9339	CTCTATCACACTTGTTCTCCTGTTATA	12	<nnxnp>	nn	16	np	247		
>ref-9343	GGGAAACAAACTGCGTCTCCTACATTC	2	<nnxnp>	nn	130	np	135		
>ref-9360	CATTATCCTACGTTCTCTCCTTGTCTC	15	<lmxll>	ll	130	lm	125		
>ref-9379	TTGTGAAAAACGGCGGCTCCCCGAATG	4	<lmxll>	ll	13	lm	252		
>ref-9379	TTGTGAAAAACGGCGGCTCCCCGAATG	22	<lmxll>	ll	13	lm	252		
>ref-9384	CTTCCTGTTACTATTCCTCCTCCTCTC	7	<nnxnp>	nn	243	np	12		
>ref-9384	CTTCCTGTTACTATTCCTCCTCCTCTC	27	<lmxll>	ll	55	lm	205		
>ref-9448	TTTACTCTCACTTACTCTCCTGGTAAT	23	<nnxnp>	nn	127	np	136		
>ref-9450	CTCCCCCTCACTATCACTCCTCCCCCC	1	<lmxll>	ll	265	lm	0		
>ref-9464	GTCCCCTCTACCGCTCCTCCTATCTAT	12	<nnxnp>	nn	131	np	133		

>ref-9482	CCCCACTTCACCCTTCCTCCTCCCCCT	24	<lmxll>	ll	130	lm	132		
>ref-9482	CCCCACTTCACCCTTCCTCCTCCCCCT	27	<lmxll>	ll	133	lm	128		
>ref-9488	TCTCTTGTGACTGCAGCTCCTCCCTGT	4	<lmxll>	ll	130	lm	137		
>ref-9512	CCTCCCCTTACTCTTTCTCCCCCTCTG	12	<hkxhk>	hh	0	hk	259	kk	4
>ref-9512	CCTCCCCTTACTCTTTCTCCCCCTCTG	27	<hkxhk>	hh	3	hk	262	kk	1
>ref-9521	AAGACAGCCACCGTGACTCCCCTAAGT	7	<lmxll>	ll	180	lm	53		
>ref-9521	AAGACAGCCACCGTGACTCCCCTAAGT	13	<hkxhk>	hh	1	hk	129	kk	130
>ref-9521	AAGACAGCCACCGTGACTCCCCTAAGT	27	<lmxll>	ll	179	lm	52		
>ref-9543	GATGACGTCACTGCCTCTCCCCGTGAC	6	<hkxhk>	hh	3	hk	270	kk	0
>ref-9543	GATGACGTCACTGCCTCTCCCCGTGAC	21	<hkxhk>	hh	28	hk	237	kk	0
>ref-9567	CTCCCCTCCACCCTCTCTCCCCCTAAC	9	<lmxll>	ll	23	lm	218		
>ref-9567	CTCCCCTCCACCCTCTCTCCCCCTAAC	24	<lmxll>	ll	17	lm	234		
>ref-9570	TCAGGCAGTACATTACCTCCGACTACT	6	<lmxll>	ll	116	lm	150		
>ref-9570	TCAGGCAGTACATTACCTCCGACTACT	25	<lmxll>	ll	116	lm	150		
>ref-9579	TGTATCTCGACTCCATCTCCCGCAAAC	6	<nnxnp>	nn	265	np	0		
>ref-9594	CCCTGTCTCACTCTTCCTCCCCTCTAC	8	<hkxhk>	hh	2	hk	264	kk	4
>ref-9594	CCCTGTCTCACTCTTCCTCCCCTCTAC	26	<hkxhk>	hh	4	hk	264	kk	2
>ref-9596	AACCTTCACACTAGCTCTCCGGAGAAA	1	<nnxnp>	nn	30	np	201		
>ref-9596	AACCTTCACACTAGCTCTCCGGAGAAA	3	<nnxnp>	nn	30	np	201		
>ref-9600	AGTGACGATACCCCTCCTCCTCCTCCT	25	<hkxhk>	hh	63	hk	129	kk	65
>ref-9600	AGTGACGATACCCCTCCTCCTCCTCCT	26	<lmxll>	ll	264	lm	1		
>ref-9615	TGTCACCCTACCTACTCTCCCTCTCCA	1	<hkxhk>	hh	0	hk	272	kk	1
>ref-9615	TGTCACCCTACCTACTCTCCCTCTCCA	5	<hkxhk>	hh	1	hk	272	kk	0
>ref-9620	TTAAGCCCTACATCTGCTCCCTCCCAC	1	<hkxhk>	hh	0	hk	173	kk	92
>ref-9620	TTAAGCCCTACATCTGCTCCCTCCCAC	3	<hkxhk>	hh	0	hk	272	kk	0
>ref-9620	TTAAGCCCTACATCTGCTCCCTCCCAC	21	<hkxhk>	hh	145	hk	121	kk	0
>ref-9620	TTAAGCCCTACATCTGCTCCCTCCCAC	24	<hkxhk>	hh	71	hk	194	kk	0
>ref-9620	TTAAGCCCTACATCTGCTCCCTCCCAC	25	<hkxhk>	hh	58	hk	211	kk	0
>ref-9622	ACTTCTGTTACTCCTACTCCTGTTGCA	23	<nnxnp>	nn	101	np	161		
>ref-9681	CCGGGAGCAACCACAGCTCCTTTTCAC	2	<nnxnp>	nn	121	np	144		
>ref-9684	ATTATTTCTACGCACACTCCCTCTACA	15	<nnxnp>	nn	131	np	128		
>ref-9709	CAGCCCGATACTGAAACTCCTTCGGCC	25	<lmxll>	ll	118	lm	140		
>ref-9716	TCTGCTCTCACCGAGTCTCCTGCTTCT	9	<nnxnp>	nn	124	np	144		
>ref-9716	TCTGCTCTCACCGAGTCTCCTGCTTCT	12	<nnxnp>	nn	131	np	139		
>ref-9719	CCTTTCCCTACTCCTCCTCCCTTATCT	25	<lmxll>	ll	140	lm	105		
>ref-9721	ATTTCAGCTACCAGCTCTCCGAAAATT	7	<hkxhk>	hh	0	hk	236	kk	25
>ref-9721	ATTTCAGCTACCAGCTCTCCGAAAATT	22	<nnxnp>	nn	269	np	1		
>ref-9721	ATTTCAGCTACCAGCTCTCCGAAAATT	26	<nnxnp>	nn	271	np	0		

>ref-9739	TCGTCTTCCACTTCCCCTCCCCTTCGT	13	<nnxnp>	nn	134	np	125		
>ref-9739	TCGTCTTCCACTTCCCCTCCCCTTCGT	23	<nnxnp>	nn	0	np	262		
>ref-9739	TCGTCTTCCACTTCCCCTCCCCTTCGT	26	<nnxnp>	nn	139	np	120		
>ref-9774	GACCATATCACCCGAGCTCCTTTGGTG	6	<nnxnp>	nn	135	np	133		
>ref-9783	ACCCCACACACCTTCCCTCCGAGATAT	9	<nnxnp>	nn	151	np	108		
>ref-9797	CGCCCCCTGACCCTCACTCCTCTCCAC	8	<nnxnp>	nn	262	np	0		
>ref-9799	TTCTCTCTAACCCCTTCTCCGTTTTCA	3	<nnxnp>	nn	267	np	1		
>ref-9805	CACTCACTCACTCACTCTCCATAAATC	3	<hkxhk>	hh	1	hk	202	kk	57
>ref-9805	CACTCACTCACTCACTCTCCATAAATC	23	<hkxhk>	hh	1	hk	192	kk	66
>ref-9805	CACTCACTCACTCACTCTCCATAAATC	24	<nnxnp>	nn	264	np	0		
>ref-9807	AACTCAACAACACAACCTCCGCAACAA	5	<lmxll>	ll	0	lm	271		
>ref-9828	GGCAAACATACAGACTCTCCTCCATGC	24	<lmxll>	ll	142	lm	108		
>ref-9833	GTGAATTCTACGTTGCCTCCTGCACTT	12	<lmxll>	ll	139	lm	112		
>ref-9858	TCTGGCAGGACTTCTCCTCCCTAACCC	14	<lmxll>	ll	128	lm	103		
>ref-9885	CCCCGCCTCACTTTCTCTCCTTCTCCC	4	<nnxnp>	nn	107	np	145		
>ref-9885	CCCCGCCTCACTTTCTCTCCTTCTCCC	5	<nnxnp>	nn	48	np	191		
>ref-9885	CCCCGCCTCACTTTCTCTCCTTCTCCC	16	<nnxnp>	nn	261	np	0		
>ref-9903	CGATGGATCACGATTTCTCCTCCCCGA	25	<nnxnp>	nn	139	np	116		
>ref-9906	TAACCCACCACTACCCCTCCACCCCCT	1	<lmxll>	ll	0	lm	266		
>ref-9906	TAACCCACCACTACCCCTCCACCCCCT	13	<lmxll>	ll	0	lm	266		
>ref-9938	TCTCGTACCACATTATCTCCCACCTAG	4	<lmxll>	ll	55	lm	199		
>ref-9945	TCCTTCCTCACTGTGGCTCCGAGGGTA	27	<lmxll>	ll	138	lm	123		
>ref-9982	CACTCGGGGACACTCTCTCCCTCTCTT	1	<hkxhk>	hh	4	hk	265	kk	0
>ref-10013	GACCGTCTTACTTGTTCTCCATGTGTA	2	<hkxhk>	hh	24	hk	122	kk	80
>ref-10040	GGGACACTCACAGCTGCTCCTACTGTC	24	<hkxhk>	hh	63	hk	147	kk	56
>ref-10047	TCCCCCCAAACCCTATCTCCCTGCCCT	7	<nnxnp>	nn	129	np	134		
>ref-10049	GGCAAACAAACCCTTCCTCCAATTCCT	21	<nnxnp>	nn	118	np	143		
>ref-10086	ACAACCTCTACGGAATCTCCTCTTGCA	8	<lmxll>	ll	122	lm	114		
>ref-10089	TCGCTCGGGACTTCCCCTCCCTCCCTC	23	<lmxll>	ll	109	lm	135		
>ref-10089	TCGCTCGGGACTTCCCCTCCCTCCCTC	24	<nnxnp>	nn	117	np	121		
>ref-10110	ATTAAAAGTACCTTGACTCCATCCCTC	9	<hkxhk>	hh	52	hk	127	kk	56
>ref-10122	CTCCCCCAAACCCTCTCTCCCCTCTCC	6	<lmxll>	ll	127	lm	136		
>ref-10126	AAGTGAAGGACATTATCTCCACTACTA	7	<hkxhk>	hh	68	hk	132	kk	44
>ref-10144	CGCACTTTTACACGCACTCCCACTCTC	14	<lmxll>	ll	114	lm	140		
>ref-10144	CGCACTTTTACACGCACTCCCACTCTC	22	<lmxll>	ll	107	lm	142		
>ref-10145	ACCTCCTGAACCCCTCCTCCTCCCCTC	1	<lmxll>	ll	2	lm	259		
>ref-10145	ACCTCCTGAACCCCTCCTCCTCCCCTC	9	<lmxll>	ll	2	lm	258		
>ref-10153	TGGGGTAAAACTGGTGCTCCTTTAGCT	23	<nnxnp>	nn	156	np	112		

>ref-10181	CTCCTCCCTACCTCTCCTCCTTTCTTC	5	<hkxhk>	hh	0	hk	272	kk	0
>ref-10181	CTCCTCCCTACCTCTCCTCCTTTCTTC	8	<hkxhk>	hh	0	hk	272	kk	0
>ref-10199	GTAAATCCAACCAAACCTCCTATAGTT	7	<lmxll>	ll	122	lm	110		
>ref-10230	GCAGAGAGTACTCCATCTCCGTGTAAA	13	<nnxnp>	nn	125	np	95		
>ref-10235	TGACCTTTGACCTTGACTCCTGTCACG	27	<nnxnp>	nn	262	np	0		
>ref-10246	CCACCTCCCACTCCACCTCCACTCCCC	3	<nnxnp>	nn	231	np	1		
>ref-10253	CACACACGCACCCCTCCTCCCCCCAAC	14	<nnxnp>	nn	111	np	145		
>ref-10268	TCTTCTTCCACTTCCACTCCACTTTCA	12	<hkxhk>	hh	3	hk	212	kk	51
>ref-10272	AGGTAGTTTACTATGGCTCCTCCTGTA	4	<lmxll>	ll	130	lm	110		
>ref-10286	ATTCCAACCACCTTTGCTCCCTTCAAT	26	<nnxnp>	nn	138	np	119		
>ref-10297	TATATGTCAACCAAGGCTCCTGGAGGC	2	<hkxhk>	hh	54	hk	154	kk	63
>ref-10302	CTTCCTAAAACAAGCACTCCTTCAGGT	23	<nnxnp>	nn	222	np	0		
>ref-10303	TCTTCCCTTACCCCTCCTCCTTGTCTC	26	<lmxll>	ll	117	lm	114		
>ref-10303	TCTTCCCTTACCCCTCCTCCTTGTCTC	27	<lmxll>	ll	118	lm	114		
>ref-10311	CATTCGTCCACTCCTCCTCCACCCTCC	13	<lmxll>	ll	264	lm	0		
>ref-10311	CATTCGTCCACTCCTCCTCCACCCTCC	14	<lmxll>	ll	264	lm	0		
>ref-10322	CATTGTACCACTTTTTCTCCCTCTCTC	5	<nnxnp>	nn	251	np	0		
>ref-10322	CATTGTACCACTTTTTCTCCCTCTCTC	13	<nnxnp>	nn	246	np	0		
>ref-10339	TTCATCATTACCATCCCTCCCTCCCTG	5	<nnxnp>	nn	113	np	110		
>ref-10347	AAACCTTAAACACCAGCTCCTCTTCCC	12	<nnxnp>	nn	107	np	114		
>ref-10366	ACTGCCTCCACACAGTCTCCCTCATAA	15	<nnxnp>	nn	150	np	107		
>ref-10366	ACTGCCTCCACACAGTCTCCCTCATAA	23	<nnxnp>	nn	131	np	140		
>ref-10366	ACTGCCTCCACACAGTCTCCCTCATAA	25	<nnxnp>	nn	126	np	144		
>ref-10376	CCATACTCGACGTCCCCTCCCACACTC	15	<lmxll>	ll	146	lm	120		
>ref-10402	CTTGGGCTTACTGATACTCCTTGCCGG	6	<nnxnp>	nn	121	np	134		
>ref-10412	ACCCCGCTCACCTACCCTCCCCCTCGA	15	<lmxll>	ll	116	lm	139		
>ref-10412	ACCCCGCTCACCTACCCTCCCCCTCGA	24	<nnxnp>	nn	132	np	125		
>ref-10441	TCCCTCCCTACCCTCACTCCATCCATC	15	<nnxnp>	nn	262	np	0		
>ref-10441	TCCCTCCCTACCCTCACTCCATCCATC	16	<nnxnp>	nn	164	np	74		
>ref-10441	TCCCTCCCTACCCTCACTCCATCCATC	21	<hkxhk>	hh	1	hk	215	kk	24
>ref-10441	TCCCTCCCTACCCTCACTCCATCCATC	25	<nnxnp>	nn	148	np	77		
>ref-10448	TCCTGACCAACTTTATCTCCCCCTCAA	6	<lmxll>	ll	134	lm	123		
>ref-10477	GTCTGTCACACAGTGCCTCCCTATTCT	9	<nnxnp>	nn	136	np	111		
>ref-10510	TCCCTCCTTACCTTCCCTCCCTCTAAG	12	<lmxll>	ll	131	lm	97		
>ref-10521	TGTTTATGAACACTTGCTCCTGTTCAT	16	<hkxhk>	hh	0	hk	270	kk	0
>ref-10525	TTCCCTTCTACCTCCACTCCACACCCC	8	<lmxll>	ll	137	lm	127		
>ref-10528	ACACACACGACGCCATCTCCAGCAGCC	2	<hkxhk>	hh	63	hk	142	kk	58
>ref-10541	CTTGAGTAAACAAATCCTCCATACTCT	25	<nnxnp>	nn	112	np	125		

>ref-10541	CTTGAGTAAACAAATCCTCCATACTCT	26	<nnxnp>	nn	113	np	124		
>ref-10543	TAAGAAGCGACTGGTGCTCCCGTGGTT	3	<hkxhk>	hh	78	hk	133	kk	55
>ref-10547	ACTCGACGCACGCCAGCTCCTACAGAC	6	<hkxhk>	hh	59	hk	156	kk	55
>ref-10555	CTGACGGGCACAGCAGCTCCGTGGGAT	22	<lmxll>	ll	124	lm	136		
>ref-10580	CCTCGTAGAACGGCTCCTCCACCCAAG	7	<lmxll>	ll	132	lm	118		
>ref-10585	CGGGAGAGCACCTACACTCCTTTAGAT	16	<nnxnp>	nn	131	np	127		
>ref-10606	ACATTTAGTACATATTCTCCTCCTTCT	4	<nnxnp>	nn	126	np	132		
>ref-10611	TTCTCTCTCACCATTTCTCCCATTCCT	15	<hkxhk>	hh	0	hk	273	kk	0
>ref-10626	TGTTTCACCACCCACTCTCCAGTCCAT	2	<lmxll>	ll	133	lm	131		
>ref-10647	AGAAGAGAAACAGAGACTCCATACATT	7	<lmxll>	ll	141	lm	103		
>ref-10658	ACCTCCTTTACAGCATCTCCTCCGAGT	13	<hkxhk>	hh	0	hk	197	kk	54
>ref-10672	CTCTTCTCTACCCTTCCTCCTTCCTCT	14	<hkxhk>	hh	0	hk	271	kk	0
>ref-10672	CTCTTCTCTACCCTTCCTCCTTCCTCT	27	<hkxhk>	hh	0	hk	271	kk	0
>ref-10680	CCAGCTCCTACCACAACTCCAGCTCCA	9	<hkxhk>	hh	0	hk	200	kk	35
>ref-10680	CCAGCTCCTACCACAACTCCAGCTCCA	13	<nnxnp>	nn	176	np	64		
>ref-10680	CCAGCTCCTACCACAACTCCAGCTCCA	24	<hkxhk>	hh	0	hk	231	kk	15
>ref-10691	GCTCAACACACGTCATCTCCATAATAC	3	<hkxhk>	hh	64	hk	127	kk	51
>ref-10727	CCCGTGTACACAAGAACTCCATACACT	14	<lmxll>	ll	132	lm	104		
>ref-10733	CTGTTCAGGACCCCAACTCCATGCTTG	12	<hkxhk>	hh	0	hk	273	kk	0
>ref-10733	CTGTTCAGGACCCCAACTCCATGCTTG	27	<hkxhk>	hh	0	hk	273	kk	0
>ref-10737	CAATCCCTCACTGTTTCTCCCCTTCTC	1	<lmxll>	ll	123	lm	130		
>ref-10740	AGTCCTCCCACACTGTCTCCCTCATCC	24	<lmxll>	ll	257	lm	0		
>ref-10740	AGTCCTCCCACACTGTCTCCCTCATCC	25	<lmxll>	ll	259	lm	0		
>ref-10744	GGCGGGTCGACCTGTGCTCCAACGACA	26	<lmxll>	ll	141	lm	132		
>ref-10748	GATACTTCCACGTGGCCTCCTCCCCCT	4	<hkxhk>	hh	51	hk	134	kk	65
>ref-10751	TCCAACCCTACTGCACCTCCCTTGTTA	15	<lmxll>	ll	129	lm	112		
>ref-10781	TGTTTTACCACACGTCCTCCCGCGCAG	13	<nnxnp>	nn	266	np	0		
>ref-10786	CCACTTCTCACGTTCTCTCCACTTCTC	4	<hkxhk>	hh	4	hk	267	kk	0
>ref-10786	CCACTTCTCACGTTCTCTCCACTTCTC	14	<nnxnp>	nn	162	np	78		
>ref-10791	ACTCTCTCCACCTCTCCTCCCCGTCCT	1	<nnxnp>	nn	4	np	264		
>ref-10791	ACTCTCTCCACCTCTCCTCCCCGTCCT	14	<nnxnp>	nn	4	np	264		
>ref-10801	CTGGGGTTCACGTGGACTCCATGTATT	1	<nnxnp>	nn	127	np	123		
>ref-10817	CTTTTCTCAACTCCCTCTCCTGTTCTT	2	<hkxhk>	hh	58	hk	130	kk	52
>ref-10830	ACCCCATTCACTTACACTCCAATACCT	13	<nnxnp>	nn	99	np	120		
>ref-10833	TATCCTCCTACTGACCCTCCTACCCTC	14	<hkxhk>	hh	1	hk	271	kk	0
>ref-10833	TATCCTCCTACTGACCCTCCTACCCTC	23	<hkxhk>	hh	1	hk	271	kk	0
>ref-10836	TCTTCTCTAACCGCAGCTCCTTTGTTC	24	<lmxll>	ll	145	lm	119		
>ref-10842	TGCAGAGAAACATCATCTCCTTACCTG	7	<lmxll>	ll	113	lm	150		

>ref-10880	CCTCCCCCTACCAATCCTCCTTCCCCC	13	<hkxhk>	hh	0	hk	263	kk	5
>ref-10880	CCTCCCCCTACCAATCCTCCTTCCCCC	24	<hkxhk>	hh	1	hk	270	kk	0
>ref-10883	TATCGGGGAACTGTCTCTCCTGATTCA	3	<nnxnp>	nn	131	np	118		
>ref-10883	TATCGGGGAACTGTCTCTCCTGATTCA	5	<nnxnp>	nn	106	np	136		
>ref-10893	TTCTCACTCACTTACGCTCCGCCCACT	2	<lmxll>	ll	146	lm	123		
>ref-10918	TTGTTAACGACTCAATCTCCTAGCCCT	7	<hkxhk>	hh	52	hk	131	kk	76
>ref-10925	ACCCTCCCAACCTTCTCTCCCCTCTCC	5	<nnxnp>	nn	83	np	151		
>ref-10925	ACCCTCCCAACCTTCTCTCCCCTCTCC	14	<nnxnp>	nn	82	np	155		
>ref-10927	CTCACCGTTACCCCCCCTCCTACCCTT	1	<nnxnp>	nn	67	np	154		
>ref-10927	CTCACCGTTACCCCCCCTCCTACCCTT	7	<lmxll>	ll	4	lm	240		
>ref-10936	CTCACCGCCACTTTTCCTCCTTCCTAA	26	<hkxhk>	hh	57	hk	143	kk	64
>ref-10936	CTCACCGCCACTTTTCCTCCTTCCTAA	27	<hkxhk>	hh	57	hk	143	kk	65
>ref-10959	TCTGGAGGAACTTGCACTCCTTACTCG	3	<hkxhk>	hh	0	hk	214	kk	49
>ref-10959	TCTGGAGGAACTTGCACTCCTTACTCG	23	<hkxhk>	hh	0	hk	272	kk	0
>ref-10989	CATCTTGCAACTTGCTCTCCTCCTCCT	22	<nnxnp>	nn	142	np	128		
>ref-10998	ACTCCCGATACCTTGTCTCCATCTCAC	6	<nnxnp>	nn	101	np	169		
>ref-11007	GCGTCTGTCACGTAGGCTCCATATCGG	2	<hkxhk>	hh	127	hk	112	kk	1
>ref-11009	CCTTTGTTTACTCCCTCTCCCCCTTCC	12	<lmxll>	ll	126	lm	127		
>ref-11015	GTTCCCCCAACACCCCCTCCTCCCAAA	3	<hkxhk>	hh	4	hk	170	kk	73
>ref-11015	GTTCCCCCAACACCCCCTCCTCCCAAA	14	<hkxhk>	hh	4	hk	170	kk	72
>ref-11018	TTGTCATCTACCTTCTCTCCCTATCAT	26	<lmxll>	ll	0	lm	254		
>ref-11027	TCGTCCCTCACCCCCTCTCCCCCTTCC	6	<nnxnp>	nn	265	np	0		
>ref-11027	TCGTCCCTCACCCCCTCTCCCCCTTCC	7	<lmxll>	ll	150	lm	97		
>ref-11028	TTCCCTCCCACGTTTCCTCCGCCCATC	2	<hkxhk>	hh	53	hk	128	kk	63
>ref-11028	TTCCCTCCCACGTTTCCTCCGCCCATC	9	<lmxll>	ll	124	lm	128		
>ref-11028	TTCCCTCCCACGTTTCCTCCGCCCATC	21	<lmxll>	ll	117	lm	133		
>ref-11040	CACCTATTCACTATCCCTCCCATCACC	9	<lmxll>	ll	152	lm	87		
>ref-11051	TACATATGCACCGCCCCTCCCCCCCTT	2	<nnxnp>	nn	32	np	230		
>ref-11051	TACATATGCACCGCCCCTCCCCCCCTT	13	<nnxnp>	nn	31	np	231		
>ref-11056	ATGGTGATCACGTGTGCTCCGCCTGAA	26	<nnxnp>	nn	146	np	123		
>ref-11089	GCTCCGCCAACCAGCACTCCAAAACAA	8	<lmxll>	ll	120	lm	107		
>ref-11095	TTATTACCCACAATGCCTCCAGAGTAA	2	<nnxnp>	nn	125	np	142		
>ref-11097	GCACTGTATACGCTCCCTCCCAGTCTG	22	<lmxll>	ll	137	lm	128		
>ref-11100	GCTCGCCTGACCGCCTCTCCTTGCCTC	3	<nnxnp>	nn	122	np	141		
>ref-11111	GTAACGCCCACTGACCCTCCTCTTGTC	13	<hkxhk>	hh	57	hk	154	kk	57
>ref-11111	GTAACGCCCACTGACCCTCCTCTTGTC	15	<nnxnp>	nn	132	np	140		
>ref-11111	GTAACGCCCACTGACCCTCCTCTTGTC	22	<lmxll>	ll	136	lm	134		
>ref-11124	AATCAAACTACCCTTTCTCCCATCCCT	4	<nnxnp>	nn	262	np	0		

>ref-11126	AAGTTAAACACCAGCTCTCCGTAATCC	3	<nnxnp>	nn	122	np	113		
>ref-11129	ATGCCTGTCACTGGCACTCCTGTCGCC	21	<hkxhk>	hh	83	hk	136	kk	49
>ref-11140	TCTTAAGCCACGCCCCCTCCTCATGGT	27	<lmxll>	ll	115	lm	155		
>ref-11217	TCTACCCCTACTCACACTCCCGGTGCT	21	<lmxll>	ll	103	lm	121		
>ref-11222	TCATCTTTCACTCCCTCTCCCTCCTCC	22	<lmxll>	ll	6	lm	262		
>ref-11222	TCATCTTTCACTCCCTCTCCCTCCTCC	24	<lmxll>	ll	27	lm	231		
>ref-11234	CGCGGCGGTACTCCTGCTCCTGATACC	16	<nnxnp>	nn	144	np	127		
>ref-11237	CCACAGGGCACGGTCTCTCCCTCCCAC	27	<nnxnp>	nn	127	np	139		
>ref-11239	GCAACAGAGACGATTCCTCCGAAAGCG	23	<nnxnp>	nn	271	np	0		
>ref-11252	ACAAAAGCCACCCTTACTCCAACACTT	14	<hkxhk>	hh	1	hk	260	kk	1
>ref-11258	CCTTTCTACACCTTTTCTCCTCTCCCC	3	<lmxll>	ll	7	lm	260		
>ref-11258	CCTTTCTACACCTTTTCTCCTCTCCCC	14	<lmxll>	ll	7	lm	260		
>ref-11264	CTTCCTTCTACTCCCGCTCCCTACTCT	5	<nnxnp>	nn	134	np	135		
>ref-11271	TACTTTTCTACAGCAACTCCTTCCTCT	3	<nnxnp>	nn	141	np	123		
>ref-11299	TCTTTAGCCACACCCCCTCCCCCTCTC	7	<lmxll>	ll	11	lm	242		
>ref-11299	TCTTTAGCCACACCCCCTCCCCCTCTC	14	<lmxll>	ll	13	lm	243		
>ref-11299	TCTTTAGCCACACCCCCTCCCCCTCTC	26	<hkxhk>	hh	2	hk	187	kk	58
>ref-11308	AATCGTTTCACGGCTTCTCCCTCTTTC	2	<lmxll>	ll	120	lm	125		
>ref-11308	AATCGTTTCACGGCTTCTCCCTCTTTC	4	<lmxll>	ll	121	lm	124		
>ref-11313	AAAGAGCCTACGTTATCTCCACCAACA	12	<lmxll>	ll	133	lm	125		
>ref-11327	TTCCCTTTAACTTCCTCTCCCTCCCCT	2	<nnxnp>	nn	127	np	125		
>ref-11327	TTCCCTTTAACTTCCTCTCCCTCCCCT	5	<nnxnp>	nn	129	np	124		
>ref-11330	ATTGCAGCCACCACTCCTCCGGGTAGT	14	<hkxhk>	hh	0	hk	273	kk	0
>ref-11330	ATTGCAGCCACCACTCCTCCGGGTAGT	24	<nnxnp>	nn	148	np	121		
>ref-11344	AATTCCTACACACCCCCTCCCCTCGCT	14	<nnxnp>	nn	102	np	152		
>ref-11357	TACTTACTTACTCTCTCTCCTTCTCCC	9	<nnxnp>	nn	263	np	0		
>ref-11416	CCCTACCCCACTCCCTCTCCATTTCCC	5	<lmxll>	ll	12	lm	227		
>ref-11416	CCCTACCCCACTCCCTCTCCATTTCCC	15	<lmxll>	ll	24	lm	207		
>ref-11419	CCATCCTACACACCCCCTCCCCCTATC	24	<hkxhk>	hh	1	hk	270	kk	0
>ref-11449	GTGTAGTCGACTATCTCTCCTCTGTCT	5	<hkxhk>	hh	70	hk	128	kk	59
>ref-11449	GTGTAGTCGACTATCTCTCCTCTGTCT	26	<lmxll>	ll	133	lm	127		
>ref-11472	GGAACACGAACTCTACCTCCAGCAGCG	21	<hkxhk>	hh	57	hk	113	kk	76
>ref-11502	TGCAGTCTCACTCCCTCTCCCCTCTCC	1	<nnxnp>	nn	270	np	0		
>ref-11502	TGCAGTCTCACTCCCTCTCCCCTCTCC	8	<lmxll>	ll	6	lm	259		
>ref-11515	CTGTCTCTCACCCTCTCTCCAACCCCC	3	<nnxnp>	nn	30	np	209		
>ref-11515	CTGTCTCTCACCCTCTCTCCAACCCCC	12	<nnxnp>	nn	108	np	138		
>ref-11547	CGACGCCCCACGCACTCTCCTCCTTGA	22	<lmxll>	ll	135	lm	135		
>ref-11555	CAAGACAGCACGTCTCCTCCTCCCCCT	4	<lmxll>	ll	122	lm	144		

>ref-11562	GTGTCTCTCACTGTCTCTCCTATCTCC	27	<lmxll>	ll	117	lm	139		
>ref-11565	ACCCGGCCCACAGACACTCCTTGCCTC	5	<nnxnp>	nn	117	np	141		
>ref-11591	CTCTCTTCTACTCTTCCTCCCTTCTCC	4	<nnxnp>	nn	89	np	157		
>ref-11591	CTCTCTTCTACTCTTCCTCCCTTCTCC	7	<lmxll>	ll	255	lm	2		
>ref-11591	CTCTCTTCTACTCTTCCTCCCTTCTCC	12	<nnxnp>	nn	100	np	149		
>ref-11591	CTCTCTTCTACTCTTCCTCCCTTCTCC	23	<lmxll>	ll	254	lm	5		
>ref-11600	TTCTCGGAGACTGATGCTCCCTCCAAA	26	<hkxhk>	hh	57	hk	126	kk	47
>ref-11610	AGCGTCTGCACTCGCCCTCCTCCCTTC	21	<lmxll>	ll	122	lm	147		
>ref-11622	TATCAGATGACAGTTTCTCCGGATACT	7	<lmxll>	ll	131	lm	128		
>ref-11624	TCACGCAACACACAACCTCCGTCTTTG	5	<lmxll>	ll	162	lm	96		
>ref-11624	TCACGCAACACACAACCTCCGTCTTTG	26	<lmxll>	ll	86	lm	158		
>ref-11635	CACACCTCCACACCTGCTCCTTTCTTC	2	<hkxhk>	hh	3	hk	115	kk	112
>ref-11638	TCACATAACACAATTGCTCCGTCAGTT	7	<nnxnp>	nn	136	np	127		
>ref-11650	ACCTCAAGAACACAGACTCCAGCAAAT	9	<lmxll>	ll	1	lm	257		
>ref-11650	ACCTCAAGAACACAGACTCCAGCAAAT	13	<lmxll>	ll	1	lm	257		
>ref-11659	TTTATTTCCACCCTTCCTCCTTCTCTC	22	<lmxll>	ll	132	lm	128		
>ref-11680	TCATCTGCAACTCCCACTCCCACTTTC	26	<hkxhk>	hh	14	hk	245	kk	0
>ref-11680	TCATCTGCAACTCCCACTCCCACTTTC	27	<hkxhk>	hh	0	hk	245	kk	15
>ref-11682	GATCCACACACTGCTCCTCCGCCCTGA	4	<hkxhk>	hh	55	hk	119	kk	50
>ref-11683	TTCTCTCAAACTCTATCTCCTCTTCCT	2	<nnxnp>	nn	127	np	133		
>ref-11691	CACTCCTTCACCCCTTCTCCTCCTACT	8	<hkxhk>	hh	5	hk	122	kk	106
>ref-11691	CACTCCTTCACCCCTTCTCCTCCTACT	12	<nnxnp>	nn	110	np	139		
>ref-11691	CACTCCTTCACCCCTTCTCCTCCTACT	13	<nnxnp>	nn	158	np	90		
>ref-11729	CCCCCACCCACCCGACCTCCTTCTCTG	4	<nnxnp>	nn	110	np	145		
>ref-11735	CCATCACTAACCCCCTCTCCTCCCCGC	27	<lmxll>	ll	136	lm	117		
>ref-11758	TTTCCACCAACTTTTCCTCCTTCTCCT	1	<nnxnp>	nn	242	np	1		
>ref-11771	ATGGTATAGACAGCAGCTCCAGGGTAT	21	<nnxnp>	nn	267	np	0		
>ref-11784	CCCAGCATCACTCATTCTCCTCCTTTT	5	<nnxnp>	nn	129	np	134		
>ref-11788	CCTATGAAAACGGCACCTCCTGTGCAA	9	<nnxnp>	nn	257	np	0		
>ref-11808	AAAAGACGGACATAAACTCCGTACATA	5	<hkxhk>	hh	70	hk	183	kk	0
>ref-11809	TTCTTTCACACCCCTTCTCCTCCCCTC	12	<nnxnp>	nn	238	np	0		
>ref-11809	TTCTTTCACACCCCTTCTCCTCCCCTC	22	<nnxnp>	nn	241	np	0		
>ref-11809	TTCTTTCACACCCCTTCTCCTCCCCTC	26	<nnxnp>	nn	238	np	0		
>ref-11820	GTGTCGATAACATTATCTCCTTTCCCC	6	<lmxll>	ll	124	lm	130		
>ref-11845	TGTATATAGACTTTACCTCCCCAGGAC	21	<nnxnp>	nn	108	np	123		
>ref-11862	TCCACATTCACACATTCTCCTCATCCA	3	<nnxnp>	nn	273	np	0		
>ref-11862	TCCACATTCACACATTCTCCTCATCCA	24	<nnxnp>	nn	269	np	3		
>ref-11867	AGGGACAGCACCCCTCCTCCCAGCATA	13	<hkxhk>	hh	11	hk	257	kk	0

>ref-11867	AGGGACAGCACCCCTCCTCCCAGCATA	22	<hkxhk>	hh	2	hk	270	kk	0
>ref-11870	TATATATATACGGATGCTCCCACACAC	22	<lmxll>	ll	126	lm	117		
>ref-11880	GAAGTGAAAACACAGTCTCCTAATTAT	1	<lmxll>	ll	95	lm	136		
>ref-11896	TTCTTCTTAACTGGCCCTCCCCAACTT	14	<lmxll>	ll	138	lm	107		
>ref-11896	TTCTTCTTAACTGGCCCTCCCCAACTT	23	<lmxll>	ll	140	lm	107		
>ref-11902	ATTATCATTACCATCACTCCCATTATT	4	<hkxhk>	hh	193	hk	41	kk	0
>ref-11902	ATTATCATTACCATCACTCCCATTATT	21	<hkxhk>	hh	0	hk	202	kk	39
>ref-11902	ATTATCATTACCATCACTCCCATTATT	24	<lmxll>	ll	0	lm	221		
>ref-11904	CTTATCCCAACCCCCCCTCCGTTACCT	12	<lmxll>	ll	130	lm	122		
>ref-11905	TCCCTCTTCACTCATCCTCCTCGTTTC	6	<lmxll>	ll	148	lm	112		
>ref-11908	TCGTTGAGAACCGACTCTCCAAACACC	3	<nnxnp>	nn	135	np	132		
>ref-11918	TTCTTTCCCACCCTCTCTCCTCCTCTT	1	<nnxnp>	nn	7	np	260		
>ref-11918	TTCTTTCCCACCCTCTCTCCTCCTCTT	3	<lmxll>	ll	35	lm	214		
>ref-11918	TTCTTTCCCACCCTCTCTCCTCCTCTT	5	<lmxll>	ll	35	lm	215		
>ref-11918	TTCTTTCCCACCCTCTCTCCTCCTCTT	12	<nnxnp>	nn	7	np	256		
>ref-11940	ATCTCTTCAACCCTTCCTCCACCTTCC	21	<nnxnp>	nn	114	np	143		
>ref-11944	GTCTTCTCTACCAACTCTCCTCCTTCT	25	<lmxll>	ll	147	lm	112		
>ref-11953	CCCTTTGCGACTGACGCTCCGCAAGAT	14	<nnxnp>	nn	127	np	132		
>ref-11971	TCTATATGTACATATCCTCCTCTTCCT	23	<lmxll>	ll	118	lm	143		
>ref-12021	TCTTTATGGACTTCTCCTCCTCCTCTT	14	<nnxnp>	nn	142	np	124		
>ref-12067	ATTATCTTTACCTTCTCTCCCTCCACA	2	<nnxnp>	nn	114	np	134		
>ref-12067	ATTATCTTTACCTTCTCTCCCTCCACA	8	<nnxnp>	nn	113	np	134		
>ref-12076	CTTATCCCCACCCGCTCTCCCTCGCGC	2	<lmxll>	ll	149	lm	113		
>ref-12087	TAATGATCTACCTCCTCTCCTTCATCT	5	<nnxnp>	nn	146	np	113		
>ref-12090	TATGCTTCCACTTCATCTCCCGTTATT	25	<lmxll>	ll	109	lm	154		
>ref-12115	AATCATTCCACTCTTCCTCCCTCCCCC	22	<lmxll>	ll	259	lm	0		
>ref-12155	TCATCATCAACATCATCTCCATCATCA	3	<lmxll>	ll	247	lm	22		
>ref-12155	TCATCATCAACATCATCTCCATCATCA	4	<hkxhk>	hh	0	hk	257	kk	12
>ref-12155	TCATCATCAACATCATCTCCATCATCA	6	<hkxhk>	hh	92	hk	171	kk	0
>ref-12155	TCATCATCAACATCATCTCCATCATCA	7	<nnxnp>	nn	169	np	92		
>ref-12155	TCATCATCAACATCATCTCCATCATCA	22	<lmxll>	ll	122	lm	131		
>ref-12155	TCATCATCAACATCATCTCCATCATCA	24	<lmxll>	ll	152	lm	115		
>ref-12155	TCATCATCAACATCATCTCCATCATCA	25	<hkxhk>	hh	0	hk	272	kk	0
>ref-12181	AACACTCTCACCTCCTCTCCTCCATCT	5	<hkxhk>	hh	46	hk	143	kk	55
>ref-12184	CAGATACACACATGCTCTCCATTTGAT	3	<lmxll>	ll	106	lm	152		
>ref-12184	CAGATACACACATGCTCTCCATTTGAT	5	<lmxll>	ll	106	lm	152		
>ref-12192	TATGCACTGACTATTCCTCCTAATACA	9	<nnxnp>	nn	116	np	114		
>ref-12200	ACCCCCTTTACATGGCCTCCTCGCCCC	22	<nnxnp>	nn	140	np	129		

>ref-12234	TCCATTGTCACGTCCTCTCCCCTGCTG	6	<hkxhk>	hh	63	hk	144	kk	56
>ref-12247	ACCTACTCTACTGCATCTCCTCTTCCA	26	<lmxll>	ll	136	lm	127		
>ref-12254	AACCGGAGCACTCGGCCTCCTTTTCCT	25	<nnxnp>	nn	115	np	143		
>ref-12259	TTCCCTCCTACTCTACCTCCTTTTCCT	13	<hkxhk>	hh	40	hk	139	kk	69
>ref-12267	GGTCTCTTTACACCTGCTCCCTGCTGT	14	<nnxnp>	nn	127	np	143		
>ref-12276	ATTTTTCTCACCTTCTCTCCCTTCTTT	27	<lmxll>	ll	124	lm	117		
>ref-12279	CCTCCACCCACGTATCCTCCAGCCCAT	7	<hkxhk>	hh	1	hk	268	kk	0
>ref-12279	CCTCCACCCACGTATCCTCCAGCCCAT	26	<lmxll>	ll	139	lm	121		
>ref-12283	AGACATTGCACCTCCACTCCCCGTGGA	15	<hkxhk>	hh	58	hk	148	kk	57
>ref-12309	CTTACTCATACTTTCACTCCTACTCAT	5	<nnxnp>	nn	131	np	131		
>ref-12314	CTTCCCTCCACTCCTACTCCTCTTCTC	3	<nnxnp>	nn	146	np	121		
>ref-12314	CTTCCCTCCACTCCTACTCCTCTTCTC	22	<nnxnp>	nn	147	np	120		
>ref-12316	CAAAAGTGAACACGTACTCCCATCAGT	13	<lmxll>	ll	10	lm	254		
>ref-12321	CCGGTCCCCACCCATCCTCCCACGCAA	2	<hkxhk>	hh	11	hk	258	kk	0
>ref-12321	CCGGTCCCCACCCATCCTCCCACGCAA	5	<hkxhk>	hh	0	hk	270	kk	2
>ref-12321	CCGGTCCCCACCCATCCTCCCACGCAA	13	<hkxhk>	hh	1	hk	272	kk	0
>ref-12321	CCGGTCCCCACCCATCCTCCCACGCAA	21	<hkxhk>	hh	2	hk	270	kk	0
>ref-12342	TTTATCACGACCGTATCTCCATTTCTA	27	<nnxnp>	nn	127	np	126		
>ref-12343	CACACGGGCACAGCCGCTCCTCCAGGG	16	<hkxhk>	hh	0	hk	273	kk	0
>ref-12343	CACACGGGCACAGCCGCTCCTCCAGGG	27	<nnxnp>	nn	37	np	232		
>ref-12358	TTTGTATCCACTAAGCCTCCTCATCCC	4	<nnxnp>	nn	127	np	140		
>ref-12358	TTTGTATCCACTAAGCCTCCTCATCCC	12	<lmxll>	ll	154	lm	105		
>ref-12365	TCCTACGCGACGAAATCTCCTTCTTCC	13	<nnxnp>	nn	109	np	148		
>ref-12379	AAAGTTATGACTCTCTCTCCTTCACAT	5	<lmxll>	ll	129	lm	130		
>ref-12381	CTCCCCCTAACCTCCCCTCCGTGTCTA	1	<nnxnp>	nn	128	np	135		
>ref-12381	CTCCCCCTAACCTCCCCTCCGTGTCTA	2	<nnxnp>	nn	128	np	135		
>ref-12386	TAAACCAATACAAGATCTCCTCGGGAA	21	<nnxnp>	nn	125	np	119		
>ref-12386	TAAACCAATACAAGATCTCCTCGGGAA	22	<nnxnp>	nn	125	np	119		
>ref-12391	CATAACAACACCATAGCTCCCCGCAAA	12	<lmxll>	ll	144	lm	121		
>ref-12396	ACTTGTACCACGGCCGCTCCAGTTAGC	15	<hkxhk>	hh	61	hk	145	kk	59
>ref-12403	ATAACATTAACATGAACTCCCTGTGTG	25	<lmxll>	ll	124	lm	128		
>ref-12413	CCTACCTTTACCTCCTCTCCACGCCTA	1	<hkxhk>	hh	0	hk	13	kk	223
>ref-12413	CCTACCTTTACCTCCTCTCCACGCCTA	22	<hkxhk>	hh	0	hk	273	kk	0
>ref-12413	CCTACCTTTACCTCCTCTCCACGCCTA	23	<lmxll>	ll	250	lm	16		
>ref-12415	CCTCCCTGCACCATGCCTCCTGCCCTC	22	<nnxnp>	nn	145	np	128		
>ref-12429	CACCAACCCACTCTACCTCCCTCTCCT	9	<hkxhk>	hh	0	hk	261	kk	6
>ref-12464	GTCCGTCTCACAATGGCTCCCTTTCTA	7	<nnxnp>	nn	137	np	133		
>ref-12468	CTCCTTCCCACATATCCTCCTTCCTCC	8	<lmxll>	ll	118	lm	128		

>ref-12469	GCCTTTTACACTCTCTCTCCTTTGCTT	27	<hkxhk>	hh	0	hk	272	kk	1
>ref-12475	CATTCTCCCACTTCTCCTCCTTCCTTT	2	<lmxll>	ll	12	lm	249		
>ref-12475	CATTCTCCCACTTCTCCTCCTTCCTTT	4	<lmxll>	ll	12	lm	248		
>ref-12497	TTCCTTCACACCTCCCCTCCCCTCTTA	13	<nnxnp>	nn	126	np	127		
>ref-12505	TCCCCTCTTACTACCCCTCCCATTCTT	7	<hkxhk>	hh	52	hk	130	kk	46
>ref-12514	TCGTATATCACTCCCTCTCCTTTCCTC	13	<hkxhk>	hh	61	hk	157	kk	41
>ref-12525	AGATGGTTGACACCTGCTCCTTCAGAT	3	<hkxhk>	hh	0	hk	272	kk	0
>ref-12525	AGATGGTTGACACCTGCTCCTTCAGAT	7	<hkxhk>	hh	0	hk	272	kk	0
>ref-12537	CTCACCCGGACGCAACCTCCTTTCGAC	3	<hkxhk>	hh	61	hk	134	kk	53
>ref-12552	CTGGGCGTCACAAATACTCCTGCAGGA	5	<nnxnp>	nn	95	np	128		
>ref-12588	ATGTCTACAACGAGATCTCCACCCTTA	5	<nnxnp>	nn	138	np	128		
>ref-12620	GCACATGACACTTTTTCTCCCTTAGGA	4	<nnxnp>	nn	132	np	113		
>ref-12634	CACACGCCCACTGCAACTCCAACTGCT	9	<lmxll>	ll	266	lm	0		
>ref-12642	TCTCCATACACCTTTTCTCCTCCCTCA	27	<nnxnp>	nn	126	np	139		
>ref-12656	CCCTGGCTCACTATAGCTCCTTCACAA	22	<lmxll>	ll	128	lm	139		
>ref-12679	CGCGCACACACACGCACTCCTCTCTCA	5	<lmxll>	ll	131	lm	136		
>ref-12721	CCATCGTTGACCTCTTCTCCGCCTCAC	3	<hkxhk>	hh	0	hk	271	kk	1
>ref-12748	GCTGCTTGAACCGTCGCTCCAGCTCGT	13	<hkxhk>	hh	0	hk	242	kk	25
>ref-12748	GCTGCTTGAACCGTCGCTCCAGCTCGT	22	<hkxhk>	hh	20	hk	250	kk	0
>ref-12769	TTAACCCAAACTTAGGCTCCGGCATTT	23	<nnxnp>	nn	132	np	100		
>ref-12774	CTCGAATCGACCACGCCTCCTTGTTAT	12	<lmxll>	ll	125	lm	107		
>ref-12774	CTCGAATCGACCACGCCTCCTTGTTAT	15	<lmxll>	ll	127	lm	107		
>ref-12780	GCCTCTCTAACTCTCTCTCCTACATAT	9	<lmxll>	ll	116	lm	125		
>ref-12780	GCCTCTCTAACTCTCTCTCCTACATAT	25	<nnxnp>	nn	135	np	114		
>ref-12785	TCTCCTTTTACAATCTCTCCCTCTTCC	3	<hkxhk>	hh	0	hk	257	kk	3
>ref-12785	TCTCCTTTTACAATCTCTCCCTCTTCC	12	<hkxhk>	hh	3	hk	260	kk	0
>ref-12850	CCCACAATCACTCTCACTCCCCCACAC	4	<nnxnp>	nn	232	np	0		
>ref-12880	GCATCGGGCACAGTTTCTCCCTCTTTA	14	<nnxnp>	nn	131	np	117		
>ref-12913	TCGTCCAATACCCTTTCTCCAACATTC	4	<nnxnp>	nn	43	np	184		
>ref-12913	TCGTCCAATACCCTTTCTCCAACATTC	25	<nnxnp>	nn	41	np	184		
>ref-12916	TTTCCCTTCACCTGGTCTCCCTCGCCA	7	<nnxnp>	nn	122	np	123		
>ref-12956	TCAGTAGCCACGCCTCCTCCTCTCCCA	27	<nnxnp>	nn	135	np	134		
>ref-12974	GTCTTCGTCACTCCCCCTCCTACGTCT	1	<nnxnp>	nn	116	np	154		
>ref-12975	TGCTCAGTTACATAACCTCCTTCACGC	5	<nnxnp>	nn	131	np	125		
>ref-13023	AACCTTTAGACCTGGTCTCCCTGTGTT	22	<nnxnp>	nn	113	np	114		
>ref-13026	GAATATGAAACATTAACTCCTTCATCG	4	<lmxll>	ll	111	lm	120		
>ref-13026	GAATATGAAACATTAACTCCTTCATCG	14	<lmxll>	ll	111	lm	120		
>ref-13028	CTTCCCGCCACTCCACCTCCACCTCCA	3	<hkxhk>	hh	0	hk	267	kk	0

>ref-13028	CTTCCCGCCACTCCACCTCCACCTCCA	5	<hkxhk>	hh	0	hk	194	kk	58
>ref-13028	CTTCCCGCCACTCCACCTCCACCTCCA	7	<lmxll>	ll	8	lm	255		
>ref-13032	CTCTACCTCACTCGCTCTCCACCACAC	14	<lmxll>	ll	0	lm	267		
>ref-13032	CTCTACCTCACTCGCTCTCCACCACAC	24	<lmxll>	ll	0	lm	267		
>ref-13057	AAATGTGGCACAATGCCTCCTCAGCCC	26	<lmxll>	ll	129	lm	137		
>ref-13073	GTTCGGCTGACGTTTGCTCCTGGAGGC	27	<hkxhk>	hh	1	hk	272	kk	0
>ref-13085	CCTCCTCCTACTCCTCCTCCAGACGCT	25	<lmxll>	ll	1	lm	272		
>ref-13085	CCTCCTCCTACTCCTCCTCCAGACGCT	27	<lmxll>	ll	1	lm	272		
>ref-13107	CTTCATTCAACCACTCCTCCTCCTCTA	1	<lmxll>	ll	229	lm	6		
>ref-13131	TGACAAGACACTATGCCTCCCCTTTGC	4	<lmxll>	ll	252	lm	18		
>ref-13131	TGACAAGACACTATGCCTCCCCTTTGC	6	<hkxhk>	hh	51	hk	205	kk	0
>ref-13131	TGACAAGACACTATGCCTCCCCTTTGC	12	<hkxhk>	hh	0	hk	273	kk	0
>ref-13131	TGACAAGACACTATGCCTCCCCTTTGC	21	<nnxnp>	nn	255	np	16		
>ref-13131	TGACAAGACACTATGCCTCCCCTTTGC	22	<nnxnp>	nn	255	np	16		
>ref-13131	TGACAAGACACTATGCCTCCCCTTTGC	27	<lmxll>	ll	2	lm	271		
>ref-13146	TACTTATACACGTGTACTCCTTTGTAT	2	<lmxll>	ll	107	lm	128		
>ref-13168	GCCCTCTCCACTCCCCCTCCCCTTCCA	1	<lmxll>	ll	12	lm	256		
>ref-13168	GCCCTCTCCACTCCCCCTCCCCTTCCA	27	<lmxll>	ll	16	lm	251		
>ref-13195	TGTCTGCCTACAGCTGCTCCATTACAG	3	<hkxhk>	hh	0	hk	273	kk	0
>ref-13195	TGTCTGCCTACAGCTGCTCCATTACAG	27	<hkxhk>	hh	75	hk	197	kk	0
>ref-13201	TTTATAGCCACGTTATCTCCCCCACAT	24	<lmxll>	ll	133	lm	118		
>ref-13202	ACTCCAGCCACTCTTTCTCCACACACT	1	<nnxnp>	nn	121	np	139		
>ref-13211	TTTCCTCATACTAACACTCCCGTTCTG	3	<nnxnp>	nn	90	np	139		
>ref-13252	CACCCACGAACTCCTCCTCCTCCTCCC	8	<hkxhk>	hh	0	hk	273	kk	0
>ref-13252	CACCCACGAACTCCTCCTCCTCCTCCC	12	<hkxhk>	hh	0	hk	193	kk	63
>ref-13252	CACCCACGAACTCCTCCTCCTCCTCCC	22	<hkxhk>	hh	0	hk	273	kk	0
>ref-13252	CACCCACGAACTCCTCCTCCTCCTCCC	26	<hkxhk>	hh	0	hk	193	kk	62
>ref-13266	GAAACAACCACACAAACTCCTGTACTC	24	<nnxnp>	nn	112	np	139		
>ref-13273	TAACCTTTGACCTCATCTCCTGCAGGA	4	<nnxnp>	nn	121	np	129		
>ref-13280	CCCCCTCCAACGCCTTCTCCTCACTTC	15	<hkxhk>	hh	0	hk	217	kk	48
>ref-13288	CTCAATTTTACTCTCTCTCCTTTCTAC	2	<lmxll>	ll	113	lm	137		
>ref-13290	CCATCTACCACCCCATCTCCACAAATA	12	<nnxnp>	nn	233	np	0		
>ref-13297	TTCTTTATTACTCCTTCTCCTCTCCTT	6	<lmxll>	ll	1	lm	268		
>ref-13297	TTCTTTATTACTCCTTCTCCTCTCCTT	21	<lmxll>	ll	1	lm	268		
>ref-13304	TGTCTTTGCACTTTATCTCCTTCGCTT	4	<lmxll>	ll	118	lm	139		
>ref-13304	TGTCTTTGCACTTTATCTCCTTCGCTT	8	<lmxll>	ll	116	lm	140		
>ref-13321	CAACCTCCAACACCTTCTCCCTCACTA	14	<hkxhk>	hh	80	hk	185	kk	0
>ref-13322	TAATAGCTCACTCCCTCTCCTCCCTTA	1	<nnxnp>	nn	116	np	142		

>ref-13322	TAATAGCTCACTCCCTCTCCTCCCTTA	16	<nnxnp>	nn	116	np	142		
>ref-13332	CTTATTCCTACTTCTCCTCCTTCTAAC	8	<nnxnp>	nn	117	np	135		
>ref-13350	TGCATAAAGACAAGACCTCCAGCCGCA	9	<nnxnp>	nn	143	np	127		
>ref-13371	CAATCCCCCACTCTCACTCCCTTTCCC	3	<nnxnp>	nn	250	np	2		
>ref-13371	CAATCCCCCACTCTCACTCCCTTTCCC	8	<nnxnp>	nn	265	np	0		
>ref-13371	CAATCCCCCACTCTCACTCCCTTTCCC	16	<nnxnp>	nn	247	np	0		
>ref-13371	CAATCCCCCACTCTCACTCCCTTTCCC	23	<nnxnp>	nn	258	np	1		
>ref-13376	TGCCCATTTACCCTCACTCCTCCCCCT	2	<lmxll>	ll	262	lm	0		
>ref-13379	CCCTTAAGGACAACAACTCCACACATA	25	<lmxll>	ll	115	lm	132		
>ref-13379	CCCTTAAGGACAACAACTCCACACATA	26	<lmxll>	ll	112	lm	133		
>ref-13383	GGCTTTGAAACAACGTCTCCATCCATT	23	<hkxhk>	hh	40	hk	202	kk	2
>ref-13405	CGTCCACCTACTTATTCTCCATCTTTA	3	<lmxll>	ll	118	lm	108		
>ref-13422	ACACCCTCTACTCCCCCTCCTCCTCAT	3	<lmxll>	ll	152	lm	96		
>ref-13422	ACACCCTCTACTCCCCCTCCTCCTCAT	27	<lmxll>	ll	155	lm	95		
>ref-13427	CTCCACCTAACATTGGCTCCGAGCAAT	2	<nnxnp>	nn	120	np	122		
>ref-13433	CGCCCCTCCACGTTCCCTCCCCTCCCC	3	<hkxhk>	hh	0	hk	273	kk	0
>ref-13433	CGCCCCTCCACGTTCCCTCCCCTCCCC	4	<hkxhk>	hh	24	hk	239	kk	0
>ref-13433	CGCCCCTCCACGTTCCCTCCCCTCCCC	5	<nnxnp>	nn	219	np	42		
>ref-13433	CGCCCCTCCACGTTCCCTCCCCTCCCC	8	<hkxhk>	hh	24	hk	239	kk	0
>ref-13446	GCTGCGCCCACAAGGCCTCCAGTCGTG	6	<hkxhk>	hh	0	hk	273	kk	0
>ref-13448	ACCACAACGACCGCAACTCCCGCAACC	26	<nnxnp>	nn	125	np	94		
>ref-13453	TATGCACACACATGCGCTCCCTCGCCC	4	<nnxnp>	nn	136	np	132		
>ref-13453	TATGCACACACATGCGCTCCCTCGCCC	5	<nnxnp>	nn	138	np	131		
>ref-13469	CATTACTTAACAGTGTCTCCATTGAAG	3	<lmxll>	ll	116	lm	142		
>ref-13470	TCCCCCTTTACCCTCTCTCCCTCCTCT	1	<lmxll>	ll	10	lm	253		
>ref-13479	GTCCACCCCACAGTGTCTCCTTGAGGG	6	<lmxll>	ll	147	lm	120		
>ref-13479	GTCCACCCCACAGTGTCTCCTTGAGGG	14	<lmxll>	ll	203	lm	45		
>ref-13496	CCCCCGTCCACTCCTACTCCCCTCCTC	12	<lmxll>	ll	146	lm	106		
>ref-13496	CCCCCGTCCACTCCTACTCCCCTCCTC	16	<lmxll>	ll	143	lm	106		
>ref-13498	ATGTGCTCAACGTAGGCTCCTGGGAAG	12	<hkxhk>	hh	13	hk	243	kk	0
>ref-13498	ATGTGCTCAACGTAGGCTCCTGGGAAG	14	<hkxhk>	hh	13	hk	242	kk	0
>ref-13513	TTAAACACGACCAAGTCTCCGCACCGC	4	<nnxnp>	nn	131	np	140		
>ref-13514	TGTTTGTACACCTGTGCTCCGCTGTAC	4	<lmxll>	ll	129	lm	135		
>ref-13520	CTGCCTCTCACCCTACCTCCCTCTCAA	15	<hkxhk>	hh	111	hk	119	kk	0
>ref-13520	CTGCCTCTCACCCTACCTCCCTCTCAA	16	<lmxll>	ll	264	lm	0		
>ref-13520	CTGCCTCTCACCCTACCTCCCTCTCAA	22	<lmxll>	ll	264	lm	0		
>ref-13520	CTGCCTCTCACCCTACCTCCCTCTCAA	26	<lmxll>	ll	264	lm	0		
>ref-13543	TACCCACTCACCCACCCTCCGAGTCCG	9	<hkxhk>	hh	58	hk	149	kk	46

>ref-13554	TCTTTTTTTACTCTCTCTCCGTCATCT	21	<nnxnp>	nn	106	np	132		
>ref-13557	TTTTAATCTACTTACGCTCCAATCTAC	27	<lmxll>	ll	23	lm	218		
>ref-13581	ACAGTTCACACCTCTTCTCCAACCAAT	4	<hkxhk>	hh	63	hk	104	kk	67
>ref-13581	ACAGTTCACACCTCTTCTCCAACCAAT	27	<lmxll>	ll	136	lm	102		
>ref-13586	CGTGGCTCCACCCTCCCTCCCTCCCTC	2	<hkxhk>	hh	1	hk	227	kk	11
>ref-13586	CGTGGCTCCACCCTCCCTCCCTCCCTC	8	<nnxnp>	nn	17	np	228		
>ref-13586	CGTGGCTCCACCCTCCCTCCCTCCCTC	13	<nnxnp>	nn	116	np	141		
>ref-13589	TTCCCCTTCACCGCCCCTCCTCCCCCT	2	<nnxnp>	nn	9	np	247		
>ref-13589	TTCCCCTTCACCGCCCCTCCTCCCCCT	27	<nnxnp>	nn	10	np	247		
>ref-13594	TGCATTATCACCCCCTCTCCTCTCCTC	2	<nnxnp>	nn	130	np	111		
>ref-13616	TCCCGCTGCACTCGCGCTCCGTCGACA	4	<lmxll>	ll	137	lm	135		
>ref-13620	CCATCGCCCACTCTCCCTCCCCCCCGT	22	<lmxll>	ll	218	lm	42		
>ref-13639	ATCTCCTCAACGCCACCTCCGACCGCC	23	<lmxll>	ll	111	lm	152		
>ref-13652	CGCCAATTTACGATGCCTCCGCGGATT	2	<nnxnp>	nn	250	np	0		
>ref-13672	GTGCATCGTACTTACCCTCCATTGATG	21	<nnxnp>	nn	127	np	134		
>ref-13681	ATACTTATAACCTCGCCTCCACCCATT	15	<nnxnp>	nn	133	np	128		
>ref-13691	GGTACAAGCACGTACCCTCCACGAGAG	26	<lmxll>	ll	118	lm	147		
>ref-13713	CTTTCCGCGACCACTTCTCCTCCTCCT	1	<lmxll>	ll	259	lm	5		
>ref-13726	CACACGCACACGGCCTCTCCCTTCGCC	8	<nnxnp>	nn	143	np	125		
>ref-13749	GTGGCCATGACCTTTCCTCCTGAACGC	22	<lmxll>	ll	144	lm	105		
>ref-13749	GTGGCCATGACCTTTCCTCCTGAACGC	24	<lmxll>	ll	27	lm	230		
>ref-13749	GTGGCCATGACCTTTCCTCCTGAACGC	25	<hkxhk>	hh	76	hk	182	kk	0
>ref-13751	AACTCGACAACGTTCCCTCCATGAAAG	12	<lmxll>	ll	128	lm	128		
>ref-13751	AACTCGACAACGTTCCCTCCATGAAAG	24	<lmxll>	ll	128	lm	127		
>ref-13760	GTCGAGGGAACCGCTCCTCCTCAACAG	2	<nnxnp>	nn	132	np	133		
>ref-13761	ACCTTAAGGACTTGTCCTCCTCTCTTT	5	<lmxll>	ll	122	lm	140		
>ref-13817	CGTCACTGCACGATTCCTCCGCTCTGA	1	<nnxnp>	nn	260	np	11		
>ref-13817	CGTCACTGCACGATTCCTCCGCTCTGA	3	<hkxhk>	hh	0	hk	179	kk	85
>ref-13817	CGTCACTGCACGATTCCTCCGCTCTGA	15	<lmxll>	ll	70	lm	195		
>ref-13821	CTCTCACTCACCATCACTCCCTCGTCT	24	<hkxhk>	hh	0	hk	270	kk	0
>ref-13821	CTCTCACTCACCATCACTCCCTCGTCT	25	<lmxll>	ll	41	lm	213		
>ref-13828	ACAGCCTACACTGTCACTCCATCTGTC	9	<hkxhk>	hh	64	hk	132	kk	54
>ref-13838	ACTCCCTTCACTCACCCTCCTCCACCC	6	<nnxnp>	nn	135	np	118		
>ref-13850	ACTACTACGACCACCTCTCCCACCATC	16	<nnxnp>	nn	107	np	138		
>ref-13858	GAAGGTAGAACAGATGCTCCAGTTCCT	2	<lmxll>	ll	122	lm	144		
>ref-13858	GAAGGTAGAACAGATGCTCCAGTTCCT	23	<lmxll>	ll	124	lm	142		
>ref-13871	CCTTCTTCTACATTCTCTCCTCCCCCA	9	<hkxhk>	hh	0	hk	238	kk	7
>ref-13871	CCTTCTTCTACATTCTCTCCTCCCCCA	27	<hkxhk>	hh	6	hk	261	kk	1

>ref-13902	GCCGACAACACTAAATCTCCAGTTGTA	23	<nnxnp>	nn	264	np	0		
>ref-13903	TGCGTTTAAACTATTTCTCCTTATATT	12	<nnxnp>	nn	110	np	111		
>ref-13917	TTCCATAGGACGCCACCTCCCCTATGC	3	<nnxnp>	nn	133	np	107		
>ref-13923	AACAGGAAGACCGAAACTCCGACAGAG	6	<lmxll>	ll	127	lm	122		
>ref-13924	AATAACTAGACACGGTCTCCCCTGTAT	24	<lmxll>	ll	116	lm	140		
>ref-13931	TCTTGTTGTACTTTCCCTCCTTCCTAC	21	<hkxhk>	hh	48	hk	129	kk	54
>ref-13952	TCCTCATTTACTATCTCTCCTCAGTCA	27	<lmxll>	ll	124	lm	131		
>ref-13956	CCTCCCTCTACCCTTCCTCCCTATCTT	2	<nnxnp>	nn	261	np	0		
>ref-13956	CCTCCCTCTACCCTTCCTCCCTATCTT	9	<nnxnp>	nn	141	np	78		
>ref-13956	CCTCCCTCTACCCTTCCTCCCTATCTT	22	<nnxnp>	nn	57	np	176		
>ref-13958	CGAATGGGCACGTCTGCTCCGCTTGCC	2	<hkxhk>	hh	0	hk	115	kk	153
>ref-13986	ACTCTGTCGACTGTTGCTCCTCTCAAG	7	<hkxhk>	hh	0	hk	273	kk	0
>ref-14061	CATCCTGCCACACTCTCTCCCCTCCCT	2	<lmxll>	ll	194	lm	56		
>ref-14061	CATCCTGCCACACTCTCTCCCCTCCCT	7	<lmxll>	ll	194	lm	55		
>ref-14066	ACTCACCTAACAAACGCTCCTTACTTT	13	<hkxhk>	hh	29	hk	128	kk	66
>ref-14066	ACTCACCTAACAAACGCTCCTTACTTT	15	<hkxhk>	hh	29	hk	127	kk	67
>ref-14070	GAAGGACTGACCTGACCTCCTTAATCA	8	<nnxnp>	nn	262	np	0		
>ref-14070	GAAGGACTGACCTGACCTCCTTAATCA	13	<hkxhk>	hh	50	hk	122	kk	67
>ref-14116	ACCACGACAACCATATCTCCTCATAAC	12	<hkxhk>	hh	9	hk	253	kk	0
>ref-14117	TCCAAATCCACACAGCCTCCCCATCCA	4	<hkxhk>	hh	6	hk	253	kk	5
>ref-14121	ACGCCCAACACTTACGCTCCCCCCTTT	16	<nnxnp>	nn	130	np	128		
>ref-14139	CCAAGGAAAACCATGTCTCCGCTATTG	3	<hkxhk>	hh	0	hk	261	kk	0
>ref-14142	TGTAGCGGGACCCAGGCTCCAGGAGGA	3	<hkxhk>	hh	0	hk	272	kk	0
>ref-14142	TGTAGCGGGACCCAGGCTCCAGGAGGA	22	<nnxnp>	nn	129	np	142		
>ref-14173	TGACATGCAACCGAACCTCCTAGGGTT	22	<nnxnp>	nn	111	np	148		
>ref-14187	CCAATGCCCACGCTGCCTCCTCTTCCC	2	<nnxnp>	nn	7	np	266		
>ref-14187	CCAATGCCCACGCTGCCTCCTCTTCCC	8	<hkxhk>	hh	58	hk	191	kk	0
>ref-14191	TCAGATTCGACCTGCTCTCCCCGCCTC	27	<hkxhk>	hh	51	hk	154	kk	61
>ref-14205	TGCCCCTCCACCCTTTCTCCCTCTTTC	2	<hkxhk>	hh	3	hk	260	kk	1
>ref-14205	TGCCCCTCCACCCTTTCTCCCTCTTTC	4	<lmxll>	ll	264	lm	0		
>ref-14205	TGCCCCTCCACCCTTTCTCCCTCTTTC	5	<nnxnp>	nn	265	np	0		
>ref-14205	TGCCCCTCCACCCTTTCTCCCTCTTTC	24	<hkxhk>	hh	2	hk	261	kk	1
>ref-14222	GGACTCTCCACCTCTCCTCCTCCTCCT	8	<lmxll>	ll	140	lm	127		
>ref-14222	GGACTCTCCACCTCTCCTCCTCCTCCT	13	<lmxll>	ll	139	lm	127		
>ref-14224	TATTGCATAACCCCATCTCCTTGTTCA	26	<nnxnp>	nn	116	np	104		
>ref-14228	TCACTTCGTACCCACCCTCCCTCCTCC	5	<hkxhk>	hh	0	hk	163	kk	64
>ref-14228	TCACTTCGTACCCACCCTCCCTCCTCC	8	<hkxhk>	hh	0	hk	162	kk	64
>ref-14249	ACTATCCTCACCATATCTCCCTTCTTC	5	<nnxnp>	nn	134	np	110		

>ref-14249	ACTATCCTCACCATATCTCCCTTCTTC	23	<nnxnp>	nn	100	np	139		
>ref-14252	CACTCGGACACCGCAGCTCCGTCAGGA	27	<hkxhk>	hh	65	hk	158	kk	39
>ref-14269	ATATCCATTACTACTCCTCCCACAGCA	3	<hkxhk>	hh	0	hk	271	kk	0
>ref-14294	CTTTGAAACACTGTCACTCCCTGTCAC	26	<nnxnp>	nn	204	np	20		
>ref-14326	ACTCCTTTTACAATGGCTCCCAACTCT	9	<nnxnp>	nn	124	np	139		
>ref-14326	ACTCCTTTTACAATGGCTCCCAACTCT	25	<nnxnp>	nn	124	np	139		
>ref-14334	CCCCTCTAGACTCTATCTCCCCTCTTT	1	<lmxll>	ll	119	lm	117		
>ref-14356	CAACCCACAACCATCACTCCCCCACAC	14	<lmxll>	ll	131	lm	135		
>ref-14356	CAACCCACAACCATCACTCCCCCACAC	22	<lmxll>	ll	131	lm	135		
>ref-14370	TATGTCATCACATTTTCTCCTTTTCTC	2	<lmxll>	ll	17	lm	229		
>ref-14370	TATGTCATCACATTTTCTCCTTTTCTC	6	<lmxll>	ll	17	lm	229		
>ref-14377	TTTTCTCTTACCCCTACTCCAGTAATG	9	<hkxhk>	hh	0	hk	205	kk	31
>ref-14405	ACCCCAACAACCGTCCCTCCAACAAGA	1	<lmxll>	ll	125	lm	142		
>ref-14443	AAATCTGACACCGTTTCTCCAGCAGCT	8	<hkxhk>	hh	0	hk	271	kk	0
>ref-14449	CACAAAACAACCTTTTCTCCAACCCAC	13	<nnxnp>	nn	133	np	111		
>ref-14451	CCCCATGTCACCTTCCCTCCCTCTCCC	1	<lmxll>	ll	260	lm	0		
>ref-14461	GGCTCGGTCACCCCACCTCCCCCTCCA	13	<hkxhk>	hh	54	hk	136	kk	67
>ref-14479	TCTATCCCCACCCCCCCTCCTCGCCAC	5	<lmxll>	ll	145	lm	123		
>ref-14487	CCACCTTTCACTTCCTCTCCCCTCTCT	3	<hkxhk>	hh	30	hk	232	kk	0
>ref-14487	CCACCTTTCACTTCCTCTCCCCTCTCT	25	<hkxhk>	hh	0	hk	230	kk	26
>ref-14502	TTACATCCTACCTTCCCTCCCCCCTCC	3	<lmxll>	ll	155	lm	81		
>ref-14502	TTACATCCTACCTTCCCTCCCCCCTCC	9	<nnxnp>	nn	265	np	0		
>ref-14502	TTACATCCTACCTTCCCTCCCCCCTCC	13	<lmxll>	ll	157	lm	79		
>ref-14519	CCCTTTCTCACCTCCTCTCCTCCCTGG	26	<nnxnp>	nn	237	np	2		
>ref-14519	CCCTTTCTCACCTCCTCTCCTCCCTGG	27	<nnxnp>	nn	236	np	1		
>ref-14542	CCATCGTCGACGGTTTCTCCGAAAAGT	5	<hkxhk>	hh	0	hk	272	kk	0
>ref-14542	CCATCGTCGACGGTTTCTCCGAAAAGT	13	<lmxll>	ll	89	lm	156		
>ref-14542	CCATCGTCGACGGTTTCTCCGAAAAGT	27	<lmxll>	ll	4	lm	262		
>ref-14562	CACCCCCACACCTCTCCTCCATCTCTC	22	<hkxhk>	hh	0	hk	272	kk	1
>ref-14585	CTCCCATTCACTCCCTCTCCCCTCCCT	7	<lmxll>	ll	10	lm	239		
>ref-14585	CTCCCATTCACTCCCTCTCCCCTCCCT	22	<lmxll>	ll	19	lm	240		
>ref-14604	GTTTATTAAACTAAACCTCCATCATCA	5	<hkxhk>	hh	4	hk	263	kk	0
>ref-14604	GTTTATTAAACTAAACCTCCATCATCA	7	<lmxll>	ll	164	lm	86		
>ref-14604	GTTTATTAAACTAAACCTCCATCATCA	14	<hkxhk>	hh	4	hk	263	kk	0
>ref-14604	GTTTATTAAACTAAACCTCCATCATCA	23	<hkxhk>	hh	20	hk	221	kk	0
>ref-14612	CCCTTTCCCACGCCCACTCCCGCCCTC	16	<nnxnp>	nn	106	np	148		
>ref-14614	TCCCTTCATACACTCCCTCCCCCCCAA	15	<nnxnp>	nn	111	np	151		
>ref-14622	AGCCAAGGCACATGTCCTCCGGAAAGT	8	<lmxll>	ll	123	lm	122		

>ref-14629	CCGTCAGCTACGATACCTCCGTCAGCC	1	<lmxll>	ll	92	lm	165		
>ref-14629	CCGTCAGCTACGATACCTCCGTCAGCC	22	<lmxll>	ll	93	lm	165		
>ref-14662	CCTCCCCTGACGTCATCTCCCTTGAAG	25	<nnxnp>	nn	263	np	0		
>ref-14681	CCTTCCCTAACTCCGTCTCCCTTTCCT	9	<hkxhk>	hh	79	hk	180	kk	0
>ref-14706	TATACACACACATAAGCTCCCTTTCTT	13	<nnxnp>	nn	136	np	121		
>ref-14733	AGTCCAATTACTCCTCCTCCTCTCCCC	1	<lmxll>	ll	230	lm	1		
>ref-14737	CCCTCACTTACTCCCTCTCCCTCTCAG	7	<hkxhk>	hh	0	hk	273	kk	0
>ref-14737	CCCTCACTTACTCCCTCTCCCTCTCAG	9	<hkxhk>	hh	0	hk	273	kk	0
>ref-14747	GCAGAGGAAACCTGTCCTCCTGAGGCA	6	<hkxhk>	hh	0	hk	272	kk	0
>ref-14747	GCAGAGGAAACCTGTCCTCCTGAGGCA	14	<hkxhk>	hh	0	hk	273	kk	0
>ref-14751	CACTGATGGACCGCTTCTCCAGAGCCT	12	<hkxhk>	hh	0	hk	273	kk	0
>ref-14751	CACTGATGGACCGCTTCTCCAGAGCCT	13	<hkxhk>	hh	0	hk	203	kk	60
>ref-14760	GCTGCTGTTACGACATCTCCCTTTTAT	1	<nnxnp>	nn	265	np	0		
>ref-14760	GCTGCTGTTACGACATCTCCCTTTTAT	2	<nnxnp>	nn	266	np	0		
>ref-14768	ACTTCTATTACTATCTCTCCACATCAG	24	<hkxhk>	hh	57	hk	137	kk	61
>ref-14771	TCCTCTATTACTCTCTCTCCCTCTGTT	5	<lmxll>	ll	142	lm	88		
>ref-14779	CTCTAATACACCCATCCTCCAATCTCC	5	<lmxll>	ll	123	lm	114		
>ref-14779	CTCTAATACACCCATCCTCCAATCTCC	16	<lmxll>	ll	122	lm	114		
>ref-14817	TTCCCCTCAACTGTCTCTCCTCGTTCT	9	<nnxnp>	nn	134	np	137		
>ref-14826	CTGGCAAGTACAGTAACTCCAGTTTAT	3	<nnxnp>	nn	246	np	0		
>ref-14831	TGCAGCCCCACTCTAACTCCAAAACTG	7	<lmxll>	ll	117	lm	122		
>ref-14831	TGCAGCCCCACTCTAACTCCAAAACTG	14	<hkxhk>	hh	4	hk	253	kk	2
>ref-14831	TGCAGCCCCACTCTAACTCCAAAACTG	25	<hkxhk>	hh	51	hk	170	kk	0
>ref-14848	TGCCCGCCCACACTTCCTCCTTCCACC	21	<lmxll>	ll	114	lm	148		
>ref-14862	TGTGAGGTCACTTTCACTCCCATTAAC	25	<lmxll>	ll	0	lm	272		
>ref-14862	TGTGAGGTCACTTTCACTCCCATTAAC	26	<lmxll>	ll	0	lm	272		
>ref-14862	TGTGAGGTCACTTTCACTCCCATTAAC	27	<lmxll>	ll	171	lm	94		
>ref-14865	TCCCCTCCTACTTTTCCTCCTCTCCCA	3	<lmxll>	ll	252	lm	1		
>ref-14865	TCCCCTCCTACTTTTCCTCCTCTCCCA	15	<hkxhk>	hh	50	hk	133	kk	50
>ref-14917	CGCTGCCCCACCCACGCTCCGGCCACG	14	<nnxnp>	nn	270	np	0		
>ref-14920	AGCGCTGTCACAATCTCTCCAGTGCGA	14	<lmxll>	ll	106	lm	152		
>ref-14931	CATCCCATCACCACACCTCCTGTCGTC	15	<hkxhk>	hh	0	hk	188	kk	75
>ref-14937	CACACACACACACAGCCTCCATGCAGC	1	<hkxhk>	hh	78	hk	193	kk	0
>ref-14937	CACACACACACACAGCCTCCATGCAGC	3	<hkxhk>	hh	63	hk	209	kk	0
>ref-14937	CACACACACACACAGCCTCCATGCAGC	14	<hkxhk>	hh	0	hk	273	kk	0
>ref-14940	GTCCGCGTCACCCTACCTCCCTTCTCC	7	<lmxll>	ll	142	lm	126		
>ref-14943	AATCACACGACAGGAACTCCGCGGGCA	2	<lmxll>	ll	125	lm	144		
>ref-14943	AATCACACGACAGGAACTCCGCGGGCA	24	<lmxll>	ll	139	lm	126		

>ref-14967	CCCCTCCCTACGCCCACTCCGCCTATT	21	<nnxnp>	nn	9	np	256		
>ref-14967	CCCCTCCCTACGCCCACTCCGCCTATT	25	<nnxnp>	nn	9	np	255		
>ref-14993	TGCCGATGTACGATGCCTCCCAAAAGT	9	<nnxnp>	nn	268	np	0		
>ref-14994	CAAGTAGAAACCGCTCCTCCCCCCAAA	12	<nnxnp>	nn	135	np	116		
>ref-14994	CAAGTAGAAACCGCTCCTCCCCCCAAA	24	<nnxnp>	nn	137	np	114		
>ref-15001	CTCCTTAACACTCTCTCTCCTTTCCTC	16	<lmxll>	ll	116	lm	131		
>ref-15013	TCTCTTGTGACCTTTTCTCCTCTCTCT	21	<lmxll>	ll	100	lm	155		
>ref-15050	TGCACACGCACGCACACTCCTCCCCTA	1	<nnxnp>	nn	139	np	131		
>ref-15057	TTGACGGGAACATATTCTCCTTCTCCC	2	<nnxnp>	nn	130	np	133		
>ref-15059	TTCCCTTCCACTCTTCCTCCTTGCATA	16	<lmxll>	ll	126	lm	126		
>ref-15060	TCATGCCCTACTCTTTCTCCCTCGCAT	9	<nnxnp>	nn	118	np	136		
>ref-15062	TGACGACTAACATAAGCTCCTTTCCAT	12	<nnxnp>	nn	142	np	125		
>ref-15074	ACTACGTAAACACTAGCTCCCGGGGAT	25	<nnxnp>	nn	129	np	99		
>ref-15074	ACTACGTAAACACTAGCTCCCGGGGAT	27	<nnxnp>	nn	134	np	96		
>ref-15082	CCTCTAGCCACGGGATCTCCTCCTTCT	16	<nnxnp>	nn	129	np	140		
>ref-15082	CCTCTAGCCACGGGATCTCCTCCTTCT	23	<lmxll>	ll	138	lm	130		
>ref-15082	CCTCTAGCCACGGGATCTCCTCCTTCT	26	<lmxll>	ll	138	lm	130		
>ref-15093	CCTCGACCAACTCCTCCTCCTCATGAC	13	<hkxhk>	hh	0	hk	272	kk	0
>ref-15104	ACAGAGCATACAATATCTCCTCCGAGT	22	<hkxhk>	hh	57	hk	123	kk	49
>ref-15138	TCTTCTCCTACCTACCCTCCGGCCTTC	22	<hkxhk>	hh	62	hk	149	kk	60
>ref-15144	CACATGCACACACATGCTCCGACCTCG	12	<nnxnp>	nn	98	np	163		
>ref-15161	CCCTCCAGCACGCCCGCTCCCTCTCAT	5	<nnxnp>	nn	124	np	133		
>ref-15170	TACCCACTTACATACTCTCCCCCCGAC	15	<lmxll>	ll	138	lm	125		
>ref-15183	AAACTATAAACTGCCTCTCCTTCGGAA	23	<hkxhk>	hh	68	hk	118	kk	46
>ref-15185	TGATTTTTTACTTGTTCTCCTGTTCTT	22	<lmxll>	ll	109	lm	110		
>ref-15192	CGTACACCCACGCTCACTCCCACACAC	1	<lmxll>	ll	168	lm	99		
>ref-15194	ATCAACACCACCTGGCCTCCTCAACAC	12	<lmxll>	ll	133	lm	129		
>ref-15218	ATCACGTGGACAGTACCTCCAGCGTTA	2	<lmxll>	ll	117	lm	141		
>ref-15237	ACCATTCTCACTCACTCTCCCTCACTC	22	<hkxhk>	hh	51	hk	154	kk	52
>ref-15249	TCCACACACACACGACCTCCCCTTCCC	14	<lmxll>	ll	16	lm	221		
>ref-15249	TCCACACACACACGACCTCCCCTTCCC	23	<lmxll>	ll	20	lm	216		
>ref-15249	TCCACACACACACGACCTCCCCTTCCC	25	<lmxll>	ll	43	lm	177		
>ref-15261	CTTTCTCCAACCCTCTCTCCCCCCTCT	9	<hkxhk>	hh	8	hk	248	kk	0
>ref-15261	CTTTCTCCAACCCTCTCTCCCCCCTCT	14	<nnxnp>	nn	49	np	182		
>ref-15261	CTTTCTCCAACCCTCTCTCCCCCCTCT	25	<hkxhk>	hh	0	hk	211	kk	32
>ref-15266	TAAATGAATACACATGCTCCCTGTGAT	6	<lmxll>	ll	143	lm	105		
>ref-15276	CGCACGATGACCTTACCTCCTTCCCTC	26	<hkxhk>	hh	58	hk	133	kk	68
>ref-15285	ACAGCTTCAACCGAGGCTCCGAGGCCC	23	<lmxll>	ll	140	lm	131		

>ref-15286	CAACCCCACACCCCCTCTCCCACAATC	5	<lmxll>	ll	118	lm	126		
>ref-15304	TCCTCTCCTACTCTATCTCCTCTCCTG	27	<hkxhk>	hh	1	hk	231	kk	28
>ref-15305	ACATTACCTACATGGACTCCTAGAATA	16	<hkxhk>	hh	55	hk	127	kk	55
>ref-15308	AATATGAATACAGAATCTCCAGGGAAA	7	<lmxll>	ll	115	lm	147		
>ref-15308	AATATGAATACAGAATCTCCAGGGAAA	27	<nnxnp>	nn	142	np	111		
>ref-15319	TCTTCCTCCACCCTCCCTCCTTCCATT	21	<nnxnp>	nn	1	np	266		
>ref-15319	TCTTCCTCCACCCTCCCTCCTTCCATT	24	<nnxnp>	nn	1	np	267		
>ref-15334	TGCTCAATCACCAATACTCCCACATAA	21	<nnxnp>	nn	136	np	130		
>ref-15335	TATACCATCACACCGTCTCCCTTCTTA	3	<nnxnp>	nn	119	np	144		
>ref-15345	ACAGCCACAACCATAACTCCTTCCCCT	27	<nnxnp>	nn	258	np	1		
>ref-15367	CGTTCATAAACTCCCACTCCCTCCTTC	12	<nnxnp>	nn	115	np	124		
>ref-15373	CGCAAGCAAACACACGCTCCCTCAGAT	5	<lmxll>	ll	136	lm	136		
>ref-15382	AGACAACAGACACAGTCTCCCAATCTA	16	<hkxhk>	hh	62	hk	143	kk	58
>ref-15402	ACTCTTTTCACTGTCTCTCCTCTTCCG	1	<nnxnp>	nn	247	np	0		
>ref-15402	ACTCTTTTCACTGTCTCTCCTCTTCCG	2	<nnxnp>	nn	248	np	0		
>ref-15419	AGACGGCGCACTTTTTCTCCTCTGCCC	1	<hkxhk>	hh	3	hk	266	kk	0
>ref-15421	AACGTTCCCACCTACCCTCCTTTACTG	3	<lmxll>	ll	116	lm	152		
>ref-15439	CTCCCTCCAACGGCGCCTCCACCGTCC	1	<nnxnp>	nn	271	np	0		
>ref-15439	CTCCCTCCAACGGCGCCTCCACCGTCC	3	<nnxnp>	nn	271	np	0		
>ref-15460	ATATGAAGGACAACTACTCCAAGCACG	24	<hkxhk>	hh	35	hk	222	kk	0
>ref-15465	CTCTTTCTCACCTCTCCTCCCTCTTTC	4	<lmxll>	ll	35	lm	214		
>ref-15465	CTCTTTCTCACCTCTCCTCCCTCTTTC	27	<lmxll>	ll	36	lm	213		
>ref-15475	CACGCACGCACACAGCCTCCCCTCACG	13	<lmxll>	ll	135	lm	129		
>ref-15487	CCTTCTGTTACGGCCCCTCCTTCCTCT	9	<nnxnp>	nn	262	np	1		
>ref-15492	GAATGAGGGACTTACTCTCCGTTTTCT	3	<lmxll>	ll	120	lm	107		
>ref-15493	CAAACCCCAACTGAACCTCCTTCTCTT	4	<lmxll>	ll	1	lm	264		
>ref-15505	CAAGCACCAACACTTCCTCCTTCAACA	4	<nnxnp>	nn	13	np	256		
>ref-15505	CAAGCACCAACACTTCCTCCTTCAACA	16	<nnxnp>	nn	13	np	256		
>ref-15521	ATGGGCCACACTGGACCTCCATGGCCG	26	<nnxnp>	nn	266	np	0		
>ref-15526	CTGTCTTTCACATCCACTCCCAGTCCA	24	<lmxll>	ll	127	lm	136		
>ref-15528	CAGTGCGCGACTGTGTCTCCCTCATTG	4	<nnxnp>	nn	133	np	137		
>ref-15541	CCACGTATGACCATCTCTCCCCCTTTT	5	<hkxhk>	hh	54	hk	134	kk	61
>ref-15541	CCACGTATGACCATCTCTCCCCCTTTT	6	<hkxhk>	hh	54	hk	134	kk	61
>ref-15541	CCACGTATGACCATCTCTCCCCCTTTT	8	<nnxnp>	nn	120	np	134		
>ref-15553	CCGCACTCTACCCAGCCTCCTACGTCT	13	<lmxll>	ll	144	lm	125		
>ref-15556	CACACACCCACACTACCTCCACCACCA	5	<hkxhk>	hh	0	hk	273	kk	0
>ref-15556	CACACACCCACACTACCTCCACCACCA	6	<hkxhk>	hh	0	hk	273	kk	0
>ref-15557	CCTCTTCCTACTTCATCTCCCTTCTCT	22	<hkxhk>	hh	0	hk	266	kk	3

>ref-15557	CCTCTTCCTACTTCATCTCCCTTCTCT	26	<nnxnp>	nn	129	np	133		
>ref-15557	CCTCTTCCTACTTCATCTCCCTTCTCT	27	<hkxhk>	hh	0	hk	266	kk	3
>ref-15564	GCAAGCCCCACTCCCTCTCCCTCTCCT	1	<lmxll>	ll	270	lm	0		
>ref-15592	TCCTCCACCACCATTCCTCCTCTTTGT	25	<hkxhk>	hh	0	hk	270	kk	0
>ref-15612	ACAAGTACGACTACCACTCCAACGGCT	25	<hkxhk>	hh	0	hk	257	kk	10
>ref-15618	CCACATTCAACACTTCCTCCACGCCCA	13	<nnxnp>	nn	127	np	140		
>ref-15634	AAAACACATACGCCTACTCCAAGACAC	12	<nnxnp>	nn	121	np	122		
>ref-15639	ACGTTTATTACATTCTCTCCCTTTGCT	15	<lmxll>	ll	138	lm	127		
>ref-15646	GGTAATCGTACAGTGCCTCCTAACCAA	13	<hkxhk>	hh	0	hk	237	kk	18
>ref-15648	CGTGATTTTACCTCTTCTCCCACGAAG	24	<hkxhk>	hh	0	hk	219	kk	23
>ref-15661	CCCAATACAACCCTCTCTCCTCCCCTT	1	<nnxnp>	nn	223	np	1		
>ref-15666	TACACTCAAACAAGTGCTCCGTTGTTT	1	<nnxnp>	nn	114	np	147		
>ref-15669	CTCTCTACCACATTCCCTCCCTCTCAT	13	<nnxnp>	nn	142	np	116		
>ref-15675	ATTTACCCCACTCCCCCTCCTCCCCTC	5	<lmxll>	ll	15	lm	215		
>ref-15675	ATTTACCCCACTCCCCCTCCTCCCCTC	7	<nnxnp>	nn	252	np	0		
>ref-15675	ATTTACCCCACTCCCCCTCCTCCCCTC	8	<lmxll>	ll	15	lm	215		
>ref-15675	ATTTACCCCACTCCCCCTCCTCCCCTC	15	<nnxnp>	nn	251	np	0		
>ref-15678	GCTTCTTTCACTTTCTCTCCCGTCTTC	1	<hkxhk>	hh	63	hk	154	kk	46
>ref-15687	TCTAATGCAACCAGATCTCCATCAGCG	22	<lmxll>	ll	135	lm	129		
>ref-15692	ATTCTCATTACCCTTACTCCTCTACCC	2	<lmxll>	ll	116	lm	146		
>ref-15715	GTCTCTTCCACTTTCCCTCCTTCATTT	5	<nnxnp>	nn	263	np	0		
>ref-15720	TGACCTGACACTCGAGCTCCAATCTTA	16	<hkxhk>	hh	65	hk	138	kk	52
>ref-15732	AGTGGCCTCACCTCGTCTCCTAAGAAT	4	<hkxhk>	hh	60	hk	146	kk	44
>ref-15732	AGTGGCCTCACCTCGTCTCCTAAGAAT	5	<hkxhk>	hh	43	hk	146	kk	60
>ref-15786	GTGCTGTCAACACGAACTCCTTGCCCA	14	<lmxll>	ll	128	lm	140		
>ref-15791	TCCTCTCTCACTGATTCTCCCTCCGTC	8	<nnxnp>	nn	121	np	129		
>ref-15800	CGCGCCATAACCCACCCTCCGTCCTCC	4	<hkxhk>	hh	1	hk	269	kk	0
>ref-15800	CGCGCCATAACCCACCCTCCGTCCTCC	5	<hkxhk>	hh	3	hk	264	kk	0
>ref-15800	CGCGCCATAACCCACCCTCCGTCCTCC	24	<lmxll>	ll	197	lm	62		
>ref-15808	GAGGCGATGACAGTTCCTCCGCAACAT	1	<lmxll>	ll	127	lm	145		
>ref-15809	AGTGACCCCACTCCTTCTCCTCTCTCT	27	<hkxhk>	hh	37	hk	129	kk	68
>ref-15811	TCTTCTTAAACAAACGCTCCGTTGAGT	16	<lmxll>	ll	120	lm	115		
>ref-15811	TCTTCTTAAACAAACGCTCCGTTGAGT	25	<nnxnp>	nn	113	np	122		
>ref-15811	TCTTCTTAAACAAACGCTCCGTTGAGT	26	<nnxnp>	nn	113	np	122		
>ref-15814	TCCCTCAGCACCCCGACTCCCTTCCTT	8	<hkxhk>	hh	58	hk	155	kk	51
>ref-15842	TCCAGACAGACAGTTCCTCCCAAGGCA	1	<lmxll>	ll	258	lm	0		
>ref-15865	GTCCTCTGTACCTCTCCTCCTTCTTGG	6	<nnxnp>	nn	151	np	114		
>ref-15865	GTCCTCTGTACCTCTCCTCCTTCTTGG	12	<lmxll>	ll	130	lm	140		

>ref-15893	CCTTTTGGCACCGGTTCTCCCTGCGTT	9	<lmxll>	ll	147	lm	120		
>ref-15897	TGGGTACTCACGGATACTCCCTCTGTT	25	<nnxnp>	nn	124	np	144		
>ref-15906	TCTATTGTGACAAGAGCTCCATTAGTC	26	<nnxnp>	nn	125	np	144		
>ref-15925	TTTCTTCAAACATTGTCTCCCGTGTTT	5	<nnxnp>	nn	265	np	0		
>ref-15925	TTTCTTCAAACATTGTCTCCCGTGTTT	25	<nnxnp>	nn	121	np	124		
>ref-15926	TTCCTTTACACCCTTCCTCCCTTATCT	26	<nnxnp>	nn	140	np	126		
>ref-15927	TTACCTTCTACAATCTCTCCCAGTACT	3	<lmxll>	ll	114	lm	142		
>ref-15946	TAACTGAGGACTTTATCTCCTGCTCAT	4	<hkxhk>	hh	0	hk	264	kk	0
>ref-15946	TAACTGAGGACTTTATCTCCTGCTCAT	5	<nnxnp>	nn	126	np	127		
>ref-15946	TAACTGAGGACTTTATCTCCTGCTCAT	8	<hkxhk>	hh	96	hk	138	kk	0
>ref-15946	TAACTGAGGACTTTATCTCCTGCTCAT	23	<lmxll>	ll	45	lm	220		
>ref-15996	TATCCTCTTACCAAACCTCCTTTCCTC	12	<nnxnp>	nn	111	np	108		
>ref-16002	GCGCTGGAGACGCCCTCTCCTTCGTCG	25	<lmxll>	ll	143	lm	120		
>ref-16028	TGGTACAAAACTGCCCCTCCTCTAGGC	24	<nnxnp>	nn	263	np	0		
>ref-16043	TTTAAGGAAACTCATTCTCCTGCTGTT	26	<nnxnp>	nn	104	np	118		
>ref-16050	AACGTTGTAACTCATCCTCCCGCTTTC	16	<hkxhk>	hh	47	hk	150	kk	63
>ref-16053	TCAGGCACTACCGTTTCTCCTTCTACT	9	<lmxll>	ll	4	lm	263		
>ref-16073	TCCCTCATCACACTCTCTCCAACACAA	8	<nnxnp>	nn	105	np	134		
>ref-16076	ATTCGAGTGACAGTATCTCCCCGTGGA	2	<lmxll>	ll	127	lm	141		
>ref-16078	CCCACTACTACCTTCTCTCCCTCCCCC	24	<hkxhk>	hh	2	hk	180	kk	59
>ref-16078	CCCACTACTACCTTCTCTCCCTCCCCC	27	<hkxhk>	hh	52	hk	185	kk	2
>ref-16080	AGGTGCATAACCGTGTCTCCCAGGGCA	2	<lmxll>	ll	105	lm	147		
>ref-16080	AGGTGCATAACCGTGTCTCCCAGGGCA	9	<lmxll>	ll	105	lm	147		
>ref-16083	TGCGTCCTCACTTTTCCTCCTCATTTC	25	<lmxll>	ll	119	lm	136		
>ref-16097	GAATACATGACACTAGCTCCAAAGAGC	25	<lmxll>	ll	116	lm	138		
>ref-16103	AACACACACACAGAGACTCCGCCCCCC	1	<lmxll>	ll	138	lm	124		
>ref-16103	AACACACACACAGAGACTCCGCCCCCC	15	<hkxhk>	hh	7	hk	262	kk	1
>ref-16103	AACACACACACAGAGACTCCGCCCCCC	26	<hkxhk>	hh	7	hk	262	kk	1
>ref-16123	GTCCGTTTCACTACTCCTCCTAAACCA	15	<lmxll>	ll	134	lm	105		
>ref-16126	GGAGAACGAACAGCGCCTCCGGAAGGA	24	<hkxhk>	hh	51	hk	146	kk	73
>ref-16147	CATATAAACACACACGCTCCACATATT	23	<nnxnp>	nn	121	np	127		
>ref-16154	TCCTCCTTTACCTATCCTCCTACGTTT	13	<lmxll>	ll	119	lm	122		
>ref-16167	GTAGAAGACACCTGGTCTCCGTTGGAC	5	<lmxll>	ll	4	lm	264		
>ref-16182	TCGTATCTTACGTGGACTCCTGATACA	3	<nnxnp>	nn	120	np	141		
>ref-16182	TCGTATCTTACGTGGACTCCTGATACA	7	<nnxnp>	nn	134	np	131		
>ref-16183	TTTCCTCATACTCTCCCTCCCTCTTTT	7	<nnxnp>	nn	61	np	184		
>ref-16183	TTTCCTCATACTCTCCCTCCCTCTTTT	24	<nnxnp>	nn	60	np	187		
>ref-16184	CACCCAAACACACACACTCCTCCTTGC	14	<nnxnp>	nn	142	np	129		

>ref-16203	GAATCATTAACAATCACTCCGAGGTAT	21	<nnxnp>	nn	182	np	77		
>ref-16203	GAATCATTAACAATCACTCCGAGGTAT	23	<hkxhk>	hh	67	hk	199	kk	0
>ref-16203	GAATCATTAACAATCACTCCGAGGTAT	24	<lmxll>	ll	79	lm	171		
>ref-16208	TTCCCAGACACCCCCTCTCCCCTCCTC	7	<lmxll>	ll	7	lm	258		
>ref-16208	TTCCCAGACACCCCCTCTCCCCTCCTC	9	<lmxll>	ll	7	lm	258		
>ref-16210	TGACTCGTCACGCCCTCTCCTGCGGTT	5	<nnxnp>	nn	132	np	138		
>ref-16222	AACTGGGACACCAACACTCCCCAGAAT	12	<lmxll>	ll	119	lm	137		
>ref-16253	TTATCGTACACCACTCCTCCTCTTGTT	2	<lmxll>	ll	145	lm	123		
>ref-16253	TTATCGTACACCACTCCTCCTCTTGTT	8	<lmxll>	ll	56	lm	205		
>ref-16253	TTATCGTACACCACTCCTCCTCTTGTT	12	<lmxll>	ll	144	lm	123		
>ref-16253	TTATCGTACACCACTCCTCCTCTTGTT	15	<lmxll>	ll	56	lm	205		
>ref-16272	GGAAAAAGCACCGTATCTCCCGCCAGA	8	<nnxnp>	nn	110	np	110		
>ref-16299	CTTACAGTAACACCAGCTCCTCAGGCA	2	<hkxhk>	hh	54	hk	140	kk	54
>ref-16302	AACAGGGATACGCCCCCTCCTTCTCCT	27	<lmxll>	ll	132	lm	94		
>ref-16314	GCTTCCCTAACCCTCCCTCCAACCCTA	4	<lmxll>	ll	125	lm	134		
>ref-16318	TGACTAATAACCCCTCCTCCTTCCTCC	7	<nnxnp>	nn	46	np	214		
>ref-16347	GAAATGGTGACCATCGCTCCGGACCGA	3	<lmxll>	ll	1	lm	270		
>ref-16347	GAAATGGTGACCATCGCTCCGGACCGA	22	<hkxhk>	hh	0	hk	229	kk	35
>ref-16347	GAAATGGTGACCATCGCTCCGGACCGA	23	<hkxhk>	hh	2	hk	265	kk	0
>ref-16354	TTCTCTGCCACAGACTCTCCCTCTTTT	9	<nnxnp>	nn	132	np	121		
>ref-16360	CTTCCTATTACTCCCCCTCCTTTCCCC	5	<nnxnp>	nn	264	np	0		
>ref-16374	GTCTGCGCCACAAAGCCTCCGTGTTCC	21	<lmxll>	ll	131	lm	131		
>ref-16455	TTCACATATACATCCTCTCCTTTATTC	14	<nnxnp>	nn	117	np	138		
>ref-16455	TTCACATATACATCCTCTCCTTTATTC	27	<nnxnp>	nn	118	np	137		
>ref-16464	ATATTTGCAACAAGGCCTCCTCACAGA	22	<hkxhk>	hh	0	hk	234	kk	35
>ref-16506	TCCAACAAAACAACTGCTCCAGCAACT	5	<hkxhk>	hh	0	hk	270	kk	2
>ref-16506	TCCAACAAAACAACTGCTCCAGCAACT	27	<hkxhk>	hh	2	hk	270	kk	0
>ref-16549	CTACATGACACACCATCTCCGCTACAT	1	<hkxhk>	hh	0	hk	266	kk	3
>ref-16554	CTCAGTTCTACTCCTCCTCCTCCCCTC	5	<lmxll>	ll	10	lm	254		
>ref-16554	CTCAGTTCTACTCCTCCTCCTCCCCTC	13	<lmxll>	ll	10	lm	254		
>ref-16571	CATAGGGCCACTCTTCCTCCATTGCAT	3	<nnxnp>	nn	128	np	133		
>ref-16584	TCCGTTTTCACTTCTCCTCCTCTTTCT	8	<nnxnp>	nn	112	np	138		
>ref-16591	ACCACCATCACACCATCTCCATCAGAA	27	<hkxhk>	hh	58	hk	153	kk	41
>ref-16593	GTGAAACCCACGACCACTCCCCTCCTT	27	<lmxll>	ll	127	lm	132		
>ref-16608	CTTCTTCCTACTTCCCCTCCTCTCTCT	5	<hkxhk>	hh	28	hk	230	kk	0
>ref-16608	CTTCTTCCTACTTCCCCTCCTCTCTCT	15	<hkxhk>	hh	0	hk	226	kk	30
>ref-16624	TCCGAGCCCACACTTTCTCCTTTCTTC	22	<nnxnp>	nn	133	np	120		
>ref-16631	TCTTTTCATACTCCGCCTCCCACTCTT	8	<lmxll>	ll	120	lm	138		

>ref-16635	GGTCGTGACACCGGACCTCCGCTACAA	23	<lmxll>	ll	123	lm	150		
>ref-16641	CTTCCCCCTACGCACGCTCCTTCGTTA	1	<nnxnp>	nn	137	np	133		
>ref-16661	CTTTCGCCGACTTCACCTCCTACGTTC	4	<hkxhk>	hh	55	hk	158	kk	54
>ref-16664	ATCCGCTTTACAACACCTCCTCCCCTC	1	<lmxll>	ll	239	lm	11		
>ref-16664	ATCCGCTTTACAACACCTCCTCCCCTC	22	<nnxnp>	nn	110	np	131		
>ref-16686	CCGTGTTGCACTCTGCCTCCCTCCCGT	2	<lmxll>	ll	120	lm	147		
>ref-16686	CCGTGTTGCACTCTGCCTCCCTCCCGT	7	<nnxnp>	nn	127	np	146		
>ref-16691	CTGCGTGTTACCCTTTCTCCTTTCCCT	21	<lmxll>	ll	139	lm	124		
>ref-16720	CGTTCGCGCACCGACCCTCCTATATAA	5	<nnxnp>	nn	118	np	135		
>ref-16741	CATCCATCCACCCCTCCTCCTCCTTAC	13	<lmxll>	ll	142	lm	126		
>ref-16768	CAATCACCCACCAATCCTCCCGGACCC	2	<lmxll>	ll	125	lm	139		
>ref-16840	TTCCTGCATACTCACACTCCAGCCCTG	1	<nnxnp>	nn	124	np	106		
>ref-16852	GCTCCTCAAACCTATCCTCCTCTTTTA	4	<lmxll>	ll	122	lm	135		
>ref-16858	TATAAGTGTACACGATCTCCCTGACCG	4	<lmxll>	ll	138	lm	129		
>ref-16879	TTCTTCTCTACAGCCTCTCCTCACTCA	22	<nnxnp>	nn	106	np	155		
>ref-16911	CAAGTGGGGACACATCCTCCCCAACAC	27	<nnxnp>	nn	93	np	160		
>ref-16918	CTCTTTATCACCCATCCTCCCCTCATG	26	<lmxll>	ll	138	lm	123		
>ref-16923	TTAAACTTGACACTGTCTCCTTCAGTG	23	<lmxll>	ll	133	lm	125		
>ref-16927	TACTTGTCAACAGATACTCCATATATA	1	<nnxnp>	nn	119	np	132		
>ref-16932	TCGTCATAAACAGCTTCTCCCAAGTTA	23	<nnxnp>	nn	142	np	127		
>ref-16939	TAAATTCACACCTCGCCTCCCACTTTA	15	<hkxhk>	hh	2	hk	244	kk	6
>ref-16939	TAAATTCACACCTCGCCTCCCACTTTA	27	<hkxhk>	hh	2	hk	244	kk	6
>ref-16948	CATAACTTAACTGCCACTCCAAATGCA	1	<lmxll>	ll	131	lm	126		
>ref-16950	ACTCCATCCACACAACCTCCTGTACCA	23	<lmxll>	ll	134	lm	132		
>ref-16960	CTACAGCTTACCTTTCCTCCTCACTGC	24	<hkxhk>	hh	67	hk	136	kk	52
>ref-16961	CATTTGGCCACTGGTTCTCCTCTTCCT	24	<nnxnp>	nn	115	np	141		
>ref-16961	CATTTGGCCACTGGTTCTCCTCTTCCT	25	<nnxnp>	nn	114	np	142		
>ref-16968	ATGGCTATGACTAGTACTCCACCTCAG	13	<nnxnp>	nn	58	np	202		
>ref-17021	TCTGCTTGTACTTGATCTCCTCCTCCA	14	<lmxll>	ll	124	lm	144		
>ref-17029	ACCGGCAGCACGAACCCTCCCGAATAA	3	<nnxnp>	nn	270	np	0		
>ref-17029	ACCGGCAGCACGAACCCTCCCGAATAA	4	<nnxnp>	nn	128	np	130		
>ref-17042	TTGTCCACTACATCATCTCCCCGTGGC	8	<nnxnp>	nn	106	np	151		
>ref-17046	TAAGTGGTAACTGCCACTCCCCGTGTT	4	<hkxhk>	hh	0	hk	257	kk	9
>ref-17046	TAAGTGGTAACTGCCACTCCCCGTGTT	27	<hkxhk>	hh	0	hk	257	kk	9
>ref-17056	CTTATCTTCACTTTCTCTCCCTCTGTT	1	<hkxhk>	hh	0	hk	266	kk	1
>ref-17056	CTTATCTTCACTTTCTCTCCCTCTGTT	4	<hkxhk>	hh	1	hk	266	kk	0
>ref-17056	CTTATCTTCACTTTCTCTCCCTCTGTT	23	<lmxll>	ll	145	lm	111		
>ref-17057	TTATGCCCAACCTCTCCTCCTTCCCCT	1	<nnxnp>	nn	143	np	119		

>ref-17057	TTATGCCCAACCTCTCCTCCTTCCCCT	26	<hkxhk>	hh	0	hk	271	kk	1
>ref-17057	TTATGCCCAACCTCTCCTCCTTCCCCT	27	<hkxhk>	hh	0	hk	271	kk	1
>ref-17063	TACATAGCTACGGTTCCTCCCTTTAAT	1	<lmxll>	ll	122	lm	98		
>ref-17089	TTTCTTCCCACACGACCTCCGCCCTAA	1	<lmxll>	ll	115	lm	119		
>ref-17092	TTCTCTTAAACCTAATCTCCTATCCCT	9	<lmxll>	ll	104	lm	153		
>ref-17092	TTCTCTTAAACCTAATCTCCTATCCCT	14	<hkxhk>	hh	49	hk	147	kk	55
>ref-17097	CGCACTCGCACCTTGACTCCTTCATCC	25	<hkxhk>	hh	53	hk	130	kk	63
>ref-17131	AGAACTTATACCAAGACTCCTTGAAAT	1	<nnxnp>	nn	106	np	126		
>ref-17150	TCCACTCTCACTTAAACTCCTACTCTC	22	<hkxhk>	hh	81	hk	145	kk	18
>ref-17156	TCACCAAATACAGCTCCTCCATCACCA	14	<hkxhk>	hh	2	hk	269	kk	0
>ref-17156	TCACCAAATACAGCTCCTCCATCACCA	15	<nnxnp>	nn	74	np	168		
>ref-17182	ACTGGAAACACTGACACTCCGCTTTCA	4	<hkxhk>	hh	65	hk	194	kk	1
>ref-17182	ACTGGAAACACTGACACTCCGCTTTCA	5	<hkxhk>	hh	1	hk	194	kk	65
>ref-17182	ACTGGAAACACTGACACTCCGCTTTCA	13	<hkxhk>	hh	3	hk	202	kk	64
>ref-17182	ACTGGAAACACTGACACTCCGCTTTCA	21	<lmxll>	ll	93	lm	163		
>ref-17182	ACTGGAAACACTGACACTCCGCTTTCA	24	<nnxnp>	nn	132	np	135		
>ref-17190	GTGACATAGACCAGTACTCCTTCGCCG	1	<hkxhk>	hh	57	hk	149	kk	59
>ref-17240	CCATGCAGCACGCCCCCTCCCTCCACG	6	<nnxnp>	nn	145	np	122		
>ref-17252	TGCTCTGTCACCCCCTCTCCTTCTCAT	8	<nnxnp>	nn	110	np	139		
>ref-17259	AAATATAGGACTTCCTCTCCATATCCT	25	<nnxnp>	nn	134	np	116		
>ref-17277	AAAAAGTAAACGTTCCCTCCATCTTCA	15	<lmxll>	ll	124	lm	99		
>ref-17295	TTCTCTATCACTCCTTCTCCTTGTGTG	7	<lmxll>	ll	134	lm	129		
>ref-17308	TACACACACACAGGGCCTCCATAGTCT	3	<hkxhk>	hh	63	hk	114	kk	44
>ref-17324	ACTTTTTCAACTCTCCCTCCCTCCCTC	6	<hkxhk>	hh	0	hk	213	kk	41
>ref-17324	ACTTTTTCAACTCTCCCTCCCTCCCTC	8	<hkxhk>	hh	2	hk	179	kk	61
>ref-17324	ACTTTTTCAACTCTCCCTCCCTCCCTC	24	<hkxhk>	hh	2	hk	178	kk	61
>ref-17335	GAGGTGGGTACGGCGGCTCCGTTGCCA	1	<lmxll>	ll	131	lm	141		
>ref-17336	ACTCCCTCCACTCCCCCTCCACTGCCC	7	<hkxhk>	hh	0	hk	273	kk	0
>ref-17336	ACTCCCTCCACTCCCCCTCCACTGCCC	24	<hkxhk>	hh	0	hk	273	kk	0
>ref-17341	TCCTCTATTACTCCTTCTCCCCTTCCT	22	<hkxhk>	hh	0	hk	266	kk	0
>ref-17341	TCCTCTATTACTCCTTCTCCCCTTCCT	25	<hkxhk>	hh	1	hk	266	kk	0
>ref-17369	CTCCATCCCACCTTCCCTCCCGCATTC	13	<nnxnp>	nn	263	np	2		
>ref-17369	CTCCATCCCACCTTCCCTCCCGCATTC	22	<nnxnp>	nn	7	np	258		
>ref-17372	ATTACTCGCACTGGTGCTCCTCCTCCT	4	<hkxhk>	hh	0	hk	273	kk	0
>ref-17372	ATTACTCGCACTGGTGCTCCTCCTCCT	16	<hkxhk>	hh	0	hk	273	kk	0
>ref-17379	CGCTCACTCACTCTCCCTCCCTCCCCT	2	<hkxhk>	hh	0	hk	250	kk	4
>ref-17379	CGCTCACTCACTCTCCCTCCCTCCCCT	15	<lmxll>	ll	41	lm	225		
>ref-17389	CTCATTCACACATGAGCTCCGGGTGCC	16	<nnxnp>	nn	137	np	127		

>ref-17406	GCTGTTCCTACCCCTCCTCCTCCTACA	9	<lmxll>	ll	135	lm	121		
>ref-17406	GCTGTTCCTACCCCTCCTCCTCCTACA	23	<lmxll>	ll	113	lm	147		
>ref-17414	TCCACCCCTACCTTTCCTCCCTCTCTC	13	<lmxll>	ll	3	lm	223		
>ref-17414	TCCACCCCTACCTTTCCTCCCTCTCTC	15	<lmxll>	ll	109	lm	121		
>ref-17414	TCCACCCCTACCTTTCCTCCCTCTCTC	21	<lmxll>	ll	4	lm	223		
>ref-17414	TCCACCCCTACCTTTCCTCCCTCTCTC	27	<lmxll>	ll	117	lm	119		
>ref-17434	CATTCACTCACTTTCTCTCCCACTCAT	4	<hkxhk>	hh	0	hk	182	kk	68
>ref-17434	CATTCACTCACTTTCTCTCCCACTCAT	21	<lmxll>	ll	189	lm	70		
>ref-17436	CTCTGTTATACTAACTCTCCTCTCCTC	22	<lmxll>	ll	128	lm	132		
>ref-17436	CTCTGTTATACTAACTCTCCTCTCCTC	25	<hkxhk>	hh	71	hk	132	kk	45
>ref-17436	CTCTGTTATACTAACTCTCCTCTCCTC	27	<hkxhk>	hh	71	hk	133	kk	45
>ref-17446	TGGATGTGTACCTTGTCTCCTCAGACC	16	<lmxll>	ll	113	lm	149		
>ref-17459	GGTTGACAGACTACCTCTCCATAAGGT	7	<hkxhk>	hh	18	hk	230	kk	0
>ref-17459	GGTTGACAGACTACCTCTCCATAAGGT	9	<hkxhk>	hh	0	hk	230	kk	18
>ref-17465	GGTATTTCTACCTTATCTCCCCTTTCC	3	<hkxhk>	hh	0	hk	270	kk	1
>ref-17477	CTTCACCTCACTCCTCCTCCACCCCCT	3	<lmxll>	ll	14	lm	240		
>ref-17477	CTTCACCTCACTCCTCCTCCACCCCCT	5	<lmxll>	ll	14	lm	234		
>ref-17493	CTTGTGGGAACCGACTCTCCTCGTCCA	5	<nnxnp>	nn	150	np	122		
>ref-17495	CTGTTCGATACGATTTCTCCAACCCTT	1	<lmxll>	ll	123	lm	139		
>ref-17500	GGTCAGCGCACTGTTCCTCCTCTGTGT	16	<lmxll>	ll	124	lm	121		
>ref-17504	CCTCTTCCGACCTATCCTCCTCCTGCT	12	<hkxhk>	hh	0	hk	273	kk	0
>ref-17504	CCTCTTCCGACCTATCCTCCTCCTGCT	24	<hkxhk>	hh	0	hk	273	kk	0
>ref-17510	TATCCACACACCTTATCTCCAGCGTCA	1	<lmxll>	ll	128	lm	120		
>ref-17520	ACAACCTCCACTACCTCTCCACCTCCC	6	<nnxnp>	nn	118	np	105		
>ref-17520	ACAACCTCCACTACCTCTCCACCTCCC	7	<lmxll>	ll	97	lm	142		
>ref-17533	CAGTTCAGAACCAAGGCTCCCCTTTCG	22	<nnxnp>	nn	117	np	130		
>ref-17539	CACAGACACACGCACGCTCCTCCCTCC	3	<nnxnp>	nn	168	np	105		
>ref-17548	CACGAGAGGACGTACCCTCCGCGCGGC	27	<lmxll>	ll	254	lm	0		
>ref-17567	GAATCGGTTACCTTTACTCCAAGCTCT	9	<lmxll>	ll	133	lm	133		
>ref-17601	CCCTCCTCTACCTTCCCTCCTCGGCAT	1	<hkxhk>	hh	1	hk	256	kk	3
>ref-17601	CCCTCCTCTACCTTCCCTCCTCGGCAT	7	<hkxhk>	hh	3	hk	255	kk	2
>ref-17601	CCCTCCTCTACCTTCCCTCCTCGGCAT	21	<hkxhk>	hh	1	hk	253	kk	6
>ref-17620	ACCTCGTAGACGAGATCTCCTGAGAGT	9	<nnxnp>	nn	136	np	128		
>ref-17626	CATCAACGGACATCACCTCCATCAACG	9	<nnxnp>	nn	265	np	3		
>ref-17626	CATCAACGGACATCACCTCCATCAACG	14	<hkxhk>	hh	29	hk	214	kk	0
>ref-17626	CATCAACGGACATCACCTCCATCAACG	27	<hkxhk>	hh	0	hk	272	kk	0
>ref-17631	TCCACCCCCACGCCCACTCCCTCTCCC	1	<hkxhk>	hh	0	hk	273	kk	0
>ref-17631	TCCACCCCCACGCCCACTCCCTCTCCC	4	<hkxhk>	hh	4	hk	265	kk	0

>ref-17631	TCCACCCCCACGCCCACTCCCTCTCCC	6	<hkxhk>	hh	0	hk	251	kk	0
>ref-17631	TCCACCCCCACGCCCACTCCCTCTCCC	12	<hkxhk>	hh	1	hk	268	kk	0
>ref-17641	TACTGCTACACGTGTTCTCCCTCCATG	2	<nnxnp>	nn	126	np	144		
>ref-17686	TACTCTGTTACTCTCCCTCCCCTTCCC	13	<lmxll>	ll	116	lm	120		
>ref-17686	TACTCTGTTACTCTCCCTCCCCTTCCC	26	<lmxll>	ll	115	lm	121		
>ref-17698	CTTATTTTGACTTATCCTCCCCACTCT	23	<lmxll>	ll	108	lm	133		
>ref-17708	GCAACCTCAACCCGACCTCCCTATCGT	24	<lmxll>	ll	151	lm	119		
>ref-17712	TAATCCTGCACGGTTACTCCTTCCGTT	6	<lmxll>	ll	139	lm	126		
>ref-17754	CTTGATTTGACCTGACCTCCTCCCTTC	7	<hkxhk>	hh	51	hk	153	kk	53
>ref-17766	GACAGACAGACGAATGCTCCCGCGAAC	6	<lmxll>	ll	140	lm	132		
>ref-17800	CACACACACACGGAAGCTCCTCCCAAC	16	<hkxhk>	hh	0	hk	254	kk	11
>ref-17800	CACACACACACGGAAGCTCCTCCCAAC	25	<hkxhk>	hh	11	hk	254	kk	0
>ref-17801	CGAAAATGAACCGCGGCTCCAGTGTGC	12	<lmxll>	ll	142	lm	93		
>ref-17824	CGCCACGCCACGTTCGCTCCCGTACCA	25	<nnxnp>	nn	263	np	0		
>ref-17837	TTATATACTACTACTACTCCTACTATT	2	<hkxhk>	hh	17	hk	201	kk	2
>ref-17837	TTATATACTACTACTACTCCTACTATT	9	<hkxhk>	hh	16	hk	203	kk	2
>ref-17841	TCTTCTTTTACTCATCCTCCTTCTTTC	3	<hkxhk>	hh	49	hk	141	kk	71
>ref-17862	CACCATCCTACTACCCCTCCTTTCGCT	7	<nnxnp>	nn	137	np	130		
>ref-17886	TGGCTCCAAACCATGTCTCCCTAGCTT	2	<nnxnp>	nn	118	np	105		
>ref-17889	TCCTTCCCTACCTCGCCTCCCCCTCCC	4	<lmxll>	ll	233	lm	22		
>ref-17889	TCCTTCCCTACCTCGCCTCCCCCTCCC	15	<lmxll>	ll	86	lm	154		
>ref-17891	ATTATCATCACTAACTCTCCTCATTAC	5	<hkxhk>	hh	58	hk	127	kk	51
>ref-17891	ATTATCATCACTAACTCTCCTCATTAC	22	<hkxhk>	hh	51	hk	127	kk	58
>ref-17894	CTGAAGGCCACCTCAACTCCCAACTAT	3	<lmxll>	ll	197	lm	59		
>ref-17898	GGAGGAGAGACAGAACCTCCTCAGACA	4	<hkxhk>	hh	0	hk	203	kk	67
>ref-17898	GGAGGAGAGACAGAACCTCCTCAGACA	21	<hkxhk>	hh	0	hk	203	kk	67
>ref-17909	CAACATAGCACAGCTTCTCCTTGATGT	2	<hkxhk>	hh	0	hk	259	kk	0
>ref-17909	CAACATAGCACAGCTTCTCCTTGATGT	5	<hkxhk>	hh	0	hk	272	kk	0
>ref-17942	TGTCTGTACACACACACTCCCATATAC	22	<lmxll>	ll	122	lm	133		
>ref-17954	CCACCATCTACTCAAGCTCCAAGAAGG	24	<hkxhk>	hh	2	hk	267	kk	0
>ref-17954	CCACCATCTACTCAAGCTCCAAGAAGG	27	<nnxnp>	nn	140	np	126		
>ref-17979	TCCTTTCGTACTGTTTCTCCTCTTTCT	22	<lmxll>	ll	145	lm	121		
>ref-18008	CTTAGGCCCACTTTGTCTCCCTTTGTC	8	<lmxll>	ll	137	lm	130		
>ref-18009	CTGCCTCCAACCTCTCCTCCGTCCCGC	2	<lmxll>	ll	98	lm	155		
>ref-18009	CTGCCTCCAACCTCTCCTCCGTCCCGC	3	<lmxll>	ll	98	lm	155		
>ref-18037	CTGGGAACGACTCTCCCTCCGTCCAAC	23	<nnxnp>	nn	268	np	0		
>ref-18050	AGGAGACACACGGCGGCTCCGGGCTTA	16	<lmxll>	ll	137	lm	131		
>ref-18063	ATGTCGAGTACGGACGCTCCGTGGTCG	9	<lmxll>	ll	266	lm	1		

>ref-18099	CTCCCCTTCACCTCCCCTCCTGTTCAC	4	<nnxnp>	nn	270	np	0		
>ref-18099	CTCCCCTTCACCTCCCCTCCTGTTCAC	26	<hkxhk>	hh	2	hk	262	kk	0
>ref-18099	CTCCCCTTCACCTCCCCTCCTGTTCAC	27	<hkxhk>	hh	0	hk	262	kk	2
>ref-18138	TCATTTCACACTTCATCTCCTCTCTTC	5	<hkxhk>	hh	61	hk	140	kk	48
>ref-18143	TCATCTCCCACCTTCTCTCCCTCATCC	3	<lmxll>	ll	3	lm	267		
>ref-18143	TCATCTCCCACCTTCTCTCCCTCATCC	13	<lmxll>	ll	3	lm	267		
>ref-18143	TCATCTCCCACCTTCTCTCCCTCATCC	14	<hkxhk>	hh	0	hk	197	kk	72
>ref-18149	GATCCCATCACCCTAACTCCTTCTCCT	1	<lmxll>	ll	255	lm	1		
>ref-18149	GATCCCATCACCCTAACTCCTTCTCCT	2	<lmxll>	ll	259	lm	0		
>ref-18151	TCTAATTCAACTCTGCCTCCCCCTATT	9	<hkxhk>	hh	53	hk	143	kk	56
>ref-18151	TCTAATTCAACTCTGCCTCCCCCTATT	27	<hkxhk>	hh	56	hk	146	kk	53
>ref-18178	TAAAAGATAACGATCCCTCCTTCGTCC	25	<lmxll>	ll	123	lm	126		
>ref-18188	TTGCCCTTCACCTGGCCTCCTTTTTAT	3	<hkxhk>	hh	44	hk	130	kk	64
>ref-18218	TCCTCTAATACCCTCTCTCCTTCTGCT	26	<lmxll>	ll	129	lm	121		
>ref-18223	AAAAAACTCACGGTTTCTCCTGCTAAT	3	<nnxnp>	nn	126	np	121		
>ref-18236	GATTCTCCTACCCTTCCTCCCCATTAT	26	<lmxll>	ll	128	lm	114		
>ref-18253	CTCGGTCTCACTTTCTCTCCTCATTCT	24	<nnxnp>	nn	104	np	158		
>ref-18253	CTCGGTCTCACTTTCTCTCCTCATTCT	25	<nnxnp>	nn	105	np	158		
>ref-18254	TCATGGATGACAGTGCCTCCTCCACCT	7	<hkxhk>	hh	0	hk	273	kk	0
>ref-18254	TCATGGATGACAGTGCCTCCTCCACCT	16	<hkxhk>	hh	0	hk	273	kk	0
>ref-18255	TCTTCTGTCACCCACTCTCCCTCTCTT	12	<nnxnp>	nn	143	np	109		
>ref-18289	CCCACTTTTACCCACTCTCCCACTTGC	13	<hkxhk>	hh	0	hk	92	kk	137
>ref-18289	CCCACTTTTACCCACTCTCCCACTTGC	15	<lmxll>	ll	28	lm	224		
>ref-18289	CCCACTTTTACCCACTCTCCCACTTGC	22	<nnxnp>	nn	217	np	37		
>ref-18289	CCCACTTTTACCCACTCTCCCACTTGC	26	<hkxhk>	hh	0	hk	92	kk	138
>ref-18295	CCCGCACGTACTTTACCTCCGAGAATG	2	<hkxhk>	hh	50	hk	150	kk	57
>ref-18304	CAAGAATCTACTTGAACTCCTTATGTC	8	<lmxll>	ll	137	lm	126		
>ref-18323	CGAGGCAAGACAGGGCCTCCGTTGCAC	24	<lmxll>	ll	137	lm	136		
>ref-18350	AAGGCTGGGACAGTTGCTCCAGGTTTG	24	<lmxll>	ll	138	lm	100		
>ref-18359	CCTCACCCTACCCTGCCTCCTTCCTCC	2	<nnxnp>	nn	257	np	0		
>ref-18359	CCTCACCCTACCCTGCCTCCTTCCTCC	7	<nnxnp>	nn	260	np	0		
>ref-18360	AGACTGTTCACGCCTGCTCCGTTAGCC	4	<hkxhk>	hh	68	hk	123	kk	49
>ref-18373	CACTAAACCACCCCTTCTCCTTGCCTT	7	<nnxnp>	nn	140	np	127		
>ref-18379	ACGCAATACACAACTCCTCCGTCCCAG	7	<nnxnp>	nn	133	np	118		
>ref-18388	AGCCTATCTACTCCCCCTCCTCCCCCT	12	<lmxll>	ll	109	lm	135		
>ref-18388	AGCCTATCTACTCCCCCTCCTCCCCCT	25	<nnxnp>	nn	122	np	128		
>ref-18394	TTTGTGCTCACAGTATCTCCTACACCA	7	<lmxll>	ll	115	lm	122		
>ref-18397	TTAGTAATCACTGAGCCTCCATTTCTA	5	<lmxll>	ll	169	lm	81		

>ref-18397	TTAGTAATCACTGAGCCTCCATTTCTA	7	<hkxhk>	hh	0	hk	260	kk	3
>ref-18397	TTAGTAATCACTGAGCCTCCATTTCTA	9	<lmxll>	ll	168	lm	82		
>ref-18397	TTAGTAATCACTGAGCCTCCATTTCTA	15	<nnxnp>	nn	147	np	112		
>ref-18397	TTAGTAATCACTGAGCCTCCATTTCTA	25	<hkxhk>	hh	0	hk	260	kk	3
>ref-18420	TCCCTACCAACTTTTCCTCCTCTCTTC	7	<hkxhk>	hh	2	hk	247	kk	14
>ref-18420	TCCCTACCAACTTTTCCTCCTCTCTTC	23	<hkxhk>	hh	13	hk	247	kk	2
>ref-18440	GCTCTCTCCACTTCCCCTCCCACACCC	1	<hkxhk>	hh	62	hk	139	kk	55
>ref-18448	ATCGTCTCCACATTCCCTCCCTTTTCC	1	<lmxll>	ll	119	lm	149		
>ref-18454	CCATTCCTCACACCATCTCCTTCCCCC	4	<hkxhk>	hh	47	hk	149	kk	62
>ref-18455	TGTGAGACTACTGCTGCTCCTACGATT	22	<lmxll>	ll	106	lm	137		
>ref-18455	TGTGAGACTACTGCTGCTCCTACGATT	23	<lmxll>	ll	107	lm	137		
>ref-18464	AAGAGAAGTACAGCCCCTCCAACCCAG	27	<nnxnp>	nn	145	np	125		
>ref-18472	ACCTCATCCACTCGAACTCCTAGGCTC	14	<nnxnp>	nn	130	np	133		
>ref-18472	ACCTCATCCACTCGAACTCCTAGGCTC	15	<nnxnp>	nn	131	np	132		
>ref-18500	TCACCTCCAACAATAGCTCCACCTCCA	15	<lmxll>	ll	110	lm	158		
>ref-18500	TCACCTCCAACAATAGCTCCACCTCCA	25	<nnxnp>	nn	186	np	72		
>ref-18503	ACACCCATCACTACCCCTCCCCAATGC	27	<nnxnp>	nn	106	np	123		
>ref-18514	CCCTCGACGACAGACTCTCCTGGGACC	2	<hkxhk>	hh	0	hk	273	kk	0
>ref-18514	CCCTCGACGACAGACTCTCCTGGGACC	12	<hkxhk>	hh	2	hk	271	kk	0
>ref-18514	CCCTCGACGACAGACTCTCCTGGGACC	26	<hkxhk>	hh	209	hk	53	kk	0
>ref-18514	CCCTCGACGACAGACTCTCCTGGGACC	27	<hkxhk>	hh	0	hk	273	kk	0
>ref-18569	GGGTTTTGCACATGCACTCCCTACACA	23	<hkxhk>	hh	150	hk	100	kk	0
>ref-18569	GGGTTTTGCACATGCACTCCCTACACA	26	<hkxhk>	hh	150	hk	100	kk	0
>ref-18598	GGGAGGAATACAAGCTCTCCTTTGAGA	8	<hkxhk>	hh	0	hk	267	kk	1
>ref-18598	GGGAGGAATACAAGCTCTCCTTTGAGA	13	<hkxhk>	hh	0	hk	267	kk	1
>ref-18622	CTGCCCCCCACGTCACCTCCGTGTCGC	3	<nnxnp>	nn	123	np	124		
>ref-18648	CACGTACTTACACCGCCTCCAGTCAAA	3	<lmxll>	ll	130	lm	119		
>ref-18662	AATTTGGGTACAGACCCTCCCGCAATG	22	<lmxll>	ll	134	lm	108		
>ref-18671	TCTCCTCCTACCCAGTCTCCCCCTCTC	22	<lmxll>	ll	258	lm	1		
>ref-18693	TCCATAATAACTGCACCTCCTCCCCCC	7	<hkxhk>	hh	46	hk	142	kk	73
>ref-18707	ACTGCTCCCACGTCCTCTCCCACCCAG	3	<hkxhk>	hh	62	hk	133	kk	64
>ref-18728	CATCCTTTCACTCTCTCTCCCTTTGTT	5	<hkxhk>	hh	0	hk	253	kk	13
>ref-18728	CATCCTTTCACTCTCTCTCCCTTTGTT	12	<hkxhk>	hh	0	hk	252	kk	13
>ref-18728	CATCCTTTCACTCTCTCTCCCTTTGTT	13	<hkxhk>	hh	2	hk	271	kk	0
>ref-18759	TGGATGAGAACCATCACTCCATTAGTA	7	<nnxnp>	nn	134	np	131		
>ref-18761	TGAATTTAGACGTCATCTCCTACATTG	23	<lmxll>	ll	140	lm	122		
>ref-18762	TCATACGTTACCAGAGCTCCGTGATGA	26	<nnxnp>	nn	105	np	124		
>ref-18766	TTCACTCTTACGGCGTCTCCATTACCG	14	<lmxll>	ll	124	lm	109		

>ref-18797	CCATTCCCTACCCTCCCTCCCTTCCTC	1	<nnxnp>	nn	245	np	17		
>ref-18797	CCATTCCCTACCCTCCCTCCCTTCCTC	23	<nnxnp>	nn	12	np	249		
>ref-18848	CTTCCAAACACGAGGGCTCCTCATGGT	8	<lmxll>	ll	141	lm	100		
>ref-18848	CTTCCAAACACGAGGGCTCCTCATGGT	27	<lmxll>	ll	144	lm	98		
>ref-18880	GAACTCCCCACATCCACTCCTCTCCCT	13	<nnxnp>	nn	106	np	144		
>ref-18896	ACACACAAAACACACACTCCATTTCTC	4	<nnxnp>	nn	128	np	141		
>ref-18926	TTACTTTCAACTCATCCTCCCTCTTAT	5	<lmxll>	ll	123	lm	122		
>ref-18926	TTACTTTCAACTCATCCTCCCTCTTAT	6	<lmxll>	ll	122	lm	122		
>ref-18932	TGTAATCCTACAGGTCCTCCTACAAGT	25	<hkxhk>	hh	0	hk	273	kk	0
>ref-18936	GGTTCAAGCACAGCCGCTCCATTATCA	6	<hkxhk>	hh	67	hk	116	kk	54
>ref-18936	GGTTCAAGCACAGCCGCTCCATTATCA	16	<hkxhk>	hh	54	hk	116	kk	66
>ref-18949	CTCCCTCCCACTCCTTCTCCTGGTCCC	7	<lmxll>	ll	109	lm	130		
>ref-18956	CTCCTCCCAACTCTGCCTCCCTCCCCT	8	<nnxnp>	nn	109	np	160		
>ref-18968	CGGTGGAGGACGTCTGCTCCATGTTCG	13	<nnxnp>	nn	145	np	124		
>ref-18972	AACAGGGGGACGAGATCTCCTCCGTGG	22	<lmxll>	ll	134	lm	133		
>ref-18978	ATCTATCGAACGAGACCTCCTATGCTC	21	<nnxnp>	nn	113	np	144		
>ref-19012	ACCACATAAACCGAACCTCCACCCCAG	25	<hkxhk>	hh	0	hk	273	kk	0
>ref-19012	ACCACATAAACCGAACCTCCACCCCAG	26	<hkxhk>	hh	5	hk	265	kk	0
>ref-19012	ACCACATAAACCGAACCTCCACCCCAG	27	<hkxhk>	hh	6	hk	265	kk	0
>ref-19079	AGAAGCAGGACGGAGCCTCCAACCCAT	14	<nnxnp>	nn	129	np	143		
>ref-19132	CACGCACAAACACAAACTCCATTTCCC	5	<lmxll>	ll	135	lm	128		
>ref-19164	TACAGCACCACAGAAACTCCGGACCCT	24	<hkxhk>	hh	55	hk	153	kk	51
>ref-19181	CTATTTCTTACGGTCTCTCCTCTCCTC	14	<lmxll>	ll	124	lm	136		
>ref-19194	CCCCGCTCAACACTCTCTCCTTTCTCT	22	<lmxll>	ll	147	lm	121		
>ref-19200	CAGATCTGCACAGTTTCTCCAACTGAC	5	<lmxll>	ll	124	lm	122		
>ref-19256	TCATGATTCACTCAGGCTCCAGGGGAT	14	<nnxnp>	nn	130	np	122		
>ref-19260	CCTCCTGCTACTTCTTCTCCATCATCA	1	<hkxhk>	hh	40	hk	126	kk	68
>ref-19278	TCCTTCCCGACCCTCGCTCCGCTTCCT	12	<nnxnp>	nn	125	np	143		
>ref-19280	ACAAATAAAACACATACTCCACATACT	24	<lmxll>	ll	116	lm	136		
>ref-19283	TATCCTCCCACCTTTCCTCCCTCTTTT	4	<lmxll>	ll	132	lm	128		
>ref-19305	ATTAAATCCACAACACCTCCCTCCCTC	16	<lmxll>	ll	138	lm	105		
>ref-19307	GTCGATAGCACTTCCGCTCCTGTATGG	4	<nnxnp>	nn	148	np	119		
>ref-19323	CACATTTATACATTGCCTCCCTTCTCA	12	<nnxnp>	nn	118	np	121		
>ref-19331	TTATAATGGACCGTCTCTCCCGTCTGT	7	<hkxhk>	hh	55	hk	152	kk	57
>ref-19331	TTATAATGGACCGTCTCTCCCGTCTGT	23	<hkxhk>	hh	55	hk	152	kk	57
>ref-19338	TCACTGGCCACCCAACCTCCTGCCTCC	3	<lmxll>	ll	240	lm	8		
>ref-19363	ACACACTTCACTCACTCTCCACAAGGT	16	<lmxll>	ll	106	lm	157		
>ref-19381	TTCACGGCAACATCCTCTCCTATATGG	23	<nnxnp>	nn	111	np	140		

>ref-19381	TTCACGGCAACATCCTCTCCTATATGG	24	<hkxhk>	hh	53	hk	179	kk	0
>ref-19381	TTCACGGCAACATCCTCTCCTATATGG	27	<nnxnp>	nn	111	np	141		
>ref-19434	TGACGACGTACCAACACTCCACTCACT	6	<lmxll>	ll	132	lm	132		
>ref-19435	ACATTCTTTACATTCTCTCCTTCTCCT	15	<lmxll>	ll	113	lm	140		
>ref-19443	GTCATTGCAACCATCACTCCTCCGGGT	14	<nnxnp>	nn	270	np	3		
>ref-19445	TTCCTGCCTACTCTCTCTCCTCCAACC	4	<hkxhk>	hh	48	hk	128	kk	62
>ref-19445	TTCCTGCCTACTCTCTCTCCTCCAACC	5	<hkxhk>	hh	62	hk	127	kk	48
>ref-19452	CTTCATCCCACACAATCTCCTGATCCT	26	<lmxll>	ll	104	lm	165		
>ref-19464	GCTAACGAGACAGCCCCTCCAGCCAAT	2	<hkxhk>	hh	53	hk	148	kk	56
>ref-19464	GCTAACGAGACAGCCCCTCCAGCCAAT	16	<hkxhk>	hh	53	hk	148	kk	56
>ref-19469	AAGACGCTCACTAGGCCTCCTTCCCAG	21	<lmxll>	ll	47	lm	218		
>ref-19469	AAGACGCTCACTAGGCCTCCTTCCCAG	27	<nnxnp>	nn	163	np	102		
>ref-19470	GAGTGGATGACTCGACCTCCAGAATCT	3	<hkxhk>	hh	0	hk	226	kk	35
>ref-19470	GAGTGGATGACTCGACCTCCAGAATCT	13	<lmxll>	ll	134	lm	126		
>ref-19470	GAGTGGATGACTCGACCTCCAGAATCT	22	<lmxll>	ll	135	lm	126		
>ref-19481	GTCTTTTTTACAAGCTCTCCCTTTCTA	6	<lmxll>	ll	107	lm	139		
>ref-19487	TACCTCCCCACGCCATCTCCCCTCTCA	12	<lmxll>	ll	137	lm	132		
>ref-19528	TCCTCTTGAACCCCCCCTCCCCCTCCC	2	<lmxll>	ll	77	lm	167		
>ref-19528	TCCTCTTGAACCCCCCCTCCCCCTCCC	8	<lmxll>	ll	41	lm	210		
>ref-19551	CCTCAACCCACTGCTACTCCTTGAACA	7	<hkxhk>	hh	43	hk	134	kk	66
>ref-19587	ATTAGTGGCACTGTATCTCCCCGAGCT	21	<nnxnp>	nn	127	np	137		
>ref-19587	ATTAGTGGCACTGTATCTCCCCGAGCT	27	<nnxnp>	nn	127	np	137		
>ref-19597	TCCTTATTTACTCCCTCTCCCCTTTTC	2	<lmxll>	ll	196	lm	53		
>ref-19597	TCCTTATTTACTCCCTCTCCCCTTTTC	6	<nnxnp>	nn	62	np	194		
>ref-19597	TCCTTATTTACTCCCTCTCCCCTTTTC	15	<lmxll>	ll	196	lm	55		
>ref-19597	TCCTTATTTACTCCCTCTCCCCTTTTC	24	<nnxnp>	nn	38	np	214		
>ref-19597	TCCTTATTTACTCCCTCTCCCCTTTTC	26	<hkxhk>	hh	0	hk	272	kk	0
>ref-19598	CACACATACACACATACTCCAGAATGC	7	<nnxnp>	nn	20	np	202		
>ref-19598	CACACATACACACATACTCCAGAATGC	15	<nnxnp>	nn	20	np	200		
>ref-19607	CATCCCCCAACACCATCTCCTTCCATA	14	<lmxll>	ll	14	lm	244		
>ref-19618	AGCGGAACGACAAAGTCTCCCCTGAAA	24	<hkxhk>	hh	0	hk	262	kk	0
>ref-19638	CCCTCCTACACCCCCACTCCCTCCCCT	8	<lmxll>	ll	194	lm	48		
>ref-19638	CCCTCCTACACCCCCACTCCCTCCCCT	15	<lmxll>	ll	241	lm	15		
>ref-19673	TATAACACCACACGAGCTCCTCATCTT	14	<lmxll>	ll	129	lm	126		
>ref-19674	AATACGATTACACAAGCTCCTTTCTCA	15	<nnxnp>	nn	143	np	105		
>ref-19748	CGCTTACTCACCCACACTCCAACCGTC	22	<nnxnp>	nn	266	np	0		
>ref-19750	TTTCCTCTCACTCAATCTCCCTCTCTT	26	<hkxhk>	hh	19	hk	240	kk	1
>ref-19750	TTTCCTCTCACTCAATCTCCCTCTCTT	27	<nnxnp>	nn	261	np	3		

>ref-19764	TGTGCATATACAAACACTCCTTTGCCT	15	<lmxll>	ll	138	lm	130		
>ref-19771	CCCCACCTCACCACCACTCCCCTCCAA	5	<lmxll>	ll	162	lm	94		
>ref-19771	CCCCACCTCACCACCACTCCCCTCCAA	6	<lmxll>	ll	62	lm	200		
>ref-19771	CCCCACCTCACCACCACTCCCCTCCAA	22	<hkxhk>	hh	141	hk	122	kk	0
>ref-19771	CCCCACCTCACCACCACTCCCCTCCAA	24	<lmxll>	ll	232	lm	31		
>ref-19777	ACCCCAAATACTGCCACTCCCTTATAC	3	<nnxnp>	nn	255	np	1		
>ref-19807	CGAATAGCCACAAAGACTCCTGAATAA	6	<lmxll>	ll	114	lm	137		
>ref-19850	CTCCACCTCACAGACTCTCCTCTGTCT	9	<nnxnp>	nn	109	np	152		
>ref-19854	GAGGCCCTAACATGACCTCCTCGTCTG	21	<hkxhk>	hh	0	hk	266	kk	2
>ref-19854	GAGGCCCTAACATGACCTCCTCGTCTG	22	<hkxhk>	hh	2	hk	266	kk	0
>ref-19856	GCTGATCAGACCACCGCTCCTCTCTTT	24	<lmxll>	ll	131	lm	137		
>ref-19878	CCTGGGGAGACATTACCTCCCGACAAA	15	<lmxll>	ll	134	lm	113		
>ref-19878	CCTGGGGAGACATTACCTCCCGACAAA	23	<lmxll>	ll	133	lm	113		
>ref-19905	TTCCCTGCCACCTCTTCTCCCCCTCCT	7	<lmxll>	ll	202	lm	52		
>ref-19905	TTCCCTGCCACCTCTTCTCCCCCTCCT	14	<lmxll>	ll	202	lm	50		
>ref-19914	GCTTCTCGCACCTGCTCTCCAACATCC	15	<nnxnp>	nn	121	np	148		
>ref-19920	CGAACAAAAACCCAAACTCCACCGAAA	8	<lmxll>	ll	103	lm	121		
>ref-19929	GTTTTGTTTACTTCAGCTCCACCTCTT	5	<lmxll>	ll	126	lm	117		
>ref-19929	GTTTTGTTTACTTCAGCTCCACCTCTT	25	<nnxnp>	nn	126	np	116		
>ref-19937	CTCCTGCCCACAGCCCCTCCCACACCC	5	<lmxll>	ll	99	lm	141		
>ref-19937	CTCCTGCCCACAGCCCCTCCCACACCC	6	<lmxll>	ll	100	lm	140		
>ref-19971	ACCTCCAGGACTCCGTCTCCTGGTAAT	3	<hkxhk>	hh	0	hk	273	kk	0
>ref-19971	ACCTCCAGGACTCCGTCTCCTGGTAAT	14	<hkxhk>	hh	0	hk	273	kk	0
>ref-19987	ATGTAGCCAACCCAAACTCCTAAAGCA	15	<hkxhk>	hh	45	hk	126	kk	66
>ref-19987	ATGTAGCCAACCCAAACTCCTAAAGCA	25	<nnxnp>	nn	259	np	0		
>ref-19987	ATGTAGCCAACCCAAACTCCTAAAGCA	26	<nnxnp>	nn	259	np	0		
>ref-19992	GTCTCTTTTACTCGCTCTCCCTTTCTC	1	<nnxnp>	nn	259	np	3		
>ref-19992	GTCTCTTTTACTCGCTCTCCCTTTCTC	23	<nnxnp>	nn	268	np	1		
>ref-19993	GGACAAACAACTCCCTCTCCCTCTCCC	1	<hkxhk>	hh	0	hk	273	kk	0
>ref-19993	GGACAAACAACTCCCTCTCCCTCTCCC	3	<hkxhk>	hh	0	hk	273	kk	0
>ref-20031	TTGTTAGTGACAGTTGCTCCTAGGTAA	15	<hkxhk>	hh	0	hk	260	kk	3
>ref-20031	TTGTTAGTGACAGTTGCTCCTAGGTAA	23	<hkxhk>	hh	0	hk	260	kk	3
>ref-20044	AGGAGTTGGACAAACACTCCTCATCCC	2	<lmxll>	ll	131	lm	139		
>ref-20048	ATTACATACACGGATCCTCCTCTGCTC	14	<nnxnp>	nn	124	np	145		
>ref-20050	ACCACCACCACCATTTCTCCTAATCCA	14	<nnxnp>	nn	115	np	148		
>ref-20050	ACCACCACCACCATTTCTCCTAATCCA	25	<nnxnp>	nn	244	np	18		
>ref-20050	ACCACCACCACCATTTCTCCTAATCCA	26	<hkxhk>	hh	166	hk	86	kk	0
>ref-20050	ACCACCACCACCATTTCTCCTAATCCA	27	<lmxll>	ll	239	lm	24		

>ref-20052	GCTTGGCAGACAGCGCCTCCAGATACA	7	<nnxnp>	nn	273	np	0		
>ref-20054	ACACTGACCACCTTAACTCCCCTCACA	26	<nnxnp>	nn	140	np	115		
>ref-20060	ACCCTTAAAACCTGGCCTCCCTCCCCT	16	<lmxll>	ll	133	lm	112		
>ref-20081	CCTGCTTCCACCATAACTCCTGCTTCT	26	<hkxhk>	hh	1	hk	122	kk	134
>ref-20081	CCTGCTTCCACCATAACTCCTGCTTCT	27	<hkxhk>	hh	134	hk	122	kk	1
>ref-20082	CACACAGGTACTCGTACTCCTAGGAAC	21	<hkxhk>	hh	44	hk	138	kk	68
>ref-20092	CAAACACGCACGTTTTCTCCTCTCTCT	26	<nnxnp>	nn	116	np	138		
>ref-20116	CACACACACACTTACCCTCCCAAGCGA	26	<nnxnp>	nn	139	np	124		
>ref-20129	GACACCCATACACACGCTCCCGTATAC	1	<nnxnp>	nn	132	np	136		
>ref-20129	GACACCCATACACACGCTCCCGTATAC	6	<nnxnp>	nn	132	np	136		
>ref-20132	ATTTTGAACACGCACCCTCCATTCTCG	26	<hkxhk>	hh	60	hk	145	kk	44
>ref-20132	ATTTTGAACACGCACCCTCCATTCTCG	27	<hkxhk>	hh	44	hk	145	kk	59
>ref-20138	AGAACTAGCACTGACTCTCCCCTACTA	12	<lmxll>	ll	115	lm	133		
>ref-20145	CATATCCACACCGTCTCTCCAACATCA	16	<nnxnp>	nn	144	np	125		
>ref-20180	AACATAGATACAGATTCTCCTCTTAAT	7	<lmxll>	ll	104	lm	121		
>ref-20194	CTACCCCTCACCCCACCTCCCTCCCTC	1	<hkxhk>	hh	0	hk	77	kk	176
>ref-20194	CTACCCCTCACCCCACCTCCCTCCCTC	3	<nnxnp>	nn	14	np	253		
>ref-20194	CTACCCCTCACCCCACCTCCCTCCCTC	8	<hkxhk>	hh	0	hk	79	kk	171
>ref-20203	CCTACCCCTACCCCTTCTCCCCCTTCT	15	<hkxhk>	hh	0	hk	238	kk	20
>ref-20207	TTCTGTTGAACTTGCACTCCGCATCAG	5	<hkxhk>	hh	60	hk	156	kk	51
>ref-20210	TGCCCTCTCACCCTCCCTCCTCTCCAT	2	<lmxll>	ll	221	lm	4		
>ref-20210	TGCCCTCTCACCCTCCCTCCTCTCCAT	26	<lmxll>	ll	233	lm	11		
>ref-20216	TGGTTCCCAACTCCTTCTCCTTTGATA	15	<lmxll>	ll	124	lm	127		
>ref-20228	TTCATTTCCACTCCCCCTCCTCTCCCT	1	<nnxnp>	nn	259	np	2		
>ref-20228	TTCATTTCCACTCCCCCTCCTCTCCCT	4	<nnxnp>	nn	258	np	1		
>ref-20228	TTCATTTCCACTCCCCCTCCTCTCCCT	5	<nnxnp>	nn	263	np	0		
>ref-20228	TTCATTTCCACTCCCCCTCCTCTCCCT	6	<nnxnp>	nn	261	np	1		
>ref-20228	TTCATTTCCACTCCCCCTCCTCTCCCT	14	<nnxnp>	nn	262	np	0		
>ref-20238	CCTTTCCTCACCTTCCCTCCTTGTTCC	23	<nnxnp>	nn	250	np	3		
>ref-20238	CCTTTCCTCACCTTCCCTCCTTGTTCC	27	<nnxnp>	nn	258	np	2		
>ref-20243	CATTCCTATACTACTACTCCTGCCTTA	4	<lmxll>	ll	111	lm	134		
>ref-20247	GCCTTCCCCACTCCGCCTCCCCTTCCC	23	<hkxhk>	hh	0	hk	139	kk	118
>ref-20267	AACAAGTCTACCTACACTCCTCGACAA	4	<hkxhk>	hh	112	hk	144	kk	0
>ref-20267	AACAAGTCTACCTACACTCCTCGACAA	6	<nnxnp>	nn	255	np	8		
>ref-20267	AACAAGTCTACCTACACTCCTCGACAA	8	<hkxhk>	hh	203	hk	54	kk	0
>ref-20267	AACAAGTCTACCTACACTCCTCGACAA	16	<nnxnp>	nn	254	np	5		
>ref-20267	AACAAGTCTACCTACACTCCTCGACAA	22	<lmxll>	ll	4	lm	256		
>ref-20267	AACAAGTCTACCTACACTCCTCGACAA	23	<hkxhk>	hh	0	hk	156	kk	94

>ref-20267	AACAAGTCTACCTACACTCCTCGACAA	24	<hkxhk>	hh	224	hk	33	kk	0
>ref-20289	TCGCTAGCCACACCCCCTCCGCCCACA	1	<nnxnp>	nn	121	np	142		
>ref-20298	TATACACGCACGAACACTCCCACACAA	23	<nnxnp>	nn	125	np	127		
>ref-20312	TGCGAGCGCACCGTGCCTCCAGCCGTG	1	<nnxnp>	nn	258	np	0		
>ref-20312	TGCGAGCGCACCGTGCCTCCAGCCGTG	8	<nnxnp>	nn	258	np	0		
>ref-20334	CCGATTCCCACTTACTCTCCTTGCCTC	8	<nnxnp>	nn	110	np	153		
>ref-20360	AAACTTTATACTCATACTCCCTGTTAA	7	<hkxhk>	hh	2	hk	267	kk	0
>ref-20363	CCCTTCCCCACTGCTCCTCCCTATTCC	5	<hkxhk>	hh	0	hk	210	kk	35
>ref-20363	CCCTTCCCCACTGCTCCTCCCTATTCC	16	<hkxhk>	hh	1	hk	258	kk	4
>ref-20363	CCCTTCCCCACTGCTCCTCCCTATTCC	23	<hkxhk>	hh	1	hk	259	kk	4
>ref-20364	TCCTACCATACTCTCCCTCCCTATAGT	1	<hkxhk>	hh	35	hk	141	kk	64
>ref-20385	GGTTGGCCTACACTCACTCCATCACCT	4	<hkxhk>	hh	51	hk	156	kk	60
>ref-20390	CACCCAAGCACCCTTACTCCCTCCCCC	23	<nnxnp>	nn	116	np	139		
>ref-20424	AGCAGAATGACCCCTCCTCCCCGTTTG	3	<hkxhk>	hh	261	hk	11	kk	0
>ref-20424	AGCAGAATGACCCCTCCTCCCCGTTTG	13	<lmxll>	ll	9	lm	260		
>ref-20424	AGCAGAATGACCCCTCCTCCCCGTTTG	16	<nnxnp>	nn	259	np	0		
>ref-20424	AGCAGAATGACCCCTCCTCCCCGTTTG	22	<hkxhk>	hh	75	hk	191	kk	0
>ref-20438	CATGCCTGCACTCCAACTCCTGGCTCG	1	<hkxhk>	hh	0	hk	273	kk	0
>ref-20438	CATGCCTGCACTCCAACTCCTGGCTCG	15	<hkxhk>	hh	0	hk	273	kk	0
>ref-20451	AGACGGGATACTACTCCTCCATTTCAC	5	<lmxll>	ll	119	lm	129		
>ref-20456	ACCACCACCACCATCCCTCCCTCAACC	21	<hkxhk>	hh	0	hk	210	kk	57
>ref-20456	ACCACCACCACCATCCCTCCCTCAACC	22	<hkxhk>	hh	0	hk	210	kk	55
>ref-20461	AACATGTAGACGTGATCTCCATATCGC	13	<nnxnp>	nn	129	np	137		
>ref-20461	AACATGTAGACGTGATCTCCATATCGC	16	<nnxnp>	nn	128	np	137		
>ref-20461	AACATGTAGACGTGATCTCCATATCGC	27	<lmxll>	ll	111	lm	155		
>ref-20472	TATTAATCCACTACTACTCCGCAGTAA	23	<lmxll>	ll	109	lm	134		
>ref-20474	TTATTTCCAACACACTCTCCTAGTGCT	7	<hkxhk>	hh	67	hk	142	kk	36
>ref-20497	CTAATCCGAACTTCCTCTCCTTTCCCG	23	<hkxhk>	hh	36	hk	139	kk	85
>ref-20531	TTAACTATTACATCTGCTCCTTCTCTC	4	<nnxnp>	nn	120	np	144		
>ref-20535	CTCTTCCTCACTCCCCCTCCCGTGTCC	6	<lmxll>	ll	135	lm	107		
>ref-20535	CTCTTCCTCACTCCCCCTCCCGTGTCC	22	<lmxll>	ll	134	lm	108		
>ref-20536	AACCCCCACACTCACCCTCCAACCCCA	4	<hkxhk>	hh	0	hk	271	kk	1
>ref-20536	AACCCCCACACTCACCCTCCAACCCCA	7	<nnxnp>	nn	42	np	218		
>ref-20536	AACCCCCACACTCACCCTCCAACCCCA	27	<hkxhk>	hh	0	hk	272	kk	0
>ref-20555	CTACTCCTTACCCTATCTCCCTCTTCC	1	<hkxhk>	hh	3	hk	257	kk	0
>ref-20555	CTACTCCTTACCCTATCTCCCTCTTCC	22	<hkxhk>	hh	0	hk	265	kk	0
>ref-20556	CTAAGCACAACTAACTCTCCGAGAATT	12	<hkxhk>	hh	1	hk	193	kk	56
>ref-20556	CTAAGCACAACTAACTCTCCGAGAATT	14	<hkxhk>	hh	54	hk	195	kk	1

>ref-20556	CTAAGCACAACTAACTCTCCGAGAATT	23	<lmxll>	ll	143	lm	104		
>ref-20557	TTCCTTTCCACCTCTCCTCCCCCCACC	4	<hkxhk>	hh	17	hk	242	kk	0
>ref-20557	TTCCTTTCCACCTCTCCTCCCCCCACC	21	<hkxhk>	hh	0	hk	243	kk	17
>ref-20557	TTCCTTTCCACCTCTCCTCCCCCCACC	24	<nnxnp>	nn	156	np	106		
>ref-20567	CTCCTTCCCACAGTCACTCCCACAGGC	5	<hkxhk>	hh	0	hk	246	kk	12
>ref-20567	CTCCTTCCCACAGTCACTCCCACAGGC	21	<hkxhk>	hh	15	hk	245	kk	0
>ref-20567	CTCCTTCCCACAGTCACTCCCACAGGC	23	<hkxhk>	hh	14	hk	245	kk	0
>ref-20590	ACAACACAGACATTTCCTCCTCGCCCT	9	<lmxll>	ll	140	lm	128		
>ref-20591	CCAACACAAACACCTCCTCCTTCCATT	8	<hkxhk>	hh	39	hk	134	kk	66
>ref-20594	CACTCTTCCACTTCTTCTCCCCCTTTC	15	<hkxhk>	hh	14	hk	244	kk	3
>ref-20594	CACTCTTCCACTTCTTCTCCCCCTTTC	24	<hkxhk>	hh	14	hk	244	kk	3
>ref-20602	ATAATTACCACCTGTACTCCAGTTGTG	6	<nnxnp>	nn	117	np	123		
>ref-20604	ACCTCCATCACCCCCTCTCCCCCGTCA	21	<hkxhk>	hh	56	hk	136	kk	64
>ref-20606	GTGGCTGGGACTGCTCCTCCCCCTCCT	9	<nnxnp>	nn	264	np	0		
>ref-20607	CCCCCTACCACTTCACCTCCCACCTCC	7	<lmxll>	ll	4	lm	263		
>ref-20607	CCCCCTACCACTTCACCTCCCACCTCC	13	<lmxll>	ll	4	lm	263		
>ref-20613	CTACCCCTTACCATATCTCCCACACAA	4	<nnxnp>	nn	98	np	153		
>ref-20618	CCTTCCCTTACTATCTCTCCCTTCCCT	13	<nnxnp>	nn	8	np	257		
>ref-20626	CTCTCCCTAACTCTCCCTCCATCTTCT	15	<lmxll>	ll	231	lm	1		
>ref-20627	CTTGTGTGTACTCGGCCTCCCTTTAGC	7	<lmxll>	ll	119	lm	134		
>ref-20635	CCTGCAAACACAGCATCTCCTTAGCGG	23	<lmxll>	ll	117	lm	126		
>ref-20637	TTCATTCGCACATCTACTCCTTCAGAA	2	<nnxnp>	nn	109	np	142		
>ref-20637	TTCATTCGCACATCTACTCCTTCAGAA	6	<lmxll>	ll	147	lm	111		
>ref-20647	TCACTATGGACTTACTCTCCATGCATC	9	<hkxhk>	hh	101	hk	138	kk	1
>ref-20647	TCACTATGGACTTACTCTCCATGCATC	26	<hkxhk>	hh	101	hk	138	kk	1
>ref-20652	CCACACTCCACACCTACTCCACACTCC	3	<hkxhk>	hh	21	hk	247	kk	0
>ref-20652	CCACACTCCACACCTACTCCACACTCC	6	<hkxhk>	hh	20	hk	248	kk	0
>ref-20652	CCACACTCCACACCTACTCCACACTCC	16	<hkxhk>	hh	47	hk	218	kk	0
>ref-20652	CCACACTCCACACCTACTCCACACTCC	24	<hkxhk>	hh	28	hk	241	kk	0
>ref-20668	CATACATCCACACTCCCTCCTCTCCCT	5	<nnxnp>	nn	127	np	129		
>ref-20678	TGGTTTAGTACTGGCCCTCCGGGTGAC	15	<lmxll>	ll	3	lm	220		
>ref-20688	CACCCCATAACCCCCTCTCCTACTCAT	14	<lmxll>	ll	148	lm	115		
>ref-20689	ATGATCATTACTTCCTCTCCATAGTAT	8	<lmxll>	ll	113	lm	113		
>ref-20694	GCTCTGGGAACCCTGGCTCCTCCTCCT	14	<lmxll>	ll	107	lm	149		
>ref-20700	TAAACCACGACCTCCTCTCCCCTATCC	22	<lmxll>	ll	126	lm	141		
>ref-20705	ATATACCACACTAACGCTCCTTCCCCC	16	<lmxll>	ll	139	lm	119		
>ref-20712	CACAATGCCACACTCTCTCCGCCTCTC	9	<nnxnp>	nn	131	np	139		
>ref-20731	CACGTGATCACTTCATCTCCTACCCGC	21	<lmxll>	ll	132	lm	104		

>ref-20750	TCCTTTTGAACACCCCCTCCTCCTCCC	13	<nnxnp>	nn	109	np	130		
>ref-20753	ACCCATCTTACGCCCACTCCTTCTCTC	22	<nnxnp>	nn	268	np	0		
>ref-20761	TTCCTCCTTACAGCCCCTCCCACCACT	1	<lmxll>	ll	145	lm	106		
>ref-20778	TCTATAAATACTTTATCTCCCTCTACT	14	<lmxll>	ll	115	lm	123		
>ref-20795	TGGAAGTCGACATTGACTCCTCCTTTA	24	<nnxnp>	nn	126	np	133		
>ref-20798	CCACTCTCCACCACCTCTCCACCAGCT	25	<nnxnp>	nn	125	np	102		
>ref-20819	AGATAACAAACTTATCCTCCCTTCCAT	7	<nnxnp>	nn	141	np	109		
>ref-20828	ATTTATTTCACGTCAACTCCGTTATTA	6	<nnxnp>	nn	108	np	125		
>ref-20856	CCCCATTACACCGCAACTCCCATACTC	4	<lmxll>	ll	126	lm	107		
>ref-20885	TTATATCTTACCTTCTCTCCCTCTCTA	6	<lmxll>	ll	94	lm	159		
>ref-20885	TTATATCTTACCTTCTCTCCCTCTCTA	24	<lmxll>	ll	94	lm	158		
>ref-20890	CATACACGCACAAAGACTCCTAAGTAA	3	<lmxll>	ll	127	lm	127		
>ref-20930	TCGTTGCACACGCCCACTCCATGTTTG	2	<lmxll>	ll	2	lm	267		
>ref-20935	GTACACCAGACTGCCTCTCCTGCTCTC	1	<lmxll>	ll	139	lm	131		
>ref-20935	GTACACCAGACTGCCTCTCCTGCTCTC	25	<nnxnp>	nn	123	np	148		
>ref-20945	CCGTCGGCCACGCCCACTCCACCGGCT	23	<nnxnp>	nn	127	np	136		
>ref-20956	CTGAACCTTACTGAACCTCCTCGGGCT	27	<nnxnp>	nn	116	np	143		
>ref-20980	TGGACGGCAACGGCTTCTCCCCAATCA	3	<nnxnp>	nn	271	np	0		
>ref-20983	CTGGAGGACACCCACCCTCCTGCCCTG	24	<nnxnp>	nn	118	np	140		
>ref-20992	TCGCGTCCTACTCTTCCTCCTCTTTCT	5	<lmxll>	ll	130	lm	112		
>ref-20992	TCGCGTCCTACTCTTCCTCCTCTTTCT	22	<lmxll>	ll	142	lm	105		
>ref-20999	TCACCACACACTGTACCTCCCTTGTTA	12	<nnxnp>	nn	141	np	116		
>ref-21010	ATTCCCCTCACATCTCCTCCTCCCTCA	6	<nnxnp>	nn	134	np	128		
>ref-21038	GGGCTTAGGACGCCGTCTCCGGGGGGA	26	<nnxnp>	nn	138	np	132		
>ref-21078	CAAACACAGACAAACACTCCTTATCAT	21	<nnxnp>	nn	140	np	129		
>ref-21086	GCCCACACGACACTGTCTCCCAGAGGA	13	<lmxll>	ll	131	lm	141		
>ref-21116	CCCGCGAAGACCTCCTCTCCAGTGCAC	8	<lmxll>	ll	146	lm	123		
>ref-21116	CCCGCGAAGACCTCCTCTCCAGTGCAC	27	<lmxll>	ll	146	lm	123		
>ref-21125	TTTGGCTAAACCCCCTCTCCGGCTCTA	24	<hkxhk>	hh	1	hk	195	kk	62
>ref-21125	TTTGGCTAAACCCCCTCTCCGGCTCTA	25	<lmxll>	ll	151	lm	109		
>ref-21126	GACCCCATAACCTTCTCTCCTCGCTTC	1	<nnxnp>	nn	157	np	107		
>ref-21126	GACCCCATAACCTTCTCTCCTCGCTTC	5	<lmxll>	ll	123	lm	140		
>ref-21126	GACCCCATAACCTTCTCTCCTCGCTTC	24	<nnxnp>	nn	157	np	107		
>ref-21132	CAGGTCTCCACCTTCACTCCTCATCAA	21	<hkxhk>	hh	58	hk	146	kk	44
>ref-21139	AGGTGCGGCACACCTTCTCCTCCTCCT	16	<hkxhk>	hh	61	hk	156	kk	51
>ref-21169	CTCCATGCTACTCCTCCTCCTTTGGCC	1	<lmxll>	ll	120	lm	124		
>ref-21173	CGTGATGTCACGGTCGCTCCTCCTCCC	21	<lmxll>	ll	115	lm	150		
>ref-21179	TCATTCCAAACCTCCGCTCCCGCCGAA	16	<hkxhk>	hh	66	hk	116	kk	42

>ref-21199	CCCTTCTCTACCTTTTCTCCTTCCCAC	7	<hkxhk>	hh	5	hk	262	kk	1
>ref-21199	CCCTTCTCTACCTTTTCTCCTTCCCAC	13	<hkxhk>	hh	5	hk	262	kk	1
>ref-21213	CACGCTAACACGCTAACTCCATCTATC	2	<hkxhk>	hh	56	hk	140	kk	63
>ref-21228	CATTCACCCACACCCTCTCCTCACCCC	14	<lmxll>	ll	137	lm	113		
>ref-21228	CATTCACCCACACCCTCTCCTCACCCC	15	<lmxll>	ll	137	lm	113		
>ref-21259	AACAGCATTACACTTCCTCCTCACGTT	22	<hkxhk>	hh	68	hk	149	kk	46
>ref-21287	TTAGTTACCACATTTGCTCCAGCATGA	21	<nnxnp>	nn	253	np	0		
>ref-21299	GACCGTTGTACCGCGTCTCCAACACGC	4	<nnxnp>	nn	129	np	127		
>ref-21299	GACCGTTGTACCGCGTCTCCAACACGC	5	<lmxll>	ll	129	lm	129		
>ref-21299	GACCGTTGTACCGCGTCTCCAACACGC	9	<lmxll>	ll	130	lm	128		
>ref-21302	AGTTTGTCAACCTTATCTCCTGACTCT	24	<nnxnp>	nn	132	np	119		
>ref-21319	GAATCACTTACATCCACTCCTACGGCT	12	<hkxhk>	hh	6	hk	240	kk	1
>ref-21322	CTCCTTCCGACTGATCCTCCTTTCATG	2	<lmxll>	ll	127	lm	137		
>ref-21325	GGGAACCTAACTTTGTCTCCTCTGTAC	21	<nnxnp>	nn	137	np	124		
>ref-21329	TCCTCCCCTACTGTCTCTCCCCTCCTT	1	<nnxnp>	nn	144	np	129		
>ref-21329	TCCTCCCCTACTGTCTCTCCCCTCCTT	15	<nnxnp>	nn	145	np	128		
>ref-21332	CCTTTTCCTACTCACCCTCCTTCTCTA	2	<lmxll>	ll	121	lm	134		
>ref-21335	CCCAAGCCAACCCTCCCTCCGTAACCT	21	<nnxnp>	nn	262	np	0		
>ref-21336	CGCACTCTCACCCTCCCTCCACCTACC	3	<lmxll>	ll	132	lm	131		
>ref-21339	TGCCAGCCTACCACACCTCCTTTTCTC	23	<hkxhk>	hh	74	hk	130	kk	44
>ref-21341	CTTTCTCCCACCATCCCTCCCTCACCT	26	<hkxhk>	hh	6	hk	263	kk	0
>ref-21370	CCCCCCTCGACCTTCCCTCCCCATCCT	9	<hkxhk>	hh	0	hk	270	kk	0
>ref-21370	CCCCCCTCGACCTTCCCTCCCCATCCT	14	<hkxhk>	hh	0	hk	271	kk	0
>ref-21399	ATAATGTCCACATTATCTCCACGCATA	24	<lmxll>	ll	112	lm	112		
>ref-21417	CAAACAACAACCTCACCTCCTCATTCA	6	<lmxll>	ll	152	lm	115		
>ref-21497	GCTGTTCCTACGCGCCCTCCGCATCTG	9	<hkxhk>	hh	0	hk	270	kk	0
>ref-21497	GCTGTTCCTACGCGCCCTCCGCATCTG	15	<hkxhk>	hh	2	hk	271	kk	0
>ref-21497	GCTGTTCCTACGCGCCCTCCGCATCTG	22	<hkxhk>	hh	41	hk	226	kk	0
>ref-21512	CCTGCACCTACAACAGCTCCAACACCT	9	<hkxhk>	hh	0	hk	273	kk	0
>ref-21512	CCTGCACCTACAACAGCTCCAACACCT	27	<nnxnp>	nn	271	np	0		
>ref-21539	TCCTGTTTAACCCTTGCTCCTATTATC	21	<hkxhk>	hh	61	hk	136	kk	51
>ref-21543	CAACGAGGCACTTCCTCTCCCCTTCAA	22	<lmxll>	ll	127	lm	144		
>ref-21590	TATCTGCCTACCTTTCCTCCTACTTAT	14	<hkxhk>	hh	0	hk	239	kk	18
>ref-21590	TATCTGCCTACCTTTCCTCCTACTTAT	24	<hkxhk>	hh	0	hk	268	kk	0
>ref-21590	TATCTGCCTACCTTTCCTCCTACTTAT	26	<hkxhk>	hh	0	hk	269	kk	0
>ref-21596	TTCGACGTCACGCTTGCTCCCTTGATA	3	<lmxll>	ll	121	lm	143		
>ref-21597	GGATTGTTTACCCTTGCTCCATCCCTC	9	<hkxhk>	hh	1	hk	212	kk	55
>ref-21610	AAAAGGATTACGTAATCTCCTATTCCG	7	<lmxll>	ll	112	lm	127		

>ref-21611	CGACGGTCCACACTAGCTCCTCGGGCA	14	<nnxnp>	nn	106	np	160		
>ref-21615	CTCCTTTTCACCCTACCTCCTCTTCTC	5	<hkxhk>	hh	0	hk	251	kk	13
>ref-21615	CTCCTTTTCACCCTACCTCCTCTTCTC	13	<hkxhk>	hh	5	hk	259	kk	0
>ref-21615	CTCCTTTTCACCCTACCTCCTCTTCTC	14	<hkxhk>	hh	0	hk	270	kk	0
>ref-21626	CCCGTAAGAACCCTCTCTCCCCCCTTT	1	<nnxnp>	nn	99	np	144		
>ref-21635	CGGTCGTGTACAGGGACTCCAGGAGCA	12	<lmxll>	ll	128	lm	124		
>ref-21652	GGAAGGACGACTGCGCCTCCCCCCTCA	27	<hkxhk>	hh	4	hk	174	kk	68
>ref-21653	CTATATGAAACAATTGCTCCTTTATCA	1	<hkxhk>	hh	46	hk	197	kk	0
>ref-21653	CTATATGAAACAATTGCTCCTTTATCA	7	<lmxll>	ll	227	lm	27		
>ref-21653	CTATATGAAACAATTGCTCCTTTATCA	15	<hkxhk>	hh	0	hk	128	kk	99
>ref-21653	CTATATGAAACAATTGCTCCTTTATCA	16	<lmxll>	ll	47	lm	191		
>ref-21653	CTATATGAAACAATTGCTCCTTTATCA	26	<hkxhk>	hh	23	hk	229	kk	0
>ref-21665	TTGTACATAACCTTTCCTCCTCATGTT	1	<nnxnp>	nn	258	np	0		
>ref-21666	TCATGTCCTACCGCTCCTCCTTGAGTT	16	<nnxnp>	nn	112	np	141		
>ref-21673	TAATTTCCGACGCGCTCTCCACCAGCA	27	<lmxll>	ll	133	lm	114		
>ref-21697	TGTTTATCCACCCTTCCTCCTTTCCAT	13	<hkxhk>	hh	66	hk	130	kk	62
>ref-21697	TGTTTATCCACCCTTCCTCCTTTCCAT	16	<nnxnp>	nn	125	np	141		
>ref-21705	ACAACAACAACTTTTACTCCCAACGAA	23	<nnxnp>	nn	103	np	134		
>ref-21710	TCATACCCTACCCCTTCTCCCAGTCCT	22	<hkxhk>	hh	37	hk	150	kk	59
>ref-21714	TTCTCACATACATCTTCTCCAGCGCCT	12	<lmxll>	ll	131	lm	113		
>ref-21734	ATTCCCTCCACCTTCCCTCCTACCTTA	9	<nnxnp>	nn	117	np	148		
>ref-21781	GACTGGCAGACTATCTCTCCGGTTGTC	14	<nnxnp>	nn	136	np	130		
>ref-21781	GACTGGCAGACTATCTCTCCGGTTGTC	16	<nnxnp>	nn	130	np	136		
>ref-21785	CCGCTCCCCACTCCCCCTCCCCTTCTC	2	<nnxnp>	nn	28	np	209		
>ref-21785	CCGCTCCCCACTCCCCCTCCCCTTCTC	4	<lmxll>	ll	117	lm	108		
>ref-21785	CCGCTCCCCACTCCCCCTCCCCTTCTC	8	<lmxll>	ll	263	lm	0		
>ref-21785	CCGCTCCCCACTCCCCCTCCCCTTCTC	16	<lmxll>	ll	258	lm	0		
>ref-21785	CCGCTCCCCACTCCCCCTCCCCTTCTC	24	<lmxll>	ll	28	lm	216		
>ref-21788	ACCTCTCTAACTTAATCTCCTCTCTCT	24	<hkxhk>	hh	61	hk	138	kk	50
>ref-21801	CTCCACCTTACCCTCCCTCCCCCTTCC	1	<nnxnp>	nn	162	np	99		
>ref-21801	CTCCACCTTACCCTCCCTCCCCCTTCC	5	<hkxhk>	hh	8	hk	231	kk	0
>ref-21801	CTCCACCTTACCCTCCCTCCCCCTTCC	14	<lmxll>	ll	85	lm	168		
>ref-21801	CTCCACCTTACCCTCCCTCCCCCTTCC	24	<nnxnp>	nn	129	np	121		
>ref-21830	GCGTCCCAGACCCGCCCTCCGCACGCA	4	<hkxhk>	hh	47	hk	144	kk	71
>ref-21832	TACACAAACACACTTGCTCCTACGGTT	23	<lmxll>	ll	119	lm	146		
>ref-21832	TACACAAACACACTTGCTCCTACGGTT	24	<hkxhk>	hh	63	hk	137	kk	62
>ref-21853	CCTGCTATTACCGCTGCTCCAGACAAG	9	<nnxnp>	nn	264	np	0		
>ref-21865	TTTATTTTCACACTTGCTCCTTCATTC	4	<hkxhk>	hh	58	hk	188	kk	1

>ref-21865	TTTATTTTCACACTTGCTCCTTCATTC	12	<hkxhk>	hh	58	hk	188	kk	1
>ref-21871	ATTCACTTAACCTGTACTCCTGCTTGG	5	<nnxnp>	nn	113	np	133		
>ref-21880	CTTCCGGACACTCCTCCTCCTTCTCCT	7	<lmxll>	ll	259	lm	0		
>ref-21892	CCTCCCCTGACCTGTCCTCCTCCTTCA	9	<lmxll>	ll	125	lm	123		
>ref-21894	ACCCTTTATACCCACACTCCCTCATCC	8	<lmxll>	ll	125	lm	123		
>ref-21896	TCCCGACCCACCCCCTCTCCACGCCCA	15	<hkxhk>	hh	3	hk	269	kk	0
>ref-21896	TCCCGACCCACCCCCTCTCCACGCCCA	21	<hkxhk>	hh	5	hk	267	kk	0
>ref-21896	TCCCGACCCACCCCCTCTCCACGCCCA	23	<nnxnp>	nn	200	np	65		
>ref-21896	TCCCGACCCACCCCCTCTCCACGCCCA	24	<lmxll>	ll	145	lm	127		
>ref-21896	TCCCGACCCACCCCCTCTCCACGCCCA	27	<hkxhk>	hh	4	hk	268	kk	0
>ref-21911	TGTTATTATACATTACCTCCGAAAGCT	15	<nnxnp>	nn	117	np	104		
>ref-21915	ATGTTTCTTACAGTAACTCCACTGTCA	27	<nnxnp>	nn	104	np	149		
>ref-21920	CGCTGTGGCACAGGGCCTCCGAGTGCC	22	<hkxhk>	hh	0	hk	273	kk	0
>ref-21931	TCTAGATCGACCTTCTCTCCCGCGTTT	3	<hkxhk>	hh	54	hk	139	kk	49
>ref-21937	CCTTAGACGACTGGGCCTCCCTACTCA	4	<nnxnp>	nn	259	np	0		
>ref-21937	CCTTAGACGACTGGGCCTCCCTACTCA	25	<nnxnp>	nn	259	np	1		
>ref-21942	TATTCCTCTACCACCCCTCCCTCTCCC	1	<nnxnp>	nn	103	np	150		
>ref-21984	ATTACTACTACTCATCCTCCTTCTTCT	3	<lmxll>	ll	140	lm	115		
>ref-21992	CGAAGGCAAACCGCACCTCCATCTCTC	26	<hkxhk>	hh	53	hk	128	kk	53
>ref-22014	TTTTTCCTCACAGTTGCTCCAGTATCT	9	<lmxll>	ll	18	lm	227		
>ref-22014	TTTTTCCTCACAGTTGCTCCAGTATCT	16	<lmxll>	ll	18	lm	226		
>ref-22063	TGCAGAACTACTCTCCCTCCCTCTTCC	25	<nnxnp>	nn	126	np	128		
>ref-22079	GTCACTTCTACCCTTCCTCCTCCTCCT	2	<hkxhk>	hh	0	hk	65	kk	188
>ref-22079	GTCACTTCTACCCTTCCTCCTCCTCCT	3	<hkxhk>	hh	0	hk	67	kk	190
>ref-22079	GTCACTTCTACCCTTCCTCCTCCTCCT	4	<hkxhk>	hh	0	hk	269	kk	3
>ref-22079	GTCACTTCTACCCTTCCTCCTCCTCCT	23	<hkxhk>	hh	0	hk	270	kk	2
>ref-22111	GAGACTAAGACAATGCCTCCCACTCTA	6	<lmxll>	ll	97	lm	130		
>ref-22131	CACACACGGACATACACTCCCAGGAAG	1	<nnxnp>	nn	114	np	107		
>ref-22140	GTGGGTGTCACATTCCCTCCTTCCCCT	22	<lmxll>	ll	143	lm	119		
>ref-22182	GTAAATGCAACACAAACTCCAAGGATA	21	<lmxll>	ll	128	lm	109		
>ref-22203	GTGCGGGGGACCCATTCTCCCTTGGAG	21	<hkxhk>	hh	8	hk	230	kk	0
>ref-22231	TAACCCCTCACCCTGCCTCCTCCCTCT	1	<hkxhk>	hh	0	hk	254	kk	9
>ref-22231	TAACCCCTCACCCTGCCTCCTCCCTCT	7	<hkxhk>	hh	9	hk	255	kk	0
>ref-22234	ACATCCCATACTGTACCTCCTCCCCAC	22	<lmxll>	ll	120	lm	125		
>ref-22256	TCCTCCTCCACTCCTCCTCCCCTTTAT	21	<nnxnp>	nn	219	np	10		
>ref-22257	CATTTGTTTACACTTCCTCCCTAGGTT	3	<nnxnp>	nn	101	np	145		
>ref-22259	CATCACTCGACGTCAGCTCCGTGGTGG	9	<nnxnp>	nn	156	np	115		
>ref-22267	CTCTCTTATACTACCTCTCCTGACATG	12	<lmxll>	ll	121	lm	136		

>ref-22314	CAGAAGCGAACACCCGCTCCCATCCTC	13	<nnxnp>	nn	122	np	148		
>ref-22337	CTCCATGCCACCCTCTCTCCCGCTTGG	1	<nnxnp>	nn	271	np	0		
>ref-22370	TCTCCGATGACTCACCCTCCCTCCTGT	2	<hkxhk>	hh	211	hk	11	kk	0
>ref-22385	GTCGCCCTAACTCCTTCTCCTGCCCTT	4	<nnxnp>	nn	143	np	128		
>ref-22385	GTCGCCCTAACTCCTTCTCCTGCCCTT	14	<lmxll>	ll	123	lm	147		
>ref-22388	CTATTCTCTACAGGCTCTCCCAATCTC	26	<hkxhk>	hh	53	hk	129	kk	46
>ref-22393	CAACATATCACATAGCCTCCCACCCTT	13	<nnxnp>	nn	123	np	133		
>ref-22396	ACTACCGTTACTACACCTCCTCCCCCT	2	<lmxll>	ll	240	lm	1		
>ref-22396	ACTACCGTTACTACACCTCCTCCCCCT	7	<nnxnp>	nn	116	np	115		
>ref-22407	CCCGCTTAGACGCTCCCTCCAGGGCCC	3	<nnxnp>	nn	129	np	136		
>ref-22408	CTTTTCCCTACTCCTCCTCCATCTTTA	2	<nnxnp>	nn	139	np	108		
>ref-22408	CTTTTCCCTACTCCTCCTCCATCTTTA	25	<nnxnp>	nn	140	np	107		
>ref-22426	ATGACACTCACCTGAACTCCGTAAGAC	22	<nnxnp>	nn	131	np	110		
>ref-22438	CAAGGTTTTACTCTCACTCCCTACTCT	2	<hkxhk>	hh	12	hk	247	kk	0
>ref-22438	CAAGGTTTTACTCTCACTCCCTACTCT	9	<hkxhk>	hh	0	hk	247	kk	12
>ref-22440	CAACGAGGTACTAGACCTCCTGGAAAG	23	<nnxnp>	nn	101	np	142		
>ref-22441	CCCCCCACCACACCCCCTCCCCTTCCT	5	<hkxhk>	hh	2	hk	228	kk	14
>ref-22441	CCCCCCACCACACCCCCTCCCCTTCCT	16	<hkxhk>	hh	2	hk	228	kk	14
>ref-22463	CTACAGTAGACATGATCTCCCTTCACC	1	<hkxhk>	hh	0	hk	273	kk	0
>ref-22463	CTACAGTAGACATGATCTCCCTTCACC	2	<hkxhk>	hh	0	hk	254	kk	11
>ref-22463	CTACAGTAGACATGATCTCCCTTCACC	9	<hkxhk>	hh	11	hk	255	kk	0
>ref-22463	CTACAGTAGACATGATCTCCCTTCACC	14	<hkxhk>	hh	49	hk	212	kk	0
>ref-22463	CTACAGTAGACATGATCTCCCTTCACC	15	<hkxhk>	hh	50	hk	213	kk	0
>ref-22463	CTACAGTAGACATGATCTCCCTTCACC	25	<hkxhk>	hh	3	hk	268	kk	0
>ref-22477	TTAGATTTCACGAAAACTCCTTAGATT	1	<lmxll>	ll	204	lm	40		
>ref-22498	CATCGCCTCACCTCTCCTCCAGTGCTC	14	<lmxll>	ll	106	lm	124		
>ref-22502	ACCACATCCACACGCTCTCCCAAATAA	2	<lmxll>	ll	235	lm	28		
>ref-22510	CGCCCACACACATTTCCTCCAGACTAC	14	<lmxll>	ll	108	lm	137		
>ref-22516	TCATTGTCTACCCCCACTCCCCTTATT	14	<hkxhk>	hh	3	hk	269	kk	0
>ref-22516	TCATTGTCTACCCCCACTCCCCTTATT	16	<nnxnp>	nn	83	np	168		
>ref-22516	TCATTGTCTACCCCCACTCCCCTTATT	22	<hkxhk>	hh	0	hk	267	kk	3
>ref-22543	GCCACCCTTACCCCTCCTCCCCCTCCC	4	<lmxll>	ll	7	lm	247		
>ref-22543	GCCACCCTTACCCCTCCTCCCCCTCCC	26	<lmxll>	ll	8	lm	248		
>ref-22546	TGTCATCTAACTATCACTCCTATCACT	9	<hkxhk>	hh	49	hk	137	kk	57
>ref-22546	TGTCATCTAACTATCACTCCTATCACT	16	<nnxnp>	nn	111	np	127		
>ref-22578	TTCATTACTACATTCACTCCCTCTATT	13	<lmxll>	ll	152	lm	110		
>ref-22583	GGCAGAGCGACAAGTTCTCCCAATCAA	9	<lmxll>	ll	115	lm	136		
>ref-22591	ACTCACAAAACCCAGTCTCCTTCTCCT	24	<nnxnp>	nn	138	np	116		

>ref-22616	AAGAAAGAGACATCTCCTCCTTCCTAT	21	<lmxll>	ll	128	lm	125		
>ref-22619	CTGTCACCAACCTATCCTCCCTGTCCT	3	<nnxnp>	nn	66	np	198		
>ref-22619	CTGTCACCAACCTATCCTCCCTGTCCT	14	<hkxhk>	hh	1	hk	272	kk	0
>ref-22619	CTGTCACCAACCTATCCTCCCTGTCCT	26	<hkxhk>	hh	17	hk	244	kk	0
>ref-22619	CTGTCACCAACCTATCCTCCCTGTCCT	27	<hkxhk>	hh	0	hk	273	kk	0
>ref-22662	CTCCCATCCACTCGCCCTCCCTCCTAC	15	<nnxnp>	nn	113	np	117		
>ref-22662	CTCCCATCCACTCGCCCTCCCTCCTAC	25	<nnxnp>	nn	110	np	118		
>ref-22666	TACACCACCACCCCACCTCCCACAGAT	2	<lmxll>	ll	62	lm	198		
>ref-22666	TACACCACCACCCCACCTCCCACAGAT	6	<lmxll>	ll	4	lm	266		
>ref-22666	TACACCACCACCCCACCTCCCACAGAT	22	<lmxll>	ll	63	lm	199		
>ref-22673	TCATTTTCCACTCCTCCTCCGGCTCTC	27	<nnxnp>	nn	122	np	134		
>ref-22676	GCCAGCTGTACAATAACTCCACTCAAA	9	<nnxnp>	nn	269	np	0		
>ref-22680	ACTTTCGAGACAGTTCCTCCCTCTCGG	26	<nnxnp>	nn	110	np	152		
>ref-22689	ATGACTAGTACATGACCTCCACCTTTG	25	<lmxll>	ll	126	lm	112		
>ref-22690	ACAACGGGAACACGCCCTCCCAACCAA	21	<nnxnp>	nn	114	np	152		
>ref-22690	ACAACGGGAACACGCCCTCCCAACCAA	22	<hkxhk>	hh	54	hk	147	kk	62
>ref-22713	TGGGTGCTGACCACAACTCCTGGCCTA	2	<lmxll>	ll	1	lm	265		
>ref-22713	TGGGTGCTGACCACAACTCCTGGCCTA	9	<hkxhk>	hh	0	hk	262	kk	5
>ref-22736	CTGACCGCTACCTTCACTCCTTCTCTG	26	<hkxhk>	hh	39	hk	151	kk	69
>ref-22761	GTGTTGATCACTGTAGCTCCGTTTGTC	15	<hkxhk>	hh	67	hk	148	kk	52
>ref-22762	GTGACACGCACCGACCCTCCTCAGGGA	2	<lmxll>	ll	122	lm	144		
>ref-22769	TAACAAAGCACAGGATCTCCATCAGGC	23	<nnxnp>	nn	141	np	126		
>ref-22777	ACATTCCTAACTGCTTCTCCTCTGCAT	7	<nnxnp>	nn	132	np	133		
>ref-22777	ACATTCCTAACTGCTTCTCCTCTGCAT	9	<nnxnp>	nn	130	np	136		
>ref-22786	CTATTCCTTACTTATCCTCCTATCACT	22	<nnxnp>	nn	103	np	124		
>ref-22787	ACTACCACGACTTCAGCTCCTCAGACA	5	<hkxhk>	hh	8	hk	259	kk	0
>ref-22787	ACTACCACGACTTCAGCTCCTCAGACA	12	<hkxhk>	hh	0	hk	268	kk	2
>ref-22808	AACCCACTTACCCACTCTCCCAACCCA	13	<nnxnp>	nn	127	np	132		
>ref-22808	AACCCACTTACCCACTCTCCCAACCCA	16	<nnxnp>	nn	124	np	133		
>ref-22827	AGAACAATCACAATCACTCCCGAGATA	12	<lmxll>	ll	131	lm	132		
>ref-22829	CTCTCTTTCACTATGTCTCCGGCTCTC	21	<nnxnp>	nn	260	np	0		
>ref-22829	CTCTCTTTCACTATGTCTCCGGCTCTC	22	<nnxnp>	nn	260	np	0		
>ref-22833	CCCAAAGTAACTGCCACTCCAGCCCCT	27	<hkxhk>	hh	61	hk	118	kk	67
>ref-22836	AACATGCACACTCGCGCTCCATGCATG	1	<nnxnp>	nn	120	np	146		
>ref-22838	ACCATCCACACAATACCTCCTTCCATC	3	<lmxll>	ll	132	lm	116		
>ref-22838	ACCATCCACACAATACCTCCTTCCATC	24	<lmxll>	ll	132	lm	116		
>ref-22863	TTCCGCGCTACATCTCCTCCTCCTCCT	4	<hkxhk>	hh	39	hk	212	kk	0
>ref-22863	TTCCGCGCTACATCTCCTCCTCCTCCT	6	<hkxhk>	hh	39	hk	212	kk	0

>ref-22885	ACCTCCTTCACCTCCTCTCCATCCATT	15	<nnxnp>	nn	119	np	149		
>ref-22922	ACTTCCTGGACGAAGCCTCCTTCTAAT	13	<nnxnp>	nn	104	np	135		
>ref-22923	CCCTCTTCCACGTCATCTCCGTTTTCT	21	<nnxnp>	nn	114	np	137		
>ref-22941	TTTGCATATACGCTAGCTCCTCAAAAT	2	<hkxhk>	hh	37	hk	119	kk	69
>ref-22941	TTTGCATATACGCTAGCTCCTCAAAAT	22	<hkxhk>	hh	38	hk	121	kk	69
>ref-22964	AGAAGTCATACATATTCTCCATATGTA	7	<lmxll>	ll	112	lm	114		
>ref-22993	GATTCCACCACCTCTTCTCCACCCACT	26	<nnxnp>	nn	249	np	0		
>ref-22993	GATTCCACCACCTCTTCTCCACCCACT	27	<nnxnp>	nn	248	np	0		
>ref-23014	CCCCTTCCAACGCCCACTCCCGCTCCC	5	<lmxll>	ll	203	lm	18		
>ref-23021	CTACCTTCTACCTGCTCTCCTGCTCCC	3	<lmxll>	ll	127	lm	144		
>ref-23025	TTCTCCTTCACTTTTTCTCCGTTATTT	3	<lmxll>	ll	154	lm	65		
>ref-23025	TTCTCCTTCACTTTTTCTCCGTTATTT	6	<lmxll>	ll	118	lm	110		
>ref-23025	TTCTCCTTCACTTTTTCTCCGTTATTT	15	<lmxll>	ll	120	lm	110		
>ref-23035	ATTTCTTAGACCATACCTCCTCTGAAT	16	<hkxhk>	hh	48	hk	143	kk	39
>ref-23068	CACGCACACACTACCCCTCCTTCTCTC	24	<nnxnp>	nn	141	np	127		
>ref-23118	GCCCCGTCTACCCCCTCTCCTACCCCA	3	<lmxll>	ll	119	lm	135		
>ref-23118	GCCCCGTCTACCCCCTCTCCTACCCCA	5	<lmxll>	ll	123	lm	133		
>ref-23118	GCCCCGTCTACCCCCTCTCCTACCCCA	7	<lmxll>	ll	122	lm	133		
>ref-23119	CGCACGGTCACACGGGCTCCTCGCGGC	24	<nnxnp>	nn	142	np	106		
>ref-23128	CTTCGTCTGACTCACTCTCCCTGTCTC	13	<nnxnp>	nn	129	np	139		
>ref-23134	TGAGTATCCACCCCCCCTCCTACCTCA	21	<lmxll>	ll	127	lm	131		
>ref-23136	TTCTATGCAACTCGTCCTCCTCCATTT	6	<hkxhk>	hh	21	hk	187	kk	52
>ref-23140	TACCATCCTACTGATCCTCCTCCTCCT	26	<lmxll>	ll	144	lm	126		
>ref-23142	CAACAGCTCACACGGGCTCCTGGCAAG	8	<hkxhk>	hh	0	hk	272	kk	0
>ref-23142	CAACAGCTCACACGGGCTCCTGGCAAG	23	<hkxhk>	hh	0	hk	272	kk	0
>ref-23150	AACCATTACACGTATTCTCCCTTCTGT	23	<lmxll>	ll	115	lm	114		
>ref-23167	GTAACCATGACCCTCCCTCCAGCCCAG	6	<lmxll>	ll	110	lm	126		
>ref-23179	GCAGCATCGACAACGGCTCCAGCACTA	14	<lmxll>	ll	110	lm	151		
>ref-23188	GAAACAGGAACAATATCTCCACTAATA	4	<hkxhk>	hh	15	hk	212	kk	2
>ref-23188	GAAACAGGAACAATATCTCCACTAATA	22	<hkxhk>	hh	15	hk	212	kk	2
>ref-23199	CTCTTTCCCACACTGTCTCCCGCGTAG	25	<lmxll>	ll	123	lm	140		
>ref-23210	TTTTCTCTCACCCCCCCTCCCCTCCTC	3	<lmxll>	ll	180	lm	41		
>ref-23210	TTTTCTCTCACCCCCCCTCCCCTCCTC	8	<hkxhk>	hh	5	hk	226	kk	3
>ref-23210	TTTTCTCTCACCCCCCCTCCCCTCCTC	12	<lmxll>	ll	113	lm	120		
>ref-23210	TTTTCTCTCACCCCCCCTCCCCTCCTC	21	<hkxhk>	hh	244	hk	1	kk	0
>ref-23220	AATAGAGCAACGTCACCTCCGGAAGGC	27	<nnxnp>	nn	249	np	0		
>ref-23228	GGGCTACTCACGTCCTCTCCCCCTTTT	1	<lmxll>	ll	123	lm	137		
>ref-23228	GGGCTACTCACGTCCTCTCCCCCTTTT	3	<lmxll>	ll	128	lm	141		

>ref-23249	CAAACAGACACATTGCCTCCGTGCTTG	3	<lmxll>	ll	136	lm	132		
>ref-23253	TAAGCCGTTACGTTGTCTCCCTGGCCA	1	<nnxnp>	nn	143	np	125		
>ref-23260	CCCTTCGTCACATTCTCTCCTGTGCCT	7	<nnxnp>	nn	142	np	129		
>ref-23285	CCCCTCTCTACATTCACTCCCCCACCT	26	<nnxnp>	nn	106	np	150		
>ref-23296	GCTCTAGGGACCCCAGCTCCAGTATCT	27	<lmxll>	ll	145	lm	123		
>ref-23297	CTCCTGCGCACGCCGCCTCCATCTCTC	6	<nnxnp>	nn	117	np	151		
>ref-23324	ACGATATCGACGCGACCTCCTTAGATG	3	<hkxhk>	hh	74	hk	136	kk	37
>ref-23343	CCACACAGAACACCCCCTCCCCCTTCC	2	<lmxll>	ll	30	lm	221		
>ref-23343	CCACACAGAACACCCCCTCCCCCTTCC	3	<lmxll>	ll	30	lm	220		
>ref-23344	TCTTCTTACACTTTCTCTCCTTCAGTC	25	<nnxnp>	nn	131	np	111		
>ref-23350	CACTTACCTACACGCACTCCCCACACT	3	<nnxnp>	nn	207	np	48		
>ref-23350	CACTTACCTACACGCACTCCCCACACT	5	<hkxhk>	hh	0	hk	158	kk	85
>ref-23350	CACTTACCTACACGCACTCCCCACACT	27	<hkxhk>	hh	0	hk	271	kk	1
>ref-23363	ACCAACTAGACGCGGTCTCCCTATATA	14	<hkxhk>	hh	53	hk	132	kk	46
>ref-23385	CCGAGAGTGACCCACCCTCCAGGCTAT	12	<hkxhk>	hh	1	hk	269	kk	0
>ref-23386	CACTACAAAACGCGAACTCCGGGCCGG	2	<lmxll>	ll	108	lm	121		
>ref-23402	TCTCCATGCACCCCCCCTCCACACACA	6	<nnxnp>	nn	264	np	0		
>ref-23403	CCTTCTATTACCTCTTCTCCCTCTCCC	1	<lmxll>	ll	19	lm	228		
>ref-23403	CCTTCTATTACCTCTTCTCCCTCTCCC	25	<lmxll>	ll	20	lm	226		
>ref-23410	ACCTCCCTTACTTAGCCTCCTTTTTCT	21	<lmxll>	ll	107	lm	139		
>ref-23460	GCATTTTGAACAGCCACTCCGATCTTT	13	<lmxll>	ll	134	lm	119		
>ref-23489	GTCTCTCTAACTGTCACTCCTTCCCTT	27	<nnxnp>	nn	261	np	1		
>ref-23507	GTTGTGGAGACGTTTCCTCCTTGAAAT	24	<nnxnp>	nn	145	np	120		
>ref-23515	TCGACATCCACAGGTCCTCCTTGACTT	1	<hkxhk>	hh	0	hk	264	kk	7
>ref-23515	TCGACATCCACAGGTCCTCCTTGACTT	5	<hkxhk>	hh	0	hk	273	kk	0
>ref-23516	CCTGCTCCAACTCCTTCTCCCCCTCTC	9	<lmxll>	ll	128	lm	126		
>ref-23516	CCTGCTCCAACTCCTTCTCCCCCTCTC	16	<lmxll>	ll	120	lm	131		
>ref-23516	CCTGCTCCAACTCCTTCTCCCCCTCTC	25	<lmxll>	ll	152	lm	112		
>ref-23520	CTCCGCTGGACCCAAACTCCCATTCCA	26	<hkxhk>	hh	49	hk	136	kk	76
>ref-23573	TATTCCACTACCCCTTCTCCCTCCCCA	26	<nnxnp>	nn	257	np	0		
>ref-23573	TATTCCACTACCCCTTCTCCCTCCCCA	27	<nnxnp>	nn	254	np	0		
>ref-23580	TCATCTTCTACACCTCCTCCCTCCATT	3	<nnxnp>	nn	142	np	127		
>ref-23589	TACATAAGCACACAAGCTCCCATGTCA	8	<lmxll>	ll	120	lm	141		
>ref-23594	GCTCCTCCTACGCGCCCTCCGTCACAC	9	<nnxnp>	nn	271	np	1		
>ref-23594	GCTCCTCCTACGCGCCCTCCGTCACAC	16	<lmxll>	ll	135	lm	135		
>ref-23594	GCTCCTCCTACGCGCCCTCCGTCACAC	21	<lmxll>	ll	135	lm	135		
>ref-23633	ATCCCCCCCACTCCCGCTCCCCCATTC	16	<hkxhk>	hh	0	hk	195	kk	35
>ref-23662	GTGGGAGGGACAGCACCTCCAGCATAT	5	<lmxll>	ll	142	lm	123		

>ref-23669	TCTCTCCCAACATCTACTCCGTCTACT	25	<nnxnp>	nn	124	np	136		
>ref-23681	CACTCTCTCACAAACACTCCCCTTCAT	1	<nnxnp>	nn	105	np	142		
>ref-23717	CACATGGGTACCCCTACTCCCCCCACC	2	<lmxll>	ll	121	lm	142		
>ref-23730	CTCCTGCACACATGCACTCCTGCACAC	12	<hkxhk>	hh	3	hk	266	kk	1
>ref-23730	CTCCTGCACACATGCACTCCTGCACAC	13	<hkxhk>	hh	1	hk	266	kk	3
>ref-23730	CTCCTGCACACATGCACTCCTGCACAC	14	<hkxhk>	hh	1	hk	232	kk	24
>ref-23730	CTCCTGCACACATGCACTCCTGCACAC	22	<hkxhk>	hh	1	hk	231	kk	25
>ref-23731	AAACACGGGACAGCGGCTCCTTCGGTG	9	<nnxnp>	nn	265	np	0		
>ref-23751	CACCTGGTCACTGAGTCTCCTTCCACA	15	<lmxll>	ll	115	lm	150		
>ref-23751	CACCTGGTCACTGAGTCTCCTTCCACA	25	<lmxll>	ll	115	lm	150		
>ref-23753	AACATAAGTACGCTGTCTCCAGGGGAC	5	<nnxnp>	nn	189	np	75		
>ref-23753	AACATAAGTACGCTGTCTCCAGGGGAC	23	<hkxhk>	hh	0	hk	260	kk	1
>ref-23753	AACATAAGTACGCTGTCTCCAGGGGAC	26	<hkxhk>	hh	12	hk	254	kk	0
>ref-23753	AACATAAGTACGCTGTCTCCAGGGGAC	27	<hkxhk>	hh	0	hk	269	kk	3
>ref-23766	ACTCCGACTACGATGTCTCCTCGCTCG	23	<nnxnp>	nn	272	np	0		
>ref-23793	AACAGCAGCACGCTCCCTCCCTATCCT	5	<lmxll>	ll	117	lm	141		
>ref-23798	TCACTCCCCACCGCTCCTCCTTCTCCC	9	<lmxll>	ll	140	lm	110		
>ref-23806	ACTACGTGGACCACATCTCCGTTCTCT	1	<nnxnp>	nn	273	np	0		
>ref-23806	ACTACGTGGACCACATCTCCGTTCTCT	2	<nnxnp>	nn	273	np	0		
>ref-23824	CTCCGCCTAACACCATCTCCATCATGT	14	<nnxnp>	nn	126	np	129		
>ref-23825	CCTCCCTGCACCCTGGCTCCTCCCCCT	5	<hkxhk>	hh	1	hk	266	kk	0
>ref-23825	CCTCCCTGCACCCTGGCTCCTCCCCCT	22	<hkxhk>	hh	1	hk	266	kk	0
>ref-23827	CCATGATGGACTTCCCCTCCTGCTCTC	12	<nnxnp>	nn	120	np	144		
>ref-23860	TATCACCATACTGCTTCTCCTCACTCC	5	<lmxll>	ll	128	lm	133		
>ref-23861	CCACCGGCCACACCCTCTCCCTCTCTC	7	<hkxhk>	hh	1	hk	255	kk	0
>ref-23861	CCACCGGCCACACCCTCTCCCTCTCTC	26	<hkxhk>	hh	1	hk	254	kk	0
>ref-23864	CTCACAGAAACGTACGCTCCGACATAT	6	<nnxnp>	nn	114	np	115		
>ref-23872	CACATTATCACCAGCGCTCCATCCAAC	24	<nnxnp>	nn	272	np	0		
>ref-23880	ACTGCGCGCACGCCTCCTCCTTAGCAG	7	<lmxll>	ll	121	lm	145		
>ref-23898	CTCTGCCTCACTATATCTCCCTCATTA	6	<hkxhk>	hh	5	hk	264	kk	0
>ref-23898	CTCTGCCTCACTATATCTCCCTCATTA	7	<hkxhk>	hh	5	hk	264	kk	0
>ref-23905	GTTGCTTTTACTCGCGCTCCCTCTCCT	25	<nnxnp>	nn	262	np	0		
>ref-23909	CTTACTCCGACTACGACTCCTCCTACT	1	<lmxll>	ll	217	lm	7		
>ref-23943	CAGGCAAGGACACTCACTCCAACCTAG	1	<nnxnp>	nn	272	np	0		
>ref-23949	TTCCTTTACACCATATCTCCTTTTCCC	27	<nnxnp>	nn	119	np	124		
>ref-23961	GAACGTAGAACACTCCCTCCATCAAAG	12	<lmxll>	ll	134	lm	114		
>ref-24040	TTGTTGCATACACACACTCCCACCCTC	9	<hkxhk>	hh	66	hk	150	kk	50
>ref-24042	CTATCCTCAACCCCCACTCCACATCCC	4	<hkxhk>	hh	1	hk	262	kk	2

>ref-24042	CTATCCTCAACCCCCACTCCACATCCC	7	<hkxhk>	hh	1	hk	262	kk	2
>ref-24052	AGTCCCATGACATCATCTCCATAGCTG	2	<nnxnp>	nn	141	np	124		
>ref-24071	TCCCTTTCCACTTTGTCTCCTGTAAAC	3	<hkxhk>	hh	14	hk	255	kk	0
>ref-24071	TCCCTTTCCACTTTGTCTCCTGTAAAC	21	<hkxhk>	hh	0	hk	269	kk	2
>ref-24071	TCCCTTTCCACTTTGTCTCCTGTAAAC	23	<hkxhk>	hh	0	hk	261	kk	4
>ref-24071	TCCCTTTCCACTTTGTCTCCTGTAAAC	25	<hkxhk>	hh	23	hk	241	kk	0
>ref-24078	ACCCCGAGTACGCCGACTCCCTCGTCG	23	<nnxnp>	nn	136	np	133		
>ref-24099	CCCAAATCCACCCCCACTCCACCCACT	4	<nnxnp>	nn	126	np	108		
>ref-24124	CATACCCTTACCCTACCTCCTTCCCTA	9	<nnxnp>	nn	92	np	134		
>ref-24124	CATACCCTTACCCTACCTCCTTCCCTA	21	<nnxnp>	nn	92	np	135		
>ref-24128	ATGATCTACACTCGTTCTCCTAAGTTA	27	<lmxll>	ll	113	lm	150		
>ref-24129	AGTATGAATACTCTGTCTCCGACTGCA	1	<lmxll>	ll	152	lm	115		
>ref-24129	AGTATGAATACTCTGTCTCCGACTGCA	2	<hkxhk>	hh	0	hk	267	kk	5
>ref-24129	AGTATGAATACTCTGTCTCCGACTGCA	16	<hkxhk>	hh	1	hk	272	kk	0
>ref-24138	ATTAACCTAACCCAGCCTCCTTATCAA	2	<lmxll>	ll	128	lm	129		
>ref-24156	ACTACCAACACACCACCTCCACCGCTA	3	<hkxhk>	hh	0	hk	273	kk	0
>ref-24168	ATAAGGAGAACCACGGCTCCTGGACTG	14	<nnxnp>	nn	121	np	149		
>ref-24213	CACCGCGCCACCGCCGCTCCTCCGCCT	4	<nnxnp>	nn	123	np	149		
>ref-24252	CTGACATGCACTTGTTCTCCTCCAATA	26	<hkxhk>	hh	72	hk	137	kk	44
>ref-24259	AAAGCAGTAACATCTGCTCCTTGGCGT	1	<lmxll>	ll	132	lm	137		
>ref-24279	CTTTCCGTGACCACTCCTCCCTAGCAA	27	<nnxnp>	nn	273	np	0		
>ref-24299	GATCCGAGTACGACAACTCCAATTCCT	9	<lmxll>	ll	46	lm	199		
>ref-24299	GATCCGAGTACGACAACTCCAATTCCT	15	<lmxll>	ll	47	lm	200		
>ref-24300	CTAAGTATAACCTTCTCTCCTTATAAA	27	<hkxhk>	hh	45	hk	135	kk	63
>ref-24306	GACGGGACTACACGTGCTCCATTTCTA	1	<lmxll>	ll	189	lm	68		
>ref-24306	GACGGGACTACACGTGCTCCATTTCTA	4	<lmxll>	ll	190	lm	68		
>ref-24333	AGCCGTCACACTTCTCCTCCTCCTCTT	1	<hkxhk>	hh	1	hk	271	kk	0
>ref-24333	AGCCGTCACACTTCTCCTCCTCCTCTT	12	<hkxhk>	hh	0	hk	271	kk	1
>ref-24333	AGCCGTCACACTTCTCCTCCTCCTCTT	16	<hkxhk>	hh	18	hk	252	kk	1
>ref-24334	CACGCTCATACCCAAGCTCCTTTGACC	24	<lmxll>	ll	126	lm	144		
>ref-24340	TCCCTCATTACATATACTCCATGTAGA	13	<lmxll>	ll	0	lm	257		
>ref-24349	ATATCATAAACGGACTCTCCCATCAAC	6	<hkxhk>	hh	0	hk	259	kk	6
>ref-24353	TCCTACGTAACGCCGCCTCCATGCTGG	21	<hkxhk>	hh	48	hk	146	kk	69
>ref-24355	GTCCCTTACACCCTCTCTCCTCGTATG	16	<hkxhk>	hh	57	hk	137	kk	54
>ref-24355	GTCCCTTACACCCTCTCTCCTCGTATG	23	<hkxhk>	hh	57	hk	137	kk	53
>ref-24361	TATCTCCCCACTTGATCTCCCCCTCTT	14	<lmxll>	ll	131	lm	118		
>ref-24361	TATCTCCCCACTTGATCTCCCCCTCTT	25	<lmxll>	ll	132	lm	118		
>ref-24373	GGAAATACCACCACGCCTCCCAATACT	27	<lmxll>	ll	130	lm	129		

>ref-24376	TCTCTTATAACAATCTCTCCCTCTGTT	15	<lmxll>	ll	262	lm	0		
>ref-24380	CATCCTCCAACCTTTCCTCCCTCCATC	2	<hkxhk>	hh	0	hk	269	kk	2
>ref-24380	CATCCTCCAACCTTTCCTCCCTCCATC	3	<lmxll>	ll	10	lm	257		
>ref-24380	CATCCTCCAACCTTTCCTCCCTCCATC	12	<nnxnp>	nn	179	np	80		
>ref-24385	TCTCTCTTCACCTTCCCTCCTCCTATC	25	<lmxll>	ll	34	lm	207		
>ref-24400	ACTAATCACACACGCGCTCCCTCTGAT	13	<hkxhk>	hh	12	hk	252	kk	0
>ref-24400	ACTAATCACACACGCGCTCCCTCTGAT	16	<hkxhk>	hh	0	hk	252	kk	11
>ref-24408	GCCCCTCGCACCCCTCCTCCTCAACAC	21	<lmxll>	ll	121	lm	141		
>ref-24408	GCCCCTCGCACCCCTCCTCCTCAACAC	22	<lmxll>	ll	121	lm	141		
>ref-24425	CTTTCACACACTCCTTCTCCTTCCCTT	9	<lmxll>	ll	23	lm	230		
>ref-24425	CTTTCACACACTCCTTCTCCTTCCCTT	16	<lmxll>	ll	23	lm	231		
>ref-24438	AACACCACAACATTGTCTCCTAAACAG	1	<lmxll>	ll	108	lm	117		
>ref-24438	AACACCACAACATTGTCTCCTAAACAG	3	<lmxll>	ll	110	lm	116		
>ref-24442	CAGAAAGGAACCAAGGCTCCTCAAGCT	21	<nnxnp>	nn	101	np	120		
>ref-24450	TCTATCTATACAGGAGCTCCCATCTCC	9	<nnxnp>	nn	256	np	0		
>ref-24456	GTCTTGAAAACCCTATCTCCTTGAGTC	24	<lmxll>	ll	116	lm	132		
>ref-24471	TCCATCTCCACCATCCCTCCCTCCACT	16	<lmxll>	ll	73	lm	191		
>ref-24482	TTTCACTTGACTCTTTCTCCTTGCTTA	25	<hkxhk>	hh	48	hk	120	kk	63
>ref-24516	ATGCATGGGACTGCAACTCCAGCTGAA	9	<nnxnp>	nn	124	np	128		
>ref-24561	TGGATAAACACTTGTCCTCCTCCTTCT	25	<hkxhk>	hh	54	hk	159	kk	49
>ref-24569	TAGGACTAAACAAGCCCTCCTTCTTCA	9	<nnxnp>	nn	102	np	144		
>ref-24576	TTCAGCCAGACCGAAGCTCCGACTCTG	9	<lmxll>	ll	136	lm	128		
>ref-24591	TCCATTCTCACATCTTCTCCTCATCAT	26	<hkxhk>	hh	67	hk	144	kk	53
>ref-24612	TTTCCTCCTACAGGAACTCCGGGCTGC	5	<hkxhk>	hh	142	hk	108	kk	0
>ref-24612	TTTCCTCCTACAGGAACTCCGGGCTGC	7	<nnxnp>	nn	5	np	265		
>ref-24612	TTTCCTCCTACAGGAACTCCGGGCTGC	13	<lmxll>	ll	11	lm	257		
>ref-24612	TTTCCTCCTACAGGAACTCCGGGCTGC	15	<lmxll>	ll	11	lm	259		
>ref-24627	CTCCCCCATACCGTCTCTCCCCTCTTC	16	<nnxnp>	nn	128	np	104		
>ref-24627	CTCCCCCATACCGTCTCTCCCCTCTTC	26	<nnxnp>	nn	122	np	111		
>ref-24641	TTGCAAGTAACACGCTCTCCAGTAACA	9	<nnxnp>	nn	261	np	0		
>ref-24659	GGAGATGCTACTCCCTCTCCCTCTCTC	4	<nnxnp>	nn	150	np	112		
>ref-24659	GGAGATGCTACTCCCTCTCCCTCTCTC	14	<nnxnp>	nn	150	np	112		
>ref-24695	ATAGTGGAAACGTCATCTCCAAATACA	1	<hkxhk>	hh	55	hk	127	kk	56
>ref-24712	CACGACACGACCCACACTCCGCGCTTC	2	<hkxhk>	hh	0	hk	273	kk	0
>ref-24712	CACGACACGACCCACACTCCGCGCTTC	8	<hkxhk>	hh	0	hk	273	kk	0
>ref-24712	CACGACACGACCCACACTCCGCGCTTC	14	<lmxll>	ll	12	lm	258		
>ref-24717	GTCGTGCTAACCTTGGCTCCTGTGTGT	5	<lmxll>	ll	123	lm	139		
>ref-24725	AACGAACAGACTATATCTCCAATCAGT	7	<lmxll>	ll	119	lm	128		

>ref-24726	TGTCTCCCTACCTTCCCTCCTCCCACT	15	<lmxll>	ll	134	lm	112		
>ref-24735	CCCTCCGCCACTCCTTCTCCCCCATCC	2	<hkxhk>	hh	25	hk	151	kk	65
>ref-24735	CCCTCCGCCACTCCTTCTCCCCCATCC	13	<lmxll>	ll	118	lm	134		
>ref-24746	TCCACCCTTACCATCCCTCCACCCTAC	7	<nnxnp>	nn	119	np	137		
>ref-24766	TTATTCCATACACTTACTCCATGATAT	13	<hkxhk>	hh	8	hk	250	kk	1
>ref-24800	CCCTCTATTACCCTCCCTCCTCCCCTT	6	<nnxnp>	nn	8	np	257		
>ref-24800	CCCTCTATTACCCTCCCTCCTCCCCTT	7	<nnxnp>	nn	7	np	258		
>ref-24805	ACACACACTACGTTTTCTCCAAGGTAG	7	<nnxnp>	nn	226	np	0		
>ref-24826	CTCCCTTTCACCTCCTCTCCCTCTTTT	2	<nnxnp>	nn	82	np	177		
>ref-24826	CTCCCTTTCACCTCCTCTCCCTCTTTT	15	<hkxhk>	hh	3	hk	260	kk	1
>ref-24826	CTCCCTTTCACCTCCTCTCCCTCTTTT	21	<nnxnp>	nn	82	np	179		
>ref-24834	CCAATCCCTACCTTCGCTCCCTTACCA	7	<nnxnp>	nn	127	np	142		
>ref-24834	CCAATCCCTACCTTCGCTCCCTTACCA	27	<hkxhk>	hh	55	hk	153	kk	55
>ref-24841	TCTTCCTCCACTCTTCCTCCCCTTTCC	16	<lmxll>	ll	80	lm	154		
>ref-24848	CCGACACCCACATTATCTCCCCGCCGC	13	<hkxhk>	hh	60	hk	143	kk	52
>ref-24861	TGGAATTACACTTCACCTCCTCTATAA	23	<lmxll>	ll	113	lm	115		
>ref-24871	AAGCAATAGACCTGAGCTCCAGTGCTC	4	<lmxll>	ll	140	lm	126		
>ref-24873	TTTCTTTCTACGCCCACTCCTCTTCCT	8	<nnxnp>	nn	136	np	83		
>ref-24884	TTCTCCCCTACTGACACTCCTAGGAAT	1	<hkxhk>	hh	0	hk	202	kk	49
>ref-24884	TTCTCCCCTACTGACACTCCTAGGAAT	3	<lmxll>	ll	159	lm	95		
>ref-24884	TTCTCCCCTACTGACACTCCTAGGAAT	6	<hkxhk>	hh	6	hk	261	kk	0
>ref-24884	TTCTCCCCTACTGACACTCCTAGGAAT	8	<lmxll>	ll	158	lm	95		
>ref-24884	TTCTCCCCTACTGACACTCCTAGGAAT	15	<hkxhk>	hh	49	hk	202	kk	0
>ref-24898	CCCACGCCTACCCCTTCTCCTCTCCCC	7	<lmxll>	ll	244	lm	0		
>ref-24899	ACACACACTACCTTTCCTCCCTGTCTC	23	<lmxll>	ll	145	lm	117		
>ref-24933	TACACCCCCACCCCCACTCCCTCACTC	1	<nnxnp>	nn	8	np	258		
>ref-24933	TACACCCCCACCCCCACTCCCTCACTC	24	<nnxnp>	nn	7	np	259		
>ref-24939	GATTAAGCTACTCACCCTCCCCTCAAT	24	<nnxnp>	nn	129	np	109		
>ref-24940	TCCCCCTCAACCCCATCTCCCTTACTC	14	<lmxll>	ll	112	lm	113		
>ref-24948	AGGCAAAACACCCTCCCTCCCATCTCT	15	<lmxll>	ll	118	lm	137		
>ref-24986	TCTTCTTCCACCTCTACTCCTTTCTTC	27	<lmxll>	ll	129	lm	108		
>ref-24987	TCCCCTTCCACAATTCCTCCGTATCTC	22	<lmxll>	ll	129	lm	119		
>ref-24995	ACCACCTCCACCCCCTCTCCCTGTTAA	14	<nnxnp>	nn	125	np	138		
>ref-24997	GACACCAGAACAACACCTCCAGGGGCG	5	<nnxnp>	nn	255	np	0		
>ref-25011	TACTCCAACACTTCTCCTCCATCACTT	22	<nnxnp>	nn	131	np	104		
>ref-25011	TACTCCAACACTTCTCCTCCATCACTT	23	<nnxnp>	nn	135	np	102		
>ref-25027	GTCTGTATCACTCCCGCTCCTCCTCTG	22	<hkxhk>	hh	62	hk	136	kk	67
>ref-25036	CCTCCCCCCACCCTCTCTCCCTCTTAA	12	<nnxnp>	nn	221	np	1		

>ref-25061	CCTCACTCAACACTCTCTCCCCTCTCT	15	<hkxhk>	hh	0	hk	272	kk	0
>ref-25061	CCTCACTCAACACTCTCTCCCCTCTCT	24	<hkxhk>	hh	0	hk	272	kk	0
>ref-25091	ACCTCATCCACTGATCCTCCTTCACCC	21	<lmxll>	ll	122	lm	141		
>ref-25105	GCCCCCTGCACCTCTCCTCCTCTCCTC	13	<lmxll>	ll	24	lm	227		
>ref-25105	GCCCCCTGCACCTCTCCTCCTCTCCTC	22	<lmxll>	ll	23	lm	225		
>ref-25106	CCCACGTGCACCTTATCTCCTTGCTTC	4	<hkxhk>	hh	61	hk	137	kk	54
>ref-25165	GATCCGCGAACGGCCGCTCCTCGTCAG	25	<lmxll>	ll	111	lm	138		
>ref-25185	TTCAACTTCACTGCAACTCCTTGGGTC	4	<lmxll>	ll	84	lm	182		
>ref-25185	TTCAACTTCACTGCAACTCCTTGGGTC	22	<hkxhk>	hh	0	hk	273	kk	0
>ref-25191	TCTCGTTTCACTTCTCCTCCTTCATGT	5	<hkxhk>	hh	64	hk	147	kk	53
>ref-25193	CATACCTTCACATATTCTCCAGTACTA	25	<nnxnp>	nn	110	np	112		
>ref-25194	TATATGTTAACCGAACCTCCGACCCGA	22	<hkxhk>	hh	27	hk	191	kk	1
>ref-25206	CCAGCATCCACCTCCTCTCCCCTGCAT	8	<nnxnp>	nn	140	np	127		
>ref-25209	CCGCCTCAAACCCAAGCTCCCTCGCAG	3	<hkxhk>	hh	66	hk	140	kk	54
>ref-25221	TACCCACCCACACCCCCTCCACTCTCC	1	<lmxll>	ll	148	lm	96		
>ref-25221	TACCCACCCACACCCCCTCCACTCTCC	2	<lmxll>	ll	148	lm	95		
>ref-25221	TACCCACCCACACCCCCTCCACTCTCC	5	<nnxnp>	nn	3	np	245		
>ref-25221	TACCCACCCACACCCCCTCCACTCTCC	21	<nnxnp>	nn	3	np	246		
>ref-25241	ATGCTATCAACTTACCCTCCTCCTTCA	24	<nnxnp>	nn	270	np	0		
>ref-25241	ATGCTATCAACTTACCCTCCTCCTTCA	27	<nnxnp>	nn	269	np	0		
>ref-25260	TTTATGAATACTCTGTCTCCTTATCAC	6	<lmxll>	ll	153	lm	107		
>ref-25263	GCCCTTCCTACAGCCCCTCCCCCCCTC	25	<nnxnp>	nn	156	np	88		
>ref-25263	GCCCTTCCTACAGCCCCTCCCCCCCTC	26	<nnxnp>	nn	156	np	89		
>ref-25272	TGCGTGTGCACAATACCTCCCTTGTGG	4	<lmxll>	ll	0	lm	272		
>ref-25272	TGCGTGTGCACAATACCTCCCTTGTGG	25	<lmxll>	ll	0	lm	272		
>ref-25279	CCTTCAACAACTCCATCTCCCATTGTA	21	<hkxhk>	hh	0	hk	270	kk	0
>ref-25284	AAATCCCTCACTTTACCTCCGGTTTCC	1	<lmxll>	ll	120	lm	110		
>ref-25284	AAATCCCTCACTTTACCTCCGGTTTCC	2	<lmxll>	ll	119	lm	110		
>ref-25288	ACATCCAGAACAAGAACTCCTCCTACT	23	<hkxhk>	hh	0	hk	272	kk	0
>ref-25288	ACATCCAGAACAAGAACTCCTCCTACT	25	<hkxhk>	hh	0	hk	272	kk	0
>ref-25332	GTAGCGGGAACCCAGGCTCCAGGAGGA	4	<lmxll>	ll	176	lm	88		
>ref-25332	GTAGCGGGAACCCAGGCTCCAGGAGGA	5	<hkxhk>	hh	0	hk	260	kk	9
>ref-25332	GTAGCGGGAACCCAGGCTCCAGGAGGA	9	<lmxll>	ll	176	lm	88		
>ref-25332	GTAGCGGGAACCCAGGCTCCAGGAGGA	25	<hkxhk>	hh	0	hk	260	kk	9
>ref-25332	GTAGCGGGAACCCAGGCTCCAGGAGGA	26	<hkxhk>	hh	0	hk	258	kk	10
>ref-25335	CATCAATACACCAATTCTCCACTCCCC	24	<lmxll>	ll	125	lm	124		
>ref-25347	AGATGTAGTACCTTGCCTCCATGAGAA	6	<lmxll>	ll	104	lm	157		
>ref-25352	ACCCGGCCTACCCCCCCTCCTCCTCCT	5	<lmxll>	ll	206	lm	21		

>ref-25352	ACCCGGCCTACCCCCCCTCCTCCTCCT	15	<lmxll>	ll	262	lm	1		
>ref-25352	ACCCGGCCTACCCCCCCTCCTCCTCCT	21	<lmxll>	ll	270	lm	0		
>ref-25356	AGCCCAGCGACTCACCCTCCCTTGTCA	3	<hkxhk>	hh	8	hk	265	kk	0
>ref-25356	AGCCCAGCGACTCACCCTCCCTTGTCA	21	<hkxhk>	hh	8	hk	265	kk	0
>ref-25359	GCATTCCCCACCTGCCCTCCACTTCCA	4	<nnxnp>	nn	127	np	143		
>ref-25363	AGAAGCTCTACCTTCTCTCCCTCAAAG	27	<nnxnp>	nn	134	np	118		
>ref-25366	TTCTTCCACACCTATCCTCCCCTCCCC	5	<lmxll>	ll	104	lm	159		
>ref-25380	CATGATCCAACTCTCACTCCGGACTCC	14	<hkxhk>	hh	55	hk	154	kk	55
>ref-25396	CACATTATAACCGCCTCTCCATTCCCT	1	<hkxhk>	hh	55	hk	152	kk	57
>ref-25423	TTGGGATCGACAGGGCCTCCAGGGTAC	6	<hkxhk>	hh	29	hk	234	kk	0
>ref-25423	TTGGGATCGACAGGGCCTCCAGGGTAC	24	<hkxhk>	hh	0	hk	268	kk	2
>ref-25429	ATGAATTGCACGTCGCCTCCTCATGGA	9	<lmxll>	ll	131	lm	133		
>ref-25433	ATAGCACCAACACCTTCTCCTCCTGCT	16	<hkxhk>	hh	65	hk	146	kk	47
>ref-25435	CCTATCCCCACCACCCCTCCCTCAACC	4	<nnxnp>	nn	117	np	133		
>ref-25435	CCTATCCCCACCACCCCTCCCTCAACC	5	<nnxnp>	nn	101	np	148		
>ref-25453	AAACCTCTGACTCGTCCTCCTGGCCGT	21	<hkxhk>	hh	0	hk	268	kk	1
>ref-25453	AAACCTCTGACTCGTCCTCCTGGCCGT	24	<hkxhk>	hh	1	hk	268	kk	0
>ref-25487	CTCTTCTGTACCTCACCTCCTTCTTTC	14	<lmxll>	ll	134	lm	115		
>ref-25487	CTCTTCTGTACCTCACCTCCTTCTTTC	22	<lmxll>	ll	107	lm	143		
>ref-25538	CCATACGTAACTTCATCTCCCACAGCT	25	<hkxhk>	hh	64	hk	136	kk	53
>ref-25540	CAAGCACCAACGGGACCTCCAACACAA	2	<hkxhk>	hh	0	hk	271	kk	0
>ref-25540	CAAGCACCAACGGGACCTCCAACACAA	26	<hkxhk>	hh	138	hk	123	kk	0
>ref-25550	CAAGCATTCACGTTATCTCCAATCCGT	8	<nnxnp>	nn	127	np	109		
>ref-25578	TCAACGACCACAGGGACTCCTCCCGAC	12	<nnxnp>	nn	273	np	0		
>ref-25586	CATTCAGCAACTCTTCCTCCTCTTCCT	14	<lmxll>	ll	108	lm	119		
>ref-25588	TATCAGTACACTGCTACTCCATTCCCC	7	<lmxll>	ll	148	lm	112		
>ref-25607	CTTACATGCACTCACCCTCCTTCATCT	22	<nnxnp>	nn	137	np	127		
>ref-25637	ATCCCACCAACACGCTCTCCATCCCTC	16	<nnxnp>	nn	117	np	152		
>ref-25655	GAAATCATCACAGAACCTCCAAGTGTT	4	<hkxhk>	hh	0	hk	273	kk	0
>ref-25655	GAAATCATCACAGAACCTCCAAGTGTT	8	<hkxhk>	hh	0	hk	273	kk	0
>ref-25655	GAAATCATCACAGAACCTCCAAGTGTT	22	<lmxll>	ll	184	lm	75		
>ref-25661	ATCAACTCCACACTACCTCCCTCCACA	23	<nnxnp>	nn	135	np	130		
>ref-25663	TTAGAATTCACTCCCTCTCCGTTAACC	23	<hkxhk>	hh	45	hk	119	kk	57
>ref-25665	CTAAACATCACGCATTCTCCTTCTTCC	12	<hkxhk>	hh	67	hk	131	kk	62
>ref-25672	TACATTCGCACTCCCTCTCCGCTGTTA	5	<nnxnp>	nn	128	np	144		
>ref-25672	TACATTCGCACTCCCTCTCCGCTGTTA	6	<hkxhk>	hh	74	hk	141	kk	53
>ref-25673	CTCCTTCCCACCCCCTCTCCCCATCAC	14	<nnxnp>	nn	97	np	150		
>ref-25673	CTCCTTCCCACCCCCTCTCCCCATCAC	24	<nnxnp>	nn	157	np	99		

>ref-25694	TGTGTGTTTACACGAGCTCCCTCTCCT	9	<hkxhk>	hh	60	hk	149	kk	50
>ref-25711	CCTTCTCCTACTCTTGCTCCCCTCCTC	14	<nnxnp>	nn	247	np	6		
>ref-25737	TCTCTATCTACTCCGCCTCCTCCGTCG	15	<nnxnp>	nn	109	np	154		
>ref-25741	TTAGTTTCCACCCTTCCTCCCTCCCCC	1	<nnxnp>	nn	120	np	139		
>ref-25758	CACGCAGCCACGCCCCCTCCTCAATAG	16	<nnxnp>	nn	118	np	137		
>ref-25764	TCATCTTCCACATCTTCTCCCTTCCCT	27	<lmxll>	ll	149	lm	115		
>ref-25766	GAGTTATTAACGAACGCTCCCAAGTCC	4	<nnxnp>	nn	111	np	143		
>ref-25774	CCTTTTCTCACTTTCCCTCCCTTCCAT	2	<lmxll>	ll	126	lm	126		
>ref-25788	GGCAGGATAACCTAGGCTCCTACAGTT	16	<lmxll>	ll	120	lm	145		
>ref-25792	AAACACACAACTCAGCCTCCGACACAC	23	<nnxnp>	nn	138	np	131		
>ref-25799	CCCATTCACACACATTCTCCATTACAC	2	<nnxnp>	nn	122	np	126		
>ref-25844	TCCCCCTTCACCTCTTCTCCTCCCTAC	2	<nnxnp>	nn	245	np	0		
>ref-25856	CTCGTGGCCACGTTTTCTCCAAACGAG	24	<lmxll>	ll	107	lm	132		
>ref-25860	GTGAAGCGCACCCTATCTCCCCGAGAG	15	<lmxll>	ll	124	lm	114		
>ref-25863	TCTTCGTCCACCGTCTCTCCTTCTTTT	5	<hkxhk>	hh	83	hk	113	kk	25
>ref-25911	TCTCTCACAACATCATCTCCCACAGCA	12	<hkxhk>	hh	60	hk	162	kk	43
>ref-25913	CCATCTCCTACCCCCCCTCCCCCACCT	27	<lmxll>	ll	29	lm	193		
>ref-25959	TCCAGCACCACCACCTCTCCTAGCTTA	25	<lmxll>	ll	117	lm	140		
>ref-25960	TCTCTTGTTACATCAACTCCCTTCCCT	2	<nnxnp>	nn	124	np	132		
>ref-25989	CTACCCTTCACTTTACCTCCTCTTCTT	3	<nnxnp>	nn	117	np	137		
>ref-25989	CTACCCTTCACTTTACCTCCTCTTCTT	4	<nnxnp>	nn	117	np	137		
>ref-25990	TCTCTTTCAACTCCACCTCCACCACCA	1	<nnxnp>	nn	55	np	201		
>ref-25990	TCTCTTTCAACTCCACCTCCACCACCA	13	<nnxnp>	nn	56	np	202		
>ref-25990	TCTCTTTCAACTCCACCTCCACCACCA	16	<nnxnp>	nn	135	np	123		
>ref-25991	GGTTAGCTAACTAGCGCTCCATACAGG	23	<nnxnp>	nn	273	np	0		
>ref-25992	CCTGTCACCACAGCCCCTCCTTTTCTC	3	<hkxhk>	hh	0	hk	272	kk	0
>ref-25992	CCTGTCACCACAGCCCCTCCTTTTCTC	5	<hkxhk>	hh	0	hk	252	kk	11
>ref-25992	CCTGTCACCACAGCCCCTCCTTTTCTC	14	<nnxnp>	nn	151	np	109		
>ref-26002	TAAAATACAACAACAACTCCACCATCA	22	<lmxll>	ll	108	lm	111		
>ref-26032	CCTCCTATTACGTTATCTCCTACTGTC	22	<nnxnp>	nn	262	np	0		
>ref-26032	CCTCCTATTACGTTATCTCCTACTGTC	24	<nnxnp>	nn	260	np	0		
>ref-26055	TAATCCTCAACTGAACCTCCCTCTTTA	16	<lmxll>	ll	127	lm	120		
>ref-26056	ATCGTGGAGACAATGTCTCCTTAATTA	13	<hkxhk>	hh	18	hk	237	kk	0
>ref-26057	TCCTTCATCACTCTCTCTCCTTTACTC	23	<nnxnp>	nn	4	np	251		
>ref-26057	TCCTTCATCACTCTCTCTCCTTTACTC	24	<nnxnp>	nn	4	np	251		
>ref-26067	ACTTCCCCCACACTCTCTCCATCCTCT	14	<hkxhk>	hh	0	hk	273	kk	0
>ref-26067	ACTTCCCCCACACTCTCTCCATCCTCT	21	<lmxll>	ll	35	lm	231		
>ref-26067	ACTTCCCCCACACTCTCTCCATCCTCT	23	<lmxll>	ll	120	lm	152		

>ref-26071	CCCCACCCAACCACAGCTCCTCTCTTT	8	<lmxll>	ll	29	lm	229		
>ref-26071	CCCCACCCAACCACAGCTCCTCTCTTT	25	<lmxll>	ll	10	lm	259		
>ref-26071	CCCCACCCAACCACAGCTCCTCTCTTT	27	<lmxll>	ll	10	lm	259		
>ref-26084	CTGTCATCTACTTTCACTCCGTATCTT	2	<lmxll>	ll	119	lm	140		
>ref-26128	ACATACTTTACTGCCACTCCTGACTTT	5	<nnxnp>	nn	145	np	115		
>ref-26132	CCCTCTTCCACGGACCCTCCACCCATC	12	<lmxll>	ll	123	lm	111		
>ref-26136	AACCGAAGAACATCACCTCCCGCACCC	26	<lmxll>	ll	143	lm	99		
>ref-26136	AACCGAAGAACATCACCTCCCGCACCC	27	<lmxll>	ll	141	lm	100		
>ref-26142	CACTCAGTCACTCTCTCTCCGACACAT	25	<nnxnp>	nn	115	np	135		
>ref-26149	GAATAAGTCACTCTCTCTCCCTTCCTC	23	<lmxll>	ll	123	lm	125		
>ref-26153	AGCGGACACACACATTCTCCTTTCATA	15	<nnxnp>	nn	119	np	125		
>ref-26153	AGCGGACACACACATTCTCCTTTCATA	16	<nnxnp>	nn	119	np	125		
>ref-26158	CCCCTCCTCACTCCCACTCCATCCTCT	16	<lmxll>	ll	15	lm	245		
>ref-26158	CCCCTCCTCACTCCCACTCCATCCTCT	23	<lmxll>	ll	14	lm	246		
>ref-26171	GGAACACAGACAGCCTCTCCTGCTGAA	22	<nnxnp>	nn	263	np	0		
>ref-26217	AGAGCTCCTACATGCTCTCCTGAACAC	21	<hkxhk>	hh	59	hk	125	kk	58
>ref-26236	GCCTCTCTTACGTAGACTCCTCCTCAG	6	<hkxhk>	hh	59	hk	142	kk	59
>ref-26247	CCACCCACAACCCACACTCCCTCCCTG	2	<lmxll>	ll	228	lm	2		
>ref-26260	GTACTCCTTACTCCTCCTCCCTTCCCT	21	<nnxnp>	nn	118	np	132		
>ref-26271	CTCCCTTTAACCCCTCCTCCGGTTTGG	22	<lmxll>	ll	127	lm	122		
>ref-26289	CACAAGATTACTCATTCTCCTATCATA	3	<hkxhk>	hh	0	hk	197	kk	51
>ref-26289	CACAAGATTACTCATTCTCCTATCATA	6	<hkxhk>	hh	0	hk	254	kk	11
>ref-26294	AGCCCTTTCACATTCCCTCCCGCGGGA	1	<lmxll>	ll	138	lm	130		
>ref-26302	ACCACCCACACCTCCTCTCCTCCTCCA	15	<lmxll>	ll	132	lm	129		
>ref-26305	CAGTTTTCCACTCCTTCTCCTTCTTCT	14	<nnxnp>	nn	126	np	134		
>ref-26342	CTTCCTCATACCACCCCTCCATCACCT	1	<nnxnp>	nn	235	np	0		
>ref-26342	CTTCCTCATACCACCCCTCCATCACCT	2	<lmxll>	ll	125	lm	96		
>ref-26342	CTTCCTCATACCACCCCTCCATCACCT	9	<nnxnp>	nn	234	np	0		
>ref-26363	ATCGCGTTCACTTATTCTCCTTTTCTA	4	<nnxnp>	nn	156	np	87		
>ref-26363	ATCGCGTTCACTTATTCTCCTTTTCTA	26	<lmxll>	ll	94	lm	138		
>ref-26374	CAACCCTTCACATCCTCTCCCTCTACA	5	<nnxnp>	nn	259	np	0		
>ref-26394	TTGCGACAGACTCACCCTCCTCTCCCC	26	<lmxll>	ll	152	lm	117		
>ref-26395	TCTGCCCCCACCTCCTCTCCGTCAATT	23	<nnxnp>	nn	103	np	129		
>ref-26414	ACTCCACCAACTCGTCCTCCTCTTGGG	23	<nnxnp>	nn	113	np	139		
>ref-26414	ACTCCACCAACTCGTCCTCCTCTTGGG	27	<nnxnp>	nn	113	np	139		
>ref-26421	ATTTGATATACATTTTCTCCATACATA	3	<nnxnp>	nn	111	np	108		
>ref-26421	ATTTGATATACATTTTCTCCATACATA	26	<nnxnp>	nn	111	np	108		
>ref-26424	TGGGGGCTCACAGCTCCTCCTGCTCCA	6	<nnxnp>	nn	137	np	132		

>ref-26424	TGGGGGCTCACAGCTCCTCCTGCTCCA	26	<nnxnp>	nn	128	np	139		
>ref-26456	TCAGGACAGACTCTCTCTCCCACGCAT	4	<lmxll>	ll	128	lm	133		
>ref-26486	ATCCTCATGACTCGGACTCCGCTGCCT	15	<lmxll>	ll	122	lm	123		
>ref-26486	ATCCTCATGACTCGGACTCCGCTGCCT	16	<lmxll>	ll	121	lm	123		
>ref-26489	CATCTTTCCACCCTCCCTCCTCCTATC	2	<hkxhk>	hh	61	hk	143	kk	45
>ref-26509	TTCTATAAAACACGTCCTCCTCAAGCT	2	<lmxll>	ll	136	lm	127		
>ref-26510	CCTTCATCAACCCCTTCTCCCTTCTCC	6	<nnxnp>	nn	267	np	0		
>ref-26533	TGGCCGTGAACTACTGCTCCGTCTCCG	5	<lmxll>	ll	136	lm	128		
>ref-26533	TGGCCGTGAACTACTGCTCCGTCTCCG	13	<lmxll>	ll	136	lm	128		
>ref-26554	CCAGTCCCAACCCCTCCTCCTTCCTCT	16	<nnxnp>	nn	123	np	134		
>ref-26560	TCTTTGTGGACACTGGCTCCCCAGACT	4	<hkxhk>	hh	0	hk	272	kk	0
>ref-26560	TCTTTGTGGACACTGGCTCCCCAGACT	26	<hkxhk>	hh	0	hk	272	kk	0
>ref-26567	AGCATTGATACGCTGGCTCCCTACGTA	3	<hkxhk>	hh	2	hk	270	kk	0
>ref-26567	AGCATTGATACGCTGGCTCCCTACGTA	12	<hkxhk>	hh	3	hk	269	kk	0
>ref-26567	AGCATTGATACGCTGGCTCCCTACGTA	24	<hkxhk>	hh	0	hk	273	kk	0
>ref-26582	TGTTTGAAGACTCTTTCTCCCCTTCTT	7	<lmxll>	ll	87	lm	155		
>ref-26582	TGTTTGAAGACTCTTTCTCCCCTTCTT	8	<lmxll>	ll	88	lm	155		
>ref-26593	CTCTTCTTTACTTTCCCTCCATCCTTC	2	<hkxhk>	hh	0	hk	265	kk	0
>ref-26593	CTCTTCTTTACTTTCCCTCCATCCTTC	7	<hkxhk>	hh	0	hk	265	kk	0
>ref-26613	AATCAATGTACTAAGGCTCCATGAGCT	2	<lmxll>	ll	78	lm	185		
>ref-26613	AATCAATGTACTAAGGCTCCATGAGCT	3	<lmxll>	ll	2	lm	269		
>ref-26613	AATCAATGTACTAAGGCTCCATGAGCT	9	<hkxhk>	hh	0	hk	272	kk	1
>ref-26613	AATCAATGTACTAAGGCTCCATGAGCT	15	<lmxll>	ll	76	lm	189		
>ref-26613	AATCAATGTACTAAGGCTCCATGAGCT	16	<lmxll>	ll	71	lm	191		
>ref-26614	TGTTTTTTGACATTTCCTCCGCGGTTG	2	<lmxll>	ll	123	lm	122		
>ref-26624	GGAACCCTCACCGGCTCTCCCGCCCTC	27	<nnxnp>	nn	92	np	129		
>ref-26629	TTCCCCCTTACCTCCCCTCCTCTTCCT	1	<lmxll>	ll	16	lm	239		
>ref-26629	TTCCCCCTTACCTCCCCTCCTCTTCCT	14	<lmxll>	ll	21	lm	236		
>ref-26632	ACTTTAGCAACACTCGCTCCATGCTTC	16	<lmxll>	ll	109	lm	134		
>ref-26632	ACTTTAGCAACACTCGCTCCATGCTTC	21	<lmxll>	ll	109	lm	134		
>ref-26646	CCCCACTCCACCTTCCCTCCCTCCCAT	4	<nnxnp>	nn	267	np	0		
>ref-26646	CCCCACTCCACCTTCCCTCCCTCCCAT	16	<nnxnp>	nn	7	np	253		
>ref-26653	CGGCTTCGGACCCCCCCTCCGTCCCTA	24	<lmxll>	ll	127	lm	128		
>ref-26665	CCCGGAGACACGTCCGCTCCCAACCCT	26	<hkxhk>	hh	55	hk	152	kk	61
>ref-26678	GCCATGCCGACCTTGCCTCCATCCCGT	9	<nnxnp>	nn	125	np	112		
>ref-26693	CCGGTCCCCACCCTTCCTCCCCTCTTC	22	<lmxll>	ll	265	lm	0		
>ref-26695	TTCATTCCCACTCTTTCTCCCTCAGTG	23	<nnxnp>	nn	127	np	117		
>ref-26697	CCAGAGACCACGTGCTCTCCGCCTGTG	15	<lmxll>	ll	135	lm	137		

>ref-26717	ATGTCATCCACTCCCTCTCCTTCTTCC	25	<nnxnp>	nn	266	np	0		
>ref-26730	CTGCTCTTTACTCCTCCTCCTCTCCGT	23	<lmxll>	ll	154	lm	109		
>ref-26731	GGAACGGTAACTTCCGCTCCGGGCGTG	8	<nnxnp>	nn	123	np	139		
>ref-26731	GGAACGGTAACTTCCGCTCCGGGCGTG	12	<lmxll>	ll	137	lm	129		
>ref-26731	GGAACGGTAACTTCCGCTCCGGGCGTG	27	<lmxll>	ll	120	lm	139		
>ref-26745	ATTAAGTATACCACCTCTCCTCTATAA	23	<hkxhk>	hh	0	hk	81	kk	153
>ref-26763	CGAAACTCCACCACGCCTCCATTTCCC	7	<lmxll>	ll	115	lm	146		
>ref-26765	TTTCTTCCAACGATCCCTCCTACGGTT	23	<lmxll>	ll	143	lm	108		
>ref-26785	TCCCTCTCTACCGCCCCTCCCTTTCTT	12	<nnxnp>	nn	141	np	118		
>ref-26798	GCAGAGATGACATTCTCTCCCTCTCTT	26	<lmxll>	ll	126	lm	127		
>ref-26799	AGCTTTAGGACTATTACTCCCCGCTTG	16	<lmxll>	ll	128	lm	123		
>ref-26804	CTCACTATGACCATTACTCCTCACCAT	3	<nnxnp>	nn	109	np	116		
>ref-26835	CTACACGTAACCTGCCCTCCTAGTTTA	13	<lmxll>	ll	122	lm	104		
>ref-26839	TTCCTTCCGACCTTCCCTCCCCCTTCT	2	<lmxll>	ll	127	lm	129		
>ref-26841	ATACCTCTCACTCTCACTCCCTGTTAA	3	<lmxll>	ll	58	lm	169		
>ref-26841	ATACCTCTCACTCTCACTCCCTGTTAA	7	<lmxll>	ll	59	lm	169		
>ref-26844	AACTTCGTAACTACCACTCCTCTTGCT	6	<lmxll>	ll	220	lm	35		
>ref-26862	ACTACCTAAACAGCTGCTCCCCTGCAT	13	<lmxll>	ll	131	lm	132		
>ref-26862	ACTACCTAAACAGCTGCTCCCCTGCAT	16	<lmxll>	ll	131	lm	132		
>ref-26867	TTTTCATAAACCCTTCCTCCTTCACAT	23	<nnxnp>	nn	125	np	121		
>ref-26871	CTCCGCACCACACCTACTCCATTCCTC	4	<hkxhk>	hh	1	hk	257	kk	5
>ref-26871	CTCCGCACCACACCTACTCCATTCCTC	8	<hkxhk>	hh	5	hk	257	kk	1
>ref-26872	TCAAGATAAACAACCTCTCCTTCTCTC	4	<nnxnp>	nn	127	np	140		
>ref-26878	TCTTAATTCACTTCACCTCCCTTCCCT	6	<lmxll>	ll	261	lm	0		
>ref-26878	TCTTAATTCACTTCACCTCCCTTCCCT	16	<nnxnp>	nn	264	np	0		
>ref-26903	CTGATGCAAACAAACTCTCCTTCCATT	1	<lmxll>	ll	102	lm	154		
>ref-26903	CTGATGCAAACAAACTCTCCTTCCATT	23	<lmxll>	ll	102	lm	154		
>ref-26931	CTCGCCTTTACTCCCCCTCCTCCCTCC	2	<hkxhk>	hh	5	hk	233	kk	7
>ref-26931	CTCGCCTTTACTCCCCCTCCTCCCTCC	3	<lmxll>	ll	19	lm	221		
>ref-26931	CTCGCCTTTACTCCCCCTCCTCCCTCC	4	<nnxnp>	nn	225	np	11		
>ref-26931	CTCGCCTTTACTCCCCCTCCTCCCTCC	25	<nnxnp>	nn	231	np	12		
>ref-26936	ACACCGTAAACGGTGACTCCAGTTTCG	13	<nnxnp>	nn	148	np	118		
>ref-26943	ACTGATTGGACTTGGTCTCCCACGACA	3	<lmxll>	ll	142	lm	131		
>ref-26961	CACACACACACATACGCTCCGACTGCT	13	<lmxll>	ll	113	lm	144		
>ref-26961	CACACACACACATACGCTCCGACTGCT	14	<lmxll>	ll	117	lm	141		
>ref-26970	CCTTCACCCACTCCTCCTCCCTCAGCA	2	<hkxhk>	hh	36	hk	231	kk	0
>ref-26970	CCTTCACCCACTCCTCCTCCCTCAGCA	25	<hkxhk>	hh	0	hk	231	kk	32
>ref-27007	CCGGCCAGTACTGGCCCTCCGGTCTCA	1	<hkxhk>	hh	0	hk	273	kk	0

>ref-27007	CCGGCCAGTACTGGCCCTCCGGTCTCA	3	<nnxnp>	nn	61	np	199		
>ref-27007	CCGGCCAGTACTGGCCCTCCGGTCTCA	4	<hkxhk>	hh	14	hk	253	kk	0
>ref-27007	CCGGCCAGTACTGGCCCTCCGGTCTCA	5	<hkxhk>	hh	0	hk	273	kk	0
>ref-27007	CCGGCCAGTACTGGCCCTCCGGTCTCA	6	<hkxhk>	hh	0	hk	273	kk	0
>ref-27023	TAAGTAAACACCGCGTCTCCAAATGTA	16	<hkxhk>	hh	44	hk	156	kk	53
>ref-27078	CTTCCTCCTACCCCTTCTCCCCTCTCA	15	<lmxll>	ll	14	lm	234		
>ref-27078	CTTCCTCCTACCCCTTCTCCCCTCTCA	25	<lmxll>	ll	9	lm	245		
>ref-27097	AATGGTGTTACTCTTTCTCCCACTAAT	23	<nnxnp>	nn	140	np	109		
>ref-27109	TTTCCATCAACCCCCTCTCCTCTGTTA	4	<nnxnp>	nn	246	np	0		
>ref-27114	TCCCCTCTCACTGCGCCTCCTCCTAAG	2	<nnxnp>	nn	137	np	119		
>ref-27119	TTGCATGGCACACCATCTCCACGCTAC	16	<lmxll>	ll	126	lm	137		
>ref-27128	TTTTCCCTCACTCCCTCTCCCAGCCAC	1	<lmxll>	ll	2	lm	228		
>ref-27128	TTTTCCCTCACTCCCTCTCCCAGCCAC	26	<lmxll>	ll	2	lm	228		
>ref-27133	CTCCCCGCCACCACTCCTCCCTCCCTC	22	<nnxnp>	nn	266	np	0		
>ref-27140	CAAGTATTTACAATCCCTCCAGAGAAT	2	<hkxhk>	hh	10	hk	233	kk	2
>ref-27140	CAAGTATTTACAATCCCTCCAGAGAAT	6	<hkxhk>	hh	10	hk	234	kk	2
>ref-27145	CACCGCCCAACCGGCACTCCTCCCTCC	6	<lmxll>	ll	120	lm	137		
>ref-27148	CCTTTTTTCACCTCTTCTCCTCTTCCC	16	<hkxhk>	hh	2	hk	194	kk	64
>ref-27170	CTCTCAGCTACTTCAGCTCCCTCGACA	6	<hkxhk>	hh	11	hk	260	kk	0
>ref-27170	CTCTCAGCTACTTCAGCTCCCTCGACA	9	<hkxhk>	hh	0	hk	129	kk	132
>ref-27170	CTCTCAGCTACTTCAGCTCCCTCGACA	14	<nnxnp>	nn	217	np	48		
>ref-27170	CTCTCAGCTACTTCAGCTCCCTCGACA	24	<hkxhk>	hh	0	hk	269	kk	4
>ref-27180	GGCAGCTCGACGAGACCTCCCGCGAGG	9	<nnxnp>	nn	157	np	115		
>ref-27182	ACATCGTCCACCTGCACTCCATCCTCC	3	<lmxll>	ll	117	lm	147		
>ref-27197	TTGGTCATAACCCATCCTCCCTTACTA	25	<lmxll>	ll	252	lm	2		
>ref-27197	TTGGTCATAACCCATCCTCCCTTACTA	26	<lmxll>	ll	236	lm	4		
>ref-27219	TTCGGTGGCACTTTGGCTCCGACCCTC	23	<nnxnp>	nn	129	np	137		
>ref-27234	CACTTCAGTACTCTCCCTCCCCTCACC	23	<lmxll>	ll	134	lm	100		
>ref-27253	CAGGGTGTAACTCCATCTCCGCATCCT	5	<lmxll>	ll	121	lm	149		
>ref-27262	CAATGTCTTACTTCTCCTCCTCTCCTC	23	<nnxnp>	nn	146	np	121		
>ref-27283	AGAAATATAACAAGCCCTCCCTGACCA	2	<hkxhk>	hh	55	hk	130	kk	55
>ref-27318	CTCTTAGCCACAGGATCTCCCAGAGTG	6	<nnxnp>	nn	221	np	38		
>ref-27318	CTCTTAGCCACAGGATCTCCCAGAGTG	8	<hkxhk>	hh	0	hk	272	kk	0
>ref-27318	CTCTTAGCCACAGGATCTCCCAGAGTG	14	<hkxhk>	hh	0	hk	44	kk	211
>ref-27349	ATTCATCCTACGCCTCCTCCTCCTTTT	1	<nnxnp>	nn	93	np	136		
>ref-27349	ATTCATCCTACGCCTCCTCCTCCTTTT	5	<nnxnp>	nn	89	np	138		
>ref-27353	CTTTGCGCCACACTCCCTCCGACTCAT	14	<lmxll>	ll	146	lm	120		
>ref-27380	CAATCTTTGACTCCACCTCCTGGGGTC	16	<hkxhk>	hh	2	hk	270	kk	0

>ref-27380	CAATCTTTGACTCCACCTCCTGGGGTC	22	<hkxhk>	hh	2	hk	270	kk	0
>ref-27412	CCTTCCCTTACCCTACCTCCCTTCTAC	4	<lmxll>	ll	9	lm	253		
>ref-27415	CAGCCTCGTACCCGTACTCCTCACATC	1	<nnxnp>	nn	165	np	100		
>ref-27415	CAGCCTCGTACCCGTACTCCTCACATC	4	<nnxnp>	nn	166	np	98		
>ref-27415	CAGCCTCGTACCCGTACTCCTCACATC	14	<nnxnp>	nn	136	np	135		
>ref-27415	CAGCCTCGTACCCGTACTCCTCACATC	15	<nnxnp>	nn	136	np	135		
>ref-27426	CGCAGGCTCACTTCCACTCCAGACTAT	2	<lmxll>	ll	121	lm	124		
>ref-27432	TTGTCGTTAACTCTATCTCCCCTCTCT	16	<lmxll>	ll	109	lm	130		
>ref-27432	TTGTCGTTAACTCTATCTCCCCTCTCT	23	<nnxnp>	nn	257	np	0		
>ref-27433	CCTTCTCCTACCCCTTCTCCATCCCTC	1	<lmxll>	ll	160	lm	82		
>ref-27433	CCTTCTCCTACCCCTTCTCCATCCCTC	22	<hkxhk>	hh	0	hk	171	kk	53
>ref-27433	CCTTCTCCTACCCCTTCTCCATCCCTC	26	<lmxll>	ll	160	lm	83		
>ref-27441	CATTCACGCACTTGAACTCCCTTTCTC	13	<lmxll>	ll	8	lm	263		
>ref-27441	CATTCACGCACTTGAACTCCCTTTCTC	14	<lmxll>	ll	8	lm	263		
>ref-27442	CTGCTACCCACTCTCTCTCCCGTGTTC	21	<nnxnp>	nn	131	np	134		
>ref-27457	CTTGGTGTGACGCTGACTCCTTATCTA	25	<nnxnp>	nn	143	np	107		
>ref-27462	TCTCCTTTAACCCTTCCTCCCTGCTAT	8	<lmxll>	ll	134	lm	123		
>ref-27473	TAAGCACACACTCCGCCTCCTTTCCCA	5	<lmxll>	ll	132	lm	132		
>ref-27484	CTGGATACTACTCTCTCTCCCTCTCCC	24	<nnxnp>	nn	262	np	0		
>ref-27492	CCTCCTCATACTCTCTCTCCCCTTCTC	3	<nnxnp>	nn	158	np	103		
>ref-27492	CCTCCTCATACTCTCTCTCCCCTTCTC	22	<nnxnp>	nn	155	np	103		
>ref-27496	AGGAAAGAAACTTATCCTCCTCAAACG	16	<lmxll>	ll	8	lm	247		
>ref-27496	AGGAAAGAAACTTATCCTCCTCAAACG	21	<lmxll>	ll	19	lm	223		
>ref-27496	AGGAAAGAAACTTATCCTCCTCAAACG	27	<lmxll>	ll	8	lm	247		
>ref-27529	GGTCCCTCCACCGCCCCTCCTCCCCAT	25	<lmxll>	ll	129	lm	137		
>ref-27554	CCAACACTTACCTCTCCTCCCTTTCCT	1	<lmxll>	ll	128	lm	117		
>ref-27562	CTTTGGTTTACGTTATCTCCACCCGTC	14	<nnxnp>	nn	0	np	243		
>ref-27595	CACACAATTACTTATACTCCTTCGAGA	15	<nnxnp>	nn	143	np	126		
>ref-27600	GACAGTAAGACAAACCCTCCTCGACAC	7	<hkxhk>	hh	54	hk	150	kk	55
>ref-27608	ACATCCTACACGTCCCCTCCGTTTCCT	26	<lmxll>	ll	134	lm	126		
>ref-27617	TTATATGTCACTTCTCCTCCTGGTTTC	12	<nnxnp>	nn	97	np	122		
>ref-27617	TTATATGTCACTTCTCCTCCTGGTTTC	14	<nnxnp>	nn	97	np	122		
>ref-27622	TCCTTTCTTACCGGCTCTCCACCTTAT	1	<hkxhk>	hh	51	hk	150	kk	60
>ref-27630	ACCCCACCCACGCACTCTCCCTTGCTT	14	<lmxll>	ll	100	lm	162		
>ref-27630	ACCCCACCCACGCACTCTCCCTTGCTT	24	<hkxhk>	hh	0	hk	137	kk	133
>ref-27661	ACTCTCCTCACTCACTCTCCTTACTTA	15	<lmxll>	ll	120	lm	144		
>ref-27666	ACTGACCCGACTTGACCTCCCTTTTCA	26	<nnxnp>	nn	123	np	127		
>ref-27668	TTTCTTCCCACTCTTTCTCCAGTCTCC	26	<lmxll>	ll	109	lm	142		

>ref-27674	AGTCCTCCCACAGCCCCTCCCTCAGTC	1	<hkxhk>	hh	31	hk	237	kk	0
>ref-27680	CTCCCATTCACCACATCTCCTCCCACT	13	<nnxnp>	nn	104	np	134		
>ref-27680	CTCCCATTCACCACATCTCCTCCCACT	15	<nnxnp>	nn	105	np	138		
>ref-27682	ACTGTTATGACCGCTCCTCCACCTTCT	1	<nnxnp>	nn	124	np	143		
>ref-27747	CGGCTTTCTACAATCACTCCCACCGAC	26	<hkxhk>	hh	20	hk	238	kk	0
>ref-27752	AAAAGGACTACAGTTTCTCCAAATCCG	6	<lmxll>	ll	132	lm	134		
>ref-27755	TCATCTACAACTGTATCTCCATCTACA	4	<nnxnp>	nn	259	np	0		
>ref-27787	AGGATGTGTACATGTACTCCAGGTATG	21	<lmxll>	ll	144	lm	126		
>ref-27799	GGGAATGGAACGTTATCTCCGTTGATG	4	<lmxll>	ll	122	lm	146		
>ref-27804	TTTTCAAATACACAACCTCCCATGCGT	21	<lmxll>	ll	97	lm	151		
>ref-27810	AACATACATACCCTCTCTCCCTCGCCC	1	<hkxhk>	hh	4	hk	266	kk	0
>ref-27810	AACATACATACCCTCTCTCCCTCGCCC	4	<hkxhk>	hh	4	hk	266	kk	0
>ref-27812	TGACAATGCACAGTTTCTCCAGAGCCA	9	<nnxnp>	nn	272	np	0		
>ref-27842	TCCCCTCCTACTCTGCCTCCCATTCTT	21	<lmxll>	ll	124	lm	121		
>ref-27844	CCCCCACCCACATCCTCTCCATCAACA	1	<hkxhk>	hh	1	hk	267	kk	2
>ref-27900	TCTTTACCGACTCCGACTCCGACTCCA	9	<hkxhk>	hh	0	hk	9	kk	233
>ref-27900	TCTTTACCGACTCCGACTCCGACTCCA	13	<lmxll>	ll	240	lm	6		
>ref-27900	TCTTTACCGACTCCGACTCCGACTCCA	14	<lmxll>	ll	243	lm	2		
>ref-27900	TCTTTACCGACTCCGACTCCGACTCCA	15	<nnxnp>	nn	246	np	1		
>ref-27900	TCTTTACCGACTCCGACTCCGACTCCA	21	<hkxhk>	hh	0	hk	10	kk	236
>ref-27920	GGTAGCACCACTTCATCTCCATGAAAG	13	<nnxnp>	nn	14	np	249		
>ref-27920	GGTAGCACCACTTCATCTCCATGAAAG	25	<nnxnp>	nn	14	np	249		
>ref-27922	GCCCCTCCTACACTTCCTCCCTCCCCT	1	<lmxll>	ll	123	lm	123		
>ref-27922	GCCCCTCCTACACTTCCTCCCTCCCCT	12	<lmxll>	ll	124	lm	120		
>ref-27928	TACTATCACACTATCTCTCCATTCTAA	2	<hkxhk>	hh	0	hk	222	kk	39
>ref-27928	TACTATCACACTATCTCTCCATTCTAA	6	<nnxnp>	nn	135	np	128		
>ref-27973	TACGCTTGTACACCTCCTCCACTCACA	6	<nnxnp>	nn	119	np	146		
>ref-27981	GGGGAGCGCACTTTGGCTCCGTCAGTG	4	<nnxnp>	nn	134	np	135		
>ref-27981	GGGGAGCGCACTTTGGCTCCGTCAGTG	21	<nnxnp>	nn	134	np	135		
>ref-28010	CTCATTCTCACTTCATCTCCAACTTTC	12	<lmxll>	ll	129	lm	129		
>ref-28024	TCTTCTTCTACCTTTTCTCCTTTCTCA	24	<lmxll>	ll	66	lm	182		
>ref-28056	CATAGTGCAACAGGAACTCCGGGCGCT	27	<hkxhk>	hh	60	hk	137	kk	57
>ref-28078	TATCTTTATACTGCCTCTCCCTTCCCC	12	<lmxll>	ll	116	lm	138		
>ref-28079	ACAGGAGTCACATTCACTCCCTCATAT	24	<lmxll>	ll	121	lm	144		
>ref-28082	CCACTTTCCACACCCCCTCCCCCGTCC	4	<lmxll>	ll	121	lm	127		
>ref-28090	CCTGTTCCAACTGTCTCTCCTAGTTGT	7	<hkxhk>	hh	52	hk	118	kk	66
>ref-28108	TCCATCTTCACCACTTCTCCCTTCCAT	8	<hkxhk>	hh	0	hk	262	kk	4
>ref-28108	TCCATCTTCACCACTTCTCCCTTCCAT	22	<nnxnp>	nn	17	np	248		

>ref-28117	TGATCACCCACTACAGCTCCTTCGCTT	23	<hkxhk>	hh	51	hk	148	kk	57
>ref-28148	GTGTTGGATACCGTGTCTCCTGGCGAC	2	<nnxnp>	nn	116	np	139		
>ref-28190	ATAACAAGGACATTGTCTCCCCCGCCA	23	<lmxll>	ll	125	lm	133		
>ref-28199	CTCCTCCACACTGCCTCTCCCCTTCCT	4	<hkxhk>	hh	49	hk	142	kk	63
>ref-28212	CCACGTTTTACCCTTTCTCCTCTTCCT	12	<nnxnp>	nn	264	np	0		
>ref-28212	CCACGTTTTACCCTTTCTCCTCTTCCT	27	<nnxnp>	nn	266	np	0		
>ref-28214	ATAACGTTCACGGAAACTCCTTTCTTT	4	<nnxnp>	nn	131	np	119		
>ref-28220	CCGCCGCTGACTCGCTCTCCGTCAGGC	6	<nnxnp>	nn	270	np	0		
>ref-28246	ATGAGACGCACGGAGCCTCCAATTATG	3	<lmxll>	ll	126	lm	131		
>ref-28254	ACTCGACCCACACATCCTCCTCTTAAA	3	<hkxhk>	hh	0	hk	272	kk	0
>ref-28255	CACATAGACACACATTCTCCCACGTAG	2	<lmxll>	ll	154	lm	105		
>ref-28264	CCTCTTCCTACTTCTTCTCCATCCCTT	3	<hkxhk>	hh	27	hk	176	kk	39
>ref-28264	CCTCTTCCTACTTCTTCTCCATCCCTT	4	<hkxhk>	hh	39	hk	176	kk	27
>ref-28296	TCCTCCTATACCTTTACTCCTCCTCTC	7	<nnxnp>	nn	117	np	130		
>ref-28300	TCCCCCTCCACCTGTGCTCCTCCCTTC	15	<lmxll>	ll	109	lm	152		
>ref-28312	GTCTTTTCTACCCTCTCTCCCTTCTTT	5	<hkxhk>	hh	10	hk	247	kk	3
>ref-28323	GATAAGTCCACGTGACCTCCAATTGCT	12	<nnxnp>	nn	129	np	142		
>ref-28342	GCTCCCGGCACATGCCCTCCTTGGAAG	13	<nnxnp>	nn	103	np	121		
>ref-28350	TTCTGCGCCACAATGACTCCCCAAGGT	7	<lmxll>	ll	126	lm	144		
>ref-28378	TACCTTTACACACCTCCTCCTTACATT	3	<hkxhk>	hh	55	hk	147	kk	56
>ref-28382	AGATCAATCACTTTAACTCCTCTCTCT	1	<nnxnp>	nn	128	np	93		
>ref-28421	ATAGTATATACTACCTCTCCTTTTCCG	5	<hkxhk>	hh	11	hk	202	kk	6
>ref-28423	CGGATGCCAACGCTTGCTCCAGTCATT	6	<nnxnp>	nn	138	np	133		
>ref-28426	TATGAACAAACAAACGCTCCACATTTC	4	<lmxll>	ll	123	lm	113		
>ref-28449	TCTCTTATCACGTGCCCTCCCTCCCCC	8	<lmxll>	ll	141	lm	124		
>ref-28451	GGTGTCGCGACGTCTGCTCCGCCTTCT	25	<nnxnp>	nn	108	np	111		
>ref-28474	CTCCTCATCACAAAGCCTCCTTCTTCG	1	<nnxnp>	nn	124	np	128		
>ref-28484	CTCCTCAAGACCTCCTCTCCCTCTCTG	27	<lmxll>	ll	139	lm	112		
>ref-28502	AGCTGCACGACTCACGCTCCCTCGTCG	23	<nnxnp>	nn	123	np	145		
>ref-28540	CTATTCCCCACTCATCCTCCTTACACT	22	<nnxnp>	nn	143	np	121		
>ref-28552	CATCGATCTACCCTATCTCCCTGTTTC	5	<nnxnp>	nn	111	np	136		
>ref-28555	AGGGTCACCACAACGGCTCCAGGGGTA	9	<lmxll>	ll	150	lm	116		
>ref-28556	TTCAACTCTACTCACTCTCCCGCCATA	22	<nnxnp>	nn	140	np	129		
>ref-28558	TCACCATGGACTTACTCTCCATACAGT	3	<hkxhk>	hh	64	hk	201	kk	0
>ref-28558	TCACCATGGACTTACTCTCCATACAGT	15	<hkxhk>	hh	0	hk	261	kk	10
>ref-28558	TCACCATGGACTTACTCTCCATACAGT	27	<hkxhk>	hh	3	hk	269	kk	0
>ref-28570	TGTGTGTGTACACACACTCCTATGTAT	13	<hkxhk>	hh	3	hk	261	kk	0
>ref-28570	TGTGTGTGTACACACACTCCTATGTAT	24	<hkxhk>	hh	0	hk	261	kk	3

>ref-28572	CTCCCCTCTACTCCACCTCCACTCTCT	26	<nnxnp>	nn	153	np	98		
>ref-28601	CTTACCTTCACAGCTTCTCCCGATCAT	4	<hkxhk>	hh	50	hk	139	kk	63
>ref-28607	GCCAACCCCACTCCTTCTCCGAGATGT	9	<lmxll>	ll	117	lm	142		
>ref-28613	CCCCTCCTCACTAACTCTCCCTCTCTT	3	<lmxll>	ll	272	lm	0		
>ref-28649	TCGCTGAGAACACGCCCTCCTTTCCCT	12	<lmxll>	ll	127	lm	128		
>ref-28649	TCGCTGAGAACACGCCCTCCTTTCCCT	15	<lmxll>	ll	265	lm	0		
>ref-28649	TCGCTGAGAACACGCCCTCCTTTCCCT	21	<lmxll>	ll	127	lm	128		
>ref-28649	TCGCTGAGAACACGCCCTCCTTTCCCT	27	<lmxll>	ll	256	lm	1		
>ref-28660	TTCTCCTCCACCTAACCTCCTTCCACC	1	<lmxll>	ll	65	lm	188		
>ref-28660	TTCTCCTCCACCTAACCTCCTTCCACC	14	<nnxnp>	nn	267	np	1		
>ref-28660	TTCTCCTCCACCTAACCTCCTTCCACC	21	<nnxnp>	nn	266	np	1		
>ref-28666	CCCGCTCCCACTCCGCCTCCCCCGCCC	1	<lmxll>	ll	104	lm	140		
>ref-28666	CCCGCTCCCACTCCGCCTCCCCCGCCC	12	<hkxhk>	hh	2	hk	178	kk	58
>ref-28666	CCCGCTCCCACTCCGCCTCCCCCGCCC	15	<lmxll>	ll	80	lm	167		
>ref-28669	CCTTCCCCCACCTCCTCTCCTACCCCT	2	<nnxnp>	nn	259	np	0		
>ref-28669	CCTTCCCCCACCTCCTCTCCTACCCCT	12	<lmxll>	ll	106	lm	130		
>ref-28669	CCTTCCCCCACCTCCTCTCCTACCCCT	13	<lmxll>	ll	101	lm	134		
>ref-28683	ATCACCATTACATCCTCTCCACCACCA	14	<lmxll>	ll	118	lm	142		
>ref-28683	ATCACCATTACATCCTCTCCACCACCA	15	<lmxll>	ll	119	lm	142		
>ref-28685	AGTTCACAGACGGCCTCTCCGACTGAG	23	<hkxhk>	hh	0	hk	273	kk	0
>ref-28718	AGAGACAAAACAGACTCTCCCCAATAG	25	<nnxnp>	nn	123	np	134		
>ref-28720	TACAGAAAAACGGTTTCTCCAGTATTG	3	<lmxll>	ll	37	lm	185		
>ref-28720	TACAGAAAAACGGTTTCTCCAGTATTG	5	<lmxll>	ll	37	lm	185		
>ref-28729	AGCCCTCGTACTCAGCCTCCTGTACTT	9	<nnxnp>	nn	264	np	0		
>ref-28759	TTCCTGCTAACTCCCTCTCCCTCTTCC	12	<nnxnp>	nn	5	np	264		
>ref-28759	TTCCTGCTAACTCCCTCTCCCTCTTCC	13	<nnxnp>	nn	5	np	264		
>ref-28778	TGACTGTACACCGCCCCTCCCGTACCT	2	<lmxll>	ll	130	lm	134		
>ref-28808	CTCTTTTCCACACCCCCTCCCTCCCCT	6	<hkxhk>	hh	1	hk	251	kk	5
>ref-28821	TCAAATATGACAGGTTCTCCAACATAT	13	<hkxhk>	hh	1	hk	271	kk	0
>ref-28821	TCAAATATGACAGGTTCTCCAACATAT	23	<lmxll>	ll	41	lm	214		
>ref-28829	GGAATTCTCACGGGGACTCCTCTATAC	12	<lmxll>	ll	112	lm	127		
>ref-28829	GGAATTCTCACGGGGACTCCTCTATAC	15	<nnxnp>	nn	130	np	112		
>ref-28841	TAAAATTGCACGGCGTCTCCTTCGTCA	9	<lmxll>	ll	251	lm	0		
>ref-28846	CACCCTCAAACACTTTCTCCCAATTTA	25	<lmxll>	ll	123	lm	122		
>ref-28849	CCTTTCCCTACTTTGCCTCCCTATCAT	16	<nnxnp>	nn	104	np	147		
>ref-28854	AAACCTGTCACTTCATCTCCCTCTTCT	22	<lmxll>	ll	135	lm	134		
>ref-28871	ACCCATACAACGACCACTCCCTCCCCA	23	<hkxhk>	hh	75	hk	194	kk	0
>ref-28871	ACCCATACAACGACCACTCCCTCCCCA	27	<hkxhk>	hh	0	hk	262	kk	5

>ref-28889	TCTTCCCCTACTCTTCCTCCTCTCATC	5	<nnxnp>	nn	128	np	141		
>ref-28907	TGTTTGCGGACATCTCCTCCTTTGGCT	25	<lmxll>	ll	128	lm	141		
>ref-28926	TGTTAGCTTACCTGCCCTCCTGTATGG	6	<hkxhk>	hh	0	hk	106	kk	149
>ref-28926	TGTTAGCTTACCTGCCCTCCTGTATGG	8	<hkxhk>	hh	0	hk	273	kk	0
>ref-28926	TGTTAGCTTACCTGCCCTCCTGTATGG	15	<lmxll>	ll	207	lm	54		
>ref-28926	TGTTAGCTTACCTGCCCTCCTGTATGG	24	<hkxhk>	hh	0	hk	273	kk	0
>ref-28934	ACAAGTACTACTTCAGCTCCCACAACG	21	<lmxll>	ll	4	lm	252		
>ref-28934	ACAAGTACTACTTCAGCTCCCACAACG	24	<lmxll>	ll	4	lm	251		
>ref-28961	CCCTCTCTTACTACCTCTCCTTCCTCC	23	<lmxll>	ll	102	lm	146		
>ref-28962	TCCTCCCCCACTTACCCTCCACGTCCT	25	<lmxll>	ll	126	lm	143		
>ref-28973	TGCCTACGCACACAACCTCCCCCCACA	23	<hkxhk>	hh	0	hk	271	kk	1
>ref-28995	GTATTCCCGACCTTCTCTCCCACATCC	22	<nnxnp>	nn	249	np	1		
>ref-29002	CACAAATACACCAACACTCCGTCACAT	2	<lmxll>	ll	85	lm	154		
>ref-29016	GCTTTCCTCACAGGCTCTCCATCCCCG	12	<nnxnp>	nn	120	np	121		
>ref-29018	TAATCATCGACCGACTCTCCATTCCCT	5	<lmxll>	ll	126	lm	137		
>ref-29018	TAATCATCGACCGACTCTCCATTCCCT	7	<lmxll>	ll	126	lm	137		
>ref-29026	TATCCCTGAACAGCGTCTCCGTTATAA	2	<hkxhk>	hh	53	hk	164	kk	46
>ref-29028	TCTACAGCCACTATGTCTCCAGCAACA	22	<hkxhk>	hh	54	hk	151	kk	56
>ref-29033	GTCTTTCTCACACTTCCTCCCTCTCTT	25	<nnxnp>	nn	133	np	116		
>ref-29042	TTTCCTTTCACTTTCCCTCCTTCGTAA	27	<lmxll>	ll	88	lm	163		
>ref-29044	GGGCTTGGTACAGAGGCTCCTGTGTGA	6	<hkxhk>	hh	64	hk	138	kk	61
>ref-29071	TTTATTCATACATTAACTCCAGAAGCG	23	<hkxhk>	hh	42	hk	189	kk	0
>ref-29079	ACCACGGCGACTTCCCCTCCGACACCT	1	<hkxhk>	hh	121	hk	145	kk	0
>ref-29079	ACCACGGCGACTTCCCCTCCGACACCT	3	<hkxhk>	hh	0	hk	264	kk	2
>ref-29079	ACCACGGCGACTTCCCCTCCGACACCT	6	<hkxhk>	hh	0	hk	264	kk	2
>ref-29079	ACCACGGCGACTTCCCCTCCGACACCT	15	<hkxhk>	hh	107	hk	155	kk	0
>ref-29087	CGCCTCGACACACATTCTCCGCTGGAG	27	<hkxhk>	hh	0	hk	272	kk	1
>ref-29091	CCCTTTTCCACATCTCCTCCTCCTCCC	12	<lmxll>	ll	114	lm	108		
>ref-29121	TATAATCTTACCTTCCCTCCATTCCTT	4	<nnxnp>	nn	110	np	109		
>ref-29131	CCATGCGGGACAGGCTCTCCAGGTGCA	8	<nnxnp>	nn	120	np	144		
>ref-29152	TGCTATAAGACTGATGCTCCGAAAATA	13	<hkxhk>	hh	56	hk	128	kk	47
>ref-29156	CAACCAGGAACCTCAGCTCCTTCACCC	8	<lmxll>	ll	136	lm	127		
>ref-29162	CATCATGCTACACAGACTCCCACTGGA	8	<nnxnp>	nn	142	np	126		
>ref-29167	CCTTTTCTAACACCCACTCCGGCGCAT	26	<lmxll>	ll	129	lm	108		
>ref-29195	GTTGTTGCTACTGGCACTCCGGTCTCT	1	<lmxll>	ll	139	lm	130		
>ref-29196	CCAAACTCCACCTTCACTCCTTCCACT	5	<lmxll>	ll	116	lm	145		
>ref-29198	GTACCTACTACAGCGCCTCCATCAGTG	5	<hkxhk>	hh	0	hk	273	kk	0
>ref-29198	GTACCTACTACAGCGCCTCCATCAGTG	7	<hkxhk>	hh	0	hk	273	kk	0

>ref-29204	TACCAGCCGACACCAACTCCACATATA	2	<nnxnp>	nn	263	np	0		
>ref-29227	TTTAAACCTACATCTGCTCCCTCTCAC	5	<lmxll>	ll	128	lm	125		
>ref-29227	TTTAAACCTACATCTGCTCCCTCTCAC	12	<lmxll>	ll	129	lm	125		
>ref-29230	CCCCACGCAACCTCTTCTCCTCCTTCC	7	<nnxnp>	nn	139	np	121		
>ref-29242	GCATCAGTCACCTCTGCTCCGCTGTCG	16	<nnxnp>	nn	139	np	124		
>ref-29260	TAGCCTTTTACAATCTCTCCATCTGTA	6	<nnxnp>	nn	129	np	130		
>ref-29260	TAGCCTTTTACAATCTCTCCATCTGTA	27	<lmxll>	ll	123	lm	136		
>ref-29275	TCCGTTGCTACCTACTCTCCCACTTCT	14	<nnxnp>	nn	137	np	132		
>ref-29307	TTTGGGGCTACATCTTCTCCAAGTGTA	9	<lmxll>	ll	267	lm	3		
>ref-29347	AGAAGTAAAACCAAAACTCCCCTCTTT	6	<hkxhk>	hh	1	hk	102	kk	131
>ref-29347	AGAAGTAAAACCAAAACTCCCCTCTTT	12	<lmxll>	ll	206	lm	33		
>ref-29347	AGAAGTAAAACCAAAACTCCCCTCTTT	21	<hkxhk>	hh	8	hk	247	kk	6
>ref-29348	ACCACGTTTACTTCCTCTCCCCTTCCT	6	<hkxhk>	hh	53	hk	142	kk	55
>ref-29355	GTTTGTCGCACAAACTCTCCATGCAGT	3	<lmxll>	ll	222	lm	38		
>ref-29355	GTTTGTCGCACAAACTCTCCATGCAGT	13	<nnxnp>	nn	238	np	31		
>ref-29355	GTTTGTCGCACAAACTCTCCATGCAGT	27	<lmxll>	ll	218	lm	41		
>ref-29394	TGCTGCCTGACTTCTGCTCCCGTGATC	21	<hkxhk>	hh	0	hk	269	kk	2
>ref-29396	TTCCCCTCCACTCCATCTCCTATCGAA	8	<hkxhk>	hh	60	hk	113	kk	59
>ref-29399	GACGTCCAAACACACCCTCCGTTTATA	8	<lmxll>	ll	125	lm	127		
>ref-29410	CCGTTCTCTACCAATTCTCCTCCTTCT	23	<lmxll>	ll	120	lm	123		
>ref-29416	ATTCTCATAACTTTCGCTCCTTCTCTT	22	<hkxhk>	hh	60	hk	145	kk	53
>ref-29432	CAACAAGCGACCCTGCCTCCCTCCCAT	1	<nnxnp>	nn	121	np	149		
>ref-29435	CACAGAGCCACACATCCTCCCGTCCAG	6	<hkxhk>	hh	68	hk	139	kk	46
>ref-29457	GCCCGGCCTACGCAGCCTCCTCCCTCT	25	<nnxnp>	nn	130	np	142		
>ref-29476	TGAGACTTCACGATGGCTCCCCCTAAG	1	<hkxhk>	hh	59	hk	140	kk	66
>ref-29476	TGAGACTTCACGATGGCTCCCCCTAAG	7	<hkxhk>	hh	59	hk	140	kk	66
>ref-29499	TTCCCCCCTACGTCTTCTCCTCCTCTC	12	<hkxhk>	hh	4	hk	263	kk	1
>ref-29499	TTCCCCCCTACGTCTTCTCCTCCTCTC	15	<hkxhk>	hh	1	hk	263	kk	4
>ref-29502	TTCTCTCTAACCCCTTCTCCCTCCCTT	2	<nnxnp>	nn	70	np	196		
>ref-29502	TTCTCTCTAACCCCTTCTCCCTCCCTT	13	<nnxnp>	nn	20	np	235		
>ref-29502	TTCTCTCTAACCCCTTCTCCCTCCCTT	16	<nnxnp>	nn	67	np	195		
>ref-29504	CACACACACACACGGGCTCCCCTCGTT	1	<lmxll>	ll	114	lm	148		
>ref-29515	TCTTTTCCCACATTCTCTCCCTCATTC	7	<hkxhk>	hh	14	hk	250	kk	0
>ref-29515	TCTTTTCCCACATTCTCTCCCTCATTC	12	<hkxhk>	hh	13	hk	250	kk	0
>ref-29520	TCCCTTCCTACGAACTCTCCCCCCTTT	6	<nnxnp>	nn	130	np	123		
>ref-29522	TAAAACCACACACACACTCCAAGCAAA	6	<nnxnp>	nn	100	np	124		
>ref-29524	GCTAAAGGTACGAGTTCTCCCTTACTT	12	<nnxnp>	nn	124	np	101		
>ref-29530	CTTTCACTCACTCCTTCTCCATCTATC	1	<lmxll>	ll	102	lm	150		

>ref-29530	CTTTCACTCACTCCTTCTCCATCTATC	15	<nnxnp>	nn	132	np	119		
>ref-29535	TCCATCTAGACCTTCCCTCCATCTAGA	3	<hkxhk>	hh	10	hk	227	kk	0
>ref-29535	TCCATCTAGACCTTCCCTCCATCTAGA	12	<hkxhk>	hh	9	hk	226	kk	0
>ref-29542	TTTTTATCCACCCTTTCTCCCTTTCCC	1	<nnxnp>	nn	269	np	1		
>ref-29542	TTTTTATCCACCCTTTCTCCCTTTCCC	2	<nnxnp>	nn	217	np	42		
>ref-29542	TTTTTATCCACCCTTTCTCCCTTTCCC	6	<nnxnp>	nn	268	np	1		
>ref-29542	TTTTTATCCACCCTTTCTCCCTTTCCC	24	<lmxll>	ll	134	lm	128		
>ref-29542	TTTTTATCCACCCTTTCTCCCTTTCCC	26	<lmxll>	ll	132	lm	129		
>ref-29569	GAAATCACAACTCTGGCTCCTTCCACC	15	<hkxhk>	hh	4	hk	268	kk	0
>ref-29575	CCTAAGGAAACATTCCCTCCTCGCTTG	2	<nnxnp>	nn	114	np	132		
>ref-29575	CCTAAGGAAACATTCCCTCCTCGCTTG	25	<nnxnp>	nn	119	np	127		
>ref-29576	ATTATGTAAACATTAACTCCCATGATA	22	<lmxll>	ll	98	lm	136		
>ref-29612	TTCTAAACCACCCATCCTCCTCCTTCT	15	<nnxnp>	nn	133	np	129		
>ref-29619	TTTCTCCTCACCTCTCCTCCCTCCGTC	4	<lmxll>	ll	32	lm	209		
>ref-29619	TTTCTCCTCACCTCTCCTCCCTCCGTC	25	<lmxll>	ll	25	lm	214		
>ref-29642	ACATGCAGGACACAATCTCCTACAGTA	2	<nnxnp>	nn	123	np	137		
>ref-29646	GTGTCTGTGACTCGTTCTCCGCAAATG	7	<lmxll>	ll	132	lm	135		
>ref-29653	TCAAATTAAACATCATCTCCAATCTCT	6	<nnxnp>	nn	113	np	112		
>ref-29653	TCAAATTAAACATCATCTCCAATCTCT	8	<nnxnp>	nn	114	np	111		
>ref-29678	TTTGTTACTACTCTCTCTCCCTCTCCC	14	<nnxnp>	nn	137	np	107		
>ref-29693	AGATCACTAACCTCTCCTCCACGTGTT	1	<hkxhk>	hh	49	hk	129	kk	76
>ref-29693	AGATCACTAACCTCTCCTCCACGTGTT	4	<nnxnp>	nn	140	np	123		
>ref-29718	GCCTGATAAACAGCATCTCCTAAAATT	6	<nnxnp>	nn	118	np	108		
>ref-29719	TTACCCCTCACCTCTCCTCCTTTCCCC	13	<lmxll>	ll	265	lm	1		
>ref-29739	TCGTCTTGTACGTCTTCTCCCTCCCTT	7	<hkxhk>	hh	45	hk	150	kk	61
>ref-29742	GGGGAATTAACTGTCTCTCCCCTATAG	15	<lmxll>	ll	16	lm	240		
>ref-29742	GGGGAATTAACTGTCTCTCCCCTATAG	27	<lmxll>	ll	152	lm	92		
>ref-29754	ACGTTTCACACGCATCCTCCCCTATAT	1	<hkxhk>	hh	59	hk	139	kk	39
>ref-29772	CCTTAAGACACAACAGCTCCCTTTTCT	4	<nnxnp>	nn	107	np	136		
>ref-29782	GGTGCAGGTACCTTCTCTCCCACACCT	1	<hkxhk>	hh	0	hk	191	kk	70
>ref-29782	GGTGCAGGTACCTTCTCTCCCACACCT	12	<nnxnp>	nn	234	np	35		
>ref-29782	GGTGCAGGTACCTTCTCTCCCACACCT	22	<hkxhk>	hh	0	hk	273	kk	0
>ref-29782	GGTGCAGGTACCTTCTCTCCCACACCT	25	<lmxll>	ll	193	lm	73		
>ref-29782	GGTGCAGGTACCTTCTCTCCCACACCT	26	<hkxhk>	hh	0	hk	62	kk	197
>ref-29797	CTCTCTTGCACTCTGCCTCCCTCTAAC	8	<lmxll>	ll	20	lm	200		
>ref-29797	CTCTCTTGCACTCTGCCTCCCTCTAAC	15	<lmxll>	ll	20	lm	201		
>ref-29809	CCTCCTTTCACCCCTCCTCCAACTCCC	15	<lmxll>	ll	140	lm	90		
>ref-29819	TCTCTGTCTACTTCCCCTCCTCCATTT	6	<hkxhk>	hh	45	hk	150	kk	63

>ref-29819	TCTCTGTCTACTTCCCCTCCTCCATTT	23	<hkxhk>	hh	63	hk	149	kk	45
>ref-29822	TGTAAAGCCACACGTGCTCCGAGACTT	15	<lmxll>	ll	128	lm	139		
>ref-29857	CATCCCATCACTGCTACTCCTTTTACT	2	<nnxnp>	nn	107	np	115		
>ref-29891	CCTCCTCTTACCGCTCCTCCCACCACT	26	<nnxnp>	nn	247	np	0		
>ref-29895	TTGCGAGACACCTTCGCTCCGTCTTTT	16	<hkxhk>	hh	56	hk	145	kk	53
>ref-29905	CTCCCTCAAACTCTATCTCCTTCACCC	22	<lmxll>	ll	143	lm	123		
>ref-29916	CCTTCCTCCACCCATCCTCCATCAACC	3	<hkxhk>	hh	0	hk	267	kk	3
>ref-29918	TCTTCCCTCACCCATTCTCCTTTAATA	25	<lmxll>	ll	122	lm	112		
>ref-29919	AGGGCCTCCACGCTGGCTCCTCTGTGG	27	<lmxll>	ll	120	lm	141		
>ref-29942	AACAAACAGACTTACTCTCCTCTCTCT	9	<nnxnp>	nn	234	np	0		
>ref-29942	AACAAACAGACTTACTCTCCTCTCTCT	13	<nnxnp>	nn	236	np	1		
>ref-29962	GCAGCTGTCACAGAGCCTCCACTCAAA	12	<hkxhk>	hh	40	hk	139	kk	57
>ref-29962	GCAGCTGTCACAGAGCCTCCACTCAAA	24	<hkxhk>	hh	40	hk	139	kk	58
>ref-29966	CTGAACACTACAACTACTCCCTCCTCA	2	<hkxhk>	hh	0	hk	272	kk	1
>ref-29966	CTGAACACTACAACTACTCCCTCCTCA	12	<hkxhk>	hh	4	hk	263	kk	0
>ref-29966	CTGAACACTACAACTACTCCCTCCTCA	21	<hkxhk>	hh	4	hk	263	kk	0
>ref-29977	CCACCCCACACCCACACTCCTACACTC	3	<lmxll>	ll	78	lm	151		
>ref-29993	ATTAATCCTACCCTCTCTCCCCTTCTC	24	<hkxhk>	hh	55	hk	144	kk	45
>ref-29993	ATTAATCCTACCCTCTCTCCCCTTCTC	25	<hkxhk>	hh	44	hk	145	kk	54
>ref-30003	CAGAAAAACACCTCATCTCCCAAACCA	13	<nnxnp>	nn	104	np	135		
>ref-30003	CAGAAAAACACCTCATCTCCCAAACCA	24	<nnxnp>	nn	104	np	135		
>ref-30004	CCGGCTTACACGGAATCTCCAAAAGCG	12	<lmxll>	ll	125	lm	116		
>ref-30013	TATCCAGCCACTCCCCCTCCCCCTCAT	2	<lmxll>	ll	76	lm	147		
>ref-30013	TATCCAGCCACTCCCCCTCCCCCTCAT	7	<lmxll>	ll	78	lm	147		
>ref-30034	CTGATCACGACAACCCCTCCCATGGCC	2	<nnxnp>	nn	264	np	4		
>ref-30034	CTGATCACGACAACCCCTCCCATGGCC	16	<nnxnp>	nn	264	np	5		
>ref-30041	TTACCCCTTACCCCTTCTCCCTTCCCC	1	<hkxhk>	hh	0	hk	202	kk	60
>ref-30041	TTACCCCTTACCCCTTCTCCCTTCCCC	9	<lmxll>	ll	132	lm	138		
>ref-30041	TTACCCCTTACCCCTTCTCCCTTCCCC	15	<nnxnp>	nn	147	np	107		
>ref-30041	TTACCCCTTACCCCTTCTCCCTTCCCC	23	<hkxhk>	hh	0	hk	141	kk	111
>ref-30041	TTACCCCTTACCCCTTCTCCCTTCCCC	24	<nnxnp>	nn	147	np	109		
>ref-30041	TTACCCCTTACCCCTTCTCCCTTCCCC	25	<lmxll>	ll	45	lm	214		
>ref-30044	TGTATGGGAACAATCCCTCCCTACATT	8	<nnxnp>	nn	141	np	129		
>ref-30054	GCGTTGGGCACTCGCACTCCAGTGTTA	5	<hkxhk>	hh	0	hk	11	kk	262
>ref-30054	GCGTTGGGCACTCGCACTCCAGTGTTA	24	<hkxhk>	hh	0	hk	11	kk	262
>ref-30122	TGCCACAACACTAACCCTCCCAACGTG	5	<nnxnp>	nn	126	np	142		
>ref-30122	TGCCACAACACTAACCCTCCCAACGTG	16	<nnxnp>	nn	125	np	143		
>ref-30146	CAATCTCCTACCTTCACTCCTTCATTC	12	<lmxll>	ll	121	lm	129		

>ref-30150	GCACTCACCACACGACCTCCCTACACA	24	<nnxnp>	nn	128	np	138		
>ref-30164	AGGAAGGTTACCTAGCCTCCTTTCCCC	14	<nnxnp>	nn	114	np	144		
>ref-30182	CCCTCCATCACCCACCCTCCACCCCTC	13	<lmxll>	ll	145	lm	74		
>ref-30191	GTGCTTATGACTTCAGCTCCGTAGTGT	25	<nnxnp>	nn	125	np	132		
>ref-30196	TTCCCCTACACCTTCTCTCCCCTTAAC	3	<nnxnp>	nn	3	np	233		
>ref-30196	TTCCCCTACACCTTCTCTCCCCTTAAC	8	<nnxnp>	nn	5	np	254		
>ref-30204	GAGACGTACACATACGCTCCGCAGTAA	1	<hkxhk>	hh	78	hk	141	kk	40
>ref-30231	CACACGCCCACACGCCCTCCACACGCC	27	<nnxnp>	nn	268	np	0		
>ref-30237	TAATATATAACCTCATCTCCAACATCT	6	<nnxnp>	nn	109	np	112		
>ref-30265	TCCTTTCTCACTTCCTCTCCTTCCGCT	23	<nnxnp>	nn	133	np	128		
>ref-30276	AAGCCAATGACCACGCCTCCTTTAGTT	6	<hkxhk>	hh	66	hk	206	kk	0
>ref-30276	AAGCCAATGACCACGCCTCCTTTAGTT	7	<hkxhk>	hh	30	hk	242	kk	0
>ref-30284	CGCGTATGCACGTCCCCTCCTGTCATG	14	<nnxnp>	nn	135	np	137		
>ref-30297	CTCCTTCTTACTCCTCCTCCCCTCGCC	8	<lmxll>	ll	166	lm	105		
>ref-30302	TGTCTCTCAACATTGTCTCCGTGTCCC	27	<lmxll>	ll	153	lm	112		
>ref-30307	CCTCACCCTACCACCTCTCCCTCTCTT	1	<hkxhk>	hh	10	hk	261	kk	0
>ref-30307	CCTCACCCTACCACCTCTCCCTCTCTT	4	<nnxnp>	nn	234	np	30		
>ref-30307	CCTCACCCTACCACCTCTCCCTCTCTT	27	<nnxnp>	nn	227	np	32		
>ref-30316	CCCTTCTCCACCCCATCTCCTTCCCTC	4	<nnxnp>	nn	170	np	81		
>ref-30316	CCCTTCTCCACCCCATCTCCTTCCCTC	15	<lmxll>	ll	6	lm	223		
>ref-30316	CCCTTCTCCACCCCATCTCCTTCCCTC	16	<nnxnp>	nn	169	np	83		
>ref-30322	TACGCCGCCACAGACTCTCCCGTTTCT	15	<lmxll>	ll	101	lm	141		
>ref-30322	TACGCCGCCACAGACTCTCCCGTTTCT	27	<lmxll>	ll	101	lm	141		
>ref-30336	TAACCCCCCACCCTCACTCCTCCCCCC	5	<hkxhk>	hh	0	hk	272	kk	0
>ref-30336	TAACCCCCCACCCTCACTCCTCCCCCC	8	<lmxll>	ll	32	lm	226		
>ref-30336	TAACCCCCCACCCTCACTCCTCCCCCC	12	<lmxll>	ll	32	lm	227		
>ref-30336	TAACCCCCCACCCTCACTCCTCCCCCC	15	<nnxnp>	nn	126	np	114		
>ref-30336	TAACCCCCCACCCTCACTCCTCCCCCC	16	<nnxnp>	nn	95	np	146		
>ref-30336	TAACCCCCCACCCTCACTCCTCCCCCC	23	<hkxhk>	hh	0	hk	272	kk	0
>ref-30349	TAAGATCTGACCCCTCCTCCTTCCCCC	24	<nnxnp>	nn	258	np	0		
>ref-30349	TAAGATCTGACCCCTCCTCCTTCCCCC	27	<nnxnp>	nn	257	np	1		
>ref-30352	CTCCTCCCTACTTGGTCTCCCGTCTCG	16	<hkxhk>	hh	52	hk	137	kk	73
>ref-30356	CAATCCTTCACTCTCTCTCCTATCTAC	3	<lmxll>	ll	125	lm	119		
>ref-30356	CAATCCTTCACTCTCTCTCCTATCTAC	12	<lmxll>	ll	126	lm	119		
>ref-30362	CCGTAGGAGACGTCCGCTCCTGCCTCT	5	<lmxll>	ll	0	lm	219		
>ref-30362	CCGTAGGAGACGTCCGCTCCTGCCTCT	24	<lmxll>	ll	0	lm	219		
>ref-30378	ATCGGCACCACTCTCTCTCCCTCCCTC	13	<nnxnp>	nn	10	np	255		
>ref-30419	GAAGGCAAAACCAAGTCTCCTAATTGC	15	<lmxll>	ll	50	lm	200		

>ref-30419	GAAGGCAAAACCAAGTCTCCTAATTGC	27	<lmxll>	ll	53	lm	199		
>ref-30420	TCACCAAGGACGCACTCTCCAAGGACG	2	<lmxll>	ll	127	lm	124		
>ref-30420	TCACCAAGGACGCACTCTCCAAGGACG	3	<hkxhk>	hh	4	hk	264	kk	0
>ref-30431	TATATATTCACATTCACTCCCTCCCTC	3	<hkxhk>	hh	0	hk	265	kk	2
>ref-30431	TATATATTCACATTCACTCCCTCCCTC	8	<lmxll>	ll	25	lm	237		
>ref-30431	TATATATTCACATTCACTCCCTCCCTC	14	<lmxll>	ll	26	lm	237		
>ref-30438	GTAAGAGGTACTCTCCCTCCCTTCCTT	2	<nnxnp>	nn	115	np	122		
>ref-30442	CAATCTTCCACCTCCTCTCCCTGCATC	4	<lmxll>	ll	127	lm	141		
>ref-30442	CAATCTTCCACCTCCTCTCCCTGCATC	15	<lmxll>	ll	127	lm	141		
>ref-30458	ATATTCTTAACTTTTTCTCCTTCTCCT	6	<lmxll>	ll	18	lm	242		
>ref-30458	ATATTCTTAACTTTTTCTCCTTCTCCT	22	<lmxll>	ll	18	lm	242		
>ref-30458	ATATTCTTAACTTTTTCTCCTTCTCCT	24	<lmxll>	ll	14	lm	249		
>ref-30458	ATATTCTTAACTTTTTCTCCTTCTCCT	26	<lmxll>	ll	14	lm	249		
>ref-30468	ACTCCCCTCACCCTCCCTCCTCTCACT	1	<hkxhk>	hh	8	hk	256	kk	0
>ref-30468	ACTCCCCTCACCCTCCCTCCTCTCACT	5	<hkxhk>	hh	10	hk	255	kk	0
>ref-30468	ACTCCCCTCACCCTCCCTCCTCTCACT	13	<nnxnp>	nn	122	np	142		
>ref-30468	ACTCCCCTCACCCTCCCTCCTCTCACT	21	<nnxnp>	nn	69	np	177		
>ref-30470	ACACTCAAGACCGTGGCTCCTGCTCCT	6	<nnxnp>	nn	136	np	134		
>ref-30494	CCTTTACAAACCTTTTCTCCATTTCTC	13	<nnxnp>	nn	111	np	109		
>ref-30495	AGCAAGATCACAGAATCTCCTCATCAG	16	<lmxll>	ll	109	lm	132		
>ref-30542	TCATCTTTTACTCTCTCTCCGTCTATT	13	<lmxll>	ll	93	lm	153		
>ref-30557	CTACCTCCCACCGACCCTCCTCACGTA	26	<hkxhk>	hh	65	hk	147	kk	52
>ref-30560	CCCTATTCCACTTTTCCTCCCTCCTTC	8	<nnxnp>	nn	123	np	124		
>ref-30586	ATCAATGAAACCTTTTCTCCTTTTCAA	13	<nnxnp>	nn	105	np	129		
>ref-30611	ATGCACTCAACAAGCTCTCCACCGCTA	7	<lmxll>	ll	120	lm	146		
>ref-30611	ATGCACTCAACAAGCTCTCCACCGCTA	23	<lmxll>	ll	123	lm	144		
>ref-30625	ATGTTAATAACGTCATCTCCAGTTACC	21	<hkxhk>	hh	73	hk	136	kk	49
>ref-30635	CCCCTTATCACTTTCTCTCCGTTTATC	13	<hkxhk>	hh	56	hk	133	kk	60
>ref-30638	CCCCATCCAACGCCACCTCCCTCCCCT	23	<nnxnp>	nn	266	np	0		
>ref-30649	GCCATAGAAACGCACGCTCCAAGTATC	1	<lmxll>	ll	129	lm	115		
>ref-30649	GCCATAGAAACGCACGCTCCAAGTATC	23	<lmxll>	ll	128	lm	115		
>ref-30658	AAGAACTAGACCTGGTCTCCCCTTCTT	14	<hkxhk>	hh	0	hk	215	kk	42
>ref-30658	AAGAACTAGACCTGGTCTCCCCTTCTT	22	<lmxll>	ll	135	lm	123		
>ref-30658	AAGAACTAGACCTGGTCTCCCCTTCTT	27	<hkxhk>	hh	0	hk	157	kk	101
>ref-30664	CCATCATCCACTATTCCTCCAGCTCCC	25	<lmxll>	ll	127	lm	133		
>ref-30691	ACAGTCCAGACCTCACCTCCTCGTCCA	5	<hkxhk>	hh	0	hk	271	kk	1
>ref-30691	ACAGTCCAGACCTCACCTCCTCGTCCA	15	<hkxhk>	hh	5	hk	265	kk	0
>ref-30691	ACAGTCCAGACCTCACCTCCTCGTCCA	25	<lmxll>	ll	1	lm	272		

>ref-30701	TCGCCGACAACTTCCGCTCCTTGTACC	8	<lmxll>	ll	111	lm	137		
>ref-30703	GTTTTAGAGACACAATCTCCTTCATCA	21	<lmxll>	ll	126	lm	124		
>ref-30721	GCATTTTCAACTCCTGCTCCGGTGACT	8	<hkxhk>	hh	0	hk	273	kk	0
>ref-30721	GCATTTTCAACTCCTGCTCCGGTGACT	21	<hkxhk>	hh	0	hk	273	kk	0
>ref-30729	CCACGCCCCACTCGTCCTCCCCTACCC	7	<nnxnp>	nn	152	np	116		
>ref-30780	AATTAACGTACAAATCCTCCTAGATTA	9	<lmxll>	ll	128	lm	113		
>ref-30780	AATTAACGTACAAATCCTCCTAGATTA	26	<nnxnp>	nn	114	np	123		
>ref-30797	CCTTTCTTTACTCTATCTCCCTTTCCG	13	<nnxnp>	nn	135	np	103		
>ref-30797	CCTTTCTTTACTCTATCTCCCTTTCCG	15	<nnxnp>	nn	136	np	104		
>ref-30803	CTAACTTCCACCGCGACTCCTTGATCG	6	<lmxll>	ll	125	lm	140		
>ref-30803	CTAACTTCCACCGCGACTCCTTGATCG	27	<nnxnp>	nn	143	np	115		
>ref-30808	ATCATCATCACATTTTCTCCTTCTTCC	12	<nnxnp>	nn	9	np	255		
>ref-30808	ATCATCATCACATTTTCTCCTTCTTCC	16	<nnxnp>	nn	9	np	257		
>ref-30819	TTCTCCTTCACTCCTTCTCCCTTTTCC	23	<hkxhk>	hh	0	hk	222	kk	22
>ref-30819	TTCTCCTTCACTCCTTCTCCCTTTTCC	27	<nnxnp>	nn	258	np	3		
>ref-30850	CCACGCATGACTGCCACTCCGCTCTCC	2	<lmxll>	ll	128	lm	140		
>ref-30851	AGATGATTTACTTGCTCTCCAGCAACT	6	<lmxll>	ll	143	lm	115		
>ref-30871	CGCGCTAACACTTCCCCTCCGACACCT	2	<hkxhk>	hh	235	hk	32	kk	0
>ref-30871	CGCGCTAACACTTCCCCTCCGACACCT	6	<nnxnp>	nn	136	np	118		
>ref-30871	CGCGCTAACACTTCCCCTCCGACACCT	9	<lmxll>	ll	158	lm	104		
>ref-30871	CGCGCTAACACTTCCCCTCCGACACCT	21	<hkxhk>	hh	0	hk	266	kk	7
>ref-30893	CCCCTCTTTACCCATCCTCCTTTCTTC	23	<hkxhk>	hh	3	hk	265	kk	1
>ref-30893	CCCCTCTTTACCCATCCTCCTTTCTTC	26	<hkxhk>	hh	2	hk	266	kk	1
>ref-30937	CATCTCATCACTCGCGCTCCCAAGCCA	24	<nnxnp>	nn	135	np	135		
>ref-30967	CCCACTTAAACGGGACCTCCCTCAAAT	12	<lmxll>	ll	118	lm	111		
>ref-30969	CATCCGCAAACGGCAGCTCCTAGCCGC	22	<nnxnp>	nn	246	np	0		
>ref-30995	TCATCAACGACCCCATCTCCCCCTAGT	9	<lmxll>	ll	56	lm	174		
>ref-30995	TCATCAACGACCCCATCTCCCCCTAGT	26	<lmxll>	ll	124	lm	97		
>ref-31003	CTTACACCTACCCCCACTCCTCCCTCT	21	<lmxll>	ll	137	lm	121		
>ref-31015	TTTCCTCACACCTCCCCTCCTCCCTCC	12	<nnxnp>	nn	234	np	0		
>ref-31039	AAGCATTGCACCGCCTCTCCCCATGAC	6	<hkxhk>	hh	0	hk	273	kk	0
>ref-31062	CGTGCAATAACCATGTCTCCAACGAAT	8	<lmxll>	ll	117	lm	127		
>ref-31062	CGTGCAATAACCATGTCTCCAACGAAT	12	<lmxll>	ll	116	lm	127		
>ref-31064	AGACACACAACAGTTACTCCAGTTCAC	12	<nnxnp>	nn	121	np	115		
>ref-31097	TGCGTTCAAACCAGCACTCCTGACCTA	7	<lmxll>	ll	123	lm	124		
>ref-31102	GGAGTTTTAACGTCGTCTCCCATTATC	14	<lmxll>	ll	118	lm	145		
>ref-31121	TCATGTTGGACGAAATCTCCCTCCGTC	23	<hkxhk>	hh	127	hk	140	kk	0
>ref-31135	TCCTCCCTCACACACTCTCCCTTTCAT	2	<nnxnp>	nn	1	np	270		

>ref-31135	TCCTCCCTCACACACTCTCCCTTTCAT	6	<nnxnp>	nn	1	np	270		
>ref-31142	TCCCTCAACACTCCTCCTCCCTCACCA	8	<hkxhk>	hh	31	hk	222	kk	1
>ref-31142	TCCCTCAACACTCCTCCTCCCTCACCA	25	<hkxhk>	hh	32	hk	222	kk	1
>ref-31145	AAGTCCTTTACCTGCGCTCCGTTCTGT	27	<hkxhk>	hh	54	hk	138	kk	55
>ref-31146	ATCCTTGCTACCTCCCCTCCCCCTCCT	22	<lmxll>	ll	120	lm	143		
>ref-31152	AAAATGTTTACACTTACTCCACACTCG	9	<nnxnp>	nn	259	np	0		
>ref-31189	CACACTCTAACAGACTCTCCAATCCTC	8	<lmxll>	ll	122	lm	129		
>ref-31197	ACAATATTTACCGTCTCTCCTTCCACT	25	<nnxnp>	nn	128	np	137		
>ref-31211	CACGTTCTCACTGAACCTCCGGCTAGA	15	<lmxll>	ll	129	lm	135		
>ref-31247	AGTTAGGTCACTTGTCCTCCGGTTTCA	14	<lmxll>	ll	123	lm	122		
>ref-31254	ACAACCACCACGACTACTCCACTACTA	12	<lmxll>	ll	179	lm	56		
>ref-31254	ACAACCACCACGACTACTCCACTACTA	15	<lmxll>	ll	115	lm	133		
>ref-31254	ACAACCACCACGACTACTCCACTACTA	16	<lmxll>	ll	115	lm	133		
>ref-31260	CCTTTACCCACACCCCCTCCCATCGCC	25	<nnxnp>	nn	153	np	79		
>ref-31281	TACCCAACCACCTCCCCTCCTCTTCTA	24	<lmxll>	ll	149	lm	108		
>ref-31283	GCTCGCTCCACCATACCTCCTTGCTCC	23	<hkxhk>	hh	61	hk	127	kk	73
>ref-31286	CAGATTTCGACAATGGCTCCGGAATGA	8	<lmxll>	ll	121	lm	119		
>ref-31293	CTCACCTTCACTTCATCTCCAACTCCC	1	<nnxnp>	nn	271	np	1		
>ref-31293	CTCACCTTCACTTCATCTCCAACTCCC	27	<hkxhk>	hh	47	hk	154	kk	66
>ref-31302	CGGGATCCAACTACCACTCCCTACAAA	1	<hkxhk>	hh	0	hk	273	kk	0
>ref-31302	CGGGATCCAACTACCACTCCCTACAAA	4	<hkxhk>	hh	0	hk	39	kk	225
>ref-31304	GTACATATCACATGCACTCCATCGCTA	24	<nnxnp>	nn	141	np	119		
>ref-31309	CAGCATAACACTCCCGCTCCGCTTTTT	1	<lmxll>	ll	255	lm	0		
>ref-31321	TGAATGCCAACAGGTTCTCCCAACTCC	8	<nnxnp>	nn	268	np	0		
>ref-31325	ATCAAGGTGACATTGTCTCCACAACAT	16	<nnxnp>	nn	127	np	137		
>ref-31326	CTCTCCCTCACACCCACTCCCACTCAC	6	<hkxhk>	hh	0	hk	271	kk	0
>ref-31326	CTCTCCCTCACACCCACTCCCACTCAC	12	<hkxhk>	hh	0	hk	273	kk	0
>ref-31361	AACGACAGAACCTAAACTCCAGGAACT	13	<lmxll>	ll	111	lm	127		
>ref-31365	TTCCTCCCCACGCCCTCTCCCTGCTCT	23	<nnxnp>	nn	132	np	138		
>ref-31373	TCTAAACGTACTTTGGCTCCAAGACAG	4	<hkxhk>	hh	43	hk	226	kk	0
>ref-31373	TCTAAACGTACTTTGGCTCCAAGACAG	9	<hkxhk>	hh	0	hk	272	kk	1
>ref-31373	TCTAAACGTACTTTGGCTCCAAGACAG	27	<hkxhk>	hh	0	hk	271	kk	0
>ref-31381	GGATTTGAAACGGTGCCTCCTCAAAAT	27	<nnxnp>	nn	116	np	110		
>ref-31385	GTCACTGTCACTCTTTCTCCATCCCTA	13	<lmxll>	ll	127	lm	141		
>ref-31394	CCTCCTCCTACGAAACCTCCTGCTCCT	1	<nnxnp>	nn	79	np	189		
>ref-31394	CCTCCTCCTACGAAACCTCCTGCTCCT	7	<hkxhk>	hh	35	hk	219	kk	0
>ref-31394	CCTCCTCCTACGAAACCTCCTGCTCCT	24	<nnxnp>	nn	78	np	190		
>ref-31408	CTCGGTCCGACCCTCACTCCTTTAGTA	23	<nnxnp>	nn	132	np	123		

>ref-31416	TCCCCTTTCACTGTCCCTCCCCATCCT	6	<hkxhk>	hh	21	hk	244	kk	0
>ref-31416	TCCCCTTTCACTGTCCCTCCCCATCCT	13	<hkxhk>	hh	0	hk	268	kk	2
>ref-31469	CGTAGCAGGACCCACTCTCCGCATCAC	1	<lmxll>	ll	116	lm	148		
>ref-31483	GGCGACATGACCAGCACTCCATAAGCG	23	<hkxhk>	hh	58	hk	129	kk	76
>ref-31485	CTATCAGTCACCTTCCCTCCCGCCCTC	22	<lmxll>	ll	90	lm	167		
>ref-31493	CCGAAAGAGACCGCTTCTCCTATCTTT	13	<lmxll>	ll	132	lm	124		
>ref-31496	CATTTGTCTACTCTTTCTCCACTCCCT	25	<nnxnp>	nn	132	np	138		
>ref-31503	ATGGCTCTTACCCACCCTCCCCCATAT	24	<nnxnp>	nn	101	np	144		
>ref-31509	CCCCCTATCACACTCTCTCCTCCCCGT	14	<lmxll>	ll	119	lm	142		
>ref-31512	GTTTAGCCGACGTTAACTCCACAACTT	2	<nnxnp>	nn	141	np	127		
>ref-31515	CAGACATTGACCCGAGCTCCATCTGGC	16	<nnxnp>	nn	265	np	1		
>ref-31539	TCATCAATGACTTCCGCTCCCTTTCCC	26	<nnxnp>	nn	105	np	154		
>ref-31582	CCAGCTTGGACGGAGCCTCCTGCGCCT	16	<hkxhk>	hh	48	hk	145	kk	71
>ref-31582	CCAGCTTGGACGGAGCCTCCTGCGCCT	26	<nnxnp>	nn	144	np	127		
>ref-31586	TCCCCCTCCACCCCCCCTCCTCTCCTT	2	<lmxll>	ll	51	lm	194		
>ref-31586	TCCCCCTCCACCCCCCCTCCTCTCCTT	4	<hkxhk>	hh	0	hk	261	kk	5
>ref-31586	TCCCCCTCCACCCCCCCTCCTCTCCTT	8	<lmxll>	ll	252	lm	11		
>ref-31586	TCCCCCTCCACCCCCCCTCCTCTCCTT	13	<hkxhk>	hh	6	hk	264	kk	0
>ref-31586	TCCCCCTCCACCCCCCCTCCTCTCCTT	15	<nnxnp>	nn	138	np	110		
>ref-31594	CCTGGGGGAACATTGCCTCCACACTCC	9	<hkxhk>	hh	2	hk	260	kk	0
>ref-31608	ACCCCACGCACCTTACCTCCTTCCTCT	24	<lmxll>	ll	130	lm	141		
>ref-31618	GAGGGACAAACTATTCCTCCTTTCACT	24	<lmxll>	ll	117	lm	143		
>ref-31624	CCCGGCTCCACTCCCTCTCCCTCTTCT	4	<lmxll>	ll	121	lm	138		
>ref-31624	CCCGGCTCCACTCCCTCTCCCTCTTCT	14	<lmxll>	ll	122	lm	139		
>ref-31638	CGTCGTCGAACTGCCTCTCCTGTACTG	2	<nnxnp>	nn	147	np	126		
>ref-31657	TGCCTATACACCCTTCCTCCGAACTCA	21	<nnxnp>	nn	122	np	125		
>ref-31657	TGCCTATACACCCTTCCTCCGAACTCA	26	<lmxll>	ll	152	lm	98		
>ref-31660	TTGTGGGCGACCAGTTCTCCTTGTTCC	3	<lmxll>	ll	91	lm	165		
>ref-31660	TTGTGGGCGACCAGTTCTCCTTGTTCC	13	<hkxhk>	hh	0	hk	273	kk	0
>ref-31660	TTGTGGGCGACCAGTTCTCCTTGTTCC	26	<nnxnp>	nn	86	np	184		
>ref-31678	ACCTTCTCTACTTGTTCTCCTTTCTAC	8	<lmxll>	ll	53	lm	193		
>ref-31678	ACCTTCTCTACTTGTTCTCCTTTCTAC	14	<lmxll>	ll	54	lm	193		
>ref-31687	CGTCGCCCAACTCCCCCTCCCGTTCCC	1	<lmxll>	ll	93	lm	170		
>ref-31713	TCCACCCACACCATCTCTCCTTCTATT	21	<nnxnp>	nn	119	np	144		
>ref-31716	CTCCCTCTAACTCCTCCTCCATTCCCT	5	<lmxll>	ll	65	lm	200		
>ref-31716	CTCCCTCTAACTCCTCCTCCATTCCCT	21	<lmxll>	ll	64	lm	200		
>ref-31723	ACACCTCTCACACCTTCTCCACTGTAT	24	<lmxll>	ll	144	lm	78		
>ref-31730	TCCACAGTAACATCCCCTCCCTGTCCT	24	<nnxnp>	nn	272	np	0		

>ref-31731	GCTTACTTTACCTTACCTCCCCTACCT	24	<lmxll>	ll	96	lm	140		
>ref-31752	CGCCTGCAAACCTAAGCTCCACGCCCG	26	<lmxll>	ll	144	lm	116		
>ref-31752	CGCCTGCAAACCTAAGCTCCACGCCCG	27	<lmxll>	ll	145	lm	116		
>ref-31762	GTCCCCCCAACCAAGTCTCCGTGCTCT	1	<lmxll>	ll	128	lm	131		
>ref-31762	GTCCCCCCAACCAAGTCTCCGTGCTCT	2	<lmxll>	ll	128	lm	131		
>ref-31764	CCCTCCCTCACACCTCCTCCTCACATC	9	<lmxll>	ll	109	lm	148		
>ref-31771	CATTTGCACACCATGACTCCATACAAT	24	<lmxll>	ll	35	lm	186		
>ref-31771	CATTTGCACACCATGACTCCATACAAT	27	<lmxll>	ll	35	lm	186		
>ref-31775	CTTTCTTTCACCACCTCTCCTTTCTCC	5	<hkxhk>	hh	28	hk	210	kk	0
>ref-31775	CTTTCTTTCACCACCTCTCCTTTCTCC	13	<hkxhk>	hh	25	hk	210	kk	0
>ref-31806	TTACCTAGTACTGGCCCTCCGGTCTTA	4	<nnxnp>	nn	242	np	2		
>ref-31806	TTACCTAGTACTGGCCCTCCGGTCTTA	5	<nnxnp>	nn	235	np	2		
>ref-31807	AAATACGCCACCGCCTCTCCCTGCCTC	3	<hkxhk>	hh	4	hk	264	kk	0
>ref-31807	AAATACGCCACCGCCTCTCCCTGCCTC	23	<hkxhk>	hh	0	hk	264	kk	4
>ref-31825	TGCCCTCCTACCCTCCCTCCCGCTTTT	4	<nnxnp>	nn	263	np	0		
>ref-31845	CACACACATACCAGCACTCCTGCCCAG	15	<lmxll>	ll	129	lm	135		
>ref-31852	TCTCCTTCTACCTCCTCTCCTCCATTT	2	<lmxll>	ll	264	lm	1		
>ref-31864	CAGGCTCGAACTTCCCCTCCTGCTAAC	13	<lmxll>	ll	145	lm	110		
>ref-31864	CAGGCTCGAACTTCCCCTCCTGCTAAC	23	<lmxll>	ll	145	lm	110		
>ref-31900	GTATCTCCCACTCCTTCTCCATCCCTC	7	<nnxnp>	nn	264	np	0		
>ref-31957	CTCCTCGCTACTCTGCCTCCTATCTCC	1	<nnxnp>	nn	129	np	122		
>ref-31975	CGCGGCAGCACTCTCACTCCTTCCTTT	27	<nnxnp>	nn	171	np	88		
>ref-32040	ACACCTACAACCACAACTCCAACCTCC	1	<nnxnp>	nn	271	np	0		
>ref-32040	ACACCTACAACCACAACTCCAACCTCC	3	<nnxnp>	nn	272	np	0		
>ref-32045	CAGCCTTATACACGAGCTCCCTCTGCT	21	<lmxll>	ll	125	lm	134		
>ref-32050	GTTTCTTCTACATTTTCTCCTGCTCTA	1	<lmxll>	ll	153	lm	83		
>ref-32050	GTTTCTTCTACATTTTCTCCTGCTCTA	22	<lmxll>	ll	152	lm	83		
>ref-32063	TCTTCTCAAACAGATTCTCCTCCGTGA	21	<nnxnp>	nn	131	np	133		
>ref-32078	ATCATCGTCACCTCCTCTCCTCTCCTC	3	<lmxll>	ll	263	lm	0		
>ref-32078	ATCATCGTCACCTCCTCTCCTCTCCTC	7	<lmxll>	ll	261	lm	0		
>ref-32094	CACGTACACACACTGACTCCTCGTATG	15	<hkxhk>	hh	63	hk	142	kk	45
>ref-32109	TACAGCAGAACTCCAACTCCCACTCCC	3	<nnxnp>	nn	238	np	0		
>ref-32111	ACACACGAAACGTGATCTCCTTCAAAT	22	<lmxll>	ll	119	lm	140		
>ref-32116	TGGTTCTTTACTACCTCTCCTCGCATG	25	<nnxnp>	nn	149	np	119		
>ref-32141	CCGCCAAACACACCGCCTCCGTGGACA	2	<lmxll>	ll	188	lm	79		
>ref-32153	ACCACAATCACCAAAGCTCCACCACCC	14	<hkxhk>	hh	51	hk	143	kk	71
>ref-32153	ACCACAATCACCAAAGCTCCACCACCC	15	<hkxhk>	hh	70	hk	143	kk	51
>ref-32169	GTAGGTGTAACTCCCCCTCCAGTGCCT	6	<nnxnp>	nn	128	np	138		

>ref-32173	CCGTGGCAGACAATTGCTCCCGGCGAG	1	<lmxll>	ll	198	lm	63			
>ref-32195	TTCACTCTCACATTCCCTCCATTCTTC	3	<nnxnp>	nn	250	np	1			
>ref-32195	TTCACTCTCACATTCCCTCCATTCTTC	4	<nnxnp>	nn	250	np	2			
>ref-32198	CCCCTTTCTACGATCACTCCTCTTCCT	6	<nnxnp>	nn	219	np	0			
>ref-32215	TTAGTTCACACTAGTCCTCCTCTACCT	1	<lmxll>	ll	120	lm	143			
>ref-32215	TTAGTTCACACTAGTCCTCCTCTACCT	14	<lmxll>	ll	120	lm	142			
>ref-32221	TTTGATATCACTATATCTCCATCTCCC	23	<lmxll>	ll	136	lm	94			
>ref-32250	CTCATCACCACACGCTCTCCAGCAGAG	23	<nnxnp>	nn	179	np	73			
>ref-32267	CTGATGGTGACCTCTCCTCCTCCTTCT	1	<hkxhk>	hh	45	hk	157	kk	57	
>ref-32267	CTGATGGTGACCTCTCCTCCTCCTTCT	4	<hkxhk>	hh	44	hk	158	kk	57	
>ref-32285	ACAAGCGATACTCCTCCTCCTCCTCCT	2	<nnxnp>	nn	241	np	5			
>ref-32300	TCACTCGGCACTTCCCCTCCGTTTCCC	6	<nnxnp>	nn	115	np	109			
>ref-32300	TCACTCGGCACTTCCCCTCCGTTTCCC	9	<nnxnp>	nn	115	np	109			
>ref-32317	TACACTTAAACTCTCTCTCCCTTCTCA	3	<lmxll>	ll	117	lm	128			
>ref-32317	TACACTTAAACTCTCTCTCCCTTCTCA	27	<lmxll>	ll	113	lm	129			
>ref-32318	CACCCTTCCACCCACTCTCCCCACTCC	3	<lmxll>	ll	140	lm	93			
>ref-32318	CACCCTTCCACCCACTCTCCCCACTCC	13	<lmxll>	ll	192	lm	51			
>ref-32322	TCAATTCAGACACGCACTCCTTCACAA	8	<nnxnp>	nn	131	np	137			
>ref-32330	TTTCTCCTTACTCCTCCTCCCTTTCCC	3	<lmxll>	ll	82	lm	177			
>ref-32330	TTTCTCCTTACTCCTCCTCCCTTTCCC	24	<lmxll>	ll	111	lm	152			
>ref-32361	CCTCCCCCTACCCTATCTCCTTTTCTC	15	<lmxll>	ll	130	lm	108			
>ref-32361	CCTCCCCCTACCCTATCTCCTTTTCTC	23	<nnxnp>	nn	104	np	132			
>ref-32361	CCTCCCCCTACCCTATCTCCTTTTCTC	24	<hkxhk>	hh	2	hk	180	kk	47	
>ref-32368	ACCTTTTCAACTACACCTCCTTTCCTT	27	<nnxnp>	nn	113	np	135			
>ref-32381	CTTACGTCCACCCTCTCTCCTCCTTCC	1	<lmxll>	ll	128	lm	140			
>ref-32381	CTTACGTCCACCCTCTCTCCTCCTTCC	5	<lmxll>	ll	134	lm	135			
>ref-32381	CTTACGTCCACCCTCTCTCCTCCTTCC	8	<lmxll>	ll	129	lm	139			
>ref-32410	GAGAGAAACACCCGCACTCCTTAAGCT	23	<hkxhk>	hh	46	hk	144	kk	69	
>ref-32424	TCCTGGCCAACAAACGCTCCGTCTTCC	3	<lmxll>	ll	258	lm	8			
>ref-32424	TCCTGGCCAACAAACGCTCCGTCTTCC	15	<hkxhk>	hh	0	hk	259	kk	0	
>ref-32439	GCCTACTTTACTTCTTCTCCTTCTCCT	13	<nnxnp>	nn	137	np	127			
>ref-32447	TTATAGAATACATGACCTCCGGGAACC	21	<nnxnp>	nn	119	np	112			
>ref-32459	AAAACATACACACTCTCTCCTCTCTCT	26	<hkxhk>	hh	31	hk	148	kk	63	
>ref-32494	CTCCTTACCACCTCCTCTCCTCCTCTT	7	<lmxll>	ll	179	lm	65			
>ref-32494	CTCCTTACCACCTCCTCTCCTCCTCTT	27	<lmxll>	ll	180	lm	68			
>ref-32512	CTAGCCTGCACCCTCCCTCCCCCTCCC	12	<lmxll>	ll	239	lm	3			
>ref-32512	CTAGCCTGCACCCTCCCTCCCCCTCCC	27	<lmxll>	ll	242	lm	0			
>ref-32514	CCACCTCTCACTCTCTCTCCCTATCTA	5	<lmxll>	ll	109	lm	139			

>ref-32537	TGAGTCGCAACTTCGCCTCCGTCTCCT	14	<lmxll>	ll	132	lm	123		
>ref-32547	ATCCAGGTAACCTCGTCTCCCTGGTGA	6	<nnxnp>	nn	112	np	132		
>ref-32548	TGTACCGCCACTTACCCTCCGCTTCAT	8	<nnxnp>	nn	147	np	125		
>ref-32563	TGGAAATCGACTGCAGCTCCGGAACCC	8	<nnxnp>	nn	116	np	109		
>ref-32567	GGACGAGATACAGATCCTCCTGCCTTA	8	<hkxhk>	hh	0	hk	273	kk	0
>ref-32581	CACTTACACACCCTGCCTCCTTCCCTT	3	<lmxll>	ll	265	lm	0		
>ref-32592	CTCACCTACACGTTCCCTCCTCATGAA	9	<nnxnp>	nn	138	np	129		
>ref-32613	CTGCACAAGACAACAGCTCCCCATTCA	26	<lmxll>	ll	147	lm	119		
>ref-32657	CCTCTTTTTACTCTTCCTCCTCCTTTA	25	<nnxnp>	nn	160	np	70		
>ref-32660	GGCCTACTGACGTGCTCTCCTTCCTAC	26	<lmxll>	ll	119	lm	148		
>ref-32661	CAATCACCCACACACACTCCCATTGCT	13	<nnxnp>	nn	153	np	112		
>ref-32702	GATCTCCACACCTCTTCTCCTGTCACT	13	<lmxll>	ll	117	lm	124		
>ref-32702	GATCTCCACACCTCTTCTCCTGTCACT	14	<nnxnp>	nn	107	np	128		
>ref-32707	TTTCCTTTTACTTTGTCTCCGAACGCT	12	<nnxnp>	nn	258	np	0		
>ref-32753	TCTCACGTGACCTTCGCTCCGTCAGGT	5	<hkxhk>	hh	0	hk	273	kk	0
>ref-32753	TCTCACGTGACCTTCGCTCCGTCAGGT	27	<nnxnp>	nn	174	np	90		
>ref-32754	CCCCCTTGAACCTCACCTCCTTCCCCC	24	<lmxll>	ll	173	lm	85		
>ref-32754	CCCCCTTGAACCTCACCTCCTTCCCCC	25	<lmxll>	ll	173	lm	86		
>ref-32801	CCTCCCCCTACCCTACCTCCTCTATTT	15	<nnxnp>	nn	224	np	2		
>ref-32801	CCTCCCCCTACCCTACCTCCTCTATTT	23	<nnxnp>	nn	233	np	0		
>ref-32816	GAAGGCGTCACAATCACTCCTCGGGTC	9	<nnxnp>	nn	118	np	123		
>ref-32840	ATGTTACCAACAACTGCTCCCTGACAT	2	<lmxll>	ll	124	lm	140		
>ref-32840	ATGTTACCAACAACTGCTCCCTGACAT	7	<lmxll>	ll	124	lm	140		
>ref-32853	TATCATCACACACTCACTCCTTCCTTC	2	<hkxhk>	hh	3	hk	268	kk	0
>ref-32853	TATCATCACACACTCACTCCTTCCTTC	9	<hkxhk>	hh	0	hk	268	kk	3
>ref-32867	CCCTCACACACGCGCGCTCCAAGCCCT	24	<hkxhk>	hh	64	hk	113	kk	45
>ref-32867	CCCTCACACACGCGCGCTCCAAGCCCT	25	<hkxhk>	hh	65	hk	113	kk	45
>ref-32875	TACTCACTCACTCACTCTCCCATGCAA	5	<hkxhk>	hh	69	hk	137	kk	52
>ref-32880	GTATTTTAAACCAAGTCTCCTTACTTG	21	<hkxhk>	hh	42	hk	173	kk	4
>ref-32891	GAACATGTAACAGATGCTCCGCGTTAT	12	<hkxhk>	hh	54	hk	138	kk	71
>ref-32897	TTCCTCTTCACCTTCTCTCCTCTCTTA	12	<hkxhk>	hh	2	hk	172	kk	60
>ref-32897	TTCCTCTTCACCTTCTCTCCTCTCTTA	24	<hkxhk>	hh	60	hk	172	kk	2
>ref-32902	TACCCCAACACCAATTCTCCATCTCCC	15	<hkxhk>	hh	0	hk	265	kk	2
>ref-32902	TACCCCAACACCAATTCTCCATCTCCC	21	<hkxhk>	hh	2	hk	265	kk	0
>ref-32922	TTAAACACGACGAGCGCTCCTAACATC	8	<nnxnp>	nn	121	np	147		
>ref-32926	CTCCCCTGTACTCCCTCTCCATTCTCT	3	<hkxhk>	hh	42	hk	136	kk	64
>ref-32927	CCTGTACCCACCCTCTCTCCTTCCTTC	4	<nnxnp>	nn	136	np	109		
>ref-32927	CCTGTACCCACCCTCTCTCCTTCCTTC	7	<nnxnp>	nn	140	np	109		

>ref-32928	CTCTGTATCACCCTTCCTCCCTCTCTG	25	<nnxnp>	nn	145	np	125		
>ref-32933	GGGGAAGAAACAGATCCTCCGTGCTCC	1	<nnxnp>	nn	116	np	152		
>ref-32958	CTCCTCCCCACCTGCTCTCCTTTCTAT	12	<nnxnp>	nn	116	np	136		
>ref-32958	CTCCTCCCCACCTGCTCTCCTTTCTAT	27	<nnxnp>	nn	120	np	135		
>ref-32960	CCGGGCAGTACGTTTGCTCCGTTTTCT	9	<nnxnp>	nn	272	np	0		
>ref-32969	CACTGTACCACGGCGGCTCCCTTATAT	3	<hkxhk>	hh	0	hk	272	kk	0
>ref-32969	CACTGTACCACGGCGGCTCCCTTATAT	21	<hkxhk>	hh	76	hk	184	kk	0
>ref-32969	CACTGTACCACGGCGGCTCCCTTATAT	22	<hkxhk>	hh	0	hk	234	kk	0
>ref-32969	CACTGTACCACGGCGGCTCCCTTATAT	26	<lmxll>	ll	0	lm	242		
>ref-32989	CCATTCGCCACATCTTCTCCCCCTCCT	13	<nnxnp>	nn	123	np	137		
>ref-33013	CACACACACACACAATCTCCCTCCATT	3	<nnxnp>	nn	101	np	161		
>ref-33013	CACACACACACACAATCTCCCTCCATT	14	<nnxnp>	nn	178	np	81		
>ref-33013	CACACACACACACAATCTCCCTCCATT	15	<lmxll>	ll	95	lm	124		
>ref-33013	CACACACACACACAATCTCCCTCCATT	23	<hkxhk>	hh	77	hk	190	kk	0
>ref-33013	CACACACACACACAATCTCCCTCCATT	24	<hkxhk>	hh	8	hk	263	kk	0
>ref-33013	CACACACACACACAATCTCCCTCCATT	25	<hkxhk>	hh	0	hk	273	kk	0
>ref-33017	CCATCATCCACTTCTTCTCCCTCCTTC	16	<lmxll>	ll	132	lm	135		
>ref-33024	AATTACTTAACCATCTCTCCCGTGTAT	9	<lmxll>	ll	119	lm	137		
>ref-33029	GAGATAATGACAATGTCTCCATTATTC	9	<lmxll>	ll	133	lm	136		
>ref-33045	GACACGTACACTGTTCCTCCAGGCACA	22	<nnxnp>	nn	144	np	123		
>ref-33052	TCCCTCCCTACACCGGCTCCCTCCCCC	1	<nnxnp>	nn	257	np	0		
>ref-33052	TCCCTCCCTACACCGGCTCCCTCCCCC	23	<nnxnp>	nn	115	np	126		
>ref-33052	TCCCTCCCTACACCGGCTCCCTCCCCC	27	<nnxnp>	nn	260	np	0		
>ref-33054	ACGCCCATAACCTTCTCTCCCTCCTCT	3	<nnxnp>	nn	119	np	141		
>ref-33077	TATCACGTGACATCATCTCCTGGCTCA	6	<nnxnp>	nn	120	np	148		
>ref-33097	TACGTAAAGACCGCCGCTCCTTTCTAA	15	<nnxnp>	nn	127	np	142		
>ref-33128	GATGGTTTTACTCTCTCTCCCGCCCAT	6	<hkxhk>	hh	52	hk	143	kk	69
>ref-33132	CCGTTCGAAACCTCTTCTCCACGGCAA	24	<nnxnp>	nn	101	np	126		
>ref-33157	CATGTTGCAACTTTTACTCCTCTTCGC	16	<nnxnp>	nn	104	np	152		
>ref-33185	AAAGACAGGACAGTGACTCCTCCTCCT	25	<lmxll>	ll	118	lm	128		
>ref-33185	AAAGACAGGACAGTGACTCCTCCTCCT	27	<lmxll>	ll	118	lm	128		
>ref-33187	CAAGGGGATACATTATCTCCTTTGCTG	3	<hkxhk>	hh	106	hk	150	kk	0
>ref-33187	CAAGGGGATACATTATCTCCTTTGCTG	7	<lmxll>	ll	197	lm	61		
>ref-33187	CAAGGGGATACATTATCTCCTTTGCTG	12	<hkxhk>	hh	104	hk	151	kk	0
>ref-33187	CAAGGGGATACATTATCTCCTTTGCTG	13	<lmxll>	ll	65	lm	186		
>ref-33187	CAAGGGGATACATTATCTCCTTTGCTG	24	<hkxhk>	hh	12	hk	258	kk	0
>ref-33193	ATCTCTCCAACAGTCTCTCCGACACAA	22	<lmxll>	ll	128	lm	140		
>ref-33194	TGCCACTATACAGCTTCTCCCTGGATG	22	<lmxll>	ll	122	lm	127		

>ref-33203	TTGCCATGGACTTACTCTCCATGTGGC	5	<lmxll>	ll	84	lm	160		
>ref-33203	TTGCCATGGACTTACTCTCCATGTGGC	6	<lmxll>	ll	83	lm	161		
>ref-33203	TTGCCATGGACTTACTCTCCATGTGGC	12	<hkxhk>	hh	0	hk	272	kk	0
>ref-33203	TTGCCATGGACTTACTCTCCATGTGGC	15	<hkxhk>	hh	0	hk	270	kk	0
>ref-33209	TTTATCTCTACCTGTCCTCCTCTTCCT	14	<lmxll>	ll	145	lm	127		
>ref-33217	CAGCAATTCACATGCTCTCCATCGGAA	6	<hkxhk>	hh	0	hk	273	kk	0
>ref-33217	CAGCAATTCACATGCTCTCCATCGGAA	23	<lmxll>	ll	260	lm	9		
>ref-33223	TACCCCTCCACCTCTTCTCCCCCTACC	12	<lmxll>	ll	179	lm	56		
>ref-33223	TACCCCTCCACCTCTTCTCCCCCTACC	15	<hkxhk>	hh	1	hk	255	kk	5
>ref-33223	TACCCCTCCACCTCTTCTCCCCCTACC	25	<nnxnp>	nn	92	np	157		
>ref-33223	TACCCCTCCACCTCTTCTCCCCCTACC	27	<nnxnp>	nn	83	np	154		
>ref-33229	CTCACACACACTTTTACTCCTCATTTT	4	<nnxnp>	nn	109	np	128		
>ref-33229	CTCACACACACTTTTACTCCTCATTTT	6	<nnxnp>	nn	110	np	127		
>ref-33244	TGTTCCCCGACACGCGCTCCTGTCTTA	9	<nnxnp>	nn	255	np	0		
>ref-33285	CTCGCACAGACTTTCCCTCCTTATTCT	1	<nnxnp>	nn	110	np	125		
>ref-33285	CTCGCACAGACTTTCCCTCCTTATTCT	15	<hkxhk>	hh	49	hk	128	kk	44
>ref-33287	ATCGTTCTCACTATTCCTCCCTCCTCC	4	<hkxhk>	hh	85	hk	149	kk	0
>ref-33287	ATCGTTCTCACTATTCCTCCCTCCTCC	7	<hkxhk>	hh	85	hk	149	kk	0
>ref-33306	ACTCGACATACTCAGACTCCTGGCATA	4	<lmxll>	ll	132	lm	136		
>ref-33312	CTACTCTCTACACCTCCTCCTCTTTCG	14	<nnxnp>	nn	111	np	158		
>ref-33318	CGATAGCAGACTTTTCCTCCGACTCTC	25	<lmxll>	ll	150	lm	108		
>ref-33321	ACAACAAGAACAGTCTCTCCAACAACA	6	<hkxhk>	hh	0	hk	272	kk	0
>ref-33375	GTATGAATAACACATTCTCCTTTGCCA	1	<nnxnp>	nn	266	np	0		
>ref-33400	TCATTTTTCACCCTTCCTCCTCTCCCT	3	<hkxhk>	hh	2	hk	266	kk	3
>ref-33400	TCATTTTTCACCCTTCCTCCTCTCCCT	9	<hkxhk>	hh	3	hk	266	kk	2
>ref-33412	CACTTTCTGACATCCCCTCCACACACA	14	<hkxhk>	hh	81	hk	177	kk	0
>ref-33418	GAAAACGTGACCCGAGCTCCGAGCCAC	23	<nnxnp>	nn	123	np	127		
>ref-33433	TATCTTCAAACACCATCTCCACCATCG	6	<nnxnp>	nn	144	np	109		
>ref-33441	TCCTCCCCCACCCAGCCTCCTTCCCCC	21	<lmxll>	ll	152	lm	108		
>ref-33442	CACACACACACGTCCTCTCCCCTCTTT	12	<lmxll>	ll	123	lm	130		
>ref-33442	CACACACACACGTCCTCTCCCCTCTTT	16	<lmxll>	ll	125	lm	127		
>ref-33442	CACACACACACGTCCTCTCCCCTCTTT	25	<lmxll>	ll	52	lm	178		
>ref-33446	AACATTTCTACTAACACTCCCCTTCTG	25	<nnxnp>	nn	131	np	98		
>ref-33482	CAGACCCCCACAGTCCCTCCCCCTCCC	5	<hkxhk>	hh	0	hk	213	kk	43
>ref-33497	CTTACTCCTACCCCTTCTCCACATCCT	12	<lmxll>	ll	256	lm	0		
>ref-33497	CTTACTCCTACCCCTTCTCCACATCCT	21	<lmxll>	ll	253	lm	0		
>ref-33504	TAACTCCCGACCCTTTCTCCACCAACA	7	<lmxll>	ll	136	lm	121		
>ref-33504	TAACTCCCGACCCTTTCTCCACCAACA	22	<lmxll>	ll	137	lm	121		

>ref-33505	CAATGTGGCACTCATACTCCTTGTAAT	4	<lmxll>	ll	123	lm	137		
>ref-33509	ATCTATCTCACGTTATCTCCTGCCTTT	21	<lmxll>	ll	135	lm	130		
>ref-33509	ATCTATCTCACGTTATCTCCTGCCTTT	22	<lmxll>	ll	136	lm	130		
>ref-33535	CCCTCTCATACCTATCCTCCCCCTTC	24	<lmxll>	ll	58	lm	196		
>ref-33541	GTGAGTGGCACTCTGGCTCCCGCCCCG	25	<nnxnp>	nn	273	np	0		
>ref-33555	AACAACTAGACCCGGTCTCCCCTACTA	1	<lmxll>	ll	21	lm	237		
>ref-33555	AACAACTAGACCCGGTCTCCCCTACTA	9	<lmxll>	ll	217	lm	36		
>ref-33555	AACAACTAGACCCGGTCTCCCCTACTA	15	<hkxhk>	hh	0	hk	261	kk	3
>ref-33555	AACAACTAGACCCGGTCTCCCCTACTA	22	<hkxhk>	hh	90	hk	148	kk	0
>ref-33555	AACAACTAGACCCGGTCTCCCCTACTA	24	<hkxhk>	hh	90	hk	148	kk	0
>ref-33555	AACAACTAGACCCGGTCTCCCCTACTA	25	<lmxll>	ll	89	lm	153		
>ref-33555	AACAACTAGACCCGGTCTCCCCTACTA	26	<lmxll>	ll	21	lm	237		
>ref-33555	AACAACTAGACCCGGTCTCCCCTACTA	27	<hkxhk>	hh	3	hk	261	kk	0
>ref-33572	TTCCACAGTACCTGGTCTCCCACAGTA	2	<hkxhk>	hh	0	hk	265	kk	4
>ref-33572	TTCCACAGTACCTGGTCTCCCACAGTA	3	<hkxhk>	hh	2	hk	264	kk	0
>ref-33572	TTCCACAGTACCTGGTCTCCCACAGTA	4	<lmxll>	ll	209	lm	47		
>ref-33590	AGCCGCTGGACATATGCTCCTGCAGCT	1	<nnxnp>	nn	261	np	11		
>ref-33590	AGCCGCTGGACATATGCTCCTGCAGCT	2	<nnxnp>	nn	261	np	11		
>ref-33595	CACACACACACATATCCTCCCTGAGAC	16	<hkxhk>	hh	0	hk	218	kk	27
>ref-33595	CACACACACACATATCCTCCCTGAGAC	27	<hkxhk>	hh	0	hk	217	kk	28
>ref-33599	CCTCCTCTCACTCGCCCTCCACTTATC	5	<nnxnp>	nn	261	np	0		
>ref-33599	CCTCCTCTCACTCGCCCTCCACTTATC	21	<lmxll>	ll	262	lm	0		
>ref-33627	TTCGCTGTCACATAAACTCCGACCTTG	26	<lmxll>	ll	148	lm	93		
>ref-33636	TACGCACGCACGCACACTCCTCCAAAT	1	<nnxnp>	nn	129	np	135		
>ref-33636	TACGCACGCACGCACACTCCTCCAAAT	27	<nnxnp>	nn	130	np	133		
>ref-33647	TCCTCCCTCACCCTCCCTCCCCTGGTT	5	<nnxnp>	nn	135	np	108		
>ref-33652	CTCTACCTTACCTGTCCTCCAGCTACC	26	<nnxnp>	nn	271	np	1		
>ref-33654	TCCTCACTAACCTCTCCTCCCTCCCTC	9	<lmxll>	ll	7	lm	264		
>ref-33654	TCCTCACTAACCTCTCCTCCCTCCCTC	24	<lmxll>	ll	20	lm	245		
>ref-33656	TATATATGTACGTATCCTCCTATCGAT	4	<lmxll>	ll	140	lm	121		
>ref-33669	TGGCGATGGACATCACCTCCAGGGAGT	1	<hkxhk>	hh	0	hk	265	kk	6
>ref-33669	TGGCGATGGACATCACCTCCAGGGAGT	5	<hkxhk>	hh	0	hk	260	kk	10
>ref-33669	TGGCGATGGACATCACCTCCAGGGAGT	6	<hkxhk>	hh	9	hk	261	kk	0
>ref-33669	TGGCGATGGACATCACCTCCAGGGAGT	7	<hkxhk>	hh	0	hk	265	kk	6
>ref-33678	CTCCCCTCCACCCCTTCTCCTCTACCC	27	<nnxnp>	nn	217	np	4		
>ref-33682	CCCCCCCTCACCTGTCCTCCCTCCCTC	4	<lmxll>	ll	246	lm	6		
>ref-33691	CCCTTTCCCACTTTCTCTCCCCATCTC	5	<lmxll>	ll	0	lm	262		
>ref-33699	ACGAAATTCACTCTATCTCCCTTGATC	13	<lmxll>	ll	94	lm	125		

>ref-33701	GCCCTTCTAACCCCCCCTCCTCCCCCA	21	<lmxll>	ll	117	lm	116		
>ref-33702	CACGCATTCACCCCACCTCCTTCCTCT	6	<hkxhk>	hh	20	hk	230	kk	1
>ref-33702	CACGCATTCACCCCACCTCCTTCCTCT	9	<hkxhk>	hh	1	hk	230	kk	19
>ref-33726	TGATATATGACACAGTCTCCCTTGTTA	15	<nnxnp>	nn	126	np	138		
>ref-33760	CTGACTATGACTCTGTCTCCGTCTCTC	21	<hkxhk>	hh	51	hk	143	kk	66
>ref-33772	GGCTCGATTACTCTCACTCCCTTTCCT	9	<nnxnp>	nn	264	np	0		
>ref-33816	CTGTTTCCCACTCTCCCTCCCTGTTTC	3	<lmxll>	ll	176	lm	71		
>ref-33816	CTGTTTCCCACTCTCCCTCCCTGTTTC	9	<nnxnp>	nn	125	np	133		
>ref-33816	CTGTTTCCCACTCTCCCTCCCTGTTTC	23	<lmxll>	ll	174	lm	70		
>ref-33816	CTGTTTCCCACTCTCCCTCCCTGTTTC	25	<nnxnp>	nn	121	np	135		
>ref-33857	ATACCTTCTACTCCACCTCCGCCTCCG	16	<nnxnp>	nn	128	np	121		
>ref-33870	CCACATATTACTCCCTCTCCTATTCTT	26	<nnxnp>	nn	95	np	134		
>ref-33879	CTCGGCACTACCTCTGCTCCTAATCAT	1	<nnxnp>	nn	125	np	123		
>ref-33879	CTCGGCACTACCTCTGCTCCTAATCAT	2	<nnxnp>	nn	106	np	143		
>ref-33890	TGCTAATTTACGATCACTCCCTGCTCC	12	<nnxnp>	nn	269	np	0		
>ref-33907	CACCCCACCACCTTATCTCCACCCCTA	4	<lmxll>	ll	131	lm	126		
>ref-33913	TACCTAAGAACCCTCCCTCCTCCTTAT	5	<nnxnp>	nn	125	np	121		
>ref-33920	CCCCCTCAGACGACCCCTCCCTTCCCT	2	<nnxnp>	nn	127	np	120		
>ref-33933	TACCCTTTTACCTTCCCTCCCTTTACT	26	<lmxll>	ll	251	lm	6		
>ref-33949	TTCGTCATGACTCCATCTCCTTTATTC	22	<lmxll>	ll	142	lm	95		
>ref-33958	CACTTTATTACACCTTCTCCACTATAA	14	<lmxll>	ll	145	lm	92		
>ref-33971	GGAGATGCGACTGATGCTCCCCTTGAG	21	<nnxnp>	nn	1	np	261		
>ref-33978	ATAACACGTACCCTTCCTCCTTTCAAT	16	<lmxll>	ll	120	lm	109		
>ref-33983	AATTCTGACACATAACCTCCAACTGAT	24	<lmxll>	ll	119	lm	108		
>ref-34006	TTGTTTACAACCAACTCTCCCGTTAGA	21	<nnxnp>	nn	131	np	117		
>ref-34013	ACGACGATAACTTCAGCTCCCAGCTCT	21	<nnxnp>	nn	266	np	0		
>ref-34017	CCTCCACCCACCAAGCCTCCCTCCCTC	25	<nnxnp>	nn	141	np	117		
>ref-34024	CCTTTCCTAACCCTCCCTCCTTCGCTC	9	<lmxll>	ll	245	lm	1		
>ref-34039	CCCTCGTCCACACCCTCTCCTTTTCCT	9	<lmxll>	ll	120	lm	136		
>ref-34048	TATAAGAACACGAAGCCTCCACACCAC	2	<hkxhk>	hh	152	hk	99	kk	0
>ref-34048	TATAAGAACACGAAGCCTCCACACCAC	15	<hkxhk>	hh	2	hk	270	kk	0
>ref-34094	AGCACACTTACCGTCACTCCCAGGGAT	5	<lmxll>	ll	119	lm	146		
>ref-34111	TATACATCTACCTCCTCTCCATCTACT	27	<lmxll>	ll	115	lm	134		
>ref-34117	TACAATGAAACTTGTTCTCCATCCTTG	26	<hkxhk>	hh	0	hk	272	kk	0
>ref-34148	TCGGCGATGACTGGCGCTCCTCCCTTT	13	<nnxnp>	nn	140	np	128		
>ref-34153	TATTCTTGCACGAATACTCCTTTCTCT	25	<lmxll>	ll	138	lm	123		
>ref-34155	CTAGAGTTTACTTCTTCTCCTTTCTCC	21	<lmxll>	ll	32	lm	234		
>ref-34155	CTAGAGTTTACTTCTTCTCCTTTCTCC	27	<lmxll>	ll	32	lm	234		

>ref-34160	TCCTCATTAACAATATCTCCATCGTTA	14	<lmxll>	ll	119	lm	106		
>ref-34200	ATTCCCTTCACGTTTTCTCCCTCTAAC	4	<lmxll>	ll	15	lm	242		
>ref-34244	GAGAGAGACACAAGTCCTCCAGCGCTG	7	<lmxll>	ll	115	lm	152		
>ref-34244	GAGAGAGACACAAGTCCTCCAGCGCTG	9	<lmxll>	ll	115	lm	152		
>ref-34245	TCCCCCAACACACGCTCTCCCTCTCTC	26	<lmxll>	ll	122	lm	100		
>ref-34245	TCCCCCAACACACGCTCTCCCTCTCTC	27	<lmxll>	ll	122	lm	101		
>ref-34261	TCCATTGTCACTTCGCCTCCCTCACGC	22	<hkxhk>	hh	60	hk	138	kk	71
>ref-34273	GATTCGTCAACTTCAGCTCCATTCCTA	14	<nnxnp>	nn	123	np	140		
>ref-34273	GATTCGTCAACTTCAGCTCCATTCCTA	26	<nnxnp>	nn	121	np	140		
>ref-34296	CGAGGTCACACCAAACCTCCGCAGGAT	13	<nnxnp>	nn	120	np	128		
>ref-34296	CGAGGTCACACCAAACCTCCGCAGGAT	24	<lmxll>	ll	148	lm	105		
>ref-34299	TCCCCCCTTACCTCATCTCCTTCCTTT	8	<lmxll>	ll	135	lm	128		
>ref-34312	ATCGTCCTCACTTTTTCTCCTTTCCCT	22	<nnxnp>	nn	127	np	128		
>ref-34327	TCCTCTCCCACCACTCCTCCCCCCTCT	6	<lmxll>	ll	219	lm	7		
>ref-34327	TCCTCTCCCACCACTCCTCCCCCCTCT	21	<lmxll>	ll	223	lm	4		
>ref-34357	AGCGTCACGACTCAGCCTCCGCTAGAT	1	<lmxll>	ll	130	lm	141		
>ref-34366	ATCTCCATTACGCAGTCTCCATCTTCT	24	<lmxll>	ll	117	lm	147		
>ref-34375	CACTCTCTCACCACCTCTCCTTCCTTC	3	<hkxhk>	hh	1	hk	269	kk	0
>ref-34375	CACTCTCTCACCACCTCTCCTTCCTTC	13	<hkxhk>	hh	1	hk	269	kk	0
>ref-34376	CTCACTACAACCCTCTCTCCACCATCC	12	<hkxhk>	hh	0	hk	222	kk	16
>ref-34376	CTCACTACAACCCTCTCTCCACCATCC	26	<hkxhk>	hh	0	hk	273	kk	0
>ref-34378	TTTCTCTCCACTTCCTCTCCTCACGTT	25	<nnxnp>	nn	82	np	159		
>ref-34378	TTTCTCTCCACTTCCTCTCCTCACGTT	26	<nnxnp>	nn	81	np	160		
>ref-34379	CGCTGAGGGACACATTCTCCCCGGGTC	7	<lmxll>	ll	264	lm	0		
>ref-34390	ATCTCGGGAACGGAGACTCCTGGGGGA	26	<hkxhk>	hh	0	hk	116	kk	138
>ref-34390	ATCTCGGGAACGGAGACTCCTGGGGGA	27	<hkxhk>	hh	139	hk	115	kk	0
>ref-34399	ACCCTCCCTACTCCTCCTCCCTCTCTT	1	<nnxnp>	nn	10	np	260		
>ref-34399	ACCCTCCCTACTCCTCCTCCCTCTCTT	2	<nnxnp>	nn	12	np	255		
>ref-34403	AAATTACAGACGAATCCTCCGCGCCAT	3	<nnxnp>	nn	223	np	0		
>ref-34432	ACAAATATCACGTACGCTCCGCCGTGT	24	<nnxnp>	nn	124	np	139		
>ref-34441	CCACAGCCCACCCCAACTCCACAGAGC	23	<lmxll>	ll	147	lm	120		
>ref-34471	TTCACATTTACGTACCCTCCCTTTCTC	21	<lmxll>	ll	131	lm	120		
>ref-34542	CCTCATCCTACCATCCCTCCCTTCCCT	1	<nnxnp>	nn	271	np	0		
>ref-34542	CCTCATCCTACCATCCCTCCCTTCCCT	3	<lmxll>	ll	145	lm	122		
>ref-34542	CCTCATCCTACCATCCCTCCCTTCCCT	5	<nnxnp>	nn	3	np	260		
>ref-34542	CCTCATCCTACCATCCCTCCCTTCCCT	13	<nnxnp>	nn	269	np	0		
>ref-34571	CCTCCCCTTACGCCTTCTCCCTTCCCC	7	<lmxll>	ll	145	lm	96		
>ref-34571	CCTCCCCTTACGCCTTCTCCCTTCCCC	9	<nnxnp>	nn	233	np	24		

>ref-34571	CCTCCCCTTACGCCTTCTCCCTTCCCC	22	<nnxnp>	nn	233	np	22		
>ref-34571	CCTCCCCTTACGCCTTCTCCCTTCCCC	25	<hkxhk>	hh	115	hk	146	kk	0
>ref-34571	CCTCCCCTTACGCCTTCTCCCTTCCCC	26	<hkxhk>	hh	12	hk	250	kk	0
>ref-34589	CACACTCCTACCCCGTCTCCGGCAGGC	14	<nnxnp>	nn	273	np	0		
>ref-34592	CTCTCCCCCACCCCTTCTCCATTCTCC	1	<lmxll>	ll	258	lm	1		
>ref-34612	CCTCCTTCCACCTCCTCTCCTTTCATC	24	<lmxll>	ll	127	lm	130		
>ref-34629	CCTTCTCTCACTCCCACTCCTGTCTCC	21	<hkxhk>	hh	18	hk	149	kk	74
>ref-34644	CGCTCACCAACAGTGTCTCCGCGTGTC	2	<hkxhk>	hh	0	hk	244	kk	0
>ref-34644	CGCTCACCAACAGTGTCTCCGCGTGTC	6	<hkxhk>	hh	0	hk	243	kk	0
>ref-34660	TTCCGGTTGACTCACCCTCCTTCCTCA	5	<nnxnp>	nn	124	np	137		
>ref-34665	AGGCAGTGAACACATCCTCCAAGAGAG	14	<hkxhk>	hh	58	hk	148	kk	59
>ref-34675	CCAGTTCGCACGCCGCCTCCGGCCCTC	1	<nnxnp>	nn	10	np	263		
>ref-34675	CCAGTTCGCACGCCGCCTCCGGCCCTC	3	<hkxhk>	hh	66	hk	202	kk	3
>ref-34675	CCAGTTCGCACGCCGCCTCCGGCCCTC	14	<nnxnp>	nn	10	np	263		
>ref-34693	CGACTTCATACACGCTCTCCTTCCTTC	3	<lmxll>	ll	107	lm	157		
>ref-34693	CGACTTCATACACGCTCTCCTTCCTTC	25	<lmxll>	ll	140	lm	132		
>ref-34706	TGACGTTTAACTGGACCTCCTGAGACT	16	<hkxhk>	hh	62	hk	141	kk	66
>ref-34714	TATTCACCCACACTGTCTCCTTTTCTC	12	<nnxnp>	nn	118	np	124		
>ref-34731	CCATTCTTCACCAACTCTCCATCTCCT	7	<nnxnp>	nn	121	np	141		
>ref-34731	CCATTCTTCACCAACTCTCCATCTCCT	26	<nnxnp>	nn	122	np	140		
>ref-34735	ACACATTTTACTTCTTCTCCTAGCTTG	1	<hkxhk>	hh	29	hk	225	kk	0
>ref-34735	ACACATTTTACTTCTTCTCCTAGCTTG	2	<hkxhk>	hh	0	hk	162	kk	85
>ref-34735	ACACATTTTACTTCTTCTCCTAGCTTG	3	<lmxll>	ll	3	lm	269		
>ref-34735	ACACATTTTACTTCTTCTCCTAGCTTG	4	<hkxhk>	hh	32	hk	223	kk	0
>ref-34735	ACACATTTTACTTCTTCTCCTAGCTTG	24	<nnxnp>	nn	237	np	21		
>ref-34750	GTATCTTTTACTCACACTCCCTTTATT	14	<nnxnp>	nn	248	np	0		
>ref-34760	ACCAAATCAACATGCACTCCCGCACCT	22	<hkxhk>	hh	0	hk	250	kk	0
>ref-34760	ACCAAATCAACATGCACTCCCGCACCT	24	<hkxhk>	hh	1	hk	249	kk	0
>ref-34760	ACCAAATCAACATGCACTCCCGCACCT	25	<hkxhk>	hh	102	hk	144	kk	0
>ref-34811	TCCCTCCCAACTCGCTCTCCATGGCAG	14	<nnxnp>	nn	132	np	135		
>ref-34836	TGATCCTGTACTCCTCCTCCTTCTCTT	8	<hkxhk>	hh	58	hk	141	kk	69
>ref-34852	TGCGTGTCCACACTTCCTCCTTCTCTT	26	<hkxhk>	hh	63	hk	138	kk	61
>ref-34859	CAGGAAATGACCTTCACTCCCATAGAA	13	<hkxhk>	hh	0	hk	267	kk	3
>ref-34859	CAGGAAATGACCTTCACTCCCATAGAA	24	<hkxhk>	hh	3	hk	267	kk	0
>ref-34860	TACAACCCCACCAACCCTCCTTCCCCA	2	<hkxhk>	hh	144	hk	127	kk	1
>ref-34860	TACAACCCCACCAACCCTCCTTCCCCA	3	<hkxhk>	hh	141	hk	24	kk	103
>ref-34860	TACAACCCCACCAACCCTCCTTCCCCA	7	<lmxll>	ll	137	lm	131		
>ref-34860	TACAACCCCACCAACCCTCCTTCCCCA	27	<hkxhk>	hh	119	hk	153	kk	0

>ref-34886	TCGCTATCAACAGCTTCTCCTAAAAAA	25	<lmxll>	ll	122	lm	116		
>ref-34922	CATTCGGCCACACGCTCTCCTTTCCTC	25	<nnxnp>	nn	121	np	137		
>ref-34927	CATTCTCCCACTCTACCTCCCTCTTTC	5	<lmxll>	ll	112	lm	146		
>ref-34930	CCGATATATACTCAAGCTCCGCGCTTA	5	<lmxll>	ll	114	lm	135		
>ref-34961	CAATTGCAAACTTTATCTCCGGGAATG	22	<nnxnp>	nn	129	np	124		
>ref-34976	AGAGCTCCTACAGTTGCTCCTGCTGAG	22	<nnxnp>	nn	128	np	141		
>ref-35012	GGGCTGACCACCCTCCCTCCTCCCCTT	27	<nnxnp>	nn	124	np	117		
>ref-35018	CCCCCCCATACACATCCTCCCCCCTCC	1	<nnxnp>	nn	104	np	152		
>ref-35018	CCCCCCCATACACATCCTCCCCCCTCC	2	<lmxll>	ll	131	lm	128		
>ref-35018	CCCCCCCATACACATCCTCCCCCCTCC	8	<hkxhk>	hh	51	hk	198	kk	9
>ref-35027	TCACGTGGTACACTTGCTCCTGTTGCC	14	<lmxll>	ll	124	lm	144		
>ref-35075	TACTCTCCTACCCTTCCTCCCCCCATC	3	<nnxnp>	nn	128	np	139		
>ref-35100	CTCCCCATCACTCCCCCTCCCCCCTCT	6	<lmxll>	ll	196	lm	56		
>ref-35100	CTCCCCATCACTCCCCCTCCCCCCTCT	9	<nnxnp>	nn	174	np	86		
>ref-35100	CTCCCCATCACTCCCCCTCCCCCCTCT	16	<nnxnp>	nn	169	np	92		
>ref-35120	ACCCAATTCACTCCCACTCCCACTCAC	15	<nnxnp>	nn	262	np	0		
>ref-35128	GCACACATGACTGGAACTCCTGGAAAA	23	<hkxhk>	hh	0	hk	217	kk	27
>ref-35134	CTTCCTCAAACTCCTTCTCCTCTTCTC	4	<nnxnp>	nn	143	np	122		
>ref-35138	TGTGACGTCACTCTCCCTCCCTCCCCC	26	<hkxhk>	hh	0	hk	273	kk	0
>ref-35161	AAGGCTTCTACGCCCTCTCCCCCGTGT	23	<lmxll>	ll	123	lm	147		
>ref-35171	AAGATCCCAACAGCAGCTCCTTTGTTC	1	<hkxhk>	hh	57	hk	147	kk	50
>ref-35174	CTGCCTCCCACTTGTCCTCCCCATGAG	1	<hkxhk>	hh	7	hk	263	kk	0
>ref-35174	CTGCCTCCCACTTGTCCTCCCCATGAG	22	<hkxhk>	hh	2	hk	269	kk	0
>ref-35174	CTGCCTCCCACTTGTCCTCCCCATGAG	27	<hkxhk>	hh	0	hk	259	kk	13
>ref-35178	CCTCGCTTCACACATCCTCCGCCTTCT	1	<hkxhk>	hh	241	hk	29	kk	0
>ref-35178	CCTCGCTTCACACATCCTCCGCCTTCT	3	<nnxnp>	nn	157	np	105		
>ref-35178	CCTCGCTTCACACATCCTCCGCCTTCT	5	<hkxhk>	hh	0	hk	24	kk	244
>ref-35249	AACCAGTCAACTACATCTCCTGTGCTG	14	<nnxnp>	nn	117	np	121		
>ref-35261	CCCACCTTCACCATCACTCCCTCCTCT	4	<hkxhk>	hh	1	hk	267	kk	0
>ref-35276	CTTGCAGATACTCTTCCTCCTTCCTCC	16	<nnxnp>	nn	234	np	0		
>ref-35301	ACCATGGCCACTCTCTCTCCCGGAAAC	2	<hkxhk>	hh	88	hk	155	kk	0
>ref-35301	ACCATGGCCACTCTCTCTCCCGGAAAC	16	<nnxnp>	nn	231	np	17		
>ref-35301	ACCATGGCCACTCTCTCTCCCGGAAAC	21	<hkxhk>	hh	27	hk	220	kk	0
>ref-35301	ACCATGGCCACTCTCTCTCCCGGAAAC	23	<hkxhk>	hh	1	hk	268	kk	0
>ref-35301	ACCATGGCCACTCTCTCTCCCGGAAAC	27	<nnxnp>	nn	273	np	0		
>ref-35302	AAAACACACACGAGGCCTCCGCTAATC	23	<lmxll>	ll	129	lm	132		
>ref-35313	TCTTAAAAAACCTGCGCTCCGACTGCT	3	<hkxhk>	hh	2	hk	256	kk	2
>ref-35313	TCTTAAAAAACCTGCGCTCCGACTGCT	9	<hkxhk>	hh	2	hk	258	kk	2

>ref-35317	TTCGATATGACTCAATCTCCTTTCTTT	5	<lmxll>	ll	139	lm	116		
>ref-35322	CCCTTCCTGACTTGGGCTCCGGCCTAT	25	<nnxnp>	nn	131	np	122		
>ref-35380	GCCAACTCTACTTGAGCTCCTTCCAAC	9	<nnxnp>	nn	129	np	140		
>ref-35384	GAAGAGGGTACAGGCCCTCCTCCCCGC	9	<lmxll>	ll	131	lm	134		
>ref-35385	GTCCGTTCGACGTTCCCTCCGCCGCAG	4	<nnxnp>	nn	144	np	125		
>ref-35386	TTGCCATGCACCACTCCTCCTGTGCTG	25	<lmxll>	ll	138	lm	130		
>ref-35419	TTCCACCCGACCCGCTCTCCGCCACAC	14	<hkxhk>	hh	12	hk	251	kk	0
>ref-35456	TCCCTTACCACAATACCTCCTTTATTT	9	<nnxnp>	nn	144	np	119		
>ref-35456	TCCCTTACCACAATACCTCCTTTATTT	24	<nnxnp>	nn	145	np	119		
>ref-35458	CTCACTCCAACCTTCCCTCCCTCCACC	4	<lmxll>	ll	71	lm	158		
>ref-35472	TAATATGCAACTGTGTCTCCAGCCTAG	22	<lmxll>	ll	133	lm	128		
>ref-35476	GCTGGGCTGACACGCCCTCCATTGTCT	6	<nnxnp>	nn	139	np	128		
>ref-35478	TCTCTTATCACCTTTTCTCCCCCGCTA	15	<nnxnp>	nn	123	np	131		
>ref-35478	TCTCTTATCACCTTTTCTCCCCCGCTA	23	<lmxll>	ll	122	lm	134		
>ref-35501	CTCTCCAACACCCTTCCTCCTTCGCTG	1	<hkxhk>	hh	83	hk	182	kk	0
>ref-35517	TTCGCCATCACTTTCCCTCCTCCTCTT	5	<hkxhk>	hh	7	hk	262	kk	0
>ref-35550	AACACACCCACAGGATCTCCGCGTCTG	12	<nnxnp>	nn	135	np	122		
>ref-35552	CTGTGAGCCACACTCACTCCTTCATGT	25	<nnxnp>	nn	123	np	123		
>ref-35556	AAACACAAAACACATCCTCCCACACAC	15	<nnxnp>	nn	122	np	143		
>ref-35640	CTACCATGTACACCACCTCCTACACCA	21	<hkxhk>	hh	59	hk	140	kk	66
>ref-35644	CAGTCTCTCACTCTCCCTCCCTCACCT	16	<lmxll>	ll	214	lm	39		
>ref-35644	CAGTCTCTCACTCTCCCTCCCTCACCT	25	<lmxll>	ll	269	lm	0		
>ref-35649	TTTCCTCTTACTCTTGCTCCCCTCCCT	12	<nnxnp>	nn	127	np	109		
>ref-35654	TGTAAACCAACCACGCCTCCCCACGTT	21	<hkxhk>	hh	52	hk	141	kk	66
>ref-35654	TGTAAACCAACCACGCCTCCCCACGTT	22	<hkxhk>	hh	66	hk	141	kk	52
>ref-35673	GCAAGTGTAACCTCTCCTCCTCCTCCT	27	<nnxnp>	nn	137	np	117		
>ref-35704	CTAGTTCCCACTCAGTCTCCGTCCTCC	9	<hkxhk>	hh	60	hk	146	kk	51
>ref-35715	GTTGTTATAACCCTCTCTCCCTCCCCT	9	<hkxhk>	hh	0	hk	267	kk	0
>ref-35715	GTTGTTATAACCCTCTCTCCCTCCCCT	24	<hkxhk>	hh	0	hk	267	kk	0
>ref-35716	AGGAGGAAGACATTTTCTCCCTCACCC	6	<lmxll>	ll	110	lm	111		
>ref-35721	ACCTGCCCGACGCCGGCTCCTGCGGCA	24	<nnxnp>	nn	149	np	121		
>ref-35737	CCCAACCCTACTTAATCTCCACATCTT	23	<lmxll>	ll	134	lm	129		
>ref-35740	CGACCTGCCACGCCTCCTCCTTCTTCA	23	<nnxnp>	nn	138	np	133		
>ref-35742	TCACACTTTACCCCAACTCCTCTTCCA	5	<hkxhk>	hh	5	hk	255	kk	1
>ref-35766	CTTGGATGGACAGGGCCTCCGCCTTTT	12	<nnxnp>	nn	58	np	206		
>ref-35766	CTTGGATGGACAGGGCCTCCGCCTTTT	15	<nnxnp>	nn	57	np	206		
>ref-35769	TATAAACCCACACCTCCTCCCTCTTCC	12	<nnxnp>	nn	244	np	0		
>ref-35779	CACCCAGCCACACCCCCTCCTTACGTC	16	<nnxnp>	nn	129	np	131		

>ref-35784	TTAGAATTGACGCCACCTCCCTCTTCC	4	<hkxhk>	hh	66	hk	183	kk	0
>ref-35789	CTGCCTCCAACTTCCCCTCCTCCCCCC	9	<lmxll>	ll	234	lm	4		
>ref-35799	AACCTTTCTACTTCTCCTCCTCTCTTT	1	<hkxhk>	hh	22	hk	164	kk	33
>ref-35816	GTGCCTCCAACTTCGTCTCCCTCGCAG	9	<nnxnp>	nn	115	np	146		
>ref-35825	CTCGTCCCCACTTATCCTCCCTTCTCC	8	<hkxhk>	hh	47	hk	161	kk	52
>ref-35845	CCCGACACAACAGAGTCTCCTCGTCAT	2	<lmxll>	ll	135	lm	132		
>ref-35846	AGCCTATATACTATCTCTCCAGGCAGT	26	<lmxll>	ll	127	lm	122		
>ref-35857	CGCTCAACCACCAGGACTCCCGTCTCC	2	<hkxhk>	hh	0	hk	273	kk	0
>ref-35857	CGCTCAACCACCAGGACTCCCGTCTCC	21	<hkxhk>	hh	0	hk	273	kk	0
>ref-35865	TTCCCTCCTACCTCTCCTCCTCTCCTC	16	<lmxll>	ll	3	lm	256		
>ref-35865	TTCCCTCCTACCTCTCCTCCTCTCCTC	26	<lmxll>	ll	3	lm	257		
>ref-35872	CAGAGCTGAACTCTCCCTCCCTCCGTC	3	<lmxll>	ll	127	lm	130		
>ref-35872	CAGAGCTGAACTCTCCCTCCCTCCGTC	15	<lmxll>	ll	125	lm	130		
>ref-35882	CACAAACATACATACACTCCATTACAT	13	<lmxll>	ll	132	lm	124		
>ref-35891	ATCGTGCAGACCAATCCTCCTCTTGAA	3	<lmxll>	ll	18	lm	251		
>ref-35894	CACTGTGCCACTATCACTCCTTGCTAC	9	<hkxhk>	hh	0	hk	112	kk	118
>ref-35894	CACTGTGCCACTATCACTCCTTGCTAC	16	<hkxhk>	hh	0	hk	111	kk	117
>ref-35909	TCAGCGCCCACACCAGCTCCGAGGACC	25	<lmxll>	ll	141	lm	121		
>ref-35911	TCTCCCTTCACTCTTACTCCCTCCTCT	2	<hkxhk>	hh	0	hk	271	kk	0
>ref-35911	TCTCCCTTCACTCTTACTCCCTCCTCT	14	<hkxhk>	hh	0	hk	271	kk	0
>ref-35946	TGACCAGGCACATCCTCTCCGGACACA	13	<hkxhk>	hh	0	hk	273	kk	0
>ref-36010	CACACACATACCCCAACTCCCACAGCC	2	<lmxll>	ll	110	lm	127		
>ref-36040	AGAAAGTTAACTCTTCCTCCTCCCTCT	5	<nnxnp>	nn	127	np	132		
>ref-36059	GCAATCGAAACACGTTCTCCTTGTGAA	8	<lmxll>	ll	111	lm	140		
>ref-36087	TTCATGCACACTTTCTCTCCTCCTATT	25	<lmxll>	ll	114	lm	148		
>ref-36090	CTCACGCCCACCCACACTCCAACCCTC	2	<nnxnp>	nn	133	np	119		
>ref-36090	CTCACGCCCACCCACACTCCAACCCTC	14	<hkxhk>	hh	55	hk	196	kk	2
>ref-36090	CTCACGCCCACCCACACTCCAACCCTC	22	<hkxhk>	hh	57	hk	195	kk	2
>ref-36090	CTCACGCCCACCCACACTCCAACCCTC	27	<hkxhk>	hh	69	hk	174	kk	1
>ref-36107	TGTCATGAAACTCTTCCTCCTCTTCAT	9	<nnxnp>	nn	126	np	126		
>ref-36110	ACCAACGCTACCCCGTCTCCACCACCG	7	<nnxnp>	nn	143	np	128		
>ref-36110	ACCAACGCTACCCCGTCTCCACCACCG	8	<nnxnp>	nn	143	np	128		
>ref-36123	CTTCCCCTCACCTCACCTCCCTTGTTT	1	<nnxnp>	nn	259	np	2		
>ref-36123	CTTCCCCTCACCTCACCTCCCTTGTTT	6	<nnxnp>	nn	268	np	0		
>ref-36126	CTAACTCCAACTCGAACTCCGCCTCCG	24	<lmxll>	ll	102	lm	118		
>ref-36132	ACAAAACCAACCGAAACTCCAGTCGTC	13	<nnxnp>	nn	108	np	141		
>ref-36152	CCTGACCCTACGCTTCCTCCCCCCCAC	7	<nnxnp>	nn	255	np	0		
>ref-36152	CCTGACCCTACGCTTCCTCCCCCCCAC	23	<nnxnp>	nn	121	np	119		

>ref-36157	TGGTCGTGGACAACGGCTCCGGCCTGG	2	<hkxhk>	hh	0	hk	271	kk	0
>ref-36157	TGGTCGTGGACAACGGCTCCGGCCTGG	26	<hkxhk>	hh	0	hk	271	kk	0
>ref-36167	CTCAGTCACACACTCTCTCCCTCGCTT	3	<lmxll>	ll	8	lm	265		
>ref-36167	CTCAGTCACACACTCTCTCCCTCGCTT	5	<lmxll>	ll	8	lm	265		
>ref-36173	TATCTGTCTACGTGTACTCCTAGATTT	15	<hkxhk>	hh	0	hk	220	kk	26
>ref-36173	TATCTGTCTACGTGTACTCCTAGATTT	24	<hkxhk>	hh	0	hk	268	kk	0
>ref-36173	TATCTGTCTACGTGTACTCCTAGATTT	27	<hkxhk>	hh	0	hk	270	kk	1
>ref-36182	TCACATTTCACTGTCTCTCCTCCCCAA	24	<hkxhk>	hh	53	hk	143	kk	54
>ref-36186	TTTTCGTGTACTTCTTCTCCTTCGCCT	2	<nnxnp>	nn	119	np	100		
>ref-36186	TTTTCGTGTACTTCTTCTCCTTCGCCT	3	<nnxnp>	nn	247	np	0		
>ref-36186	TTTTCGTGTACTTCTTCTCCTTCGCCT	24	<nnxnp>	nn	237	np	4		
>ref-36196	CCTTTTTCTACTCACACTCCCTCTATC	1	<lmxll>	ll	115	lm	111		
>ref-36225	AATAATACCACATACCCTCCCTCATAT	26	<hkxhk>	hh	6	hk	227	kk	6
>ref-36269	TACACCCTCACCCCCACTCCCTCCCTT	21	<lmxll>	ll	121	lm	112		
>ref-36286	TATCTCACAACACACCCTCCTTGTCTT	7	<nnxnp>	nn	127	np	139		
>ref-36286	TATCTCACAACACACCCTCCTTGTCTT	23	<lmxll>	ll	136	lm	130		
>ref-36299	ACTTCCTCTACCTCACCTCCACCCTAA	26	<lmxll>	ll	114	lm	135		
>ref-36302	TCGCTCTCCACCCACTCTCCCCTCTCT	27	<lmxll>	ll	4	lm	260		
>ref-36320	TTCCCCAGCACTCTGACTCCTTTCTCC	8	<lmxll>	ll	258	lm	0		
>ref-36320	TTCCCCAGCACTCTGACTCCTTTCTCC	26	<lmxll>	ll	259	lm	0		
>ref-36359	CCTATAGCTACATTTTCTCCACCATCA	4	<lmxll>	ll	123	lm	142		
>ref-36394	GAGCATTTAACATATGCTCCACCTGGA	3	<lmxll>	ll	178	lm	56		
>ref-36394	GAGCATTTAACATATGCTCCACCTGGA	12	<nnxnp>	nn	77	np	177		
>ref-36394	GAGCATTTAACATATGCTCCACCTGGA	22	<lmxll>	ll	103	lm	133		
>ref-36402	TGAATCCTAACGCATTCTCCTCTTCCC	26	<hkxhk>	hh	60	hk	147	kk	48
>ref-36429	GGTATATATACTACGGCTCCGGTGCCA	2	<lmxll>	ll	243	lm	4		
>ref-36456	ATCCTAGTAACATATGCTCCCACTGCG	4	<lmxll>	ll	18	lm	245		
>ref-36456	ATCCTAGTAACATATGCTCCCACTGCG	9	<hkxhk>	hh	33	hk	235	kk	0
>ref-36456	ATCCTAGTAACATATGCTCCCACTGCG	12	<hkxhk>	hh	14	hk	244	kk	0
>ref-36456	ATCCTAGTAACATATGCTCCCACTGCG	15	<lmxll>	ll	200	lm	54		
>ref-36456	ATCCTAGTAACATATGCTCCCACTGCG	16	<lmxll>	ll	45	lm	210		
>ref-36458	CCCTTACGCACGTCTTCTCCCCTCTTC	8	<hkxhk>	hh	48	hk	133	kk	74
>ref-36471	CTTTCCCCAACCTCTCCTCCAACTCCC	2	<hkxhk>	hh	12	hk	241	kk	0
>ref-36471	CTTTCCCCAACCTCTCCTCCAACTCCC	3	<lmxll>	ll	55	lm	186		
>ref-36476	CATCTTACCACTCTCTCTCCCCTTCTC	2	<hkxhk>	hh	0	hk	273	kk	0
>ref-36476	CATCTTACCACTCTCTCTCCCCTTCTC	3	<lmxll>	ll	16	lm	242		
>ref-36476	CATCTTACCACTCTCTCTCCCCTTCTC	13	<nnxnp>	nn	163	np	105		
>ref-36484	CAGCACCACACAGAAACTCCGGCGCTT	21	<lmxll>	ll	92	lm	143		

>ref-36513	CCTACTCTTACACACCCTCCTCCTTGC	9	<hkxhk>	hh	45	hk	144	kk	71
>ref-36513	CCTACTCTTACACACCCTCCTCCTTGC	27	<nnxnp>	nn	272	np	0		
>ref-36521	CCAACGGAGACGCACCCTCCTGCAGCT	3	<hkxhk>	hh	0	hk	252	kk	0
>ref-36521	CCAACGGAGACGCACCCTCCTGCAGCT	5	<hkxhk>	hh	226	hk	23	kk	0
>ref-36521	CCAACGGAGACGCACCCTCCTGCAGCT	14	<hkxhk>	hh	9	hk	242	kk	0
>ref-36521	CCAACGGAGACGCACCCTCCTGCAGCT	15	<hkxhk>	hh	198	hk	43	kk	0
>ref-36526	ACACACACTACTGTCGCTCCTTTCCCC	3	<nnxnp>	nn	252	np	5		
>ref-36526	ACACACACTACTGTCGCTCCTTTCCCC	27	<nnxnp>	nn	252	np	0		
>ref-36531	AACAAACAAACCCACCCTCCCTACCAA	15	<hkxhk>	hh	14	hk	254	kk	0
>ref-36531	AACAAACAAACCCACCCTCCCTACCAA	22	<hkxhk>	hh	0	hk	159	kk	93
>ref-36531	AACAAACAAACCCACCCTCCCTACCAA	24	<hkxhk>	hh	0	hk	159	kk	93
>ref-36549	CCTTTCCATACCCCCTCTCCCTCCCAT	15	<nnxnp>	nn	140	np	116		
>ref-36550	CCATCTGTCACCTCACCTCCCTTTTCC	5	<nnxnp>	nn	259	np	0		
>ref-36550	CCATCTGTCACCTCACCTCCCTTTTCC	13	<nnxnp>	nn	121	np	130		
>ref-36563	ACACTCTCCACCCCCCCTCCCACACAC	4	<lmxll>	ll	55	lm	203		
>ref-36563	ACACTCTCCACCCCCCCTCCCACACAC	5	<lmxll>	ll	54	lm	204		
>ref-36572	CACTCAGACACATTTCCTCCCTCATAC	15	<nnxnp>	nn	113	np	142		
>ref-36576	ACATTTATCACTCCCCCTCCCCCATGG	4	<hkxhk>	hh	2	hk	265	kk	2
>ref-36576	ACATTTATCACTCCCCCTCCCCCATGG	12	<hkxhk>	hh	0	hk	142	kk	108
>ref-36592	CCTTCCCCCACCCTCGCTCCTTACCCT	21	<lmxll>	ll	136	lm	125		
>ref-36600	AAACACTAAACAAAGCCTCCGCCTCGA	24	<lmxll>	ll	139	lm	127		
>ref-36633	CTCCTTCCAACCCACCCTCCCTTCCCT	6	<hkxhk>	hh	3	hk	254	kk	4
>ref-36633	CTCCTTCCAACCCACCCTCCCTTCCCT	14	<lmxll>	ll	202	lm	25		
>ref-36633	CTCCTTCCAACCCACCCTCCCTTCCCT	15	<lmxll>	ll	123	lm	140		
>ref-36633	CTCCTTCCAACCCACCCTCCCTTCCCT	21	<hkxhk>	hh	3	hk	254	kk	3
>ref-36633	CTCCTTCCAACCCACCCTCCCTTCCCT	22	<lmxll>	ll	226	lm	21		
>ref-36639	CCCAATCCCACTTCCCCTCCTACCCTC	2	<nnxnp>	nn	267	np	0		
>ref-36639	CCCAATCCCACTTCCCCTCCTACCCTC	22	<nnxnp>	nn	264	np	1		
>ref-36666	ATTGCGTTCACTCGGGCTCCCAATTCC	15	<hkxhk>	hh	0	hk	262	kk	9
>ref-36666	ATTGCGTTCACTCGGGCTCCCAATTCC	22	<hkxhk>	hh	53	hk	210	kk	0
>ref-36666	ATTGCGTTCACTCGGGCTCCCAATTCC	26	<hkxhk>	hh	41	hk	227	kk	0
>ref-36670	TTCGATTTCACTATCTCTCCTTTCTCT	13	<lmxll>	ll	108	lm	146		
>ref-36670	TTCGATTTCACTATCTCTCCTTTCTCT	14	<lmxll>	ll	108	lm	146		
>ref-36679	TCTGCTTCTACTTCTGCTCCCTTTCTT	5	<nnxnp>	nn	114	np	133		
>ref-36701	ACTCTCGCTACCCTCCCTCCCTCCCCC	1	<nnxnp>	nn	45	np	178		
>ref-36702	CGAGGGGACACTGGAGCTCCTGTGTGC	4	<lmxll>	ll	36	lm	186		
>ref-36702	CGAGGGGACACTGGAGCTCCTGTGTGC	5	<lmxll>	ll	36	lm	186		
>ref-36702	CGAGGGGACACTGGAGCTCCTGTGTGC	6	<lmxll>	ll	35	lm	186		

>ref-36702	CGAGGGGACACTGGAGCTCCTGTGTGC	7	<lmxll>	ll	36	lm	186		
>ref-36702	CGAGGGGACACTGGAGCTCCTGTGTGC	8	<lmxll>	ll	36	lm	186		
>ref-36702	CGAGGGGACACTGGAGCTCCTGTGTGC	9	<lmxll>	ll	36	lm	186		
>ref-36702	CGAGGGGACACTGGAGCTCCTGTGTGC	11	<lmxll>	ll	36	lm	186		
>ref-36702	CGAGGGGACACTGGAGCTCCTGTGTGC	12	<lmxll>	ll	36	lm	186		
>ref-36702	CGAGGGGACACTGGAGCTCCTGTGTGC	13	<lmxll>	ll	36	lm	186		
>ref-36702	CGAGGGGACACTGGAGCTCCTGTGTGC	14	<lmxll>	ll	36	lm	186		
>ref-36702	CGAGGGGACACTGGAGCTCCTGTGTGC	15	<lmxll>	ll	36	lm	186		
>ref-36702	CGAGGGGACACTGGAGCTCCTGTGTGC	16	<lmxll>	ll	36	lm	187		
>ref-36702	CGAGGGGACACTGGAGCTCCTGTGTGC	17	<lmxll>	ll	36	lm	186		
>ref-36702	CGAGGGGACACTGGAGCTCCTGTGTGC	19	<lmxll>	ll	36	lm	186		
>ref-36702	CGAGGGGACACTGGAGCTCCTGTGTGC	20	<lmxll>	ll	36	lm	186		
>ref-36702	CGAGGGGACACTGGAGCTCCTGTGTGC	21	<lmxll>	ll	36	lm	186		
>ref-36702	CGAGGGGACACTGGAGCTCCTGTGTGC	22	<lmxll>	ll	36	lm	186		
>ref-36702	CGAGGGGACACTGGAGCTCCTGTGTGC	23	<lmxll>	ll	36	lm	187		
>ref-36702	CGAGGGGACACTGGAGCTCCTGTGTGC	25	<lmxll>	ll	35	lm	186		
>ref-36715	AACGAGGCCACGAATTCTCCCACTTAT	8	<hkxhk>	hh	12	hk	221	kk	1
>ref-36715	AACGAGGCCACGAATTCTCCCACTTAT	9	<hkxhk>	hh	1	hk	221	kk	12
>ref-36727	GCACGCACCACACCCCCTCCACACAAG	8	<nnxnp>	nn	260	np	0		
>ref-36741	TCCTTAACGACCATCACTCCTGCGATA	21	<lmxll>	ll	126	lm	146		
>ref-36741	TCCTTAACGACCATCACTCCTGCGATA	23	<lmxll>	ll	132	lm	135		
>ref-36751	CCCCTCCCAACCTTTCCTCCATTTCCC	14	<lmxll>	ll	27	lm	204		
>ref-36765	CCTTCTTCCACATTTTCTCCTTTCCCC	8	<lmxll>	ll	12	lm	228		
>ref-36770	TCTGTTGCAACTTCGTCTCCTTTTTAT	6	<nnxnp>	nn	126	np	103		
>ref-36813	TTCCATGTAACCACATCTCCACCATGG	1	<lmxll>	ll	120	lm	129		
>ref-36816	ATGTGTCTCACTCCTCCTCCTCCTTCT	23	<nnxnp>	nn	130	np	140		
>ref-36824	CACAAACGTACACCCGCTCCTCCCCTT	9	<nnxnp>	nn	130	np	141		
>ref-36825	AAAAGAGTCACACGCCCTCCCTTCATC	23	<nnxnp>	nn	258	np	0		
>ref-36843	CATAAGCACACGCACACTCCACACTAC	4	<lmxll>	ll	128	lm	138		
>ref-36843	CATAAGCACACGCACACTCCACACTAC	5	<lmxll>	ll	128	lm	138		
>ref-36850	CCCTTCCCAACACACCCTCCCCTCCCT	1	<nnxnp>	nn	82	np	167		
>ref-36850	CCCTTCCCAACACACCCTCCCCTCCCT	6	<hkxhk>	hh	2	hk	269	kk	0
>ref-36850	CCCTTCCCAACACACCCTCCCCTCCCT	9	<hkxhk>	hh	1	hk	270	kk	0
>ref-36850	CCCTTCCCAACACACCCTCCCCTCCCT	14	<nnxnp>	nn	77	np	170		
>ref-36855	AGTTAATTGACCCATTCTCCTGCTTTC	23	<lmxll>	ll	142	lm	92		
>ref-36880	TCCCTCCCCACATCTCCTCCGACTCCG	1	<lmxll>	ll	116	lm	137		
>ref-36889	TCATCAGAGACTAAGTCTCCAACTCCA	4	<hkxhk>	hh	0	hk	273	kk	0
>ref-36889	TCATCAGAGACTAAGTCTCCAACTCCA	16	<hkxhk>	hh	0	hk	257	kk	10

>ref-36905	AGAGGAACCACTCGATCTCCTGCCACG	12	<nnxnp>	nn	127	np	140		
>ref-36908	TTCTCCAACACCCATTCTCCAACACTC	2	<hkxhk>	hh	0	hk	270	kk	2
>ref-36908	TTCTCCAACACCCATTCTCCAACACTC	12	<hkxhk>	hh	0	hk	268	kk	0
>ref-36908	TTCTCCAACACCCATTCTCCAACACTC	14	<hkxhk>	hh	0	hk	269	kk	0
>ref-36908	TTCTCCAACACCCATTCTCCAACACTC	16	<lmxll>	ll	117	lm	138		
>ref-36917	CTCTTCTCAACTCCTCCTCCTTCATCA	22	<nnxnp>	nn	121	np	135		
>ref-36924	GTCCTTATTACTTCGGCTCCTCCTGCG	21	<lmxll>	ll	121	lm	132		
>ref-36937	CCGTCCACCACATAACCTCCAACATCA	15	<lmxll>	ll	112	lm	159		
>ref-36939	TCTTCCCACACCCCCCCTCCCCGTACC	25	<nnxnp>	nn	112	np	121		
>ref-36949	TGTCCTATTACTTGTCCTCCTACACCA	24	<hkxhk>	hh	1	hk	269	kk	0
>ref-36953	GTAAGTCTCACCTTCACTCCATTCATT	3	<lmxll>	ll	124	lm	141		
>ref-36975	TCCAGAGACACTGCATCTCCTTTAACA	7	<hkxhk>	hh	0	hk	255	kk	7
>ref-36981	TGCACACGCACATCACCTCCGCGGATT	9	<nnxnp>	nn	124	np	143		
>ref-36985	GCACACATAACCCCTTCTCCCTCTCTC	15	<nnxnp>	nn	95	np	136		
>ref-37020	TGCGTGGAAACAAAGCCTCCTTGCAGT	25	<nnxnp>	nn	138	np	135		
>ref-37036	CTTTCTCTTACTCCCTCTCCTTCCCAC	4	<lmxll>	ll	84	lm	171		
>ref-37036	CTTTCTCTTACTCCCTCTCCTTCCCAC	15	<lmxll>	ll	160	lm	98		
>ref-37072	CTCTCACTTACTTTCCCTCCCTCCCCA	26	<nnxnp>	nn	87	np	155		
>ref-37077	ACAACAACAACCACCACTCCAACCACA	3	<hkxhk>	hh	10	hk	255	kk	1
>ref-37077	ACAACAACAACCACCACTCCAACCACA	24	<hkxhk>	hh	1	hk	256	kk	10
>ref-37081	ATGGCACCTACGTCCCCTCCCCAAGCT	4	<nnxnp>	nn	112	np	151		
>ref-37082	TCTTCTCAAACTCGCGCTCCTGAACCA	24	<nnxnp>	nn	105	np	142		
>ref-37120	TACGCTCCTACGCCCACTCCCACGCTC	1	<nnxnp>	nn	164	np	105		
>ref-37120	TACGCTCCTACGCCCACTCCCACGCTC	3	<nnxnp>	nn	152	np	121		
>ref-37120	TACGCTCCTACGCCCACTCCCACGCTC	7	<nnxnp>	nn	167	np	104		
>ref-37120	TACGCTCCTACGCCCACTCCCACGCTC	21	<hkxhk>	hh	5	hk	266	kk	0
>ref-37120	TACGCTCCTACGCCCACTCCCACGCTC	24	<nnxnp>	nn	166	np	105		
>ref-37128	AGGAGTCGGACTCAATCTCCCTGTTCC	26	<hkxhk>	hh	50	hk	142	kk	51
>ref-37132	CCTCCTCCCACCCTACCTCCCCATTCT	2	<lmxll>	ll	245	lm	21		
>ref-37132	CCTCCTCCCACCCTACCTCCCCATTCT	3	<lmxll>	ll	247	lm	22		
>ref-37132	CCTCCTCCCACCCTACCTCCCCATTCT	13	<nnxnp>	nn	194	np	64		
>ref-37134	CCTCCTCCAACAGCCACTCCATGTCCT	21	<hkxhk>	hh	57	hk	164	kk	1
>ref-37134	CCTCCTCCAACAGCCACTCCATGTCCT	23	<hkxhk>	hh	1	hk	160	kk	65
>ref-37167	CTCTGCTTCACCCTCCCTCCCCCATTT	5	<hkxhk>	hh	1	hk	260	kk	3
>ref-37167	CTCTGCTTCACCCTCCCTCCCCCATTT	15	<hkxhk>	hh	1	hk	260	kk	3
>ref-37172	TTTGCAACAACATCGGCTCCAAGAAGT	14	<hkxhk>	hh	65	hk	117	kk	37
>ref-37183	GGTAAGGTCACAAGGTCTCCTTTGTGG	2	<hkxhk>	hh	21	hk	245	kk	0
>ref-37183	GGTAAGGTCACAAGGTCTCCTTTGTGG	4	<lmxll>	ll	163	lm	94		

>ref-37183	GGTAAGGTCACAAGGTCTCCTTTGTGG	5	<hkxhk>	hh	0	hk	245	kk	21
>ref-37192	TGCCAGGTAACCCCTGCTCCCAGACCG	3	<hkxhk>	hh	1	hk	265	kk	0
>ref-37192	TGCCAGGTAACCCCTGCTCCCAGACCG	5	<nnxnp>	nn	143	np	118		
>ref-37192	TGCCAGGTAACCCCTGCTCCCAGACCG	26	<hkxhk>	hh	0	hk	265	kk	1
>ref-37194	TGACAGCGCACTCGTCCTCCCAACCCT	25	<nnxnp>	nn	138	np	133		
>ref-37198	CGGCCGTCCACCGCTGCTCCTGCGCCT	7	<lmxll>	ll	169	lm	104		
>ref-37198	CGGCCGTCCACCGCTGCTCCTGCGCCT	23	<nnxnp>	nn	110	np	162		
>ref-37201	TCACGTCTGACCCTCCCTCCATCTTTT	3	<nnxnp>	nn	108	np	136		
>ref-37205	AAATCACACACAATAACTCCTGAGCTA	25	<lmxll>	ll	123	lm	145		
>ref-37212	CTCTCTCAAACTCTTTCTCCTTCCTAC	15	<lmxll>	ll	232	lm	4		
>ref-37215	CATGCATAAACGCACCCTCCCCCTCAC	8	<lmxll>	ll	117	lm	145		
>ref-37215	CATGCATAAACGCACCCTCCCCCTCAC	12	<lmxll>	ll	117	lm	145		
>ref-37228	CACAGACAAACAAAGACTCCCATTCAT	1	<hkxhk>	hh	47	hk	155	kk	61
>ref-37228	CACAGACAAACAAAGACTCCCATTCAT	6	<hkxhk>	hh	47	hk	155	kk	60
>ref-37248	GTTTTAAGCACGTACTCTCCCTTCCCT	8	<nnxnp>	nn	127	np	125		
>ref-37263	GGCTTTTTAACTCTCTCTCCCCCTCTC	5	<hkxhk>	hh	3	hk	267	kk	1
>ref-37263	GGCTTTTTAACTCTCTCTCCCCCTCTC	7	<hkxhk>	hh	3	hk	267	kk	1
>ref-37336	CATCTCTTCACTCCCTCTCCCTCATTA	3	<nnxnp>	nn	3	np	262		
>ref-37336	CATCTCTTCACTCCCTCTCCCTCATTA	14	<nnxnp>	nn	3	np	262		
>ref-37336	CATCTCTTCACTCCCTCTCCCTCATTA	23	<nnxnp>	nn	156	np	108		
>ref-37351	CTATCCCCTACCTCCTCTCCTTTTCTC	24	<nnxnp>	nn	105	np	128		
>ref-37359	TTCAGACGAACGCTCACTCCATCGCCC	7	<lmxll>	ll	118	lm	151		
>ref-37400	CTCCGTCTAACTTCCTCTCCACGCACA	24	<nnxnp>	nn	134	np	133		
>ref-37402	CCCCTCTCGACTGTGTCTCCTTTCTCG	1	<nnxnp>	nn	240	np	0		
>ref-37402	CCCCTCTCGACTGTGTCTCCTTTCTCG	3	<nnxnp>	nn	240	np	0		
>ref-37416	TATTCTACTACTTCCACTCCTCCTCCC	9	<lmxll>	ll	124	lm	109		
>ref-37421	ACATATCCTACCTACCCTCCCATACCC	4	<nnxnp>	nn	104	np	158		
>ref-37421	ACATATCCTACCTACCCTCCCATACCC	24	<nnxnp>	nn	104	np	158		
>ref-37432	ATCATGATAACTGCATCTCCCTACCTT	5	<nnxnp>	nn	129	np	119		
>ref-37451	CATCACTACACTCCTTCTCCTCCTTCT	14	<hkxhk>	hh	69	hk	140	kk	47
>ref-37455	CTTCCCATAACATCCCCTCCCTTCCCT	8	<nnxnp>	nn	96	np	171		
>ref-37461	AGGTGGCGGACACTGTCTCCCTTCTCT	1	<lmxll>	ll	143	lm	125		
>ref-37461	AGGTGGCGGACACTGTCTCCCTTCTCT	23	<hkxhk>	hh	52	hk	134	kk	74
>ref-37462	CACAGAAGCACTGCATCTCCCTAAGTT	2	<nnxnp>	nn	119	np	113		
>ref-37494	TCCTCTCTGACCCCCTCTCCCCCATCC	4	<hkxhk>	hh	0	hk	244	kk	11
>ref-37494	TCCTCTCTGACCCCCTCTCCCCCATCC	9	<hkxhk>	hh	0	hk	246	kk	11
>ref-37509	CACACATGAACTGTATCTCCCTTATAA	14	<hkxhk>	hh	56	hk	123	kk	60
>ref-37509	CACACATGAACTGTATCTCCCTTATAA	22	<hkxhk>	hh	56	hk	123	kk	60

>ref-37531	ACCCAAATAACAAGTACTCCCAAATAA	7	<hkxhk>	hh	0	hk	252	kk	9
>ref-37544	CCTTTAGCCACTGAAACTCCCTTACAG	23	<nnxnp>	nn	113	np	115		
>ref-37555	TGGCGCGCCACAGAAACTCCGTAGCGT	2	<hkxhk>	hh	0	hk	250	kk	8
>ref-37555	TGGCGCGCCACAGAAACTCCGTAGCGT	9	<hkxhk>	hh	8	hk	250	kk	0
>ref-37565	ATCATCCATACTTCCTCTCCGCTGCAC	22	<lmxll>	ll	138	lm	125		
>ref-37574	TTCCTCATAACCTTTCCTCCTTTCCTC	23	<hkxhk>	hh	0	hk	273	kk	0
>ref-37574	TTCCTCATAACCTTTCCTCCTTTCCTC	24	<hkxhk>	hh	15	hk	248	kk	0
>ref-37579	TGACAGGTGACACCCTCTCCTCTGTGA	5	<lmxll>	ll	119	lm	145		
>ref-37592	CTTCTCTTCACTCCCTCTCCCTTCCTT	16	<hkxhk>	hh	0	hk	269	kk	3
>ref-37592	CTTCTCTTCACTCCCTCTCCCTTCCTT	26	<hkxhk>	hh	0	hk	269	kk	3
>ref-37616	CTCCTATCCACTCTATCTCCTCTCTTT	1	<lmxll>	ll	17	lm	248		
>ref-37616	CTCCTATCCACTCTATCTCCTCTCTTT	7	<lmxll>	ll	0	lm	271		
>ref-37617	TTCTCAAAAACCTCATCTCCACTGTCT	24	<nnxnp>	nn	141	np	105		
>ref-37633	TGAACACCAACTACCGCTCCCAAGATC	22	<lmxll>	ll	123	lm	108		
>ref-37655	TTCCCTCCCACTGACCCTCCTTGCTTC	26	<nnxnp>	nn	128	np	134		
>ref-37659	CTTTACCCAACTCCTTCTCCCTTTCTC	16	<nnxnp>	nn	124	np	132		
>ref-37672	ACCACTACTACTATTACTCCAGTTCTA	6	<nnxnp>	nn	114	np	118		
>ref-37692	CCTCCTCACACGACCTCTCCAGAGCTG	23	<hkxhk>	hh	45	hk	152	kk	61
>ref-37702	TCCCTCTTCACTACCCCTCCCTCTCCT	1	<lmxll>	ll	166	lm	84		
>ref-37702	TCCCTCTTCACTACCCCTCCCTCTCCT	8	<lmxll>	ll	164	lm	85		
>ref-37721	TCCACGAGTACTTCCCCTCCAGGCCCC	4	<hkxhk>	hh	46	hk	131	kk	76
>ref-37731	AGTAAATTCACGAAATCTCCGAGTGAA	23	<hkxhk>	hh	0	hk	200	kk	36
>ref-37731	AGTAAATTCACGAAATCTCCGAGTGAA	25	<hkxhk>	hh	0	hk	163	kk	69
>ref-37735	CATTACCTAACTCATACTCCTAGAGGT	21	<lmxll>	ll	135	lm	111		
>ref-37740	CCTCCCCACACCTCATCTCCCCATCCG	1	<nnxnp>	nn	241	np	0		
>ref-37752	CCCTCTCCAACCGCTGCTCCCGTCCTT	27	<lmxll>	ll	108	lm	137		
>ref-37761	CTCCCTCCTACTCGCCCTCCACACGAA	5	<lmxll>	ll	129	lm	137		
>ref-37774	CCCCTCTCCACTCCTTCTCCTCTCGTC	4	<lmxll>	ll	128	lm	119		
>ref-37781	ACTCTTCAGACACCTCCTCCTCCTCCT	12	<nnxnp>	nn	218	np	17		
>ref-37785	TGAATGACTACTGATACTCCTTCTGAT	15	<lmxll>	ll	113	lm	149		
>ref-37785	TGAATGACTACTGATACTCCTTCTGAT	16	<lmxll>	ll	113	lm	150		
>ref-37835	TATGAATAAACATGTTCTCCACGCATA	14	<hkxhk>	hh	43	hk	148	kk	52
>ref-37851	CTCTCTCTCACCCTCTCTCCGAATTTC	22	<nnxnp>	nn	216	np	4		
>ref-37857	AGCTTCGATACCTTACCTCCTTGTACC	13	<nnxnp>	nn	133	np	138		
>ref-37860	CACTTTTAAACACATACTCCGCTAACA	24	<lmxll>	ll	124	lm	103		
>ref-37886	TCTTCCCTCACTCTCCCTCCATTTTAT	22	<hkxhk>	hh	0	hk	236	kk	22
>ref-37886	TCTTCCCTCACTCTCCCTCCATTTTAT	26	<hkxhk>	hh	22	hk	236	kk	0
>ref-37902	CTCCTACGGACCTCCACTCCTCTATCC	7	<nnxnp>	nn	130	np	133		

>ref-37906	CGCTTTCTCACTCCCACTCCCCTCCCC	27	<nnxnp>	nn	246	np	2		
>ref-37932	CCTACGCTCACATCCTCTCCCTCACTC	9	<hkxhk>	hh	63	hk	136	kk	66
>ref-37932	CCTACGCTCACATCCTCTCCCTCACTC	21	<lmxll>	ll	139	lm	133		
>ref-37935	TCCACCTCCACCATATCTCCTTCAATC	3	<nnxnp>	nn	269	np	1		
>ref-37936	TTAAGCTTGACACAGCCTCCCGCGGCT	2	<lmxll>	ll	3	lm	268		
>ref-37936	TTAAGCTTGACACAGCCTCCCGCGGCT	27	<lmxll>	ll	3	lm	268		
>ref-37937	CATTCAGCAACATTTTCTCCTCGGTAA	25	<lmxll>	ll	125	lm	137		
>ref-37940	CTTCAACACACCTTCTCTCCTCCCTCC	14	<hkxhk>	hh	52	hk	159	kk	47
>ref-37961	CAAACACACACACCCACTCCCTCCTCC	14	<nnxnp>	nn	18	np	240		
>ref-37966	CTCACCTTTACTATTTCTCCCTTTGTG	7	<hkxhk>	hh	60	hk	137	kk	69
>ref-37973	CCCACTCCAACACAATCTCCCTCTCTC	1	<lmxll>	ll	133	lm	136		
>ref-37995	CTCACGCCCACGAGCTCTCCCCCGCCC	16	<hkxhk>	hh	63	hk	127	kk	72
>ref-38021	TATGTCGTTACCCCCTCTCCCATTATT	13	<lmxll>	ll	113	lm	122		
>ref-38024	CAGGTAAATACGCCTCCTCCTCCTCCT	9	<hkxhk>	hh	2	hk	269	kk	0
>ref-38024	CAGGTAAATACGCCTCCTCCTCCTCCT	27	<lmxll>	ll	153	lm	102		
>ref-38079	CCGCCCTTGACCAATCCTCCTACCAAT	2	<hkxhk>	hh	1	hk	271	kk	0
>ref-38079	CCGCCCTTGACCAATCCTCCTACCAAT	8	<hkxhk>	hh	0	hk	273	kk	0
>ref-38079	CCGCCCTTGACCAATCCTCCTACCAAT	9	<hkxhk>	hh	0	hk	10	kk	235
>ref-38079	CCGCCCTTGACCAATCCTCCTACCAAT	13	<hkxhk>	hh	104	hk	149	kk	0
>ref-38079	CCGCCCTTGACCAATCCTCCTACCAAT	22	<lmxll>	ll	93	lm	171		
>ref-38090	CCCATCCCGACCTTCCCTCCCCATCCC	25	<hkxhk>	hh	60	hk	182	kk	0
>ref-38091	TCTCTTACTACTCTATCTCCCTCCCGT	23	<lmxll>	ll	134	lm	108		
>ref-38091	TCTCTTACTACTCTATCTCCCTCCCGT	24	<lmxll>	ll	107	lm	134		
>ref-38091	TCTCTTACTACTCTATCTCCCTCCCGT	26	<lmxll>	ll	256	lm	2		
>ref-38091	TCTCTTACTACTCTATCTCCCTCCCGT	27	<lmxll>	ll	261	lm	0		
>ref-38102	GGACCCCTCACTCAGCCTCCACCAATG	7	<lmxll>	ll	134	lm	133		
>ref-38102	GGACCCCTCACTCAGCCTCCACCAATG	15	<nnxnp>	nn	128	np	140		
>ref-38102	GGACCCCTCACTCAGCCTCCACCAATG	24	<nnxnp>	nn	128	np	140		
>ref-38135	CACTCTCCCACCTCATCTCCCTCCTCT	2	<hkxhk>	hh	1	hk	270	kk	0
>ref-38135	CACTCTCCCACCTCATCTCCCTCCTCT	4	<hkxhk>	hh	0	hk	271	kk	1
>ref-38156	GTGTGTGAGACTCTCCCTCCCTTTCTT	8	<nnxnp>	nn	127	np	139		
>ref-38172	AGTGTATGGACACCTCCTCCTCCTCCT	13	<hkxhk>	hh	71	hk	125	kk	57
>ref-38174	CAGCCAACGACTAATCCTCCAAATCAT	25	<nnxnp>	nn	118	np	145		
>ref-38188	CTTTCAATCACCGTCTCTCCTCCACAT	1	<nnxnp>	nn	142	np	123		
>ref-38188	CTTTCAATCACCGTCTCTCCTCCACAT	14	<nnxnp>	nn	142	np	124		
>ref-38201	CCATCCTCCACCTAATCTCCTTCCTCC	22	<nnxnp>	nn	113	np	129		
>ref-38244	CTCCCTCCAACACTATCTCCAACACTA	22	<lmxll>	ll	126	lm	95		
>ref-38256	ACAAAAACTACTCACACTCCTAGAACA	8	<hkxhk>	hh	2	hk	226	kk	34

>ref-38256	ACAAAAACTACTCACACTCCTAGAACA	15	<hkxhk>	hh	34	hk	228	kk	2
>ref-38275	TCTTAGTCAACACATGCTCCATCAACC	4	<lmxll>	ll	0	lm	269		
>ref-38275	TCTTAGTCAACACATGCTCCATCAACC	6	<lmxll>	ll	0	lm	269		
>ref-38286	GTTTTCTCTACCTCCTCTCCTTTCCTT	9	<lmxll>	ll	107	lm	117		
>ref-38307	GGGTGGGCCACGCTCTCTCCCGCCCAT	4	<nnxnp>	nn	143	np	124		
>ref-38325	AGTGTCGTCACTCCACCTCCTTTTCTT	21	<lmxll>	ll	262	lm	0		
>ref-38325	AGTGTCGTCACTCCACCTCCTTTTCTT	25	<lmxll>	ll	262	lm	0		
>ref-38336	TCCATCTCCACTACCACTCCCACCCTG	12	<nnxnp>	nn	133	np	120		
>ref-38336	TCCATCTCCACTACCACTCCCACCCTG	13	<nnxnp>	nn	133	np	120		
>ref-38349	CCCTCTTCCACTCGCTCTCCTCACCCT	15	<nnxnp>	nn	108	np	132		
>ref-38353	CCCCTTCCTACCGTTTCTCCTTCCCTG	27	<hkxhk>	hh	7	hk	259	kk	0
>ref-38355	CTAACATTAACCAACACTCCAGATCCG	14	<lmxll>	ll	107	lm	139		
>ref-38355	CTAACATTAACCAACACTCCAGATCCG	15	<lmxll>	ll	105	lm	139		
>ref-38382	CACATATCCACATACCCTCCTGCTCCA	2	<hkxhk>	hh	30	hk	240	kk	0
>ref-38382	CACATATCCACATACCCTCCTGCTCCA	7	<hkxhk>	hh	0	hk	273	kk	0
>ref-38382	CACATATCCACATACCCTCCTGCTCCA	23	<hkxhk>	hh	0	hk	269	kk	0
>ref-38382	CACATATCCACATACCCTCCTGCTCCA	24	<hkxhk>	hh	0	hk	272	kk	0
>ref-38391	TGTGTGTCAACAGATGCTCCAACAAGC	1	<nnxnp>	nn	126	np	141		
>ref-38397	CTGATTTCTACTCACCCTCCCTCTCTC	14	<hkxhk>	hh	59	hk	133	kk	57
>ref-38424	CGGATCATCACTAAGTCTCCCACAACT	27	<hkxhk>	hh	0	hk	272	kk	0
>ref-38433	CACGCACGCACACGTACTCCCATGTAC	3	<nnxnp>	nn	145	np	122		
>ref-38433	CACGCACGCACACGTACTCCCATGTAC	5	<nnxnp>	nn	146	np	122		
>ref-38448	ACGTTCACGACACGCTCTCCAGACCAC	4	<hkxhk>	hh	69	hk	141	kk	60
>ref-38483	CATCGTATAACCTCTTCTCCCTCCCCC	4	<hkxhk>	hh	60	hk	154	kk	44
>ref-38505	ACCAAATACACATTTCCTCCACTTGCA	7	<lmxll>	ll	113	lm	123		
>ref-38510	TTGCCCTTCACCCTCCCTCCTCCTCTT	2	<nnxnp>	nn	94	np	144		
>ref-38510	TTGCCCTTCACCCTCCCTCCTCCTCTT	7	<lmxll>	ll	268	lm	0		
>ref-38510	TTGCCCTTCACCCTCCCTCCTCCTCTT	13	<lmxll>	ll	269	lm	0		
>ref-38510	TTGCCCTTCACCCTCCCTCCTCCTCTT	15	<nnxnp>	nn	97	np	143		
>ref-38512	TGGAGTGGAACCGTCTCTCCACTTCAT	24	<lmxll>	ll	129	lm	137		
>ref-38512	TGGAGTGGAACCGTCTCTCCACTTCAT	27	<lmxll>	ll	128	lm	137		
>ref-38543	CTCCATCCTACCGTCTCTCCTCTTTCC	5	<hkxhk>	hh	2	hk	246	kk	4
>ref-38543	CTCCATCCTACCGTCTCTCCTCTTTCC	23	<hkxhk>	hh	4	hk	246	kk	2
>ref-38550	CCAGCGATGACGTCATCTCCTTCTTGG	5	<nnxnp>	nn	115	np	151		
>ref-38562	ACATCCATCACGGTCGCTCCATCCACC	8	<hkxhk>	hh	0	hk	273	kk	0
>ref-38573	TCCTATGTCACCCATTCTCCTCCTTCT	25	<lmxll>	ll	136	lm	128		
>ref-38578	ATGACTACTACACCAGCTCCACGGTGT	4	<hkxhk>	hh	0	hk	273	kk	0
>ref-38578	ATGACTACTACACCAGCTCCACGGTGT	13	<hkxhk>	hh	0	hk	273	kk	0

>ref-38578	ATGACTACTACACCAGCTCCACGGTGT	22	<hkxhk>	hh	23	hk	239	kk	0
>ref-38584	CACTTTCTCACTTTTTCTCCTCATTCC	1	<lmxll>	ll	249	lm	0		
>ref-38627	AAACAGAACACCAAGCCTCCACATGGT	23	<lmxll>	ll	146	lm	122		
>ref-38642	CTCTCCCTTACCCTTCCTCCGTCCATT	21	<nnxnp>	nn	19	np	246		
>ref-38642	CTCTCCCTTACCCTTCCTCCGTCCATT	27	<nnxnp>	nn	20	np	246		
>ref-38672	TTCAGCAGAACGCCGGCTCCCTTTTGG	26	<lmxll>	ll	117	lm	141		
>ref-38674	TCAACACACACAAAGACTCCTTGTCCG	13	<lmxll>	ll	125	lm	128		
>ref-38675	CCTTACGCCACCACACCTCCCCTACCT	1	<lmxll>	ll	31	lm	212		
>ref-38675	CCTTACGCCACCACACCTCCCCTACCT	2	<nnxnp>	nn	271	np	0		
>ref-38675	CCTTACGCCACCACACCTCCCCTACCT	7	<lmxll>	ll	31	lm	210		
>ref-38675	CCTTACGCCACCACACCTCCCCTACCT	8	<hkxhk>	hh	3	hk	181	kk	73
>ref-38716	TCTTCTCCCACGTTCCCTCCCCCCTCT	12	<hkxhk>	hh	13	hk	239	kk	0
>ref-38716	TCTTCTCCCACGTTCCCTCCCCCCTCT	14	<hkxhk>	hh	0	hk	238	kk	15
>ref-38724	ATGATGGAGACTGCATCTCCGAAACGT	6	<hkxhk>	hh	0	hk	264	kk	5
>ref-38724	ATGATGGAGACTGCATCTCCGAAACGT	9	<hkxhk>	hh	0	hk	147	kk	108
>ref-38724	ATGATGGAGACTGCATCTCCGAAACGT	14	<lmxll>	ll	102	lm	158		
>ref-38724	ATGATGGAGACTGCATCTCCGAAACGT	15	<lmxll>	ll	104	lm	158		
>ref-38724	ATGATGGAGACTGCATCTCCGAAACGT	22	<hkxhk>	hh	1	hk	244	kk	0
>ref-38740	GGATGCGACACCCCATCTCCTCGCATG	22	<lmxll>	ll	126	lm	137		
>ref-38741	GGCCTCCCCACTCCCCCTCCCTCTCCT	3	<nnxnp>	nn	226	np	0		
>ref-38783	AAATGTAACACGCGGTCTCCTCAGCAT	9	<lmxll>	ll	141	lm	129		
>ref-38793	ATGCACTTTACACGCCCTCCTGCTCTG	27	<nnxnp>	nn	263	np	0		
>ref-38807	TTCCTCTGTACTCCCTCTCCTCCCATT	21	<nnxnp>	nn	121	np	109		
>ref-38812	CCTCCTCTTACTTCTCCTCCGTCCCTC	1	<nnxnp>	nn	161	np	102		
>ref-38812	CCTCCTCTTACTTCTCCTCCGTCCCTC	21	<nnxnp>	nn	27	np	231		
>ref-38814	CTGGGGACTACTGATGCTCCTTCGCCG	6	<hkxhk>	hh	59	hk	154	kk	53
>ref-38837	TTTCCCCCAACGTCTCCTCCTTTACTT	14	<hkxhk>	hh	58	hk	151	kk	53
>ref-38837	TTTCCCCCAACGTCTCCTCCTTTACTT	22	<nnxnp>	nn	126	np	136		
>ref-38860	CCCACTTCCACTTCTCCTCCAACTCTC	26	<lmxll>	ll	264	lm	0		
>ref-38863	AACTCGACCACTAATACTCCCGTCCTT	6	<nnxnp>	nn	98	np	129		
>ref-38865	CAAGCCTCAACCCGTTCTCCTCCCCCA	8	<hkxhk>	hh	62	hk	129	kk	68
>ref-38889	AAACACACGACCCGACCTCCTGTGGCC	22	<lmxll>	ll	115	lm	139		
>ref-38901	ATTAAATGCACAGTCCCTCCTTTGCAA	6	<lmxll>	ll	129	lm	133		
>ref-38932	TCGCTCGTAACTATCGCTCCATCAGTG	15	<nnxnp>	nn	130	np	130		
>ref-38932	TCGCTCGTAACTATCGCTCCATCAGTG	24	<hkxhk>	hh	57	hk	143	kk	54
>ref-38944	TGTACCTGCACCTGTTCTCCGGTAGGT	7	<lmxll>	ll	121	lm	146		
>ref-38959	TTCCTTCCCACCTTCTCTCCTGTCGAC	14	<nnxnp>	nn	122	np	149		
>ref-38964	CATGCCATGACCTTAGCTCCTTCCGGG	14	<hkxhk>	hh	51	hk	131	kk	53

>ref-38972	CATCCTCCTACAACTTCTCCTTAACAT	26	<lmxll>	ll	106	lm	132		
>ref-38976	GTTGAGAATACACCTCCTCCTTCATCT	3	<hkxhk>	hh	51	hk	146	kk	60
>ref-38987	TGTACAGGGACAGAACCTCCAGTGCCC	7	<nnxnp>	nn	141	np	124		
>ref-39005	CTCATCCCCACCACCCCTCCTCCATCC	1	<lmxll>	ll	210	lm	16		
>ref-39025	TTTCTTGGAACAGATACTCCTCCACCG	6	<lmxll>	ll	129	lm	138		
>ref-39025	TTTCTTGGAACAGATACTCCTCCACCG	16	<nnxnp>	nn	3	np	267		
>ref-39046	CACACACAAACCGCTACTCCCTTCTCA	26	<nnxnp>	nn	114	np	152		
>ref-39049	CATTCCCACACCCTCCCTCCCCCCACA	2	<hkxhk>	hh	9	hk	202	kk	31
>ref-39049	CATTCCCACACCCTCCCTCCCCCCACA	21	<hkxhk>	hh	9	hk	202	kk	29
>ref-39065	GCTATCACCACCGTCCCTCCTCGCTCT	23	<lmxll>	ll	124	lm	147		
>ref-39071	ACCTCCTTCACCCAACCTCCCTCCCTC	1	<nnxnp>	nn	147	np	102		
>ref-39094	TCATATTCGACGCACCCTCCCCTTCCC	27	<lmxll>	ll	126	lm	133		
>ref-39097	CTTTCCTATACACTCCCTCCTCCTCTT	5	<nnxnp>	nn	124	np	133		
>ref-39118	ATCTATCTAACTATCTCTCCCATCATG	6	<lmxll>	ll	120	lm	130		
>ref-39122	TGCAACAAGACAAGTGCTCCTAAATAC	5	<nnxnp>	nn	109	np	130		
>ref-39181	CCCTCCCCTACTCCCCCTCCTCCATCC	12	<lmxll>	ll	88	lm	137		
>ref-39185	ACAGGAACCACTGGCTCTCCATATCTG	12	<nnxnp>	nn	138	np	124		
>ref-39185	ACAGGAACCACTGGCTCTCCATATCTG	13	<nnxnp>	nn	139	np	124		
>ref-39187	TATTCCTTTACCTCCTCTCCTTTCAAT	3	<hkxhk>	hh	51	hk	148	kk	59
>ref-39204	CTCTTCATTACGATCCCTCCCCCGTCC	12	<nnxnp>	nn	129	np	105		
>ref-39206	CTCTCTCACACCCTTTCTCCCTTATAC	4	<hkxhk>	hh	0	hk	251	kk	7
>ref-39206	CTCTCTCACACCCTTTCTCCCTTATAC	16	<hkxhk>	hh	0	hk	248	kk	8
>ref-39210	AGCATCTACACGCTTTCTCCTGTTTTT	14	<lmxll>	ll	105	lm	128		
>ref-39237	TGCTTCTCCACCCTTTCTCCTACCTTT	1	<lmxll>	ll	60	lm	201		
>ref-39246	TTCACTGTCACATAATCTCCTGCTCTA	7	<lmxll>	ll	126	lm	110		
>ref-39257	CCGAGTACAACTCTAACTCCTCGGACA	7	<lmxll>	ll	7	lm	263		
>ref-39264	GATCGCCAGACAGCATCTCCACATGGA	5	<lmxll>	ll	155	lm	108		
>ref-39270	CAATGATTTACCTATTCTCCCAACCGT	22	<hkxhk>	hh	101	hk	145	kk	0
>ref-39270	CAATGATTTACCTATTCTCCCAACCGT	25	<lmxll>	ll	224	lm	12		
>ref-39270	CAATGATTTACCTATTCTCCCAACCGT	26	<hkxhk>	hh	0	hk	145	kk	100
>ref-39270	CAATGATTTACCTATTCTCCCAACCGT	27	<hkxhk>	hh	1	hk	267	kk	0
>ref-39292	CTCACTCCCACGTCTACTCCACCTATC	12	<nnxnp>	nn	266	np	0		
>ref-39305	TCCCTGTGAACGATCACTCCCTTGCAG	15	<hkxhk>	hh	92	hk	162	kk	0
>ref-39305	TCCCTGTGAACGATCACTCCCTTGCAG	24	<hkxhk>	hh	0	hk	163	kk	91
>ref-39323	AGGGCTGACACTAGGTCTCCAGAGGAA	2	<nnxnp>	nn	143	np	129		
>ref-39328	CCTCCGCCCACTTTTTCTCCTTCTTTT	16	<hkxhk>	hh	0	hk	267	kk	2
>ref-39358	AGTAAGGTCACGCAGCCTCCTTTAAAA	1	<lmxll>	ll	159	lm	76		
>ref-39363	ACACACACAACTCCCTCTCCACGTACA	1	<lmxll>	ll	139	lm	80		

>ref-39365	CCCTTCCTTACTCTGTCTCCTTTACTC	27	<nnxnp>	nn	139	np	129		
>ref-39394	CCTCTTCCCACTTTCCCTCCGTTTGCT	9	<nnxnp>	nn	138	np	114		
>ref-39394	CCTCTTCCCACTTTCCCTCCGTTTGCT	25	<nnxnp>	nn	133	np	115		
>ref-39400	GGGCTGGCCACGGGTCCTCCTCGCCTT	12	<nnxnp>	nn	147	np	125		
>ref-39406	ATTACCTGTACAACTACTCCGCGGGAA	22	<lmxll>	ll	120	lm	124		
>ref-39428	GCAACATAGACAGCCCCTCCCGTCAAC	6	<lmxll>	ll	135	lm	123		
>ref-39432	TTCTTCCCCACTTGTCCTCCTTCCTCC	26	<lmxll>	ll	113	lm	144		
>ref-39433	CCCTCCATCACAGTCTCTCCTCTTGTC	21	<hkxhk>	hh	58	hk	147	kk	59
>ref-39478	TCACTCTATACGTTCTCTCCCTCTTTT	21	<nnxnp>	nn	104	np	138		
>ref-39481	TTGTTCTTTACTGTCTCTCCGTTTGTT	27	<nnxnp>	nn	120	np	141		
>ref-39496	GTGATGTGTACGTACGCTCCCAGAGAT	23	<nnxnp>	nn	147	np	117		
>ref-39506	GCACTGAACACCGACCCTCCTGGTCCC	22	<hkxhk>	hh	0	hk	271	kk	1
>ref-39508	AGGGGAATAACGCCCCCTCCCTCCCCG	7	<lmxll>	ll	118	lm	142		
>ref-39510	TTCACCCTCACCCTTCCTCCATTCCTC	8	<lmxll>	ll	21	lm	233		
>ref-39510	TTCACCCTCACCCTTCCTCCATTCCTC	27	<lmxll>	ll	19	lm	232		
>ref-39554	GTCAATCCTACTCGAGCTCCAACCACG	2	<hkxhk>	hh	55	hk	139	kk	53
>ref-39556	CTTCATCCCACCATTTCTCCCTCCTTC	21	<lmxll>	ll	128	lm	136		
>ref-39556	CTTCATCCCACCATTTCTCCCTCCTTC	24	<lmxll>	ll	128	lm	136		
>ref-39578	TCTGTGTCCACCCCACCTCCTCCTCCC	22	<lmxll>	ll	128	lm	124		
>ref-39585	ACCAGCTGGACCAACCCTCCCTTGCCC	13	<nnxnp>	nn	143	np	126		
>ref-39596	GCCTCTTTTACTTTCTCTCCCGCGTTG	16	<nnxnp>	nn	137	np	130		
>ref-39596	GCCTCTTTTACTTTCTCTCCCGCGTTG	24	<nnxnp>	nn	137	np	130		
>ref-39599	TCTTTTCACACTCATCCTCCCCCCTCT	15	<nnxnp>	nn	126	np	123		
>ref-39608	GCCAGCGTGACCTCTTCTCCCTCTCTT	13	<nnxnp>	nn	131	np	133		
>ref-39613	CTTGCGTGGACGTTACCTCCTAATTTC	6	<nnxnp>	nn	134	np	120		
>ref-39617	ACCTTCCTTACCGTCTCTCCCTGCCAC	27	<nnxnp>	nn	112	np	124		
>ref-39628	CCTTCTCTCACAATGTCTCCCTCTCCC	1	<hkxhk>	hh	6	hk	265	kk	0
>ref-39628	CCTTCTCTCACAATGTCTCCCTCTCCC	12	<hkxhk>	hh	6	hk	265	kk	0
>ref-39639	TCTTGAGAAACACTTTCTCCTTCGGGA	23	<lmxll>	ll	126	lm	110		
>ref-39649	TTATCATCAACGGTTTCTCCAACAAGT	6	<hkxhk>	hh	0	hk	273	kk	0
>ref-39649	TTATCATCAACGGTTTCTCCAACAAGT	26	<hkxhk>	hh	0	hk	273	kk	0
>ref-39649	TTATCATCAACGGTTTCTCCAACAAGT	27	<hkxhk>	hh	0	hk	264	kk	4
>ref-39652	AATCCAGCGACAGCCTCTCCAGGGCAT	12	<nnxnp>	nn	123	np	130		
>ref-39652	AATCCAGCGACAGCCTCTCCAGGGCAT	21	<nnxnp>	nn	124	np	130		
>ref-39694	CCTTTATCCACGCTTCCTCCTTAGTAT	14	<hkxhk>	hh	60	hk	139	kk	49
>ref-39704	ACACACATGACCACGCCTCCTCCACCT	2	<lmxll>	ll	267	lm	4		
>ref-39706	CAGACACAGACACGCACTCCGTCCTCA	3	<nnxnp>	nn	270	np	0		
>ref-39706	CAGACACAGACACGCACTCCGTCCTCA	9	<nnxnp>	nn	270	np	0		

>ref-39707	ACGCAGAAAACACCGTCTCCCATACAG	5	<nnxnp>	nn	125	np	128		
>ref-39715	ACAACACACACTTCTACTCCGCCTACA	12	<nnxnp>	nn	144	np	123		
>ref-39731	AGGTGGGCTACTCCCTCTCCGCGCCAG	8	<nnxnp>	nn	120	np	148		
>ref-39761	TCTTTCTCAACCTACCCTCCTGTTCAT	12	<nnxnp>	nn	116	np	142		
>ref-39784	TCTCCCGCGACCAATTCTCCTTCCCTC	25	<lmxll>	ll	151	lm	118		
>ref-39843	ATTCCCCGAACCTTCTCTCCAATCGCC	6	<hkxhk>	hh	53	hk	135	kk	66
>ref-39849	CTTTCTCGTACCCCTACTCCCTCTCTC	7	<hkxhk>	hh	67	hk	138	kk	45
>ref-39853	TGAAATCTAACCCTTACTCCATGGACT	25	<hkxhk>	hh	44	hk	221	kk	0
>ref-39864	GAGAGAGAAACGTACACTCCAAACGCC	7	<nnxnp>	nn	124	np	140		
>ref-39870	GCATACACAACTAACTCTCCTGCTTCT	2	<nnxnp>	nn	139	np	125		
>ref-39876	GATCTGGCAACTTTAGCTCCATTCCTA	27	<lmxll>	ll	126	lm	127		
>ref-39910	CCACCGCAGACACTCGCTCCCACGCCC	4	<hkxhk>	hh	0	hk	245	kk	12
>ref-39910	CCACCGCAGACACTCGCTCCCACGCCC	26	<nnxnp>	nn	72	np	176		
>ref-39910	CCACCGCAGACACTCGCTCCCACGCCC	27	<nnxnp>	nn	173	np	94		
>ref-39939	GAAGTTTCTACTCTCTCTCCTTCTCTT	2	<hkxhk>	hh	1	hk	264	kk	0
>ref-39939	GAAGTTTCTACTCTCTCTCCTTCTCTT	4	<hkxhk>	hh	1	hk	264	kk	0
>ref-39939	GAAGTTTCTACTCTCTCTCCTTCTCTT	8	<nnxnp>	nn	147	np	107		
>ref-39952	CGACGCCCTACCCGCCCTCCCTCTCCC	6	<nnxnp>	nn	222	np	0		
>ref-39956	GAACTCTTCACGAGTTCTCCTTCAGAC	5	<lmxll>	ll	1	lm	264		
>ref-39956	GAACTCTTCACGAGTTCTCCTTCAGAC	21	<lmxll>	ll	1	lm	264		
>ref-39970	CGCCCTCTCACGCCCTCTCCCAGTTTT	4	<lmxll>	ll	242	lm	0		
>ref-39981	CCCCAACACACCTTTCCTCCCTCCATC	7	<nnxnp>	nn	138	np	132		
>ref-39995	CATGGCTAGACTCTGGCTCCGCCCCCA	26	<nnxnp>	nn	264	np	0		
>ref-39995	CATGGCTAGACTCTGGCTCCGCCCCCA	27	<nnxnp>	nn	263	np	0		
>ref-39997	CTTCCACATACTCCACCTCCACCTCTT	8	<hkxhk>	hh	4	hk	260	kk	0
>ref-40045	TCCGTCGCCACGCGCGCTCCGGCCTCC	21	<lmxll>	ll	140	lm	131		
>ref-40050	TTTCTCAACACTTTCTCTCCCTCTCTC	3	<nnxnp>	nn	251	np	0		
>ref-40050	TTTCTCAACACTTTCTCTCCCTCTCTC	13	<nnxnp>	nn	232	np	7		
>ref-40059	TCCAACCCTACCCCAACTCCTGGCTTT	27	<nnxnp>	nn	129	np	130		
>ref-40080	GGTCGCTCCACAGAGGCTCCCAACACA	25	<hkxhk>	hh	0	hk	273	kk	0
>ref-40116	TCTAGCGTAACATTTTCTCCCTATGTA	22	<hkxhk>	hh	61	hk	125	kk	49
>ref-40121	TCTATCCCTACCATCCCTCCCCTACCC	5	<lmxll>	ll	119	lm	140		
>ref-40149	GACATCCTTACTCATCCTCCTTATTCT	2	<hkxhk>	hh	48	hk	199	kk	0
>ref-40149	GACATCCTTACTCATCCTCCTTATTCT	9	<hkxhk>	hh	0	hk	157	kk	99
>ref-40149	GACATCCTTACTCATCCTCCTTATTCT	21	<hkxhk>	hh	0	hk	224	kk	34
>ref-40149	GACATCCTTACTCATCCTCCTTATTCT	26	<lmxll>	ll	131	lm	109		
>ref-40180	AGCACCCTCACAAGTGCTCCTCCTCCA	27	<nnxnp>	nn	114	np	143		
>ref-40195	TGTACTTCCACTTATCCTCCTTTCTTC	7	<nnxnp>	nn	128	np	142		

>ref-40195	TGTACTTCCACTTATCCTCCTTTCTTC	9	<nnxnp>	nn	128	np	142		
>ref-40204	ATTATTCGTACTTATGCTCCCATTCTA	9	<lmxll>	ll	109	lm	125		
>ref-40206	CACACAATTACTTCATCTCCTCAGCCC	25	<hkxhk>	hh	65	hk	150	kk	42
>ref-40223	CATAGCCTTACTGATACTCCTATTACT	25	<lmxll>	ll	15	lm	223		
>ref-40224	GTTGAACGTACAAGGCCTCCGTCTCCA	9	<nnxnp>	nn	272	np	0		
>ref-40229	TCCTCCCCTACTAATTCTCCCCAACTT	12	<nnxnp>	nn	224	np	32		
>ref-40236	CGAATTATCACGGAGCCTCCCGCTTCT	24	<nnxnp>	nn	119	np	136		
>ref-40254	TCTCTCTCTACCCATGCTCCCTCATCC	6	<nnxnp>	nn	118	np	151		
>ref-40273	TGTAAGGCCACACTCGCTCCTCTGCGA	25	<lmxll>	ll	135	lm	132		
>ref-40288	CCCATCCTTACAATTACTCCCTACCTT	2	<nnxnp>	nn	95	np	149		
>ref-40349	CACGGAGAGACTCTCCCTCCTCCTCCT	26	<nnxnp>	nn	137	np	126		
>ref-40349	CACGGAGAGACTCTCCCTCCTCCTCCT	27	<nnxnp>	nn	134	np	128		
>ref-40351	AGGGAATGAACTCTTGCTCCTGTGGCT	5	<lmxll>	ll	125	lm	130		
>ref-40355	CAGCGGCATACGCCCCCTCCAGCTAGT	9	<hkxhk>	hh	47	hk	143	kk	65
>ref-40355	CAGCGGCATACGCCCCCTCCAGCTAGT	25	<nnxnp>	nn	125	np	141		
>ref-40357	ACTTTATCAACCCTCTCTCCTTCTCTC	1	<hkxhk>	hh	7	hk	264	kk	0
>ref-40357	ACTTTATCAACCCTCTCTCCTTCTCTC	26	<hkxhk>	hh	0	hk	264	kk	7
>ref-40358	AACCTACTCACCATCTCTCCTACTCCC	6	<lmxll>	ll	111	lm	132		
>ref-40365	TAATGTCACACGCGTTCTCCCACCTGA	13	<nnxnp>	nn	145	np	119		
>ref-40365	TAATGTCACACGCGTTCTCCCACCTGA	23	<nnxnp>	nn	145	np	119		
>ref-40409	TGCTTGTTAACTGTTGCTCCGGCCGTG	24	<hkxhk>	hh	48	hk	145	kk	69
>ref-40410	CACGAGGTTACTCAAGCTCCTTAACCT	4	<nnxnp>	nn	140	np	126		
>ref-40431	TCATCTTCTACCTCTCCTCCTTCCCTC	1	<lmxll>	ll	130	lm	134		
>ref-40435	AAATCACAAACTTATTCTCCTTCCTTC	9	<lmxll>	ll	119	lm	141		
>ref-40436	TTCTCCCTCACAATTCCTCCTTCCATC	16	<hkxhk>	hh	14	hk	177	kk	48
>ref-40436	TTCTCCCTCACAATTCCTCCTTCCATC	24	<lmxll>	ll	136	lm	106		
>ref-40436	TTCTCCCTCACAATTCCTCCTTCCATC	25	<lmxll>	ll	137	lm	107		
>ref-40441	TCACCCTCTACTTTCACTCCCCCTTTA	16	<lmxll>	ll	131	lm	117		
>ref-40456	GCAGCTGTGACCTTTACTCCTGCCGAT	25	<nnxnp>	nn	121	np	142		
>ref-40462	GAACCAGAAACTGAAGCTCCAACAGAA	3	<hkxhk>	hh	0	hk	270	kk	0
>ref-40462	GAACCAGAAACTGAAGCTCCAACAGAA	9	<nnxnp>	nn	1	np	268		
>ref-40473	CAGCTGATGACACAAGCTCCTCGATGT	23	<hkxhk>	hh	0	hk	273	kk	0
>ref-40473	CAGCTGATGACACAAGCTCCTCGATGT	27	<nnxnp>	nn	241	np	8		
>ref-40493	ACCTCTTCCACCCCCCCTCCATACCAC	21	<lmxll>	ll	29	lm	214		
>ref-40493	ACCTCTTCCACCCCCCCTCCATACCAC	26	<lmxll>	ll	27	lm	213		
>ref-40527	CTCTTTTCCACGCTCCCTCCCTCTCGC	1	<lmxll>	ll	147	lm	110		
>ref-40537	ACCACTCACACACTCCCTCCCTCTCGT	25	<lmxll>	ll	150	lm	119		
>ref-40539	ATTCCAATCACCCAATCTCCCAATTCC	3	<lmxll>	ll	106	lm	134		

>ref-40548	TTTGAGTTTACTTTCTCTCCTTCCTTG	4	<nnxnp>	nn	125	np	103		
>ref-40557	TAACCCCCAACTACCCCTCCCTCTACC	4	<nnxnp>	nn	115	np	130		
>ref-40558	TATTAAGACACTCTCTCTCCACTTCTC	25	<lmxll>	ll	20	lm	227		
>ref-40593	CAGCAGCACACAAGGACTCCCCAGTCC	12	<nnxnp>	nn	158	np	110		
>ref-40616	TGGTCCTATACAACCACTCCGGCTTAC	2	<nnxnp>	nn	250	np	0		
>ref-40617	ACAAATACAACAACAACTCCTTTAAAC	5	<lmxll>	ll	0	lm	268		
>ref-40617	ACAAATACAACAACAACTCCTTTAAAC	6	<lmxll>	ll	0	lm	270		
>ref-40617	ACAAATACAACAACAACTCCTTTAAAC	16	<hkxhk>	hh	61	hk	182	kk	0
>ref-40663	CCTTCCTTCACTCCCTCTCCCGTTTCC	13	<nnxnp>	nn	99	np	129		
>ref-40665	TCCCCATTCACCCTGCCTCCTCCCCCT	8	<lmxll>	ll	125	lm	120		
>ref-40679	CCATTTAGAACCGTTGCTCCGGTGATA	12	<nnxnp>	nn	138	np	116		
>ref-40692	CTTCCCTCTACATCCTCTCCACCTTAC	21	<hkxhk>	hh	0	hk	239	kk	15
>ref-40692	CTTCCCTCTACATCCTCTCCACCTTAC	23	<hkxhk>	hh	32	hk	192	kk	1
>ref-40703	GCCCTCATTACGTTCCCTCCTCGTCAC	1	<hkxhk>	hh	105	hk	161	kk	0
>ref-40703	GCCCTCATTACGTTCCCTCCTCGTCAC	23	<hkxhk>	hh	0	hk	272	kk	0
>ref-40703	GCCCTCATTACGTTCCCTCCTCGTCAC	27	<nnxnp>	nn	219	np	45		
>ref-40716	TGTTAGTAAACAAATCCTCCCGTTAGA	27	<lmxll>	ll	110	lm	121		
>ref-40717	AGGGCCAGGACTTGATCTCCGTTGTTG	2	<hkxhk>	hh	55	hk	154	kk	52
>ref-40725	AACAAGCAAACGCTCCCTCCTACACGC	12	<lmxll>	ll	138	lm	129		
>ref-40725	AACAAGCAAACGCTCCCTCCTACACGC	27	<lmxll>	ll	138	lm	129		
>ref-40773	CACGTATCTACCTACCCTCCCTCTCTC	7	<nnxnp>	nn	9	np	251		
>ref-40773	CACGTATCTACCTACCCTCCCTCTCTC	9	<nnxnp>	nn	7	np	252		
>ref-40785	TCCCCCTCTACATCCTCTCCTTCTCCC	2	<lmxll>	ll	267	lm	0		
>ref-40785	TCCCCCTCTACATCCTCTCCTTCTCCC	5	<lmxll>	ll	26	lm	227		
>ref-40785	TCCCCCTCTACATCCTCTCCTTCTCCC	6	<lmxll>	ll	25	lm	228		
>ref-40785	TCCCCCTCTACATCCTCTCCTTCTCCC	21	<lmxll>	ll	268	lm	0		
>ref-40803	TCTCGTGTGACACTTGCTCCTTCAAAT	15	<nnxnp>	nn	122	np	116		
>ref-40812	GCTCACGTCACGTCATCTCCTTTTCTG	1	<hkxhk>	hh	48	hk	143	kk	67
>ref-40815	ATTTTCGCCACCCTTTCTCCTCTTCCC	27	<nnxnp>	nn	241	np	2		
>ref-40821	CTGCATGGCACGCCTCCTCCCGCGTTT	24	<hkxhk>	hh	63	hk	153	kk	52
>ref-40826	ACCCTATTTACCACTTCTCCTTGGATC	3	<hkxhk>	hh	0	hk	165	kk	75
>ref-40826	ACCCTATTTACCACTTCTCCTTGGATC	6	<hkxhk>	hh	4	hk	255	kk	0
>ref-40826	ACCCTATTTACCACTTCTCCTTGGATC	22	<lmxll>	ll	64	lm	172		
>ref-40833	CCTACCACTACGCATCCTCCCCTCCCC	21	<lmxll>	ll	102	lm	154		
>ref-40839	AAAGTAATCACAAGGTCTCCACACCTG	22	<nnxnp>	nn	118	np	128		
>ref-40841	TATTCTGCAACCAACTCTCCTCTCAAA	16	<nnxnp>	nn	123	np	127		
>ref-40842	TATTATCATACTTTCGCTCCGCTCGCT	7	<hkxhk>	hh	12	hk	240	kk	0
>ref-40855	TTTCTTCTCACTCCTTCTCCTATCTCC	8	<nnxnp>	nn	235	np	0		

>ref-40866	TGTAACAAAACGCTTGCTCCTGTCGCC	13	<lmxll>	ll	131	lm	131		
>ref-40866	TGTAACAAAACGCTTGCTCCTGTCGCC	24	<nnxnp>	nn	144	np	123		
>ref-40887	GGTTGACGAACTACTCCTCCTTCATGT	14	<lmxll>	ll	125	lm	131		
>ref-40910	ACAATCATGACCCCCACTCCTCCACCA	14	<nnxnp>	nn	120	np	133		
>ref-40915	GTCCCTCTCACCCCTTCTCCACTCCCT	2	<nnxnp>	nn	268	np	0		
>ref-40915	GTCCCTCTCACCCCTTCTCCACTCCCT	23	<lmxll>	ll	137	lm	129		
>ref-40917	CTATCCCCAACCTCCCCTCCTCACCCT	7	<hkxhk>	hh	51	hk	166	kk	18
>ref-40927	CTTCCCCACACTACCCCTCCTCTTTCC	9	<hkxhk>	hh	5	hk	227	kk	9
>ref-40927	CTTCCCCACACTACCCCTCCTCTTTCC	13	<hkxhk>	hh	5	hk	227	kk	10
>ref-40949	ACCTTGCTAACATGCTCTCCATGCTCT	7	<lmxll>	ll	141	lm	131		
>ref-40949	ACCTTGCTAACATGCTCTCCATGCTCT	12	<nnxnp>	nn	140	np	131		
>ref-40962	CTTCCCTACACCATGTCTCCTTCTCCT	25	<nnxnp>	nn	132	np	138		
>ref-40963	CTCTCTCTTACCCTTGCTCCCCAAGCT	14	<nnxnp>	nn	265	np	0		
>ref-40981	AGGGCAGCCACCTCAACTCCCAGATAG	13	<hkxhk>	hh	0	hk	261	kk	2
>ref-40981	AGGGCAGCCACCTCAACTCCCAGATAG	23	<hkxhk>	hh	0	hk	261	kk	2
>ref-40985	AACTTCAAGACCCCTACTCCCAGACCC	25	<nnxnp>	nn	252	np	0		
>ref-40987	CTCCCTCACACTCCCCCTCCTCTTCTT	1	<lmxll>	ll	111	lm	146		
>ref-41011	GATCTTCCTACTCTTCCTCCTCTTTTT	15	<nnxnp>	nn	112	np	139		
>ref-41017	AAAACATCCACGTTTGCTCCATTTCTT	6	<nnxnp>	nn	114	np	116		
>ref-41038	CACGCCCACACCGACTCTCCCTCTCTC	13	<hkxhk>	hh	62	hk	210	kk	0
>ref-41038	CACGCCCACACCGACTCTCCCTCTCTC	26	<nnxnp>	nn	136	np	132		
>ref-41041	CGTTCTTAGACGTCTGCTCCTTTGTCC	2	<lmxll>	ll	146	lm	123		
>ref-41047	GCCTGGCCCACACTAACTCCCATGTCC	26	<lmxll>	ll	123	lm	126		
>ref-41054	TCCCTTCGTACTCTCTCTCCCCTCCCC	27	<nnxnp>	nn	38	np	205		
>ref-41098	TAGAACTCTACGCGCACTCCTCCAGCA	22	<hkxhk>	hh	52	hk	137	kk	55
>ref-41098	TAGAACTCTACGCGCACTCCTCCAGCA	23	<hkxhk>	hh	55	hk	137	kk	52
>ref-41099	CCTCCCCCTACCCCCTCTCCTCCCTTG	2	<nnxnp>	nn	94	np	133		
>ref-41099	CCTCCCCCTACCCCCTCTCCTCCCTTG	5	<nnxnp>	nn	9	np	253		
>ref-41099	CCTCCCCCTACCCCCTCTCCTCCCTTG	16	<nnxnp>	nn	91	np	135		
>ref-41148	TCCATCACAACGCTTTCTCCAACACTA	5	<lmxll>	ll	122	lm	143		
>ref-41148	TCCATCACAACGCTTTCTCCAACACTA	9	<nnxnp>	nn	118	np	146		
>ref-41205	CACATACTTACATTCTCTCCCCGCCAC	5	<nnxnp>	nn	129	np	143		
>ref-41216	CTACGTACAACTATGCCTCCTCCAGAT	12	<nnxnp>	nn	247	np	0		
>ref-41219	TCCCTTGCCACCCCCCCTCCCTCCATC	25	<nnxnp>	nn	49	np	171		
>ref-41224	CGCCGTACGACACTCACTCCAGCATTC	27	<nnxnp>	nn	141	np	125		
>ref-41227	AGCGAAACCACCGTTACTCCCACAACA	25	<nnxnp>	nn	261	np	0		
>ref-41298	TTCTCCTCTACACTTTCTCCTATCTGT	6	<nnxnp>	nn	135	np	123		
>ref-41303	CGCTCTCACACCTTATCTCCGCTGTTA	4	<hkxhk>	hh	60	hk	144	kk	61

>ref-41303	CGCTCTCACACCTTATCTCCGCTGTTA	23	<nnxnp>	nn	134	np	132		
>ref-41318	TATGCCAGGACAGGAGCTCCGTAAGCC	15	<lmxll>	ll	128	lm	127		
>ref-41322	GAAGAAGCCACACATCCTCCCTTCCTT	14	<nnxnp>	nn	134	np	126		
>ref-41324	ATCTTATCTACTTTCCCTCCCTCTCCC	5	<hkxhk>	hh	0	hk	182	kk	66
>ref-41324	ATCTTATCTACTTTCCCTCCCTCTCCC	13	<hkxhk>	hh	0	hk	182	kk	66
>ref-41327	ACAGGAACGACTGAGTCTCCAAACTCT	12	<lmxll>	ll	137	lm	127		
>ref-41329	CTCCCCCCTACTCCTCCTCCTCCCCAA	9	<hkxhk>	hh	0	hk	154	kk	79
>ref-41329	CTCCCCCCTACTCCTCCTCCTCCCCAA	12	<lmxll>	ll	235	lm	28		
>ref-41329	CTCCCCCCTACTCCTCCTCCTCCCCAA	26	<lmxll>	ll	5	lm	263		
>ref-41329	CTCCCCCCTACTCCTCCTCCTCCCCAA	27	<hkxhk>	hh	0	hk	227	kk	1
>ref-41331	GCAAACCTTACGGCTTCTCCAGCGCCA	4	<nnxnp>	nn	110	np	127		
>ref-41349	ACAATATCGACATTTTCTCCATCCCCT	5	<nnxnp>	nn	130	np	111		
>ref-41351	AAGATGGTAACGTAGCCTCCCATGTTG	4	<nnxnp>	nn	113	np	111		
>ref-41352	ATCTCGTTCACACGCCCTCCAAGACTT	14	<hkxhk>	hh	67	hk	133	kk	51
>ref-41375	GTGAACCCGACCGTTGCTCCTCTGCTG	15	<nnxnp>	nn	123	np	140		
>ref-41409	ACTACTCCGACTACAGCTCCTTATGCA	27	<hkxhk>	hh	64	hk	124	kk	50
>ref-41425	TTAATGCCTACTGCAGCTCCCTCTCAC	1	<nnxnp>	nn	123	np	137		
>ref-41425	TTAATGCCTACTGCAGCTCCCTCTCAC	6	<nnxnp>	nn	123	np	137		
>ref-41426	ATGCCTTTTACCTCTTCTCCCTGGCAT	5	<nnxnp>	nn	264	np	0		
>ref-41428	TTTGTCTTTACTCTATCTCCCTCTCTA	13	<lmxll>	ll	103	lm	140		
>ref-41435	CAACCCCCAACACCCCCTCCACACACA	9	<nnxnp>	nn	131	np	125		
>ref-41435	CAACCCCCAACACCCCCTCCACACACA	13	<nnxnp>	nn	132	np	124		
>ref-41450	CTCTTTTCTACCTATTCTCCTCCTATT	2	<nnxnp>	nn	99	np	120		
>ref-41452	GCTTTACACACTCGCCCTCCACGCCGC	21	<nnxnp>	nn	123	np	137		
>ref-41454	CTTCCTCAGACGCTGCCTCCTCCTTGT	14	<lmxll>	ll	132	lm	134		
>ref-41462	AACACCTTAACCTCCCCTCCCTCGTTT	14	<nnxnp>	nn	8	np	259		
>ref-41462	AACACCTTAACCTCCCCTCCCTCGTTT	24	<nnxnp>	nn	7	np	259		
>ref-41501	TAGCGATTAACGACGGCTCCTTATTTC	3	<nnxnp>	nn	127	np	140		
>ref-41501	TAGCGATTAACGACGGCTCCTTATTTC	6	<lmxll>	ll	128	lm	140		
>ref-41519	TTTCTTATTACTATTTCTCCTTGTCTC	6	<nnxnp>	nn	110	np	119		
>ref-41524	CATATGCACACTCTCTCTCCTTCTCCT	8	<nnxnp>	nn	106	np	159		
>ref-41524	CATATGCACACTCTCTCTCCTTCTCCT	15	<nnxnp>	nn	107	np	158		
>ref-41558	AACTGTGCCACATGAACTCCATAACTT	3	<lmxll>	ll	129	lm	119		
>ref-41560	ACCACAACAACAGCTACTCCAACTACA	12	<nnxnp>	nn	23	np	242		
>ref-41560	ACCACAACAACAGCTACTCCAACTACA	15	<nnxnp>	nn	140	np	133		
>ref-41560	ACCACAACAACAGCTACTCCAACTACA	16	<hkxhk>	hh	1	hk	272	kk	0
>ref-41582	CTTCATTTTACACTACCTCCAAGCTAT	5	<hkxhk>	hh	80	hk	164	kk	0
>ref-41582	CTTCATTTTACACTACCTCCAAGCTAT	7	<hkxhk>	hh	0	hk	163	kk	80

>ref-41582	CTTCATTTTACACTACCTCCAAGCTAT	15	<hkxhk>	hh	12	hk	256	kk	0
>ref-41623	TTTTCCATCACCCCTTCTCCCCCTTTC	1	<nnxnp>	nn	87	np	162		
>ref-41623	TTTTCCATCACCCCTTCTCCCCCTTTC	7	<hkxhk>	hh	32	hk	206	kk	5
>ref-41652	GAAGCAGAAACAGAAGCTCCGTTTAAT	24	<hkxhk>	hh	49	hk	167	kk	54
>ref-41672	CACTGGACCACCTGTTCTCCTGGCATA	23	<nnxnp>	nn	140	np	131		
>ref-41700	CCCTCTCCAACCCCATCTCCCTGTTAC	26	<nnxnp>	nn	114	np	115		
>ref-41702	TATATATCTACTAAATCTCCATCATAA	25	<nnxnp>	nn	127	np	92		
>ref-41722	TATCATCCTACATCCTCTCCCTTCTCC	2	<hkxhk>	hh	2	hk	269	kk	0
>ref-41722	TATCATCCTACATCCTCTCCCTTCTCC	6	<hkxhk>	hh	0	hk	269	kk	2
>ref-41736	GTATAATACACTCTCCCTCCCTACCTC	1	<lmxll>	ll	3	lm	259		
>ref-41736	GTATAATACACTCTCCCTCCCTACCTC	6	<lmxll>	ll	3	lm	259		
>ref-41763	CCCTTTCTAACCTTTTCTCCCTCCCTC	5	<lmxll>	ll	114	lm	116		
>ref-41763	CCCTTTCTAACCTTTTCTCCCTCCCTC	12	<nnxnp>	nn	59	np	166		
>ref-41772	ATACCCTCCACGTTGCCTCCCTCCCTT	3	<lmxll>	ll	139	lm	124		
>ref-41791	TCAGCCATGACCCAAGCTCCTGCATCA	13	<lmxll>	ll	130	lm	133		
>ref-41805	TTCTCATCTACTCTTTCTCCTCTTCTC	6	<nnxnp>	nn	11	np	256		
>ref-41805	TTCTCATCTACTCTTTCTCCTCTTCTC	24	<nnxnp>	nn	11	np	256		
>ref-41806	ATCTCACTCACCTTCCCTCCTTGCCTC	14	<lmxll>	ll	113	lm	153		
>ref-41806	ATCTCACTCACCTTCCCTCCTTGCCTC	21	<lmxll>	ll	112	lm	153		
>ref-41816	CATCTCCTCACTCCCCCTCCACCTGTT	13	<lmxll>	ll	122	lm	117		
>ref-41816	CATCTCCTCACTCCCCCTCCACCTGTT	21	<lmxll>	ll	144	lm	107		
>ref-41816	CATCTCCTCACTCCCCCTCCACCTGTT	22	<lmxll>	ll	119	lm	118		
>ref-41816	CATCTCCTCACTCCCCCTCCACCTGTT	25	<lmxll>	ll	144	lm	107		
>ref-41854	ACTGTCACCACTCTAGCTCCTGCTGCT	2	<nnxnp>	nn	269	np	0		
>ref-41861	AGAACACGTACCGCCTCTCCTGCGGCC	23	<nnxnp>	nn	131	np	140		
>ref-41874	CACCCTTCCACCTCCTCTCCTCCCTCC	2	<lmxll>	ll	14	lm	243		
>ref-41874	CACCCTTCCACCTCCTCTCCTCCCTCC	6	<lmxll>	ll	8	lm	254		
>ref-41874	CACCCTTCCACCTCCTCTCCTCCCTCC	12	<lmxll>	ll	9	lm	254		
>ref-41874	CACCCTTCCACCTCCTCTCCTCCCTCC	22	<nnxnp>	nn	270	np	0		
>ref-41940	TATCCTCTTACTCCCTCTCCTTCTCCC	14	<lmxll>	ll	137	lm	120		
>ref-41984	TAAAGCGAAACCGACACTCCTTTACCC	16	<lmxll>	ll	105	lm	134		
>ref-41989	CACACACACACACTCTCTCCGACTGAC	15	<lmxll>	ll	139	lm	122		
>ref-41989	CACACACACACACTCTCTCCGACTGAC	24	<lmxll>	ll	140	lm	122		
>ref-41991	TCTCCTCTTACCTTTTCTCCTCCTTTC	8	<hkxhk>	hh	0	hk	178	kk	80
>ref-41991	TCTCCTCTTACCTTTTCTCCTCCTTTC	15	<hkxhk>	hh	0	hk	179	kk	80
>ref-41991	TCTCCTCTTACCTTTTCTCCTCCTTTC	23	<nnxnp>	nn	2	np	268		
>ref-41996	TCTTTTTTCACTGGCGCTCCTCCCCGC	14	<nnxnp>	nn	115	np	104		
>ref-41998	CTCGACGGAACTGCTCCTCCTCGGACT	22	<lmxll>	ll	135	lm	129		

>ref-42056	CGAAGTCCGACCGAAGCTCCGACTCAA	3	<nnxnp>	nn	256	np	0		
>ref-42058	CGCCATCATACTTTCCCTCCTTCACTC	24	<lmxll>	ll	123	lm	137		
>ref-42074	TCTTTCCTCACTCTTCCTCCCTTGCCT	13	<lmxll>	ll	122	lm	143		
>ref-42088	TCCTCCCTCACTTCATCTCCCCCCCAT	6	<lmxll>	ll	260	lm	1		
>ref-42088	TCCTCCCTCACTTCATCTCCCCCCCAT	7	<nnxnp>	nn	260	np	0		
>ref-42094	TCTGCCTCCACTTTCACTCCCCTTCCC	5	<nnxnp>	nn	265	np	0		
>ref-42103	TGGATTTTCACTTTCCCTCCGGCTCTG	12	<lmxll>	ll	272	lm	0		
>ref-42122	ACATCATGGACGGAATCTCCTCGGTCT	1	<nnxnp>	nn	269	np	0		
>ref-42122	ACATCATGGACGGAATCTCCTCGGTCT	3	<nnxnp>	nn	271	np	0		
>ref-42123	CAGCTCCTGACTCTGTCTCCATGTTGC	1	<lmxll>	ll	229	lm	39		
>ref-42123	CAGCTCCTGACTCTGTCTCCATGTTGC	14	<lmxll>	ll	229	lm	38		
>ref-42151	ACCCACAACACACCCCCTCCCCCACAC	24	<nnxnp>	nn	132	np	132		
>ref-42153	GACGAAGTGACTGCGGCTCCTTCAAGT	3	<nnxnp>	nn	146	np	112		
>ref-42214	GCACGCGCGACCGGAACTCCAGGAAAA	2	<hkxhk>	hh	40	hk	200	kk	0
>ref-42214	GCACGCGCGACCGGAACTCCAGGAAAA	21	<hkxhk>	hh	6	hk	259	kk	0
>ref-42214	GCACGCGCGACCGGAACTCCAGGAAAA	24	<hkxhk>	hh	6	hk	259	kk	0
>ref-42215	CTTTCCCCTACCTCCCCTCCTCTTTCA	25	<nnxnp>	nn	241	np	3		
>ref-42215	CTTTCCCCTACCTCCCCTCCTCTTTCA	27	<nnxnp>	nn	232	np	4		
>ref-42219	GGGCGACTGACACGCCCTCCTTCCCGT	6	<lmxll>	ll	122	lm	146		
>ref-42224	AGTATCTACACTAACTCTCCTCTCCCC	1	<nnxnp>	nn	259	np	0		
>ref-42266	TCAGAGCTAACTGCATCTCCCATCACT	9	<hkxhk>	hh	1	hk	272	kk	0
>ref-42271	TCTCTCATGACCTTTCCTCCAGGTGTG	23	<nnxnp>	nn	110	np	141		
>ref-42287	GACATACTTACCCCCCCTCCCCCTCTC	1	<lmxll>	ll	132	lm	107		
>ref-42287	GACATACTTACCCCCCCTCCCCCTCTC	26	<lmxll>	ll	126	lm	107		
>ref-42294	TGCATGTGAACGCACGCTCCGCCCTTC	8	<nnxnp>	nn	122	np	143		
>ref-42317	TCTCCCCTTACCACTCCTCCTCTCCTT	4	<lmxll>	ll	4	lm	267		
>ref-42317	TCTCCCCTTACCACTCCTCCTCTCCTT	8	<lmxll>	ll	14	lm	250		
>ref-42317	TCTCCCCTTACCACTCCTCCTCTCCTT	14	<lmxll>	ll	14	lm	250		
>ref-42335	TCCTCGCACACCCCTCCTCCTTCCCAT	13	<lmxll>	ll	124	lm	134		
>ref-42337	GCTTCCAAAACATTGCCTCCAATGCAA	23	<nnxnp>	nn	120	np	107		
>ref-42342	ACTTACCTTACCCATCCTCCCCATCCT	1	<nnxnp>	nn	135	np	133		
>ref-42342	ACTTACCTTACCCATCCTCCCCATCCT	3	<hkxhk>	hh	53	hk	157	kk	57
>ref-42345	ACACTCTCTACCCGCACTCCCTGTCCA	14	<lmxll>	ll	130	lm	140		
>ref-42349	TTTTCTCTTACCTTTTCTCCTTCCTAC	6	<hkxhk>	hh	57	hk	116	kk	53
>ref-42350	ACAAAACCCACGATATCTCCTCGACCT	22	<lmxll>	ll	134	lm	103		
>ref-42353	ACTACTACTACTGCTACTCCTACACTA	12	<nnxnp>	nn	2	np	261		
>ref-42355	CTTTTCTTTACTTTCTCTCCTTCTCCC	1	<nnxnp>	nn	112	np	119		
>ref-42355	CTTTTCTTTACTTTCTCTCCTTCTCCC	15	<lmxll>	ll	122	lm	108		

>ref-42355	CTTTTCTTTACTTTCTCTCCTTCTCCC	25	<nnxnp>	nn	111	np	118		
>ref-42363	TCCATTTCCACCCTTCCTCCACCCTAT	22	<nnxnp>	nn	107	np	151		
>ref-42367	CATTTCCCCACATCCTCTCCCATTCTC	6	<hkxhk>	hh	2	hk	261	kk	5
>ref-42367	CATTTCCCCACATCCTCTCCCATTCTC	22	<hkxhk>	hh	2	hk	261	kk	5
>ref-42370	TCATTACTGACCTTCACTCCCCTACGC	16	<lmxll>	ll	144	lm	117		
>ref-42372	CGCACACAGACGGCGGCTCCACAACTA	25	<lmxll>	ll	132	lm	114		
>ref-42376	TCCTCTTAAACTTTCCCTCCTTCTCCC	8	<lmxll>	ll	129	lm	99		
>ref-42376	TCCTCTTAAACTTTCCCTCCTTCTCCC	21	<lmxll>	ll	2	lm	248		
>ref-42380	GCCCCTCCTACTCGACCTCCCGCCCAA	26	<nnxnp>	nn	140	np	128		
>ref-42380	GCCCCTCCTACTCGACCTCCCGCCCAA	27	<nnxnp>	nn	140	np	128		
>ref-42386	GCCCTTTCCACCTTTTCTCCCTCTATA	4	<hkxhk>	hh	58	hk	143	kk	50
>ref-42387	ACCTCCTCTACTTCTACTCCTCTCTCT	26	<hkxhk>	hh	55	hk	162	kk	43
>ref-42394	CGTCTTCCTACTCTTACTCCTCATCTC	6	<lmxll>	ll	123	lm	112		
>ref-42449	GCATAGCACACTTTTCCTCCTTTGAAT	27	<hkxhk>	hh	53	hk	117	kk	80
>ref-42453	TCTCTCCTCACCTTCCCTCCCACACCC	3	<nnxnp>	nn	0	np	253		
>ref-42453	TCTCTCCTCACCTTCCCTCCCACACCC	4	<lmxll>	ll	265	lm	0		
>ref-42475	CCCTTCTCTACCCCATCTCCCCCTCAC	8	<hkxhk>	hh	10	hk	154	kk	69
>ref-42499	GATTGACACACATACACTCCCTGACAA	27	<nnxnp>	nn	110	np	129		
>ref-42517	AATGTCTAGACACGACCTCCGCGCCCA	15	<nnxnp>	nn	123	np	145		
>ref-42528	GTTCCTGCTACACCCCCTCCCTCTCGT	7	<nnxnp>	nn	126	np	133		
>ref-42533	CCGTCTCCCACTCTCCCTCCCTTATAC	1	<nnxnp>	nn	129	np	135		
>ref-42533	CCGTCTCCCACTCTCCCTCCCTTATAC	16	<nnxnp>	nn	131	np	131		
>ref-42538	AGTTCGCTTACACATCCTCCCACTCAG	1	<nnxnp>	nn	104	np	134		
>ref-42538	AGTTCGCTTACACATCCTCCCACTCAG	26	<nnxnp>	nn	103	np	134		
>ref-42556	ACCCTTCATACTTGCCCTCCTTTCCCA	22	<nnxnp>	nn	127	np	131		
>ref-42577	ACGCCAAGTACGCCATCTCCCTCTCCC	12	<nnxnp>	nn	159	np	101		
>ref-42577	ACGCCAAGTACGCCATCTCCCTCTCCC	22	<hkxhk>	hh	0	hk	273	kk	0
>ref-42583	TCTCCTTCAACTCTTCCTCCCCCTTTA	23	<lmxll>	ll	111	lm	130		
>ref-42592	CCGCAGTTTACCTTTTCTCCTCGTTAC	16	<nnxnp>	nn	137	np	129		
>ref-42624	AGTTTCTTTACTTCCTCTCCTTCCTCA	9	<hkxhk>	hh	0	hk	270	kk	0
>ref-42626	TAAAAATACACTCTGACTCCATCATCA	5	<lmxll>	ll	105	lm	143		
>ref-42626	TAAAAATACACTCTGACTCCATCATCA	21	<lmxll>	ll	181	lm	71		
>ref-42626	TAAAAATACACTCTGACTCCATCATCA	25	<hkxhk>	hh	0	hk	271	kk	2
>ref-42626	TAAAAATACACTCTGACTCCATCATCA	26	<nnxnp>	nn	174	np	81		
>ref-42634	CCCTCTCCCACTCCTACTCCTATTAAT	9	<nnxnp>	nn	31	np	213		
>ref-42634	CCCTCTCCCACTCCTACTCCTATTAAT	25	<nnxnp>	nn	28	np	215		
>ref-42636	CATTATCCAACTCCCTCTCCCACTCCC	25	<lmxll>	ll	86	lm	142		
>ref-42660	CTCTCCTTTACCAACTCTCCCCCTCCC	6	<nnxnp>	nn	125	np	138		

>ref-42667	TGTTGTCATACCCATCCTCCTTTAATT	22	<lmxll>	ll	145	lm	80		
>ref-42671	TCTTTGTCAACTTTTACTCCACAATCA	2	<hkxhk>	hh	0	hk	255	kk	5
>ref-42671	TCTTTGTCAACTTTTACTCCACAATCA	14	<hkxhk>	hh	0	hk	255	kk	5
>ref-42671	TCTTTGTCAACTTTTACTCCACAATCA	26	<hkxhk>	hh	41	hk	188	kk	0
>ref-42685	AGTAAGTTTACCAATACTCCCTTCTAA	26	<nnxnp>	nn	104	np	147		
>ref-42723	TTACCTCTCACATTCTCTCCTTTCCTT	25	<lmxll>	ll	151	lm	114		
>ref-42734	TAGAAGTATACAGAACCTCCATTAACA	3	<lmxll>	ll	137	lm	118		
>ref-42734	TAGAAGTATACAGAACCTCCATTAACA	8	<lmxll>	ll	139	lm	117		
>ref-42761	TCTTTCTGCACGATTGCTCCCTTGACA	2	<nnxnp>	nn	131	np	139		
>ref-42761	TCTTTCTGCACGATTGCTCCCTTGACA	3	<nnxnp>	nn	131	np	139		
>ref-42773	CTCTCTTGTACCCAGTCTCCCTCTCCC	5	<nnxnp>	nn	149	np	123		
>ref-42786	TTTCCTCCTACAACTCCTCCCTTTCTC	13	<nnxnp>	nn	13	np	235		
>ref-42786	TTTCCTCCTACAACTCCTCCCTTTCTC	21	<nnxnp>	nn	14	np	235		
>ref-42801	ATCTGATATACATACCCTCCACAGATC	1	<lmxll>	ll	116	lm	104		
>ref-42840	TCAGCCCCCACCCACTCTCCCTTAGCT	4	<lmxll>	ll	124	lm	140		
>ref-42840	TCAGCCCCCACCCACTCTCCCTTAGCT	14	<lmxll>	ll	123	lm	140		
>ref-42843	CTCGCCCAAACCACGCCTCCCTCGCCC	2	<lmxll>	ll	64	lm	172		
>ref-42843	CTCGCCCAAACCACGCCTCCCTCGCCC	3	<nnxnp>	nn	145	np	115		
>ref-42843	CTCGCCCAAACCACGCCTCCCTCGCCC	23	<nnxnp>	nn	205	np	42		
>ref-42843	CTCGCCCAAACCACGCCTCCCTCGCCC	24	<hkxhk>	hh	6	hk	258	kk	0
>ref-42843	CTCGCCCAAACCACGCCTCCCTCGCCC	26	<lmxll>	ll	65	lm	173		
>ref-42877	AGAGAGGAAACTGTCGCTCCAACGATT	13	<lmxll>	ll	127	lm	135		
>ref-42888	TTCCGTGCAACAGCTCCTCCTGCCGGC	6	<lmxll>	ll	141	lm	124		
>ref-42901	TTTGTCACCACAGGACCTCCAACGTCA	25	<lmxll>	ll	143	lm	123		
>ref-42914	TTGCCCCCTACCCCTCCTCCCCTCCAT	3	<lmxll>	ll	11	lm	252		
>ref-42914	TTGCCCCCTACCCCTCCTCCCCTCCAT	7	<lmxll>	ll	11	lm	252		
>ref-42958	TCATCCGGCACAGTTGCTCCCATTCCT	5	<lmxll>	ll	135	lm	133		
>ref-42958	TCATCCGGCACAGTTGCTCCCATTCCT	26	<hkxhk>	hh	0	hk	273	kk	0
>ref-42962	CATCTACTGACGCCCTCTCCCTCCCCA	15	<hkxhk>	hh	45	hk	219	kk	0
>ref-42970	CATAACACGACCACCCCTCCCTCCCCA	5	<nnxnp>	nn	158	np	111		
>ref-42986	CCTCCCTCTACGTATTCTCCTCCTCTT	5	<lmxll>	ll	136	lm	130		
>ref-42986	CCTCCCTCTACGTATTCTCCTCCTCTT	16	<hkxhk>	hh	0	hk	207	kk	48
>ref-42986	CCTCCCTCTACGTATTCTCCTCCTCTT	24	<hkxhk>	hh	0	hk	208	kk	47
>ref-43005	GGCCAAGCCACACAAGCTCCACCACCT	2	<nnxnp>	nn	2	np	270		
>ref-43005	GGCCAAGCCACACAAGCTCCACCACCT	14	<nnxnp>	nn	2	np	270		
>ref-43005	GGCCAAGCCACACAAGCTCCACCACCT	15	<lmxll>	ll	0	lm	270		
>ref-43005	GGCCAAGCCACACAAGCTCCACCACCT	25	<lmxll>	ll	0	lm	270		
>ref-43011	GTTGTCTTCACTTATCCTCCTCTACCC	25	<hkxhk>	hh	62	hk	146	kk	39

>ref-43026	TCTGTATCTACCGTTTCTCCTTTCATT	4	<hkxhk>	hh	61	hk	146	kk	56
>ref-43034	TCCCACACCACACCCTCTCCCCCTTCC	24	<lmxll>	ll	84	lm	141		
>ref-43034	TCCCACACCACACCCTCTCCCCCTTCC	26	<lmxll>	ll	15	lm	235		
>ref-43041	GGTGTCCAGACAAGAGCTCCTCGAACA	9	<hkxhk>	hh	0	hk	262	kk	0
>ref-43046	ATTACCTCTACCTTCACTCCTACCACG	3	<nnxnp>	nn	133	np	133		
>ref-43051	TACTACACTACCCCTACTCCCTACCCT	26	<lmxll>	ll	119	lm	145		
>ref-43051	TACTACACTACCCCTACTCCCTACCCT	27	<lmxll>	ll	119	lm	145		
>ref-43052	AACACTACTACTACCCCTCCTCAATGC	2	<lmxll>	ll	97	lm	150		
>ref-43052	AACACTACTACTACCCCTCCTCAATGC	3	<hkxhk>	hh	4	hk	264	kk	0
>ref-43052	AACACTACTACTACCCCTCCTCAATGC	12	<hkxhk>	hh	0	hk	264	kk	4
>ref-43052	AACACTACTACTACCCCTCCTCAATGC	16	<hkxhk>	hh	1	hk	271	kk	0
>ref-43052	AACACTACTACTACCCCTCCTCAATGC	24	<hkxhk>	hh	1	hk	271	kk	0
>ref-43052	AACACTACTACTACCCCTCCTCAATGC	27	<lmxll>	ll	97	lm	150		
>ref-43066	AACATCCACACACTTTCTCCTTCCTCC	8	<lmxll>	ll	121	lm	128		
>ref-43073	TGAGTGTGCACCGGTTCTCCTTCTCTT	1	<lmxll>	ll	134	lm	136		
>ref-43073	TGAGTGTGCACCGGTTCTCCTTCTCTT	8	<lmxll>	ll	135	lm	136		
>ref-43101	GCTTCCCCCACCTCCCCTCCCCGCCTC	27	<nnxnp>	nn	141	np	111		
>ref-43115	TCTGCATTCACGTGACCTCCTCTGGGG	23	<nnxnp>	nn	261	np	0		
>ref-43125	CGCCAATCCACCCTTCCTCCTGCTCCT	2	<lmxll>	ll	130	lm	140		
>ref-43125	CGCCAATCCACCCTTCCTCCTGCTCCT	24	<lmxll>	ll	133	lm	138		
>ref-43144	CCAAAGAAAACGGAAACTCCCTATTCT	13	<hkxhk>	hh	0	hk	207	kk	23
>ref-43167	CCATTTATCACTCCCACTCCCCCCATC	8	<hkxhk>	hh	1	hk	262	kk	2
>ref-43167	CCATTTATCACTCCCACTCCCCCCATC	25	<hkxhk>	hh	2	hk	262	kk	1
>ref-43213	TTACCCTCTACTTGCTCTCCCCTCTCG	13	<hkxhk>	hh	0	hk	272	kk	1
>ref-43232	TTTCCCAGAACGAAGTCTCCGCTGCTT	6	<lmxll>	ll	121	lm	141		
>ref-43232	TTTCCCAGAACGAAGTCTCCGCTGCTT	12	<lmxll>	ll	120	lm	141		
>ref-43292	CTCTCGTAAACCTCTCCTCCCGCAAAC	1	<nnxnp>	nn	256	np	1		
>ref-43303	CCTTCCCTTACTTCTCCTCCTTTCCAG	13	<lmxll>	ll	252	lm	0		
>ref-43303	CCTTCCCTTACTTCTCCTCCTTTCCAG	22	<lmxll>	ll	254	lm	0		
>ref-43312	CCTGTTATCACCCTTTCTCCCTCTTAC	4	<nnxnp>	nn	121	np	146		
>ref-43320	CCCAACAAAACCCATTCTCCCTGACTC	16	<hkxhk>	hh	0	hk	249	kk	15
>ref-43320	CCCAACAAAACCCATTCTCCCTGACTC	22	<hkxhk>	hh	0	hk	258	kk	12
>ref-43335	ATCCGAATTACAACGCCTCCGTCCTTA	9	<nnxnp>	nn	136	np	129		
>ref-43354	GTCGATATGACTTTATCTCCATTAGCT	9	<nnxnp>	nn	147	np	112		
>ref-43400	ACCATCACTACTACTTCTCCATGAGGT	8	<hkxhk>	hh	1	hk	270	kk	0
>ref-43400	ACCATCACTACTACTTCTCCATGAGGT	25	<hkxhk>	hh	0	hk	176	kk	76
>ref-43400	ACCATCACTACTACTTCTCCATGAGGT	26	<hkxhk>	hh	0	hk	175	kk	76
>ref-43462	GCACTTCCCACAGGCACTCCTCACAAG	25	<hkxhk>	hh	21	hk	213	kk	0

>ref-43462	GCACTTCCCACAGGCACTCCTCACAAG	26	<hkxhk>	hh	20	hk	215	kk	0
>ref-43465	TTCTCCTCCACCCCTCCTCCGTCGCCG	2	<hkxhk>	hh	48	hk	205	kk	2
>ref-43465	TTCTCCTCCACCCCTCCTCCGTCGCCG	12	<hkxhk>	hh	47	hk	205	kk	3
>ref-43500	AATAGAATGACTGAGCCTCCTGAGAAC	4	<hkxhk>	hh	2	hk	270	kk	0
>ref-43500	AATAGAATGACTGAGCCTCCTGAGAAC	12	<lmxll>	ll	146	lm	93		
>ref-43500	AATAGAATGACTGAGCCTCCTGAGAAC	21	<nnxnp>	nn	124	np	136		
>ref-43515	CTGCCCCGCACTCCTCCTCCCTCATCG	8	<nnxnp>	nn	264	np	0		
>ref-43520	AACCGAGATACTGTTTCTCCTCTCTGT	4	<nnxnp>	nn	117	np	146		
>ref-43535	CTTGCGCCAACCTTTACTCCTCTGTTT	12	<nnxnp>	nn	125	np	134		
>ref-43560	TTCTCCTTCACTTCCCCTCCTTTCTCA	3	<lmxll>	ll	265	lm	1		
>ref-43560	TTCTCCTTCACTTCCCCTCCTTTCTCA	14	<lmxll>	ll	265	lm	0		
>ref-43575	GACCAACAAACCCCTGCTCCGCAATAA	1	<hkxhk>	hh	69	hk	172	kk	0
>ref-43575	GACCAACAAACCCCTGCTCCGCAATAA	3	<hkxhk>	hh	0	hk	263	kk	6
>ref-43575	GACCAACAAACCCCTGCTCCGCAATAA	14	<nnxnp>	nn	186	np	71		
>ref-43575	GACCAACAAACCCCTGCTCCGCAATAA	27	<hkxhk>	hh	0	hk	263	kk	5
>ref-43583	TCTTTTGCCACTTTATCTCCTTTAACT	15	<hkxhk>	hh	58	hk	118	kk	51
>ref-43589	ACGTGTTCAACCTGAACTCCCGCTCCC	1	<lmxll>	ll	191	lm	32		
>ref-43589	ACGTGTTCAACCTGAACTCCCGCTCCC	3	<lmxll>	ll	249	lm	6		
>ref-43592	TTCCTCTTCACTCCCTCTCCGTCACAG	5	<hkxhk>	hh	59	hk	161	kk	46
>ref-43608	TGCATTGTCACATAACCTCCATCACTT	16	<lmxll>	ll	136	lm	129		
>ref-43648	CCTCACTCCACATGCCCTCCCCTTCCT	14	<hkxhk>	hh	0	hk	272	kk	0
>ref-43648	CCTCACTCCACATGCCCTCCCCTTCCT	26	<hkxhk>	hh	0	hk	269	kk	2
>ref-43660	AGAGACCTAACCTAATCTCCCTTTGCT	2	<nnxnp>	nn	141	np	129		
>ref-43662	CCCTGCAACACGCTCTCTCCTCAGAAT	1	<nnxnp>	nn	268	np	0		
>ref-43662	CCCTGCAACACGCTCTCTCCTCAGAAT	3	<nnxnp>	nn	268	np	0		
>ref-43671	TCCCTCACAACTCCCCCTCCATCTCTC	7	<hkxhk>	hh	2	hk	266	kk	0
>ref-43671	TCCCTCACAACTCCCCCTCCATCTCTC	21	<hkxhk>	hh	1	hk	245	kk	0
>ref-43688	ACCTCTCCCACAGAACCTCCACAGCAG	4	<lmxll>	ll	259	lm	10		
>ref-43688	ACCTCTCCCACAGAACCTCCACAGCAG	7	<lmxll>	ll	202	lm	65		
>ref-43688	ACCTCTCCCACAGAACCTCCACAGCAG	24	<hkxhk>	hh	0	hk	267	kk	4
>ref-43688	ACCTCTCCCACAGAACCTCCACAGCAG	27	<hkxhk>	hh	0	hk	154	kk	112
>ref-43691	CTTTATCTCACTTTCCCTCCCCCTTCT	9	<lmxll>	ll	250	lm	1		
>ref-43691	CTTTATCTCACTTTCCCTCCCCCTTCT	14	<lmxll>	ll	255	lm	1		
>ref-43715	TCTCTCCCAACCTTCACTCCCTTCCTT	1	<nnxnp>	nn	13	np	244		
>ref-43715	TCTCTCCCAACCTTCACTCCCTTCCTT	2	<nnxnp>	nn	267	np	0		
>ref-43716	CCTCTGTCTACCTTTCCTCCTTCCTTG	6	<nnxnp>	nn	124	np	137		
>ref-43716	CCTCTGTCTACCTTTCCTCCTTCCTTG	12	<nnxnp>	nn	119	np	136		
>ref-43730	CTCTACCCCACCTTCTCTCCCTCTTCC	1	<nnxnp>	nn	233	np	0		

>ref-43730	CTCTACCCCACCTTCTCTCCCTCTTCC	4	<lmxll>	ll	231	lm	0			
>ref-43730	CTCTACCCCACCTTCTCTCCCTCTTCC	6	<nnxnp>	nn	227	np	3			
>ref-43730	CTCTACCCCACCTTCTCTCCCTCTTCC	12	<lmxll>	ll	220	lm	7			
>ref-43730	CTCTACCCCACCTTCTCTCCCTCTTCC	16	<nnxnp>	nn	222	np	7			
>ref-43743	TGCAAAGGTACTGCCACTCCGTTCCAA	2	<hkxhk>	hh	64	hk	87	kk	83
>ref-43744	CTCTCATCAACCCTCCCTCCCTTCCCT	4	<nnxnp>	nn	102	np	119		
>ref-43744	CTCTCATCAACCCTCCCTCCCTTCCCT	6	<nnxnp>	nn	217	np	3		
>ref-43744	CTCTCATCAACCCTCCCTCCCTTCCCT	7	<nnxnp>	nn	236	np	1		
>ref-43744	CTCTCATCAACCCTCCCTCCCTTCCCT	15	<nnxnp>	nn	112	np	114		
>ref-43744	CTCTCATCAACCCTCCCTCCCTTCCCT	16	<nnxnp>	nn	218	np	9		
>ref-43744	CTCTCATCAACCCTCCCTCCCTTCCCT	24	<lmxll>	ll	134	lm	86		
>ref-43747	TCCACCTCCACGGTATCTCCCTCTTCC	2	<hkxhk>	hh	56	hk	137	kk	55
>ref-43755	ATTAATATCACATCATCTCCACGTCCA	9	<hkxhk>	hh	49	hk	120	kk	55
>ref-43775	GCGGTCCTAACGGCCCCTCCTTCCCTT	25	<lmxll>	ll	145	lm	114		
>ref-43831	AGACTGTTAACTGAGGCTCCTTTTCCG	4	<lmxll>	ll	122	lm	121		
>ref-43847	CTTCTTACCACCTCCCCTCCTTCCCTA	5	<nnxnp>	nn	129	np	123		
>ref-43859	AAGATCCTCACATCCACTCCTTCACAC	14	<lmxll>	ll	120	lm	139		
>ref-43883	CCCTTCTGTACCCCTCCTCCTTCTCTT	14	<nnxnp>	nn	257	np	0		
>ref-43883	CCCTTCTGTACCCCTCCTCCTTCTCTT	22	<nnxnp>	nn	254	np	1		
>ref-43901	AAGATACCAACAACGCCTCCAGTAGAC	16	<lmxll>	ll	137	lm	107		
>ref-43912	CCAAATGCGACTTGAACTCCCTTGTCA	6	<nnxnp>	nn	2	np	254		
>ref-43927	GAATCATAAACACAGTCTCCTCATAAT	3	<nnxnp>	nn	112	np	138		
>ref-44031	ATTTAAGGGACCATATCTCCTCAACGA	1	<lmxll>	ll	131	lm	126		
>ref-44031	ATTTAAGGGACCATATCTCCTCAACGA	14	<nnxnp>	nn	122	np	141		
>ref-44037	CCCCCTCCTACAACCTCTCCCTTCCTC	1	<nnxnp>	nn	59	np	202		
>ref-44037	CCCCCTCCTACAACCTCTCCCTTCCTC	7	<lmxll>	ll	130	lm	133		
>ref-44037	CCCCCTCCTACAACCTCTCCCTTCCTC	8	<lmxll>	ll	130	lm	133		
>ref-44053	TACTCCTGCACTCCTGCTCCTTCTTTC	21	<hkxhk>	hh	41	hk	164	kk	57
>ref-44083	AAGATCTGAACCTTCCCTCCTCCTCTC	21	<hkxhk>	hh	55	hk	130	kk	62
>ref-44092	CTTTCTCTCACTGCCCCTCCTCCTGCC	14	<lmxll>	ll	128	lm	130		
>ref-44104	ACGAGTACGACGTGCCCTCCAGCTCGA	22	<nnxnp>	nn	149	np	124		
>ref-44156	GGCTCTGTTACTTTCCCTCCTTCTCTT	6	<hkxhk>	hh	0	hk	211	kk	49
>ref-44156	GGCTCTGTTACTTTCCCTCCTTCTCTT	16	<nnxnp>	nn	155	np	109		
>ref-44156	GGCTCTGTTACTTTCCCTCCTTCTCTT	21	<hkxhk>	hh	0	hk	211	kk	51
>ref-44160	CAATGGCTGACCAATCCTCCTTGTCCA	8	<lmxll>	ll	138	lm	130		
>ref-44160	CAATGGCTGACCAATCCTCCTTGTCCA	9	<lmxll>	ll	271	lm	0		
>ref-44160	CAATGGCTGACCAATCCTCCTTGTCCA	24	<nnxnp>	nn	129	np	141		
>ref-44164	ACCTCTATGACCTGACCTCCATCGCTT	7	<lmxll>	ll	126	lm	144		

>ref-44167	CGCCTCCAAACTCCACCTCCACCTCCA	2	<lmxll>	ll	89	lm	141		
>ref-44167	CGCCTCCAAACTCCACCTCCACCTCCA	22	<lmxll>	ll	148	lm	86		
>ref-44178	AGCTATGACACACGCGCTCCTCTCTCT	1	<hkxhk>	hh	51	hk	142	kk	73
>ref-44184	CTTTACCCTACATTCTCTCCCTTTTCC	5	<nnxnp>	nn	15	np	252		
>ref-44184	CTTTACCCTACATTCTCTCCCTTTTCC	9	<nnxnp>	nn	15	np	252		
>ref-44247	AAATAATAAACATCCCCTCCTTCCTCT	23	<nnxnp>	nn	135	np	113		
>ref-44250	TCTTCTCTCACCCTGTCTCCCTGTCTC	3	<lmxll>	ll	8	lm	262		
>ref-44250	TCTTCTCTCACCCTGTCTCCCTGTCTC	8	<lmxll>	ll	8	lm	262		
>ref-44264	CCTTTTTTTACTATCTCTCCCTCTCTC	16	<lmxll>	ll	113	lm	106		
>ref-44267	ATATGCGGGACATAAACTCCACATATA	7	<nnxnp>	nn	132	np	124		
>ref-44271	CGCATTCGGACAAGGGCTCCGTATGAC	27	<nnxnp>	nn	273	np	0		
>ref-44276	CCCGTTACTACGAATCCTCCCTCTCCT	21	<lmxll>	ll	134	lm	127		
>ref-44277	TTCTGGATCACCCCTCCTCCTCCTCCT	5	<nnxnp>	nn	267	np	1		
>ref-44277	TTCTGGATCACCCCTCCTCCTCCTCCT	6	<nnxnp>	nn	270	np	1		
>ref-44278	ACAACACAAACCATCTCTCCTTGAACT	27	<hkxhk>	hh	52	hk	150	kk	58
>ref-44293	TTATCAATCACTCTCACTCCCTCTATC	25	<lmxll>	ll	219	lm	25		
>ref-44295	GTGTCTTACACTCCATCTCCAGGCATG	27	<lmxll>	ll	134	lm	133		
>ref-44297	TTTCTTGGCACCAGCTCTCCTTCCCTT	4	<lmxll>	ll	264	lm	0		
>ref-44297	TTTCTTGGCACCAGCTCTCCTTCCCTT	23	<nnxnp>	nn	123	np	137		
>ref-44309	CGCCTCTCGACCTTCGCTCCTCCGCCC	8	<lmxll>	ll	115	lm	145		
>ref-44336	ATCCCCTCTACCGACCCTCCATTCCCT	13	<hkxhk>	hh	1	hk	265	kk	1
>ref-44375	AAAACAACAACAACAACTCCAACTGCT	3	<lmxll>	ll	135	lm	128		
>ref-44378	GCTCGCTCTACCATTCCTCCTTCTCCC	12	<nnxnp>	nn	131	np	137		
>ref-44379	GATTGACGTACCTGACCTCCCCAGTAT	1	<nnxnp>	nn	233	np	30		
>ref-44379	GATTGACGTACCTGACCTCCCCAGTAT	3	<nnxnp>	nn	233	np	30		
>ref-44393	AGCAAGGGGACTCTGCCTCCTGGACCC	4	<hkxhk>	hh	2	hk	253	kk	8
>ref-44400	CAGACACGAACAGTAACTCCACAAATA	4	<hkxhk>	hh	6	hk	265	kk	0
>ref-44400	CAGACACGAACAGTAACTCCACAAATA	8	<hkxhk>	hh	0	hk	238	kk	8
>ref-44400	CAGACACGAACAGTAACTCCACAAATA	21	<nnxnp>	nn	99	np	159		
>ref-44400	CAGACACGAACAGTAACTCCACAAATA	25	<hkxhk>	hh	0	hk	273	kk	0
>ref-44422	AGCCTTTCAACATGTCCTCCTCAAAAT	13	<lmxll>	ll	111	lm	123		
>ref-44467	TCTTCACACACCCCCCCTCCCCTTCCC	8	<lmxll>	ll	229	lm	4		
>ref-44467	TCTTCACACACCCCCCCTCCCCTTCCC	12	<lmxll>	ll	226	lm	4		
>ref-44467	TCTTCACACACCCCCCCTCCCCTTCCC	14	<lmxll>	ll	205	lm	30		
>ref-44467	TCTTCACACACCCCCCCTCCCCTTCCC	27	<lmxll>	ll	243	lm	0		
>ref-44469	TCTACGATGACCTTAACTCCACCATCG	6	<lmxll>	ll	188	lm	72		
>ref-44469	TCTACGATGACCTTAACTCCACCATCG	12	<nnxnp>	nn	139	np	117		
>ref-44469	TCTACGATGACCTTAACTCCACCATCG	21	<nnxnp>	nn	140	np	116		

>ref-44475	TCCCAACGAACTTTAGCTCCAAGACAG	9	<hkxhk>	hh	0	hk	273	kk	0
>ref-44475	TCCCAACGAACTTTAGCTCCAAGACAG	21	<hkxhk>	hh	0	hk	272	kk	1
>ref-44520	TCGTTTAGTACAGTTGCTCCACGTGCA	4	<hkxhk>	hh	69	hk	111	kk	47
>ref-44548	CCCACTTCTACCTCTCCTCCCTCTCTT	3	<nnxnp>	nn	146	np	126		
>ref-44548	CCCACTTCTACCTCTCCTCCCTCTCTT	15	<hkxhk>	hh	21	hk	174	kk	74
>ref-44570	AGCCTGGTAACACTGGCTCCTTTCTCT	12	<lmxll>	ll	267	lm	0		
>ref-44595	AACTACTTAACCACCTCTCCCTCCTAT	14	<lmxll>	ll	119	lm	136		
>ref-44629	TTCCACCCGACCCTCTCTCCCTCGCCT	5	<hkxhk>	hh	79	hk	142	kk	43
>ref-44636	CTCACACACACCTCCACTCCTCCATCC	12	<nnxnp>	nn	108	np	161		
>ref-44636	CTCACACACACCTCCACTCCTCCATCC	23	<nnxnp>	nn	108	np	161		
>ref-44638	CATACAAATACAAGCTCTCCCTCTCTT	15	<nnxnp>	nn	134	np	108		
>ref-44638	CATACAAATACAAGCTCTCCCTCTCTT	25	<lmxll>	ll	116	lm	117		
>ref-44638	CATACAAATACAAGCTCTCCCTCTCTT	27	<lmxll>	ll	108	lm	119		
>ref-44646	TAAAGACAAACACTAACTCCTCGGAGG	26	<lmxll>	ll	112	lm	135		
>ref-44652	CCTTTCCCCACCCATCCTCCCTTCCAC	3	<nnxnp>	nn	113	np	112		
>ref-44652	CCTTTCCCCACCCATCCTCCCTTCCAC	26	<nnxnp>	nn	105	np	114		
>ref-44655	CCCAAAGGCACAAAGCCTCCTGTATAA	15	<nnxnp>	nn	118	np	105		
>ref-44657	TGCCCATTAACACATCCTCCTATGCCT	2	<nnxnp>	nn	125	np	141		
>ref-44657	TGCCCATTAACACATCCTCCTATGCCT	15	<nnxnp>	nn	125	np	141		
>ref-44658	ACCTCTGCCACAAAATCTCCCTGGTCT	14	<nnxnp>	nn	141	np	102		
>ref-44694	GTCTGGGTTACCAATGCTCCCTGTGGA	6	<nnxnp>	nn	270	np	0		
>ref-44696	TACCTGAATACCTTCTCTCCGCATACA	15	<nnxnp>	nn	15	np	215		
>ref-44696	TACCTGAATACCTTCTCTCCGCATACA	26	<nnxnp>	nn	15	np	215		
>ref-44718	TCTCTCCTCACCTGCCCTCCCATCCTT	27	<nnxnp>	nn	127	np	135		
>ref-44726	GCTGGCTCAACCGATGCTCCTTCATAT	23	<lmxll>	ll	124	lm	147		
>ref-44780	ATAGGACTAACAGTCCCTCCCGCTTCT	7	<lmxll>	ll	221	lm	29		
>ref-44783	GCCCTTTAAACAAAAGCTCCATGCCCC	15	<lmxll>	ll	112	lm	125		
>ref-44783	GCCCTTTAAACAAAAGCTCCATGCCCC	27	<lmxll>	ll	110	lm	126		
>ref-44797	AAGCATCATACTCAGCCTCCTGTGTAT	3	<lmxll>	ll	119	lm	148		
>ref-44799	ATCTCCTTCACTCTCCCTCCTTCCCCC	2	<hkxhk>	hh	0	hk	254	kk	12
>ref-44799	ATCTCCTTCACTCTCCCTCCTTCCCCC	6	<hkxhk>	hh	14	hk	252	kk	0
>ref-44816	CGTCTTCCTACTTGTCCTCCTCTCCTT	23	<nnxnp>	nn	249	np	1		
>ref-44820	AGAGCTGCCACGCCTTCTCCCGATGTC	27	<nnxnp>	nn	12	np	246		
>ref-44822	CTCGCTCCTACAGCCTCTCCCTCCTTC	27	<hkxhk>	hh	70	hk	138	kk	51
>ref-44829	CCAACATTAACCCAACCTCCACAGATT	4	<lmxll>	ll	127	lm	114		
>ref-44844	TCGCCGGTAACTATGGCTCCATCGACT	26	<nnxnp>	nn	267	np	0		
>ref-44849	CTTCATCGTACTTTCCCTCCTCCCCAT	5	<nnxnp>	nn	99	np	131		
>ref-44877	TGCATATATACCTCTCCTCCTCTCTCC	6	<nnxnp>	nn	112	np	139		

>ref-44886	CATTCAATTACGCTTCCTCCTTTCTTC	7	<nnxnp>	nn	128	np	132		
>ref-44900	ATCTTTATTACCCTCCCTCCCTTCTTT	25	<nnxnp>	nn	104	np	139		
>ref-44932	AAAACCCGCACTCACACTCCCGTACCC	6	<lmxll>	ll	100	lm	154		
>ref-44944	TCCCTCCCCACACACACTCCTTCAGCT	26	<hkxhk>	hh	54	hk	141	kk	58
>ref-44973	CAGTCTCTCACTCCCTCTCCTTTTATC	22	<nnxnp>	nn	122	np	143		
>ref-44983	CACAACCCCACTCCCCCTCCACACACA	1	<lmxll>	ll	134	lm	130		
>ref-44990	CACGAATTCACCATTACTCCTTGCCCT	13	<lmxll>	ll	125	lm	138		
>ref-45012	ATCTTAATCACTTTGACTCCGGCGACG	13	<lmxll>	ll	98	lm	138		
>ref-45049	GTCTTCTGTACTGTCTCTCCTCCCATT	1	<lmxll>	ll	108	lm	152		
>ref-45104	CTCGCGTCCACACTGCCTCCCTCCCCA	6	<nnxnp>	nn	124	np	145		
>ref-45106	ATTCTATTAACCTCTCCTCCGCCTCTT	13	<lmxll>	ll	120	lm	140		
>ref-45106	ATTCTATTAACCTCTCCTCCGCCTCTT	27	<lmxll>	ll	131	lm	128		
>ref-45111	ACCCCTTTCACCTTCCCTCCCTATCTC	7	<nnxnp>	nn	120	np	141		
>ref-45111	ACCCCTTTCACCTTCCCTCCCTATCTC	8	<nnxnp>	nn	267	np	0		
>ref-45123	CCTAAAACTACCCTCCCTCCCCCACCG	25	<nnxnp>	nn	120	np	112		
>ref-45126	ACATGAACAACAATCCCTCCCTTACCC	4	<lmxll>	ll	110	lm	129		
>ref-45182	CATCCTTTCACATTCTCTCCCATCCTC	7	<lmxll>	ll	132	lm	118		
>ref-45185	ATTCCCTATACCCATCCTCCTTCTCTT	13	<nnxnp>	nn	127	np	134		
>ref-45191	GAAGTTGTCACGCAAGCTCCTATGACA	7	<lmxll>	ll	143	lm	117		
>ref-45232	CCGCAACCTACTTCCTCTCCTTTCCCT	3	<nnxnp>	nn	133	np	130		
>ref-45246	GCCACCACAACCGATGCTCCCACAACC	21	<nnxnp>	nn	119	np	134		
>ref-45247	GATACCGTCACGGCCCCTCCTTCCCTC	21	<nnxnp>	nn	144	np	122		
>ref-45254	AGTTTGGCAACATGCGCTCCCCTCTCT	5	<nnxnp>	nn	259	np	0		
>ref-45254	AGTTTGGCAACATGCGCTCCCCTCTCT	26	<nnxnp>	nn	262	np	0		
>ref-45261	AGCCCGCCGACCTAGTCTCCTCCGCTG	5	<nnxnp>	nn	137	np	132		
>ref-45272	TCAATCATCACCAAAGCTCCATTAGCA	16	<nnxnp>	nn	147	np	119		
>ref-45272	TCAATCATCACCAAAGCTCCATTAGCA	25	<lmxll>	ll	101	lm	166		
>ref-45279	CTATCTCTAACGCCATCTCCCTCTCCA	15	<lmxll>	ll	144	lm	127		
>ref-45301	CTCAACCTCACCCCTCCTCCTTTCCCT	3	<lmxll>	ll	256	lm	0		
>ref-45310	TGTACATCCACCAAACCTCCCTTTTAC	8	<hkxhk>	hh	1	hk	272	kk	0
>ref-45310	TGTACATCCACCAAACCTCCCTTTTAC	15	<hkxhk>	hh	1	hk	272	kk	0
>ref-45310	TGTACATCCACCAAACCTCCCTTTTAC	25	<hkxhk>	hh	0	hk	269	kk	1
>ref-45313	CCACCATCCACATCTCCTCCTTCTCCC	24	<nnxnp>	nn	5	np	244		
>ref-45313	CCACCATCCACATCTCCTCCTTCTCCC	25	<nnxnp>	nn	5	np	246		
>ref-45318	TCCTGCCTAACAAGCCCTCCTCGCAAA	23	<hkxhk>	hh	2	hk	264	kk	2
>ref-45318	TCCTGCCTAACAAGCCCTCCTCGCAAA	26	<hkxhk>	hh	2	hk	264	kk	2
>ref-45320	TTACCCGACACTCCCCCTCCTCTCTTC	6	<nnxnp>	nn	123	np	133		
>ref-45337	AAGCATCTAACCGCTCCTCCGACCTGT	24	<nnxnp>	nn	125	np	142		

>ref-45341	CAAGTAGAAACCCTCCCTCCACTGCCT	3	<hkxhk>	hh	67	hk	147	kk	53
>ref-45352	TTTCAACTCACTCCCTCTCCTTTCCTC	6	<nnxnp>	nn	256	np	4		
>ref-45352	TTTCAACTCACTCCCTCTCCTTTCCTC	26	<nnxnp>	nn	255	np	4		
>ref-45361	TTTTTCACCACTTAAGCTCCAGTGTCC	15	<nnxnp>	nn	122	np	98		
>ref-45361	TTTTTCACCACTTAAGCTCCAGTGTCC	22	<nnxnp>	nn	122	np	97		
>ref-45367	TCCCCTCCCACATAATCTCCCCTCAGC	26	<nnxnp>	nn	0	np	246		
>ref-45377	TTACCCTCAACCCCCCCTCCCCCTCCC	3	<nnxnp>	nn	61	np	175		
>ref-45377	TTACCCTCAACCCCCCCTCCCCCTCCC	9	<nnxnp>	nn	19	np	227		
>ref-45377	TTACCCTCAACCCCCCCTCCCCCTCCC	12	<nnxnp>	nn	26	np	234		
>ref-45377	TTACCCTCAACCCCCCCTCCCCCTCCC	15	<nnxnp>	nn	89	np	150		
>ref-45389	AGGATGTGCACGGGAGCTCCGGGCAAG	4	<hkxhk>	hh	47	hk	157	kk	64
>ref-45403	CGTGTAAAAACTATTCCTCCTCCTCCT	2	<lmxll>	ll	15	lm	232		
>ref-45403	CGTGTAAAAACTATTCCTCCTCCTCCT	5	<lmxll>	ll	16	lm	232		
>ref-45403	CGTGTAAAAACTATTCCTCCTCCTCCT	26	<hkxhk>	hh	3	hk	253	kk	2
>ref-45412	TCTTCGACAACTTGTTCTCCTTCAGAT	7	<hkxhk>	hh	4	hk	261	kk	0
>ref-45412	TCTTCGACAACTTGTTCTCCTTCAGAT	27	<hkxhk>	hh	0	hk	260	kk	4
>ref-45417	GCCAATTCGACGTCATCTCCCACGAAC	21	<lmxll>	ll	101	lm	132		
>ref-45456	CTCCCACCTACCTCAACTCCCTTTCTC	1	<nnxnp>	nn	270	np	0		
>ref-45464	TGTGCTGCCACATGGTCTCCAGGTATT	1	<hkxhk>	hh	0	hk	125	kk	132
>ref-45464	TGTGCTGCCACATGGTCTCCAGGTATT	9	<hkxhk>	hh	0	hk	124	kk	133
>ref-45464	TGTGCTGCCACATGGTCTCCAGGTATT	21	<lmxll>	ll	116	lm	139		
>ref-45464	TGTGCTGCCACATGGTCTCCAGGTATT	22	<nnxnp>	nn	16	np	248		
>ref-45466	ATCTTCTCTACAGCCCCTCCTCGCTGT	6	<nnxnp>	nn	265	np	0		
>ref-45480	CGTGCAGACACCCCCACTCCCCTATTT	21	<nnxnp>	nn	114	np	149		
>ref-45481	CCACGCCCCACCCATCCTCCTCCTACC	9	<hkxhk>	hh	9	hk	261	kk	0
>ref-45481	CCACGCCCCACCCATCCTCCTCCTACC	13	<hkxhk>	hh	0	hk	273	kk	0
>ref-45491	CTTCCTTTTACTAAGTCTCCCTATCCA	21	<nnxnp>	nn	113	np	117		
>ref-45505	TCGAGAGGGACACCAGCTCCATGACAG	24	<hkxhk>	hh	1	hk	271	kk	0
>ref-45518	ATGCAAATGACCCTTTCTCCTTGAAAT	22	<nnxnp>	nn	118	np	116		
>ref-45541	TTGCCATCTACACCATCTCCATCACCT	2	<nnxnp>	nn	146	np	109		
>ref-45561	ACAGTCATTACTCTCTCTCCTCTCTCT	13	<hkxhk>	hh	0	hk	269	kk	2
>ref-45561	ACAGTCATTACTCTCTCTCCTCTCTCT	14	<hkxhk>	hh	0	hk	269	kk	2
>ref-45565	CTTGTAAACACCATAACTCCCTTGTCA	24	<hkxhk>	hh	0	hk	205	kk	35
>ref-45565	CTTGTAAACACCATAACTCCCTTGTCA	26	<hkxhk>	hh	35	hk	204	kk	0
>ref-45569	TCTCTGGCAACTTCTCCTCCCTGTCTC	12	<lmxll>	ll	112	lm	145		
>ref-45569	TCTCTGGCAACTTCTCCTCCCTGTCTC	14	<lmxll>	ll	112	lm	146		
>ref-45577	AGCCAATACACGTGTCCTCCTGCATCT	24	<nnxnp>	nn	132	np	138		
>ref-45585	ACAAGAGGGACACATCCTCCACCTGTT	2	<hkxhk>	hh	4	hk	267	kk	0

>ref-45585	ACAAGAGGGACACATCCTCCACCTGTT	3	<hkxhk>	hh	4	hk	267	kk	0
>ref-45589	CTCTGAAAAACACCACCTCCCGCCCCT	24	<nnxnp>	nn	110	np	119		
>ref-45594	TCTGTGCCAACGCAGACTCCGGAACAA	3	<nnxnp>	nn	134	np	136		
>ref-45602	AGAACATAAACCAGACCTCCTTTTAGC	1	<lmxll>	ll	154	lm	105		
>ref-45602	AGAACATAAACCAGACCTCCTTTTAGC	2	<lmxll>	ll	155	lm	105		
>ref-45622	CGTGCGCGTACGTGTGCTCCATGCATG	14	<nnxnp>	nn	132	np	134		
>ref-45646	CCCCCCCTAACCCCTTCTCCTCCACTC	24	<nnxnp>	nn	230	np	5		
>ref-45687	TCTACCCATACTCACACTCCTGCTTTC	4	<nnxnp>	nn	145	np	114		
>ref-45687	TCTACCCATACTCACACTCCTGCTTTC	7	<nnxnp>	nn	145	np	114		
>ref-45701	AACTAAAATACTCTCTCTCCTTCTCTT	24	<lmxll>	ll	113	lm	128		
>ref-45711	CGCCCCCCCACAGGACCTCCCTGCACT	24	<lmxll>	ll	114	lm	150		
>ref-45737	CCTTAAAACACATATACTCCCTCCCCT	3	<nnxnp>	nn	141	np	104		
>ref-45737	CCTTAAAACACATATACTCCCTCCCCT	4	<hkxhk>	hh	0	hk	243	kk	20
>ref-45737	CCTTAAAACACATATACTCCCTCCCCT	13	<hkxhk>	hh	0	hk	233	kk	31
>ref-45737	CCTTAAAACACATATACTCCCTCCCCT	21	<nnxnp>	nn	141	np	104		
>ref-45737	CCTTAAAACACATATACTCCCTCCCCT	23	<lmxll>	ll	235	lm	18		
>ref-45737	CCTTAAAACACATATACTCCCTCCCCT	26	<nnxnp>	nn	72	np	165		
>ref-45743	AACACTGCTACTTAACCTCCAGGAGAC	22	<nnxnp>	nn	122	np	128		
>ref-45773	GTCTCCCTTACCTTTCCTCCCCTTTTT	12	<lmxll>	ll	195	lm	56		
>ref-45780	CACTCCCTAACCGTCTCTCCTCACTCC	25	<nnxnp>	nn	138	np	123		
>ref-45786	ACTTCTAGAACCTCCACTCCAAGGATT	3	<nnxnp>	nn	122	np	142		
>ref-45788	CGTGTAGGTACCCCACCTCCACTCTTC	14	<hkxhk>	hh	79	hk	182	kk	0
>ref-45789	ATTACGACTACTACTACTCCTATCATC	27	<hkxhk>	hh	6	hk	224	kk	4
>ref-45863	CTTTACTACACACCGTCTCCCTAATGA	14	<nnxnp>	nn	108	np	140		
>ref-45867	TTGCAACATACCGTCCCTCCAATATCA	2	<nnxnp>	nn	129	np	130		
>ref-45869	AATACTCATACTCATACTCCCTCACAC	1	<hkxhk>	hh	0	hk	267	kk	1
>ref-45869	AATACTCATACTCATACTCCCTCACAC	26	<hkxhk>	hh	0	hk	269	kk	0
>ref-45872	CAAGCACCCACGTAACCTCCATATAAG	6	<lmxll>	ll	120	lm	137		
>ref-45872	CAAGCACCCACGTAACCTCCATATAAG	27	<nnxnp>	nn	270	np	0		
>ref-45899	TACGAGAGTACAGTTACTCCTCCCTTG	27	<lmxll>	ll	111	lm	130		
>ref-45907	CCTTCTCCCACCCCACCTCCTTCTCTC	14	<hkxhk>	hh	1	hk	266	kk	1
>ref-45907	CCTTCTCCCACCCCACCTCCTTCTCTC	21	<hkxhk>	hh	1	hk	267	kk	1
>ref-45908	AACACGGCCACGTTATCTCCTTATCAG	26	<lmxll>	ll	137	lm	123		
>ref-45922	ATTCTCTCCACCTCATCTCCTCCAATC	9	<nnxnp>	nn	133	np	131		
>ref-45955	GTCTATCCCACTTCGCCTCCCTTCTAC	14	<lmxll>	ll	126	lm	123		
>ref-45962	CATCTCCACACACGTTCTCCAACACAC	27	<nnxnp>	nn	122	np	146		
>ref-45964	ATCCCCACCACCTCCCCTCCATCCCCA	8	<lmxll>	ll	162	lm	91		
>ref-45964	ATCCCCACCACCTCCCCTCCATCCCCA	9	<nnxnp>	nn	9	np	258		

>ref-45964	ATCCCCACCACCTCCCCTCCATCCCCA	14	<hkxhk>	hh	130	hk	137	kk	0
>ref-45979	CGGTCGAGGACGAGCACTCCGGTGACA	6	<nnxnp>	nn	138	np	132		
>ref-45979	CGGTCGAGGACGAGCACTCCGGTGACA	21	<lmxll>	ll	142	lm	128		
>ref-45979	CGGTCGAGGACGAGCACTCCGGTGACA	23	<nnxnp>	nn	138	np	132		
>ref-46013	CTCCTCTTAACCTTTCCTCCCTCTCTC	3	<lmxll>	ll	272	lm	0		
>ref-46013	CTCCTCTTAACCTTTCCTCCCTCTCTC	8	<hkxhk>	hh	0	hk	261	kk	5
>ref-46013	CTCCTCTTAACCTTTCCTCCCTCTCTC	14	<hkxhk>	hh	0	hk	260	kk	6
>ref-46020	TAGCCCTTAACCAGTACTCCAGTCTTA	1	<nnxnp>	nn	122	np	147		
>ref-46025	GCAATAGACACAATAGCTCCAAAGTCA	24	<lmxll>	ll	100	lm	123		
>ref-46026	TTGATATTCACGATGCCTCCGCGCCTC	3	<lmxll>	ll	129	lm	135		
>ref-46037	TCTCCTTTGACTTGTTCTCCTTCCTCA	2	<hkxhk>	hh	138	hk	115	kk	0
>ref-46037	TCTCCTTTGACTTGTTCTCCTTCCTCA	5	<hkxhk>	hh	140	hk	109	kk	0
>ref-46042	TCCTCTCCCACCTTTTCTCCTTGCAAT	1	<lmxll>	ll	138	lm	127		
>ref-46056	ACACCACATACACACACTCCAACCATA	21	<lmxll>	ll	152	lm	112		
>ref-46076	CCTTTTACAACTCCGCCTCCTTCTCTC	27	<lmxll>	ll	219	lm	3		
>ref-46096	TTCTCCCTCACTCCTCCTCCTTTATCT	21	<hkxhk>	hh	0	hk	225	kk	25
>ref-46096	TTCTCCCTCACTCCTCCTCCTTTATCT	27	<hkxhk>	hh	0	hk	225	kk	23
>ref-46102	CTCTTCCCTACTTCTTCTCCACCATCT	1	<nnxnp>	nn	139	np	134		
>ref-46102	CTCTTCCCTACTTCTTCTCCACCATCT	3	<hkxhk>	hh	5	hk	265	kk	0
>ref-46161	CTCCCACTCACGCGCCCTCCCGCCATA	23	<hkxhk>	hh	78	hk	133	kk	49
>ref-46165	AATCCCTCTACTCCCCCTCCTTCCCTC	2	<lmxll>	ll	236	lm	1		
>ref-46165	AATCCCTCTACTCCCCCTCCTTCCCTC	14	<lmxll>	ll	242	lm	2		
>ref-46167	GCTAGGCGCACCACCTCTCCAGGACCG	14	<nnxnp>	nn	142	np	128		
>ref-46177	TTCATTTTAACCCTCTCTCCTTTTATC	1	<lmxll>	ll	112	lm	138		
>ref-46187	ACAGACAACACGATCCCTCCAAAGCAA	24	<lmxll>	ll	134	lm	125		
>ref-46203	CCAGCTTGGACGAAGCCTCCTCTACCC	2	<nnxnp>	nn	115	np	144		
>ref-46213	TGCTCTTTCACCCCCACTCCAAGCCAG	4	<lmxll>	ll	104	lm	135		
>ref-46225	ACTGTTACGACCACCACTCCTAAACAT	2	<lmxll>	ll	121	lm	127		
>ref-46226	CACCACGTGACTCCTGCTCCTCATGTC	3	<lmxll>	ll	133	lm	133		
>ref-46234	CGTGGCTTCACTCTCCCTCCTTCCTTT	4	<nnxnp>	nn	132	np	137		
>ref-46235	TCCCTTCGTACCTTCTCTCCCCCCCTT	4	<lmxll>	ll	264	lm	0		
>ref-46235	TCCCTTCGTACCTTCTCTCCCCCCCTT	8	<lmxll>	ll	80	lm	163		
>ref-46235	TCCCTTCGTACCTTCTCTCCCCCCCTT	25	<hkxhk>	hh	34	hk	218	kk	0
>ref-46235	TCCCTTCGTACCTTCTCTCCCCCCCTT	27	<nnxnp>	nn	128	np	132		
>ref-46238	CACCTTTTTACCCCTCCTCCTCCTCCG	1	<nnxnp>	nn	0	np	254		
>ref-46276	ACATCCTCCACCACCACTCCTACCTCC	4	<hkxhk>	hh	44	hk	167	kk	56
>ref-46290	CTCTCCTTCACTCCCCCTCCTTGGGCT	22	<nnxnp>	nn	147	np	119		
>ref-46290	CTCTCCTTCACTCCCCCTCCTTGGGCT	27	<lmxll>	ll	272	lm	0		

>ref-46297	TGTTGATCCACATAAACTCCCAAGTTT	8	<hkxhk>	hh	0	hk	207	kk	36
>ref-46297	TGTTGATCCACATAAACTCCCAAGTTT	12	<hkxhk>	hh	141	hk	86	kk	0
>ref-46297	TGTTGATCCACATAAACTCCCAAGTTT	15	<hkxhk>	hh	35	hk	208	kk	0
>ref-46297	TGTTGATCCACATAAACTCCCAAGTTT	26	<hkxhk>	hh	0	hk	239	kk	14
>ref-46308	GCCCTGCCAACACTTCCTCCCTGCTGA	9	<nnxnp>	nn	139	np	126		
>ref-46321	GCTTTATTTACCTCGCCTCCTCTTCCT	1	<lmxll>	ll	131	lm	127		
>ref-46321	GCTTTATTTACCTCGCCTCCTCTTCCT	3	<lmxll>	ll	132	lm	128		
>ref-46351	CTCCGTTCCACTCTCCCTCCTCCCTTA	1	<lmxll>	ll	129	lm	133		
>ref-46383	CTCTTCCCTACCTTTTCTCCTCTCACG	25	<lmxll>	ll	124	lm	120		
>ref-46389	ACTCAAACCACCCCACCTCCTACCTCG	7	<nnxnp>	nn	128	np	116		
>ref-46389	ACTCAAACCACCCCACCTCCTACCTCG	9	<nnxnp>	nn	129	np	116		
>ref-46406	TAGAAGCATACTGGGTCTCCGGGTCTG	6	<hkxhk>	hh	0	hk	273	kk	0
>ref-46406	TAGAAGCATACTGGGTCTCCGGGTCTG	13	<hkxhk>	hh	0	hk	273	kk	0
>ref-46409	GCGCCTGCAACAGCGTCTCCACGTCCT	16	<nnxnp>	nn	267	np	0		
>ref-46411	AGAGTCGCGACCGGCACTCCATCAGCG	15	<nnxnp>	nn	136	np	131		
>ref-46412	AAAACATCCACACTCTCTCCACACCTT	15	<nnxnp>	nn	134	np	127		
>ref-46416	CCTCTTCACACTCCATCTCCCATATTC	1	<nnxnp>	nn	99	np	143		
>ref-46444	AAGCCAAGCACGGCCCCTCCCCTTGAG	5	<nnxnp>	nn	137	np	131		
>ref-46472	GTGGAGCGCACCAGTTCTCCAAGTGCA	26	<nnxnp>	nn	273	np	0		
>ref-46477	CACCCAAACACACACGCTCCTTTACTT	5	<hkxhk>	hh	0	hk	273	kk	0
>ref-46490	ATGACGTTGACCGTGGCTCCCATGGCG	12	<hkxhk>	hh	55	hk	140	kk	55
>ref-46495	CCCTCCTTCACCCGCTCTCCTTTCCTT	1	<nnxnp>	nn	264	np	0		
>ref-46495	CCCTCCTTCACCCGCTCTCCTTTCCTT	14	<nnxnp>	nn	253	np	2		
>ref-46515	ATCCTTCTTACTTCTTCTCCTCCTCCC	13	<nnxnp>	nn	175	np	62		
>ref-46515	ATCCTTCTTACTTCTTCTCCTCCTCCC	16	<nnxnp>	nn	167	np	68		
>ref-46515	ATCCTTCTTACTTCTTCTCCTCCTCCC	27	<nnxnp>	nn	259	np	2		
>ref-46522	ACTACAGATACGTTTTCTCCTCGAATT	5	<lmxll>	ll	109	lm	130		
>ref-46529	CCTTACCCTACCCTTCCTCCCCCTTAC	15	<nnxnp>	nn	225	np	0		
>ref-46539	AGCTGCTCCACATCTCCTCCCACGAGT	1	<hkxhk>	hh	61	hk	139	kk	60
>ref-46559	CAGAGGAACACAGTGCCTCCATCAAGT	6	<hkxhk>	hh	73	hk	190	kk	0
>ref-46559	CAGAGGAACACAGTGCCTCCATCAAGT	15	<hkxhk>	hh	0	hk	32	kk	237
>ref-46559	CAGAGGAACACAGTGCCTCCATCAAGT	27	<hkxhk>	hh	0	hk	272	kk	1
>ref-46574	TCTTCGTACACCTTCCCTCCCCCTTCC	1	<lmxll>	ll	16	lm	246		
>ref-46574	TCTTCGTACACCTTCCCTCCCCCTTCC	6	<lmxll>	ll	11	lm	251		
>ref-46574	TCTTCGTACACCTTCCCTCCCCCTTCC	8	<lmxll>	ll	17	lm	246		
>ref-46574	TCTTCGTACACCTTCCCTCCCCCTTCC	9	<lmxll>	ll	134	lm	123		
>ref-46574	TCTTCGTACACCTTCCCTCCCCCTTCC	12	<lmxll>	ll	11	lm	252		
>ref-46583	CAGACTATCACTGCAACTCCTTTTATT	3	<nnxnp>	nn	117	np	147		

>ref-46583	CAGACTATCACTGCAACTCCTTTTATT	24	<lmxll>	ll	124	lm	135		
>ref-46599	GTCTATATCACCTCCTCTCCCTTCCCC	1	<nnxnp>	nn	262	np	1		
>ref-46599	GTCTATATCACCTCCTCTCCCTTCCCC	24	<nnxnp>	nn	262	np	1		
>ref-46599	GTCTATATCACCTCCTCTCCCTTCCCC	26	<lmxll>	ll	264	lm	0		
>ref-46615	TGAGGCAGGACGAGGACTCCAGCGACT	4	<hkxhk>	hh	0	hk	273	kk	0
>ref-46615	TGAGGCAGGACGAGGACTCCAGCGACT	6	<hkxhk>	hh	9	hk	261	kk	0
>ref-46636	CGAAATAACACTGACACTCCTACTGTA	13	<hkxhk>	hh	42	hk	143	kk	71
>ref-46648	CCTTCTACCACCTCTTCTCCCGTCTTT	8	<lmxll>	ll	112	lm	153		
>ref-46648	CCTTCTACCACCTCTTCTCCCGTCTTT	22	<lmxll>	ll	113	lm	152		
>ref-46648	CCTTCTACCACCTCTTCTCCCGTCTTT	25	<hkxhk>	hh	51	hk	134	kk	70
>ref-46653	GCTTCGGATACACCACCTCCGCTAGCA	25	<hkxhk>	hh	54	hk	126	kk	57
>ref-46679	CATACTGCAACATGAACTCCGGTCGCC	2	<lmxll>	ll	187	lm	63		
>ref-46716	TGGTGCAAAACAGGATCTCCAGCTACC	23	<lmxll>	ll	125	lm	144		
>ref-46728	TGAGACGCCACCTCAGCTCCATCCTCC	1	<lmxll>	ll	140	lm	125		
>ref-46731	AGGATCCTAACCCACGCTCCACAGACA	12	<lmxll>	ll	133	lm	132		
>ref-46731	AGGATCCTAACCCACGCTCCACAGACA	14	<lmxll>	ll	133	lm	132		
>ref-46743	ATCATCCCCACGTCTCCTCCCCACCCC	13	<nnxnp>	nn	131	np	136		
>ref-46779	TTCCCTTTTACTCTTTCTCCCATTCCC	1	<nnxnp>	nn	141	np	117		
>ref-46779	TTCCCTTTTACTCTTTCTCCCATTCCC	23	<nnxnp>	nn	142	np	117		
>ref-46789	AGGCTAGTGACGTTTGCTCCTCCCCAC	4	<lmxll>	ll	140	lm	113		
>ref-46789	AGGCTAGTGACGTTTGCTCCTCCCCAC	6	<lmxll>	ll	142	lm	112		
>ref-46789	AGGCTAGTGACGTTTGCTCCTCCCCAC	13	<hkxhk>	hh	0	hk	273	kk	0
>ref-46809	CCCTTTCTCACCTCCCCTCCTTTCCCC	5	<nnxnp>	nn	121	np	126		
>ref-46809	CCCTTTCTCACCTCCCCTCCTTTCCCC	21	<nnxnp>	nn	119	np	127		
>ref-46835	GAGGGTTGCACAGTGTCTCCCCAGAGG	5	<nnxnp>	nn	131	np	129		
>ref-46835	GAGGGTTGCACAGTGTCTCCCCAGAGG	25	<hkxhk>	hh	224	hk	38	kk	0
>ref-46835	GAGGGTTGCACAGTGTCTCCCCAGAGG	26	<nnxnp>	nn	104	np	135		
>ref-46851	CTCCTCATTACCACCTCTCCTTCTCCC	1	<hkxhk>	hh	0	hk	146	kk	101
>ref-46853	GTTCCTGAAACCACAGCTCCAAATACT	4	<hkxhk>	hh	0	hk	270	kk	2
>ref-46855	TTCCCACACACCCCTCCTCCTTCCTTT	23	<hkxhk>	hh	1	hk	269	kk	0
>ref-46855	TTCCCACACACCCCTCCTCCTTCCTTT	25	<hkxhk>	hh	0	hk	269	kk	1
>ref-46874	CATGACAACACTCCACCTCCATCCCCT	25	<nnxnp>	nn	140	np	122		
>ref-46881	GAGAAGCAAACAGTGTCTCCTGGAGCA	8	<lmxll>	ll	129	lm	137		
>ref-46904	ACTATCATCACCCTGTCTCCTTTATTA	7	<nnxnp>	nn	124	np	137		
>ref-46924	TACGCCCAAACTCCCCCTCCTACATCC	23	<lmxll>	ll	261	lm	0		
>ref-46924	TACGCCCAAACTCCCCCTCCTACATCC	27	<lmxll>	ll	253	lm	1		
>ref-46938	ACGGCGTGGACAGCCACTCCCATGAAG	6	<lmxll>	ll	22	lm	234		
>ref-46938	ACGGCGTGGACAGCCACTCCCATGAAG	23	<hkxhk>	hh	0	hk	246	kk	17

>ref-46940	CCCTCTCCTACCCTACCTCCCCCCTTT	23	<lmxll>	ll	2	lm	268		
>ref-46964	ATTTATGTGACACCTTCTCCTTCACCC	14	<lmxll>	ll	127	lm	139		
>ref-46966	AATAACAGAACAGTTCCTCCGTTCACA	3	<hkxhk>	hh	64	hk	158	kk	41
>ref-46972	TACGTTATTACGAGGTCTCCTTCGCCT	24	<lmxll>	ll	122	lm	139		
>ref-46972	TACGTTATTACGAGGTCTCCTTCGCCT	25	<lmxll>	ll	140	lm	120		
>ref-46973	CGCACTCTCACTCTCTCTCCTAATATT	2	<nnxnp>	nn	129	np	124		
>ref-46998	ACCACCCTCACCACCTCTCCACCACCC	1	<nnxnp>	nn	272	np	0		
>ref-47005	TAAACCCCTACTTCTCCTCCTCCTTCT	9	<nnxnp>	nn	127	np	140		
>ref-47054	CCCATCACAACATGACCTCCGCTCGAC	16	<lmxll>	ll	126	lm	140		
>ref-47061	TTAAAAATAACTCTCTCTCCCTCTCTC	1	<hkxhk>	hh	0	hk	222	kk	24
>ref-47061	TTAAAAATAACTCTCTCTCCCTCTCTC	2	<hkxhk>	hh	0	hk	262	kk	3
>ref-47061	TTAAAAATAACTCTCTCTCCCTCTCTC	5	<hkxhk>	hh	24	hk	221	kk	0
>ref-47061	TTAAAAATAACTCTCTCTCCCTCTCTC	14	<hkxhk>	hh	0	hk	249	kk	1
>ref-47061	TTAAAAATAACTCTCTCTCCCTCTCTC	24	<hkxhk>	hh	0	hk	56	kk	182
>ref-47076	CGGTCCACTACAACACCTCCCCCTGAA	3	<nnxnp>	nn	184	np	82		
>ref-47076	CGGTCCACTACAACACCTCCCCCTGAA	26	<nnxnp>	nn	267	np	3		
>ref-47085	TACCTTGTTACCCTGCCTCCTTGTCTG	24	<hkxhk>	hh	72	hk	147	kk	51
>ref-47093	GAATTAAGTACATAGCCTCCTTCGTCA	24	<lmxll>	ll	111	lm	131		
>ref-47097	CTCTCTCCAACACCAACTCCCTGCCTT	14	<lmxll>	ll	126	lm	135		
>ref-47113	GGGCAACACACACTTACTCCCGTATAT	2	<nnxnp>	nn	116	np	130		
>ref-47147	TATATATGCACACAGCCTCCCTCTCTG	2	<lmxll>	ll	80	lm	151		
>ref-47152	ACCACCAACACGCTCTCTCCCACAACA	12	<nnxnp>	nn	117	np	136		
>ref-47157	CATCAAGTTACCATACCTCCAACTTAC	4	<nnxnp>	nn	114	np	152		
>ref-47157	CATCAAGTTACCATACCTCCAACTTAC	5	<hkxhk>	hh	73	hk	191	kk	4
>ref-47157	CATCAAGTTACCATACCTCCAACTTAC	9	<hkxhk>	hh	3	hk	191	kk	73
>ref-47157	CATCAAGTTACCATACCTCCAACTTAC	13	<nnxnp>	nn	102	np	137		
>ref-47175	CTGTCCTCGACCCCCACTCCCTGTCCT	9	<nnxnp>	nn	16	np	254		
>ref-47175	CTGTCCTCGACCCCCACTCCCTGTCCT	14	<nnxnp>	nn	39	np	222		
>ref-47182	TTTCTCTTTACTTTCCCTCCTCTCTGT	4	<lmxll>	ll	144	lm	103		
>ref-47196	CTCTGACTGACTCTGTCTCCACCTATA	25	<lmxll>	ll	4	lm	227		
>ref-47196	CTCTGACTGACTCTGTCTCCACCTATA	26	<lmxll>	ll	4	lm	226		
>ref-47198	TATCGCTACACCATCACTCCCTTCTTG	15	<nnxnp>	nn	129	np	133		
>ref-47199	TCTCCCCCTACTAATGCTCCTTGTTGA	23	<lmxll>	ll	140	lm	107		
>ref-47236	TTATTCCTCACTCACCCTCCACGCTTC	3	<lmxll>	ll	256	lm	0		
>ref-47236	TTATTCCTCACTCACCCTCCACGCTTC	6	<lmxll>	ll	256	lm	0		
>ref-47247	CATGAAGTCACACTCACTCCTTTCCCC	1	<lmxll>	ll	107	lm	157		
>ref-47255	CTAATTCAAACTCATCCTCCTTCCTTC	13	<nnxnp>	nn	119	np	131		
>ref-47256	CAAATCCCAACTTGATCTCCGATGATC	12	<hkxhk>	hh	0	hk	271	kk	0

>ref-47263	GATTACAGAACAGCATCTCCGAGGACA	16	<lmxll>	ll	110	lm	134		
>ref-47263	GATTACAGAACAGCATCTCCGAGGACA	25	<lmxll>	ll	109	lm	135		
>ref-47264	GCAATCATTACTCAACCTCCTCATCTC	5	<hkxhk>	hh	56	hk	159	kk	48
>ref-47267	CAGAAAGTAACTTTGTCTCCTTTAGAC	15	<lmxll>	ll	121	lm	109		
>ref-47278	CCCTACCATACCTTCTCTCCTTTACCT	16	<nnxnp>	nn	120	np	127		
>ref-47283	ATCCTCTTTACTCTCTCTCCGCTTGTC	21	<nnxnp>	nn	134	np	136		
>ref-47312	CCCTCCACCACTTCCCCTCCTCCCCAC	5	<nnxnp>	nn	75	np	162		
>ref-47319	CCTCCCAACACATCCTCTCCCTCCCCC	9	<lmxll>	ll	151	lm	105		
>ref-47325	TTTCACCTCACGGGTTCTCCTCCTACT	8	<hkxhk>	hh	53	hk	129	kk	62
>ref-47346	CACCCTTTTACCCATCCTCCCTCTTTC	9	<hkxhk>	hh	0	hk	260	kk	7
>ref-47348	GACAATAACACAATCGCTCCCCATATG	1	<nnxnp>	nn	132	np	112		
>ref-47355	CACACTCTTACATGCACTCCCTCGCAG	25	<nnxnp>	nn	130	np	134		
>ref-47356	AAGGCTGGGACACTAACTCCACTTCTT	1	<lmxll>	ll	5	lm	266		
>ref-47356	AAGGCTGGGACACTAACTCCACTTCTT	4	<lmxll>	ll	148	lm	124		
>ref-47356	AAGGCTGGGACACTAACTCCACTTCTT	5	<lmxll>	ll	5	lm	266		
>ref-47357	TCCCCGATCACTCTCTCTCCTTCTCCC	1	<lmxll>	ll	134	lm	121		
>ref-47357	TCCCCGATCACTCTCTCTCCTTCTCCC	6	<lmxll>	ll	125	lm	123		
>ref-47405	GCTGCTTTTACTTCTGCTCCTTTTCTG	7	<nnxnp>	nn	128	np	120		
>ref-47493	TTCCTCCCTACATACCCTCCCTCTCCT	7	<hkxhk>	hh	3	hk	267	kk	0
>ref-47493	TTCCTCCCTACATACCCTCCCTCTCCT	9	<hkxhk>	hh	0	hk	267	kk	3
>ref-47499	GTCATTCTCACACTTTCTCCCTTTCTC	4	<nnxnp>	nn	265	np	1		
>ref-47499	GTCATTCTCACACTTTCTCCCTTTCTC	12	<nnxnp>	nn	255	np	4		
>ref-47505	GAATACAAGACGATTTCTCCCTCTTGT	7	<lmxll>	ll	120	lm	147		
>ref-47518	CGAAGCAAAACCCGCCCTCCTAATAAT	12	<nnxnp>	nn	123	np	116		
>ref-47519	GAACGTAAAACGGGCACTCCCACACAC	1	<nnxnp>	nn	134	np	134		
>ref-47519	GAACGTAAAACGGGCACTCCCACACAC	16	<lmxll>	ll	135	lm	133		
>ref-47539	CTTGCGCCAACCCAGCCTCCTGGCATA	4	<hkxhk>	hh	45	hk	152	kk	61
>ref-47549	CAGCCCATGACCCACTCTCCTTCGTTC	3	<hkxhk>	hh	0	hk	273	kk	0
>ref-47549	CAGCCCATGACCCACTCTCCTTCGTTC	14	<hkxhk>	hh	2	hk	271	kk	0
>ref-47561	CACGGCCACACCCTTCCTCCCCTCCTT	13	<nnxnp>	nn	124	np	144		
>ref-47564	ACCACCCGCACCCCCTCTCCTCCTCGA	6	<lmxll>	ll	142	lm	121		
>ref-47595	TTTCTCTCAACTCTTTCTCCCTCCTTC	24	<hkxhk>	hh	0	hk	273	kk	0
>ref-47596	TGAGCTTGGACGTCGACTCCTTGTTCA	2	<nnxnp>	nn	189	np	75		
>ref-47596	TGAGCTTGGACGTCGACTCCTTGTTCA	5	<hkxhk>	hh	0	hk	227	kk	40
>ref-47596	TGAGCTTGGACGTCGACTCCTTGTTCA	21	<hkxhk>	hh	0	hk	273	kk	0
>ref-47623	CCACCTCCAACTCCTCCTCCAATAGCA	6	<hkxhk>	hh	0	hk	273	kk	0
>ref-47623	CCACCTCCAACTCCTCCTCCAATAGCA	8	<nnxnp>	nn	144	np	120		
>ref-47623	CCACCTCCAACTCCTCCTCCAATAGCA	9	<hkxhk>	hh	0	hk	273	kk	0

>ref-47625	CTGGTATGAACTGCACCTCCTTTGGCT	2	<nnxnp>	nn	129	np	140		
>ref-47626	ACGTCCATGACCCAATCTCCTCCTCCT	26	<nnxnp>	nn	134	np	116		
>ref-47632	CACACACCAACCCTTCCTCCCTCCCCC	4	<nnxnp>	nn	28	np	220		
>ref-47633	CGTCCTTTTACTTCTCCTCCTCCTTGT	3	<lmxll>	ll	139	lm	125		
>ref-47636	TGTTTTATCACTGATACTCCTTCCGAC	7	<hkxhk>	hh	54	hk	111	kk	55
>ref-47661	ATTATGCCCACCTCCTCTCCCCCCTTC	7	<nnxnp>	nn	269	np	0		
>ref-47679	TCAACCTTGACATACTCTCCTCCTTCT	9	<hkxhk>	hh	0	hk	200	kk	65
>ref-47679	TCAACCTTGACATACTCTCCTCCTTCT	26	<hkxhk>	hh	0	hk	199	kk	66
>ref-47719	TGCAGCCACACTGTCGCTCCACAGCCT	23	<lmxll>	ll	130	lm	136		
>ref-47721	GATACATATACTCAGTCTCCCTCAGTA	3	<lmxll>	ll	133	lm	134		
>ref-47747	CTTAGCCTCACCTCCCCTCCCTCACTC	2	<lmxll>	ll	266	lm	0		
>ref-47747	CTTAGCCTCACCTCCCCTCCCTCACTC	24	<lmxll>	ll	260	lm	0		
>ref-47761	CCTCGCCGCACGTGCACTCCTGCGCGT	2	<lmxll>	ll	123	lm	142		
>ref-47763	GCCCTTGCAACCGTTTCTCCTTTGCTG	16	<lmxll>	ll	127	lm	135		
>ref-47771	CGGCGCGCCACCCTGACTCCCACCTCC	1	<nnxnp>	nn	261	np	1		
>ref-47795	CCTCCACGTACCCCTCCTCCCTCCACG	1	<lmxll>	ll	18	lm	225		
>ref-47795	CCTCCACGTACCCCTCCTCCCTCCACG	6	<lmxll>	ll	16	lm	226		
>ref-47796	CCTCCTCTTACTCATCCTCCCCTCTCT	14	<nnxnp>	nn	23	np	220		
>ref-47796	CCTCCTCTTACTCATCCTCCCCTCTCT	15	<nnxnp>	nn	124	np	108		
>ref-47796	CCTCCTCTTACTCATCCTCCCCTCTCT	24	<nnxnp>	nn	211	np	31		
>ref-47805	TGACGGGCGACAACTTCTCCTTGAACG	15	<lmxll>	ll	112	lm	129		
>ref-47807	GTGAAATCGACTGTTCCTCCTTTTGTC	3	<lmxll>	ll	140	lm	124		
>ref-47810	CCCACTCCAACCTCTTCTCCCCTCCTA	21	<lmxll>	ll	107	lm	139		
>ref-47836	TCAGATGTAACTTTCTCTCCTCAGTAA	15	<lmxll>	ll	127	lm	124		
>ref-47860	CTCTTGTCCACTTTTTCTCCTTTTCCT	24	<nnxnp>	nn	114	np	115		
>ref-47868	AAGTCTGATACATCAACTCCAATAAGG	5	<hkxhk>	hh	4	hk	268	kk	0
>ref-47868	AAGTCTGATACATCAACTCCAATAAGG	15	<lmxll>	ll	8	lm	259		
>ref-47868	AAGTCTGATACATCAACTCCAATAAGG	22	<hkxhk>	hh	37	hk	216	kk	0
>ref-47868	AAGTCTGATACATCAACTCCAATAAGG	23	<lmxll>	ll	14	lm	253		
>ref-47869	TCACCTGTCACCATTACTCCTTATCAG	15	<lmxll>	ll	100	lm	136		
>ref-47895	CATTACACCACCTGCTCTCCCTTATCA	1	<nnxnp>	nn	130	np	135		
>ref-47895	CATTACACCACCTGCTCTCCCTTATCA	2	<nnxnp>	nn	130	np	135		
>ref-47896	ATTTACACCACAGTAACTCCAATGCCA	1	<nnxnp>	nn	271	np	0		
>ref-47920	TCCATTGATACATCTCCTCCTTTGTCC	9	<lmxll>	ll	259	lm	0		
>ref-47937	AACACCAACACTCACCCTCCAACACAT	26	<nnxnp>	nn	120	np	120		
>ref-47937	AACACCAACACTCACCCTCCAACACAT	27	<nnxnp>	nn	120	np	119		
>ref-47938	TCTCCCTCCACTCTTTCTCCTCTCCTA	27	<lmxll>	ll	147	lm	107		
>ref-47941	TTCCCATACACACCCTCTCCCTTACAC	26	<nnxnp>	nn	135	np	136		

>ref-47945	CCTTCTTCTACTACTTCTCCTGTTGCT	26	<lmxll>	ll	113	lm	140		
>ref-47950	ATCCCTTGCACTCGGCCTCCTCCGATC	13	<nnxnp>	nn	132	np	138		
>ref-47974	CATAAGAGAACACTTCCTCCAATACTC	15	<hkxhk>	hh	1	hk	261	kk	11
>ref-47974	CATAAGAGAACACTTCCTCCAATACTC	23	<hkxhk>	hh	1	hk	261	kk	11
>ref-47986	TGCATTTCCACACTTCCTCCGTTTCAT	16	<lmxll>	ll	147	lm	117		
>ref-47989	GAAGAAAACACTGCTTCTCCAACGGGG	5	<nnxnp>	nn	162	np	103		
>ref-47989	GAAGAAAACACTGCTTCTCCAACGGGG	6	<lmxll>	ll	144	lm	116		
>ref-47989	GAAGAAAACACTGCTTCTCCAACGGGG	8	<nnxnp>	nn	139	np	128		
>ref-47995	AACTCTCTCACCCTTCCTCCTCGACAT	26	<nnxnp>	nn	134	np	135		
>ref-48035	CTCGTCTTCACTATCACTCCCTCTTCA	12	<nnxnp>	nn	141	np	121		
>ref-48035	CTCGTCTTCACTATCACTCCCTCTTCA	26	<lmxll>	ll	143	lm	124		
>ref-48040	TTACCTTTCACGATTACTCCTGTCACC	25	<lmxll>	ll	116	lm	139		
>ref-48057	ATTCGTTAAACACAGGCTCCTCTTGCT	15	<nnxnp>	nn	125	np	120		
>ref-48061	ATCCCCAACACTCCACCTCCTCTTTCC	2	<hkxhk>	hh	0	hk	218	kk	43
>ref-48061	ATCCCCAACACTCCACCTCCTCTTTCC	3	<hkxhk>	hh	0	hk	217	kk	44
>ref-48075	TGAGGGGTGACACTTCCTCCTTGCCAC	2	<lmxll>	ll	123	lm	140		
>ref-48075	TGAGGGGTGACACTTCCTCCTTGCCAC	26	<lmxll>	ll	139	lm	128		
>ref-48076	ATTCCTCCCACACCTGCTCCTTAGTCC	23	<lmxll>	ll	108	lm	147		
>ref-48096	CTTCCCTAAACTATCTCTCCTTTACAT	23	<nnxnp>	nn	103	np	132		
>ref-48103	ACCTCTACCACCACCTCTCCTTCCTCT	14	<nnxnp>	nn	125	np	138		
>ref-48108	AGCCTTACAACTCGATCTCCCTCACAC	6	<nnxnp>	nn	120	np	135		
>ref-48122	TCCATGTTGACCGCACCTCCGCCCATT	1	<nnxnp>	nn	273	np	0		
>ref-48122	TCCATGTTGACCGCACCTCCGCCCATT	2	<nnxnp>	nn	273	np	0		
>ref-48131	CTTCTCTTCACGATTCCTCCTTCCCCT	24	<lmxll>	ll	131	lm	141		
>ref-48157	TATTCCCCAACAGCCTCTCCCCCTCCA	27	<lmxll>	ll	120	lm	142		
>ref-48166	CTCCCCTACACCCATCCTCCTCCCCTT	4	<hkxhk>	hh	1	hk	262	kk	0
>ref-48176	AGATTTCTCACTCTTTCTCCCAGTTAC	1	<lmxll>	ll	135	lm	107		
>ref-48183	CCCCATGCGACTGCCTCTCCCACAGTC	1	<hkxhk>	hh	0	hk	273	kk	0
>ref-48202	GCTCCTCCAACCCCCTCTCCTACGCCC	6	<hkxhk>	hh	44	hk	125	kk	59
>ref-48214	GAACACAGTACACGCGCTCCTCAGCAC	3	<lmxll>	ll	124	lm	138		
>ref-48243	ACTCTCACTACTTTCTCTCCTCTTCTC	21	<nnxnp>	nn	120	np	145		
>ref-48247	ATACTAGCTACACAGTCTCCTCACTTT	7	<hkxhk>	hh	55	hk	149	kk	50
>ref-48251	TCCACGGGTACCCCAACTCCTTCCCCT	15	<lmxll>	ll	112	lm	149		
>ref-48254	ACATAACACACGCAGCCTCCTCTCGTT	7	<nnxnp>	nn	115	np	156		
>ref-48274	CTTTTTGTCACGCACACTCCTGATGAT	3	<nnxnp>	nn	116	np	133		
>ref-48282	CATAATCCCACAACCACTCCCACAACC	5	<lmxll>	ll	133	lm	127		
>ref-48282	CATAATCCCACAACCACTCCCACAACC	7	<lmxll>	ll	133	lm	127		
>ref-48288	TTATTCCTTACTTGCTCTCCCTTCTCA	14	<nnxnp>	nn	132	np	134		

>ref-48294	TGTAAGGAAACCAGGTCTCCTTATGCA	2	<nnxnp>	nn	151	np	120		
>ref-48344	TTCCTTCCCACCCTTTCTCCTTCTTTA	26	<hkxhk>	hh	0	hk	273	kk	0
>ref-48347	CATACTCATACTTACACTCCCTTATAC	13	<hkxhk>	hh	60	hk	143	kk	57
>ref-48361	CTCTTCCCGACGTCCACTCCCTTCTTC	1	<nnxnp>	nn	269	np	0		
>ref-48364	CATCAGTCTACCATTCCTCCAACATAC	22	<hkxhk>	hh	0	hk	251	kk	7
>ref-48364	CATCAGTCTACCATTCCTCCAACATAC	27	<hkxhk>	hh	0	hk	251	kk	7
>ref-48367	CGCACGCCCACATGCACTCCAAAACCC	16	<lmxll>	ll	236	lm	0		
>ref-48370	AACAAACACACACTCACTCCCAATTAA	14	<lmxll>	ll	121	lm	126		
>ref-48370	AACAAACACACACTCACTCCCAATTAA	22	<lmxll>	ll	121	lm	126		
>ref-48370	AACAAACACACACTCACTCCCAATTAA	26	<lmxll>	ll	56	lm	193		
>ref-48386	TTAGGACATACGGCCCCTCCTCTCCCC	3	<lmxll>	ll	141	lm	130		
>ref-48387	TATCCTATCACCCTCCCTCCCTTCCAC	5	<lmxll>	ll	262	lm	0		
>ref-48387	TATCCTATCACCCTCCCTCCCTTCCAC	22	<lmxll>	ll	265	lm	0		
>ref-48401	TTGTCACTCACTCCGTCTCCCAACCCC	14	<hkxhk>	hh	50	hk	132	kk	86
>ref-48413	CACATTTAAACAAGCACTCCTACAAAT	7	<hkxhk>	hh	0	hk	148	kk	112
>ref-48436	CCGCTACCCACTTTCTCTCCTTCCTCT	3	<lmxll>	ll	1	lm	268		
>ref-48468	ATAGACGAGACAGTCCCTCCCTCATTT	2	<lmxll>	ll	138	lm	127		
>ref-48468	ATAGACGAGACAGTCCCTCCCTCATTT	13	<lmxll>	ll	138	lm	127		
>ref-48476	TACCAGTCCACCCGGCCTCCTCTTCTT	15	<nnxnp>	nn	134	np	134		
>ref-48489	GTCCCTTACACGTATCCTCCTCAGGTA	26	<nnxnp>	nn	133	np	134		
>ref-48509	TCTCCTTCAACCCCTCCTCCCCGCCCC	16	<lmxll>	ll	127	lm	139		
>ref-48521	TACACCCTCACCCCTCCTCCCCACCCC	24	<lmxll>	ll	106	lm	144		
>ref-48525	CTGGGACAAACAAACACTCCTATACTC	3	<lmxll>	ll	126	lm	112		
>ref-48525	CTGGGACAAACAAACACTCCTATACTC	9	<lmxll>	ll	125	lm	112		
>ref-48528	TTATTCAAGACAGCTTCTCCCTCCCTC	26	<nnxnp>	nn	137	np	127		
>ref-48578	GCCCATAAAACACGTTCTCCCTCCCCA	13	<hkxhk>	hh	0	hk	250	kk	10
>ref-48606	TTGCTTCCTACCATTCCTCCTTTCTCT	2	<hkxhk>	hh	60	hk	136	kk	70
>ref-48615	AGCTCTACCACCCGTCCTCCACTCGGG	25	<hkxhk>	hh	0	hk	259	kk	10
>ref-48617	ACTAAACCAACTGGCCCTCCGGTCTCA	1	<lmxll>	ll	111	lm	122		
>ref-48630	GCTCTCAAAACTAGCCCTCCCTATCAA	14	<nnxnp>	nn	118	np	124		
>ref-48630	GCTCTCAAAACTAGCCCTCCCTATCAA	16	<nnxnp>	nn	117	np	125		
>ref-48708	CCTCCTGCCACTCCTCCTCCCTCCTGT	15	<hkxhk>	hh	0	hk	217	kk	25
>ref-48708	CCTCCTGCCACTCCTCCTCCCTCCTGT	22	<hkxhk>	hh	0	hk	217	kk	25
>ref-48730	GCCATCATCACTCATACTCCGGGGACG	3	<nnxnp>	nn	254	np	0		
>ref-48730	GCCATCATCACTCATACTCCGGGGACG	25	<lmxll>	ll	120	lm	115		
>ref-48740	GCTGCAAGCACTCCTCCTCCTTCTTCT	25	<lmxll>	ll	109	lm	148		
>ref-48741	CCTGAGGTAACTTAGGCTCCCTGCCAA	3	<nnxnp>	nn	147	np	111		
>ref-48751	TGGTATTTAACACTGACTCCTGTACTG	24	<hkxhk>	hh	57	hk	145	kk	61

>ref-48764	TCCGTTTCTACCCCTTCTCCGTTTCTA	13	<lmxll>	ll	260	lm	0		
>ref-48776	ACCCCTCCCACCTTACCTCCCCCCTCC	5	<lmxll>	ll	11	lm	261		
>ref-48776	ACCCCTCCCACCTTACCTCCCCCCTCC	15	<lmxll>	ll	10	lm	257		
>ref-48796	CCTATCCTTACTCCTTCTCCCTACTCC	24	<hkxhk>	hh	36	hk	217	kk	0
>ref-48810	AGGAAAAGCACAAATCCTCCTTGTACG	23	<nnxnp>	nn	127	np	105		
>ref-48819	TCTCGCTCGACTCCTCCTCCTTTCCCT	27	<nnxnp>	nn	142	np	118		
>ref-48822	CCATTCTTTACTATCTCTCCTTCCTCC	6	<hkxhk>	hh	5	hk	234	kk	1
>ref-48822	CCATTCTTTACTATCTCTCCTTCCTCC	9	<hkxhk>	hh	1	hk	235	kk	5
>ref-48829	TAATTGGCAACTCCTTCTCCACAATGT	1	<lmxll>	ll	116	lm	132		
>ref-48850	GGCGTTCGGACCGTTCCTCCTTATCGA	1	<nnxnp>	nn	137	np	131		
>ref-48851	CAAAGACAAACAAACTCTCCGGCCCAA	1	<nnxnp>	nn	146	np	119		
>ref-48854	TCCACACTCACTCACTCTCCCACAAGA	2	<nnxnp>	nn	260	np	1		
>ref-48854	TCCACACTCACTCACTCTCCCACAAGA	6	<nnxnp>	nn	264	np	1		
>ref-48857	CACACTTACACTCGCTCTCCTCATCAC	6	<nnxnp>	nn	159	np	112		
>ref-48858	GTTTCCCTTACCTCCTCTCCTTACTCT	4	<nnxnp>	nn	122	np	133		
>ref-48867	ATCCTCCCTACCCTTTCTCCCCTGATT	4	<lmxll>	ll	131	lm	105		
>ref-48871	AGCTAACTAACCCCCCCTCCCCCCTCT	21	<nnxnp>	nn	117	np	136		
>ref-48871	AGCTAACTAACCCCCCCTCCCCCCTCT	23	<nnxnp>	nn	120	np	134		
>ref-48871	AGCTAACTAACCCCCCCTCCCCCCTCT	27	<nnxnp>	nn	114	np	137		
>ref-48907	AGGTCGGGAACTTTATCTCCCGGACAG	24	<nnxnp>	nn	121	np	133		
>ref-48907	AGGTCGGGAACTTTATCTCCCGGACAG	25	<nnxnp>	nn	121	np	134		
>ref-48955	AACATCCACACAGGGTCTCCAGGCTCC	1	<nnxnp>	nn	129	np	140		
>ref-48957	ACTAGGATCACACTCGCTCCATAGCTA	26	<hkxhk>	hh	47	hk	160	kk	56
>ref-48959	GAGGAAAGAACTAGACCTCCCTTCAAT	1	<hkxhk>	hh	64	hk	200	kk	0
>ref-48959	GAGGAAAGAACTAGACCTCCCTTCAAT	6	<nnxnp>	nn	152	np	110		
>ref-48959	GAGGAAAGAACTAGACCTCCCTTCAAT	23	<hkxhk>	hh	0	hk	273	kk	0
>ref-48965	AGCAACACCACGAAGGCTCCTCCTCGA	13	<lmxll>	ll	200	lm	46		
>ref-49000	TGGCCAGCTACCTCGGCTCCGTGCACA	1	<lmxll>	ll	136	lm	123		
>ref-49031	CGACCCATCACCTTCTCTCCAGCCAGC	15	<hkxhk>	hh	1	hk	271	kk	0
>ref-49039	CCTCCGTCGACGAGTCCTCCGTCGACG	12	<lmxll>	ll	251	lm	12		
>ref-49042	TTATAAACCACGCCCCCTCCTATACCC	6	<nnxnp>	nn	85	np	147		
>ref-49053	TGAAAACCCACCGCTTCTCCCCTCTAA	23	<hkxhk>	hh	50	hk	117	kk	55
>ref-49086	TCTCTCCCAACCTCCTCTCCGTCTCCT	9	<nnxnp>	nn	115	np	142		
>ref-49087	ACCCCCTTTACCCCTCCTCCTCTCTCC	1	<nnxnp>	nn	209	np	32		
>ref-49087	ACCCCCTTTACCCCTCCTCCTCTCTCC	3	<lmxll>	ll	26	lm	208		
>ref-49087	ACCCCCTTTACCCCTCCTCCTCTCTCC	13	<lmxll>	ll	88	lm	160		
>ref-49087	ACCCCCTTTACCCCTCCTCCTCTCTCC	21	<lmxll>	ll	5	lm	221		
>ref-49087	ACCCCCTTTACCCCTCCTCCTCTCTCC	23	<nnxnp>	nn	206	np	33		

>ref-49095	CTTTTTCCTACCTTCTCTCCATCCCTT	21	<hkxhk>	hh	2	hk	255	kk	3
>ref-49095	CTTTTTCCTACCTTCTCTCCATCCCTT	26	<hkxhk>	hh	3	hk	256	kk	3
>ref-49115	CATCCTCATACTTCTCCTCCTTTCTCC	3	<lmxll>	ll	135	lm	109		
>ref-49124	CGGAAGGACACGGCCTCTCCCACGGCG	24	<lmxll>	ll	125	lm	144		
>ref-49124	CGGAAGGACACGGCCTCTCCCACGGCG	27	<lmxll>	ll	124	lm	144		
>ref-49152	CAAGGTGTTACTTTTTCTCCCAGTGAT	2	<nnxnp>	nn	13	np	222		
>ref-49186	CTTATATTAACAGTCACTCCCAACATC	6	<lmxll>	ll	136	lm	107		
>ref-49191	CTTTCTATCACATTTTCTCCCTCTTCT	1	<hkxhk>	hh	21	hk	235	kk	1
>ref-49191	CTTTCTATCACATTTTCTCCCTCTTCT	15	<hkxhk>	hh	1	hk	234	kk	21
>ref-49223	CCTATCCGCACTATATCTCCCACAATT	22	<nnxnp>	nn	144	np	114		
>ref-49230	AGAACCAACACCAGACCTCCCAACAGT	1	<hkxhk>	hh	1	hk	268	kk	0
>ref-49230	AGAACCAACACCAGACCTCCCAACAGT	16	<hkxhk>	hh	33	hk	219	kk	0
>ref-49238	CATCCATATACCCTTCCTCCTTCTCCA	21	<nnxnp>	nn	125	np	142		
>ref-49238	CATCCATATACCCTTCCTCCTTCTCCA	23	<nnxnp>	nn	125	np	142		
>ref-49241	ATTTTCATCACTGCTCCTCCTCCTCTT	1	<lmxll>	ll	140	lm	118		
>ref-49245	TCCATCTCTACGTCTCCTCCTTCCTTT	21	<hkxhk>	hh	52	hk	151	kk	65
>ref-49248	TCCTCTTCCACTACGTCTCCACCTCTC	7	<lmxll>	ll	129	lm	129		
>ref-49257	CCAACACACACACAACCTCCCCAACAC	27	<hkxhk>	hh	17	hk	248	kk	0
>ref-49258	CCTTCCTCTACCCCACCTCCCCACTCT	26	<nnxnp>	nn	114	np	144		
>ref-49289	TTGCCTAAGACTCTATCTCCCTTACCT	3	<nnxnp>	nn	126	np	127		
>ref-49298	ATAAAGTGAACGCGTTCTCCCTTCTCC	21	<hkxhk>	hh	6	hk	262	kk	0
>ref-49298	ATAAAGTGAACGCGTTCTCCCTTCTCC	22	<hkxhk>	hh	0	hk	262	kk	6
>ref-49305	GATCAGATGACTCCATCTCCTCTCGTT	25	<lmxll>	ll	129	lm	121		
>ref-49329	CCTTCCGCTACCCATTCTCCCTTTCCC	1	<nnxnp>	nn	130	np	129		
>ref-49385	AATATCCCCACATCTTCTCCTTCTCCA	13	<lmxll>	ll	141	lm	120		
>ref-49385	AATATCCCCACATCTTCTCCTTCTCCA	26	<lmxll>	ll	140	lm	120		
>ref-49388	CCGCGCGTTACGGTCCCTCCCAACCCC	13	<nnxnp>	nn	134	np	132		
>ref-49398	TCACGTGCAACGACGGCTCCTGCATCG	5	<lmxll>	ll	161	lm	104		
>ref-49398	TCACGTGCAACGACGGCTCCTGCATCG	24	<lmxll>	ll	161	lm	104		
>ref-49425	TATGCCATCACGATCACTCCACGCCCA	2	<hkxhk>	hh	65	hk	137	kk	65
>ref-49425	TATGCCATCACGATCACTCCACGCCCA	8	<hkxhk>	hh	64	hk	137	kk	65
>ref-49453	TTCTCGCTTACTCACACTCCTCATATA	4	<lmxll>	ll	120	lm	123		
>ref-49453	TTCTCGCTTACTCACACTCCTCATATA	16	<lmxll>	ll	119	lm	123		
>ref-49458	TCTCTCCTTACTCCATCTCCCTTCCCC	6	<lmxll>	ll	137	lm	126		
>ref-49485	ACCTCCTCCACCTTTTCTCCCCAACAT	23	<lmxll>	ll	9	lm	242		
>ref-49495	TCCTTATCTACCCTCTCTCCACCCTTC	15	<hkxhk>	hh	63	hk	132	kk	56
>ref-49499	AAGAGAGATACGGTGGCTCCCTCTGTC	6	<lmxll>	ll	114	lm	146		
>ref-49499	AAGAGAGATACGGTGGCTCCCTCTGTC	16	<nnxnp>	nn	268	np	1		

>ref-49513	CCCCAGCCTACCTTGTCTCCCCCTTCT	4	<lmxll>	ll	143	lm	117		
>ref-49536	CACTAGGCAACCCTTCCTCCCGACCAA	25	<lmxll>	ll	125	lm	109		
>ref-49544	CTGGCCTCTACCACTCCTCCATCCCCT	1	<lmxll>	ll	116	lm	149		
>ref-49606	TCCCTACCCACTTACCCTCCCTCACTA	13	<lmxll>	ll	127	lm	125		
>ref-49606	TCCCTACCCACTTACCCTCCCTCACTA	22	<nnxnp>	nn	124	np	122		
>ref-49606	TCCCTACCCACTTACCCTCCCTCACTA	23	<lmxll>	ll	127	lm	125		
>ref-49606	TCCCTACCCACTTACCCTCCCTCACTA	24	<nnxnp>	nn	121	np	122		
>ref-49612	ACAGAATAGACAGTAACTCCGGAGCAA	8	<hkxhk>	hh	52	hk	141	kk	66
>ref-49615	TTCCTTCTCACTCTCTCTCCTGACGAG	2	<nnxnp>	nn	111	np	117		
>ref-49615	TTCCTTCTCACTCTCTCTCCTGACGAG	25	<nnxnp>	nn	112	np	117		
>ref-49625	ATTTGATATACTGTTCCTCCTTTTACA	9	<nnxnp>	nn	27	np	209		
>ref-49625	ATTTGATATACTGTTCCTCCTTTTACA	12	<lmxll>	ll	13	lm	240		
>ref-49635	TTCTCCCCTACGTCTCCTCCATTCTCT	13	<nnxnp>	nn	123	np	147		
>ref-49671	CCTCCGCCCACTCCGCCTCCCAAGCCT	6	<nnxnp>	nn	271	np	0		
>ref-49674	CCTTTCCATACCTAGCCTCCACGCAGA	2	<nnxnp>	nn	122	np	134		
>ref-49674	CCTTTCCATACCTAGCCTCCACGCAGA	13	<hkxhk>	hh	0	hk	263	kk	1
>ref-49686	TTCATGTCTACTCCCCCTCCTCCTCTT	5	<hkxhk>	hh	50	hk	143	kk	68
>ref-49696	CCAGTCCTCACCATGCCTCCTTTCAAT	7	<lmxll>	ll	136	lm	127		
>ref-49709	GGGAGTGGCACTCGTCCTCCTCGAGCA	26	<lmxll>	ll	267	lm	4		
>ref-49709	GGGAGTGGCACTCGTCCTCCTCGAGCA	27	<lmxll>	ll	268	lm	5		
>ref-49712	TGTATACACACCGTTGCTCCAGCAGGA	2	<lmxll>	ll	136	lm	117		
>ref-49719	ATCTGTATCACAGTTTCTCCCTTTCTG	13	<nnxnp>	nn	138	np	128		
>ref-49726	CAATTCAACACCATCACTCCTTTAAAG	3	<hkxhk>	hh	49	hk	139	kk	72
>ref-49746	CTGTCTATTACACTTTCTCCCTCCCCT	14	<lmxll>	ll	114	lm	145		
>ref-49807	CGTGTAAACACGCACACTCCCTGACAC	24	<lmxll>	ll	151	lm	114		
>ref-49825	CACACCCACACTTACACTCCCTTCCTT	5	<hkxhk>	hh	77	hk	191	kk	0
>ref-49825	CACACCCACACTTACACTCCCTTCCTT	27	<hkxhk>	hh	1	hk	271	kk	1
>ref-49857	TGAAGTTTAACATTTTCTCCATCAACT	1	<hkxhk>	hh	0	hk	75	kk	176
>ref-49857	TGAAGTTTAACATTTTCTCCATCAACT	2	<hkxhk>	hh	0	hk	76	kk	178
>ref-49857	TGAAGTTTAACATTTTCTCCATCAACT	21	<nnxnp>	nn	200	np	57		
>ref-49857	TGAAGTTTAACATTTTCTCCATCAACT	25	<hkxhk>	hh	4	hk	269	kk	0
>ref-49864	TGTATGTTTACGAGTTCTCCATACTCT	6	<lmxll>	ll	0	lm	253		
>ref-49864	TGTATGTTTACGAGTTCTCCATACTCT	26	<lmxll>	ll	0	lm	253		
>ref-49888	TTTCGCTACACCCTTCCTCCACCCTCC	16	<nnxnp>	nn	259	np	0		
>ref-49906	ATCTCCATCACAGCTTCTCCTCCATCA	1	<hkxhk>	hh	19	hk	244	kk	0
>ref-49908	CATACACATACACTCACTCCCTCTTTC	8	<lmxll>	ll	100	lm	158		
>ref-49908	CATACACATACACTCACTCCCTCTTTC	24	<lmxll>	ll	99	lm	157		
>ref-49908	CATACACATACACTCACTCCCTCTTTC	26	<lmxll>	ll	142	lm	112		

>ref-49908	CATACACATACACTCACTCCCTCTTTC	27	<lmxll>	ll	141	lm	113		
>ref-49909	CAGAGCGCCACAAGACCTCCGCCTCCC	13	<lmxll>	ll	136	lm	127		
>ref-49913	CTTGGTTTCACCCCTTCTCCTCCTCTT	1	<lmxll>	ll	151	lm	96		
>ref-49914	GAAAAAGACACAGAAGCTCCTGAGGAT	6	<lmxll>	ll	119	lm	141		
>ref-49962	GTACAAGTGACTTCTTCTCCATCTCCC	12	<lmxll>	ll	142	lm	125		
>ref-49983	CCCTCCCTTACCCTCCCTCCCTCTAAG	13	<nnxnp>	nn	173	np	73		
>ref-49996	CCACCGCTTACGAAACCTCCGCTTATA	8	<nnxnp>	nn	46	np	205		
>ref-49996	CCACCGCTTACGAAACCTCCGCTTATA	24	<hkxhk>	hh	0	hk	18	kk	242
>ref-50021	GCACCGGTCACCTTACCTCCATGGCAA	3	<lmxll>	ll	134	lm	133		
>ref-50021	GCACCGGTCACCTTACCTCCATGGCAA	4	<lmxll>	ll	134	lm	133		
>ref-50021	GCACCGGTCACCTTACCTCCATGGCAA	15	<lmxll>	ll	129	lm	135		
>ref-50026	CTTTCATCCACTTTCTCTCCTATTCCT	6	<nnxnp>	nn	130	np	116		
>ref-50026	CTTTCATCCACTTTCTCTCCTATTCCT	27	<lmxll>	ll	28	lm	207		
>ref-50030	GAGGATAACACTAAGCCTCCGCCGACG	9	<hkxhk>	hh	0	hk	273	kk	0
>ref-50030	GAGGATAACACTAAGCCTCCGCCGACG	14	<hkxhk>	hh	0	hk	263	kk	0
>ref-50030	GAGGATAACACTAAGCCTCCGCCGACG	21	<hkxhk>	hh	0	hk	258	kk	11
>ref-50045	CCTTACTGCACAATAGCTCCTACAAGA	6	<hkxhk>	hh	64	hk	128	kk	49
>ref-50045	CCTTACTGCACAATAGCTCCTACAAGA	16	<nnxnp>	nn	143	np	103		
>ref-50087	ACCTCCGCCACGCCTACTCCCGCCCAC	8	<hkxhk>	hh	0	hk	267	kk	4
>ref-50087	ACCTCCGCCACGCCTACTCCCGCCCAC	16	<hkxhk>	hh	0	hk	273	kk	0
>ref-50099	AGTGGATTCACTTTGTCTCCTTTCTCT	24	<lmxll>	ll	136	lm	135		
>ref-50099	AGTGGATTCACTTTGTCTCCTTTCTCT	27	<lmxll>	ll	136	lm	135		
>ref-50128	CAGCCTACTACCCTTCCTCCCCTCGCT	3	<lmxll>	ll	148	lm	118		
>ref-50142	TCTCCCCCAACCTTCTCTCCCTCCTCC	7	<lmxll>	ll	119	lm	135		
>ref-50142	TCTCCCCCAACCTTCTCTCCCTCCTCC	9	<lmxll>	ll	102	lm	146		
>ref-50160	CGAATATTCACACATACTCCAAGATCA	1	<nnxnp>	nn	101	np	165		
>ref-50162	ACTGCCTTAACTGACTCTCCTTCCTGA	23	<nnxnp>	nn	158	np	111		
>ref-50166	CCACCCGCCACTGGCCCTCCATCACCT	15	<lmxll>	ll	105	lm	166		
>ref-50166	CCACCCGCCACTGGCCCTCCATCACCT	16	<lmxll>	ll	105	lm	166		
>ref-50166	CCACCCGCCACTGGCCCTCCATCACCT	23	<nnxnp>	nn	156	np	112		
>ref-50192	ATGAAAAAAACAATTCCTCCCCGGCGG	5	<hkxhk>	hh	1	hk	264	kk	0
>ref-50192	ATGAAAAAAACAATTCCTCCCCGGCGG	6	<hkxhk>	hh	1	hk	269	kk	0
>ref-50192	ATGAAAAAAACAATTCCTCCCCGGCGG	13	<hkxhk>	hh	1	hk	269	kk	0
>ref-50192	ATGAAAAAAACAATTCCTCCCCGGCGG	15	<hkxhk>	hh	0	hk	264	kk	1
>ref-50218	CAGTGTCTGACTTGGACTCCGTACACC	5	<nnxnp>	nn	111	np	114		
>ref-50218	CAGTGTCTGACTTGGACTCCGTACACC	16	<nnxnp>	nn	112	np	114		
>ref-50225	TCACACTCTACTCCCTCTCCCCCTCCT	3	<nnxnp>	nn	119	np	122		
>ref-50232	ACTCTTTCTACAATGACTCCTCTACCC	8	<nnxnp>	nn	266	np	0		

>ref-50232	ACTCTTTCTACAATGACTCCTCTACCC	9	<nnxnp>	nn	266	np	0		
>ref-50232	ACTCTTTCTACAATGACTCCTCTACCC	26	<nnxnp>	nn	266	np	0		
>ref-50235	ACATCCTCCACTCCATCTCCTCACATG	6	<hkxhk>	hh	81	hk	184	kk	0
>ref-50235	ACATCCTCCACTCCATCTCCTCACATG	23	<hkxhk>	hh	5	hk	264	kk	0
>ref-50235	ACATCCTCCACTCCATCTCCTCACATG	25	<hkxhk>	hh	6	hk	264	kk	0
>ref-50258	GAATGAGAAACAGAGACTCCTAACAAC	1	<nnxnp>	nn	123	np	125		
>ref-50312	CACTCTAAAACCTCGCCTCCTCTCTAT	25	<lmxll>	ll	133	lm	97		
>ref-50313	CACATAGATACTACGCCTCCATTAGCA	25	<nnxnp>	nn	0	np	268		
>ref-50337	TACGCCCCTACATCTCCTCCTACCCCC	2	<lmxll>	ll	122	lm	129		
>ref-50339	TGTGTATACACAAACACTCCCATTCAT	2	<hkxhk>	hh	0	hk	272	kk	0
>ref-50339	TGTGTATACACAAACACTCCCATTCAT	6	<hkxhk>	hh	1	hk	272	kk	0
>ref-50339	TGTGTATACACAAACACTCCCATTCAT	13	<hkxhk>	hh	0	hk	247	kk	0
>ref-50339	TGTGTATACACAAACACTCCCATTCAT	27	<hkxhk>	hh	1	hk	269	kk	3
>ref-50347	GAACAAACAACGTTATCTCCAAGAATG	13	<lmxll>	ll	105	lm	125		
>ref-50352	ACGTCAGCCACTAATGCTCCTTCTCAC	12	<lmxll>	ll	131	lm	131		
>ref-50352	ACGTCAGCCACTAATGCTCCTTCTCAC	13	<lmxll>	ll	131	lm	131		
>ref-50359	ACAACACAAACCCACCCTCCACTTAAC	7	<hkxhk>	hh	50	hk	140	kk	57
>ref-50362	GTCCCCTCAACCCTTTCTCCTGCCTGT	23	<lmxll>	ll	111	lm	150		
>ref-50386	TCTGTGTACACGAAGACTCCTTTCTAT	22	<nnxnp>	nn	125	np	106		
>ref-50424	ACCCGCTTTACTCCAACTCCGATTTTA	5	<nnxnp>	nn	252	np	6		
>ref-50424	ACCCGCTTTACTCCAACTCCGATTTTA	9	<lmxll>	ll	193	lm	37		
>ref-50424	ACCCGCTTTACTCCAACTCCGATTTTA	23	<nnxnp>	nn	193	np	52		
>ref-50428	AGATACGAGACAAAGCCTCCGCGTTTA	21	<nnxnp>	nn	146	np	113		
>ref-50428	AGATACGAGACAAAGCCTCCGCGTTTA	26	<nnxnp>	nn	146	np	113		
>ref-50441	ATCTGCTTAACATTGTCTCCCTACGCA	5	<hkxhk>	hh	54	hk	141	kk	66
>ref-50445	CTGCCAGTGACTCCTCCTCCTCAACCT	22	<hkxhk>	hh	119	hk	140	kk	0
>ref-50452	TTTCAATTTACACCGCCTCCCTCAAGA	26	<hkxhk>	hh	0	hk	254	kk	1
>ref-50459	CAGAACGCAACCGCATCTCCGTTTATT	1	<nnxnp>	nn	128	np	120		
>ref-50460	GGGGAATATACTTACCCTCCCCTTCCC	5	<lmxll>	ll	133	lm	130		
>ref-50467	ATCTCTGACACATTGACTCCTCCTCAT	2	<lmxll>	ll	126	lm	145		
>ref-50469	TGTATCCACACCTCTCCTCCTCTTCCT	7	<lmxll>	ll	123	lm	135		
>ref-50473	CGATCCCTGACCCCCGCTCCTCTCTAC	25	<lmxll>	ll	118	lm	145		
>ref-50475	CCTCCAACAACTCTCTCTCCTCCCTCT	27	<lmxll>	ll	150	lm	115		
>ref-50480	TACATACATACTTACACTCCCTTCTAT	25	<hkxhk>	hh	0	hk	242	kk	13
>ref-50510	CCCACCCCCACCCCCACTCCCTCCACG	9	<hkxhk>	hh	8	hk	213	kk	6
>ref-50515	CTTCTCCTTACCTTGGCTCCTCCTATT	23	<lmxll>	ll	145	lm	87		
>ref-50540	TTCTTCGCTACGTCATCTCCCGACCCG	6	<hkxhk>	hh	45	hk	137	kk	53
>ref-50551	AACAAATTAACATAATCTCCACGAGCT	8	<nnxnp>	nn	71	np	187		

>ref-50585	CACTCCCTTACCTCCCCTCCCCCACAC	26	<lmxll>	ll	30	lm	199		
>ref-50601	CACTGCATTACTCCAACTCCCACTGAT	22	<nnxnp>	nn	117	np	125		
>ref-50613	TCCTACTCTACTATCCCTCCATCCATT	21	<hkxhk>	hh	57	hk	134	kk	63
>ref-50625	CTCTCCGTTACCCTGCCTCCGTACATA	15	<nnxnp>	nn	132	np	103		
>ref-50637	TATATTCCCACCCTTCCTCCTTTCCTC	2	<hkxhk>	hh	8	hk	195	kk	40
>ref-50637	TATATTCCCACCCTTCCTCCTTTCCTC	4	<hkxhk>	hh	8	hk	198	kk	39
>ref-50645	ACTTTCTTGACTGTTCCTCCTCTTGCA	5	<hkxhk>	hh	42	hk	135	kk	83
>ref-50654	TTCGCCCTTACCCCCCCTCCCTCTCCT	27	<lmxll>	ll	227	lm	2		
>ref-50660	CCACCTCGCACACTCGCTCCCTCCCCT	12	<lmxll>	ll	144	lm	127		
>ref-50673	AACGTACTCACAGATACTCCTATCCTT	27	<nnxnp>	nn	126	np	142		
>ref-50690	CCCCTCTACACAACCTCTCCAGCCTGC	1	<lmxll>	ll	148	lm	118		
>ref-50691	CGTGTACACACCTTGACTCCAGCTTCC	4	<lmxll>	ll	127	lm	145		
>ref-50705	ACGACCTCTACAGCCCCTCCAAGAGGG	23	<lmxll>	ll	242	lm	27		
>ref-50705	ACGACCTCTACAGCCCCTCCAAGAGGG	26	<nnxnp>	nn	268	np	5		
>ref-50705	ACGACCTCTACAGCCCCTCCAAGAGGG	27	<hkxhk>	hh	0	hk	261	kk	10
>ref-50760	CTCTCCTTTACCCTTTCTCCTTGCTCT	14	<lmxll>	ll	42	lm	206		
>ref-50760	CTCTCCTTTACCCTTTCTCCTTGCTCT	23	<lmxll>	ll	41	lm	208		
>ref-50778	CCAGCATAAACTCTGCCTCCAGCATTC	27	<hkxhk>	hh	0	hk	272	kk	1
>ref-50799	AGTATAACGACCCCATCTCCTTCCCAA	24	<nnxnp>	nn	93	np	143		
>ref-50802	TCTTCCTCCACCAACCCTCCTTCTTCG	26	<lmxll>	ll	269	lm	0		
>ref-50828	CTCACCTCCACCTCCTCTCCTTTCCCT	7	<hkxhk>	hh	0	hk	266	kk	1
>ref-50828	CTCACCTCCACCTCCTCTCCTTTCCCT	14	<hkxhk>	hh	1	hk	264	kk	2
>ref-50837	AACACCCCCACGCCATCTCCTCAAGCC	21	<lmxll>	ll	117	lm	148		
>ref-50860	CAATCTCCTACTCGTTCTCCTCTCTTG	6	<lmxll>	ll	138	lm	116		
>ref-50866	CAAGGGGAGACAAAGCCTCCTCCAAGA	15	<lmxll>	ll	125	lm	125		
>ref-50887	CTGCCCCCCACCCACCCTCCATGTATC	24	<nnxnp>	nn	120	np	140		
>ref-50908	GGCGGAACTACAAAGCCTCCAACTAAG	22	<nnxnp>	nn	265	np	0		
>ref-50928	ACCTCATCCACCTCATCTCCGTCCCCC	3	<lmxll>	ll	265	lm	0		
>ref-50928	ACCTCATCCACCTCATCTCCGTCCCCC	24	<nnxnp>	nn	136	np	127		
>ref-50944	CACACACTCACACTCACTCCCAGTTGT	23	<nnxnp>	nn	256	np	2		
>ref-50945	CTCTCGCTCACTACCTCTCCTCCTACT	1	<lmxll>	ll	243	lm	11		
>ref-50945	CTCTCGCTCACTACCTCTCCTCCTACT	3	<nnxnp>	nn	249	np	1		
>ref-50956	AATCGCTAAACCGAAGCTCCTTTCGGA	24	<nnxnp>	nn	108	np	159		
>ref-50960	TTTCTCTTCACTCTCACTCCCTCCCCT	15	<nnxnp>	nn	69	np	194		
>ref-50960	TTTCTCTTCACTCTCACTCCCTCCCCT	16	<nnxnp>	nn	64	np	197		
>ref-50988	GGGACCGCGACCTCGCCTCCTCGGATG	8	<lmxll>	ll	123	lm	137		
>ref-50989	CACATGCATACATGCACTCCTTCAAAT	25	<nnxnp>	nn	101	np	142		
>ref-50994	TCCTCAGACACCAAGTCTCCTCTCCCA	2	<nnxnp>	nn	131	np	120		

>ref-51002	TTGGCGTCCACCGTGTCTCCTCGTCCT	6	<nnxnp>	nn	146	np	117		
>ref-51002	TTGGCGTCCACCGTGTCTCCTCGTCCT	12	<lmxll>	ll	7	lm	257		
>ref-51002	TTGGCGTCCACCGTGTCTCCTCGTCCT	25	<nnxnp>	nn	208	np	61		
>ref-51014	GTCCTCCTCACCGAGGCTCCCATGAAT	6	<lmxll>	ll	147	lm	111		
>ref-51017	AAAGGTGATACCTCCGCTCCCGTTGCT	5	<hkxhk>	hh	52	hk	143	kk	52
>ref-51021	CACTGCCACACCCGCCCTCCTTCTCTT	7	<lmxll>	ll	142	lm	128		
>ref-51021	CACTGCCACACCCGCCCTCCTTCTCTT	16	<nnxnp>	nn	103	np	167		
>ref-51021	CACTGCCACACCCGCCCTCCTTCTCTT	21	<nnxnp>	nn	103	np	167		
>ref-51040	AGCATTAATACTCCCTCTCCCTCTCTC	2	<hkxhk>	hh	3	hk	264	kk	0
>ref-51040	AGCATTAATACTCCCTCTCCCTCTCTC	3	<hkxhk>	hh	0	hk	264	kk	3
>ref-51044	AATGAAGAGACGCCGACTCCCAGCATC	21	<nnxnp>	nn	143	np	123		
>ref-51087	TAGTGCATGACTCCCCCTCCTCCTCAT	21	<lmxll>	ll	56	lm	208		
>ref-51087	TAGTGCATGACTCCCCCTCCTCCTCAT	24	<hkxhk>	hh	3	hk	261	kk	5
>ref-51087	TAGTGCATGACTCCCCCTCCTCCTCAT	26	<lmxll>	ll	49	lm	212		
>ref-51091	CTTGGATGCACCCACGCTCCAGGCATT	27	<hkxhk>	hh	0	hk	236	kk	34
>ref-51105	TTTCTCTGAACCCCATCTCCCTTCCCC	4	<hkxhk>	hh	17	hk	246	kk	0
>ref-51105	TTTCTCTGAACCCCATCTCCCTTCCCC	8	<nnxnp>	nn	150	np	90		
>ref-51105	TTTCTCTGAACCCCATCTCCCTTCCCC	16	<hkxhk>	hh	0	hk	161	kk	93
>ref-51114	CCTACCCCCACCTACTCTCCCCTCCTC	16	<lmxll>	ll	1	lm	262		
>ref-51114	CCTACCCCCACCTACTCTCCCCTCCTC	21	<lmxll>	ll	1	lm	262		
>ref-51138	AAGATCTTAACCTATCCTCCTCCTTCT	7	<lmxll>	ll	127	lm	138		
>ref-51154	ATACGCAACACTAACACTCCTCCCCCC	25	<nnxnp>	nn	129	np	136		
>ref-51192	CTCTTTTACACTATCCCTCCTATTCCT	1	<hkxhk>	hh	67	hk	175	kk	12
>ref-51202	CAAATTCCCACTTCCACTCCCTCTCAC	7	<hkxhk>	hh	8	hk	261	kk	0
>ref-51202	CAAATTCCCACTTCCACTCCCTCTCAC	14	<hkxhk>	hh	8	hk	261	kk	0
>ref-51232	GTCCTGTCCACTCTCCCTCCTCCCCCT	12	<nnxnp>	nn	28	np	231		
>ref-51232	GTCCTGTCCACTCTCCCTCCTCCCCCT	26	<nnxnp>	nn	134	np	136		
>ref-51235	GCGAAGAGGACGAAGACTCCAGCGAAA	2	<hkxhk>	hh	3	hk	266	kk	0
>ref-51235	GCGAAGAGGACGAAGACTCCAGCGAAA	12	<hkxhk>	hh	0	hk	273	kk	0
>ref-51235	GCGAAGAGGACGAAGACTCCAGCGAAA	15	<lmxll>	ll	183	lm	69		
>ref-51261	GCAAACCTAACCTTTCCTCCCTTCTAT	2	<hkxhk>	hh	16	hk	241	kk	0
>ref-51261	GCAAACCTAACCTTTCCTCCCTTCTAT	14	<hkxhk>	hh	0	hk	258	kk	9
>ref-51261	GCAAACCTAACCTTTCCTCCCTTCTAT	21	<hkxhk>	hh	9	hk	258	kk	0
>ref-51264	CTCCTACGGACCACTCCTCCTCCTCCT	1	<hkxhk>	hh	231	hk	10	kk	0
>ref-51271	ATGGACTGGACACCACCTCCTCCTCTT	6	<lmxll>	ll	137	lm	129		
>ref-51277	GTGTGAGTGACTGCGCCTCCTTCTCCG	12	<lmxll>	ll	114	lm	153		
>ref-51288	AAAATGTTCACTTCCTCTCCTTTCTCC	26	<nnxnp>	nn	125	np	113		
>ref-51290	CTCACCTACACTTTCTCTCCCTCACTT	8	<nnxnp>	nn	223	np	24		

>ref-51290	CTCACCTACACTTTCTCTCCCTCACTT	13	<nnxnp>	nn	227	np	24		
>ref-51303	ACACGTCGCACTCTTCCTCCGTCCGTC	21	<nnxnp>	nn	129	np	138		
>ref-51304	TAGTATGCTACTGTGTCTCCCGAAGAC	26	<lmxll>	ll	1	lm	267		
>ref-51317	TTTTCCTTGACATTCTCTCCCTCTCTC	8	<lmxll>	ll	5	lm	256		
>ref-51317	TTTTCCTTGACATTCTCTCCCTCTCTC	9	<lmxll>	ll	12	lm	245		
>ref-51317	TTTTCCTTGACATTCTCTCCCTCTCTC	24	<lmxll>	ll	12	lm	246		
>ref-51319	ATGCGTCTCACCCTCCCTCCCTTTCTC	1	<nnxnp>	nn	263	np	1		
>ref-51319	ATGCGTCTCACCCTCCCTCCCTTTCTC	23	<nnxnp>	nn	260	np	1		
>ref-51331	TCACCTTCCACCCACTCTCCCTCCCCT	6	<nnxnp>	nn	251	np	10		
>ref-51331	TCACCTTCCACCCACTCTCCCTCCCCT	9	<lmxll>	ll	31	lm	231		
>ref-51358	CTCAATACTACCTTGTCTCCTCTCCCA	27	<lmxll>	ll	107	lm	150		
>ref-51363	CTTATCGAAACCAAATCTCCAACAATT	1	<lmxll>	ll	119	lm	119		
>ref-51372	CTCTCTGCTACCCTTCCTCCCTCTGGT	25	<lmxll>	ll	16	lm	241		
>ref-51372	CTCTCTGCTACCCTTCCTCCCTCTGGT	26	<lmxll>	ll	16	lm	242		
>ref-51377	CGTCTCCTGACACCTTCTCCTTCCCTT	25	<nnxnp>	nn	128	np	141		
>ref-51394	TATCTTTCAACCAGTCCTCCTTTTCAC	16	<nnxnp>	nn	137	np	126		
>ref-51401	CTAAGCCCTACCCCTCCTCCTCCTCCC	5	<nnxnp>	nn	177	np	69		
>ref-51411	TCTCTCACAACGTTATCTCCCGTACTC	6	<lmxll>	ll	132	lm	134		
>ref-51411	TCTCTCACAACGTTATCTCCCGTACTC	7	<hkxhk>	hh	56	hk	143	kk	64
>ref-51411	TCTCTCACAACGTTATCTCCCGTACTC	26	<hkxhk>	hh	64	hk	143	kk	56
>ref-51417	CTGATGGTCACTGTGGCTCCTCCTTTG	9	<hkxhk>	hh	63	hk	145	kk	58
>ref-51444	TCTCTCTCTACTTCCTCTCCGATTTCA	6	<lmxll>	ll	148	lm	116		
>ref-51444	TCTCTCTCTACTTCCTCTCCGATTTCA	8	<lmxll>	ll	153	lm	111		
>ref-51452	CCTCTGCCCACACAGCCTCCTATCCCC	6	<nnxnp>	nn	23	np	234		
>ref-51452	CCTCTGCCCACACAGCCTCCTATCCCC	15	<nnxnp>	nn	22	np	234		
>ref-51456	CATTCCCCTACCTTATCTCCCCTCCCC	1	<lmxll>	ll	166	lm	58		
>ref-51456	CATTCCCCTACCTTATCTCCCCTCCCC	2	<lmxll>	ll	167	lm	57		
>ref-51469	AAAATATCTACATCTCCTCCTCCTCCT	1	<nnxnp>	nn	229	np	38		
>ref-51469	AAAATATCTACATCTCCTCCTCCTCCT	4	<nnxnp>	nn	179	np	88		
>ref-51469	AAAATATCTACATCTCCTCCTCCTCCT	5	<lmxll>	ll	241	lm	26		
>ref-51469	AAAATATCTACATCTCCTCCTCCTCCT	9	<lmxll>	ll	104	lm	129		
>ref-51471	GGTTGCACAACGTTGTCTCCCTTACTC	22	<lmxll>	ll	121	lm	127		
>ref-51472	TATAAGTCGACTTTCTCTCCTTTCCCT	3	<lmxll>	ll	119	lm	104		
>ref-51472	TATAAGTCGACTTTCTCTCCTTTCCCT	27	<lmxll>	ll	118	lm	105		
>ref-51488	CACGACGGCACTTCCTCTCCCACCTCT	3	<lmxll>	ll	129	lm	131		
>ref-51491	AGAAGGCCGACGGCACCTCCTCGCCCT	1	<nnxnp>	nn	123	np	144		
>ref-51492	TCTCTTATCACTTATTCTCCCTCTTAC	1	<hkxhk>	hh	0	hk	255	kk	5
>ref-51492	TCTCTTATCACTTATTCTCCCTCTTAC	3	<lmxll>	ll	134	lm	122		

>ref-51492	TCTCTTATCACTTATTCTCCCTCTTAC	8	<hkxhk>	hh	0	hk	255	kk	5
>ref-51515	CGTCAACCCACACCTTCTCCAGTCCTC	9	<lmxll>	ll	141	lm	120		
>ref-51516	ATGTCTGTAACATCCACTCCTGTAACT	6	<nnxnp>	nn	133	np	115		
>ref-51535	CGGCCTTCAACACGCCCTCCCGGAGAT	5	<nnxnp>	nn	127	np	136		
>ref-51569	CTCCCTCATACCCAGCCTCCCCCACTT	8	<hkxhk>	hh	1	hk	262	kk	2
>ref-51578	TTCCTATCCACACACCCTCCTAATCCT	14	<nnxnp>	nn	99	np	134		
>ref-51580	CACTCACTCACTCACTCTCCCGAGGTA	2	<lmxll>	ll	132	lm	136		
>ref-51581	CTCTTAGAAACTTGCTCTCCATTGATC	1	<lmxll>	ll	111	lm	126		
>ref-51585	ACTATAACAACCTTCTCTCCATCAATG	7	<nnxnp>	nn	114	np	127		
>ref-51585	ACTATAACAACCTTCTCTCCATCAATG	24	<nnxnp>	nn	114	np	127		
>ref-51589	TTCCCCTTCACTTTATCTCCCTCCCCC	7	<lmxll>	ll	5	lm	265		
>ref-51589	TTCCCCTTCACTTTATCTCCCTCCCCC	13	<lmxll>	ll	5	lm	265		
>ref-51615	TGTCTTATTACCACGCCTCCAACGGTC	13	<hkxhk>	hh	29	hk	236	kk	0
>ref-51615	TGTCTTATTACCACGCCTCCAACGGTC	24	<hkxhk>	hh	0	hk	271	kk	2
>ref-51615	TGTCTTATTACCACGCCTCCAACGGTC	25	<hkxhk>	hh	0	hk	273	kk	0
>ref-51628	CTCTCTTACACACTTTCTCCTTGTCTT	8	<hkxhk>	hh	53	hk	147	kk	51
>ref-51636	TCTTAAATCACTTTCACTCCCTCCCTC	1	<lmxll>	ll	85	lm	135		
>ref-51651	CACGCTTGCACATGATCTCCATTTAGT	23	<nnxnp>	nn	121	np	132		
>ref-51695	CTGCTCGATACCACCCCTCCTTCCATC	1	<nnxnp>	nn	125	np	147		
>ref-51712	TGGATGTACACGGCGACTCCTTTCCAC	21	<hkxhk>	hh	0	hk	270	kk	1
>ref-51722	GTGCTCGGCACTGAACCTCCAGTCGGG	2	<lmxll>	ll	126	lm	142		
>ref-51742	TCCTACTCCACTCCCCCTCCCTTTTTA	8	<hkxhk>	hh	59	hk	185	kk	1
>ref-51742	TCCTACTCCACTCCCCCTCCCTTTTTA	13	<nnxnp>	nn	74	np	149		
>ref-51742	TCCTACTCCACTCCCCCTCCCTTTTTA	24	<hkxhk>	hh	1	hk	186	kk	60
>ref-51744	CTTCTCATGACTAAATCTCCTTCTCCC	25	<nnxnp>	nn	117	np	109		
>ref-51754	TATCCCCCCACCTCTTCTCCTTGCCTT	5	<lmxll>	ll	7	lm	255		
>ref-51754	TATCCCCCCACCTCTTCTCCTTGCCTT	25	<lmxll>	ll	7	lm	255		
>ref-51758	CGGGCGTTTACCATGTCTCCCTGTATC	16	<lmxll>	ll	112	lm	148		
>ref-51766	CACATGTACACCCACTCTCCTACCCCC	13	<nnxnp>	nn	122	np	147		
>ref-51779	GCCATGGCAACCGGATCTCCATGACTG	7	<lmxll>	ll	131	lm	136		
>ref-51779	GCCATGGCAACCGGATCTCCATGACTG	25	<nnxnp>	nn	133	np	130		
>ref-51811	CACAAATGCACAGCTACTCCCCCACCT	15	<lmxll>	ll	138	lm	115		
>ref-51833	TACCACCATACCCCTTCTCCCATACTA	12	<hkxhk>	hh	8	hk	251	kk	0
>ref-51833	TACCACCATACCCCTTCTCCCATACTA	16	<hkxhk>	hh	0	hk	251	kk	9
>ref-51852	TTCTCTTCTACCCTTTCTCCCTTAATC	12	<lmxll>	ll	130	lm	122		
>ref-51852	TTCTCTTCTACCCTTTCTCCCTTAATC	25	<nnxnp>	nn	123	np	121		
>ref-51871	TGATGTACCACAGTTCCTCCATTTCCT	7	<nnxnp>	nn	85	np	136		
>ref-51877	ATCGTCCTAACTGCATCTCCCTGTCAT	7	<lmxll>	ll	125	lm	145		

>ref-51883	TCCTGCTTAACACCTTCTCCCTCTTTT	3	<nnxnp>	nn	120	np	145		
>ref-51891	CCCCTTGCAACTCTGCCTCCAATTCTG	8	<lmxll>	ll	148	lm	109		
>ref-51891	CCCCTTGCAACTCTGCCTCCAATTCTG	15	<lmxll>	ll	148	lm	109		
>ref-51895	CCTCTCTTAACCCTTTCTCCCTCTTCC	4	<nnxnp>	nn	157	np	82		
>ref-51895	CCTCTCTTAACCCTTTCTCCCTCTTCC	16	<nnxnp>	nn	155	np	83		
>ref-51903	GCGTGTGAGACTATGGCTCCTGTGGTC	8	<hkxhk>	hh	53	hk	147	kk	61
>ref-51909	TATGCATTTACCTGCTCTCCCCTTTGT	25	<nnxnp>	nn	229	np	0		
>ref-51920	TACATATATACACTCCCTCCTCTCCCT	9	<hkxhk>	hh	131	hk	108	kk	0
>ref-51920	TACATATATACACTCCCTCCTCTCCCT	16	<hkxhk>	hh	129	hk	107	kk	0
>ref-51922	TCCTTTCCTACTCCCCCTCCCCCACCT	3	<hkxhk>	hh	0	hk	271	kk	0
>ref-51922	TCCTTTCCTACTCCCCCTCCCCCACCT	5	<lmxll>	ll	242	lm	24		
>ref-51922	TCCTTTCCTACTCCCCCTCCCCCACCT	21	<lmxll>	ll	1	lm	270		
>ref-51922	TCCTTTCCTACTCCCCCTCCCCCACCT	24	<hkxhk>	hh	0	hk	273	kk	0
>ref-51943	TATGATAATACATCCCCTCCCTCCCTC	5	<lmxll>	ll	118	lm	140		
>ref-51951	CTCTCTCCGACTTAATCTCCCTCTCCC	5	<nnxnp>	nn	137	np	130		
>ref-51963	CGACCCTATACAGCCGCTCCCCACTAT	3	<lmxll>	ll	1	lm	271		
>ref-51969	TTCCTATCTACTCTCCCTCCTCTCCCT	5	<lmxll>	ll	262	lm	7		
>ref-51969	TTCCTATCTACTCTCCCTCCTCTCCCT	24	<lmxll>	ll	264	lm	3		
>ref-51969	TTCCTATCTACTCTCCCTCCTCTCCCT	26	<lmxll>	ll	132	lm	126		
>ref-51985	CAGAATTTTACATGCTCTCCTTGCATA	6	<nnxnp>	nn	131	np	114		
>ref-52000	CGAGGTTTTACACACACTCCATCTTTC	2	<hkxhk>	hh	6	hk	244	kk	2
>ref-52011	CCTTCCCTCACCCAACCTCCCCCTTCC	1	<hkxhk>	hh	0	hk	273	kk	0
>ref-52011	CCTTCCCTCACCCAACCTCCCCCTTCC	22	<nnxnp>	nn	11	np	255		
>ref-52011	CCTTCCCTCACCCAACCTCCCCCTTCC	24	<nnxnp>	nn	12	np	254		
>ref-52011	CCTTCCCTCACCCAACCTCCCCCTTCC	27	<lmxll>	ll	259	lm	11		
>ref-52013	TAGGTTCTGACCAGTCCTCCATGCCTC	3	<nnxnp>	nn	144	np	125		
>ref-52077	CTGCCCTCAACCCTCCCTCCCTCCGTC	3	<lmxll>	ll	147	lm	74		
>ref-52085	GCCTCCTCGACGGCCTCTCCTCCGCCA	24	<lmxll>	ll	119	lm	144		
>ref-52106	CTCTCCAACACTACTTCTCCCTCCTAT	14	<nnxnp>	nn	133	np	117		
>ref-52111	CGAGAACAGACTCATTCTCCTTTCACT	8	<nnxnp>	nn	121	np	141		
>ref-52126	CACCCACCCACCCCTTCTCCTCCTATC	21	<lmxll>	ll	6	lm	255		
>ref-52126	CACCCACCCACCCCTTCTCCTCCTATC	22	<lmxll>	ll	6	lm	255		
>ref-52129	TCCATCCATACTCTCTCTCCATACACA	6	<lmxll>	ll	121	lm	134		
>ref-52143	GACACACATACTCTTTCTCCTTTCTCG	7	<nnxnp>	nn	124	np	136		
>ref-52153	TACTGTAAGACGTTCCCTCCTCACTGC	24	<lmxll>	ll	127	lm	143		
>ref-52155	TCTTCCTCTACATCCTCTCCTTCATTC	2	<nnxnp>	nn	139	np	112		
>ref-52155	TCTTCCTCTACATCCTCTCCTTCATTC	3	<lmxll>	ll	105	lm	140		
>ref-52155	TCTTCCTCTACATCCTCTCCTTCATTC	8	<lmxll>	ll	105	lm	140		

>ref-52195	ATCACCACCACTTCATCTCCACCACTT	7	<nnxnp>	nn	3	np	266		
>ref-52205	TCCCCCTGCACCCCCTCTCCCCCTGCA	21	<nnxnp>	nn	249	np	0		
>ref-52210	ATGGTGCTGACACAATCTCCCGGCAGC	4	<hkxhk>	hh	0	hk	243	kk	20
>ref-52210	ATGGTGCTGACACAATCTCCCGGCAGC	14	<lmxll>	ll	3	lm	269		
>ref-52210	ATGGTGCTGACACAATCTCCCGGCAGC	21	<hkxhk>	hh	5	hk	267	kk	0
>ref-52216	CAGCGAGGCACAGCATCTCCCTCCTCT	25	<nnxnp>	nn	137	np	133		
>ref-52218	AGTGTGTGTACTTTGCCTCCGGACGTG	25	<lmxll>	ll	142	lm	130		
>ref-52238	TCCTCTGTTACGTCCTCTCCCTCATCC	12	<hkxhk>	hh	47	hk	149	kk	57
>ref-52255	TCCCCCCCAACCAAGCCTCCCTCTCAC	4	<nnxnp>	nn	147	np	118		
>ref-52255	TCCCCCCCAACCAAGCCTCCCTCTCAC	16	<hkxhk>	hh	78	hk	184	kk	0
>ref-52261	TGCTGAACTACTCCCCCTCCTAAGCCA	1	<lmxll>	ll	123	lm	133		
>ref-52261	TGCTGAACTACTCCCCCTCCTAAGCCA	13	<nnxnp>	nn	130	np	128		
>ref-52265	CTCTGCGCGACATTGTCTCCCATTGTG	21	<nnxnp>	nn	261	np	0		
>ref-52282	CAATCACTCACTCCCCCTCCTCTCCTC	13	<lmxll>	ll	111	lm	147		
>ref-52284	ATACTTCCCACCCTTTCTCCCTCACTC	6	<lmxll>	ll	253	lm	1		
>ref-52296	CACGCTCCAACGTTTTCTCCATCACCA	8	<hkxhk>	hh	43	hk	158	kk	50
>ref-52296	CACGCTCCAACGTTTTCTCCATCACCA	25	<hkxhk>	hh	43	hk	158	kk	50
>ref-52316	CCTTACCTAACAGCAGCTCCCTTCTTG	26	<hkxhk>	hh	56	hk	137	kk	58
>ref-52325	AACACAGTAACGCTCTCTCCTCCCTCG	23	<nnxnp>	nn	271	np	0		
>ref-52332	GACAAAACAACACGGACTCCAGCAGAC	13	<lmxll>	ll	120	lm	146		
>ref-52332	GACAAAACAACACGGACTCCAGCAGAC	16	<lmxll>	ll	120	lm	146		
>ref-52345	AGGTCTAGGACCCCTCCTCCGACAAGC	25	<lmxll>	ll	0	lm	273		
>ref-52355	TTTGCCTTCACCTCCCCTCCCCTCCCC	1	<nnxnp>	nn	234	np	1		
>ref-52355	TTTGCCTTCACCTCCCCTCCCCTCCCC	7	<nnxnp>	nn	112	np	124		
>ref-52355	TTTGCCTTCACCTCCCCTCCCCTCCCC	14	<nnxnp>	nn	251	np	4		
>ref-52355	TTTGCCTTCACCTCCCCTCCCCTCCCC	21	<nnxnp>	nn	120	np	120		
>ref-52355	TTTGCCTTCACCTCCCCTCCCCTCCCC	24	<lmxll>	ll	258	lm	1		
>ref-52355	TTTGCCTTCACCTCCCCTCCCCTCCCC	26	<nnxnp>	nn	256	np	0		
>ref-52360	CTCCTTGTAACCTTAACTCCTTTCCTG	1	<hkxhk>	hh	73	hk	119	kk	36
>ref-52360	CTCCTTGTAACCTTAACTCCTTTCCTG	12	<hkxhk>	hh	36	hk	118	kk	73
>ref-52379	AAGCAGTACACTGAACCTCCAAAGCAG	2	<lmxll>	ll	123	lm	139		
>ref-52379	AAGCAGTACACTGAACCTCCAAAGCAG	13	<lmxll>	ll	128	lm	138		
>ref-52385	TTTAGGAAGACATTTACTCCGTCGCAA	24	<nnxnp>	nn	118	np	121		
>ref-52386	CTGCCTGTCACTAACCCTCCCCATATA	25	<nnxnp>	nn	122	np	112		
>ref-52407	GACAGGCCAACCTCCTCTCCTCCTCCT	7	<lmxll>	ll	134	lm	124		
>ref-52412	CCTTCTTTCACCTTTCCTCCTTATTAT	6	<lmxll>	ll	9	lm	260		
>ref-52412	CCTTCTTTCACCTTTCCTCCTTATTAT	7	<lmxll>	ll	8	lm	261		
>ref-52437	GAAGGCATCACTGCTGCTCCTCCATGA	3	<hkxhk>	hh	56	hk	211	kk	0

>ref-52437	GAAGGCATCACTGCTGCTCCTCCATGA	16	<hkxhk>	hh	0	hk	211	kk	56
>ref-52510	AGGGCTAAAACAGCTTCTCCAACATAT	27	<nnxnp>	nn	138	np	129		
>ref-52523	CAAGCACACACATACACTCCCAAAGAC	21	<nnxnp>	nn	119	np	134		
>ref-52527	ATCATAATCACCTGTTCTCCCCGCCGG	27	<hkxhk>	hh	63	hk	123	kk	57
>ref-52559	CTGCTTGCTACTTTGCCTCCTCCTCCT	4	<hkxhk>	hh	61	hk	148	kk	57
>ref-52559	CTGCTTGCTACTTTGCCTCCTCCTCCT	9	<hkxhk>	hh	56	hk	146	kk	64
>ref-52564	TTCGTAGTCACACTTACTCCCTATTCT	1	<hkxhk>	hh	0	hk	270	kk	0
>ref-52564	TTCGTAGTCACACTTACTCCCTATTCT	4	<hkxhk>	hh	81	hk	180	kk	0
>ref-52599	GAGTAAATTACTTCCACTCCCTCAGCA	7	<lmxll>	ll	116	lm	142		
>ref-52620	AGGCCCTGCACCGAAGCTCCATGGCAA	21	<hkxhk>	hh	77	hk	142	kk	50
>ref-52652	CTTACTCCTACCCAGCCTCCCTCGTCT	24	<nnxnp>	nn	132	np	134		
>ref-52660	CGGGACGGGACTCACGCTCCAGGGTGA	25	<lmxll>	ll	161	lm	105		
>ref-52671	AAAACCAAAACGCGTCCTCCGATGCCC	16	<lmxll>	ll	134	lm	131		
>ref-52678	CTATCACCCACTCCCCCTCCTTTCTTC	13	<nnxnp>	nn	96	np	162		
>ref-52681	TCACCCTTTACCTACCCTCCCTCCCTC	3	<nnxnp>	nn	207	np	58		
>ref-52681	TCACCCTTTACCTACCCTCCCTCCCTC	15	<lmxll>	ll	115	lm	149		
>ref-52681	TCACCCTTTACCTACCCTCCCTCCCTC	21	<hkxhk>	hh	2	hk	270	kk	0
>ref-52701	AGAAAACAAACAAGGGCTCCAAACAAG	2	<lmxll>	ll	107	lm	146		
>ref-52701	AGAAAACAAACAAGGGCTCCAAACAAG	3	<lmxll>	ll	135	lm	112		
>ref-52701	AGAAAACAAACAAGGGCTCCAAACAAG	6	<hkxhk>	hh	51	hk	213	kk	1
>ref-52701	AGAAAACAAACAAGGGCTCCAAACAAG	21	<hkxhk>	hh	51	hk	205	kk	0
>ref-52710	AGATTCTACACCGCAACTCCCTTACTG	22	<hkxhk>	hh	57	hk	142	kk	67
>ref-52710	AGATTCTACACCGCAACTCCCTTACTG	26	<lmxll>	ll	132	lm	135		
>ref-52713	TCGAAAATAACCCAATCTCCGCCTCTG	1	<nnxnp>	nn	127	np	95		
>ref-52714	TAAACCCATACTGTTCCTCCGAGACTC	1	<hkxhk>	hh	0	hk	47	kk	205
>ref-52714	TAAACCCATACTGTTCCTCCGAGACTC	6	<hkxhk>	hh	0	hk	14	kk	256
>ref-52714	TAAACCCATACTGTTCCTCCGAGACTC	9	<lmxll>	ll	5	lm	267		
>ref-52715	TACCCCAAGACACGCCCTCCTCCCCCT	26	<nnxnp>	nn	99	np	147		
>ref-52715	TACCCCAAGACACGCCCTCCTCCCCCT	27	<nnxnp>	nn	100	np	146		
>ref-52719	AGGATGCAAACACACACTCCGACTCAT	1	<nnxnp>	nn	116	np	152		
>ref-52730	GCTGGTTAAACCCTCCCTCCTCAGTTT	27	<nnxnp>	nn	219	np	0		
>ref-52754	ATGAACCATACAATAACTCCTTTCCTG	4	<lmxll>	ll	117	lm	110		
>ref-52764	CTATTCATTACCTCAACTCCATGCCCT	7	<nnxnp>	nn	118	np	104		
>ref-52774	CAGTGGTCCACGACAACTCCTCCCCCT	2	<lmxll>	ll	183	lm	72		
>ref-52774	CAGTGGTCCACGACAACTCCTCCCCCT	5	<nnxnp>	nn	200	np	57		
>ref-52774	CAGTGGTCCACGACAACTCCTCCCCCT	6	<hkxhk>	hh	0	hk	166	kk	94
>ref-52774	CAGTGGTCCACGACAACTCCTCCCCCT	8	<lmxll>	ll	185	lm	71		
>ref-52776	ACATGACTAACGTCTTCTCCGGTTGCA	25	<nnxnp>	nn	133	np	137		

>ref-52795	TTCACACTAACCTTCCCTCCCTCCTTT	6	<lmxll>	ll	262	lm	0		
>ref-52795	TTCACACTAACCTTCCCTCCCTCCTTT	9	<lmxll>	ll	261	lm	0		
>ref-52795	TTCACACTAACCTTCCCTCCCTCCTTT	26	<hkxhk>	hh	128	hk	130	kk	0
>ref-52796	CCTCTCCCTACAACCCCTCCTCCTTTA	26	<nnxnp>	nn	115	np	128		
>ref-52810	CCCCCGTTAACCCTCCCTCCCTCTTCC	6	<lmxll>	ll	58	lm	180		
>ref-52810	CCCCCGTTAACCCTCCCTCCCTCTTCC	12	<lmxll>	ll	62	lm	178		
>ref-52811	CTTCCCTTTACCTCTCCTCCCTCAGGC	26	<nnxnp>	nn	117	np	149		
>ref-52819	ACATGAACAACAGATACTCCTCTGTCA	4	<nnxnp>	nn	99	np	135		
>ref-52826	ATATTTCTTACTCTTCCTCCATCTGCT	27	<nnxnp>	nn	110	np	134		
>ref-52857	CAAGGCTTCACCGAGGCTCCACCGAGG	2	<hkxhk>	hh	4	hk	262	kk	0
>ref-52857	CAAGGCTTCACCGAGGCTCCACCGAGG	7	<hkxhk>	hh	0	hk	272	kk	0
>ref-52857	CAAGGCTTCACCGAGGCTCCACCGAGG	23	<lmxll>	ll	175	lm	90		
>ref-52865	GTACTACAGACGCTCACTCCTCTAATC	4	<hkxhk>	hh	3	hk	270	kk	0
>ref-52865	GTACTACAGACGCTCACTCCTCTAATC	14	<lmxll>	ll	133	lm	134		
>ref-52885	CATACTGTCACACAGGCTCCTGAAGCA	4	<hkxhk>	hh	0	hk	273	kk	0
>ref-52885	CATACTGTCACACAGGCTCCTGAAGCA	14	<hkxhk>	hh	0	hk	273	kk	0
>ref-52915	TTTCCTCGCACGTTTCCTCCCTACTTG	12	<nnxnp>	nn	139	np	116		
>ref-52925	ACTTTATGTACATCTTCTCCTCCTTTT	8	<nnxnp>	nn	123	np	129		
>ref-52940	GGCAGAGGGACTGGGGCTCCGTTGGTT	15	<nnxnp>	nn	115	np	129		
>ref-52963	CCCACATCCACAGCTGCTCCCATGTCC	26	<lmxll>	ll	135	lm	136		
>ref-52979	CCATCACTCACTATCTCTCCATCACTC	1	<lmxll>	ll	5	lm	267		
>ref-52979	CCATCACTCACTATCTCTCCATCACTC	3	<lmxll>	ll	5	lm	267		
>ref-52979	CCATCACTCACTATCTCTCCATCACTC	22	<lmxll>	ll	44	lm	201		
>ref-52984	ACAGCTCCCACACTTGCTCCCGTTACG	1	<lmxll>	ll	120	lm	146		
>ref-52984	ACAGCTCCCACACTTGCTCCCGTTACG	16	<nnxnp>	nn	129	np	133		
>ref-52991	TCCCCTTTCACACTCCCTCCCTCGTTT	2	<nnxnp>	nn	116	np	142		
>ref-53026	ACATTTTCTACTTCCTCTCCCCCCTCC	22	<nnxnp>	nn	114	np	114		
>ref-53041	GGACTATGCACGTTCTCTCCTTGAACG	1	<hkxhk>	hh	25	hk	242	kk	0
>ref-53041	GGACTATGCACGTTCTCTCCTTGAACG	25	<nnxnp>	nn	227	np	36		
>ref-53041	GGACTATGCACGTTCTCTCCTTGAACG	26	<hkxhk>	hh	19	hk	252	kk	0
>ref-53054	CCCTCTTCCACCACCTCTCCATCCCCT	22	<hkxhk>	hh	0	hk	160	kk	75
>ref-53069	CTCTCTCTCACTTACTCTCCTACATTC	6	<lmxll>	ll	121	lm	146		
>ref-53073	CTACGTCTAACCCTCTCTCCTCCTTTC	1	<nnxnp>	nn	149	np	120		
>ref-53073	CTACGTCTAACCCTCTCTCCTCCTTTC	3	<nnxnp>	nn	149	np	120		
>ref-53083	ATTCACTTCACTCCGGCTCCATTTTCT	26	<nnxnp>	nn	245	np	0		
>ref-53108	TTCTCCTTTACGCATCCTCCTTTTCCC	16	<lmxll>	ll	108	lm	144		
>ref-53146	CCTCCACGTACAGCAACTCCACCCACA	1	<nnxnp>	nn	257	np	0		
>ref-53146	CCTCCACGTACAGCAACTCCACCCACA	2	<lmxll>	ll	121	lm	113		

>ref-53157	ATCTGTCTGACGATCCCTCCATATATT	23	<lmxll>	ll	99	lm	136		
>ref-53191	CGCTTCCCCACACCTCCTCCTCCCCAC	26	<hkxhk>	hh	82	hk	131	kk	48
>ref-53194	TGTATGCACACGCATACTCCACATATA	16	<lmxll>	ll	132	lm	130		
>ref-53232	TCCTCCCCCACCCTTCCTCCCCCCTCC	7	<hkxhk>	hh	0	hk	273	kk	0
>ref-53232	TCCTCCCCCACCCTTCCTCCCCCCTCC	21	<hkxhk>	hh	2	hk	271	kk	0
>ref-53232	TCCTCCCCCACCCTTCCTCCCCCCTCC	22	<hkxhk>	hh	0	hk	273	kk	0
>ref-53237	CCTTTCTCTACGTACCCTCCTCTTCTT	15	<hkxhk>	hh	58	hk	132	kk	57
>ref-53239	CCTCACTTTACACACTCTCCTCACTAT	23	<hkxhk>	hh	0	hk	273	kk	0
>ref-53239	CCTCACTTTACACACTCTCCTCACTAT	24	<lmxll>	ll	234	lm	31		
>ref-53239	CCTCACTTTACACACTCTCCTCACTAT	25	<lmxll>	ll	103	lm	152		
>ref-53239	CCTCACTTTACACACTCTCCTCACTAT	26	<hkxhk>	hh	0	hk	273	kk	0
>ref-53239	CCTCACTTTACACACTCTCCTCACTAT	27	<lmxll>	ll	181	lm	77		
>ref-53248	TCCTTACTTACCTTATCTCCTCCTCAC	24	<nnxnp>	nn	103	np	130		
>ref-53248	TCCTTACTTACCTTATCTCCTCCTCAC	25	<nnxnp>	nn	103	np	130		
>ref-53264	ACGTTTCGAACTTTCTCTCCCTCTCCT	8	<lmxll>	ll	125	lm	130		
>ref-53274	CCCCAACCCACCTACTCTCCCAGTCCA	1	<nnxnp>	nn	200	np	45		
>ref-53274	CCCCAACCCACCTACTCTCCCAGTCCA	26	<lmxll>	ll	81	lm	156		
>ref-53294	CATCAACTAACCCATCCTCCCTTATTA	16	<nnxnp>	nn	32	np	233		
>ref-53294	CATCAACTAACCCATCCTCCCTTATTA	26	<lmxll>	ll	13	lm	257		
>ref-53294	CATCAACTAACCCATCCTCCCTTATTA	27	<lmxll>	ll	13	lm	257		
>ref-53309	CCAAGGCCTACTCTACCTCCAATAACA	12	<lmxll>	ll	36	lm	219		
>ref-53326	GTACTGTCGACTGCCTCTCCTGGTGAT	13	<lmxll>	ll	136	lm	123		
>ref-53327	CCCTGACGTACACTCGCTCCCTCATTA	5	<lmxll>	ll	127	lm	139		
>ref-53342	GTGCTACAGACGTCTTCTCCGCATCAG	2	<hkxhk>	hh	0	hk	268	kk	2
>ref-53342	GTGCTACAGACGTCTTCTCCGCATCAG	22	<hkxhk>	hh	3	hk	270	kk	0
>ref-53367	ATTAAAACGACACACTCTCCCACGCAC	24	<nnxnp>	nn	127	np	135		
>ref-53395	AGACGAACGACTGTGTCTCCGTCGTAA	4	<nnxnp>	nn	136	np	128		
>ref-53418	TACACATTCACCTTATCTCCTGCTTTT	2	<lmxll>	ll	110	lm	145		
>ref-53418	TACACATTCACCTTATCTCCTGCTTTT	3	<lmxll>	ll	110	lm	146		
>ref-53426	CGCACGCTCACCCCCTCTCCCATTCAC	12	<nnxnp>	nn	228	np	38		
>ref-53430	GCCGGACGGACGGACTCTCCCCTTGGA	3	<nnxnp>	nn	113	np	112		
>ref-53444	CCTACTGTCACGGTCACTCCTACTGTC	12	<hkxhk>	hh	2	hk	270	kk	0
>ref-53444	CCTACTGTCACGGTCACTCCTACTGTC	24	<hkxhk>	hh	0	hk	111	kk	141
>ref-53448	CATCTATTTACCCCCCCTCCCCCACAC	8	<nnxnp>	nn	111	np	134		
>ref-53449	CTTTCTTTTACTCTACCTCCTTCTCTT	23	<hkxhk>	hh	32	hk	133	kk	65
>ref-53450	AAGTAGAAAACCCTTTCTCCCAGTGTT	26	<nnxnp>	nn	234	np	0		
>ref-53454	TCCCCACTAACGCCTCCTCCTCTTCCT	27	<hkxhk>	hh	6	hk	260	kk	0
>ref-53456	TTTCTTCGTACTCAGTCTCCTTCCGTT	14	<hkxhk>	hh	64	hk	139	kk	61

>ref-53467	TATACGCTTACACCATCTCCAACCCCC	2	<lmxll>	ll	136	lm	106		
>ref-53468	CCATTTGCCACAGCATCTCCTTGTCCC	25	<lmxll>	ll	11	lm	255		
>ref-53478	CAGCCCCACACACGCACTCCCACTCTC	14	<hkxhk>	hh	121	hk	120	kk	0
>ref-53478	CAGCCCCACACACGCACTCCCACTCTC	21	<nnxnp>	nn	138	np	130		
>ref-53478	CAGCCCCACACACGCACTCCCACTCTC	26	<hkxhk>	hh	0	hk	186	kk	71
>ref-53485	AGACAGAAGACCTTCCCTCCCGAGGTA	24	<nnxnp>	nn	137	np	131		
>ref-53505	TGCAATGGAACACATCCTCCCTCCTAA	12	<lmxll>	ll	117	lm	138		
>ref-53506	AATACACTCACAAATTCTCCTAAAGCA	1	<nnxnp>	nn	108	np	128		
>ref-53541	CCCTCCTTCACATACCCTCCACAACCC	15	<hkxhk>	hh	9	hk	263	kk	0
>ref-53541	CCCTCCTTCACATACCCTCCACAACCC	25	<hkxhk>	hh	9	hk	263	kk	0
>ref-53541	CCCTCCTTCACATACCCTCCACAACCC	26	<hkxhk>	hh	5	hk	263	kk	0
>ref-53550	TTGCCTTTCACTCTGCCTCCCCAAGCC	2	<lmxll>	ll	115	lm	137		
>ref-53567	CACTTACTCACTTCCACTCCTTCCCAC	25	<nnxnp>	nn	261	np	0		
>ref-53570	ACCCTTTCCACTTGCCCTCCTCCCGCT	27	<nnxnp>	nn	152	np	119		
>ref-53571	CGAATTCAAACGCATCCTCCTTCTTCT	25	<lmxll>	ll	116	lm	134		
>ref-53584	ATTACGCTCACAATATCTCCTCATTAG	1	<nnxnp>	nn	7	np	256		
>ref-53584	ATTACGCTCACAATATCTCCTCATTAG	5	<nnxnp>	nn	7	np	256		
>ref-53609	ACTACCACTACTACAACTCCTAGTACC	14	<nnxnp>	nn	148	np	80		
>ref-53649	GCGGTTGTTACTGTAGCTCCTGTTGTG	13	<nnxnp>	nn	134	np	125		
>ref-53663	CTCTCACGCACGTCTTCTCCCTCACGT	5	<lmxll>	ll	10	lm	258		
>ref-53668	TACATACAGACGCTTCCTCCATCGCGA	5	<hkxhk>	hh	0	hk	187	kk	76
>ref-53668	TACATACAGACGCTTCCTCCATCGCGA	23	<hkxhk>	hh	0	hk	273	kk	0
>ref-53668	TACATACAGACGCTTCCTCCATCGCGA	26	<hkxhk>	hh	0	hk	270	kk	3
>ref-53672	CAGATGCATACATATCCTCCATTTGAT	3	<lmxll>	ll	2	lm	269		
>ref-53672	CAGATGCATACATATCCTCCATTTGAT	5	<hkxhk>	hh	0	hk	264	kk	6
>ref-53672	CAGATGCATACATATCCTCCATTTGAT	7	<nnxnp>	nn	236	np	25		
>ref-53672	CAGATGCATACATATCCTCCATTTGAT	9	<lmxll>	ll	124	lm	133		
>ref-53672	CAGATGCATACATATCCTCCATTTGAT	12	<hkxhk>	hh	2	hk	270	kk	0
>ref-53672	CAGATGCATACATATCCTCCATTTGAT	15	<hkxhk>	hh	0	hk	213	kk	45
>ref-53672	CAGATGCATACATATCCTCCATTTGAT	22	<lmxll>	ll	32	lm	229		
>ref-53698	ATTTGCATAACGTCAGCTCCACGATAA	1	<nnxnp>	nn	145	np	117		
>ref-53701	CTCCCTCTAACCCCCACTCCTCTCCAG	4	<nnxnp>	nn	169	np	86		
>ref-53715	TGAAATGAGACAATAACTCCTTTGAAA	1	<lmxll>	ll	58	lm	206		
>ref-53715	TGAAATGAGACAATAACTCCTTTGAAA	2	<hkxhk>	hh	0	hk	14	kk	254
>ref-53715	TGAAATGAGACAATAACTCCTTTGAAA	5	<nnxnp>	nn	273	np	0		
>ref-53715	TGAAATGAGACAATAACTCCTTTGAAA	13	<nnxnp>	nn	94	np	162		
>ref-53715	TGAAATGAGACAATAACTCCTTTGAAA	25	<nnxnp>	nn	216	np	48		
>ref-53739	TCCTCTCCCACCACCCCTCCCCTCCCC	13	<hkxhk>	hh	7	hk	262	kk	1

>ref-53810	TTATCATCAACGTCATCTCCATTATCA	2	<lmxll>	ll	95	lm	150		
>ref-53810	TTATCATCAACGTCATCTCCATTATCA	9	<lmxll>	ll	95	lm	150		
>ref-53815	TTCCTCGCGACTGTTTCTCCCTCCATA	16	<hkxhk>	hh	49	hk	149	kk	47
>ref-53822	TCCGCCCCTACTTCTCCTCCTGCTTCT	2	<nnxnp>	nn	268	np	0		
>ref-53822	TCCGCCCCTACTTCTCCTCCTGCTTCT	6	<nnxnp>	nn	268	np	0		
>ref-53822	TCCGCCCCTACTTCTCCTCCTGCTTCT	15	<nnxnp>	nn	115	np	144		
>ref-53822	TCCGCCCCTACTTCTCCTCCTGCTTCT	22	<nnxnp>	nn	249	np	2		
>ref-53833	TGCGCCTTCACCATTGCTCCCTAAAGA	5	<lmxll>	ll	121	lm	140		
>ref-53834	GCTCTTTGCACCCAACCTCCAGCATGT	8	<lmxll>	ll	153	lm	118		
>ref-53837	CAACGTTCGACCCCCCCTCCCCCTTTT	16	<nnxnp>	nn	111	np	127		
>ref-53867	CACCTGGCTACCCTCCCTCCAACCCTC	7	<nnxnp>	nn	122	np	129		
>ref-53879	AATGTTACAACACGAGCTCCGAATGCT	26	<lmxll>	ll	139	lm	127		
>ref-53885	CAACTTCCTACCTCCCCTCCTCCCCTT	3	<nnxnp>	nn	3	np	249		
>ref-53885	CAACTTCCTACCTCCCCTCCTCCCCTT	5	<lmxll>	ll	262	lm	0		
>ref-53885	CAACTTCCTACCTCCCCTCCTCCCCTT	6	<nnxnp>	nn	4	np	250		
>ref-53885	CAACTTCCTACCTCCCCTCCTCCCCTT	12	<lmxll>	ll	261	lm	0		
>ref-53921	TTCGCCCTGACACTGGCTCCCCCTCAC	12	<hkxhk>	hh	6	hk	264	kk	0
>ref-53937	CTACCTCTCACTCCCCCTCCCTTTCCA	3	<lmxll>	ll	1	lm	220		
>ref-53940	GACTACATTACAAGGCCTCCCAATGCA	2	<nnxnp>	nn	271	np	0		
>ref-53943	CCCTCTTTCACCTCTGCTCCCTCCTTC	6	<hkxhk>	hh	0	hk	270	kk	1
>ref-53943	CCCTCTTTCACCTCTGCTCCCTCCTTC	15	<hkxhk>	hh	0	hk	270	kk	1
>ref-53952	AAAGCTGGCACTCTCCCTCCTCCTCCT	26	<hkxhk>	hh	57	hk	140	kk	56
>ref-53959	TTAATAATTACCATCTCTCCAGTGTAA	6	<nnxnp>	nn	210	np	49		
>ref-53965	CCCTCTCTCACCCTCCCTCCCTCTTGA	3	<lmxll>	ll	25	lm	236		
>ref-53965	CCCTCTCTCACCCTCCCTCCCTCTTGA	5	<lmxll>	ll	25	lm	235		
>ref-53984	GACTGATACACACTCTCTCCCGTCTCA	1	<nnxnp>	nn	123	np	123		
>ref-53995	GAAGCCCGTACACCTTCTCCGCACAGC	24	<hkxhk>	hh	63	hk	142	kk	56
>ref-53999	CTCCGGTTGACACTTCCTCCCCGAACG	2	<lmxll>	ll	76	lm	174		
>ref-54017	TCCCCTTCCACACCTTCTCCCATGCAC	12	<nnxnp>	nn	273	np	0		
>ref-54095	GCCGCTGTAACTGGATCTCCTCCCTTG	24	<nnxnp>	nn	142	np	116		
>ref-54105	GGAAGTAAAACGCGGTCTCCGTTTCTC	2	<nnxnp>	nn	111	np	159		
>ref-54106	CTACCTTCCACTTAACCTCCGGCGTCC	8	<nnxnp>	nn	141	np	130		
>ref-54126	CTTTACTCCACCTGTTCTCCTGCTTCT	25	<lmxll>	ll	114	lm	153		
>ref-54135	TATAATATAACGAACCCTCCCTCTGTT	5	<lmxll>	ll	121	lm	123		
>ref-54135	TATAATATAACGAACCCTCCCTCTGTT	12	<lmxll>	ll	119	lm	123		
>ref-54136	CAGAGGCCCACTCCCCCTCCCCTCATG	1	<nnxnp>	nn	129	np	139		
>ref-54137	CCCAATCACACCCGCTCTCCCTCTCCC	24	<hkxhk>	hh	0	hk	264	kk	9
>ref-54137	CCCAATCACACCCGCTCTCCCTCTCCC	25	<hkxhk>	hh	9	hk	264	kk	0

>ref-54145	TGCATAAGGACATCGGCTCCTCTGTGT	14	<nnxnp>	nn	133	np	137		
>ref-54151	CACCCACCAACCCAGCCTCCAGTCCAC	1	<lmxll>	ll	127	lm	134		
>ref-54153	GTCTACCGTACATGCCCTCCGCCTAAT	26	<lmxll>	ll	117	lm	148		
>ref-54154	GTAGGGAAAACTCTTGCTCCTTAGCAA	2	<nnxnp>	nn	108	np	140		
>ref-54222	AAGTACAACACACATCCTCCAAGCGCT	3	<lmxll>	ll	129	lm	143		
>ref-54265	GGTATCATCACAAGCTCTCCCGGAGAT	23	<lmxll>	ll	141	lm	120		
>ref-54266	CTGCATGTCACTTAAGCTCCATGGCAT	3	<hkxhk>	hh	0	hk	273	kk	0
>ref-54266	CTGCATGTCACTTAAGCTCCATGGCAT	9	<nnxnp>	nn	69	np	183		
>ref-54266	CTGCATGTCACTTAAGCTCCATGGCAT	24	<hkxhk>	hh	8	hk	259	kk	0
>ref-54275	AGACTTGGGACAGACACTCCATAGTAT	21	<lmxll>	ll	130	lm	138		
>ref-54285	GAGACAACAACGCCTGCTCCTTGGTCA	26	<hkxhk>	hh	53	hk	156	kk	50
>ref-54285	GAGACAACAACGCCTGCTCCTTGGTCA	27	<hkxhk>	hh	53	hk	157	kk	49
>ref-54307	ATTCCACGCACAGCCCCTCCAGCTAGT	8	<lmxll>	ll	123	lm	143		
>ref-54338	TTATGAGGAACCATGACTCCTAGATAT	8	<hkxhk>	hh	0	hk	49	kk	218
>ref-54338	TTATGAGGAACCATGACTCCTAGATAT	14	<nnxnp>	nn	65	np	199		
>ref-54340	CTACAGTGGACATGATCTCCCTTCGAC	8	<nnxnp>	nn	5	np	265		
>ref-54340	CTACAGTGGACATGATCTCCCTTCGAC	27	<nnxnp>	nn	5	np	265		
>ref-54350	ACCCTTCCAACCCCCTCTCCCCCACCT	5	<hkxhk>	hh	0	hk	272	kk	0
>ref-54350	ACCCTTCCAACCCCCTCTCCCCCACCT	24	<hkxhk>	hh	0	hk	272	kk	0
>ref-54350	ACCCTTCCAACCCCCTCTCCCCCACCT	26	<nnxnp>	nn	135	np	125		
>ref-54356	AAATGTTGCACTTCGACTCCTCAACTT	6	<lmxll>	ll	125	lm	137		
>ref-54384	TTCCTACCCACCCTTTCTCCCTTCAAC	1	<nnxnp>	nn	248	np	0		
>ref-54384	TTCCTACCCACCCTTTCTCCCTTCAAC	6	<nnxnp>	nn	249	np	0		
>ref-54384	TTCCTACCCACCCTTTCTCCCTTCAAC	25	<nnxnp>	nn	251	np	0		
>ref-54401	CCCCCTACCACCCTATCTCCCCCTCCC	1	<lmxll>	ll	157	lm	100		
>ref-54401	CCCCCTACCACCCTATCTCCCCCTCCC	5	<nnxnp>	nn	174	np	75		
>ref-54401	CCCCCTACCACCCTATCTCCCCCTCCC	13	<hkxhk>	hh	9	hk	256	kk	0
>ref-54401	CCCCCTACCACCCTATCTCCCCCTCCC	25	<nnxnp>	nn	127	np	126		
>ref-54404	TATCACCCCACTTTCGCTCCTTCATTA	7	<nnxnp>	nn	105	np	124		
>ref-54407	CACAGACTCACCCCCACTCCCTCACCA	8	<lmxll>	ll	130	lm	130		
>ref-54407	CACAGACTCACCCCCACTCCCTCACCA	12	<lmxll>	ll	132	lm	129		
>ref-54442	ATCACCACAACACCGTCTCCTCACCAT	25	<nnxnp>	nn	144	np	120		
>ref-54459	TCTCCCCCAACCTTCCCTCCCTTATAT	6	<nnxnp>	nn	119	np	116		
>ref-54463	TATCCTCCCACAGTTTCTCCATCCTCT	26	<nnxnp>	nn	142	np	122		
>ref-54470	TCCTCTCTTACGTCCTCTCCTCTCTCC	1	<hkxhk>	hh	49	hk	150	kk	66
>ref-54470	TCCTCTCTTACGTCCTCTCCTCTCTCC	2	<hkxhk>	hh	66	hk	151	kk	49
>ref-54489	TAAAATCGTACCCTCTCTCCCTCTCTC	27	<lmxll>	ll	129	lm	124		
>ref-54499	TGTGGATGCACATGCTCTCCCTCTCTC	3	<hkxhk>	hh	52	hk	128	kk	69

>ref-54550	CTCTCTCTAACGGTCCCTCCTTCACCT	9	<hkxhk>	hh	52	hk	144	kk	64
>ref-54556	GATGCACACACTCGTTCTCCTTCCATC	9	<nnxnp>	nn	160	np	109		
>ref-54583	ATTATGATGACCCTCGCTCCAGCTCTC	7	<nnxnp>	nn	109	np	154		
>ref-54584	CTTTCCGCTACCAACTCTCCTCTATCC	3	<lmxll>	ll	132	lm	132		
>ref-54585	ATTCCGTTTACGCAATCTCCTTCCTTC	6	<nnxnp>	nn	114	np	132		
>ref-54590	TCTCAAGTAACCTTTCCTCCTCGCCTT	5	<lmxll>	ll	132	lm	131		
>ref-54593	GAAACCATTACTATTCCTCCAGCGATA	23	<lmxll>	ll	21	lm	236		
>ref-54593	GAAACCATTACTATTCCTCCAGCGATA	24	<lmxll>	ll	11	lm	249		
>ref-54599	TGGCGCCTGACTGGGTCTCCTTCCCCT	16	<nnxnp>	nn	130	np	141		
>ref-54603	TTTCTATCTACTTTATCTCCTTTTCCT	22	<hkxhk>	hh	3	hk	239	kk	16
>ref-54603	TTTCTATCTACTTTATCTCCTTTTCCT	24	<hkxhk>	hh	1	hk	149	kk	95
>ref-54647	GGGTTTTAGACTTATGCTCCCGTTCCG	15	<nnxnp>	nn	114	np	147		
>ref-54667	CCGCATGTCACCTTTCCTCCTCCTCCT	21	<nnxnp>	nn	113	np	144		
>ref-54689	TATCTGCTTACCCCTCCTCCCCTCTCC	2	<lmxll>	ll	5	lm	258		
>ref-54689	TATCTGCTTACCCCTCCTCCCCTCTCC	27	<lmxll>	ll	5	lm	258		
>ref-54695	CTTGCCATGACGTCATCTCCTTGCCAT	1	<hkxhk>	hh	0	hk	273	kk	0
>ref-54695	CTTGCCATGACGTCATCTCCTTGCCAT	5	<hkxhk>	hh	0	hk	273	kk	0
>ref-54695	CTTGCCATGACGTCATCTCCTTGCCAT	24	<hkxhk>	hh	16	hk	247	kk	0
>ref-54705	CCTCTTAGCACTCTCTCTCCCCCTTCC	8	<lmxll>	ll	116	lm	144		
>ref-54727	TCATCCCTTACTTTGCCTCCATCCCAT	4	<nnxnp>	nn	130	np	136		
>ref-54753	TTCAGCTCCACCTCCCCTCCACCTTCA	22	<lmxll>	ll	122	lm	102		
>ref-54773	GTCATCATCACCCCAGCTCCCCTCCTT	26	<nnxnp>	nn	130	np	132		
>ref-54805	CGCTCTCTCACTCGCACTCCATCACAT	2	<nnxnp>	nn	135	np	138		
>ref-54819	CACTTCACAACAGCCGCTCCTTCTGGC	21	<lmxll>	ll	125	lm	143		
>ref-54826	CCACTAACAACAAAATCTCCTCCTCCT	12	<nnxnp>	nn	234	np	1		
>ref-54832	TGTCTTCGCACGTCCTCTCCCGACCGC	12	<lmxll>	ll	116	lm	156		
>ref-54832	TGTCTTCGCACGTCCTCTCCCGACCGC	16	<lmxll>	ll	116	lm	156		
>ref-54860	AGATCAATAACTTTCTCTCCTAAGGCC	21	<hkxhk>	hh	0	hk	270	kk	1
>ref-54860	AGATCAATAACTTTCTCTCCTAAGGCC	23	<hkxhk>	hh	27	hk	227	kk	0
>ref-54908	TCAATCATTACAATAGCTCCGTAATTA	3	<nnxnp>	nn	107	np	145		
>ref-54916	TCCTTCCATACCCCTCCTCCTTTCCCA	15	<hkxhk>	hh	0	hk	237	kk	13
>ref-54916	TCCTTCCATACCCCTCCTCCTTTCCCA	26	<hkxhk>	hh	13	hk	237	kk	0
>ref-54918	CTTCCTACCACAGCATCTCCTCCCCTT	4	<lmxll>	ll	124	lm	140		
>ref-54919	TTAAGTCCAACATAAACTCCTTAAGAT	9	<hkxhk>	hh	60	hk	148	kk	47
>ref-54930	AAAAGCAGAACTAACACTCCCCCCTCC	5	<lmxll>	ll	123	lm	127		
>ref-54937	AAGAGCCCCACTCTCCCTCCCTTTTCA	25	<nnxnp>	nn	92	np	134		
>ref-54939	TCCTCGTGTACCTCATCTCCTTCTTAT	8	<lmxll>	ll	139	lm	131		
>ref-54939	TCCTCGTGTACCTCATCTCCTTCTTAT	26	<nnxnp>	nn	133	np	127		

>ref-54941	GGAAGTTCCACAAGCACTCCAAACCCG	12	<nnxnp>	nn	267	np	0		
>ref-54955	TTCCAAAATACCTACGCTCCGACTGCT	1	<lmxll>	ll	113	lm	117		
>ref-54983	TTCTCTTCGACCTCTTCTCCTTCTCTC	9	<hkxhk>	hh	5	hk	261	kk	3
>ref-54983	TTCTCTTCGACCTCTTCTCCTTCTCTC	23	<hkxhk>	hh	5	hk	262	kk	3
>ref-54989	TTCATCCCGACTCATTCTCCGTCGTCC	13	<nnxnp>	nn	143	np	110		
>ref-54993	ACTCTTCCAACCCCTCCTCCACCCTTC	25	<hkxhk>	hh	0	hk	267	kk	4
>ref-55059	ACTAATTCCACAACTACTCCCTCCACA	14	<nnxnp>	nn	3	np	261		
>ref-55059	ACTAATTCCACAACTACTCCCTCCACA	16	<nnxnp>	nn	3	np	261		
>ref-55069	AGAGTTCAGACGCCCTCTCCGTCTTGA	15	<nnxnp>	nn	116	np	139		
>ref-55071	CGGTGTCCCACACCTCCTCCTGTTCTA	23	<nnxnp>	nn	123	np	145		
>ref-55075	CTTTAACCAACACGTCCTCCAAAGTTA	12	<lmxll>	ll	118	lm	129		
>ref-55075	CTTTAACCAACACGTCCTCCAAAGTTA	13	<lmxll>	ll	119	lm	129		
>ref-55078	ACGATAAAGACCTTAACTCCTTAACTA	4	<nnxnp>	nn	144	np	120		
>ref-55084	AGCCCAGAGACCCTCCCTCCACCTCCG	21	<lmxll>	ll	137	lm	129		
>ref-55087	TAGACAAGGACATTGGCTCCTCGGTGG	2	<nnxnp>	nn	111	np	150		
>ref-55087	TAGACAAGGACATTGGCTCCTCGGTGG	6	<nnxnp>	nn	111	np	150		
>ref-55087	TAGACAAGGACATTGGCTCCTCGGTGG	25	<lmxll>	ll	109	lm	159		
>ref-55091	TCCTTACCCACCGACCCTCCCTTTTCC	12	<lmxll>	ll	126	lm	132		
>ref-55103	CCCTCGTCTACTACCCCTCCCCCACTT	15	<nnxnp>	nn	263	np	0		
>ref-55112	ATAATTGGGACGCTTCCTCCTAGTTTA	1	<lmxll>	ll	3	lm	264		
>ref-55112	ATAATTGGGACGCTTCCTCCTAGTTTA	12	<lmxll>	ll	3	lm	264		
>ref-55138	AGGATTTTTACTCTTCCTCCTCCTCCT	3	<hkxhk>	hh	0	hk	271	kk	0
>ref-55138	AGGATTTTTACTCTTCCTCCTCCTCCT	6	<hkxhk>	hh	0	hk	270	kk	1
>ref-55143	CCCGTCTCGACCTTGGCTCCGGCCGTC	25	<lmxll>	ll	114	lm	106		
>ref-55144	CTAGGGTTTACCATCTCTCCTGAAGGT	22	<hkxhk>	hh	0	hk	194	kk	52
>ref-55152	GGATGATTCACGTTGCCTCCTTCTGTA	24	<hkxhk>	hh	52	hk	140	kk	72
>ref-55195	TCCTTCACCACCTCCCCTCCCCCTCCC	1	<lmxll>	ll	234	lm	24		
>ref-55195	TCCTTCACCACCTCCCCTCCCCCTCCC	27	<lmxll>	ll	248	lm	10		
>ref-55219	CACACAGGCACTTACACTCCAGCACAC	8	<nnxnp>	nn	144	np	129		
>ref-55220	GCAGTGTCGACACTCGCTCCAGAGAGG	26	<nnxnp>	nn	220	np	0		
>ref-55220	GCAGTGTCGACACTCGCTCCAGAGAGG	27	<nnxnp>	nn	219	np	1		
>ref-55229	TCCTTTGCCACGTTCACTCCTGTCCAC	24	<nnxnp>	nn	142	np	127		
>ref-55235	TCCTTCCTCACTTCCTCTCCTCTTCGT	23	<lmxll>	ll	263	lm	0		
>ref-55235	TCCTTCCTCACTTCCTCTCCTCTTCGT	26	<lmxll>	ll	255	lm	4		
>ref-55281	GTAATTATTACAGTCACTCCTGTCTCC	2	<hkxhk>	hh	0	hk	258	kk	6
>ref-55281	GTAATTATTACAGTCACTCCTGTCTCC	8	<hkxhk>	hh	0	hk	141	kk	111
>ref-55281	GTAATTATTACAGTCACTCCTGTCTCC	9	<hkxhk>	hh	0	hk	259	kk	7
>ref-55281	GTAATTATTACAGTCACTCCTGTCTCC	12	<hkxhk>	hh	45	hk	209	kk	0

>ref-55282	CCTTCTCTAACCCCTTCTCCTAGTCTT	25	<lmxll>	ll	154	lm	76		
>ref-55283	CTCTCTTTCACCTAATCTCCCTGCCCC	24	<lmxll>	ll	135	lm	106		
>ref-55293	CATCCACCTACGTGCGCTCCCCTCCTT	22	<lmxll>	ll	137	lm	132		
>ref-55323	GTTCTTTACACTTTCGCTCCATCCCCT	9	<nnxnp>	nn	125	np	133		
>ref-55345	CCCTTTCTCACCTGTCCTCCCCTTCCC	6	<lmxll>	ll	135	lm	132		
>ref-55350	AGCATCCCAACCTCATCTCCATCCCTC	21	<lmxll>	ll	130	lm	137		
>ref-55353	GCAGATCTCACTCTCCCTCCCTCCCTC	2	<nnxnp>	nn	223	np	0		
>ref-55368	GCCCTTGGAACAGGAACTCCCAAACCT	7	<lmxll>	ll	132	lm	117		
>ref-55386	GAGATAGAGACGCCCCCTCCTCCTCAT	3	<nnxnp>	nn	129	np	143		
>ref-55386	GAGATAGAGACGCCCCCTCCTCCTCAT	5	<hkxhk>	hh	66	hk	131	kk	67
>ref-55386	GAGATAGAGACGCCCCCTCCTCCTCAT	23	<lmxll>	ll	137	lm	131		
>ref-55386	GAGATAGAGACGCCCCCTCCTCCTCAT	24	<nnxnp>	nn	136	np	134		
>ref-55395	ATCCAGTATACTACATCTCCTGTTTAC	1	<hkxhk>	hh	57	hk	135	kk	43
>ref-55395	ATCCAGTATACTACATCTCCTGTTTAC	2	<hkxhk>	hh	44	hk	135	kk	57
>ref-55405	ACTACACCCACTACAACTCCAACAATA	7	<nnxnp>	nn	126	np	121		
>ref-55405	ACTACACCCACTACAACTCCAACAATA	13	<hkxhk>	hh	116	hk	120	kk	0
>ref-55405	ACTACACCCACTACAACTCCAACAATA	16	<nnxnp>	nn	154	np	87		
>ref-55414	CTTCCCCCAACTGTGTCTCCCTTTTCT	9	<nnxnp>	nn	116	np	138		
>ref-55414	CTTCCCCCAACTGTGTCTCCCTTTTCT	25	<nnxnp>	nn	120	np	136		
>ref-55437	GCTGCCGTGACTCATCCTCCTGGCGCT	5	<lmxll>	ll	124	lm	130		
>ref-55447	CATTCGCAGACAATCCCTCCCAGAGAC	25	<hkxhk>	hh	50	hk	152	kk	42
>ref-55460	TTTCCCTCCACGCTACCTCCGACCTTT	23	<nnxnp>	nn	130	np	130		
>ref-55482	CAACCTCCGACCTTCTCTCCCTTTCAT	22	<lmxll>	ll	79	lm	175		
>ref-55482	CAACCTCCGACCTTCTCTCCCTTTCAT	25	<lmxll>	ll	79	lm	175		
>ref-55485	TCCCTGAAAACTCGCTCTCCTTGATCA	24	<lmxll>	ll	111	lm	115		
>ref-55508	ACCCCAATCACCACCTCTCCCCCTCCC	5	<nnxnp>	nn	141	np	127		
>ref-55559	GAATCAAGCACATCTACTCCATATGAT	7	<hkxhk>	hh	123	hk	135	kk	0
>ref-55578	CATCCATCCACCTCCCCTCCCTGCCTC	13	<lmxll>	ll	264	lm	0		
>ref-55578	CATCCATCCACCTCCCCTCCCTGCCTC	23	<nnxnp>	nn	100	np	124		
>ref-55651	GACACCATAACTTCCCCTCCTCTTCCT	5	<hkxhk>	hh	62	hk	134	kk	71
>ref-55656	CACCTTTCTACTCCTTCTCCCAACTTT	16	<nnxnp>	nn	109	np	112		
>ref-55661	ATACCTTCTACATACACTCCACATTCA	6	<lmxll>	ll	135	lm	107		
>ref-55665	GCTCCTTCTACCCTTCCTCCACCCTTT	1	<nnxnp>	nn	161	np	95		
>ref-55665	GCTCCTTCTACCCTTCCTCCACCCTTT	3	<nnxnp>	nn	164	np	95		
>ref-55668	GCCTTCGAGACTCCGGCTCCATATTCT	8	<lmxll>	ll	0	lm	258		
>ref-55668	GCCTTCGAGACTCCGGCTCCATATTCT	22	<lmxll>	ll	0	lm	258		
>ref-55680	GCATCCTGCACGTTATCTCCCTTATTT	7	<nnxnp>	nn	121	np	140		
>ref-55728	AGGACCCTGACAGGTGCTCCACTGGAT	26	<nnxnp>	nn	270	np	0		

>ref-55729	CCTATTGGTACTATTTCTCCAAACTCA	2	<lmxll>	ll	117	lm	123		
>ref-55794	AATACACAAACTTATTCTCCTCTTTAA	3	<hkxhk>	hh	0	hk	201	kk	29
>ref-55794	AATACACAAACTTATTCTCCTCTTTAA	5	<hkxhk>	hh	1	hk	224	kk	11
>ref-55794	AATACACAAACTTATTCTCCTCTTTAA	16	<hkxhk>	hh	0	hk	200	kk	29
>ref-55794	AATACACAAACTTATTCTCCTCTTTAA	21	<lmxll>	ll	164	lm	63		
>ref-55799	TGTGTAACTACTATTGCTCCAACTACT	8	<lmxll>	ll	117	lm	129		
>ref-55807	ACCCGCCATACCCCTCCTCCTCCCTCC	22	<nnxnp>	nn	117	np	146		
>ref-55813	ATCCGTGTAACCACAGCTCCATCAGCC	23	<lmxll>	ll	126	lm	140		
>ref-55815	ATCATTTGTACTCCGTCTCCTCCTCTT	1	<lmxll>	ll	127	lm	134		
>ref-55818	TAACAAGCAACCTTCCCTCCCTGCAGG	6	<lmxll>	ll	137	lm	130		
>ref-55837	GTCAAGTCAACAGATCCTCCTACGTAT	12	<nnxnp>	nn	116	np	125		
>ref-55841	TCCTGCTACACACATACTCCTCACTCC	5	<lmxll>	ll	265	lm	0		
>ref-55851	ATAAACAATACGGTCTCTCCTCAACTC	26	<lmxll>	ll	135	lm	124		
>ref-55863	CAAGCACGCACGAAACCTCCCTCCCAC	15	<hkxhk>	hh	60	hk	148	kk	52
>ref-55872	CCACTGCCCACCCCTTCTCCCTCTGCC	26	<hkxhk>	hh	0	hk	230	kk	12
>ref-55873	CCCTTTCCTACCTCATCTCCCCCAATA	22	<hkxhk>	hh	39	hk	142	kk	64
>ref-55885	TCCAAAACAACAGGGTCTCCAGTTGGC	13	<hkxhk>	hh	5	hk	257	kk	0
>ref-55885	TCCAAAACAACAGGGTCTCCAGTTGGC	25	<hkxhk>	hh	5	hk	257	kk	0
>ref-55889	GATTATCAGACCACGGCTCCCTGACTG	24	<lmxll>	ll	131	lm	129		
>ref-55933	CCTCCTCCAACACCCTCTCCTCCTACC	12	<lmxll>	ll	61	lm	193		
>ref-55933	CCTCCTCCAACACCCTCTCCTCCTACC	13	<hkxhk>	hh	149	hk	102	kk	0
>ref-55933	CCTCCTCCAACACCCTCTCCTCCTACC	16	<lmxll>	ll	55	lm	200		
>ref-55933	CCTCCTCCAACACCCTCTCCTCCTACC	25	<hkxhk>	hh	2	hk	254	kk	0
>ref-55933	CCTCCTCCAACACCCTCTCCTCCTACC	27	<nnxnp>	nn	236	np	34		
>ref-55934	CCCGCACTCACTCTGTCTCCTTCTTTT	3	<lmxll>	ll	114	lm	125		
>ref-55934	CCCGCACTCACTCTGTCTCCTTCTTTT	6	<lmxll>	ll	114	lm	124		
>ref-55942	TTCTTTCTTACTCAGTCTCCCGTTAAT	3	<hkxhk>	hh	34	hk	208	kk	0
>ref-55947	TCTCCCTTAACCCTCCCTCCCTCCCCA	2	<nnxnp>	nn	180	np	53		
>ref-55947	TCTCCCTTAACCCTCCCTCCCTCCCCA	7	<nnxnp>	nn	171	np	56		
>ref-55947	TCTCCCTTAACCCTCCCTCCCTCCCCA	21	<lmxll>	ll	2	lm	220		
>ref-55947	TCTCCCTTAACCCTCCCTCCCTCCCCA	24	<lmxll>	ll	1	lm	218		
>ref-55956	AACAGACGCACAGCCTCTCCATTTCGA	6	<nnxnp>	nn	268	np	0		
>ref-55982	TCTCAGCATACTGGGACTCCATGAGTG	1	<nnxnp>	nn	118	np	135		
>ref-55982	TCTCAGCATACTGGGACTCCATGAGTG	27	<nnxnp>	nn	271	np	0		
>ref-55983	TTCCCGCTCACACACTCTCCGTCCTGC	5	<nnxnp>	nn	140	np	128		
>ref-55992	TTAATCAACACTTCAACTCCCGATGCA	7	<lmxll>	ll	114	lm	126		
>ref-55996	CACAAACAAACATCCACTCCCCCACCT	16	<hkxhk>	hh	2	hk	270	kk	0
>ref-55996	CACAAACAAACATCCACTCCCCCACCT	24	<hkxhk>	hh	2	hk	270	kk	0

>ref-56009	ACCACCACCACCATCTCTCCACCTGCA	4	<nnxnp>	nn	136	np	131		
>ref-56021	TTTGAACAGACAACCGCTCCCACATAT	23	<hkxhk>	hh	14	hk	244	kk	0
>ref-56021	TTTGAACAGACAACCGCTCCCACATAT	24	<hkxhk>	hh	14	hk	244	kk	0
>ref-56023	CGAGGAACTACACGCTCTCCACTACGG	21	<lmxll>	ll	110	lm	149		
>ref-56038	GAAATGCCTACAATGACTCCTCTACAA	8	<lmxll>	ll	114	lm	124		
>ref-56062	CCTCTCGACACTCACGCTCCTTTATCT	6	<lmxll>	ll	128	lm	130		
>ref-56067	ACTAAACACACACACGCTCCGCTCTCT	1	<hkxhk>	hh	60	hk	150	kk	56
>ref-56068	AGCAAACACACACGTACTCCTCCCCCA	2	<nnxnp>	nn	121	np	134		
>ref-56070	TTTGTCTCCACTTTTTCTCCCTCATCT	2	<nnxnp>	nn	135	np	134		
>ref-56073	CTTTTGTAGACGCCCCCTCCCTATGTT	23	<lmxll>	ll	134	lm	127		
>ref-56087	GGTAAGGTCACAAGGTCTCCTAATTGA	2	<nnxnp>	nn	266	np	3		
>ref-56087	GGTAAGGTCACAAGGTCTCCTAATTGA	15	<lmxll>	ll	257	lm	9		
>ref-56087	GGTAAGGTCACAAGGTCTCCTAATTGA	26	<hkxhk>	hh	0	hk	141	kk	122
>ref-56088	TCTAGTGGAACGGTATCTCCTCTCTCC	22	<hkxhk>	hh	134	hk	110	kk	1
>ref-56092	TAATATCGAACAGCGTCTCCAACTCTT	26	<lmxll>	ll	124	lm	142		
>ref-56098	ATTGGACACACGGTCCCTCCAGGTATA	13	<nnxnp>	nn	208	np	50		
>ref-56098	ATTGGACACACGGTCCCTCCAGGTATA	14	<hkxhk>	hh	0	hk	216	kk	48
>ref-56105	TGGAGCTTCACGTCACCTCCTGGAATG	1	<hkxhk>	hh	0	hk	225	kk	31
>ref-56105	TGGAGCTTCACGTCACCTCCTGGAATG	16	<hkxhk>	hh	0	hk	269	kk	1
>ref-56105	TGGAGCTTCACGTCACCTCCTGGAATG	27	<hkxhk>	hh	0	hk	269	kk	1
>ref-56119	TTCTGTCTTACAGCAACTCCTCTCATC	4	<lmxll>	ll	141	lm	128		
>ref-56152	CTCATTACTACCCCTTCTCCTTCTCCT	1	<nnxnp>	nn	133	np	117		
>ref-56165	AGCCACAGGACCGCGCCTCCATCTGGG	15	<hkxhk>	hh	0	hk	38	kk	227
>ref-56173	CTCTCTTTCACCTCCCCTCCACATCAA	2	<lmxll>	ll	116	lm	136		
>ref-56199	ATTCCGGCCACTGTCTCTCCACCCTAC	23	<nnxnp>	nn	109	np	126		
>ref-56212	ACCTATCTCACCCCACCTCCTATCCAT	14	<hkxhk>	hh	72	hk	128	kk	40
>ref-56212	ACCTATCTCACCCCACCTCCTATCCAT	16	<lmxll>	ll	125	lm	130		
>ref-56212	ACCTATCTCACCCCACCTCCTATCCAT	27	<hkxhk>	hh	40	hk	128	kk	72
>ref-56229	ACTCAAGGCACCTCCGCTCCCCCTCTC	15	<nnxnp>	nn	124	np	144		
>ref-56256	TTCTTCTTTACTTCTTCTCCTTCCTTA	24	<nnxnp>	nn	250	np	0		
>ref-56256	TTCTTCTTTACTTCTTCTCCTTCCTTA	27	<nnxnp>	nn	237	np	0		
>ref-56286	CTCCATCACACTCTTCCTCCCGCACCT	15	<nnxnp>	nn	10	np	258		
>ref-56295	TAAGAAAATACACTCCCTCCTATTATT	5	<lmxll>	ll	103	lm	121		
>ref-56306	CTAAAGCATACTTTCCCTCCAAGCTCT	25	<nnxnp>	nn	146	np	97		
>ref-56344	TCTCTTTGAACATCCCCTCCATTTCTC	23	<nnxnp>	nn	125	np	139		
>ref-56346	TCCCTCTCCACTCCCACTCCCATCCCC	2	<nnxnp>	nn	265	np	0		
>ref-56346	TCCCTCTCCACTCCCACTCCCATCCCC	15	<hkxhk>	hh	212	hk	39	kk	0
>ref-56346	TCCCTCTCCACTCCCACTCCCATCCCC	16	<lmxll>	ll	261	lm	1		

>ref-56346	TCCCTCTCCACTCCCACTCCCATCCCC	22	<lmxll>	ll	257	lm	3		
>ref-56346	TCCCTCTCCACTCCCACTCCCATCCCC	23	<hkxhk>	hh	0	hk	40	kk	211
>ref-56346	TCCCTCTCCACTCCCACTCCCATCCCC	26	<nnxnp>	nn	0	np	264		
>ref-56354	ATCTCCCTCACAGCATCTCCCTCAGTA	13	<nnxnp>	nn	257	np	16		
>ref-56380	TAGTATCCCACTGCCTCTCCTGCCCCT	9	<nnxnp>	nn	130	np	141		
>ref-56397	ACAGGTCCCACAGCGCCTCCGACAGAT	27	<hkxhk>	hh	0	hk	273	kk	0
>ref-56401	CCCCTCCCCACTTCTCCTCCTTCCAGT	27	<nnxnp>	nn	115	np	130		
>ref-56409	CGACTCTCGACACCGACTCCCGACACC	2	<hkxhk>	hh	0	hk	265	kk	5
>ref-56409	CGACTCTCGACACCGACTCCCGACACC	7	<hkxhk>	hh	0	hk	270	kk	0
>ref-56409	CGACTCTCGACACCGACTCCCGACACC	12	<nnxnp>	nn	217	np	36		
>ref-56416	TACCCCCCCACCCCCACTCCACCGCTC	4	<nnxnp>	nn	118	np	126		
>ref-56416	TACCCCCCCACCCCCACTCCACCGCTC	21	<lmxll>	ll	136	lm	96		
>ref-56416	TACCCCCCCACCCCCACTCCACCGCTC	24	<lmxll>	ll	138	lm	95		
>ref-56424	TCCTCTGACACCGCCTCTCCCTCGTTT	26	<nnxnp>	nn	127	np	140		
>ref-56425	CGAAATCAGACTCCATCTCCTCCGCCT	8	<lmxll>	ll	107	lm	120		
>ref-56436	TCCATTTTGACTCCTTCTCCTTCTCTT	26	<nnxnp>	nn	218	np	2		
>ref-56458	TTTAAACAAACGACGTCTCCAGTAGTT	22	<hkxhk>	hh	68	hk	142	kk	53
>ref-56521	TGACATTTAACGAGCTCTCCCCTTTGC	1	<hkxhk>	hh	46	hk	122	kk	85
>ref-56538	CCGGTGTTAACACTAGCTCCATCTGCA	27	<hkxhk>	hh	0	hk	272	kk	1
>ref-56594	TTATCTCGCACTCGTTCTCCGTCTCCA	3	<nnxnp>	nn	137	np	135		
>ref-56602	AGTTGTCACACAAATCCTCCGTCAACT	25	<hkxhk>	hh	1	hk	269	kk	0
>ref-56602	AGTTGTCACACAAATCCTCCGTCAACT	26	<hkxhk>	hh	7	hk	259	kk	0
>ref-56614	GTCCGTCCGACGCTCCCTCCTGCCTCC	13	<nnxnp>	nn	132	np	102		
>ref-56615	ATCCTAGTAACTAGCTCTCCCCTCTCT	5	<nnxnp>	nn	129	np	128		
>ref-56624	ATTATTACAACCCCCCCTCCCCCTCCC	1	<lmxll>	ll	8	lm	235		
>ref-56624	ATTATTACAACCCCCCCTCCCCCTCCC	4	<lmxll>	ll	3	lm	241		
>ref-56644	AATTCTCTTACCCACTCTCCTTCTCAT	5	<nnxnp>	nn	140	np	122		
>ref-56644	AATTCTCTTACCCACTCTCCTTCTCAT	7	<nnxnp>	nn	140	np	122		
>ref-56685	CCACTCCTTACTTCTCCTCCCCCATTT	3	<lmxll>	ll	4	lm	267		
>ref-56685	CCACTCCTTACTTCTCCTCCCCCATTT	25	<hkxhk>	hh	1	hk	202	kk	63
>ref-56685	CCACTCCTTACTTCTCCTCCCCCATTT	26	<lmxll>	ll	4	lm	267		
>ref-56709	TTGTTCTCCACCATTCCTCCCCCTAAC	1	<lmxll>	ll	4	lm	253		
>ref-56709	TTGTTCTCCACCATTCCTCCCCCTAAC	3	<lmxll>	ll	113	lm	119		
>ref-56709	TTGTTCTCCACCATTCCTCCCCCTAAC	5	<lmxll>	ll	113	lm	119		
>ref-56720	AATCATCATACTCTTTCTCCATGACCC	27	<nnxnp>	nn	235	np	0		
>ref-56727	TAGGCCTCTACAGCCACTCCAGACAAT	24	<lmxll>	ll	15	lm	241		
>ref-56729	CACACCTATACAATCGCTCCCACGCAT	15	<hkxhk>	hh	63	hk	135	kk	62
>ref-56739	CAGATCGTGACGTTCCCTCCTTCGCGG	25	<nnxnp>	nn	125	np	112		

>ref-56754	TTATTATATACCATTTCTCCGTCTGGT	14	<hkxhk>	hh	1	hk	248	kk	1
>ref-56755	CTCATTCTCACCTTCACTCCTACCTCT	4	<lmxll>	ll	113	lm	108		
>ref-56755	CTCATTCTCACCTTCACTCCTACCTCT	23	<lmxll>	ll	113	lm	108		
>ref-56761	AATCTATATACAAGACCTCCATTTGCA	4	<lmxll>	ll	135	lm	94		
>ref-56762	TCCTCAGAAACAGACGCTCCATGCTGA	7	<hkxhk>	hh	0	hk	271	kk	0
>ref-56762	TCCTCAGAAACAGACGCTCCATGCTGA	21	<hkxhk>	hh	0	hk	271	kk	0
>ref-56762	TCCTCAGAAACAGACGCTCCATGCTGA	26	<lmxll>	ll	162	lm	98		
>ref-56775	TCCCCTACCACACAATCTCCTTCACCT	22	<hkxhk>	hh	35	hk	204	kk	1
>ref-56781	TCCTTCTCTACCCTGTCTCCTATTTCT	5	<hkxhk>	hh	4	hk	258	kk	7
>ref-56781	TCCTTCTCTACCCTGTCTCCTATTTCT	22	<hkxhk>	hh	7	hk	258	kk	4
>ref-56783	ATGAAGGGAACGAGAACTCCACCCCTT	3	<hkxhk>	hh	46	hk	161	kk	57
>ref-56785	TTCCCTTCCACCTTCCCTCCTCCTCTG	3	<lmxll>	ll	138	lm	122		
>ref-56803	ACCAGGTGCACCATTCCTCCGAGGACT	5	<lmxll>	ll	17	lm	243		
>ref-56803	ACCAGGTGCACCATTCCTCCGAGGACT	23	<lmxll>	ll	184	lm	75		
>ref-56820	CGCCCACCCACTCATCCTCCCCACACC	1	<nnxnp>	nn	265	np	0		
>ref-56882	TAACCTACAACGGACACTCCCCCAACA	25	<nnxnp>	nn	98	np	128		
>ref-56894	TGGTCACGAACTTGAACTCCAGTATTA	8	<lmxll>	ll	111	lm	108		
>ref-56897	AGAAACCTCACGCACACTCCACACCAG	26	<hkxhk>	hh	0	hk	133	kk	121
>ref-56912	GATAACGCGACGTCTTCTCCTCTCTCT	13	<lmxll>	ll	141	lm	126		
>ref-56946	TCTTTCCGAACCGTTTCTCCCACCTAT	21	<nnxnp>	nn	110	np	152		
>ref-56972	CTCGATTCGACTTCGTCTCCTTGTTTA	3	<lmxll>	ll	117	lm	147		
>ref-56981	TCTTCCCTTACTCCATCTCCTCCCCAT	2	<nnxnp>	nn	267	np	0		
>ref-56989	TCCTCTTCCACTTCATCTCCCCTTCTG	2	<nnxnp>	nn	119	np	146		
>ref-56994	TTCTCCTTCACCGTTTCTCCCTTCCCT	26	<nnxnp>	nn	178	np	72		
>ref-57002	CACCACGCGACTCCATCTCCTCCTCCT	1	<lmxll>	ll	263	lm	0		
>ref-57003	CCGCCGTCGACGTGCGCTCCCTCAGCG	9	<nnxnp>	nn	270	np	0		
>ref-57017	GCTTAACCTACTCCCCCTCCTTTCCTC	27	<nnxnp>	nn	122	np	142		
>ref-57050	AACCAGCGCACAACCGCTCCTGCAAAA	5	<lmxll>	ll	134	lm	136		
>ref-57078	CTTTTCATCACTGCACCTCCTTGCCCC	23	<nnxnp>	nn	264	np	0		
>ref-57093	CCTCATCGTACTCTGTCTCCCCTCTTC	1	<hkxhk>	hh	65	hk	142	kk	52
>ref-57100	TAACTTCTTACTTTCTCTCCTCCCCCC	4	<nnxnp>	nn	140	np	118		
>ref-57108	CGCATGGATACAGACACTCCATATACA	2	<nnxnp>	nn	104	np	121		
>ref-57113	CTTCGTCCTACGCCCTCTCCTCCTCCC	5	<lmxll>	ll	259	lm	0		
>ref-57113	CTTCGTCCTACGCCCTCTCCTCCTCCC	12	<lmxll>	ll	259	lm	0		
>ref-57123	ATACACAGTACATAACCTCCCTATTAC	9	<hkxhk>	hh	46	hk	125	kk	71
>ref-57140	CTGGTCCACACCAGCCCTCCTACCTCG	12	<hkxhk>	hh	0	hk	272	kk	0
>ref-57140	CTGGTCCACACCAGCCCTCCTACCTCG	15	<hkxhk>	hh	0	hk	272	kk	0
>ref-57140	CTGGTCCACACCAGCCCTCCTACCTCG	22	<hkxhk>	hh	0	hk	272	kk	0

>ref-57182	ACTTTGCATACACACTCTCCCTTCTTC	27	<nnxnp>	nn	127	np	128		
>ref-57195	TTGTATCTGACTCTGCCTCCATCTCTG	15	<nnxnp>	nn	134	np	134		
>ref-57196	GAAGAATCAACTCGTCCTCCACCTGAA	23	<hkxhk>	hh	38	hk	213	kk	0
>ref-57197	TTTCCTCTTACCCTTTCTCCGTTTATT	1	<nnxnp>	nn	98	np	138		
>ref-57197	TTTCCTCTTACCCTTTCTCCGTTTATT	13	<nnxnp>	nn	152	np	84		
>ref-57197	TTTCCTCTTACCCTTTCTCCGTTTATT	25	<nnxnp>	nn	97	np	140		
>ref-57202	TAAACCCACACGGCTCCTCCGCCAGGG	22	<lmxll>	ll	130	lm	128		
>ref-57212	CTCTCCTTCACACCCCCTCCTTCCCCC	3	<lmxll>	ll	238	lm	13		
>ref-57212	CTCTCCTTCACACCCCCTCCTTCCCCC	4	<nnxnp>	nn	38	np	204		
>ref-57212	CTCTCCTTCACACCCCCTCCTTCCCCC	16	<lmxll>	ll	261	lm	1		
>ref-57212	CTCTCCTTCACACCCCCTCCTTCCCCC	26	<nnxnp>	nn	36	np	204		
>ref-57238	TCAGCGCACACTTCTCCTCCTGTGATA	7	<nnxnp>	nn	160	np	106		
>ref-57262	AAATCAGCAACAAAATCTCCCCCAAAA	4	<nnxnp>	nn	17	np	216		
>ref-57262	AAATCAGCAACAAAATCTCCCCCAAAA	6	<nnxnp>	nn	17	np	216		
>ref-57262	AAATCAGCAACAAAATCTCCCCCAAAA	21	<nnxnp>	nn	116	np	125		
>ref-57262	AAATCAGCAACAAAATCTCCCCCAAAA	27	<hkxhk>	hh	68	hk	161	kk	5
>ref-57271	TTCTTTTTTACTGTCTCTCCTCTCTTT	22	<lmxll>	ll	160	lm	70		
>ref-57277	CTTACCTGTACCTCCTCTCCGCGTCTA	6	<nnxnp>	nn	127	np	133		
>ref-57277	CTTACCTGTACCTCCTCTCCGCGTCTA	8	<hkxhk>	hh	51	hk	143	kk	58
>ref-57277	CTTACCTGTACCTCCTCTCCGCGTCTA	15	<lmxll>	ll	124	lm	132		
>ref-57278	GAAAGAGAGACGTAACCTCCATTCATT	24	<nnxnp>	nn	158	np	100		
>ref-57285	AATGAAAGCACTGGCACTCCGTCTCCC	2	<nnxnp>	nn	217	np	3		
>ref-57295	GCCCTACCAACTTCCCCTCCTCCTCCT	26	<hkxhk>	hh	49	hk	193	kk	1
>ref-57295	GCCCTACCAACTTCCCCTCCTCCTCCT	27	<hkxhk>	hh	1	hk	195	kk	47
>ref-57308	CCCTCCATTACATTCCCTCCTTTCTCT	1	<hkxhk>	hh	53	hk	118	kk	53
>ref-57316	GCCACTCTGACGTCGTCTCCACCTCTA	15	<hkxhk>	hh	0	hk	272	kk	1
>ref-57316	GCCACTCTGACGTCGTCTCCACCTCTA	27	<lmxll>	ll	98	lm	172		
>ref-57321	CCCACAGCAACGCCTCCTCCACTTCCC	7	<nnxnp>	nn	130	np	140		
>ref-57337	TCCTGTTTTACCTTTACTCCTTATTCT	12	<lmxll>	ll	119	lm	135		
>ref-57355	TCCTCCCTGACCTGGCCTCCAACCTAG	9	<hkxhk>	hh	0	hk	10	kk	262
>ref-57355	TCCTCCCTGACCTGGCCTCCAACCTAG	13	<hkxhk>	hh	0	hk	272	kk	0
>ref-57355	TCCTCCCTGACCTGGCCTCCAACCTAG	15	<nnxnp>	nn	232	np	34		
>ref-57355	TCCTCCCTGACCTGGCCTCCAACCTAG	25	<hkxhk>	hh	0	hk	30	kk	230
>ref-57355	TCCTCCCTGACCTGGCCTCCAACCTAG	26	<hkxhk>	hh	236	hk	32	kk	0
>ref-57360	TACCACCTCACCCTATCTCCCTTTTTC	26	<lmxll>	ll	129	lm	124		
>ref-57372	TTCTCAATCACCTATTCTCCCTCTTCC	21	<hkxhk>	hh	1	hk	198	kk	65
>ref-57392	ACGCCCCCAACAAAGCCTCCAGTACTT	8	<hkxhk>	hh	1	hk	271	kk	0
>ref-57392	ACGCCCCCAACAAAGCCTCCAGTACTT	23	<hkxhk>	hh	0	hk	268	kk	2

>ref-57399	ACCTAATCCACAGAAACTCCTCCACAG	1	<nnxnp>	nn	128	np	108		
>ref-57399	ACCTAATCCACAGAAACTCCTCCACAG	4	<nnxnp>	nn	132	np	106		
>ref-57442	TTAAGGCTTACATTCCCTCCTTTTGTT	16	<hkxhk>	hh	70	hk	139	kk	49
>ref-57458	TTCTGCATTACTCCCTCTCCTCCTCCC	15	<nnxnp>	nn	248	np	0		
>ref-57458	TTCTGCATTACTCCCTCTCCTCCTCCC	22	<hkxhk>	hh	111	hk	80	kk	34
>ref-57460	TATGATAACACTTTAACTCCCTTTTTC	4	<hkxhk>	hh	0	hk	258	kk	8
>ref-57460	TATGATAACACTTTAACTCCCTTTTTC	6	<lmxll>	ll	28	lm	216		
>ref-57460	TATGATAACACTTTAACTCCCTTTTTC	26	<lmxll>	ll	6	lm	246		
>ref-57460	TATGATAACACTTTAACTCCCTTTTTC	27	<nnxnp>	nn	90	np	157		
>ref-57462	AGCGCTCTAACCACTACTCCATCGCGG	3	<nnxnp>	nn	19	np	240		
>ref-57462	AGCGCTCTAACCACTACTCCATCGCGG	16	<nnxnp>	nn	19	np	239		
>ref-57486	TCCCCCCCTACCCTTCCTCCCTCATCA	23	<nnxnp>	nn	89	np	134		
>ref-57486	TCCCCCCCTACCCTTCCTCCCTCATCA	27	<nnxnp>	nn	39	np	190		
>ref-57494	CTCTCTCTGACCTGTTCTCCTTTATTC	3	<hkxhk>	hh	47	hk	125	kk	60
>ref-57499	TCAACAAGCACAAATCCTCCGTCACCA	3	<lmxll>	ll	3	lm	268		
>ref-57499	TCAACAAGCACAAATCCTCCGTCACCA	21	<hkxhk>	hh	0	hk	192	kk	71
>ref-57503	GCCTTTCTTACCATACCTCCTTTTCCT	4	<lmxll>	ll	118	lm	106		
>ref-57517	TGTCCCCCTACCTTCTCTCCCACTCAC	5	<nnxnp>	nn	267	np	0		
>ref-57526	CAACCAAACACCCCCACTCCCATGCCC	21	<lmxll>	ll	115	lm	131		
>ref-57537	CATACCCCTACCATCTCTCCTTCCTCC	4	<lmxll>	ll	58	lm	207		
>ref-57537	CATACCCCTACCATCTCTCCTTCCTCC	25	<hkxhk>	hh	0	hk	207	kk	58
>ref-57538	CCATTGTTAACCCTCTCTCCCTTCCCC	5	<nnxnp>	nn	135	np	131		
>ref-57542	AGGTGAAACACGTCGCCTCCTCCGTTT	24	<lmxll>	ll	121	lm	143		
>ref-57543	CTGCTGTCTACATTATCTCCCTTAGTG	3	<lmxll>	ll	119	lm	126		
>ref-57545	CCCCTCTCCACACCCTCTCCCACCCTC	3	<nnxnp>	nn	141	np	120		
>ref-57545	CCCCTCTCCACACCCTCTCCCACCCTC	25	<nnxnp>	nn	141	np	119		
>ref-57557	CCTTTCTCAACTCGCCCTCCCCCTCCT	5	<nnxnp>	nn	119	np	121		
>ref-57557	CCTTTCTCAACTCGCCCTCCCCCTCCT	21	<nnxnp>	nn	118	np	120		
>ref-57582	ATCCCCCTTACCCTCTCTCCCATCCCC	12	<lmxll>	ll	20	lm	233		
>ref-57582	ATCCCCCTTACCCTCTCTCCCATCCCC	22	<hkxhk>	hh	18	hk	233	kk	0
>ref-57588	TAACGGCCTACGTGAGCTCCCTGTGCC	21	<hkxhk>	hh	49	hk	147	kk	70
>ref-57590	TTCCCTTGTACTCTCTCTCCTTTTCCC	6	<hkxhk>	hh	12	hk	230	kk	1
>ref-57590	TTCCCTTGTACTCTCTCTCCTTTTCCC	24	<hkxhk>	hh	12	hk	230	kk	1
>ref-57595	TAAAAGATCACTCAGACTCCCTACTAT	25	<hkxhk>	hh	0	hk	229	kk	15
>ref-57595	TAAAAGATCACTCAGACTCCCTACTAT	27	<hkxhk>	hh	0	hk	232	kk	20
>ref-57598	TACTCTAGAACACCTCCTCCAGCCCCT	13	<lmxll>	ll	115	lm	137		
>ref-57612	AACAGTCTGACCTCTTCTCCTACTACG	22	<lmxll>	ll	101	lm	134		
>ref-57620	AAGCTTCCAACCAAGTCTCCGCTTAAG	3	<nnxnp>	nn	272	np	0		

>ref-57638	TTCCACGCAACGTATGCTCCAATGATT	7	<lmxll>	ll	132	lm	119		
>ref-57700	TAACGCAGGACTCTCTCTCCCTTATTT	4	<hkxhk>	hh	61	hk	148	kk	57
>ref-57700	TAACGCAGGACTCTCTCTCCCTTATTT	24	<hkxhk>	hh	57	hk	148	kk	61
>ref-57702	ACTCTTCTCACCTTCTCTCCCTACCCC	1	<lmxll>	ll	133	lm	88		
>ref-57702	ACTCTTCTCACCTTCTCTCCCTACCCC	7	<lmxll>	ll	140	lm	86		
>ref-57707	GTTTACCCTACTCTCCCTCCACCTCCC	1	<hkxhk>	hh	0	hk	129	kk	97
>ref-57707	GTTTACCCTACTCTCCCTCCACCTCCC	5	<hkxhk>	hh	98	hk	129	kk	0
>ref-57708	GCCCGGTGAACCCTCTCTCCGTTTTGT	22	<nnxnp>	nn	102	np	122		
>ref-57710	CACCCACCCACGCCCACTCCCTTACAC	24	<lmxll>	ll	6	lm	260		
>ref-57724	TTTATTGAGACACTGTCTCCAGAAGGG	5	<nnxnp>	nn	119	np	130		
>ref-57724	TTTATTGAGACACTGTCTCCAGAAGGG	7	<hkxhk>	hh	0	hk	242	kk	5
>ref-57724	TTTATTGAGACACTGTCTCCAGAAGGG	22	<nnxnp>	nn	78	np	178		
>ref-57724	TTTATTGAGACACTGTCTCCAGAAGGG	25	<nnxnp>	nn	265	np	5		
>ref-57724	TTTATTGAGACACTGTCTCCAGAAGGG	27	<lmxll>	ll	89	lm	159		
>ref-57732	ATAATTACTACTACCGCTCCTACAGCT	8	<nnxnp>	nn	127	np	129		
>ref-57735	CTTAAGACCACACACACTCCATGGACG	4	<nnxnp>	nn	129	np	134		
>ref-57735	CTTAAGACCACACACACTCCATGGACG	13	<nnxnp>	nn	128	np	136		
>ref-57736	CGCTTTTGAACGTGAACTCCTTTATCA	12	<hkxhk>	hh	0	hk	217	kk	39
>ref-57736	CGCTTTTGAACGTGAACTCCTTTATCA	14	<nnxnp>	nn	236	np	25		
>ref-57737	TCCCCCATTACCTTCCCTCCCTTCCAT	21	<lmxll>	ll	30	lm	232		
>ref-57737	TCCCCCATTACCTTCCCTCCCTTCCAT	26	<hkxhk>	hh	0	hk	71	kk	185
>ref-57737	TCCCCCATTACCTTCCCTCCCTTCCAT	27	<lmxll>	ll	256	lm	3		
>ref-57752	ATTCAGCGCACGTGGTCTCCTGCAATG	4	<lmxll>	ll	135	lm	122		
>ref-57754	ATGGCTATTACCCTTCCTCCTCCCTGT	2	<nnxnp>	nn	133	np	122		
>ref-57754	ATGGCTATTACCCTTCCTCCTCCCTGT	14	<lmxll>	ll	127	lm	102		
>ref-57754	ATGGCTATTACCCTTCCTCCTCCCTGT	26	<nnxnp>	nn	132	np	122		
>ref-57757	TATGCCTATACATTCTCTCCCCTTCTT	4	<hkxhk>	hh	58	hk	147	kk	45
>ref-57757	TATGCCTATACATTCTCTCCCCTTCTT	12	<hkxhk>	hh	45	hk	146	kk	57
>ref-57763	CCACCTGCAACTCCTCCTCCCACCCCT	7	<hkxhk>	hh	55	hk	135	kk	65
>ref-57769	CCTCTCTTAACTTTCCCTCCAACCTCT	2	<nnxnp>	nn	115	np	147		
>ref-57772	TGTTACATTACATTTGCTCCGTGTCGC	21	<nnxnp>	nn	116	np	133		
>ref-57780	CAGATATCAACAATCACTCCCTCGTGC	5	<nnxnp>	nn	109	np	157		
>ref-57791	ATGGTTACGACACCGCCTCCTAGAACA	13	<lmxll>	ll	46	lm	213		
>ref-57791	ATGGTTACGACACCGCCTCCTAGAACA	14	<lmxll>	ll	46	lm	213		
>ref-57791	ATGGTTACGACACCGCCTCCTAGAACA	22	<hkxhk>	hh	0	hk	273	kk	0
>ref-57794	GTCTTATGCACAGCTGCTCCCTTTTTG	2	<hkxhk>	hh	0	hk	268	kk	3
>ref-57794	GTCTTATGCACAGCTGCTCCCTTTTTG	26	<hkxhk>	hh	0	hk	273	kk	0
>ref-57804	TCCCCCGCTACTACTGCTCCTGCTACT	1	<nnxnp>	nn	134	np	128		

>ref-57804	TCCCCCGCTACTACTGCTCCTGCTACT	2	<nnxnp>	nn	135	np	127		
>ref-57804	TCCCCCGCTACTACTGCTCCTGCTACT	13	<lmxll>	ll	110	lm	151		
>ref-57819	TAATGGCCTACTCCCTCTCCATACCCT	9	<hkxhk>	hh	0	hk	255	kk	12
>ref-57819	TAATGGCCTACTCCCTCTCCATACCCT	12	<hkxhk>	hh	0	hk	271	kk	1
>ref-57819	TAATGGCCTACTCCCTCTCCATACCCT	22	<hkxhk>	hh	0	hk	239	kk	32
>ref-57827	AGAAAGAGAACAAAAGCTCCCGGCACT	26	<hkxhk>	hh	49	hk	163	kk	53
>ref-57840	CCTCTATCCACACCCTCTCCCCTCACC	14	<lmxll>	ll	101	lm	157		
>ref-57860	TCTAATGTTACGCCGACTCCGATACTC	26	<lmxll>	ll	127	lm	127		
>ref-57865	AAAACCCTCACTCTCTCTCCCTCCCTC	4	<nnxnp>	nn	123	np	131		
>ref-57865	AAAACCCTCACTCTCTCTCCCTCCCTC	22	<nnxnp>	nn	12	np	254		
>ref-57865	AAAACCCTCACTCTCTCTCCCTCCCTC	23	<nnxnp>	nn	145	np	115		
>ref-57865	AAAACCCTCACTCTCTCTCCCTCCCTC	26	<lmxll>	ll	21	lm	235		
>ref-57869	GACCTTTTGACGTCCTCTCCGTGTCCA	4	<lmxll>	ll	134	lm	134		
>ref-57883	CCTCCCACCACCCCTGCTCCCCCTCCT	1	<hkxhk>	hh	1	hk	270	kk	0
>ref-57883	CCTCCCACCACCCCTGCTCCCCCTCCT	16	<hkxhk>	hh	0	hk	240	kk	21
>ref-57887	TCAACTTACACTCTCCCTCCCTCCCTT	23	<hkxhk>	hh	43	hk	128	kk	54
>ref-57888	CACATATATACACCTCCTCCCCTCCAC	1	<nnxnp>	nn	262	np	0		
>ref-57889	GCAGCTGGGACAGAGTCTCCTCGCAGT	5	<lmxll>	ll	141	lm	129		
>ref-57926	CAGATGATTACAGAGTCTCCACAAGGC	7	<lmxll>	ll	106	lm	115		
>ref-57945	TTGTCTTACACTTCCCCTCCCACCATC	14	<lmxll>	ll	115	lm	145		
>ref-57956	TGGGATGAAACAAGCCCTCCTGGAGTA	16	<lmxll>	ll	216	lm	35		
>ref-57956	TGGGATGAAACAAGCCCTCCTGGAGTA	22	<nnxnp>	nn	176	np	80		
>ref-57960	TTCACCTCCACTCTCTCTCCAACACTT	21	<lmxll>	ll	53	lm	212		
>ref-57963	GGAAACAGGACATTCTCTCCTCTCTCT	9	<lmxll>	ll	121	lm	143		
>ref-57963	GGAAACAGGACATTCTCTCCTCTCTCT	15	<lmxll>	ll	144	lm	119		
>ref-57965	TCCCTCCATACTGCTCCTCCACCCCCC	22	<nnxnp>	nn	129	np	133		
>ref-57967	CCCGTTCAGACGCGGCCTCCACCCTCC	8	<nnxnp>	nn	138	np	133		
>ref-57973	AGACTTATGACACTGGCTCCACGTGTC	21	<nnxnp>	nn	271	np	1		
>ref-57994	CACACATACACAACACCTCCCTTTCCT	15	<nnxnp>	nn	123	np	142		
>ref-57995	TCACCCGCGACCTAGCCTCCGGGCTAG	27	<lmxll>	ll	136	lm	130		
>ref-58008	CGGGGTAGAACGAGGTCTCCGATACAT	26	<hkxhk>	hh	53	hk	156	kk	60
>ref-58029	AACGCGTACACGCATCCTCCCCACACA	22	<nnxnp>	nn	116	np	153		
>ref-58059	TTACCCTAAACCTCCTCTCCCTTACCC	6	<hkxhk>	hh	85	hk	176	kk	0
>ref-58059	TTACCCTAAACCTCCTCTCCCTTACCC	13	<hkxhk>	hh	0	hk	267	kk	2
>ref-58059	TTACCCTAAACCTCCTCTCCCTTACCC	14	<hkxhk>	hh	14	hk	244	kk	0
>ref-58059	TTACCCTAAACCTCCTCTCCCTTACCC	15	<hkxhk>	hh	2	hk	267	kk	0
>ref-58096	TCCTCATTTACTCTCTCTCCTACATCT	1	<hkxhk>	hh	0	hk	271	kk	1
>ref-58096	TCCTCATTTACTCTCTCTCCTACATCT	21	<hkxhk>	hh	0	hk	271	kk	1

>ref-58102	TTACTCATCACTCATTCTCCCGTCATT	22	<lmxll>	ll	146	lm	124		
>ref-58185	CGTTTATCTACGTTCTCTCCCCATCTT	1	<lmxll>	ll	132	lm	128		
>ref-58185	CGTTTATCTACGTTCTCTCCCCATCTT	2	<hkxhk>	hh	57	hk	145	kk	56
>ref-58192	TAATCACGCACAGCCACTCCACTAAAA	7	<nnxnp>	nn	127	np	132		
>ref-58197	CTGATTGATACACAACCTCCTCGCGCG	6	<hkxhk>	hh	60	hk	142	kk	54
>ref-58238	TTATGAAACACTTCATCTCCTCCTTTG	12	<hkxhk>	hh	51	hk	148	kk	61
>ref-58240	CAACTTTCCACACCACCTCCCTCGTGA	22	<nnxnp>	nn	251	np	0		
>ref-58244	GTGTCCTGTACGCGCCCTCCTGATCGT	2	<nnxnp>	nn	121	np	133		
>ref-58244	GTGTCCTGTACGCGCCCTCCTGATCGT	22	<hkxhk>	hh	53	hk	146	kk	49
>ref-58244	GTGTCCTGTACGCGCCCTCCTGATCGT	23	<hkxhk>	hh	49	hk	146	kk	53
>ref-58263	TCTCTGGCCACTCCCACTCCGCTCAAG	24	<nnxnp>	nn	152	np	120		
>ref-58280	TACTTTTGTACTGCTACTCCTGTGGTT	24	<lmxll>	ll	114	lm	153		
>ref-58309	TCCAGCATGACCCATTCTCCATACGTA	26	<nnxnp>	nn	123	np	145		
>ref-58347	GTATTGTGGACCCCGTCTCCGACGGTG	12	<lmxll>	ll	212	lm	47		
>ref-58351	AACAAGCGAACCCGTGCTCCTCCTCCA	2	<nnxnp>	nn	124	np	140		
>ref-58351	AACAAGCGAACCCGTGCTCCTCCTCCA	3	<nnxnp>	nn	124	np	140		
>ref-58354	CTATCCCCCACCTCTCCTCCTTCTCTA	23	<nnxnp>	nn	108	np	141		
>ref-58354	CTATCCCCCACCTCTCCTCCTTCTCTA	25	<nnxnp>	nn	135	np	111		
>ref-58387	GGCGAGTGGACGTGCTCTCCTCATCAT	24	<nnxnp>	nn	267	np	0		
>ref-58387	GGCGAGTGGACGTGCTCTCCTCATCAT	25	<nnxnp>	nn	267	np	0		
>ref-58406	GTCTACTTTACCCTCTCTCCTTCTCCT	3	<hkxhk>	hh	48	hk	133	kk	69
>ref-58406	GTCTACTTTACCCTCTCTCCTTCTCCT	22	<hkxhk>	hh	70	hk	133	kk	46
>ref-58416	CTGCCATGCACACAGACTCCTATCTCT	4	<nnxnp>	nn	270	np	0		
>ref-58427	GACCTGGGCACGGTGACTCCTGGGAGC	4	<hkxhk>	hh	0	hk	273	kk	0
>ref-58427	GACCTGGGCACGGTGACTCCTGGGAGC	8	<hkxhk>	hh	0	hk	272	kk	0
>ref-58427	GACCTGGGCACGGTGACTCCTGGGAGC	27	<hkxhk>	hh	0	hk	273	kk	0
>ref-58440	TCCCCCTCTACTCTCCCTCCAATGGCA	2	<hkxhk>	hh	57	hk	136	kk	63
>ref-58442	TGGGCCCCAACACCCGCTCCTATTTTC	27	<lmxll>	ll	182	lm	66		
>ref-58510	CGCCCTTCCACCCACACTCCTCCCCAC	24	<nnxnp>	nn	121	np	143		
>ref-58510	CGCCCTTCCACCCACACTCCTCCCCAC	26	<nnxnp>	nn	123	np	143		
>ref-58515	ACTTTCCTCACTCTCCCTCCTTTCGTC	1	<hkxhk>	hh	1	hk	259	kk	4
>ref-58515	ACTTTCCTCACTCTCCCTCCTTTCGTC	2	<hkxhk>	hh	1	hk	259	kk	4
>ref-58515	ACTTTCCTCACTCTCCCTCCTTTCGTC	9	<lmxll>	ll	10	lm	251		
>ref-58515	ACTTTCCTCACTCTCCCTCCTTTCGTC	25	<lmxll>	ll	8	lm	253		
>ref-58533	CCTAAATGCACTTACCCTCCTTCTCTG	22	<lmxll>	ll	129	lm	130		
>ref-58533	CCTAAATGCACTTACCCTCCTTCTCTG	25	<lmxll>	ll	129	lm	130		
>ref-58536	CCATCGCCCACAGCCTCTCCTCCTTCT	7	<hkxhk>	hh	56	hk	145	kk	59
>ref-58555	CTCACCCTCACCCCGACTCCCACTTCA	5	<lmxll>	ll	113	lm	146		

>ref-58562	GACAGCAGGACCCAACCTCCCTTTCCC	12	<nnxnp>	nn	131	np	139		
>ref-58568	ATTTCACTGACCGAAACTCCTCGAATC	13	<lmxll>	ll	134	lm	108		
>ref-58578	CTGTACTCTACACACCCTCCCCCTCCC	1	<lmxll>	ll	3	lm	266		
>ref-58578	CTGTACTCTACACACCCTCCCCCTCCC	4	<lmxll>	ll	3	lm	266		
>ref-58605	TCCATAGCCACCCTTTCTCCTTCCTTT	2	<nnxnp>	nn	132	np	132		
>ref-58618	TCTCCCTACACCCCCCCTCCGACCATC	21	<nnxnp>	nn	133	np	127		
>ref-58618	TCTCCCTACACCCCCCCTCCGACCATC	27	<nnxnp>	nn	132	np	127		
>ref-58631	GACACCATGACCGCTGCTCCGAGCTAT	22	<lmxll>	ll	116	lm	152		
>ref-58639	GGACCTTCTACTCTTGCTCCTCCTCCT	3	<lmxll>	ll	262	lm	0		
>ref-58639	GGACCTTCTACTCTTGCTCCTCCTCCT	16	<lmxll>	ll	213	lm	19		
>ref-58650	TTTTCCCCTACCCTTTCTCCGCTCTTC	2	<hkxhk>	hh	19	hk	219	kk	3
>ref-58650	TTTTCCCCTACCCTTTCTCCGCTCTTC	6	<hkxhk>	hh	246	hk	6	kk	0
>ref-58650	TTTTCCCCTACCCTTTCTCCGCTCTTC	9	<hkxhk>	hh	0	hk	7	kk	245
>ref-58650	TTTTCCCCTACCCTTTCTCCGCTCTTC	21	<hkxhk>	hh	19	hk	220	kk	2
>ref-58653	CTCCTACCAACTCCCTCTCCCTTTCCC	5	<nnxnp>	nn	150	np	105		
>ref-58653	CTCCTACCAACTCCCTCTCCCTTTCCC	6	<nnxnp>	nn	130	np	115		
>ref-58678	GCTCCTTCCACCTTTGCTCCTTCTCTC	15	<lmxll>	ll	133	lm	128		
>ref-58678	GCTCCTTCCACCTTTGCTCCTTCTCTC	21	<lmxll>	ll	133	lm	128		
>ref-58688	CAAAGGCCAACAAAGCCTCCACTCCTT	24	<lmxll>	ll	155	lm	116		
>ref-58700	ATCGGGCGTACGTGCGCTCCTGGCAGT	9	<nnxnp>	nn	267	np	1		
>ref-58719	TCACGTCCAACTCCTCCTCCTCCCATT	7	<hkxhk>	hh	2	hk	270	kk	1
>ref-58719	TCACGTCCAACTCCTCCTCCTCCCATT	14	<lmxll>	ll	130	lm	140		
>ref-58719	TCACGTCCAACTCCTCCTCCTCCCATT	25	<hkxhk>	hh	1	hk	270	kk	1
>ref-58728	CTCCCTCCAACACCCTCTCCCTCCTCC	7	<lmxll>	ll	180	lm	74		
>ref-58728	CTCCCTCCAACACCCTCTCCCTCCTCC	8	<lmxll>	ll	178	lm	74		
>ref-58729	TGCAAGTTCACTGGTGCTCCCAAATAT	13	<nnxnp>	nn	128	np	108		
>ref-58731	CACACACACACAACAGCTCCATATCAC	23	<hkxhk>	hh	62	hk	138	kk	59
>ref-58749	TGGGTAGAAACAAACCCTCCTCCTCAT	26	<hkxhk>	hh	0	hk	257	kk	9
>ref-58755	GCGTCTTTCACTATTTCTCCTTTCTCT	2	<nnxnp>	nn	112	np	146		
>ref-58755	GCGTCTTTCACTATTTCTCCTTTCTCT	15	<nnxnp>	nn	143	np	121		
>ref-58757	GCACTTCTAACATATCCTCCCGTCCCA	8	<nnxnp>	nn	113	np	138		
>ref-58757	GCACTTCTAACATATCCTCCCGTCCCA	14	<hkxhk>	hh	45	hk	128	kk	70
>ref-58759	CAAGTGGATACATGTCCTCCGTGTGTT	8	<nnxnp>	nn	134	np	135		
>ref-58767	CAACACTGAACGCTGGCTCCTTCTGTC	8	<lmxll>	ll	123	lm	146		
>ref-58826	ACTTCGCACACTCCCTCTCCCTCCCTC	3	<nnxnp>	nn	141	np	123		
>ref-58826	ACTTCGCACACTCCCTCTCCCTCCCTC	4	<nnxnp>	nn	7	np	263		
>ref-58826	ACTTCGCACACTCCCTCTCCCTCCCTC	8	<nnxnp>	nn	133	np	126		
>ref-58826	ACTTCGCACACTCCCTCTCCCTCCCTC	14	<nnxnp>	nn	7	np	263		

>ref-58843	GGCAGACTAACCTAACCTCCATCTATT	21	<lmxll>	ll	106	lm	145		
>ref-58867	TATACTTACACACAGCCTCCATGCAGT	15	<hkxhk>	hh	58	hk	146	kk	57
>ref-58878	ATTTATAGGACGGCTTCTCCTTAGAAT	8	<nnxnp>	nn	137	np	102		
>ref-58879	GTACCAGGTACTGTACCTCCCTTCACA	4	<lmxll>	ll	110	lm	145		
>ref-58933	CTGCGCAACACCGGCTCTCCGTGTTTA	27	<hkxhk>	hh	59	hk	145	kk	61
>ref-58942	ATGTATGACACGACACCTCCCACTCTG	27	<nnxnp>	nn	135	np	133		
>ref-58946	TCCAACAGCACATCCACTCCTTCCAGC	5	<lmxll>	ll	122	lm	133		
>ref-58957	TTTTCATTCACCCTTTCTCCTCTACTT	6	<lmxll>	ll	116	lm	129		
>ref-58957	TTTTCATTCACCCTTTCTCCTCTACTT	12	<lmxll>	ll	114	lm	130		
>ref-58967	TTCTTCTTTACATTGTCTCCTCTCTCC	6	<lmxll>	ll	144	lm	117		
>ref-58976	TAAGAGAACACTATCTCTCCTACACTC	4	<lmxll>	ll	133	lm	138		
>ref-58977	CATCACCCGACCCTCACTCCTTGAATG	1	<nnxnp>	nn	260	np	0		
>ref-58977	CATCACCCGACCCTCACTCCTTGAATG	2	<nnxnp>	nn	259	np	0		
>ref-59013	TTTTCTCTAACTTCTTCTCCCTCTCTC	26	<nnxnp>	nn	72	np	150		
>ref-59015	TACCCTCCAACTCATTCTCCTTCCTTC	9	<hkxhk>	hh	1	hk	266	kk	0
>ref-59015	TACCCTCCAACTCATTCTCCTTCCTTC	21	<hkxhk>	hh	0	hk	267	kk	1
>ref-59024	GCTACTCAGACTCAGTCTCCCTATATG	1	<hkxhk>	hh	56	hk	135	kk	67
>ref-59029	TTGAAGAGTACAGAGTCTCCGGCTTGT	9	<nnxnp>	nn	237	np	0		
>ref-59046	ACCTCCGAAACCACTACTCCCTCCGAG	14	<hkxhk>	hh	1	hk	212	kk	9
>ref-59046	ACCTCCGAAACCACTACTCCCTCCGAG	25	<nnxnp>	nn	142	np	83		
>ref-59048	AAAAGAGGCACCAGGCCTCCCATGCAC	7	<lmxll>	ll	132	lm	123		
>ref-59048	AAAAGAGGCACCAGGCCTCCCATGCAC	14	<lmxll>	ll	52	lm	208		
>ref-59048	AAAAGAGGCACCAGGCCTCCCATGCAC	24	<lmxll>	ll	19	lm	241		
>ref-59066	GGTGGCACCACAGGCTCTCCCGCGGTG	6	<lmxll>	ll	155	lm	118		
>ref-59077	GCTTATCAAACTGTATCTCCACTCATC	13	<hkxhk>	hh	44	hk	147	kk	69
>ref-59089	CTTGGGCTGACAATTTCTCCCCGCCTG	4	<lmxll>	ll	125	lm	118		
>ref-59092	AAATGAGACACAAAGGCTCCCTCACAA	25	<hkxhk>	hh	63	hk	103	kk	57
>ref-59096	AAATCTCTCACCTGAACTCCTCTCCAT	24	<lmxll>	ll	77	lm	174		
>ref-59096	AAATCTCTCACCTGAACTCCTCTCCAT	26	<hkxhk>	hh	2	hk	259	kk	2
>ref-59109	CCTCCTCGTACCTGACCTCCCAAATAT	14	<nnxnp>	nn	131	np	113		
>ref-59110	CTCGCTCTGACACCTTCTCCAGAGCCA	3	<nnxnp>	nn	105	np	161		
>ref-59147	CGTTCTGTTACTCTCTCTCCCTCTTCT	25	<lmxll>	ll	260	lm	1		
>ref-59149	CTATAATTAACTGCATCTCCTCCCCCC	27	<nnxnp>	nn	115	np	109		
>ref-59165	TTTATCTCTACCTTCTCTCCTTTTCTC	15	<lmxll>	ll	185	lm	61		
>ref-59176	CTCACGAACACCTTCCCTCCCCCCTCT	6	<hkxhk>	hh	0	hk	272	kk	0
>ref-59176	CTCACGAACACCTTCCCTCCCCCCTCT	8	<hkxhk>	hh	0	hk	272	kk	0
>ref-59178	TATTTTCTTACTCATCCTCCTCTTCTC	22	<hkxhk>	hh	53	hk	122	kk	48
>ref-59213	TTGTATGTGACGTGGCCTCCGCGGCTG	27	<lmxll>	ll	122	lm	144		

>ref-59218	GACTTACCTACTGGGTCTCCGTGGCCT	9	<hkxhk>	hh	76	hk	157	kk	34
>ref-59226	AGTACACACACATGTCCTCCCGTGGTC	6	<lmxll>	ll	132	lm	140		
>ref-59243	GAAACCATCACACAGCCTCCTTGATTG	16	<lmxll>	ll	21	lm	243		
>ref-59243	GAAACCATCACACAGCCTCCTTGATTG	21	<lmxll>	ll	22	lm	243		
>ref-59282	ACTGAGATAACTACAACTCCTGAGATA	1	<lmxll>	ll	119	lm	143		
>ref-59282	ACTGAGATAACTACAACTCCTGAGATA	3	<hkxhk>	hh	0	hk	273	kk	0
>ref-59282	ACTGAGATAACTACAACTCCTGAGATA	13	<hkxhk>	hh	19	hk	251	kk	0
>ref-59282	ACTGAGATAACTACAACTCCTGAGATA	21	<hkxhk>	hh	0	hk	271	kk	2
>ref-59282	ACTGAGATAACTACAACTCCTGAGATA	24	<hkxhk>	hh	0	hk	273	kk	0
>ref-59284	TAGACTTTTACAGCTCCTCCTTCCGTT	13	<nnxnp>	nn	120	np	140		
>ref-59289	TGACGTCTTACGCCGCCTCCAACTGCC	12	<nnxnp>	nn	140	np	129		
>ref-59306	GACACAACAACAACGCCTCCAAATCCT	1	<lmxll>	ll	121	lm	144		
>ref-59310	GTCTACAGAACCGCTTCTCCTTGCGTA	15	<hkxhk>	hh	57	hk	143	kk	69
>ref-59311	GTACTTTTCACGAAAACTCCGTGGAGC	1	<hkxhk>	hh	0	hk	50	kk	205
>ref-59311	GTACTTTTCACGAAAACTCCGTGGAGC	22	<hkxhk>	hh	0	hk	272	kk	0
>ref-59329	AAACTGATTACTTGTTCTCCTTATAGA	14	<lmxll>	ll	124	lm	135		
>ref-59332	TGCACCGTAACAAACCCTCCCCCATAC	6	<nnxnp>	nn	121	np	109		
>ref-59349	TGAATAAGAACTCACTCTCCTTTTTCC	14	<nnxnp>	nn	99	np	130		
>ref-59355	CCTTCTCAAACATCACCTCCCTCTCCC	13	<nnxnp>	nn	131	np	128		
>ref-59370	ATGCGCACTACTGTCACTCCTTTCCAC	23	<hkxhk>	hh	0	hk	251	kk	7
>ref-59379	AACATCCTCACAGAATCTCCCACTTTT	1	<hkxhk>	hh	2	hk	247	kk	6
>ref-59379	AACATCCTCACAGAATCTCCCACTTTT	8	<hkxhk>	hh	5	hk	248	kk	3
>ref-59442	TCTCTCTCCACTAGTTCTCCCTCTCCT	13	<lmxll>	ll	113	lm	144		
>ref-59465	CTCTTGAAAACTCTCCCTCCCTCTCTC	24	<hkxhk>	hh	0	hk	270	kk	0
>ref-59465	CTCTTGAAAACTCTCCCTCCCTCTCTC	27	<hkxhk>	hh	0	hk	270	kk	0
>ref-59467	CCCTACCCTACCTTCCCTCCCCCTACC	2	<nnxnp>	nn	99	np	139		
>ref-59467	CCCTACCCTACCTTCCCTCCCCCTACC	9	<nnxnp>	nn	101	np	137		
>ref-59477	AGCGATGCAACGGGAGCTCCCTAGGGT	24	<lmxll>	ll	138	lm	132		
>ref-59477	AGCGATGCAACGGGAGCTCCCTAGGGT	25	<lmxll>	ll	138	lm	132		
>ref-59502	TTAAACATCACCCTCTCTCCACCCGAC	25	<hkxhk>	hh	33	hk	208	kk	5
>ref-59502	TTAAACATCACCCTCTCTCCACCCGAC	26	<hkxhk>	hh	5	hk	208	kk	32
>ref-59524	GGGCTGTAAACATGGCCTCCTTCTAGC	16	<nnxnp>	nn	122	np	138		
>ref-59544	GACGTCAGAACATCATCTCCATATACG	22	<nnxnp>	nn	148	np	103		
>ref-59553	GAAGAGTTTACAGACACTCCTAACCGT	2	<lmxll>	ll	120	lm	141		
>ref-59556	AACAAACCGACACAATCTCCTTAGACA	5	<hkxhk>	hh	43	hk	129	kk	52
>ref-59562	ACTTCCTCCACTCCTGCTCCTTACCCC	12	<nnxnp>	nn	259	np	0		
>ref-59594	ATCTGCATCACCATGTCTCCGTTGTCG	9	<lmxll>	ll	99	lm	162		
>ref-59633	CGTTGCTTGACCTCTCCTCCACTAAAT	4	<lmxll>	ll	119	lm	120		

>ref-59633	CGTTGCTTGACCTCTCCTCCACTAAAT	6	<hkxhk>	hh	59	hk	132	kk	47
>ref-59648	CACGCTCTCACTTCCTCTCCTTCTACT	14	<lmxll>	ll	44	lm	211		
>ref-59648	CACGCTCTCACTTCCTCTCCTTCTACT	25	<lmxll>	ll	43	lm	211		
>ref-59654	AAGTTCGATACCGCCCCTCCGACTCTG	23	<lmxll>	ll	122	lm	144		
>ref-59660	TTCCTTCTTACTCCTACTCCTCTTCTC	3	<lmxll>	ll	104	lm	134		
>ref-59660	TTCCTTCTTACTCCTACTCCTCTTCTC	13	<lmxll>	ll	107	lm	132		
>ref-59668	ACCCTTTTGACTCATCCTCCCGTTCTC	16	<lmxll>	ll	117	lm	145		
>ref-59680	CACGACCACACGCTCGCTCCGCACTCC	23	<lmxll>	ll	112	lm	150		
>ref-59692	CGCACTCGTACTACTACTCCCACTCGT	1	<nnxnp>	nn	169	np	99		
>ref-59692	CGCACTCGTACTACTACTCCCACTCGT	6	<hkxhk>	hh	0	hk	273	kk	0
>ref-59692	CGCACTCGTACTACTACTCCCACTCGT	12	<nnxnp>	nn	171	np	98		
>ref-59692	CGCACTCGTACTACTACTCCCACTCGT	16	<lmxll>	ll	195	lm	71		
>ref-59692	CGCACTCGTACTACTACTCCCACTCGT	26	<hkxhk>	hh	0	hk	162	kk	99
>ref-59712	TCACAGCAGACAGCAGCTCCACCATAT	13	<hkxhk>	hh	0	hk	264	kk	4
>ref-59712	TCACAGCAGACAGCAGCTCCACCATAT	27	<hkxhk>	hh	1	hk	266	kk	0
>ref-59715	ATTCTTTCCACCCGACCTCCAGAATCA	8	<lmxll>	ll	118	lm	125		
>ref-59739	AATGTGGGGACTCTTTCTCCTTCTCCT	24	<lmxll>	ll	117	lm	142		
>ref-59764	CACCACATCACCCTTACTCCACCACAT	15	<lmxll>	ll	110	lm	147		
>ref-59770	TCATGGCCTACATTGTCTCCCGCGAGG	15	<nnxnp>	nn	137	np	108		
>ref-59789	AAGCCCCATACCTCCCCTCCCGCCCCC	1	<hkxhk>	hh	0	hk	273	kk	0
>ref-59789	AAGCCCCATACCTCCCCTCCCGCCCCC	15	<hkxhk>	hh	0	hk	273	kk	0
>ref-59789	AAGCCCCATACCTCCCCTCCCGCCCCC	22	<hkxhk>	hh	123	hk	130	kk	0
>ref-59789	AAGCCCCATACCTCCCCTCCCGCCCCC	25	<hkxhk>	hh	2	hk	270	kk	0
>ref-59794	AGCCATTTCACTTTCCCTCCTCGAACC	3	<nnxnp>	nn	129	np	129		
>ref-59796	CACACACACACACGAGCTCCTCGAAAA	13	<nnxnp>	nn	111	np	127		
>ref-59827	GCTCTCCTTACCGCCCCTCCTCCCTCT	24	<lmxll>	ll	126	lm	145		
>ref-59828	ACTGAAAAGACTCTCCCTCCTGTTTTG	3	<lmxll>	ll	142	lm	95		
>ref-59828	ACTGAAAAGACTCTCCCTCCTGTTTTG	16	<lmxll>	ll	96	lm	145		
>ref-59837	TAGTTTAACACTTTCGCTCCCCTTCCC	25	<lmxll>	ll	138	lm	109		
>ref-59848	TCTGTATCCACCTATTCTCCTTCTCAT	22	<nnxnp>	nn	122	np	131		
>ref-59856	CCCCCTCCTACTCCTGCTCCCTTGCCG	16	<nnxnp>	nn	136	np	124		
>ref-59867	CTCCTCATCACCATCTCTCCCTCATCT	4	<lmxll>	ll	139	lm	127		
>ref-59873	ATCAGTTACACCATTCCTCCCTCTGTG	2	<lmxll>	ll	123	lm	133		
>ref-59874	GCACAGTGAACTATAACTCCTCCCCCT	3	<nnxnp>	nn	10	np	250		
>ref-59879	CCCGGGTACACTCGTCCTCCTTCAGAC	2	<hkxhk>	hh	0	hk	268	kk	4
>ref-59879	CCCGGGTACACTCGTCCTCCTTCAGAC	14	<hkxhk>	hh	0	hk	269	kk	3
>ref-59879	CCCGGGTACACTCGTCCTCCTTCAGAC	21	<hkxhk>	hh	1	hk	271	kk	0
>ref-59879	CCCGGGTACACTCGTCCTCCTTCAGAC	23	<nnxnp>	nn	245	np	24		

>ref-59879	CCCGGGTACACTCGTCCTCCTTCAGAC	25	<hkxhk>	hh	0	hk	232	kk	27
>ref-59879	CCCGGGTACACTCGTCCTCCTTCAGAC	26	<lmxll>	ll	0	lm	273		
>ref-59889	TTCCGTGGCACGACGCCTCCAGACGAT	27	<lmxll>	ll	122	lm	148		
>ref-59905	CATACATATACCCCCTCTCCTCTCCCC	9	<nnxnp>	nn	239	np	0		
>ref-59930	TGTCGGTTCACTCTCTCTCCCTCCTTC	23	<lmxll>	ll	264	lm	0		
>ref-59964	TCACCCTGCACCCTATCTCCAATCCCT	12	<lmxll>	ll	122	lm	134		
>ref-59975	CTGCATATCACCTGTTCTCCACCAGGA	26	<nnxnp>	nn	137	np	118		
>ref-59975	CTGCATATCACCTGTTCTCCACCAGGA	27	<nnxnp>	nn	138	np	119		
>ref-59982	TTCCTTCCTACTTCACCTCCCCCGTAT	15	<lmxll>	ll	10	lm	260		
>ref-59982	TTCCTTCCTACTTCACCTCCCCCGTAT	24	<lmxll>	ll	9	lm	261		
>ref-59986	CTCTCGAGAACAAGGACTCCTCGAGGG	5	<hkxhk>	hh	1	hk	266	kk	3
>ref-59996	TCTATCTTCACCTCCTCTCCCCTCTAT	26	<nnxnp>	nn	116	np	154		
>ref-60001	GAGAAAGGCACCCTCGCTCCTCCTCCT	26	<hkxhk>	hh	60	hk	128	kk	74
>ref-60021	TTCACTCCTACGCCTCCTCCTCATTCA	8	<nnxnp>	nn	27	np	232		
>ref-60021	TTCACTCCTACGCCTCCTCCTCATTCA	16	<nnxnp>	nn	5	np	264		
>ref-60032	GTCATTATTACTCCTCCTCCTACTACT	24	<lmxll>	ll	114	lm	121		
>ref-60033	TTATGAAGTACAGTCTCTCCCTCTCCC	9	<lmxll>	ll	247	lm	0		
>ref-60041	TCCCGCCCAACCAACTCTCCTTCTCAA	15	<nnxnp>	nn	125	np	128		
>ref-60052	TATTCTCTTACTTCATCTCCCACACCT	21	<lmxll>	ll	130	lm	127		
>ref-60065	TGTTCTTAGACGAGTTCTCCGATCACC	21	<nnxnp>	nn	37	np	224		
>ref-60077	CATCCTCTCACCATCTCTCCACCACAG	16	<nnxnp>	nn	125	np	139		
>ref-60083	TTATAAGATACTCATCCTCCAGATTAT	3	<lmxll>	ll	114	lm	131		
>ref-60092	AAAGGAATTACAAGCCCTCCAGTTTGT	26	<nnxnp>	nn	242	np	0		
>ref-60094	CTCCTTCGGACATATACTCCTTGATGT	5	<nnxnp>	nn	141	np	122		
>ref-60104	CAGTACTACACTGTTCCTCCCGAAGAA	6	<nnxnp>	nn	270	np	0		
>ref-60104	CAGTACTACACTGTTCCTCCCGAAGAA	9	<nnxnp>	nn	269	np	0		
>ref-60125	TACTCCCTCACTCCCTCTCCTCCTGAC	25	<lmxll>	ll	260	lm	1		
>ref-60126	ACCGGAAACACGCCTGCTCCGTCAAAA	14	<nnxnp>	nn	121	np	113		
>ref-60140	TTCTCTTTGACATCTGCTCCCTATCAT	9	<lmxll>	ll	137	lm	123		
>ref-60162	TTCCCCCTCACACCATCTCCCTCCCCT	3	<lmxll>	ll	38	lm	220		
>ref-60162	TTCCCCCTCACACCATCTCCCTCCCCT	8	<lmxll>	ll	37	lm	221		
>ref-60188	TCCTCTCCTACTTCTCCTCCACCTTGT	21	<nnxnp>	nn	263	np	0		
>ref-60202	ACCTTCGTAACTTCCACTCCTTGCATA	5	<hkxhk>	hh	0	hk	186	kk	74
>ref-60202	ACCTTCGTAACTTCCACTCCTTGCATA	7	<nnxnp>	nn	233	np	33		
>ref-60202	ACCTTCGTAACTTCCACTCCTTGCATA	21	<lmxll>	ll	44	lm	214		
>ref-60217	CTAGAATATACCAGCACTCCCTGCAAG	8	<nnxnp>	nn	98	np	135		
>ref-60222	GATCCCCTAACATGTTCTCCAAGTCCT	2	<hkxhk>	hh	1	hk	271	kk	0
>ref-60224	GTATCTGTCACTTTTTCTCCCTCTTGA	27	<hkxhk>	hh	0	hk	130	kk	124

>ref-60243	CCACGTCACACCCTTCCTCCCCTCCCA	26	<lmxll>	ll	118	lm	106		
>ref-60267	CCCCGTAAAACCGAATCTCCAATTCCC	23	<nnxnp>	nn	117	np	138		
>ref-60268	ATAGTTGCAACAACAACTCCACCCATA	13	<lmxll>	ll	112	lm	142		
>ref-60277	ATATCCACCACATAGTCTCCATATATC	4	<lmxll>	ll	4	lm	263		
>ref-60277	ATATCCACCACATAGTCTCCATATATC	24	<hkxhk>	hh	0	hk	2	kk	268
>ref-60277	ATATCCACCACATAGTCTCCATATATC	26	<lmxll>	ll	199	lm	55		
>ref-60286	CCCCCCCTTACTCCTTCTCCTTTTCCC	1	<nnxnp>	nn	264	np	0		
>ref-60338	TAGTCCAACACCAACCCTCCCCTCTTC	3	<nnxnp>	nn	120	np	125		
>ref-60374	ACCCCGTCGACTGCGGCTCCTTCTACA	9	<lmxll>	ll	218	lm	1		
>ref-60379	AGACAGTAGACCTTGACTCCCTGGTCA	21	<nnxnp>	nn	136	np	130		
>ref-60380	CTTCATCGTACAGATTCTCCCCCACTT	23	<lmxll>	ll	132	lm	112		
>ref-60380	CTTCATCGTACAGATTCTCCCCCACTT	25	<nnxnp>	nn	121	np	122		
>ref-60385	CTTCCCTTCACTCCCCCTCCCCGCACT	1	<nnxnp>	nn	266	np	1		
>ref-60385	CTTCCCTTCACTCCCCCTCCCCGCACT	8	<hkxhk>	hh	0	hk	227	kk	19
>ref-60385	CTTCCCTTCACTCCCCCTCCCCGCACT	23	<hkxhk>	hh	0	hk	229	kk	15
>ref-60395	CAATAAACGACGAGCCCTCCATGTGTT	8	<hkxhk>	hh	0	hk	259	kk	0
>ref-60395	CAATAAACGACGAGCCCTCCATGTGTT	14	<lmxll>	ll	142	lm	106		
>ref-60395	CAATAAACGACGAGCCCTCCATGTGTT	15	<lmxll>	ll	155	lm	89		
>ref-60395	CAATAAACGACGAGCCCTCCATGTGTT	16	<hkxhk>	hh	10	hk	241	kk	0
>ref-60397	TGTCCCTATACAACCGCTCCTGTACAC	26	<nnxnp>	nn	130	np	129		
>ref-60406	ATTATTCCGACCTTTACTCCAGGCAGA	2	<lmxll>	ll	133	lm	107		
>ref-60406	ATTATTCCGACCTTTACTCCAGGCAGA	23	<lmxll>	ll	132	lm	108		
>ref-60415	CCACTCCAGACTCCCACTCCTTAATAC	26	<lmxll>	ll	114	lm	122		
>ref-60416	TCCTTCACTACGTCCCCTCCTCTTCCT	15	<lmxll>	ll	91	lm	160		
>ref-60419	AGATGCCCAACAGATCCTCCGCAAACA	4	<hkxhk>	hh	58	hk	153	kk	58
>ref-60468	GCTTTGGCCACAGTCTCTCCTCTCCCG	13	<nnxnp>	nn	116	np	126		
>ref-60468	GCTTTGGCCACAGTCTCTCCTCTCCCG	25	<nnxnp>	nn	115	np	126		
>ref-60476	CCCTATTTTACATTCTCTCCTCCATCA	6	<lmxll>	ll	82	lm	167		
>ref-60489	GCTCCCAGAACACCCCCTCCCTTCACA	1	<lmxll>	ll	231	lm	36		
>ref-60489	GCTCCCAGAACACCCCCTCCCTTCACA	16	<hkxhk>	hh	0	hk	270	kk	0
>ref-60491	CCTGTCTCAACTCCCTCTCCTCAACCC	21	<lmxll>	ll	1	lm	266		
>ref-60491	CCTGTCTCAACTCCCTCTCCTCAACCC	23	<lmxll>	ll	13	lm	245		
>ref-60497	ACTCCGGGTACTGGCCCTCCGGTTTCA	24	<hkxhk>	hh	0	hk	272	kk	1
>ref-60500	CCACCCTCCACCTCATCTCCCACACTC	5	<nnxnp>	nn	220	np	0		
>ref-60531	CTCGCTGCGACTGTCACTCCCACTCGC	13	<nnxnp>	nn	123	np	141		
>ref-60533	CACAAACAAACATTTCCTCCACACAAA	14	<hkxhk>	hh	0	hk	263	kk	1
>ref-60533	CACAAACAAACATTTCCTCCACACAAA	15	<hkxhk>	hh	0	hk	263	kk	1
>ref-60537	CCCCTTTACACTGCCTCTCCTCTTCCT	8	<nnxnp>	nn	114	np	138		

>ref-60545	GCACAAGTAACCCCACCTCCCATCCTG	2	<lmxll>	ll	133	lm	120		
>ref-60550	GCGCCGCCCACGTAGCCTCCATTAAGT	26	<lmxll>	ll	167	lm	98		
>ref-60550	GCGCCGCCCACGTAGCCTCCATTAAGT	27	<lmxll>	ll	166	lm	98		
>ref-60556	TATTATCCCACAAATTCTCCGTCGACA	16	<nnxnp>	nn	137	np	119		
>ref-60580	ACCCTCTCCACCTCTCCTCCAGCCTTT	25	<lmxll>	ll	106	lm	150		
>ref-60611	TTCCCATATACTCTTCCTCCTCCTCCA	5	<nnxnp>	nn	240	np	1		
>ref-60611	TTCCCATATACTCTTCCTCCTCCTCCA	8	<nnxnp>	nn	218	np	5		
>ref-60627	CCCCCATAAACCTTCCCTCCTTCCTTC	6	<nnxnp>	nn	23	np	235		
>ref-60627	CCCCCATAAACCTTCCCTCCTTCCTTC	7	<nnxnp>	nn	8	np	257		
>ref-60665	CCAGCGCCGACATCTACTCCTTCGGTG	9	<nnxnp>	nn	273	np	0		
>ref-60677	TCTCCTTCTACTCCTCCTCCACCTCTG	4	<lmxll>	ll	273	lm	0		
>ref-60677	TCTCCTTCTACTCCTCCTCCACCTCTG	21	<hkxhk>	hh	0	hk	273	kk	0
>ref-60677	TCTCCTTCTACTCCTCCTCCACCTCTG	26	<lmxll>	ll	0	lm	273		
>ref-60687	GCCGGAGAGACATGATCTCCCTTGTGC	3	<lmxll>	ll	109	lm	156		
>ref-60688	GGAGGCTTTACTCTTTCTCCTGTGAAA	24	<lmxll>	ll	114	lm	119		
>ref-60707	AACCCCAAGACACGACCTCCCTTTGCT	5	<lmxll>	ll	160	lm	111		
>ref-60707	AACCCCAAGACACGACCTCCCTTTGCT	7	<lmxll>	ll	159	lm	111		
>ref-60735	TTATTCATTACCCCTCCTCCTTTCCCT	1	<lmxll>	ll	242	lm	22		
>ref-60735	TTATTCATTACCCCTCCTCCTTTCCCT	4	<lmxll>	ll	242	lm	22		
>ref-60735	TTATTCATTACCCCTCCTCCTTTCCCT	6	<nnxnp>	nn	153	np	101		
>ref-60735	TTATTCATTACCCCTCCTCCTTTCCCT	7	<hkxhk>	hh	2	hk	270	kk	1
>ref-60735	TTATTCATTACCCCTCCTCCTTTCCCT	12	<hkxhk>	hh	1	hk	270	kk	2
>ref-60736	TACCCCTCCACAACCTCTCCCCACCCC	24	<lmxll>	ll	115	lm	118		
>ref-60736	TACCCCTCCACAACCTCTCCCCACCCC	25	<lmxll>	ll	115	lm	118		
>ref-60751	CTGCCCCCGACCAAGCCTCCTTCATCC	6	<lmxll>	ll	139	lm	133		
>ref-60769	TTCATTTTCACTTCCTCTCCATTCCTT	14	<nnxnp>	nn	109	np	131		
>ref-60769	TTCATTTTCACTTCCTCTCCATTCCTT	26	<nnxnp>	nn	109	np	131		
>ref-60774	CCCTCTGGCACAAGAACTCCCCTTCCC	14	<nnxnp>	nn	268	np	0		
>ref-60776	GGTATTAAGACATTCTCTCCTGCACTT	1	<nnxnp>	nn	137	np	116		
>ref-60776	GGTATTAAGACATTCTCTCCTGCACTT	15	<nnxnp>	nn	264	np	0		
>ref-60789	TCTGACATCACCTGCCCTCCTGCCTCG	14	<hkxhk>	hh	61	hk	145	kk	59
>ref-60817	CTCCACCCCACTCTCCCTCCCCACTCC	25	<nnxnp>	nn	235	np	0		
>ref-60823	TTTCCATTTACTATTTCTCCCTTTCTT	6	<hkxhk>	hh	0	hk	259	kk	10
>ref-60823	TTTCCATTTACTATTTCTCCCTTTCTT	23	<nnxnp>	nn	79	np	179		
>ref-60823	TTTCCATTTACTATTTCTCCCTTTCTT	26	<hkxhk>	hh	0	hk	259	kk	10
>ref-60840	ATTCGGACAACTATAACTCCGGACACG	2	<nnxnp>	nn	115	np	143		
>ref-60849	TATCCCCGCACTTTATCTCCTTAAGTG	6	<lmxll>	ll	131	lm	125		
>ref-60856	CTCAGTTTCACTTTTTCTCCCTCCCTC	9	<hkxhk>	hh	3	hk	266	kk	0

>ref-60856	CTCAGTTTCACTTTTTCTCCCTCCCTC	16	<hkxhk>	hh	0	hk	267	kk	2
>ref-60879	TCACTCATGACAGATACTCCTCACTTA	5	<hkxhk>	hh	0	hk	272	kk	1
>ref-60879	TCACTCATGACAGATACTCCTCACTTA	14	<hkxhk>	hh	1	hk	272	kk	0
>ref-60891	CTAAGTCCCACTTTCCCTCCTTCCCTC	2	<nnxnp>	nn	7	np	261		
>ref-60922	GTCGAGCAGACGCGTCCTCCGGCCATC	13	<nnxnp>	nn	130	np	140		
>ref-60960	TTGCCCTTCACCTCCCCTCCTCCCTTT	4	<hkxhk>	hh	7	hk	255	kk	0
>ref-60960	TTGCCCTTCACCTCCCCTCCTCCCTTT	13	<hkxhk>	hh	0	hk	255	kk	8
>ref-61001	AATATTGCTACCCCCACTCCCTTTCCA	16	<hkxhk>	hh	27	hk	204	kk	0
>ref-61001	AATATTGCTACCCCCACTCCCTTTCCA	21	<hkxhk>	hh	0	hk	204	kk	27
>ref-61054	TAATATACCACAAGAGCTCCTAATACA	3	<lmxll>	ll	112	lm	119		
>ref-61081	CTTAACACGACCTCGCCTCCACTTCAG	26	<hkxhk>	hh	61	hk	149	kk	49
>ref-61083	AGTCATCCCACTTCATCTCCTTTTCCT	8	<nnxnp>	nn	115	np	141		
>ref-61108	TCCTATCCCACTCTCCCTCCCCCCTCT	4	<lmxll>	ll	117	lm	138		
>ref-61108	TCCTATCCCACTCTCCCTCCCCCCTCT	7	<lmxll>	ll	120	lm	137		
>ref-61114	TCCCTTATAACTTCCCCTCCCTCCCCT	8	<nnxnp>	nn	119	np	137		
>ref-61122	CAGTCCTTCACTCTATCTCCCTTGTCT	21	<nnxnp>	nn	158	np	98		
>ref-61122	CAGTCCTTCACTCTATCTCCCTTGTCT	24	<nnxnp>	nn	157	np	98		
>ref-61163	GCACTTGCCACTGCTCCTCCTCCTTCC	3	<nnxnp>	nn	102	np	133		
>ref-61164	CACTCTTTAACGGTCTCTCCTGATATC	4	<lmxll>	ll	111	lm	147		
>ref-61164	CACTCTTTAACGGTCTCTCCTGATATC	7	<lmxll>	ll	111	lm	147		
>ref-61275	AGAGTCGATACTGCTGCTCCGGTGTAC	7	<nnxnp>	nn	143	np	127		
>ref-61275	AGAGTCGATACTGCTGCTCCGGTGTAC	23	<nnxnp>	nn	148	np	123		
>ref-61278	AACTCACATACACACACTCCCACAAGT	13	<lmxll>	ll	138	lm	128		
>ref-61281	AGTACTAAGACACACACTCCCGAGCCC	23	<nnxnp>	nn	140	np	129		
>ref-61305	CCTTGGCCAACCAGACCTCCCATACCA	2	<nnxnp>	nn	132	np	133		
>ref-61314	CCCCACTCGACTCCCTCTCCTTTACTT	15	<lmxll>	ll	113	lm	128		
>ref-61319	CTCCTTTTCACTCTATCTCCCCTCCCT	4	<hkxhk>	hh	110	hk	121	kk	0
>ref-61319	CTCCTTTTCACTCTATCTCCCCTCCCT	15	<hkxhk>	hh	0	hk	266	kk	2
>ref-61319	CTCCTTTTCACTCTATCTCCCCTCCCT	21	<hkxhk>	hh	0	hk	121	kk	109
>ref-61319	CTCCTTTTCACTCTATCTCCCCTCCCT	25	<hkxhk>	hh	0	hk	266	kk	2
>ref-61330	CACTAGACCACTAGACCTCCATCATCA	26	<nnxnp>	nn	127	np	110		
>ref-61331	TACACCCTTACACCTACTCCCTTGCCC	14	<lmxll>	ll	136	lm	118		
>ref-61335	TCCTTCTCCACCACGCCTCCTTCGCTT	9	<lmxll>	ll	127	lm	141		
>ref-61349	CTAGTTGAAACCAACACTCCACGGCCC	14	<nnxnp>	nn	126	np	139		
>ref-61372	TCCCTTCCAACTCCCTCTCCTCAAGCG	12	<hkxhk>	hh	44	hk	148	kk	63
>ref-61377	GTGTTGGTGACTCATCCTCCATACATT	14	<hkxhk>	hh	76	hk	132	kk	51
>ref-61406	GGAATCCTAACTATAGCTCCACGTAAG	14	<nnxnp>	nn	124	np	118		
>ref-61425	TTCTTTCTTACCTTTTCTCCCCCCTCT	13	<nnxnp>	nn	111	np	147		

>ref-61448	TCGCCTTCAACCTTCCCTCCTTCCCTT	3	<lmxll>	ll	2	lm	233		
>ref-61448	TCGCCTTCAACCTTCCCTCCTTCCCTT	13	<lmxll>	ll	117	lm	134		
>ref-61452	TATGTGATGACTCATTCTCCTTCGTAA	27	<nnxnp>	nn	160	np	88		
>ref-61461	GTCCCTTTCACTCTCTCTCCTGCCCAC	26	<lmxll>	ll	268	lm	0		
>ref-61468	TGTCCTTTTACTATTTCTCCAATTACA	1	<nnxnp>	nn	238	np	0		
>ref-61469	ATCACCCTAACCGCGCCTCCACGACAT	25	<nnxnp>	nn	131	np	138		
>ref-61471	CCGCCGTACACGCTCTCTCCCACCCAT	1	<nnxnp>	nn	132	np	91		
>ref-61509	TTCACCATCACCATCTCTCCCTCTCTT	26	<lmxll>	ll	114	lm	142		
>ref-61535	CCGAGAATGACCTCCGCTCCGAACGAC	2	<nnxnp>	nn	231	np	0		
>ref-61542	CTACTTTCAACATACGCTCCCGCTCAG	24	<nnxnp>	nn	110	np	118		
>ref-61569	CGATTCTCAACTGTGTCTCCATCTCAT	13	<nnxnp>	nn	250	np	0		
>ref-61575	CTCGGTTCCACGAAGCCTCCAGACTAC	4	<hkxhk>	hh	60	hk	144	kk	62
>ref-61575	CTCGGTTCCACGAAGCCTCCAGACTAC	5	<hkxhk>	hh	60	hk	144	kk	62
>ref-61591	GAGGAGAATACAAGGCCTCCTCCTCAA	22	<lmxll>	ll	143	lm	126		
>ref-61626	AGGCATAAGACGTGTTCTCCGTTCACT	3	<nnxnp>	nn	128	np	139		
>ref-61626	AGGCATAAGACGTGTTCTCCGTTCACT	4	<nnxnp>	nn	128	np	139		
>ref-61630	AGTTGACAAACAACATCTCCATAAGGT	1	<lmxll>	ll	101	lm	144		
>ref-61644	ATAATAGCTACCTCTCCTCCTCCCCTC	13	<nnxnp>	nn	100	np	139		
>ref-61644	ATAATAGCTACCTCTCCTCCTCCCCTC	23	<nnxnp>	nn	99	np	139		
>ref-61660	CCTCCATGTACCCTCCCTCCTCCATCA	7	<nnxnp>	nn	126	np	131		
>ref-61660	CCTCCATGTACCCTCCCTCCTCCATCA	8	<nnxnp>	nn	121	np	121		
>ref-61661	CCCACAACGACACAAACTCCCCCACAA	2	<hkxhk>	hh	0	hk	262	kk	0
>ref-61661	CCCACAACGACACAAACTCCCCCACAA	3	<lmxll>	ll	138	lm	111		
>ref-61661	CCCACAACGACACAAACTCCCCCACAA	7	<hkxhk>	hh	0	hk	262	kk	0
>ref-61661	CCCACAACGACACAAACTCCCCCACAA	12	<hkxhk>	hh	168	hk	86	kk	0
>ref-61661	CCCACAACGACACAAACTCCCCCACAA	23	<nnxnp>	nn	234	np	26		
>ref-61700	AATCAACCCACTTAGTCTCCTTCTGCG	6	<nnxnp>	nn	119	np	130		
>ref-61703	CTTATCTTCACCTTTTCTCCCTTCGTT	5	<lmxll>	ll	125	lm	136		
>ref-61716	TCTTCTCCTACGCCTACTCCGACAGGG	2	<hkxhk>	hh	12	hk	242	kk	1
>ref-61716	TCTTCTCCTACGCCTACTCCGACAGGG	14	<hkxhk>	hh	12	hk	242	kk	1
>ref-61726	TCCGAGGGGACACACTCTCCCGCCATT	26	<nnxnp>	nn	272	np	0		
>ref-61755	AATAATTCAACATTCACTCCTTCGCTG	2	<lmxll>	ll	120	lm	131		
>ref-61779	TACAGTATCACACCACCTCCTATACCG	1	<nnxnp>	nn	114	np	138		
>ref-61779	TACAGTATCACACCACCTCCTATACCG	8	<nnxnp>	nn	114	np	138		
>ref-61779	TACAGTATCACACCACCTCCTATACCG	16	<lmxll>	ll	134	lm	125		
>ref-61839	ATATAACACACTTTTCCTCCCACCGAA	4	<hkxhk>	hh	36	hk	211	kk	4
>ref-61839	ATATAACACACTTTTCCTCCCACCGAA	5	<hkxhk>	hh	4	hk	211	kk	36
>ref-61839	ATATAACACACTTTTCCTCCCACCGAA	15	<hkxhk>	hh	0	hk	175	kk	72

>ref-61839	ATATAACACACTTTTCCTCCCACCGAA	25	<hkxhk>	hh	0	hk	175	kk	72
>ref-61864	CATATATATACACCCCCTCCATCTCTG	3	<lmxll>	ll	114	lm	114		
>ref-61867	CTCCTTCCCACTCTTTCTCCTCAACAT	1	<hkxhk>	hh	3	hk	263	kk	0
>ref-61867	CTCCTTCCCACTCTTTCTCCTCAACAT	8	<hkxhk>	hh	3	hk	264	kk	0
>ref-61893	CTGGACAGAACAGTGACTCCCCATCAG	25	<nnxnp>	nn	125	np	146		
>ref-61896	CGACCACCGACAGTGTCTCCAAAAGGG	27	<nnxnp>	nn	135	np	126		
>ref-61919	GTGTCACTCACTTAGTCTCCTAACTGA	16	<lmxll>	ll	260	lm	0		
>ref-61986	ATCTTTGATACTGCTTCTCCATTTTAT	26	<hkxhk>	hh	1	hk	224	kk	20
>ref-61992	ACGATGTTCACTATTTCTCCTCCCATA	4	<hkxhk>	hh	0	hk	273	kk	0
>ref-61992	ACGATGTTCACTATTTCTCCTCCCATA	6	<lmxll>	ll	40	lm	209		
>ref-61992	ACGATGTTCACTATTTCTCCTCCCATA	8	<nnxnp>	nn	165	np	97		
>ref-61992	ACGATGTTCACTATTTCTCCTCCCATA	9	<lmxll>	ll	176	lm	61		
>ref-62008	TGGGTGCCCACCTACCCTCCTCAGCAT	4	<lmxll>	ll	272	lm	0		
>ref-62008	TGGGTGCCCACCTACCCTCCTCAGCAT	15	<lmxll>	ll	136	lm	123		
>ref-62008	TGGGTGCCCACCTACCCTCCTCAGCAT	16	<hkxhk>	hh	59	hk	203	kk	0
>ref-62008	TGGGTGCCCACCTACCCTCCTCAGCAT	22	<hkxhk>	hh	0	hk	272	kk	1
>ref-62011	TGATTGTAAACCTTCGCTCCTCCTTCT	25	<nnxnp>	nn	134	np	118		
>ref-62019	CCACTCGTCACCCTTACTCCAAACAAT	27	<hkxhk>	hh	62	hk	137	kk	58
>ref-62029	GCTAGTATCACTCAACCTCCAGGATTT	12	<nnxnp>	nn	220	np	0		
>ref-62029	GCTAGTATCACTCAACCTCCAGGATTT	14	<nnxnp>	nn	219	np	0		
>ref-62047	CTCCCTTCCACCTTTCCTCCTCTATTT	27	<lmxll>	ll	126	lm	123		
>ref-62063	ACCTACACCACACAACCTCCTCCTCCT	4	<hkxhk>	hh	2	hk	192	kk	71
>ref-62063	ACCTACACCACACAACCTCCTCCTCCT	5	<hkxhk>	hh	71	hk	192	kk	2
>ref-62078	CCCCAACACACCCCGTCTCCCCCAAAA	5	<nnxnp>	nn	104	np	128		
>ref-62106	CCCTCCCTAACTCTCTCTCCGTGCCAC	9	<lmxll>	ll	219	lm	0		
>ref-62132	CTTTCCTCCACCCTCTCTCCCTCCTAC	1	<nnxnp>	nn	244	np	0		
>ref-62132	CTTTCCTCCACCCTCTCTCCCTCCTAC	8	<nnxnp>	nn	244	np	1		
>ref-62132	CTTTCCTCCACCCTCTCTCCCTCCTAC	16	<nnxnp>	nn	227	np	8		
>ref-62138	ATACTCCCCACTTTCCCTCCTCCCTCT	2	<nnxnp>	nn	147	np	121		
>ref-62138	ATACTCCCCACTTTCCCTCCTCCCTCT	14	<nnxnp>	nn	147	np	121		
>ref-62144	CATATTGCTACGTAATCTCCCTGCAAA	3	<hkxhk>	hh	49	hk	133	kk	67
>ref-62153	CCCTTTCTGACTTCCTCTCCCTCTTCT	5	<lmxll>	ll	140	lm	125		
>ref-62153	CCCTTTCTGACTTCCTCTCCCTCTTCT	9	<nnxnp>	nn	269	np	0		
>ref-62169	CTGTGTGTTACTCTCTCTCCTTTGCCT	23	<nnxnp>	nn	126	np	140		
>ref-62170	ACCATTCCTACTCCTCCTCCCTCCAAC	26	<nnxnp>	nn	123	np	133		
>ref-62172	AGCGTTATGACATGACCTCCCTCTTAT	24	<nnxnp>	nn	134	np	129		
>ref-62198	GGAGCAGCCACGATGTCTCCAGCATTC	27	<hkxhk>	hh	0	hk	268	kk	4
>ref-62214	CCGCGGCCGACCCAATCTCCCCCCGAG	4	<nnxnp>	nn	259	np	0		

>ref-62214	CCGCGGCCGACCCAATCTCCCCCCGAG	13	<lmxll>	ll	130	lm	119		
>ref-62218	TGCGCACACACACACTCTCCTGGGTCC	4	<lmxll>	ll	126	lm	143		
>ref-62220	TCCTCCTGCACTGCATCTCCCTTGTCC	23	<lmxll>	ll	125	lm	141		
>ref-62249	TTCTCAGCCACTCACCCTCCCTCTCCA	14	<hkxhk>	hh	4	hk	265	kk	1
>ref-62249	TTCTCAGCCACTCACCCTCCCTCTCCA	26	<hkxhk>	hh	4	hk	265	kk	1
>ref-62250	TTTCCAGTCACCCTTTCTCCCGGCATA	6	<lmxll>	ll	110	lm	139		
>ref-62254	CTCTCTCCTACCTCCCCTCCACTCCCT	8	<lmxll>	ll	69	lm	194		
>ref-62284	ATTTCTTCTACCCTTTCTCCTTCCTCT	3	<hkxhk>	hh	0	hk	269	kk	0
>ref-62284	ATTTCTTCTACCCTTTCTCCTTCCTCT	15	<hkxhk>	hh	0	hk	269	kk	0
>ref-62327	CCTCCTCCTACAGCGCCTCCGTCAGGT	2	<hkxhk>	hh	4	hk	266	kk	0
>ref-62327	CCTCCTCCTACAGCGCCTCCGTCAGGT	15	<lmxll>	ll	42	lm	223		
>ref-62366	CTTGGCAGAACTCTCCCTCCCTCGCAC	5	<lmxll>	ll	149	lm	117		
>ref-62368	CCGCGGCCGACGCCGCCTCCTCCTGCA	1	<nnxnp>	nn	138	np	134		
>ref-62376	GAAAAGGAAACCGCAACTCCTCAAATG	14	<lmxll>	ll	135	lm	89		
>ref-62391	TCGTGCTACACTCGCCCTCCGTCACGG	15	<nnxnp>	nn	255	np	0		
>ref-62393	TTTATTATCACGGTTCCTCCAGCACCA	4	<nnxnp>	nn	97	np	140		
>ref-62404	TGAGTAACAACGGGGTCTCCTGTCACG	12	<nnxnp>	nn	127	np	138		
>ref-62404	TGAGTAACAACGGGGTCTCCTGTCACG	13	<nnxnp>	nn	127	np	138		
>ref-62411	TCCTCTTCTACTCTCTCTCCCTTCTCT	12	<lmxll>	ll	118	lm	136		
>ref-62411	TCCTCTTCTACTCTCTCTCCCTTCTCT	24	<nnxnp>	nn	265	np	0		
>ref-62411	TCCTCTTCTACTCTCTCTCCCTTCTCT	25	<nnxnp>	nn	260	np	3		
>ref-62417	ACACCCTAAACAAGCACTCCACTCTCA	5	<nnxnp>	nn	127	np	143		
>ref-62439	CTGCCCTTCACTCTCTCTCCTTCCCTT	6	<lmxll>	ll	231	lm	2		
>ref-62440	TACACTCATACACTTCCTCCCTATTCA	22	<nnxnp>	nn	129	np	134		
>ref-62446	CCGCCGTGTACCCTTTCTCCCTTTTCT	25	<nnxnp>	nn	13	np	251		
>ref-62458	CTCCTCTCTACCTCTCCTCCCCTCTTT	2	<lmxll>	ll	40	lm	220		
>ref-62458	CTCCTCTCTACCTCTCCTCCCCTCTTT	25	<lmxll>	ll	40	lm	219		
>ref-62472	CCTCTTTCTACACCTTCTCCCTCTCTC	15	<nnxnp>	nn	246	np	1		
>ref-62483	AAGAGCCCGACATTCCCTCCATTGTCT	1	<nnxnp>	nn	254	np	0		
>ref-62497	TACACACATACGTAGCCTCCGGTGGCG	2	<nnxnp>	nn	244	np	0		
>ref-62517	CTCTTTCTCACACGCCCTCCCGCGCTC	2	<nnxnp>	nn	131	np	135		
>ref-62532	TCTCCCCCAACTCTCGCTCCTTCCTCC	3	<hkxhk>	hh	0	hk	256	kk	9
>ref-62532	TCTCCCCCAACTCTCGCTCCTTCCTCC	16	<hkxhk>	hh	0	hk	256	kk	8
>ref-62566	TTTCTCTCCACATTACCTCCCACCATC	21	<lmxll>	ll	128	lm	141		
>ref-62567	TCCACATTCACTTTCTCTCCACCTTTA	25	<lmxll>	ll	110	lm	116		
>ref-62594	GAGCCTTGGACTAAACCTCCTTTAAGG	9	<hkxhk>	hh	69	hk	119	kk	38
>ref-62600	CTCCTCCTCACCCTTTCTCCTTACCTC	4	<lmxll>	ll	131	lm	121		
>ref-62600	CTCCTCCTCACCCTTTCTCCTTACCTC	16	<hkxhk>	hh	0	hk	214	kk	41

>ref-62600	CTCCTCCTCACCCTTTCTCCTTACCTC	22	<hkxhk>	hh	0	hk	215	kk	42
>ref-62632	TTCCTACCCACTTGCTCTCCCTTTACT	4	<lmxll>	ll	137	lm	122		
>ref-62632	TTCCTACCCACTTGCTCTCCCTTTACT	15	<lmxll>	ll	138	lm	122		
>ref-62654	CCTCTCCCCACACTAGCTCCCTCTCCT	24	<lmxll>	ll	151	lm	88		
>ref-62654	CCTCTCCCCACACTAGCTCCCTCTCCT	27	<lmxll>	ll	148	lm	89		
>ref-62657	GAAAGTGTTACTGCGGCTCCGTACCCT	13	<nnxnp>	nn	128	np	133		
>ref-62657	GAAAGTGTTACTGCGGCTCCGTACCCT	14	<nnxnp>	nn	128	np	133		
>ref-62657	GAAAGTGTTACTGCGGCTCCGTACCCT	15	<nnxnp>	nn	125	np	137		
>ref-62663	TAGTGATACACGGCATCTCCTTTGAAT	7	<lmxll>	ll	8	lm	257		
>ref-62698	TCTCTGAACACCTTTCCTCCTCTGGGC	5	<nnxnp>	nn	254	np	1		
>ref-62710	CCTCCAGGTACTCCCTCTCCTCCTCCC	23	<hkxhk>	hh	60	hk	134	kk	59
>ref-62713	GAATAGCTTACTTCATCTCCTCAGTGT	6	<lmxll>	ll	141	lm	112		
>ref-62717	TACGTACACACTCCTCCTCCTCCCCCC	4	<lmxll>	ll	238	lm	1		
>ref-62717	TACGTACACACTCCTCCTCCTCCCCCC	5	<lmxll>	ll	245	lm	0		
>ref-62734	TCCTCCTCTACCGTTTCTCCTTCGTTC	24	<lmxll>	ll	123	lm	150		
>ref-62773	AAACGCACAACAGCACCTCCCACATCA	16	<lmxll>	ll	140	lm	125		
>ref-62780	TAGAAGGCAACAGGGCCTCCCATGAAT	13	<hkxhk>	hh	0	hk	270	kk	3
>ref-62780	TAGAAGGCAACAGGGCCTCCCATGAAT	24	<hkxhk>	hh	0	hk	270	kk	3
>ref-62786	TGAACCTTCACATTACCTCCTGCCCTA	4	<lmxll>	ll	121	lm	126		
>ref-62789	ATCGTAATTACTTTCCCTCCACTGCAG	8	<lmxll>	ll	130	lm	132		
>ref-62798	CGTGCCCGTACTCTATCTCCCCTCTAA	15	<lmxll>	ll	113	lm	120		
>ref-62800	CGCTCCCGGACTTACCCTCCATTCACT	5	<lmxll>	ll	95	lm	140		
>ref-62805	TTACCACTAACTCCCCCTCCTCCCCCA	9	<hkxhk>	hh	0	hk	263	kk	2
>ref-62805	TTACCACTAACTCCCCCTCCTCCCCCA	15	<hkxhk>	hh	0	hk	263	kk	2
>ref-62806	TCACTCCCAACCTCCTCTCCTTCCTCT	3	<hkxhk>	hh	66	hk	141	kk	46
>ref-62837	CACATCCCGACGACTGCTCCAAGGGCT	2	<hkxhk>	hh	8	hk	263	kk	0
>ref-62846	CCATCTAGAACCTCCGCTCCATCGGAG	24	<lmxll>	ll	105	lm	147		
>ref-62847	TGCCCCAACACTCCTCCTCCTTCCCTC	2	<nnxnp>	nn	136	np	120		
>ref-62854	CATCGCCATACCCATCCTCCTCTTCCT	24	<nnxnp>	nn	101	np	169		
>ref-62855	TTCCTCTCCACCCTTTCTCCTTCCCTT	4	<hkxhk>	hh	1	hk	270	kk	0
>ref-62855	TTCCTCTCCACCCTTTCTCCTTCCCTT	23	<hkxhk>	hh	1	hk	270	kk	0
>ref-62863	GTTTTTCTCACTCCGTCTCCTTCCCCT	26	<lmxll>	ll	126	lm	117		
>ref-62868	CTTATTCGGACTCCTCCTCCGCGGCTG	13	<nnxnp>	nn	127	np	142		
>ref-62886	GTATTCATGACATATACTCCTATGCAT	1	<hkxhk>	hh	2	hk	253	kk	2
>ref-62886	GTATTCATGACATATACTCCTATGCAT	7	<hkxhk>	hh	6	hk	246	kk	1
>ref-62886	GTATTCATGACATATACTCCTATGCAT	14	<lmxll>	ll	130	lm	109		
>ref-62895	AACCTTAGGACCTCCTCTCCCGCTGCC	21	<nnxnp>	nn	117	np	151		
>ref-62895	AACCTTAGGACCTCCTCTCCCGCTGCC	24	<nnxnp>	nn	116	np	151		

>ref-62906	GCCACGCCCACTCCCACTCCTACTTCA	13	<hkxhk>	hh	0	hk	53	kk	201
>ref-62906	GCCACGCCCACTCCCACTCCTACTTCA	22	<hkxhk>	hh	0	hk	53	kk	199
>ref-62924	TTTTGACAAACTTCTACTCCATCGTTT	1	<hkxhk>	hh	0	hk	252	kk	11
>ref-62924	TTTTGACAAACTTCTACTCCATCGTTT	16	<lmxll>	ll	35	lm	205		
>ref-62924	TTTTGACAAACTTCTACTCCATCGTTT	24	<lmxll>	ll	53	lm	200		
>ref-62944	ATATCACTTACTCTGGCTCCCGCTGCA	27	<nnxnp>	nn	127	np	142		
>ref-62985	GACTTCGTAACTTACCCTCCCCATCTG	1	<nnxnp>	nn	136	np	136		
>ref-62991	CCCCCCTCCACATTCCCTCCGCATTCC	3	<hkxhk>	hh	27	hk	230	kk	8
>ref-63006	TTCGTCCTTACTCCTCCTCCTTCATCC	4	<lmxll>	ll	116	lm	129		
>ref-63021	CCTAATCACACACACCCTCCCCATCCC	14	<lmxll>	ll	124	lm	142		
>ref-63028	GATTCTATAACCTCCTCTCCACCCCTT	1	<lmxll>	ll	3	lm	253		
>ref-63028	GATTCTATAACCTCCTCTCCACCCCTT	7	<lmxll>	ll	5	lm	251		
>ref-63046	AATGGATATACAACTTCTCCCAGCAAT	7	<lmxll>	ll	134	lm	100		
>ref-63066	GACTTCATAACCGGCGCTCCTTTAGGT	3	<nnxnp>	nn	143	np	120		
>ref-63080	TCCCCCATCACCATCCCTCCTCTTCCT	13	<lmxll>	ll	65	lm	171		
>ref-63080	TCCCCCATCACCATCCCTCCTCTTCCT	26	<lmxll>	ll	65	lm	169		
>ref-63098	TACACACACACGCTCTCTCCCTCTTTC	1	<hkxhk>	hh	0	hk	273	kk	0
>ref-63098	TACACACACACGCTCTCTCCCTCTTTC	3	<lmxll>	ll	142	lm	119		
>ref-63098	TACACACACACGCTCTCTCCCTCTTTC	25	<hkxhk>	hh	0	hk	273	kk	0
>ref-63102	GCCCCTCCAACCTCCCCTCCCTCACCC	1	<nnxnp>	nn	164	np	84		
>ref-63102	GCCCCTCCAACCTCCCCTCCCTCACCC	26	<hkxhk>	hh	0	hk	271	kk	0
>ref-63112	ATGGACTCCACCTTGTCTCCGACCATC	3	<lmxll>	ll	127	lm	142		
>ref-63112	ATGGACTCCACCTTGTCTCCGACCATC	21	<nnxnp>	nn	163	np	109		
>ref-63163	CAGTCCGTCACAGGGCCTCCCTCTACT	7	<nnxnp>	nn	141	np	130		
>ref-63172	CTCACTAAAACCGAGCCTCCCACACAT	13	<lmxll>	ll	118	lm	149		
>ref-63182	TTGCCTTCAACATTATCTCCGGAAGGG	14	<lmxll>	ll	128	lm	121		
>ref-63189	TCCTCGTATACGTTAACTCCTCTCTCT	2	<lmxll>	ll	134	lm	122		
>ref-63189	TCCTCGTATACGTTAACTCCTCTCTCT	12	<lmxll>	ll	135	lm	121		
>ref-63206	AGATCCAGAACGATATCTCCGGCGTAT	12	<nnxnp>	nn	131	np	126		
>ref-63213	ATTACCATGACTCACTCTCCATTACTC	24	<nnxnp>	nn	131	np	123		
>ref-63231	TAATCGGACACGAGAACTCCACCTTCA	12	<lmxll>	ll	107	lm	120		
>ref-63242	TCTCCTTCAACCTCCTCTCCCTCTTCC	8	<hkxhk>	hh	0	hk	270	kk	3
>ref-63242	TCTCCTTCAACCTCCTCTCCCTCTTCC	22	<hkxhk>	hh	0	hk	272	kk	0
>ref-63264	GGCGCGGTAACATTTTCTCCTCTTCAT	8	<hkxhk>	hh	3	hk	259	kk	7
>ref-63264	GGCGCGGTAACATTTTCTCCTCTTCAT	13	<hkxhk>	hh	3	hk	259	kk	7
>ref-63269	TTATCCCTCACCTTACCTCCTGTTTAT	2	<nnxnp>	nn	125	np	115		
>ref-63273	AATCCAACCACAAGGACTCCTAAACCC	14	<nnxnp>	nn	137	np	129		
>ref-63273	AATCCAACCACAAGGACTCCTAAACCC	21	<hkxhk>	hh	66	hk	138	kk	57

>ref-63280	CCCAACATCACTCCATCTCCTCCCTCT	7	<hkxhk>	hh	1	hk	270	kk	1
>ref-63280	CCCAACATCACTCCATCTCCTCCCTCT	27	<hkxhk>	hh	1	hk	270	kk	1
>ref-63281	ATAAACCACACACACACTCCCTACCGA	6	<hkxhk>	hh	0	hk	193	kk	64
>ref-63281	ATAAACCACACACACACTCCCTACCGA	7	<hkxhk>	hh	63	hk	192	kk	0
>ref-63286	TTTCTATCTACCTTTTCTCCTCTTCCT	6	<lmxll>	ll	70	lm	149		
>ref-63286	TTTCTATCTACCTTTTCTCCTCTTCCT	13	<lmxll>	ll	84	lm	140		
>ref-63293	AACTCCCCCACTCTTCCTCCTTCGTCT	3	<nnxnp>	nn	136	np	124		
>ref-63320	CCCATAAACACCCGTCCTCCCTCACCT	3	<nnxnp>	nn	124	np	124		
>ref-63325	TTCATTTTTACTATTCCTCCGCTGTAC	16	<hkxhk>	hh	4	hk	261	kk	0
>ref-63333	CACCTGTGCACTTCCACTCCTCCCTCC	15	<hkxhk>	hh	79	hk	129	kk	46
>ref-63337	ATCAGAAATACGCTGTCTCCGGGGGGC	25	<nnxnp>	nn	119	np	132		
>ref-63337	ATCAGAAATACGCTGTCTCCGGGGGGC	26	<lmxll>	ll	131	lm	123		
>ref-63337	ATCAGAAATACGCTGTCTCCGGGGGGC	27	<lmxll>	ll	132	lm	122		
>ref-63340	TTTATATATACCCTGTCTCCCACTGAA	2	<hkxhk>	hh	0	hk	97	kk	156
>ref-63340	TTTATATATACCCTGTCTCCCACTGAA	13	<hkxhk>	hh	0	hk	61	kk	197
>ref-63342	ATTTCGTATACCTAGCCTCCGCAGTTT	21	<lmxll>	ll	127	lm	140		
>ref-63380	TTAGTCTGAACTCCGCCTCCTATCCTA	8	<hkxhk>	hh	1	hk	264	kk	0
>ref-63392	TGTAGGTCGACGCAAACTCCTTTATCT	15	<lmxll>	ll	120	lm	143		
>ref-63403	ACTGACATCACGCTAGCTCCTGGCAAC	23	<hkxhk>	hh	0	hk	272	kk	1
>ref-63403	ACTGACATCACGCTAGCTCCTGGCAAC	24	<hkxhk>	hh	0	hk	273	kk	0
>ref-63458	GTGCTGCCCACCTCCCCTCCCCTACAA	2	<hkxhk>	hh	49	hk	147	kk	57
>ref-63464	CTATCACCAACTGCTCCTCCTTCCTCT	6	<nnxnp>	nn	146	np	117		
>ref-63465	CAGTCATGCACGCCTTCTCCATTTTCT	2	<lmxll>	ll	155	lm	108		
>ref-63465	CAGTCATGCACGCCTTCTCCATTTTCT	14	<lmxll>	ll	160	lm	106		
>ref-63470	TGTAACCTTACCTGGACTCCACAGGAA	4	<nnxnp>	nn	117	np	124		
>ref-63475	GAGGGTCACACCTTACCTCCCTCTCCT	16	<nnxnp>	nn	139	np	125		
>ref-63487	GTCTCCTTCACTTAATCTCCCCTTGTC	25	<lmxll>	ll	145	lm	122		
>ref-63504	TTGTAGTGTACTGGGCCTCCTCGTCGC	9	<nnxnp>	nn	236	np	0		
>ref-63506	GGAAAATCAACTTGCCCTCCGTCGTTT	2	<lmxll>	ll	122	lm	103		
>ref-63519	GTGCAATGAACGGCGTCTCCTTATCCT	22	<hkxhk>	hh	71	hk	125	kk	67
>ref-63531	TATGCCAGTACAGCTGCTCCCCGCTCT	5	<lmxll>	ll	114	lm	130		
>ref-63531	TATGCCAGTACAGCTGCTCCCCGCTCT	24	<lmxll>	ll	132	lm	112		
>ref-63560	TTGACCAGGACGCCATCTCCTCTGTCA	9	<nnxnp>	nn	97	np	127		
>ref-63568	AGGAATCTTACTCTTCCTCCTTTCCCC	4	<nnxnp>	nn	105	np	157		
>ref-63575	ACCCTAACCACAATTACTCCTGACTCC	4	<lmxll>	ll	130	lm	122		
>ref-63608	AAGTATGACACTCGATCTCCTCATCCC	26	<nnxnp>	nn	0	np	253		
>ref-63612	CACACACATACATACACTCCTCATCTG	6	<hkxhk>	hh	66	hk	136	kk	55
>ref-63622	AACAGCGACACTTTCTCTCCAGATATT	1	<lmxll>	ll	115	lm	143		

>ref-63622	AACAGCGACACTTTCTCTCCAGATATT	2	<lmxll>	ll	115	lm	143		
>ref-63662	CACAACTAGACCCGGTCTCCCCTATAT	12	<hkxhk>	hh	20	hk	230	kk	0
>ref-63662	CACAACTAGACCCGGTCTCCCCTATAT	21	<nnxnp>	nn	212	np	33		
>ref-63662	CACAACTAGACCCGGTCTCCCCTATAT	26	<hkxhk>	hh	16	hk	232	kk	0
>ref-63671	CCCTCCCCCACCTTCCCTCCAATCCCC	5	<hkxhk>	hh	24	hk	223	kk	1
>ref-63671	CCCTCCCCCACCTTCCCTCCAATCCCC	23	<nnxnp>	nn	157	np	77		
>ref-63689	TACGACCCCACTTCCGCTCCCCCACCT	6	<lmxll>	ll	151	lm	121		
>ref-63729	TATACAGGGACGATTCCTCCCATACTG	7	<nnxnp>	nn	124	np	123		
>ref-63731	CCACGCCACACAAGACCTCCGAAGTGA	2	<nnxnp>	nn	270	np	0		
>ref-63739	CACCCTCCTACTCATCCTCCCTCTCAT	21	<lmxll>	ll	122	lm	139		
>ref-63743	CCGTGGTAGACCTATACTCCAGGACGT	2	<hkxhk>	hh	0	hk	272	kk	0
>ref-63743	CCGTGGTAGACCTATACTCCAGGACGT	7	<hkxhk>	hh	0	hk	256	kk	13
>ref-63743	CCGTGGTAGACCTATACTCCAGGACGT	9	<hkxhk>	hh	0	hk	256	kk	13
>ref-63743	CCGTGGTAGACCTATACTCCAGGACGT	25	<lmxll>	ll	40	lm	215		
>ref-63743	CCGTGGTAGACCTATACTCCAGGACGT	26	<hkxhk>	hh	0	hk	252	kk	15
>ref-63752	AGACAAAGCACAAGTCCTCCCGCTAGA	15	<nnxnp>	nn	127	np	142		
>ref-63766	ACTTATGTCACCATGTCTCCTTCTTTA	5	<nnxnp>	nn	106	np	128		
>ref-63767	TGGTGGGCCACTGCAGCTCCGACGTCG	8	<lmxll>	ll	123	lm	141		
>ref-63785	TCCCCAAACACATCCCCTCCCCCTCCA	6	<hkxhk>	hh	0	hk	271	kk	0
>ref-63795	CTTCTTGCCACTCCTTCTCCTCCTTCC	7	<lmxll>	ll	5	lm	261		
>ref-63795	CTTCTTGCCACTCCTTCTCCTCCTTCC	9	<lmxll>	ll	6	lm	257		
>ref-63799	GTATCGTTAACATTTCCTCCTCCTTCA	16	<lmxll>	ll	130	lm	139		
>ref-63808	TGTGTAGCTACACTTTCTCCTTCTATC	1	<hkxhk>	hh	0	hk	236	kk	26
>ref-63808	TGTGTAGCTACACTTTCTCCTTCTATC	4	<lmxll>	ll	199	lm	61		
>ref-63808	TGTGTAGCTACACTTTCTCCTTCTATC	25	<hkxhk>	hh	0	hk	273	kk	0
>ref-63813	TTTCCATTAACAGTTTCTCCATTTCCC	5	<lmxll>	ll	148	lm	113		
>ref-63822	CCCACGTAAACCTGGTCTCCCACACGA	27	<lmxll>	ll	137	lm	128		
>ref-63874	ATTTCCTCTACTTTCTCTCCTCCTCCC	13	<nnxnp>	nn	141	np	124		
>ref-63877	AGAGAGGAAACGAGATCTCCCCCTCGG	7	<hkxhk>	hh	0	hk	261	kk	5
>ref-63877	AGAGAGGAAACGAGATCTCCCCCTCGG	16	<lmxll>	ll	122	lm	114		
>ref-63877	AGAGAGGAAACGAGATCTCCCCCTCGG	21	<hkxhk>	hh	1	hk	267	kk	0
>ref-63877	AGAGAGGAAACGAGATCTCCCCCTCGG	22	<hkxhk>	hh	5	hk	261	kk	0
>ref-63879	CCCCGAGGAACTGTCTCTCCCCTCCTC	8	<lmxll>	ll	241	lm	1		
>ref-63898	CACCCCCCAACTCCCTCTCCCTGACTC	22	<nnxnp>	nn	117	np	149		
>ref-63908	GACCGGCTGACTGACTCTCCATCCCTG	14	<nnxnp>	nn	137	np	111		
>ref-63908	GACCGGCTGACTGACTCTCCATCCCTG	21	<hkxhk>	hh	83	hk	115	kk	50
>ref-63949	TAAGAATTTACCTTTTCTCCTCTTCCT	8	<nnxnp>	nn	111	np	135		
>ref-64011	GCTCCATGAACGTCTCCTCCCCAGTTA	22	<nnxnp>	nn	140	np	128		

>ref-64025	CTTACATCCACCTCCTCTCCCTCCTCC	5	<lmxll>	ll	16	lm	254		
>ref-64025	CTTACATCCACCTCCTCTCCCTCCTCC	9	<lmxll>	ll	16	lm	254		
>ref-64035	GGTATCTGAACCTCCTCTCCTTTTGCA	24	<hkxhk>	hh	46	hk	146	kk	64
>ref-64037	TTCATTCTCACACCTTCTCCTCCCTCC	6	<nnxnp>	nn	96	np	141		
>ref-64044	GCCGCCAGAACTTCCCCTCCGGCAGTA	25	<nnxnp>	nn	121	np	143		
>ref-64057	ACCTCCTCCACCCCCTCTCCTGCCCCC	1	<hkxhk>	hh	1	hk	262	kk	0
>ref-64057	ACCTCCTCCACCCCCTCTCCTGCCCCC	24	<nnxnp>	nn	151	np	105		
>ref-64059	ACTCCCATCACGATCTCTCCACTAGAA	7	<lmxll>	ll	185	lm	72		
>ref-64059	ACTCCCATCACGATCTCTCCACTAGAA	9	<lmxll>	ll	234	lm	33		
>ref-64059	ACTCCCATCACGATCTCTCCACTAGAA	24	<lmxll>	ll	3	lm	270		
>ref-64093	CTCACCAGTACACACTCTCCCACGCAC	24	<nnxnp>	nn	100	np	163		
>ref-64133	CTCCCCATAACCCCCACTCCTCCTCCC	5	<lmxll>	ll	2	lm	265		
>ref-64133	CTCCCCATAACCCCCACTCCTCCTCCC	25	<lmxll>	ll	2	lm	265		
>ref-64137	CTTCCTGCCACTTCTCCTCCTTCTATT	22	<hkxhk>	hh	57	hk	142	kk	62
>ref-64147	TTGTTGTCTACGAAGCCTCCCCCAGAG	3	<lmxll>	ll	126	lm	138		
>ref-64159	CGTCTCCTTACAAATCCTCCTCCACTT	16	<lmxll>	ll	85	lm	139		
>ref-64159	CGTCTCCTTACAAATCCTCCTCCACTT	24	<lmxll>	ll	82	lm	138		
>ref-64177	GATAAACTGACCTTTTCTCCATATACA	16	<lmxll>	ll	111	lm	131		
>ref-64179	TCCATTTTCACTTTCTCTCCTCGTTCG	4	<nnxnp>	nn	121	np	126		
>ref-64216	GAGATAGTAACCGGCTCTCCCTTCAAG	14	<lmxll>	ll	140	lm	112		
>ref-64236	CCATACACTACAGCAACTCCGCCGTTG	2	<nnxnp>	nn	273	np	0		
>ref-64252	CAGCACGTGACCTTCCCTCCTTCCCTC	13	<nnxnp>	nn	109	np	158		
>ref-64259	TGACAAGCCACTTCGTCTCCGCGCCAT	4	<lmxll>	ll	141	lm	131		
>ref-64316	GGAGGCTTGACACTGGCTCCTGCACTC	15	<lmxll>	ll	260	lm	10		
>ref-64316	GGAGGCTTGACACTGGCTCCTGCACTC	23	<hkxhk>	hh	25	hk	242	kk	0
>ref-64331	TCCTTCCCAACCCTTCCTCCCCTCCTA	2	<nnxnp>	nn	127	np	122		
>ref-64332	AGTAGAGATACAATGCCTCCACAGATA	9	<nnxnp>	nn	264	np	0		
>ref-64346	TCCATCGATACAGCGGCTCCTCGAAAC	1	<lmxll>	ll	4	lm	269		
>ref-64346	TCCATCGATACAGCGGCTCCTCGAAAC	4	<lmxll>	ll	4	lm	269		
>ref-64365	AGACGCGGTACAACCCCTCCCTCTCCC	2	<hkxhk>	hh	59	hk	138	kk	67
>ref-64420	CTCCTCAATACCTTCCCTCCCCCTCTC	2	<hkxhk>	hh	0	hk	268	kk	3
>ref-64420	CTCCTCAATACCTTCCCTCCCCCTCTC	8	<hkxhk>	hh	3	hk	268	kk	0
>ref-64420	CTCCTCAATACCTTCCCTCCCCCTCTC	24	<nnxnp>	nn	118	np	132		
>ref-64450	TCCCCTGGCACCTCCCCTCCTTTCTCC	2	<hkxhk>	hh	0	hk	264	kk	0
>ref-64484	TCGTCGTATACCTCCCCTCCCTCCCTC	24	<hkxhk>	hh	0	hk	250	kk	6
>ref-64484	TCGTCGTATACCTCCCCTCCCTCCCTC	25	<hkxhk>	hh	11	hk	244	kk	0
>ref-64517	CTACCCCCTACCCTTTCTCCTCCTCCC	7	<lmxll>	ll	0	lm	267		
>ref-64517	CTACCCCCTACCCTTTCTCCTCCTCCC	8	<lmxll>	ll	0	lm	267		

>ref-64532	TCCCTTCCGACTCTCCCTCCTCACCTC	12	<nnxnp>	nn	134	np	128		
>ref-64541	TCTACCTGAACCCCTTCTCCCTCTCCT	1	<hkxhk>	hh	42	hk	134	kk	66
>ref-64543	AGCTAATGCACTTCATCTCCTCGTGGG	26	<nnxnp>	nn	117	np	142		
>ref-64552	AGTGATGGAACATTATCTCCATCCATC	23	<lmxll>	ll	99	lm	125		
>ref-64555	CCCTCCTCTACAACCCCTCCCCTTCCT	2	<hkxhk>	hh	20	hk	241	kk	0
>ref-64555	CCCTCCTCTACAACCCCTCCCCTTCCT	9	<hkxhk>	hh	0	hk	240	kk	22
>ref-64597	AGGTCATATACTCCCTCTCCCTTTTCT	12	<hkxhk>	hh	0	hk	195	kk	57
>ref-64597	AGGTCATATACTCCCTCTCCCTTTTCT	13	<hkxhk>	hh	13	hk	252	kk	0
>ref-64597	AGGTCATATACTCCCTCTCCCTTTTCT	21	<hkxhk>	hh	57	hk	195	kk	0
>ref-64597	AGGTCATATACTCCCTCTCCCTTTTCT	22	<hkxhk>	hh	0	hk	252	kk	13
>ref-64597	AGGTCATATACTCCCTCTCCCTTTTCT	23	<hkxhk>	hh	0	hk	270	kk	1
>ref-64597	AGGTCATATACTCCCTCTCCCTTTTCT	26	<hkxhk>	hh	1	hk	270	kk	0
>ref-64625	TCTCCCCCAACCATCCCTCCCTTCTCT	24	<lmxll>	ll	119	lm	147		
>ref-64625	TCTCCCCCAACCATCCCTCCCTTCTCT	25	<lmxll>	ll	119	lm	147		
>ref-64642	TAGGTAATGACACCGCCTCCTCCCCTC	8	<hkxhk>	hh	2	hk	266	kk	2
>ref-64661	ACCCTCACCACCACCTCTCCTTCACAT	21	<nnxnp>	nn	117	np	143		
>ref-64677	AACTCACGAACCGAAACTCCGGCCCCT	24	<hkxhk>	hh	25	hk	216	kk	0
>ref-64680	TGCCCGGCCACTCTCCCTCCTCTCCCT	5	<nnxnp>	nn	140	np	126		
>ref-64689	CTTGCCTGTACACCCCCTCCCCCCCTC	21	<lmxll>	ll	150	lm	88		
>ref-64695	CCTCGTTAAACATCCTCTCCATTCCTT	9	<nnxnp>	nn	128	np	135		
>ref-64713	CAACCGCTGACAACCTCTCCTCAACCC	1	<lmxll>	ll	0	lm	265		
>ref-64713	CAACCGCTGACAACCTCTCCTCAACCC	6	<lmxll>	ll	88	lm	162		
>ref-64713	CAACCGCTGACAACCTCTCCTCAACCC	21	<lmxll>	ll	89	lm	162		
>ref-64715	CATACCGACACCCCAACTCCCCGACAT	22	<lmxll>	ll	1	lm	265		
>ref-64740	TTTCTTTTCACCTTTCCTCCTTCCTCC	1	<lmxll>	ll	9	lm	264		
>ref-64740	TTTCTTTTCACCTTTCCTCCTTCCTCC	6	<lmxll>	ll	9	lm	264		
>ref-64767	GTTCCCTTAACCCCCTCTCCCCTTTCC	9	<lmxll>	ll	125	lm	131		
>ref-64767	GTTCCCTTAACCCCCTCTCCCCTTTCC	24	<lmxll>	ll	130	lm	127		
>ref-64770	AACGCATGCACAGGCGCTCCAAACTGT	27	<hkxhk>	hh	52	hk	135	kk	50
>ref-64782	ACCCTCACCACTCTCTCTCCCCTTTCC	23	<nnxnp>	nn	95	np	128		
>ref-64807	TTCGTTTATACTTACGCTCCCTGAGGA	3	<nnxnp>	nn	135	np	108		
>ref-64807	TTCGTTTATACTTACGCTCCCTGAGGA	4	<nnxnp>	nn	133	np	109		
>ref-64831	ATATCGCAAACTCTTTCTCCCCACACA	4	<nnxnp>	nn	266	np	6		
>ref-64831	ATATCGCAAACTCTTTCTCCCCACACA	9	<nnxnp>	nn	63	np	188		
>ref-64831	ATATCGCAAACTCTTTCTCCCCACACA	14	<nnxnp>	nn	223	np	34		
>ref-64853	CGTTCTCGTACTTTTTCTCCTTCTCCT	22	<nnxnp>	nn	124	np	133		
>ref-64853	CGTTCTCGTACTTTTTCTCCTTCTCCT	26	<nnxnp>	nn	125	np	131		
>ref-64886	CACTTCCACACCTTTCCTCCCTCTTAA	26	<lmxll>	ll	125	lm	140		

>ref-64899	AGGTATGGCACTGCACCTCCTCTCTGG	9	<nnxnp>	nn	262	np	0		
>ref-64900	CAGCCATCCACCTAAACTCCAGGAATA	16	<nnxnp>	nn	147	np	122		
>ref-64908	CACCTGCTAACCGTGGCTCCACGTATC	4	<hkxhk>	hh	2	hk	271	kk	0
>ref-64908	CACCTGCTAACCGTGGCTCCACGTATC	9	<hkxhk>	hh	2	hk	271	kk	0
>ref-64908	CACCTGCTAACCGTGGCTCCACGTATC	13	<hkxhk>	hh	0	hk	273	kk	0
>ref-64908	CACCTGCTAACCGTGGCTCCACGTATC	16	<lmxll>	ll	65	lm	190		
>ref-64908	CACCTGCTAACCGTGGCTCCACGTATC	23	<lmxll>	ll	111	lm	142		
>ref-64908	CACCTGCTAACCGTGGCTCCACGTATC	25	<hkxhk>	hh	0	hk	273	kk	0
>ref-64945	CACCGCCAGACCCTCCCTCCTACCATA	23	<nnxnp>	nn	105	np	118		
>ref-64947	CTTCCCTGCACTTGCCCTCCTCCTTAC	8	<lmxll>	ll	197	lm	50		
>ref-64947	CTTCCCTGCACTTGCCCTCCTCCTTAC	14	<lmxll>	ll	199	lm	48		
>ref-64961	AACGAGCCCACTGGAGCTCCCTTGTGG	4	<nnxnp>	nn	123	np	124		
>ref-64962	AACATCAAAACTGTAACTCCTTCTGAA	14	<hkxhk>	hh	0	hk	270	kk	1
>ref-64962	AACATCAAAACTGTAACTCCTTCTGAA	22	<hkxhk>	hh	0	hk	270	kk	1
>ref-65040	TCCACCCCTACCCTTCCTCCCCATCTC	4	<nnxnp>	nn	45	np	176		
>ref-65040	TCCACCCCTACCCTTCCTCCCCATCTC	5	<nnxnp>	nn	46	np	176		
>ref-65047	CTCCCCTTCACCCACTCTCCCCTCTCT	3	<nnxnp>	nn	186	np	76		
>ref-65047	CTCCCCTTCACCCACTCTCCCCTCTCT	7	<nnxnp>	nn	182	np	77		
>ref-65047	CTCCCCTTCACCCACTCTCCCCTCTCT	12	<lmxll>	ll	154	lm	107		
>ref-65047	CTCCCCTTCACCCACTCTCCCCTCTCT	15	<lmxll>	ll	124	lm	108		
>ref-65047	CTCCCCTTCACCCACTCTCCCCTCTCT	21	<nnxnp>	nn	45	np	206		
>ref-65047	CTCCCCTTCACCCACTCTCCCCTCTCT	24	<nnxnp>	nn	45	np	209		
>ref-65057	ATCATGCGAACCCCAACTCCAAACCTA	5	<hkxhk>	hh	0	hk	266	kk	2
>ref-65057	ATCATGCGAACCCCAACTCCAAACCTA	14	<hkxhk>	hh	0	hk	269	kk	0
>ref-65068	CACTCTGACACTGACACTCCGCCACAC	4	<nnxnp>	nn	139	np	134		
>ref-65072	TCCTTCCTTACCCCTCCTCCATACTCC	1	<hkxhk>	hh	1	hk	259	kk	8
>ref-65072	TCCTTCCTTACCCCTCCTCCATACTCC	12	<nnxnp>	nn	145	np	120		
>ref-65072	TCCTTCCTTACCCCTCCTCCATACTCC	21	<nnxnp>	nn	149	np	119		
>ref-65072	TCCTTCCTTACCCCTCCTCCATACTCC	22	<lmxll>	ll	5	lm	262		
>ref-65072	TCCTTCCTTACCCCTCCTCCATACTCC	23	<lmxll>	ll	5	lm	263		
>ref-65081	TACTCAGCAACTCTATCTCCCTGCCTA	27	<nnxnp>	nn	131	np	131		
>ref-65132	TTTCCTCCTACCTCCCCTCCACTCCCT	5	<nnxnp>	nn	3	np	261		
>ref-65132	TTTCCTCCTACCTCCCCTCCACTCCCT	12	<nnxnp>	nn	3	np	261		
>ref-65136	TGATTTCGCACTTCCCCTCCTCTCTCC	25	<lmxll>	ll	93	lm	138		
>ref-65136	TGATTTCGCACTTCCCCTCCTCTCTCC	26	<lmxll>	ll	94	lm	137		
>ref-65142	TGTCTTCCAACTCCCCCTCCCCCTTCT	22	<lmxll>	ll	129	lm	132		
>ref-65166	CTCTGTCTCACCCTTTCTCCCGCGAAA	26	<lmxll>	ll	271	lm	0		
>ref-65170	CCCAGCTCAACCTGTCCTCCCTCCCTC	9	<lmxll>	ll	3	lm	268		

>ref-65190	AGGAAAAAGACCTCTGCTCCCACTTAC	2	<hkxhk>	hh	0	hk	236	kk	14
>ref-65190	AGGAAAAAGACCTCTGCTCCCACTTAC	16	<hkxhk>	hh	14	hk	237	kk	0
>ref-65203	CTTCCCCAAACTTCCCCTCCCTCCTCT	3	<hkxhk>	hh	0	hk	269	kk	1
>ref-65222	CAGAAGATGACCCGCGCTCCTAAACGA	15	<nnxnp>	nn	120	np	132		
>ref-65228	TTATCCTCCACCCACTCTCCACTCTTC	25	<lmxll>	ll	143	lm	116		
>ref-65228	TTATCCTCCACCCACTCTCCACTCTTC	26	<lmxll>	ll	144	lm	116		
>ref-65231	AAGGCAGCCACCTCAACTCCCTCGATG	4	<hkxhk>	hh	0	hk	264	kk	3
>ref-65231	AAGGCAGCCACCTCAACTCCCTCGATG	15	<lmxll>	ll	155	lm	113		
>ref-65238	CGCTCCCTCACTCCGTCTCCCCTTCTC	15	<hkxhk>	hh	2	hk	263	kk	0
>ref-65241	CCGTTACTAACCCTCTCTCCTCTTCCC	24	<hkxhk>	hh	64	hk	137	kk	60
>ref-65245	CAGACGTTCACTGTCTCTCCCATTCTC	3	<lmxll>	ll	144	lm	115		
>ref-65275	TCGTGTGGGACGTCGTCTCCGAACCCT	24	<lmxll>	ll	135	lm	94		
>ref-65300	TTCCTCACCACTTTCTCTCCTCCTTCT	3	<lmxll>	ll	120	lm	110		
>ref-65300	TTCCTCACCACTTTCTCTCCTCCTTCT	4	<lmxll>	ll	118	lm	109		
>ref-65314	CATCCTCGGACGCAAACTCCGTATTCT	6	<lmxll>	ll	127	lm	130		
>ref-65314	CATCCTCGGACGCAAACTCCGTATTCT	9	<lmxll>	ll	121	lm	140		
>ref-65344	TTACCAGTTACTGTTGCTCCTCCTCCA	2	<lmxll>	ll	116	lm	140		
>ref-65376	GTTTCTCTCACTCTCTCTCCGTATTCA	1	<hkxhk>	hh	41	hk	141	kk	71
>ref-65379	CCGGGCCTGACCAGCCCTCCAGGCTTC	3	<lmxll>	ll	116	lm	145		
>ref-65383	TTTTCTCCCACCTTTCCTCCCGTCTTC	8	<lmxll>	ll	0	lm	268		
>ref-65383	TTTTCTCCCACCTTTCCTCCCGTCTTC	27	<lmxll>	ll	0	lm	268		
>ref-65399	TCTTTCTCTACGTCCCCTCCTCTTACC	24	<nnxnp>	nn	230	np	0		
>ref-65399	TCTTTCTCTACGTCCCCTCCTCTTACC	25	<nnxnp>	nn	229	np	0		
>ref-65399	TCTTTCTCTACGTCCCCTCCTCTTACC	27	<nnxnp>	nn	230	np	0		
>ref-65421	TTACTCCCTACTCTCACTCCCCTTTCA	12	<lmxll>	ll	116	lm	131		
>ref-65426	ACCACCACCACAGCATCTCCCGCTTGC	2	<lmxll>	ll	124	lm	145		
>ref-65437	CTCACACACACACATTCTCCACGCATA	23	<nnxnp>	nn	140	np	128		
>ref-65437	CTCACACACACACATTCTCCACGCATA	24	<nnxnp>	nn	140	np	128		
>ref-65456	TTTGGTAAAACACTGTCTCCGGGCAAT	27	<hkxhk>	hh	59	hk	148	kk	43
>ref-65487	CAGAAAGAGACTTTCCCTCCGAGCTTT	1	<lmxll>	ll	137	lm	126		
>ref-65499	CTCTATATTACTCCCTCTCCCTAACCC	15	<lmxll>	ll	129	lm	115		
>ref-65507	TTCTTATCGACTAAATCTCCTTCACCC	21	<nnxnp>	nn	129	np	133		
>ref-65521	TAATCCTACACACCCGCTCCTCAACAC	2	<nnxnp>	nn	148	np	124		
>ref-65521	TAATCCTACACACCCGCTCCTCAACAC	6	<hkxhk>	hh	80	hk	166	kk	0
>ref-65539	ACGCTTGAAACACATCCTCCGGCTGTT	23	<nnxnp>	nn	137	np	131		
>ref-65548	ATCTCGGTCACTCTCTCTCCCTTTCCC	6	<nnxnp>	nn	232	np	12		
>ref-65553	CCCTCTTCCACATCGCCTCCCCCTCCC	25	<hkxhk>	hh	0	hk	273	kk	0
>ref-65555	CGTCTCTACACCACCCCTCCCTCTCCC	1	<nnxnp>	nn	107	np	154		

>ref-65555	CGTCTCTACACCACCCCTCCCTCTCCC	2	<hkxhk>	hh	0	hk	251	kk	13
>ref-65555	CGTCTCTACACCACCCCTCCCTCTCCC	3	<hkxhk>	hh	0	hk	272	kk	0
>ref-65555	CGTCTCTACACCACCCCTCCCTCTCCC	6	<hkxhk>	hh	27	hk	233	kk	0
>ref-65555	CGTCTCTACACCACCCCTCCCTCTCCC	14	<hkxhk>	hh	0	hk	270	kk	0
>ref-65569	CCCTGCATTACCTCTTCTCCTCTCCTT	5	<hkxhk>	hh	0	hk	257	kk	8
>ref-65569	CCCTGCATTACCTCTTCTCCTCTCCTT	22	<hkxhk>	hh	8	hk	257	kk	0
>ref-65596	CGGCTGGCGACGCCTCCTCCTCGCAAC	27	<nnxnp>	nn	130	np	139		
>ref-65610	TTCCCTACCACGCTATCTCCCTCCTCT	5	<lmxll>	ll	130	lm	122		
>ref-65616	GTCCTTCTCACCTCCTCTCCTTTTCTT	1	<hkxhk>	hh	109	hk	138	kk	1
>ref-65616	GTCCTTCTCACCTCCTCTCCTTTTCTT	2	<hkxhk>	hh	105	hk	141	kk	1
>ref-65616	GTCCTTCTCACCTCCTCTCCTTTTCTT	3	<nnxnp>	nn	263	np	0		
>ref-65616	GTCCTTCTCACCTCCTCTCCTTTTCTT	14	<lmxll>	ll	261	lm	1		
>ref-65616	GTCCTTCTCACCTCCTCTCCTTTTCTT	23	<lmxll>	ll	259	lm	2		
>ref-65616	GTCCTTCTCACCTCCTCTCCTTTTCTT	27	<lmxll>	ll	259	lm	3		
>ref-65625	ACAACCCACACAACCCCTCCCCCACAC	15	<nnxnp>	nn	128	np	132		
>ref-65627	AGTACTGGAACACCTGCTCCTCTGATG	9	<lmxll>	ll	113	lm	155		
>ref-65629	CTACCTTTAACTATATCTCCCTCTCTA	4	<lmxll>	ll	18	lm	230		
>ref-65629	CTACCTTTAACTATATCTCCCTCTCTA	23	<lmxll>	ll	18	lm	231		
>ref-65630	TCTCCACCGACCCCCTCTCCCCACTCT	3	<hkxhk>	hh	1	hk	262	kk	3
>ref-65631	TAATTGGGAACTTCGGCTCCCTCCTTG	3	<hkxhk>	hh	58	hk	132	kk	49
>ref-65654	GGGCGGCCGACACCCACTCCCGCTGAA	16	<nnxnp>	nn	116	np	149		
>ref-65661	CAGACAGCTACATTCTCTCCGTGGAGA	3	<nnxnp>	nn	75	np	175		
>ref-65661	CAGACAGCTACATTCTCTCCGTGGAGA	5	<hkxhk>	hh	35	hk	223	kk	0
>ref-65661	CAGACAGCTACATTCTCTCCGTGGAGA	23	<hkxhk>	hh	92	hk	153	kk	0
>ref-65673	GTACCCCTAACCCTCCCTCCCCTTCCC	2	<lmxll>	ll	104	lm	135		
>ref-65674	AGATCTTTGACAGCATCTCCTATAGCA	14	<lmxll>	ll	144	lm	117		
>ref-65675	CCCTCCTGCACTATCCCTCCGCTTCCC	14	<lmxll>	ll	138	lm	128		
>ref-65682	CCCTCAAACACCATTCCTCCCTTTCCT	4	<lmxll>	ll	117	lm	147		
>ref-65683	GATTCACACACTCACTCTCCCACTTAC	1	<hkxhk>	hh	47	hk	141	kk	72
>ref-65688	GCTAATATTACGCGGCCTCCCCTTCCC	15	<nnxnp>	nn	113	np	138		
>ref-65715	CACACACACACATAGACTCCTCCATAG	1	<lmxll>	ll	112	lm	119		
>ref-65715	CACACACACACATAGACTCCTCCATAG	5	<lmxll>	ll	122	lm	114		
>ref-65730	TTCCTCTTTACCTTTCCTCCCTTTCCC	3	<nnxnp>	nn	120	np	130		
>ref-65730	TTCCTCTTTACCTTTCCTCCCTTTCCC	13	<nnxnp>	nn	119	np	130		
>ref-65738	TTACCTTGCACTCACCCTCCCTCTCCC	3	<hkxhk>	hh	45	hk	150	kk	52
>ref-65746	TCGTTCCTCACCCCACCTCCTCACCCC	25	<nnxnp>	nn	114	np	136		
>ref-65751	GCCACTTAAACTTCTCCTCCATCTTCA	8	<nnxnp>	nn	132	np	134		
>ref-65764	AGTCCTCACACTCACTCTCCCTCACTC	25	<hkxhk>	hh	0	hk	272	kk	0

>ref-65777	AACTTAATAACTCCCCCTCCCTTCCCC	24	<hkxhk>	hh	49	hk	129	kk	59
>ref-65812	TGTCGTTTTACTCCGTCTCCTTGCAAA	23	<nnxnp>	nn	148	np	114		
>ref-65826	CCAACAAAAACGTATTCTCCATCGATG	13	<nnxnp>	nn	142	np	107		
>ref-65855	ACTAACGCAACCCCTCCTCCTCTTCTA	8	<hkxhk>	hh	40	hk	138	kk	75
>ref-65864	TGTGGACGCACACGCCCTCCCATGATT	21	<hkxhk>	hh	1	hk	150	kk	119
>ref-65866	TCTTCAGCCACCTTTCCTCCTCCTTTC	3	<nnxnp>	nn	7	np	261		
>ref-65866	TCTTCAGCCACCTTTCCTCCTCCTTTC	22	<nnxnp>	nn	178	np	84		
>ref-65871	TACCCCCCCACCTCCTCTCCAAGCCTT	16	<nnxnp>	nn	122	np	134		
>ref-65875	CACCTCCCCACTCTTGCTCCATTCCTC	22	<lmxll>	ll	123	lm	140		
>ref-65886	CTGAGAATCACTTTCACTCCCCGGCCT	3	<nnxnp>	nn	271	np	0		
>ref-65886	CTGAGAATCACTTTCACTCCCCGGCCT	24	<nnxnp>	nn	271	np	0		
>ref-65911	AGGGAGCAAACATTCTCTCCCTTTCCG	1	<hkxhk>	hh	58	hk	134	kk	61
>ref-65970	CCTTCCTTGACTCTCGCTCCCTGCATT	27	<lmxll>	ll	134	lm	133		
>ref-65982	TCCATCCCCACCCTGCCTCCCCTCATC	5	<nnxnp>	nn	19	np	202		
>ref-65982	TCCATCCCCACCCTGCCTCCCCTCATC	8	<nnxnp>	nn	195	np	61		
>ref-65986	TTCCTCCTTACTCCCTCTCCCCTCCCT	12	<lmxll>	ll	178	lm	67		
>ref-65986	TTCCTCCTTACTCCCTCTCCCCTCCCT	27	<nnxnp>	nn	124	np	121		
>ref-66004	CACACACACACCCGTCCTCCAAGGAAA	12	<lmxll>	ll	7	lm	233		
>ref-66004	CACACACACACCCGTCCTCCAAGGAAA	21	<lmxll>	ll	7	lm	233		
>ref-66020	GAATCTGTCACGCGACCTCCCTCGCAC	2	<hkxhk>	hh	60	hk	142	kk	65
>ref-66038	TCGATTTACACTCCTCCTCCTCCCTTC	3	<nnxnp>	nn	105	np	150		
>ref-66042	ACAATTATTACCCTCTCTCCCTGTTTT	2	<nnxnp>	nn	228	np	0		
>ref-66043	TTCTCCCATACCCTTCCTCCCTATGAT	4	<lmxll>	ll	120	lm	118		
>ref-66043	TTCTCCCATACCCTTCCTCCCTATGAT	14	<lmxll>	ll	118	lm	117		
>ref-66044	TCCTGCATCACTTTCTCTCCCCTCAAT	8	<nnxnp>	nn	121	np	148		
>ref-66079	TCCTCTTCCACCTTCCCTCCCCAACCT	23	<nnxnp>	nn	215	np	7		
>ref-66082	CCTACTGTTACGCGTTCTCCAGCACAA	3	<hkxhk>	hh	61	hk	153	kk	45
>ref-66093	CCCTCTACCACAACCTCTCCCCCTCTA	24	<lmxll>	ll	9	lm	262		
>ref-66093	CCCTCTACCACAACCTCTCCCCCTCTA	27	<lmxll>	ll	8	lm	263		
>ref-66103	TGTCTTAGCACTTCCTCTCCTTGTGGA	5	<hkxhk>	hh	0	hk	270	kk	2
>ref-66103	TGTCTTAGCACTTCCTCTCCTTGTGGA	15	<hkxhk>	hh	0	hk	270	kk	1
>ref-66103	TGTCTTAGCACTTCCTCTCCTTGTGGA	27	<hkxhk>	hh	0	hk	273	kk	0
>ref-66122	AATAAGCACACAAATACTCCTACACGC	15	<lmxll>	ll	130	lm	135		
>ref-66180	TACCTTACAACCTTCTCTCCCCTTCTC	8	<lmxll>	ll	124	lm	141		
>ref-66182	CCTATTTCCACGTCCTCTCCTGTCCCA	13	<nnxnp>	nn	134	np	127		
>ref-66196	ATCGCACAAACCGTATCTCCTAGCGAG	6	<lmxll>	ll	119	lm	145		
>ref-66200	CTCTCATTCACTTCCTCTCCTTCAAAT	26	<lmxll>	ll	127	lm	115		
>ref-66236	TGCCAAACGACCCTTCCTCCTCCTCTA	4	<nnxnp>	nn	134	np	126		

>ref-66241	TCGCCCTCCACTTCTTCTCCTTTCTTC	3	<lmxll>	ll	21	lm	236		
>ref-66241	TCGCCCTCCACTTCTTCTCCTTTCTTC	9	<lmxll>	ll	24	lm	235		
>ref-66269	CTCGAATCCACTCGACCTCCAACCTCC	5	<lmxll>	ll	0	lm	272		
>ref-66269	CTCGAATCCACTCGACCTCCAACCTCC	7	<lmxll>	ll	0	lm	272		
>ref-66294	AAGATAACGACTCTGCCTCCTTTGTCC	1	<nnxnp>	nn	114	np	141		
>ref-66307	CTTCCACACACTCATCCTCCATCCCCC	9	<lmxll>	ll	142	lm	127		
>ref-66308	CTCTCCACCACTCCGACTCCGACTCCC	13	<nnxnp>	nn	111	np	145		
>ref-66316	CACATCTTCACACACCCTCCTCTCCAT	14	<hkxhk>	hh	54	hk	148	kk	62
>ref-66327	TATTTGTTGACCGATACTCCCTTTGCT	21	<hkxhk>	hh	2	hk	110	kk	107
>ref-66347	ATTTGCTTCACTCCCTCTCCTTCTTTC	22	<nnxnp>	nn	134	np	107		
>ref-66354	CTCTCTCTTACTTCATCTCCACCTAAA	23	<hkxhk>	hh	65	hk	139	kk	43
>ref-66360	CCTCCCCCCACCACACCTCCATTGTCA	16	<nnxnp>	nn	117	np	144		
>ref-66364	AGCCCCGGCACGAAACCTCCTGCTATC	5	<nnxnp>	nn	112	np	144		
>ref-66368	CCCCTATTCACTGGCACTCCTTTATCG	15	<nnxnp>	nn	125	np	109		
>ref-66411	TGTTCTCTCACTTCGCCTCCTGGGAAA	25	<lmxll>	ll	125	lm	131		
>ref-66421	TCATGCAGGACCTTTTCTCCTTCAGGC	25	<nnxnp>	nn	127	np	137		
>ref-66442	TCCTTCTACACTCGTCCTCCGTCACTA	22	<lmxll>	ll	130	lm	126		
>ref-66442	TCCTTCTACACTCGTCCTCCGTCACTA	26	<lmxll>	ll	131	lm	127		
>ref-66466	CCTCACCCTACCCTTACTCCTTCTTTC	9	<nnxnp>	nn	250	np	0		
>ref-66512	ACCCAAGCCACTGCCTCTCCCTCCCCA	23	<lmxll>	ll	113	lm	139		
>ref-66557	CCCTCTCGGACCTGAACTCCTCCGGGC	24	<nnxnp>	nn	272	np	0		
>ref-66583	AATGCATCCACTGTGTCTCCACGTGAG	3	<nnxnp>	nn	126	np	137		
>ref-66591	GTCTTCTTAACGCTTCCTCCCTCTCCT	24	<hkxhk>	hh	49	hk	133	kk	73
>ref-66602	CTCCGACTCACTCCACCTCCTCCTCTC	2	<nnxnp>	nn	121	np	113		
>ref-66606	AAGAAAAGCACAAGCACTCCCACCTCT	14	<hkxhk>	hh	0	hk	197	kk	40
>ref-66606	AAGAAAAGCACAAGCACTCCCACCTCT	23	<hkxhk>	hh	3	hk	255	kk	0
>ref-66613	TTCCTCCTAACTTCTCCTCCCTATCCT	8	<lmxll>	ll	268	lm	0		
>ref-66629	TTCTCACAGACTCTCTCTCCATTGCTC	6	<hkxhk>	hh	52	hk	128	kk	84
>ref-66629	TTCTCACAGACTCTCTCTCCATTGCTC	14	<hkxhk>	hh	84	hk	128	kk	52
>ref-66633	TCAATTTCCACTCTTTCTCCATCACCA	1	<nnxnp>	nn	143	np	121		
>ref-66675	CTATTTCCCACTCTCGCTCCTTTCTCT	24	<lmxll>	ll	121	lm	128		
>ref-66686	AGACACTGCACTCTCTCTCCCTCTCTC	9	<hkxhk>	hh	2	hk	270	kk	0
>ref-66686	AGACACTGCACTCTCTCTCCCTCTCTC	13	<nnxnp>	nn	138	np	130		
>ref-66686	AGACACTGCACTCTCTCTCCCTCTCTC	23	<hkxhk>	hh	2	hk	270	kk	0
>ref-66691	ATCTCACCTACAGTACCTCCATATAAT	1	<hkxhk>	hh	6	hk	240	kk	0
>ref-66691	ATCTCACCTACAGTACCTCCATATAAT	9	<nnxnp>	nn	56	np	165		
>ref-66691	ATCTCACCTACAGTACCTCCATATAAT	24	<nnxnp>	nn	190	np	40		
>ref-66691	ATCTCACCTACAGTACCTCCATATAAT	27	<hkxhk>	hh	0	hk	150	kk	75

>ref-66701	CCCATCCTCACCATTCCTCCTTTTTAA	3	<hkxhk>	hh	68	hk	140	kk	39
>ref-66701	CCCATCCTCACCATTCCTCCTTTTTAA	26	<hkxhk>	hh	68	hk	139	kk	39
>ref-66723	AGTTATCAAACGCACGCTCCAAATATC	1	<hkxhk>	hh	1	hk	235	kk	9
>ref-66723	AGTTATCAAACGCACGCTCCAAATATC	8	<hkxhk>	hh	1	hk	234	kk	10
>ref-66724	TATGACACCACCTTAGCTCCTTTCCTC	5	<nnxnp>	nn	136	np	126		
>ref-66735	AAGATAAACACATAAGCTCCGTGTATA	26	<hkxhk>	hh	137	hk	124	kk	0
>ref-66736	CTCTCTCTCACCCTCTCTCCAGCGTTG	5	<nnxnp>	nn	127	np	119		
>ref-66736	CTCTCTCTCACCCTCTCTCCAGCGTTG	25	<nnxnp>	nn	126	np	120		
>ref-66750	TGGCCAGAAACCGGGACTCCAGAAGTA	12	<lmxll>	ll	113	lm	131		
>ref-66763	CACTTTTTTACCTCTTCTCCTTCCCCC	24	<nnxnp>	nn	225	np	0		
>ref-66781	CCCTTAACCACCCGACCTCCCTCTCCT	15	<hkxhk>	hh	8	hk	260	kk	0
>ref-66792	GACACAAGTACCCGCTCTCCCTCGCTC	12	<lmxll>	ll	131	lm	132		
>ref-66795	ACCCCTCCTACCTCCCCTCCCTTCACC	2	<lmxll>	ll	270	lm	0		
>ref-66798	AGAATGTAAACCAGCGCTCCTCGAAAG	25	<lmxll>	ll	110	lm	129		
>ref-66807	TTATATCTAACCCATTCTCCTCTCCCT	23	<lmxll>	ll	130	lm	136		
>ref-66816	TGCTCATCCACACATCCTCCACACATG	9	<hkxhk>	hh	0	hk	271	kk	2
>ref-66836	TGTTTGCTCACACCTCCTCCTCTTGCG	25	<lmxll>	ll	131	lm	139		
>ref-66838	AAAGCAATAACACGGCCTCCAATCGTG	26	<nnxnp>	nn	272	np	0		
>ref-66843	TCCTCCTCTACTCGTCCTCCCAATTCA	14	<lmxll>	ll	109	lm	142		
>ref-66864	CCACTCCCCACATCCGCTCCATCACTT	3	<nnxnp>	nn	128	np	126		
>ref-66866	ACATCCATCACCCCGTCTCCATCCATC	1	<lmxll>	ll	116	lm	137		
>ref-66887	TTCTACGGCACAGAAACTCCGCCCCCA	14	<lmxll>	ll	249	lm	0		
>ref-66893	CAGACACTCACCTTTGCTCCAAGTCCA	3	<nnxnp>	nn	262	np	0		
>ref-66897	CACAAGTCCACTTACTCTCCCCGTGTG	15	<lmxll>	ll	122	lm	149		
>ref-66937	TCCTTGGCGACCTTTCCTCCGTGTCTT	15	<nnxnp>	nn	134	np	136		
>ref-66943	CCCTCTACTACTACCTCTCCTTCAGCT	22	<nnxnp>	nn	109	np	134		
>ref-66945	CCCCACAGTACCCTCCCTCCTTTCCCC	14	<lmxll>	ll	149	lm	103		
>ref-66990	CCTAATAGCACAGAACCTCCTGTTCTA	27	<nnxnp>	nn	128	np	92		
>ref-66998	ATCGCGCGAACTCCACCTCCACAGGTC	21	<hkxhk>	hh	48	hk	128	kk	50
>ref-67002	GAGAGCAAGACATGCCCTCCCCTTAGA	6	<nnxnp>	nn	126	np	143		
>ref-67006	ATGCTGCTGACTTTCACTCCAAAGTAT	16	<nnxnp>	nn	136	np	123		
>ref-67023	TGGGTTGGTACACCTACTCCTGGTCTG	6	<lmxll>	ll	5	lm	267		
>ref-67023	TGGGTTGGTACACCTACTCCTGGTCTG	26	<lmxll>	ll	6	lm	265		
>ref-67038	CAGTACTAGACCGTATCTCCCTCTTTG	27	<nnxnp>	nn	130	np	118		
>ref-67051	ATCCGCCTGACATCCCCTCCTGAGATC	6	<lmxll>	ll	122	lm	139		
>ref-67054	CCCACCCTTACCTTCCCTCCTTCACCC	8	<nnxnp>	nn	148	np	121		
>ref-67077	CACGTGTACACCATGTCTCCCGTGACT	13	<lmxll>	ll	143	lm	126		
>ref-67087	GCACGCGTTACTTCCGCTCCCGGTTAC	23	<lmxll>	ll	145	lm	127		

>ref-67092	CTTCCTATTACCCTCCCTCCCCTCCTT	2	<lmxll>	ll	29	lm	226		
>ref-67092	CTTCCTATTACCCTCCCTCCCCTCCTT	5	<nnxnp>	nn	267	np	0		
>ref-67092	CTTCCTATTACCCTCCCTCCCCTCCTT	7	<lmxll>	ll	28	lm	225		
>ref-67098	CCCACACGTACACGAGCTCCCATACCG	3	<lmxll>	ll	119	lm	105		
>ref-67107	CATTTATCCACATCCACTCCGCATCTC	24	<hkxhk>	hh	61	hk	151	kk	55
>ref-67125	GGCATACACACGCGTGCTCCGGGCGTA	7	<nnxnp>	nn	151	np	117		
>ref-67189	CCCACTTCAACACCTCCTCCACCACCT	14	<lmxll>	ll	270	lm	0		
>ref-67195	ATTTCAAACACGCCTCCTCCCCTGTTT	24	<nnxnp>	nn	133	np	134		
>ref-67196	ATACTTACGACATGAACTCCCCCTCGT	25	<lmxll>	ll	120	lm	127		
>ref-67210	CCCTCTGAAACAAGCCCTCCTCATGCA	7	<nnxnp>	nn	116	np	140		
>ref-67210	CCCTCTGAAACAAGCCCTCCTCATGCA	23	<nnxnp>	nn	113	np	144		
>ref-67222	GTCACCGTTACATTTCCTCCCATAGGA	1	<nnxnp>	nn	129	np	108		
>ref-67222	GTCACCGTTACATTTCCTCCCATAGGA	6	<lmxll>	ll	109	lm	118		
>ref-67253	AATTTGGAAACAACGACTCCTATCGCT	12	<nnxnp>	nn	96	np	154		
>ref-67253	AATTTGGAAACAACGACTCCTATCGCT	14	<nnxnp>	nn	140	np	116		
>ref-67253	AATTTGGAAACAACGACTCCTATCGCT	22	<hkxhk>	hh	136	hk	113	kk	0
>ref-67253	AATTTGGAAACAACGACTCCTATCGCT	24	<hkxhk>	hh	136	hk	113	kk	0
>ref-67256	AGCATGTCTACCTGTCCTCCTCCGTCT	4	<hkxhk>	hh	67	hk	152	kk	49
>ref-67259	CGCCCTACCACCCTGCCTCCACCCTCC	21	<hkxhk>	hh	2	hk	270	kk	0
>ref-67297	CGCGCCCAAACTTTGCCTCCACCTCCT	2	<hkxhk>	hh	0	hk	271	kk	1
>ref-67297	CGCGCCCAAACTTTGCCTCCACCTCCT	16	<nnxnp>	nn	241	np	25		
>ref-67299	TCCCCTCCCACACGTCCTCCGCCCATA	26	<nnxnp>	nn	273	np	0		
>ref-67299	TCCCCTCCCACACGTCCTCCGCCCATA	27	<nnxnp>	nn	273	np	0		
>ref-67304	GCATTTTCAACATCTTCTCCTGAGCTG	21	<hkxhk>	hh	39	hk	143	kk	65
>ref-67318	CCACCCTCCACCTCCACTCCCTTCCTC	6	<nnxnp>	nn	160	np	78		
>ref-67336	TTGTTTAGAACACGTCCTCCTTCCCTC	15	<hkxhk>	hh	63	hk	146	kk	55
>ref-67343	CACAATGGCACTCCGGCTCCTCAGGGC	14	<lmxll>	ll	145	lm	124		
>ref-67347	CACACATATACATGGCCTCCGCTGTGC	26	<nnxnp>	nn	112	np	135		
>ref-67347	CACACATATACATGGCCTCCGCTGTGC	27	<nnxnp>	nn	112	np	134		
>ref-67348	TTCAAACTGACCTTCTCTCCCTCCCTT	7	<nnxnp>	nn	125	np	123		
>ref-67359	GCCTCCCCAACCCTCTCTCCCCTCACT	4	<lmxll>	ll	6	lm	263		
>ref-67359	GCCTCCCCAACCCTCTCTCCCCTCACT	5	<nnxnp>	nn	141	np	124		
>ref-67359	GCCTCCCCAACCCTCTCTCCCCTCACT	7	<lmxll>	ll	6	lm	263		
>ref-67359	GCCTCCCCAACCCTCTCTCCCCTCACT	25	<nnxnp>	nn	142	np	123		
>ref-67378	CACAGAAACACCCCACCTCCACTATAT	23	<lmxll>	ll	110	lm	111		
>ref-67394	AACAGAATGACAGTGCCTCCAGATCCC	1	<nnxnp>	nn	134	np	125		
>ref-67400	TTGCCTTGTACCCGCACTCCCTCGACC	7	<nnxnp>	nn	133	np	132		
>ref-67409	AACAGCCACACAGTTTCTCCAACAATC	8	<hkxhk>	hh	73	hk	139	kk	41

>ref-67411	CTCCGCTTCACTTCTTCTCCTCCATCT	25	<nnxnp>	nn	124	np	122		
>ref-67416	AAGCAACTGACCTCGCCTCCGTTAACA	12	<nnxnp>	nn	142	np	115		
>ref-67416	AAGCAACTGACCTCGCCTCCGTTAACA	21	<lmxll>	ll	130	lm	130		
>ref-67422	AATTCCTTTACCACACCTCCACCTCAT	3	<nnxnp>	nn	110	np	112		
>ref-67422	AATTCCTTTACCACACCTCCACCTCAT	5	<nnxnp>	nn	110	np	112		
>ref-67422	AATTCCTTTACCACACCTCCACCTCAT	26	<lmxll>	ll	113	lm	109		
>ref-67425	TACCTTTCTACCTCTTCTCCTTCCCTT	5	<hkxhk>	hh	0	hk	205	kk	24
>ref-67425	TACCTTTCTACCTCTTCTCCTTCCCTT	13	<hkxhk>	hh	0	hk	205	kk	24
>ref-67435	TCATCCTCCACCTCTCCTCCCTTTCCT	6	<hkxhk>	hh	2	hk	267	kk	0
>ref-67435	TCATCCTCCACCTCTCCTCCCTTTCCT	21	<hkxhk>	hh	0	hk	267	kk	2
>ref-67449	AGGAATATCACATCCTCTCCATTATTA	21	<hkxhk>	hh	42	hk	126	kk	69
>ref-67449	AGGAATATCACATCCTCTCCATTATTA	26	<nnxnp>	nn	114	np	122		
>ref-67479	AGAGCAATGACATCATCTCCTTACATA	2	<lmxll>	ll	136	lm	110		
>ref-67516	GAAATGCATACCAATCCTCCCCCATCT	8	<lmxll>	ll	138	lm	131		
>ref-67528	TCCCTCATCACTCATTCTCCTCCTTTT	8	<hkxhk>	hh	3	hk	257	kk	2
>ref-67528	TCCCTCATCACTCATTCTCCTCCTTTT	23	<lmxll>	ll	13	lm	245		
>ref-67543	AGACATGTCACAATGTCTCCTTTCCTT	27	<lmxll>	ll	123	lm	137		
>ref-67562	TTTTAGCCAACGAAACCTCCTTTTAAC	26	<hkxhk>	hh	51	hk	142	kk	40
>ref-67565	TCCCCCCCAACACACCCTCCTCCTCCC	15	<nnxnp>	nn	1	np	239		
>ref-67581	TCATCCTCCACTCCTTCTCCCCTTTCT	9	<lmxll>	ll	134	lm	118		
>ref-67590	TCTTCCTCCACGTCTCCTCCAGCTCCA	23	<lmxll>	ll	120	lm	146		
>ref-67590	TCTTCCTCCACGTCTCCTCCAGCTCCA	26	<lmxll>	ll	120	lm	146		
>ref-67601	CTAGGTGTGACCTCCTCTCCTTGCTCC	5	<nnxnp>	nn	132	np	136		
>ref-67602	GTTCTCCCCACCTTCCCTCCCTACCTT	12	<nnxnp>	nn	124	np	131		
>ref-67602	GTTCTCCCCACCTTCCCTCCCTACCTT	27	<lmxll>	ll	141	lm	123		
>ref-67608	CGCACTTACACGCCCCCTCCCTCGCAC	8	<lmxll>	ll	129	lm	141		
>ref-67623	GTGCCCAGTACTTCATCTCCAATGGGC	9	<lmxll>	ll	272	lm	0		
>ref-67644	CACACACAAACTCAACCTCCTCCCAAG	24	<lmxll>	ll	135	lm	130		
>ref-67651	CCTCCCCCTACCTCATCTCCTCTCTTT	1	<lmxll>	ll	6	lm	260		
>ref-67651	CCTCCCCCTACCTCATCTCCTCTCTTT	2	<hkxhk>	hh	71	hk	190	kk	0
>ref-67651	CCTCCCCCTACCTCATCTCCTCTCTTT	14	<nnxnp>	nn	147	np	113		
>ref-67651	CCTCCCCCTACCTCATCTCCTCTCTTT	24	<nnxnp>	nn	147	np	113		
>ref-67651	CCTCCCCCTACCTCATCTCCTCTCTTT	26	<lmxll>	ll	7	lm	260		
>ref-67681	CTTCCTCTCACTCTTCCTCCACCATTC	7	<hkxhk>	hh	214	hk	40	kk	0
>ref-67681	CTTCCTCTCACTCTTCCTCCACCATTC	14	<hkxhk>	hh	0	hk	46	kk	211
>ref-67681	CTTCCTCTCACTCTTCCTCCACCATTC	23	<nnxnp>	nn	131	np	139		
>ref-67698	TTCCCTCCCACACCTCCTCCCTCACCT	4	<lmxll>	ll	260	lm	1		
>ref-67708	CACCTTTTAACCTCCCCTCCTCCCTTC	3	<nnxnp>	nn	122	np	119		

>ref-67711	TTACAGATTACATGGACTCCCGTATCT	7	<hkxhk>	hh	0	hk	271	kk	1
>ref-67713	CTATATATAACGCCCCCTCCTCCTCCC	23	<lmxll>	ll	135	lm	124		
>ref-67716	CTCTTTCTCACACTCCCTCCGTACATT	1	<nnxnp>	nn	113	np	147		
>ref-67716	CTCTTTCTCACACTCCCTCCGTACATT	21	<nnxnp>	nn	113	np	147		
>ref-67720	GTCCCTTCCACGCTCCCTCCTCGCCCC	2	<lmxll>	ll	116	lm	135		
>ref-67720	GTCCCTTCCACGCTCCCTCCTCGCCCC	16	<lmxll>	ll	120	lm	131		
>ref-67721	CATTGACCTACTCTCTCTCCTCCTCTT	16	<lmxll>	ll	221	lm	44		
>ref-67726	CAAACACACACTCACGCTCCCATCACA	12	<nnxnp>	nn	266	np	0		
>ref-67745	CCCTCATCCACACCTTCTCCTCCGTCT	1	<lmxll>	ll	115	lm	149		
>ref-67750	AAACCTAACACAACATCTCCTCTCATA	25	<lmxll>	ll	143	lm	119		
>ref-67766	CTATCCCGAACTCTTACTCCTCGTTGA	1	<nnxnp>	nn	190	np	63		
>ref-67781	AAAGGCATGACAGTTTCTCCTTCCTAA	4	<nnxnp>	nn	128	np	126		
>ref-67797	TAACCCCTCACCCCTTCTCCCTCACTA	27	<lmxll>	ll	119	lm	138		
>ref-67810	AACACTGCTACTACTACTCCCTCCTCA	21	<hkxhk>	hh	62	hk	190	kk	0
>ref-67817	CTTATTTTTACCTCCCCTCCCCCTTCC	1	<nnxnp>	nn	252	np	0		
>ref-67819	ACGTAGCGGACGTGACCTCCAGCGTCC	5	<hkxhk>	hh	0	hk	271	kk	0
>ref-67825	CCTACCCCTACCCCTCCTCCTTCTACA	4	<lmxll>	ll	241	lm	5		
>ref-67825	CCTACCCCTACCCCTCCTCCTTCTACA	25	<lmxll>	ll	228	lm	8		
>ref-67830	TCTTTCGTTACCCATTCTCCTAACCCT	5	<nnxnp>	nn	114	np	137		
>ref-67830	TCTTTCGTTACCCATTCTCCTAACCCT	6	<nnxnp>	nn	114	np	137		
>ref-67834	CTCTCTTCCACCCACGCTCCTTCATCC	8	<nnxnp>	nn	119	np	141		
>ref-67846	TCTTTTCTTACGCCCCCTCCTTTCCCC	3	<hkxhk>	hh	56	hk	135	kk	55
>ref-67853	GCCTCTTCAACTTACCCTCCTATTCCT	26	<nnxnp>	nn	270	np	0		
>ref-67853	GCCTCTTCAACTTACCCTCCTATTCCT	27	<nnxnp>	nn	270	np	0		
>ref-67884	AAAGTTGTGACGTCATCTCCCCCTGAA	1	<hkxhk>	hh	58	hk	143	kk	60
>ref-67884	AAAGTTGTGACGTCATCTCCCCCTGAA	2	<hkxhk>	hh	58	hk	143	kk	60
>ref-67907	AAGAAAGCAACGTTTCCTCCACAATAA	7	<nnxnp>	nn	140	np	121		
>ref-67913	TTCTCCTCTACCTCCTCTCCTTACTCC	5	<lmxll>	ll	153	lm	106		
>ref-67917	TTGAGCGCAACTCCAACTCCAGCGTAG	9	<hkxhk>	hh	116	hk	146	kk	0
>ref-67921	AGCCTCCCAACGCCAACTCCTGCAAGA	5	<hkxhk>	hh	63	hk	131	kk	61
>ref-67966	TATTAGAGTACTAGCTCTCCATTCATC	4	<hkxhk>	hh	7	hk	264	kk	0
>ref-67966	TATTAGAGTACTAGCTCTCCATTCATC	5	<hkxhk>	hh	68	hk	192	kk	0
>ref-67966	TATTAGAGTACTAGCTCTCCATTCATC	14	<hkxhk>	hh	0	hk	264	kk	7
>ref-67971	GCGCCTGTGACTCGAACTCCCGAACTC	9	<nnxnp>	nn	249	np	0		
>ref-67972	TCCTCATTTACTGCCTCTCCTCGAAGA	22	<hkxhk>	hh	53	hk	157	kk	51
>ref-67974	CCGATGCTTACAGAAGCTCCTTTGATC	3	<lmxll>	ll	128	lm	132		
>ref-67974	CCGATGCTTACAGAAGCTCCTTTGATC	7	<lmxll>	ll	130	lm	126		
>ref-67985	TTCGTCTAGACTGTTTCTCCGTTCGTG	1	<lmxll>	ll	130	lm	131		

>ref-68000	CTGTGGATTACCAGTTCTCCAAAGCCT	9	<nnxnp>	nn	264	np	0		
>ref-68007	CTTATACTCACTCACACTCCGACATAT	1	<nnxnp>	nn	0	np	252		
>ref-68008	CTAGCTTGAACACGCTCTCCCCTTCAC	4	<nnxnp>	nn	267	np	0		
>ref-68013	GGAGTAACCACCAACCCTCCCAAAATT	15	<nnxnp>	nn	54	np	204		
>ref-68013	GGAGTAACCACCAACCCTCCCAAAATT	25	<lmxll>	ll	234	lm	35		
>ref-68035	CCACGCTATACCATACCTCCCTACTGC	24	<hkxhk>	hh	0	hk	272	kk	0
>ref-68035	CCACGCTATACCATACCTCCCTACTGC	26	<hkxhk>	hh	0	hk	271	kk	0
>ref-68046	CATCTTGGGACGGCTCCTCCTCCTCCT	1	<nnxnp>	nn	244	np	0		
>ref-68046	CATCTTGGGACGGCTCCTCCTCCTCCT	2	<nnxnp>	nn	246	np	0		
>ref-68069	TACACTTACACTTTCGCTCCATTCTTA	2	<hkxhk>	hh	3	hk	268	kk	0
>ref-68099	TCTATTACTACAGCTGCTCCCGGTCTA	1	<lmxll>	ll	100	lm	141		
>ref-68111	TGCGACGAAACGTGCCCTCCTGAGTAC	24	<lmxll>	ll	137	lm	83		
>ref-68116	AATGCATACACTCCTCCTCCTGATAGA	7	<lmxll>	ll	140	lm	130		
>ref-68125	CACCTTGCTACCTGTGCTCCTTGGTGT	25	<hkxhk>	hh	0	hk	268	kk	4
>ref-68125	CACCTTGCTACCTGTGCTCCTTGGTGT	27	<hkxhk>	hh	0	hk	267	kk	4
>ref-68139	TAAAGCTTAACCTTCGCTCCTTCTGTA	5	<hkxhk>	hh	46	hk	118	kk	62
>ref-68154	CTCTCGCCAACACCAACTCCGCCTGTT	6	<lmxll>	ll	114	lm	152		
>ref-68175	AAGCTGACGACTTGTGCTCCGCCCTGG	27	<nnxnp>	nn	267	np	0		
>ref-68201	TTCTCCATCACTGCGCCTCCCCCACCC	25	<hkxhk>	hh	0	hk	273	kk	0
>ref-68209	CATGCAAGGACTTACTCTCCTTGTTTA	6	<hkxhk>	hh	127	hk	133	kk	0
>ref-68209	CATGCAAGGACTTACTCTCCTTGTTTA	12	<hkxhk>	hh	0	hk	269	kk	4
>ref-68209	CATGCAAGGACTTACTCTCCTTGTTTA	14	<hkxhk>	hh	0	hk	272	kk	0
>ref-68209	CATGCAAGGACTTACTCTCCTTGTTTA	23	<nnxnp>	nn	220	np	44		
>ref-68241	TCTCCACCCACTCCATCTCCCCATTCA	1	<lmxll>	ll	130	lm	96		
>ref-68241	TCTCCACCCACTCCATCTCCCCATTCA	5	<lmxll>	ll	129	lm	96		
>ref-68241	TCTCCACCCACTCCATCTCCCCATTCA	21	<lmxll>	ll	104	lm	119		
>ref-68255	CCTCCTCTAACCCCAGCTCCCCCTCCC	14	<lmxll>	ll	143	lm	106		
>ref-68266	CACACACTCACACACGCTCCATATAAC	1	<hkxhk>	hh	53	hk	136	kk	61
>ref-68266	CACACACTCACACACGCTCCATATAAC	22	<hkxhk>	hh	53	hk	137	kk	60
>ref-68283	AGATGGCGGACGGAGGCTCCTTCGTAC	13	<lmxll>	ll	151	lm	121		
>ref-68290	CAACGCATCACTCTCCCTCCCCATCCC	22	<nnxnp>	nn	142	np	131		
>ref-68303	CCTTTCGCCACCCCTGCTCCGTTTAGC	25	<hkxhk>	hh	71	hk	125	kk	67
>ref-68307	CTTCCCTTCACTCCTTCTCCCTCGCAT	4	<nnxnp>	nn	129	np	137		
>ref-68324	TAATGTCTTACAAATCCTCCTGGAAAT	23	<hkxhk>	hh	2	hk	271	kk	0
>ref-68327	GAGCCGCCGACGAGGCCTCCATGCCCC	14	<nnxnp>	nn	136	np	133		
>ref-68333	AGCCCAGTCACAAACCCTCCTACAATC	12	<hkxhk>	hh	5	hk	264	kk	0
>ref-68333	AGCCCAGTCACAAACCCTCCTACAATC	15	<lmxll>	ll	39	lm	213		
>ref-68348	CAGTTACCCACTAATCCTCCATTATAG	25	<lmxll>	ll	177	lm	65		

>ref-68348	CAGTTACCCACTAATCCTCCATTATAG	26	<nnxnp>	nn	199	np	39		
>ref-68359	TCGTTACCCACGCCTGCTCCTTCGGTA	13	<lmxll>	ll	148	lm	122		
>ref-68359	TCGTTACCCACGCCTGCTCCTTCGGTA	16	<lmxll>	ll	148	lm	122		
>ref-68361	CCTTCATCCACATTCCCTCCCCTCTTT	4	<lmxll>	ll	70	lm	193		
>ref-68361	CCTTCATCCACATTCCCTCCCCTCTTT	6	<lmxll>	ll	70	lm	193		
>ref-68362	CCTTTCACCACACTCTCTCCTCTCTTC	26	<nnxnp>	nn	92	np	128		
>ref-68363	GGGGACCCCACTCGAACTCCTCGCAGT	9	<lmxll>	ll	137	lm	132		
>ref-68386	CACACACACACGCGCGCTCCTCTTATT	12	<hkxhk>	hh	66	hk	144	kk	58
>ref-68417	CTGCCTTTTACCCTCTCTCCTTCCTCT	2	<nnxnp>	nn	144	np	111		
>ref-68417	CTGCCTTTTACCCTCTCTCCTTCCTCT	3	<nnxnp>	nn	135	np	118		
>ref-68417	CTGCCTTTTACCCTCTCTCCTTCCTCT	23	<lmxll>	ll	76	lm	184		
>ref-68417	CTGCCTTTTACCCTCTCTCCTTCCTCT	24	<lmxll>	ll	80	lm	183		
>ref-68430	GAGAAAAAGACCGGGCCTCCTCCCTAT	14	<hkxhk>	hh	45	hk	153	kk	51
>ref-68464	TCCTTTTCTACTCCCCCTCCCTCTACC	9	<hkxhk>	hh	0	hk	180	kk	59
>ref-68464	TCCTTTTCTACTCCCCCTCCCTCTACC	22	<hkxhk>	hh	0	hk	181	kk	57
>ref-68475	ATCTCTTCCACTTCAACTCCAGTCCAC	3	<lmxll>	ll	135	lm	127		
>ref-68510	TCACAGCTGACAGTGACTCCTTGACAG	2	<hkxhk>	hh	1	hk	271	kk	0
>ref-68514	CGTGCACACACCCTCTCTCCCCAGCTG	13	<lmxll>	ll	133	lm	132		
>ref-68519	ACAATCATTACACCATCTCCATACTTG	1	<nnxnp>	nn	262	np	0		
>ref-68519	ACAATCATTACACCATCTCCATACTTG	2	<nnxnp>	nn	261	np	0		
>ref-68524	CTCGTTCCAACATAATCTCCCTTATCA	16	<nnxnp>	nn	126	np	118		
>ref-68545	GCTTAAGGAACCATACCTCCTGAGAGA	5	<lmxll>	ll	25	lm	223		
>ref-68545	GCTTAAGGAACCATACCTCCTGAGAGA	21	<lmxll>	ll	25	lm	223		
>ref-68548	ATCTTCTGAACTACATCTCCTTTGTAT	3	<lmxll>	ll	117	lm	128		
>ref-68551	CTAAATCTCACTCTCACTCCTTCTTTC	5	<nnxnp>	nn	250	np	6		
>ref-68554	TTCATTCTCACTCTTTCTCCGCCATTC	3	<hkxhk>	hh	0	hk	262	kk	10
>ref-68554	TTCATTCTCACTCTTTCTCCGCCATTC	14	<hkxhk>	hh	0	hk	184	kk	83
>ref-68554	TTCATTCTCACTCTTTCTCCGCCATTC	21	<hkxhk>	hh	82	hk	184	kk	0
>ref-68563	CATCTATTCACCGTGTCTCCATCTTTT	2	<lmxll>	ll	110	lm	150		
>ref-68574	AACGGGATAACGCTTTCTCCCTCACGC	2	<nnxnp>	nn	133	np	137		
>ref-68589	CCAAATTACACTTCATCTCCCTAATTA	15	<hkxhk>	hh	51	hk	215	kk	1
>ref-68604	ACAAACATCACTCCATCTCCTACACAG	12	<hkxhk>	hh	0	hk	206	kk	58
>ref-68607	TTGGCCTCCACCTCCTCTCCTTGGCCT	1	<lmxll>	ll	137	lm	125		
>ref-68661	CTTCCATCTACTCTCCCTCCCGACACG	6	<hkxhk>	hh	1	hk	266	kk	3
>ref-68661	CTTCCATCTACTCTCCCTCCCGACACG	9	<hkxhk>	hh	0	hk	264	kk	4
>ref-68661	CTTCCATCTACTCTCCCTCCCGACACG	21	<hkxhk>	hh	0	hk	262	kk	2
>ref-68661	CTTCCATCTACTCTCCCTCCCGACACG	27	<hkxhk>	hh	0	hk	200	kk	47
>ref-68690	TGAGGCAAGACTAGAGCTCCCAGAAGC	23	<nnxnp>	nn	138	np	118		

>ref-68694	CACCTACCAACTCGACCTCCTCTCTCC	9	<hkxhk>	hh	64	hk	148	kk	44
>ref-68698	CCCCTATTCACCCCTTCTCCAATCCCC	8	<lmxll>	ll	101	lm	123		
>ref-68701	ACCCCACCCACTCCCTCTCCCCTTCCT	26	<lmxll>	ll	128	lm	139		
>ref-68702	AAATCTGGCACTGTGCCTCCTTCGTCC	26	<nnxnp>	nn	140	np	129		
>ref-68710	CACTGTTTCACTGTCTCTCCCTCTCCT	21	<hkxhk>	hh	0	hk	273	kk	0
>ref-68710	CACTGTTTCACTGTCTCTCCCTCTCCT	26	<hkxhk>	hh	0	hk	273	kk	0
>ref-68716	GACTTAAATACACTATCTCCACCTTTT	22	<hkxhk>	hh	17	hk	254	kk	0
>ref-68716	GACTTAAATACACTATCTCCACCTTTT	27	<hkxhk>	hh	0	hk	158	kk	98
>ref-68764	CTCTCGTAAACATCTCCTCCTCTCTCT	22	<lmxll>	ll	122	lm	138		
>ref-68799	GCCAGATGCACACAGACTCCAGTGTTC	23	<nnxnp>	nn	121	np	148		
>ref-68812	CCTCCGCGCACCCTGCCTCCCTCTCCG	16	<lmxll>	ll	147	lm	125		
>ref-68822	CTCCACCCAACCCAATCTCCTCTTATC	8	<nnxnp>	nn	125	np	112		
>ref-68824	AGCCCCCGCACCCTTTCTCCCTAATCA	22	<nnxnp>	nn	110	np	146		
>ref-68828	AGGAAAATTACACACGCTCCTCCCTAC	24	<nnxnp>	nn	120	np	129		
>ref-68831	TACAATCACACCTTCCCTCCTTCCCTC	2	<nnxnp>	nn	262	np	2		
>ref-68831	TACAATCACACCTTCCCTCCTTCCCTC	8	<nnxnp>	nn	263	np	1		
>ref-68831	TACAATCACACCTTCCCTCCTTCCCTC	16	<nnxnp>	nn	120	np	131		
>ref-68850	ATGCTTAGCACGAATCCTCCTTTTTAT	16	<lmxll>	ll	125	lm	120		
>ref-68854	ACCAAATACACCAATCCTCCTATCTCC	21	<lmxll>	ll	119	lm	142		
>ref-68859	CCAAGCAGGACGCACGCTCCTACAAAC	21	<nnxnp>	nn	122	np	146		
>ref-68871	AGAACTCCGACACCTCCTCCTCCTTTT	13	<nnxnp>	nn	123	np	135		
>ref-68873	CCGCCTTCCACTTCCTCTCCTCTTGTC	3	<nnxnp>	nn	137	np	130		
>ref-68873	CCGCCTTCCACTTCCTCTCCTCTTGTC	4	<nnxnp>	nn	137	np	130		
>ref-68883	TTCGTCCTTACCCACGCTCCCTCCTTT	4	<hkxhk>	hh	226	hk	10	kk	0
>ref-68883	TTCGTCCTTACCCACGCTCCCTCCTTT	5	<hkxhk>	hh	0	hk	10	kk	228
>ref-68895	ATTCTTTTTACTTTCCCTCCTCCCTTC	24	<nnxnp>	nn	106	np	124		
>ref-68895	ATTCTTTTTACTTTCCCTCCTCCCTTC	27	<nnxnp>	nn	108	np	124		
>ref-68896	CGGTCGGGCACTGGCGCTCCTGACAAC	16	<nnxnp>	nn	110	np	144		
>ref-68913	CTCTCTTCCACATCCTCTCCTAACCCC	5	<nnxnp>	nn	116	np	132		
>ref-68930	CACATACAAACCTGTGCTCCAACACAC	1	<hkxhk>	hh	19	hk	249	kk	0
>ref-68944	ATATATATAACAATCGCTCCAACACAC	5	<hkxhk>	hh	0	hk	244	kk	0
>ref-68964	CTGTCTCTCACACCCCCTCCCTCTCCC	14	<hkxhk>	hh	185	hk	66	kk	0
>ref-68964	CTGTCTCTCACACCCCCTCCCTCTCCC	16	<hkxhk>	hh	0	hk	258	kk	0
>ref-68967	CAAATGATAACAATCCCTCCCTCCCTC	8	<lmxll>	ll	143	lm	113		
>ref-68975	TCTCCCCTTACTCATTCTCCTTTTGTT	25	<hkxhk>	hh	0	hk	267	kk	5
>ref-68979	TACCCCTTTACCACCCCTCCCTACCTC	2	<hkxhk>	hh	1	hk	262	kk	0
>ref-68979	TACCCCTTTACCACCCCTCCCTACCTC	8	<hkxhk>	hh	0	hk	262	kk	1
>ref-68981	GTCTCTTCGACGCCTCCTCCAGCATGG	12	<nnxnp>	nn	244	np	0		

>ref-68997	ACAATAGCAACATTGTCTCCACAGAGA	26	<hkxhk>	hh	6	hk	252	kk	0
>ref-69027	GCACCTACGACCGCTGCTCCAACTACA	7	<hkxhk>	hh	96	hk	164	kk	0
>ref-69039	TTTTCCACAACCTACCCTCCTTCGCCA	14	<lmxll>	ll	107	lm	130		
>ref-69042	TTTCTCGTTACCCTTCCTCCTCTTATC	23	<nnxnp>	nn	106	np	135		
>ref-69048	CTTCTACTTACTCTTTCTCCCTCTCCG	5	<lmxll>	ll	108	lm	114		
>ref-69054	TGACCTTTAACCCAGCCTCCGGGCTAG	2	<hkxhk>	hh	1	hk	191	kk	59
>ref-69054	TGACCTTTAACCCAGCCTCCGGGCTAG	13	<hkxhk>	hh	1	hk	191	kk	59
>ref-69058	CCACTTCCTACTGCTCCTCCTTTTCTC	7	<hkxhk>	hh	63	hk	197	kk	0
>ref-69058	CCACTTCCTACTGCTCCTCCTTTTCTC	13	<hkxhk>	hh	0	hk	198	kk	58
>ref-69058	CCACTTCCTACTGCTCCTCCTTTTCTC	16	<nnxnp>	nn	7	np	256		
>ref-69058	CCACTTCCTACTGCTCCTCCTTTTCTC	27	<lmxll>	ll	128	lm	132		
>ref-69093	TACATGTGTACGTGATCTCCCTCTTTC	23	<nnxnp>	nn	126	np	124		
>ref-69124	TTATATGATACTTTCTCTCCTCCTTTG	27	<nnxnp>	nn	233	np	1		
>ref-69133	CCATCTCACACTTCCTCTCCCTCTTTT	1	<hkxhk>	hh	0	hk	266	kk	7
>ref-69156	CTGATTTTGACCCCATCTCCTCAGATG	16	<hkxhk>	hh	6	hk	217	kk	9
>ref-69175	CAGGATGCGACAGCTACTCCTCACCCA	7	<nnxnp>	nn	139	np	125		
>ref-69177	TTCCTCCCCACCCATCCTCCCTTCCTT	23	<nnxnp>	nn	155	np	106		
>ref-69199	TGATCCGCGACCATCCCTCCACCGCAC	15	<nnxnp>	nn	139	np	132		
>ref-69223	TTCGTCCCCACTTTATCTCCGGTAGCT	16	<nnxnp>	nn	96	np	164		
>ref-69233	TCTCGTGGAACGGACTCTCCTGCGCTC	24	<nnxnp>	nn	134	np	138		
>ref-69253	GATGCAACGACGTCAGCTCCGTCGATT	7	<nnxnp>	nn	128	np	136		
>ref-69253	GATGCAACGACGTCAGCTCCGTCGATT	13	<nnxnp>	nn	128	np	136		
>ref-69272	CCATTCACCACCCTTTCTCCTTCCCTC	7	<hkxhk>	hh	1	hk	264	kk	0
>ref-69272	CCATTCACCACCCTTTCTCCTTCCCTC	14	<hkxhk>	hh	0	hk	264	kk	1
>ref-69296	TTACTGTAGACCTATACTCCCTATCGT	6	<nnxnp>	nn	130	np	130		
>ref-69306	GGGAAATGAACCATATCTCCCTCGAAT	23	<lmxll>	ll	113	lm	136		
>ref-69316	TGGACCCCCACCCCCCCTCCTTGTCCC	7	<nnxnp>	nn	236	np	0		
>ref-69316	TGGACCCCCACCCCCCCTCCTTGTCCC	23	<nnxnp>	nn	230	np	0		
>ref-69319	CGAGCCCTGACGCTCTCTCCCCAATCC	2	<hkxhk>	hh	0	hk	272	kk	1
>ref-69330	GAATACATCACGGCTCCTCCTGCCCCT	22	<lmxll>	ll	125	lm	144		
>ref-69331	GCAGATTCCACCTTTTCTCCAATCTCT	4	<lmxll>	ll	117	lm	146		
>ref-69341	TGTATACATACCTATACTCCCTCTCTC	2	<lmxll>	ll	30	lm	237		
>ref-69341	TGTATACATACCTATACTCCCTCTCTC	12	<lmxll>	ll	31	lm	237		
>ref-69349	GCACCGCTTACTCTCTCTCCTACCTGA	5	<hkxhk>	hh	0	hk	273	kk	0
>ref-69349	GCACCGCTTACTCTCTCTCCTACCTGA	14	<hkxhk>	hh	0	hk	168	kk	95
>ref-69398	TATCGTTTCACCCATTCTCCCTTCCCC	15	<lmxll>	ll	124	lm	135		
>ref-69402	CGTCCACCCACTCTACCTCCTACTTCC	15	<hkxhk>	hh	201	hk	24	kk	0
>ref-69414	TCTCCAAGCACTGTACCTCCTTCTCGT	3	<lmxll>	ll	122	lm	141		

>ref-69426	TCCTATTCCACTATTCCTCCTCTTTAT	9	<lmxll>	ll	118	lm	126		
>ref-69443	GAAGATACTACGGACTCTCCCGTTTGT	12	<lmxll>	ll	133	lm	126		
>ref-69443	GAAGATACTACGGACTCTCCCGTTTGT	15	<lmxll>	ll	133	lm	126		
>ref-69464	TCACATATCACTCTTTCTCCCGAAGTA	3	<nnxnp>	nn	129	np	133		
>ref-69476	CCTACACCTACACCACCTCCCTCCCTC	4	<lmxll>	ll	104	lm	150		
>ref-69480	TTCGAAATGACTTTCTCTCCTGACATA	9	<nnxnp>	nn	122	np	131		
>ref-69483	TGCCTTCATACTTGCACTCCTTTCTAC	3	<nnxnp>	nn	126	np	118		
>ref-69490	TCCTGGAAGACACTAGCTCCTGACCGA	2	<lmxll>	ll	123	lm	140		
>ref-69492	ATGCCCATCACTCTTCCTCCACCCCCC	23	<lmxll>	ll	102	lm	157		
>ref-69508	ACCATCAACACCACCTCTCCTTCACCC	21	<lmxll>	ll	126	lm	142		
>ref-69510	CCTACCTTTACCTCTACTCCTCAGACA	2	<lmxll>	ll	247	lm	17		
>ref-69510	CCTACCTTTACCTCTACTCCTCAGACA	14	<hkxhk>	hh	8	hk	262	kk	0
>ref-69512	TGCAACAGCACAGAATCTCCCCTCTTA	16	<hkxhk>	hh	58	hk	137	kk	57
>ref-69513	GCCTGAGGGACGTTCGCTCCGTTAGTA	9	<nnxnp>	nn	138	np	135		
>ref-69516	CCCCCGCCCACTCTCTCTCCCACACAC	9	<nnxnp>	nn	127	np	137		
>ref-69516	CCCCCGCCCACTCTCTCTCCCACACAC	16	<nnxnp>	nn	125	np	137		
>ref-69536	CCTTCCCCCACCTCCTCTCCCCTCTTT	5	<lmxll>	ll	11	lm	246		
>ref-69536	CCTTCCCCCACCTCCTCTCCCCTCTTT	6	<lmxll>	ll	12	lm	245		
>ref-69550	AGGTCACCAACCTCCACTCCAATACCT	6	<lmxll>	ll	53	lm	180		
>ref-69550	AGGTCACCAACCTCCACTCCAATACCT	27	<lmxll>	ll	52	lm	180		
>ref-69551	CGTGCCTCCACCCTCCCTCCTTCCTCT	21	<nnxnp>	nn	101	np	133		
>ref-69574	CACAAGTGCACAGACACTCCAAACAAA	16	<hkxhk>	hh	63	hk	145	kk	57
>ref-69605	CACAATTAAACACCCACTCCCACACCC	26	<nnxnp>	nn	127	np	142		
>ref-69619	CACTATACAACCTCGCCTCCATTACCC	21	<nnxnp>	nn	129	np	140		
>ref-69619	CACTATACAACCTCGCCTCCATTACCC	22	<nnxnp>	nn	129	np	140		
>ref-69627	CCTCTCCCCACCCTATCTCCTTTCTTC	1	<hkxhk>	hh	2	hk	236	kk	7
>ref-69627	CCTCTCCCCACCCTATCTCCTTTCTTC	15	<nnxnp>	nn	42	np	187		
>ref-69627	CCTCTCCCCACCCTATCTCCTTTCTTC	23	<nnxnp>	nn	42	np	187		
>ref-69627	CCTCTCCCCACCCTATCTCCTTTCTTC	24	<lmxll>	ll	4	lm	243		
>ref-69656	AGCTTGTACACCTTCACTCCATCGCTC	5	<lmxll>	ll	129	lm	137		
>ref-69656	AGCTTGTACACCTTCACTCCATCGCTC	21	<lmxll>	ll	129	lm	137		
>ref-69683	TTCACCTCCACTTACCCTCCTCCTTCT	8	<hkxhk>	hh	0	hk	266	kk	4
>ref-69683	TTCACCTCCACTTACCCTCCTCCTTCT	15	<hkxhk>	hh	0	hk	266	kk	4
>ref-69688	GTTTTTAATACTGCTGCTCCACCTCCA	3	<nnxnp>	nn	118	np	126		
>ref-69702	ATCTATTACACATTCACTCCTTTCTTT	25	<nnxnp>	nn	127	np	139		
>ref-69732	AACTCCCCAACTGTTCCTCCTTTCCTT	2	<nnxnp>	nn	133	np	125		
>ref-69745	GCACTAGCAACCACAACTCCATCTTTT	5	<nnxnp>	nn	115	np	114		
>ref-69745	GCACTAGCAACCACAACTCCATCTTTT	7	<nnxnp>	nn	117	np	114		

>ref-69745	GCACTAGCAACCACAACTCCATCTTTT	27	<nnxnp>	nn	248	np	0		
>ref-69760	CTCCCACCAACCCCTCCTCCTCCATAT	3	<hkxhk>	hh	49	hk	207	kk	0
>ref-69760	CTCCCACCAACCCCTCCTCCTCCATAT	24	<hkxhk>	hh	0	hk	205	kk	50
>ref-69764	GAAACTTTTACACATTCTCCAATACCA	3	<hkxhk>	hh	1	hk	269	kk	1
>ref-69764	GAAACTTTTACACATTCTCCAATACCA	15	<hkxhk>	hh	0	hk	253	kk	6
>ref-69765	AAATCCAGGACTCCAGCTCCTGACTCC	8	<lmxll>	ll	55	lm	197		
>ref-69765	AAATCCAGGACTCCAGCTCCTGACTCC	16	<lmxll>	ll	57	lm	195		
>ref-69770	CTGTCTCTCACCAAGTCTCCGAAGTTG	1	<hkxhk>	hh	0	hk	272	kk	1
>ref-69770	CTGTCTCTCACCAAGTCTCCGAAGTTG	6	<hkxhk>	hh	0	hk	250	kk	15
>ref-69773	ACTCTCATCACACTCGCTCCCCCTGAC	4	<hkxhk>	hh	60	hk	144	kk	57
>ref-69773	ACTCTCATCACACTCGCTCCCCCTGAC	7	<hkxhk>	hh	57	hk	144	kk	60
>ref-69789	CTCCGTCTTACCTCCGCTCCCCTCTTT	4	<hkxhk>	hh	54	hk	137	kk	62
>ref-69792	CTCTTACTCACTATCACTCCCTCCCTT	2	<nnxnp>	nn	259	np	0		
>ref-69792	CTCTTACTCACTATCACTCCCTCCCTT	6	<nnxnp>	nn	251	np	5		
>ref-69792	CTCTTACTCACTATCACTCCCTCCCTT	13	<nnxnp>	nn	210	np	27		
>ref-69792	CTCTTACTCACTATCACTCCCTCCCTT	21	<nnxnp>	nn	264	np	0		
>ref-69802	CCCTCCTTCACTCGTCCTCCCCTCCCT	2	<hkxhk>	hh	6	hk	266	kk	0
>ref-69802	CCCTCCTTCACTCGTCCTCCCCTCCCT	26	<hkxhk>	hh	0	hk	266	kk	6
>ref-69803	TAACCAATTACCTCCTCTCCAATGATA	16	<nnxnp>	nn	123	np	106		
>ref-69827	CCAGCTCCCACTTCCTCTCCTTCCTCC	2	<hkxhk>	hh	4	hk	267	kk	0
>ref-69827	CCAGCTCCCACTTCCTCTCCTTCCTCC	4	<hkxhk>	hh	0	hk	267	kk	4
>ref-69847	CTTCGCTCCACCCTATCTCCTCCCCTA	15	<hkxhk>	hh	54	hk	146	kk	58
>ref-69873	GTTAAGTATACGCGAGCTCCCAGAGTA	23	<nnxnp>	nn	132	np	110		
>ref-69874	CATGCACGCACATCCACTCCATATGTA	1	<nnxnp>	nn	117	np	151		
>ref-69874	CATGCACGCACATCCACTCCATATGTA	3	<lmxll>	ll	154	lm	117		
>ref-69877	TTCTCTTTGACTTCATCTCCTTATTCC	2	<lmxll>	ll	130	lm	135		
>ref-69879	TACATCTCCACTCCCCCTCCTCCTCCC	2	<lmxll>	ll	0	lm	254		
>ref-69879	TACATCTCCACTCCCCCTCCTCCTCCC	4	<lmxll>	ll	171	lm	59		
>ref-69879	TACATCTCCACTCCCCCTCCTCCTCCC	13	<nnxnp>	nn	258	np	0		
>ref-69879	TACATCTCCACTCCCCCTCCTCCTCCC	25	<lmxll>	ll	261	lm	0		
>ref-69879	TACATCTCCACTCCCCCTCCTCCTCCC	27	<hkxhk>	hh	106	hk	145	kk	0
>ref-69901	CGGCGCACCACACTTCCTCCACACTCT	5	<lmxll>	ll	139	lm	118		
>ref-69901	CGGCGCACCACACTTCCTCCACACTCT	27	<lmxll>	ll	139	lm	118		
>ref-69911	TCTTTATCCACACTTCCTCCTTTTCTC	9	<lmxll>	ll	150	lm	71		
>ref-69922	GTCTTGCCTACTTGTCCTCCTTTCTCT	3	<lmxll>	ll	137	lm	124		
>ref-69928	TTAATTTGCACATTTTCTCCGACAAAT	3	<hkxhk>	hh	92	hk	160	kk	0
>ref-69928	TTAATTTGCACATTTTCTCCGACAAAT	4	<hkxhk>	hh	2	hk	267	kk	0
>ref-69928	TTAATTTGCACATTTTCTCCGACAAAT	5	<lmxll>	ll	120	lm	136		

>ref-69928	TTAATTTGCACATTTTCTCCGACAAAT	21	<hkxhk>	hh	0	hk	239	kk	22
>ref-69928	TTAATTTGCACATTTTCTCCGACAAAT	26	<hkxhk>	hh	91	hk	160	kk	0
>ref-69971	ACTTTTTCCACTCCATCTCCTCATCTT	7	<hkxhk>	hh	7	hk	245	kk	3
>ref-69971	ACTTTTTCCACTCCATCTCCTCATCTT	15	<hkxhk>	hh	3	hk	245	kk	7
>ref-69996	AGCCCCTGTACAGATTCTCCAGCATCC	8	<hkxhk>	hh	1	hk	248	kk	10
>ref-69996	AGCCCCTGTACAGATTCTCCAGCATCC	22	<hkxhk>	hh	1	hk	248	kk	9
>ref-69997	CAGTCTTTCACCAACTCTCCCTTCCCT	13	<nnxnp>	nn	111	np	154		
>ref-69998	GCCCCACAGACTGCCACTCCCCTTATA	9	<nnxnp>	nn	155	np	108		
>ref-70015	TCGCTCTCCACGTCGTCTCCAGACACG	21	<hkxhk>	hh	0	hk	271	kk	1
>ref-70015	TCGCTCTCCACGTCGTCTCCAGACACG	24	<nnxnp>	nn	140	np	93		
>ref-70015	TCGCTCTCCACGTCGTCTCCAGACACG	26	<hkxhk>	hh	0	hk	271	kk	1
>ref-70015	TCGCTCTCCACGTCGTCTCCAGACACG	27	<hkxhk>	hh	66	hk	178	kk	0
>ref-70083	TCTCCTAACACTACTACTCCTTCTTCT	7	<nnxnp>	nn	23	np	232		
>ref-70083	TCTCCTAACACTACTACTCCTTCTTCT	8	<nnxnp>	nn	22	np	232		
>ref-70083	TCTCCTAACACTACTACTCCTTCTTCT	26	<nnxnp>	nn	147	np	101		
>ref-70103	ACACACCCCACTCCTGCTCCTCCTTCA	2	<nnxnp>	nn	145	np	121		
>ref-70103	ACACACCCCACTCCTGCTCCTCCTTCA	9	<hkxhk>	hh	5	hk	263	kk	0
>ref-70130	CATCCTCCTACTTCCCCTCCTTCTATC	8	<lmxll>	ll	135	lm	129		
>ref-70160	CGAGTCAAAACGGTTTCTCCTCTCCCA	9	<hkxhk>	hh	48	hk	130	kk	46
>ref-70160	CGAGTCAAAACGGTTTCTCCTCTCCCA	14	<hkxhk>	hh	48	hk	130	kk	47
>ref-70215	TTCTGCTTCACCGTAACTCCGTCAGGT	3	<nnxnp>	nn	157	np	116		
>ref-70219	AACACATATACAAGTCCTCCGTTCCTT	2	<nnxnp>	nn	117	np	132		
>ref-70219	AACACATATACAAGTCCTCCGTTCCTT	4	<lmxll>	ll	137	lm	122		
>ref-70224	ATACACTTTACACCTTCTCCCGCCTAC	4	<lmxll>	ll	139	lm	126		
>ref-70256	ACACCCATCACCCAGCCTCCAAGAGAT	26	<nnxnp>	nn	273	np	0		
>ref-70262	TCCAACTTAACGATTCCTCCCGTCTCT	24	<lmxll>	ll	137	lm	119		
>ref-70263	TATATCGAAACACAATCTCCTCCCAGT	21	<nnxnp>	nn	122	np	129		
>ref-70269	TTTCTTCCCACACCCCCTCCCCCCTCA	5	<nnxnp>	nn	34	np	197		
>ref-70269	TTTCTTCCCACACCCCCTCCCCCCTCA	25	<lmxll>	ll	127	lm	106		
>ref-70269	TTTCTTCCCACACCCCCTCCCCCCTCA	26	<lmxll>	ll	130	lm	106		
>ref-70289	CATGTTTATACTCACGCTCCCTTGATC	1	<hkxhk>	hh	72	hk	134	kk	59
>ref-70302	ACCCCACCCACCTCTCCTCCTCCCCTC	6	<hkxhk>	hh	1	hk	262	kk	3
>ref-70302	ACCCCACCCACCTCTCCTCCTCCCCTC	8	<hkxhk>	hh	1	hk	263	kk	3
>ref-70331	TCCCCTCACACCCCTTCTCCCCTCCTT	12	<nnxnp>	nn	6	np	249		
>ref-70373	CTATCTTTCACTTCATCTCCCCTCTTC	6	<hkxhk>	hh	17	hk	241	kk	1
>ref-70373	CTATCTTTCACTTCATCTCCCCTCTTC	12	<hkxhk>	hh	17	hk	243	kk	1
>ref-70404	CACTCACTCACTAACTCTCCCGTTCGT	9	<hkxhk>	hh	0	hk	213	kk	57
>ref-70414	ACATCCATAACTAAGACTCCATAACTA	3	<nnxnp>	nn	124	np	128		

>ref-70419	GAAGGCGTAACTCAATCTCCGGCGAAG	21	<nnxnp>	nn	111	np	139		
>ref-70420	TGTTGAAAAACCTCTCCTCCATTGTCC	25	<nnxnp>	nn	122	np	104		
>ref-70421	CAGCTTGTAACACTCACTCCCGAGGCT	8	<lmxll>	ll	137	lm	127		
>ref-70421	CAGCTTGTAACACTCACTCCCGAGGCT	12	<lmxll>	ll	124	lm	142		
>ref-70426	CACACAGTCACATCATCTCCATCATAA	8	<nnxnp>	nn	134	np	130		
>ref-70431	GGGCAGAAGACAATGGCTCCGCGGCTT	14	<lmxll>	ll	131	lm	137		
>ref-70435	AATACTCCAACAAATACTCCAACGCGT	21	<lmxll>	ll	114	lm	138		
>ref-70435	AATACTCCAACAAATACTCCAACGCGT	27	<nnxnp>	nn	259	np	0		
>ref-70443	ATGACTGGCACCACCCCTCCTCTACTA	4	<hkxhk>	hh	0	hk	273	kk	0
>ref-70443	ATGACTGGCACCACCCCTCCTCTACTA	7	<hkxhk>	hh	0	hk	258	kk	9
>ref-70443	ATGACTGGCACCACCCCTCCTCTACTA	12	<hkxhk>	hh	0	hk	259	kk	9
>ref-70443	ATGACTGGCACCACCCCTCCTCTACTA	16	<lmxll>	ll	9	lm	262		
>ref-70454	TTCGTTATCACGTTATCTCCGGCGGTC	3	<lmxll>	ll	129	lm	138		
>ref-70455	AGTGTATCTACTTTCCCTCCTTCCCCT	9	<hkxhk>	hh	31	hk	181	kk	44
>ref-70455	AGTGTATCTACTTTCCCTCCTTCCCCT	13	<lmxll>	ll	208	lm	57		
>ref-70463	GTTGCCATGACAACCTCTCCGCCGCCG	5	<lmxll>	ll	134	lm	138		
>ref-70480	CCCTCCTATACCCCGTCTCCTCCTCCC	8	<nnxnp>	nn	260	np	0		
>ref-70480	CCCTCCTATACCCCGTCTCCTCCTCCC	15	<nnxnp>	nn	252	np	0		
>ref-70486	CATTTCGGCACATATACTCCGTCTCCT	21	<nnxnp>	nn	150	np	105		
>ref-70502	ACTTACCTTACCTTTACTCCCCTCTCC	3	<nnxnp>	nn	242	np	0		
>ref-70508	CTACCCCCTACCCTCCCTCCATGTCTC	14	<nnxnp>	nn	107	np	148		
>ref-70510	ACGTTTAACACCATGTCTCCTCCCGTC	13	<nnxnp>	nn	135	np	130		
>ref-70533	CTGCCTCTGACTCCCTCTCCGCGGGGA	3	<nnxnp>	nn	134	np	129		
>ref-70553	TCCTTTCTCACTCACACTCCCTCGTCT	9	<hkxhk>	hh	0	hk	137	kk	128
>ref-70553	TCCTTTCTCACTCACACTCCCTCGTCT	13	<hkxhk>	hh	0	hk	137	kk	128
>ref-70553	TCCTTTCTCACTCACACTCCCTCGTCT	16	<nnxnp>	nn	273	np	0		
>ref-70553	TCCTTTCTCACTCACACTCCCTCGTCT	24	<nnxnp>	nn	272	np	0		
>ref-70562	AGGTACCAAACTCTTTCTCCATCTCCC	23	<nnxnp>	nn	112	np	108		
>ref-70585	TTGTCTCCCACTCTTCCTCCTCGTAAG	5	<nnxnp>	nn	137	np	103		
>ref-70585	TTGTCTCCCACTCTTCCTCCTCGTAAG	13	<hkxhk>	hh	101	hk	139	kk	2
>ref-70585	TTGTCTCCCACTCTTCCTCCTCGTAAG	27	<nnxnp>	nn	160	np	73		
>ref-70588	TCCTACCCAACATTCCCTCCCTCCCTA	4	<hkxhk>	hh	3	hk	263	kk	0
>ref-70607	TTGCCAAGGACATTCTCTCCCTCTCTC	15	<nnxnp>	nn	133	np	135		
>ref-70612	CAATAAATCACGTGACCTCCATCCACG	16	<lmxll>	ll	128	lm	133		
>ref-70616	CCCATGCTTACCCATTCTCCTTACCCA	2	<nnxnp>	nn	132	np	122		
>ref-70625	ATCTTTGTCACTTAGTCTCCATCTCTC	7	<hkxhk>	hh	21	hk	242	kk	0
>ref-70625	ATCTTTGTCACTTAGTCTCCATCTCTC	14	<lmxll>	ll	149	lm	118		
>ref-70625	ATCTTTGTCACTTAGTCTCCATCTCTC	15	<nnxnp>	nn	212	np	50		

>ref-70652	CCAGCATGAACCGAAGCTCCCGGTGCA	13	<lmxll>	ll	132	lm	135		
>ref-70676	CTCTCCCACACGCCCTCTCCTACCTCT	9	<lmxll>	ll	141	lm	127		
>ref-70680	GTGCTTTGAACGTTTCCTCCCTCATAC	12	<nnxnp>	nn	126	np	137		
>ref-70681	CAGACACATACGCCCTCTCCCATTGTC	7	<lmxll>	ll	146	lm	123		
>ref-70681	CAGACACATACGCCCTCTCCCATTGTC	14	<lmxll>	ll	146	lm	123		
>ref-70735	AGTGTTTTCACGGTCCCTCCATTGGTT	1	<hkxhk>	hh	59	hk	143	kk	57
>ref-70735	AGTGTTTTCACGGTCCCTCCATTGGTT	9	<lmxll>	ll	116	lm	143		
>ref-70771	TTGTTAGCCACCTCACCTCCTGTAGCT	5	<lmxll>	ll	143	lm	128		
>ref-70788	TACTCCTTTACTGTTTCTCCTTTGCTT	22	<lmxll>	ll	113	lm	150		
>ref-70788	TACTCCTTTACTGTTTCTCCTTTGCTT	24	<lmxll>	ll	114	lm	150		
>ref-70811	CTTTCTCTCACCTTCTCTCCATTATCT	14	<lmxll>	ll	126	lm	131		
>ref-70822	CCCTTCTGCACTCATCCTCCCCTCTTT	22	<hkxhk>	hh	49	hk	146	kk	56
>ref-70836	CCCACCACCACCAGTTCTCCTTTCTCC	3	<nnxnp>	nn	133	np	132		
>ref-70931	TTGCCTCTCACTCCGCCTCCTCCCCTC	15	<hkxhk>	hh	51	hk	140	kk	59
>ref-70939	CTCTCATTAACTGTCACTCCCTAACAC	22	<lmxll>	ll	0	lm	273		
>ref-70939	CTCTCATTAACTGTCACTCCCTAACAC	23	<lmxll>	ll	0	lm	273		
>ref-70957	AACCCTCTCACCCTCCCTCCTGCCCTA	22	<nnxnp>	nn	266	np	0		
>ref-70957	AACCCTCTCACCCTCCCTCCTGCCCTA	27	<nnxnp>	nn	265	np	0		
>ref-70969	TCAGTAATCACGGCACCTCCCACTGGT	3	<lmxll>	ll	0	lm	272		
>ref-70980	GAGACAAAAACGGGCTCTCCTCCCTCT	7	<lmxll>	ll	117	lm	132		
>ref-71022	CACAGAATTACTCGTTCTCCAACAATA	13	<lmxll>	ll	145	lm	109		
>ref-71022	CACAGAATTACTCGTTCTCCAACAATA	22	<hkxhk>	hh	39	hk	136	kk	72
>ref-71032	TAATCTTCCACCTCCTCTCCTTCCTCT	26	<nnxnp>	nn	128	np	139		
>ref-71033	TGGGCTATCACTCATTCTCCAATTTGT	2	<lmxll>	ll	114	lm	118		
>ref-71060	ATCCTCTCAACTACGCCTCCGTCCCCT	14	<hkxhk>	hh	0	hk	270	kk	3
>ref-71060	ATCCTCTCAACTACGCCTCCGTCCCCT	21	<hkxhk>	hh	0	hk	270	kk	3
>ref-71062	GTTATACATACTCATGCTCCTCGCCCA	12	<lmxll>	ll	116	lm	150		
>ref-71062	GTTATACATACTCATGCTCCTCGCCCA	27	<hkxhk>	hh	59	hk	140	kk	58
>ref-71086	CTCTCATTCACCTTCTCTCCCTATCTT	23	<nnxnp>	nn	9	np	264		
>ref-71086	CTCTCATTCACCTTCTCTCCCTATCTT	26	<nnxnp>	nn	9	np	264		
>ref-71088	CCTATCGACACTTCGCCTCCTCTTCTA	14	<nnxnp>	nn	155	np	102		
>ref-71088	CCTATCGACACTTCGCCTCCTCTTCTA	27	<lmxll>	ll	131	lm	136		
>ref-71107	AACGTGTTCACTCTCTCTCCCTTTGTG	6	<lmxll>	ll	131	lm	136		
>ref-71108	CTCACTCCCACCCTTTCTCCTCTCCCC	4	<nnxnp>	nn	24	np	225		
>ref-71139	CACCAGCCCACCTCCCCTCCCCCTCAA	6	<nnxnp>	nn	212	np	53		
>ref-71139	CACCAGCCCACCTCCCCTCCCCCTCAA	15	<hkxhk>	hh	10	hk	261	kk	0
>ref-71161	TTTAATGCCACACCTCCTCCTATTGTC	15	<lmxll>	ll	251	lm	0		
>ref-71181	TCATTATTCACTAACGCTCCTTTGTTA	16	<nnxnp>	nn	141	np	123		

>ref-71192	ACCTCCAATACCATCTCTCCTTGAGCT	4	<lmxll>	ll	127	lm	125		
>ref-71195	CCATACACAACACTTACTCCTTTAATG	15	<nnxnp>	nn	113	np	116		
>ref-71197	CACCCCTCTACCCCCCCTCCCACATAC	23	<nnxnp>	nn	160	np	89		
>ref-71208	CTTCGTCTAACACATACTCCTTCCTTT	4	<lmxll>	ll	115	lm	135		
>ref-71209	TGCACACAGACTCTCTCTCCCTCCTCT	4	<nnxnp>	nn	115	np	146		
>ref-71214	TTTATCTAAACTTCCGCTCCTCGTCAT	1	<lmxll>	ll	265	lm	0		
>ref-71249	TCCTGCTTCACATTCTCTCCTCTTCTC	7	<lmxll>	ll	116	lm	134		
>ref-71260	TACCCTCTCACTCCTCCTCCTTCGACC	7	<nnxnp>	nn	122	np	142		
>ref-71275	TACCTCCCTACACCTACTCCCTCCCTC	2	<nnxnp>	nn	161	np	78		
>ref-71275	TACCTCCCTACACCTACTCCCTCCCTC	15	<nnxnp>	nn	113	np	134		
>ref-71275	TACCTCCCTACACCTACTCCCTCCCTC	16	<nnxnp>	nn	163	np	75		
>ref-71294	CATATGTATACGTACGCTCCTATATAC	16	<nnxnp>	nn	100	np	122		
>ref-71299	TCCTCAGACACAGGCTCTCCATTTAGT	1	<lmxll>	ll	118	lm	134		
>ref-71308	GTGGTATGTACATACACTCCTCGTTCC	5	<lmxll>	ll	102	lm	163		
>ref-71308	GTGGTATGTACATACACTCCTCGTTCC	14	<hkxhk>	hh	0	hk	273	kk	0
>ref-71308	GTGGTATGTACATACACTCCTCGTTCC	15	<lmxll>	ll	112	lm	153		
>ref-71308	GTGGTATGTACATACACTCCTCGTTCC	16	<hkxhk>	hh	0	hk	273	kk	0
>ref-71308	GTGGTATGTACATACACTCCTCGTTCC	25	<lmxll>	ll	101	lm	163		
>ref-71313	TTCTTTCTTACACGTTCTCCGCTTCCT	21	<lmxll>	ll	152	lm	114		
>ref-71314	CATCCCCCCACTCAACCTCCCCCTTCC	14	<nnxnp>	nn	267	np	0		
>ref-71314	CATCCCCCCACTCAACCTCCCCCTTCC	15	<nnxnp>	nn	260	np	1		
>ref-71379	TGTAAATCTACATCATCTCCCTTTGTT	8	<nnxnp>	nn	130	np	111		
>ref-71380	CACACGCGCACCTACCCTCCCCCAACA	21	<lmxll>	ll	132	lm	128		
>ref-71394	CCCTCCTCCACCAGTGCTCCACTCTTG	9	<nnxnp>	nn	113	np	155		
>ref-71397	TAATCCCTCACACTCCCTCCCTCTCTA	23	<nnxnp>	nn	134	np	121		
>ref-71422	CAAGTCTGCACCGAGTCTCCGTGCAAG	2	<hkxhk>	hh	76	hk	145	kk	45
>ref-71455	AATACTTACACCGGAACTCCCCAGCAA	8	<lmxll>	ll	108	lm	126		
>ref-71455	AATACTTACACCGGAACTCCCCAGCAA	9	<lmxll>	ll	108	lm	126		
>ref-71470	CAGAGCGGGACTCTTTCTCCGTCTTCG	6	<lmxll>	ll	124	lm	127		
>ref-71478	TCCCAAATCACTTTCTCTCCCTCCATC	25	<lmxll>	ll	113	lm	139		
>ref-71523	GCGGCTCTCACGGTCTCTCCTTATTCA	16	<lmxll>	ll	110	lm	133		
>ref-71526	TAAAGGAGTACTGAGGCTCCTTGGCAA	21	<nnxnp>	nn	127	np	135		
>ref-71526	TAAAGGAGTACTGAGGCTCCTTGGCAA	24	<nnxnp>	nn	124	np	136		
>ref-71529	TTATTAGTCACTCACCCTCCCCCCTCT	24	<lmxll>	ll	111	lm	134		
>ref-71543	TCTCTTCCCACCATCCCTCCCTCTCTA	1	<hkxhk>	hh	0	hk	259	kk	5
>ref-71543	TCTCTTCCCACCATCCCTCCCTCTCTA	4	<hkxhk>	hh	5	hk	259	kk	0
>ref-71557	TTTACAATCACAATATCTCCTACTTTC	21	<hkxhk>	hh	0	hk	237	kk	20
>ref-71557	TTTACAATCACAATATCTCCTACTTTC	26	<lmxll>	ll	48	lm	193		

>ref-71557	TTTACAATCACAATATCTCCTACTTTC	27	<hkxhk>	hh	11	hk	247	kk	1
>ref-71560	CCCCCTCTCACTTACCCTCCCCACTTC	15	<nnxnp>	nn	225	np	0		
>ref-71562	CCCGCCATCACTTACCCTCCAATACTT	2	<hkxhk>	hh	0	hk	256	kk	16
>ref-71562	CCCGCCATCACTTACCCTCCAATACTT	9	<lmxll>	ll	7	lm	262		
>ref-71562	CCCGCCATCACTTACCCTCCAATACTT	14	<lmxll>	ll	7	lm	262		
>ref-71562	CCCGCCATCACTTACCCTCCAATACTT	21	<nnxnp>	nn	210	np	50		
>ref-71562	CCCGCCATCACTTACCCTCCAATACTT	23	<hkxhk>	hh	0	hk	86	kk	177
>ref-71581	CGGACACTGACTCTTCCTCCTCCCCTT	24	<hkxhk>	hh	0	hk	272	kk	0
>ref-71581	CGGACACTGACTCTTCCTCCTCCCCTT	26	<hkxhk>	hh	0	hk	272	kk	0
>ref-71596	ACCCTCGCGACGCTGCCTCCGCCGCCT	7	<lmxll>	ll	137	lm	134		
>ref-71619	GGGGATTTAACAGACGCTCCCAGCTCT	4	<lmxll>	ll	120	lm	139		
>ref-71619	GGGGATTTAACAGACGCTCCCAGCTCT	12	<lmxll>	ll	120	lm	139		
>ref-71633	TTCATCAACACCCCATCTCCCCTTATT	9	<nnxnp>	nn	121	np	127		
>ref-71646	ATATGGCCTACGGCAGCTCCTAGTATC	2	<nnxnp>	nn	128	np	134		
>ref-71654	AATCCATTCACCTTTTCTCCTCCTCCT	5	<hkxhk>	hh	37	hk	141	kk	67
>ref-71654	AATCCATTCACCTTTTCTCCTCCTCCT	12	<lmxll>	ll	147	lm	104		
>ref-71714	AGCCGACACACCTCAGCTCCAGTCCCT	12	<nnxnp>	nn	235	np	28		
>ref-71742	TACACATGCACGTACACTCCCAGATAC	9	<hkxhk>	hh	55	hk	140	kk	54
>ref-71749	GTTTATGGAACGTTTCCTCCCTCTACA	24	<lmxll>	ll	119	lm	148		
>ref-71764	CATTGCAATACTGGCGCTCCTGGTATA	5	<nnxnp>	nn	257	np	0		
>ref-71764	CATTGCAATACTGGCGCTCCTGGTATA	6	<nnxnp>	nn	132	np	109		
>ref-71802	GAATCAAAAACTCATCCTCCTTCGGAA	23	<nnxnp>	nn	233	np	0		
>ref-71838	CCCCTGGATACAGTGGCTCCTCAGGAA	21	<hkxhk>	hh	0	hk	273	kk	0
>ref-71846	TTGCGTAACACTTGACCTCCTTTGGTC	24	<hkxhk>	hh	64	hk	146	kk	54
>ref-71864	CGCACACTCACCGCTTCTCCGCCGCAC	3	<nnxnp>	nn	256	np	0		
>ref-71871	CGGGCGCCCACGACAACTCCGTGAAGG	25	<nnxnp>	nn	259	np	0		
>ref-71871	CGGGCGCCCACGACAACTCCGTGAAGG	26	<nnxnp>	nn	258	np	0		
>ref-71928	TTATATATAACTCCCCCTCCCCCCTCT	2	<lmxll>	ll	35	lm	192		
>ref-71928	TTATATATAACTCCCCCTCCCCCCTCT	9	<lmxll>	ll	35	lm	193		
>ref-71954	GGCTCCCTGACTCCCCCTCCCTTCCTT	21	<nnxnp>	nn	118	np	131		
>ref-71993	TGAGACTAGACGCAACCTCCACGGATC	9	<lmxll>	ll	127	lm	133		
>ref-72052	CTCACTCTCACTCTTGCTCCTCCTTCA	25	<lmxll>	ll	107	lm	150		
>ref-72054	TATACGTATACACCCCCTCCACACACA	2	<lmxll>	ll	3	lm	259		
>ref-72054	TATACGTATACACCCCCTCCACACACA	6	<lmxll>	ll	4	lm	259		
>ref-72054	TATACGTATACACCCCCTCCACACACA	8	<lmxll>	ll	171	lm	74		
>ref-72064	TACGTATATACGTCTTCTCCGAGTCTA	6	<hkxhk>	hh	43	hk	145	kk	76
>ref-72083	TGGCCCGAAACAACCACTCCACTACAA	16	<nnxnp>	nn	135	np	135		
>ref-72104	TCCTCCTCCACCTCCGCTCCTACCCCC	1	<hkxhk>	hh	0	hk	273	kk	0

>ref-72104	TCCTCCTCCACCTCCGCTCCTACCCCC	15	<lmxll>	ll	73	lm	183		
>ref-72104	TCCTCCTCCACCTCCGCTCCTACCCCC	23	<hkxhk>	hh	0	hk	266	kk	6
>ref-72104	TCCTCCTCCACCTCCGCTCCTACCCCC	25	<lmxll>	ll	68	lm	187		
>ref-72105	TCTTTCTCTACTTTCACTCCTCCCCCT	22	<nnxnp>	nn	270	np	1		
>ref-72105	TCTTTCTCTACTTTCACTCCTCCCCCT	24	<nnxnp>	nn	266	np	2		
>ref-72170	ATCTTACAAACAGTGCCTCCTGGACAT	27	<lmxll>	ll	119	lm	108		
>ref-72178	ACTTTAGTTACGTTTTCTCCTCGTCAT	1	<lmxll>	ll	114	lm	145		
>ref-72178	ACTTTAGTTACGTTTTCTCCTCGTCAT	5	<lmxll>	ll	118	lm	145		
>ref-72183	ATCACCATCACCACCCCTCCTCACAAT	21	<nnxnp>	nn	142	np	124		
>ref-72188	GTGTCTGTCACTCCCTCTCCTTCCCCC	7	<nnxnp>	nn	244	np	4		
>ref-72191	AAGAGTTCCACCTGCGCTCCTTTCTGT	16	<nnxnp>	nn	138	np	130		
>ref-72191	AAGAGTTCCACCTGCGCTCCTTTCTGT	23	<nnxnp>	nn	137	np	131		
>ref-72197	CCCTACCCTACATACCCTCCACCTCTT	5	<lmxll>	ll	4	lm	264		
>ref-72197	CCCTACCCTACATACCCTCCACCTCTT	23	<hkxhk>	hh	33	hk	221	kk	1
>ref-72197	CCCTACCCTACATACCCTCCACCTCTT	27	<hkxhk>	hh	2	hk	223	kk	28
>ref-72221	TATGTATACACTTCCTCTCCTCGTCCT	23	<nnxnp>	nn	131	np	131		
>ref-72224	ATCTAAACGACACTCCCTCCACCCGGT	6	<nnxnp>	nn	113	np	133		
>ref-72224	ATCTAAACGACACTCCCTCCACCCGGT	7	<nnxnp>	nn	114	np	133		
>ref-72238	AAATACGCCACAATATCTCCTAACCAC	27	<lmxll>	ll	119	lm	121		
>ref-72261	CTCTCACTCACCTCCTCTCCGCCTCCC	2	<nnxnp>	nn	3	np	267		
>ref-72261	CTCTCACTCACCTCCTCTCCGCCTCCC	21	<nnxnp>	nn	3	np	267		
>ref-72262	CTCCCTTCCACCCTCACTCCACCCAAC	1	<nnxnp>	nn	129	np	133		
>ref-72262	CTCCCTTCCACCCTCACTCCACCCAAC	6	<lmxll>	ll	119	lm	108		
>ref-72262	CTCCCTTCCACCCTCACTCCACCCAAC	7	<lmxll>	ll	117	lm	109		
>ref-72262	CTCCCTTCCACCCTCACTCCACCCAAC	12	<hkxhk>	hh	10	hk	249	kk	0
>ref-72282	TCCCCCTCTACCCTTCCTCCTTACCCC	3	<hkxhk>	hh	0	hk	263	kk	1
>ref-72282	TCCCCCTCTACCCTTCCTCCTTACCCC	4	<hkxhk>	hh	1	hk	263	kk	0
>ref-72282	TCCCCCTCTACCCTTCCTCCTTACCCC	15	<lmxll>	ll	175	lm	69		
>ref-72282	TCCCCCTCTACCCTTCCTCCTTACCCC	23	<lmxll>	ll	174	lm	71		
>ref-72316	TCTCTCTTTACCTTTCCTCCTTACCTC	4	<hkxhk>	hh	83	hk	174	kk	0
>ref-72316	TCTCTCTTTACCTTTCCTCCTTACCTC	16	<hkxhk>	hh	0	hk	273	kk	0
>ref-72316	TCTCTCTTTACCTTTCCTCCTTACCTC	23	<nnxnp>	nn	13	np	249		
>ref-72316	TCTCTCTTTACCTTTCCTCCTTACCTC	24	<hkxhk>	hh	0	hk	174	kk	82
>ref-72353	CTTCCTCTTACTCTCACTCCGTTATTA	27	<hkxhk>	hh	5	hk	250	kk	2
>ref-72373	GCCTCACCTACTATTGCTCCCAGGTTC	5	<nnxnp>	nn	108	np	121		
>ref-72373	GCCTCACCTACTATTGCTCCCAGGTTC	9	<nnxnp>	nn	107	np	121		
>ref-72380	CCCACTTACACTCTATCTCCTTCTCCC	14	<hkxhk>	hh	0	hk	273	kk	0
>ref-72380	CCCACTTACACTCTATCTCCTTCTCCC	22	<hkxhk>	hh	0	hk	273	kk	0

>ref-72382	CCAATCTTTACCACTTCTCCTCTGCCT	12	<nnxnp>	nn	121	np	148		
>ref-72388	GAAAAGACCACATCACCTCCTCTCACA	24	<lmxll>	ll	126	lm	112		
>ref-72403	TACTTAGACACACACTCTCCCTCTTCC	4	<nnxnp>	nn	130	np	133		
>ref-72418	CTCGACTTTACCTTCCCTCCTGCATTA	4	<hkxhk>	hh	46	hk	152	kk	52
>ref-72420	TATACTCAAACCGTCCCTCCTGGCAGA	12	<lmxll>	ll	262	lm	0		
>ref-72431	AGGCTACCTACTCCCTCTCCTCTCGCT	4	<lmxll>	ll	264	lm	0		
>ref-72433	TCCCTTTCTACCCAGACTCCCTCCTTC	15	<hkxhk>	hh	0	hk	263	kk	0
>ref-72448	ACAGTTTACACACACGCTCCCTTGGGA	4	<lmxll>	ll	0	lm	268		
>ref-72448	ACAGTTTACACACACGCTCCCTTGGGA	25	<lmxll>	ll	0	lm	268		
>ref-72458	CTAATTCCTACATTTCCTCCTTTACCT	27	<lmxll>	ll	144	lm	124		
>ref-72467	GCACACACCACAGGTACTCCTCACAGA	15	<lmxll>	ll	124	lm	132		
>ref-72470	ATGCCATCGACTCCCCCTCCTTGATGT	14	<nnxnp>	nn	132	np	135		
>ref-72472	CCCCCGGGCACACTCCCTCCCTCAATC	7	<lmxll>	ll	24	lm	233		
>ref-72472	CCCCCGGGCACACTCCCTCCCTCAATC	9	<hkxhk>	hh	22	hk	238	kk	0
>ref-72472	CCCCCGGGCACACTCCCTCCCTCAATC	16	<lmxll>	ll	23	lm	233		
>ref-72476	TCTCTCTCTACTCTCACTCCTGTTCCC	26	<lmxll>	ll	116	lm	113		
>ref-72476	TCTCTCTCTACTCTCACTCCTGTTCCC	27	<lmxll>	ll	116	lm	113		
>ref-72486	ACATATGGGACACGCTCTCCTGTTCTG	24	<lmxll>	ll	153	lm	113		
>ref-72530	TTTTTACGTACATCTCCTCCTCGCACT	26	<lmxll>	ll	113	lm	125		
>ref-72556	CAATGTTATACAAATCCTCCTAAACTA	25	<lmxll>	ll	109	lm	139		
>ref-72578	TTCTTTTCTACTGCTACTCCTATGGAT	27	<lmxll>	ll	142	lm	92		
>ref-72593	CTGACCTGCACCTCATCTCCGCGCTTG	14	<nnxnp>	nn	136	np	134		
>ref-72603	GTATAACATACAGTACCTCCCTTGTGT	22	<lmxll>	ll	106	lm	120		
>ref-72613	TTCCTCCCTACCCCCTCTCCCCATCTC	7	<hkxhk>	hh	2	hk	270	kk	0
>ref-72613	TTCCTCCCTACCCCCTCTCCCCATCTC	13	<hkxhk>	hh	1	hk	270	kk	0
>ref-72614	ACTTACTCCACCTCTTCTCCTTCCACC	15	<lmxll>	ll	8	lm	215		
>ref-72650	TGATGTTAAACCATTCCTCCTCGTTGG	6	<nnxnp>	nn	241	np	0		
>ref-72662	CATGGGCCAACTCCCCCTCCTCCCCAT	24	<nnxnp>	nn	130	np	136		
>ref-72680	AACCGCCACACTCATCCTCCAACAACG	5	<lmxll>	ll	130	lm	132		
>ref-72688	CTGTCAAGAACCAGCCCTCCCAGGTAA	16	<nnxnp>	nn	146	np	109		
>ref-72711	CCAAGTCAAACGGAGCCTCCCCGTGCG	25	<nnxnp>	nn	156	np	105		
>ref-72719	ACCCCGGCTACCCCTTCTCCTCTAACA	23	<hkxhk>	hh	0	hk	273	kk	0
>ref-72719	ACCCCGGCTACCCCTTCTCCTCTAACA	24	<hkxhk>	hh	1	hk	270	kk	0
>ref-72719	ACCCCGGCTACCCCTTCTCCTCTAACA	25	<hkxhk>	hh	0	hk	273	kk	0
>ref-72719	ACCCCGGCTACCCCTTCTCCTCTAACA	26	<hkxhk>	hh	0	hk	233	kk	0
>ref-72724	CCCTTCCCCACCCTGTCTCCTTGTCAG	13	<lmxll>	ll	118	lm	137		
>ref-72727	TACTAACATACTTCTTCTCCATTCCAC	7	<nnxnp>	nn	104	np	126		
>ref-72732	ATACATGCGACTCCACCTCCCTACCCT	21	<lmxll>	ll	116	lm	145		

>ref-72734	GTGGTATGTACATGCACTCCTTCTCAT	14	<hkxhk>	hh	1	hk	272	kk	0
>ref-72734	GTGGTATGTACATGCACTCCTTCTCAT	26	<hkxhk>	hh	0	hk	272	kk	1
>ref-72739	CCCTTTTATACACGCACTCCCTTTCCT	2	<nnxnp>	nn	261	np	0		
>ref-72746	ATTCCCTGTACTGTCGCTCCTACTACT	4	<lmxll>	ll	127	lm	114		
>ref-72758	AGACTCACGACGTCCTCTCCGTCGCCT	15	<hkxhk>	hh	62	hk	145	kk	55
>ref-72795	AGTCCAGAGACTCGGCCTCCACTACAC	14	<hkxhk>	hh	45	hk	149	kk	60
>ref-72801	TTTTCCTCCACATTCTCTCCACTTACT	5	<nnxnp>	nn	115	np	124		
>ref-72801	TTTTCCTCCACATTCTCTCCACTTACT	27	<nnxnp>	nn	257	np	0		
>ref-72804	CAAAGATATACTTCAGCTCCAAACCTT	6	<nnxnp>	nn	119	np	116		
>ref-72816	CATAGTCCAACCGATCCTCCGACCTGA	3	<hkxhk>	hh	0	hk	154	kk	102
>ref-72816	CATAGTCCAACCGATCCTCCGACCTGA	14	<hkxhk>	hh	124	hk	131	kk	0
>ref-72816	CATAGTCCAACCGATCCTCCGACCTGA	15	<hkxhk>	hh	0	hk	154	kk	102
>ref-72816	CATAGTCCAACCGATCCTCCGACCTGA	26	<hkxhk>	hh	0	hk	273	kk	0
>ref-72816	CATAGTCCAACCGATCCTCCGACCTGA	27	<hkxhk>	hh	0	hk	273	kk	0
>ref-72820	CACACCCACACCTCCACTCCCATCCCC	13	<nnxnp>	nn	134	np	136		
>ref-72820	CACACCCACACCTCCACTCCCATCCCC	14	<nnxnp>	nn	132	np	136		
>ref-72828	GCGACAGGAACGCAAACTCCCACACCC	7	<nnxnp>	nn	114	np	122		
>ref-72832	CTTTTATCCACACTCTCTCCTCCCGTC	12	<nnxnp>	nn	124	np	130		
>ref-72852	ACCACCACCACCTTCTCTCCCTCCTCT	5	<lmxll>	ll	132	lm	127		
>ref-72852	ACCACCACCACCTTCTCTCCCTCCTCT	13	<lmxll>	ll	131	lm	126		
>ref-72852	ACCACCACCACCTTCTCTCCCTCCTCT	16	<hkxhk>	hh	0	hk	273	kk	0
>ref-72852	ACCACCACCACCTTCTCTCCCTCCTCT	21	<hkxhk>	hh	231	hk	35	kk	0
>ref-72852	ACCACCACCACCTTCTCTCCCTCCTCT	24	<hkxhk>	hh	231	hk	35	kk	0
>ref-72852	ACCACCACCACCTTCTCTCCCTCCTCT	25	<hkxhk>	hh	0	hk	273	kk	0
>ref-72866	TCCCCACCTACTTCCCCTCCCCACCTA	21	<hkxhk>	hh	0	hk	250	kk	3
>ref-72866	TCCCCACCTACTTCCCCTCCCCACCTA	27	<hkxhk>	hh	15	hk	225	kk	0
>ref-72915	CAGTCAGTCACTCTCTCTCCCACCTCT	3	<hkxhk>	hh	2	hk	259	kk	1
>ref-72920	ATCTTCTTTACAACATCTCCTTTTCTC	12	<hkxhk>	hh	49	hk	146	kk	46
>ref-72920	ATCTTCTTTACAACATCTCCTTTTCTC	13	<hkxhk>	hh	49	hk	146	kk	46
>ref-72958	AACTCACTAACACCTTCTCCTTCCTCT	15	<nnxnp>	nn	124	np	136		
>ref-72958	AACTCACTAACACCTTCTCCTTCCTCT	25	<nnxnp>	nn	121	np	139		
>ref-72981	ATGATATTGACTCCGCCTCCCTCTTCC	7	<nnxnp>	nn	124	np	123		
>ref-72981	ATGATATTGACTCCGCCTCCCTCTTCC	16	<nnxnp>	nn	124	np	122		
>ref-73010	GAGTAAACAACGTTTTCTCCTTACGTC	2	<hkxhk>	hh	94	hk	169	kk	0
>ref-73010	GAGTAAACAACGTTTTCTCCTTACGTC	21	<hkxhk>	hh	0	hk	184	kk	82
>ref-73011	TCTGTCTCTACTCCTCCTCCTCACCCC	27	<nnxnp>	nn	239	np	0		
>ref-73023	TCCCCTCTTACAACCTCTCCCACTCCC	21	<lmxll>	ll	134	lm	124		
>ref-73032	TTCAGCATCACCGCTACTCCCTTTTCA	2	<hkxhk>	hh	0	hk	244	kk	23

>ref-73032	TTCAGCATCACCGCTACTCCCTTTTCA	4	<nnxnp>	nn	242	np	17		
>ref-73032	TTCAGCATCACCGCTACTCCCTTTTCA	5	<hkxhk>	hh	0	hk	117	kk	135
>ref-73032	TTCAGCATCACCGCTACTCCCTTTTCA	14	<lmxll>	ll	116	lm	126		
>ref-73032	TTCAGCATCACCGCTACTCCCTTTTCA	27	<lmxll>	ll	131	lm	119		
>ref-73034	AAATGAGGCACTCCTCCTCCCTCCATC	24	<lmxll>	ll	132	lm	130		
>ref-73048	TCGTCTTACACCTAACCTCCCTCTCCT	14	<lmxll>	ll	123	lm	126		
>ref-73048	TCGTCTTACACCTAACCTCCCTCTCCT	25	<lmxll>	ll	122	lm	126		
>ref-73049	TGTTTTTCCACCTCAACTCCATCTTAC	14	<nnxnp>	nn	111	np	146		
>ref-73061	CTCTCTAGAACATTCTCTCCTCTTTTC	5	<lmxll>	ll	12	lm	250		
>ref-73061	CTCTCTAGAACATTCTCTCCTCTTTTC	9	<lmxll>	ll	12	lm	251		
>ref-73064	CGATCTCCAACTCCATCTCCTCCACCA	12	<nnxnp>	nn	145	np	112		
>ref-73078	CGCTCCTCCACCACTCCTCCACCGCTC	2	<hkxhk>	hh	0	hk	142	kk	89
>ref-73078	CGCTCCTCCACCACTCCTCCACCGCTC	3	<hkxhk>	hh	0	hk	145	kk	83
>ref-73078	CGCTCCTCCACCACTCCTCCACCGCTC	13	<nnxnp>	nn	201	np	32		
>ref-73078	CGCTCCTCCACCACTCCTCCACCGCTC	16	<nnxnp>	nn	203	np	33		
>ref-73084	CTCCTCCTCACGCGCTCTCCATACTCC	3	<nnxnp>	nn	123	np	139		
>ref-73084	CTCCTCCTCACGCGCTCTCCATACTCC	4	<nnxnp>	nn	123	np	139		
>ref-73089	TAAGCGTGTACTGAGGCTCCCATGGAA	12	<nnxnp>	nn	129	np	138		
>ref-73089	TAAGCGTGTACTGAGGCTCCCATGGAA	13	<nnxnp>	nn	129	np	138		
>ref-73095	ACTCGCACGACACAGACTCCTTGTTTC	1	<lmxll>	ll	114	lm	148		
>ref-73100	GACTGGCGCACGAGTTCTCCGCGAGAA	7	<hkxhk>	hh	0	hk	273	kk	0
>ref-73100	GACTGGCGCACGAGTTCTCCGCGAGAA	23	<hkxhk>	hh	0	hk	273	kk	0
>ref-73104	CACTCTTTCACCCTCACTCCCTCATCC	3	<nnxnp>	nn	132	np	97		
>ref-73110	AGCATTGACACGCACACTCCCTTATAA	6	<lmxll>	ll	123	lm	145		
>ref-73110	AGCATTGACACGCACACTCCCTTATAA	23	<lmxll>	ll	123	lm	145		
>ref-73114	CTTCTCTGTACCCCCCCTCCCCCTCTC	8	<lmxll>	ll	52	lm	206		
>ref-73119	AGGCCTCGAACTGGCCCTCCGGGTATG	2	<lmxll>	ll	122	lm	128		
>ref-73143	CTCAGCTTTACTCCCTCTCCGTCCCTC	27	<hkxhk>	hh	48	hk	151	kk	59
>ref-73150	CCAGCAACCACATACGCTCCCTGAAAG	3	<lmxll>	ll	120	lm	139		
>ref-73157	TACCCTTCTACTTCTCCTCCTTGTCCC	2	<nnxnp>	nn	116	np	137		
>ref-73164	TCTCCCCCCACTTCCTCTCCATCCCCC	1	<hkxhk>	hh	0	hk	158	kk	65
>ref-73164	TCTCCCCCCACTTCCTCTCCATCCCCC	4	<nnxnp>	nn	238	np	1		
>ref-73164	TCTCCCCCCACTTCCTCTCCATCCCCC	27	<lmxll>	ll	111	lm	114		
>ref-73207	CTAGCAAGAACCTTTACTCCAACAACA	1	<nnxnp>	nn	113	np	121		
>ref-73228	AACAGCTAAACCGAACCTCCAATATCA	2	<lmxll>	ll	104	lm	136		
>ref-73230	CCAGTACACACATCGCCTCCAGCACAC	1	<nnxnp>	nn	110	np	147		
>ref-73247	TGCGCTCACACCCTTTCTCCCTCTTTG	1	<nnxnp>	nn	109	np	132		
>ref-73259	AATATAGTGACTATACCTCCCTGACAT	23	<nnxnp>	nn	227	np	0		

>ref-73265	TGCTTCTCCACACGTCCTCCCTCCCTA	24	<lmxll>	ll	128	lm	122		
>ref-73267	AACACCCTCACCCCCACTCCTACCCTA	6	<hkxhk>	hh	0	hk	168	kk	91
>ref-73267	AACACCCTCACCCCCACTCCTACCCTA	15	<nnxnp>	nn	15	np	243		
>ref-73270	TCTACTCTTACCCCTACTCCTACTCCT	8	<hkxhk>	hh	3	hk	255	kk	6
>ref-73270	TCTACTCTTACCCCTACTCCTACTCCT	12	<lmxll>	ll	0	lm	236		
>ref-73270	TCTACTCTTACCCCTACTCCTACTCCT	22	<nnxnp>	nn	58	np	193		
>ref-73271	TCTAATGTGACACGTCCTCCGGCCGGC	2	<hkxhk>	hh	0	hk	273	kk	0
>ref-73271	TCTAATGTGACACGTCCTCCGGCCGGC	9	<hkxhk>	hh	0	hk	107	kk	154
>ref-73281	AGCTGATTTACACATCCTCCATGAGGT	1	<hkxhk>	hh	0	hk	273	kk	0
>ref-73281	AGCTGATTTACACATCCTCCATGAGGT	21	<lmxll>	ll	182	lm	83		
>ref-73281	AGCTGATTTACACATCCTCCATGAGGT	23	<hkxhk>	hh	0	hk	228	kk	35
>ref-73290	ACCTTCTCTACCCTTTCTCCTTCACTC	7	<nnxnp>	nn	132	np	137		
>ref-73308	AGGAGTTTGACTGGATCTCCCGGCATC	1	<nnxnp>	nn	119	np	141		
>ref-73314	ACTGCGCACACCTTGCCTCCCATGCCC	8	<lmxll>	ll	121	lm	146		
>ref-73315	ACCCCCCAAACTCCTTCTCCCCCTCCT	27	<lmxll>	ll	260	lm	3		
>ref-73319	CCTCTCTTAACTTTCCCTCCATCTCCT	26	<lmxll>	ll	140	lm	119		
>ref-73323	AGATCACTAACGAAAGCTCCTTACAGC	22	<lmxll>	ll	120	lm	124		
>ref-73326	CCCCACTCTACCTGAGCTCCCTGATCC	16	<lmxll>	ll	139	lm	120		
>ref-73336	AATTCAAAGACAAACACTCCCATCTTA	22	<lmxll>	ll	113	lm	143		
>ref-73341	AACCTCATTACTCTTTCTCCCTTCTCT	24	<lmxll>	ll	114	lm	134		
>ref-73356	TCCTCTCTCACTTATACTCCATCCTCT	14	<nnxnp>	nn	10	np	260		
>ref-73356	TCCTCTCTCACTTATACTCCATCCTCT	25	<nnxnp>	nn	30	np	226		
>ref-73366	AGTGAACTCACCTCCTCTCCCTCTTTG	26	<nnxnp>	nn	114	np	156		
>ref-73367	ATACCTGTGACAGGTGCTCCTCGAGGT	12	<lmxll>	ll	140	lm	122		
>ref-73375	AGACAAGCCACTCTACCTCCCGGGCAA	24	<lmxll>	ll	131	lm	138		
>ref-73381	ATGCGATACACCCCCCCTCCTCCTCCT	7	<lmxll>	ll	74	lm	183		
>ref-73381	ATGCGATACACCCCCCCTCCTCCTCCT	12	<lmxll>	ll	74	lm	183		
>ref-73402	CACTCTCTCACACACTCTCCCTCCACA	12	<hkxhk>	hh	0	hk	258	kk	4
>ref-73402	CACTCTCTCACACACTCTCCCTCCACA	13	<lmxll>	ll	92	lm	153		
>ref-73402	CACTCTCTCACACACTCTCCCTCCACA	22	<hkxhk>	hh	4	hk	260	kk	0
>ref-73437	GCAAACATGACAAGATCTCCGGAACTT	25	<nnxnp>	nn	249	np	0		
>ref-73444	TCACCCTCCACTCTCCCTCCCAAGTCC	6	<lmxll>	ll	140	lm	105		
>ref-73445	TACGATAATACTTCTGCTCCTTTCCCT	2	<nnxnp>	nn	86	np	140		
>ref-73506	TAACCATATACTTGGCCTCCTTAGGAG	5	<hkxhk>	hh	0	hk	271	kk	0
>ref-73506	TAACCATATACTTGGCCTCCTTAGGAG	15	<hkxhk>	hh	0	hk	271	kk	0
>ref-73522	TGAAGGATTACCAAGACTCCTTTTCAT	9	<nnxnp>	nn	271	np	0		
>ref-73528	ATGATAATTACAAACTCTCCCGCCTAT	21	<hkxhk>	hh	34	hk	152	kk	49
>ref-73560	CCTATTATCACCCCCTCTCCTCCTCTC	5	<nnxnp>	nn	8	np	254		

>ref-73560	CCTATTATCACCCCCTCTCCTCCTCTC	7	<nnxnp>	nn	8	np	254		
>ref-73571	ATAAACACAACGTTCACTCCAGCGGTG	21	<nnxnp>	nn	126	np	147		
>ref-73571	ATAAACACAACGTTCACTCCAGCGGTG	23	<nnxnp>	nn	126	np	147		
>ref-73594	CTCTCTTTCACTTTGCCTCCTACTCTT	14	<hkxhk>	hh	9	hk	178	kk	62
>ref-73594	CTCTCTTTCACTTTGCCTCCTACTCTT	15	<nnxnp>	nn	13	np	234		
>ref-73600	GGGCCACTTACCGGCTCTCCACCTTTC	5	<nnxnp>	nn	136	np	132		
>ref-73667	CTCTCTTCCACATCCTCTCCCTATCTC	25	<nnxnp>	nn	267	np	0		
>ref-73667	CTCTCTTCCACATCCTCTCCCTATCTC	27	<nnxnp>	nn	264	np	0		
>ref-73690	CTCGGCCTAACTCCACCTCCTCCCTTC	1	<nnxnp>	nn	260	np	1		
>ref-73690	CTCGGCCTAACTCCACCTCCTCCCTTC	6	<nnxnp>	nn	259	np	0		
>ref-73695	GGAGAGGGTACGCCGTCTCCTCTCGTA	7	<nnxnp>	nn	134	np	135		
>ref-73704	CATTCACCCACCCATCCTCCTACAAAA	3	<hkxhk>	hh	0	hk	190	kk	64
>ref-73707	CTCACACACACCCATTCTCCTACACAG	2	<lmxll>	ll	102	lm	154		
>ref-73728	CCCTGCTTAACGCTCTCTCCTGCGACG	6	<lmxll>	ll	215	lm	41		
>ref-73729	CTCCTCCCCACTCTACCTCCTATACCT	5	<hkxhk>	hh	3	hk	265	kk	2
>ref-73729	CTCCTCCCCACTCTACCTCCTATACCT	22	<hkxhk>	hh	2	hk	264	kk	3
>ref-73747	CCCCCATGAACCATCGCTCCCTTAAAG	8	<lmxll>	ll	210	lm	30		
>ref-73747	CCCCCATGAACCATCGCTCCCTTAAAG	13	<lmxll>	ll	135	lm	84		
>ref-73747	CCCCCATGAACCATCGCTCCCTTAAAG	16	<lmxll>	ll	6	lm	252		
>ref-73752	ATCCATATAACCCTTACTCCCTCATCG	5	<lmxll>	ll	132	lm	136		
>ref-73757	TGAACACACACACTCTCTCCCTCCCTG	5	<lmxll>	ll	172	lm	92		
>ref-73757	TGAACACACACACTCTCTCCCTCCCTG	6	<hkxhk>	hh	0	hk	273	kk	0
>ref-73757	TGAACACACACACTCTCTCCCTCCCTG	24	<hkxhk>	hh	0	hk	273	kk	0
>ref-73757	TGAACACACACACTCTCTCCCTCCCTG	27	<lmxll>	ll	157	lm	104		
>ref-73812	AACCTCCCAACACCTCCTCCTTCTTTT	14	<lmxll>	ll	105	lm	164		
>ref-73826	TCGACCCCCACCACCCCTCCCCCCAAT	3	<hkxhk>	hh	7	hk	261	kk	0
>ref-73826	TCGACCCCCACCACCCCTCCCCCCAAT	9	<hkxhk>	hh	0	hk	138	kk	108
>ref-73826	TCGACCCCCACCACCCCTCCCCCCAAT	14	<hkxhk>	hh	185	hk	70	kk	0
>ref-73826	TCGACCCCCACCACCCCTCCCCCCAAT	26	<lmxll>	ll	143	lm	95		
>ref-73836	CCCTCCCTGACCAGGACTCCAGTCTCT	5	<lmxll>	ll	140	lm	126		
>ref-73836	CCCTCCCTGACCAGGACTCCAGTCTCT	15	<lmxll>	ll	95	lm	159		
>ref-73836	CCCTCCCTGACCAGGACTCCAGTCTCT	22	<lmxll>	ll	5	lm	265		
>ref-73840	ACTTCTCATACAAGAACTCCCTCATAC	4	<hkxhk>	hh	0	hk	260	kk	9
>ref-73840	ACTTCTCATACAAGAACTCCCTCATAC	12	<hkxhk>	hh	2	hk	271	kk	0
>ref-73844	GCACGTAGTACACGCCCTCCGTGTTGT	1	<hkxhk>	hh	81	hk	178	kk	0
>ref-73844	GCACGTAGTACACGCCCTCCGTGTTGT	9	<hkxhk>	hh	0	hk	273	kk	0
>ref-73845	GAAGCTGTCACATCCTCTCCTTTCCCC	25	<lmxll>	ll	152	lm	117		
>ref-73864	ACTTCCCTCACTCTTTCTCCTCACTTC	2	<nnxnp>	nn	266	np	0		

>ref-73884	TCTTTCTCGACTCATTCTCCTTCTCCT	13	<lmxll>	ll	154	lm	104		
>ref-73897	GAGTCACAGACAACTACTCCATAGCCG	22	<hkxhk>	hh	0	hk	270	kk	1
>ref-73897	GAGTCACAGACAACTACTCCATAGCCG	27	<hkxhk>	hh	0	hk	270	kk	1
>ref-73912	CCTCTCTCAACCCTCCCTCCTCTCCAG	13	<nnxnp>	nn	251	np	0		
>ref-73949	GACAAACAGACAAATTCTCCCCGACGG	27	<lmxll>	ll	116	lm	140		
>ref-73958	CTCCATCTAACCATCCCTCCCTCTCCT	23	<lmxll>	ll	107	lm	130		
>ref-73964	TGACCTGCCACCCCCCCTCCCCCCTTT	12	<hkxhk>	hh	0	hk	268	kk	2
>ref-73964	TGACCTGCCACCCCCCCTCCCCCCTTT	26	<hkxhk>	hh	0	hk	270	kk	0
>ref-73975	ATTTCCTTTACTCTCTCTCCCCTACCT	23	<nnxnp>	nn	128	np	101		
>ref-74001	CCCGAAAACACTCCCTCTCCGCCTTCC	16	<lmxll>	ll	118	lm	143		
>ref-74015	TATGTATACACGCTCTCTCCTTCCCTG	8	<nnxnp>	nn	125	np	145		
>ref-74015	TATGTATACACGCTCTCTCCTTCCCTG	27	<nnxnp>	nn	125	np	145		
>ref-74067	CTTGTCTCCACCTTCCCTCCTCACTCC	4	<nnxnp>	nn	255	np	1		
>ref-74067	CTTGTCTCCACCTTCCCTCCTCACTCC	5	<nnxnp>	nn	250	np	2		
>ref-74082	TTTTGGTCAACATTGCCTCCAAGATTA	27	<lmxll>	ll	126	lm	100		
>ref-74085	AAAAGCACAACACCATCTCCAACTGCC	13	<lmxll>	ll	116	lm	117		
>ref-74085	AAAAGCACAACACCATCTCCAACTGCC	16	<lmxll>	ll	114	lm	117		
>ref-74096	TACAGTCTCACTCTCCCTCCCCTCTCT	5	<lmxll>	ll	268	lm	0		
>ref-74096	TACAGTCTCACTCTCCCTCCCCTCTCT	25	<lmxll>	ll	123	lm	141		
>ref-74144	ACGAACTGCACTATAACTCCTGTCGAT	23	<lmxll>	ll	114	lm	149		
>ref-74155	TGAACGTGCACGTGCTCTCCCCAGCCC	13	<hkxhk>	hh	42	hk	154	kk	68
>ref-74171	TAGGCCGATACCCTCGCTCCCCAACAC	12	<nnxnp>	nn	87	np	173		
>ref-74178	CGCGCACATACTCTGTCTCCTTCCCTC	1	<lmxll>	ll	110	lm	156		
>ref-74178	CGCGCACATACTCTGTCTCCTTCCCTC	24	<hkxhk>	hh	56	hk	149	kk	61
>ref-74181	GTGATAGAAACGGGTGCTCCTGACTAG	12	<lmxll>	ll	121	lm	129		
>ref-74263	CCTGCCAATACCCCCTCTCCCTCACCA	4	<hkxhk>	hh	2	hk	206	kk	54
>ref-74263	CCTGCCAATACCCCCTCTCCCTCACCA	13	<hkxhk>	hh	54	hk	206	kk	1
>ref-74263	CCTGCCAATACCCCCTCTCCCTCACCA	24	<lmxll>	ll	4	lm	263		
>ref-74263	CCTGCCAATACCCCCTCTCCCTCACCA	26	<lmxll>	ll	4	lm	263		
>ref-74268	AGGGTTAAGACAGCGGCTCCGCGTGAC	25	<nnxnp>	nn	119	np	153		
>ref-74274	AGCAAGAAAACTGCGCCTCCAGATTTA	13	<nnxnp>	nn	121	np	141		
>ref-74275	TTTTCTATGACCTTCCCTCCCTTTGCC	1	<hkxhk>	hh	0	hk	273	kk	0
>ref-74275	TTTTCTATGACCTTCCCTCCCTTTGCC	5	<lmxll>	ll	1	lm	270		
>ref-74280	ATTTAAGACACGCCTCCTCCCCTCAAT	13	<nnxnp>	nn	271	np	0		
>ref-74292	ACCCCTGGCACCTCTCCTCCTCTCCCC	3	<lmxll>	ll	127	lm	125		
>ref-74303	CCGTATCTCACTCCCTCTCCTTTATTT	3	<hkxhk>	hh	22	hk	241	kk	0
>ref-74303	CCGTATCTCACTCCCTCTCCTTTATTT	7	<hkxhk>	hh	22	hk	240	kk	0
>ref-74305	CACACATACACATATACTCCCTTACAA	1	<hkxhk>	hh	52	hk	142	kk	37

>ref-74312	CTCTCTTCTACCTTCTCTCCTCCCCTT	6	<nnxnp>	nn	149	np	115		
>ref-74312	CTCTCTTCTACCTTCTCTCCTCCCCTT	12	<lmxll>	ll	97	lm	150		
>ref-74312	CTCTCTTCTACCTTCTCTCCTCCCCTT	21	<lmxll>	ll	107	lm	143		
>ref-74312	CTCTCTTCTACCTTCTCTCCTCCCCTT	24	<nnxnp>	nn	148	np	114		
>ref-74326	AGGCCCTTAACCCCCCCTCCCCCTCTC	3	<hkxhk>	hh	0	hk	253	kk	4
>ref-74326	AGGCCCTTAACCCCCCCTCCCCCTCTC	6	<hkxhk>	hh	5	hk	253	kk	0
>ref-74326	AGGCCCTTAACCCCCCCTCCCCCTCTC	22	<lmxll>	ll	128	lm	128		
>ref-74327	AATATAGTAACATATGCTCCCACTGCG	3	<lmxll>	ll	4	lm	252		
>ref-74327	AATATAGTAACATATGCTCCCACTGCG	21	<lmxll>	ll	2	lm	228		
>ref-74327	AATATAGTAACATATGCTCCCACTGCG	26	<lmxll>	ll	4	lm	252		
>ref-74327	AATATAGTAACATATGCTCCCACTGCG	27	<lmxll>	ll	2	lm	227		
>ref-74334	TATTCTGTCACTCTCCCTCCTCTTTAC	12	<lmxll>	ll	132	lm	87		
>ref-74349	TTCCGCTTGACGTACTCTCCTCTCTCT	2	<nnxnp>	nn	267	np	0		
>ref-74351	CATCTTGTTACAGGATCTCCTCTGCCA	2	<lmxll>	ll	140	lm	128		
>ref-74351	CATCTTGTTACAGGATCTCCTCTGCCA	4	<lmxll>	ll	115	lm	142		
>ref-74351	CATCTTGTTACAGGATCTCCTCTGCCA	23	<hkxhk>	hh	61	hk	131	kk	67
>ref-74351	CATCTTGTTACAGGATCTCCTCTGCCA	27	<lmxll>	ll	115	lm	142		
>ref-74366	AACTATTATACATCTACTCCCTCGCTT	24	<lmxll>	ll	104	lm	121		
>ref-74373	CCGTCCCCAACTCCGTCTCCGCGCCAC	3	<lmxll>	ll	155	lm	111		
>ref-74378	CCTCCTCCTACTTAGTCTCCTTCTTAT	7	<nnxnp>	nn	3	np	243		
>ref-74384	TCCTCCTGTACTCCCTCTCCTTCTGTT	2	<lmxll>	ll	124	lm	136		
>ref-74387	GCCATGACCACTCCCCCTCCAACACAT	27	<nnxnp>	nn	267	np	0		
>ref-74418	CTCCCCTTTACAACTCCTCCCTCCCTC	25	<nnxnp>	nn	117	np	130		
>ref-74420	ACAACAGACACACTTACTCCTGCAGGA	4	<lmxll>	ll	10	lm	259		
>ref-74443	AACCTCATTACCCTTCCTCCTTCTCCC	22	<lmxll>	ll	266	lm	1		
>ref-74475	CCTCCTTGGACACCTCCTCCTCCTCCT	8	<lmxll>	ll	86	lm	175		
>ref-74475	CCTCCTTGGACACCTCCTCCTCCTCCT	9	<nnxnp>	nn	222	np	24		
>ref-74475	CCTCCTTGGACACCTCCTCCTCCTCCT	12	<lmxll>	ll	255	lm	7		
>ref-74489	ACAAAGTTAACTGCCTCTCCAACTATA	1	<nnxnp>	nn	120	np	112		
>ref-74489	ACAAAGTTAACTGCCTCTCCAACTATA	4	<lmxll>	ll	103	lm	123		
>ref-74511	GAAGGACGTACTTGTTCTCCCTCTGAC	23	<lmxll>	ll	129	lm	136		
>ref-74518	AGAACTAACACGCAAACTCCGCCTCTA	14	<nnxnp>	nn	128	np	143		
>ref-74538	GGCACTCCTACCTCTACTCCATGCACT	21	<lmxll>	ll	118	lm	139		
>ref-74544	ACTGCTTTTACTCCACCTCCATCAGCT	25	<nnxnp>	nn	120	np	128		
>ref-74546	CCATATAACACGCGAACTCCCTCGGTT	1	<nnxnp>	nn	239	np	2		
>ref-74546	CCATATAACACGCGAACTCCCTCGGTT	2	<nnxnp>	nn	248	np	0		
>ref-74546	CCATATAACACGCGAACTCCCTCGGTT	13	<lmxll>	ll	110	lm	124		
>ref-74550	CTGGCCAATACACAATCTCCTGAGTCA	22	<lmxll>	ll	110	lm	151		

>ref-74561	ATCCACCTTACCATCCCTCCGTTCTCT	5	<hkxhk>	hh	39	hk	225	kk	0
>ref-74561	ATCCACCTTACCATCCCTCCGTTCTCT	16	<lmxll>	ll	2	lm	270		
>ref-74561	ATCCACCTTACCATCCCTCCGTTCTCT	27	<hkxhk>	hh	0	hk	272	kk	0
>ref-74572	TGATTGCAAACATGACCTCCTTCCAGT	24	<lmxll>	ll	155	lm	114		
>ref-74613	TCTCCTTCTACCCCCCCTCCTTTCCCT	27	<lmxll>	ll	46	lm	196		
>ref-74645	ACCCAGTTCACCTCACCTCCCATTCTT	15	<hkxhk>	hh	72	hk	138	kk	52
>ref-74669	CCACCCTGTACTAATCCTCCCCTGCCT	13	<lmxll>	ll	120	lm	137		
>ref-74696	CCTTCATCTACTCTTCCTCCTACACTA	16	<nnxnp>	nn	163	np	103		
>ref-74703	CTATCAACCACCCGTCCTCCTTACTCC	7	<nnxnp>	nn	116	np	143		
>ref-74723	GCACCATGGACGAGATCTCCTCCGGGT	1	<hkxhk>	hh	29	hk	203	kk	0
>ref-74723	GCACCATGGACGAGATCTCCTCCGGGT	23	<hkxhk>	hh	0	hk	273	kk	0
>ref-74736	CGCCGTCTAACTTGGCCTCCGCCCGTT	3	<hkxhk>	hh	4	hk	267	kk	0
>ref-74736	CGCCGTCTAACTTGGCCTCCGCCCGTT	25	<hkxhk>	hh	0	hk	267	kk	3
>ref-74737	TACCTACCTACCCCCCCTCCCCCTGCC	5	<lmxll>	ll	115	lm	116		
>ref-74737	TACCTACCTACCCCCCCTCCCCCTGCC	8	<lmxll>	ll	115	lm	114		
>ref-74737	TACCTACCTACCCCCCCTCCCCCTGCC	25	<lmxll>	ll	55	lm	182		
>ref-74759	CAGATTATCACTGCCTCTCCCCCATCC	5	<hkxhk>	hh	46	hk	118	kk	63
>ref-74766	CTCCTCATTACTCCCTCTCCTCTCTCT	7	<lmxll>	ll	96	lm	152		
>ref-74766	CTCCTCATTACTCCCTCTCCTCTCTCT	12	<lmxll>	ll	105	lm	146		
>ref-74776	GGATCCCCTACTTCCCCTCCCTTCCCT	8	<hkxhk>	hh	53	hk	131	kk	46
>ref-74787	AAAATAATCACAGTTACTCCTCATGTA	4	<hkxhk>	hh	23	hk	233	kk	1
>ref-74787	AAAATAATCACAGTTACTCCTCATGTA	6	<hkxhk>	hh	23	hk	233	kk	1
>ref-74798	CCCACTCCCACCCACACTCCAACACTT	7	<lmxll>	ll	104	lm	164		
>ref-74807	AGCATTTCGACACAAACTCCTGTTCTT	26	<lmxll>	ll	116	lm	128		
>ref-74809	TCAGCCTCGACGTATCCTCCGTCATTG	27	<nnxnp>	nn	126	np	143		
>ref-74819	GTCCTCCTTACGGGCCCTCCTCCTCCT	23	<nnxnp>	nn	111	np	144		
>ref-74826	CCTAGTGGTACAGGCACTCCTGGAACC	15	<lmxll>	ll	161	lm	85		
>ref-74826	CCTAGTGGTACAGGCACTCCTGGAACC	21	<lmxll>	ll	93	lm	146		
>ref-74832	GTCCCTCCCACTCCCTCTCCATCCCGC	14	<lmxll>	ll	10	lm	251		
>ref-74832	GTCCCTCCCACTCCCTCTCCATCCCGC	16	<lmxll>	ll	10	lm	252		
>ref-74838	TAAACCGAAACCATCTCTCCCTCTCCC	14	<lmxll>	ll	252	lm	0		
>ref-74852	CGTTTCTCCACTTTCCCTCCGTCTTCC	16	<lmxll>	ll	119	lm	119		
>ref-74871	CACATTTACACCTTCCCTCCCTCCCTC	6	<hkxhk>	hh	4	hk	255	kk	9
>ref-74871	CACATTTACACCTTCCCTCCCTCCCTC	8	<nnxnp>	nn	63	np	159		
>ref-74871	CACATTTACACCTTCCCTCCCTCCCTC	12	<hkxhk>	hh	3	hk	260	kk	5
>ref-74876	CCTTAATACACATCCTCTCCTCCCCCA	13	<lmxll>	ll	126	lm	123		
>ref-74885	GGTGCAAGGACACAGCCTCCTCACCCC	25	<hkxhk>	hh	0	hk	272	kk	0
>ref-74901	ACTCTTCTCACTCTCTCTCCTTCACTT	2	<nnxnp>	nn	146	np	120		

>ref-74925	CCCCACACCACTTCCACTCCCACTCTT	13	<hkxhk>	hh	117	hk	111	kk	0
>ref-74955	GTGTCCCTCACTCTTTCTCCCTCCCTC	1	<hkxhk>	hh	0	hk	248	kk	0
>ref-74955	GTGTCCCTCACTCTTTCTCCCTCCCTC	3	<hkxhk>	hh	98	hk	164	kk	0
>ref-74955	GTGTCCCTCACTCTTTCTCCCTCCCTC	6	<hkxhk>	hh	270	hk	3	kk	0
>ref-74955	GTGTCCCTCACTCTTTCTCCCTCCCTC	16	<hkxhk>	hh	0	hk	268	kk	3
>ref-74992	GTTTTTGTGACTGAACCTCCATGTGTT	1	<hkxhk>	hh	32	hk	214	kk	0
>ref-74992	GTTTTTGTGACTGAACCTCCATGTGTT	12	<hkxhk>	hh	0	hk	214	kk	33
>ref-74992	GTTTTTGTGACTGAACCTCCATGTGTT	21	<hkxhk>	hh	7	hk	246	kk	3
>ref-74995	TGCCGCCGCACTGATGCTCCGCTCCCT	13	<lmxll>	ll	272	lm	0		
>ref-74997	GCTCTAATGACCCGCTCTCCCTTTACA	22	<lmxll>	ll	125	lm	139		
>ref-75000	GTGAAGTGTACGAATTCTCCCAACGTG	8	<lmxll>	ll	16	lm	240		
>ref-75000	GTGAAGTGTACGAATTCTCCCAACGTG	12	<lmxll>	ll	53	lm	194		
>ref-75042	TACATACATACTCTCACTCCTTACACT	9	<nnxnp>	nn	244	np	0		
>ref-75044	TTTTTGTGAACATTTTCTCCTTTCTGT	7	<nnxnp>	nn	242	np	0		
>ref-75050	CTCCTTTCAACCTCCCCTCCCTCCTTT	3	<hkxhk>	hh	30	hk	214	kk	1
>ref-75050	CTCCTTTCAACCTCCCCTCCCTCCTTT	22	<hkxhk>	hh	1	hk	214	kk	30
>ref-75051	ATCCAGAACACCATTCCTCCATTTGGT	4	<nnxnp>	nn	272	np	1		
>ref-75051	ATCCAGAACACCATTCCTCCATTTGGT	24	<nnxnp>	nn	264	np	4		
>ref-75056	CGTTCCTACACCCCTCCTCCATCAACC	2	<lmxll>	ll	135	lm	106		
>ref-75067	GCCACCTATACAGTGTCTCCATGAACT	25	<nnxnp>	nn	102	np	122		
>ref-75090	CCTGATGCTACATCGCCTCCTTTTATG	8	<lmxll>	ll	129	lm	130		
>ref-75094	CCCTTCCTCACTTCTTCTCCCTCCTTC	5	<nnxnp>	nn	46	np	209		
>ref-75094	CCCTTCCTCACTTCTTCTCCCTCCTTC	13	<nnxnp>	nn	39	np	207		
>ref-75094	CCCTTCCTCACTTCTTCTCCCTCCTTC	27	<hkxhk>	hh	37	hk	218	kk	0
>ref-75104	GTCACTCACACCCTCCCTCCCTCATTC	1	<nnxnp>	nn	122	np	135		
>ref-75104	GTCACTCACACCCTCCCTCCCTCATTC	2	<lmxll>	ll	118	lm	142		
>ref-75104	GTCACTCACACCCTCCCTCCCTCATTC	8	<nnxnp>	nn	257	np	5		
>ref-75104	GTCACTCACACCCTCCCTCCCTCATTC	24	<hkxhk>	hh	243	hk	11	kk	0
>ref-75122	CACTTACTAACTTCCTCTCCAGTAACT	2	<lmxll>	ll	28	lm	208		
>ref-75122	CACTTACTAACTTCCTCTCCAGTAACT	7	<nnxnp>	nn	154	np	88		
>ref-75122	CACTTACTAACTTCCTCTCCAGTAACT	15	<lmxll>	ll	28	lm	209		
>ref-75122	CACTTACTAACTTCCTCTCCAGTAACT	26	<nnxnp>	nn	154	np	88		
>ref-75128	CTTTGGTGCACTTCTACTCCCCAACAC	23	<lmxll>	ll	130	lm	136		
>ref-75144	ATCACGGTCACCCGGGCTCCCTCCGCG	14	<nnxnp>	nn	213	np	37		
>ref-75144	ATCACGGTCACCCGGGCTCCCTCCGCG	16	<lmxll>	ll	159	lm	83		
>ref-75144	ATCACGGTCACCCGGGCTCCCTCCGCG	23	<lmxll>	ll	165	lm	89		
>ref-75165	TTCCAATCCACCTAATCTCCCCTTTCC	16	<hkxhk>	hh	0	hk	269	kk	0
>ref-75165	TTCCAATCCACCTAATCTCCCCTTTCC	26	<hkxhk>	hh	0	hk	269	kk	0

>ref-75169	CTTTCCTCCACCCACCCTCCCCCCTTC	5	<nnxnp>	nn	247	np	0		
>ref-75172	TCTTCCGGTACCTTCCCTCCAGCTCTT	27	<lmxll>	ll	104	lm	161		
>ref-75194	CCACACTGCACGCACACTCCCGAACTC	22	<nnxnp>	nn	112	np	154		
>ref-75234	CTCTCCCCCACTCCCTCTCCCTTTGAA	1	<nnxnp>	nn	10	np	263		
>ref-75234	CTCTCCCCCACTCCCTCTCCCTTTGAA	24	<nnxnp>	nn	10	np	262		
>ref-75239	TCCTCAATCACCCTCCCTCCTTCTCAT	21	<nnxnp>	nn	127	np	139		
>ref-75247	CTCCCCTTCACCTCCTCTCCCCACATC	1	<hkxhk>	hh	2	hk	262	kk	0
>ref-75247	CTCCCCTTCACCTCCTCTCCCCACATC	12	<lmxll>	ll	255	lm	6		
>ref-75254	CCAACACCCACTAGACCTCCTCCACCT	7	<hkxhk>	hh	78	hk	195	kk	0
>ref-75256	CTCCCTCTCACACTTCCTCCCATCCTG	3	<hkxhk>	hh	194	hk	52	kk	0
>ref-75256	CTCCCTCTCACACTTCCTCCCATCCTG	27	<hkxhk>	hh	0	hk	55	kk	192
>ref-75264	GAGAGACGAACTACTTCTCCGGGAAAA	4	<nnxnp>	nn	261	np	0		
>ref-75264	GAGAGACGAACTACTTCTCCGGGAAAA	8	<lmxll>	ll	134	lm	122		
>ref-75268	ACAGCAACCACCCATTCTCCTCCTGCT	23	<nnxnp>	nn	131	np	131		
>ref-75278	TGGTTTTAGACTTGACCTCCTTCCAGT	25	<lmxll>	ll	91	lm	130		
>ref-75318	ACATTTTAAACAAACACTCCGATGCCT	4	<hkxhk>	hh	0	hk	269	kk	0
>ref-75335	CCTGCACATACCACAGCTCCAGGCGCA	5	<hkxhk>	hh	5	hk	262	kk	0
>ref-75335	CCTGCACATACCACAGCTCCAGGCGCA	7	<hkxhk>	hh	5	hk	262	kk	0
>ref-75335	CCTGCACATACCACAGCTCCAGGCGCA	9	<hkxhk>	hh	0	hk	202	kk	69
>ref-75342	TAACCCGTGACTGACCCTCCATAAGCA	5	<lmxll>	ll	107	lm	138		
>ref-75343	CATTTCATTACTCTTGCTCCACCTCAG	14	<lmxll>	ll	125	lm	137		
>ref-75344	CTCTCCCTCACACTCGCTCCGCTCCCT	13	<lmxll>	ll	0	lm	272		
>ref-75344	CTCTCCCTCACACTCGCTCCGCTCCCT	16	<lmxll>	ll	0	lm	271		
>ref-75347	CATACACAAACTCTCTCTCCCCACACA	24	<lmxll>	ll	127	lm	132		
>ref-75353	TTCTTCTTCACCACCTCTCCTTCTCCT	23	<nnxnp>	nn	122	np	142		
>ref-75356	AGGAGTTAAACCCAAACTCCAAGGGTA	3	<lmxll>	ll	23	lm	240		
>ref-75356	AGGAGTTAAACCCAAACTCCAAGGGTA	7	<nnxnp>	nn	140	np	129		
>ref-75356	AGGAGTTAAACCCAAACTCCAAGGGTA	24	<lmxll>	ll	154	lm	109		
>ref-75358	AGAGCCAAGACCCCATCTCCGTCAAGC	15	<lmxll>	ll	130	lm	135		
>ref-75405	TAAAAATGTACTCGTTCTCCGACGATG	22	<lmxll>	ll	125	lm	122		
>ref-75419	CCGAATCTTACAGACTCTCCCATATAT	14	<hkxhk>	hh	46	hk	202	kk	0
>ref-75419	CCGAATCTTACAGACTCTCCCATATAT	16	<hkxhk>	hh	0	hk	168	kk	73
>ref-75419	CCGAATCTTACAGACTCTCCCATATAT	21	<hkxhk>	hh	74	hk	167	kk	0
>ref-75419	CCGAATCTTACAGACTCTCCCATATAT	22	<hkxhk>	hh	22	hk	235	kk	0
>ref-75420	TTGCTATTTACGCCTCCTCCTCCTCTT	3	<lmxll>	ll	253	lm	1		
>ref-75428	CTCTTATTGACCCCCCCTCCCTCACTG	2	<lmxll>	ll	103	lm	121		
>ref-75460	GCAATACCAACAGCTGCTCCACACGAT	24	<lmxll>	ll	118	lm	116		
>ref-75526	TATTTGCTTACTTCTCCTCCTCCTCCT	7	<lmxll>	ll	30	lm	229		

>ref-75526	TATTTGCTTACTTCTCCTCCTCCTCCT	8	<nnxnp>	nn	111	np	129		
>ref-75552	GATATTACTACATTATCTCCCTCCTTG	26	<lmxll>	ll	121	lm	133		
>ref-75568	TTCCTTTTTACCTTGCCTCCTCTCCCC	12	<nnxnp>	nn	130	np	122		
>ref-75607	TACAACAGAACACTCTCTCCTTGATTT	1	<lmxll>	ll	126	lm	117		
>ref-75607	TACAACAGAACACTCTCTCCTTGATTT	24	<lmxll>	ll	126	lm	117		
>ref-75608	CACACCCTCACCCCCTCTCCTAATGCA	8	<lmxll>	ll	153	lm	112		
>ref-75621	CAGACAGGCACAGCATCTCCTCCTTAT	24	<lmxll>	ll	125	lm	143		
>ref-75627	CCCTCTCCCACCTGCTCTCCTTCCCTT	1	<lmxll>	ll	262	lm	1		
>ref-75627	CCCTCTCCCACCTGCTCTCCTTCCCTT	3	<lmxll>	ll	259	lm	0		
>ref-75627	CCCTCTCCCACCTGCTCTCCTTCCCTT	6	<lmxll>	ll	11	lm	243		
>ref-75627	CCCTCTCCCACCTGCTCTCCTTCCCTT	14	<lmxll>	ll	10	lm	251		
>ref-75643	CCCTCTCCGACTTCTCCTCCTTCCCCT	12	<hkxhk>	hh	1	hk	151	kk	110
>ref-75643	CCCTCTCCGACTTCTCCTCCTTCCCCT	14	<hkxhk>	hh	0	hk	259	kk	9
>ref-75643	CCCTCTCCGACTTCTCCTCCTTCCCCT	16	<hkxhk>	hh	0	hk	259	kk	9
>ref-75648	CACACAATTACACACTCTCCATCTGCA	13	<lmxll>	ll	134	lm	126		
>ref-75665	TTCCTTTAGACATGGCCTCCCCTTTAA	23	<nnxnp>	nn	256	np	0		
>ref-75675	ACGAACTTCACTTCTCCTCCTCCCCCT	22	<nnxnp>	nn	145	np	113		
>ref-75676	AGCCCCTTAACACGCCCTCCCCTTCTC	1	<hkxhk>	hh	80	hk	126	kk	51
>ref-75676	AGCCCCTTAACACGCCCTCCCCTTCTC	24	<hkxhk>	hh	51	hk	126	kk	79
>ref-75683	CCTCTTCCTACTTCCCCTCCCACACCT	4	<lmxll>	ll	139	lm	131		
>ref-75692	CACTTTTACACAATAACTCCTGCACCG	24	<lmxll>	ll	113	lm	127		
>ref-75711	TTTCCCGCCACTATAGCTCCCATCCAG	7	<lmxll>	ll	116	lm	147		
>ref-75715	TCTCCCCCTACTCTGTCTCCCACTCTC	15	<nnxnp>	nn	53	np	187		
>ref-75717	GAATGGCCGACTTTACCTCCGTAGCCC	27	<nnxnp>	nn	116	np	137		
>ref-75720	TCCAAGTCAACAAATTCTCCTGTAATG	2	<hkxhk>	hh	98	hk	123	kk	0
>ref-75720	TCCAAGTCAACAAATTCTCCTGTAATG	15	<hkxhk>	hh	0	hk	224	kk	18
>ref-75720	TCCAAGTCAACAAATTCTCCTGTAATG	24	<hkxhk>	hh	18	hk	225	kk	0
>ref-75721	ATATCAACCACTCAGCCTCCCACTACA	2	<hkxhk>	hh	0	hk	273	kk	0
>ref-75721	ATATCAACCACTCAGCCTCCCACTACA	3	<nnxnp>	nn	267	np	6		
>ref-75721	ATATCAACCACTCAGCCTCCCACTACA	4	<hkxhk>	hh	0	hk	273	kk	0
>ref-75722	CGGAGAACTACCAAGACTCCTCAGACT	3	<lmxll>	ll	124	lm	131		
>ref-75760	ACACTGACCACGCCCCCTCCCCCTCCC	25	<lmxll>	ll	115	lm	137		
>ref-75762	CCTTCATCTACTCTTCCTCCAATTCCC	15	<hkxhk>	hh	2	hk	256	kk	5
>ref-75769	TGTAAAAGGACGCGCACTCCTTCACAG	15	<lmxll>	ll	143	lm	122		
>ref-75794	AGATATCTCACCTCTTCTCCTTCCCCT	7	<lmxll>	ll	266	lm	0		
>ref-75803	ACGACAGCCACAGCCACTCCAACGCCT	1	<lmxll>	ll	4	lm	262		
>ref-75803	ACGACAGCCACAGCCACTCCAACGCCT	3	<lmxll>	ll	4	lm	262		
>ref-75823	GGAGTCCGTACCTACACTCCCTACTGC	9	<nnxnp>	nn	272	np	0		

>ref-75828	AGGAGTCTAACCCCTTCTCCTCCCCCT	24	<lmxll>	ll	135	lm	133		
>ref-75828	AGGAGTCTAACCCCTTCTCCTCCCCCT	25	<lmxll>	ll	137	lm	131		
>ref-75850	TTCTCCTTAACCTCTCCTCCTCCCATA	9	<hkxhk>	hh	0	hk	255	kk	6
>ref-75850	TTCTCCTTAACCTCTCCTCCTCCCATA	27	<hkxhk>	hh	0	hk	257	kk	5
>ref-75889	GTATGACCGACCAGACCTCCGCTGCAC	21	<hkxhk>	hh	50	hk	149	kk	61
>ref-75892	ATTCCCGTAACCCTCTCTCCTTCCCTC	25	<nnxnp>	nn	130	np	124		
>ref-75898	TCTCATCGTACCCTCTCTCCACTCTGT	21	<nnxnp>	nn	99	np	158		
>ref-75982	TCCCTTACTACCTTCCCTCCCCCCTAA	9	<lmxll>	ll	2	lm	267		
>ref-75982	TCCCTTACTACCTTCCCTCCCCCCTAA	27	<lmxll>	ll	2	lm	267		
>ref-75984	GTGATCACTACGGTTTCTCCTGTCTTT	1	<nnxnp>	nn	127	np	122		
>ref-75986	ACGTCCACAACTGCCCCTCCCTCTTCC	13	<nnxnp>	nn	144	np	122		
>ref-75992	CCCTTCTCGACACCCCCTCCTCATTCC	3	<nnxnp>	nn	129	np	112		
>ref-76002	CCATTTCATACTCCTCCTCCTTTCTCT	5	<lmxll>	ll	117	lm	126		
>ref-76002	CCATTTCATACTCCTCCTCCTTTCTCT	16	<lmxll>	ll	119	lm	129		
>ref-76010	TTAAACTATACTCTTCCTCCTCCTCTT	1	<nnxnp>	nn	17	np	245		
>ref-76010	TTAAACTATACTCTTCCTCCTCCTCTT	3	<nnxnp>	nn	3	np	255		
>ref-76010	TTAAACTATACTCTTCCTCCTCCTCTT	6	<nnxnp>	nn	17	np	245		
>ref-76013	TCCCGAGGCACGTCCACTCCAGCCGAC	13	<nnxnp>	nn	270	np	0		
>ref-76017	AGTTAAGAGACGGGAACTCCCTTGTAA	23	<nnxnp>	nn	144	np	116		
>ref-76038	TGTCTACTCACTTTCTCTCCCTCACCC	9	<lmxll>	ll	263	lm	9		
>ref-76038	TGTCTACTCACTTTCTCTCCCTCACCC	15	<nnxnp>	nn	149	np	117		
>ref-76046	TGTTTCTTAACTGGTTCTCCCCCACCA	23	<nnxnp>	nn	106	np	136		
>ref-76050	CAGAGCTACACAGGAGCTCCTCACAAT	12	<lmxll>	ll	93	lm	166		
>ref-76050	CAGAGCTACACAGGAGCTCCTCACAAT	23	<hkxhk>	hh	11	hk	257	kk	0
>ref-76050	CAGAGCTACACAGGAGCTCCTCACAAT	24	<nnxnp>	nn	262	np	10		
>ref-76050	CAGAGCTACACAGGAGCTCCTCACAAT	27	<lmxll>	ll	0	lm	273		
>ref-76121	CCTCCATTAACAATGTCTCCTGAAGCA	2	<hkxhk>	hh	0	hk	257	kk	10
>ref-76121	CCTCCATTAACAATGTCTCCTGAAGCA	4	<hkxhk>	hh	11	hk	255	kk	0
>ref-76121	CCTCCATTAACAATGTCTCCTGAAGCA	13	<hkxhk>	hh	58	hk	204	kk	0
>ref-76121	CCTCCATTAACAATGTCTCCTGAAGCA	25	<hkxhk>	hh	0	hk	222	kk	8
>ref-76140	CACCTTCTCACCTTTTCTCCATCACCT	27	<nnxnp>	nn	257	np	0		
>ref-76157	CTTTCTTTCACCCGCCCTCCTCCCTCT	3	<lmxll>	ll	80	lm	147		
>ref-76157	CTTTCTTTCACCCGCCCTCCTCCCTCT	14	<lmxll>	ll	65	lm	157		
>ref-76164	CTTCCTCTTACAGTTACTCCAACTGCT	2	<nnxnp>	nn	137	np	128		
>ref-76164	CTTCCTCTTACAGTTACTCCAACTGCT	3	<nnxnp>	nn	137	np	128		
>ref-76168	ACCTCCCTAACTACCTCTCCCCCTCTC	21	<hkxhk>	hh	65	hk	142	kk	34
>ref-76172	TATCATGTTACTCCAACTCCCTTCCAC	1	<lmxll>	ll	96	lm	145		
>ref-76172	TATCATGTTACTCCAACTCCCTTCCAC	2	<lmxll>	ll	97	lm	144		

>ref-76172	TATCATGTTACTCCAACTCCCTTCCAC	27	<hkxhk>	hh	2	hk	270	kk	0
>ref-76184	GTCCCTCCCACATGCTCTCCTCTCCTC	1	<hkxhk>	hh	0	hk	271	kk	0
>ref-76184	GTCCCTCCCACATGCTCTCCTCTCCTC	2	<hkxhk>	hh	0	hk	271	kk	0
>ref-76200	ATAAAATCCACGTTTCCTCCTCCTCCT	12	<hkxhk>	hh	5	hk	260	kk	2
>ref-76200	ATAAAATCCACGTTTCCTCCTCCTCCT	27	<hkxhk>	hh	2	hk	260	kk	5
>ref-76207	AACCAACCTACCCTACCTCCCTCCCCC	26	<nnxnp>	nn	127	np	127		
>ref-76235	CATGACTCCACCTTCCCTCCTTCTTTC	25	<hkxhk>	hh	1	hk	270	kk	0
>ref-76253	CGGCGCCGGACTTTCTCTCCTCGTAAC	21	<nnxnp>	nn	118	np	139		
>ref-76253	CGGCGCCGGACTTTCTCTCCTCGTAAC	23	<nnxnp>	nn	119	np	139		
>ref-76256	TCCACAGGTACTTAGTCTCCAAGGAGT	9	<nnxnp>	nn	256	np	0		
>ref-76273	TTCCTCCGTACGTGCCCTCCTTTAACC	7	<nnxnp>	nn	119	np	136		
>ref-76323	CACCACAGCACATTGTCTCCTTCCATA	7	<lmxll>	ll	132	lm	137		
>ref-76324	GTGGCCTTCACCTCACCTCCACCCCCC	5	<lmxll>	ll	119	lm	133		
>ref-76325	CTTAGGACTACCAGCGCTCCTTCCAGA	1	<nnxnp>	nn	148	np	119		
>ref-76325	CTTAGGACTACCAGCGCTCCTTCCAGA	14	<nnxnp>	nn	143	np	128		
>ref-76338	TCTTCTATCACTCTACCTCCTTCCCCT	7	<hkxhk>	hh	6	hk	261	kk	0
>ref-76338	TCTTCTATCACTCTACCTCCTTCCCCT	16	<lmxll>	ll	136	lm	132		
>ref-76338	TCTTCTATCACTCTACCTCCTTCCCCT	22	<hkxhk>	hh	0	hk	261	kk	6
>ref-76341	AGTTAATGAACAATGGCTCCCTTTCTT	8	<lmxll>	ll	138	lm	96		
>ref-76341	AGTTAATGAACAATGGCTCCCTTTCTT	14	<lmxll>	ll	136	lm	97		
>ref-76347	TTTAAACGCACTATTTCTCCCCCTCTC	3	<nnxnp>	nn	96	np	132		
>ref-76403	CCTCCCTCTACGATTCCTCCTCCTCAT	1	<lmxll>	ll	106	lm	161		
>ref-76409	CTCTCTGAAACTCATCCTCCGGCGCCA	15	<hkxhk>	hh	0	hk	218	kk	49
>ref-76413	TTCGAATTAACTTGACCTCCCTTCGCT	13	<lmxll>	ll	134	lm	120		
>ref-76423	ACCACCCTTACCCCGACTCCACTGGGT	4	<lmxll>	ll	131	lm	112		
>ref-76432	AAATGCTATACACCTCCTCCCTCATCG	13	<lmxll>	ll	130	lm	116		
>ref-76476	CACACACACACACACCCTCCCCAAGCG	24	<nnxnp>	nn	246	np	6		
>ref-76476	CACACACACACACACCCTCCCCAAGCG	26	<nnxnp>	nn	254	np	3		
>ref-76484	AGTTAGAATACTTCATCTCCCAGTCAT	12	<nnxnp>	nn	109	np	149		
>ref-76500	CCTCTTTTTACTTTGTCTCCATTTCAC	4	<nnxnp>	nn	110	np	109		
>ref-76512	TCTCCCTCCACACCCTCTCCCTTCTGT	3	<lmxll>	ll	133	lm	98		
>ref-76622	TAAAATGGAACAGTGCCTCCCCTAGGG	24	<hkxhk>	hh	49	hk	134	kk	62
>ref-76622	TAAAATGGAACAGTGCCTCCCCTAGGG	25	<hkxhk>	hh	62	hk	134	kk	49
>ref-76630	ATCACCTTCACTACTTCTCCGCCCACA	4	<nnxnp>	nn	132	np	128		
>ref-76652	GATTGTGTTACTCTGCCTCCTTGTCCT	14	<nnxnp>	nn	141	np	126		
>ref-76652	GATTGTGTTACTCTGCCTCCTTGTCCT	15	<hkxhk>	hh	62	hk	150	kk	50
>ref-76655	CTCCTTCGTACCGTCCCTCCTCCCCCT	12	<lmxll>	ll	46	lm	196		
>ref-76655	CTCCTTCGTACCGTCCCTCCTCCCCCT	13	<lmxll>	ll	42	lm	196		

>ref-76661	AACCCTCGCACTTGCTCTCCATTTTGT	1	<hkxhk>	hh	0	hk	272	kk	0
>ref-76661	AACCCTCGCACTTGCTCTCCATTTTGT	4	<lmxll>	ll	97	lm	148		
>ref-76661	AACCCTCGCACTTGCTCTCCATTTTGT	14	<nnxnp>	nn	259	np	9		
>ref-76661	AACCCTCGCACTTGCTCTCCATTTTGT	15	<lmxll>	ll	96	lm	147		
>ref-76661	AACCCTCGCACTTGCTCTCCATTTTGT	27	<nnxnp>	nn	258	np	10		
>ref-76671	CTAAACACTACAACCACTCCACTCCAC	2	<hkxhk>	hh	0	hk	254	kk	9
>ref-76673	TATATCCTCACCATCTCTCCATCTCAA	13	<lmxll>	ll	114	lm	132		
>ref-76676	TAAGATAAAACACAATCTCCGTGATTC	2	<lmxll>	ll	113	lm	113		
>ref-76686	TCCTTACCAACACATTCTCCATCCCTG	27	<lmxll>	ll	262	lm	0		
>ref-76705	GCAGCTCCCACTCGGACTCCCACGATA	14	<nnxnp>	nn	136	np	136		
>ref-76732	GCTTTCTTCACCTGGCCTCCGTGCGAG	22	<nnxnp>	nn	150	np	122		
>ref-76734	ATCGGCTTAACTCCCTCTCCATCATCG	13	<nnxnp>	nn	110	np	135		
>ref-76753	GTAACCTTGACAGTAGCTCCTCAGTAT	21	<nnxnp>	nn	134	np	128		
>ref-76761	ACTAAGCACACAGCTCCTCCTACGCAC	7	<hkxhk>	hh	55	hk	144	kk	61
>ref-76785	CTCTCTCACACACACTCTCCAGCGATC	1	<nnxnp>	nn	134	np	135		
>ref-76785	CTCTCTCACACACACTCTCCAGCGATC	4	<nnxnp>	nn	129	np	138		
>ref-76785	CTCTCTCACACACACTCTCCAGCGATC	6	<nnxnp>	nn	129	np	138		
>ref-76804	GCCGGCCCAACCCACGCTCCTCCATCA	15	<hkxhk>	hh	47	hk	151	kk	62
>ref-76810	CCACCGTCGACCTGCGCTCCCATTGGT	3	<lmxll>	ll	146	lm	122		
>ref-76810	CCACCGTCGACCTGCGCTCCCATTGGT	6	<lmxll>	ll	146	lm	121		
>ref-76821	TCTCCTATTACCCCCCCTCCCCTCCCC	4	<hkxhk>	hh	12	hk	246	kk	2
>ref-76821	TCTCCTATTACCCCCCCTCCCCTCCCC	6	<hkxhk>	hh	2	hk	255	kk	9
>ref-76821	TCTCCTATTACCCCCCCTCCCCTCCCC	24	<lmxll>	ll	180	lm	61		
>ref-76829	GGCTATCCTACGTGGTCTCCTCGGATC	5	<lmxll>	ll	131	lm	136		
>ref-76831	ACTCCCTCTACTCCCCCTCCCCGTCCA	16	<nnxnp>	nn	131	np	125		
>ref-76836	TTCTCCGTCACTAAGCCTCCTTTTATT	8	<hkxhk>	hh	1	hk	262	kk	8
>ref-76836	TTCTCCGTCACTAAGCCTCCTTTTATT	9	<hkxhk>	hh	1	hk	271	kk	0
>ref-76836	TTCTCCGTCACTAAGCCTCCTTTTATT	13	<hkxhk>	hh	8	hk	262	kk	1
>ref-76858	ACCCTAGCAACTTTCTCTCCTTCCGAC	16	<lmxll>	ll	126	lm	138		
>ref-76881	ACCTCAACCACCCCCACTCCCACCTCC	2	<nnxnp>	nn	216	np	18		
>ref-76881	ACCTCAACCACCCCCACTCCCACCTCC	26	<nnxnp>	nn	134	np	125		
>ref-76886	TACGGGGTCACCAAACCTCCGTTAACT	5	<nnxnp>	nn	262	np	0		
>ref-76888	TCCCTCTACACCCTCCCTCCCTTGGTG	1	<hkxhk>	hh	64	hk	139	kk	51
>ref-76888	TCCCTCTACACCCTCCCTCCCTTGGTG	16	<lmxll>	ll	121	lm	142		
>ref-76912	TCGACTTCCACCCTCACTCCACTCTCT	4	<lmxll>	ll	103	lm	139		
>ref-76912	TCGACTTCCACCCTCACTCCACTCTCT	14	<lmxll>	ll	106	lm	140		
>ref-76918	TCTCCCCTTACCGTTCCTCCTCATCAT	15	<lmxll>	ll	3	lm	270		
>ref-76918	TCTCCCCTTACCGTTCCTCCTCATCAT	26	<lmxll>	ll	3	lm	270		

>ref-76920	CCCCTCTCCACTGACCCTCCCCCCTCT	14	<lmxll>	ll	162	lm	101		
>ref-76920	CCCCTCTCCACTGACCCTCCCCCCTCT	15	<lmxll>	ll	163	lm	101		
>ref-76920	CCCCTCTCCACTGACCCTCCCCCCTCT	23	<hkxhk>	hh	115	hk	135	kk	0
>ref-76920	CCCCTCTCCACTGACCCTCCCCCCTCT	27	<hkxhk>	hh	0	hk	137	kk	112
>ref-76947	ACACTTCCCACTCCCTCTCCCTTCTAC	9	<lmxll>	ll	109	lm	144		
>ref-76947	ACACTTCCCACTCCCTCTCCCTTCTAC	15	<lmxll>	ll	109	lm	144		
>ref-76954	CTGTCAGTAACTAACCCTCCTCCCCAT	2	<nnxnp>	nn	144	np	113		
>ref-76954	CTGTCAGTAACTAACCCTCCTCCCCAT	6	<nnxnp>	nn	144	np	113		
>ref-76962	CAGTTTAGAACGAAGCCTCCTCCGTTA	24	<lmxll>	ll	124	lm	136		
>ref-76972	TACCTAAACACCTATTCTCCTTGGGGA	21	<lmxll>	ll	152	lm	113		
>ref-76986	TATCAGTTTACCTCATCTCCGCCTTCG	1	<nnxnp>	nn	269	np	0		
>ref-76988	CTCCCCATTACTCTTTCTCCCTTTCTC	13	<lmxll>	ll	103	lm	116		
>ref-76994	CCACATCTTACTGGCTCTCCATCCCAC	21	<lmxll>	ll	194	lm	76		
>ref-76994	CCACATCTTACTGGCTCTCCATCCCAC	26	<lmxll>	ll	213	lm	36		
>ref-77009	CACCTGACCACCTCTACTCCCTCCCTA	9	<nnxnp>	nn	139	np	127		
>ref-77026	ACCTATGTCACTCATCCTCCCCCCCAC	23	<lmxll>	ll	128	lm	135		
>ref-77041	CCCTTCCCTACATCCCCTCCTCTTCCT	6	<nnxnp>	nn	147	np	118		
>ref-77048	CACACGCCCACCTGCCCTCCCGGATTA	6	<nnxnp>	nn	142	np	120		
>ref-77057	TCTCATCTCACCCAATCTCCTTCTCCC	27	<lmxll>	ll	122	lm	145		
>ref-77073	TGAAGGGCTACACGTCCTCCCCTCCGC	4	<lmxll>	ll	0	lm	272		
>ref-77073	TGAAGGGCTACACGTCCTCCCCTCCGC	12	<lmxll>	ll	2	lm	268		
>ref-77107	GGAAGTACCACCTACTCTCCCTACGGT	14	<nnxnp>	nn	114	np	146		
>ref-77121	TTTACTTCTACTCTTCCTCCCTATCTC	26	<lmxll>	ll	141	lm	104		
>ref-77168	CCTCTTCTAACGCCTCCTCCAATTCCT	1	<nnxnp>	nn	129	np	134		
>ref-77184	CCCCTTCTTACCTTTCCTCCTTCATTC	9	<nnxnp>	nn	123	np	139		
>ref-77235	GATTCAAGCACAGCACCTCCATCAGCT	23	<lmxll>	ll	138	lm	133		
>ref-77244	CCCCCAAAGACCACTCCTCCTCTTCCC	23	<nnxnp>	nn	263	np	0		
>ref-77247	GAACAGCAAACTCTTGCTCCTCCCAAC	2	<nnxnp>	nn	121	np	135		
>ref-77247	GAACAGCAAACTCTTGCTCCTCCCAAC	7	<hkxhk>	hh	62	hk	145	kk	47
>ref-77263	CATCCACCCACCCCCTCTCCTTTCCAC	26	<lmxll>	ll	229	lm	2		
>ref-77273	CATACTTCTACCTCTTCTCCTAATGGG	5	<hkxhk>	hh	71	hk	127	kk	43
>ref-77276	TCACCAACCACTCCCTCTCCCCTCCCT	1	<hkxhk>	hh	2	hk	271	kk	0
>ref-77276	TCACCAACCACTCCCTCTCCCCTCCCT	3	<hkxhk>	hh	81	hk	164	kk	0
>ref-77276	TCACCAACCACTCCCTCTCCCCTCCCT	14	<hkxhk>	hh	0	hk	271	kk	2
>ref-77276	TCACCAACCACTCCCTCTCCCCTCCCT	26	<hkxhk>	hh	83	hk	161	kk	0
>ref-77303	GCCTCTGAAACATCGTCTCCTGCAATG	14	<nnxnp>	nn	132	np	132		
>ref-77307	ATCAAGATTACACACTCTCCCTGGAAT	5	<lmxll>	ll	124	lm	110		
>ref-77307	ATCAAGATTACACACTCTCCCTGGAAT	24	<lmxll>	ll	95	lm	130		

>ref-77314	CAGGATGATACGCGGACTCCGACTGCT	12	<lmxll>	ll	125	lm	136		
>ref-77333	TCTGCCTGGACATCTTCTCCGTCAGCC	24	<nnxnp>	nn	140	np	133		
>ref-77358	AGAGACGGCACAGTAGCTCCCCCTGTG	2	<nnxnp>	nn	135	np	132		
>ref-77359	GATATGGAAACATCTACTCCACCACTG	25	<nnxnp>	nn	105	np	126		
>ref-77371	TCTCTCTCTACATTCCCTCCCCTCATA	12	<nnxnp>	nn	267	np	0		
>ref-77374	ATAATGATAACGTTTTCTCCTTTTTCT	8	<lmxll>	ll	98	lm	134		
>ref-77412	CAAGAAGACACTTTCTCTCCGTCTTAA	21	<nnxnp>	nn	120	np	118		
>ref-77421	GTCACTTGCACGTCGCCTCCTTCTCCT	7	<lmxll>	ll	124	lm	146		
>ref-77421	GTCACTTGCACGTCGCCTCCTTCTCCT	22	<lmxll>	ll	122	lm	147		
>ref-77431	TGTCATTCCACCCTGCCTCCCTCCCCC	23	<lmxll>	ll	116	lm	132		
>ref-77433	TTCCTCTCTACTGAAACTCCTTCCTCA	5	<lmxll>	ll	113	lm	106		
>ref-77433	TTCCTCTCTACTGAAACTCCTTCCTCA	8	<lmxll>	ll	113	lm	106		
>ref-77450	CATCCTCACACGCCCCCTCCACCTCCA	3	<hkxhk>	hh	47	hk	154	kk	68
>ref-77450	CATCCTCACACGCCCCCTCCACCTCCA	5	<hkxhk>	hh	68	hk	154	kk	47
>ref-77452	TTTGTCATCACTTCAACTCCTGACTAC	4	<hkxhk>	hh	0	hk	218	kk	41
>ref-77452	TTTGTCATCACTTCAACTCCTGACTAC	26	<hkxhk>	hh	0	hk	256	kk	0
>ref-77501	AAAAGGCAGACCATCTCTCCAGGCGGC	23	<hkxhk>	hh	63	hk	135	kk	52
>ref-77518	AATGCTGAGACATATCCTCCAGGTCTC	6	<lmxll>	ll	66	lm	196		
>ref-77518	AATGCTGAGACATATCCTCCAGGTCTC	12	<hkxhk>	hh	11	hk	258	kk	0
>ref-77518	AATGCTGAGACATATCCTCCAGGTCTC	25	<nnxnp>	nn	13	np	256		
>ref-77518	AATGCTGAGACATATCCTCCAGGTCTC	27	<hkxhk>	hh	256	hk	13	kk	0
>ref-77523	GACCCGATGACGCAATCTCCACCACAG	1	<lmxll>	ll	10	lm	259		
>ref-77550	AAGGCTCAAACAGCAGCTCCTTCAGAG	23	<lmxll>	ll	125	lm	146		
>ref-77557	TTTGTTTGGACTCTCGCTCCATCCACT	9	<lmxll>	ll	127	lm	136		
>ref-77565	TTCCTTTTGACTGATCCTCCGTCGATT	8	<hkxhk>	hh	0	hk	243	kk	12
>ref-77568	TCATCCGCTACCTCCTCTCCTCCTCCC	22	<nnxnp>	nn	244	np	1		
>ref-77568	TCATCCGCTACCTCCTCTCCTCCTCCC	25	<lmxll>	ll	243	lm	1		
>ref-77568	TCATCCGCTACCTCCTCTCCTCCTCCC	27	<nnxnp>	nn	218	np	15		
>ref-77575	TTATTTTTAACCAATTCTCCTTCATAA	6	<hkxhk>	hh	0	hk	161	kk	59
>ref-77575	TTATTTTTAACCAATTCTCCTTCATAA	24	<hkxhk>	hh	59	hk	161	kk	0
>ref-77577	CTCTCTCCGACCCCCTCTCCATCCTCC	3	<lmxll>	ll	145	lm	114		
>ref-77577	CTCTCTCCGACCCCCTCTCCATCCTCC	9	<nnxnp>	nn	32	np	203		
>ref-77577	CTCTCTCCGACCCCCTCTCCATCCTCC	21	<nnxnp>	nn	36	np	204		
>ref-77584	CAGGAGCCAACCTCGCCTCCAACAGGT	5	<nnxnp>	nn	127	np	142		
>ref-77598	CAATATTCGACTCGTTCTCCCGAGAGT	23	<hkxhk>	hh	47	hk	144	kk	57
>ref-77611	CTCGCCCCTACCTCCTCTCCCTCTCTC	5	<hkxhk>	hh	1	hk	272	kk	0
>ref-77611	CTCGCCCCTACCTCCTCTCCCTCTCTC	25	<hkxhk>	hh	1	hk	272	kk	0
>ref-77624	CAAAGGACCACGTGACCTCCACTTTTC	27	<lmxll>	ll	118	lm	102		

>ref-77628	TTCCTCATAACTATCGCTCCCTCATCA	7	<lmxll>	ll	146	lm	122		
>ref-77628	TTCCTCATAACTATCGCTCCCTCATCA	22	<lmxll>	ll	145	lm	122		
>ref-77646	CTCGTTCAAACAAATACTCCTCCCACA	26	<nnxnp>	nn	222	np	0		
>ref-77650	ATTGAGAAGACCTTCCCTCCACAGTGC	24	<hkxhk>	hh	0	hk	260	kk	0
>ref-77650	ATTGAGAAGACCTTCCCTCCACAGTGC	26	<nnxnp>	nn	245	np	14		
>ref-77650	ATTGAGAAGACCTTCCCTCCACAGTGC	27	<hkxhk>	hh	0	hk	260	kk	0
>ref-77676	TCTACTTGTACCTTTCCTCCTTCTCTT	15	<lmxll>	ll	135	lm	130		
>ref-77677	TGCATCCCAACCAGATCTCCACATATC	9	<nnxnp>	nn	138	np	113		
>ref-77688	TATCTCCCTACATTCTCTCCCACTTCC	2	<lmxll>	ll	6	lm	237		
>ref-77688	TATCTCCCTACATTCTCTCCCACTTCC	15	<lmxll>	ll	6	lm	236		
>ref-77710	TGCAGATACACACCAGCTCCCACACAC	25	<nnxnp>	nn	143	np	127		
>ref-77723	CTGCTGCGAACGGCGCCTCCCCCCCAC	24	<nnxnp>	nn	128	np	127		
>ref-77723	CTGCTGCGAACGGCGCCTCCCCCCCAC	26	<nnxnp>	nn	127	np	128		
>ref-77744	TCATTTATGACTCTCCCTCCGTCTGTC	14	<nnxnp>	nn	130	np	122		
>ref-77753	CGCATTCGGACGTCAGCTCCTTACCCA	22	<lmxll>	ll	125	lm	124		
>ref-77770	ATGCTAAAGACTGAAGCTCCATTTTCA	6	<lmxll>	ll	128	lm	128		
>ref-77783	GCCGCAGTTACAAGATCTCCCTCGTAC	2	<lmxll>	ll	1	lm	269		
>ref-77783	GCCGCAGTTACAAGATCTCCCTCGTAC	5	<lmxll>	ll	1	lm	269		
>ref-77801	TATTTTTCTACCTTTCCTCCTCTTTCT	6	<lmxll>	ll	132	lm	126		
>ref-77801	TATTTTTCTACCTTTCCTCCTCTTTCT	24	<nnxnp>	nn	9	np	244		
>ref-77809	CCTCATTAGACTTTTCCTCCCTTGCTG	23	<nnxnp>	nn	131	np	116		
>ref-77813	TTCACCCTTACCCCCACTCCCTCTCTT	3	<nnxnp>	nn	261	np	0		
>ref-77813	TTCACCCTTACCCCCACTCCCTCTCTT	5	<nnxnp>	nn	261	np	0		
>ref-77817	CCTAGCGCTACCTCATCTCCCCCTGAT	12	<lmxll>	ll	132	lm	136		
>ref-77837	CCCCTCCTCACTCTCTCTCCGTTCCAC	7	<hkxhk>	hh	103	hk	131	kk	0
>ref-77853	CTTCCTCAAACTCTTCCTCCCTAATTA	25	<nnxnp>	nn	117	np	137		
>ref-77861	CTGCCCACTACCTTGTCTCCTTCGCCG	9	<lmxll>	ll	149	lm	123		
>ref-77864	TTAATTCCTACAGAACCTCCAATAGCA	23	<nnxnp>	nn	132	np	124		
>ref-77926	TTTTCCACCACCCTCCCTCCCCTCCCT	7	<lmxll>	ll	232	lm	2		
>ref-77926	TTTTCCACCACCCTCCCTCCCCTCCCT	22	<lmxll>	ll	253	lm	0		
>ref-77937	CTCTCGTTTACCTCCTCTCCTTTCTAC	9	<nnxnp>	nn	120	np	140		
>ref-77940	TTTGCTTCAACCCTCCCTCCTCCGCCT	14	<nnxnp>	nn	128	np	142		
>ref-77942	ACACACACAACCTTTCCTCCTTGCAGA	3	<nnxnp>	nn	264	np	0		
>ref-77943	TCCTATTTTACTAATCCTCCCTTGTTC	7	<nnxnp>	nn	127	np	135		
>ref-77963	TTCCCTGCCACCCTTCCTCCTTTCACA	24	<nnxnp>	nn	144	np	115		
>ref-77969	CTACCCTATACTCTCTCTCCCTCCGTC	23	<nnxnp>	nn	257	np	0		
>ref-77981	TTCCCCTTTACTTCTTCTCCTCGCTAC	7	<lmxll>	ll	101	lm	121		
>ref-77981	TTCCCCTTTACTTCTTCTCCTCGCTAC	8	<lmxll>	ll	104	lm	119		

>ref-78015	ACAGTAACAACAGTAACTCCAACTCCA	3	<hkxhk>	hh	221	hk	7	kk	0
>ref-78015	ACAGTAACAACAGTAACTCCAACTCCA	13	<lmxll>	ll	151	lm	72		
>ref-78031	TGTTGGTCAACGTGTCCTCCGATTCAA	23	<hkxhk>	hh	0	hk	269	kk	2
>ref-78036	ACCGACTACACCACTCCTCCTCCTTCT	1	<lmxll>	ll	219	lm	5		
>ref-78039	TTCGGTCTCACTCGTTCTCCTTCTCTG	23	<nnxnp>	nn	138	np	134		
>ref-78040	CGCCTGGATACCCCCCCTCCTTCCCCC	9	<lmxll>	ll	220	lm	0		
>ref-78042	GTCGTTAGAACCTCCTCTCCCGCAATT	22	<lmxll>	ll	111	lm	156		
>ref-78052	CCTAATGCCACCTTATCTCCCCCCTCC	15	<lmxll>	ll	92	lm	159		
>ref-78056	TAGATACATACAGCTACTCCACAACAA	14	<lmxll>	ll	118	lm	134		
>ref-78082	CTCGCAAGGACGGCGCCTCCACTTCGT	13	<lmxll>	ll	128	lm	136		
>ref-78083	TGTCCCCCAACTCCTTCTCCTTGTCCC	2	<nnxnp>	nn	244	np	0		
>ref-78083	TGTCCCCCAACTCCTTCTCCTTGTCCC	23	<nnxnp>	nn	238	np	1		
>ref-78087	AGGATAAAAACTGCTCCTCCAAGACTC	21	<nnxnp>	nn	131	np	118		
>ref-78087	AGGATAAAAACTGCTCCTCCAAGACTC	23	<nnxnp>	nn	130	np	118		
>ref-78094	GAGAAAAATACACAATCTCCGCAACCA	26	<nnxnp>	nn	261	np	0		
>ref-78094	GAGAAAAATACACAATCTCCGCAACCA	27	<nnxnp>	nn	260	np	0		
>ref-78105	ATCCCCAACACAGCCTCTCCTCCACAG	5	<hkxhk>	hh	73	hk	181	kk	0
>ref-78105	ATCCCCAACACAGCCTCTCCTCCACAG	15	<hkxhk>	hh	1	hk	272	kk	0
>ref-78108	GCCTCGGGCACTTTTTCTCCTCTAATA	26	<nnxnp>	nn	269	np	0		
>ref-78108	GCCTCGGGCACTTTTTCTCCTCTAATA	27	<nnxnp>	nn	268	np	0		
>ref-78135	TCCCTTCCAACCTCTCCTCCCCTCACC	22	<lmxll>	ll	147	lm	103		
>ref-78137	TCAGCAATCACTGGCCCTCCAGCAGTC	13	<hkxhk>	hh	0	hk	149	kk	122
>ref-78145	GACTTCCAAACGCGGTCTCCACATCAA	9	<hkxhk>	hh	49	hk	142	kk	68
>ref-78148	TTAGTATGTACTGAATCTCCTTCACGC	2	<lmxll>	ll	114	lm	137		
>ref-78148	TTAGTATGTACTGAATCTCCTTCACGC	26	<nnxnp>	nn	132	np	129		
>ref-78150	GGTAATGGTACTCTTGCTCCTAATGCA	21	<hkxhk>	hh	0	hk	244	kk	4
>ref-78150	GGTAATGGTACTCTTGCTCCTAATGCA	26	<nnxnp>	nn	38	np	192		
>ref-78176	CTTTTACTCACTGTCACTCCTTCGTAT	7	<nnxnp>	nn	120	np	131		
>ref-78185	ATGACAATCACTCACTCTCCCTACCTT	4	<lmxll>	ll	110	lm	155		
>ref-78191	TGCTCACCCACAGTCTCTCCATATATC	7	<nnxnp>	nn	117	np	129		
>ref-78210	CCCCTCTTCACATCCCCTCCGTCTCCC	15	<nnxnp>	nn	267	np	0		
>ref-78216	CGTGGACTGACATTATCTCCTGAGCTG	2	<lmxll>	ll	122	lm	140		
>ref-78236	ACAAGAGCGACAAGCTCTCCCACACCC	4	<hkxhk>	hh	48	hk	151	kk	69
>ref-78245	TCAGCCCCTACTCTCTCTCCCTCCTCT	7	<lmxll>	ll	7	lm	265		
>ref-78245	TCAGCCCCTACTCTCTCTCCCTCCTCT	23	<lmxll>	ll	7	lm	265		
>ref-78247	CTTTACCCAACCTTCCCTCCCTTCCTT	23	<nnxnp>	nn	132	np	126		
>ref-78282	TACATACACACACACACTCCGTCTTCA	3	<hkxhk>	hh	54	hk	199	kk	0
>ref-78282	TACATACACACACACACTCCGTCTTCA	7	<nnxnp>	nn	131	np	108		

>ref-78292	AACCCACTCACCCGCCCTCCTCTCCCC	12	<nnxnp>	nn	108	np	141		
>ref-78292	AACCCACTCACCCGCCCTCCTCTCCCC	14	<lmxll>	ll	129	lm	129		
>ref-78296	CGCACGCGCACACACGCTCCCATCCAC	27	<lmxll>	ll	117	lm	153		
>ref-78315	TCCTCTTTAACCTCCCCTCCTCCTTCG	23	<lmxll>	ll	109	lm	143		
>ref-78315	TCCTCTTTAACCTCCCCTCCTCCTTCG	27	<lmxll>	ll	102	lm	142		
>ref-78331	TATTTCTCCACTTCCACTCCTTTTCTC	3	<lmxll>	ll	118	lm	149		
>ref-78344	TAAAAAGAAACTGCTGCTCCGTTAGTC	7	<nnxnp>	nn	120	np	123		
>ref-78372	AAGGACCAGACCCCCCCTCCCTCACTG	7	<lmxll>	ll	151	lm	101		
>ref-78372	AAGGACCAGACCCCCCCTCCCTCACTG	9	<lmxll>	ll	151	lm	101		
>ref-78379	CCTCCAACCACTTCCACTCCTCTTCCT	24	<lmxll>	ll	127	lm	133		
>ref-78401	CCCACCACCACCACCCCTCCTTCCCCT	1	<hkxhk>	hh	1	hk	203	kk	52
>ref-78401	CCCACCACCACCACCCCTCCTTCCCCT	5	<hkxhk>	hh	53	hk	203	kk	1
>ref-78427	CTACCATTGACTCCTTCTCCTCCTCCA	27	<hkxhk>	hh	1	hk	271	kk	0
>ref-78447	TAAATCAAGACTTCCTCTCCAGTCTGG	9	<lmxll>	ll	164	lm	77		
>ref-78447	TAAATCAAGACTTCCTCTCCAGTCTGG	27	<hkxhk>	hh	1	hk	247	kk	9
>ref-78448	TCATTTCTTACAGTGGCTCCTTTCCTC	2	<lmxll>	ll	114	lm	116		
>ref-78448	TCATTTCTTACAGTGGCTCCTTTCCTC	16	<nnxnp>	nn	242	np	0		
>ref-78463	ACTTCTTTTACTTCTCCTCCGCCTTGT	15	<nnxnp>	nn	127	np	133		
>ref-78472	CCTGAGGTCACTGTCCCTCCTTCCTCT	4	<lmxll>	ll	102	lm	151		
>ref-78507	TGATAGACAACATTACCTCCTGATCAC	1	<nnxnp>	nn	134	np	133		
>ref-78540	CCCCCTTCTACTCCACCTCCTTTGCCT	27	<lmxll>	ll	259	lm	2		
>ref-78558	ACATCGTTAACCCCAGCTCCTTCTACG	14	<nnxnp>	nn	121	np	140		
>ref-78560	TTTGCTGCCACTCGTCCTCCCTCATAT	6	<lmxll>	ll	124	lm	137		
>ref-78571	TGATCACAAACACAAACTCCCTTCACC	12	<lmxll>	ll	120	lm	109		
>ref-78571	TGATCACAAACACAAACTCCCTTCACC	27	<lmxll>	ll	118	lm	109		
>ref-78591	TGCGACTCAACTGTTGCTCCCGCATGC	7	<nnxnp>	nn	243	np	24		
>ref-78592	TCAAAGAATACTCAGTCTCCTGCTCTG	14	<nnxnp>	nn	105	np	116		
>ref-78615	CCTCCCTGAACTGGCCCTCCGGTCTCA	9	<lmxll>	ll	151	lm	107		
>ref-78625	TCTATCCCTACCCTTACTCCCTTCTTT	12	<hkxhk>	hh	49	hk	134	kk	52
>ref-78625	TCTATCCCTACCCTTACTCCCTTCTTT	25	<lmxll>	ll	120	lm	119		
>ref-78630	CTTTTCTTCACTCTCACTCCTTTCCCC	5	<lmxll>	ll	117	lm	130		
>ref-78631	ATGCATTCCACTGGTTCTCCTAGTCCC	16	<hkxhk>	hh	0	hk	257	kk	13
>ref-78634	CCGGTGTTTACATGAACTCCGGTGCTC	25	<lmxll>	ll	130	lm	137		
>ref-78649	CGCGGTCCGACAGAATCTCCATCGATA	8	<nnxnp>	nn	122	np	140		
>ref-78652	AGAGAACAAACTGACTCTCCGAATTAC	2	<lmxll>	ll	148	lm	73		
>ref-78652	AGAGAACAAACTGACTCTCCGAATTAC	27	<hkxhk>	hh	1	hk	126	kk	94
>ref-78660	CTTCCTCATACCCTCCCTCCCTCATAC	1	<nnxnp>	nn	272	np	0		
>ref-78673	CCTTCTCACACTCCCCCTCCCTCTGGA	3	<lmxll>	ll	116	lm	140		

>ref-78673	CCTTCTCACACTCCCCCTCCCTCTGGA	22	<lmxll>	ll	118	lm	140		
>ref-78685	CTCCCTTCCACGCCCTCTCCCCTCCCC	15	<nnxnp>	nn	265	np	0		
>ref-78712	CTATCCCCTACTTTTTCTCCCTCTCCT	1	<nnxnp>	nn	251	np	1		
>ref-78777	GTCACTTTAACCTCCTCTCCATTTACT	23	<hkxhk>	hh	55	hk	132	kk	64
>ref-78796	TTACAACAAACCCCTCCTCCCCCTCCT	5	<lmxll>	ll	232	lm	0		
>ref-78845	ACTTTGTTCACCGTGTCTCCAGGTGTT	16	<lmxll>	ll	121	lm	147		
>ref-78852	TTATTCCACACTGCCACTCCAAGAAAG	15	<hkxhk>	hh	2	hk	262	kk	1
>ref-78852	TTATTCCACACTGCCACTCCAAGAAAG	24	<hkxhk>	hh	1	hk	261	kk	2
>ref-78870	ACAGCACGAACTCCATCTCCAGCAGGT	4	<lmxll>	ll	250	lm	9		
>ref-78881	GACGAGAGGACCCGGGCTCCAGTGTAG	8	<nnxnp>	nn	21	np	240		
>ref-78881	GACGAGAGGACCCGGGCTCCAGTGTAG	23	<nnxnp>	nn	20	np	241		
>ref-78889	TGCGCTGAAACCCGCTCTCCTCCGCCT	7	<lmxll>	ll	140	lm	130		
>ref-78905	CCTCATCCTACTTATTCTCCTGACCTT	7	<lmxll>	ll	25	lm	235		
>ref-78905	CCTCATCCTACTTATTCTCCTGACCTT	27	<nnxnp>	nn	141	np	107		
>ref-78906	CTCCTTTCTACCCTCCCTCCCTGTCTT	7	<lmxll>	ll	134	lm	105		
>ref-78906	CTCCTTTCTACCCTCCCTCCCTGTCTT	23	<hkxhk>	hh	0	hk	222	kk	0
>ref-78911	TTCTCCCTCACCTCCCCTCCCCCTTCT	2	<hkxhk>	hh	0	hk	268	kk	3
>ref-78911	TTCTCCCTCACCTCCCCTCCCCCTTCT	6	<lmxll>	ll	193	lm	62		
>ref-78911	TTCTCCCTCACCTCCCCTCCCCCTTCT	9	<lmxll>	ll	148	lm	106		
>ref-78911	TTCTCCCTCACCTCCCCTCCCCCTTCT	16	<hkxhk>	hh	2	hk	268	kk	0
>ref-78935	CATCACTGAACTCCTCCTCCATCACGT	25	<nnxnp>	nn	128	np	142		
>ref-78935	CATCACTGAACTCCTCCTCCATCACGT	26	<nnxnp>	nn	159	np	96		
>ref-78938	GGAGCCGCGACTGCTACTCCTTGCGCT	24	<nnxnp>	nn	142	np	127		
>ref-78947	TAACGACAGACCTTTCCTCCTCCTTCC	25	<lmxll>	ll	120	lm	150		
>ref-78950	CTCCATGTCACCCTATCTCCATGTCAC	3	<hkxhk>	hh	58	hk	129	kk	60
>ref-78955	AGCTTTCTGACACATTCTCCTTGGTAA	3	<nnxnp>	nn	128	np	109		
>ref-78960	ATCAACATTACTTACCCTCCTCGTCTT	15	<hkxhk>	hh	66	hk	129	kk	59
>ref-78972	AACCCTTCCACACTAACTCCTCTCCAT	21	<nnxnp>	nn	135	np	105		
>ref-78976	ACCTCCCCTACTCCCCCTCCCCGCTAT	16	<nnxnp>	nn	265	np	0		
>ref-78979	TCCACCTCCACCTTGCCTCCTTCTCCG	27	<lmxll>	ll	148	lm	117		
>ref-78983	AATAAAAAAACACGATCTCCCTCAAGC	1	<nnxnp>	nn	223	np	0		
>ref-79053	GTTTCTCTCACCATATCTCCCCTCCTC	12	<nnxnp>	nn	243	np	0		
>ref-79056	CTCTCTCTCACCCTATCTCCCCCTTAC	3	<lmxll>	ll	164	lm	92		
>ref-79056	CTCTCTCTCACCCTATCTCCCCCTTAC	21	<hkxhk>	hh	0	hk	260	kk	5
>ref-79059	CACTCATGTACGCATACTCCACTAATA	4	<nnxnp>	nn	139	np	124		
>ref-79061	GTGTGCGTAACACATGCTCCAGCAAGG	5	<nnxnp>	nn	263	np	0		
>ref-79070	GGCGTCCAAACCCTCTCTCCGCCATCA	14	<nnxnp>	nn	127	np	142		
>ref-79074	TATTTTCCGACAAGTTCTCCCGAATAT	2	<hkxhk>	hh	3	hk	260	kk	0

>ref-79074	TATTTTCCGACAAGTTCTCCCGAATAT	8	<hkxhk>	hh	0	hk	260	kk	3
>ref-79097	TAAAGTTATACCCATTCTCCCTTAACT	8	<hkxhk>	hh	2	hk	246	kk	2
>ref-79106	TCTGAATTTACTGTACCTCCCTTCTTC	13	<hkxhk>	hh	2	hk	192	kk	27
>ref-79106	TCTGAATTTACTGTACCTCCCTTCTTC	16	<hkxhk>	hh	27	hk	192	kk	2
>ref-79113	CAAACACACACAGACACTCCTGATACT	1	<lmxll>	ll	146	lm	125		
>ref-79113	CAAACACACACAGACACTCCTGATACT	3	<lmxll>	ll	146	lm	125		
>ref-79116	TCCACTCCTACCACCCCTCCCTCTCCT	22	<nnxnp>	nn	127	np	134		
>ref-79123	GACAGGCACACCAACTCTCCCACAGTC	6	<nnxnp>	nn	138	np	131		
>ref-79123	GACAGGCACACCAACTCTCCCACAGTC	8	<nnxnp>	nn	139	np	131		
>ref-79133	TCGTTGTGGACCCTCCCTCCCTCTCTC	6	<nnxnp>	nn	251	np	1		
>ref-79133	TCGTTGTGGACCCTCCCTCCCTCTCTC	21	<lmxll>	ll	249	lm	0		
>ref-79133	TCGTTGTGGACCCTCCCTCCCTCTCTC	23	<nnxnp>	nn	248	np	0		
>ref-79137	ACACACAACACGCCACCTCCACACCAC	4	<nnxnp>	nn	130	np	135		
>ref-79137	ACACACAACACGCCACCTCCACACCAC	6	<lmxll>	ll	139	lm	130		
>ref-79137	ACACACAACACGCCACCTCCACACCAC	8	<hkxhk>	hh	52	hk	154	kk	59
>ref-79142	GCGACATTAACACGGCCTCCCGCTGTC	21	<lmxll>	ll	145	lm	124		
>ref-79142	GCGACATTAACACGGCCTCCCGCTGTC	26	<hkxhk>	hh	83	hk	131	kk	49
>ref-79165	TCCAAATCCACTTTCTCTCCTTCTCTT	2	<lmxll>	ll	119	lm	129		
>ref-79168	CTGCTCCTCACCCTGTCTCCCATCTTC	26	<nnxnp>	nn	270	np	0		
>ref-79186	TGCCCTCTCACCCTTACTCCCTGCTCC	3	<nnxnp>	nn	265	np	0		
>ref-79193	ATGGTGGCCACGAGCTCTCCGCAAATG	25	<nnxnp>	nn	128	np	127		
>ref-79221	ACTCTTTCCACGCCCACTCCCGAGGCA	1	<nnxnp>	nn	114	np	155		
>ref-79244	AGGCCGCAAACCGCGCCTCCGACTCCA	5	<hkxhk>	hh	66	hk	131	kk	65
>ref-79271	CACACACGTACGCACACTCCTACGCGG	9	<nnxnp>	nn	268	np	0		
>ref-79290	TTCCTTCTCACCACACCTCCCTCATTC	6	<lmxll>	ll	116	lm	149		
>ref-79290	TTCCTTCTCACCACACCTCCCTCATTC	24	<lmxll>	ll	116	lm	149		
>ref-79328	CCCACCTGGACGCTCTCTCCACCTCAG	12	<lmxll>	ll	133	lm	133		
>ref-79329	TGTATGACCACGTGTCCTCCCCTCAGG	24	<hkxhk>	hh	21	hk	250	kk	0
>ref-79329	TGTATGACCACGTGTCCTCCCCTCAGG	26	<hkxhk>	hh	4	hk	269	kk	0
>ref-79330	TCTCTCGCCACTTCCGCTCCACATCAC	16	<hkxhk>	hh	4	hk	264	kk	0
>ref-79330	TCTCTCGCCACTTCCGCTCCACATCAC	23	<hkxhk>	hh	4	hk	264	kk	0
>ref-79348	TCCTTTATCACGTCCTCTCCCCTTCTT	3	<nnxnp>	nn	130	np	139		
>ref-79351	AGATCATCTACGAGTCCTCCAGTAGAT	12	<lmxll>	ll	134	lm	136		
>ref-79357	TTCCCTTCAACAACACCTCCCATTAAC	21	<hkxhk>	hh	100	hk	159	kk	0
>ref-79392	CCCTTCTCCACCATCTCTCCTTCCTCT	1	<lmxll>	ll	8	lm	265		
>ref-79392	CCCTTCTCCACCATCTCTCCTTCCTCT	7	<lmxll>	ll	8	lm	265		
>ref-79395	CCACCACCCACCTCCCCTCCCCTCACT	5	<lmxll>	ll	123	lm	111		
>ref-79409	TCTCAAGGTACACACGCTCCTCCTTAC	14	<hkxhk>	hh	1	hk	272	kk	0

>ref-79409	TCTCAAGGTACACACGCTCCTCCTTAC	21	<lmxll>	ll	147	lm	126		
>ref-79410	TGCTTGCCCACCTAGCCTCCTACCTTC	15	<hkxhk>	hh	55	hk	150	kk	65
>ref-79440	ACAACAACAACAAACACTCCACCACAA	25	<hkxhk>	hh	0	hk	114	kk	143
>ref-79497	GGTTTTCCTACCTCTCCTCCGCTCCAC	25	<lmxll>	ll	126	lm	129		
>ref-79497	GGTTTTCCTACCTCTCCTCCGCTCCAC	26	<lmxll>	ll	126	lm	129		
>ref-79498	TTCCCTTCAACTGCTACTCCTACCTCT	16	<lmxll>	ll	259	lm	0		
>ref-79515	ATTCTGCTGACATCCACTCCCGCACAC	26	<lmxll>	ll	123	lm	143		
>ref-79542	ACTACCTCCACTTCCTCTCCCTCCTAC	8	<nnxnp>	nn	123	np	132		
>ref-79543	TTCTCACTTACTCACTCTCCCTCAATT	1	<hkxhk>	hh	50	hk	139	kk	68
>ref-79568	GCATTCCTAACCCCTCCTCCCTTCCAC	26	<nnxnp>	nn	133	np	128		
>ref-79575	ACGGCGCTCACTCTGCCTCCCGCCGGA	7	<nnxnp>	nn	99	np	138		
>ref-79580	TGCATGCACACCCCCACTCCCACTCCC	2	<lmxll>	ll	186	lm	33		
>ref-79595	CCACCCCACACCTCCACTCCACTCGTC	9	<lmxll>	ll	111	lm	125		
>ref-79595	CCACCCCACACCTCCACTCCACTCGTC	14	<lmxll>	ll	135	lm	121		
>ref-79595	CCACCCCACACCTCCACTCCACTCGTC	21	<hkxhk>	hh	9	hk	258	kk	0
>ref-79595	CCACCCCACACCTCCACTCCACTCGTC	27	<hkxhk>	hh	5	hk	265	kk	0
>ref-79602	AACCTTTTAACTACCACTCCCCCCTTT	13	<hkxhk>	hh	1	hk	239	kk	15
>ref-79602	AACCTTTTAACTACCACTCCCCCCTTT	14	<hkxhk>	hh	1	hk	239	kk	15
>ref-79608	CATACCTTCACCATAACTCCCACCACA	6	<lmxll>	ll	156	lm	106		
>ref-79610	CGGATCCTAACCTCCACTCCTCCTTAT	2	<lmxll>	ll	86	lm	161		
>ref-79631	GAGAGAGAGACTCCCTCTCCCCTTAGT	3	<lmxll>	ll	145	lm	119		
>ref-79662	CCCTTTCTCACCCCCTCTCCATCTATC	22	<hkxhk>	hh	29	hk	162	kk	57
>ref-79664	CCCCCACTCACAACCCCTCCCCTTCCT	5	<hkxhk>	hh	1	hk	265	kk	1
>ref-79664	CCCCCACTCACAACCCCTCCCCTTCCT	6	<hkxhk>	hh	1	hk	265	kk	1
>ref-79672	TAAGCAAACACATCTCCTCCCACCTCC	23	<lmxll>	ll	129	lm	139		
>ref-79712	TCTCCAACAACTCTCTCTCCTCCTCTT	2	<nnxnp>	nn	135	np	128		
>ref-79712	TCTCCAACAACTCTCTCTCCTCCTCTT	15	<nnxnp>	nn	133	np	128		
>ref-79718	CACCAAACGACACCCCCTCCCCCCTCC	16	<hkxhk>	hh	0	hk	263	kk	3
>ref-79722	TCTTCCCTTACCTCTCCTCCCTACCCA	3	<hkxhk>	hh	0	hk	182	kk	79
>ref-79722	TCTTCCCTTACCTCTCCTCCCTACCCA	5	<hkxhk>	hh	80	hk	182	kk	0
>ref-79740	GAAGACCCCACAAATCCTCCCTGCCCC	9	<nnxnp>	nn	128	np	144		
>ref-79751	CCAGCAGGAACCCCGTCTCCTTATGGA	23	<nnxnp>	nn	141	np	124		
>ref-79764	CCTGCGGCTACCTCTCCTCCAAAATTC	25	<lmxll>	ll	130	lm	133		
>ref-79781	CAATAATTTACGTTTTCTCCTCGTTGT	3	<hkxhk>	hh	1	hk	260	kk	0
>ref-79781	CAATAATTTACGTTTTCTCCTCGTTGT	8	<nnxnp>	nn	144	np	115		
>ref-79801	TCCTCCCCAACTTCCCCTCCTCAGAGT	12	<hkxhk>	hh	0	hk	260	kk	8
>ref-79801	TCCTCCCCAACTTCCCCTCCTCAGAGT	22	<nnxnp>	nn	184	np	75		
>ref-79801	TCCTCCCCAACTTCCCCTCCTCAGAGT	24	<hkxhk>	hh	0	hk	273	kk	0

>ref-79836	CAACCCCAGACCCTTCCTCCTACTCCT	6	<lmxll>	ll	128	lm	141		
>ref-79866	TTGGCAGTCACCATGTCTCCATGATGT	7	<hkxhk>	hh	0	hk	236	kk	28
>ref-79866	TTGGCAGTCACCATGTCTCCATGATGT	23	<hkxhk>	hh	0	hk	146	kk	102
>ref-79866	TTGGCAGTCACCATGTCTCCATGATGT	27	<hkxhk>	hh	0	hk	109	kk	146
>ref-79867	CCTTATCCCACTGAGCCTCCTCCCACA	21	<lmxll>	ll	143	lm	116		
>ref-79881	ATCCTTTTCACTCAGTCTCCTCTCTCT	15	<lmxll>	ll	127	lm	95		
>ref-79885	CCCTCTCCCACTCCCTCTCCCTAGCAC	23	<hkxhk>	hh	0	hk	273	kk	0
>ref-79885	CCCTCTCCCACTCCCTCTCCCTAGCAC	24	<nnxnp>	nn	22	np	200		
>ref-79889	CCCCCTCTTACCCATTCTCCTTCCCCC	1	<nnxnp>	nn	262	np	0		
>ref-79894	AATGTTCTCACGCGAACTCCGATGAAG	24	<lmxll>	ll	6	lm	264		
>ref-79899	CCTCCTCGGACATCTCCTCCTCGGACA	25	<hkxhk>	hh	107	hk	148	kk	0
>ref-79899	CCTCCTCGGACATCTCCTCCTCGGACA	26	<nnxnp>	nn	262	np	10		
>ref-79907	TTCGTTAAAACTGCATCTCCATGCTAG	9	<lmxll>	ll	257	lm	0		
>ref-79936	CCACATCCAACACCCTCTCCCCTTCCT	21	<hkxhk>	hh	3	hk	267	kk	0
>ref-79948	AGCCAGACAACGCAGCCTCCACGATCG	24	<nnxnp>	nn	130	np	137		
>ref-79961	TCTCCTCCGACCTCCTCTCCTCAATTT	7	<hkxhk>	hh	57	hk	149	kk	57
>ref-79961	TCTCCTCCGACCTCCTCTCCTCAATTT	26	<hkxhk>	hh	57	hk	149	kk	57
>ref-79969	CACTAATGCACACTCACTCCTACTCTT	5	<hkxhk>	hh	32	hk	207	kk	0
>ref-79970	CTCTCCCCTACTCCCTCTCCCTATATC	9	<hkxhk>	hh	0	hk	273	kk	0
>ref-79970	CTCTCCCCTACTCCCTCTCCCTATATC	12	<lmxll>	ll	163	lm	94		
>ref-79970	CTCTCCCCTACTCCCTCTCCCTATATC	14	<nnxnp>	nn	167	np	91		
>ref-79970	CTCTCCCCTACTCCCTCTCCCTATATC	23	<hkxhk>	hh	0	hk	250	kk	15
>ref-79970	CTCTCCCCTACTCCCTCTCCCTATATC	25	<hkxhk>	hh	5	hk	259	kk	0
>ref-79986	CATATTTGCACACACCCTCCCTCTGCC	8	<hkxhk>	hh	48	hk	143	kk	56
>ref-80000	ACGCACAAGACTTTCCCTCCATCGCGT	21	<hkxhk>	hh	64	hk	135	kk	53
>ref-80023	AAGTCGATAACTCTATCTCCTTCATTT	4	<lmxll>	ll	132	lm	114		
>ref-80023	AAGTCGATAACTCTATCTCCTTCATTT	5	<lmxll>	ll	132	lm	114		
>ref-80023	AAGTCGATAACTCTATCTCCTTCATTT	25	<lmxll>	ll	130	lm	123		
>ref-80040	TTGTGGAGGACGACGACTCCATCCTTG	3	<hkxhk>	hh	0	hk	268	kk	4
>ref-80041	GCTCGTGGCACCACCTCTCCCAGCATT	2	<nnxnp>	nn	243	np	26		
>ref-80041	GCTCGTGGCACCACCTCTCCCAGCATT	5	<lmxll>	ll	16	lm	252		
>ref-80041	GCTCGTGGCACCACCTCTCCCAGCATT	13	<hkxhk>	hh	2	hk	269	kk	0
>ref-80041	GCTCGTGGCACCACCTCTCCCAGCATT	21	<hkxhk>	hh	10	hk	253	kk	0
>ref-80041	GCTCGTGGCACCACCTCTCCCAGCATT	23	<hkxhk>	hh	0	hk	253	kk	11
>ref-80046	TGCCATAGAACGAAAGCTCCCCTTAAT	1	<hkxhk>	hh	54	hk	137	kk	56
>ref-80085	CAGGAAAGAACTGGAGCTCCTCCACAT	16	<hkxhk>	hh	42	hk	153	kk	69
>ref-80099	ACCATTACCACACAGACTCCTATTGCA	22	<lmxll>	ll	123	lm	142		
>ref-80104	CTTTTACCGACTTTGCCTCCCTCTTTC	16	<nnxnp>	nn	246	np	1		

>ref-80133	CCTCTTCCAACGCCCTCTCCTTTCCAA	6	<hkxhk>	hh	70	hk	127	kk	59
>ref-80175	ACACGGTGCACGTGGTCTCCCCTGAGA	5	<lmxll>	ll	163	lm	110		
>ref-80189	ATCCTATCCACTCGTTCTCCTCCCCAC	9	<lmxll>	ll	138	lm	127		
>ref-80195	CATACTTTCACAGCCCCTCCTTGTCAC	5	<hkxhk>	hh	55	hk	134	kk	75
>ref-80219	CGCGTATTTACTCTCTCTCCGTTTCTC	2	<nnxnp>	nn	133	np	131		
>ref-80219	CGCGTATTTACTCTCTCTCCGTTTCTC	16	<nnxnp>	nn	132	np	133		
>ref-80233	CCACTCAAAACCCCAGCTCCTTGTAGT	4	<nnxnp>	nn	124	np	140		
>ref-80234	AAACGCAACACAGACGCTCCCCCTCTC	8	<nnxnp>	nn	102	np	166		
>ref-80251	ACCACAATTACGTGCGCTCCTGCCCTT	22	<lmxll>	ll	143	lm	124		
>ref-80256	CGTGTCGGTACCATTTCTCCACGACAT	21	<nnxnp>	nn	5	np	264		
>ref-80257	TGACTACGGACGAAGGCTCCCGTCTAT	26	<nnxnp>	nn	114	np	149		
>ref-80273	TTCAACCGAACAGACACTCCCTGTAAG	5	<lmxll>	ll	119	lm	126		
>ref-80274	ATGCTGAGAACATTTACTCCCGCTTCT	12	<nnxnp>	nn	136	np	123		
>ref-80289	TCTAACTTAACCCACCCTCCCTACAAC	27	<nnxnp>	nn	249	np	0		
>ref-80292	GGCGGAGGGACGGGGTCTCCTGCTTCT	9	<nnxnp>	nn	270	np	0		
>ref-80312	GCTCCGCCCACCCGAACTCCACCCCTT	6	<hkxhk>	hh	70	hk	115	kk	63
>ref-80319	CTCAGCTGAACTCTCTCTCCCTTCCAT	2	<hkxhk>	hh	49	hk	161	kk	55
>ref-80319	CTCAGCTGAACTCTCTCTCCCTTCCAT	7	<hkxhk>	hh	45	hk	164	kk	57
>ref-80328	ACTTTTCCGACCACGTCTCCTTTCCTA	15	<lmxll>	ll	132	lm	115		
>ref-80344	CATACAAACACCATTGCTCCACGAAGT	26	<nnxnp>	nn	148	np	125		
>ref-80345	TCCCTCATTACTCCTCCTCCTTCCCCC	2	<lmxll>	ll	178	lm	79		
>ref-80349	TACATCTCCACTCCACCTCCACCTCCT	4	<nnxnp>	nn	266	np	0		
>ref-80349	TACATCTCCACTCCACCTCCACCTCCT	5	<lmxll>	ll	222	lm	30		
>ref-80349	TACATCTCCACTCCACCTCCACCTCCT	21	<lmxll>	ll	139	lm	91		
>ref-80349	TACATCTCCACTCCACCTCCACCTCCT	22	<lmxll>	ll	140	lm	92		
>ref-80359	TGTACCTTGACATTGGCTCCCTTCCCT	12	<nnxnp>	nn	98	np	166		
>ref-80368	CTTGCCCCCACATTTGCTCCTCTGTCT	5	<hkxhk>	hh	6	hk	265	kk	0
>ref-80408	ACTACACCCACTTTCCCTCCTTCTCTT	26	<nnxnp>	nn	124	np	147		
>ref-80409	TTTACCTGAACAAAAACTCCATGGAAA	6	<hkxhk>	hh	4	hk	252	kk	2
>ref-80409	TTTACCTGAACAAAAACTCCATGGAAA	12	<nnxnp>	nn	266	np	0		
>ref-80409	TTTACCTGAACAAAAACTCCATGGAAA	23	<hkxhk>	hh	3	hk	257	kk	2
>ref-80417	TTCGTCTTCACCCGTTCTCCTCTCTTC	22	<nnxnp>	nn	124	np	139		
>ref-80432	CGTCTTCCAACTCCTCCTCCTACCCTT	24	<nnxnp>	nn	225	np	2		
>ref-80438	CATTGTAAGACTTTAACTCCGCCGCAC	7	<hkxhk>	hh	60	hk	158	kk	47
>ref-80450	TGCTCCCCTACCCCCACTCCCTCCCTC	5	<nnxnp>	nn	256	np	0		
>ref-80450	TGCTCCCCTACCCCCACTCCCTCCCTC	25	<nnxnp>	nn	240	np	1		
>ref-80450	TGCTCCCCTACCCCCACTCCCTCCCTC	26	<nnxnp>	nn	250	np	0		
>ref-80465	TCCTGGCTGACTGCATCTCCCCCTTTC	24	<lmxll>	ll	126	lm	140		

>ref-80465	TCCTGGCTGACTGCATCTCCCCCTTTC	27	<lmxll>	ll	126	lm	140		
>ref-80469	CCTCTCTCCACCCCATCTCCTCCTTCC	15	<lmxll>	ll	189	lm	56		
>ref-80479	CTCCCCATCACCCACCCTCCTTCCCCT	16	<lmxll>	ll	237	lm	0		
>ref-80503	ATAATCAATACACAATCTCCAATTGCC	14	<lmxll>	ll	132	lm	103		
>ref-80548	ACATCCCAAACGCAGACTCCTGAACGG	7	<hkxhk>	hh	58	hk	206	kk	0
>ref-80549	GGACACGAAACCGACTCTCCAAGCGGA	7	<nnxnp>	nn	133	np	136		
>ref-80560	CCGCTCACTACGTGTACTCCGACCTCT	4	<nnxnp>	nn	128	np	130		
>ref-80584	AAAACAAATACTTTATCTCCTCCTCTT	23	<lmxll>	ll	112	lm	125		
>ref-80584	AAAACAAATACTTTATCTCCTCCTCTT	27	<lmxll>	ll	111	lm	125		
>ref-80628	GGACATGGGACCTGCACTCCTGAATCT	7	<nnxnp>	nn	113	np	126		
>ref-80629	CACATACACACACCACCTCCCCTTAAT	4	<nnxnp>	nn	269	np	0		
>ref-80629	CACATACACACACCACCTCCCCTTAAT	27	<nnxnp>	nn	123	np	134		
>ref-80637	AGCCCGTCGACTCTCACTCCTTTTCAC	2	<nnxnp>	nn	145	np	124		
>ref-80658	ACGCCCTCCACCTCCCCTCCCCCTTCC	3	<lmxll>	ll	20	lm	235		
>ref-80658	ACGCCCTCCACCTCCCCTCCCCCTTCC	21	<nnxnp>	nn	10	np	262		
>ref-80658	ACGCCCTCCACCTCCCCTCCCCCTTCC	22	<nnxnp>	nn	10	np	262		
>ref-80658	ACGCCCTCCACCTCCCCTCCCCCTTCC	24	<lmxll>	ll	28	lm	229		
>ref-80665	ACATCCTGTACGCTTCCTCCTGAGCAG	8	<hkxhk>	hh	7	hk	262	kk	0
>ref-80665	ACATCCTGTACGCTTCCTCCTGAGCAG	12	<hkxhk>	hh	0	hk	262	kk	7
>ref-80665	ACATCCTGTACGCTTCCTCCTGAGCAG	13	<hkxhk>	hh	3	hk	269	kk	0
>ref-80665	ACATCCTGTACGCTTCCTCCTGAGCAG	21	<hkxhk>	hh	0	hk	269	kk	3
>ref-80672	ATATCTTTCACCCTTCCTCCTCCAACA	5	<lmxll>	ll	117	lm	125		
>ref-80684	CACACCGCCACGCAATCTCCCCACCAT	24	<nnxnp>	nn	127	np	137		
>ref-80685	TGTATACATACATTTCCTCCCACAGAC	2	<lmxll>	ll	191	lm	59		
>ref-80691	TACACACAGACACTCACTCCAAACTAT	16	<nnxnp>	nn	119	np	140		
>ref-80707	TCCTCCCCAACCCTCTCTCCATGTTTT	2	<lmxll>	ll	261	lm	0		
>ref-80717	CTCCCTCTCACTTCCCCTCCATTCCTT	1	<nnxnp>	nn	147	np	114		
>ref-80717	CTCCCTCTCACTTCCCCTCCATTCCTT	12	<nnxnp>	nn	77	np	164		
>ref-80717	CTCCCTCTCACTTCCCCTCCATTCCTT	21	<nnxnp>	nn	63	np	185		
>ref-80719	ATGACGTCAACGGGACCTCCAGTATCT	26	<hkxhk>	hh	64	hk	147	kk	50
>ref-80737	GACAGCCTTACTATCTCTCCGCGTTAC	2	<lmxll>	ll	127	lm	134		
>ref-80737	GACAGCCTTACTATCTCTCCGCGTTAC	5	<hkxhk>	hh	53	hk	146	kk	58
>ref-80745	TTCCTTCCTACCCCTCCTCCCCCCTAC	4	<hkxhk>	hh	5	hk	160	kk	63
>ref-80745	TTCCTTCCTACCCCTCCTCCCCCCTAC	21	<hkxhk>	hh	5	hk	158	kk	65
>ref-80747	TCTTACTCTACACCCTCTCCCCCTCCC	1	<hkxhk>	hh	28	hk	225	kk	3
>ref-80747	TCTTACTCTACACCCTCTCCCCCTCCC	2	<hkxhk>	hh	22	hk	234	kk	3
>ref-80752	ATGGTTCTCACGTGCCCTCCAGTTGCT	15	<nnxnp>	nn	129	np	144		
>ref-80785	TTTGCAACAACCCCCCCTCCACTCCCT	13	<lmxll>	ll	5	lm	248		

>ref-80785	TTTGCAACAACCCCCCCTCCACTCCCT	21	<lmxll>	ll	5	lm	247		
>ref-80794	CTACCCACCACCACCACTCCACTCATT	1	<lmxll>	ll	116	lm	117		
>ref-80794	CTACCCACCACCACCACTCCACTCATT	2	<lmxll>	ll	116	lm	118		
>ref-80799	CTGCACCCAACACCCTCTCCTTCCATA	26	<lmxll>	ll	98	lm	125		
>ref-80832	CTCCCCTCTACTCTTTCTCCCTCCCCC	24	<nnxnp>	nn	120	np	133		
>ref-80832	CTCCCCTCTACTCTTTCTCCCTCCCCC	25	<nnxnp>	nn	126	np	131		
>ref-80872	ATTGTTGTTACTCACACTCCTCATGCT	3	<lmxll>	ll	137	lm	131		
>ref-80881	TCCTCCATCACGTTCCCTCCCGGCTCC	5	<lmxll>	ll	132	lm	138		
>ref-80910	CCCCTCTCCACACGTCCTCCCACTTCC	23	<lmxll>	ll	167	lm	100		
>ref-80924	CACTCATCCACCTTCCCTCCTCCCAAC	3	<nnxnp>	nn	121	np	113		
>ref-80939	TACACACGTACGCCAACTCCCATCAGT	8	<lmxll>	ll	131	lm	136		
>ref-80944	TGGTGCCTGACAGGCTCTCCGTTCCCG	27	<nnxnp>	nn	150	np	123		
>ref-80970	CCCTACCCTACTCCCTCTCCCCTCACC	23	<hkxhk>	hh	0	hk	240	kk	9
>ref-80970	CCCTACCCTACTCCCTCTCCCCTCACC	24	<hkxhk>	hh	9	hk	240	kk	0
>ref-81011	ACCCGTCACACAGCATCTCCTCCTGAA	5	<lmxll>	ll	141	lm	125		
>ref-81028	TTTTGTGGTACTTAAACTCCTTTCTCT	8	<nnxnp>	nn	130	np	116		
>ref-81039	CAAGAAGATACCTCCTCTCCCTCCTTG	3	<hkxhk>	hh	52	hk	123	kk	52
>ref-81064	GGCCCGGTAACCCGCTCTCCTGCTTCG	6	<nnxnp>	nn	269	np	0		
>ref-81100	TGTTGGTGAACTTTATCTCCGGCCGCC	1	<nnxnp>	nn	267	np	0		
>ref-81100	TGTTGGTGAACTTTATCTCCGGCCGCC	2	<nnxnp>	nn	267	np	0		
>ref-81118	GCTTTGTTTACTCCTTCTCCAATTCCT	15	<lmxll>	ll	132	lm	122		
>ref-81119	CTTCCTCCTACCAAACCTCCCTCTCCT	1	<hkxhk>	hh	0	hk	209	kk	49
>ref-81119	CTTCCTCCTACCAAACCTCCCTCTCCT	16	<lmxll>	ll	11	lm	257		
>ref-81129	CCATCCCCCACACCCTCTCCCCCACAC	1	<lmxll>	ll	130	lm	98		
>ref-81135	GAATACCATACACCTCCTCCAAAGGTA	24	<hkxhk>	hh	0	hk	254	kk	10
>ref-81154	TAAACTTCTACAGCTTCTCCAGGCTTT	24	<hkxhk>	hh	60	hk	144	kk	39
>ref-81179	GACGCCAAAACATCGGCTCCTTTGCTT	22	<lmxll>	ll	135	lm	121		
>ref-81185	CCTCTCATGACGACTGCTCCTCCTCCT	1	<lmxll>	ll	226	lm	15		
>ref-81188	CCTGTCTCTACCTTCCCTCCTCCTTGT	14	<hkxhk>	hh	60	hk	146	kk	61
>ref-81201	TATTCACTCACACTTCCTCCTACTCTT	8	<nnxnp>	nn	120	np	109		
>ref-81222	CGCTGTACCACGCCCCCTCCCCGCCCC	14	<nnxnp>	nn	117	np	142		
>ref-81223	AGGAGTATCACTTTATCTCCGACATTA	8	<nnxnp>	nn	167	np	89		
>ref-81223	AGGAGTATCACTTTATCTCCGACATTA	27	<nnxnp>	nn	167	np	89		
>ref-81235	TGGCTGGAAACCGCCCCTCCCGCGCAC	6	<nnxnp>	nn	140	np	130		
>ref-81241	ATGATGCGGACTTTTACTCCGGCGACA	26	<nnxnp>	nn	267	np	0		
>ref-81254	TCACCCTTCACGTTCCCTCCACGCCCT	25	<hkxhk>	hh	0	hk	273	kk	0
>ref-81270	CTTCCTCCAACCTCTTCTCCTCCATCC	16	<lmxll>	ll	231	lm	1		
>ref-81274	CTCTTCTCTACTCCTTCTCCCTTTTAT	3	<nnxnp>	nn	244	np	0		

>ref-81280	AGCAAGCAGACTCTGCCTCCGTTAGAT	16	<nnxnp>	nn	147	np	120		
>ref-81344	ACCCCAATCACACCACCTCCATGTTCA	2	<nnxnp>	nn	119	np	147		
>ref-81344	ACCCCAATCACACCACCTCCATGTTCA	24	<nnxnp>	nn	119	np	147		
>ref-81367	CCTAGCCTAACCTCGACTCCCATGTTA	14	<hkxhk>	hh	6	hk	263	kk	0
>ref-81367	CCTAGCCTAACCTCGACTCCCATGTTA	22	<hkxhk>	hh	34	hk	222	kk	0
>ref-81376	ATTCCTCCCACTCGTCCTCCTCCGCCA	16	<lmxll>	ll	123	lm	146		
>ref-81377	ATTTCCAGCACAGTGCCTCCAGAGTGT	7	<nnxnp>	nn	133	np	121		
>ref-81388	ATCCCTCCTACTGCTCCTCCTCCCGCT	1	<nnxnp>	nn	135	np	134		
>ref-81389	CTCACTTAAACGATGCCTCCTTGTTGT	16	<lmxll>	ll	113	lm	145		
>ref-81406	AGCGCGACAACTCACACTCCTCGGACA	14	<hkxhk>	hh	88	hk	173	kk	0
>ref-81465	AAATCATCCACTCGACCTCCAGGAAAC	1	<lmxll>	ll	131	lm	97		
>ref-81466	CTTTCTTCCACCTTCTCTCCCTTCTTT	14	<nnxnp>	nn	8	np	252		
>ref-81466	CTTTCTTCCACCTTCTCTCCCTTCTTT	22	<nnxnp>	nn	7	np	252		
>ref-81466	CTTTCTTCCACCTTCTCTCCCTTCTTT	27	<lmxll>	ll	151	lm	105		
>ref-81468	CGGTGGTGTACACATCCTCCCGCGCCT	14	<hkxhk>	hh	0	hk	272	kk	1
>ref-81468	CGGTGGTGTACACATCCTCCCGCGCCT	22	<hkxhk>	hh	0	hk	272	kk	1
>ref-81499	CTCCCCACCACCACCCCTCCTAACCCC	2	<nnxnp>	nn	130	np	112		
>ref-81499	CTCCCCACCACCACCCCTCCTAACCCC	6	<hkxhk>	hh	3	hk	171	kk	65
>ref-81499	CTCCCCACCACCACCCCTCCTAACCCC	8	<nnxnp>	nn	132	np	112		
>ref-81509	GGCGGGACCACAAACTCTCCTTTGTCT	8	<nnxnp>	nn	142	np	128		
>ref-81517	CCCTTCGCCACGCTTTCTCCCTCATCC	5	<nnxnp>	nn	126	np	143		
>ref-81533	ATCCGCGTGACAGGTTCTCCACGGAAT	6	<nnxnp>	nn	116	np	139		
>ref-81563	CATTTAATCACCATACCTCCATGTACA	26	<nnxnp>	nn	123	np	116		
>ref-81592	CGCGGGAAAACATTTGCTCCTCCGAGT	4	<lmxll>	ll	148	lm	121		
>ref-81592	CGCGGGAAAACATTTGCTCCTCCGAGT	27	<lmxll>	ll	6	lm	265		
>ref-81599	AGGACTCGAACCCACTCTCCCTAGATA	22	<hkxhk>	hh	1	hk	269	kk	0
>ref-81599	AGGACTCGAACCCACTCTCCCTAGATA	25	<hkxhk>	hh	0	hk	269	kk	1
>ref-81605	AATTGGAGGACCTCCGCTCCTCCTCGG	5	<lmxll>	ll	127	lm	139		
>ref-81633	AGTTGAGAAACGCTAGCTCCATTTACT	12	<nnxnp>	nn	120	np	148		
>ref-81646	TTCCCTCGCACCCTTTCTCCTTCCATC	15	<nnxnp>	nn	262	np	0		
>ref-81646	TTCCCTCGCACCCTTTCTCCTTCCATC	25	<nnxnp>	nn	259	np	1		
>ref-81684	TACAGTTTCACCTTACCTCCTCAGTCT	8	<hkxhk>	hh	54	hk	145	kk	40
>ref-81691	CCATGCACGACAAAGGCTCCCACGCAA	21	<lmxll>	ll	145	lm	125		
>ref-81700	CCTAACCTAACCTAACCTCCCTAACTT	6	<lmxll>	ll	186	lm	63		
>ref-81700	CCTAACCTAACCTAACCTCCCTAACTT	15	<nnxnp>	nn	77	np	184		
>ref-81700	CCTAACCTAACCTAACCTCCCTAACTT	22	<hkxhk>	hh	0	hk	169	kk	87
>ref-81700	CCTAACCTAACCTAACCTCCCTAACTT	26	<lmxll>	ll	13	lm	255		
>ref-81721	AACATTCCCACGCTCACTCCCACGCCC	21	<nnxnp>	nn	129	np	138		

>ref-81743	CCAGTCTTCACTTTCCCTCCTCCCCCC	5	<nnxnp>	nn	118	np	137		
>ref-81753	TGCTCCTCCACTCCCCCTCCCTCTTCC	2	<lmxll>	ll	31	lm	223		
>ref-81753	TGCTCCTCCACTCCCCCTCCCTCTTCC	6	<nnxnp>	nn	251	np	13		
>ref-81753	TGCTCCTCCACTCCCCCTCCCTCTTCC	13	<nnxnp>	nn	250	np	15		
>ref-81753	TGCTCCTCCACTCCCCCTCCCTCTTCC	26	<lmxll>	ll	47	lm	205		
>ref-81762	TGTACAAGCACTTCCGCTCCCTAAACC	7	<hkxhk>	hh	56	hk	155	kk	53
>ref-81765	AGGTGTAAAACCATTTCTCCCTCCCAT	3	<nnxnp>	nn	124	np	121		
>ref-81768	CATTGGCCCACTTTCCCTCCCAGACAT	2	<lmxll>	ll	123	lm	138		
>ref-81794	TTTTCATCCACCCAGCCTCCCACCTCC	15	<nnxnp>	nn	0	np	260		
>ref-81818	CCAACCGCCACTTCTCCTCCTTGACTG	6	<lmxll>	ll	133	lm	127		
>ref-81831	CTGGCGATGACCGTTTCTCCTCGCTGG	24	<nnxnp>	nn	127	np	134		
>ref-81850	AGAAAGGGTACCAAGACTCCTCCCTCG	8	<nnxnp>	nn	125	np	143		
>ref-81851	ATGGTGTTGACTGTAACTCCGGTGAGA	3	<lmxll>	ll	31	lm	203		
>ref-81851	ATGGTGTTGACTGTAACTCCGGTGAGA	4	<lmxll>	ll	30	lm	203		
>ref-81853	ACCTTCTCCACCTTCTCTCCTTCATCC	23	<lmxll>	ll	144	lm	125		
>ref-81878	TGCCACCGAACACTACCTCCTCCCCCT	16	<hkxhk>	hh	55	hk	153	kk	55
>ref-81900	TCCCATTCAACTCTCTCTCCTACCCTT	25	<lmxll>	ll	100	lm	160		
>ref-81900	TCCCATTCAACTCTCTCTCCTACCCTT	27	<lmxll>	ll	100	lm	160		
>ref-81924	ATCGCTTCCACAGTTCCTCCCGTTCAT	6	<lmxll>	ll	55	lm	206		
>ref-81924	ATCGCTTCCACAGTTCCTCCCGTTCAT	7	<hkxhk>	hh	0	hk	268	kk	4
>ref-81924	ATCGCTTCCACAGTTCCTCCCGTTCAT	8	<hkxhk>	hh	3	hk	269	kk	0
>ref-81924	ATCGCTTCCACAGTTCCTCCCGTTCAT	24	<hkxhk>	hh	0	hk	239	kk	23
>ref-81924	ATCGCTTCCACAGTTCCTCCCGTTCAT	25	<lmxll>	ll	55	lm	206		
>ref-81935	TGTATATTAACGCCCTCTCCTTTATCA	4	<nnxnp>	nn	114	np	152		
>ref-81952	CGAGTCCAGACTTCATCTCCAAAGCAG	2	<nnxnp>	nn	268	np	0		
>ref-81954	TTCCACCCTACTCCCTCTCCTTCCATT	26	<lmxll>	ll	255	lm	0		
>ref-81954	TTCCACCCTACTCCCTCTCCTTCCATT	27	<lmxll>	ll	253	lm	0		
>ref-81955	CTTACCCTTACCACAGCTCCCTCTGCC	21	<nnxnp>	nn	125	np	134		
>ref-82035	GCAAAGCCCACAGGACCTCCGCAGGTG	12	<hkxhk>	hh	2	hk	267	kk	1
>ref-82035	GCAAAGCCCACAGGACCTCCGCAGGTG	25	<hkxhk>	hh	1	hk	267	kk	2
>ref-82061	CGTTTTCATACTCCTCCTCCCCTCTCT	1	<lmxll>	ll	83	lm	155		
>ref-82061	CGTTTTCATACTCCTCCTCCCCTCTCT	8	<hkxhk>	hh	5	hk	252	kk	4
>ref-82061	CGTTTTCATACTCCTCCTCCCCTCTCT	12	<lmxll>	ll	83	lm	155		
>ref-82061	CGTTTTCATACTCCTCCTCCCCTCTCT	16	<hkxhk>	hh	5	hk	252	kk	4
>ref-82114	CTCATCCCCACCTTCTCTCCCCATCTC	2	<lmxll>	ll	125	lm	120		
>ref-82153	CTCATACACACTCCACCTCCTACATCT	1	<nnxnp>	nn	85	np	139		
>ref-82153	CTCATACACACTCCACCTCCTACATCT	14	<hkxhk>	hh	1	hk	271	kk	0
>ref-82153	CTCATACACACTCCACCTCCTACATCT	15	<lmxll>	ll	111	lm	148		

>ref-82153	CTCATACACACTCCACCTCCTACATCT	25	<lmxll>	ll	110	lm	146		
>ref-82153	CTCATACACACTCCACCTCCTACATCT	27	<nnxnp>	nn	123	np	138		
>ref-82181	TAGAGATCCACATCGTCTCCATCACTG	12	<lmxll>	ll	138	lm	130		
>ref-82191	AGTTTATTAACAAGATCTCCTAACCTC	26	<nnxnp>	nn	124	np	106		
>ref-82211	ACTGAGGCGACCGCATCTCCGTGGGAA	21	<nnxnp>	nn	263	np	0		
>ref-82221	GTCCCTGCCACTGCCTCTCCCCCCCTT	2	<hkxhk>	hh	34	hk	126	kk	82
>ref-82221	GTCCCTGCCACTGCCTCTCCCCCCCTT	16	<hkxhk>	hh	34	hk	126	kk	82
>ref-82256	TTCCAACTCACGCCATCTCCCTCCTTC	25	<hkxhk>	hh	54	hk	133	kk	50
>ref-82261	AGGCCGAGTACACACGCTCCTTGGTCA	15	<lmxll>	ll	139	lm	131		
>ref-82264	CATGTTCAGACATTGACTCCGCCATTA	4	<lmxll>	ll	138	lm	131		
>ref-82281	ATTAAAGACACTTCTGCTCCCGTATCA	2	<hkxhk>	hh	0	hk	227	kk	32
>ref-82281	ATTAAAGACACTTCTGCTCCCGTATCA	7	<lmxll>	ll	214	lm	39		
>ref-82281	ATTAAAGACACTTCTGCTCCCGTATCA	21	<hkxhk>	hh	28	hk	225	kk	0
>ref-82296	CACTCCCCGACAGGGCCTCCTTTCCCC	23	<nnxnp>	nn	130	np	135		
>ref-82305	CGTCTCCCAACGTCTGCTCCGATGGAA	1	<hkxhk>	hh	2	hk	194	kk	71
>ref-82305	CGTCTCCCAACGTCTGCTCCGATGGAA	2	<hkxhk>	hh	1	hk	268	kk	3
>ref-82305	CGTCTCCCAACGTCTGCTCCGATGGAA	12	<hkxhk>	hh	2	hk	194	kk	71
>ref-82305	CGTCTCCCAACGTCTGCTCCGATGGAA	25	<hkxhk>	hh	1	hk	268	kk	2
>ref-82310	ATACCACCCACAAACACTCCCTTCCCA	9	<hkxhk>	hh	1	hk	130	kk	137
>ref-82310	ATACCACCCACAAACACTCCCTTCCCA	26	<hkxhk>	hh	138	hk	130	kk	1
>ref-82336	ACGAGAGACACATTATCTCCAGCAAAC	1	<lmxll>	ll	118	lm	147		
>ref-82352	CCCTCCCCCACACGCCCTCCGCCCTCT	22	<lmxll>	ll	259	lm	0		
>ref-82353	ACATTGCACACTCTCTCTCCAACTATC	25	<nnxnp>	nn	233	np	0		
>ref-82353	ACATTGCACACTCTCTCTCCAACTATC	27	<nnxnp>	nn	233	np	2		
>ref-82364	TCATGTTCTACAACATCTCCACTCTCT	1	<lmxll>	ll	129	lm	138		
>ref-82402	CTTCCTCTCACTCTCCCTCCCACAACC	8	<nnxnp>	nn	20	np	239		
>ref-82408	ACCCCGTGAACGAGTCCTCCTCCAACA	1	<hkxhk>	hh	0	hk	273	kk	0
>ref-82408	ACCCCGTGAACGAGTCCTCCTCCAACA	4	<hkxhk>	hh	0	hk	273	kk	0
>ref-82408	ACCCCGTGAACGAGTCCTCCTCCAACA	6	<hkxhk>	hh	0	hk	273	kk	0
>ref-82408	ACCCCGTGAACGAGTCCTCCTCCAACA	27	<lmxll>	ll	162	lm	105		
>ref-82412	AGCCAGCGCACAACCTCTCCCTTCTCC	21	<nnxnp>	nn	103	np	133		
>ref-82436	GACTGGCAGACTCTCTCTCCCTTTCTC	6	<lmxll>	ll	8	lm	262		
>ref-82436	GACTGGCAGACTCTCTCTCCCTTTCTC	13	<lmxll>	ll	8	lm	261		
>ref-82449	TTCCTATTTACCTTTTCTCCTTTCTTT	3	<hkxhk>	hh	0	hk	249	kk	10
>ref-82449	TTCCTATTTACCTTTTCTCCTTTCTTT	12	<hkxhk>	hh	0	hk	249	kk	11
>ref-82467	GCCGTTGCGACACGGTCTCCGTGCGCG	3	<nnxnp>	nn	273	np	0		
>ref-82518	GCAGGCATCACAAACCCTCCTCCTTTC	26	<nnxnp>	nn	124	np	137		
>ref-82519	AAATCTGTGACCAATTCTCCCTCGGTG	5	<hkxhk>	hh	49	hk	142	kk	56

>ref-82537	CACAGGAGGACCCAGTCTCCTTTGTCC	22	<lmxll>	ll	121	lm	132		
>ref-82549	CGCCACCTCACACCCCCTCCTTTCCCC	4	<nnxnp>	nn	269	np	0		
>ref-82550	CTTACCACCACACCCCCTCCCCTCCCC	4	<nnxnp>	nn	246	np	0		
>ref-82554	TCTCTATCTACCCGTCCTCCCCTCTTC	2	<hkxhk>	hh	1	hk	260	kk	1
>ref-82554	TCTCTATCTACCCGTCCTCCCCTCTTC	13	<hkxhk>	hh	0	hk	261	kk	1
>ref-82560	CTATTAAACACTCGCCCTCCGCTGTAC	13	<nnxnp>	nn	126	np	142		
>ref-82587	TTCTCCTTCACCTCTCCTCCTAGTCAT	6	<nnxnp>	nn	119	np	140		
>ref-82597	TCCTCCTGTACCTCCCCTCCCATCTAC	25	<hkxhk>	hh	38	hk	108	kk	76
>ref-82597	TCCTCCTGTACCTCCCCTCCCATCTAC	26	<hkxhk>	hh	74	hk	109	kk	39
>ref-82606	TTTTTCCCCACCATCTCTCCTCCCTTT	5	<hkxhk>	hh	3	hk	245	kk	2
>ref-82606	TTTTTCCCCACCATCTCTCCTCCCTTT	24	<hkxhk>	hh	56	hk	193	kk	0
>ref-82613	GGCCTACTTACACCCTCTCCCACTGGC	26	<nnxnp>	nn	155	np	107		
>ref-82614	TCCTTCACCACCCCCACTCCATCCCCT	25	<lmxll>	ll	128	lm	139		
>ref-82618	AGCTTGGCAACAATTTCTCCAGGCTTT	2	<hkxhk>	hh	0	hk	213	kk	44
>ref-82618	AGCTTGGCAACAATTTCTCCAGGCTTT	14	<hkxhk>	hh	0	hk	272	kk	0
>ref-82618	AGCTTGGCAACAATTTCTCCAGGCTTT	21	<hkxhk>	hh	5	hk	267	kk	0
>ref-82618	AGCTTGGCAACAATTTCTCCAGGCTTT	23	<hkxhk>	hh	0	hk	272	kk	1
>ref-82657	CACTTCTGCACCCTTTCTCCCTCTCAC	8	<lmxll>	ll	148	lm	125		
>ref-82675	CCTTTTCCTACTCTTCCTCCTAAGCCT	2	<nnxnp>	nn	251	np	0		
>ref-82677	GAAAAGAAGACTCTCTCTCCCCTCCCT	4	<hkxhk>	hh	0	hk	270	kk	0
>ref-82677	GAAAAGAAGACTCTCTCTCCCCTCCCT	27	<hkxhk>	hh	0	hk	270	kk	0
>ref-82682	GACATGGACACTGGTTCTCCTTAAACA	5	<nnxnp>	nn	116	np	138		
>ref-82696	CCTTCTTAAACACCCTCTCCCTCCCAC	12	<lmxll>	ll	122	lm	127		
>ref-82699	TTCTCTTTGACTCTCTCTCCTGGAATG	6	<hkxhk>	hh	0	hk	269	kk	0
>ref-82699	TTCTCTTTGACTCTCTCTCCTGGAATG	9	<hkxhk>	hh	0	hk	269	kk	0
>ref-82699	TTCTCTTTGACTCTCTCTCCTGGAATG	26	<hkxhk>	hh	0	hk	247	kk	8
>ref-82703	CAACTCGACACGATATCTCCTTGTGTA	1	<lmxll>	ll	90	lm	133		
>ref-82730	ATTTGTCTCACCGATTCTCCAAGACAG	15	<lmxll>	ll	132	lm	129		
>ref-82730	ATTTGTCTCACCGATTCTCCAAGACAG	24	<nnxnp>	nn	112	np	151		
>ref-82737	GCCCTCATAACAAGGGCTCCGGAGGGC	21	<hkxhk>	hh	0	hk	131	kk	129
>ref-82737	GCCCTCATAACAAGGGCTCCGGAGGGC	22	<hkxhk>	hh	0	hk	13	kk	256
>ref-82753	ACTGCTGGCACGCCGCCTCCTTGTACA	9	<lmxll>	ll	137	lm	135		
>ref-82756	CGCGCCAAGACTTCTCCTCCCTTCCCC	5	<lmxll>	ll	63	lm	187		
>ref-82765	ATGAGGTCAACCGACTCTCCAAACTTT	14	<nnxnp>	nn	163	np	76		
>ref-82766	GTGTCTGCCACTGGCCCTCCGCGGATG	8	<lmxll>	ll	127	lm	141		
>ref-82768	GGATACAATACATGTACTCCTGCCTAC	26	<nnxnp>	nn	263	np	1		
>ref-82788	CTCTTTCCCACTCCCTCTCCTACCTTT	4	<lmxll>	ll	267	lm	0		
>ref-82788	CTCTTTCCCACTCCCTCTCCTACCTTT	24	<lmxll>	ll	267	lm	0		

>ref-82854	TTTGTTACGACTTTTCCTCCGTCCATT	24	<lmxll>	ll	120	lm	151		
>ref-82859	TTCCGACGAACTCCCCCTCCCCTTCTT	12	<nnxnp>	nn	257	np	0		
>ref-82874	AGTCAAAACACCTAAGCTCCCCATCTA	22	<lmxll>	ll	124	lm	130		
>ref-82875	TGCTTGTACACACCATCTCCTTTTTTG	25	<lmxll>	ll	116	lm	132		
>ref-82878	AGCGCGCCCACCGCCCCTCCCTCCCTC	13	<hkxhk>	hh	87	hk	132	kk	0
>ref-82887	CAGCCCCCGACGCCCTCTCCTTCCCGA	21	<lmxll>	ll	114	lm	127		
>ref-82893	TTCTTCACCACTCCCTCTCCTCCTCCA	6	<lmxll>	ll	120	lm	130		
>ref-82901	CACACACCCACACCCGCTCCCGTCTCG	14	<nnxnp>	nn	128	np	124		
>ref-82904	GGCTCCTGTACCAGTGCTCCAACCTGC	12	<lmxll>	ll	94	lm	164		
>ref-82910	ACTCCTACCACATCCTCTCCCTCTACT	7	<lmxll>	ll	129	lm	134		
>ref-82919	CCAACACTAACACCTCCTCCTCCTCTC	2	<hkxhk>	hh	0	hk	207	kk	62
>ref-82919	CCAACACTAACACCTCCTCCTCCTCTC	14	<lmxll>	ll	206	lm	63		
>ref-82940	CCTCCTCCCACCGTCCCTCCAACCTCC	14	<lmxll>	ll	121	lm	122		
>ref-82949	CCCTTTCTTACCTGACCTCCAACACCA	4	<hkxhk>	hh	61	hk	133	kk	60
>ref-82949	CCCTTTCTTACCTGACCTCCAACACCA	24	<hkxhk>	hh	60	hk	133	kk	62
>ref-82951	AATTATACCACACGATCTCCCTAGCTC	23	<lmxll>	ll	104	lm	117		
>ref-82952	ACTCCGATTACTCCTACTCCCATTCCT	1	<lmxll>	ll	163	lm	63		
>ref-82953	ATCATACCCACCCTCACTCCGCCTCTT	12	<nnxnp>	nn	121	np	113		
>ref-82957	TTCCCTTCCACACCTTCTCCTTCCTTT	22	<nnxnp>	nn	122	np	148		
>ref-82957	TTCCCTTCCACACCTTCTCCTTCCTTT	23	<nnxnp>	nn	122	np	148		
>ref-82959	CAAATGAACACAGAGACTCCACCTTCT	27	<nnxnp>	nn	117	np	145		
>ref-82972	GTGCTGGTTACTCTATCTCCCAACCCT	1	<nnxnp>	nn	19	np	244		
>ref-82977	CTGAGTCCCACCCTTCCTCCGTCTTTC	3	<nnxnp>	nn	107	np	155		
>ref-82977	CTGAGTCCCACCCTTCCTCCGTCTTTC	25	<nnxnp>	nn	106	np	155		
>ref-82980	CTCTCTACCACATCATCTCCCTCTCCC	7	<lmxll>	ll	2	lm	271		
>ref-82990	CTCCCTCCCACACCGCCTCCGCTTCCA	3	<hkxhk>	hh	3	hk	268	kk	0
>ref-82990	CTCCCTCCCACACCGCCTCCGCTTCCA	23	<hkxhk>	hh	1	hk	260	kk	7
>ref-83000	CCTTTATATACTCTCCCTCCCGTCCCT	21	<lmxll>	ll	113	lm	145		
>ref-83001	TCTCCTCTTACCCTCACTCCATTCCTC	22	<hkxhk>	hh	44	hk	160	kk	65
>ref-83005	ACTTCTGATACACCCTCTCCTTCTCCA	14	<lmxll>	ll	129	lm	133		
>ref-83005	ACTTCTGATACACCCTCTCCTTCTCCA	27	<lmxll>	ll	126	lm	133		
>ref-83022	CAGCGATTTACTTCAACTCCTGTTACA	25	<hkxhk>	hh	62	hk	126	kk	54
>ref-83027	ACCTCCTTAACTGCTTCTCCTCTTCTT	14	<nnxnp>	nn	124	np	132		
>ref-83027	ACCTCCTTAACTGCTTCTCCTCTTCTT	26	<nnxnp>	nn	125	np	131		
>ref-83029	CGTTCACCAACCCCCCCTCCCGCTCTC	2	<lmxll>	ll	109	lm	151		
>ref-83044	TGCAGCAGCACTCCCACTCCCCCACAT	24	<nnxnp>	nn	124	np	137		
>ref-83048	ACAAGTAAAACTTCCCCTCCTCTCCAC	24	<lmxll>	ll	135	lm	132		
>ref-83053	CGGCTCAGAACACAGACTCCTCGCCAT	9	<nnxnp>	nn	139	np	128		

>ref-83072	ACCGGCACCACCTCCCCTCCGTCTTAC	9	<hkxhk>	hh	63	hk	141	kk	67
>ref-83072	ACCGGCACCACCTCCCCTCCGTCTTAC	22	<hkxhk>	hh	67	hk	141	kk	63
>ref-83095	CCCTTCATCACCTATTCTCCTTCCCTT	9	<hkxhk>	hh	1	hk	268	kk	0
>ref-83096	CACCCCTCAACATCTTCTCCCCCTCCC	26	<hkxhk>	hh	8	hk	263	kk	0
>ref-83102	CCTCCATCAACCCCTTCTCCATCCGTT	25	<hkxhk>	hh	51	hk	153	kk	51
>ref-83136	AATGCCCTTACTCCACCTCCTACCGTC	21	<lmxll>	ll	130	lm	139		
>ref-83137	AGGCTCACCACCGCCCCTCCAGCCAAG	1	<nnxnp>	nn	131	np	133		
>ref-83141	CTCCCCCCCACACTTTCTCCCCCTCCT	9	<hkxhk>	hh	32	hk	160	kk	29
>ref-83145	CTGCCTTCCACGACTCCTCCCCCCTCC	16	<nnxnp>	nn	259	np	0		
>ref-83150	TACACACTAACATTAGCTCCAAGTTAC	15	<nnxnp>	nn	273	np	0		
>ref-83152	AAGTTAACTACTCGCCCTCCACGTTCA	1	<lmxll>	ll	143	lm	118		
>ref-83168	CCGCGAACTACAGATCCTCCTCCTCCT	12	<lmxll>	ll	39	lm	231		
>ref-83172	TGCATATAAACTGACTCTCCACACTCC	15	<nnxnp>	nn	133	np	125		
>ref-83200	TTATCCCTAACCCCCACTCCTCACCCT	1	<nnxnp>	nn	95	np	157		
>ref-83205	ATCACTTGGACATTCCCTCCTCTTCCT	24	<lmxll>	ll	131	lm	126		
>ref-83233	GCTCTCACTACTTCTTCTCCGCCGTCT	6	<nnxnp>	nn	269	np	0		
>ref-83250	TTCCGTTATACTTTTTCTCCTTCTTCT	12	<lmxll>	ll	95	lm	130		
>ref-83250	TTCCGTTATACTTTTTCTCCTTCTTCT	25	<lmxll>	ll	97	lm	128		
>ref-83257	ACCTCAACCACCGAGACTCCCAACGCT	1	<lmxll>	ll	111	lm	145		
>ref-83268	ACTCAAATTACTCTGTCTCCTCCTCAT	26	<nnxnp>	nn	122	np	123		
>ref-83295	TCTACTTCTACTCGCTCTCCCTCTCCT	2	<nnxnp>	nn	159	np	102		
>ref-83295	TCTACTTCTACTCGCTCTCCCTCTCCT	5	<nnxnp>	nn	158	np	102		
>ref-83295	TCTACTTCTACTCGCTCTCCCTCTCCT	6	<hkxhk>	hh	50	hk	206	kk	3
>ref-83295	TCTACTTCTACTCGCTCTCCCTCTCCT	27	<hkxhk>	hh	49	hk	206	kk	3
>ref-83300	GCCTCCGTTACCCTCGCTCCTTGCTCA	6	<nnxnp>	nn	79	np	174		
>ref-83300	GCCTCCGTTACCCTCGCTCCTTGCTCA	24	<nnxnp>	nn	76	np	175		
>ref-83328	GCTCTGATGACATGACCTCCGTGCATA	23	<lmxll>	ll	107	lm	145		
>ref-83338	ATATTAGCAACCAATTCTCCCCCGTGA	21	<nnxnp>	nn	129	np	119		
>ref-83341	ACAACGAAGACTTACACTCCTGATGGA	8	<nnxnp>	nn	129	np	131		
>ref-83358	CATGATTTCACCGTACCTCCTGCCTCC	12	<hkxhk>	hh	63	hk	144	kk	51
>ref-83390	TCGACGCTAACATTAACTCCTTCGCCA	8	<nnxnp>	nn	135	np	123		
>ref-83390	TCGACGCTAACATTAACTCCTTCGCCA	26	<nnxnp>	nn	134	np	123		
>ref-83397	CTTTCTTCTACCCCATCTCCTCTCTTT	12	<hkxhk>	hh	85	hk	147	kk	0
>ref-83397	CTTTCTTCTACCCCATCTCCTCTCTTT	22	<hkxhk>	hh	87	hk	147	kk	0
>ref-83411	TTCTTTAGCACTCGCTCTCCCAGTCTA	13	<lmxll>	ll	142	lm	116		
>ref-83411	TTCTTTAGCACTCGCTCTCCCAGTCTA	14	<lmxll>	ll	142	lm	116		
>ref-83416	ACGTCCACTACAACCCCTCCCTTGCCC	26	<lmxll>	ll	140	lm	128		
>ref-83416	ACGTCCACTACAACCCCTCCCTTGCCC	27	<lmxll>	ll	141	lm	128		

>ref-83424	AATGTTTACACCTTAACTCCTTCATTT	25	<hkxhk>	hh	27	hk	192	kk	0
>ref-83424	AATGTTTACACCTTAACTCCTTCATTT	27	<hkxhk>	hh	74	hk	146	kk	0
>ref-83467	GATATGTCTACTCACCCTCCCCCCTCT	27	<lmxll>	ll	137	lm	114		
>ref-83482	CAAACACACACCATACCTCCACCATAA	16	<lmxll>	ll	135	lm	134		
>ref-83492	GTGTCCTAAACCGCCTCTCCAGCAGGT	13	<nnxnp>	nn	136	np	127		
>ref-83493	TCCCATCCTACTTACTCTCCCATTCCC	14	<hkxhk>	hh	76	hk	187	kk	2
>ref-83493	TCCCATCCTACTTACTCTCCCATTCCC	22	<hkxhk>	hh	8	hk	265	kk	0
>ref-83493	TCCCATCCTACTTACTCTCCCATTCCC	23	<hkxhk>	hh	1	hk	264	kk	8
>ref-83510	CCACGCCCGACACCCCCTCCTTCCTTC	9	<nnxnp>	nn	123	np	130		
>ref-83523	CCCTTCATCACGTCCTCTCCTCCTCCT	13	<lmxll>	ll	141	lm	131		
>ref-83527	TTTCCCTCCACTCATCCTCCCTCCTTT	15	<lmxll>	ll	263	lm	1		
>ref-83527	TTTCCCTCCACTCATCCTCCCTCCTTT	25	<lmxll>	ll	260	lm	4		
>ref-83538	TGCCTGTGGACCATGACTCCATACCTT	3	<hkxhk>	hh	66	hk	183	kk	16
>ref-83538	TGCCTGTGGACCATGACTCCATACCTT	5	<lmxll>	ll	112	lm	155		
>ref-83538	TGCCTGTGGACCATGACTCCATACCTT	8	<hkxhk>	hh	16	hk	183	kk	66
>ref-83548	CTATCCACGACCTTAACTCCCAGGTTT	13	<lmxll>	ll	121	lm	133		
>ref-83556	ATAATGCTGACAACCACTCCAATAGCA	7	<nnxnp>	nn	121	np	124		
>ref-83563	GGCGCAAGGACTGACTCTCCTCAGCGT	1	<lmxll>	ll	137	lm	126		
>ref-83592	AGGACACTAACCCCCCCTCCCGTCCAA	13	<hkxhk>	hh	56	hk	143	kk	61
>ref-83599	TTTCATTTCACTCCATCTCCTTTCTCA	14	<lmxll>	ll	121	lm	104		
>ref-83626	CTCGGTACGACGGTTTCTCCCCCGTGT	13	<lmxll>	ll	105	lm	158		
>ref-83656	CCTCTCACCACTTGCTCTCCTTCCTTC	4	<lmxll>	ll	114	lm	149		
>ref-83656	CCTCTCACCACTTGCTCTCCTTCCTTC	5	<lmxll>	ll	114	lm	149		
>ref-83661	CGCCTTTCCACCCTTCCTCCACCCCTC	22	<nnxnp>	nn	269	np	1		
>ref-83671	TCGCGTTGCACTTTCCCTCCTTTTATC	13	<nnxnp>	nn	138	np	126		
>ref-83672	CATACCCCTACATAGGCTCCTGCATAC	22	<lmxll>	ll	129	lm	95		
>ref-83710	AACAAATCCACATTTTCTCCTGACCAT	22	<nnxnp>	nn	256	np	0		
>ref-83723	CTACCCACCACCGTTTCTCCTGCTGTT	13	<nnxnp>	nn	138	np	119		
>ref-83728	CCCTGCCTCACCCTGCCTCCCTTTTTC	26	<nnxnp>	nn	121	np	131		
>ref-83728	CCCTGCCTCACCCTGCCTCCCTTTTTC	27	<nnxnp>	nn	121	np	131		
>ref-83729	TCTCCCCTTACTTTCCCTCCCTTTTCT	3	<hkxhk>	hh	0	hk	262	kk	4
>ref-83729	TCTCCCCTTACTTTCCCTCCCTTTTCT	24	<hkxhk>	hh	0	hk	263	kk	4
>ref-83738	GGCGTTCAAACATATGCTCCTTCGCTT	6	<hkxhk>	hh	52	hk	144	kk	60
>ref-83743	TTTGAATAAACTTCCCCTCCCTCCCTC	26	<lmxll>	ll	166	lm	95		
>ref-83745	GACTCCCTCACTATGCCTCCAATCACC	1	<lmxll>	ll	115	lm	146		
>ref-83745	GACTCCCTCACTATGCCTCCAATCACC	5	<lmxll>	ll	115	lm	146		
>ref-83773	CCCAATCGCACCCAAACTCCTGCCGTG	16	<nnxnp>	nn	264	np	0		
>ref-83776	TTCTTCTTCACGCTCTCTCCCTGCTTC	1	<lmxll>	ll	148	lm	121		

>ref-83781	TCTTCCCCACCTCCCCTCCTTCCCTT	7	<nnxnp>	nn	254	np	5		
>ref-83805	CTCTCCTCTACTTCCTCTCCCATTACT	6	<lmxll>	ll	119	lm	122		
>ref-83832	ACCTTCTCCACTCTTCCTCCCTTTCTT	13	<nnxnp>	nn	130	np	96		
>ref-83836	ACTACTATTACTACAACTCCACGTTCT	7	<nnxnp>	nn	104	np	148		
>ref-83852	AGAACACCAACACCAACTCCTCGTGGC	5	<nnxnp>	nn	119	np	153		
>ref-83871	AACAGTCACACCCTATCTCCTTGCGAA	2	<nnxnp>	nn	130	np	122		
>ref-83881	CTAGGTACTACCTTTCCTCCTGGACTA	3	<nnxnp>	nn	113	np	145		
>ref-83888	CTCCTCCCTACCTCCCCTCCCCCCATA	9	<lmxll>	ll	89	lm	131		
>ref-83888	CTCCTCCCTACCTCCCCTCCCCCCATA	15	<nnxnp>	nn	2	np	260		
>ref-83888	CTCCTCCCTACCTCCCCTCCCCCCATA	25	<lmxll>	ll	84	lm	138		
>ref-83898	CCACGGCAGACAGCTCCTCCAGTGCGT	9	<hkxhk>	hh	0	hk	273	kk	0
>ref-83898	CCACGGCAGACAGCTCCTCCAGTGCGT	21	<lmxll>	ll	0	lm	273		
>ref-83912	CCTTCCTCCACCCCATCTCCTCACCAC	27	<nnxnp>	nn	156	np	111		
>ref-83929	CCCCTCTCTACACACTCTCCTCTCTCT	3	<nnxnp>	nn	238	np	1		
>ref-83937	CTTCCTTTTACCTGTCCTCCTATCCCT	26	<hkxhk>	hh	61	hk	143	kk	52
>ref-83954	AGACTGAACACACAAACTCCAAAGACA	3	<hkxhk>	hh	51	hk	130	kk	56
>ref-83972	TCTTCTCCTACTTTCTCTCCTTTTCCA	25	<hkxhk>	hh	30	hk	190	kk	14
>ref-83977	GCCCGTTCCACAATCTCTCCAGGATTC	25	<hkxhk>	hh	59	hk	145	kk	60
>ref-84000	CCCCTGCCAACGCGGTCTCCTCTCTTC	22	<nnxnp>	nn	267	np	0		
>ref-84001	TTCGTCATTACGTCCCCTCCATTTGCC	25	<hkxhk>	hh	0	hk	127	kk	133
>ref-84012	TCCGCTTTCACCCCCCCTCCTCTCCCA	27	<nnxnp>	nn	138	np	108		
>ref-84036	CAACCACCGACGTTCCCTCCCGTAGCT	6	<hkxhk>	hh	46	hk	142	kk	71
>ref-84047	ATGCCCAGCACTTCATCTCCCTGTGTT	5	<nnxnp>	nn	145	np	124		
>ref-84063	ACATACGCAACCTCAGCTCCGGACTGA	23	<nnxnp>	nn	233	np	0		
>ref-84084	CAGTGGCTGACCCTCTCTCCACCTCCC	7	<hkxhk>	hh	54	hk	140	kk	62
>ref-84097	TTTACGTCAACCATATCTCCGTTTCGT	9	<lmxll>	ll	237	lm	26		
>ref-84097	TTTACGTCAACCATATCTCCGTTTCGT	25	<nnxnp>	nn	178	np	88		
>ref-84117	TCTTCACTTACTCCTTCTCCCATTCTC	8	<lmxll>	ll	107	lm	144		
>ref-84123	GGCCTAACGACTCAGGCTCCGACTCAT	15	<lmxll>	ll	114	lm	158		
>ref-84139	CCTCCCCTAACTCGTTCTCCCCGAGTG	9	<nnxnp>	nn	124	np	127		
>ref-84150	CCCAACACCACAACTTCTCCCCAACAC	9	<nnxnp>	nn	226	np	5		
>ref-84171	ACTACTGTTACTGCTGCTCCAGTTACT	4	<lmxll>	ll	43	lm	207		
>ref-84171	ACTACTGTTACTGCTGCTCCAGTTACT	27	<lmxll>	ll	43	lm	204		
>ref-84175	ATTAAGGAAACCTCTCCTCCGTCTCAT	4	<nnxnp>	nn	142	np	119		
>ref-84196	CCCTCCTTCACTCACACTCCTCTAGGG	14	<nnxnp>	nn	126	np	130		
>ref-84200	AGTTATATCACATTACCTCCTTTTCTC	25	<lmxll>	ll	121	lm	111		
>ref-84261	TCTGGTGACACCGCTGCTCCCAACCTC	3	<hkxhk>	hh	0	hk	84	kk	170
>ref-84261	TCTGGTGACACCGCTGCTCCCAACCTC	14	<hkxhk>	hh	0	hk	269	kk	1

>ref-84265	CATTCTCTTACCTTCTCTCCGTCTTTC	25	<lmxll>	ll	121	lm	133		
>ref-84273	TCATCTCGTACTTGCTCTCCTTTATCA	9	<lmxll>	ll	262	lm	0		
>ref-84276	CAAGATTAAACACCGCCTCCCACCTCC	25	<lmxll>	ll	107	lm	156		
>ref-84283	AAGGCTATCACGCTCCCTCCTCTTCCT	2	<lmxll>	ll	121	lm	151		
>ref-84303	TACTTTCTCACTTTCTCTCCCTTCTGT	5	<nnxnp>	nn	115	np	123		
>ref-84309	TGGCGCCCGACAGCACCTCCTTCACCG	27	<lmxll>	ll	269	lm	0		
>ref-84321	CACGCAACGACAGCGCCTCCTCCAACG	26	<nnxnp>	nn	132	np	138		
>ref-84331	GTGGCACTCACAGCGGCTCCTCCAAGT	24	<hkxhk>	hh	0	hk	273	kk	0
>ref-84331	GTGGCACTCACAGCGGCTCCTCCAAGT	25	<lmxll>	ll	4	lm	267		
>ref-84359	TCACCCTCCACCCTTCCTCCATCTTTC	1	<nnxnp>	nn	6	np	235		
>ref-84359	TCACCCTCCACCCTTCCTCCATCTTTC	24	<lmxll>	ll	236	lm	10		
>ref-84361	CCACTCTCCACCCTTTCTCCCCTTCCA	4	<nnxnp>	nn	236	np	4		
>ref-84361	CCACTCTCCACCCTTTCTCCCCTTCCA	7	<nnxnp>	nn	235	np	5		
>ref-84361	CCACTCTCCACCCTTTCTCCCCTTCCA	9	<hkxhk>	hh	128	hk	110	kk	0
>ref-84361	CCACTCTCCACCCTTTCTCCCCTTCCA	21	<hkxhk>	hh	96	hk	137	kk	0
>ref-84366	TATTTGCTTACACCTTCTCCCTCCTTT	27	<lmxll>	ll	123	lm	140		
>ref-84367	TCCCCCTAAACTTCCCCTCCCTCTCCT	3	<hkxhk>	hh	51	hk	216	kk	0
>ref-84367	TCCCCCTAAACTTCCCCTCCCTCTCCT	27	<hkxhk>	hh	0	hk	211	kk	30
>ref-84379	TATGCTCCCACACAATCTCCCAGAGAA	9	<hkxhk>	hh	37	hk	150	kk	53
>ref-84387	TAACTACCTACCACGCCTCCTTTTCGA	1	<hkxhk>	hh	0	hk	235	kk	26
>ref-84391	AAAACCTGAACCTCCCCTCCTCTTTAA	14	<lmxll>	ll	115	lm	139		
>ref-84395	TCACTCTCAACACCCCCTCCCTCCAAT	14	<nnxnp>	nn	263	np	0		
>ref-84401	CTCTCTCTCACCCTTTCTCCAATTCAA	3	<nnxnp>	nn	271	np	1		
>ref-84405	ACCAACGATACCTCTCCTCCCAGTATC	27	<hkxhk>	hh	60	hk	143	kk	45
>ref-84414	CCTCCGTAAACTTGATCTCCTGATTTG	8	<lmxll>	ll	134	lm	101		
>ref-84418	ACTCAAAACACGCCATCTCCTCTCGAC	27	<nnxnp>	nn	273	np	0		
>ref-84430	TCCCTAAACACCCTCCCTCCTCCTCCC	3	<nnxnp>	nn	126	np	120		
>ref-84430	TCCCTAAACACCCTCCCTCCTCCTCCC	4	<hkxhk>	hh	3	hk	266	kk	0
>ref-84430	TCCCTAAACACCCTCCCTCCTCCTCCC	13	<hkxhk>	hh	13	hk	255	kk	0
>ref-84436	TTCTCTTACACTCACGCTCCTTCTGGC	15	<nnxnp>	nn	127	np	141		
>ref-84436	TTCTCTTACACTCACGCTCCTTCTGGC	16	<lmxll>	ll	144	lm	126		
>ref-84461	AACACGCCCACACTCCCTCCCACACCT	7	<nnxnp>	nn	123	np	133		
>ref-84485	ATTTATTACACACATCCTCCATTCTCT	22	<lmxll>	ll	134	lm	129		
>ref-84494	TAATTCGTTACCCTTTCTCCCGTGCAG	24	<lmxll>	ll	123	lm	145		
>ref-84523	GACGACCGCACAATCCCTCCTGTACTT	23	<nnxnp>	nn	137	np	136		
>ref-84537	AGACTATCCACTCTAGCTCCCCTGGCT	14	<nnxnp>	nn	136	np	134		
>ref-84537	AGACTATCCACTCTAGCTCCCCTGGCT	25	<hkxhk>	hh	0	hk	201	kk	62
>ref-84550	CACCATTCCACTCCTCCTCCACCCCTA	2	<nnxnp>	nn	254	np	0		

>ref-84557	ATCTTCAAAACATACTCTCCTTACTAT	12	<lmxll>	ll	112	lm	125		
>ref-84567	TCTCCCCTTACCTCTTCTCCCTTCCTT	4	<lmxll>	ll	6	lm	266		
>ref-84567	TCTCCCCTTACCTCTTCTCCCTTCCTT	7	<lmxll>	ll	6	lm	266		
>ref-84575	GCAACTACCACTGTTCCTCCTCTCATT	8	<lmxll>	ll	129	lm	137		
>ref-84579	CTTGTGGGCACCTGACCTCCTCCTTTA	27	<nnxnp>	nn	123	np	140		
>ref-84602	TCCCATGCCACACTCCCTCCTCTTCCT	16	<nnxnp>	nn	110	np	145		
>ref-84622	ACTCTTAGTACTAATACTCCTCTTCCC	4	<nnxnp>	nn	120	np	108		
>ref-84638	TATTTCAAGACGCAAACTCCATTCAGT	26	<hkxhk>	hh	0	hk	269	kk	0
>ref-84646	AGACAGAAAACCTTGACTCCGAGCTTA	2	<nnxnp>	nn	264	np	0		
>ref-84654	CTTCCATCTACCCTCTCTCCCTCCTAT	24	<lmxll>	ll	112	lm	144		
>ref-84673	ACCCCAGGCACCGTCTCTCCCCCAACG	7	<hkxhk>	hh	44	hk	137	kk	47
>ref-84677	TGCTGGGACACGCTGTCTCCTTAAGTT	26	<nnxnp>	nn	114	np	148		
>ref-84680	TCGTCTTAGACATAGACTCCCTGACTC	14	<lmxll>	ll	136	lm	132		
>ref-84680	TCGTCTTAGACATAGACTCCCTGACTC	23	<lmxll>	ll	136	lm	132		
>ref-84689	CCTCTGTCTACCCAATCTCCAACTCCT	2	<lmxll>	ll	132	lm	105		
>ref-84689	CCTCTGTCTACCCAATCTCCAACTCCT	3	<lmxll>	ll	117	lm	113		
>ref-84689	CCTCTGTCTACCCAATCTCCAACTCCT	15	<lmxll>	ll	137	lm	108		
>ref-84691	CTTCACAACACCCTCTCTCCCCCCACA	16	<lmxll>	ll	119	lm	143		
>ref-84696	CATCCATACACAGGTGCTCCCATAAAT	16	<hkxhk>	hh	4	hk	249	kk	0
>ref-84696	CATCCATACACAGGTGCTCCCATAAAT	22	<hkxhk>	hh	0	hk	249	kk	4
>ref-84698	CGCTGACCAACCAATCCTCCTCCGGGT	2	<nnxnp>	nn	262	np	0		
>ref-84719	AAGGAAGCGACTTCGACTCCGCACTCT	8	<nnxnp>	nn	126	np	119		
>ref-84730	ATGAACAACACCACTTCTCCTTTCCCA	25	<hkxhk>	hh	43	hk	139	kk	70
>ref-84738	CTGCCCTCCACCTCCCCTCCCACTCTA	15	<nnxnp>	nn	112	np	144		
>ref-84741	TATGTCTGAACACTCTCTCCCTCTCTC	8	<nnxnp>	nn	131	np	135		
>ref-84758	GGAAGGGGAACTCCCTCTCCCTGTCAC	2	<hkxhk>	hh	0	hk	159	kk	103
>ref-84758	GGAAGGGGAACTCCCTCTCCCTGTCAC	13	<hkxhk>	hh	125	hk	137	kk	0
>ref-84769	GAGAGAGAGACTGGCCCTCCAGAACCA	16	<lmxll>	ll	114	lm	142		
>ref-84806	TCTCTTTCTACTTTCTCTCCTTTTATT	4	<lmxll>	ll	139	lm	116		
>ref-84806	TCTCTTTCTACTTTCTCTCCTTTTATT	5	<lmxll>	ll	138	lm	116		
>ref-84806	TCTCTTTCTACTTTCTCTCCTTTTATT	13	<lmxll>	ll	9	lm	246		
>ref-84806	TCTCTTTCTACTTTCTCTCCTTTTATT	15	<lmxll>	ll	9	lm	246		
>ref-84810	CGTACCATCACTGTTACTCCTCCAAAG	14	<lmxll>	ll	269	lm	0		
>ref-84867	AATGACTGAACTATTTCTCCGCCGAAC	26	<nnxnp>	nn	119	np	142		
>ref-84887	GTAGCGGTAACTATTACTCCGGACCAC	23	<hkxhk>	hh	0	hk	264	kk	4
>ref-84887	GTAGCGGTAACTATTACTCCGGACCAC	27	<hkxhk>	hh	0	hk	264	kk	4
>ref-84908	CTTTCTCGTACAGATTCTCCTCCTCCT	7	<nnxnp>	nn	166	np	93		
>ref-84908	CTTTCTCGTACAGATTCTCCTCCTCCT	23	<hkxhk>	hh	0	hk	273	kk	0

>ref-84909	TTGCCTTACACCTACACTCCTTTCCTT	23	<lmxll>	ll	114	lm	131		
>ref-84909	TTGCCTTACACCTACACTCCTTTCCTT	25	<lmxll>	ll	114	lm	131		
>ref-84911	GTTGCAGTCACTACATCTCCCTGCCAT	27	<nnxnp>	nn	124	np	134		
>ref-84915	TCTTCCTTTACTCCATCTCCTCATTCG	5	<hkxhk>	hh	53	hk	112	kk	77
>ref-84933	CTGTGTGTTACCTAAGCTCCTTCCTTT	3	<lmxll>	ll	114	lm	135		
>ref-84944	AACTAAGTGACAGTGTCTCCTTGCGTG	5	<nnxnp>	nn	137	np	130		
>ref-84945	CGCCTCCACACTATCGCTCCCTCCGCA	21	<lmxll>	ll	129	lm	94		
>ref-84964	CTTCCTTCTACCCCATCTCCTCCTTCA	26	<lmxll>	ll	135	lm	130		
>ref-84977	CCAGCTTGGACGGAGACTCCTCAGCTT	14	<lmxll>	ll	80	lm	184		
>ref-85008	TTCCCCATCACCCCATCTCCTCCTCTT	1	<nnxnp>	nn	136	np	106		
>ref-85008	TTCCCCATCACCCCATCTCCTCCTCTT	2	<nnxnp>	nn	136	np	106		
>ref-85022	TTACCCCCAACCCCATCTCCTCCTCTT	7	<lmxll>	ll	14	lm	244		
>ref-85022	TTACCCCCAACCCCATCTCCTCCTCTT	24	<hkxhk>	hh	0	hk	260	kk	3
>ref-85028	CATCCTCCCACGCGTTCTCCGATGCAT	9	<nnxnp>	nn	147	np	124		
>ref-85033	ATTTCTCTAACTACCTCTCCCTCCCTT	1	<nnxnp>	nn	188	np	38		
>ref-85048	TCCGTTTACACCCCTCCTCCCCCTCCC	7	<nnxnp>	nn	128	np	132		
>ref-85052	CGACGACTGACTTCCCCTCCCTCTTCC	9	<nnxnp>	nn	270	np	0		
>ref-85056	CCCCCTGGCACCGAAGCTCCGTCCGTG	21	<nnxnp>	nn	130	np	125		
>ref-85065	GACCCCTTAACTTTATCTCCGTCTGGT	2	<hkxhk>	hh	65	hk	146	kk	47
>ref-85066	AATTTGTATACAATTCCTCCTGCACTC	13	<hkxhk>	hh	6	hk	257	kk	0
>ref-85087	CCCTCTTCCACTTCGCCTCCTCTTCTT	8	<lmxll>	ll	5	lm	267		
>ref-85087	CCCTCTTCCACTTCGCCTCCTCTTCTT	15	<lmxll>	ll	5	lm	267		
>ref-85087	CCCTCTTCCACTTCGCCTCCTCTTCTT	26	<hkxhk>	hh	3	hk	202	kk	65
>ref-85089	CAAACCAACACCAATTCTCCTTATCAT	8	<nnxnp>	nn	101	np	154		
>ref-85109	CTTTTCATTACTCCTCCTCCCCCCTTC	5	<hkxhk>	hh	0	hk	258	kk	4
>ref-85109	CTTTTCATTACTCCTCCTCCCCCCTTC	7	<hkxhk>	hh	4	hk	258	kk	0
>ref-85121	CCCCCAATAACCCTTCCTCCTCCACTC	23	<lmxll>	ll	106	lm	150		
>ref-85142	TATCTCTACACCTTATCTCCCTATCTT	27	<hkxhk>	hh	52	hk	160	kk	43
>ref-85160	TCGCTTTCCACTTTCGCTCCTGCCATC	22	<hkxhk>	hh	38	hk	139	kk	61
>ref-85166	CAACGCTATACCCTGCCTCCCCCTGTC	22	<lmxll>	ll	115	lm	147		
>ref-85176	AGCCCCCTCACTGGCTCTCCTAAGGTA	9	<lmxll>	ll	123	lm	136		
>ref-85176	AGCCCCCTCACTGGCTCTCCTAAGGTA	25	<nnxnp>	nn	132	np	127		
>ref-85199	TCCCCTCCCACCCCCTCTCCTCAGCTG	23	<lmxll>	ll	112	lm	127		
>ref-85228	GAGAGGGTAACAGCGGCTCCTCTCGTG	2	<lmxll>	ll	122	lm	145		
>ref-85230	TCAGCCACCACCAAAGCTCCTCTGCTT	12	<lmxll>	ll	133	lm	134		
>ref-85254	TTCCCTTTGACACCCTCTCCTCTTTGA	2	<hkxhk>	hh	2	hk	270	kk	0
>ref-85254	TTCCCTTTGACACCCTCTCCTCTTTGA	4	<hkxhk>	hh	3	hk	262	kk	0
>ref-85276	CTGGCCCTAACCCCACCTCCCTGCCCT	23	<lmxll>	ll	263	lm	0		

>ref-85277	CCTTAAACCACAATGTCTCCGTCATTA	3	<nnxnp>	nn	121	np	106		
>ref-85282	GACTCGTTAACAATAGCTCCAGCTAAC	8	<nnxnp>	nn	125	np	144		
>ref-85293	ATCTCAGTAACATTTGCTCCCCAACAG	1	<hkxhk>	hh	2	hk	258	kk	3
>ref-85312	TATTTATTTACTTTCTCTCCTTTTCTC	1	<hkxhk>	hh	0	hk	216	kk	22
>ref-85312	TATTTATTTACTTTCTCTCCTTTTCTC	4	<hkxhk>	hh	0	hk	168	kk	76
>ref-85312	TATTTATTTACTTTCTCTCCTTTTCTC	5	<hkxhk>	hh	0	hk	254	kk	1
>ref-85312	TATTTATTTACTTTCTCTCCTTTTCTC	7	<lmxll>	ll	186	lm	61		
>ref-85312	TATTTATTTACTTTCTCTCCTTTTCTC	13	<hkxhk>	hh	0	hk	256	kk	1
>ref-85324	TCCTGTCACACTCCCTCTCCCTCAGTG	4	<hkxhk>	hh	77	hk	137	kk	38
>ref-85353	AGCGCCCCGACTTTTCCTCCCGAATCC	5	<nnxnp>	nn	112	np	137		
>ref-85394	GCATCAGGAACAGCATCTCCGTCTGCC	1	<lmxll>	ll	148	lm	120		
>ref-85398	CCATCTCTAACTTCCCCTCCCTTCGTT	9	<hkxhk>	hh	69	hk	149	kk	51
>ref-85400	CTTACTCTCACGCTCCCTCCCGCCGTC	12	<nnxnp>	nn	121	np	144		
>ref-85405	TTCTTTCCGACATCTCCTCCGCTCTTT	6	<hkxhk>	hh	59	hk	137	kk	62
>ref-85425	CCTCTCTCAACCCTCTCTCCTTTACAC	2	<nnxnp>	nn	114	np	135		
>ref-85437	TCTTCCGACACCTGCGCTCCGACGACA	1	<nnxnp>	nn	271	np	0		
>ref-85437	TCTTCCGACACCTGCGCTCCGACGACA	2	<nnxnp>	nn	270	np	0		
>ref-85453	TCGTCCTGAACCCCCTCTCCCTCCCCC	4	<lmxll>	ll	0	lm	258		
>ref-85494	GTATCCTGGACTCCTGCTCCTTCGATT	23	<hkxhk>	hh	1	hk	272	kk	0
>ref-85506	CCTCTTCTCACTTTTCCTCCTCTCCAT	1	<lmxll>	ll	117	lm	142		
>ref-85511	CGCTCCCTGACACCCGCTCCCTGACAC	2	<hkxhk>	hh	0	hk	273	kk	0
>ref-85511	CGCTCCCTGACACCCGCTCCCTGACAC	7	<hkxhk>	hh	0	hk	273	kk	0
>ref-85511	CGCTCCCTGACACCCGCTCCCTGACAC	16	<hkxhk>	hh	0	hk	273	kk	0
>ref-85511	CGCTCCCTGACACCCGCTCCCTGACAC	21	<hkxhk>	hh	0	hk	273	kk	0
>ref-85544	CCCCATCTTACCCCTACTCCCACGCCC	25	<hkxhk>	hh	0	hk	170	kk	57
>ref-85548	ACTGCCTCTACTCCTCCTCCCCCTCTT	8	<nnxnp>	nn	137	np	127		
>ref-85552	CCACAATATACTCTCTCTCCCACAACA	26	<nnxnp>	nn	232	np	33		
>ref-85580	GGGGGTGATACCCTCACTCCCTTTAAA	26	<lmxll>	ll	117	lm	103		
>ref-85610	TTTTTCTTTACGCATTCTCCATTTCCT	7	<nnxnp>	nn	132	np	127		
>ref-85610	TTTTTCTTTACGCATTCTCCATTTCCT	8	<lmxll>	ll	108	lm	129		
>ref-85619	CCCTCTTCTACCGTTCCTCCCCATCCT	7	<nnxnp>	nn	101	np	145		
>ref-85620	TCCTTATCCACCCTTCCTCCTTCCTCT	3	<hkxhk>	hh	67	hk	199	kk	0
>ref-85620	TCCTTATCCACCCTTCCTCCTTCCTCT	23	<hkxhk>	hh	66	hk	199	kk	0
>ref-85636	TTCCCCCGCACCCTCACTCCTGACCAG	1	<nnxnp>	nn	142	np	118		
>ref-85648	CATGATGCAACTCTCACTCCACATACT	2	<nnxnp>	nn	118	np	145		
>ref-85667	CCTCCACACACACACCCTCCCTCCTCA	2	<nnxnp>	nn	264	np	0		
>ref-85667	CCTCCACACACACACCCTCCCTCCTCA	5	<hkxhk>	hh	35	hk	225	kk	0
>ref-85667	CCTCCACACACACACCCTCCCTCCTCA	14	<lmxll>	ll	4	lm	266		

>ref-85667	CCTCCACACACACACCCTCCCTCCTCA	23	<lmxll>	ll	52	lm	200		
>ref-85684	AGGCATGGAACTTTTCCTCCGCACATT	25	<lmxll>	ll	122	lm	147		
>ref-85686	TCCCTCTCTACCCAGCCTCCGCCCCTG	14	<nnxnp>	nn	136	np	133		
>ref-85686	TCCCTCTCTACCCAGCCTCCGCCCCTG	21	<nnxnp>	nn	136	np	133		
>ref-85688	AACAGCAAAACAAACTCTCCACGAACA	5	<lmxll>	ll	131	lm	129		
>ref-85715	AATATGACAACGCCCCCTCCCCACCCT	25	<nnxnp>	nn	135	np	130		
>ref-85719	ACTGACACTACTACTACTCCTTTAATA	26	<lmxll>	ll	113	lm	119		
>ref-85729	GTAGGCTTTACTCACTCTCCGTTTCCT	8	<hkxhk>	hh	42	hk	160	kk	50
>ref-85791	CTTCTCTTGACTCTTTCTCCCCCTTCC	9	<lmxll>	ll	129	lm	118		
>ref-85823	CACACACACACTCTTCCTCCAGCGAGA	3	<lmxll>	ll	148	lm	123		
>ref-85823	CACACACACACTCTTCCTCCAGCGAGA	21	<lmxll>	ll	149	lm	122		
>ref-85826	AGAGGAACCACCGCCTCTCCGACAGGC	12	<nnxnp>	nn	129	np	138		
>ref-85827	TTCTGACACACTCTTTCTCCCTTAAAA	12	<nnxnp>	nn	85	np	136		
>ref-85833	CCTTCCCCTACCCTACCTCCAATGAAT	2	<lmxll>	ll	132	lm	130		
>ref-85858	CAGCCAACAACTGATACTCCCAGCATC	3	<lmxll>	ll	104	lm	121		
>ref-85861	CCACCTTATACTGGCTCTCCTTCCTCC	4	<nnxnp>	nn	127	np	117		
>ref-85867	CTTACCTACACCTTTTCTCCTCTCATT	26	<nnxnp>	nn	142	np	114		
>ref-85868	TTCTCTCGCACCTTCCCTCCAACTCCA	5	<nnxnp>	nn	137	np	122		
>ref-85868	TTCTCTCGCACCTTCCCTCCAACTCCA	26	<nnxnp>	nn	133	np	123		
>ref-85869	GAGCCTCCCACGCGCCCTCCCTTTGCA	3	<nnxnp>	nn	131	np	141		
>ref-85869	GAGCCTCCCACGCGCCCTCCCTTTGCA	25	<lmxll>	ll	145	lm	125		
>ref-85871	TCTGTCCATACAGTCCCTCCATGGCTT	8	<hkxhk>	hh	37	hk	215	kk	0
>ref-85871	TCTGTCCATACAGTCCCTCCATGGCTT	12	<hkxhk>	hh	37	hk	215	kk	0
>ref-85871	TCTGTCCATACAGTCCCTCCATGGCTT	25	<hkxhk>	hh	2	hk	269	kk	0
>ref-85887	TACAAACCAACCAAAGCTCCTCTTTAA	2	<hkxhk>	hh	43	hk	217	kk	0
>ref-85887	TACAAACCAACCAAAGCTCCTCTTTAA	7	<hkxhk>	hh	0	hk	273	kk	0
>ref-85887	TACAAACCAACCAAAGCTCCTCTTTAA	8	<lmxll>	ll	37	lm	218		
>ref-85913	TCTATGATAACCCTTTCTCCCTCCATC	6	<hkxhk>	hh	27	hk	227	kk	1
>ref-85913	TCTATGATAACCCTTTCTCCCTCCATC	9	<hkxhk>	hh	27	hk	227	kk	1
>ref-85942	CTATTCGCTACGCACCCTCCCTTACTT	21	<nnxnp>	nn	119	np	146		
>ref-85942	CTATTCGCTACGCACCCTCCCTTACTT	27	<nnxnp>	nn	119	np	146		
>ref-85947	TCTTTTCCCACTTCTGCTCCTTCCCTT	16	<hkxhk>	hh	56	hk	125	kk	54
>ref-85950	ACTCTGCTTACTCCTTCTCCCTCATTT	15	<nnxnp>	nn	129	np	142		
>ref-85953	CACACACAAACACTGCCTCCTCTGCCC	2	<lmxll>	ll	151	lm	112		
>ref-85953	CACACACAAACACTGCCTCCTCTGCCC	24	<nnxnp>	nn	128	np	140		
>ref-85957	CAGGCTGTCACGAGGACTCCGGTGATG	26	<nnxnp>	nn	143	np	129		
>ref-85959	GAACATGTCACTCACACTCCGTAGATA	21	<hkxhk>	hh	86	hk	129	kk	46
>ref-85999	CACTTACCTACGCACCCTCCCACCCAG	21	<lmxll>	ll	112	lm	123		

>ref-86001	TACGCTCTCACACTTTCTCCTGTCAAC	4	<lmxll>	ll	81	lm	164		
>ref-86001	TACGCTCTCACACTTTCTCCTGTCAAC	27	<lmxll>	ll	81	lm	165		
>ref-86044	GACATACCCACACCACCTCCTTCCCTA	14	<lmxll>	ll	122	lm	119		
>ref-86048	GACGTCTGTACTCCCTCTCCCTCTCTC	26	<lmxll>	ll	139	lm	120		
>ref-86056	CAAGAGCCCACTTGACCTCCTTCAACC	6	<hkxhk>	hh	70	hk	132	kk	55
>ref-86071	GAGAGAGAGACTCACTCTCCCCAGCGC	9	<lmxll>	ll	0	lm	262		
>ref-86071	GAGAGAGAGACTCACTCTCCCCAGCGC	14	<lmxll>	ll	0	lm	262		
>ref-86077	TCCTCCTCAACATCATCTCCTTTCCAT	1	<hkxhk>	hh	46	hk	149	kk	67
>ref-86089	ACATCCGCGACTGTTTCTCCCTCGGTG	21	<nnxnp>	nn	112	np	150		
>ref-86091	CACCCCAACACCTTCCCTCCCCCTCCT	22	<nnxnp>	nn	55	np	210		
>ref-86091	CACCCCAACACCTTCCCTCCCCCTCCT	24	<nnxnp>	nn	56	np	210		
>ref-86132	CCAATCCCAACATTAGCTCCATCACCC	27	<nnxnp>	nn	106	np	127		
>ref-86156	CGGTGCTGCACTGGCCCTCCGCCGAGT	2	<lmxll>	ll	155	lm	117		
>ref-86166	ACGGGTGCCACTGTCCCTCCCCCAACC	2	<nnxnp>	nn	262	np	6		
>ref-86166	ACGGGTGCCACTGTCCCTCCCCCAACC	3	<hkxhk>	hh	241	hk	27	kk	0
>ref-86166	ACGGGTGCCACTGTCCCTCCCCCAACC	15	<nnxnp>	nn	262	np	7		
>ref-86176	TTCTTGTCAACATTCTCTCCTTGTATC	1	<hkxhk>	hh	0	hk	248	kk	14
>ref-86176	TTCTTGTCAACATTCTCTCCTTGTATC	5	<nnxnp>	nn	227	np	27		
>ref-86176	TTCTTGTCAACATTCTCTCCTTGTATC	16	<hkxhk>	hh	0	hk	24	kk	232
>ref-86176	TTCTTGTCAACATTCTCTCCTTGTATC	21	<hkxhk>	hh	0	hk	267	kk	2
>ref-86176	TTCTTGTCAACATTCTCTCCTTGTATC	26	<nnxnp>	nn	226	np	28		
>ref-86177	TCAGCGAGGACCCACCCTCCCTGACTG	3	<lmxll>	ll	262	lm	11		
>ref-86177	TCAGCGAGGACCCACCCTCCCTGACTG	5	<hkxhk>	hh	212	hk	52	kk	0
>ref-86177	TCAGCGAGGACCCACCCTCCCTGACTG	6	<lmxll>	ll	71	lm	194		
>ref-86177	TCAGCGAGGACCCACCCTCCCTGACTG	26	<hkxhk>	hh	0	hk	175	kk	78
>ref-86186	CCTATCCCAACACTCACTCCTTCCCTC	14	<hkxhk>	hh	0	hk	270	kk	1
>ref-86204	GAAGTCATTACGATTACTCCGTACACC	22	<nnxnp>	nn	131	np	125		
>ref-86220	CAAAGACAGACACCGCCTCCCACCCAC	13	<lmxll>	ll	114	lm	156		
>ref-86235	ATGGATTAAACCAGCACTCCATCGGAT	16	<lmxll>	ll	141	lm	128		
>ref-86236	ATAACGTTGACGCAGACTCCTATCGTA	23	<hkxhk>	hh	0	hk	221	kk	43
>ref-86238	TAACGACATACGCACCCTCCAGCAAAC	16	<nnxnp>	nn	129	np	138		
>ref-86249	TTTAAAACAACTTCAGCTCCAATGTAT	9	<lmxll>	ll	1	lm	259		
>ref-86254	CACAATTCAACCCCCCCTCCCTTCCCC	3	<hkxhk>	hh	8	hk	263	kk	0
>ref-86254	CACAATTCAACCCCCCCTCCCTTCCCC	15	<lmxll>	ll	193	lm	70		
>ref-86254	CACAATTCAACCCCCCCTCCCTTCCCC	16	<hkxhk>	hh	8	hk	263	kk	0
>ref-86254	CACAATTCAACCCCCCCTCCCTTCCCC	21	<hkxhk>	hh	0	hk	220	kk	37
>ref-86254	CACAATTCAACCCCCCCTCCCTTCCCC	23	<hkxhk>	hh	0	hk	220	kk	37
>ref-86267	TCACCCTTCACCCCCCCTCCCTTCACA	23	<nnxnp>	nn	137	np	124		

>ref-86275	TGCAAAGTGACACCTACTCCTTGCTAT	27	<nnxnp>	nn	268	np	0		
>ref-86307	CTTGTTGACACCCTTCCTCCCGCTTTC	5	<lmxll>	ll	119	lm	130		
>ref-86315	CTTTCCTCTACCGTCCCTCCCTCTTTC	2	<hkxhk>	hh	0	hk	271	kk	0
>ref-86315	CTTTCCTCTACCGTCCCTCCCTCTTTC	13	<hkxhk>	hh	1	hk	271	kk	0
>ref-86327	CTGTAAGTGACCTTCCCTCCTCTTCCC	5	<nnxnp>	nn	273	np	0		
>ref-86344	AGATGTGGCACTCGCTCTCCCCTGGCA	9	<lmxll>	ll	153	lm	114		
>ref-86359	ATTCCCCCTACCTTCCCTCCTTCCCAC	9	<nnxnp>	nn	105	np	136		
>ref-86372	GCGTCACAAACTTAGGCTCCACACTCT	23	<hkxhk>	hh	55	hk	131	kk	69
>ref-86375	GCTCAGCGGACCTTAGCTCCATTAAAT	6	<lmxll>	ll	116	lm	149		
>ref-86405	ATTTCTCGTACCATATCTCCATTCCTC	13	<lmxll>	ll	113	lm	129		
>ref-86418	CACACACAGACTCTTTCTCCTCTCCCT	2	<lmxll>	ll	7	lm	262		
>ref-86418	CACACACAGACTCTTTCTCCTCTCCCT	3	<hkxhk>	hh	0	hk	272	kk	0
>ref-86418	CACACACAGACTCTTTCTCCTCTCCCT	27	<lmxll>	ll	7	lm	262		
>ref-86431	ATCCCCCCTACCCCCGCTCCCTATCCC	12	<hkxhk>	hh	57	hk	153	kk	32
>ref-86456	CAGCTCATCACGCTCGCTCCGGGGTAC	25	<lmxll>	ll	132	lm	132		
>ref-86457	GCGAGCACGACCCTGTCTCCAGGCACA	3	<hkxhk>	hh	0	hk	273	kk	0
>ref-86468	TCCTTTCCCACGTCCTCTCCTTTTCAT	8	<hkxhk>	hh	48	hk	133	kk	64
>ref-86490	CTTCCCATAACACCCTCTCCCCTACCT	3	<hkxhk>	hh	0	hk	179	kk	87
>ref-86490	CTTCCCATAACACCCTCTCCCCTACCT	5	<hkxhk>	hh	87	hk	179	kk	0
>ref-86492	ATCCAGGGCACGGCGACTCCTCTCCTA	5	<lmxll>	ll	143	lm	127		
>ref-86499	CATACTATAACCTCTCCTCCTCTCCCT	8	<lmxll>	ll	0	lm	249		
>ref-86499	CATACTATAACCTCTCCTCCTCTCCCT	9	<lmxll>	ll	0	lm	249		
>ref-86521	ACTCCTGATACTATACCTCCTTCCCCT	1	<lmxll>	ll	4	lm	264		
>ref-86521	ACTCCTGATACTATACCTCCTTCCCCT	9	<lmxll>	ll	4	lm	264		
>ref-86533	CTCTCCTTCACTCCTTCTCCCTCTTAA	1	<nnxnp>	nn	259	np	0		
>ref-86533	CTCTCCTTCACTCCTTCTCCCTCTTAA	5	<lmxll>	ll	115	lm	129		
>ref-86535	AACATCATAACACAGTCTCCCGTACCA	7	<nnxnp>	nn	118	np	145		
>ref-86538	CAATCCCTCACTCCTCCTCCTTCATTC	24	<lmxll>	ll	57	lm	200		
>ref-86544	CGATCTCTTACGTAGCCTCCGCCAGCC	8	<lmxll>	ll	101	lm	121		
>ref-86570	GCATACGTTACGTAACCTCCTTCCCCA	26	<lmxll>	ll	134	lm	132		
>ref-86584	GGCGGTTGAACCGTGGCTCCGGGCGAC	2	<hkxhk>	hh	59	hk	136	kk	63
>ref-86648	TTCTATACCACGTTATCTCCTTTTCAT	5	<lmxll>	ll	136	lm	111		
>ref-86662	CTTTCCTTAACGCTTTCTCCCCCGCCC	14	<hkxhk>	hh	43	hk	129	kk	62
>ref-86664	CCAACTCAAACACCACCTCCACCCTCA	7	<lmxll>	ll	130	lm	135		
>ref-86665	TTTATCGTAACCCCTCCTCCTTCCCTC	16	<lmxll>	ll	119	lm	123		
>ref-86666	CATAGCATTACAAACACTCCAACAGAC	1	<nnxnp>	nn	108	np	114		
>ref-86671	CCTCGACCAACACCCGCTCCAACTACA	27	<hkxhk>	hh	0	hk	271	kk	0
>ref-86693	TGAGTTCGGACGTGGGCTCCTCGCAGC	1	<hkxhk>	hh	0	hk	125	kk	126

>ref-86693	TGAGTTCGGACGTGGGCTCCTCGCAGC	7	<hkxhk>	hh	9	hk	261	kk	0
>ref-86693	TGAGTTCGGACGTGGGCTCCTCGCAGC	16	<hkxhk>	hh	0	hk	262	kk	11
>ref-86693	TGAGTTCGGACGTGGGCTCCTCGCAGC	22	<hkxhk>	hh	107	hk	152	kk	0
>ref-86693	TGAGTTCGGACGTGGGCTCCTCGCAGC	26	<hkxhk>	hh	0	hk	148	kk	108
>ref-86695	CAGATAAAGACAGATTCTCCTGAGTTT	3	<nnxnp>	nn	124	np	121		
>ref-86707	AACACAAGGACGTCGGCTCCACATTCT	26	<lmxll>	ll	166	lm	84		
>ref-86707	AACACAAGGACGTCGGCTCCACATTCT	27	<lmxll>	ll	166	lm	84		
>ref-86709	CTTCTTTTCACTTCTGCTCCTCCTTCT	2	<lmxll>	ll	2	lm	262		
>ref-86709	CTTCTTTTCACTTCTGCTCCTCCTTCT	22	<lmxll>	ll	2	lm	262		
>ref-86726	TCCAATTCCACTTCTTCTCCTTCGTCT	8	<nnxnp>	nn	86	np	133		
>ref-86738	CTCTTCATGACTTCCTCTCCACTCTTC	9	<lmxll>	ll	136	lm	127		
>ref-86738	CTCTTCATGACTTCCTCTCCACTCTTC	12	<lmxll>	ll	137	lm	127		
>ref-86746	GGCCGCGGCACCAGCGCTCCAAGACCT	5	<lmxll>	ll	117	lm	142		
>ref-86747	TCTCTCAGAACTCCCCCTCCCATCTCC	24	<nnxnp>	nn	119	np	148		
>ref-86749	TCACCTTTCACAGTCCCTCCTCCACTT	7	<lmxll>	ll	162	lm	99		
>ref-86749	TCACCTTTCACAGTCCCTCCTCCACTT	14	<hkxhk>	hh	0	hk	273	kk	0
>ref-86749	TCACCTTTCACAGTCCCTCCTCCACTT	24	<hkxhk>	hh	0	hk	273	kk	0
>ref-86770	CCTGAACCGACCTGCCCTCCGACCGTT	13	<nnxnp>	nn	219	np	0		
>ref-86781	ACTCCCCCTACTTCCCCTCCCTAATTT	4	<nnxnp>	nn	241	np	0		
>ref-86781	ACTCCCCCTACTTCCCCTCCCTAATTT	5	<nnxnp>	nn	239	np	0		
>ref-86788	ATCTCTACCACTCCCACTCCCCCCTCC	2	<lmxll>	ll	258	lm	0		
>ref-86789	CTACCAGGTACAGCTGCTCCAAATACT	12	<nnxnp>	nn	171	np	90		
>ref-86789	CTACCAGGTACAGCTGCTCCAAATACT	14	<hkxhk>	hh	0	hk	220	kk	41
>ref-86789	CTACCAGGTACAGCTGCTCCAAATACT	16	<hkxhk>	hh	0	hk	270	kk	1
>ref-86789	CTACCAGGTACAGCTGCTCCAAATACT	27	<hkxhk>	hh	0	hk	270	kk	1
>ref-86805	CAGATCCACACCGTCTCTCCCTGGATC	3	<lmxll>	ll	143	lm	123		
>ref-86805	CAGATCCACACCGTCTCTCCCTGGATC	24	<lmxll>	ll	143	lm	123		
>ref-86823	ATTACCCCAACGGCACCTCCTACCCTT	12	<hkxhk>	hh	64	hk	139	kk	50
>ref-86827	CCTCTTCCTACTCTTCCTCCAGTCACT	5	<hkxhk>	hh	43	hk	152	kk	48
>ref-86842	TCATACAACACTGTCACTCCCTGCCAT	23	<nnxnp>	nn	117	np	148		
>ref-86855	GATGGCTCCACGTGTGCTCCGCAGCAA	1	<hkxhk>	hh	0	hk	268	kk	3
>ref-86863	TTCTCTTATACACCCACTCCCACTCTC	4	<hkxhk>	hh	11	hk	248	kk	3
>ref-86863	TTCTCTTATACACCCACTCCCACTCTC	22	<hkxhk>	hh	3	hk	248	kk	11
>ref-86865	CCTCCGTTAACTCCCCCTCCTTCTTCT	2	<nnxnp>	nn	134	np	132		
>ref-86865	CCTCCGTTAACTCCCCCTCCTTCTTCT	3	<nnxnp>	nn	134	np	132		
>ref-86868	ATCGGCCTTACCCTTCCTCCCCTTATA	9	<lmxll>	ll	133	lm	134		
>ref-86891	ACGAGTACGACAGGTCCTCCCGTACAG	26	<nnxnp>	nn	240	np	0		
>ref-86898	TGGTGCACAACCTCCCCTCCCACACAT	24	<hkxhk>	hh	59	hk	140	kk	70

>ref-86901	ATGAATATCACTCGTCCTCCTTTCCTT	21	<hkxhk>	hh	65	hk	146	kk	54
>ref-86904	GCTTACTCTACCTCATCTCCTCCATAA	24	<hkxhk>	hh	3	hk	267	kk	1
>ref-86934	ACCTCACTCACCCTTCCTCCTCCTTCC	7	<hkxhk>	hh	29	hk	218	kk	6
>ref-86951	CTTACCATCACTTTGCCTCCCCCCGAA	1	<nnxnp>	nn	266	np	0		
>ref-87005	CGCTCCCTCACTCCCCCTCCTACTCCC	6	<nnxnp>	nn	265	np	3		
>ref-87006	CCCTCGCTCACTCTTCCTCCTTCCTCA	6	<lmxll>	ll	21	lm	210		
>ref-87006	CCCTCGCTCACTCTTCCTCCTTCCTCA	25	<lmxll>	ll	262	lm	0		
>ref-87008	TCGGACTTTACCTGTCCTCCGTTTGTT	9	<hkxhk>	hh	61	hk	142	kk	60
>ref-87011	TTGTATAAGACCTTCTCTCCTTCTCCT	26	<nnxnp>	nn	111	np	148		
>ref-87026	TTTACCCTTACCCTACCTCCCTCTCTT	26	<nnxnp>	nn	134	np	118		
>ref-87041	ACCATCCACACTGATCCTCCAGCTGCC	22	<nnxnp>	nn	116	np	112		
>ref-87053	TCCCTCTTCACATTTTCTCCCTCATTT	24	<hkxhk>	hh	169	hk	81	kk	0
>ref-87058	GCCAGAAGTACCTGCCCTCCCCCTACT	27	<nnxnp>	nn	136	np	111		
>ref-87069	CCGATTCCTACGTTACCTCCGAATCCA	7	<lmxll>	ll	147	lm	97		
>ref-87083	ATATATACCACATATGCTCCTTTAGGT	16	<nnxnp>	nn	119	np	101		
>ref-87090	CCCTGTCCTACCCAGCCTCCTACCGTA	5	<hkxhk>	hh	57	hk	210	kk	0
>ref-87093	TCTCTACTTACTCCTCCTCCTAATCCT	12	<nnxnp>	nn	226	np	0		
>ref-87102	TCAAACCAGACTGTTTCTCCACATTGA	9	<lmxll>	ll	240	lm	0		
>ref-87111	TTCCTCCTCACCTTCCCTCCTGTTCTT	24	<lmxll>	ll	122	lm	140		
>ref-87158	TCTCTCATCACCCTTCCTCCTTCCCCT	2	<lmxll>	ll	131	lm	138		
>ref-87166	GAATGTCTCACTCGGTCTCCTTGATTG	1	<lmxll>	ll	125	lm	140		
>ref-87196	TGTATTCCCACGTTCTCTCCTTAGTTC	5	<lmxll>	ll	127	lm	135		
>ref-87201	CACATACACACCACAGCTCCTTTCCTA	5	<lmxll>	ll	117	lm	140		
>ref-87201	CACATACACACCACAGCTCCTTTCCTA	22	<hkxhk>	hh	52	hk	143	kk	60
>ref-87201	CACATACACACCACAGCTCCTTTCCTA	23	<nnxnp>	nn	141	np	117		
>ref-87233	CTTTATCCCACGCGCCCTCCCTCCTTC	8	<hkxhk>	hh	50	hk	140	kk	60
>ref-87233	CTTTATCCCACGCGCCCTCCCTCCTTC	13	<lmxll>	ll	118	lm	131		
>ref-87259	TTCCCCTCAACCCCCCCTCCCCCCATC	14	<nnxnp>	nn	153	np	85		
>ref-87277	CCAGTTGCAACGTTGCCTCCCTCCCCT	2	<nnxnp>	nn	123	np	138		
>ref-87298	TCTGAATGTACGTTCGCTCCTCCTCCT	12	<nnxnp>	nn	151	np	116		
>ref-87324	CGTGTCGGAACTCCTCCTCCTTTTCTT	26	<nnxnp>	nn	268	np	0		
>ref-87328	CCCTATTCCACTTCCCCTCCCATTCCC	1	<lmxll>	ll	242	lm	0		
>ref-87328	CCCTATTCCACTTCCCCTCCCATTCCC	22	<lmxll>	ll	211	lm	13		
>ref-87337	TCTTATTGGACAGCATCTCCTTGTGTA	7	<nnxnp>	nn	141	np	122		
>ref-87341	CTTTACCCTACTCTATCTCCTGGTATT	25	<nnxnp>	nn	80	np	157		
>ref-87352	TCTCCTCCAACCCCATCTCCACATCCA	24	<hkxhk>	hh	71	hk	186	kk	2
>ref-87352	TCTCCTCCAACCCCATCTCCACATCCA	25	<hkxhk>	hh	71	hk	186	kk	2
>ref-87360	ACGCTCTCTACTTCTTCTCCCTCCCCC	4	<lmxll>	ll	261	lm	1		

>ref-87428	GTTTCTCCGACCTCAACTCCTCATAAA	7	<lmxll>	ll	106	lm	135		
>ref-87464	ACAATCAAGACTTTTCCTCCACCAAGT	8	<hkxhk>	hh	43	hk	139	kk	61
>ref-87465	CTTCCCTCCACCCCAACTCCCACTCCC	1	<lmxll>	ll	247	lm	0		
>ref-87470	TCTTCTCCTACCTCCCCTCCCTTCCCC	1	<nnxnp>	nn	162	np	78		
>ref-87470	TCTTCTCCTACCTCCCCTCCCTTCCCC	6	<lmxll>	ll	67	lm	168		
>ref-87470	TCTTCTCCTACCTCCCCTCCCTTCCCC	9	<nnxnp>	nn	269	np	0		
>ref-87470	TCTTCTCCTACCTCCCCTCCCTTCCCC	12	<nnxnp>	nn	269	np	0		
>ref-87470	TCTTCTCCTACCTCCCCTCCCTTCCCC	22	<lmxll>	ll	10	lm	254		
>ref-87470	TCTTCTCCTACCTCCCCTCCCTTCCCC	26	<nnxnp>	nn	269	np	0		
>ref-87475	CACGCCCAAACACCTGCTCCCGCCGCC	8	<lmxll>	ll	132	lm	141		
>ref-87481	ATATGACATACCGAATCTCCCGATCAC	8	<lmxll>	ll	128	lm	117		
>ref-87495	ATCCTCCCCACTATCCCTCCTCCCAAC	1	<hkxhk>	hh	63	hk	119	kk	42
>ref-87510	GAATGAGATACTTGAGCTCCTAGTTGT	5	<hkxhk>	hh	39	hk	145	kk	57
>ref-87521	CTGAACACCACGGCATCTCCTCCTCCT	12	<lmxll>	ll	136	lm	133		
>ref-87524	CCACATCGCACCTTCACTCCCCAAGGC	5	<lmxll>	ll	29	lm	223		
>ref-87524	CCACATCGCACCTTCACTCCCCAAGGC	7	<nnxnp>	nn	52	np	203		
>ref-87528	CACCTATCCACCCATTCTCCCACATAT	12	<nnxnp>	nn	116	np	148		
>ref-87543	AATTTGTTTACTCTGACTCCTGACTGC	1	<lmxll>	ll	127	lm	125		
>ref-87588	CCCTCTTTTACTGCCTCTCCCCTTCCT	16	<lmxll>	ll	114	lm	136		
>ref-87592	CAACCACCAACCCTTTCTCCCAACCCA	27	<nnxnp>	nn	106	np	134		
>ref-87594	GCTAACTGGACCCACTCTCCTCACGTG	24	<hkxhk>	hh	61	hk	139	kk	65
>ref-87620	GAGGGAATGACTGATGCTCCTTCTATC	9	<lmxll>	ll	133	lm	134		
>ref-87621	ACCGAAGACACATGAACTCCACTTTGT	13	<lmxll>	ll	120	lm	130		
>ref-87624	CCTCCCCTCACTTCTCCTCCTCTACTC	15	<lmxll>	ll	6	lm	263		
>ref-87624	CCTCCCCTCACTTCTCCTCCTCTACTC	24	<lmxll>	ll	5	lm	263		
>ref-87624	CCTCCCCTCACTTCTCCTCCTCTACTC	25	<lmxll>	ll	146	lm	121		
>ref-87635	TCCATTTCGACCGGATCTCCCTCCCCA	5	<lmxll>	ll	115	lm	152		
>ref-87636	CCTTTCCTGACTCCCTCTCCCCCCTTC	9	<nnxnp>	nn	122	np	120		
>ref-87636	CCTTTCCTGACTCCCTCTCCCCCCTTC	13	<nnxnp>	nn	128	np	117		
>ref-87636	CCTTTCCTGACTCCCTCTCCCCCCTTC	16	<hkxhk>	hh	19	hk	154	kk	68
>ref-87651	CCTTCATTAACACCATCTCCCTCCTTC	16	<nnxnp>	nn	112	np	136		
>ref-87653	CCTTATTCCACCCTCTCTCCACCCCAT	1	<nnxnp>	nn	264	np	0		
>ref-87653	CCTTATTCCACCCTCTCTCCACCCCAT	4	<hkxhk>	hh	7	hk	247	kk	0
>ref-87663	CCCATTTCAACCCCTCCTCCTCTCCTC	16	<lmxll>	ll	7	lm	261		
>ref-87663	CCCATTTCAACCCCTCCTCCTCTCCTC	22	<lmxll>	ll	7	lm	261		
>ref-87675	AGCACCCCCACCTCCACTCCGCACGCA	1	<hkxhk>	hh	1	hk	268	kk	0
>ref-87675	AGCACCCCCACCTCCACTCCGCACGCA	6	<hkxhk>	hh	0	hk	263	kk	3
>ref-87675	AGCACCCCCACCTCCACTCCGCACGCA	8	<hkxhk>	hh	93	hk	168	kk	0

>ref-87678	CCTCCGTCAACTCTTTCTCCTGCTTCT	12	<lmxll>	ll	5	lm	265		
>ref-87741	ACACCGCCCACACCCTCTCCCTCCCTA	3	<hkxhk>	hh	0	hk	273	kk	0
>ref-87741	ACACCGCCCACACCCTCTCCCTCCCTA	15	<hkxhk>	hh	0	hk	273	kk	0
>ref-87770	ATCGCCTCCACAATATCTCCTTCAAGA	6	<lmxll>	ll	127	lm	130		
>ref-87775	TTTTGTCGCACCCTCTCTCCAGTCTGC	27	<nnxnp>	nn	114	np	140		
>ref-87777	CCCTCCTCCACGCTCACTCCCTTCCCC	9	<lmxll>	ll	1	lm	255		
>ref-87777	CCCTCCTCCACGCTCACTCCCTTCCCC	16	<lmxll>	ll	2	lm	266		
>ref-87811	TCCTCTCCTACTTCCCCTCCTCGCCCA	4	<nnxnp>	nn	4	np	262		
>ref-87811	TCCTCTCCTACTTCCCCTCCTCGCCCA	12	<nnxnp>	nn	67	np	189		
>ref-87811	TCCTCTCCTACTTCCCCTCCTCGCCCA	13	<lmxll>	ll	20	lm	243		
>ref-87811	TCCTCTCCTACTTCCCCTCCTCGCCCA	26	<nnxnp>	nn	134	np	122		
>ref-87828	TGCATTCCCACTCACCCTCCACATGCC	7	<lmxll>	ll	120	lm	147		
>ref-87840	CCTCCTCGCACATGCTCTCCCTCCCAT	14	<nnxnp>	nn	153	np	91		
>ref-87857	TTTACCCCAACCCTCCCTCCTCCTTTA	6	<nnxnp>	nn	247	np	9		
>ref-87863	CCACCGCAGACTCTCCCTCCGTTTTTC	21	<lmxll>	ll	156	lm	102		
>ref-87872	TTTCTCTCCACTTCTCCTCCCTTCCTC	25	<nnxnp>	nn	66	np	191		
>ref-87886	ACTGGAAGCACACCACCTCCATTGCAT	13	<lmxll>	ll	127	lm	142		
>ref-87889	ACCAACAAAACGATTCCTCCTTTTGTG	1	<lmxll>	ll	130	lm	129		
>ref-87904	TTATATCTTACCTCCTCTCCTTTCCCT	27	<nnxnp>	nn	120	np	140		
>ref-87926	CACACACCCACACCATCTCCGTCACCC	6	<lmxll>	ll	168	lm	69		
>ref-87926	CACACACCCACACCATCTCCGTCACCC	22	<lmxll>	ll	168	lm	69		
>ref-87928	CCTGATTTGACTCTCTCTCCTTTTCCT	16	<hkxhk>	hh	45	hk	139	kk	68
>ref-87931	ACTCGATTTACCTTATCTCCATTAACA	14	<lmxll>	ll	112	lm	137		
>ref-87943	TTCTCCATTACTGTTCCTCCAGTGTGT	27	<lmxll>	ll	151	lm	106		
>ref-87970	CAAGGTTACACTTCCTCTCCTCGTATG	23	<nnxnp>	nn	119	np	129		
>ref-88007	AAATATGAGACTTGTCCTCCGCCCTAA	14	<hkxhk>	hh	64	hk	136	kk	63
>ref-88025	AATAACCTCACGCCCACTCCACCGACT	5	<nnxnp>	nn	59	np	194		
>ref-88025	AATAACCTCACGCCCACTCCACCGACT	23	<nnxnp>	nn	59	np	194		
>ref-88041	GAGCTGTTCACGGCAGCTCCGAAGAAA	6	<lmxll>	ll	116	lm	145		
>ref-88047	CCCCTCTACACCCCCTCTCCACCCCTC	8	<nnxnp>	nn	177	np	69		
>ref-88047	CCCCTCTACACCCCCTCTCCACCCCTC	27	<nnxnp>	nn	209	np	45		
>ref-88060	TCCTCTTCTACCATTCCTCCCCCACTC	13	<hkxhk>	hh	71	hk	136	kk	57
>ref-88060	TCCTCTTCTACCATTCCTCCCCCACTC	27	<lmxll>	ll	129	lm	140		
>ref-88068	TCTCCTCCCACTCTTCCTCCTTGCCCA	7	<lmxll>	ll	138	lm	119		
>ref-88068	TCTCCTCCCACTCTTCCTCCTTGCCCA	23	<lmxll>	ll	136	lm	119		
>ref-88085	ATTCCTTCAACCTCGTCTCCTTCTCCT	14	<lmxll>	ll	127	lm	142		
>ref-88087	CCCAAGCACACATCCTCTCCCTCACCT	13	<lmxll>	ll	130	lm	138		
>ref-88087	CCCAAGCACACATCCTCTCCCTCACCT	22	<lmxll>	ll	130	lm	138		

>ref-88090	AATCCTCCCACCCCCCCTCCTTTAGTT	2	<lmxll>	ll	120	lm	118		
>ref-88126	CATAAGCACACAAATCCTCCTTCACAC	8	<nnxnp>	nn	134	np	132		
>ref-88137	AGAGGGGAAACGTTACCTCCCTCCCTC	24	<hkxhk>	hh	0	hk	273	kk	0
>ref-88137	AGAGGGGAAACGTTACCTCCCTCCCTC	27	<hkxhk>	hh	2	hk	271	kk	0
>ref-88152	TATGTTGGTACTCCGTCTCCCTCCTAG	12	<nnxnp>	nn	263	np	0		
>ref-88171	TTTCCCCATACCTCTCCTCCCTTACTC	14	<hkxhk>	hh	59	hk	132	kk	54
>ref-88190	GGAGTCAGTACCTCCCCTCCGACTCAG	2	<lmxll>	ll	93	lm	144		
>ref-88190	GGAGTCAGTACCTCCCCTCCGACTCAG	23	<lmxll>	ll	94	lm	141		
>ref-88192	CTCCTATTTACTCATTCTCCTTCTTCT	4	<lmxll>	ll	269	lm	0		
>ref-88192	CTCCTATTTACTCATTCTCCTTCTTCT	14	<nnxnp>	nn	131	np	89		
>ref-88195	AGAACTCCAACAACTGCTCCCTCACAC	16	<nnxnp>	nn	259	np	0		
>ref-88207	ACAAGCTAAACTGTCCCTCCACGATTG	24	<hkxhk>	hh	54	hk	152	kk	54
>ref-88208	TTTTCCCTGACCATTACTCCGTTGGTA	1	<nnxnp>	nn	243	np	0		
>ref-88208	TTTTCCCTGACCATTACTCCGTTGGTA	5	<nnxnp>	nn	243	np	0		
>ref-88227	TTCCTCATAACCCGCTCTCCCTTACCT	25	<nnxnp>	nn	122	np	148		
>ref-88228	CGAAAAAACACGTTGACTCCGTGTAAG	26	<lmxll>	ll	128	lm	122		
>ref-88234	TCCCACTGCACCCCTCCTCCATGTGAG	6	<lmxll>	ll	152	lm	108		
>ref-88240	GTTTACATTACCCTCCCTCCCCTCCCT	24	<nnxnp>	nn	102	np	128		
>ref-88270	CCGAACACTACAACAACTCCCCCATCA	23	<hkxhk>	hh	3	hk	262	kk	0
>ref-88278	ATGCCAAGGACGCCGCCTCCTACCCCG	8	<hkxhk>	hh	48	hk	143	kk	66
>ref-88292	CGACCCCCTACAACCCCTCCTCGCCCC	1	<nnxnp>	nn	173	np	75		
>ref-88292	CGACCCCCTACAACCCCTCCTCGCCCC	13	<hkxhk>	hh	0	hk	262	kk	5
>ref-88292	CGACCCCCTACAACCCCTCCTCGCCCC	14	<lmxll>	ll	131	lm	139		
>ref-88292	CGACCCCCTACAACCCCTCCTCGCCCC	26	<hkxhk>	hh	66	hk	201	kk	4
>ref-88293	TCCCTCCTAACTCTATCTCCTCCCTCT	13	<nnxnp>	nn	153	np	96		
>ref-88302	CTCCCACCCACATTCACTCCCTCATAC	12	<hkxhk>	hh	7	hk	252	kk	0
>ref-88302	CTCCCACCCACATTCACTCCCTCATAC	24	<hkxhk>	hh	7	hk	253	kk	0
>ref-88310	CCCATCCCCACCTCTTCTCCCAACCCT	9	<nnxnp>	nn	181	np	80		
>ref-88310	CCCATCCCCACCTCTTCTCCCAACCCT	12	<lmxll>	ll	53	lm	211		
>ref-88354	TTCTTTTACACAGTCCCTCCCTTCGCT	3	<nnxnp>	nn	130	np	137		
>ref-88356	TGATACAACACCACCTCTCCCCTCTCA	7	<hkxhk>	hh	29	hk	229	kk	0
>ref-88376	CGCTGCACCACACGCACTCCGCAACCA	6	<lmxll>	ll	117	lm	148		
>ref-88401	CATACACACACAGCTCCTCCCTCCCTC	3	<lmxll>	ll	132	lm	133		
>ref-88401	CATACACACACAGCTCCTCCCTCCCTC	5	<lmxll>	ll	136	lm	133		
>ref-88419	ATTTCTCTCACTGCCCCTCCAAACTCA	26	<hkxhk>	hh	6	hk	251	kk	1
>ref-88472	ATACATCACACCCGGGCTCCTTTTCGG	21	<nnxnp>	nn	128	np	135		
>ref-88472	ATACATCACACCCGGGCTCCTTTTCGG	26	<nnxnp>	nn	127	np	137		
>ref-88491	AGGTTCTTCACCGCCCCTCCCCTCCCA	13	<lmxll>	ll	139	lm	96		

>ref-88498	TTCTCCCTCACTCCTGCTCCTGCTCTT	16	<nnxnp>	nn	257	np	7		
>ref-88498	TTCTCCCTCACTCCTGCTCCTGCTCTT	22	<nnxnp>	nn	265	np	0		
>ref-88498	TTCTCCCTCACTCCTGCTCCTGCTCTT	26	<nnxnp>	nn	271	np	0		
>ref-88505	CCCCTTTTCACTATCACTCCTTTTCAT	15	<nnxnp>	nn	120	np	114		
>ref-88517	CTACCCACTACCTCTTCTCCCCTGATC	1	<lmxll>	ll	246	lm	0		
>ref-88590	GTCCTTCTAACCCCCCCTCCCCCCTCC	5	<hkxhk>	hh	0	hk	256	kk	4
>ref-88590	GTCCTTCTAACCCCCCCTCCCCCCTCC	9	<hkxhk>	hh	1	hk	260	kk	0
>ref-88602	ATGTTCTTCACATCAGCTCCCGAAGGC	6	<nnxnp>	nn	182	np	61		
>ref-88602	ATGTTCTTCACATCAGCTCCCGAAGGC	9	<nnxnp>	nn	182	np	61		
>ref-88604	ATGATGATTACACTAACTCCTTAAGAA	2	<nnxnp>	nn	120	np	129		
>ref-88607	GCTCCCCCAACCCACACTCCAACATAA	21	<nnxnp>	nn	135	np	124		
>ref-88622	TTAACTACAACCGCAGCTCCTCCACCC	12	<lmxll>	ll	111	lm	155		
>ref-88636	CGTCGAAGTACCCGAGCTCCAGGTGCA	9	<lmxll>	ll	266	lm	1		
>ref-88652	CTTACCCGAACGGGCGCTCCTCACCTA	5	<lmxll>	ll	109	lm	120		
>ref-88653	TCCCCATCCACTTCCCCTCCCCCTCCA	3	<lmxll>	ll	5	lm	252		
>ref-88653	TCCCCATCCACTTCCCCTCCCCCTCCA	9	<lmxll>	ll	5	lm	252		
>ref-88653	TCCCCATCCACTTCCCCTCCCCCTCCA	12	<lmxll>	ll	115	lm	118		
>ref-88653	TCCCCATCCACTTCCCCTCCCCCTCCA	13	<nnxnp>	nn	175	np	63		
>ref-88653	TCCCCATCCACTTCCCCTCCCCCTCCA	15	<lmxll>	ll	136	lm	121		
>ref-88660	CTCTCACCCACTTACTCTCCTTATCTA	4	<lmxll>	ll	107	lm	154		
>ref-88666	CCCTTTGTCACCGGTTCTCCACGTCAT	13	<nnxnp>	nn	120	np	151		
>ref-88695	CCCATTTCAACCCCAACTCCACCTTTT	16	<lmxll>	ll	137	lm	108		
>ref-88712	TTCCGTATCACCCCTCCTCCTCCTTTC	25	<lmxll>	ll	119	lm	135		
>ref-88732	AGCACATCTACCGCATCTCCAACATCC	12	<hkxhk>	hh	76	hk	131	kk	46
>ref-88738	CTTCATTGCACTTCCACTCCTCCTCCC	24	<nnxnp>	nn	253	np	0		
>ref-88753	TTTCATATTACATTTTCTCCTTGTGGA	4	<nnxnp>	nn	147	np	81		
>ref-88757	GAACTGAAGACAAGAGCTCCATTGCAA	26	<nnxnp>	nn	124	np	144		
>ref-88763	TATGCTACAACAAAATCTCCAGCCAGG	26	<nnxnp>	nn	108	np	111		
>ref-88782	CACCTCTTAACTCTTTCTCCTTCTCTC	22	<lmxll>	ll	5	lm	268		
>ref-88801	TTCGGTCTAACGGTCGCTCCATTAAGA	5	<hkxhk>	hh	91	hk	120	kk	36
>ref-88821	GAAAGAAACACTTCAGCTCCTCTCAGC	1	<hkxhk>	hh	0	hk	264	kk	3
>ref-88821	GAAAGAAACACTTCAGCTCCTCTCAGC	15	<lmxll>	ll	255	lm	13		
>ref-88828	TTTTCTTATACACAGGCTCCCTTGTAA	12	<hkxhk>	hh	54	hk	127	kk	63
>ref-88841	TCCTCCCTCACTCATTCTCCCTCGCCA	7	<lmxll>	ll	238	lm	25		
>ref-88841	TCCTCCCTCACTCATTCTCCCTCGCCA	12	<hkxhk>	hh	0	hk	273	kk	0
>ref-88841	TCCTCCCTCACTCATTCTCCCTCGCCA	14	<hkxhk>	hh	0	hk	272	kk	0
>ref-88867	ATCACGAACACAGTGTCTCCGCACAAC	13	<lmxll>	ll	142	lm	127		
>ref-88903	CTCGCTTACACGGTCACTCCATGTGCC	9	<hkxhk>	hh	61	hk	146	kk	53

>ref-88929	CACTTACACACACCCTCTCCTCGACAG	5	<lmxll>	ll	128	lm	141		
>ref-88931	GAGCTCCTCACAGAACCTCCTGTGAGA	24	<lmxll>	ll	133	lm	119		
>ref-88942	CTCTTTTCAACCATCCCTCCTTCCCTA	7	<nnxnp>	nn	119	np	137		
>ref-88946	AGGAAATGCACTTTGTCTCCCGAGACG	25	<hkxhk>	hh	0	hk	273	kk	0
>ref-88950	CTCTTGTCAACCTCACCTCCTGACAAC	3	<nnxnp>	nn	137	np	131		
>ref-88950	CTCTTGTCAACCTCACCTCCTGACAAC	6	<nnxnp>	nn	126	np	138		
>ref-88957	AGTACACAGACAGAATCTCCGAAGGAC	2	<nnxnp>	nn	110	np	140		
>ref-88963	TCATCTACTACATCCTCTCCTCTATCT	25	<nnxnp>	nn	130	np	134		
>ref-89018	ACACATTGAACATATACTCCAATCACT	1	<hkxhk>	hh	47	hk	149	kk	59
>ref-89018	ACACATTGAACATATACTCCAATCACT	26	<nnxnp>	nn	126	np	134		
>ref-89025	CCCCTACATACTTCCTCTCCTCCTACC	3	<hkxhk>	hh	67	hk	177	kk	19
>ref-89025	CCCCTACATACTTCCTCTCCTCCTACC	4	<lmxll>	ll	73	lm	193		
>ref-89025	CCCCTACATACTTCCTCTCCTCCTACC	23	<lmxll>	ll	73	lm	193		
>ref-89044	CATATCCCTACTCCCCCTCCCCCTTCT	8	<nnxnp>	nn	114	np	141		
>ref-89053	TGTCTAATTACCCTTTCTCCTTTTGCT	9	<hkxhk>	hh	53	hk	140	kk	53
>ref-89086	CCTCTTCCTACCCCGTCTCCCTCCTAC	14	<nnxnp>	nn	123	np	138		
>ref-89091	ACCATCCTCACCCTCCCTCCCTCTCCT	3	<nnxnp>	nn	256	np	0		
>ref-89091	ACCATCCTCACCCTCCCTCCCTCTCCT	6	<nnxnp>	nn	248	np	1		
>ref-89100	AAGCCTCGCACCTCATCTCCTTCTCTC	9	<hkxhk>	hh	71	hk	139	kk	55
>ref-89129	CGAAACAGAACCGCTCCTCCAATGATA	13	<lmxll>	ll	127	lm	133		
>ref-89167	ATCCGTTTCACAAACCCTCCTTTAAAT	13	<nnxnp>	nn	107	np	130		
>ref-89187	CTTCCAACCACCACAACTCCAGCAACA	1	<nnxnp>	nn	256	np	0		
>ref-89190	GCCTCTCCCACTTCCACTCCCCCATCT	1	<hkxhk>	hh	1	hk	267	kk	0
>ref-89201	GTTGTTATCACCTCATCTCCGTTTTCT	5	<nnxnp>	nn	126	np	133		
>ref-89221	AAAACATACACATACACTCCCTTACAC	3	<lmxll>	ll	13	lm	250		
>ref-89221	AAAACATACACATACACTCCCTTACAC	9	<hkxhk>	hh	0	hk	260	kk	10
>ref-89221	AAAACATACACATACACTCCCTTACAC	27	<hkxhk>	hh	3	hk	269	kk	0
>ref-89245	CTTGGGCAGACCCCTTCTCCCTCCCCC	9	<nnxnp>	nn	254	np	2		
>ref-89253	GTCTTCCTCACACACTCTCCCCCTCCC	5	<lmxll>	ll	0	lm	271		
>ref-89253	GTCTTCCTCACACACTCTCCCCCTCCC	12	<lmxll>	ll	0	lm	271		
>ref-89265	TCCCCTTACACCACCTCTCCTTCTGTT	3	<hkxhk>	hh	49	hk	115	kk	75
>ref-89278	CCCTCTCTCACCATAGCTCCCTCACTA	5	<lmxll>	ll	114	lm	141		
>ref-89278	CCCTCTCTCACCATAGCTCCCTCACTA	8	<lmxll>	ll	134	lm	124		
>ref-89283	CTACCTCATACCCCCTCTCCTCCCCCT	3	<lmxll>	ll	254	lm	1		
>ref-89283	CTACCTCATACCCCCTCTCCTCCCCCT	4	<nnxnp>	nn	263	np	0		
>ref-89289	CATCACCCAACGCCGCCTCCACTCCCT	4	<nnxnp>	nn	145	np	125		
>ref-89303	TGAATATCTACACGCTCTCCCCATCTC	9	<lmxll>	ll	131	lm	133		
>ref-89325	TCTCTCACGACAGTTTCTCCTTTCCAA	22	<nnxnp>	nn	121	np	143		

>ref-89327	TACTCCATCACCATCCCTCCTTGTCTT	23	<lmxll>	ll	131	lm	120		
>ref-89331	CTCCCCCCTACCCCAGCTCCCTGCTCC	4	<hkxhk>	hh	69	hk	181	kk	0
>ref-89331	CTCCCCCCTACCCCAGCTCCCTGCTCC	23	<hkxhk>	hh	0	hk	182	kk	66
>ref-89344	CCACCTCCCACCTTCCCTCCCCCCACC	15	<nnxnp>	nn	185	np	66		
>ref-89344	CCACCTCCCACCTTCCCTCCCCCCACC	16	<lmxll>	ll	170	lm	53		
>ref-89397	TTGCATGCTACCTCCTCTCCATTATGT	7	<nnxnp>	nn	143	np	124		
>ref-89407	TCACTCCACACCCTCTCTCCAACATTC	12	<hkxhk>	hh	43	hk	130	kk	57
>ref-89409	CACAGAATGACGATTACTCCTTTCTAT	22	<lmxll>	ll	111	lm	153		
>ref-89415	CCTTCTCCCACCCTCTCTCCACTCCAC	16	<lmxll>	ll	135	lm	121		
>ref-89437	TCTCTTCTTACCTTTCCTCCTTTCTTC	6	<lmxll>	ll	3	lm	265		
>ref-89437	TCTCTTCTTACCTTTCCTCCTTTCTTC	13	<lmxll>	ll	3	lm	265		
>ref-89443	TATTAAAACACTCAGGCTCCAAGCTAT	24	<lmxll>	ll	120	lm	129		
>ref-89456	TTATTTCACACTCTTTCTCCCCTTTTC	1	<hkxhk>	hh	4	hk	222	kk	12
>ref-89456	TTATTTCACACTCTTTCTCCCCTTTTC	24	<hkxhk>	hh	5	hk	223	kk	12
>ref-89465	ATCAACGCCACGAGGACTCCCCCAGCA	14	<lmxll>	ll	143	lm	127		
>ref-89466	CACTTACGTACACCATCTCCACTACCC	9	<lmxll>	ll	225	lm	0		
>ref-89475	CCTTCCTTCACTCCCTCTCCCTGGCAT	9	<hkxhk>	hh	0	hk	263	kk	2
>ref-89475	CCTTCCTTCACTCCCTCTCCCTGGCAT	24	<hkxhk>	hh	0	hk	263	kk	2
>ref-89485	CTATCTCTAACCTATTCTCCTTCACAT	22	<nnxnp>	nn	113	np	138		
>ref-89500	TTTCTTCCAACTTCATCTCCTTTTCCC	5	<nnxnp>	nn	136	np	129		
>ref-89500	TTTCTTCCAACTTCATCTCCTTTTCCC	8	<nnxnp>	nn	135	np	129		
>ref-89510	CACCACATCACACCGACTCCTCAGCCT	2	<lmxll>	ll	128	lm	141		
>ref-89513	ATCGGAATTACTCTTCCTCCCTTTCTT	4	<lmxll>	ll	51	lm	196		
>ref-89523	TCCCTTCCAACGCCCCCTCCCCCTTTT	9	<nnxnp>	nn	21	np	243		
>ref-89523	TCCCTTCCAACGCCCCCTCCCCCTTTT	12	<nnxnp>	nn	21	np	243		
>ref-89524	CGACGTCCCACTCTCCCTCCGTCTTTC	24	<hkxhk>	hh	55	hk	161	kk	51
>ref-89524	CGACGTCCCACTCTCCCTCCGTCTTTC	25	<hkxhk>	hh	57	hk	160	kk	50
>ref-89525	ATTCATAAAACGTGAGCTCCTTGATAC	26	<nnxnp>	nn	132	np	119		
>ref-89531	CTTCCTTCCACCCAACCTCCCTTTCCC	2	<hkxhk>	hh	0	hk	226	kk	27
>ref-89531	CTTCCTTCCACCCAACCTCCCTTTCCC	15	<hkxhk>	hh	25	hk	230	kk	0
>ref-89550	ATCCTCGGCACTATACCTCCTTCTACA	27	<nnxnp>	nn	142	np	127		
>ref-89562	AATAAGGGTACAAAATCTCCATTACTG	6	<lmxll>	ll	6	lm	226		
>ref-89563	TTCCTACATACCGAATCTCCTTCCCCT	27	<hkxhk>	hh	53	hk	134	kk	66
>ref-89565	CTTTCCATCACTCTACCTCCTTCAATA	13	<lmxll>	ll	99	lm	134		
>ref-89575	ATTTCGGACACGGCTGCTCCTCGGCTG	13	<lmxll>	ll	128	lm	130		
>ref-89585	TTGTCAATCACATTTCCTCCGTTGGTT	25	<lmxll>	ll	156	lm	112		
>ref-89590	CTTCCTGCTACTCTTCCTCCCCCCCAT	7	<nnxnp>	nn	103	np	144		
>ref-89590	CTTCCTGCTACTCTTCCTCCCCCCCAT	21	<nnxnp>	nn	101	np	147		

>ref-89599	CGGATATGTACCCTACCTCCACTTGCA	5	<hkxhk>	hh	38	hk	135	kk	56
>ref-89606	CAATAAATCACTGGTCCTCCTTGAATC	4	<lmxll>	ll	147	lm	89		
>ref-89606	CAATAAATCACTGGTCCTCCTTGAATC	23	<hkxhk>	hh	0	hk	233	kk	11
>ref-89633	CCAACCACAACAATTTCTCCGCCTACT	23	<lmxll>	ll	111	lm	110		
>ref-89633	CCAACCACAACAATTTCTCCGCCTACT	25	<nnxnp>	nn	105	np	117		
>ref-89635	CTTGGCCCCACTCTGACTCCTCGCGAA	27	<nnxnp>	nn	121	np	130		
>ref-89682	CACCTCACTACACCCCCTCCCCTGTCA	25	<nnxnp>	nn	116	np	148		
>ref-89715	AAAACAACAACCGAGACTCCACTGTTT	23	<hkxhk>	hh	65	hk	135	kk	46
>ref-89717	TCTGCTGAGACGAAAGCTCCTTACCAG	7	<nnxnp>	nn	100	np	141		
>ref-89719	TTTCCTTGCACAATTACTCCTCTATTA	21	<lmxll>	ll	105	lm	115		
>ref-89731	GGCTCGCGGACTAGCTCTCCTAATGCT	21	<hkxhk>	hh	0	hk	270	kk	2
>ref-89731	GGCTCGCGGACTAGCTCTCCTAATGCT	22	<hkxhk>	hh	1	hk	271	kk	0
>ref-89731	GGCTCGCGGACTAGCTCTCCTAATGCT	27	<hkxhk>	hh	0	hk	273	kk	0
>ref-89747	CTATTCCTCACCCCTTCTCCCCCATTC	27	<nnxnp>	nn	122	np	147		
>ref-89758	ATTCCTACCACCCTCTCTCCATGCTCC	5	<nnxnp>	nn	118	np	145		
>ref-89758	ATTCCTACCACCCTCTCTCCATGCTCC	7	<nnxnp>	nn	118	np	145		
>ref-89774	CACCCCCATACCCTCTCTCCGCCTTCC	6	<lmxll>	ll	129	lm	137		
>ref-89784	CTCAACTGAACAGATTCTCCTCAAACC	2	<hkxhk>	hh	60	hk	136	kk	31
>ref-89784	CTCAACTGAACAGATTCTCCTCAAACC	24	<nnxnp>	nn	110	np	127		
>ref-89790	CTTCATCCAACCGAAGCTCCTCGTTCC	14	<hkxhk>	hh	59	hk	144	kk	51
>ref-89817	CACACCCCCACAGTCCCTCCCACACCC	2	<hkxhk>	hh	0	hk	273	kk	0
>ref-89817	CACACCCCCACAGTCCCTCCCACACCC	3	<hkxhk>	hh	0	hk	273	kk	0
>ref-89817	CACACCCCCACAGTCCCTCCCACACCC	22	<hkxhk>	hh	1	hk	272	kk	0
>ref-89817	CACACCCCCACAGTCCCTCCCACACCC	23	<hkxhk>	hh	0	hk	272	kk	0
>ref-89877	GCAATTCCAACCTACACTCCACCCACG	2	<lmxll>	ll	130	lm	124		
>ref-89880	TACTTCTCTACTCCCCCTCCTCCCAGC	22	<lmxll>	ll	33	lm	192		
>ref-89884	AAGGAAGTAACGTGGCCTCCGCGGATG	15	<hkxhk>	hh	57	hk	151	kk	58
>ref-89894	CCCCTGCTGACCCCCTCTCCTCGCCTC	14	<lmxll>	ll	128	lm	132		
>ref-89905	CATCTCTATACCCTCTCTCCGTTTCCC	21	<nnxnp>	nn	273	np	0		
>ref-89905	CATCTCTATACCCTCTCTCCGTTTCCC	22	<hkxhk>	hh	0	hk	272	kk	0
>ref-89908	CAGAGCGTGACATGCTCTCCCGCAGTC	4	<lmxll>	ll	144	lm	123		
>ref-89931	TTAATACCCACAATTACTCCGAACGCT	21	<hkxhk>	hh	0	hk	272	kk	0
>ref-89931	TTAATACCCACAATTACTCCGAACGCT	23	<hkxhk>	hh	0	hk	272	kk	0
>ref-89936	AGTCTGAATACTGGAACTCCCACTACC	27	<nnxnp>	nn	251	np	1		
>ref-89953	ATGCTAAAGACTTAGTCTCCTAACGCC	5	<lmxll>	ll	121	lm	141		
>ref-89963	TCCCACTTCACAGAAGCTCCTACATGT	4	<nnxnp>	nn	123	np	104		
>ref-89964	TTATCTTCGACCTATCCTCCGCTGAGT	9	<lmxll>	ll	1	lm	270		
>ref-89980	GGATAGAAAACCCTCTCTCCCTGTTTA	4	<nnxnp>	nn	127	np	119		

>ref-89982	ATTCTATGTACATGCTCTCCATCCCAT	21	<lmxll>	ll	122	lm	144		
>ref-89982	ATTCTATGTACATGCTCTCCATCCCAT	22	<lmxll>	ll	130	lm	137		
>ref-90005	GGCACGGGTACGGCCTCTCCCCCGTGT	5	<hkxhk>	hh	6	hk	264	kk	0
>ref-90005	GGCACGGGTACGGCCTCTCCCCCGTGT	6	<hkxhk>	hh	2	hk	271	kk	0
>ref-90005	GGCACGGGTACGGCCTCTCCCCCGTGT	7	<hkxhk>	hh	0	hk	272	kk	0
>ref-90005	GGCACGGGTACGGCCTCTCCCCCGTGT	15	<hkxhk>	hh	1	hk	271	kk	0
>ref-90005	GGCACGGGTACGGCCTCTCCCCCGTGT	16	<hkxhk>	hh	0	hk	266	kk	5
>ref-90014	CCTAATTATACTCTCTCTCCTCAAGAC	9	<nnxnp>	nn	125	np	106		
>ref-90014	CCTAATTATACTCTCTCTCCTCAAGAC	25	<nnxnp>	nn	125	np	106		
>ref-90075	GGCCTCTCCACCGTTTCTCCATCCCCT	26	<hkxhk>	hh	60	hk	140	kk	68
>ref-90089	AGCACGACAACTCAAACTCCTAGGCCA	3	<nnxnp>	nn	31	np	220		
>ref-90089	AGCACGACAACTCAAACTCCTAGGCCA	25	<nnxnp>	nn	29	np	220		
>ref-90131	TTTCCTCCCACCTCCTCTCCCTCCCAT	5	<hkxhk>	hh	20	hk	246	kk	0
>ref-90131	TTTCCTCCCACCTCCTCTCCCTCCCAT	7	<hkxhk>	hh	0	hk	224	kk	35
>ref-90131	TTTCCTCCCACCTCCTCTCCCTCCCAT	12	<hkxhk>	hh	0	hk	225	kk	34
>ref-90131	TTTCCTCCCACCTCCTCTCCCTCCCAT	16	<hkxhk>	hh	0	hk	247	kk	19
>ref-90131	TTTCCTCCCACCTCCTCTCCCTCCCAT	23	<nnxnp>	nn	213	np	48		
>ref-90131	TTTCCTCCCACCTCCTCTCCCTCCCAT	25	<hkxhk>	hh	0	hk	261	kk	3
>ref-90131	TTTCCTCCCACCTCCTCTCCCTCCCAT	26	<hkxhk>	hh	3	hk	246	kk	0
>ref-90141	CTTCTCAGCACTCCTCCTCCTCTCCCC	2	<lmxll>	ll	168	lm	83		
>ref-90141	CTTCTCAGCACTCCTCCTCCTCTCCCC	4	<lmxll>	ll	168	lm	82		
>ref-90141	CTTCTCAGCACTCCTCCTCCTCTCCCC	6	<hkxhk>	hh	2	hk	244	kk	9
>ref-90141	CTTCTCAGCACTCCTCCTCCTCTCCCC	14	<hkxhk>	hh	2	hk	244	kk	9
>ref-90171	TTAACTGACACCTTCTCTCCCCGCTGG	9	<lmxll>	ll	120	lm	115		
>ref-90176	TGCCCCTTCACCCTGTCTCCTCCCTTT	1	<hkxhk>	hh	58	hk	155	kk	55
>ref-90176	TGCCCCTTCACCCTGTCTCCTCCCTTT	2	<hkxhk>	hh	58	hk	155	kk	55
>ref-90189	GGGCACCTTACCCTCTCTCCTCCCTTC	14	<hkxhk>	hh	0	hk	272	kk	0
>ref-90189	GGGCACCTTACCCTCTCTCCTCCCTTC	22	<hkxhk>	hh	66	hk	205	kk	0
>ref-90189	GGGCACCTTACCCTCTCTCCTCCCTTC	26	<lmxll>	ll	140	lm	128		
>ref-90211	GACGCCATGACGGTTGCTCCAGGCATG	16	<hkxhk>	hh	0	hk	273	kk	0
>ref-90211	GACGCCATGACGGTTGCTCCAGGCATG	21	<hkxhk>	hh	0	hk	273	kk	0
>ref-90217	TCTTCGATGACTCAATCTCCTTCGCAC	15	<lmxll>	ll	113	lm	129		
>ref-90217	TCTTCGATGACTCAATCTCCTTCGCAC	27	<lmxll>	ll	113	lm	129		
>ref-90231	CCCTTGCGAACGAGCTCTCCCCTTCTC	3	<nnxnp>	nn	271	np	0		
>ref-90234	GGACCCACAACAAGCTCTCCTTCTCGC	12	<nnxnp>	nn	136	np	125		
>ref-90271	CCGAAGATCACTGTTGCTCCAAATGAA	5	<hkxhk>	hh	1	hk	251	kk	14
>ref-90271	CCGAAGATCACTGTTGCTCCAAATGAA	22	<nnxnp>	nn	93	np	145		
>ref-90271	CCGAAGATCACTGTTGCTCCAAATGAA	24	<hkxhk>	hh	14	hk	251	kk	1

>ref-90275	CTCGAATACACAGACCCTCCAGGAGAG	12	<nnxnp>	nn	273	np	0		
>ref-90281	CCTTTTACCACACCACCTCCTCTACCT	9	<nnxnp>	nn	108	np	116		
>ref-90281	CCTTTTACCACACCACCTCCTCTACCT	12	<lmxll>	ll	122	lm	112		
>ref-90305	GGCTGCTTTACCCTGCCTCCTGCTGCC	25	<lmxll>	ll	133	lm	137		
>ref-90329	CTTTCGTAAACGTTTTCTCCATCACTT	8	<hkxhk>	hh	1	hk	261	kk	1
>ref-90329	CTTTCGTAAACGTTTTCTCCATCACTT	25	<hkxhk>	hh	0	hk	222	kk	24
>ref-90331	ACCTCCGTCACCAACACTCCTCCCTGG	14	<lmxll>	ll	117	lm	143		
>ref-90362	AGAGGGCTTACGCCGCCTCCCTCTTCG	9	<nnxnp>	nn	273	np	0		
>ref-90368	TTTTCCTCTACATCTTCTCCCTTCCCT	2	<nnxnp>	nn	0	np	271		
>ref-90368	TTTTCCTCTACATCTTCTCCCTTCCCT	7	<nnxnp>	nn	0	np	271		
>ref-90368	TTTTCCTCTACATCTTCTCCCTTCCCT	8	<lmxll>	ll	20	lm	234		
>ref-90368	TTTTCCTCTACATCTTCTCCCTTCCCT	23	<lmxll>	ll	20	lm	233		
>ref-90381	ACTCTTTGTACTTACGCTCCCTTTCTG	9	<lmxll>	ll	127	lm	123		
>ref-90398	CTAGTCTTCACCCTTACTCCATTCCCT	15	<lmxll>	ll	111	lm	139		
>ref-90405	TTTTAACCCACTTCCTCTCCTTCTCCC	1	<lmxll>	ll	3	lm	224		
>ref-90406	GCCTGGAGCACCTCTCCTCCTCTTCCT	23	<nnxnp>	nn	122	np	136		
>ref-90416	AACATCAACACCACAACTCCAAACACA	23	<nnxnp>	nn	131	np	135		
>ref-90417	AGCATAGATACCACTGCTCCACTTAAG	9	<lmxll>	ll	20	lm	204		
>ref-90417	AGCATAGATACCACTGCTCCACTTAAG	13	<hkxhk>	hh	30	hk	188	kk	3
>ref-90417	AGCATAGATACCACTGCTCCACTTAAG	14	<lmxll>	ll	20	lm	204		
>ref-90424	CAGTATAAAACACTTGCTCCAGCGTCC	22	<lmxll>	ll	113	lm	131		
>ref-90432	CCACCTTCTACTCCTTCTCCTGCCTCC	4	<lmxll>	ll	124	lm	141		
>ref-90432	CCACCTTCTACTCCTTCTCCTGCCTCC	8	<lmxll>	ll	140	lm	127		
>ref-90441	TGCTCCTTCACCTAGTCTCCCGTCTTT	2	<lmxll>	ll	134	lm	132		
>ref-90443	TTTAACGACACTGTCCCTCCAAGGTAA	1	<nnxnp>	nn	262	np	0		
>ref-90452	GAATGATACACGTGAACTCCACCCTCT	21	<nnxnp>	nn	132	np	128		
>ref-90478	CGAAGAAAAACAAAATCTCCTCGCCTA	1	<lmxll>	ll	109	lm	116		
>ref-90478	CGAAGAAAAACAAAATCTCCTCGCCTA	2	<lmxll>	ll	110	lm	116		
>ref-90486	ACGCCCAATACCCCTCCTCCTCCTCCT	3	<hkxhk>	hh	0	hk	270	kk	2
>ref-90486	ACGCCCAATACCCCTCCTCCTCCTCCT	16	<hkxhk>	hh	77	hk	191	kk	0
>ref-90486	ACGCCCAATACCCCTCCTCCTCCTCCT	26	<hkxhk>	hh	2	hk	270	kk	0
>ref-90531	CAGACACAAACCCCCTCTCCTCCCACA	26	<lmxll>	ll	243	lm	0		
>ref-90532	ACTTATCTGACCTTTTCTCCTCTTCCC	1	<hkxhk>	hh	78	hk	169	kk	0
>ref-90532	ACTTATCTGACCTTTTCTCCTCTTCCC	27	<hkxhk>	hh	77	hk	169	kk	0
>ref-90541	TTCACGGAGACATATTCTCCCTTCACA	15	<lmxll>	ll	121	lm	139		
>ref-90574	AATATTTGTACATGTTCTCCTTGGTGA	22	<hkxhk>	hh	0	hk	253	kk	9
>ref-90581	CACTCCTCAACCTTCCCTCCCTCCTCT	9	<lmxll>	ll	82	lm	177		
>ref-90581	CACTCCTCAACCTTCCCTCCCTCCTCT	14	<lmxll>	ll	83	lm	176		

>ref-90586	CACTCTACCACTCCCACTCCACTCCCA	2	<nnxnp>	nn	59	np	166		
>ref-90586	CACTCTACCACTCCCACTCCACTCCCA	7	<nnxnp>	nn	63	np	169		
>ref-90587	ATCTTTCTCACCTTCTCTCCTGTTGTT	3	<nnxnp>	nn	96	np	138		
>ref-90587	ATCTTTCTCACCTTCTCTCCTGTTGTT	13	<nnxnp>	nn	96	np	138		
>ref-90588	TCCCCCATCACTCACACTCCATGATTC	2	<nnxnp>	nn	128	np	119		
>ref-90588	TCCCCCATCACTCACACTCCATGATTC	6	<nnxnp>	nn	113	np	135		
>ref-90590	TTGAAGAGAACTATCACTCCACTTGTT	14	<hkxhk>	hh	52	hk	129	kk	50
>ref-90604	CTCTCCGCCACACCGTCTCCTTTCCTG	23	<nnxnp>	nn	122	np	138		
>ref-90608	CACACGCTGACTCTTCCTCCTGGGACG	26	<nnxnp>	nn	93	np	150		
>ref-90641	CTTTGCCTCACTCTCTCTCCCTCGATC	5	<nnxnp>	nn	261	np	3		
>ref-90641	CTTTGCCTCACTCTCTCTCCCTCGATC	24	<nnxnp>	nn	241	np	0		
>ref-90641	CTTTGCCTCACTCTCTCTCCCTCGATC	25	<nnxnp>	nn	233	np	7		
>ref-90647	ATCCCCCCCACCCTTCCTCCCTCCAAC	7	<nnxnp>	nn	109	np	129		
>ref-90657	TCCTTTCTCACCTCTTCTCCCCTTCTT	21	<nnxnp>	nn	10	np	262		
>ref-90685	CTGACCTCTACTTCGACTCCGATCTTG	26	<lmxll>	ll	147	lm	82		
>ref-90692	GGAACCGCGACTCTCCCTCCCTCCGTG	16	<hkxhk>	hh	0	hk	273	kk	0
>ref-90710	CTATTTCTAACTTTGTCTCCATGTATA	1	<hkxhk>	hh	7	hk	256	kk	1
>ref-90710	CTATTTCTAACTTTGTCTCCATGTATA	21	<hkxhk>	hh	7	hk	256	kk	1
>ref-90710	CTATTTCTAACTTTGTCTCCATGTATA	25	<lmxll>	ll	144	lm	114		
>ref-90732	ATGTGGTTCACTATGACTCCATCCGTC	7	<nnxnp>	nn	124	np	142		
>ref-90734	GATGTACGTACGTTCGCTCCCGAGATC	14	<hkxhk>	hh	108	hk	97	kk	44
>ref-90738	TTTCTTCCCACTTCATCTCCTCTCCCC	4	<nnxnp>	nn	268	np	0		
>ref-90738	TTTCTTCCCACTTCATCTCCTCTCCCC	27	<lmxll>	ll	267	lm	1		
>ref-90747	CACCTTACTACATTAACTCCTCGTACC	23	<nnxnp>	nn	124	np	133		
>ref-90757	AGGAAGGAAACACCTGCTCCTCGAGGC	21	<hkxhk>	hh	0	hk	266	kk	5
>ref-90767	TTCAGCAGTACACCCTCTCCGCTGCCT	9	<lmxll>	ll	141	lm	118		
>ref-90803	CTTCTAGAGACAGTGTCTCCATAAAAA	7	<lmxll>	ll	7	lm	247		
>ref-90803	CTTCTAGAGACAGTGTCTCCATAAAAA	8	<hkxhk>	hh	21	hk	235	kk	0
>ref-90803	CTTCTAGAGACAGTGTCTCCATAAAAA	9	<hkxhk>	hh	0	hk	240	kk	13
>ref-90803	CTTCTAGAGACAGTGTCTCCATAAAAA	26	<nnxnp>	nn	55	np	174		
>ref-90813	GAACCCTCCACTAACCCTCCCTTGCCC	9	<lmxll>	ll	136	lm	111		
>ref-90813	GAACCCTCCACTAACCCTCCCTTGCCC	23	<hkxhk>	hh	44	hk	157	kk	46
>ref-90814	GTCTGCACTACAGCAACTCCCCTTCCC	22	<nnxnp>	nn	271	np	0		
>ref-90814	GTCTGCACTACAGCAACTCCCCTTCCC	24	<nnxnp>	nn	267	np	0		
>ref-90856	TTTGCACGAACCTTCCCTCCCTCTCGC	27	<nnxnp>	nn	125	np	144		
>ref-90863	ACACCACTCACGAACCCTCCACGGCCT	22	<nnxnp>	nn	130	np	141		
>ref-90868	GAACCGTACACAATATCTCCTAACGCC	6	<lmxll>	ll	106	lm	140		
>ref-90870	AACTCCACGACTCCGACTCCACAGCCC	4	<hkxhk>	hh	0	hk	65	kk	199

>ref-90878	CTACCCTACACACCTTCTCCTTCTCCC	5	<nnxnp>	nn	267	np	0		
>ref-90885	GTCAGGCCAACACTAGCTCCTCTTTTC	26	<lmxll>	ll	114	lm	140		
>ref-90891	CAGAATGCGACGACCACTCCTCTCGGC	12	<hkxhk>	hh	57	hk	153	kk	56
>ref-90891	CAGAATGCGACGACCACTCCTCTCGGC	13	<hkxhk>	hh	57	hk	153	kk	56
>ref-90906	CCCACCACAACCCTTCCTCCCTCCCCT	26	<lmxll>	ll	125	lm	129		
>ref-90919	TACATGGATACAGAGGCTCCTCATTCG	9	<nnxnp>	nn	259	np	0		
>ref-90927	TGATATCGAACTTATGCTCCCACCAAA	3	<lmxll>	ll	108	lm	118		
>ref-90927	TGATATCGAACTTATGCTCCCACCAAA	24	<nnxnp>	nn	117	np	108		
>ref-90936	CTTGTGAAAACTCTTCCTCCTAGAGAT	12	<hkxhk>	hh	2	hk	238	kk	5
>ref-90941	CTCCCCCGCACCTTCTCTCCTCACCCT	6	<hkxhk>	hh	1	hk	269	kk	0
>ref-90941	CTCCCCCGCACCTTCTCTCCTCACCCT	13	<hkxhk>	hh	0	hk	269	kk	1
>ref-90943	GACTCCCCCACCCTTTCTCCCTCCCTC	2	<nnxnp>	nn	121	np	125		
>ref-90943	GACTCCCCCACCCTTTCTCCCTCCCTC	23	<nnxnp>	nn	140	np	118		
>ref-90961	GACGCGAATACCTCCCCTCCCACATTA	5	<hkxhk>	hh	59	hk	136	kk	66
>ref-90964	AATTTCACCACCCCTTCTCCTCTATTT	3	<hkxhk>	hh	2	hk	238	kk	5
>ref-90977	AAAACATCCACACGAGCTCCAAACCAA	26	<nnxnp>	nn	112	np	135		
>ref-90986	AGCAACTCGACGCCCTCTCCCTCCCTC	23	<nnxnp>	nn	137	np	131		
>ref-90986	AGCAACTCGACGCCCTCTCCCTCCCTC	26	<hkxhk>	hh	0	hk	208	kk	57
>ref-90997	TATGCAGAAACAAATTCTCCTTCATGT	2	<hkxhk>	hh	183	hk	69	kk	0
>ref-90997	TATGCAGAAACAAATTCTCCTTCATGT	3	<hkxhk>	hh	0	hk	234	kk	0
>ref-90997	TATGCAGAAACAAATTCTCCTTCATGT	12	<nnxnp>	nn	57	np	203		
>ref-91014	GATTACGAGACTTAATCTCCTTAACAA	21	<lmxll>	ll	131	lm	119		
>ref-91034	TCTCCCTTAACCTCTTCTCCTCTCTCT	2	<nnxnp>	nn	202	np	58		
>ref-91034	TCTCCCTTAACCTCTTCTCCTCTCTCT	8	<nnxnp>	nn	200	np	59		
>ref-91034	TCTCCCTTAACCTCTTCTCCTCTCTCT	22	<hkxhk>	hh	0	hk	250	kk	13
>ref-91072	CATTCCTTCACTTATCCTCCCCCATTC	25	<lmxll>	ll	134	lm	124		
>ref-91078	TATGGATGAACGTTTCCTCCTCCTCCT	27	<lmxll>	ll	226	lm	1		
>ref-91103	AGGGGGAGAACCTGGACTCCACACCCG	12	<lmxll>	ll	144	lm	119		
>ref-91105	CACTCTCACACTATCACTCCCCTAAGT	22	<nnxnp>	nn	103	np	153		
>ref-91112	GAGAGTAAAACACCTTCTCCTCTTCTC	1	<nnxnp>	nn	128	np	121		
>ref-91123	TATTCCCTTACCCTCTCTCCTCACTCA	13	<nnxnp>	nn	124	np	140		
>ref-91142	TGGGGCTTCACCTAGCCTCCCCCTCGA	9	<nnxnp>	nn	272	np	0		
>ref-91163	ACACAAATAACACAAACTCCACGAACA	26	<nnxnp>	nn	232	np	0		
>ref-91179	AACTACAGTACATAAGCTCCCTCACTC	8	<lmxll>	ll	133	lm	131		
>ref-91180	GGGATCGCCACGTCGACTCCACCACAC	25	<nnxnp>	nn	269	np	0		
>ref-91180	GGGATCGCCACGTCGACTCCACCACAC	27	<nnxnp>	nn	270	np	0		
>ref-91197	CTTCCCTTTACTCCCTCTCCTTCTTAC	6	<hkxhk>	hh	56	hk	144	kk	44
>ref-91204	TCCTTATTCACTCCCTCTCCTCTTCAT	9	<lmxll>	ll	190	lm	67		

>ref-91204	TCCTTATTCACTCCCTCTCCTCTTCAT	26	<lmxll>	ll	188	lm	67		
>ref-91296	ATTCCCCACACCTCTCCTCCTCATTTA	8	<hkxhk>	hh	65	hk	123	kk	35
>ref-91298	TGTGTTCTTACATGAGCTCCGACTTAA	9	<nnxnp>	nn	263	np	0		
>ref-91319	TCGTCTATTACTTTCGCTCCGCTTATT	3	<lmxll>	ll	114	lm	141		
>ref-91319	TCGTCTATTACTTTCGCTCCGCTTATT	5	<hkxhk>	hh	52	hk	145	kk	59
>ref-91331	CCGGAATAAACATGATCTCCGTGTCCG	6	<lmxll>	ll	233	lm	0		
>ref-91341	CCATCTCCAACCTCTTCTCCCCCACTC	3	<nnxnp>	nn	117	np	150		
>ref-91341	CCATCTCCAACCTCTTCTCCCCCACTC	21	<hkxhk>	hh	0	hk	273	kk	0
>ref-91361	TCTACCCATACCTTCCCTCCCATCCTT	4	<hkxhk>	hh	62	hk	184	kk	0
>ref-91371	ATCAAGAAAACACGTGCTCCGCAGTTC	4	<lmxll>	ll	115	lm	146		
>ref-91371	ATCAAGAAAACACGTGCTCCGCAGTTC	7	<lmxll>	ll	116	lm	146		
>ref-91379	TCGGCTTACACGGTTCCTCCCACGCGG	3	<lmxll>	ll	138	lm	131		
>ref-91391	TTATCTCCAACCCCCTCTCCCCTCCCA	8	<lmxll>	ll	257	lm	0		
>ref-91391	TTATCTCCAACCCCCTCTCCCCTCCCA	16	<nnxnp>	nn	115	np	130		
>ref-91399	TGTTCTCCCACAAGGCCTCCAGTCCAC	5	<lmxll>	ll	120	lm	121		
>ref-91439	TTTTCCTCCACCTCCTCTCCCCCATTT	6	<nnxnp>	nn	228	np	0		
>ref-91446	CGGGAACCTACATCAGCTCCTCTTCCT	13	<nnxnp>	nn	133	np	136		
>ref-91446	CGGGAACCTACATCAGCTCCTCTTCCT	24	<nnxnp>	nn	133	np	135		
>ref-91449	GCCATGCTTACGTCTCCTCCAGTTCTG	24	<hkxhk>	hh	0	hk	153	kk	106
>ref-91462	AGAGTGTCTACACTAGCTCCCTGTGAC	16	<lmxll>	ll	128	lm	132		
>ref-91509	ACTTCGCCAACATCCCCTCCACTACTC	6	<lmxll>	ll	128	lm	127		
>ref-91513	CTGCCCCCTACCCATCCTCCTAGCTAC	8	<lmxll>	ll	152	lm	99		
>ref-91537	CACACCCTTACCCCTCCTCCTCCCCCC	2	<nnxnp>	nn	238	np	3		
>ref-91564	CACCGCTGCACCAGAGCTCCACCAAAT	22	<nnxnp>	nn	123	np	139		
>ref-91575	CTCCACCCTACTATTCCTCCCTCCTCC	2	<hkxhk>	hh	16	hk	246	kk	1
>ref-91575	CTCCACCCTACTATTCCTCCCTCCTCC	13	<hkxhk>	hh	1	hk	171	kk	86
>ref-91587	CCTTCCTACACATTTCCTCCCTCCCTC	7	<hkxhk>	hh	3	hk	246	kk	5
>ref-91587	CCTTCCTACACATTTCCTCCCTCCCTC	9	<hkxhk>	hh	3	hk	246	kk	5
>ref-91604	CTTTCCCGAACTTTGTCTCCATCGCAT	1	<lmxll>	ll	37	lm	220		
>ref-91604	CTTTCCCGAACTTTGTCTCCATCGCAT	25	<lmxll>	ll	72	lm	180		
>ref-91618	GCCCAAAGCACAAAGACTCCGTTACAT	1	<nnxnp>	nn	135	np	135		
>ref-91618	GCCCAAAGCACAAAGACTCCGTTACAT	2	<nnxnp>	nn	136	np	135		
>ref-91624	CATCCTCGTACAGCCGCTCCCTCTCCA	13	<hkxhk>	hh	51	hk	152	kk	62
>ref-91624	CATCCTCGTACAGCCGCTCCCTCTCCA	14	<hkxhk>	hh	52	hk	152	kk	62
>ref-91631	GCATGGGCAACAGCTTCTCCTCACATT	3	<lmxll>	ll	17	lm	253		
>ref-91631	GCATGGGCAACAGCTTCTCCTCACATT	4	<hkxhk>	hh	0	hk	273	kk	0
>ref-91631	GCATGGGCAACAGCTTCTCCTCACATT	15	<lmxll>	ll	17	lm	253		
>ref-91631	GCATGGGCAACAGCTTCTCCTCACATT	23	<lmxll>	ll	179	lm	78		

>ref-91631	GCATGGGCAACAGCTTCTCCTCACATT	25	<hkxhk>	hh	1	hk	272	kk	0
>ref-91669	GATTCCAGTACGTTTTCTCCCCGAAGT	14	<nnxnp>	nn	109	np	112		
>ref-91669	GATTCCAGTACGTTTTCTCCCCGAAGT	23	<nnxnp>	nn	109	np	112		
>ref-91694	TTAGCAACCACGGTCACTCCAGGCCAG	21	<lmxll>	ll	134	lm	129		
>ref-91705	ACGCTATCTACTTCCCCTCCATCGTCA	12	<nnxnp>	nn	120	np	147		
>ref-91739	CATATTCGTACTTTTTCTCCTTCGTTC	14	<lmxll>	ll	113	lm	121		
>ref-91743	TAGAATCTGACCCGAGCTCCGAGCTTC	13	<nnxnp>	nn	135	np	117		
>ref-91750	TAACACGAGACATTCCCTCCAAAACAC	23	<nnxnp>	nn	256	np	0		
>ref-91762	AAATAGGAGACATTCCCTCCCTTCTCT	1	<lmxll>	ll	246	lm	0		
>ref-91762	AAATAGGAGACATTCCCTCCCTTCTCT	7	<lmxll>	ll	126	lm	106		
>ref-91764	CAATATGCAACGTCCTCTCCTCATCAG	21	<hkxhk>	hh	52	hk	150	kk	61
>ref-91770	GTACCACTCACTGTTCCTCCGATTATT	8	<lmxll>	ll	109	lm	127		
>ref-91770	GTACCACTCACTGTTCCTCCGATTATT	24	<lmxll>	ll	110	lm	127		
>ref-91783	CATGAAATCACACGATCTCCTGAGATT	3	<hkxhk>	hh	0	hk	270	kk	0
>ref-91783	CATGAAATCACACGATCTCCTGAGATT	7	<hkxhk>	hh	0	hk	269	kk	0
>ref-91783	CATGAAATCACACGATCTCCTGAGATT	23	<hkxhk>	hh	9	hk	251	kk	0
>ref-91783	CATGAAATCACACGATCTCCTGAGATT	27	<hkxhk>	hh	0	hk	251	kk	9
>ref-91785	GCCATAACAACAACTGCTCCTTTGGTT	3	<hkxhk>	hh	3	hk	264	kk	1
>ref-91786	TGCACAGGTACCAGATCTCCCAGAGTG	22	<nnxnp>	nn	109	np	147		
>ref-91790	TCCACCACTACCCTATCTCCACCTCTC	2	<nnxnp>	nn	178	np	77		
>ref-91790	TCCACCACTACCCTATCTCCACCTCTC	9	<hkxhk>	hh	4	hk	266	kk	0
>ref-91790	TCCACCACTACCCTATCTCCACCTCTC	14	<hkxhk>	hh	0	hk	266	kk	4
>ref-91797	ATCAACTACACTCTCTCTCCTTCAATC	27	<nnxnp>	nn	112	np	120		
>ref-91820	TTTCCTCCTACTACTACTCCTACCACC	13	<nnxnp>	nn	69	np	172		
>ref-91820	TTTCCTCCTACTACTACTCCTACCACC	25	<nnxnp>	nn	68	np	172		
>ref-91821	AATGCCGAGACCCGAGCTCCACGAGCT	27	<nnxnp>	nn	260	np	0		
>ref-91824	CATACAAGAACACATTCTCCCTCTCAA	21	<nnxnp>	nn	127	np	137		
>ref-91848	CTTACGTTCACTTACCCTCCCCCCTTC	27	<lmxll>	ll	128	lm	135		
>ref-91865	TATTTCCATACATCCTCTCCTTGCCCT	14	<lmxll>	ll	135	lm	130		
>ref-91876	CTACCTTCCACTTTTTCTCCATCCCAT	16	<lmxll>	ll	121	lm	130		
>ref-91882	ACTGTGGCTACCTCTGCTCCCTGACCA	13	<lmxll>	ll	147	lm	123		
>ref-91925	AGTACCTGGACGAGATCTCCGAGGACG	3	<hkxhk>	hh	17	hk	244	kk	0
>ref-91958	TCCTAGCGCACTACTGCTCCCTCCAGT	4	<lmxll>	ll	116	lm	153		
>ref-91964	CTACTCCTCACCCTCTCTCCCTTCTTC	12	<lmxll>	ll	113	lm	151		
>ref-91964	CTACTCCTCACCCTCTCTCCCTTCTTC	21	<lmxll>	ll	113	lm	154		
>ref-91989	ACCTCTCTTACCTCCTCTCCCCTACCC	8	<nnxnp>	nn	112	np	118		
>ref-92003	CTCTCGTCCACCCCCACTCCCTCTATC	21	<lmxll>	ll	117	lm	140		
>ref-92003	CTCTCGTCCACCCCCACTCCCTCTATC	23	<nnxnp>	nn	138	np	113		

>ref-92003	CTCTCGTCCACCCCCACTCCCTCTATC	27	<nnxnp>	nn	146	np	115		
>ref-92024	CTCTCCCACACTCTTCCTCCCCCACAC	16	<hkxhk>	hh	93	hk	165	kk	0
>ref-92026	GGTAGGCATACGCGACCTCCCTCAGCC	9	<nnxnp>	nn	267	np	0		
>ref-92037	ATCACACAAACCCCTCCTCCCTCTGCG	9	<lmxll>	ll	136	lm	132		
>ref-92041	AAAGGGCCCACGTCCCCTCCCCATGCT	5	<lmxll>	ll	100	lm	155		
>ref-92072	AGGTCTCGCACAGGTCCTCCCTCCTTG	27	<lmxll>	ll	131	lm	138		
>ref-92083	CATGCATACACGTCCTCTCCGCCCCAT	21	<lmxll>	ll	120	lm	146		
>ref-92087	TGAACATGCACATGACCTCCAGGGACG	3	<nnxnp>	nn	104	np	154		
>ref-92087	TGAACATGCACATGACCTCCAGGGACG	6	<nnxnp>	nn	138	np	111		
>ref-92087	TGAACATGCACATGACCTCCAGGGACG	7	<hkxhk>	hh	0	hk	268	kk	3
>ref-92087	TGAACATGCACATGACCTCCAGGGACG	26	<nnxnp>	nn	168	np	85		
>ref-92087	TGAACATGCACATGACCTCCAGGGACG	27	<hkxhk>	hh	0	hk	240	kk	14
>ref-92096	ACTACAGCAACAAGCACTCCAATCACA	21	<hkxhk>	hh	73	hk	135	kk	52
>ref-92096	ACTACAGCAACAAGCACTCCAATCACA	25	<hkxhk>	hh	73	hk	136	kk	51
>ref-92102	TCTCTTATTACCGTCTCTCCTACCACT	22	<nnxnp>	nn	130	np	140		
>ref-92105	TTCACACCAACCACCTCTCCCGACACA	14	<nnxnp>	nn	104	np	117		
>ref-92161	AAGAAACTTACACATCCTCCTATACCA	5	<hkxhk>	hh	0	hk	270	kk	0
>ref-92161	AAGAAACTTACACATCCTCCTATACCA	13	<hkxhk>	hh	19	hk	229	kk	0
>ref-92161	AAGAAACTTACACATCCTCCTATACCA	14	<hkxhk>	hh	0	hk	270	kk	0
>ref-92161	AAGAAACTTACACATCCTCCTATACCA	25	<hkxhk>	hh	0	hk	229	kk	19
>ref-92164	TACTGTAAAACTCAATCTCCTCCTGGT	8	<hkxhk>	hh	2	hk	270	kk	0
>ref-92164	TACTGTAAAACTCAATCTCCTCCTGGT	23	<lmxll>	ll	19	lm	245		
>ref-92191	CATTAAATGACTTTTCCTCCTTTATTG	9	<hkxhk>	hh	29	hk	221	kk	0
>ref-92191	CATTAAATGACTTTTCCTCCTTTATTG	16	<hkxhk>	hh	29	hk	221	kk	0
>ref-92208	CCCTTTCCCACCACTTCTCCTTCATCA	24	<lmxll>	ll	121	lm	122		
>ref-92211	TGGTTTGCGACCGCCTCTCCTGGGAAA	8	<hkxhk>	hh	25	hk	227	kk	0
>ref-92211	TGGTTTGCGACCGCCTCTCCTGGGAAA	13	<nnxnp>	nn	16	np	249		
>ref-92233	ACCCCTTCCACCCCTTCTCCTCACCTC	6	<hkxhk>	hh	17	hk	165	kk	68
>ref-92271	TAGATAGATACTCTTTCTCCTGGTCCA	4	<hkxhk>	hh	55	hk	181	kk	11
>ref-92299	TTAATGACCACGAACGCTCCTTATGCA	13	<nnxnp>	nn	129	np	125		
>ref-92306	CCCGGGCACACTCCCCCTCCCTCCCAC	4	<lmxll>	ll	137	lm	116		
>ref-92313	TCAAGTTCCACTCCCTCTCCCTCACCG	3	<hkxhk>	hh	50	hk	145	kk	60
>ref-92313	TCAAGTTCCACTCCCTCTCCCTCACCG	5	<nnxnp>	nn	269	np	0		
>ref-92314	CACACCTATACGACTCCTCCGATTCTC	8	<hkxhk>	hh	56	hk	137	kk	58
>ref-92322	CTATCTATCACTCTATCTCCCCACCTA	1	<lmxll>	ll	244	lm	1		
>ref-92340	TCCATCCGAACGCTAACTCCCTTAGTT	4	<nnxnp>	nn	132	np	135		
>ref-92360	AAAGATCGTACAATTTCTCCAAAACCA	6	<nnxnp>	nn	135	np	95		
>ref-92361	AATGTTATCACCCACTCTCCAAGAAAG	22	<lmxll>	ll	130	lm	106		

>ref-92417	CCTTCCCCCACTTTCCCTCCCTTTTTC	2	<nnxnp>	nn	228	np	1		
>ref-92418	ACAGGAGCAACCCGCCCTCCGTTACCA	2	<nnxnp>	nn	108	np	154		
>ref-92443	CACTCTCCCACCATCACTCCCGCATTA	13	<hkxhk>	hh	55	hk	127	kk	52
>ref-92445	AAGACGGTCACAGTTTCTCCTCCAGCG	12	<hkxhk>	hh	59	hk	132	kk	55
>ref-92452	GTTGGTGACACTCCTCCTCCTTTCGTC	13	<lmxll>	ll	142	lm	118		
>ref-92462	CCTCCAACCACACAACCTCCACCTTCG	25	<lmxll>	ll	6	lm	264		
>ref-92470	CTCTTATTAACAAACGCTCCTCTTACC	3	<nnxnp>	nn	221	np	0		
>ref-92496	AGAGAGAACACAAGATCTCCCGACAAG	7	<hkxhk>	hh	67	hk	145	kk	55
>ref-92498	CACGCCGGGACCGAGGCTCCCTGCATC	2	<hkxhk>	hh	0	hk	267	kk	0
>ref-92498	CACGCCGGGACCGAGGCTCCCTGCATC	16	<hkxhk>	hh	0	hk	269	kk	0
>ref-92498	CACGCCGGGACCGAGGCTCCCTGCATC	21	<hkxhk>	hh	25	hk	241	kk	0
>ref-92501	CTCTCTCTTACTCTTTCTCCTCTGTAG	12	<lmxll>	ll	99	lm	162		
>ref-92503	TCTATCCACACCCTCTCTCCTCTCTCC	5	<lmxll>	ll	244	lm	1		
>ref-92508	CTGTTTCCCACCCTCACTCCAAACTTC	9	<lmxll>	ll	124	lm	125		
>ref-92508	CTGTTTCCCACCCTCACTCCAAACTTC	12	<lmxll>	ll	138	lm	112		
>ref-92508	CTGTTTCCCACCCTCACTCCAAACTTC	13	<nnxnp>	nn	152	np	108		
>ref-92508	CTGTTTCCCACCCTCACTCCAAACTTC	14	<hkxhk>	hh	0	hk	270	kk	2
>ref-92523	CTCATGCCCACGTCATCTCCGTACACA	24	<nnxnp>	nn	138	np	129		
>ref-92540	CTCCCCTCCACTTCCCCTCCCGCCCTT	1	<hkxhk>	hh	1	hk	266	kk	0
>ref-92544	TTTGTTTTTACTGTTCCTCCTCCTCCT	25	<hkxhk>	hh	66	hk	139	kk	39
>ref-92548	AGAACGACGACTGCTGCTCCTCCGCTC	23	<nnxnp>	nn	265	np	0		
>ref-92577	GACAATACCACTGACACTCCCCCGCCC	8	<lmxll>	ll	112	lm	121		
>ref-92583	TCCATTGGCACGAACACTCCCTTCTCC	26	<lmxll>	ll	133	lm	102		
>ref-92627	TGTTCCCAAACTTGGCCTCCGGAATTC	5	<lmxll>	ll	66	lm	154		
>ref-92653	CCTAGATCCACACTTCCTCCCCTCCCC	26	<nnxnp>	nn	254	np	0		
>ref-92665	TAAACACGTACCTAGGCTCCCTTATAG	7	<hkxhk>	hh	71	hk	146	kk	42
>ref-92666	TACTTCCGCACTCCCCCTCCCCCGTTC	5	<hkxhk>	hh	0	hk	203	kk	45
>ref-92685	CATGTTCCAACTCTCCCTCCTCTCTTT	9	<nnxnp>	nn	142	np	125		
>ref-92695	ACAGAAGACACTATCTCTCCCCATGGT	15	<nnxnp>	nn	122	np	115		
>ref-92700	TGATTTCCTACAGATCCTCCTCCTGGT	26	<nnxnp>	nn	128	np	129		
>ref-92703	TCCTCTCTGACCAGGACTCCGGGTATG	1	<lmxll>	ll	9	lm	258		
>ref-92703	TCCTCTCTGACCAGGACTCCGGGTATG	4	<lmxll>	ll	9	lm	258		
>ref-92703	TCCTCTCTGACCAGGACTCCGGGTATG	16	<nnxnp>	nn	124	np	143		
>ref-92714	AAACCTGAAACTATACCTCCCGTGACG	27	<nnxnp>	nn	132	np	120		
>ref-92722	CAGGTCATGACTTCTCCTCCTATTAGT	24	<lmxll>	ll	126	lm	130		
>ref-92727	CCCCTTCCTACTTTCTCTCCCCTTGTT	6	<nnxnp>	nn	144	np	112		
>ref-92728	ATACCGATAACGATATCTCCAACAACG	4	<nnxnp>	nn	119	np	145		
>ref-92728	ATACCGATAACGATATCTCCAACAACG	27	<nnxnp>	nn	118	np	145		

>ref-92730	CCATTTGCAACAGAAGCTCCTGGTGCC	13	<nnxnp>	nn	111	np	151		
>ref-92730	CCATTTGCAACAGAAGCTCCTGGTGCC	15	<nnxnp>	nn	111	np	151		
>ref-92768	CCGTTGCGCACTGTATCTCCCTTGGCC	6	<nnxnp>	nn	109	np	131		
>ref-92779	ACCTATTTTACCCTTTCTCCTTTCCAT	26	<hkxhk>	hh	62	hk	127	kk	55
>ref-92780	AATTACAATACTACTCCTCCTTCTCCT	26	<hkxhk>	hh	46	hk	139	kk	56
>ref-92780	AATTACAATACTACTCCTCCTTCTCCT	27	<hkxhk>	hh	55	hk	137	kk	46
>ref-92808	GGAGACCCTACCTTCCCTCCGTGTAAA	24	<nnxnp>	nn	125	np	126		
>ref-92821	GTAGTTGCCACTTGCCCTCCACATCAA	22	<lmxll>	ll	131	lm	138		
>ref-92830	TGCTTATGTACACACTCTCCACTCTCT	21	<lmxll>	ll	110	lm	150		
>ref-92848	TATTTGCATACTTTTCCTCCTCCTCAT	6	<lmxll>	ll	261	lm	0		
>ref-92848	TATTTGCATACTTTTCCTCCTCCTCAT	9	<nnxnp>	nn	263	np	0		
>ref-92848	TATTTGCATACTTTTCCTCCTCCTCAT	12	<lmxll>	ll	263	lm	0		
>ref-92854	CTCTATCCTACTCCCTCTCCCACTGTT	25	<hkxhk>	hh	72	hk	169	kk	0
>ref-92874	CACACGCGCACACGACCTCCATGCCAT	4	<nnxnp>	nn	123	np	150		
>ref-92874	CACACGCGCACACGACCTCCATGCCAT	6	<nnxnp>	nn	144	np	127		
>ref-92874	CACACGCGCACACGACCTCCATGCCAT	21	<nnxnp>	nn	144	np	127		
>ref-92878	CTCCCACACACTTTCCCTCCCTCATTC	7	<lmxll>	ll	99	lm	135		
>ref-92878	CTCCCACACACTTTCCCTCCCTCATTC	24	<lmxll>	ll	76	lm	152		
>ref-92907	CACTACCATACTCCCCCTCCCCCCACA	3	<nnxnp>	nn	135	np	123		
>ref-92910	TCTTTCCCCACTTTCTCTCCTACACAT	12	<nnxnp>	nn	106	np	129		
>ref-92914	TCCTTTCATACAGTGTCTCCCAACGCC	12	<hkxhk>	hh	50	hk	166	kk	45
>ref-92917	ATCATCATCACCATCACTCCTCCTTCT	3	<nnxnp>	nn	104	np	146		
>ref-92917	ATCATCATCACCATCACTCCTCCTTCT	5	<lmxll>	ll	0	lm	272		
>ref-92917	ATCATCATCACCATCACTCCTCCTTCT	22	<hkxhk>	hh	2	hk	269	kk	0
>ref-92917	ATCATCATCACCATCACTCCTCCTTCT	24	<hkxhk>	hh	0	hk	269	kk	2
>ref-92925	CACCACCTTACCTATCCTCCATCACTA	9	<lmxll>	ll	104	lm	140		
>ref-92925	CACCACCTTACCTATCCTCCATCACTA	22	<lmxll>	ll	104	lm	140		
>ref-92928	CCCCGATCCACCCCTTCTCCCACTACA	12	<nnxnp>	nn	105	np	133		
>ref-92931	CCCTTTCTCACCCTTTCTCCCGTCTCT	16	<nnxnp>	nn	126	np	124		
>ref-92931	CCCTTTCTCACCCTTTCTCCCGTCTCT	21	<nnxnp>	nn	122	np	123		
>ref-92938	GTCGCTCACACACACACTCCCTCACTC	7	<hkxhk>	hh	0	hk	273	kk	0
>ref-92980	AAGGACAGGACAGAGCCTCCAATCCCA	27	<hkxhk>	hh	0	hk	272	kk	0
>ref-92996	TTCCCATCTACACCCCCTCCCTTCCTC	5	<nnxnp>	nn	272	np	0		
>ref-92996	TTCCCATCTACACCCCCTCCCTTCCTC	24	<nnxnp>	nn	272	np	0		
>ref-93017	CCTGACCTCACTTTCACTCCGGGCCGA	9	<hkxhk>	hh	38	hk	218	kk	0
>ref-93017	CCTGACCTCACTTTCACTCCGGGCCGA	14	<nnxnp>	nn	228	np	39		
>ref-93017	CCTGACCTCACTTTCACTCCGGGCCGA	24	<nnxnp>	nn	126	np	133		
>ref-93017	CCTGACCTCACTTTCACTCCGGGCCGA	26	<hkxhk>	hh	0	hk	135	kk	127

>ref-93034	TCTGAGTGAACAGACTCTCCTCCTACT	14	<lmxll>	ll	125	lm	140		
>ref-93045	GTAGAACGTACAATATCTCCTCATCTG	1	<lmxll>	ll	102	lm	142		
>ref-93074	TTCCCTTCCACCGCCCCTCCCCACGTT	7	<lmxll>	ll	131	lm	133		
>ref-93074	TTCCCTTCCACCGCCCCTCCCCACGTT	23	<hkxhk>	hh	67	hk	201	kk	0
>ref-93095	AACCTTTGAACGAGTGCTCCTCAGTCC	21	<lmxll>	ll	141	lm	127		
>ref-93096	TAGAGTCATACCCATTCTCCTATAACT	13	<lmxll>	ll	114	lm	133		
>ref-93097	GACACCAAGACACGCTCTCCCGTCGAC	25	<lmxll>	ll	108	lm	156		
>ref-93132	CTCACCCTCACGCTCTCTCCTCTTATC	24	<nnxnp>	nn	132	np	138		
>ref-93145	CATCCCTCCACCCCCCCTCCTATCCCA	4	<lmxll>	ll	114	lm	106		
>ref-93157	GAGTGGATGACCCCCCCTCCTCTCCAC	16	<nnxnp>	nn	105	np	145		
>ref-93174	ATGCTCGCAACATATGCTCCAGTCATT	23	<hkxhk>	hh	45	hk	145	kk	66
>ref-93201	TCACCCAAAACACTTTCTCCCTCTCCT	1	<lmxll>	ll	145	lm	119		
>ref-93202	AGCATAAGCACGTCATCTCCGTCCCGG	22	<lmxll>	ll	136	lm	133		
>ref-93219	AGGTTCGTAACGAAGTCTCCTTATCAC	15	<nnxnp>	nn	117	np	109		
>ref-93220	CCCCTCCACACCTACCCTCCTCCCAAT	14	<nnxnp>	nn	261	np	0		
>ref-93220	CCCCTCCACACCTACCCTCCTCCCAAT	25	<nnxnp>	nn	259	np	0		
>ref-93234	CCACCCCCCACACCCTCTCCCTGCTCC	13	<lmxll>	ll	73	lm	182		
>ref-93265	CGCCTCGCCACGGCCCCTCCCAAGCCA	1	<nnxnp>	nn	268	np	0		
>ref-93268	CACACACCCACTCACCCTCCCTTTATA	8	<nnxnp>	nn	114	np	122		
>ref-93275	CGATTGGAAACGGATACTCCCATGTTG	21	<lmxll>	ll	121	lm	115		
>ref-93279	CCTCACCCTACGCCCCCTCCCCCCTCT	26	<nnxnp>	nn	128	np	112		
>ref-93279	CCTCACCCTACGCCCCCTCCCCCCTCT	27	<nnxnp>	nn	125	np	112		
>ref-93291	GAGACGGGTACACCAGCTCCTTAGCGG	6	<nnxnp>	nn	152	np	117		
>ref-93293	CGATTCCCAACTCCTTCTCCCTCTCTA	6	<nnxnp>	nn	138	np	125		
>ref-93293	CGATTCCCAACTCCTTCTCCCTCTCTA	14	<nnxnp>	nn	138	np	125		
>ref-93295	CTTTCTTACACTCTCCCTCCCCTTTCT	13	<hkxhk>	hh	57	hk	127	kk	43
>ref-93333	TCTAGCACTACACCGCCTCCATATCTA	5	<lmxll>	ll	258	lm	0		
>ref-93348	GGCCTGCATACCCTACCTCCCTCCCTT	15	<lmxll>	ll	260	lm	0		
>ref-93351	TCCGAGCCCACACTGTCTCCTAGAACA	5	<nnxnp>	nn	118	np	120		
>ref-93366	TTCTCGACGACTGCCTCTCCCGCTGTC	1	<nnxnp>	nn	271	np	0		
>ref-93366	TTCTCGACGACTGCCTCTCCCGCTGTC	5	<nnxnp>	nn	125	np	135		
>ref-93370	CCAAGCACCACGTCTTCTCCTTCGAGC	26	<hkxhk>	hh	0	hk	272	kk	0
>ref-93389	CAAACAAACACAACCCCTCCACAAACA	7	<nnxnp>	nn	8	np	243		
>ref-93389	CAAACAAACACAACCCCTCCACAAACA	9	<hkxhk>	hh	93	hk	140	kk	0
>ref-93389	CAAACAAACACAACCCCTCCACAAACA	14	<hkxhk>	hh	32	hk	216	kk	0
>ref-93389	CAAACAAACACAACCCCTCCACAAACA	15	<nnxnp>	nn	8	np	243		
>ref-93396	CAATCTCTTACATATACTCCCTGGAGC	5	<lmxll>	ll	143	lm	94		
>ref-93399	ATTCTCATCACCCTGTCTCCTCCCTCT	26	<lmxll>	ll	260	lm	0		

>ref-93399	ATTCTCATCACCCTGTCTCCTCCCTCT	27	<lmxll>	ll	257	lm	0		
>ref-93409	TGCTTATCCACCTTTTCTCCTTCGCCT	2	<lmxll>	ll	176	lm	61		
>ref-93409	TGCTTATCCACCTTTTCTCCTTCGCCT	22	<lmxll>	ll	176	lm	61		
>ref-93424	GATTCAATGACCTAACCTCCATTGTAG	14	<lmxll>	ll	102	lm	126		
>ref-93425	AGCAGCAGGACTCGCCCTCCACGTACT	8	<lmxll>	ll	2	lm	270		
>ref-93426	GTTGGACCGACCGAGCCTCCTGAAGAA	13	<hkxhk>	hh	58	hk	132	kk	61
>ref-93433	AATAACTAAACAAACACTCCTTGAAAT	7	<lmxll>	ll	109	lm	147		
>ref-93441	TCTGGGCCCACACTTTCTCCTGTGATC	1	<nnxnp>	nn	99	np	130		
>ref-93461	GAAAGTCGTACGTTTCCTCCGGTCATT	1	<lmxll>	ll	122	lm	116		
>ref-93461	GAAAGTCGTACGTTTCCTCCGGTCATT	2	<lmxll>	ll	121	lm	116		
>ref-93466	TCTCATTGCACATCTGCTCCCCTGTAG	21	<nnxnp>	nn	137	np	134		
>ref-93484	ATCTTCCTTACATTCACTCCTCCCCTT	2	<hkxhk>	hh	0	hk	269	kk	3
>ref-93484	ATCTTCCTTACATTCACTCCTCCCCTT	24	<hkxhk>	hh	2	hk	269	kk	0
>ref-93484	ATCTTCCTTACATTCACTCCTCCCCTT	27	<hkxhk>	hh	0	hk	273	kk	0
>ref-93488	TCGCTTCTTACTTTTCCTCCCTTTTCT	2	<hkxhk>	hh	20	hk	223	kk	0
>ref-93488	TCGCTTCTTACTTTTCCTCCCTTTTCT	21	<hkxhk>	hh	70	hk	158	kk	0
>ref-93507	CACATGCACACTGCTTCTCCCCGCCCA	8	<lmxll>	ll	122	lm	144		
>ref-93511	CTACACATAACCTTTTCTCCATGTAAT	26	<nnxnp>	nn	130	np	113		
>ref-93512	TACCCACCCACTCCCTCTCCTCTTATA	21	<lmxll>	ll	4	lm	254		
>ref-93512	TACCCACCCACTCCCTCTCCTCTTATA	25	<hkxhk>	hh	51	hk	185	kk	0
>ref-93519	ACCCCTTCTACTCTTTCTCCACTTCAT	14	<lmxll>	ll	123	lm	136		
>ref-93520	CTGCTAGATACTTCCTCTCCTTACCCT	6	<hkxhk>	hh	72	hk	111	kk	48
>ref-93532	GTCTGGGCTACCGCCTCTCCTTTATCC	27	<nnxnp>	nn	121	np	140		
>ref-93548	GCCATAATCACCCGCTCTCCTGCTCTC	22	<nnxnp>	nn	137	np	126		
>ref-93548	GCCATAATCACCCGCTCTCCTGCTCTC	25	<nnxnp>	nn	137	np	126		
>ref-93581	CTAGCAGTGACAAGGCCTCCTCGATGC	4	<lmxll>	ll	15	lm	248		
>ref-93613	TCGAGAAAAACTATGTCTCCTCTTTTG	5	<lmxll>	ll	166	lm	100		
>ref-93613	TCGAGAAAAACTATGTCTCCTCTTTTG	13	<lmxll>	ll	166	lm	100		
>ref-93618	CCAAAGTCAACTGTACCTCCCTGCCTG	8	<nnxnp>	nn	113	np	122		
>ref-93624	ACAAGATACACAGTCCCTCCAGAGAAA	9	<lmxll>	ll	119	lm	136		
>ref-93627	AGAGAAGGTACTGCTGCTCCATCCGAA	25	<nnxnp>	nn	147	np	119		
>ref-93632	GGGTTCCGTACCTCCCCTCCTCTCTCT	4	<lmxll>	ll	132	lm	129		
>ref-93644	ACTCTTAACACCTTCCCTCCTTCCTTC	13	<lmxll>	ll	120	lm	138		
>ref-93649	CTATCTATTACAGTGGCTCCCAAACAT	21	<nnxnp>	nn	64	np	196		
>ref-93651	GCACTTAAAACTAGTTCTCCATTGAGG	3	<nnxnp>	nn	136	np	123		
>ref-93655	AGAGGGCCTACCTGACCTCCCTTATTT	8	<nnxnp>	nn	122	np	147		
>ref-93667	CTCCCCCTTACTGTTGCTCCTCCCCTT	3	<nnxnp>	nn	122	np	125		
>ref-93667	CTCCCCCTTACTGTTGCTCCTCCCCTT	25	<nnxnp>	nn	121	np	124		

>ref-93674	CTCCTCCATACCTCTCCTCCCTCCCCA	3	<hkxhk>	hh	0	hk	269	kk	1
>ref-93674	CTCCTCCATACCTCTCCTCCCTCCCCA	9	<hkxhk>	hh	1	hk	269	kk	0
>ref-93702	CACCCCCTCACCCTATCTCCTACCTCC	14	<nnxnp>	nn	10	np	261		
>ref-93702	CACCCCCTCACCCTATCTCCTACCTCC	22	<nnxnp>	nn	10	np	261		
>ref-93703	TAATGTCATACCCCATCTCCCTAACCA	16	<lmxll>	ll	110	lm	115		
>ref-93739	CACCACTTTACCCTCCCTCCTACCCAA	4	<hkxhk>	hh	98	hk	135	kk	0
>ref-93739	CACCACTTTACCCTCCCTCCTACCCAA	25	<hkxhk>	hh	9	hk	246	kk	1
>ref-93739	CACCACTTTACCCTCCCTCCTACCCAA	27	<hkxhk>	hh	3	hk	266	kk	1
>ref-93748	CTGGTGTTTACACCTCCTCCTCCTCAC	4	<lmxll>	ll	124	lm	134		
>ref-93753	AACCCGGCGACTATCCCTCCCTCACAT	1	<hkxhk>	hh	63	hk	144	kk	51
>ref-93755	CACGGCTTGACTTGACCTCCCTGTGTA	25	<nnxnp>	nn	133	np	138		
>ref-93758	TCTATACATACACTATCTCCGTCTATT	21	<nnxnp>	nn	111	np	126		
>ref-93758	TCTATACATACACTATCTCCGTCTATT	23	<lmxll>	ll	111	lm	122		
>ref-93771	GTTACCAGAACGAGGTCTCCAGCGACA	5	<nnxnp>	nn	270	np	0		
>ref-93781	CGGGAATGTACTTCGGCTCCTGAGCAT	1	<lmxll>	ll	138	lm	131		
>ref-93813	TCTTCCCTAACCTCTTCTCCCTCCACC	24	<nnxnp>	nn	146	np	116		
>ref-93835	CTAGTCATCACCCATCCTCCTACCTAG	2	<hkxhk>	hh	5	hk	249	kk	1
>ref-93835	CTAGTCATCACCCATCCTCCTACCTAG	3	<hkxhk>	hh	1	hk	246	kk	5
>ref-93849	TGCTACCCTACTCCTTCTCCTATCCTT	25	<lmxll>	ll	134	lm	133		
>ref-93853	GTCGCAAAAACGGACACTCCCACGTAC	27	<hkxhk>	hh	54	hk	135	kk	61
>ref-93890	CGTCGTTCCACAATGGCTCCAAGCGAT	25	<nnxnp>	nn	111	np	140		
>ref-93899	TAATTGACAACAATGTCTCCCCATAGG	9	<lmxll>	ll	97	lm	150		
>ref-93934	TTCCTCTCCACTTCCTCTCCCTTCCTT	22	<nnxnp>	nn	21	np	247		
>ref-93942	AACTGTTATACTTAGACTCCCATAAAT	22	<hkxhk>	hh	58	hk	180	kk	3
>ref-93985	TGTTGGAGGACCGCTGCTCCTCCACCA	12	<nnxnp>	nn	111	np	156		
>ref-93986	CCTTTAAAAACATAACCTCCTTGTCTG	5	<lmxll>	ll	140	lm	99		
>ref-93986	CCTTTAAAAACATAACCTCCTTGTCTG	27	<lmxll>	ll	141	lm	99		
>ref-94003	CCTTCTCCAACGTCTTCTCCTTACCTC	13	<lmxll>	ll	107	lm	112		
>ref-94011	GCAAGCACAACATAGGCTCCCAAATAC	9	<nnxnp>	nn	271	np	0		
>ref-94015	ACACCTATTACTGCCGCTCCAAGCCCC	4	<lmxll>	ll	122	lm	143		
>ref-94018	CCCTTAGCTACTCCTCCTCCTGCCACC	23	<nnxnp>	nn	118	np	105		
>ref-94036	ACAACGGCTACCTCTTCTCCACTGTGG	1	<lmxll>	ll	248	lm	2		
>ref-94036	ACAACGGCTACCTCTTCTCCACTGTGG	3	<lmxll>	ll	257	lm	2		
>ref-94058	ATACTTGTCACCCTTCCTCCCCTCCCT	24	<lmxll>	ll	133	lm	136		
>ref-94073	ATCATACACACTCCCCCTCCCCCCCAC	9	<nnxnp>	nn	248	np	19		
>ref-94083	GACAGACAAACAAACACTCCTCTTGTC	5	<nnxnp>	nn	133	np	131		
>ref-94089	CACAGTGCCACGCCTACTCCTGGCATT	4	<hkxhk>	hh	68	hk	138	kk	64
>ref-94106	GCCCTGACCACCCTTTCTCCTTCTTCT	23	<nnxnp>	nn	145	np	128		

>ref-94107	GTTCTCTATACCCCCTCTCCCCTCTTC	1	<hkxhk>	hh	40	hk	198	kk	3
>ref-94107	GTTCTCTATACCCCCTCTCCCCTCTTC	26	<hkxhk>	hh	41	hk	200	kk	4
>ref-94124	CACCTCACCACGCAGCCTCCTAAACCT	2	<lmxll>	ll	136	lm	128		
>ref-94124	CACCTCACCACGCAGCCTCCTAAACCT	3	<lmxll>	ll	137	lm	128		
>ref-94129	CTCCCTCAAACAGTCCCTCCTTCTCTC	7	<nnxnp>	nn	225	np	35		
>ref-94153	CCCCCCTCCACCCCCTCTCCCTGCTCC	13	<lmxll>	ll	27	lm	219		
>ref-94158	ACTTCTAATACCCTTCCTCCCGTCAAT	14	<lmxll>	ll	131	lm	121		
>ref-94159	TCCTTTCACACTCACACTCCTCCACAT	2	<nnxnp>	nn	123	np	138		
>ref-94164	GCAATGTCAACTCGCCCTCCCTCACCA	24	<hkxhk>	hh	56	hk	154	kk	53
>ref-94175	TTTATAAGAACCACGACTCCTTTAACC	8	<hkxhk>	hh	69	hk	185	kk	3
>ref-94175	TTTATAAGAACCACGACTCCTTTAACC	21	<nnxnp>	nn	145	np	109		
>ref-94187	TTGCCATCCACCTTATCTCCTAACCCC	4	<hkxhk>	hh	0	hk	273	kk	0
>ref-94187	TTGCCATCCACCTTATCTCCTAACCCC	13	<hkxhk>	hh	0	hk	273	kk	0
>ref-94193	AAAACACCCACACAGGCTCCTCATTCC	27	<lmxll>	ll	126	lm	119		
>ref-94211	CCTTACGTAACCGAGTCTCCCTCAGGT	21	<lmxll>	ll	130	lm	127		
>ref-94253	CCTTATCAAACCCTCTCTCCCTCTTTC	6	<hkxhk>	hh	3	hk	264	kk	3
>ref-94253	CCTTATCAAACCCTCTCTCCCTCTTTC	21	<hkxhk>	hh	3	hk	264	kk	3
>ref-94258	AGCTGAACTACAAGCCCTCCTCACAGA	2	<hkxhk>	hh	0	hk	80	kk	181
>ref-94258	AGCTGAACTACAAGCCCTCCTCACAGA	8	<nnxnp>	nn	238	np	25		
>ref-94258	AGCTGAACTACAAGCCCTCCTCACAGA	14	<lmxll>	ll	43	lm	219		
>ref-94258	AGCTGAACTACAAGCCCTCCTCACAGA	15	<hkxhk>	hh	30	hk	237	kk	0
>ref-94258	AGCTGAACTACAAGCCCTCCTCACAGA	22	<nnxnp>	nn	238	np	25		
>ref-94280	ACGAACCATACATGTTCTCCGTCCGTG	2	<nnxnp>	nn	106	np	130		
>ref-94296	ACAACACCAACAACAGCTCCAACACAC	1	<lmxll>	ll	87	lm	178		
>ref-94302	CTCTCCGTCACTTCCCCTCCTCCTTTG	5	<lmxll>	ll	128	lm	144		
>ref-94314	TGGACAAAGACTGACTCTCCCAGGCCC	5	<lmxll>	ll	155	lm	100		
>ref-94317	GAGACAGGCACAGGACCTCCAACTAAG	6	<hkxhk>	hh	57	hk	140	kk	67
>ref-94319	CCCTTTCTCACGCCTCCTCCCTTCCCC	26	<lmxll>	ll	125	lm	125		
>ref-94327	CCTGGGACGACCACTTCTCCTCCATCG	22	<lmxll>	ll	246	lm	0		
>ref-94341	TTAGCGATAACCTTGACTCCCGCAGCA	27	<lmxll>	ll	142	lm	120		
>ref-94347	CTACCTCCAACTCCTCCTCCTCCTGCC	25	<hkxhk>	hh	1	hk	270	kk	0
>ref-94347	CTACCTCCAACTCCTCCTCCTCCTGCC	27	<hkxhk>	hh	1	hk	271	kk	0
>ref-94358	ATTGTCTCCACGTCGCCTCCCACCCGA	24	<nnxnp>	nn	272	np	0		
>ref-94359	CACGATAAAACGACAGCTCCATCTACA	13	<lmxll>	ll	121	lm	147		
>ref-94367	TCACCGGCGACTCCCTCTCCCCGTCCC	22	<lmxll>	ll	123	lm	137		
>ref-94384	ATTGTTTATACGTCTTCTCCTGCATTC	25	<lmxll>	ll	130	lm	132		
>ref-94387	AACACCACCACGCCATCTCCATCACCA	2	<lmxll>	ll	4	lm	269		
>ref-94387	AACACCACCACGCCATCTCCATCACCA	4	<hkxhk>	hh	6	hk	262	kk	0

>ref-94387	AACACCACCACGCCATCTCCATCACCA	7	<hkxhk>	hh	30	hk	236	kk	0
>ref-94387	AACACCACCACGCCATCTCCATCACCA	22	<hkxhk>	hh	0	hk	273	kk	0
>ref-94389	ACAATACTCACTTCCTCTCCTCCTTCC	1	<nnxnp>	nn	22	np	235		
>ref-94389	ACAATACTCACTTCCTCTCCTCCTTCC	12	<nnxnp>	nn	22	np	236		
>ref-94394	TTACCCAGCACCCTCTCTCCATTATAC	1	<hkxhk>	hh	1	hk	236	kk	13
>ref-94394	TTACCCAGCACCCTCTCTCCATTATAC	16	<hkxhk>	hh	1	hk	236	kk	13
>ref-94401	TGACCACCTACTAAGGCTCCAGAGACT	21	<hkxhk>	hh	45	hk	216	kk	0
>ref-94401	TGACCACCTACTAAGGCTCCAGAGACT	24	<hkxhk>	hh	45	hk	216	kk	0
>ref-94401	TGACCACCTACTAAGGCTCCAGAGACT	27	<hkxhk>	hh	0	hk	273	kk	0
>ref-94403	ATTAGCCACACAGCCCCTCCCTCTCTC	3	<lmxll>	ll	125	lm	142		
>ref-94403	ATTAGCCACACAGCCCCTCCCTCTCTC	4	<lmxll>	ll	124	lm	142		
>ref-94407	CAAGCATACACTTTTACTCCTACATTA	12	<lmxll>	ll	117	lm	131		
>ref-94407	CAAGCATACACTTTTACTCCTACATTA	22	<lmxll>	ll	117	lm	131		
>ref-94425	GCATGACTCACCTGCCCTCCCGACAAA	5	<hkxhk>	hh	66	hk	149	kk	47
>ref-94425	GCATGACTCACCTGCCCTCCCGACAAA	12	<lmxll>	ll	127	lm	138		
>ref-94435	CTGGCTCTCACTCCCTCTCCCCCTACT	15	<lmxll>	ll	128	lm	129		
>ref-94439	GAGGACTTCACTGCACCTCCCTTATCG	16	<lmxll>	ll	139	lm	130		
>ref-94439	GAGGACTTCACTGCACCTCCCTTATCG	21	<lmxll>	ll	119	lm	147		
>ref-94474	TCATTCTAAACCCCTTCTCCCTCATTT	27	<hkxhk>	hh	0	hk	263	kk	3
>ref-94476	TCCCGTGTAACTGAGGCTCCTCTGTGT	3	<hkxhk>	hh	82	hk	120	kk	54
>ref-94478	AATTACAATACACACGCTCCATGCAAA	27	<hkxhk>	hh	21	hk	232	kk	2
>ref-94487	AGGCGCATGACAAGAGCTCCTGAAGTG	3	<lmxll>	ll	141	lm	129		
>ref-94524	TCCCCTCTCACCTTTTCTCCTTTCCTC	16	<lmxll>	ll	0	lm	268		
>ref-94524	TCCCCTCTCACCTTTTCTCCTTTCCTC	21	<lmxll>	ll	0	lm	268		
>ref-94526	CTCCTTTTTACTTTCTCTCCTTCTGTC	15	<nnxnp>	nn	107	np	118		
>ref-94580	CCCCATTCCACCGCCGCTCCACTTCCT	13	<lmxll>	ll	45	lm	204		
>ref-94650	TTAATGAGGACTTAACCTCCTTAGGAA	1	<lmxll>	ll	133	lm	121		
>ref-94652	TCTCCTCTCACCCTCACTCCTTTCTTC	4	<lmxll>	ll	267	lm	0		
>ref-94653	CGAACGAAGACAGAGCCTCCTTGCATC	4	<hkxhk>	hh	62	hk	135	kk	54
>ref-94653	CGAACGAAGACAGAGCCTCCTTGCATC	6	<hkxhk>	hh	54	hk	135	kk	63
>ref-94673	CAGAGTTCAACGGCACCTCCTCTTGCG	15	<lmxll>	ll	137	lm	134		
>ref-94673	CAGAGTTCAACGGCACCTCCTCTTGCG	23	<nnxnp>	nn	127	np	146		
>ref-94692	TCCACCTACACTAACCCTCCCTCCATC	12	<hkxhk>	hh	0	hk	215	kk	28
>ref-94692	TCCACCTACACTAACCCTCCCTCCATC	15	<lmxll>	ll	20	lm	228		
>ref-94692	TCCACCTACACTAACCCTCCCTCCATC	22	<lmxll>	ll	21	lm	227		
>ref-94706	ATACACTCTACTGCGTCTCCCGACTCT	2	<nnxnp>	nn	131	np	135		
>ref-94710	CCTCCCCTTACTTTGTCTCCTCCACTT	1	<hkxhk>	hh	0	hk	111	kk	124
>ref-94710	CCTCCCCTTACTTTGTCTCCTCCACTT	5	<lmxll>	ll	118	lm	106		

>ref-94714	TCCCCTGTTACTCTTCCTCCCCCCAAA	7	<hkxhk>	hh	92	hk	165	kk	0
>ref-94718	TCGTTGCTTACCCTTCCTCCAGTTCCC	7	<lmxll>	ll	138	lm	118		
>ref-94719	ATTCCTAATACTCCCTCTCCCTTTCCC	4	<lmxll>	ll	132	lm	120		
>ref-94749	TACTTCATAACCCCACCTCCACCTGCT	21	<hkxhk>	hh	57	hk	139	kk	47
>ref-94764	ATTTCCTTCACCACTCCTCCCCTCCTT	12	<nnxnp>	nn	262	np	0		
>ref-94767	ACACTGTTCACGTGTTCTCCTCGCTAA	24	<lmxll>	ll	125	lm	143		
>ref-94769	CTCCAGTATACTTGCGCTCCCTCACTT	4	<lmxll>	ll	131	lm	134		
>ref-94769	CTCCAGTATACTTGCGCTCCCTCACTT	5	<lmxll>	ll	130	lm	134		
>ref-94770	TAGATCATCACTGAATCTCCAACTTGT	9	<nnxnp>	nn	127	np	135		
>ref-94788	TCTTGGTCCACACAGCCTCCTATGCAC	2	<nnxnp>	nn	131	np	120		
>ref-94790	CATTCCATCACTCCAACTCCTTAAGCA	4	<hkxhk>	hh	62	hk	128	kk	71
>ref-94822	TTTCTCTACACCCGCCCTCCCTTCTCG	8	<hkxhk>	hh	62	hk	131	kk	56
>ref-94822	TTTCTCTACACCCGCCCTCCCTTCTCG	14	<nnxnp>	nn	117	np	130		
>ref-94829	ACACAAACGACTCAAGCTCCTTTGTTT	6	<nnxnp>	nn	131	np	135		
>ref-94838	TATCGCGCTACATGACCTCCCCACTTC	21	<hkxhk>	hh	48	hk	148	kk	61
>ref-94873	CTCTCCTTTACTCCACCTCCCTTTTCG	4	<lmxll>	ll	64	lm	171		
>ref-94873	CTCTCCTTTACTCCACCTCCCTTTTCG	21	<lmxll>	ll	128	lm	105		
>ref-94882	AGCAGGACCACGCAGCCTCCTCGTCTC	5	<nnxnp>	nn	124	np	137		
>ref-94883	CACTCACCAACGTACTCTCCCTCACTC	12	<hkxhk>	hh	58	hk	160	kk	48
>ref-94893	CACGTACTCACTTACCCTCCTCATACA	5	<nnxnp>	nn	135	np	84		
>ref-94915	CCCCGAGCAACATTTTCTCCTATGTCG	22	<nnxnp>	nn	119	np	129		
>ref-94945	GTCCCTTGCACTCTCCCTCCCACCTTT	1	<nnxnp>	nn	0	np	269		
>ref-94965	TCCCATACCACCTCATCTCCTTGCTCA	24	<hkxhk>	hh	69	hk	129	kk	63
>ref-94969	AAGTCGATAACTCTATCTCCTTCTTCA	4	<hkxhk>	hh	0	hk	258	kk	6
>ref-94969	AAGTCGATAACTCTATCTCCTTCTTCA	5	<hkxhk>	hh	7	hk	258	kk	0
>ref-94969	AAGTCGATAACTCTATCTCCTTCTTCA	15	<lmxll>	ll	16	lm	246		
>ref-94977	TCTTATAACACGTGCGCTCCTCCGTGT	22	<lmxll>	ll	234	lm	29		
>ref-94983	TGGGTGAATACGTGCTCTCCTTTGGTG	3	<lmxll>	ll	113	lm	144		
>ref-94986	TCCTTAATTACTTCCTCTCCTTTGTCC	4	<lmxll>	ll	118	lm	133		
>ref-94992	TCCCGGAAGACCGCGCCTCCGCCAAAC	1	<hkxhk>	hh	0	hk	268	kk	0
>ref-94992	TCCCGGAAGACCGCGCCTCCGCCAAAC	22	<nnxnp>	nn	268	np	0		
>ref-94999	ACACACCTTACACGATCTCCTCCGTCT	22	<hkxhk>	hh	62	hk	163	kk	45
>ref-95018	ATACCGGAAACGTCAGCTCCAATTAAA	15	<hkxhk>	hh	47	hk	134	kk	58
>ref-95074	ACTTCCTTCACCCACTCTCCAGCGCCC	13	<lmxll>	ll	4	lm	267		
>ref-95074	ACTTCCTTCACCCACTCTCCAGCGCCC	21	<hkxhk>	hh	91	hk	175	kk	0
>ref-95074	ACTTCCTTCACCCACTCTCCAGCGCCC	22	<lmxll>	ll	48	lm	218		
>ref-95096	ACTACTCTCACAGTGGCTCCACTACAG	1	<lmxll>	ll	125	lm	136		
>ref-95139	CCCACCCCTACCAACCCTCCCATCTCC	9	<nnxnp>	nn	129	np	110		

>ref-95147	CTCTCTTTCACACTGACTCCTTCTCCT	9	<lmxll>	ll	131	lm	99		
>ref-95163	TCCTCGCTAACTCTCACTCCTTCCTTT	7	<lmxll>	ll	125	lm	144		
>ref-95163	TCCTCGCTAACTCTCACTCCTTCCTTT	16	<lmxll>	ll	124	lm	144		
>ref-95170	CTATTGACCACGCCCCCTCCTCTCTAT	2	<nnxnp>	nn	132	np	138		
>ref-95173	CCCTCTCTTACCTCACCTCCTCCTTCC	9	<nnxnp>	nn	258	np	0		
>ref-95180	TATTACAATACACCTACTCCCAACACA	1	<nnxnp>	nn	118	np	132		
>ref-95182	AGGTCATACACTTCTCCTCCCCCTTCA	12	<nnxnp>	nn	119	np	114		
>ref-95205	CCTTATTCCACCATTCCTCCTGTTCAG	1	<nnxnp>	nn	58	np	191		
>ref-95205	CCTTATTCCACCATTCCTCCTGTTCAG	3	<lmxll>	ll	175	lm	82		
>ref-95205	CCTTATTCCACCATTCCTCCTGTTCAG	27	<hkxhk>	hh	0	hk	245	kk	12
>ref-95214	CACCCTTACACTCTCTCTCCTTCCCTT	12	<nnxnp>	nn	136	np	130		
>ref-95227	CTCCTGTTCACCCTCCCTCCTCCCTTC	7	<lmxll>	ll	259	lm	2		
>ref-95227	CTCCTGTTCACCCTCCCTCCTCCCTTC	9	<lmxll>	ll	263	lm	0		
>ref-95234	TTTTCCTTAACTTCTTCTCCCTTTTCT	13	<lmxll>	ll	8	lm	243		
>ref-95234	TTTTCCTTAACTTCTTCTCCCTTTTCT	25	<lmxll>	ll	9	lm	243		
>ref-95279	TTCCTTCTCACCTATTCTCCTCCTTCT	12	<nnxnp>	nn	106	np	140		
>ref-95279	TTCCTTCTCACCTATTCTCCTCCTTCT	13	<nnxnp>	nn	109	np	137		
>ref-95300	CTATCTATCACCTTGACTCCCTCTCTT	27	<lmxll>	ll	91	lm	165		
>ref-95315	TCCTTTCTAACCAATCCTCCCTCCCTC	23	<nnxnp>	nn	110	np	130		
>ref-95328	GCTTCCTTTACCCTTCCTCCTGCTTCC	12	<hkxhk>	hh	1	hk	270	kk	0
>ref-95341	CTCCCGTTTACTTTTACTCCTCATTCA	8	<nnxnp>	nn	244	np	0		
>ref-95346	TTGCTGCGTACTTGCGCTCCTTGCGTG	26	<lmxll>	ll	269	lm	0		
>ref-95370	TTCATGCGTACCGGTTCTCCTTGTGTG	22	<nnxnp>	nn	117	np	149		
>ref-95372	TCTAAACGAACCTCATCTCCCTAGCAT	1	<nnxnp>	nn	272	np	0		
>ref-95372	TCTAAACGAACCTCATCTCCCTAGCAT	2	<nnxnp>	nn	272	np	0		
>ref-95373	CATATATTCACTGAAGCTCCAGCCTAA	2	<lmxll>	ll	143	lm	122		
>ref-95381	GTGGAGAAGACTTACTCTCCTGTCTCA	3	<hkxhk>	hh	0	hk	238	kk	22
>ref-95381	GTGGAGAAGACTTACTCTCCTGTCTCA	9	<lmxll>	ll	183	lm	74		
>ref-95381	GTGGAGAAGACTTACTCTCCTGTCTCA	12	<hkxhk>	hh	0	hk	168	kk	73
>ref-95381	GTGGAGAAGACTTACTCTCCTGTCTCA	24	<lmxll>	ll	183	lm	73		
>ref-95381	GTGGAGAAGACTTACTCTCCTGTCTCA	27	<hkxhk>	hh	72	hk	170	kk	0
>ref-95386	CCACGCCTTACGGCCACTCCTTCACTC	12	<nnxnp>	nn	127	np	140		
>ref-95443	CACTCGTTGACCCTCACTCCTCACAAT	15	<lmxll>	ll	130	lm	124		
>ref-95450	AACCTCAAGACAGGGCCTCCTGCGTTG	12	<nnxnp>	nn	127	np	146		
>ref-95453	ATCGACTCCACCTCACCTCCACATCTT	6	<nnxnp>	nn	266	np	0		
>ref-95470	TTAGGGCCTACTGCTGCTCCAAGAGCC	3	<lmxll>	ll	55	lm	193		
>ref-95470	TTAGGGCCTACTGCTGCTCCAAGAGCC	26	<lmxll>	ll	235	lm	24		
>ref-95470	TTAGGGCCTACTGCTGCTCCAAGAGCC	27	<hkxhk>	hh	61	hk	192	kk	0

>ref-95510	TCACCTGGCACCTCTTCTCCTCTCCCT	14	<lmxll>	ll	152	lm	115		
>ref-95526	GTGGGACGGACTTCATCTCCAGTACAG	13	<lmxll>	ll	118	lm	146		
>ref-95526	GTGGGACGGACTTCATCTCCAGTACAG	14	<lmxll>	ll	139	lm	129		
>ref-95534	ATAACGGTGACTAGATCTCCTGTAATA	3	<nnxnp>	nn	113	np	140		
>ref-95541	CACCCCCCTACCCCTCCTCCCATCCCC	4	<hkxhk>	hh	14	hk	218	kk	8
>ref-95553	CCAACAATCACACCTCCTCCAACAATC	4	<hkxhk>	hh	153	hk	103	kk	0
>ref-95553	CCAACAATCACACCTCCTCCAACAATC	15	<hkxhk>	hh	0	hk	246	kk	21
>ref-95553	CCAACAATCACACCTCCTCCAACAATC	22	<hkxhk>	hh	75	hk	196	kk	0
>ref-95593	GTTGTCCAAACAGTTACTCCATTTTCT	15	<nnxnp>	nn	117	np	141		
>ref-95603	CCACGTCCCACTCCTTCTCCCTCCCAC	13	<lmxll>	ll	125	lm	134		
>ref-95647	GTGGTTAGAACACTGACTCCGACCCTC	3	<lmxll>	ll	271	lm	1		
>ref-95647	GTGGTTAGAACACTGACTCCGACCCTC	8	<nnxnp>	nn	259	np	11		
>ref-95647	GTGGTTAGAACACTGACTCCGACCCTC	16	<lmxll>	ll	268	lm	4		
>ref-95655	ATATCTCTCACCCACCCTCCTATAGAA	16	<nnxnp>	nn	129	np	104		
>ref-95676	CCACCCCACACACACCCTCCACCCAAC	16	<lmxll>	ll	143	lm	93		
>ref-95676	CCACCCCACACACACCCTCCACCCAAC	22	<lmxll>	ll	143	lm	92		
>ref-95693	ACTACTCCTACCCAGCCTCCCCTTCCT	1	<lmxll>	ll	132	lm	130		
>ref-95697	CACCCCAACACCCTCCCTCCTACCCCA	21	<nnxnp>	nn	216	np	20		
>ref-95697	CACCCCAACACCCTCCCTCCTACCCCA	22	<nnxnp>	nn	211	np	20		
>ref-95730	CCCCTTCTGACCTCCCCTCCTCCTCAT	6	<lmxll>	ll	9	lm	263		
>ref-95730	CCCCTTCTGACCTCCCCTCCTCCTCAT	26	<lmxll>	ll	9	lm	263		
>ref-95741	TCTACTTCTACAATTTCTCCTTCGTTC	13	<hkxhk>	hh	10	hk	229	kk	1
>ref-95741	TCTACTTCTACAATTTCTCCTTCGTTC	27	<hkxhk>	hh	2	hk	239	kk	2
>ref-95754	GAATTCGTCACGGGATCTCCCAGAGTG	23	<lmxll>	ll	123	lm	116		
>ref-95782	GCAGAGCGCACATCTCCTCCACCTCCG	9	<lmxll>	ll	104	lm	135		
>ref-95782	GCAGAGCGCACATCTCCTCCACCTCCG	15	<nnxnp>	nn	104	np	131		
>ref-95782	GCAGAGCGCACATCTCCTCCACCTCCG	27	<nnxnp>	nn	100	np	131		
>ref-95789	GCCCTTCAAACGCCTTCTCCTACCACA	21	<hkxhk>	hh	44	hk	144	kk	66
>ref-95823	TCTCTCTCGACCCTTCCTCCTGCTCTC	6	<nnxnp>	nn	126	np	121		
>ref-95834	TTGCGGTCGACAACGCCTCCGCCAGTG	24	<nnxnp>	nn	122	np	141		
>ref-95836	TTGTGTGAGACGTTTCCTCCGGCGGCA	25	<nnxnp>	nn	121	np	145		
>ref-95839	ATGTCTCTCACTCAATCTCCCATTCCC	3	<hkxhk>	hh	49	hk	161	kk	55
>ref-95839	ATGTCTCTCACTCAATCTCCCATTCCC	5	<nnxnp>	nn	270	np	0		
>ref-95850	AAACAGAAGACAAACGCTCCCACGGAC	25	<nnxnp>	nn	114	np	129		
>ref-95851	CCCAACCCCACCTACCCTCCCTTATCC	3	<hkxhk>	hh	0	hk	210	kk	51
>ref-95851	CCCAACCCCACCTACCCTCCCTTATCC	7	<hkxhk>	hh	21	hk	205	kk	0
>ref-95851	CCCAACCCCACCTACCCTCCCTTATCC	8	<nnxnp>	nn	101	np	148		
>ref-95862	CCTCCTTAGACTCCTCCTCCTTTTTC	1	<nnxnp>	nn	127	np	101		

>ref-95862	CCTCCTTAGACTCCTCCTCCTTTTTTC	2	<nnxnp>	nn	126	np	101		
>ref-95904	CCTCTGCTTACTCGTTCTCCTCATGTT	27	<nnxnp>	nn	262	np	0		
>ref-95917	TTCTCCCTTACCTTACCTCCCTCCTTA	6	<hkxhk>	hh	52	hk	141	kk	54
>ref-95927	CCCTAGGCCACTACCTCTCCCACGGTC	21	<nnxnp>	nn	128	np	135		
>ref-95941	TCCTCCCATACCCGGCCTCCTTCTCTA	3	<nnxnp>	nn	121	np	132		
>ref-95960	CATATTCAAACACTCTCTCCCATACAC	24	<nnxnp>	nn	124	np	129		
>ref-96003	GAGCCACTTACCCGTCCTCCTCCGCCG	1	<nnxnp>	nn	115	np	155		
>ref-96003	GAGCCACTTACCCGTCCTCCTCCGCCG	3	<nnxnp>	nn	115	np	155		
>ref-96006	CCTCCGGCGACCTGACCTCCGGCGACC	7	<lmxll>	ll	131	lm	124		
>ref-96006	CCTCCGGCGACCTGACCTCCGGCGACC	8	<lmxll>	ll	133	lm	123		
>ref-96006	CCTCCGGCGACCTGACCTCCGGCGACC	22	<hkxhk>	hh	0	hk	257	kk	14
>ref-96006	CCTCCGGCGACCTGACCTCCGGCGACC	23	<hkxhk>	hh	14	hk	257	kk	0
>ref-96006	CCTCCGGCGACCTGACCTCCGGCGACC	26	<hkxhk>	hh	16	hk	253	kk	0
>ref-96016	ACTCGCAGCACCTGTTCTCCGTGCCAT	1	<nnxnp>	nn	138	np	133		
>ref-96016	ACTCGCAGCACCTGTTCTCCGTGCCAT	5	<nnxnp>	nn	138	np	133		
>ref-96025	GTTGAGTGTACGAGTTCTCCTTTTATA	8	<hkxhk>	hh	30	hk	234	kk	0
>ref-96042	GACACCAACACTCTTCCTCCCGTCTTC	15	<lmxll>	ll	142	lm	125		
>ref-96059	GAACCATCTACTCCTCCTCCTCTCTCT	26	<nnxnp>	nn	269	np	0		
>ref-96072	CCCACAGCAACACACCCTCCCACATTC	1	<lmxll>	ll	19	lm	240		
>ref-96072	CCCACAGCAACACACCCTCCCACATTC	12	<lmxll>	ll	50	lm	202		
>ref-96072	CCCACAGCAACACACCCTCCCACATTC	26	<lmxll>	ll	49	lm	202		
>ref-96074	ACCTCTATCACACTCCCTCCTTTTCTC	13	<lmxll>	ll	130	lm	127		
>ref-96088	AGGCCATCTACTGTATCTCCCACATAG	26	<hkxhk>	hh	44	hk	124	kk	69
>ref-96088	AGGCCATCTACTGTATCTCCCACATAG	27	<hkxhk>	hh	69	hk	124	kk	43
>ref-96104	ATCTCCATCACCATCACTCCCATTCCA	7	<hkxhk>	hh	30	hk	227	kk	0
>ref-96104	ATCTCCATCACCATCACTCCCATTCCA	13	<lmxll>	ll	136	lm	132		
>ref-96104	ATCTCCATCACCATCACTCCCATTCCA	24	<nnxnp>	nn	129	np	136		
>ref-96104	ATCTCCATCACCATCACTCCCATTCCA	27	<hkxhk>	hh	0	hk	269	kk	1
>ref-96111	CCATCGCCTACCCCCCCTCCTCTCCCA	6	<nnxnp>	nn	220	np	19		
>ref-96111	CCATCGCCTACCCCCCCTCCTCTCCCA	21	<nnxnp>	nn	222	np	18		
>ref-96124	TCGTGACCTACTTCCGCTCCTCTAATC	3	<nnxnp>	nn	142	np	117		
>ref-96173	TGCGACCGCACGGGTCCTCCATCAGGC	16	<lmxll>	ll	119	lm	134		
>ref-96211	CGGCAGTTTACCCCTCCTCCCTCTGGT	27	<nnxnp>	nn	147	np	82		
>ref-96216	CCCCGCCACACCCCCTCTCCCCCTCTG	1	<lmxll>	ll	154	lm	86		
>ref-96216	CCCCGCCACACCCCCTCTCCCCCTCTG	5	<hkxhk>	hh	0	hk	201	kk	48
>ref-96216	CCCCGCCACACCCCCTCTCCCCCTCTG	26	<hkxhk>	hh	0	hk	257	kk	6
>ref-96216	CCCCGCCACACCCCCTCTCCCCCTCTG	27	<nnxnp>	nn	203	np	47		
>ref-96217	TTGTTTGTGACTCTTTCTCCAAACGCT	14	<nnxnp>	nn	115	np	130		

>ref-96219	CCCTAAATCACCTCGCCTCCCTTTGCA	16	<nnxnp>	nn	116	np	151		
>ref-96223	CCCGTTCCCACTTCTACTCCTCCTGCC	4	<hkxhk>	hh	0	hk	195	kk	64
>ref-96241	CCTTCTTCCACCCCTTCTCCCCCATCT	12	<lmxll>	ll	119	lm	127		
>ref-96241	CCTTCTTCCACCCCTTCTCCCCCATCT	13	<lmxll>	ll	126	lm	123		
>ref-96241	CCTTCTTCCACCCCTTCTCCCCCATCT	24	<nnxnp>	nn	121	np	118		
>ref-96275	AGCGTCCTAACGAAACCTCCTTTCCTG	3	<lmxll>	ll	144	lm	126		
>ref-96293	ATATCCCTCACCAACCCTCCTCCTCTC	1	<nnxnp>	nn	113	np	146		
>ref-96309	ACCCAGGTCACAGAGCCTCCGGGCTAG	8	<hkxhk>	hh	0	hk	273	kk	0
>ref-96309	ACCCAGGTCACAGAGCCTCCGGGCTAG	12	<lmxll>	ll	4	lm	269		
>ref-96309	ACCCAGGTCACAGAGCCTCCGGGCTAG	24	<hkxhk>	hh	51	hk	203	kk	0
>ref-96310	CACACGCTCACGCACACTCCCTCACTA	5	<nnxnp>	nn	146	np	126		
>ref-96327	GCACCCCTCACATGGTCTCCCTGCCAG	12	<lmxll>	ll	127	lm	132		
>ref-96333	CGGCTGTAAACTCCTCCTCCTTCGCTG	6	<lmxll>	ll	132	lm	124		
>ref-96363	ATTCCCCATACAATCTCTCCCTTCTTA	24	<nnxnp>	nn	129	np	121		
>ref-96363	ATTCCCCATACAATCTCTCCCTTCTTA	26	<nnxnp>	nn	128	np	121		
>ref-96368	CTCGCCCGTACGCCCACTCCCGCACCT	1	<lmxll>	ll	127	lm	145		
>ref-96368	CTCGCCCGTACGCCCACTCCCGCACCT	2	<lmxll>	ll	45	lm	217		
>ref-96368	CTCGCCCGTACGCCCACTCCCGCACCT	4	<lmxll>	ll	47	lm	217		
>ref-96370	TCCTCATTCACAGTCTCTCCTCAACAG	24	<hkxhk>	hh	49	hk	119	kk	63
>ref-96393	TTTCATCCCACCTCCTCTCCTCCTTCA	8	<nnxnp>	nn	255	np	0		
>ref-96398	ACTCCACCAACAGCAGCTCCACAATTA	16	<hkxhk>	hh	55	hk	144	kk	68
>ref-96412	CACAATACCACAAATTCTCCGGAGTTT	4	<lmxll>	ll	128	lm	115		
>ref-96421	TTATCTTTCACTTTTCCTCCTTTTATC	7	<hkxhk>	hh	0	hk	124	kk	108
>ref-96467	ACTGCCCCCACGCGCACTCCCGGGGTA	1	<lmxll>	ll	244	lm	20		
>ref-96467	ACTGCCCCCACGCGCACTCCCGGGGTA	2	<hkxhk>	hh	0	hk	273	kk	0
>ref-96467	ACTGCCCCCACGCGCACTCCCGGGGTA	8	<nnxnp>	nn	68	np	183		
>ref-96467	ACTGCCCCCACGCGCACTCCCGGGGTA	16	<nnxnp>	nn	155	np	103		
>ref-96467	ACTGCCCCCACGCGCACTCCCGGGGTA	21	<hkxhk>	hh	135	hk	116	kk	0
>ref-96467	ACTGCCCCCACGCGCACTCCCGGGGTA	22	<hkxhk>	hh	92	hk	155	kk	0
>ref-96467	ACTGCCCCCACGCGCACTCCCGGGGTA	25	<lmxll>	ll	245	lm	19		
>ref-96479	CCTCTCGCAACCCACTCTCCACCGCCT	9	<lmxll>	ll	149	lm	121		
>ref-96484	GCCCCTCTCACTCCTCCTCCTATCTTT	12	<nnxnp>	nn	239	np	0		
>ref-96496	TTATTATCCACTTATTCTCCTTCCTTT	3	<lmxll>	ll	6	lm	253		
>ref-96496	TTATTATCCACTTATTCTCCTTCCTTT	27	<lmxll>	ll	6	lm	252		
>ref-96508	TGTGTTGTTACCTCCCCTCCTCCTCCC	23	<hkxhk>	hh	64	hk	140	kk	62
>ref-96510	AGTTCCTCAACCTTTCCTCCTCCACAC	23	<lmxll>	ll	133	lm	131		
>ref-96512	TCCTTCCATACCCTCCCTCCTCTCTTC	5	<lmxll>	ll	41	lm	211		
>ref-96512	TCCTTCCATACCCTCCCTCCTCTCTTC	8	<hkxhk>	hh	0	hk	273	kk	0

>ref-96512	TCCTTCCATACCCTCCCTCCTCTCTTC	9	<hkxhk>	hh	0	hk	268	kk	2
>ref-96512	TCCTTCCATACCCTCCCTCCTCTCTTC	24	<lmxll>	ll	40	lm	212		
>ref-96522	CCATTTCCCACATCTCCTCCAGCCTCA	26	<lmxll>	ll	131	lm	134		
>ref-96528	TGAATACTGACCCCCCCTCCACTCTCT	9	<lmxll>	ll	240	lm	2		
>ref-96534	TGATTCCCAACGGTTTCTCCTGTCTTT	8	<nnxnp>	nn	104	np	123		
>ref-96560	CGTCCTGACACATGCACTCCATCCATT	23	<lmxll>	ll	129	lm	140		
>ref-96591	AGACCTATAACGTTCTCTCCCAGAGGA	7	<lmxll>	ll	125	lm	116		
>ref-96600	CCCCACTGCACTTTCTCTCCCTCTTTC	25	<lmxll>	ll	126	lm	139		
>ref-96600	CCCCACTGCACTTTCTCTCCCTCTTTC	27	<nnxnp>	nn	139	np	124		
>ref-96642	CTTTCCCCAACTCCACCTCCCCCCTTG	4	<hkxhk>	hh	0	hk	53	kk	190
>ref-96685	TCCTGCCCCACCACATCTCCCTCCTCA	5	<lmxll>	ll	139	lm	130		
>ref-96690	CTGTGGCTCACCGTTCCTCCCGATAGA	13	<lmxll>	ll	133	lm	120		
>ref-96754	TACTCCCATACTAACACTCCCTCCCTC	6	<hkxhk>	hh	4	hk	265	kk	0
>ref-96754	TACTCCCATACTAACACTCCCTCCCTC	21	<hkxhk>	hh	58	hk	204	kk	1
>ref-96754	TACTCCCATACTAACACTCCCTCCCTC	23	<lmxll>	ll	177	lm	73		
>ref-96767	TCCTTTTGCACTCTTCCTCCCTCTCCT	4	<lmxll>	ll	148	lm	120		
>ref-96767	TCCTTTTGCACTCTTCCTCCCTCTCCT	5	<lmxll>	ll	146	lm	121		
>ref-96778	GTCGCCAGAACCTGACCTCCTTACCTT	4	<hkxhk>	hh	0	hk	146	kk	112
>ref-96778	GTCGCCAGAACCTGACCTCCTTACCTT	5	<hkxhk>	hh	0	hk	271	kk	0
>ref-96778	GTCGCCAGAACCTGACCTCCTTACCTT	16	<hkxhk>	hh	0	hk	271	kk	0
>ref-96779	TTTCTCCCTACCCTTTCTCCTCCACCT	5	<lmxll>	ll	110	lm	117		
>ref-96786	GCCACTTGTACAGGGCCTCCCTTCACT	3	<nnxnp>	nn	125	np	148		
>ref-96801	ATGCCCCCCACCCTTACTCCAGCTCCA	9	<lmxll>	ll	103	lm	116		
>ref-96821	CTCCTTCCTACCACCGCTCCCTTCTCT	27	<nnxnp>	nn	265	np	0		
>ref-96849	CTCAATCTCACTCTCCCTCCCTTTCAT	5	<lmxll>	ll	2	lm	237		
>ref-96849	CTCAATCTCACTCTCCCTCCCTTTCAT	7	<lmxll>	ll	3	lm	245		
>ref-96856	TCTCAGCCCACCGTCCCTCCCTCTCTC	14	<lmxll>	ll	261	lm	0		
>ref-96856	TCTCAGCCCACCGTCCCTCCCTCTCTC	26	<lmxll>	ll	259	lm	0		
>ref-96859	CCCAATTCCACCCGACCTCCTCCTGTC	23	<hkxhk>	hh	47	hk	150	kk	65
>ref-96859	CCCAATTCCACCCGACCTCCTCCTGTC	25	<lmxll>	ll	114	lm	150		
>ref-96919	CAACCATTGACATGATCTCCATACGAC	21	<hkxhk>	hh	66	hk	199	kk	0
>ref-96919	CAACCATTGACATGATCTCCATACGAC	24	<hkxhk>	hh	0	hk	199	kk	66
>ref-96928	CTCACTGTCACTACTCCTCCTCCTTCT	1	<lmxll>	ll	253	lm	11		
>ref-96947	CCATACCACACAGCCCCTCCCATCCCC	15	<lmxll>	ll	106	lm	133		
>ref-96948	CTGTCCTCCACAACGTCTCCTTTTTAC	12	<lmxll>	ll	118	lm	146		
>ref-96949	ACATTGTGGACTTACTCTCCATGCAGC	1	<nnxnp>	nn	129	np	132		
>ref-96966	ACCCTATACACACAACCTCCTTATAAT	6	<hkxhk>	hh	38	hk	125	kk	58
>ref-96969	GGAAGTACCACCCCATCTCCCTCCTCA	2	<hkxhk>	hh	0	hk	270	kk	0

>ref-96969	GGAAGTACCACCCCATCTCCCTCCTCA	12	<hkxhk>	hh	0	hk	270	kk	0
>ref-96970	TTCTCCCTGACCCCTACTCCCTGCCCC	25	<nnxnp>	nn	133	np	132		
>ref-96984	AAGAAGAAAACTGCTTCTCCTCGGCAC	23	<nnxnp>	nn	112	np	156		
>ref-96984	AAGAAGAAAACTGCTTCTCCTCGGCAC	24	<nnxnp>	nn	111	np	156		
>ref-96985	TCCAAAAGGACGCTGCCTCCTCCTGGG	2	<lmxll>	ll	125	lm	127		
>ref-96996	TTCCTTTCTACCCTCTCTCCCATCCTC	3	<nnxnp>	nn	34	np	215		
>ref-96996	TTCCTTTCTACCCTCTCTCCCATCCTC	12	<lmxll>	ll	63	lm	183		
>ref-96996	TTCCTTTCTACCCTCTCTCCCATCCTC	27	<nnxnp>	nn	38	np	212		
>ref-97029	CACACATGCACACGATCTCCTTCCCAC	3	<nnxnp>	nn	149	np	122		
>ref-97029	CACACATGCACACGATCTCCTTCCCAC	13	<lmxll>	ll	162	lm	109		
>ref-97029	CACACATGCACACGATCTCCTTCCCAC	26	<nnxnp>	nn	149	np	122		
>ref-97032	CCGCCAGGGACGTAGCCTCCGCCGCCT	8	<nnxnp>	nn	130	np	133		
>ref-97039	TCCTCCGAGACAAATACTCCACTTCCC	1	<lmxll>	ll	124	lm	109		
>ref-97061	TTGTTCTCTACTATCTCTCCGTTCCCC	4	<nnxnp>	nn	259	np	0		
>ref-97062	GTTGCCATTACTGTTACTCCTACTATC	27	<nnxnp>	nn	110	np	143		
>ref-97073	CCTGCAACAACCATAACTCCACCTGCA	22	<hkxhk>	hh	59	hk	132	kk	75
>ref-97093	GCACAGCTGACCGTATCTCCGACAGCG	2	<lmxll>	ll	119	lm	138		
>ref-97093	GCACAGCTGACCGTATCTCCGACAGCG	5	<lmxll>	ll	119	lm	138		
>ref-97103	TGCCTTAAAACACATACTCCCCTCTTC	1	<nnxnp>	nn	188	np	66		
>ref-97103	TGCCTTAAAACACATACTCCCCTCTTC	22	<nnxnp>	nn	189	np	66		
>ref-97126	CGTCCAAGCACACTCACTCCAATGTAT	3	<nnxnp>	nn	138	np	127		
>ref-97130	TATAAAGCTACGCAGGCTCCGCCCCTT	21	<hkxhk>	hh	48	hk	139	kk	53
>ref-97153	GATCATTCCACTTTGCCTCCTCCCTTT	24	<nnxnp>	nn	113	np	143		
>ref-97158	TGTAGTGATACCTTAACTCCTCAAGGT	5	<lmxll>	ll	5	lm	259		
>ref-97158	TGTAGTGATACCTTAACTCCTCAAGGT	14	<lmxll>	ll	22	lm	235		
>ref-97158	TGTAGTGATACCTTAACTCCTCAAGGT	15	<lmxll>	ll	7	lm	261		
>ref-97158	TGTAGTGATACCTTAACTCCTCAAGGT	22	<lmxll>	ll	22	lm	235		
>ref-97161	CCTCCTCCCACTCTCCCTCCACCCTTA	21	<nnxnp>	nn	114	np	129		
>ref-97161	CCTCCTCCCACTCTCCCTCCACCCTTA	27	<nnxnp>	nn	115	np	131		
>ref-97168	TCTGGTAACACCACCCCTCCCCCCACA	5	<lmxll>	ll	128	lm	116		
>ref-97168	TCTGGTAACACCACCCCTCCCCCCACA	16	<nnxnp>	nn	113	np	128		
>ref-97172	CACGCAAGAACAACGTCTCCCTTGCAA	4	<lmxll>	ll	133	lm	133		
>ref-97172	CACGCAAGAACAACGTCTCCCTTGCAA	13	<lmxll>	ll	127	lm	140		
>ref-97175	ATCCCTCTCACATTGCCTCCCTCCCTT	12	<nnxnp>	nn	262	np	0		
>ref-97175	ATCCCTCTCACATTGCCTCCCTCCCTT	27	<nnxnp>	nn	261	np	0		
>ref-97184	AGAATTTCCACTGGATCTCCCGCACCT	12	<lmxll>	ll	124	lm	136		
>ref-97207	TTCCTTCCTACTTTCCCTCCTTCCTGT	1	<lmxll>	ll	127	lm	135		
>ref-97207	TTCCTTCCTACTTTCCCTCCTTCCTGT	7	<lmxll>	ll	125	lm	135		

>ref-97222	CTCCCATGCACTTGTTCTCCCTCGTAC	1	<nnxnp>	nn	273	np	0		
>ref-97225	ACACGAGCCACTTCCCCTCCCACACCT	21	<lmxll>	ll	127	lm	130		
>ref-97245	CCCCTTCACACCATTTCTCCTCTCATA	2	<nnxnp>	nn	132	np	120		
>ref-97257	TTATAAAGAACCAGCTCTCCCCTTGAC	26	<lmxll>	ll	92	lm	127		
>ref-97269	CCGACGTTCACCTTCTCTCCTCCGATT	6	<hkxhk>	hh	52	hk	154	kk	51
>ref-97276	ATTCTCGCTACTCCTTCTCCCATTCCT	21	<nnxnp>	nn	110	np	146		
>ref-97276	ATTCTCGCTACTCCTTCTCCCATTCCT	22	<nnxnp>	nn	110	np	146		
>ref-97294	CCTCTCTAAACCCTCTCTCCCTCCCCC	8	<nnxnp>	nn	143	np	102		
>ref-97294	CCTCTCTAAACCCTCTCTCCCTCCCCC	14	<nnxnp>	nn	143	np	106		
>ref-97299	TGTCTCCTCACCTCTTCTCCTTTGCTC	2	<hkxhk>	hh	67	hk	130	kk	56
>ref-97323	CTATTCCTTACTTCCGCTCCATCCTCT	6	<nnxnp>	nn	137	np	131		
>ref-97337	CCAATACTGACCGAGTCTCCTTCAGAA	21	<lmxll>	ll	127	lm	131		
>ref-97345	CATCCCTCCACTTTCCCTCCCCTGTTT	4	<nnxnp>	nn	235	np	0		
>ref-97363	TATATATACACACACCCTCCCGCGTCG	22	<lmxll>	ll	126	lm	125		
>ref-97375	CTCTCACTCACCCTTCCTCCGTGATAA	14	<hkxhk>	hh	52	hk	126	kk	63
>ref-97386	GAAAGAGAGACTATAGCTCCAAGCGGA	21	<nnxnp>	nn	128	np	137		
>ref-97412	AATAAGCGAACCGCCGCTCCGGTGATT	6	<lmxll>	ll	127	lm	126		
>ref-97414	GTGGTATGTACTGCGTCTCCTCATTGA	1	<nnxnp>	nn	141	np	126		
>ref-97424	TACACATACACACTTTCTCCTTCTCCT	5	<lmxll>	ll	3	lm	264		
>ref-97424	TACACATACACACTTTCTCCTTCTCCT	7	<lmxll>	ll	3	lm	264		
>ref-97431	CTTGCTCTCACCCCCTCTCCTCACACC	6	<hkxhk>	hh	57	hk	202	kk	5
>ref-97457	ACATCTCCCACCCTCCCTCCACATCTC	6	<lmxll>	ll	175	lm	81		
>ref-97457	ACATCTCCCACCCTCCCTCCACATCTC	8	<lmxll>	ll	175	lm	81		
>ref-97463	GTTGATCGCACTCCGTCTCCATACCGT	3	<nnxnp>	nn	141	np	128		
>ref-97468	GTGTTGCCAACAGGCTCTCCTTTCTCT	21	<lmxll>	ll	129	lm	140		
>ref-97487	TCCTTCCTTACTTTTTCTCCTTCTCTT	9	<hkxhk>	hh	11	hk	238	kk	6
>ref-97487	TCCTTCCTTACTTTTTCTCCTTCTCTT	13	<lmxll>	ll	269	lm	1		
>ref-97487	TCCTTCCTTACTTTTTCTCCTTCTCTT	15	<hkxhk>	hh	11	hk	238	kk	6
>ref-97487	TCCTTCCTTACTTTTTCTCCTTCTCTT	16	<nnxnp>	nn	129	np	133		
>ref-97487	TCCTTCCTTACTTTTTCTCCTTCTCTT	25	<lmxll>	ll	272	lm	0		
>ref-97491	TCACCTTTAACCATAACTCCTTTCCCT	3	<nnxnp>	nn	102	np	121		
>ref-97497	TATATACAAACAGCAGCTCCAACACAC	5	<nnxnp>	nn	121	np	101		
>ref-97500	CTCTTTATCACGTCCTCTCCTCCCTTA	12	<nnxnp>	nn	140	np	111		
>ref-97500	CTCTTTATCACGTCCTCTCCTCCCTTA	15	<nnxnp>	nn	140	np	111		
>ref-97525	CATGACGTCACAGGCCCTCCTTGCCTT	12	<lmxll>	ll	125	lm	148		
>ref-97527	CTATGAACCACTGGCACTCCCTAAAGT	21	<nnxnp>	nn	122	np	134		
>ref-97547	CATCCCCTCACTTCCCCTCCGTTCCCT	1	<lmxll>	ll	264	lm	1		
>ref-97547	CATCCCCTCACTTCCCCTCCGTTCCCT	2	<lmxll>	ll	75	lm	158		

>ref-97547	CATCCCCTCACTTCCCCTCCGTTCCCT	21	<lmxll>	ll	52	lm	189		
>ref-97548	ATTCAATGAACTCTTTCTCCTTGTTGG	1	<nnxnp>	nn	248	np	0		
>ref-97598	ATGATGGTTACATAACCTCCTTAGTAG	15	<lmxll>	ll	139	lm	124		
>ref-97607	TCTAAGCCAACCGTGTCTCCACGGACC	5	<nnxnp>	nn	111	np	148		
>ref-97618	TACAATTTCACAATTACTCCCAAACAC	23	<hkxhk>	hh	1	hk	270	kk	0
>ref-97629	TCCCGGAGAACTCTCCCTCCCTTCCCT	5	<lmxll>	ll	252	lm	0		
>ref-97636	AGTAGAACGACAGAGTCTCCCCTTAAA	3	<nnxnp>	nn	142	np	100		
>ref-97636	AGTAGAACGACAGAGTCTCCCCTTAAA	8	<hkxhk>	hh	9	hk	224	kk	1
>ref-97641	CTATCTGTCACACACTCTCCTTCCCCC	26	<lmxll>	ll	98	lm	154		
>ref-97684	AATGGGCGGACGGGGACTCCAGGCGCT	21	<nnxnp>	nn	119	np	145		
>ref-97684	AATGGGCGGACGGGGACTCCAGGCGCT	23	<lmxll>	ll	123	lm	147		
>ref-97690	AGTAAGAACACGTAACCTCCCAACGCG	16	<lmxll>	ll	116	lm	108		
>ref-97717	CCTCCCCTGACACTGCCTCCCCTAATA	23	<nnxnp>	nn	254	np	0		
>ref-97721	ACAGGGTCCACAGGGTCTCCCGAGTCT	15	<nnxnp>	nn	138	np	134		
>ref-97736	GCAAATAAAACATCTACTCCACTGTCC	3	<nnxnp>	nn	262	np	0		
>ref-97739	GTCTCGTATACTCCTCCTCCTCGTCGT	15	<nnxnp>	nn	108	np	152		
>ref-97746	ACACTATTAACCCAATCTCCCCGTATG	2	<hkxhk>	hh	1	hk	253	kk	6
>ref-97746	ACACTATTAACCCAATCTCCCCGTATG	15	<lmxll>	ll	142	lm	107		
>ref-97755	AGGAATACCACAGGCACTCCCAGGGTT	14	<hkxhk>	hh	0	hk	261	kk	10
>ref-97787	CCTGGGTCCACTGACTCTCCTCCACTC	2	<hkxhk>	hh	2	hk	266	kk	0
>ref-97787	CCTGGGTCCACTGACTCTCCTCCACTC	5	<lmxll>	ll	162	lm	100		
>ref-97787	CCTGGGTCCACTGACTCTCCTCCACTC	12	<hkxhk>	hh	0	hk	266	kk	2
>ref-97787	CCTGGGTCCACTGACTCTCCTCCACTC	25	<lmxll>	ll	162	lm	101		
>ref-97805	CTCTGTCATACGCCTTCTCCAAGCCTA	6	<hkxhk>	hh	0	hk	225	kk	43
>ref-97805	CTCTGTCATACGCCTTCTCCAAGCCTA	24	<hkxhk>	hh	43	hk	225	kk	0
>ref-97833	CACGCCATCACTCCCCCTCCTCTCCAC	21	<nnxnp>	nn	116	np	134		
>ref-97839	AGATTGAAGACTGTGCCTCCCCGCCCA	21	<lmxll>	ll	116	lm	120		
>ref-97853	ACCTTTCACACCCTACCTCCTTTGCCG	25	<lmxll>	ll	124	lm	138		
>ref-97860	TCTCATTCCACACTCACTCCTCACCAA	5	<hkxhk>	hh	3	hk	267	kk	0
>ref-97867	AACGACAAAACGTGTCCTCCTAAACAT	24	<lmxll>	ll	122	lm	146		
>ref-97879	TAAACTAAAACACTTTCTCCTTTTAGT	26	<lmxll>	ll	125	lm	111		
>ref-97884	TCATAGTTTACATTACCTCCTATGTTG	23	<nnxnp>	nn	220	np	0		
>ref-97892	CCCTCCCCAACTTTCCCTCCCCAACTT	21	<nnxnp>	nn	245	np	22		
>ref-97892	CCCTCCCCAACTTTCCCTCCCCAACTT	24	<hkxhk>	hh	0	hk	272	kk	1
>ref-97892	CCCTCCCCAACTTTCCCTCCCCAACTT	25	<nnxnp>	nn	245	np	22		
>ref-97917	ATTCCTACCACCTCGTCTCCTCCTTCT	25	<nnxnp>	nn	120	np	137		
>ref-97932	TCCTTTCCCACCTTTCCTCCTTCCTGC	14	<hkxhk>	hh	0	hk	272	kk	0
>ref-97932	TCCTTTCCCACCTTTCCTCCTTCCTGC	24	<lmxll>	ll	20	lm	241		

>ref-97932	TCCTTTCCCACCTTTCCTCCTTCCTGC	26	<hkxhk>	hh	0	hk	271	kk	0
>ref-97945	CATACTACTACCTCTGCTCCCTCTCTC	14	<nnxnp>	nn	122	np	139		
>ref-97950	CTCATCCCTACTCTTTCTCCTTCTTCC	25	<nnxnp>	nn	239	np	3		
>ref-97992	AGCATCAGCACCTGCACTCCCTTTTCA	6	<nnxnp>	nn	136	np	135		
>ref-97997	CCTCACTCGACCATAGCTCCTCCCACT	4	<nnxnp>	nn	147	np	117		
>ref-97997	CCTCACTCGACCATAGCTCCTCCCACT	22	<hkxhk>	hh	42	hk	210	kk	0
>ref-97997	CCTCACTCGACCATAGCTCCTCCCACT	24	<nnxnp>	nn	21	np	249		
>ref-98035	AACGGGGCCACGGTTGCTCCTCCTCAG	26	<hkxhk>	hh	63	hk	138	kk	69
>ref-98036	GAAATATAAACAGCATCTCCTTCCTGT	22	<lmxll>	ll	111	lm	121		
>ref-98037	TCTCCTGTTACTCTCTCTCCTGCGTCC	3	<nnxnp>	nn	113	np	157		
>ref-98049	TCACGCTTTACTGATGCTCCACAACGG	8	<nnxnp>	nn	230	np	27		
>ref-98061	TATTCTTACACTCATTCTCCTCATTTC	8	<lmxll>	ll	126	lm	140		
>ref-98073	CATCTATCCACCCGTCCTCCCACCCAT	14	<nnxnp>	nn	257	np	0		
>ref-98073	CATCTATCCACCCGTCCTCCCACCCAT	27	<nnxnp>	nn	263	np	0		
>ref-98113	CTGCTTCCCACCATCCCTCCCTTTCCT	4	<nnxnp>	nn	153	np	100		
>ref-98118	TCATTACGGACAGTCTCTCCGGCCGCT	23	<lmxll>	ll	148	lm	118		
>ref-98119	TGTTCCGACACTCTTGCTCCTCCAGAC	5	<nnxnp>	nn	113	np	151		
>ref-98142	TGCAAGCATACATAGCCTCCCTGACAG	6	<hkxhk>	hh	83	hk	131	kk	48
>ref-98169	CTACCCAAGACAGAGACTCCATTAACA	26	<lmxll>	ll	134	lm	132		
>ref-98171	CTTTCTACAACAGAGTCTCCTTGCTTC	24	<nnxnp>	nn	265	np	0		
>ref-98172	ACGACAACGACAGTAACTCCAGCTTCT	23	<nnxnp>	nn	271	np	0		
>ref-98185	TCCATGTCAACTGCTTCTCCCTTGTCC	9	<hkxhk>	hh	58	hk	146	kk	66
>ref-98192	TCTGCCACTACCACAACTCCGCTAGCA	7	<lmxll>	ll	127	lm	120		
>ref-98193	TAATTAACCACTCAATCTCCTCTCGCT	8	<lmxll>	ll	116	lm	109		
>ref-98194	TTCCTCTCTACATCATCTCCCATCTCT	22	<lmxll>	ll	104	lm	125		
>ref-98195	GTTCCCAACACTACTTCTCCTGCGCGC	13	<hkxhk>	hh	102	hk	158	kk	0
>ref-98220	ATGTTGTATACTGACTCTCCACACCCC	22	<nnxnp>	nn	131	np	121		
>ref-98231	TTAGCCAATACCTTCCCTCCTCTCTTT	23	<nnxnp>	nn	118	np	123		
>ref-98231	TTAGCCAATACCTTCCCTCCTCTCTTT	27	<nnxnp>	nn	116	np	123		
>ref-98238	CACACACACACTTTATCTCCCGCTAAC	21	<nnxnp>	nn	120	np	133		
>ref-98242	CTTACTATTACCAGTTCTCCTAACTCC	25	<lmxll>	ll	125	lm	114		
>ref-98244	GTGTATTGTACCCTCTCTCCTTCCTTC	4	<lmxll>	ll	134	lm	110		
>ref-98244	GTGTATTGTACCCTCTCTCCTTCCTTC	25	<lmxll>	ll	133	lm	110		
>ref-98255	TTCTTCTCCACAGAACCTCCTTTGACT	9	<lmxll>	ll	125	lm	133		
>ref-98266	TCCGACCCGACGCCATCTCCTGCTCTC	8	<hkxhk>	hh	63	hk	145	kk	54
>ref-98266	TCCGACCCGACGCCATCTCCTGCTCTC	22	<hkxhk>	hh	54	hk	145	kk	64
>ref-98267	CCCTCCGTGACCTCCCCTCCTCTCCCC	27	<lmxll>	ll	60	lm	187		
>ref-98268	CTCTCCACGACCCTTTCTCCCTCATTG	7	<nnxnp>	nn	101	np	140		

>ref-98268	CTCTCCACGACCCTTTCTCCCTCATTG	9	<nnxnp>	nn	101	np	139		
>ref-98270	ACTCTTTAAACACCACCTCCTTCGCTC	1	<nnxnp>	nn	122	np	144		
>ref-98290	CAACCCCCAACCTCCACTCCCTCGCTT	6	<nnxnp>	nn	126	np	126		
>ref-98317	CGTAAACACACTCCTCCTCCTTCTCCT	5	<lmxll>	ll	126	lm	123		
>ref-98344	GAAATAGGAACTGGACCTCCTTTACAT	8	<lmxll>	ll	130	lm	124		
>ref-98360	TTCACTATCACCGGCGCTCCTTCTAGA	15	<lmxll>	ll	139	lm	127		
>ref-98367	TTATTACGCACCACGTCTCCCTTACTT	5	<nnxnp>	nn	137	np	131		
>ref-98373	TACACATTGACCAGACCTCCATTATAG	14	<lmxll>	ll	121	lm	102		
>ref-98376	GTATCACACACCACCACTCCCTCTGTT	23	<hkxhk>	hh	60	hk	145	kk	62
>ref-98385	AATCAACGTACTCTCTCTCCTTCCTCT	3	<nnxnp>	nn	118	np	130		
>ref-98401	CACAATCACACCCCCCCTCCACACACA	15	<hkxhk>	hh	0	hk	272	kk	1
>ref-98401	CACAATCACACCCCCCCTCCACACACA	24	<hkxhk>	hh	1	hk	272	kk	0
>ref-98459	CACCCCCCTACCTTACCTCCCTGGCCT	21	<nnxnp>	nn	133	np	127		
>ref-98464	TTGTGTGTGACCATCGCTCCCTCACCT	7	<nnxnp>	nn	133	np	139		
>ref-98465	ATCTGTTCCACTTCCTCTCCCTTCAAC	14	<hkxhk>	hh	66	hk	137	kk	52
>ref-98499	ACCACTTTCACGTTATCTCCAACACCT	14	<nnxnp>	nn	135	np	131		
>ref-98507	ATGTTGTCAACAGTCTCTCCTTTCATC	13	<hkxhk>	hh	1	hk	268	kk	2
>ref-98549	TTGTTTGCTACAGCATCTCCTCTTCAC	1	<hkxhk>	hh	0	hk	273	kk	0
>ref-98549	TTGTTTGCTACAGCATCTCCTCTTCAC	2	<hkxhk>	hh	0	hk	273	kk	0
>ref-98590	CTTGTTCCCACCTATCCTCCTCCCCCT	4	<hkxhk>	hh	0	hk	241	kk	18
>ref-98590	CTTGTTCCCACCTATCCTCCTCCCCCT	6	<hkxhk>	hh	0	hk	241	kk	19
>ref-98644	CATCCACCCACACAACCTCCTCCCTTC	3	<lmxll>	ll	1	lm	271		
>ref-98644	CATCCACCCACACAACCTCCTCCCTTC	4	<hkxhk>	hh	0	hk	261	kk	8
>ref-98644	CATCCACCCACACAACCTCCTCCCTTC	14	<hkxhk>	hh	8	hk	261	kk	0
>ref-98644	CATCCACCCACACAACCTCCTCCCTTC	16	<lmxll>	ll	66	lm	183		
>ref-98644	CATCCACCCACACAACCTCCTCCCTTC	21	<lmxll>	ll	1	lm	271		
>ref-98645	ATTACTACCACCATTACTCCACGAAAA	7	<hkxhk>	hh	62	hk	132	kk	40
>ref-98645	ATTACTACCACCATTACTCCACGAAAA	27	<lmxll>	ll	136	lm	104		
>ref-98650	TTCTTCCCTACGCCCCCTCCCCCCATT	24	<lmxll>	ll	129	lm	120		
>ref-98681	TATGCTCCCACACCCTCTCCCTCTGTT	26	<nnxnp>	nn	131	np	128		
>ref-98704	ATCTCCCCCACCTCCCCTCCTTCATCT	13	<hkxhk>	hh	0	hk	204	kk	39
>ref-98704	ATCTCCCCCACCTCCCCTCCTTCATCT	24	<hkxhk>	hh	37	hk	204	kk	0
>ref-98706	TTCACTACCACCCCTACTCCTCTCCCT	6	<hkxhk>	hh	0	hk	256	kk	6
>ref-98725	TCCACCTCCACCTGTACTCCACTACAG	5	<lmxll>	ll	121	lm	131		
>ref-98727	CCGCAGGCGACCTTCACTCCCAACTCC	4	<nnxnp>	nn	266	np	0		
>ref-98735	TCTGTATATACTCTCCCTCCCCCGGAA	9	<lmxll>	ll	112	lm	110		
>ref-98752	CAGCGACCAACGCTAGCTCCTCTCGCT	27	<lmxll>	ll	129	lm	134		
>ref-98793	TACAGTGTTACTCTCCCTCCCTCAGAC	6	<hkxhk>	hh	0	hk	165	kk	73

>ref-98793	TACAGTGTTACTCTCCCTCCCTCAGAC	16	<hkxhk>	hh	109	hk	117	kk	0
>ref-98793	TACAGTGTTACTCTCCCTCCCTCAGAC	23	<nnxnp>	nn	146	np	115		
>ref-98793	TACAGTGTTACTCTCCCTCCCTCAGAC	27	<hkxhk>	hh	0	hk	96	kk	143
>ref-98806	GGAAATCTGACAGAGGCTCCTAACAAT	1	<hkxhk>	hh	6	hk	211	kk	19
>ref-98806	GGAAATCTGACAGAGGCTCCTAACAAT	15	<hkxhk>	hh	19	hk	211	kk	6
>ref-98839	GCCCTCCCTACCCCAACTCCACCCCCC	22	<lmxll>	ll	176	lm	64		
>ref-98839	GCCCTCCCTACCCCAACTCCACCCCCC	27	<hkxhk>	hh	43	hk	198	kk	3
>ref-98852	CTCTGTTTCACCCATTCTCCTGCTCCG	3	<hkxhk>	hh	0	hk	163	kk	96
>ref-98852	CTCTGTTTCACCCATTCTCCTGCTCCG	6	<hkxhk>	hh	0	hk	161	kk	98
>ref-98852	CTCTGTTTCACCCATTCTCCTGCTCCG	22	<lmxll>	ll	141	lm	122		
>ref-98866	GTCTTCTCTACCCTTCCTCCCCTTTTC	23	<nnxnp>	nn	240	np	0		
>ref-98928	TTGTTCCTTACCCAATCTCCTTGCTTG	27	<lmxll>	ll	114	lm	123		
>ref-98929	AAGTATCACACTTCAACTCCTTTTAGT	3	<hkxhk>	hh	1	hk	233	kk	5
>ref-98929	AAGTATCACACTTCAACTCCTTTTAGT	26	<hkxhk>	hh	79	hk	154	kk	0
>ref-98947	TCTGTTCTTACTGTTGCTCCATTTCAT	14	<nnxnp>	nn	117	np	148		
>ref-98948	CCAACTCCCACTTACACTCCAGTCCTT	3	<nnxnp>	nn	113	np	132		
>ref-98957	AACATACTCACTCCACCTCCTCCCCCT	3	<nnxnp>	nn	118	np	135		
>ref-98957	AACATACTCACTCCACCTCCTCCCCCT	4	<lmxll>	ll	262	lm	0		
>ref-98967	TTGATCTCCACTTCAACTCCAAACACC	9	<hkxhk>	hh	20	hk	247	kk	0
>ref-98967	TTGATCTCCACTTCAACTCCAAACACC	16	<nnxnp>	nn	221	np	41		
>ref-98967	TTGATCTCCACTTCAACTCCAAACACC	25	<hkxhk>	hh	1	hk	271	kk	0
>ref-98974	CATTTTTCTACTCTCACTCCCTTTCAT	4	<nnxnp>	nn	94	np	142		
>ref-98996	CTCCTCCTTACCCTCCCTCCCTTATCC	13	<hkxhk>	hh	9	hk	261	kk	0
>ref-98996	CTCCTCCTTACCCTCCCTCCCTTATCC	24	<hkxhk>	hh	9	hk	261	kk	0
>ref-98999	CATGCATGCACGCACACTCCTTCCTTC	1	<nnxnp>	nn	157	np	115		
>ref-99019	TCACCCTCCACCTCTTCTCCCTTCCTC	6	<hkxhk>	hh	6	hk	260	kk	0
>ref-99019	TCACCCTCCACCTCTTCTCCCTTCCTC	25	<hkxhk>	hh	0	hk	260	kk	6
>ref-99040	CCCTTCTGTACCTATTCTCCTTCTTCC	3	<nnxnp>	nn	260	np	0		
>ref-99040	CCCTTCTGTACCTATTCTCCTTCTTCC	4	<nnxnp>	nn	263	np	0		
>ref-99047	CTATCTCTCACACATTCTCCTTTTCTT	3	<hkxhk>	hh	0	hk	257	kk	0
>ref-99054	AGCAAATGAACGAGGACTCCGTTGGCT	3	<hkxhk>	hh	47	hk	142	kk	70
>ref-99080	GTAATACAAACCTCCTCTCCCTCTCTC	5	<lmxll>	ll	77	lm	179		
>ref-99080	GTAATACAAACCTCCTCTCCCTCTCTC	12	<lmxll>	ll	77	lm	179		
>ref-99082	TGTCAGCATACAGGAGCTCCACCTGAA	2	<lmxll>	ll	124	lm	139		
>ref-99087	CTCTCTCTGACTCACCCTCCTGACTTG	12	<nnxnp>	nn	262	np	0		
>ref-99091	TGGTAAATCACAGGTCCTCCTTGACTT	3	<nnxnp>	nn	105	np	153		
>ref-99092	TGCGGCACCACTCTCTCTCCCTCGGCT	6	<hkxhk>	hh	1	hk	215	kk	53
>ref-99102	CTTCCCTCTACAGTAGCTCCGGATTAC	4	<lmxll>	ll	121	lm	100		

>ref-99107	AATATAATAACCGTCACTCCTCGAACA	15	<lmxll>	ll	127	lm	135		
>ref-99108	TCCCCTCCGACTCTCCCTCCATTTCTT	1	<lmxll>	ll	119	lm	144		
>ref-99108	TCCCCTCCGACTCTCCCTCCATTTCTT	2	<lmxll>	ll	119	lm	144		
>ref-99151	CTTCACATCACGAATTCTCCTTGCAGC	4	<hkxhk>	hh	6	hk	258	kk	0
>ref-99151	CTTCACATCACGAATTCTCCTTGCAGC	12	<hkxhk>	hh	0	hk	270	kk	1
>ref-99151	CTTCACATCACGAATTCTCCTTGCAGC	25	<hkxhk>	hh	1	hk	270	kk	0
>ref-99164	TATCCCTCTACAGCGTCTCCTTGAGCA	23	<nnxnp>	nn	134	np	136		
>ref-99178	GCATTCTATACTCTCTCTCCCTGTCAC	13	<lmxll>	ll	109	lm	123		
>ref-99179	CCGACAGCAACAACGCCTCCCCTCTCT	15	<lmxll>	ll	0	lm	272		
>ref-99179	CCGACAGCAACAACGCCTCCCCTCTCT	27	<lmxll>	ll	0	lm	272		
>ref-99181	CTCTCCCGGACTCTCCCTCCGCACGAC	6	<nnxnp>	nn	122	np	135		
>ref-99181	CTCTCCCGGACTCTCCCTCCGCACGAC	7	<nnxnp>	nn	123	np	135		
>ref-99203	GGGGAAAGTACCTCATCTCCTTCACTC	23	<nnxnp>	nn	117	np	143		
>ref-99207	ACAGTACGGACATCACCTCCATCAACG	14	<hkxhk>	hh	42	hk	131	kk	62
>ref-99232	TCTTCCCTGACTCTCACTCCCTCCTTC	16	<hkxhk>	hh	14	hk	243	kk	0
>ref-99232	TCTTCCCTGACTCTCACTCCCTCCTTC	27	<hkxhk>	hh	17	hk	238	kk	0
>ref-99236	CTTCTTTACACTCGTCCTCCTCCTATT	8	<hkxhk>	hh	0	hk	271	kk	0
>ref-99236	CTTCTTTACACTCGTCCTCCTCCTATT	25	<hkxhk>	hh	0	hk	271	kk	0
>ref-99262	CTCTCTCTCACCCTATCTCCTCATTCT	9	<hkxhk>	hh	0	hk	272	kk	1
>ref-99262	CTCTCTCTCACCCTATCTCCTCATTCT	15	<hkxhk>	hh	1	hk	272	kk	0
>ref-99306	TTCATTTCAACTTATTCTCCTTGTTAC	14	<lmxll>	ll	106	lm	120		
>ref-99316	CTCCTGGCCACCCCCTCTCCGCTGATG	4	<hkxhk>	hh	50	hk	142	kk	51
>ref-99316	CTCCTGGCCACCCCCTCTCCGCTGATG	6	<nnxnp>	nn	120	np	131		
>ref-99393	ATCAACCCCACTGTTCCTCCATCACCC	13	<lmxll>	ll	129	lm	92		
>ref-99450	TCTATGTTCACCTCCTCTCCTTTTCCC	1	<hkxhk>	hh	13	hk	238	kk	1
>ref-99450	TCTATGTTCACCTCCTCTCCTTTTCCC	7	<hkxhk>	hh	13	hk	238	kk	1
>ref-99470	TTGCCTAGGACTTCCTCTCCCAGAATT	2	<nnxnp>	nn	94	np	153		
>ref-99470	TTGCCTAGGACTTCCTCTCCCAGAATT	4	<nnxnp>	nn	95	np	151		
>ref-99470	TTGCCTAGGACTTCCTCTCCCAGAATT	14	<hkxhk>	hh	4	hk	264	kk	0
>ref-99470	TTGCCTAGGACTTCCTCTCCCAGAATT	24	<hkxhk>	hh	4	hk	264	kk	0
>ref-99475	TCTTCTTCCACATTCCCTCCCTCCTCC	16	<hkxhk>	hh	1	hk	204	kk	50
>ref-99475	TCTTCTTCCACATTCCCTCCCTCCTCC	27	<hkxhk>	hh	2	hk	205	kk	49
>ref-99492	TCGCCTACCACTTCCTCTCCCTCTACT	15	<nnxnp>	nn	128	np	135		
>ref-99537	CTGCCAGCGACAGTGCCTCCTTCACCA	4	<lmxll>	ll	149	lm	110		
>ref-99555	CATACAGAGACATCAACTCCAGCATTG	15	<lmxll>	ll	134	lm	136		
>ref-99563	TATAAACAAACAACTCCTCCCTACACT	16	<lmxll>	ll	114	lm	112		
>ref-99565	TGCTAATTCACCCAATCTCCTCGACCT	23	<nnxnp>	nn	130	np	133		
>ref-99571	ATCACGCTCACGAACTCTCCTTTTATT	22	<lmxll>	ll	116	lm	122		

>ref-99578	TTTAACTTCACCCATCCTCCATGGATT	7	<lmxll>	ll	105	lm	134			
>ref-99631	CCTTTCTTCACTCTCTCTCCTTTTCTA	2	<lmxll>	ll	157	lm	84			
>ref-99631	CCTTTCTTCACTCTCTCTCCTTTTCTA	7	<lmxll>	ll	158	lm	84			
>ref-99631	CCTTTCTTCACTCTCTCTCCTTTTCTA	21	<lmxll>	ll	142	lm	117			
>ref-99681	CTTACACTGACCGATTCTCCACTCTTC	12	<lmxll>	ll	30	lm	236			
>ref-99685	GCCAGAATTACAATTCCTCCTGAAGGG	2	<nnxnp>	nn	263	np	0			
>ref-99690	AATATCGTCACAATAACTCCCTGAATA	2	<lmxll>	ll	101	lm	161			
>ref-99748	CCCTGAGGGACTGAAGCTCCTGCTGCT	2	<nnxnp>	nn	252	np	0			
>ref-99748	CCCTGAGGGACTGAAGCTCCTGCTGCT	23	<nnxnp>	nn	240	np	0			
>ref-99780	TTTTCGTTTACATGCTCTCCCTTTACT	9	<hkxhk>	hh	64	hk	145	kk	42
>ref-99780	TTTTCGTTTACATGCTCTCCCTTTACT	25	<hkxhk>	hh	41	hk	146	kk	65
>ref-99791	TGAGTGTCTACTGGCGCTCCCTCTCCA	8	<nnxnp>	nn	140	np	130			
>ref-99816	TTCCTTCTCACTTCGCCTCCTCCTCTA	1	<nnxnp>	nn	262	np	0			
>ref-99830	CTGCTCTGCACTCTCCCTCCCTCCCCC	22	<lmxll>	ll	0	lm	220			
>ref-99843	TTTTATCCTACTTTGCCTCCTCCTCCT	15	<nnxnp>	nn	213	np	6			
>ref-99845	CACTGCAAGACCCTCCCTCCATTACTC	23	<nnxnp>	nn	124	np	125			
>ref-99859	TGCACGCAAACACAGCCTCCTAATGCA	1	<lmxll>	ll	2	lm	268			
>ref-99859	TGCACGCAAACACAGCCTCCTAATGCA	2	<lmxll>	ll	2	lm	268			
>ref-99873	CTCCTTGCAACCTTGCCTCCCTTTCCG	7	<hkxhk>	hh	0	hk	254	kk	11
>ref-99873	CTCCTTGCAACCTTGCCTCCCTTTCCG	27	<hkxhk>	hh	0	hk	267	kk	4
>ref-99901	CCCTCCCGCACCTCCACTCCTTCACAC	14	<hkxhk>	hh	0	hk	273	kk	0
>ref-99901	CCCTCCCGCACCTCCACTCCTTCACAC	16	<hkxhk>	hh	24	hk	247	kk	0
>ref-99901	CCCTCCCGCACCTCCACTCCTTCACAC	23	<hkxhk>	hh	238	hk	30	kk	0
>ref-99901	CCCTCCCGCACCTCCACTCCTTCACAC	26	<hkxhk>	hh	0	hk	273	kk	0
>ref-99901	CCCTCCCGCACCTCCACTCCTTCACAC	27	<hkxhk>	hh	60	hk	208	kk	0
>ref-99906	TTTGGTGTTACTCTTTCTCCCTTTCTC	1	<hkxhk>	hh	0	hk	189	kk	56
>ref-99906	TTTGGTGTTACTCTTTCTCCCTTTCTC	7	<hkxhk>	hh	0	hk	191	kk	54
>ref-99931	TTTAGTACAACCCCCACTCCCCTCCCT	21	<lmxll>	ll	132	lm	109			
>ref-99932	ACTCCCCCCACTCCCACTCCACCTCCA	4	<lmxll>	ll	259	lm	0			
>ref-99932	ACTCCCCCCACTCCCACTCCACCTCCA	25	<nnxnp>	nn	188	np	46			
>ref-99939	CTCTCTCCTACTCATTCTCCTCTCTTC	15	<nnxnp>	nn	125	np	129			
>ref-99952	GAGGTAACGACACGGCCTCCATGACCA	22	<lmxll>	ll	124	lm	146			
>ref-99963	CGCCCCCCGACTCCCCCTCCCTCTCTC	2	<nnxnp>	nn	65	np	179			
>ref-99963	CGCCCCCCGACTCCCCCTCCCTCTCTC	9	<hkxhk>	hh	1	hk	247	kk	8
>ref-99963	CGCCCCCCGACTCCCCCTCCCTCTCTC	23	<lmxll>	ll	134	lm	115			
>ref-99963	CGCCCCCCGACTCCCCCTCCCTCTCTC	27	<nnxnp>	nn	262	np	1			
>ref-99974	ATCTTGTCCACCTTTTCTCCGCTTCCC	23	<nnxnp>	nn	124	np	141			
>ref-99987	CTTTATTTTACAGAACCTCCGCGATAA	4	<lmxll>	ll	115	lm	137			

>ref-99987	CTTTATTTTACAGAACCTCCGCGATAA	6	<lmxll>	ll	130	lm	125		
>ref-99987	CTTTATTTTACAGAACCTCCGCGATAA	13	<lmxll>	ll	130	lm	124		
>ref-100015	ACATCGAAGACCTCAGCTCCGAGATGA	21	<nnxnp>	nn	136	np	117		
>ref-100015	ACATCGAAGACCTCAGCTCCGAGATGA	27	<nnxnp>	nn	136	np	117		
>ref-100020	TTCCAGCCCACTCCCCCTCCTTCCTAT	15	<nnxnp>	nn	109	np	140		
>ref-100032	CAATAAAACACCTCTGCTCCCTGTCTT	21	<nnxnp>	nn	104	np	130		
>ref-100050	CCCACGCAAACCTTTCCTCCTGGCAAC	7	<hkxhk>	hh	60	hk	111	kk	60
>ref-100058	ATATCCTCTACGCCACCTCCAACTCCT	23	<nnxnp>	nn	118	np	143		
>ref-100075	TCCCTCCCGACCAGCGCTCCCTCGGCC	9	<nnxnp>	nn	266	np	0		
>ref-100103	CACCCATTCACCCTCCCTCCTCCTCCT	8	<hkxhk>	hh	0	hk	175	kk	83
>ref-100103	CACCCATTCACCCTCCCTCCTCCTCCT	23	<hkxhk>	hh	91	hk	174	kk	0
>ref-100132	GGAGGTCGCACTCCGTCTCCTCGGCCG	9	<lmxll>	ll	146	lm	126		
>ref-100141	GAGGCTTCCACTTTTACTCCTTTCTTT	22	<nnxnp>	nn	139	np	132		
>ref-100142	GAGAGCAATACGACGACTCCGACTGTT	13	<hkxhk>	hh	0	hk	273	kk	0
>ref-100153	TGGTCTTCTACGGCCTCTCCCTCAGCG	5	<lmxll>	ll	8	lm	263		
>ref-100153	TGGTCTTCTACGGCCTCTCCCTCAGCG	7	<lmxll>	ll	8	lm	263		
>ref-100176	CCCTTTTTAACATCATCTCCTTCTTCT	22	<lmxll>	ll	127	lm	122		
>ref-100178	TAACCGCCAACCTCTGCTCCAGAGATG	3	<nnxnp>	nn	137	np	129		
>ref-100183	AGATAATAAACAAACACTCCTTGTTTA	14	<hkxhk>	hh	56	hk	129	kk	45
>ref-100195	CGGTGGCTGACGGGCGCTCCTTTCCTT	15	<lmxll>	ll	120	lm	145		
>ref-100253	CTGCATCGCACTGTATCTCCTCGCTTG	3	<nnxnp>	nn	139	np	131		
>ref-100261	TTCTAGGTCACGCTCTCTCCTCTCCCT	1	<lmxll>	ll	112	lm	128		
>ref-100268	ATCCTTAAAACCTTATCTCCAACACCA	14	<lmxll>	ll	109	lm	129		
>ref-100268	ATCCTTAAAACCTTATCTCCAACACCA	22	<nnxnp>	nn	117	np	120		
>ref-100275	ATAGTCTTAACCTTTACTCCATGTGAC	4	<nnxnp>	nn	136	np	109		
>ref-100280	ACACACACAACAACCCCTCCTTCCACA	12	<nnxnp>	nn	167	np	97		
>ref-100280	ACACACACAACAACCCCTCCTTCCACA	24	<nnxnp>	nn	167	np	97		
>ref-100281	TCTGTTTCTACTCTCCCTCCTCCTCTC	22	<lmxll>	ll	48	lm	212		
>ref-100281	TCTGTTTCTACTCTCCCTCCTCCTCTC	27	<lmxll>	ll	126	lm	130		
>ref-100283	CGCTGTAACACTGATACTCCAAGTCTT	21	<nnxnp>	nn	114	np	147		
>ref-100285	GACGGCAAAACACATCCTCCCTCACTC	7	<lmxll>	ll	129	lm	143		
>ref-100296	TTCCCCTTCACTTTACCTCCTTCTCTC	7	<nnxnp>	nn	4	np	262		
>ref-100296	TTCCCCTTCACTTTACCTCCTTCTCTC	15	<nnxnp>	nn	3	np	264		
>ref-100296	TTCCCCTTCACTTTACCTCCTTCTCTC	25	<lmxll>	ll	197	lm	58		
>ref-100303	CTCCCTTCCACTCCCACTCCCAATCTT	22	<nnxnp>	nn	90	np	158		
>ref-100303	CTCCCTTCCACTCCCACTCCCAATCTT	23	<nnxnp>	nn	80	np	157		
>ref-100304	CACCCCTCCACTCCTCCTCCCACACCT	4	<nnxnp>	nn	51	np	206		
>ref-100304	CACCCCTCCACTCCTCCTCCCACACCT	21	<hkxhk>	hh	0	hk	245	kk	22

>ref-100319	GGCGTCGAAACATAGACTCCACTCGCT	1	<nnxnp>	nn	139	np	130		
>ref-100319	GGCGTCGAAACATAGACTCCACTCGCT	4	<nnxnp>	nn	139	np	130		
>ref-100319	GGCGTCGAAACATAGACTCCACTCGCT	9	<hkxhk>	hh	1	hk	198	kk	68
>ref-100330	CGCTGCACCACACAACCTCCGCCACTG	26	<lmxll>	ll	117	lm	139		
>ref-100330	CGCTGCACCACACAACCTCCGCCACTG	27	<lmxll>	ll	117	lm	139		
>ref-100337	ACTCACCTTACATTACCTCCCTTTATC	23	<nnxnp>	nn	124	np	107		
>ref-100386	GTAAATTAGACACGGGCTCCGGCGCCG	23	<lmxll>	ll	112	lm	139		
>ref-100394	TCTCTTCTCACCCTATCTCCTCCTTCC	14	<nnxnp>	nn	119	np	144		
>ref-100394	TCTCTTCTCACCCTATCTCCTCCTTCC	27	<nnxnp>	nn	115	np	144		
>ref-100426	TCCTCCACCACCTATACTCCACGCCTT	12	<nnxnp>	nn	125	np	143		
>ref-100428	AAGCGGGCGACGTCGGCTCCCGTGTCT	16	<hkxhk>	hh	0	hk	270	kk	0
>ref-100428	AAGCGGGCGACGTCGGCTCCCGTGTCT	25	<hkxhk>	hh	0	hk	270	kk	0
>ref-100462	CCCTATTCAACCCTACCTCCCTCTACC	16	<nnxnp>	nn	134	np	107		
>ref-100488	TCCCTGACAACACTAACTCCCCCCCAA	25	<hkxhk>	hh	0	hk	102	kk	121
>ref-100543	TCCACCAACACCTCTCCTCCCTGGTCA	22	<lmxll>	ll	128	lm	135		
>ref-100581	CAGTTTCCCACTCTCTCTCCTCCTCTC	3	<nnxnp>	nn	116	np	131		
>ref-100581	CAGTTTCCCACTCTCTCTCCTCCTCTC	27	<lmxll>	ll	220	lm	0		
>ref-100596	TAAATCTCAACAAAGCCTCCCTCTCCC	26	<lmxll>	ll	119	lm	136		
>ref-100603	CCTATAGTTACCTCCTCTCCTCTTAAC	2	<nnxnp>	nn	124	np	137		
>ref-100603	CCTATAGTTACCTCCTCTCCTCTTAAC	21	<nnxnp>	nn	124	np	137		
>ref-100607	AACACGGGCACGCACGCTCCCACACGC	26	<nnxnp>	nn	272	np	0		
>ref-100607	AACACGGGCACGCACGCTCCCACACGC	27	<nnxnp>	nn	272	np	0		
>ref-100611	CTGCTTATCACACGCCCTCCCCGTACC	24	<nnxnp>	nn	151	np	115		
>ref-100617	TGTGTTATCACTTCCACTCCTCCCTGT	24	<nnxnp>	nn	131	np	140		
>ref-100617	TGTGTTATCACTTCCACTCCTCCCTGT	26	<hkxhk>	hh	0	hk	129	kk	133
>ref-100619	CACTTAATGACCCCTCCTCCACCCCCA	13	<lmxll>	ll	142	lm	124		
>ref-100619	CACTTAATGACCCCTCCTCCACCCCCA	22	<lmxll>	ll	261	lm	7		
>ref-100622	CTCACACAAACCCATCCTCCCATTTTA	24	<nnxnp>	nn	147	np	113		
>ref-100622	CTCACACAAACCCATCCTCCCATTTTA	26	<nnxnp>	nn	205	np	45		
>ref-100629	ATGCCGGGCACACTGCCTCCTGTTGGT	8	<lmxll>	ll	120	lm	144		
>ref-100640	CCACCTTCCACTCTCTCTCCCATTCCC	3	<lmxll>	ll	209	lm	51		
>ref-100640	CCACCTTCCACTCTCTCTCCCATTCCC	7	<hkxhk>	hh	2	hk	269	kk	0
>ref-100640	CCACCTTCCACTCTCTCTCCCATTCCC	13	<hkxhk>	hh	1	hk	267	kk	0
>ref-100640	CCACCTTCCACTCTCTCTCCCATTCCC	21	<nnxnp>	nn	184	np	75		
>ref-100640	CCACCTTCCACTCTCTCTCCCATTCCC	24	<hkxhk>	hh	0	hk	233	kk	1
>ref-100661	AAATAGGGAACTTCCTCTCCTTCAAAT	26	<lmxll>	ll	119	lm	128		
>ref-100678	CCCAAACCGACCTAAACTCCACCCTTA	25	<hkxhk>	hh	12	hk	256	kk	0
>ref-100678	CCCAAACCGACCTAAACTCCACCCTTA	27	<hkxhk>	hh	0	hk	256	kk	12

>ref-100716	GTTTCTCATACTCTGCCTCCGACGGAC	23	<hkxhk>	hh	52	hk	153	kk	60
>ref-100716	GTTTCTCATACTCTGCCTCCGACGGAC	27	<hkxhk>	hh	52	hk	152	kk	60
>ref-100721	CCTCCTGCTACTGTTGCTCCTCCTCCA	1	<lmxll>	ll	137	lm	133		
>ref-100721	CCTCCTGCTACTGTTGCTCCTCCTCCA	9	<lmxll>	ll	138	lm	133		
>ref-100738	TTTTTCTTAACACTCTCTCCTTTATCA	9	<nnxnp>	nn	152	np	82		
>ref-100738	TTTTTCTTAACACTCTCTCCTTTATCA	12	<hkxhk>	hh	82	hk	157	kk	0
>ref-100738	TTTTTCTTAACACTCTCTCCTTTATCA	16	<hkxhk>	hh	1	hk	226	kk	17
>ref-100740	CCTCCTGTCACTCCTGCTCCTCTTCTC	6	<nnxnp>	nn	148	np	92		
>ref-100755	CTTCCTGCCACCTCTCCTCCGTTGCCT	12	<lmxll>	ll	122	lm	146		
>ref-100755	CTTCCTGCCACCTCTCCTCCGTTGCCT	21	<nnxnp>	nn	145	np	121		
>ref-100755	CTTCCTGCCACCTCTCCTCCGTTGCCT	27	<nnxnp>	nn	145	np	121		
>ref-100761	TCTGCCCAAACTTTCCCTCCCTGGCTA	9	<hkxhk>	hh	73	hk	182	kk	0
>ref-100761	TCTGCCCAAACTTTCCCTCCCTGGCTA	15	<lmxll>	ll	171	lm	79		
>ref-100779	TAAGCATGTACGAAACCTCCGTCAGAT	16	<lmxll>	ll	112	lm	122		
>ref-100791	TCTCTCCACACCACCTCTCCTCTCCTT	5	<lmxll>	ll	8	lm	263		
>ref-100791	TCTCTCCACACCACCTCTCCTCTCCTT	13	<lmxll>	ll	8	lm	263		
>ref-100805	ATTCTCCAAACGTAGTCTCCTGTCCTG	23	<nnxnp>	nn	224	np	0		
>ref-100810	GTCGAGCCCACACCAGCTCCAGTCGAA	6	<hkxhk>	hh	13	hk	223	kk	0
>ref-100810	GTCGAGCCCACACCAGCTCCAGTCGAA	27	<hkxhk>	hh	6	hk	233	kk	0
>ref-100819	GTAACGATCACAAGCCCTCCGTCTCTA	12	<lmxll>	ll	135	lm	131		
>ref-100819	GTAACGATCACAAGCCCTCCGTCTCTA	22	<nnxnp>	nn	139	np	122		
>ref-100844	TACCTTTCAACACCATCTCCACCATTA	7	<hkxhk>	hh	0	hk	271	kk	1
>ref-100844	TACCTTTCAACACCATCTCCACCATTA	14	<nnxnp>	nn	142	np	119		
>ref-100845	TCCTCTTATACTCCCTCTCCCCTTCTG	3	<nnxnp>	nn	129	np	138		
>ref-100846	TGATCCATTACATTCTCTCCGGTGAAA	5	<lmxll>	ll	116	lm	125		
>ref-100855	ATCTCGAGGACTCTATCTCCCCTCCCT	5	<lmxll>	ll	135	lm	127		
>ref-100876	CACAACATAACACTCCCTCCTGGTCCT	14	<lmxll>	ll	131	lm	134		
>ref-100879	AACTCTCTTACTCTTCCTCCCCTCGCT	27	<hkxhk>	hh	35	hk	156	kk	65
>ref-100880	TCTCCGGACACCTCCTCTCCCAACATT	21	<nnxnp>	nn	136	np	118		
>ref-100887	GAGGCCGTCACCGCTGCTCCCTCAGCC	14	<lmxll>	ll	151	lm	113		
>ref-100892	TACGTACCTACTCCTACTCCTTGACAC	3	<lmxll>	ll	139	lm	124		
>ref-100929	CTCCATGGCACCGCTCCTCCTCTTCCT	4	<lmxll>	ll	114	lm	156		
>ref-100951	TTTTCCCCAACTCCTACTCCCTTCCCC	1	<lmxll>	ll	38	lm	209		
>ref-100951	TTTTCCCCAACTCCTACTCCCTTCCCC	9	<lmxll>	ll	38	lm	209		
>ref-100971	GACTCTGCAACAGACTCTCCGGCTGTA	2	<hkxhk>	hh	49	hk	133	kk	57
>ref-100982	CCGCGCATAACTCAGCCTCCTTCAGGA	2	<nnxnp>	nn	129	np	138		
>ref-100988	CTCCTATTAACCCTCCCTCCATTCATC	13	<lmxll>	ll	103	lm	143		
>ref-101002	CTGTGCTACACTATTCCTCCCATGAAG	2	<hkxhk>	hh	4	hk	233	kk	2

>ref-101002	CTGTGCTACACTATTCCTCCCATGAAG	12	<hkxhk>	hh	4	hk	234	kk	2
>ref-101011	CATACCCACACTATCTCTCCAACATCC	13	<hkxhk>	hh	33	hk	231	kk	0
>ref-101011	CATACCCACACTATCTCTCCAACATCC	24	<hkxhk>	hh	0	hk	273	kk	0
>ref-101011	CATACCCACACTATCTCTCCAACATCC	26	<hkxhk>	hh	9	hk	234	kk	0
>ref-101016	AGCCATACCACTCAAGCTCCGCGTTGG	14	<hkxhk>	hh	48	hk	151	kk	47
>ref-101027	CCCATATTCACTCTCCCTCCCTATCCT	3	<hkxhk>	hh	2	hk	178	kk	69
>ref-101032	CGCAAACATACACTCGCTCCCACACAC	16	<lmxll>	ll	129	lm	131		
>ref-101041	CCGTCGATCACTTTCTCTCCAACACCA	7	<lmxll>	ll	100	lm	160		
>ref-101064	TTGCAGGGGACACAGCCTCCTGAATTA	23	<hkxhk>	hh	2	hk	266	kk	1
>ref-101064	TTGCAGGGGACACAGCCTCCTGAATTA	27	<hkxhk>	hh	2	hk	268	kk	0
>ref-101100	TTCTCTTTTACTTCCTCTCCTTTTCCT	1	<lmxll>	ll	3	lm	267		
>ref-101100	TTCTCTTTTACTTCCTCTCCTTTTCCT	4	<lmxll>	ll	3	lm	266		
>ref-101100	TTCTCTTTTACTTCCTCTCCTTTTCCT	8	<hkxhk>	hh	0	hk	209	kk	57
>ref-101100	TTCTCTTTTACTTCCTCTCCTTTTCCT	22	<hkxhk>	hh	0	hk	209	kk	57
>ref-101114	GAATGAAAAACACTGGCTCCCCGCAAC	26	<nnxnp>	nn	142	np	120		
>ref-101122	TCTACTTCTACTTCTTCTCCAAGCGTC	4	<nnxnp>	nn	130	np	140		
>ref-101126	TCCTGTTCCACATCCTCTCCCACCTCT	3	<lmxll>	ll	136	lm	135		
>ref-101127	CAGCTGGGCACAGAGTCTCCGTCTGAG	21	<lmxll>	ll	142	lm	129		
>ref-101149	TATTACGTTACGTATTCTCCCTCTCAT	1	<lmxll>	ll	119	lm	122		
>ref-101159	CAGGCAAAAACCTGCCCTCCGCCTATC	2	<lmxll>	ll	1	lm	266		
>ref-101159	CAGGCAAAAACCTGCCCTCCGCCTATC	8	<lmxll>	ll	1	lm	266		
>ref-101161	CCCTCCCACACCTATCCTCCCTTCCTC	2	<nnxnp>	nn	12	np	246		
>ref-101161	CCCTCCCACACCTATCCTCCCTTCCTC	8	<nnxnp>	nn	12	np	245		
>ref-101161	CCCTCCCACACCTATCCTCCCTTCCTC	14	<nnxnp>	nn	6	np	261		
>ref-101161	CCCTCCCACACCTATCCTCCCTTCCTC	25	<nnxnp>	nn	8	np	261		
>ref-101163	GCCAATACAACGCTCTCTCCATTCTAG	24	<hkxhk>	hh	63	hk	131	kk	72
>ref-101170	TACGTAATAACACTTTCTCCTGCTTAA	9	<lmxll>	ll	128	lm	117		
>ref-101198	ATTCCTCCTACTACTACTCCTCTTGGT	8	<hkxhk>	hh	59	hk	138	kk	56
>ref-101211	TTCATCACAACGAACTCTCCTTCATGC	27	<lmxll>	ll	131	lm	134		
>ref-101223	ATAATTCTCACATCCCCTCCACCAATG	15	<hkxhk>	hh	46	hk	122	kk	64
>ref-101236	TTCTTGTCCACATAGTCTCCACCCTTT	15	<nnxnp>	nn	133	np	132		
>ref-101236	TTCTTGTCCACATAGTCTCCACCCTTT	26	<nnxnp>	nn	134	np	132		
>ref-101241	TTCCAACCAACAACATCTCCAAACTAT	23	<lmxll>	ll	93	lm	127		
>ref-101246	TCACCCTTCACCCCCTCTCCCTCCACT	2	<nnxnp>	nn	265	np	2		
>ref-101246	TCACCCTTCACCCCCTCTCCCTCCACT	25	<nnxnp>	nn	246	np	8		
>ref-101256	TAAACAAAAACTGGATCTCCGTTATTT	22	<nnxnp>	nn	117	np	116		
>ref-101266	CCATTTCCCACGTACTCTCCTCTTCTC	2	<hkxhk>	hh	0	hk	156	kk	98
>ref-101266	CCATTTCCCACGTACTCTCCTCTTCTC	4	<hkxhk>	hh	0	hk	273	kk	0

>ref-101266	CCATTTCCCACGTACTCTCCTCTTCTC	14	<hkxhk>	hh	0	hk	273	kk	0
>ref-101271	TAACCGCAAACTTACTCTCCGTCTTCT	6	<hkxhk>	hh	2	hk	271	kk	0
>ref-101271	TAACCGCAAACTTACTCTCCGTCTTCT	7	<lmxll>	ll	168	lm	100		
>ref-101278	TTCTGTAATACAATGTCTCCATCGTTT	7	<hkxhk>	hh	58	hk	142	kk	54
>ref-101278	TTCTGTAATACAATGTCTCCATCGTTT	16	<nnxnp>	nn	127	np	130		
>ref-101287	CATATCTATACATCCTCTCCGTCTTTT	21	<lmxll>	ll	67	lm	183		
>ref-101287	CATATCTATACATCCTCTCCGTCTTTT	26	<lmxll>	ll	67	lm	183		
>ref-101307	TTCATTCTCACTGTACCTCCATCTCTT	21	<lmxll>	ll	126	lm	130		
>ref-101332	ATAAAGAGAACAGTCACTCCCGAAACA	9	<lmxll>	ll	117	lm	119		
>ref-101342	CTACCTTTCACTTCGTCTCCCTCTTTT	24	<lmxll>	ll	8	lm	265		
>ref-101342	CTACCTTTCACTTCGTCTCCCTCTTTT	27	<lmxll>	ll	8	lm	265		
>ref-101350	TGCGTTCCCACTCATCCTCCTTCCGTT	21	<lmxll>	ll	130	lm	135		
>ref-101360	CTCAGTTGAACTTGCTCTCCGATACTT	1	<nnxnp>	nn	104	np	140		
>ref-101368	CTATCCCCCACCCCCCCTCCTTTCCTT	2	<lmxll>	ll	103	lm	147		
>ref-101368	CTATCCCCCACCCCCCCTCCTTTCCTT	3	<lmxll>	ll	13	lm	245		
>ref-101368	CTATCCCCCACCCCCCCTCCTTTCCTT	22	<lmxll>	ll	13	lm	246		
>ref-101380	CCTCTTCTTACACATCCTCCTTTCCCT	13	<hkxhk>	hh	41	hk	141	kk	60
>ref-101388	CTCTCGTTCACCCTTTCTCCCTCTGGA	27	<nnxnp>	nn	100	np	122		
>ref-101391	ACCATTACTACTATTGCTCCCGCTACC	5	<lmxll>	ll	153	lm	105		
>ref-101391	ACCATTACTACTATTGCTCCCGCTACC	8	<lmxll>	ll	153	lm	105		
>ref-101399	CTCTCTGTCACACTGTCTCCTTCATCG	5	<nnxnp>	nn	151	np	121		
>ref-101402	AGACACTTCACTCGATCTCCAAGGACT	26	<lmxll>	ll	125	lm	125		
>ref-101407	CTATATCCAACCATCCCTCCGTCATCC	14	<nnxnp>	nn	114	np	141		
>ref-101421	TAAGCCCCTACTTTATCTCCCTTTTCT	2	<hkxhk>	hh	6	hk	246	kk	5
>ref-101421	TAAGCCCCTACTTTATCTCCCTTTTCT	14	<hkxhk>	hh	0	hk	234	kk	17
>ref-101448	CACCGCCCGACGAGGTCTCCTCCCGAA	24	<nnxnp>	nn	125	np	143		
>ref-101464	AAGCCCAGCACATCTCCTCCTTCCGGA	12	<lmxll>	ll	144	lm	121		
>ref-101471	AACCTCCTCACCCACTCTCCCTCTCCA	7	<hkxhk>	hh	1	hk	270	kk	0
>ref-101477	ACACTAAATACTTGAACTCCTTAACTT	3	<lmxll>	ll	144	lm	118		
>ref-101477	ACACTAAATACTTGAACTCCTTAACTT	6	<lmxll>	ll	145	lm	116		
>ref-101524	ATGACATTTACTGAATCTCCTACACCT	13	<lmxll>	ll	119	lm	116		
>ref-101553	TCCCTTCCAACGTTGCCTCCTCTCTCT	25	<lmxll>	ll	95	lm	165		
>ref-101560	TAATTTTCTACCTGCTCTCCATCTCTT	1	<hkxhk>	hh	0	hk	268	kk	1
>ref-101574	AGCACCGTCACGGATACTCCCTTCTAT	4	<nnxnp>	nn	123	np	135		
>ref-101574	AGCACCGTCACGGATACTCCCTTCTAT	12	<nnxnp>	nn	120	np	136		
>ref-101582	ATATCAATCACTATTCCTCCCTCTCTC	6	<hkxhk>	hh	135	hk	111	kk	0
>ref-101633	CTCATCTCAACCACTCCTCCTCCTCCT	9	<lmxll>	ll	131	lm	125		
>ref-101633	CTCATCTCAACCACTCCTCCTCCTCCT	13	<lmxll>	ll	111	lm	123		

>ref-101636	ATTTCACCCACTCCCCCTCCTCCCTCT	1	<lmxll>	ll	73	lm	162		
>ref-101636	ATTTCACCCACTCCCCCTCCTCCCTCT	27	<lmxll>	ll	73	lm	160		
>ref-101683	TGAGGGCCAACCCCATCTCCTTTCGCA	2	<hkxhk>	hh	56	hk	153	kk	55
>ref-101684	ACTCTCACCACAACGGCTCCACTTAAG	21	<hkxhk>	hh	48	hk	141	kk	56
>ref-101690	ATCGGTCCTACGTTTCCTCCCTACGGT	12	<hkxhk>	hh	46	hk	135	kk	57
>ref-101698	TGATAACACACTTCATCTCCACAGCAT	7	<lmxll>	ll	121	lm	104		
>ref-101708	TTCTATTTCACTCCTTCTCCCTTCTCC	1	<hkxhk>	hh	0	hk	271	kk	1
>ref-101708	TTCTATTTCACTCCTTCTCCCTTCTCC	16	<hkxhk>	hh	0	hk	271	kk	1
>ref-101718	TCCCCATTTACCTTCCCTCCTTCCCTT	7	<hkxhk>	hh	0	hk	262	kk	0
>ref-101730	AAGAGCATAACAATGTCTCCTTAAACC	2	<lmxll>	ll	124	lm	105		
>ref-101730	AAGAGCATAACAATGTCTCCTTAAACC	15	<lmxll>	ll	124	lm	105		
>ref-101800	TGTTCTGTGACCCTTCCTCCCTTAAAT	5	<lmxll>	ll	139	lm	118		
>ref-101807	AGAGGGGACACCATCCCTCCTGCTCTA	24	<hkxhk>	hh	50	hk	132	kk	47
>ref-101810	TCCTTCCCCACTTATTCTCCTGCCTCC	7	<hkxhk>	hh	71	hk	139	kk	25
>ref-101818	CACGCATGTACGCTCTCTCCTGTACAC	4	<nnxnp>	nn	130	np	132		
>ref-101852	TCCTCTTCTACCTACCCTCCCTTCTCC	8	<lmxll>	ll	5	lm	262		
>ref-101852	TCCTCTTCTACCTACCCTCCCTTCTCC	22	<lmxll>	ll	5	lm	262		
>ref-101872	CCCACTTACACCCACCCTCCTCCCTCT	24	<lmxll>	ll	162	lm	98		
>ref-101872	CCCACTTACACCCACCCTCCTCCCTCT	25	<lmxll>	ll	161	lm	99		
>ref-101918	AACTCCTCTACTTCGTCTCCATAACTC	25	<nnxnp>	nn	144	np	103		
>ref-101949	TCCTCCCCTACCACACCTCCCTACCTT	9	<lmxll>	ll	263	lm	0		
>ref-101949	TCCTCCCCTACCACACCTCCCTACCTT	23	<lmxll>	ll	260	lm	0		
>ref-101965	CTTTGTCTCACCCTACCTCCCTCCCTT	21	<nnxnp>	nn	239	np	1		
>ref-101965	CTTTGTCTCACCCTACCTCCCTCCCTT	27	<lmxll>	ll	219	lm	6		
>ref-101967	CACGCATTTACTCACTCTCCTTCACCT	25	<nnxnp>	nn	153	np	117		
>ref-101984	CTCTCCTACACCTACACTCCTCCCTCT	13	<lmxll>	ll	138	lm	127		
>ref-101986	TCTCTTTCTACATCTCCTCCATCGTTC	8	<nnxnp>	nn	122	np	134		
>ref-101999	TCCTCACTCACCATCCCTCCTTTCCCT	12	<lmxll>	ll	239	lm	22		
>ref-101999	TCCTCACTCACCATCCCTCCTTTCCCT	13	<lmxll>	ll	240	lm	22		
>ref-102074	TCACTCCCTACACCTGCTCCCTCGGAT	13	<lmxll>	ll	118	lm	146		
>ref-102074	TCACTCCCTACACCTGCTCCCTCGGAT	25	<lmxll>	ll	141	lm	122		
>ref-102076	CACATACCCACACACTCTCCCCTTCCC	22	<nnxnp>	nn	128	np	138		
>ref-102080	TTCCTTCCTACGCTTCCTCCTCCTTCT	3	<nnxnp>	nn	252	np	7		
>ref-102080	TTCCTTCCTACGCTTCCTCCTCCTTCT	12	<nnxnp>	nn	195	np	31		
>ref-102089	CCCTTACACACCTTTCCTCCATTTTCC	12	<nnxnp>	nn	118	np	144		
>ref-102124	CTCGCCTCTACTGTCTCTCCTTCCGAG	23	<lmxll>	ll	129	lm	135		
>ref-102126	CCGATGTACACGGCCGCTCCACGAATG	22	<nnxnp>	nn	132	np	123		
>ref-102137	AACCCCCACACATCGCCTCCCACATAT	12	<hkxhk>	hh	60	hk	159	kk	41

>ref-102143	GTCACTCTCACTCTCACTCCTTTTCTC	24	<hkxhk>	hh	0	hk	53	kk	210
>ref-102146	ACCCTTGGCACAACCCCTCCCTCTCAT	26	<hkxhk>	hh	0	hk	272	kk	0
>ref-102158	TGATGTTCCACTCAGGCTCCAGAGGCC	7	<nnxnp>	nn	118	np	128		
>ref-102175	CCACACGAAACAGATGCTCCTCACATA	23	<nnxnp>	nn	109	np	152		
>ref-102181	TCTCGATACACGACCTCTCCAGGTGCC	9	<hkxhk>	hh	1	hk	142	kk	124
>ref-102182	AACCTCTATACCCTTTCTCCTTTTCTC	1	<hkxhk>	hh	0	hk	252	kk	4
>ref-102182	AACCTCTATACCCTTTCTCCTTTTCTC	13	<hkxhk>	hh	4	hk	252	kk	0
>ref-102186	CCGGCCCCCACTCTTCCTCCTTGCCTG	5	<lmxll>	ll	129	lm	124		
>ref-102210	GAGGCCCTTACTCCTCCTCCTCCTCCT	27	<nnxnp>	nn	126	np	140		
>ref-102225	TTCTCCAAAACCACATCTCCGCACTTT	9	<hkxhk>	hh	0	hk	270	kk	0
>ref-102225	TTCTCCAAAACCACATCTCCGCACTTT	21	<hkxhk>	hh	0	hk	271	kk	0
>ref-102227	CCCTCCTTGACTCGCCCTCCTTCCTCC	9	<nnxnp>	nn	0	np	257		
>ref-102227	CCCTCCTTGACTCGCCCTCCTTCCTCC	14	<nnxnp>	nn	0	np	257		
>ref-102231	CCAGCGAATACACATCCTCCAGCGGAG	4	<nnxnp>	nn	106	np	148		
>ref-102253	CTGTTATGGACGGTGCCTCCTTTACTA	1	<hkxhk>	hh	0	hk	220	kk	24
>ref-102253	CTGTTATGGACGGTGCCTCCTTTACTA	3	<hkxhk>	hh	24	hk	220	kk	0
>ref-102276	CCTCTTTCTACTCTTCCTCCTCTCCTA	9	<nnxnp>	nn	127	np	134		
>ref-102285	CCCCGTCACACTTCCTCTCCCCTCTCA	27	<lmxll>	ll	113	lm	144		
>ref-102295	CGAGCTTTTACACACACTCCTTGTGGG	2	<lmxll>	ll	27	lm	222		
>ref-102295	CGAGCTTTTACACACACTCCTTGTGGG	5	<hkxhk>	hh	2	hk	230	kk	32
>ref-102295	CGAGCTTTTACACACACTCCTTGTGGG	6	<nnxnp>	nn	161	np	98		
>ref-102295	CGAGCTTTTACACACACTCCTTGTGGG	21	<lmxll>	ll	59	lm	196		
>ref-102295	CGAGCTTTTACACACACTCCTTGTGGG	27	<lmxll>	ll	60	lm	195		
>ref-102312	TCCCCACCCACTCTGCCTCCTCCTTCC	4	<nnxnp>	nn	268	np	0		
>ref-102312	TCCCCACCCACTCTGCCTCCTCCTTCC	15	<lmxll>	ll	27	lm	229		
>ref-102389	TCCGCCACCACCAACACTCCACCATCA	8	<hkxhk>	hh	0	hk	267	kk	2
>ref-102389	TCCGCCACCACCAACACTCCACCATCA	14	<hkxhk>	hh	0	hk	267	kk	2
>ref-102390	ACTCCTTCAACTCACTCTCCCCCTCCC	15	<hkxhk>	hh	21	hk	215	kk	0
>ref-102390	ACTCCTTCAACTCACTCTCCCCCTCCC	21	<hkxhk>	hh	21	hk	214	kk	0
>ref-102391	CAGTCGACGACCCCCTCTCCTGAAGCT	14	<lmxll>	ll	130	lm	93		
>ref-102413	TCGTATCCTACCCTACCTCCACCCTAC	3	<lmxll>	ll	120	lm	145		
>ref-102440	CATACACACACAGAAGCTCCAAAACAC	25	<lmxll>	ll	176	lm	75		
>ref-102446	TCATCACCCACCTTTTCTCCCTGGTCA	2	<lmxll>	ll	108	lm	153		
>ref-102450	TCACTCAAAACTCCCTCTCCCTCTCTT	8	<hkxhk>	hh	6	hk	258	kk	0
>ref-102450	TCACTCAAAACTCCCTCTCCCTCTCTT	12	<hkxhk>	hh	0	hk	257	kk	7
>ref-102455	CACTCACCTACTCACTCTCCTCTTGAT	5	<nnxnp>	nn	141	np	83		
>ref-102490	CTTTCTCCAACAACTCCTCCAACAACT	15	<lmxll>	ll	12	lm	257		
>ref-102490	CTTTCTCCAACAACTCCTCCAACAACT	25	<lmxll>	ll	12	lm	257		

>ref-102499	CTCTATTCTACTGTCACTCCCTCCCCT	14	<lmxll>	ll	116	lm	130		
>ref-102499	CTCTATTCTACTGTCACTCCCTCCCCT	16	<lmxll>	ll	116	lm	129		
>ref-102504	TCACCCCCCACCCCCGCTCCCCCTCTC	1	<lmxll>	ll	66	lm	161		
>ref-102529	TTTTATCTTACTTTCGCTCCTGTTCTC	13	<lmxll>	ll	126	lm	126		
>ref-102543	TACCCACCAACCCTTTCTCCATCACCC	1	<hkxhk>	hh	0	hk	267	kk	3
>ref-102543	TACCCACCAACCCTTTCTCCATCACCC	26	<hkxhk>	hh	3	hk	267	kk	0
>ref-102547	ATCATTACAACCTCCTCTCCCCACATC	24	<hkxhk>	hh	4	hk	258	kk	1
>ref-102547	ATCATTACAACCTCCTCTCCCCACATC	26	<hkxhk>	hh	1	hk	258	kk	4
>ref-102556	TTTCTGTCTACCCCCTCTCCCCCCATT	6	<lmxll>	ll	29	lm	192		
>ref-102556	TTTCTGTCTACCCCCTCTCCCCCCATT	27	<lmxll>	ll	29	lm	193		
>ref-102559	TGCACTCCGACCACCGCTCCTGTCTTG	26	<nnxnp>	nn	130	np	137		
>ref-102565	ATTCCCCTAACCCACTCTCCCCCAACC	2	<lmxll>	ll	44	lm	180		
>ref-102565	ATTCCCCTAACCCACTCTCCCCCAACC	23	<lmxll>	ll	45	lm	180		
>ref-102566	ATTCACGTTACTCTATCTCCATTTCTC	9	<lmxll>	ll	264	lm	0		
>ref-102571	CTTACACGCACGCTCGCTCCGTGTCAG	7	<lmxll>	ll	141	lm	119		
>ref-102589	TGACCAGTGACTCTGCCTCCTTAACCC	9	<nnxnp>	nn	130	np	141		
>ref-102598	CCACGCTCGACCCCTCCTCCATCCATC	15	<hkxhk>	hh	63	hk	138	kk	53
>ref-102610	TCTCTTCCAACTTTCCCTCCTACTCTC	12	<hkxhk>	hh	0	hk	269	kk	1
>ref-102610	TCTCTTCCAACTTTCCCTCCTACTCTC	21	<hkxhk>	hh	0	hk	269	kk	1
>ref-102611	CAAGGCCTAACTGTGTCTCCTTACCTT	4	<hkxhk>	hh	0	hk	263	kk	7
>ref-102611	CAAGGCCTAACTGTGTCTCCTTACCTT	13	<lmxll>	ll	8	lm	258		
>ref-102612	TGGCCGGCCACAGTGCCTCCCTCTTTC	3	<lmxll>	ll	125	lm	141		
>ref-102613	TCTTTCTTCACCCCCCCCTCCCCCGCTT	4	<hkxhk>	hh	59	hk	173	kk	3
>ref-102613	TCTTTCTTCACCCCCCCCTCCCCCGCTT	9	<hkxhk>	hh	58	hk	173	kk	3
>ref-102624	AACCAAGGTACTTAAACTCCTGGACTC	16	<nnxnp>	nn	89	np	172		
>ref-102624	AACCAAGGTACTTAAACTCCTGGACTC	22	<nnxnp>	nn	92	np	156		
>ref-102626	CCAACTGTGACTCAGCCTCCTGCAGCA	25	<nnxnp>	nn	126	np	143		
>ref-102626	CCAACTGTGACTCAGCCTCCTGCAGCA	26	<nnxnp>	nn	126	np	143		
>ref-102645	TGCTAGGGTACCTTGTCTCCTGTCCAG	12	<hkxhk>	hh	54	hk	122	kk	54
>ref-102699	AGCCAGCTCACCATTGCTCCATCCAGG	24	<nnxnp>	nn	272	np	0		
>ref-102724	TTAAGACGTACTCCTCCTCCTATCGCT	5	<lmxll>	ll	125	lm	142		
>ref-102731	TTAGTGCTAACCTTCCCTCCTTTTCCT	7	<lmxll>	ll	138	lm	126		
>ref-102735	ATTTGCATGACAGCTGCTCCTTCCTAT	3	<nnxnp>	nn	133	np	131		
>ref-102758	GTTTCCCGCACCGACTCTCCTTACACC	23	<nnxnp>	nn	119	np	119		
>ref-102758	GTTTCCCGCACCGACTCTCCTTACACC	25	<nnxnp>	nn	115	np	120		
>ref-102774	CTACCCATCACTCACTCTCCATTACCC	9	<lmxll>	ll	159	lm	111		
>ref-102774	CTACCCATCACTCACTCTCCATTACCC	26	<hkxhk>	hh	0	hk	273	kk	0
>ref-102791	CGTGGCATTACCATTTCTCCGTATTCA	6	<nnxnp>	nn	138	np	116		

>ref-102811	ACACACACGACACGACCTCCTCGTGTA	16	<nnxnp>	nn	129	np	140		
>ref-102821	CCTGTATCTACGCCACCTCCTCCTTCT	5	<hkxhk>	hh	53	hk	123	kk	68
>ref-102827	TACTCTTCCACTCCTCCTCCTCCTTTA	21	<lmxll>	ll	7	lm	262		
>ref-102827	TACTCTTCCACTCCTCCTCCTCCTTTA	24	<lmxll>	ll	7	lm	262		
>ref-102830	AATTGTTGAACATTTACTCCTCCACTA	24	<hkxhk>	hh	45	hk	129	kk	63
>ref-102859	AGTGTTTTGACTTTGACTCCCTCCCTC	24	<hkxhk>	hh	1	hk	266	kk	5
>ref-102861	TCTATCTGTACCCCCCCTCCCCATATA	8	<nnxnp>	nn	100	np	138		
>ref-102861	TCTATCTGTACCCCCCCTCCCCATATA	24	<nnxnp>	nn	102	np	138		
>ref-102892	TTCTGACCCACCGCCCCTCCCCATTTC	3	<lmxll>	ll	115	lm	121		
>ref-102894	TGCGTATGTACGTACTCTCCCGGAGTG	2	<nnxnp>	nn	161	np	111		
>ref-102894	TGCGTATGTACGTACTCTCCCGGAGTG	13	<hkxhk>	hh	0	hk	207	kk	64
>ref-102894	TGCGTATGTACGTACTCTCCCGGAGTG	22	<hkxhk>	hh	0	hk	273	kk	0
>ref-102904	TTCCTACCCACTTTCCCTCCTACGGCA	6	<lmxll>	ll	107	lm	150		
>ref-102924	TTCTATTCCACCTTCCCTCCCTCTCTT	13	<lmxll>	ll	124	lm	111		
>ref-102932	ATCACATCCACCAACCCTCCCCCATCA	14	<lmxll>	ll	137	lm	127		
>ref-102934	AACGCAGAAACGTCGACTCCGTTGGCG	4	<hkxhk>	hh	0	hk	246	kk	19
>ref-102953	AAATCCCAAACCCAAACTCCCAACCTC	24	<lmxll>	ll	154	lm	81		
>ref-102953	AAATCCCAAACCCAAACTCCCAACCTC	26	<hkxhk>	hh	0	hk	206	kk	29
>ref-102953	AAATCCCAAACCCAAACTCCCAACCTC	27	<hkxhk>	hh	0	hk	154	kk	75
>ref-102963	ACCGTCCTCACCATCCCTCCCCCTCTC	4	<lmxll>	ll	42	lm	185		
>ref-102963	ACCGTCCTCACCATCCCTCCCCCTCTC	13	<lmxll>	ll	47	lm	188		
>ref-102967	TTCTCCTCCACACCACCTCCTCTCCTC	3	<nnxnp>	nn	234	np	0		
>ref-102967	TTCTCCTCCACACCACCTCCTCTCCTC	13	<nnxnp>	nn	239	np	1		
>ref-102973	CCTTCTGCCACTCCCCCTCCTGCTCCA	27	<nnxnp>	nn	269	np	0		
>ref-102975	CACTCGCCAACGGCTTCTCCAACCCGC	2	<lmxll>	ll	127	lm	107		
>ref-102993	ACGTTATTAACTCTCTCTCCCTCTCTC	3	<hkxhk>	hh	23	hk	232	kk	0
>ref-102993	ACGTTATTAACTCTCTCTCCCTCTCTC	7	<hkxhk>	hh	0	hk	231	kk	24
>ref-102998	GAAGTTAAAACAATCTCTCCTTCAGTA	8	<nnxnp>	nn	110	np	130		
>ref-102998	GAAGTTAAAACAATCTCTCCTTCAGTA	22	<hkxhk>	hh	64	hk	126	kk	46
>ref-103016	AATGCAAAGACGCCTACTCCTACACTG	23	<lmxll>	ll	208	lm	56		
>ref-103036	ACCCTCCCCACACCCTCTCCCTTTCCC	6	<nnxnp>	nn	271	np	0		
>ref-103036	ACCCTCCCCACACCCTCTCCCTTTCCC	12	<nnxnp>	nn	10	np	251		
>ref-103039	TATGTATTTACATTCCCTCCATCACAT	7	<lmxll>	ll	113	lm	150		
>ref-103056	CTCTCCTCTACCCCCTCTCCGTCTCTC	25	<nnxnp>	nn	124	np	141		
>ref-103056	CTCTCCTCTACCCCCTCTCCGTCTCTC	27	<lmxll>	ll	141	lm	126		
>ref-103061	TCCCCACATACCTTCCCTCCCATTCTC	4	<lmxll>	ll	253	lm	0		
>ref-103065	TGCATCGCCACCCTTCCTCCTCCTCTA	14	<hkxhk>	hh	49	hk	151	kk	54
>ref-103087	TTTCTCTCTACCCCGTCTCCTTCTCCC	15	<hkxhk>	hh	225	hk	1	kk	0

>ref-103087	TTTCTCTCTACCCCGTCTCCTTCTCCC	21	<nnxnp>	nn	249	np	1		
>ref-103112	ATTACTGTTACACAAACTCCTGGCTCG	1	<hkxhk>	hh	65	hk	126	kk	52
>ref-103112	ATTACTGTTACACAAACTCCTGGCTCG	5	<hkxhk>	hh	65	hk	126	kk	52
>ref-103117	CTCCATTTCACTTCCTCTCCTCCCTTC	5	<lmxll>	ll	60	lm	190		
>ref-103117	CTCCATTTCACTTCCTCTCCTCCCTTC	8	<lmxll>	ll	56	lm	197		
>ref-103145	CTCCGTGAAACTCTTTCTCCTTCTTTC	3	<lmxll>	ll	119	lm	140		
>ref-103150	GCTTGCGACACAAAACCTCCAGCATGA	6	<nnxnp>	nn	264	np	0		
>ref-103150	GCTTGCGACACAAAACCTCCAGCATGA	21	<nnxnp>	nn	119	np	128		
>ref-103175	CGTCTGAGGACAGACACTCCGCAGACT	6	<hkxhk>	hh	68	hk	146	kk	50
>ref-103177	AGACGAATAACGGTCCCTCCATTAGTT	3	<hkxhk>	hh	0	hk	272	kk	0
>ref-103177	AGACGAATAACGGTCCCTCCATTAGTT	8	<hkxhk>	hh	0	hk	271	kk	0
>ref-103177	AGACGAATAACGGTCCCTCCATTAGTT	16	<hkxhk>	hh	0	hk	272	kk	0
>ref-103184	ACCCTACGTACACATTCTCCCTCACAT	6	<hkxhk>	hh	55	hk	137	kk	62
>ref-103184	ACCCTACGTACACATTCTCCCTCACAT	25	<hkxhk>	hh	55	hk	137	kk	62
>ref-103198	CTCACCTTTACCCCTTCTCCTTCTTCT	14	<nnxnp>	nn	125	np	140		
>ref-103199	CTCGCTTCTACTAAAACTCCTTCGATG	26	<nnxnp>	nn	141	np	93		
>ref-103205	CTTCCATGTACACATCCTCCTCCCTCC	23	<hkxhk>	hh	63	hk	143	kk	49
>ref-103215	TAAACGGACACTCGGCCTCCTCTTCCT	26	<nnxnp>	nn	124	np	134		
>ref-103216	TGTTATCAAACTCTTCCTCCTTCTCTT	2	<hkxhk>	hh	0	hk	271	kk	1
>ref-103216	TGTTATCAAACTCTTCCTCCTTCTCTT	14	<hkxhk>	hh	0	hk	271	kk	1
>ref-103221	ACGCAGTCCACTGCTTCTCCCTTTTCC	16	<lmxll>	ll	122	lm	122		
>ref-103226	GTCGGTGTAACTTTCCCTCCTCACTCA	21	<hkxhk>	hh	50	hk	141	kk	55
>ref-103243	CCAAGATGGACGTGCCCTCCCTGCCTG	21	<hkxhk>	hh	0	hk	273	kk	0
>ref-103243	CCAAGATGGACGTGCCCTCCCTGCCTG	27	<hkxhk>	hh	0	hk	273	kk	0
>ref-103247	TCTGAGTTGACAACCCCTCCAAGCTAT	4	<lmxll>	ll	133	lm	114		
>ref-103258	AAGCAAAAGACCATTCCTCCACCAGCC	12	<lmxll>	ll	144	lm	127		
>ref-103263	AGCTTTACAACCTTTCCTCCCTCTTTC	2	<nnxnp>	nn	138	np	121		
>ref-103266	TGTAACTCCACCCATCCTCCTTTCCCC	8	<nnxnp>	nn	127	np	136		
>ref-103268	CGCCATCGCACAGCGGCTCCTCCAGCC	1	<lmxll>	ll	238	lm	3		
>ref-103276	CTTTCGCCCACTTACTCTCCTTTATCT	6	<lmxll>	ll	119	lm	142		
>ref-103289	CTACGCAACACACAACCTCCCCTCCCC	3	<nnxnp>	nn	218	np	1		
>ref-103296	CTTCAGACCACGCCCACTCCCGCGTTC	2	<lmxll>	ll	122	lm	141		
>ref-103311	GTTTTACTTACCCAGTCTCCCACTGTT	24	<hkxhk>	hh	1	hk	256	kk	0
>ref-103311	GTTTTACTTACCCAGTCTCCCACTGTT	26	<hkxhk>	hh	1	hk	256	kk	0
>ref-103321	ATGAAATCTACCATGCCTCCATGATGT	23	<nnxnp>	nn	119	np	140		
>ref-103337	TTCCCTTTAACTTTTCCTCCTTTTCCA	21	<nnxnp>	nn	117	np	119		
>ref-103363	TCTTTCGTGACAGCTTCTCCTTCTCCC	21	<lmxll>	ll	141	lm	109		
>ref-103371	TCCTCATTCACTTTCCCTCCTTCCTCC	23	<hkxhk>	hh	61	hk	188	kk	1

>ref-103371	TCCTCATTCACTTTCCCTCCTTCCTCC	26	<hkxhk>	hh	62	hk	189	kk	1
>ref-103398	TTCACGCCCACTTTCCCTCCCGCCGAG	27	<nnxnp>	nn	132	np	123		
>ref-103446	TCCCTTGCAACCCTCCCTCCCTTCCAC	5	<nnxnp>	nn	132	np	136		
>ref-103455	TCCCCTGACACTGCTTCTCCCTGCTTC	3	<hkxhk>	hh	7	hk	213	kk	0
>ref-103468	ATATTCTTAACTCAGCCTCCGCAGCGT	2	<nnxnp>	nn	120	np	143		
>ref-103494	CGCCCCTTCACCCCCTCTCCTTCACCC	3	<lmxll>	ll	250	lm	0		
>ref-103494	CGCCCCTTCACCCCCTCTCCTTCACCC	25	<lmxll>	ll	250	lm	0		
>ref-103497	CTTCTTTATACACTCTCTCCTTCTCTC	8	<lmxll>	ll	139	lm	102		
>ref-103514	TCCTTCAACACATACTCTCCTCGGGTC	3	<lmxll>	ll	122	lm	132		
>ref-103537	TATCTTCGGACCTCACCTCCCTCCCTC	16	<nnxnp>	nn	248	np	0		
>ref-103540	TATATTTGTACATTCACTCCTCAGCCT	3	<hkxhk>	hh	38	hk	150	kk	69
>ref-103547	TGAGGCAATACGCCGTCTCCCCAATTA	9	<nnxnp>	nn	209	np	48		
>ref-103553	TCCCCTTTCACGGCTCCTCCTCTTATC	8	<nnxnp>	nn	128	np	138		
>ref-103565	GTGTAGGTCACCGAGCCTCCACGCTTG	1	<lmxll>	ll	169	lm	103		
>ref-103590	CCACGTACAACCGACGCTCCACTTAAT	2	<hkxhk>	hh	54	hk	126	kk	42
>ref-103599	CTATGCCTCACGCGATCTCCCACTTTA	21	<nnxnp>	nn	132	np	135		
>ref-103609	TACACACGCACATACACTCCCAGCGAA	9	<lmxll>	ll	122	lm	147		
>ref-103626	AACTGGCCGACATGACCTCCCTTATTT	25	<lmxll>	ll	110	lm	128		
>ref-103630	CCACCTGTCACTCCTTCTCCTCCTCTT	9	<hkxhk>	hh	3	hk	265	kk	0
>ref-103630	CCACCTGTCACTCCTTCTCCTCCTCTT	21	<hkxhk>	hh	0	hk	265	kk	3
>ref-103645	TTCCACTTCACTCACTCTCCACTTTCC	3	<nnxnp>	nn	124	np	137		
>ref-103677	CATATATTCACTCTCTCTCCCTCCTTC	3	<lmxll>	ll	135	lm	131		
>ref-103677	CATATATTCACTCTCTCTCCCTCCTTC	26	<lmxll>	ll	136	lm	131		
>ref-103679	GTGCGCCGCACTCGCGCTCCCACGAGC	24	<nnxnp>	nn	139	np	129		
>ref-103689	GCGAAACCGACCTCTCCTCCCCAACCC	1	<hkxhk>	hh	0	hk	272	kk	0
>ref-103689	GCGAAACCGACCTCTCCTCCCCAACCC	2	<hkxhk>	hh	2	hk	268	kk	0
>ref-103689	GCGAAACCGACCTCTCCTCCCCAACCC	3	<lmxll>	ll	23	lm	237		
>ref-103689	GCGAAACCGACCTCTCCTCCCCAACCC	6	<hkxhk>	hh	2	hk	268	kk	0
>ref-103694	CCTCCCACCACTCTTCCTCCTTCACTC	9	<hkxhk>	hh	0	hk	267	kk	1
>ref-103701	CGTGTGGTAACCTGTTCTCCGTGTGCG	26	<nnxnp>	nn	108	np	146		
>ref-103705	TCCATGAACACACCTGCTCCTGACTGT	3	<lmxll>	ll	126	lm	145		
>ref-103727	CCCACCACCACCACTACTCCTACCTCC	3	<hkxhk>	hh	0	hk	264	kk	3
>ref-103727	CCCACCACCACCACTACTCCTACCTCC	25	<hkxhk>	hh	0	hk	262	kk	2
>ref-103733	TGATGTCCCACAGTTCCTCCGTTCGGT	5	<nnxnp>	nn	140	np	118		
>ref-103735	CCTCTCTTAACTTTCTCTCCTCCCTTC	8	<hkxhk>	hh	0	hk	265	kk	5
>ref-103735	CCTCTCTTAACTTTCTCTCCTCCCTTC	12	<lmxll>	ll	48	lm	212		
>ref-103735	CCTCTCTTAACTTTCTCTCCTCCCTTC	13	<hkxhk>	hh	0	hk	257	kk	6
>ref-103735	CCTCTCTTAACTTTCTCTCCTCCCTTC	15	<hkxhk>	hh	6	hk	257	kk	0

>ref-103735	CCTCTCTTAACTTTCTCTCCTCCCTTC	25	<hkxhk>	hh	0	hk	270	kk	3
>ref-103743	ACTCCCTCAACCCCTCCTCCCTTCTCC	1	<lmxll>	ll	6	lm	227		
>ref-103743	ACTCCCTCAACCCCTCCTCCCTTCTCC	9	<nnxnp>	nn	251	np	1		
>ref-103743	ACTCCCTCAACCCCTCCTCCCTTCTCC	15	<lmxll>	ll	6	lm	223		
>ref-103743	ACTCCCTCAACCCCTCCTCCCTTCTCC	21	<nnxnp>	nn	263	np	0		
>ref-103763	TCTCCTTCAACTTTTTCTCCTTGTTAT	14	<lmxll>	ll	120	lm	127		
>ref-103763	TCTCCTTCAACTTTTTCTCCTTGTTAT	24	<lmxll>	ll	119	lm	127		
>ref-103769	CTCAACACAACCCACTCTCCCTAGCTC	2	<nnxnp>	nn	141	np	117		
>ref-103769	CTCAACACAACCCACTCTCCCTAGCTC	15	<nnxnp>	nn	140	np	117		
>ref-103770	TCTTAATTTACTTTTGCTCCTCATTTT	9	<lmxll>	ll	121	lm	106		
>ref-103771	AACTCCGAAACGCCGGCTCCCGCGTGC	22	<lmxll>	ll	138	lm	134		
>ref-103801	CTCTCTCTAACTCGACCTCCTATTCGT	6	<hkxhk>	hh	0	hk	270	kk	1
>ref-103801	CTCTCTCTAACTCGACCTCCTATTCGT	7	<hkxhk>	hh	221	hk	38	kk	0
>ref-103801	CTCTCTCTAACTCGACCTCCTATTCGT	15	<lmxll>	ll	190	lm	55		
>ref-103806	CGATCACATACTCTTCCTCCCCTCTCA	21	<lmxll>	ll	111	lm	112		
>ref-103831	GTAACGAAAACAACATCTCCCTTTGTC	5	<nnxnp>	nn	154	np	101		
>ref-103831	GTAACGAAAACAACATCTCCCTTTGTC	26	<nnxnp>	nn	154	np	101		
>ref-103837	CGTCCAACAACAACGGCTCCTCCATCT	1	<lmxll>	ll	250	lm	5		
>ref-103837	CGTCCAACAACAACGGCTCCTCCATCT	2	<lmxll>	ll	261	lm	5		
>ref-103852	GATTGCTGCACAAAATCTCCACCTCAC	7	<nnxnp>	nn	271	np	0		
>ref-103867	ACCATACATACATTAACTCCTCGGACA	1	<lmxll>	ll	133	lm	128		
>ref-103867	ACCATACATACATTAACTCCTCGGACA	14	<lmxll>	ll	133	lm	128		
>ref-103882	ACACACACAACCACCGCTCCCACACGC	21	<lmxll>	ll	143	lm	126		
>ref-103883	TACCATGAGACCTTTGCTCCTCTTGGT	26	<hkxhk>	hh	1	hk	272	kk	0
>ref-103904	TTCAACTACACTGTTCCTCCTTCGTTT	5	<lmxll>	ll	136	lm	131		
>ref-103912	CTAACCCTTACCTTCCCTCCCCTACCT	3	<hkxhk>	hh	2	hk	269	kk	0
>ref-103912	CTAACCCTTACCTTCCCTCCCCTACCT	14	<hkxhk>	hh	0	hk	269	kk	2
>ref-103930	CCCACCTCTACTATCCCTCCCTCTCTC	1	<nnxnp>	nn	248	np	0		
>ref-103976	ACTTCCCTAACACCCCCTCCCCCCCTT	13	<nnxnp>	nn	270	np	0		
>ref-103991	CCCCTCCCCACCATTCCTCCTCCGTCT	4	<lmxll>	ll	129	lm	144		
>ref-103991	CCCCTCCCCACCATTCCTCCTCCGTCT	5	<lmxll>	ll	46	lm	211		
>ref-103991	CCCCTCCCCACCATTCCTCCTCCGTCT	24	<lmxll>	ll	44	lm	212		
>ref-104001	ATTCTGTCAACTAGGACTCCGTACACA	2	<lmxll>	ll	0	lm	256		
>ref-104001	ATTCTGTCAACTAGGACTCCGTACACA	9	<lmxll>	ll	0	lm	256		
>ref-104014	TCTACACCCACTTCTTCTCCCCTTCTC	15	<nnxnp>	nn	130	np	124		
>ref-104014	TCTACACCCACTTCTTCTCCCCTTCTC	24	<nnxnp>	nn	127	np	125		
>ref-104038	AATCAAATTACCCTCTCTCCCTCTCCC	27	<lmxll>	ll	124	lm	125		
>ref-104045	TGATGTTGAACTCCCTCTCCTGCATTG	2	<nnxnp>	nn	132	np	128		

>ref-104059	TCTCCCTACACTCTCTCTCCCTACACT	27	<hkxhk>	hh	0	hk	149	kk	111
>ref-104076	CACACCTCCACATACCCTCCCCCTACT	21	<hkxhk>	hh	3	hk	244	kk	1
>ref-104076	CACACCTCCACATACCCTCCCCCTACT	25	<hkxhk>	hh	4	hk	263	kk	0
>ref-104081	TCTCCCTTCACGGCTACTCCTCTTCCT	13	<hkxhk>	hh	56	hk	132	kk	52
>ref-104123	TCTTATCTTACAGTAACTCCAACAGGT	2	<hkxhk>	hh	0	hk	265	kk	4
>ref-104123	TCTTATCTTACAGTAACTCCAACAGGT	15	<hkxhk>	hh	0	hk	273	kk	0
>ref-104123	TCTTATCTTACAGTAACTCCAACAGGT	16	<hkxhk>	hh	107	hk	151	kk	0
>ref-104123	TCTTATCTTACAGTAACTCCAACAGGT	24	<nnxnp>	nn	160	np	99		
>ref-104130	CCCACTTATACTCCCTCTCCCTTTCAT	4	<hkxhk>	hh	56	hk	213	kk	0
>ref-104130	CCCACTTATACTCCCTCTCCCTTTCAT	12	<nnxnp>	nn	124	np	146		
>ref-104130	CCCACTTATACTCCCTCTCCCTTTCAT	14	<hkxhk>	hh	3	hk	248	kk	17
>ref-104160	CACGTATCCACGTGTCCTCCCCCGCGC	21	<lmxll>	ll	134	lm	122		
>ref-104166	TTATCTACAACTCAGACTCCTATCATA	16	<nnxnp>	nn	97	np	124		
>ref-104199	TGGATGGTGACCCTGGCTCCTTTCGAT	16	<nnxnp>	nn	119	np	143		
>ref-104199	TGGATGGTGACCCTGGCTCCTTTCGAT	21	<nnxnp>	nn	119	np	143		
>ref-104201	TACAATGCCACCCCCTCTCCATATTCA	13	<hkxhk>	hh	0	hk	273	kk	0
>ref-104228	AATCCAGACACTCCAACTCCTTCTCCT	22	<lmxll>	ll	139	lm	84		
>ref-104232	GAAATCCTCACTGTAACTCCCATTGAC	6	<hkxhk>	hh	14	hk	247	kk	0
>ref-104232	GAAATCCTCACTGTAACTCCCATTGAC	14	<lmxll>	ll	217	lm	38		
>ref-104285	TATTATCTTACTCTCCCTCCTCTCTCT	2	<hkxhk>	hh	72	hk	197	kk	0
>ref-104285	TATTATCTTACTCTCCCTCCTCTCTCT	7	<hkxhk>	hh	0	hk	208	kk	50
>ref-104285	TATTATCTTACTCTCCCTCCTCTCTCT	22	<hkxhk>	hh	129	hk	124	kk	0
>ref-104294	TGAACGACCACCATCACTCCGCTTGGG	22	<lmxll>	ll	109	lm	120		
>ref-104304	ACTGTAAGGACTACGCCTCCAGCATTG	4	<lmxll>	ll	138	lm	128		
>ref-104304	ACTGTAAGGACTACGCCTCCAGCATTG	13	<nnxnp>	nn	130	np	135		
>ref-104307	TTCTTTCGCACGCTCCCTCCTTCTCTT	3	<nnxnp>	nn	123	np	143		
>ref-104307	TTCTTTCGCACGCTCCCTCCTTCTCTT	27	<lmxll>	ll	232	lm	3		
>ref-104308	ACCCCATCCACCTACCCTCCACACCTC	2	<nnxnp>	nn	258	np	0		
>ref-104308	ACCCCATCCACCTACCCTCCACACCTC	3	<lmxll>	ll	135	lm	118		
>ref-104308	ACCCCATCCACCTACCCTCCACACCTC	25	<lmxll>	ll	135	lm	117		
>ref-104343	TCCATCATCACTTCATCTCCCTCGAGT	3	<hkxhk>	hh	71	hk	128	kk	37
>ref-104371	CACACCCACACAGTCACTCCGGGGTCA	13	<hkxhk>	hh	0	hk	273	kk	0
>ref-104374	GCAAAGCAGACTCCATCTCCTTGCGCA	1	<hkxhk>	hh	0	hk	208	kk	40
>ref-104374	GCAAAGCAGACTCCATCTCCTTGCGCA	13	<lmxll>	ll	25	lm	239		
>ref-104374	GCAAAGCAGACTCCATCTCCTTGCGCA	24	<lmxll>	ll	24	lm	241		
>ref-104374	GCAAAGCAGACTCCATCTCCTTGCGCA	27	<hkxhk>	hh	39	hk	208	kk	0
>ref-104382	GGGCTTTGCACTTGTCCTCCGCATGCA	4	<lmxll>	ll	131	lm	140		
>ref-104386	CCTACACCAACCCCCTCTCCACCTCCT	2	<lmxll>	ll	120	lm	137		

>ref-104413	TCCCCTCACACCCCATCTCCCTCCATC	27	<nnxnp>	nn	247	np	0		
>ref-104417	CTCCTCTCCACCTCCCCTCCCTTCCTT	1	<nnxnp>	nn	270	np	0		
>ref-104417	CTCCTCTCCACCTCCCCTCCCTTCCTT	4	<nnxnp>	nn	270	np	0		
>ref-104417	CTCCTCTCCACCTCCCCTCCCTTCCTT	5	<hkxhk>	hh	0	hk	259	kk	8
>ref-104417	CTCCTCTCCACCTCCCCTCCCTTCCTT	9	<hkxhk>	hh	0	hk	258	kk	9
>ref-104417	CTCCTCTCCACCTCCCCTCCCTTCCTT	22	<nnxnp>	nn	16	np	249		
>ref-104441	GATTCGTTTACCGCGACTCCTTCGTCA	26	<lmxll>	ll	122	lm	134		
>ref-104470	TGGATTCGCACCTGCGCTCCAATCTCT	2	<hkxhk>	hh	34	hk	163	kk	34
>ref-104470	TGGATTCGCACCTGCGCTCCAATCTCT	16	<nnxnp>	nn	110	np	117		
>ref-104470	TGGATTCGCACCTGCGCTCCAATCTCT	21	<lmxll>	ll	108	lm	116		
>ref-104474	GCGAGCGTGACCAGTACTCCGGGTGGA	15	<nnxnp>	nn	233	np	0		
>ref-104494	CACACAAATACACACACTCCTCATTAC	21	<lmxll>	ll	158	lm	107		
>ref-104494	CACACAAATACACACACTCCTCATTAC	27	<lmxll>	ll	158	lm	107		
>ref-104496	AGAGTACACACCGTTCCTCCTAAATTT	4	<lmxll>	ll	111	lm	135		
>ref-104528	GGGGACACAACAACCACTCCCTCCTCT	15	<hkxhk>	hh	11	hk	256	kk	1
>ref-104528	GGGGACACAACAACCACTCCCTCCTCT	24	<hkxhk>	hh	1	hk	256	kk	12
>ref-104530	CAGAAAGAAACAGAGACTCCGACAGAC	22	<lmxll>	ll	125	lm	131		
>ref-104538	CCCAACGATACCCTTTCTCCTTCCAGA	9	<nnxnp>	nn	9	np	251		
>ref-104555	TCATATACGACGAACACTCCAATGAAA	12	<hkxhk>	hh	2	hk	269	kk	0
>ref-104562	CAGAGACTCACATGCGCTCCATAATCT	16	<hkxhk>	hh	0	hk	271	kk	1
>ref-104565	CTCGGTAAGACAGTTCCTCCGAGTCAG	15	<hkxhk>	hh	36	hk	125	kk	74
>ref-104565	CTCGGTAAGACAGTTCCTCCGAGTCAG	23	<hkxhk>	hh	36	hk	125	kk	73
>ref-104590	CCACATGCAACCCCCTCTCCCCCCAAC	6	<lmxll>	ll	1	lm	266		
>ref-104591	GGTGCTCACACCACCTCTCCTGGACCG	7	<nnxnp>	nn	121	np	140		
>ref-104595	ACCCATAGTACGCTCACTCCTCCATAA	9	<lmxll>	ll	264	lm	0		
>ref-104595	ACCCATAGTACGCTCACTCCTCCATAA	12	<nnxnp>	nn	121	np	139		
>ref-104595	ACCCATAGTACGCTCACTCCTCCATAA	26	<lmxll>	ll	264	lm	0		
>ref-104598	AGCCCACCCACCCCTACTCCAGCTCCA	1	<hkxhk>	hh	33	hk	218	kk	0
>ref-104598	AGCCCACCCACCCCTACTCCAGCTCCA	3	<hkxhk>	hh	2	hk	267	kk	1
>ref-104598	AGCCCACCCACCCCTACTCCAGCTCCA	8	<hkxhk>	hh	0	hk	207	kk	39
>ref-104598	AGCCCACCCACCCCTACTCCAGCTCCA	22	<hkxhk>	hh	1	hk	267	kk	3
>ref-104598	AGCCCACCCACCCCTACTCCAGCTCCA	27	<lmxll>	ll	54	lm	172		
>ref-104607	CACACCCAAACCTCTACTCCACAGAAA	26	<lmxll>	ll	123	lm	132		
>ref-104668	TTTCTTGAGACAAAAGCTCCAGAGAAT	7	<nnxnp>	nn	112	np	135		
>ref-104670	CCATCCCATACCCCCTCTCCACCCCTC	9	<hkxhk>	hh	0	hk	266	kk	3
>ref-104670	CCATCCCATACCCCCTCTCCACCCCTC	15	<hkxhk>	hh	0	hk	266	kk	3
>ref-104671	TGTTCACTTACCCTCACTCCATAAGAC	3	<lmxll>	ll	112	lm	129		
>ref-104681	TGTCCATCAACCAAGTCTCCTTAGTTG	12	<lmxll>	ll	122	lm	145		

>ref-104701	TCTTCGCCCACCACTTCTCCTCCCGTT	2	<lmxll>	ll	220	lm	6		
>ref-104727	CACAGCTACACCTTGACTCCACTTTAA	23	<nnxnp>	nn	143	np	121		
>ref-104765	CTTTTCTCCACCTTTTCTCCACCTTTT	6	<hkxhk>	hh	1	hk	266	kk	2
>ref-104765	CTTTTCTCCACCTTTTCTCCACCTTTT	25	<hkxhk>	hh	2	hk	266	kk	1
>ref-104767	GTGTGTTGAACTCATTCTCCAAATCGT	26	<lmxll>	ll	133	lm	118		
>ref-104769	CATTACGTCACAGCGCCTCCTTTCTGT	21	<nnxnp>	nn	135	np	137		
>ref-104784	CTCCCCCCAACATCGCCTCCTCCTCCT	26	<nnxnp>	nn	122	np	140		
>ref-104805	AGAACGGTCACAATTTCTCCTTTTACA	24	<lmxll>	ll	103	lm	131		
>ref-104811	GCTGGAGATACAGATGCTCCATATAGA	8	<lmxll>	ll	124	lm	141		
>ref-104812	TGCCTTCCCACTTTCTCTCCTTCGTGA	22	<nnxnp>	nn	121	np	138		
>ref-104834	GAAAGGTCAACATACTCTCCACGCTCT	13	<nnxnp>	nn	124	np	142		
>ref-104853	CGCTTCACCACTCCCCCTCCCCTCTTT	1	<lmxll>	ll	266	lm	1		
>ref-104856	AGAGCGATGACCTTTCCTCCTCATCCG	7	<nnxnp>	nn	117	np	141		
>ref-104889	ATATGAAACACTGATTCTCCGGCACTG	23	<nnxnp>	nn	126	np	105		
>ref-104890	TTTTGTTGTACTTGTGCTCCTTGTGCT	3	<lmxll>	ll	119	lm	146		
>ref-104900	TTTCTCCCTACTCTTCCTCCTTTTCTC	7	<lmxll>	ll	107	lm	131		
>ref-104900	TTTCTCCCTACTCTTCCTCCTTTTCTC	8	<lmxll>	ll	105	lm	131		
>ref-104907	AATATTGCAACACTAGCTCCTAGTCTA	22	<lmxll>	ll	134	lm	132		
>ref-104909	ACTTTCTTTACTTAGTCTCCGTAGTCT	7	<lmxll>	ll	109	lm	115		
>ref-104909	ACTTTCTTTACTTAGTCTCCGTAGTCT	12	<lmxll>	ll	109	lm	115		
>ref-104913	TTCTGTGTCACAGTAGCTCCAGACTAA	12	<lmxll>	ll	110	lm	137		
>ref-104932	GGCACTCCGACCTTGTCTCCCCCCAAA	2	<lmxll>	ll	143	lm	109		
>ref-104932	GGCACTCCGACCTTGTCTCCCCCCAAA	24	<hkxhk>	hh	6	hk	253	kk	3
>ref-104933	TCCTCTGACACTGTTTCTCCATAGATC	2	<nnxnp>	nn	125	np	141		
>ref-104976	ACTGGTCAGACTGCACCTCCCTCATGG	27	<nnxnp>	nn	224	np	0		
>ref-104987	AACTATACTACTCTCTCTCCCTCCCTC	5	<lmxll>	ll	128	lm	126		
>ref-104987	AACTATACTACTCTCTCTCCCTCCCTC	6	<lmxll>	ll	131	lm	125		
>ref-105038	GCCTGTACAACCCGTCCTCCAGTCCCT	12	<nnxnp>	nn	135	np	129		
>ref-105038	GCCTGTACAACCCGTCCTCCAGTCCCT	15	<nnxnp>	nn	136	np	129		
>ref-105040	TCTCGGCGCACTTCATCTCCACGCCTT	5	<nnxnp>	nn	129	np	129		
>ref-105061	ACCGCCATCACTCAAACTCCTCATTCA	16	<lmxll>	ll	138	lm	122		
>ref-105078	CCACAGCAAACAGACTCTCCCAGCTTA	4	<nnxnp>	nn	128	np	139		
>ref-105112	TCTCACCCCACCCCTTCTCCTCCCTTT	3	<hkxhk>	hh	0	hk	170	kk	79
>ref-105112	TCTCACCCCACCCCTTCTCCTCCCTTT	16	<hkxhk>	hh	33	hk	205	kk	6
>ref-105112	TCTCACCCCACCCCTTCTCCTCCCTTT	22	<hkxhk>	hh	79	hk	170	kk	0
>ref-105160	TTATCTGTTACCTTCCCTCCACCCCTT	22	<nnxnp>	nn	130	np	117		
>ref-105174	TTAAAATCCACCTCCTCTCCATCTCTC	5	<lmxll>	ll	130	lm	115		
>ref-105174	TTAAAATCCACCTCCTCTCCATCTCTC	27	<hkxhk>	hh	0	hk	273	kk	0

>ref-105189	TCTTCCTCCACCCTCACTCCTCCATTT	5	<nnxnp>	nn	258	np	0		
>ref-105190	TGAATTTGAACTGTGTCTCCCTCGCTG	27	<nnxnp>	nn	128	np	135		
>ref-105227	GCCTCCTCCACCGTTCCTCCATCTCCA	21	<hkxhk>	hh	68	hk	139	kk	43
>ref-105231	AAGCAGCCCACACAGACTCCTCATGTG	21	<lmxll>	ll	138	lm	130		
>ref-105240	GCAGAGAACACGGCCGCTCCCTCTTTT	1	<nnxnp>	nn	221	np	8		
>ref-105253	GGGGAACCCACAGCAGCTCCATCGATC	3	<hkxhk>	hh	0	hk	273	kk	0
>ref-105253	GGGGAACCCACAGCAGCTCCATCGATC	16	<hkxhk>	hh	0	hk	273	kk	0
>ref-105276	AACCCTTCAACGCCTCCTCCCCTTCCT	23	<lmxll>	ll	143	lm	124		
>ref-105319	TCCTCCCTAACCTCTCCTCCCCCATCC	16	<nnxnp>	nn	125	np	141		
>ref-105343	ACCATTTTAACCTTCCCTCCTGTATTT	16	<hkxhk>	hh	2	hk	239	kk	7
>ref-105343	ACCATTTTAACCTTCCCTCCTGTATTT	22	<lmxll>	ll	67	lm	154		
>ref-105344	CCCCCTCCCACACTCTCTCCCCCACCA	4	<nnxnp>	nn	265	np	0		
>ref-105352	ACAAAGATAACAGTGACTCCCTGTGTA	3	<lmxll>	ll	115	lm	128		
>ref-105352	ACAAAGATAACAGTGACTCCCTGTGTA	6	<lmxll>	ll	135	lm	102		
>ref-105357	TCCGTTCACACTATCTCTCCTCTTCCT	22	<lmxll>	ll	143	lm	95		
>ref-105358	CGCCCTCGCACACGCTCTCCTCAGTCG	4	<nnxnp>	nn	139	np	131		
>ref-105370	AGTCCCGCGACCACAGCTCCGGCCTCT	27	<lmxll>	ll	118	lm	139		
>ref-105388	CAACACAGTACTGGCCCTCCGGGTATG	15	<hkxhk>	hh	82	hk	166	kk	0
>ref-105406	CTGTCACTAACCCGACCTCCTCGACCT	9	<lmxll>	ll	101	lm	139		
>ref-105414	TATCTGTTGACAATATCTCCGTTTCTT	26	<nnxnp>	nn	259	np	1		
>ref-105418	GTACAAAGTACGTCCTCTCCATCATTC	25	<lmxll>	ll	125	lm	140		
>ref-105420	AAACGACCCACGGACACTCCCTAGTCA	14	<lmxll>	ll	121	lm	144		
>ref-105438	GAGAGACATACTAAACCTCCCTTCGTG	7	<lmxll>	ll	126	lm	144		
>ref-105465	TCCCCCTTAACCCCTTCTCCTCATCCT	2	<nnxnp>	nn	266	np	0		
>ref-105465	TCCCCCTTAACCCCTTCTCCTCATCCT	3	<nnxnp>	nn	146	np	111		
>ref-105465	TCCCCCTTAACCCCTTCTCCTCATCCT	16	<nnxnp>	nn	264	np	1		
>ref-105465	TCCCCCTTAACCCCTTCTCCTCATCCT	23	<nnxnp>	nn	139	np	118		
>ref-105487	TTTAATAACACCATAGCTCCTTGTGTA	23	<lmxll>	ll	103	lm	138		
>ref-105503	CGCCCTCCAACCTGTCCTCCCTCCCTC	2	<hkxhk>	hh	9	hk	252	kk	0
>ref-105503	CGCCCTCCAACCTGTCCTCCCTCCCTC	14	<nnxnp>	nn	244	np	14		
>ref-105517	AATTCAACGACACTCTCTCCCTGGTGT	8	<lmxll>	ll	127	lm	137		
>ref-105517	AATTCAACGACACTCTCTCCCTGGTGT	23	<nnxnp>	nn	133	np	128		
>ref-105539	GTTATTTGCACGAGTCCTCCGGGAGCG	12	<nnxnp>	nn	150	np	92		
>ref-105539	GTTATTTGCACGAGTCCTCCGGGAGCG	21	<lmxll>	ll	1	lm	248		
>ref-105548	GAAGACCCCACTCTCTCTCCCTCCCTC	26	<nnxnp>	nn	123	np	117		
>ref-105550	GTCATTCTCACTCTCTCTCCCCCATCT	1	<nnxnp>	nn	214	np	10		
>ref-105550	GTCATTCTCACTCTCTCTCCCCCATCT	4	<lmxll>	ll	226	lm	8		
>ref-105550	GTCATTCTCACTCTCTCTCCCCCATCT	24	<nnxnp>	nn	224	np	3		

>ref-105550	GTCATTCTCACTCTCTCTCCCCCATCT	26	<hkxhk>	hh	237	hk	1	kk	0
>ref-105550	GTCATTCTCACTCTCTCTCCCCCATCT	27	<nnxnp>	nn	239	np	0		
>ref-105595	AGAAAGAAGACAAGAACTCCAAACATC	2	<nnxnp>	nn	223	np	43		
>ref-105614	TTACCAGTCACATTCCCTCCGCCAGCG	4	<nnxnp>	nn	142	np	120		
>ref-105614	TTACCAGTCACATTCCCTCCGCCAGCG	26	<nnxnp>	nn	141	np	121		
>ref-105637	CACTCCTCCACCTACTCTCCACCCCTC	5	<hkxhk>	hh	14	hk	248	kk	0
>ref-105637	CACTCCTCCACCTACTCTCCACCCCTC	8	<hkxhk>	hh	28	hk	230	kk	0
>ref-105637	CACTCCTCCACCTACTCTCCACCCCTC	9	<hkxhk>	hh	2	hk	269	kk	0
>ref-105655	TCCGAAGGGACGCCCACTCCCTTGTCC	13	<hkxhk>	hh	42	hk	148	kk	65
>ref-105673	CCCTAGCCCACTCTCCCTCCATCCCTT	23	<lmxll>	ll	128	lm	128		
>ref-105674	ATGAACAACACGAGGTCTCCTTCGCCC	21	<lmxll>	ll	264	lm	0		
>ref-105683	CGGCCTTCTACCCTTCCTCCTCATTCC	3	<lmxll>	ll	121	lm	140		
>ref-105683	CGGCCTTCTACCCTTCCTCCTCATTCC	24	<lmxll>	ll	121	lm	140		
>ref-105688	TCTCTCCCTACCTCCTCTCCCTTCATC	6	<nnxnp>	nn	35	np	199		
>ref-105688	TCTCTCCCTACCTCCTCTCCCTTCATC	15	<nnxnp>	nn	35	np	196		
>ref-105689	TAACCATACACAAGACCTCCGTTGAAA	26	<nnxnp>	nn	126	np	136		
>ref-105691	TGTCCTTGCACCAACACTCCAACACCT	22	<nnxnp>	nn	31	np	239		
>ref-105691	TGTCCTTGCACCAACACTCCAACACCT	26	<hkxhk>	hh	0	hk	273	kk	0
>ref-105722	GTAACCTCCACCCATCCTCCATCCTCC	25	<nnxnp>	nn	130	np	139		
>ref-105722	GTAACCTCCACCCATCCTCCATCCTCC	26	<nnxnp>	nn	129	np	139		
>ref-105744	CTTCATATCACTCTCCCTCCCACCTTG	6	<nnxnp>	nn	102	np	141		
>ref-105752	GTCGCCTTTACAAGGACTCCTTCCAAT	4	<lmxll>	ll	116	lm	129		
>ref-105795	CCCGCAAAGACACGTCCTCCGTAGATC	16	<lmxll>	ll	110	lm	147		
>ref-105833	TTCTCCCCCACTCCCTCTCCCTGTATT	23	<lmxll>	ll	259	lm	2		
>ref-105833	TTCTCCCCCACTCCCTCTCCCTGTATT	27	<lmxll>	ll	260	lm	2		
>ref-105843	TCTTCCTTAACCCCCCCTCCTCCCCCC	4	<lmxll>	ll	98	lm	150		
>ref-105843	TCTTCCTTAACCCCCCCTCCTCCCCCC	26	<hkxhk>	hh	103	hk	138	kk	0
>ref-105845	TCTTTATTTACTCTCTCTCCCCTTTTT	1	<nnxnp>	nn	40	np	179		
>ref-105870	CGTTCTGCCACTGGAACTCCCCAACCT	2	<hkxhk>	hh	163	hk	78	kk	0
>ref-105870	CGTTCTGCCACTGGAACTCCCCAACCT	3	<hkxhk>	hh	0	hk	262	kk	4
>ref-105870	CGTTCTGCCACTGGAACTCCCCAACCT	24	<hkxhk>	hh	3	hk	270	kk	0
>ref-105890	GTTAGTCCTACTCCTCCTCCGCCTCCT	5	<nnxnp>	nn	260	np	2		
>ref-105890	GTTAGTCCTACTCCTCCTCCGCCTCCT	21	<nnxnp>	nn	207	np	34		
>ref-105895	TTCTTCATCACTCGTTCTCCTTGCAGC	4	<hkxhk>	hh	0	hk	208	kk	60
>ref-105895	TTCTTCATCACTCGTTCTCCTTGCAGC	26	<lmxll>	ll	198	lm	63		
>ref-105895	TTCTTCATCACTCGTTCTCCTTGCAGC	27	<hkxhk>	hh	60	hk	208	kk	0
>ref-105937	ATATATAGGACGTCTCCTCCTCCGGCC	1	<nnxnp>	nn	32	np	221		
>ref-105937	ATATATAGGACGTCTCCTCCTCCGGCC	3	<lmxll>	ll	230	lm	21		

>ref-105937	ATATATAGGACGTCTCCTCCTCCGGCC	4	<lmxll>	ll	11	lm	230		
>ref-105950	CGTTCTGTGACTCCTACTCCTTGTGTC	9	<nnxnp>	nn	263	np	0		
>ref-105956	AATTTCTCCACTCTCTCTCCTTCTCCC	12	<nnxnp>	nn	49	np	218		
>ref-105965	TTGTGGCTTACAGTGTCTCCTGCGAGA	1	<hkxhk>	hh	57	hk	207	kk	0
>ref-105985	ACACTCGCTACTCACTCTCCAGGCCAG	7	<hkxhk>	hh	44	hk	161	kk	57
>ref-105985	ACACTCGCTACTCACTCTCCAGGCCAG	8	<hkxhk>	hh	57	hk	161	kk	44
>ref-106018	TTACATAACACAGGAACTCCACGACTC	23	<lmxll>	ll	104	lm	136		
>ref-106048	CTCTTCCTAACCCCTCCTCCTCCTGCC	7	<nnxnp>	nn	117	np	111		
>ref-106048	CTCTTCCTAACCCCTCCTCCTCCTGCC	14	<nnxnp>	nn	118	np	111		
>ref-106049	TTTGGATTCACTTTCACTCCTAGCACA	4	<nnxnp>	nn	50	np	187		
>ref-106049	TTTGGATTCACTTTCACTCCTAGCACA	16	<lmxll>	ll	134	lm	110		
>ref-106052	AATAGCACAACTCTCCCTCCGCTCTTT	16	<lmxll>	ll	118	lm	149		
>ref-106062	CACATTAAGACCTGAACTCCTCATCTG	27	<nnxnp>	nn	119	np	140		
>ref-106077	TCTTCCCTCACCCCCCCTCCTTCTTAC	1	<lmxll>	ll	242	lm	0		
>ref-106077	TCTTCCCTCACCCCCCCTCCTTCTTAC	14	<lmxll>	ll	110	lm	115		
>ref-106077	TCTTCCCTCACCCCCCCTCCTTCTTAC	23	<nnxnp>	nn	246	np	0		
>ref-106077	TCTTCCCTCACCCCCCCTCCTTCTTAC	24	<nnxnp>	nn	240	np	0		
>ref-106077	TCTTCCCTCACCCCCCCTCCTTCTTAC	25	<lmxll>	ll	111	lm	115		
>ref-106094	GTGCGTATGACTGTCCCTCCTTGTCAT	16	<lmxll>	ll	148	lm	122		
>ref-106100	TCCTCGTCCACTTACCCTCCTCCCCCT	1	<nnxnp>	nn	1	np	250		
>ref-106100	TCCTCGTCCACTTACCCTCCTCCCCCT	2	<lmxll>	ll	264	lm	0		
>ref-106100	TCCTCGTCCACTTACCCTCCTCCCCCT	14	<nnxnp>	nn	1	np	249		
>ref-106101	GACACCACCACCGCCTCTCCCTCCACG	2	<hkxhk>	hh	36	hk	134	kk	52
>ref-106114	ACGACACCAACACTCTCTCCTCTTCTC	13	<hkxhk>	hh	56	hk	117	kk	60
>ref-106118	AAGCAGCAGACGCATTCTCCACGAGAC	2	<hkxhk>	hh	0	hk	257	kk	11
>ref-106119	GAAAGACACACCCTTTCTCCCTGCCAT	25	<nnxnp>	nn	120	np	121		
>ref-106125	ATGATGCCTACGTGCCCTCCGCTCAAA	21	<nnxnp>	nn	137	np	133		
>ref-106134	CCCTTATTTACCTACCCTCCTTCCCTT	13	<nnxnp>	nn	142	np	100		
>ref-106149	TAGTATGATACTCTGACTCCGTATGTT	25	<nnxnp>	nn	119	np	129		
>ref-106160	TCTCATATCACCGCATCTCCCTTTCTC	15	<lmxll>	ll	130	lm	130		
>ref-106174	CGACTCAGCACACTGTCTCCTCCCTCT	22	<hkxhk>	hh	70	hk	200	kk	0
>ref-106212	AGCCAATACACATTTCCTCCCACCGCC	1	<hkxhk>	hh	17	hk	190	kk	43
>ref-106212	AGCCAATACACATTTCCTCCCACCGCC	22	<hkxhk>	hh	49	hk	212	kk	0
>ref-106243	TCTTGTCCTACCCCCCCTCCTGCCACC	6	<lmxll>	ll	119	lm	145		
>ref-106245	AGTGCCTATACCTGGCCTCCCATTATT	8	<lmxll>	ll	135	lm	136		
>ref-106247	ACAAATGCTACACGGACTCCTTTGATG	1	<nnxnp>	nn	125	np	139		
>ref-106252	CCTTCACTTACCCTTCCTCCATTCCTC	13	<hkxhk>	hh	48	hk	151	kk	48
>ref-106263	TTCTGGGTCACTTGCCCTCCTCTTATA	23	<nnxnp>	nn	115	np	134		

>ref-106272	GGGACGAAGACGTGTTCTCCTTTGCCT	5	<hkxhk>	hh	1	hk	269	kk	0
>ref-106272	GGGACGAAGACGTGTTCTCCTTTGCCT	15	<hkxhk>	hh	0	hk	269	kk	1
>ref-106274	GAAGGCAATACTAGACCTCCACTACCA	1	<hkxhk>	hh	0	hk	100	kk	159
>ref-106274	GAAGGCAATACTAGACCTCCACTACCA	3	<hkxhk>	hh	0	hk	267	kk	1
>ref-106274	GAAGGCAATACTAGACCTCCACTACCA	16	<lmxll>	ll	234	lm	26		
>ref-106274	GAAGGCAATACTAGACCTCCACTACCA	22	<hkxhk>	hh	106	hk	155	kk	0
>ref-106277	TTGCCGTCTACAACTCCTCCTCGCCTT	9	<lmxll>	ll	142	lm	128		
>ref-106294	CGAAGAACTACCCTCGCTCCTCACATT	6	<nnxnp>	nn	259	np	0		
>ref-106295	CCTCCTCGAACTGGGCCTCCCCCTCGT	9	<nnxnp>	nn	264	np	0		
>ref-106299	CCTGGTCTGACCTACTCTCCCATTTAC	14	<lmxll>	ll	127	lm	110		
>ref-106317	ACTTCCACTACTCTTACTCCGACTGAT	2	<hkxhk>	hh	63	hk	122	kk	53
>ref-106320	CCTTTGTCTACATAATCTCCTTTACTT	14	<lmxll>	ll	250	lm	0		
>ref-106346	AAGCACTCGACGCTGTCTCCACCTAAT	12	<lmxll>	ll	133	lm	140		
>ref-106351	TGTTCCTCAACAACATCTCCGGCGTTA	9	<lmxll>	ll	121	lm	146		
>ref-106353	TTGCTTCAGACTATCCCTCCTTTTCTC	13	<lmxll>	ll	10	lm	252		
>ref-106353	TTGCTTCAGACTATCCCTCCTTTTCTC	21	<lmxll>	ll	10	lm	252		
>ref-106353	TTGCTTCAGACTATCCCTCCTTTTCTC	24	<lmxll>	ll	146	lm	122		
>ref-106354	GTCACAGCTACACACTCTCCATCATAA	13	<lmxll>	ll	103	lm	166		
>ref-106359	CAAGGCTCCACATAAGCTCCATGTGGA	27	<hkxhk>	hh	60	hk	143	kk	44
>ref-106369	AAAACGACCACTTGAGCTCCTTTGCAA	2	<hkxhk>	hh	35	hk	131	kk	76
>ref-106375	AATGTTACTACTAATTCTCCTCTTCCT	5	<hkxhk>	hh	64	hk	133	kk	42
>ref-106386	CCTTTTGTTACTTCCTCTCCTCTCTTT	7	<lmxll>	ll	200	lm	54		
>ref-106386	CCTTTTGTTACTTCCTCTCCTCTCTTT	22	<lmxll>	ll	190	lm	59		
>ref-106412	CATACACACACCACACCTCCTCCTCTC	16	<nnxnp>	nn	130	np	124		
>ref-106427	TACAGCAAAACTCTCTCTCCCCTTTCA	27	<nnxnp>	nn	86	np	135		
>ref-106461	CTCGTCTTCACTCTATCTCCTTCCTCC	15	<lmxll>	ll	126	lm	119		
>ref-106473	AGGTCAGAAACGTCATCTCCTCTAACT	3	<lmxll>	ll	126	lm	137		
>ref-106549	GATGATAGAACCTCCTCTCCACCCGCT	13	<nnxnp>	nn	137	np	131		
>ref-106555	CATCTCGTCACACCCTCTCCCCCCTTC	1	<nnxnp>	nn	172	np	90		
>ref-106555	CATCTCGTCACACCCTCTCCCCCCTTC	7	<hkxhk>	hh	0	hk	269	kk	0
>ref-106558	CCACGGCGGACACCCTCTCCGGGCCCT	26	<nnxnp>	nn	116	np	127		
>ref-106560	TCTTCTCCAACTCTCCCTCCTTCATCT	2	<nnxnp>	nn	175	np	87		
>ref-106560	TCTTCTCCAACTCTCCCTCCTTCATCT	21	<hkxhk>	hh	0	hk	271	kk	2
>ref-106560	TCTTCTCCAACTCTCCCTCCTTCATCT	24	<hkxhk>	hh	2	hk	271	kk	0
>ref-106575	AAATGTAACACACACACTCCCACACAC	24	<lmxll>	ll	115	lm	142		
>ref-106585	ACCACAACTACAACGACTCCTCGACCC	5	<nnxnp>	nn	115	np	151		
>ref-106597	CTCATCCCTACACTCCCTCCATCCCCT	21	<nnxnp>	nn	104	np	130		
>ref-106650	CTGCTGTTGACTCGAACTCCCGAGCGT	4	<lmxll>	ll	50	lm	187		

>ref-106650	CTGCTGTTGACTCGAACTCCCGAGCGT	9	<nnxnp>	nn	139	np	105		
>ref-106652	AACGATGAAACACAGACTCCTCTGTGA	14	<lmxll>	ll	112	lm	151		
>ref-106654	CTATCCCTTACACTCCCTCCTGGATAC	2	<lmxll>	ll	63	lm	199		
>ref-106654	CTATCCCTTACACTCCCTCCTGGATAC	13	<lmxll>	ll	62	lm	200		
>ref-106655	CCACCTTATACTCCCTCTCCACCCACT	1	<nnxnp>	nn	137	np	118		
>ref-106657	GCCCGTCTAACTTCGCCTCCGTCCGTT	24	<nnxnp>	nn	134	np	129		
>ref-106657	GCCCGTCTAACTTCGCCTCCGTCCGTT	27	<nnxnp>	nn	133	np	130		
>ref-106668	CTCTCAACAACACAAACTCCCAAACAT	4	<hkxhk>	hh	0	hk	251	kk	14
>ref-106668	CTCTCAACAACACAAACTCCCAAACAT	7	<lmxll>	ll	157	lm	89		
>ref-106668	CTCTCAACAACACAAACTCCCAAACAT	27	<lmxll>	ll	157	lm	88		
>ref-106674	AACGTCACAACTGTTGCTCCAAGATGA	22	<lmxll>	ll	266	lm	0		
>ref-106676	AACGTTATTACTGCCACTCCATTTTCA	15	<nnxnp>	nn	98	np	130		
>ref-106678	CACAGGAGGACGGTCTCTCCCCCCCAC	22	<lmxll>	ll	141	lm	129		
>ref-106679	ATCCGCAGTACTATTCCTCCATTTCTG	8	<nnxnp>	nn	119	np	138		
>ref-106679	ATCCGCAGTACTATTCCTCCATTTCTG	14	<nnxnp>	nn	108	np	143		
>ref-106712	CACCACCCCACCCCCTCTCCTCTCCCC	5	<lmxll>	ll	63	lm	174		
>ref-106715	TTCCGCCCTACCCAGCCTCCCGTTCCT	6	<lmxll>	ll	131	lm	136		
>ref-106730	CCCGCCGACACCCCTCCTCCCCTCCTT	5	<lmxll>	ll	117	lm	119		
>ref-106766	CCAGCACATACACACACTCCGTCCTTT	22	<nnxnp>	nn	132	np	128		
>ref-106771	CATCCCTTTACCACCTCTCCCTTGTCT	12	<lmxll>	ll	125	lm	135		
>ref-106771	CATCCCTTTACCACCTCTCCCTTGTCT	23	<lmxll>	ll	126	lm	134		
>ref-106797	TCTATTTCGACGGCACCTCCTATTGGC	6	<hkxhk>	hh	5	hk	234	kk	2
>ref-106803	ATTTATTTAACTTCCTCTCCCCCGCCT	6	<nnxnp>	nn	125	np	124		
>ref-106805	TTTTGATTGACACCTCCTCCCACTATC	13	<hkxhk>	hh	37	hk	105	kk	83
>ref-106821	CTGACTCTTACAAGCACTCCTTCTTCC	2	<hkxhk>	hh	0	hk	261	kk	3
>ref-106821	CTGACTCTTACAAGCACTCCTTCTTCC	3	<hkxhk>	hh	0	hk	254	kk	8
>ref-106824	TAAAACCCTACGTCTGCTCCCTCTCAA	6	<nnxnp>	nn	116	np	104		
>ref-106826	CATACATACACGCCATCTCCCATCTAT	3	<lmxll>	ll	113	lm	121		
>ref-106839	TCCCTCTCTACTCCTCCTCCATCTCTG	14	<nnxnp>	nn	112	np	140		
>ref-106842	TTTAGAAAAACCGTGCCTCCTTGCTTG	12	<lmxll>	ll	102	lm	138		
>ref-106870	GGTCAGAACACTTTACCTCCACTGACT	5	<lmxll>	ll	206	lm	58		
>ref-106870	GGTCAGAACACTTTACCTCCACTGACT	27	<hkxhk>	hh	0	hk	273	kk	0
>ref-106871	TACCTCATTACCCCCTCTCCTTCGCCC	14	<lmxll>	ll	148	lm	118		
>ref-106912	ACTCCCTCTACCCCCCCTCCCCTACCC	7	<hkxhk>	hh	0	hk	216	kk	35
>ref-106912	ACTCCCTCTACCCCCCCTCCCCTACCC	8	<hkxhk>	hh	35	hk	218	kk	0
>ref-106912	ACTCCCTCTACCCCCCCTCCCCTACCC	23	<hkxhk>	hh	0	hk	236	kk	22
>ref-106912	ACTCCCTCTACCCCCCCTCCCCTACCC	27	<hkxhk>	hh	21	hk	236	kk	0
>ref-106929	TCATCTCCCACTCATCCTCCGGCCTTT	13	<lmxll>	ll	135	lm	137		

>ref-106936	CTCACCCGCACTCTTCCTCCTCTCTCA	4	<nnxnp>	nn	111	np	156		
>ref-106938	TCCCACACAACGCTGTCTCCCTAACAA	12	<lmxll>	ll	125	lm	127		
>ref-106975	CACACACCGACCCCTCCTCCTCCCCTC	15	<hkxhk>	hh	0	hk	263	kk	6
>ref-106975	CACACACCGACCCCTCCTCCTCCCCTC	23	<hkxhk>	hh	6	hk	263	kk	0
>ref-106981	TCCACTTGTACTTCCTCTCCCACTCCT	12	<lmxll>	ll	124	lm	131		
>ref-106990	ACCTCTTCCACAACCTCTCCACCTCAT	6	<hkxhk>	hh	0	hk	273	kk	0
>ref-106990	ACCTCTTCCACAACCTCTCCACCTCAT	12	<lmxll>	ll	51	lm	207		
>ref-106990	ACCTCTTCCACAACCTCTCCACCTCAT	23	<lmxll>	ll	210	lm	44		
>ref-106990	ACCTCTTCCACAACCTCTCCACCTCAT	26	<lmxll>	ll	52	lm	210		
>ref-106995	TGGCATTACACACTGCCTCCTTCAATT	16	<nnxnp>	nn	124	np	132		
>ref-107013	TTCCTATTTACTTTCCCTCCTGTCGCT	3	<hkxhk>	hh	4	hk	259	kk	0
>ref-107030	CTTCCTTTCACCCTTTCTCCTGCCTCA	3	<hkxhk>	hh	3	hk	141	kk	98
>ref-107030	CTTCCTTTCACCCTTTCTCCTGCCTCA	16	<nnxnp>	nn	118	np	135		
>ref-107030	CTTCCTTTCACCCTTTCTCCTGCCTCA	21	<nnxnp>	nn	6	np	247		
>ref-107030	CTTCCTTTCACCCTTTCTCCTGCCTCA	22	<nnxnp>	nn	6	np	246		
>ref-107039	CCCTGACTCACCCACCCTCCTGACTCA	3	<lmxll>	ll	149	lm	115		
>ref-107039	CCCTGACTCACCCACCCTCCTGACTCA	8	<nnxnp>	nn	32	np	223		
>ref-107039	CCCTGACTCACCCACCCTCCTGACTCA	12	<nnxnp>	nn	32	np	224		
>ref-107042	ACCAGCCCTACCTCTCCTCCTTTCCAT	15	<nnxnp>	nn	118	np	144		
>ref-107059	GCCCAACATACCCATTCTCCTTCATCA	7	<lmxll>	ll	133	lm	123		
>ref-107069	CGTTAGGCCACACGGTCTCCGTGTAAG	13	<hkxhk>	hh	0	hk	272	kk	0
>ref-107069	CGTTAGGCCACACGGTCTCCGTGTAAG	23	<hkxhk>	hh	0	hk	266	kk	4
>ref-107070	CGCTATGACACTAACTCTCCGGAATAA	3	<nnxnp>	nn	139	np	83		
>ref-107085	CTATGTCTCACCCTCCCTCCCTCTCCC	5	<hkxhk>	hh	27	hk	243	kk	0
>ref-107093	AAACCCTCCACCAATGCTCCCTCTCGC	4	<lmxll>	ll	128	lm	136		
>ref-107101	AAGGAATAAACAAAAACTCCATTAACA	9	<hkxhk>	hh	1	hk	240	kk	9
>ref-107116	ACACACAGCACACCCACTCCCTCCCTC	27	<nnxnp>	nn	129	np	120		
>ref-107128	CGAGTGGGGACGTCATCTCCTTAGAGA	8	<hkxhk>	hh	63	hk	125	kk	40
>ref-107128	CGAGTGGGGACGTCATCTCCTTAGAGA	24	<nnxnp>	nn	251	np	0		
>ref-107140	TACCCGGCCACAGTGGCTCCCCCCCTT	7	<nnxnp>	nn	263	np	0		
>ref-107173	CCCATCTCTACCTCTTCTCCTCGACAG	7	<nnxnp>	nn	111	np	117		
>ref-107197	AACTTCAGAACATCGACTCCATTAGTC	8	<nnxnp>	nn	1	np	238		
>ref-107234	CCTCCCTCCACCCACTCTCCAATCTCC	5	<nnxnp>	nn	114	np	145		
>ref-107249	ATACTAACTACCCCCCCTCCCTCCAAC	13	<nnxnp>	nn	102	np	129		
>ref-107275	CGTGCATGTACCTCGGCTCCTGCCTCT	5	<lmxll>	ll	135	lm	138		
>ref-107280	ATACTGTAGACAGCGACTCCTAGGCTC	21	<hkxhk>	hh	56	hk	147	kk	59
>ref-107287	TTCTTTAAGACCTTTCCTCCTCTTTTA	1	<nnxnp>	nn	118	np	113		
>ref-107301	CAGGCGATGACTTGGGCTCCGTTTGCG	1	<nnxnp>	nn	267	np	0		

>ref-107302	TCCCACTCTACGTTCTCTCCCCCACTT	12	<hkxhk>	hh	54	hk	74	kk	112
>ref-107339	TCTACCCTCACCCTTTCTCCCTGACCA	1	<lmxll>	ll	133	lm	126		
>ref-107393	TCTCATCTCACTGTTTCTCCCTCATCT	1	<lmxll>	ll	165	lm	87		
>ref-107409	AACTTTAACACTTTAACTCCAGTGAAC	7	<lmxll>	ll	113	lm	115		
>ref-107457	TCTCTGTTCACCGATTCTCCTTCCTCT	27	<lmxll>	ll	2	lm	262		
>ref-107469	AAGGCTTCGACAATGCCTCCTAACTGG	3	<lmxll>	ll	148	lm	109		
>ref-107476	AACCCTTTGACAAAACCTCCACTTACT	9	<nnxnp>	nn	268	np	0		
>ref-107477	TCCTACCCCACACGCACTCCTAACACA	14	<hkxhk>	hh	49	hk	126	kk	60
>ref-107490	TCTCTCTCTACATCACCTCCCTCCATA	25	<nnxnp>	nn	128	np	133		
>ref-107506	TGATAAGACACTATGCCTCCTATTGGT	16	<hkxhk>	hh	47	hk	151	kk	47
>ref-107511	TCTCATTCCACCTTTTCTCCGTGTTTG	26	<lmxll>	ll	110	lm	129		
>ref-107519	CCCTCTTTCACTCCTCCTCCTTCATTA	2	<lmxll>	ll	113	lm	156		
>ref-107602	TGCTGTGCCACTAATACTCCTTCGGGC	16	<nnxnp>	nn	124	np	143		
>ref-107633	ATTCACAAAACATTAACTCCGGCGTAA	4	<nnxnp>	nn	113	np	122		
>ref-107633	ATTCACAAAACATTAACTCCGGCGTAA	22	<nnxnp>	nn	110	np	123		
>ref-107639	CTCTCCTACACTAACCCTCCTTTCCCC	1	<nnxnp>	nn	263	np	0		
>ref-107639	CTCTCCTACACTAACCCTCCTTTCCCC	13	<nnxnp>	nn	133	np	124		
>ref-107650	ATGTCTCCAACCTTCCCTCCCTCTATC	2	<hkxhk>	hh	0	hk	267	kk	5
>ref-107650	ATGTCTCCAACCTTCCCTCCCTCTATC	3	<hkxhk>	hh	0	hk	267	kk	5
>ref-107666	ATTCCCTTCACCTGCTCTCCTCCCGTC	25	<lmxll>	ll	121	lm	142		
>ref-107684	CTATAAATAACCCGCTCTCCTCTGCGT	1	<hkxhk>	hh	88	hk	160	kk	0
>ref-107684	CTATAAATAACCCGCTCTCCTCTGCGT	6	<nnxnp>	nn	243	np	27		
>ref-107684	CTATAAATAACCCGCTCTCCTCTGCGT	15	<nnxnp>	nn	243	np	27		
>ref-107684	CTATAAATAACCCGCTCTCCTCTGCGT	25	<lmxll>	ll	112	lm	139		
>ref-107684	CTATAAATAACCCGCTCTCCTCTGCGT	27	<lmxll>	ll	112	lm	139		
>ref-107696	TTTCTTCCCACCTTTTCTCCTCTTCAG	26	<nnxnp>	nn	93	np	145		
>ref-107696	TTTCTTCCCACCTTTTCTCCTCTTCAG	27	<nnxnp>	nn	94	np	145		
>ref-107710	GTGATCCTTACTCTCTCTCCTCTATCA	14	<nnxnp>	nn	124	np	140		
>ref-107715	ATGATAACAACAAAGGCTCCGTGTAAT	3	<nnxnp>	nn	168	np	94		
>ref-107715	ATGATAACAACAAAGGCTCCGTGTAAT	22	<lmxll>	ll	139	lm	124		
>ref-107715	ATGATAACAACAAAGGCTCCGTGTAAT	25	<lmxll>	ll	139	lm	124		
>ref-107733	TTAAGTCCTACATCGACTCCGTGTCAT	7	<hkxhk>	hh	1	hk	269	kk	1
>ref-107733	TTAAGTCCTACATCGACTCCGTGTCAT	12	<hkxhk>	hh	1	hk	269	kk	1
>ref-107750	TTGAACAGTACATATGCTCCAACTTTA	2	<nnxnp>	nn	103	np	125		
>ref-107761	TGCAAACACACATCCACTCCAACACGC	13	<lmxll>	ll	83	lm	169		
>ref-107761	TGCAAACACACATCCACTCCAACACGC	26	<lmxll>	ll	84	lm	170		
>ref-107787	CTTCTTTAAACTCGTTCTCCTCCTCCT	12	<lmxll>	ll	152	lm	99		
>ref-107787	CTTCTTTAAACTCGTTCTCCTCCTCCT	27	<lmxll>	ll	149	lm	100		

>ref-107811	TGTTAGTGCACATGTGCTCCTATGTGT	25	<nnxnp>	nn	131	np	134		
>ref-107821	AAATACTTAACTCCCTCTCCTCTAGAT	21	<lmxll>	ll	121	lm	114		
>ref-107830	ACCTCCATCACCTAACCTCCCCTCTCT	5	<hkxhk>	hh	68	hk	133	kk	47
>ref-107839	TCTCCATGAACCGAATCTCCTTCTCTC	13	<lmxll>	ll	151	lm	119		
>ref-107853	TTTACCGCTACTATGCCTCCCGCAGTA	3	<nnxnp>	nn	115	np	153		
>ref-107858	CGGCTCCTCACTCGCTCTCCCTCTTTC	2	<lmxll>	ll	126	lm	134		
>ref-107898	TCTCGCAGGACGCTCTCTCCTGACGAA	15	<lmxll>	ll	135	lm	130		
>ref-107899	CCACTTTCAACCTCCCCTCCCCCTTTA	3	<lmxll>	ll	110	lm	115		
>ref-107899	CCACTTTCAACCTCCCCTCCCCCTTTA	27	<lmxll>	ll	106	lm	117		
>ref-107910	AAGACTAGAACTAAAACTCCTATACTG	8	<nnxnp>	nn	114	np	133		
>ref-107918	CGGTGTGCCACTCTCCCTCCCTCCCTT	27	<hkxhk>	hh	22	hk	127	kk	81
>ref-107921	TACTCATATACACACACTCCCTTTCGC	23	<lmxll>	ll	221	lm	0		
>ref-107923	ACATGAAAAACACTTCCTCCTGTGTGT	27	<hkxhk>	hh	36	hk	99	kk	92
>ref-107934	CTCTCTCTCACGCCGCCTCCAGTAAAC	4	<lmxll>	ll	134	lm	132		
>ref-107976	AGGAAAGTAACATCAACTCCGATATTG	4	<lmxll>	ll	121	lm	143		
>ref-107989	TCTTTGACAACCTCCCCTCCCCCCCTC	3	<hkxhk>	hh	17	hk	239	kk	1
>ref-107989	TCTTTGACAACCTCCCCTCCCCCCCTC	4	<hkxhk>	hh	17	hk	239	kk	1
>ref-108015	ACATCATCCACACAGTCTCCTCCTCCC	23	<hkxhk>	hh	0	hk	272	kk	0
>ref-108059	TACATTTCAACAGAAGCTCCTTACGCT	3	<lmxll>	ll	138	lm	124		
>ref-108067	TACACCTTCACATATGCTCCTGCATTA	9	<nnxnp>	nn	140	np	119		
>ref-108071	GTGCAACATACCTGTTCTCCCACGGAG	25	<lmxll>	ll	129	lm	131		
>ref-108080	GTAACAAACACATACACTCCCACTCAT	3	<hkxhk>	hh	0	hk	270	kk	0
>ref-108080	GTAACAAACACATACACTCCCACTCAT	22	<lmxll>	ll	137	lm	119		
>ref-108085	TGGTACGTAACTTGTGCTCCCAAGCTG	27	<nnxnp>	nn	271	np	0		
>ref-108087	TGAGTGTAAACGCCCCCTCCCCCCTGT	3	<lmxll>	ll	125	lm	133		
>ref-108088	TCCACCTTAACCAGCACTCCATGTTCT	15	<nnxnp>	nn	209	np	45		
>ref-108093	CAACATCAAACCCTATCTCCTCGACCC	8	<hkxhk>	hh	46	hk	89	kk	92
>ref-108093	CAACATCAAACCCTATCTCCTCGACCC	12	<hkxhk>	hh	46	hk	89	kk	92
>ref-108116	CACCGCTGTACAGAATCTCCTGAAGAC	13	<hkxhk>	hh	63	hk	122	kk	53
>ref-108152	AGAGCTACAACACGAACTCCATCTCCA	1	<hkxhk>	hh	6	hk	258	kk	0
>ref-108152	AGAGCTACAACACGAACTCCATCTCCA	2	<hkxhk>	hh	0	hk	266	kk	5
>ref-108152	AGAGCTACAACACGAACTCCATCTCCA	4	<hkxhk>	hh	106	hk	154	kk	0
>ref-108152	AGAGCTACAACACGAACTCCATCTCCA	8	<lmxll>	ll	6	lm	261		
>ref-108152	AGAGCTACAACACGAACTCCATCTCCA	21	<hkxhk>	hh	106	hk	154	kk	0
>ref-108152	AGAGCTACAACACGAACTCCATCTCCA	23	<lmxll>	ll	6	lm	262		
>ref-108173	CTCTATCAAACTCCTTCTCCTACTCTC	13	<nnxnp>	nn	1	np	269		
>ref-108173	CTCTATCAAACTCCTTCTCCTACTCTC	22	<nnxnp>	nn	1	np	269		
>ref-108177	CACACTTACACTCACACTCCCATAAAT	6	<hkxhk>	hh	0	hk	233	kk	16

>ref-108177	CACACTTACACTCACACTCCCATAAAT	8	<hkxhk>	hh	16	hk	234	kk	0
>ref-108180	CAGGGATGGACGATTACTCCGTAGCAG	16	<lmxll>	ll	128	lm	128		
>ref-108186	ACTTCCCTCACTCGTTCTCCATGGGAC	5	<nnxnp>	nn	259	np	0		
>ref-108222	CCTTATTCTACATCCTCTCCTCCACCC	5	<nnxnp>	nn	160	np	94		
>ref-108222	CCTTATTCTACATCCTCTCCTCCACCC	22	<lmxll>	ll	5	lm	253		
>ref-108222	CCTTATTCTACATCCTCTCCTCCACCC	26	<lmxll>	ll	5	lm	254		
>ref-108238	CTCTTTCATACTTGGACTCCCACTCTC	22	<lmxll>	ll	111	lm	120		
>ref-108246	CTCACATATACACGTGCTCCCACGCAC	8	<nnxnp>	nn	273	np	0		
>ref-108250	ATCACCTACACCACCTCTCCCCCGTAT	4	<nnxnp>	nn	131	np	135		
>ref-108273	TGTGAACCAACCCCTACTCCCTCCCTC	15	<nnxnp>	nn	116	np	127		
>ref-108275	CACACTCCGACGCTTACTCCTCTACTT	24	<lmxll>	ll	132	lm	120		
>ref-108279	AACTACCACACCATCGCTCCACGTCAT	2	<lmxll>	ll	7	lm	264		
>ref-108279	AACTACCACACCATCGCTCCACGTCAT	23	<lmxll>	ll	2	lm	271		
>ref-108302	ACCACCCACACCTCTCCTCCGCCCACC	4	<nnxnp>	nn	222	np	0		
>ref-108307	ATACACACAACTACGTCTCCAGCCAAA	14	<lmxll>	ll	136	lm	133		
>ref-108371	CCCTCACTCACATACTCTCCTCCCCTT	13	<nnxnp>	nn	110	np	148		
>ref-108422	CCCCCTCCCACGCTCCCTCCCACACCC	12	<lmxll>	ll	14	lm	229		
>ref-108422	CCCCCTCCCACGCTCCCTCCCACACCC	24	<lmxll>	ll	15	lm	232		
>ref-108430	GCACCTACTACTGCGCCTCCTTCGCAA	12	<lmxll>	ll	132	lm	125		
>ref-108439	CCACTGAATACGTTCCCTCCCCATGTG	16	<lmxll>	ll	117	lm	148		
>ref-108459	CCAGCAGCTACATTTCCTCCTTGAGCA	2	<lmxll>	ll	9	lm	253		
>ref-108459	CCAGCAGCTACATTTCCTCCTTGAGCA	27	<lmxll>	ll	9	lm	252		
>ref-108471	AGATAATACACACTCACTCCACAGTCA	22	<lmxll>	ll	136	lm	131		
>ref-108486	AACGCCCCTACTTCCTCTCCCTATCTC	1	<lmxll>	ll	155	lm	115		
>ref-108492	GACACACTAACCCTTGCTCCTGCTTGT	22	<lmxll>	ll	140	lm	128		
>ref-108494	GTCCCCCTCACCCTATCTCCCCCACTC	4	<lmxll>	ll	270	lm	0		
>ref-108498	AACCACACCACACCACCTCCACCACCT	7	<nnxnp>	nn	118	np	151		
>ref-108519	AGAACCCAGACTCTCGCTCCTCTTCCC	4	<hkxhk>	hh	52	hk	148	kk	60
>ref-108521	ACACCCTCTACTTCATCTCCTACACCC	4	<hkxhk>	hh	0	hk	272	kk	0
>ref-108521	ACACCCTCTACTTCATCTCCTACACCC	21	<hkxhk>	hh	0	hk	241	kk	24
>ref-108521	ACACCCTCTACTTCATCTCCTACACCC	22	<hkxhk>	hh	8	hk	255	kk	0
>ref-108570	TAAAACAGGACTTCAACTCCAACCATG	7	<nnxnp>	nn	132	np	129		
>ref-108574	TCCCCACTCACCCTCACTCCTTCCTCA	22	<nnxnp>	nn	129	np	131		
>ref-108582	TCACCGATCACCGACTCTCCTTATTCA	24	<lmxll>	ll	132	lm	132		
>ref-108584	TCTGTTACTACAACAACTCCTGATCCA	12	<lmxll>	ll	119	lm	108		
>ref-108588	AGTATGTATACAGTCGCTCCTCTATTT	3	<nnxnp>	nn	149	np	111		
>ref-108600	GAAAACATTACATTGGCTCCTGGACTA	9	<nnxnp>	nn	127	np	125		
>ref-108611	TAGAGGGATACAACACCTCCTCCTCCT	14	<lmxll>	ll	233	lm	0		

>ref-108612	AACCCTACTACCCCCTCTCCCCCAATA	6	<lmxll>	ll	119	lm	130		
>ref-108642	CAGCCACTCACTCACTCTCCCCGTCTC	5	<nnxnp>	nn	107	np	147		
>ref-108663	ATAATTAGTACAGCGCCTCCCGTAACA	1	<nnxnp>	nn	263	np	0		
>ref-108673	TTTGAATTCACGTGTTCTCCTTCTTAG	25	<hkxhk>	hh	47	hk	127	kk	78
>ref-108712	TGCATAACAACGCTTACTCCAGCATCC	12	<lmxll>	ll	149	lm	110		
>ref-108714	CTCCCTTCCACCCTCACTCCCCCAATC	4	<nnxnp>	nn	267	np	1		
>ref-108714	CTCCCTTCCACCCTCACTCCCCCAATC	22	<nnxnp>	nn	255	np	4		
>ref-108728	CTTGCCGCCACCCTCCCTCCTCCCCTT	7	<lmxll>	ll	257	lm	0		
>ref-108728	CTTGCCGCCACCCTCCCTCCTCCCCTT	14	<lmxll>	ll	258	lm	0		
>ref-108764	GCCTTCTCTACTTTCTCTCCCTCTTCT	25	<nnxnp>	nn	122	np	137		
>ref-108772	ATGGATCTCACATACCCTCCTGTGATT	25	<lmxll>	ll	133	lm	134		
>ref-108776	TTTCCTCTCACTCGATCTCCTTGTTTC	9	<nnxnp>	nn	129	np	111		
>ref-108789	CCATCACCAACTACAGCTCCACCACCA	24	<hkxhk>	hh	0	hk	273	kk	0
>ref-108789	CCATCACCAACTACAGCTCCACCACCA	25	<hkxhk>	hh	10	hk	259	kk	0
>ref-108794	ACCTCTTACACGTCATCTCCTCCTGAA	6	<nnxnp>	nn	129	np	137		
>ref-108802	TACCTACCTACCTCCACTCCCCTTCTG	16	<hkxhk>	hh	0	hk	255	kk	10
>ref-108802	TACCTACCTACCTCCACTCCCCTTCTG	27	<hkxhk>	hh	10	hk	255	kk	0
>ref-108813	ATCACCATCACCATCACTCCCGTGCAC	5	<nnxnp>	nn	134	np	136		
>ref-108815	ATGGAACGTACGAACACTCCAACAAGA	7	<hkxhk>	hh	69	hk	123	kk	50
>ref-108821	TCAAAATGTACACCTGCTCCTCTTAAC	25	<nnxnp>	nn	111	np	131		
>ref-108827	AAAACACACACAGTGTCTCCAGTGTGA	16	<lmxll>	ll	116	lm	142		
>ref-108865	TACCTATTCACTCTTACTCCTATCGCT	26	<nnxnp>	nn	267	np	0		
>ref-108873	TTTCTTCCTACCTATTCTCCCCCTCTC	3	<hkxhk>	hh	0	hk	121	kk	128
>ref-108882	AAACTCACCACGCCAACTCCAACTTTC	2	<nnxnp>	nn	6	np	255		
>ref-108887	AGCCCTTGGACGAAGCCTCCATCCCCT	21	<nnxnp>	nn	132	np	139		
>ref-108890	TCTCTTTGCACTGCATCTCCCGTGTTG	6	<lmxll>	ll	138	lm	134		
>ref-108898	CAACCTCCAACCCCACCTCCAACCTCC	2	<lmxll>	ll	7	lm	263		
>ref-108905	GCCTTAACCACAGGGGCTCCCAAACAC	3	<hkxhk>	hh	2	hk	262	kk	0
>ref-108905	GCCTTAACCACAGGGGCTCCCAAACAC	15	<hkxhk>	hh	0	hk	262	kk	2
>ref-108921	TCGTGTTCAACATGGCCTCCTTCCTCT	27	<hkxhk>	hh	39	hk	135	kk	68
>ref-108936	CCCCCTCACACACACCCTCCCGTCCCA	16	<nnxnp>	nn	125	np	137		
>ref-108951	GTCAACAAAACACGCTCTCCTCACATG	3	<hkxhk>	hh	0	hk	272	kk	0
>ref-108981	TGGTGTGGTACACGGACTCCGACCTGC	23	<lmxll>	ll	116	lm	125		
>ref-108994	CTTGCCATCACTTTATCTCCCCTCCCC	4	<nnxnp>	nn	5	np	260		
>ref-108994	CTTGCCATCACTTTATCTCCCCTCCCC	13	<hkxhk>	hh	3	hk	254	kk	6
>ref-108994	CTTGCCATCACTTTATCTCCCCTCCCC	14	<nnxnp>	nn	5	np	260		
>ref-109006	ATGTCACTCACCTTCCCTCCCTCTCCC	1	<lmxll>	ll	266	lm	1		
>ref-109006	ATGTCACTCACCTTCCCTCCCTCTCCC	6	<hkxhk>	hh	265	hk	0	kk	0

>ref-109010	ACTGGTGCCACTCCCCCTCCCGGCTCT	25	<lmxll>	ll	129	lm	133		
>ref-109014	GGTTTAAGAACATGCCCTCCCTCCATA	1	<hkxhk>	hh	0	hk	248	kk	4
>ref-109014	GGTTTAAGAACATGCCCTCCCTCCATA	21	<hkxhk>	hh	20	hk	246	kk	0
>ref-109022	ACCACCGCTACTATTCCTCCTCCTCTT	1	<lmxll>	ll	116	lm	124		
>ref-109038	TCCACTTCCACCTCCTCTCCATTTCCA	16	<hkxhk>	hh	0	hk	264	kk	7
>ref-109038	TCCACTTCCACCTCCTCTCCATTTCCA	27	<hkxhk>	hh	7	hk	264	kk	0
>ref-109046	ACATCCGGGACAGTTCCTCCGCCAGGG	3	<nnxnp>	nn	265	np	0		
>ref-109049	GATGGTGACACTTCTGCTCCTTTTGAG	4	<hkxhk>	hh	0	hk	249	kk	8
>ref-109067	AACACCCATACATTCACTCCTAAACAC	7	<nnxnp>	nn	122	np	122		
>ref-109082	ACTCTTATCACTCTCTCTCCTTCTTTC	13	<nnxnp>	nn	197	np	58		
>ref-109085	GAGACAGTCACTGATGCTCCAGTGACT	23	<hkxhk>	hh	0	hk	195	kk	78
>ref-109085	GAGACAGTCACTGATGCTCCAGTGACT	27	<hkxhk>	hh	0	hk	259	kk	11
>ref-109130	CACACACACACACTTCCTCCTCTACTA	16	<nnxnp>	nn	131	np	134		
>ref-109142	TTGGGATACACGACTTCTCCCCCAGAA	21	<lmxll>	ll	147	lm	90		
>ref-109151	TTTACGCTCACTCATGCTCCTCCTCCT	22	<nnxnp>	nn	139	np	129		
>ref-109154	CCTATTACCACCGTCCCTCCCACCCTC	3	<nnxnp>	nn	119	np	139		
>ref-109159	CAAGGTGGAACGCTCGCTCCGCTGCTT	8	<nnxnp>	nn	149	np	120		
>ref-109162	GATCTACCCACACCTTCTCCTCGTCCA	5	<hkxhk>	hh	56	hk	158	kk	42
>ref-109162	GATCTACCCACACCTTCTCCTCGTCCA	7	<hkxhk>	hh	42	hk	159	kk	56
>ref-109162	GATCTACCCACACCTTCTCCTCGTCCA	9	<nnxnp>	nn	134	np	132		
>ref-109164	ACCTCTTTTACCCTCCCTCCCTTCCCC	9	<nnxnp>	nn	2	np	269		
>ref-109164	ACCTCTTTTACCCTCCCTCCCTTCCCC	14	<hkxhk>	hh	0	hk	170	kk	97
>ref-109164	ACCTCTTTTACCCTCCCTCCCTTCCCC	16	<nnxnp>	nn	2	np	269		
>ref-109164	ACCTCTTTTACCCTCCCTCCCTTCCCC	25	<hkxhk>	hh	98	hk	168	kk	0
>ref-109170	CACCCTGCTACCTTCTCTCCTTTCTTT	13	<nnxnp>	nn	114	np	135		
>ref-109175	ATACCTTGGACTTAAGCTCCTCCCCCT	15	<nnxnp>	nn	121	np	144		
>ref-109182	GTAATCAAGACTGTGGCTCCGCTCCTG	22	<lmxll>	ll	6	lm	265		
>ref-109186	CACGAAAGCACTAGCGCTCCCTGCGGC	6	<hkxhk>	hh	61	hk	143	kk	59
>ref-109228	TAGCCACACACGTCTCCTCCCTGGCTC	3	<lmxll>	ll	143	lm	120		
>ref-109237	CTAATTATCACCCTATCTCCCTCACAT	12	<lmxll>	ll	1	lm	259		
>ref-109237	CTAATTATCACCCTATCTCCCTCACAT	14	<lmxll>	ll	1	lm	259		
>ref-109268	AGTAACGTGACTGCAACTCCTACAGCT	7	<hkxhk>	hh	1	hk	270	kk	0
>ref-109283	GTAAATGACACTGCATCTCCAAAACTA	13	<hkxhk>	hh	0	hk	236	kk	17
>ref-109283	GTAAATGACACTGCATCTCCAAAACTA	25	<hkxhk>	hh	0	hk	261	kk	3
>ref-109302	CAGCATCTAACTCCCCCTCCCTCTCCC	3	<lmxll>	ll	4	lm	250		
>ref-109302	CAGCATCTAACTCCCCCTCCCTCTCCC	5	<lmxll>	ll	4	lm	249		
>ref-109336	CTTGCTGAGACCTTCCCTCCACTCCCT	2	<hkxhk>	hh	0	hk	269	kk	2
>ref-109336	CTTGCTGAGACCTTCCCTCCACTCCCT	7	<lmxll>	ll	60	lm	197		

>ref-109336	CTTGCTGAGACCTTCCCTCCACTCCCT	26	<hkxhk>	hh	0	hk	223	kk	42
>ref-109415	TTCGTCCTTACCAGGCCTCCCAGACCT	15	<lmxll>	ll	137	lm	123		
>ref-109419	GGGGGTAGGACCGGCTCTCCGCTTTCT	12	<nnxnp>	nn	265	np	0		
>ref-109446	GTATGTATGACCACGTCTCCTTCCCTT	2	<nnxnp>	nn	133	np	131		
>ref-109446	GTATGTATGACCACGTCTCCTTCCCTT	3	<nnxnp>	nn	129	np	134		
>ref-109456	TTCTTCGCCACGTCTCCTCCTCACGTG	2	<lmxll>	ll	111	lm	153		
>ref-109464	GATGACTGTACTGACTCTCCCGCTCCC	15	<lmxll>	ll	143	lm	113		
>ref-109470	TCACCTCGAACTACGTCTCCACACAGA	2	<hkxhk>	hh	43	hk	132	kk	58
>ref-109497	CTCCTCTCCACCTCTCCTCCCTCGCCC	3	<hkxhk>	hh	7	hk	249	kk	0
>ref-109497	CTCCTCTCCACCTCTCCTCCCTCGCCC	4	<hkxhk>	hh	6	hk	249	kk	0
>ref-109497	CTCCTCTCCACCTCTCCTCCCTCGCCC	6	<nnxnp>	nn	86	np	150		
>ref-109497	CTCCTCTCCACCTCTCCTCCCTCGCCC	24	<nnxnp>	nn	70	np	163		
>ref-109524	ACTCACCCGACAATCTCTCCGACCTCT	8	<lmxll>	ll	138	lm	129		
>ref-109531	CCTCATCCAACCCCCTCTCCCTTTCCC	1	<hkxhk>	hh	0	hk	266	kk	3
>ref-109531	CCTCATCCAACCCCCTCTCCCTTTCCC	5	<hkxhk>	hh	3	hk	259	kk	0
>ref-109531	CCTCATCCAACCCCCTCTCCCTTTCCC	8	<nnxnp>	nn	76	np	178		
>ref-109531	CCTCATCCAACCCCCTCTCCCTTTCCC	23	<nnxnp>	nn	70	np	185		
>ref-109537	TTTCCGGCTACGTCCTCTCCCTCTTCC	12	<lmxll>	ll	130	lm	120		
>ref-109537	TTTCCGGCTACGTCCTCTCCCTCTTCC	26	<lmxll>	ll	126	lm	124		
>ref-109567	CGCCCGATAACGTCAGCTCCATTTCTC	3	<nnxnp>	nn	144	np	127		
>ref-109576	GGAGCGCCCACACCACCTCCGCAACCC	22	<lmxll>	ll	168	lm	104		
>ref-109576	GGAGCGCCCACACCACCTCCGCAACCC	23	<nnxnp>	nn	103	np	166		
>ref-109582	CATGGAAATACTTTGGCTCCGATCAAG	1	<lmxll>	ll	116	lm	127		
>ref-109601	AACCCACGCACATCCCCTCCTCCTCTC	24	<lmxll>	ll	123	lm	97		
>ref-109625	TAGATATAAACTTTTCCTCCGCCCATG	21	<nnxnp>	nn	117	np	129		
>ref-109641	ACCTAGCTAACCCTGTCTCCCACTCTC	12	<hkxhk>	hh	0	hk	272	kk	0
>ref-109641	ACCTAGCTAACCCTGTCTCCCACTCTC	22	<hkxhk>	hh	0	hk	273	kk	0
>ref-109656	TCTCTTTCCACCTTCCCTCCTGTTCTC	7	<nnxnp>	nn	117	np	146		
>ref-109667	AACGCCCCAACCCCCTCTCCCTTCTCC	22	<hkxhk>	hh	0	hk	189	kk	67
>ref-109684	CCCTACTTTACCTTACCTCCATATCTC	4	<nnxnp>	nn	125	np	133		
>ref-109699	AAAATGATAACAACTTCTCCTTACGTG	1	<hkxhk>	hh	5	hk	267	kk	0
>ref-109703	CACTTATGAACCCGCCCTCCGCCTCCC	7	<nnxnp>	nn	129	np	136		
>ref-109769	CGCACGCACACGCACTCTCCATCCATA	12	<lmxll>	ll	128	lm	143		
>ref-109794	TTGAAATGGACGCCGACTCCGCGGGAG	3	<nnxnp>	nn	125	np	135		
>ref-109801	TGTAAGGGAACTTGCTCTCCGCTTAGA	25	<lmxll>	ll	135	lm	120		
>ref-109814	CAGACACAAACTTGCTCTCCAGACTGT	3	<hkxhk>	hh	48	hk	139	kk	63
>ref-109832	ATGGCTTCCACTAATTCTCCTTGAACG	6	<nnxnp>	nn	122	np	135		
>ref-109837	TATTCACTAACGTATGCTCCGACGCCT	14	<hkxhk>	hh	36	hk	216	kk	0

>ref-109837	TATTCACTAACGTATGCTCCGACGCCT	24	<hkxhk>	hh	0	hk	222	kk	30
>ref-109860	CAATTGGTGACCTTCTCTCCGGCGCAG	16	<lmxll>	ll	140	lm	130		
>ref-109869	GAGGGACTGACTGCCTCTCCCTTGCCT	2	<nnxnp>	nn	124	np	145		
>ref-109878	AAGTTGTTTACATATGCTCCTTGGTAA	5	<nnxnp>	nn	113	np	112		
>ref-109879	ATGAACGAGACGATGCCTCCCTTACGT	8	<lmxll>	ll	129	lm	137		
>ref-109909	TGATAGCATACTTCGTCTCCGAGAAAA	15	<lmxll>	ll	129	lm	113		
>ref-109912	GCTACCACCACACTTGCTCCCAGGCGA	2	<lmxll>	ll	109	lm	150		
>ref-109916	AAAGCACTGACTTCGACTCCAAACCCA	22	<nnxnp>	nn	142	np	122		
>ref-109933	TCTTTTTACACTTTCACTCCTTTTCCA	3	<nnxnp>	nn	96	np	129		
>ref-109944	TACTGTAATACTATAGCTCCAGATATA	12	<lmxll>	ll	123	lm	110		
>ref-109950	AGCCCGCGAACCCCAACTCCCTTTCCC	3	<nnxnp>	nn	126	np	109		
>ref-109973	AAAACTAAGACGCCTTCTCCATCGACA	6	<lmxll>	ll	148	lm	115		
>ref-110026	ACGCCCCCAACCATTTCTCCACCGGTT	16	<nnxnp>	nn	126	np	124		
>ref-110049	GACAAGAAGACTTACCCTCCGGCGGTG	27	<hkxhk>	hh	0	hk	272	kk	1
>ref-110059	ACTTGCGGGACCTCCTCTCCGACAGCG	4	<hkxhk>	hh	0	hk	272	kk	0
>ref-110059	ACTTGCGGGACCTCCTCTCCGACAGCG	9	<hkxhk>	hh	0	hk	272	kk	0
>ref-110090	AACGGCGGTACTTTAACTCCTCTGGTA	25	<nnxnp>	nn	106	np	149		
>ref-110099	TCCGGTAGGACCCATTCTCCCGCCGCT	9	<lmxll>	ll	259	lm	0		
>ref-110117	AGTAACTCGACGGTTCCTCCGCACCAT	5	<lmxll>	ll	130	lm	137		
>ref-110117	AGTAACTCGACGGTTCCTCCGCACCAT	22	<nnxnp>	nn	127	np	142		
>ref-110132	CTCTGCGCCACATCATCTCCTCCCGGG	3	<hkxhk>	hh	0	hk	270	kk	0
>ref-110132	CTCTGCGCCACATCATCTCCTCCCGGG	14	<hkxhk>	hh	43	hk	214	kk	0
>ref-110132	CTCTGCGCCACATCATCTCCTCCCGGG	15	<hkxhk>	hh	43	hk	214	kk	0
>ref-110143	AAGGTTTCGACCCCCTCTCCCGGGTCG	27	<nnxnp>	nn	260	np	0		
>ref-110159	GTTTCTGGCACAGCAACTCCATCAGCT	16	<lmxll>	ll	109	lm	144		
>ref-110185	CCCCCCCGCACCCATCCTCCTTCCCAT	1	<nnxnp>	nn	230	np	1		
>ref-110211	ACCCACCCTACTCTCCCTCCTCTTCAT	4	<nnxnp>	nn	117	np	141		
>ref-110258	CCGAGAGAAACGCCCTCTCCATGCGTT	26	<hkxhk>	hh	2	hk	271	kk	0
>ref-110259	GACAGACACACAACTTCTCCCAACAAA	8	<hkxhk>	hh	99	hk	153	kk	0
>ref-110259	GACAGACACACAACTTCTCCCAACAAA	14	<hkxhk>	hh	98	hk	153	kk	0
>ref-110283	ATTCGCCCCACTACCCCTCCGCCCCAG	24	<nnxnp>	nn	121	np	136		
>ref-110305	TTGAAAAAAACGTCATCTCCTTTCCTT	3	<hkxhk>	hh	5	hk	264	kk	0
>ref-110305	TTGAAAAAAACGTCATCTCCTTTCCTT	4	<hkxhk>	hh	5	hk	264	kk	0
>ref-110325	TGAAACTCCACCTTCCCTCCTTCCCCC	6	<lmxll>	ll	246	lm	0		
>ref-110325	TGAAACTCCACCTTCCCTCCTTCCCCC	8	<lmxll>	ll	246	lm	0		
>ref-110327	CCTTAGGTCACTTTCCCTCCCTCTCGT	12	<lmxll>	ll	125	lm	145		
>ref-110341	CTCCCCCTCACTCTCTCTCCACCAGAT	23	<lmxll>	ll	124	lm	114		
>ref-110349	TAATTAGCAACATGTCCTCCGTTCATC	4	<nnxnp>	nn	116	np	154		

>ref-110349	TAATTAGCAACATGTCCTCCGTTCATC	22	<nnxnp>	nn	116	np	154		
>ref-110380	ATCGAGTTCACAGTCTCTCCTTCTGTT	21	<hkxhk>	hh	52	hk	138	kk	62
>ref-110392	CTCATTGACACCATATCTCCCTCACAA	5	<nnxnp>	nn	96	np	146		
>ref-110392	CTCATTGACACCATATCTCCCTCACAA	16	<nnxnp>	nn	96	np	146		
>ref-110443	CTCATTCCAACCTTCCCTCCACCCTCA	6	<lmxll>	ll	133	lm	121		
>ref-110457	TCCTGCGTCACCAATCCTCCAATCTCT	8	<nnxnp>	nn	124	np	141		
>ref-110488	TGGTCAGTAACTTTCTCTCCTGATGGT	15	<nnxnp>	nn	253	np	0		
>ref-110502	CCCTTCTTCACCCCGTCTCCACCTCAT	2	<lmxll>	ll	133	lm	134		
>ref-110537	GCAGAGGGAACGTCTTCTCCTCTGACA	12	<hkxhk>	hh	48	hk	131	kk	70
>ref-110575	CCTCGTCTAACGCCACCTCCTCTCATC	5	<lmxll>	ll	67	lm	194		
>ref-110602	CCACTTTGCACTTGCACTCCAACCTTG	14	<lmxll>	ll	140	lm	131		
>ref-110610	TACACCCAAACTGTTTCTCCCTGTAAT	1	<hkxhk>	hh	57	hk	127	kk	60
>ref-110704	CCCATAGGCACTACCTCTCCGTCCTTC	9	<hkxhk>	hh	55	hk	215	kk	0
>ref-110706	ACACCCTTCACCCTTCCTCCATCTCTT	21	<nnxnp>	nn	126	np	135		
>ref-110710	CACTCAAATACGGACTCTCCCCCACAC	2	<lmxll>	ll	129	lm	140		
>ref-110712	AAGTAAACCACGCCCCCTCCCCTGAAT	26	<nnxnp>	nn	125	np	122		
>ref-110725	CTGGGCAACACGGAGACTCCCTGCCCC	4	<nnxnp>	nn	122	np	146		
>ref-110727	TCCCTAACGACCTCAGCTCCCTTTTAA	5	<nnxnp>	nn	125	np	133		
>ref-110731	TGAGGCACCACACCAGCTCCTGCGTCC	1	<hkxhk>	hh	0	hk	272	kk	0
>ref-110731	TGAGGCACCACACCAGCTCCTGCGTCC	12	<hkxhk>	hh	0	hk	272	kk	0
>ref-110740	CGAGAGCGGACTTCCCCTCCCCTCCCT	9	<hkxhk>	hh	68	hk	103	kk	52
>ref-110783	GCGTCAGGTACGTGATCTCCGTGCGTG	24	<lmxll>	ll	150	lm	119		
>ref-110807	TCTACCAACACTACCACTCCCAGTTCC	25	<hkxhk>	hh	64	hk	130	kk	55
>ref-110819	GCAACACTAACTCTTCCTCCTTCAACA	13	<lmxll>	ll	111	lm	130		
>ref-110819	GCAACACTAACTCTTCCTCCTTCAACA	27	<lmxll>	ll	111	lm	129		
>ref-110848	ATCGGTATGACCAGTTCTCCCGCTTTC	6	<lmxll>	ll	130	lm	139		
>ref-110851	TATTTACACACAGCCCCTCCCCACCCC	13	<nnxnp>	nn	136	np	128		
>ref-110872	CATCACCCAACTTCCCCTCCTCCTCCC	22	<hkxhk>	hh	52	hk	134	kk	69
>ref-110879	CCTCCTCATACTCATACTCCTTCCCTT	3	<lmxll>	ll	142	lm	119		
>ref-110879	CCTCCTCATACTCATACTCCTTCCCTT	23	<lmxll>	ll	141	lm	119		
>ref-110898	CTCTCCTTCACATTATCTCCCTCACTC	2	<nnxnp>	nn	118	np	141		
>ref-110916	GCCATGCTCACCATCACTCCAACATCA	2	<hkxhk>	hh	0	hk	273	kk	0
>ref-110916	GCCATGCTCACCATCACTCCAACATCA	6	<hkxhk>	hh	0	hk	273	kk	0
>ref-110918	TTAAATGTTACGCCAACTCCCCAAATA	4	<nnxnp>	nn	100	np	133		
>ref-110942	CAGCCTTATACTCTCCCTCCCCCCTCC	3	<lmxll>	ll	142	lm	117		
>ref-110947	GGTGCGGACACTGTATCTCCTGAGCAA	26	<lmxll>	ll	129	lm	136		
>ref-110961	GAGGGGAGGACGCCGACTCCGACCAGA	6	<lmxll>	ll	82	lm	177		
>ref-110965	GTTTCTCCAACCTCGTCTCCATCCTCT	12	<nnxnp>	nn	234	np	0		

>ref-110972	TTATAATTAACCAGGGCTCCTTCGGGA	4	<nnxnp>	nn	260	np	0		
>ref-110975	ATACTACTCACCTTAACTCCTCTTTAA	25	<nnxnp>	nn	128	np	119		
>ref-110997	TATTCCACAACAGCACCTCCATAACGA	15	<hkxhk>	hh	0	hk	249	kk	15
>ref-110998	CTTTCTCGCACTGTTTCTCCATTAATC	5	<nnxnp>	nn	15	np	227		
>ref-111003	ATCATCCTAACGGTGTCTCCGTTCAGT	5	<hkxhk>	hh	0	hk	256	kk	14
>ref-111010	ATAAAACTTACTTGATCTCCATCTCCA	8	<nnxnp>	nn	119	np	123		
>ref-111010	ATAAAACTTACTTGATCTCCATCTCCA	9	<nnxnp>	nn	120	np	122		
>ref-111025	AATTGATTTACCTTCACTCCTTCACTT	23	<lmxll>	ll	133	lm	123		
>ref-111069	ATCGGCCGGACAGACACTCCATCCCGG	1	<lmxll>	ll	243	lm	10		
>ref-111069	ATCGGCCGGACAGACACTCCATCCCGG	7	<hkxhk>	hh	84	hk	163	kk	0
>ref-111069	ATCGGCCGGACAGACACTCCATCCCGG	8	<hkxhk>	hh	0	hk	254	kk	0
>ref-111069	ATCGGCCGGACAGACACTCCATCCCGG	14	<hkxhk>	hh	241	hk	9	kk	0
>ref-111069	ATCGGCCGGACAGACACTCCATCCCGG	22	<hkxhk>	hh	0	hk	254	kk	0
>ref-111074	ATCAGGCATACCATCTCTCCCTCGACG	23	<hkxhk>	hh	12	hk	218	kk	2
>ref-111074	ATCAGGCATACCATCTCTCCCTCGACG	24	<hkxhk>	hh	2	hk	218	kk	12
>ref-111091	CCTTCCCCCACCTCCTCTCCTAGCATC	21	<lmxll>	ll	70	lm	166		
>ref-111091	CCTTCCCCCACCTCCTCTCCTAGCATC	26	<lmxll>	ll	71	lm	166		
>ref-111141	TCCCCCACAACCCTTCCTCCCCACTCA	15	<nnxnp>	nn	266	np	0		
>ref-111141	TCCCCCACAACCCTTCCTCCCCACTCA	25	<nnxnp>	nn	267	np	0		
>ref-111164	GTCCCCCTAACCCCCCCTCCTTCTCAT	26	<nnxnp>	nn	246	np	1		
>ref-111167	GTACCCTCGACGGGTCCTCCTGTCCCT	27	<nnxnp>	nn	137	np	127		
>ref-111178	GACGCGAGCACGCCTCCTCCTCCTCCT	6	<hkxhk>	hh	0	hk	270	kk	0
>ref-111178	GACGCGAGCACGCCTCCTCCTCCTCCT	7	<hkxhk>	hh	0	hk	270	kk	0
>ref-111186	CCGATGCTCACCTGATCTCCGCTGCCT	25	<hkxhk>	hh	72	hk	138	kk	58
>ref-111208	GTTATTACCACCATTACTCCTAGTAGT	3	<nnxnp>	nn	119	np	114		
>ref-111209	CCAAATCCTACAAATCCTCCTTCCCTC	2	<nnxnp>	nn	142	np	131		
>ref-111209	CCAAATCCTACAAATCCTCCTTCCCTC	7	<lmxll>	ll	13	lm	252		
>ref-111215	AGTTACCAAACTATATCTCCTTAATCT	23	<lmxll>	ll	123	lm	121		
>ref-111233	GATTGTCTCACTCTTCCTCCCTCCCTT	1	<nnxnp>	nn	163	np	87		
>ref-111233	GATTGTCTCACTCTTCCTCCCTCCCTT	5	<nnxnp>	nn	163	np	87		
>ref-111254	ATATTAAAGACATTAACTCCTAACTAC	9	<nnxnp>	nn	120	np	130		
>ref-111272	TACCCCTCCACCCTTTCTCCCCATCTC	21	<lmxll>	ll	5	lm	265		
>ref-111272	TACCCCTCCACCCTTTCTCCCCATCTC	24	<lmxll>	ll	5	lm	265		
>ref-111312	GCTTGTTAAACATCTGCTCCTTTCATA	22	<lmxll>	ll	118	lm	141		
>ref-111316	CGCATGCGGACACGCACTCCAGGGTAG	12	<nnxnp>	nn	131	np	139		
>ref-111344	ATCTATCCCACCCTCCCTCCACCCTTC	6	<nnxnp>	nn	8	np	264		
>ref-111350	TCTTCCCTCACCCTTACTCCCTTCTCC	1	<lmxll>	ll	182	lm	72		
>ref-111350	TCTTCCCTCACCCTTACTCCCTTCTCC	15	<lmxll>	ll	183	lm	70		

>ref-111368	CCAGGGGGGACTTGTCCTCCGAGAGGA	4	<nnxnp>	nn	192	np	64		
>ref-111368	CCAGGGGGGACTTGTCCTCCGAGAGGA	5	<lmxll>	ll	0	lm	268		
>ref-111368	CCAGGGGGGACTTGTCCTCCGAGAGGA	7	<nnxnp>	nn	228	np	33		
>ref-111386	CACCCCCTTACCCCCTCTCCCAGCTCC	21	<nnxnp>	nn	10	np	241		
>ref-111402	ACAACAGCAACATATGCTCCATCCATT	2	<lmxll>	ll	126	lm	132		
>ref-111448	CTCTTCTCTACCTCCCCTCCGTCTTCC	22	<lmxll>	ll	126	lm	120		
>ref-111468	CTTCATCGCACCCCATCTCCATCTCCC	26	<nnxnp>	nn	5	np	258		
>ref-111468	CTTCATCGCACCCCATCTCCATCTCCC	27	<nnxnp>	nn	5	np	258		
>ref-111501	ATCATTATCACTCCCACTCCTTGTCTT	23	<lmxll>	ll	128	lm	97		
>ref-111514	TCCACCCTTACCCCCACTCCCCACTCA	2	<hkxhk>	hh	42	hk	138	kk	58
>ref-111514	TCCACCCTTACCCCCACTCCCCACTCA	25	<nnxnp>	nn	135	np	113		
>ref-111525	CCATCACTTACAGGATCTCCCTCACGT	15	<nnxnp>	nn	112	np	145		
>ref-111536	AGGTTAAGCACTCAGACTCCGCGATTC	2	<hkxhk>	hh	65	hk	149	kk	45
>ref-111560	GGATCTCTTACGTCCGCTCCTGCAGGC	16	<lmxll>	ll	264	lm	0		
>ref-111586	CCCTCCCTCACCCCCACTCCTCCACTC	8	<lmxll>	ll	78	lm	152		
>ref-111591	ATCTCAGCTACCCATCCTCCTTCTCTC	21	<lmxll>	ll	137	lm	129		
>ref-111598	AGAAAGAAAACAGCCTCTCCTCCCGAG	14	<nnxnp>	nn	108	np	116		
>ref-111600	GAAGCCTCAACGCCCCCTCCTCCCCTC	12	<nnxnp>	nn	265	np	0		
>ref-111607	AGATGTGGCACATGTTCTCCAGGCTGC	26	<hkxhk>	hh	69	hk	146	kk	47
>ref-111612	GAGGGCCGCACGCACACTCCACGCCGA	12	<hkxhk>	hh	1	hk	220	kk	47
>ref-111612	GAGGGCCGCACGCACACTCCACGCCGA	27	<hkxhk>	hh	48	hk	218	kk	1
>ref-111624	TATCTGTCTACTTATCCTCCTCTTTCC	24	<hkxhk>	hh	55	hk	190	kk	0
>ref-111624	TATCTGTCTACTTATCCTCCTCTTTCC	27	<nnxnp>	nn	202	np	58		
>ref-111645	AGGGCCAGGACCTGCACTCCTGGGCTT	1	<hkxhk>	hh	37	hk	223	kk	0
>ref-111645	AGGGCCAGGACCTGCACTCCTGGGCTT	3	<hkxhk>	hh	0	hk	126	kk	139
>ref-111645	AGGGCCAGGACCTGCACTCCTGGGCTT	12	<hkxhk>	hh	59	hk	200	kk	0
>ref-111645	AGGGCCAGGACCTGCACTCCTGGGCTT	13	<hkxhk>	hh	0	hk	222	kk	38
>ref-111645	AGGGCCAGGACCTGCACTCCTGGGCTT	14	<hkxhk>	hh	0	hk	198	kk	59
>ref-111645	AGGGCCAGGACCTGCACTCCTGGGCTT	21	<hkxhk>	hh	0	hk	126	kk	139
>ref-111649	TGGCGAGTCACAATGCCTCCTCAGTTG	5	<nnxnp>	nn	135	np	133		
>ref-111659	GTACATAACACCCTTTCTCCCAATCCC	2	<lmxll>	ll	149	lm	105		
>ref-111659	GTACATAACACCCTTTCTCCCAATCCC	12	<lmxll>	ll	147	lm	105		
>ref-111662	CTTTAGCAGACAACCCCTCCTTCTCCT	14	<nnxnp>	nn	124	np	133		
>ref-111672	CAGCCTGGAACTAACTCTCCAAAGGGA	26	<nnxnp>	nn	107	np	142		
>ref-111682	AACACACATACACATACTCCCACATCA	9	<nnxnp>	nn	88	np	146		
>ref-111682	AACACACATACACATACTCCCACATCA	26	<nnxnp>	nn	94	np	141		
>ref-111692	TTTCCTATCACCGTTACTCCCTCCCTG	9	<lmxll>	ll	126	lm	136		
>ref-111692	TTTCCTATCACCGTTACTCCCTCCCTG	15	<hkxhk>	hh	74	hk	136	kk	52

>ref-111692	TTTCCTATCACCGTTACTCCCTCCCTG	27	<nnxnp>	nn	114	np	147		
>ref-111722	CCACGCACTACCCATTCTCCCCACCTC	4	<nnxnp>	nn	273	np	0		
>ref-111743	CTCTCCAACACAATCCCTCCCCCTTCC	12	<lmxll>	ll	0	lm	265		
>ref-111743	CTCTCCAACACAATCCCTCCCCCTTCC	24	<lmxll>	ll	0	lm	265		
>ref-111757	GCTGTTTTCACACTGTCTCCGGAACGG	24	<lmxll>	ll	118	lm	128		
>ref-111757	GCTGTTTTCACACTGTCTCCGGAACGG	26	<lmxll>	ll	119	lm	128		
>ref-111767	AGGCACACAACGGACTCTCCTGCATGG	23	<lmxll>	ll	109	lm	122		
>ref-111769	GCGGAAACGACTGCGGCTCCGGTTTAG	27	<nnxnp>	nn	248	np	0		
>ref-111775	CACTACACCACTGACACTCCCCGAGCA	8	<nnxnp>	nn	143	np	125		
>ref-111792	TGGGGAGGAACTGCCCCTCCTAGCCCC	21	<lmxll>	ll	95	lm	150		
>ref-111808	TCCTTCATCACCCAGCCTCCTCTCCAT	1	<nnxnp>	nn	219	np	0		
>ref-111826	CTCAGATTGACTCTGGCTCCACTCACT	8	<lmxll>	ll	144	lm	125		
>ref-111829	ACCGCCAACACTCCCACTCCCACTACT	12	<hkxhk>	hh	135	hk	108	kk	1
>ref-111829	ACCGCCAACACTCCCACTCCCACTACT	13	<hkxhk>	hh	137	hk	110	kk	1
>ref-111832	TTAATTTTCACGCAAACTCCTCTCTTA	2	<nnxnp>	nn	114	np	115		
>ref-111864	GATCAACAAACACAAACTCCACAAAGA	9	<lmxll>	ll	113	lm	146		
>ref-111870	CGCATTCATACTTTCCCTCCTTTAGTA	2	<nnxnp>	nn	138	np	82		
>ref-111882	CCTCCTTCCACATTACCTCCCTCATTT	25	<hkxhk>	hh	0	hk	273	kk	0
>ref-111884	CCACCTCCAACACCCGCTCCTTATCCT	8	<hkxhk>	hh	67	hk	148	kk	50
>ref-111884	CCACCTCCAACACCCGCTCCTTATCCT	25	<hkxhk>	hh	67	hk	148	kk	50
>ref-111894	TCCTATCACACCGCCCCTCCCCTCCCT	27	<nnxnp>	nn	100	np	152		
>ref-111912	TATAAGCGTACAATCCCTCCTTACATG	15	<nnxnp>	nn	117	np	151		
>ref-111929	GACCGGTGTACCTGCCCTCCTCTTCCC	26	<lmxll>	ll	256	lm	0		
>ref-111932	CCCCTATGCACCCTCCCTCCTCTCTCC	1	<nnxnp>	nn	113	np	140		
>ref-111958	TAATCCCCAACCCCCCCTCCCCATCCT	24	<hkxhk>	hh	0	hk	201	kk	39
>ref-111971	CTGGCTCAAACCCGACCTCCCGGCGAG	8	<hkxhk>	hh	8	hk	261	kk	0
>ref-111971	CTGGCTCAAACCCGACCTCCCGGCGAG	16	<hkxhk>	hh	8	hk	261	kk	0
>ref-111984	ACCAATACCACGGCTGCTCCTTGGGTG	16	<lmxll>	ll	112	lm	151		
>ref-111998	ATTATCCGCACACCAACTCCACTATCC	22	<lmxll>	ll	120	lm	130		
>ref-111999	GTGAACAATACCAACCCTCCTTTCTCT	23	<lmxll>	ll	128	lm	128		
>ref-112022	TCCTCTCCAACACCCTCTCCCCCTTCC	4	<lmxll>	ll	140	lm	129		
>ref-112022	TCCTCTCCAACACCCTCTCCCCCTTCC	6	<lmxll>	ll	139	lm	129		
>ref-112024	CTGCCTCTTACTCCCTCTCCCCCTGCC	15	<lmxll>	ll	110	lm	118		
>ref-112049	ACTCATTACACCCATCCTCCCTTATTC	12	<nnxnp>	nn	108	np	143		
>ref-112054	CAATCATTTACACTTTCTCCTTCCTCC	4	<hkxhk>	hh	0	hk	273	kk	0
>ref-112054	CAATCATTTACACTTTCTCCTTCCTCC	5	<nnxnp>	nn	231	np	34		
>ref-112054	CAATCATTTACACTTTCTCCTTCCTCC	9	<hkxhk>	hh	0	hk	273	kk	0
>ref-112054	CAATCATTTACACTTTCTCCTTCCTCC	27	<hkxhk>	hh	0	hk	250	kk	17

>ref-112077	ACCCACCACACAAGAGCTCCCTTTGAT	23	<lmxll>	ll	139	lm	124		
>ref-112078	GTCCTGAAGACATGCACTCCATTCCTG	1	<nnxnp>	nn	106	np	127		
>ref-112100	TTCCTTACAACCTTTTCTCCTCCTTCC	9	<hkxhk>	hh	2	hk	224	kk	26
>ref-112100	TTCCTTACAACCTTTTCTCCTCCTTCC	21	<hkxhk>	hh	26	hk	225	kk	2
>ref-112107	CCAATGGAGACGCCCCCTCCTGCAGAC	16	<lmxll>	ll	127	lm	134		
>ref-112136	CTTTGAAATACACAAGCTCCTCTTTTT	5	<nnxnp>	nn	249	np	0		
>ref-112136	CTTTGAAATACACAAGCTCCTCTTTTT	21	<hkxhk>	hh	48	hk	131	kk	53
>ref-112146	CTGTTCACTACTTACACTCCTTATCGT	9	<nnxnp>	nn	105	np	138		
>ref-112157	CTTTTGCTAACCTTCTCTCCTCCATCT	1	<hkxhk>	hh	37	hk	217	kk	0
>ref-112157	CTTTTGCTAACCTTCTCTCCTCCATCT	2	<hkxhk>	hh	0	hk	217	kk	37
>ref-112157	CTTTTGCTAACCTTCTCTCCTCCATCT	5	<lmxll>	ll	76	lm	180		
>ref-112157	CTTTTGCTAACCTTCTCTCCTCCATCT	6	<lmxll>	ll	76	lm	180		
>ref-112163	TGATTGTGTACGGTCTCTCCGTGTTGT	16	<lmxll>	ll	109	lm	132		
>ref-112188	CACACTCGCACTCGCACTCCTTCTGCT	21	<hkxhk>	hh	62	hk	137	kk	68
>ref-112233	TGCAATTAGACATTTACTCCACGGTAT	25	<nnxnp>	nn	133	np	136		
>ref-112250	CATAAACGTACATACACTCCCACAAAC	3	<lmxll>	ll	107	lm	118		
>ref-112259	CGATTGAACACTTCTTCTCCTGCCTTC	4	<lmxll>	ll	145	lm	127		
>ref-112259	CGATTGAACACTTCTTCTCCTGCCTTC	5	<lmxll>	ll	145	lm	127		
>ref-112260	CCCTCTCCAACACCCACTCCTCCTTCT	3	<hkxhk>	hh	4	hk	264	kk	0
>ref-112260	CCCTCTCCAACACCCACTCCTCCTTCT	13	<hkxhk>	hh	4	hk	264	kk	0
>ref-112270	ATAAAAACCACATTATCTCCGCTTTAA	21	<hkxhk>	hh	57	hk	139	kk	62
>ref-112305	CAATATCGGACGGGTTCTCCCCGGGTT	27	<hkxhk>	hh	53	hk	141	kk	53
>ref-112326	CTCCCTCTCACTTCCCCTCCGTTTATT	16	<lmxll>	ll	5	lm	256		
>ref-112326	CTCCCTCTCACTTCCCCTCCGTTTATT	23	<lmxll>	ll	5	lm	256		
>ref-112329	AAGCGGTACACGATTTCTCCATGTATA	3	<lmxll>	ll	123	lm	117		
>ref-112338	TGTGGAGACACTCCGTCTCCTTTCTTG	5	<hkxhk>	hh	122	hk	121	kk	3
>ref-112346	CTCTCCTCCACACTCCCTCCTTCTCTT	13	<nnxnp>	nn	152	np	112		
>ref-112346	CTCTCCTCCACACTCCCTCCTTCTCTT	22	<nnxnp>	nn	144	np	116		
>ref-112372	CTCCGCCCCACCGGTCCTCCTCTCCCT	6	<lmxll>	ll	262	lm	0		
>ref-112379	CGGGCGTGCACACATCCTCCCTACAAA	23	<nnxnp>	nn	128	np	135		
>ref-112392	TTCACACAGACGGACTCTCCCAGCTGT	2	<nnxnp>	nn	247	np	1		
>ref-112392	TTCACACAGACGGACTCTCCCAGCTGT	3	<nnxnp>	nn	249	np	0		
>ref-112399	ACTCGGAGAACAGAATCTCCAGTGACA	13	<hkxhk>	hh	0	hk	273	kk	0
>ref-112414	TCATCAAGAACATCATCTCCATTAGCA	26	<lmxll>	ll	130	lm	96		
>ref-112428	GGCCATTGGACGGAGACTCCCGCCCTT	2	<lmxll>	ll	128	lm	121		
>ref-112430	ATTATCCTGACGGCCTCTCCTCGAGCT	12	<nnxnp>	nn	141	np	118		
>ref-112430	ATTATCCTGACGGCCTCTCCTCGAGCT	22	<hkxhk>	hh	80	hk	187	kk	0
>ref-112430	ATTATCCTGACGGCCTCTCCTCGAGCT	23	<nnxnp>	nn	146	np	119		

>ref-112462	CGTACCCTTACCACCTCTCCTCCTGCT	2	<lmxll>	ll	266	lm	2		
>ref-112494	CTGTCTCCTACTCTCTCTCCGTCCTTT	4	<hkxhk>	hh	60	hk	140	kk	62
>ref-112499	TCTTTCCCCACACCCTCTCCTTCTTCC	14	<nnxnp>	nn	110	np	134		
>ref-112499	TCTTTCCCCACACCCTCTCCTTCTTCC	23	<nnxnp>	nn	109	np	134		
>ref-112501	ACACATCCCACTCTATCTCCCTCTCCC	1	<nnxnp>	nn	119	np	119		
>ref-112501	ACACATCCCACTCTATCTCCCTCTCCC	3	<nnxnp>	nn	124	np	117		
>ref-112501	ACACATCCCACTCTATCTCCCTCTCCC	5	<nnxnp>	nn	258	np	0		
>ref-112507	CTGACCCTGACACTGACTCCGTAAATC	9	<nnxnp>	nn	256	np	2		
>ref-112537	TAGAAACACACACGCGCTCCCTTAGAC	4	<nnxnp>	nn	127	np	141		
>ref-112553	AATACTAAGACCACAACTCCGTTGCAT	2	<lmxll>	ll	121	lm	149		
>ref-112575	TACACTCGCACTGTTCCTCCTCTCTCT	3	<lmxll>	ll	111	lm	148		
>ref-112585	CACATCACAACCCCCCCTCCCCCCATT	8	<hkxhk>	hh	2	hk	214	kk	7
>ref-112585	CACATCACAACCCCCCCTCCCCCCATT	26	<hkxhk>	hh	7	hk	214	kk	2
>ref-112586	ATGTTGTTTACCTCCTCTCCCATAGAA	6	<lmxll>	ll	38	lm	191		
>ref-112586	ATGTTGTTTACCTCCTCTCCCATAGAA	14	<lmxll>	ll	38	lm	191		
>ref-112586	ATGTTGTTTACCTCCTCTCCCATAGAA	21	<hkxhk>	hh	0	hk	180	kk	54
>ref-112586	ATGTTGTTTACCTCCTCTCCCATAGAA	25	<hkxhk>	hh	56	hk	179	kk	0
>ref-112588	CATCCGCAAACCTCTTCTCCTGCCTAA	8	<nnxnp>	nn	139	np	128		
>ref-112634	AACCCCCTCACCCCTTCTCCTTCCCTC	1	<lmxll>	ll	1	lm	263		
>ref-112634	AACCCCCTCACCCCTTCTCCTTCCCTC	21	<lmxll>	ll	1	lm	263		
>ref-112655	CTCACCATTACTCTGCCTCCTCCCTCA	15	<hkxhk>	hh	0	hk	267	kk	1
>ref-112658	CATTGCCAAACAAAGCCTCCCTAACTG	26	<hkxhk>	hh	31	hk	114	kk	85
>ref-112662	ATTCCCTTCACGTAACCTCCTTTCCCT	15	<hkxhk>	hh	68	hk	142	kk	45
>ref-112681	TAACCATTAACAGTTTCTCCTCTTGGT	3	<nnxnp>	nn	130	np	122		
>ref-112687	CTCGGGCCCACACTTTCTCCCGCAACC	24	<hkxhk>	hh	4	hk	269	kk	0
>ref-112687	CTCGGGCCCACACTTTCTCCCGCAACC	25	<hkxhk>	hh	4	hk	269	kk	0
>ref-112697	TTAATGTGTACAATAGCTCCCCTCACC	9	<nnxnp>	nn	137	np	107		
>ref-112698	ACTCCCACAACCAGCTCTCCCGAAGAA	6	<nnxnp>	nn	14	np	240		
>ref-112698	ACTCCCACAACCAGCTCTCCCGAAGAA	21	<nnxnp>	nn	14	np	240		
>ref-112704	CGATCTCCTACTGCATCTCCTCCCTTT	2	<nnxnp>	nn	112	np	139		
>ref-112704	CGATCTCCTACTGCATCTCCTCCCTTT	24	<nnxnp>	nn	112	np	139		
>ref-112736	TCTTTTCTCACCCTCACTCCCCATCTC	9	<lmxll>	ll	259	lm	0		
>ref-112743	TTCTTCTTCACCCTAACTCCCACCTTT	5	<lmxll>	ll	103	lm	155		
>ref-112743	TTCTTCTTCACCCTAACTCCCACCTTT	9	<lmxll>	ll	102	lm	155		
>ref-112746	GATCTGCTGACACTTTCTCCCATGTCA	9	<nnxnp>	nn	268	np	0		
>ref-112756	TGCGCTGTCACATGATCTCCAGGTATT	6	<hkxhk>	hh	0	hk	259	kk	8
>ref-112756	TGCGCTGTCACATGATCTCCAGGTATT	14	<hkxhk>	hh	0	hk	259	kk	8
>ref-112758	TACATATACACCTCCCCTCCCCACACA	6	<lmxll>	ll	207	lm	42		

>ref-112758	TACATATACACCTCCCCTCCCCACACA	13	<lmxll>	ll	132	lm	116		
>ref-112767	AAGCCTGTTACGTCATCTCCATTTTCA	27	<hkxhk>	hh	52	hk	131	kk	59
>ref-112782	GATCACTCGACTTAATCTCCTTCCCGC	22	<lmxll>	ll	106	lm	144		
>ref-112789	CTCTCTGGCACAAGTTCTCCGTCTTTC	3	<lmxll>	ll	161	lm	79		
>ref-112789	CTCTCTGGCACAAGTTCTCCGTCTTTC	5	<lmxll>	ll	160	lm	79		
>ref-112793	TACTTAATTACATCGTCTCCTAAGGTA	22	<nnxnp>	nn	105	np	134		
>ref-112815	TCTCTCTTCACCTTATCTCCGTCACTA	8	<nnxnp>	nn	114	np	143		
>ref-112837	TATCATGTCACAGTGACTCCTCCATAG	12	<hkxhk>	hh	26	hk	234	kk	0
>ref-112837	TATCATGTCACAGTGACTCCTCCATAG	22	<nnxnp>	nn	107	np	152		
>ref-112837	TATCATGTCACAGTGACTCCTCCATAG	27	<hkxhk>	hh	3	hk	266	kk	0
>ref-112886	TCTGTACACACGTCTCCTCCATCCGGA	25	<lmxll>	ll	146	lm	124		
>ref-112889	AGCAAACAAACGCCCACTCCGACTTGC	21	<hkxhk>	hh	0	hk	273	kk	0
>ref-112889	AGCAAACAAACGCCCACTCCGACTTGC	26	<hkxhk>	hh	44	hk	218	kk	0
>ref-112890	CTGATATTTACAATGACTCCTTCATAA	13	<lmxll>	ll	105	lm	145		
>ref-112896	TCCCTCTCCACCCTCCCTCCCTTTTCA	24	<lmxll>	ll	128	lm	116		
>ref-112896	TCCCTCTCCACCCTCCCTCCCTTTTCA	25	<nnxnp>	nn	256	np	3		
>ref-112926	GTTAATCACACTCTTGCTCCCCCTCCT	23	<lmxll>	ll	270	lm	0		
>ref-112933	CGTCCGCTAACCACCCCTCCGTCCGTC	8	<nnxnp>	nn	263	np	0		
>ref-112982	TTCGCCTCTACATCACCTCCCTTTTCT	8	<lmxll>	ll	113	lm	130		
>ref-112983	TCACTAACCACCTCCCCTCCTCTCCTC	3	<nnxnp>	nn	123	np	128		
>ref-112983	TCACTAACCACCTCCCCTCCTCTCCTC	24	<nnxnp>	nn	122	np	128		
>ref-112992	GATGTACCGACTCTCTCTCCCTCGTAT	13	<nnxnp>	nn	118	np	151		
>ref-112997	AAAGTAATTACAGATCCTCCACTAGCT	8	<hkxhk>	hh	0	hk	269	kk	3
>ref-112997	AAAGTAATTACAGATCCTCCACTAGCT	26	<hkxhk>	hh	0	hk	269	kk	3
>ref-113000	TCTTATATCACATCTTCTCCTTCCTCC	23	<hkxhk>	hh	52	hk	145	kk	51
>ref-113007	TCCTTCCACACAATCTCTCCACCCTCG	27	<nnxnp>	nn	130	np	125		
>ref-113024	CCGCGCCAAACAAATCCTCCTCCTCTT	12	<nnxnp>	nn	124	np	137		
>ref-113034	AAGCTTTATACGACCTCTCCCTCGCCC	16	<hkxhk>	hh	66	hk	142	kk	58
>ref-113034	AAGCTTTATACGACCTCTCCCTCGCCC	26	<nnxnp>	nn	143	np	120		
>ref-113062	GCTCCTCCTACCCCCACTCCCAAGCCG	1	<nnxnp>	nn	269	np	0		
>ref-113067	CAACCTCCAACCTCTTCTCCTGTCATT	21	<lmxll>	ll	135	lm	131		
>ref-113109	CACACACACACCATACCTCCTTTCTTC	21	<lmxll>	ll	135	lm	122		
>ref-113168	TTCCTTCACACTCACCCTCCATCCCTC	4	<lmxll>	ll	134	lm	128		
>ref-113168	TTCCTTCACACTCACCCTCCATCCCTC	21	<lmxll>	ll	258	lm	1		
>ref-113193	CAGGTAAGCACCAGTACTCCTCCAGCA	4	<hkxhk>	hh	0	hk	262	kk	9
>ref-113193	CAGGTAAGCACCAGTACTCCTCCAGCA	22	<nnxnp>	nn	178	np	85		
>ref-113200	TTAATATTGACAATTTCTCCCTTTCCT	21	<lmxll>	ll	122	lm	126		
>ref-113247	TCTGCACGAACCCGATCTCCTTATACT	4	<nnxnp>	nn	141	np	120		

>ref-113296	CCATATTTCACGTAATCTCCTCTGTAT	6	<hkxhk>	hh	56	hk	145	kk	38
>ref-113309	ACTCTTGTTACCTAATCTCCTGTGCAT	4	<nnxnp>	nn	152	np	104		
>ref-113309	ACTCTTGTTACCTAATCTCCTGTGCAT	21	<nnxnp>	nn	151	np	105		
>ref-113316	CGTGAATATACCGTTACTCCTTCTTTG	3	<nnxnp>	nn	115	np	109		
>ref-113316	CGTGAATATACCGTTACTCCTTCTTTG	13	<lmxll>	ll	117	lm	102		
>ref-113321	TCCCTTATTACCCCCTCTCCCTCACCA	2	<lmxll>	ll	137	lm	130		
>ref-113324	CCGTTGTGCACATGTTCTCCTTCATAG	3	<hkxhk>	hh	46	hk	133	kk	53
>ref-113358	CCTCCTCCCACGCACCCTCCCAGCCTC	15	<hkxhk>	hh	13	hk	236	kk	7
>ref-113358	CCTCCTCCCACGCACCCTCCCAGCCTC	22	<hkxhk>	hh	7	hk	237	kk	10
>ref-113359	TCCTCTTTCACTTTCCCTCCTCCTTCC	21	<lmxll>	ll	4	lm	217		
>ref-113367	GTAACATGTACATAATCTCCTCGTGCA	9	<hkxhk>	hh	59	hk	149	kk	48
>ref-113370	AGAAGTGCCACACCATCTCCTCTCTTA	12	<hkxhk>	hh	64	hk	141	kk	58
>ref-113371	TGGATCAAAACAATATCTCCCAGCACC	6	<hkxhk>	hh	34	hk	140	kk	68
>ref-113376	TACACTCTCACAAACGCTCCATACCCC	16	<nnxnp>	nn	136	np	126		
>ref-113384	CATTCGACCACTCACCCTCCCTCTCCC	6	<lmxll>	ll	145	lm	126		
>ref-113384	CATTCGACCACTCACCCTCCCTCTCCC	7	<lmxll>	ll	145	lm	126		
>ref-113410	CTGTCTGCCACCCTCTCTCCCTTTCTA	12	<lmxll>	ll	128	lm	142		
>ref-113450	TCACGCCTCACCCCCCCTCCATCTCTC	6	<nnxnp>	nn	269	np	0		
>ref-113472	GAGTAGTAAACAGATACTCCTCCTCCT	23	<lmxll>	ll	225	lm	0		
>ref-113514	AATTCTCCCACGCTCTCTCCTCCAACA	6	<lmxll>	ll	31	lm	222		
>ref-113514	AATTCTCCCACGCTCTCTCCTCCAACA	12	<lmxll>	ll	30	lm	222		
>ref-113521	GTGGCGACTACAGTGACTCCTTTGCTG	3	<nnxnp>	nn	154	np	118		
>ref-113531	CCTCCAACAACTCGTCCTCCTCCTTCG	1	<lmxll>	ll	124	lm	149		
>ref-113531	CCTCCAACAACTCGTCCTCCTCCTTCG	16	<hkxhk>	hh	220	hk	42	kk	0
>ref-113531	CCTCCAACAACTCGTCCTCCTCCTTCG	21	<hkxhk>	hh	0	hk	271	kk	2
>ref-113626	TACATGGTTACCTTTTCTCCATTTATG	9	<lmxll>	ll	224	lm	0		
>ref-113643	GACTTTGTGACAGGCGCTCCTGCACAT	12	<lmxll>	ll	127	lm	129		
>ref-113643	GACTTTGTGACAGGCGCTCCTGCACAT	26	<lmxll>	ll	124	lm	129		
>ref-113653	GTACACCGCACAAGGACTCCTTTAACA	8	<nnxnp>	nn	157	np	109		
>ref-113666	TGGCATCTCACCTCACCTCCAAATCCC	5	<nnxnp>	nn	267	np	0		
>ref-113697	ATACACTCTACACTCTCTCCCTCACTC	13	<nnxnp>	nn	113	np	137		
>ref-113761	TTCTCGCCAACCTCTCCTCCTCATCTC	23	<nnxnp>	nn	265	np	0		
>ref-113763	CCTATCCACACATCCTCTCCTCCTCTT	25	<hkxhk>	hh	27	hk	192	kk	24
>ref-113766	TACTTCAATACTAGCACTCCTTGCAAC	13	<hkxhk>	hh	59	hk	138	kk	62
>ref-113778	AATACACACACCTCATCTCCTCCTATC	16	<nnxnp>	nn	125	np	136		
>ref-113782	CATTAGCTTACACCGCCTCCACGTACT	1	<lmxll>	ll	111	lm	123		
>ref-113806	CTCCCCATCACCTCTTCTCCTCCCCTG	27	<lmxll>	ll	113	lm	106		
>ref-113820	CGGCTAACAACGTCCGCTCCTTCGTTA	4	<nnxnp>	nn	132	np	138		

>ref-113882	CCTTGATTCACTCTCCCTCCTCTCCTT	7	<lmxll>	ll	161	lm	107		
>ref-113882	CCTTGATTCACTCTCCCTCCTCTCCTT	24	<lmxll>	ll	160	lm	108		
>ref-113892	ATAGACAGAACCCACACTCCTCTCATC	27	<nnxnp>	nn	249	np	16		
>ref-113987	CCCTTTGCAACGTGCACTCCATGTTGA	2	<nnxnp>	nn	264	np	0		
>ref-113988	CCCACACGCACGCGCGCTCCCCTCCCC	24	<lmxll>	ll	122	lm	136		
>ref-114013	AACCAAACCACCATCACTCCATCACCA	25	<nnxnp>	nn	113	np	135		
>ref-114017	CTAACCTCTACACTCTCTCCCTAACCC	7	<nnxnp>	nn	138	np	99		
>ref-114017	CTAACCTCTACACTCTCTCCCTAACCC	8	<nnxnp>	nn	138	np	99		
>ref-114044	CCCTCTGCCACTTTCACTCCCGCTCCG	1	<nnxnp>	nn	217	np	19		
>ref-114049	AGGAAACACACAGACCCTCCCTGACAC	14	<hkxhk>	hh	119	hk	146	kk	2
>ref-114065	ATTTAATCTACTTCGCCTCCTTATCAT	1	<nnxnp>	nn	135	np	110		
>ref-114086	GGCTTCCAGACCTACACTCCGGGATAG	4	<hkxhk>	hh	0	hk	273	kk	0
>ref-114086	GGCTTCCAGACCTACACTCCGGGATAG	9	<hkxhk>	hh	0	hk	273	kk	0
>ref-114086	GGCTTCCAGACCTACACTCCGGGATAG	15	<lmxll>	ll	126	lm	140		
>ref-114094	TCTCCAGGGACCCTCCCTCCCTCTCTC	9	<nnxnp>	nn	7	np	252		
>ref-114094	TCTCCAGGGACCCTCCCTCCCTCTCTC	23	<nnxnp>	nn	7	np	252		
>ref-114096	CCTTCTCTTACCTCCCCTCCCTTTCTC	9	<lmxll>	ll	0	lm	271		
>ref-114096	CCTTCTCTTACCTCCCCTCCCTTTCTC	13	<lmxll>	ll	96	lm	151		
>ref-114098	CCTTCCCCAACCTCCCCTCCCTCACCT	14	<lmxll>	ll	98	lm	154		
>ref-114100	CCATCCCCTACCTCATCTCCCTTCCCC	7	<nnxnp>	nn	166	np	94		
>ref-114100	CCATCCCCTACCTCATCTCCCTTCCCC	13	<nnxnp>	nn	204	np	55		
>ref-114100	CCATCCCCTACCTCATCTCCCTTCCCC	22	<nnxnp>	nn	165	np	96		
>ref-114104	CCTAATCTTACCCACCCTCCTCTTACC	2	<hkxhk>	hh	63	hk	176	kk	4
>ref-114104	CCTAATCTTACCCACCCTCCTCTTACC	21	<nnxnp>	nn	137	np	86		
>ref-114104	CCTAATCTTACCCACCCTCCTCTTACC	22	<nnxnp>	nn	137	np	86		
>ref-114123	TTACACTGGACTTTCCCTCCTTCGAAT	12	<lmxll>	ll	131	lm	134		
>ref-114123	TTACACTGGACTTTCCCTCCTTCGAAT	14	<lmxll>	ll	123	lm	141		
>ref-114123	TTACACTGGACTTTCCCTCCTTCGAAT	15	<lmxll>	ll	123	lm	141		
>ref-114144	AAGAGCTTTACGCTCTCTCCTGGCAAG	9	<hkxhk>	hh	0	hk	268	kk	0
>ref-114162	TTTGATTGCACCGCGGCTCCTTTCGCT	15	<hkxhk>	hh	70	hk	144	kk	53
>ref-114176	GTCCTCCCCACTCTCTCTCCCGTCTCT	27	<lmxll>	ll	125	lm	138		
>ref-114182	CGATATATCACACCCACTCCTCTCGCA	6	<lmxll>	ll	106	lm	135		
>ref-114186	GCCCCCTCTACCTCTCCTCCTATCCAT	1	<hkxhk>	hh	0	hk	261	kk	3
>ref-114186	GCCCCCTCTACCTCTCCTCCTATCCAT	2	<hkxhk>	hh	3	hk	263	kk	0
>ref-114188	TCACTGATAACATGAACTCCGGTGTCG	6	<lmxll>	ll	147	lm	105		
>ref-114195	TGCCCTGGAACCCCTACTCCCTCTCCC	2	<nnxnp>	nn	100	np	131		
>ref-114195	TGCCCTGGAACCCCTACTCCCTCTCCC	4	<hkxhk>	hh	3	hk	265	kk	0
>ref-114195	TGCCCTGGAACCCCTACTCCCTCTCCC	8	<nnxnp>	nn	150	np	117		

>ref-114204	TTTGTAACTACTTGTCCTCCTTCCCTA	1	<nnxnp>	nn	131	np	135		
>ref-114223	GTGACCATAACAACAGCTCCCACCCTT	15	<lmxll>	ll	111	lm	124		
>ref-114234	GAGAGAGAGACAGAACCTCCACCTCCA	24	<hkxhk>	hh	2	hk	259	kk	7
>ref-114234	GAGAGAGAGACAGAACCTCCACCTCCA	27	<hkxhk>	hh	7	hk	259	kk	2
>ref-114242	CTAATGGGCACCCAACCTCCCCAGTGT	1	<nnxnp>	nn	252	np	0		
>ref-114273	GACCTGTGAACGCCTCCTCCCCCTCTG	15	<nnxnp>	nn	125	np	136		
>ref-114273	GACCTGTGAACGCCTCCTCCCCCTCTG	21	<nnxnp>	nn	125	np	136		
>ref-114292	TTACCGCCAACTCCTCCTCCTTCTTCT	26	<lmxll>	ll	186	lm	69		
>ref-114299	CTCTCCACAACCTTCCCTCCTCGCCCT	12	<nnxnp>	nn	135	np	137		
>ref-114340	TCAACACACACGCTGTCTCCTTGCAAT	12	<hkxhk>	hh	0	hk	271	kk	0
>ref-114353	TTTGACTTCACTGACGCTCCTTGACTT	4	<lmxll>	ll	133	lm	137		
>ref-114362	ACCCCTCCCACACCCACTCCCCTACCT	2	<nnxnp>	nn	131	np	134		
>ref-114383	CTCCCCATCACTCTTCCTCCCCTCCCC	25	<lmxll>	ll	123	lm	137		
>ref-114436	CCTGCAATCACTCCACCTCCTCCACCT	24	<hkxhk>	hh	57	hk	214	kk	0
>ref-114464	GTGTTGATGACTGTGCCTCCGAGTATC	13	<hkxhk>	hh	64	hk	140	kk	46
>ref-114471	AGGCACACGACCCCCTCTCCCCTCCCC	22	<hkxhk>	hh	26	hk	229	kk	0
>ref-114471	AGGCACACGACCCCCTCTCCCCTCCCC	23	<hkxhk>	hh	0	hk	259	kk	9
>ref-114471	AGGCACACGACCCCCTCTCCCCTCCCC	25	<nnxnp>	nn	168	np	96		
>ref-114477	CCATCCTTCACCCTTCCTCCTATCCAT	9	<nnxnp>	nn	128	np	142		
>ref-114491	CCTCGGCTGACCTTGGCTCCTCCTCGA	24	<lmxll>	ll	149	lm	95		
>ref-114491	CCTCGGCTGACCTTGGCTCCTCCTCGA	27	<lmxll>	ll	144	lm	95		
>ref-114501	ATATCACAAACCACCTCTCCTAAAGCT	13	<hkxhk>	hh	58	hk	117	kk	52
>ref-114502	ACACAGAACACGGCGTCTCCAATACTG	25	<hkxhk>	hh	79	hk	129	kk	14
>ref-114508	GCCCCAACAACTTTCTCTCCTACTACT	22	<lmxll>	ll	104	lm	147		
>ref-114515	CACTCACCCACTCACACTCCACAAGAT	4	<nnxnp>	nn	237	np	0		
>ref-114515	CACTCACCCACTCACACTCCACAAGAT	12	<nnxnp>	nn	236	np	0		
>ref-114542	GCACTTTGCACACTTACTCCATCTGCC	15	<lmxll>	ll	118	lm	148		
>ref-114556	CTCAACCACACCTTATCTCCCTCAACC	6	<hkxhk>	hh	2	hk	246	kk	13
>ref-114558	TCTTTCTTAACCTCTCCTCCCCCCTCT	6	<hkxhk>	hh	5	hk	254	kk	0
>ref-114558	TCTTTCTTAACCTCTCCTCCCCCCTCT	7	<hkxhk>	hh	2	hk	260	kk	1
>ref-114558	TCTTTCTTAACCTCTCCTCCCCCCTCT	9	<hkxhk>	hh	1	hk	260	kk	2
>ref-114558	TCTTTCTTAACCTCTCCTCCCCCCTCT	23	<hkxhk>	hh	5	hk	254	kk	0
>ref-114559	TCAGCAGTGACGGGTGCTCCTGCAACA	4	<nnxnp>	nn	134	np	138		
>ref-114559	TCAGCAGTGACGGGTGCTCCTGCAACA	14	<hkxhk>	hh	0	hk	273	kk	0
>ref-114559	TCAGCAGTGACGGGTGCTCCTGCAACA	24	<hkxhk>	hh	1	hk	271	kk	0
>ref-114583	CCTGGCTCCACACCAACTCCTGCCCCA	9	<hkxhk>	hh	0	hk	272	kk	1
>ref-114598	CCCTTTAGTACTCGTTCTCCCTCCCTC	26	<lmxll>	ll	129	lm	123		
>ref-114611	TTCGTTAATACCGTGCCTCCTTTTTTA	9	<lmxll>	ll	113	lm	113		

>ref-114639	GTATCTTTCACCCGTCCTCCTCCTCCT	6	<nnxnp>	nn	6	np	261		
>ref-114639	GTATCTTTCACCCGTCCTCCTCCTCCT	8	<nnxnp>	nn	6	np	261		
>ref-114640	TCAACAAACACAACAGCTCCAAAGCCT	2	<nnxnp>	nn	270	np	1		
>ref-114641	CCTTCACCTACTCTTCCTCCCTCTTTA	15	<lmxll>	ll	189	lm	60		
>ref-114686	CAACGGGTTACTTGTCCTCCTCTACAG	15	<lmxll>	ll	125	lm	135		
>ref-114692	ATCTCCCTTACCCCGCCTCCACTCTCC	5	<lmxll>	ll	171	lm	82		
>ref-114695	TTTCTATCTACTCTATCTCCTATCTGC	3	<nnxnp>	nn	158	np	102		
>ref-114703	GCTACCCCTACACTCACTCCCCGCCAT	26	<hkxhk>	hh	57	hk	153	kk	53
>ref-114705	CATCCATGCACGCACTCTCCTCTCTCT	6	<lmxll>	ll	134	lm	135		
>ref-114705	CATCCATGCACGCACTCTCCTCTCTCT	22	<lmxll>	ll	134	lm	135		
>ref-114730	TATAGTGCCACTTGTACTCCGGACTAA	24	<hkxhk>	hh	71	hk	120	kk	43
>ref-114731	AGTGGGGACACGTCCTCTCCACATTAT	1	<hkxhk>	hh	1	hk	232	kk	0
>ref-114731	AGTGGGGACACGTCCTCTCCACATTAT	22	<hkxhk>	hh	92	hk	169	kk	0
>ref-114731	AGTGGGGACACGTCCTCTCCACATTAT	23	<hkxhk>	hh	181	hk	86	kk	0
>ref-114731	AGTGGGGACACGTCCTCTCCACATTAT	24	<hkxhk>	hh	0	hk	87	kk	179
>ref-114731	AGTGGGGACACGTCCTCTCCACATTAT	25	<hkxhk>	hh	0	hk	273	kk	0
>ref-114743	CCCCCCGTAACATATGCTCCTCCAAGC	4	<nnxnp>	nn	109	np	115		
>ref-114747	CATCTCGTCACTTGATCTCCAGACCAT	1	<nnxnp>	nn	132	np	126		
>ref-114764	TACTCACTCACTACCTCTCCCTATCCC	13	<lmxll>	ll	220	lm	10		
>ref-114764	TACTCACTCACTACCTCTCCCTATCCC	23	<lmxll>	ll	222	lm	12		
>ref-114775	CGTACTGTGACGTCATCTCCGGATCCA	9	<nnxnp>	nn	96	np	153		
>ref-114776	CACGAAATAACCTAAACTCCTGATACA	26	<lmxll>	ll	103	lm	130		
>ref-114782	CTTACTCCTACAGTTTCTCCTCTTCCT	15	<nnxnp>	nn	108	np	132		
>ref-114799	AAAACAGGAACTTAATCTCCCAATTAC	1	<lmxll>	ll	119	lm	108		
>ref-114799	AAAACAGGAACTTAATCTCCCAATTAC	2	<lmxll>	ll	117	lm	109		
>ref-114815	TGACCTCGTACTGTAGCTCCGACTATC	1	<hkxhk>	hh	58	hk	133	kk	57
>ref-114822	GACACGCCCACAGATCCTCCCACAGAC	25	<hkxhk>	hh	0	hk	260	kk	5
>ref-114822	GACACGCCCACAGATCCTCCCACAGAC	26	<hkxhk>	hh	6	hk	258	kk	0
>ref-114822	GACACGCCCACAGATCCTCCCACAGAC	27	<nnxnp>	nn	251	np	12		
>ref-114863	GTTTTCCTTACTCGGTCTCCTCTCTCT	23	<lmxll>	ll	111	lm	141		
>ref-114872	TTTTATCACACAGTTCCTCCGTGCCTC	21	<lmxll>	ll	118	lm	133		
>ref-114895	TACACTCCAACCCACCCTCCTCCTTCA	14	<nnxnp>	nn	109	np	123		
>ref-114896	TCCCATCTGACACTCCCTCCCTTGCTT	7	<lmxll>	ll	113	lm	146		
>ref-114914	TTCTCATTTACGCCACCTCCCCCATCC	24	<lmxll>	ll	128	lm	126		
>ref-114917	CCATTCCCCACGTCCTCTCCCCTCCTT	6	<nnxnp>	nn	140	np	123		
>ref-114936	CACACCAGAACAGAGCCTCCTGCCTTG	6	<lmxll>	ll	138	lm	127		
>ref-114938	CGCTTAAGAACTTCCTCTCCCTGATGC	1	<nnxnp>	nn	121	np	142		
>ref-114940	TCTGCCCTTACCTGCCCTCCGTTCCCT	2	<hkxhk>	hh	51	hk	162	kk	46

>ref-114951	TATCCCTCCACCGTACCTCCACCACAA	16	<lmxll>	ll	134	lm	128		
>ref-114973	CCTTTCACCACCTCATCTCCTTTTCGT	24	<lmxll>	ll	100	lm	122		
>ref-114989	CCCACTCCCACTACCTCTCCTTCTTTC	4	<hkxhk>	hh	0	hk	268	kk	0
>ref-114989	CCCACTCCCACTACCTCTCCTTCTTTC	25	<hkxhk>	hh	0	hk	65	kk	200
>ref-114999	TCGTCTGCTACCCTCTCTCCTTGCTTC	5	<nnxnp>	nn	135	np	128		
>ref-115002	TTGCCTTTCACTCCTCCTCCTCGACCT	9	<lmxll>	ll	135	lm	123		
>ref-115020	TCGGTTCACACAACAGCTCCATAATTC	21	<lmxll>	ll	112	lm	132		
>ref-115024	TATGAAGTGACGACATCTCCAGCGTTC	21	<nnxnp>	nn	130	np	140		
>ref-115044	GACGCTAGAACGGGAGCTCCTTTGGCC	25	<lmxll>	ll	130	lm	138		
>ref-115052	ACTTCTTCCACTATTCCTCCTTTCTCT	21	<nnxnp>	nn	143	np	119		
>ref-115074	CTAATACACACTTATACTCCACCCCCT	22	<nnxnp>	nn	102	np	138		
>ref-115087	TACGCTTCCACTTCTTCTCCCCCTTTT	6	<lmxll>	ll	128	lm	139		
>ref-115090	TCTCCCTCCACCCTTTCTCCCCCAATC	5	<hkxhk>	hh	2	hk	175	kk	91
>ref-115090	TCTCCCTCCACCCTTTCTCCCCCAATC	25	<hkxhk>	hh	2	hk	172	kk	93
>ref-115102	CCTCCTCCTACTCCAGCTCCCTCCCCA	4	<hkxhk>	hh	0	hk	60	kk	201
>ref-115107	TCGTATCCCACTATCCCTCCTCCTCTC	24	<nnxnp>	nn	119	np	136		
>ref-115115	CTCTCACTAACATTCACTCCTTCCCTT	8	<lmxll>	ll	136	lm	131		
>ref-115121	CGTAGACACACACATCCTCCACATGCA	22	<lmxll>	ll	130	lm	135		
>ref-115127	CTTTAGTTTACCCATCCTCCCTCTTTC	7	<lmxll>	ll	111	lm	130		
>ref-115127	CTTTAGTTTACCCATCCTCCCTCTTTC	13	<lmxll>	ll	110	lm	130		
>ref-115133	CTTTCTTACACCCTCCCTCCCACACTC	12	<hkxhk>	hh	4	hk	260	kk	0
>ref-115133	CTTTCTTACACCCTCCCTCCCACACTC	26	<hkxhk>	hh	0	hk	257	kk	4
>ref-115164	AATTGGTTTACAGGCTCTCCCAGCGCC	12	<nnxnp>	nn	254	np	0		
>ref-115210	CCTCCTTTTACTCTCCCTCCATGCCTA	1	<nnxnp>	nn	116	np	145		
>ref-115228	CCCTTTCCAACTTTCCCTCCTAACTTT	8	<lmxll>	ll	145	lm	91		
>ref-115237	CAATCTTTCACTCTCCCTCCCCTTCTT	16	<nnxnp>	nn	115	np	125		
>ref-115237	CAATCTTTCACTCTCCCTCCCCTTCTT	25	<nnxnp>	nn	115	np	125		
>ref-115271	CCAACACCTACACCCACTCCCTCCCCT	4	<lmxll>	ll	63	lm	195		
>ref-115274	AGAACAGCCACAGCTCCTCCTAACAGA	1	<lmxll>	ll	137	lm	118		
>ref-115280	GTCTGTCCCACTCTCCCTCCCCGCAGC	23	<nnxnp>	nn	130	np	131		
>ref-115287	TCACAGACCACCTCTACTCCTCTCCAC	2	<nnxnp>	nn	147	np	124		
>ref-115309	AGCAAACAAACACAACCTCCTCGACAC	25	<nnxnp>	nn	261	np	0		
>ref-115311	ACATACCTCACGCGCTCTCCCTCTCGC	4	<nnxnp>	nn	106	np	147		
>ref-115316	CCGACCCTCACCCTCCCTCCCTGCCCC	22	<lmxll>	ll	137	lm	97		
>ref-115316	CCGACCCTCACCCTCCCTCCCTGCCCC	23	<lmxll>	ll	116	lm	103		
>ref-115360	GTATCCTGTACCTTCTCTCCTGTCACT	5	<lmxll>	ll	133	lm	131		
>ref-115379	CATTATTACACATATCCTCCCTCTCAT	12	<lmxll>	ll	114	lm	115		
>ref-115392	GTACTCCTTACATCCCCTCCTCCTCCT	6	<lmxll>	ll	38	lm	217		

>ref-115392	GTACTCCTTACATCCCCTCCTCCTCCT	25	<lmxll>	ll	40	lm	216		
>ref-115407	TGAGATCACACCCTCCCTCCCCTCCTT	25	<nnxnp>	nn	131	np	126		
>ref-115433	AATGAAGCAACCTCCTCTCCTGGGAAT	3	<hkxhk>	hh	4	hk	256	kk	1
>ref-115433	AATGAAGCAACCTCCTCTCCTGGGAAT	4	<hkxhk>	hh	4	hk	256	kk	1
>ref-115442	TGTATATAAACTGTCCCTCCGTGTTAT	15	<hkxhk>	hh	59	hk	137	kk	50
>ref-115443	ATTTCCACCACATGCCCTCCTCCTCCT	14	<hkxhk>	hh	46	hk	207	kk	2
>ref-115451	CTACCGTCAACCATTTCTCCAGCTCAG	6	<nnxnp>	nn	146	np	108		
>ref-115454	TGAGAGGCTACGTCGACTCCGTGGATG	14	<nnxnp>	nn	129	np	142		
>ref-115455	TTCCTTCAGACCCTACCTCCCCCTCCG	7	<hkxhk>	hh	40	hk	144	kk	62
>ref-115458	ACCGGACACACTCACTCTCCATCTCCA	14	<hkxhk>	hh	70	hk	152	kk	44
>ref-115459	AACAGGGCGACACCCTCTCCCAAAACT	13	<hkxhk>	hh	0	hk	217	kk	14
>ref-115459	AACAGGGCGACACCCTCTCCCAAAACT	15	<hkxhk>	hh	14	hk	217	kk	0
>ref-115470	AGGATTTTCACCAAGCCTCCTTAACCG	1	<nnxnp>	nn	128	np	128		
>ref-115470	AGGATTTTCACCAAGCCTCCTTAACCG	2	<lmxll>	ll	122	lm	132		
>ref-115474	ACGTTGCCCACAATCACTCCGCGCCCC	27	<nnxnp>	nn	139	np	120		
>ref-115493	CCCTTTAACACACACTCTCCTTAGAAA	5	<hkxhk>	hh	36	hk	134	kk	62
>ref-115493	CCCTTTAACACACACTCTCCTTAGAAA	27	<hkxhk>	hh	62	hk	133	kk	35
>ref-115517	TCTCCTACAACTCCTACTCCTCCCTCC	8	<lmxll>	ll	105	lm	128		
>ref-115529	AAGCAAAGCACACAATCTCCCTGTAAT	1	<lmxll>	ll	153	lm	105		
>ref-115530	ACCTACAACACTCCATCTCCAGCAGGT	7	<lmxll>	ll	145	lm	127		
>ref-115609	CCTGGTGCTACAAGTCCTCCTCTTGGC	26	<nnxnp>	nn	267	np	0		
>ref-115653	AACGAAATAACCTCGGCTCCGTTCCAC	4	<lmxll>	ll	122	lm	136		
>ref-115653	AACGAAATAACCTCGGCTCCGTTCCAC	9	<lmxll>	ll	131	lm	131		
>ref-115667	TGCAACGTCACGCTCTCTCCATTGCTC	26	<lmxll>	ll	137	lm	134		
>ref-115674	CTCACAGTGACACGATCTCCTTCGCTC	27	<hkxhk>	hh	32	hk	115	kk	78
>ref-115676	GACTGGTTAACTGGTTCTCCTTTCATC	4	<nnxnp>	nn	135	np	134		
>ref-115676	GACTGGTTAACTGGTTCTCCTTTCATC	5	<nnxnp>	nn	134	np	134		
>ref-115696	CCCTGCTCCACCACCCCTCCAGCCTTC	4	<hkxhk>	hh	0	hk	259	kk	7
>ref-115696	CCCTGCTCCACCACCCCTCCAGCCTTC	25	<hkxhk>	hh	0	hk	259	kk	7
>ref-115740	CTTAGTGTGACCTGACCTCCGTGTAGT	24	<hkxhk>	hh	62	hk	140	kk	46
>ref-115745	TATGAACCGACTAAAGCTCCTACGGTA	21	<nnxnp>	nn	141	np	130		
>ref-115756	TGTGATTCCACTCGTTCTCCCTTGCTT	16	<lmxll>	ll	126	lm	132		
>ref-115756	TGTGATTCCACTCGTTCTCCCTTGCTT	21	<hkxhk>	hh	74	hk	120	kk	64
>ref-115764	TCTTCCCCCACTCATTCTCCTCCTTCC	8	<lmxll>	ll	7	lm	260		
>ref-115764	TCTTCCCCCACTCATTCTCCTCCTTCC	21	<lmxll>	ll	50	lm	200		
>ref-115786	ATTTACTACACACCAGCTCCAGACACT	15	<hkxhk>	hh	116	hk	140	kk	0
>ref-115794	GTATTTGCGACCTTGCCTCCAAGCCGT	1	<lmxll>	ll	121	lm	149		
>ref-115805	GTGGCCCTGACCCCGCCTCCCCCGCTC	3	<lmxll>	ll	130	lm	141		

>ref-115829	GATTTCTAAACCGTCCCTCCTACCGAA	3	<lmxll>	ll	120	lm	139		
>ref-115846	TTTTCTCCCACACCATCTCCCTCCCTT	12	<nnxnp>	nn	242	np	0		
>ref-115846	TTTTCTCCCACACCATCTCCCTCCCTT	15	<hkxhk>	hh	56	hk	131	kk	39
>ref-115846	TTTTCTCCCACACCATCTCCCTCCCTT	25	<nnxnp>	nn	254	np	1		
>ref-115850	CCTCTCCCTACCTCCACTCCCCCCACT	25	<nnxnp>	nn	63	np	194		
>ref-115873	TGCTTGATTACTGTGTCTCCCGAGCAT	16	<nnxnp>	nn	120	np	120		
>ref-115876	CGGCTTCAAACCGGGGCTCCTTATCTC	1	<lmxll>	ll	151	lm	98		
>ref-115899	CACGGAAGCACACCAACTCCAACGCAA	2	<nnxnp>	nn	147	np	122		
>ref-115901	ACCGACTGCACAGGGTCTCCTGGATCT	8	<lmxll>	ll	103	lm	158		
>ref-115901	ACCGACTGCACAGGGTCTCCTGGATCT	9	<hkxhk>	hh	0	hk	221	kk	37
>ref-115901	ACCGACTGCACAGGGTCTCCTGGATCT	15	<hkxhk>	hh	36	hk	221	kk	0
>ref-115901	ACCGACTGCACAGGGTCTCCTGGATCT	22	<nnxnp>	nn	204	np	58		
>ref-115929	CTCACTCACACACTCCCTCCCTCTTCT	23	<lmxll>	ll	144	lm	113		
>ref-115929	CTCACTCACACACTCCCTCCCTCTTCT	26	<lmxll>	ll	142	lm	113		
>ref-115939	TATGCACGCACTGTTTCTCCTTTCTCT	6	<hkxhk>	hh	63	hk	146	kk	50
>ref-115956	TCACCTCAAACACTCCCTCCTTCAGAC	8	<hkxhk>	hh	5	hk	267	kk	0
>ref-115956	TCACCTCAAACACTCCCTCCTTCAGAC	25	<hkxhk>	hh	0	hk	273	kk	0
>ref-115956	TCACCTCAAACACTCCCTCCTTCAGAC	26	<hkxhk>	hh	0	hk	273	kk	0
>ref-115967	GAGCCTCTCACTCACCCTCCTTTCTCT	4	<nnxnp>	nn	268	np	0		
>ref-115980	ACCTAGGAGACGCCACCTCCTTCCTGA	23	<lmxll>	ll	149	lm	117		
>ref-116041	AGGAGCCCGACTTAAACTCCAGCCTCG	5	<lmxll>	ll	97	lm	170		
>ref-116041	AGGAGCCCGACTTAAACTCCAGCCTCG	7	<lmxll>	ll	187	lm	75		
>ref-116041	AGGAGCCCGACTTAAACTCCAGCCTCG	13	<lmxll>	ll	255	lm	16		
>ref-116041	AGGAGCCCGACTTAAACTCCAGCCTCG	21	<hkxhk>	hh	9	hk	264	kk	0
>ref-116041	AGGAGCCCGACTTAAACTCCAGCCTCG	23	<hkxhk>	hh	5	hk	267	kk	0
>ref-116041	AGGAGCCCGACTTAAACTCCAGCCTCG	26	<hkxhk>	hh	9	hk	264	kk	0
>ref-116052	ATGAGTTCGACCTTGCCTCCCTGGAAC	1	<lmxll>	ll	15	lm	254		
>ref-116052	ATGAGTTCGACCTTGCCTCCCTGGAAC	25	<hkxhk>	hh	0	hk	273	kk	0
>ref-116060	GTCGGTGTCACTCTCTCTCCTTCCTTG	27	<lmxll>	ll	270	lm	0		
>ref-116063	AGTAACTCTACAATCCCTCCTACATAA	8	<lmxll>	ll	112	lm	118		
>ref-116077	GCTTTCAGGACTCCGGCTCCAGATTTT	2	<lmxll>	ll	202	lm	37		
>ref-116077	GCTTTCAGGACTCCGGCTCCAGATTTT	3	<lmxll>	ll	10	lm	242		
>ref-116077	GCTTTCAGGACTCCGGCTCCAGATTTT	6	<nnxnp>	nn	157	np	72		
>ref-116077	GCTTTCAGGACTCCGGCTCCAGATTTT	13	<nnxnp>	nn	157	np	73		
>ref-116080	TTCTCCCTCACTCTCCCTCCTCTATTT	24	<nnxnp>	nn	173	np	62		
>ref-116087	TTTCGTCTTACTCTTACTCCCTTATTT	4	<nnxnp>	nn	135	np	88		
>ref-116099	CTAATGCCCACTCTCGCTCCTTCCCTC	16	<lmxll>	ll	135	lm	133		
>ref-116101	TCGTTGCACACACACGCTCCTACACAC	2	<nnxnp>	nn	124	np	131		

>ref-116115	TTATTGCTCACTTCCTCTCCGATCGCT	26	<lmxll>	ll	128	lm	133		
>ref-116136	CTCCCATCCACCTCCTCTCCTCCCACT	23	<lmxll>	ll	120	lm	129		
>ref-116143	TTTTTCCTTACCCTTCCTCCTCTACTT	16	<lmxll>	ll	130	lm	112		
>ref-116170	GGACATTATACTTCTTCTCCTCCAGGG	8	<lmxll>	ll	95	lm	135		
>ref-116178	ACCTGGAACACCTCTTCTCCCTCCCTC	16	<lmxll>	ll	126	lm	135		
>ref-116249	GTTGTGCTGACGTGGTCTCCCTCCTCC	15	<nnxnp>	nn	116	np	151		
>ref-116249	GTTGTGCTGACGTGGTCTCCCTCCTCC	27	<lmxll>	ll	138	lm	131		
>ref-116270	TGGCGATATACAGACTCTCCGCTATCC	1	<hkxhk>	hh	60	hk	143	kk	65
>ref-116294	TATTCTGCTACTCCCCCTCCCCCTCTC	13	<lmxll>	ll	135	lm	124		
>ref-116319	ACCTAACAAACCACACCTCCTCGCCTC	26	<lmxll>	ll	138	lm	130		
>ref-116322	CTTTTCCTCACCTCCCCTCCCTCCATC	8	<lmxll>	ll	32	lm	213		
>ref-116322	CTTTTCCTCACCTCCCCTCCCTCCATC	13	<lmxll>	ll	32	lm	214		
>ref-116339	AGAGCTCCTACTGCCACTCCTGGGCCA	4	<hkxhk>	hh	56	hk	139	kk	62
>ref-116339	AGAGCTCCTACTGCCACTCCTGGGCCA	14	<hkxhk>	hh	61	hk	139	kk	56
>ref-116363	CTAACATAGACACAAACTCCCAGCCAT	23	<nnxnp>	nn	265	np	0		
>ref-116372	ATGCTGTGGACTGGCCCTCCCGTTCTC	4	<hkxhk>	hh	2	hk	269	kk	0
>ref-116372	ATGCTGTGGACTGGCCCTCCCGTTCTC	15	<hkxhk>	hh	2	hk	269	kk	0
>ref-116372	ATGCTGTGGACTGGCCCTCCCGTTCTC	25	<hkxhk>	hh	2	hk	267	kk	1
>ref-116373	ACACTCCCTACGCACCCTCCCTGACCT	2	<nnxnp>	nn	267	np	0		
>ref-116373	ACACTCCCTACGCACCCTCCCTGACCT	5	<hkxhk>	hh	53	hk	148	kk	58
>ref-116373	ACACTCCCTACGCACCCTCCCTGACCT	23	<nnxnp>	nn	266	np	1		
>ref-116377	ACATATCCCACGGGGCCTCCCTCTGCA	1	<hkxhk>	hh	9	hk	238	kk	0
>ref-116377	ACATATCCCACGGGGCCTCCCTCTGCA	15	<lmxll>	ll	197	lm	56		
>ref-116405	CCGCCTCGCACTCGGCCTCCTGAGTGT	13	<lmxll>	ll	122	lm	149		
>ref-116407	CTATCCCCCACCCTATCTCCTTATCCC	6	<lmxll>	ll	112	lm	129		
>ref-116411	GAGGGTCGAACCGTATCTCCACTGAAA	21	<nnxnp>	nn	138	np	129		
>ref-116439	CTCACTCTAACACCAACTCCCACTCTC	23	<hkxhk>	hh	63	hk	163	kk	34
>ref-116441	ATAATGATAACGGTAGCTCCGATAATA	12	<nnxnp>	nn	110	np	111		
>ref-116446	TGTTACTGCACTGCCTCTCCTCTCTCT	7	<hkxhk>	hh	4	hk	269	kk	0
>ref-116446	TGTTACTGCACTGCCTCTCCTCTCTCT	9	<hkxhk>	hh	4	hk	265	kk	0
>ref-116446	TGTTACTGCACTGCCTCTCCTCTCTCT	24	<hkxhk>	hh	35	hk	228	kk	0
>ref-116448	ACAATGAGAACGGAATCTCCTTTATTA	6	<nnxnp>	nn	125	np	113		
>ref-116448	ACAATGAGAACGGAATCTCCTTTATTA	12	<nnxnp>	nn	125	np	113		
>ref-116497	CGTTCAGGGACCATAGCTCCGCCCTCA	26	<hkxhk>	hh	6	hk	129	kk	102
>ref-116513	CACTTCCACACTGTCCCTCCCTTATTT	13	<lmxll>	ll	122	lm	99		
>ref-116516	CTATCTTCAACTCCTCCTCCTGTTTGT	16	<nnxnp>	nn	122	np	137		
>ref-116526	AATTACTCCACTTCCTCTCCTTGACCT	3	<hkxhk>	hh	77	hk	136	kk	47
>ref-116526	AATTACTCCACTTCCTCTCCTTGACCT	4	<hkxhk>	hh	76	hk	136	kk	47

>ref-116542	TATAGAGTGACTGGCTCTCCCTTTCCC	15	<nnxnp>	nn	133	np	128		
>ref-116564	CCACCATCGACTGTATCTCCGCCACCA	9	<lmxll>	ll	144	lm	125		
>ref-116567	ATCGACTCCACCCGCACTCCCGCTTCC	1	<nnxnp>	nn	260	np	1		
>ref-116567	ATCGACTCCACCCGCACTCCCGCTTCC	9	<lmxll>	ll	130	lm	124		
>ref-116584	TACCCGCGCACGCTCGCTCCTATTGTC	15	<nnxnp>	nn	131	np	138		
>ref-116616	TTATCCTTTACGCTCCCTCCATTCTTC	2	<nnxnp>	nn	128	np	137		
>ref-116659	CGCTCTCACACATGCCCTCCTCCGTTA	2	<hkxhk>	hh	62	hk	148	kk	50
>ref-116669	TCTGTCCGCACCTATTCTCCTTATTCC	27	<nnxnp>	nn	136	np	128		
>ref-116688	TATAAACATACATATCCTCCTTCCTCT	5	<hkxhk>	hh	58	hk	197	kk	0
>ref-116688	TATAAACATACATATCCTCCTTCCTCT	7	<hkxhk>	hh	58	hk	198	kk	0
>ref-116705	AATCTCTTAACCTCTTCTCCGGGTTTG	9	<nnxnp>	nn	113	np	134		
>ref-116715	GCTGTCCATACCTGCCCTCCTTCTGTT	5	<hkxhk>	hh	58	hk	144	kk	62
>ref-116724	CAGCCTCTCACGGCGGCTCCTTCGGCC	3	<nnxnp>	nn	137	np	126		
>ref-116740	CCCTTGGAAACACCCTCTCCTTCCATG	24	<lmxll>	ll	140	lm	130		
>ref-116745	CTTCTTTTCACCCTCTCTCCACCATCT	5	<nnxnp>	nn	108	np	116		
>ref-116745	CTTCTTTTCACCCTCTCTCCACCATCT	12	<nnxnp>	nn	106	np	116		
>ref-116761	CTCTTTCTCACTCTTCCTCCCTGTGAT	4	<hkxhk>	hh	0	hk	273	kk	0
>ref-116761	CTCTTTCTCACTCTTCCTCCCTGTGAT	16	<hkxhk>	hh	0	hk	273	kk	0
>ref-116768	GAAACCATTACACAGACTCCTAAGACA	13	<lmxll>	ll	129	lm	130		
>ref-116774	TCGTCTCCTACTCCTTCTCCCTCCACT	16	<lmxll>	ll	163	lm	107		
>ref-116792	TCATCACCAACACCATCTCCTCCTCTT	21	<lmxll>	ll	266	lm	0		
>ref-116807	AGACGCGGCACTCAATCTCCTGGGGAT	14	<nnxnp>	nn	114	np	152		
>ref-116807	AGACGCGGCACTCAATCTCCTGGGGAT	15	<nnxnp>	nn	114	np	152		
>ref-116830	GCTTAGATGACGGACGCTCCGATGCTA	24	<nnxnp>	nn	134	np	137		
>ref-116878	TTCTATATGACATCATCTCCATACATC	2	<lmxll>	ll	115	lm	133		
>ref-116882	ATGACCTTAACTTCTCCTCCCGCCACC	14	<lmxll>	ll	133	lm	109		
>ref-116905	TTCGGTTACACGTTCTCTCCTCTCCTT	12	<nnxnp>	nn	132	np	128		
>ref-116907	TCATCCCCTACCTCTTCTCCCTACCTT	3	<nnxnp>	nn	135	np	128		
>ref-116907	TCATCCCCTACCTCTTCTCCCTACCTT	23	<nnxnp>	nn	135	np	129		
>ref-116914	TTCTTCTCCACCTCTCCTCCTTATTAC	8	<nnxnp>	nn	119	np	101		
>ref-116918	ACCATTTCCACCTCCCCTCCACCAGAC	15	<nnxnp>	nn	94	np	155		
>ref-116922	CCTACCCCAACCTCCCCTCCTCCCTAC	15	<lmxll>	ll	18	lm	236		
>ref-116922	CCTACCCCAACCTCCCCTCCTCCCTAC	16	<lmxll>	ll	18	lm	236		
>ref-116924	TTTCCTCCCACACTCCCTCCATTTTCT	7	<nnxnp>	nn	256	np	0		
>ref-116938	GTGATAATAACAATGTCTCCCGCCAGT	8	<nnxnp>	nn	272	np	0		
>ref-116959	TCACCCTCCACCCTCACTCCGCCCATC	1	<hkxhk>	hh	1	hk	233	kk	12
>ref-116959	TCACCCTCCACCCTCACTCCGCCCATC	7	<hkxhk>	hh	1	hk	212	kk	24
>ref-116973	TTCCCTAGCACCTCCTCTCCCTCTCGC	7	<lmxll>	ll	138	lm	119		

>ref-116973	TTCCCTAGCACCTCCTCTCCCTCTCGC	15	<lmxll>	ll	138	lm	119		
>ref-116987	CCAGCATGCACCCCCTCTCCACCCCCC	16	<lmxll>	ll	234	lm	0		
>ref-116998	CTACTCCTCACTTTCTCTCCCTCTTCT	3	<lmxll>	ll	60	lm	182		
>ref-116998	CTACTCCTCACTTTCTCTCCCTCTTCT	27	<lmxll>	ll	125	lm	118		
>ref-117015	CCCCGACCAACCCTTTCTCCTTCCCTC	1	<nnxnp>	nn	111	np	128		
>ref-117015	CCCCGACCAACCCTTTCTCCTTCCCTC	7	<nnxnp>	nn	110	np	129		
>ref-117024	CTCTGTAGAACATAATCTCCCTTTCGA	23	<hkxhk>	hh	22	hk	234	kk	2
>ref-117024	CTCTGTAGAACATAATCTCCCTTTCGA	26	<hkxhk>	hh	21	hk	235	kk	2
>ref-117026	TCCTCTCCTACTCCCCCTCCCTCCTCA	12	<lmxll>	ll	8	lm	257		
>ref-117026	TCCTCTCCTACTCCCCCTCCCTCCTCA	27	<lmxll>	ll	7	lm	256		
>ref-117031	CCCCTCAAAACGACCTCTCCCTCAAAA	1	<hkxhk>	hh	41	hk	231	kk	0
>ref-117031	CCCCTCAAAACGACCTCTCCCTCAAAA	4	<lmxll>	ll	160	lm	97		
>ref-117031	CCCCTCAAAACGACCTCTCCCTCAAAA	7	<lmxll>	ll	69	lm	178		
>ref-117031	CCCCTCAAAACGACCTCTCCCTCAAAA	26	<hkxhk>	hh	65	hk	190	kk	0
>ref-117038	GTCGTCGAAACACCTCCTCCCCTCTTC	5	<nnxnp>	nn	128	np	139		
>ref-117038	GTCGTCGAAACACCTCCTCCCCTCTTC	22	<nnxnp>	nn	127	np	137		
>ref-117056	CCAGTTCTTACTCCTGCTCCAACATTA	5	<hkxhk>	hh	2	hk	228	kk	15
>ref-117056	CCAGTTCTTACTCCTGCTCCAACATTA	8	<hkxhk>	hh	2	hk	227	kk	16
>ref-117072	GGCGAGCACACCTCTCCTCCTTGCGAT	1	<nnxnp>	nn	143	np	111		
>ref-117087	ATATACAAGACTGCTACTCCCATGGCT	6	<nnxnp>	nn	142	np	107		
>ref-117136	CCCTCTCCCACTCTCTCTCCCTTCCGA	26	<lmxll>	ll	102	lm	131		
>ref-117138	TTACATCCCACAATTTCTCCTCTCCTT	22	<nnxnp>	nn	138	np	120		
>ref-117144	ACCCTCCCCACCTTCTCTCCTCCTTCC	2	<hkxhk>	hh	0	hk	258	kk	5
>ref-117170	CCGGTCACGACCCGCTCTCCCCGCGAC	25	<hkxhk>	hh	0	hk	261	kk	3
>ref-117172	ATGTCTGTAACCTTTCCTCCTCCTCTG	7	<lmxll>	ll	110	lm	127		
>ref-117184	AGCCACTCAACTTCAGCTCCTTACTGG	16	<lmxll>	ll	163	lm	95		
>ref-117191	CTTGCCCCTACTGCCTCTCCCCTCTTA	1	<lmxll>	ll	118	lm	139		
>ref-117191	CTTGCCCCTACTGCCTCTCCCCTCTTA	13	<lmxll>	ll	118	lm	139		
>ref-117196	ACCATTACCACCTCTTCTCCCTCTACT	22	<nnxnp>	nn	130	np	138		
>ref-117199	ATCGCCTCCACTTGCTCTCCTTTATCA	14	<lmxll>	ll	132	lm	140		
>ref-117204	CATTCCTTAACCTCCCCTCCTTCTCTC	2	<lmxll>	ll	152	lm	107		
>ref-117204	CATTCCTTAACCTCCCCTCCTTCTCTC	3	<lmxll>	ll	120	lm	106		
>ref-117214	TGTCTGAATACATTGTCTCCAAGTGTA	23	<nnxnp>	nn	131	np	136		
>ref-117230	ATGAGCCCCACTGATTCTCCTGAGTCT	12	<lmxll>	ll	7	lm	263		
>ref-117230	ATGAGCCCCACTGATTCTCCTGAGTCT	27	<lmxll>	ll	7	lm	263		
>ref-117246	TTCTTTGCCACTCTTCCTCCTCTCTTT	3	<lmxll>	ll	103	lm	118		
>ref-117246	TTCTTTGCCACTCTTCCTCCTCTCTTT	23	<hkxhk>	hh	48	hk	191	kk	4
>ref-117279	TCCATACACACCAGATCTCCTCCTTCC	25	<lmxll>	ll	131	lm	113		

>ref-117284	CGCGCTGCGACATAACCTCCGTTTCGC	15	<hkxhk>	hh	0	hk	271	kk	1
>ref-117284	CGCGCTGCGACATAACCTCCGTTTCGC	26	<hkxhk>	hh	0	hk	271	kk	1
>ref-117288	ATTATCTTCACTCCATCTCCGTTTCTC	25	<lmxll>	ll	105	lm	137		
>ref-117339	TCCTTCCACACCCTCTCTCCTCTGTTC	8	<nnxnp>	nn	259	np	0		
>ref-117339	TCCTTCCACACCCTCTCTCCTCTGTTC	24	<nnxnp>	nn	258	np	0		
>ref-117341	CCCCTTACCACACCAACTCCTCCCGTC	26	<lmxll>	ll	149	lm	100		
>ref-117351	CTCACCATCACCTTAACTCCCCCCCAC	13	<hkxhk>	hh	51	hk	139	kk	49
>ref-117355	TACTTCCTTACTCTCCCTCCCACTCCC	1	<lmxll>	ll	14	lm	247		
>ref-117355	TACTTCCTTACTCTCCCTCCCACTCCC	22	<hkxhk>	hh	0	hk	273	kk	0
>ref-117355	TACTTCCTTACTCTCCCTCCCACTCCC	25	<lmxll>	ll	14	lm	246		
>ref-117355	TACTTCCTTACTCTCCCTCCCACTCCC	27	<hkxhk>	hh	4	hk	258	kk	0
>ref-117366	TCCTTATCTACCTTCCCTCCTCCCTCT	6	<lmxll>	ll	2	lm	271		
>ref-117366	TCCTTATCTACCTTCCCTCCTCCCTCT	21	<lmxll>	ll	3	lm	270		
>ref-117379	ACTTTACTTACTCTTTCTCCCTCTACC	3	<nnxnp>	nn	106	np	119		
>ref-117398	AAAATCGTGACTCCCTCTCCTCCAATC	7	<lmxll>	ll	129	lm	121		
>ref-117400	GAATGCCGAACAAAGCCTCCCAAGCGC	5	<lmxll>	ll	143	lm	92		
>ref-117423	ATTTATCTTACCGTCTCTCCTTCCTTC	5	<nnxnp>	nn	108	np	129		
>ref-117426	CACGCTCATACCCGTCCTCCCTCCCGC	12	<lmxll>	ll	135	lm	126		
>ref-117426	CACGCTCATACCCGTCCTCCCTCCCGC	13	<lmxll>	ll	138	lm	126		
>ref-117438	TTATCATCCACCTCTTCTCCCCCTCTC	4	<nnxnp>	nn	126	np	142		
>ref-117443	GAAGGCACGACGTCAGCTCCAGCGACC	9	<nnxnp>	nn	128	np	141		
>ref-117451	ATCCACACGACGTGGCCTCCATATGTT	6	<hkxhk>	hh	102	hk	160	kk	0
>ref-117458	TCCCCAATCACCTGTGCTCCTCTCACC	2	<lmxll>	ll	128	lm	128		
>ref-117462	ATCTATCCCACTCCCTCTCCTTCTCCT	1	<hkxhk>	hh	0	hk	43	kk	191
>ref-117462	ATCTATCCCACTCCCTCTCCTTCTCCT	2	<hkxhk>	hh	0	hk	206	kk	57
>ref-117462	ATCTATCCCACTCCCTCTCCTTCTCCT	5	<hkxhk>	hh	112	hk	129	kk	1
>ref-117462	ATCTATCCCACTCCCTCTCCTTCTCCT	24	<hkxhk>	hh	2	hk	264	kk	5
>ref-117466	CTGTTACTGACCTGGGCTCCCTTGGTG	16	<lmxll>	ll	124	lm	113		
>ref-117467	GGATCCCGCACCTCAACTCCACCAATG	26	<lmxll>	ll	104	lm	135		
>ref-117524	TTCTCCTCAACCCGCCCTCCTTTCCCT	14	<lmxll>	ll	121	lm	139		
>ref-117537	AATTGGCAAACGCGGACTCCTCTCTCG	14	<lmxll>	ll	124	lm	144		
>ref-117537	AATTGGCAAACGCGGACTCCTCTCTCG	23	<lmxll>	ll	124	lm	144		
>ref-117539	CTTTTTCATACTGTTTCTCCCCTCCTA	6	<hkxhk>	hh	5	hk	126	kk	97
>ref-117545	CCTCTTCAAACCCATTCTCCTTCCTGC	8	<hkxhk>	hh	25	hk	213	kk	0
>ref-117545	CCTCTTCAAACCCATTCTCCTTCCTGC	25	<hkxhk>	hh	0	hk	212	kk	25
>ref-117563	GTGTCTCGGACTCGGCCTCCGTGGTCA	27	<nnxnp>	nn	126	np	119		
>ref-117567	CGCCATAAAACCGCATCTCCCCACCCA	1	<lmxll>	ll	261	lm	0		
>ref-117567	CGCCATAAAACCGCATCTCCCCACCCA	13	<lmxll>	ll	146	lm	104		

>ref-117578	CCCCCACACACACACACTCCCCAATTC	8	<hkxhk>	hh	4	hk	267	kk	0
>ref-117578	CCCCCACACACACACACTCCCCAATTC	16	<hkxhk>	hh	7	hk	258	kk	1
>ref-117578	CCCCCACACACACACACTCCCCAATTC	21	<hkxhk>	hh	4	hk	267	kk	0
>ref-117578	CCCCCACACACACACACTCCCCAATTC	22	<hkxhk>	hh	1	hk	259	kk	6
>ref-117580	TCTATCATTACCAATCCTCCTATCAAT	24	<lmxll>	ll	132	lm	120		
>ref-117589	GCACTTCCAACTGCACCTCCACACACA	8	<hkxhk>	hh	47	hk	121	kk	89
>ref-117615	TAAGTCCTCACCGACCCTCCTCGTACT	8	<lmxll>	ll	135	lm	132		
>ref-117622	ACACTCAAGACACCCTCTCCCTCCCTT	27	<lmxll>	ll	137	lm	122		
>ref-117651	CTGAGGTAAACCGCTCCTCCCCTTCCC	15	<hkxhk>	hh	66	hk	137	kk	52
>ref-117657	TGGATATAAACACTGTCTCCTGTGATT	2	<nnxnp>	nn	129	np	129		
>ref-117709	GGCACAAGGACTAGCTCTCCCAGGTAG	27	<lmxll>	ll	146	lm	120		
>ref-117721	CTAATTCACACCCTCCCTCCCCCTCTC	14	<lmxll>	ll	154	lm	103		
>ref-117737	GCCCAAATTACCCGCTCTCCCTTTATC	9	<hkxhk>	hh	56	hk	143	kk	46
>ref-117752	ACGTTCTATACTTTATCTCCGTTTATT	2	<lmxll>	ll	123	lm	130		
>ref-117761	CTTCTTCCTACTCCTACTCCTCACCTC	2	<lmxll>	ll	258	lm	0		
>ref-117762	TTCCCCTCTACCTTCTCTCCCTTCTCC	3	<hkxhk>	hh	27	hk	234	kk	0
>ref-117762	TTCCCCTCTACCTTCTCTCCCTTCTCC	16	<hkxhk>	hh	0	hk	234	kk	27
>ref-117769	CGAGGTGCGACCGCTGCTCCTCGGCGT	14	<nnxnp>	nn	266	np	0		
>ref-117780	AACCGAGGCACCGTCTCTCCCCTCCCA	13	<nnxnp>	nn	137	np	135		
>ref-117792	GCACGCCTTACCCTTTCTCCATGCCCG	8	<lmxll>	ll	121	lm	145		
>ref-117843	TCATCGCTAACGTCGCCTCCAGTTAGG	23	<lmxll>	ll	120	lm	151		
>ref-117865	TATAGGCTTACAGCTCCTCCTTAATGA	27	<nnxnp>	nn	123	np	126		
>ref-117875	TCGCCTGCAACCGCGCCTCCTCCGCCA	5	<hkxhk>	hh	0	hk	266	kk	1
>ref-117875	TCGCCTGCAACCGCGCCTCCTCCGCCA	24	<hkxhk>	hh	0	hk	265	kk	2
>ref-117885	CAAAATCACACTTTACCTCCTTGTAAT	1	<nnxnp>	nn	137	np	82		
>ref-117895	ATAAATGCCACTCCCTCTCCCTCAGGA	27	<nnxnp>	nn	127	np	119		
>ref-117896	CGACCTCGTACATCACCTCCTTCACCT	3	<lmxll>	ll	272	lm	0		
>ref-117896	CGACCTCGTACATCACCTCCTTCACCT	4	<lmxll>	ll	271	lm	0		
>ref-117907	ATATATAGTACTCGCCCTCCGCTTGGT	16	<lmxll>	ll	107	lm	141		
>ref-117955	TTTAGAACAACCCCCCCTCCTCCTCCT	5	<lmxll>	ll	161	lm	81		
>ref-117982	CAAGTTCTTACTTTGACTCCTGCTGTC	2	<nnxnp>	nn	124	np	115		
>ref-118011	GTCTGTCTTACTCTCTCTCCCTTCTTC	22	<hkxhk>	hh	48	hk	148	kk	67
>ref-118016	AATCCTTCAACCTTCCCTCCTGCGCCC	1	<nnxnp>	nn	102	np	162		
>ref-118026	AATCGTTTGACTCCAACTCCTCTGCAC	13	<lmxll>	ll	133	lm	127		
>ref-118035	TACTCCCTCACCTACCCTCCTACCCAC	12	<nnxnp>	nn	121	np	133		
>ref-118041	TTCCCTACTACTCCTTCTCCTCTTCAT	26	<lmxll>	ll	267	lm	0		
>ref-118069	CCTGCCATAACTGGTTCTCCATTTACT	4	<nnxnp>	nn	110	np	118		
>ref-118069	CCTGCCATAACTGGTTCTCCATTTACT	6	<nnxnp>	nn	110	np	118		

>ref-118097	CTCTTCATTACGACCTCTCCTCTATTC	9	<nnxnp>	nn	125	np	138		
>ref-118105	CTTCCACTCACACCCTCTCCTCGTGCC	6	<lmxll>	ll	117	lm	127		
>ref-118105	CTTCCACTCACACCCTCTCCTCGTGCC	9	<lmxll>	ll	118	lm	127		
>ref-118119	CTGTTCCTCACTTACCCTCCTTCCCTC	3	<nnxnp>	nn	249	np	4		
>ref-118119	CTGTTCCTCACTTACCCTCCTTCCCTC	9	<nnxnp>	nn	260	np	0		
>ref-118119	CTGTTCCTCACTTACCCTCCTTCCCTC	13	<nnxnp>	nn	256	np	1		
>ref-118119	CTGTTCCTCACTTACCCTCCTTCCCTC	21	<nnxnp>	nn	258	np	0		
>ref-118120	CAAACTCACACTTACACTCCTATCTTT	22	<nnxnp>	nn	114	np	139		
>ref-118138	CTACCCAACACATACCCTCCTCCTTCC	3	<lmxll>	ll	141	lm	117		
>ref-118138	CTACCCAACACATACCCTCCTCCTTCC	7	<lmxll>	ll	141	lm	117		
>ref-118181	TATTAAATCACCTTCTCTCCTTTACAC	7	<hkxhk>	hh	0	hk	254	kk	7
>ref-118185	CACCTCCTCACTAAACCTCCCTACTTC	24	<lmxll>	ll	118	lm	132		
>ref-118241	TGTTCGCCCACTCCTTCTCCCACTCTC	13	<nnxnp>	nn	133	np	140		
>ref-118263	CTTCGATTGACTAAATCTCCGTCGGCT	7	<hkxhk>	hh	0	hk	272	kk	0
>ref-118263	CTTCGATTGACTAAATCTCCGTCGGCT	13	<nnxnp>	nn	270	np	3		
>ref-118264	CTTCTCTGTACTCTGTCTCCTCTTCTC	8	<hkxhk>	hh	24	hk	238	kk	0
>ref-118274	CTTATTCACACCCTTCCTCCATTCTTC	7	<lmxll>	ll	103	lm	140		
>ref-118304	TATGCACTCACGTATCCTCCCACACCA	7	<nnxnp>	nn	110	np	110		
>ref-118304	TATGCACTCACGTATCCTCCCACACCA	21	<nnxnp>	nn	109	np	110		
>ref-118317	CCCCCTTTCACGCCCCCTCCTTCGCCC	8	<nnxnp>	nn	164	np	99		
>ref-118337	TTCTTTTACACTCCCGCTCCCCCATCC	13	<hkxhk>	hh	47	hk	119	kk	67
>ref-118368	CACACACATACCCAACCTCCTGTCTTA	24	<nnxnp>	nn	126	np	131		
>ref-118371	CTGGCGGCAACACGAGCTCCTCGCGCT	16	<nnxnp>	nn	145	np	126		
>ref-118398	CACACACACACTTTCGCTCCCACACCT	12	<lmxll>	ll	75	lm	196		
>ref-118398	CACACACACACTTTCGCTCCCACACCT	22	<lmxll>	ll	75	lm	196		
>ref-118407	CTTACTACTACTCAACCTCCTCCTCTA	4	<nnxnp>	nn	97	np	151		
>ref-118407	CTTACTACTACTCAACCTCCTCCTCTA	5	<nnxnp>	nn	99	np	148		
>ref-118429	GCGTCTATCACCCCATCTCCCGGGAGA	21	<nnxnp>	nn	118	np	120		
>ref-118430	ATCTCTTTAACTGTTCCTCCCCACTCC	26	<lmxll>	ll	128	lm	123		
>ref-118432	TTTTGAGGGACTATTTCTCCTCAGTGA	12	<hkxhk>	hh	8	hk	255	kk	1
>ref-118432	TTTTGAGGGACTATTTCTCCTCAGTGA	15	<hkxhk>	hh	8	hk	255	kk	1
>ref-118447	TGACTCTTCACCTGTTCTCCTCACCTT	16	<nnxnp>	nn	120	np	146		
>ref-118475	TAGCAACTCACCTACTCTCCATGGAAG	13	<lmxll>	ll	115	lm	139		
>ref-118475	TAGCAACTCACCTACTCTCCATGGAAG	23	<lmxll>	ll	115	lm	139		
>ref-118484	GTGTCCGCCACAATGCCTCCCCCTATC	23	<nnxnp>	nn	136	np	127		
>ref-118498	CCCATCTCCACTTTCCCTCCCCACCTC	5	<lmxll>	ll	13	lm	238		
>ref-118498	CCCATCTCCACTTTCCCTCCCCACCTC	15	<lmxll>	ll	21	lm	241		
>ref-118506	TAGTCCTATACACTAGCTCCAGGGGGA	15	<nnxnp>	nn	114	np	138		

>ref-118528	AAGTCCCCAACTTCTTCTCCATTAGCT	2	<hkxhk>	hh	90	hk	127	kk	6
>ref-118530	AACAACAACACTAATCCTCCTCCTCCT	15	<hkxhk>	hh	0	hk	206	kk	21
>ref-118530	AACAACAACACTAATCCTCCTCCTCCT	16	<hkxhk>	hh	0	hk	207	kk	20
>ref-118536	CTTGGCCTCACCTCTCCTCCCCTCTCC	4	<nnxnp>	nn	7	np	255		
>ref-118536	CTTGGCCTCACCTCTCCTCCCCTCTCC	5	<nnxnp>	nn	8	np	253		
>ref-118536	CTTGGCCTCACCTCTCCTCCCCTCTCC	7	<hkxhk>	hh	77	hk	188	kk	0
>ref-118537	ATCAGCTTAACACCTTCTCCCTCTCTC	4	<lmxll>	ll	121	lm	147		
>ref-118546	AGTTCCTTCACAACCACTCCTCCCTCT	12	<hkxhk>	hh	38	hk	118	kk	63
>ref-118549	AGACTTACAACCCTCCCTCCTTCAAGA	1	<lmxll>	ll	129	lm	124		
>ref-118559	CTCTCACAAACAAACTCTCCCTCACAA	5	<lmxll>	ll	117	lm	136		
>ref-118570	ATCTGGAATACAAAATCTCCTCCACAT	1	<hkxhk>	hh	11	hk	255	kk	0
>ref-118570	ATCTGGAATACAAAATCTCCTCCACAT	21	<hkxhk>	hh	0	hk	255	kk	11
>ref-118573	ATCTAGAATACTCCCCCTCCCACTTTC	14	<nnxnp>	nn	110	np	116		
>ref-118579	ATGGCTGCTACAGGTACTCCCCCTTCA	14	<hkxhk>	hh	0	hk	191	kk	55
>ref-118579	ATGGCTGCTACAGGTACTCCCCCTTCA	25	<hkxhk>	hh	0	hk	200	kk	47
>ref-118597	TGCCTTGTCACCTCTGCTCCGCCCTCT	22	<nnxnp>	nn	268	np	1		
>ref-118609	CATTTTCTCACTCTCTCTCCTTCATCC	21	<lmxll>	ll	122	lm	132		
>ref-118658	TCCTCACCAACCTATCCTCCTCGCCAT	6	<hkxhk>	hh	37	hk	232	kk	0
>ref-118658	TCCTCACCAACCTATCCTCCTCGCCAT	22	<hkxhk>	hh	37	hk	232	kk	0
>ref-118658	TCCTCACCAACCTATCCTCCTCGCCAT	23	<hkxhk>	hh	0	hk	271	kk	0
>ref-118663	TCTCTTCTCACACGTACTCCTTACCCT	5	<nnxnp>	nn	117	np	133		
>ref-118688	GCTACTCCTACTGCCGCTCCTGCTCTG	13	<nnxnp>	nn	139	np	133		
>ref-118689	CTCCCCCGCACCCTCCCTCCGCATGTG	7	<hkxhk>	hh	80	hk	127	kk	50
>ref-118692	CACCCCTCGACCATCCCTCCTCCTCCC	22	<nnxnp>	nn	122	np	141		
>ref-118727	GGCTTCGAAACGTTTCCTCCTTTCTGC	4	<nnxnp>	nn	124	np	140		
>ref-118728	CGCTCTCCAACCTCCCCTCCCCCCTCT	3	<lmxll>	ll	258	lm	0		
>ref-118731	ATCAAGATAACACATTCTCCCTCTAAA	4	<hkxhk>	hh	1	hk	264	kk	1
>ref-118742	TCCTAAAGAACGGGTTCTCCATTTCTC	27	<lmxll>	ll	110	lm	127		
>ref-118797	CCTCCGCCCACCCCTCCTCCATCATCA	25	<lmxll>	ll	120	lm	144		
>ref-118823	GCAGGGATAACCGACGCTCCTTGCCCT	6	<nnxnp>	nn	135	np	128		
>ref-118840	TGCCTCTCTACTTTCTCTCCCGACATT	13	<lmxll>	ll	123	lm	123		
>ref-118889	TCGTCCCCTACTGATCCTCCTATTGAA	21	<lmxll>	ll	123	lm	130		
>ref-118897	TGCCATGGGACGCTCTCTCCCTCCTTT	7	<hkxhk>	hh	1	hk	146	kk	117
>ref-118902	CCATCTCACACCTCGTCTCCCATCTCC	1	<hkxhk>	hh	3	hk	269	kk	0
>ref-118902	CCATCTCACACCTCGTCTCCCATCTCC	6	<nnxnp>	nn	212	np	54		
>ref-118902	CCATCTCACACCTCGTCTCCCATCTCC	7	<nnxnp>	nn	212	np	54		
>ref-118902	CCATCTCACACCTCGTCTCCCATCTCC	15	<nnxnp>	nn	234	np	29		
>ref-118920	CACACATACACTATTTCTCCACCTCTC	7	<nnxnp>	nn	130	np	129		

>ref-118929	GCGCTACCCACTCTGCCTCCTCCTCTT	23	<hkxhk>	hh	62	hk	132	kk	63
>ref-118948	TAACATGGAACAGCACCTCCATTATGA	26	<lmxll>	ll	218	lm	7		
>ref-118965	CATGCATATACTCCTCCTCCGCTTCCT	22	<lmxll>	ll	143	lm	124		
>ref-118968	CACTTGAAGACAATGACTCCATTATTT	3	<nnxnp>	nn	121	np	120		
>ref-118968	CACTTGAAGACAATGACTCCATTATTT	8	<hkxhk>	hh	0	hk	273	kk	0
>ref-118970	TCCCCACTAACTAACCCTCCCTAACTC	12	<lmxll>	ll	127	lm	130		
>ref-118986	TTCAATACAACGGCCTCTCCTTCCTCT	12	<nnxnp>	nn	115	np	149		
>ref-118994	GATCTTTTCACCCTCCCTCCTTCCCTC	5	<lmxll>	ll	205	lm	16		
>ref-119049	TAAGCAATTACTAGACCTCCTTCCCCT	25	<nnxnp>	nn	120	np	139		
>ref-119055	CAGGTATATACACTCTCTCCCTTTCTA	9	<nnxnp>	nn	260	np	0		
>ref-119055	CAGGTATATACACTCTCTCCCTTTCTA	24	<nnxnp>	nn	138	np	114		
>ref-119064	ATAAAGCGTACTTGAGCTCCGTCTCGG	3	<nnxnp>	nn	128	np	130		
>ref-119064	ATAAAGCGTACTTGAGCTCCGTCTCGG	6	<nnxnp>	nn	127	np	130		
>ref-119078	CCCGTTTCAACCTCCTCTCCATCGCGC	1	<lmxll>	ll	140	lm	120		
>ref-119094	GAGACACTAACCCCCCCTCCCCAGCAC	23	<nnxnp>	nn	137	np	120		
>ref-119106	TCTCCTTCCACCCCCACTCCCCAATAT	15	<nnxnp>	nn	121	np	128		
>ref-119155	TTTCGATTTACTTTGCCTCCTTCGGTA	15	<nnxnp>	nn	131	np	123		
>ref-119155	TTTCGATTTACTTTGCCTCCTTCGGTA	25	<nnxnp>	nn	119	np	137		
>ref-119173	CCCCTCTCCACCCCACCTCCTCTACCT	6	<lmxll>	ll	23	lm	241		
>ref-119173	CCCCTCTCCACCCCACCTCCTCTACCT	8	<nnxnp>	nn	141	np	128		
>ref-119173	CCCCTCTCCACCCCACCTCCTCTACCT	9	<nnxnp>	nn	141	np	128		
>ref-119173	CCCCTCTCCACCCCACCTCCTCTACCT	22	<lmxll>	ll	22	lm	242		
>ref-119186	AACTCATCGACCTGCTCTCCCGTTCTC	26	<lmxll>	ll	111	lm	153		
>ref-119255	TTACTCCTTACTCCTTCTCCTTATTCT	14	<nnxnp>	nn	115	np	131		
>ref-119262	GAGACTGTCACGGTTTCTCCTGTCTTC	8	<lmxll>	ll	130	lm	135		
>ref-119262	GAGACTGTCACGGTTTCTCCTGTCTTC	25	<nnxnp>	nn	124	np	136		
>ref-119268	CCCCAACCAACCTCTCCTCCCCTCCTC	1	<hkxhk>	hh	3	hk	259	kk	0
>ref-119268	CCCCAACCAACCTCTCCTCCCCTCCTC	2	<nnxnp>	nn	13	np	245		
>ref-119268	CCCCAACCAACCTCTCCTCCCCTCCTC	5	<nnxnp>	nn	144	np	112		
>ref-119268	CCCCAACCAACCTCTCCTCCCCTCCTC	9	<nnxnp>	nn	123	np	115		
>ref-119268	CCCCAACCAACCTCTCCTCCCCTCCTC	27	<hkxhk>	hh	0	hk	265	kk	0
>ref-119269	CCTTCTTCCACCTCCTCTCCCCCTCTG	6	<nnxnp>	nn	130	np	123		
>ref-119273	CTCTGATCTACATTCGCTCCGATTGCC	24	<nnxnp>	nn	269	np	0		
>ref-119278	AACTGTCATACCTCTGCTCCTTCCTAT	27	<lmxll>	ll	129	lm	139		
>ref-119282	TCCTATTCTACTCTCCCTCCCTCTCCC	5	<lmxll>	ll	87	lm	147		
>ref-119282	TCCTATTCTACTCTCCCTCCCTCTCCC	6	<lmxll>	ll	104	lm	147		
>ref-119299	CCAGTTGCGACCTGAGCTCCACGATCT	23	<lmxll>	ll	0	lm	269		
>ref-119299	CCAGTTGCGACCTGAGCTCCACGATCT	26	<hkxhk>	hh	2	hk	269	kk	0

>ref-119307	TGAAACAGAACATAACCTCCCCCCTAA	5	<nnxnp>	nn	254	np	0		
>ref-119317	GGATCCTTTACTTTGTCTCCGACTTCT	6	<nnxnp>	nn	121	np	133		
>ref-119351	CCCATACGTACACATACTCCCATACGT	1	<lmxll>	ll	17	lm	246		
>ref-119351	CCCATACGTACACATACTCCCATACGT	2	<nnxnp>	nn	213	np	43		
>ref-119351	CCCATACGTACACATACTCCCATACGT	15	<lmxll>	ll	181	lm	75		
>ref-119351	CCCATACGTACACATACTCCCATACGT	21	<nnxnp>	nn	201	np	57		
>ref-119371	GTTGCTTAGACACCCCCTCCCCCCCTC	23	<nnxnp>	nn	120	np	130		
>ref-119377	TCTCCCTCCACCTCTGCTCCCATCCCT	27	<hkxhk>	hh	0	hk	194	kk	65
>ref-119385	TACCCTCTCACCCCTCCTCCCTTGCTC	3	<nnxnp>	nn	270	np	0		
>ref-119402	ATCGAGGCCACTGGTCCTCCGGCCTCT	13	<lmxll>	ll	121	lm	142		
>ref-119412	TACCCTTTCACACAGCCTCCGCTCGTC	26	<hkxhk>	hh	0	hk	272	kk	1
>ref-119415	CCCGGTCGTACGCCTTCTCCAGGTCCA	1	<hkxhk>	hh	11	hk	255	kk	0
>ref-119415	CCCGGTCGTACGCCTTCTCCAGGTCCA	4	<nnxnp>	nn	234	np	10		
>ref-119415	CCCGGTCGTACGCCTTCTCCAGGTCCA	25	<hkxhk>	hh	15	hk	251	kk	0
>ref-119424	TACCCGTACACAAACACTCCTAGTTAT	9	<nnxnp>	nn	121	np	121		
>ref-119441	ACCACCCTCACTTTCTCTCCTCCCTCA	5	<hkxhk>	hh	75	hk	141	kk	37
>ref-119441	ACCACCCTCACTTTCTCTCCTCCCTCA	12	<lmxll>	ll	104	lm	147		
>ref-119447	CATCTCCCCACTATCGCTCCCAACCAT	22	<nnxnp>	nn	132	np	127		
>ref-119451	TGTCCGCCAACTTCATCTCCTTGTTTT	3	<lmxll>	ll	133	lm	126		
>ref-119453	CTATGACCAACAACACCTCCACTATCT	25	<hkxhk>	hh	65	hk	149	kk	42
>ref-119453	CTATGACCAACAACACCTCCACTATCT	26	<hkxhk>	hh	42	hk	149	kk	65
>ref-119474	TTCTGTGCAACTGCTTCTCCTCCTCTT	9	<nnxnp>	nn	121	np	111		
>ref-119476	TCCCTTCCCACTCCCTCTCCTCCCCTT	2	<nnxnp>	nn	5	np	262		
>ref-119476	TCCCTTCCCACTCCCTCTCCTCCCCTT	15	<nnxnp>	nn	6	np	260		
>ref-119479	CATTCACTCACTCACTCTCCTAAGAGG	26	<lmxll>	ll	263	lm	4		
>ref-119479	CATTCACTCACTCACTCTCCTAAGAGG	27	<lmxll>	ll	263	lm	5		
>ref-119486	AATCCACGCACCCACCCTCCCATCCAA	23	<nnxnp>	nn	124	np	129		
>ref-119517	CACACACGCACGCACACTCCAACCCAA	5	<hkxhk>	hh	0	hk	273	kk	0
>ref-119517	CACACACGCACGCACACTCCAACCCAA	7	<lmxll>	ll	7	lm	266		
>ref-119517	CACACACGCACGCACACTCCAACCCAA	24	<hkxhk>	hh	0	hk	273	kk	0
>ref-119534	CACCATCTTACCTCGTCTCCTCTTTTC	14	<nnxnp>	nn	130	np	131		
>ref-119541	AAGTGCTGCACTAAGCCTCCAACTCTC	2	<hkxhk>	hh	57	hk	147	kk	64
>ref-119570	TCCTTTCCTACCCTCCCTCCACCTAAG	24	<hkxhk>	hh	48	hk	139	kk	64
>ref-119571	TTCCCCACCACATCTTCTCCAACCTCT	2	<nnxnp>	nn	122	np	144		
>ref-119593	ATGTAGCGGACTCCTGCTCCCAGTGGC	7	<hkxhk>	hh	0	hk	168	kk	68
>ref-119593	ATGTAGCGGACTCCTGCTCCCAGTGGC	16	<lmxll>	ll	60	lm	170		
>ref-119593	ATGTAGCGGACTCCTGCTCCCAGTGGC	22	<lmxll>	ll	172	lm	66		
>ref-119630	TAACAGCCCACAGAAGCTCCCGCAGTG	4	<nnxnp>	nn	128	np	143		

>ref-119630	TAACAGCCCACAGAAGCTCCCGCAGTG	13	<hkxhk>	hh	67	hk	143	kk	56
>ref-119632	AACACCACCACCCACTCTCCCTCACCT	14	<lmxll>	ll	134	lm	137		
>ref-119646	TCTCTTATTACTCTTCCTCCTAGTCCT	16	<lmxll>	ll	263	lm	0		
>ref-119646	TCTCTTATTACTCTTCCTCCTAGTCCT	21	<lmxll>	ll	134	lm	121		
>ref-119646	TCTCTTATTACTCTTCCTCCTAGTCCT	23	<lmxll>	ll	248	lm	2		
>ref-119685	CACCTCCACACCCCAGCTCCACCCCCC	9	<lmxll>	ll	133	lm	122		
>ref-119689	CCCCATCCCACCCACGCTCCCTTACCT	1	<hkxhk>	hh	13	hk	251	kk	0
>ref-119689	CCCCATCCCACCCACGCTCCCTTACCT	4	<hkxhk>	hh	3	hk	264	kk	1
>ref-119689	CCCCATCCCACCCACGCTCCCTTACCT	5	<hkxhk>	hh	13	hk	252	kk	0
>ref-119709	AGTGCCTCGACTGCGGCTCCGACATCA	6	<lmxll>	ll	119	lm	149		
>ref-119735	GACTCGTGCACTGTCCCTCCTTCTCTC	27	<nnxnp>	nn	128	np	145		
>ref-119736	CTCTGGGCAACACACGCTCCTTCGACC	12	<hkxhk>	hh	64	hk	139	kk	55
>ref-119737	TGACAAATCACCCAGACTCCAACCCAT	2	<hkxhk>	hh	0	hk	262	kk	3
>ref-119737	TGACAAATCACCCAGACTCCAACCCAT	4	<hkxhk>	hh	0	hk	272	kk	0
>ref-119761	GACAAGAGAACTTCATCTCCCGAAGAC	22	<lmxll>	ll	121	lm	143		
>ref-119768	CCTGCCAACACCGCCACTCCTCTTCCG	6	<nnxnp>	nn	92	np	168		
>ref-119768	CCTGCCAACACCGCCACTCCTCTTCCG	27	<nnxnp>	nn	112	np	149		
>ref-119778	ACCATCAACACCAAGTCTCCCCTGTCA	14	<nnxnp>	nn	118	np	150		
>ref-119778	ACCATCAACACCAAGTCTCCCCTGTCA	15	<nnxnp>	nn	118	np	150		
>ref-119780	ATGGTCTTTACTTCTCCTCCACTCCTT	21	<nnxnp>	nn	2	np	260		
>ref-119780	ATGGTCTTTACTTCTCCTCCACTCCTT	22	<nnxnp>	nn	2	np	260		
>ref-119798	CATCCAGTGACATGTCCTCCGCCCCTG	2	<lmxll>	ll	126	lm	119		
>ref-119805	ACCATGAAAACCCTCCCTCCCTCAACA	15	<nnxnp>	nn	118	np	118		
>ref-119841	CTCGCAGCAACGCACCCTCCCTTTCTT	23	<lmxll>	ll	131	lm	136		
>ref-119846	CTGTTATATACACTCTCTCCCTATCTA	15	<lmxll>	ll	131	lm	123		
>ref-119861	TTACAAATTACACGTTCTCCGTGTAGT	13	<nnxnp>	nn	122	np	113		
>ref-119919	ATCCCCACCACTCCTACTCCTCTCTTA	12	<lmxll>	ll	139	lm	119		
>ref-119934	GTCCCTCCTACCCATCCTCCATACGCA	3	<nnxnp>	nn	135	np	120		
>ref-119936	CCACGGCTTACTTTCTCTCCTTTCTCC	26	<lmxll>	ll	143	lm	111		
>ref-119940	ACAGCCACGACCCCGCCTCCCCACGGA	5	<nnxnp>	nn	271	np	0		
>ref-119940	ACAGCCACGACCCCGCCTCCCCACGGA	23	<hkxhk>	hh	48	hk	132	kk	83
>ref-119950	TCCAGTTCTACATGCTCTCCTTCTCCT	22	<lmxll>	ll	137	lm	126		
>ref-119955	AACATAAACACACTTCCTCCAACCCAT	13	<lmxll>	ll	118	lm	132		
>ref-119972	GGTATATGTACCCACCCTCCCCTTCTC	3	<hkxhk>	hh	67	hk	134	kk	56
>ref-120020	ACAGGCACGACCTCCTCTCCTTCCAAT	4	<hkxhk>	hh	0	hk	271	kk	2
>ref-120020	ACAGGCACGACCTCCTCTCCTTCCAAT	13	<lmxll>	ll	21	lm	246		
>ref-120020	ACAGGCACGACCTCCTCTCCTTCCAAT	22	<lmxll>	ll	21	lm	247		
>ref-120021	CATGACTGCACTGCTCCTCCAACCTTA	15	<nnxnp>	nn	124	np	141		

>ref-120026	CCCTATCACACCACCCCTCCATTTCCC	9	<hkxhk>	hh	0	hk	251	kk	16
>ref-120026	CCCTATCACACCACCCCTCCATTTCCC	13	<nnxnp>	nn	239	np	24		
>ref-120030	CGGGGGTATACACACACTCCAAGAAAT	9	<lmxll>	ll	131	lm	126		
>ref-120055	AGTCATCGAACCAGACCTCCAAAGGGT	24	<nnxnp>	nn	239	np	0		
>ref-120055	AGTCATCGAACCAGACCTCCAAAGGGT	26	<nnxnp>	nn	237	np	1		
>ref-120063	CACCTTGACACGTCATCTCCTTGACAC	14	<lmxll>	ll	131	lm	130		
>ref-120093	ACCAACCCGACAAGATCTCCGCAAACA	1	<nnxnp>	nn	123	np	130		
>ref-120099	CCTCACAGAACCTTTTCTCCACAGAAC	8	<hkxhk>	hh	0	hk	195	kk	37
>ref-120099	CCTCACAGAACCTTTTCTCCACAGAAC	24	<nnxnp>	nn	231	np	11		
>ref-120119	TCCTGCGGTACAGAGCCTCCGCGTACT	14	<lmxll>	ll	125	lm	145		
>ref-120134	ACCCCTCATACACGCTCTCCGTCCTAC	25	<nnxnp>	nn	133	np	136		
>ref-120137	CTCCCCCTGACTCCCTCTCCCTTCAGC	1	<hkxhk>	hh	2	hk	255	kk	1
>ref-120137	CTCCCCCTGACTCCCTCTCCCTTCAGC	3	<hkxhk>	hh	12	hk	237	kk	2
>ref-120150	CGTCTTCAAACCGTCTCTCCAACTCTT	13	<hkxhk>	hh	0	hk	21	kk	247
>ref-120153	TGCGTCTTCACTATCTCTCCTGATCTC	25	<hkxhk>	hh	165	hk	91	kk	0
>ref-120167	TTTCTCTTTACCATACCTCCTCGCGAC	14	<hkxhk>	hh	65	hk	127	kk	56
>ref-120170	GAAACAGACACAGAGCCTCCGCCAGTG	1	<nnxnp>	nn	132	np	135		
>ref-120179	TCTAACCTCACAATCCCTCCATTGCCC	2	<hkxhk>	hh	1	hk	265	kk	0
>ref-120179	TCTAACCTCACAATCCCTCCATTGCCC	3	<hkxhk>	hh	0	hk	234	kk	19
>ref-120179	TCTAACCTCACAATCCCTCCATTGCCC	23	<hkxhk>	hh	0	hk	233	kk	18
>ref-120192	TATCTCCTCACCTACCCTCCAACCATC	4	<lmxll>	ll	265	lm	0		
>ref-120193	CTCGCTCTTACATCCCCTCCCCCCCTC	8	<lmxll>	ll	227	lm	0		
>ref-120193	CTCGCTCTTACATCCCCTCCCCCCCTC	25	<nnxnp>	nn	220	np	2		
>ref-120193	CTCGCTCTTACATCCCCTCCCCCCCTC	27	<nnxnp>	nn	224	np	0		
>ref-120197	CACACCCCCACAACACCTCCAAAGAGC	26	<nnxnp>	nn	248	np	0		
>ref-120199	ATATTCACAACAGCTACTCCTGCTACC	22	<nnxnp>	nn	122	np	127		
>ref-120199	ATATTCACAACAGCTACTCCTGCTACC	27	<nnxnp>	nn	122	np	127		
>ref-120207	TTTGTTAACACAACTTCTCCTTGTCGG	1	<hkxhk>	hh	3	hk	269	kk	0
>ref-120207	TTTGTTAACACAACTTCTCCTTGTCGG	16	<hkxhk>	hh	3	hk	269	kk	0
>ref-120209	CCCTTCTCTACCCACCCTCCCCCTTTC	1	<nnxnp>	nn	119	np	111		
>ref-120209	CCCTTCTCTACCCACCCTCCCCCTTTC	2	<nnxnp>	nn	8	np	220		
>ref-120210	ACGATCACAACCCCGACTCCGCGTACT	1	<nnxnp>	nn	146	np	113		
>ref-120210	ACGATCACAACCCCGACTCCGCGTACT	14	<nnxnp>	nn	144	np	113		
>ref-120218	TCTCCACCAACCCCGCCTCCGCGCCCA	5	<lmxll>	ll	15	lm	253		
>ref-120218	TCTCCACCAACCCCGCCTCCGCGCCCA	13	<hkxhk>	hh	37	hk	225	kk	0
>ref-120218	TCTCCACCAACCCCGCCTCCGCGCCCA	14	<hkxhk>	hh	123	hk	135	kk	0
>ref-120218	TCTCCACCAACCCCGCCTCCGCGCCCA	22	<hkxhk>	hh	123	hk	134	kk	0
>ref-120222	ACCATAACCACAGTAGCTCCTACTACC	5	<nnxnp>	nn	58	np	164		

>ref-120224	TCTGAGCAGACTGAGGCTCCTCCAGCT	9	<nnxnp>	nn	261	np	0		
>ref-120240	TATGCCTCGACTCTTCCTCCTCCTCCT	26	<nnxnp>	nn	122	np	116		
>ref-120271	TCGAACTCAACCTTGACTCCCAAGGAA	12	<nnxnp>	nn	118	np	139		
>ref-120308	CTCTCCTCTACATCCCCTCCCTCTCAT	12	<nnxnp>	nn	114	np	138		
>ref-120331	AAGCCTTGGACAGTCACTCCAGGTGGA	3	<hkxhk>	hh	0	hk	186	kk	80
>ref-120331	AAGCCTTGGACAGTCACTCCAGGTGGA	22	<hkxhk>	hh	0	hk	272	kk	0
>ref-120331	AAGCCTTGGACAGTCACTCCAGGTGGA	23	<lmxll>	ll	14	lm	247		
>ref-120331	AAGCCTTGGACAGTCACTCCAGGTGGA	24	<hkxhk>	hh	0	hk	267	kk	4
>ref-120348	TTCCTCCTCACCTGTTCTCCGGGATTA	6	<nnxnp>	nn	108	np	156		
>ref-120356	CCTCATCCCACCTCATCTCCACCCGTC	9	<lmxll>	ll	119	lm	143		
>ref-120357	TTATTAATGACCCTCTCTCCTTTTCCC	12	<nnxnp>	nn	95	np	124		
>ref-120385	CCCCGACACACACACCCTCCACACATG	5	<lmxll>	ll	101	lm	142		
>ref-120393	GATGTTTGCACTCATTCTCCATTGTTA	14	<nnxnp>	nn	138	np	125		
>ref-120399	CATTTTATCACTCTTTCTCCCACGAAT	21	<lmxll>	ll	121	lm	106		
>ref-120406	CGCCCCTTTACTCTCTCTCCACTTTCT	5	<nnxnp>	nn	222	np	1		
>ref-120416	ACGATTACAACAGCACCTCCTATTGAA	3	<hkxhk>	hh	80	hk	110	kk	33
>ref-120429	CCGCCGCTGACATTCACTCCGCTCCTT	21	<nnxnp>	nn	261	np	0		
>ref-120432	TCTACCTCCACCCCTCCTCCCTTCATT	21	<nnxnp>	nn	259	np	2		
>ref-120437	GAGATGTCGACTTGACCTCCTTAACAG	3	<nnxnp>	nn	123	np	129		
>ref-120439	CATACGCACACACGCTCTCCAGCCCAC	16	<hkxhk>	hh	0	hk	265	kk	8
>ref-120439	CATACGCACACACGCTCTCCAGCCCAC	24	<hkxhk>	hh	0	hk	265	kk	8
>ref-120439	CATACGCACACACGCTCTCCAGCCCAC	26	<nnxnp>	nn	141	np	130		
>ref-120448	GGTGCTGTTACCTCTCCTCCTGCCGTC	1	<nnxnp>	nn	265	np	0		
>ref-120463	CATCTTATAACCCTCTCTCCTTCTCAC	13	<nnxnp>	nn	119	np	135		
>ref-120464	ACATGCTATACTCTCTCTCCATCTCTG	13	<nnxnp>	nn	132	np	130		
>ref-120467	TTTCCCCTTACTCTTTCTCCCTCTCCT	7	<lmxll>	ll	73	lm	175		
>ref-120467	TTTCCCCTTACTCTTTCTCCCTCTCCT	25	<lmxll>	ll	74	lm	176		
>ref-120476	TTCTTTACAACCCTTTCTCCATCAGTC	23	<nnxnp>	nn	139	np	119		
>ref-120486	CCCACCCCTACCATCCCTCCTACCCAC	16	<lmxll>	ll	118	lm	118		
>ref-120486	CCCACCCCTACCATCCCTCCTACCCAC	25	<lmxll>	ll	119	lm	118		
>ref-120495	TCTTCCTGTACACATGCTCCTTGCTCA	5	<nnxnp>	nn	132	np	124		
>ref-120495	TCTTCCTGTACACATGCTCCTTGCTCA	12	<nnxnp>	nn	132	np	124		
>ref-120536	ATCGACCTTACTGACTCTCCGTGGCTG	25	<lmxll>	ll	137	lm	130		
>ref-120550	CCCTATCCTACCTCCCCTCCTCCCTAT	3	<hkxhk>	hh	0	hk	193	kk	64
>ref-120550	CCCTATCCTACCTCCCCTCCTCCCTAT	23	<hkxhk>	hh	73	hk	155	kk	0
>ref-120554	ACCGCCTCCACTCTCTCTCCACACCCT	15	<lmxll>	ll	127	lm	145		
>ref-120569	CTGCCTCCTACCCGCCCTCCTAATTGT	6	<lmxll>	ll	140	lm	114		
>ref-120602	CCTCAATCTACTTTTCCTCCCTTTCAC	5	<hkxhk>	hh	5	hk	262	kk	0

>ref-120602	CCTCAATCTACTTTTCCTCCCTTTCAC	12	<hkxhk>	hh	0	hk	262	kk	5
>ref-120610	GCTTTAAATACAGCGTCTCCAGCCATT	14	<nnxnp>	nn	258	np	0		
>ref-120614	CTTATCACTACCACACCTCCTTCATAC	24	<lmxll>	ll	114	lm	132		
>ref-120623	GGCACCATAACCCTTCCTCCCTTATGG	7	<lmxll>	ll	2	lm	264		
>ref-120623	GGCACCATAACCCTTCCTCCCTTATGG	27	<nnxnp>	nn	110	np	155		
>ref-120663	CTCTTTTATACCTCCTCTCCCTTTCCT	4	<hkxhk>	hh	0	hk	114	kk	140
>ref-120663	CTCTTTTATACCTCCTCTCCCTTTCCT	6	<hkxhk>	hh	0	hk	254	kk	8
>ref-120674	ACTTACAAAACACACTCTCCTACATTA	25	<nnxnp>	nn	102	np	139		
>ref-120705	ATTTATATAACGCCGTCTCCTCGGTCC	1	<lmxll>	ll	129	lm	131		
>ref-120705	ATTTATATAACGCCGTCTCCTCGGTCC	2	<hkxhk>	hh	45	hk	155	kk	56
>ref-120718	TCTCCCTTCACCCTCCCTCCTTTTGCC	1	<lmxll>	ll	17	lm	248		
>ref-120718	TCTCCCTTCACCCTCCCTCCTTTTGCC	22	<lmxll>	ll	44	lm	208		
>ref-120731	CGGTTTGTTACTCACCCTCCATGTCCA	22	<lmxll>	ll	138	lm	106		
>ref-120733	ATTATCCCAACCTTCTCTCCTCCCCTT	12	<lmxll>	ll	138	lm	108		
>ref-120733	ATTATCCCAACCTTCTCTCCTCCCCTT	22	<lmxll>	ll	142	lm	108		
>ref-120736	CTCCGCCCTACGCCGTCTCCCTCTCCC	21	<nnxnp>	nn	271	np	0		
>ref-120742	TCCTTACCCACTCTTTCTCCCCCTTCC	5	<lmxll>	ll	113	lm	128		
>ref-120747	ACTGTATATACGCCACCTCCCTCCCTC	7	<hkxhk>	hh	76	hk	136	kk	49
>ref-120747	ACTGTATATACGCCACCTCCCTCCCTC	26	<lmxll>	ll	115	lm	154		
>ref-120754	CCCTCTTGCACTTTCTCTCCTGCTCAC	1	<nnxnp>	nn	228	np	1		
>ref-120754	CCCTCTTGCACTTTCTCTCCTGCTCAC	3	<nnxnp>	nn	247	np	0		
>ref-120777	CCTGCAAGTACTCCTTCTCCTCAGGAA	22	<lmxll>	ll	118	lm	145		
>ref-120779	CCTACTCCTACCCTTACTCCCTTACCT	6	<hkxhk>	hh	22	hk	215	kk	1
>ref-120779	CCTACTCCTACCCTTACTCCCTTACCT	16	<hkxhk>	hh	1	hk	215	kk	22
>ref-120791	GTGAGGGTTACCATTACTCCAATAACG	2	<hkxhk>	hh	0	hk	218	kk	33
>ref-120791	GTGAGGGTTACCATTACTCCAATAACG	5	<hkxhk>	hh	0	hk	230	kk	18
>ref-120791	GTGAGGGTTACCATTACTCCAATAACG	8	<hkxhk>	hh	0	hk	230	kk	26
>ref-120791	GTGAGGGTTACCATTACTCCAATAACG	22	<hkxhk>	hh	33	hk	218	kk	0
>ref-120810	CCCCTCTTTACTCTCTCTCCACTTTCA	1	<nnxnp>	nn	134	np	120		
>ref-120834	AGCTGGAGAACAAAACCTCCAAGACGT	5	<lmxll>	ll	118	lm	127		
>ref-120870	ACGACTGCGACTGCTGCTCCGGCGAGA	23	<hkxhk>	hh	35	hk	222	kk	0
>ref-120870	ACGACTGCGACTGCTGCTCCGGCGAGA	27	<hkxhk>	hh	0	hk	270	kk	0
>ref-120872	ACATGCAAAACTGACACTCCCTTCTTA	24	<lmxll>	ll	129	lm	112		
>ref-120884	GCGCGTGTTACACTCTCTCCCCCTCTC	2	<nnxnp>	nn	138	np	128		
>ref-120884	GCGCGTGTTACACTCTCTCCCCCTCTC	27	<hkxhk>	hh	72	hk	200	kk	0
>ref-120894	TTCGCTCGCACTGGCCCTCCTGCATCC	13	<lmxll>	ll	144	lm	126		
>ref-120897	ACACTCCCGACCCATCCTCCGCTCTTT	21	<hkxhk>	hh	54	hk	145	kk	44
>ref-120899	AAACCTCGCACTCCACCTCCACCCGCA	14	<lmxll>	ll	143	lm	124		

>ref-120950	AGGAGTTGTACCCAGGCTCCTTTTGAT	4	<hkxhk>	hh	53	hk	198	kk	0
>ref-120950	AGGAGTTGTACCCAGGCTCCTTTTGAT	22	<hkxhk>	hh	0	hk	170	kk	92
>ref-120952	GGCCTTGGCACTCCCCCTCCCCCCCTT	1	<hkxhk>	hh	2	hk	261	kk	0
>ref-120952	GGCCTTGGCACTCCCCCTCCCCCCCTT	5	<hkxhk>	hh	2	hk	261	kk	0
>ref-120965	GATGACGTCACTCTCCCTCCGTTCATT	12	<hkxhk>	hh	0	hk	174	kk	92
>ref-120984	CCTACTTCAACTACCTCTCCTCCTTCT	1	<lmxll>	ll	238	lm	6		
>ref-121035	CCTGCCCTCACCCCATCTCCAGCCTTT	26	<lmxll>	ll	103	lm	152		
>ref-121055	TCCCCACCCACCCTTCCTCCCACTCTT	7	<nnxnp>	nn	17	np	230		
>ref-121055	TCCCCACCCACCCTTCCTCCCACTCTT	26	<nnxnp>	nn	15	np	232		
>ref-121062	CACATGAAGACCGCCGCTCCAACGAGA	12	<nnxnp>	nn	143	np	126		
>ref-121071	CAGATCTCGACAATCTCTCCCTTTTCA	14	<lmxll>	ll	113	lm	125		
>ref-121072	CGGCAAATAACACGAGCTCCTAGGAAA	25	<nnxnp>	nn	113	np	137		
>ref-121088	GAGAGGTATACACCACCTCCTCCTATT	25	<nnxnp>	nn	241	np	0		
>ref-121092	CACCGGCCCACGCTCGCTCCCTATTTG	5	<lmxll>	ll	148	lm	118		
>ref-121134	GCTGCTGTGACCCCGCCTCCTCGTTCC	22	<nnxnp>	nn	124	np	148		
>ref-121149	CCCACACACACCCGGACTCCGACCCTC	21	<lmxll>	ll	129	lm	128		
>ref-121152	CCTAAGTACACACAGTCTCCTTCCTCA	27	<nnxnp>	nn	142	np	118		
>ref-121164	TCCTGTTCTACTCTCTCTCCTTTACTT	8	<lmxll>	ll	137	lm	100		
>ref-121167	GGAATCCCTACGCCCACTCCTCCTTTC	9	<lmxll>	ll	2	lm	260		
>ref-121238	TACCCCATTACTCTCCCTCCCCTCATT	1	<hkxhk>	hh	38	hk	162	kk	51
>ref-121238	TACCCCATTACTCTCCCTCCCCTCATT	2	<hkxhk>	hh	51	hk	161	kk	39
>ref-121241	CGTTAAGTAACATAATCTCCTCTCTTT	6	<lmxll>	ll	109	lm	138		
>ref-121245	CTCTCTCCCACAAACTCTCCATCCTCA	22	<nnxnp>	nn	4	np	227		
>ref-121289	TCCTTTCCAACACCACCTCCAACATCC	5	<nnxnp>	nn	119	np	125		
>ref-121316	ATCTCCGTAACTTTCCCTCCCGGTCTG	4	<nnxnp>	nn	110	np	152		
>ref-121341	TTCTCTCCCACCATCCCTCCATTAGTT	9	<lmxll>	ll	135	lm	116		
>ref-121344	GGGACAAGGACCTGCTCTCCGTCAGGG	5	<nnxnp>	nn	139	np	123		
>ref-121355	CATGCCTTAACACGGTCTCCTTGTTAA	2	<hkxhk>	hh	58	hk	145	kk	63
>ref-121356	CTCTGACCCACAGCAACTCCAGTTCAT	5	<hkxhk>	hh	0	hk	272	kk	1
>ref-121356	CTCTGACCCACAGCAACTCCAGTTCAT	8	<hkxhk>	hh	13	hk	253	kk	0
>ref-121356	CTCTGACCCACAGCAACTCCAGTTCAT	22	<hkxhk>	hh	0	hk	272	kk	1
>ref-121370	TCGTCTTTCACTTCATCTCCTGTCTCC	3	<hkxhk>	hh	45	hk	187	kk	0
>ref-121370	TCGTCTTTCACTTCATCTCCTGTCTCC	26	<nnxnp>	nn	120	np	128		
>ref-121394	CTCCTTCACACCTCCCCTCCCCTTCTT	2	<hkxhk>	hh	0	hk	245	kk	14
>ref-121394	CTCCTTCACACCTCCCCTCCCCTTCTT	8	<lmxll>	ll	10	lm	251		
>ref-121394	CTCCTTCACACCTCCCCTCCCCTTCTT	23	<hkxhk>	hh	0	hk	246	kk	14
>ref-121394	CTCCTTCACACCTCCCCTCCCCTTCTT	27	<lmxll>	ll	13	lm	251		
>ref-121395	CGGACGGCCACTCTCCCTCCGCCAGAT	22	<lmxll>	ll	153	lm	120		

>ref-121407	TTTTCATTCACTTTCTCTCCGTCTATT	15	<nnxnp>	nn	149	np	119		
>ref-121415	GGTTTTCCTACGCGATCTCCGTCCTTT	1	<nnxnp>	nn	265	np	0		
>ref-121465	ACTTTCAAAACTCTCTCTCCTTCCTTC	6	<nnxnp>	nn	112	np	118		
>ref-121484	ATCCTTTCCACCACCACTCCCAGCGTC	26	<nnxnp>	nn	240	np	0		
>ref-121490	CCTATGTCTACTTTCTCTCCCACAGCC	14	<hkxhk>	hh	58	hk	155	kk	46
>ref-121506	CGTTCTGCTACTCCTACTCCGCACGCA	14	<nnxnp>	nn	142	np	127		
>ref-121507	CTGCTTTCTACTCCCTCTCCGTTTCCT	3	<hkxhk>	hh	52	hk	151	kk	51
>ref-121507	CTGCTTTCTACTCCCTCTCCGTTTCCT	21	<hkxhk>	hh	51	hk	151	kk	52
>ref-121512	TTCCCCTTAACTCCGTCTCCCTTGGAC	26	<hkxhk>	hh	0	hk	252	kk	17
>ref-121512	TTCCCCTTAACTCCGTCTCCCTTGGAC	27	<lmxll>	ll	4	lm	267		
>ref-121517	CCCGATCAAACTATCCCTCCTCCATCT	3	<nnxnp>	nn	140	np	108		
>ref-121537	TCACTCTATACCCTGCCTCCCATCTCT	1	<lmxll>	ll	36	lm	222		
>ref-121537	TCACTCTATACCCTGCCTCCCATCTCT	15	<lmxll>	ll	13	lm	252		
>ref-121539	CTTATCTTAACGAACTCTCCTTGCATT	7	<lmxll>	ll	127	lm	117		
>ref-121540	GAAATTCGGACAGAACCTCCTGTAGTA	7	<lmxll>	ll	39	lm	208		
>ref-121540	GAAATTCGGACAGAACCTCCTGTAGTA	25	<lmxll>	ll	73	lm	173		
>ref-121557	TCTTTCCTAACCCTCTCTCCTTTCCTG	4	<lmxll>	ll	122	lm	139		
>ref-121557	TCTTTCCTAACCCTCTCTCCTTTCCTG	9	<lmxll>	ll	121	lm	140		
>ref-121559	ACATACCTCACGAACACTCCTATCTAC	8	<nnxnp>	nn	121	np	134		
>ref-121570	GAATATTTGACGTATTCTCCATCCTCT	23	<nnxnp>	nn	109	np	150		
>ref-121570	GAATATTTGACGTATTCTCCATCCTCT	27	<lmxll>	ll	137	lm	124		
>ref-121575	TGACTGTACACCTATACTCCAACTACA	15	<nnxnp>	nn	133	np	131		
>ref-121579	TCCCCATTCACTTCGTCTCCCCCTCCT	15	<lmxll>	ll	72	lm	184		
>ref-121592	CTCCGTCCCACCATCCCTCCTTCCTGT	9	<lmxll>	ll	117	lm	136		
>ref-121592	CTCCGTCCCACCATCCCTCCTTCCTGT	13	<lmxll>	ll	116	lm	136		
>ref-121592	CTCCGTCCCACCATCCCTCCTTCCTGT	16	<lmxll>	ll	127	lm	122		
>ref-121597	CTGTTAGACACATAACCTCCGGAAGCA	24	<hkxhk>	hh	47	hk	138	kk	66
>ref-121627	ACCCTTTTTACTTACTCTCCTCTGTTC	23	<nnxnp>	nn	130	np	113		
>ref-121627	ACCCTTTTTACTTACTCTCCTCTGTTC	26	<hkxhk>	hh	54	hk	121	kk	49
>ref-121664	TCAGGGGAAACATTTCCTCCTTTCGTG	21	<lmxll>	ll	127	lm	118		
>ref-121666	CTTCCCCACACTTCATCTCCTCCTCCT	8	<nnxnp>	nn	119	np	134		
>ref-121666	CTTCCCCACACTTCATCTCCTCCTCCT	23	<nnxnp>	nn	124	np	132		
>ref-121675	CAATCTCTCACTCTTTCTCCTTGTCTT	3	<hkxhk>	hh	53	hk	181	kk	0
>ref-121675	CAATCTCTCACTCTTTCTCCTTGTCTT	7	<lmxll>	ll	140	lm	94		
>ref-121675	CAATCTCTCACTCTTTCTCCTTGTCTT	21	<hkxhk>	hh	0	hk	181	kk	55
>ref-121702	CACGATCACACAACTGCTCCATCACAG	3	<nnxnp>	nn	175	np	91		
>ref-121714	TCCCTTTGTACCCTAGCTCCTTTCTCT	27	<lmxll>	ll	262	lm	0		
>ref-121724	CCTTAGGCCACCTTCCCTCCCCCAACA	2	<hkxhk>	hh	125	hk	99	kk	0

>ref-121724	CCTTAGGCCACCTTCCCTCCCCCAACA	12	<nnxnp>	nn	96	np	140		
>ref-121724	CCTTAGGCCACCTTCCCTCCCCCAACA	23	<hkxhk>	hh	15	hk	223	kk	0
>ref-121724	CCTTAGGCCACCTTCCCTCCCCCAACA	26	<nnxnp>	nn	101	np	138		
>ref-121732	CCGGGCGGGACCGCGCCTCCTGCTGCT	5	<lmxll>	ll	142	lm	123		
>ref-121732	CCGGGCGGGACCGCGCCTCCTGCTGCT	7	<lmxll>	ll	142	lm	123		
>ref-121740	TCTTGTATTACATTTTCTCCTGACCAG	2	<nnxnp>	nn	236	np	0		
>ref-121763	CCCTCCCATACCTCCCCTCCTCCCCAA	8	<nnxnp>	nn	8	np	256		
>ref-121763	CCCTCCCATACCTCCCCTCCTCCCCAA	9	<hkxhk>	hh	0	hk	266	kk	0
>ref-121763	CCCTCCCATACCTCCCCTCCTCCCCAA	26	<nnxnp>	nn	8	np	256		
>ref-121763	CCCTCCCATACCTCCCCTCCTCCCCAA	27	<nnxnp>	nn	162	np	100		
>ref-121781	CGATCCCCAACACTATCTCCCTCCCCC	8	<lmxll>	ll	121	lm	135		
>ref-121792	AGCCGTGTAACTTTCGCTCCATTACTG	16	<lmxll>	ll	111	lm	134		
>ref-121816	CATGTGATAACGGAATCTCCACGTGGC	24	<hkxhk>	hh	50	hk	123	kk	51
>ref-121821	CCACAATTCACCTCCTCTCCATCTTCC	21	<nnxnp>	nn	130	np	125		
>ref-121823	CCATCTGTCACCCTGTCTCCGACATAT	1	<hkxhk>	hh	8	hk	263	kk	0
>ref-121823	CCATCTGTCACCCTGTCTCCGACATAT	2	<lmxll>	ll	121	lm	134		
>ref-121823	CCATCTGTCACCCTGTCTCCGACATAT	5	<lmxll>	ll	65	lm	187		
>ref-121823	CCATCTGTCACCCTGTCTCCGACATAT	9	<lmxll>	ll	65	lm	187		
>ref-121823	CCATCTGTCACCCTGTCTCCGACATAT	12	<lmxll>	ll	121	lm	138		
>ref-121823	CCATCTGTCACCCTGTCTCCGACATAT	13	<nnxnp>	nn	42	np	227		
>ref-121823	CCATCTGTCACCCTGTCTCCGACATAT	25	<nnxnp>	nn	43	np	225		
>ref-121823	CCATCTGTCACCCTGTCTCCGACATAT	27	<hkxhk>	hh	0	hk	263	kk	8
>ref-121825	ATTTCCTATACTCTTCCTCCTCTTCCT	4	<lmxll>	ll	5	lm	267		
>ref-121825	ATTTCCTATACTCTTCCTCCTCTTCCT	26	<lmxll>	ll	5	lm	267		
>ref-121857	CCCTCCCCTACACTCTCTCCCATCTCT	6	<nnxnp>	nn	147	np	120		
>ref-121857	CCCTCCCCTACACTCTCTCCCATCTCT	23	<lmxll>	ll	4	lm	266		
>ref-121857	CCCTCCCCTACACTCTCTCCCATCTCT	27	<lmxll>	ll	3	lm	266		
>ref-121866	GGTTGCCCGACGGACGCTCCCTGTGTC	12	<lmxll>	ll	117	lm	153		
>ref-121877	CCTGTCCCAACCAGTTCTCCATATTTA	27	<hkxhk>	hh	44	hk	123	kk	75
>ref-121897	AAACTTTAAACTCTTCCTCCTCCTCTT	27	<hkxhk>	hh	34	hk	205	kk	5
>ref-121910	TCCGCCACTACAGGTCCTCCAACGGCA	6	<hkxhk>	hh	122	hk	140	kk	0
>ref-121913	TCCTTCCTCACCCCATCTCCCCTTCTC	5	<hkxhk>	hh	0	hk	263	kk	2
>ref-121913	TCCTTCCTCACCCCATCTCCCCTTCTC	6	<hkxhk>	hh	0	hk	259	kk	4
>ref-121950	AGCAGCCTAACTCCCTCTCCCGCCCCC	22	<hkxhk>	hh	46	hk	155	kk	63
>ref-121950	AGCAGCCTAACTCCCTCTCCCGCCCCC	23	<hkxhk>	hh	46	hk	155	kk	63
>ref-121951	TCTTTTCCCACTTTCCCTCCCTTTCCT	1	<lmxll>	ll	171	lm	82		
>ref-121951	TCTTTTCCCACTTTCCCTCCCTTTCCT	16	<lmxll>	ll	169	lm	82		
>ref-122000	ACTGCCACCACTACTACTCCATCTACT	3	<hkxhk>	hh	41	hk	122	kk	56

>ref-122014	CTCCTTTCTACTCGTCCTCCTTATCGC	16	<nnxnp>	nn	259	np	0		
>ref-122014	CTCCTTTCTACTCGTCCTCCTTATCGC	22	<hkxhk>	hh	32	hk	132	kk	75
>ref-122024	TACGCATTTACTCATCCTCCTTATTCA	26	<nnxnp>	nn	130	np	126		
>ref-122034	GAGCGAGGAACAGCGTCTCCGCTACCT	4	<lmxll>	ll	132	lm	138		
>ref-122035	ACCCCTCTCACCTCCTCTCCTCCTTCT	1	<nnxnp>	nn	245	np	5		
>ref-122035	ACCCCTCTCACCTCCTCTCCTCCTTCT	8	<nnxnp>	nn	256	np	3		
>ref-122035	ACCCCTCTCACCTCCTCTCCTCCTTCT	25	<nnxnp>	nn	251	np	6		
>ref-122037	TACCTTCCTACCTCCTCTCCTCCCTCC	21	<lmxll>	ll	36	lm	211		
>ref-122037	TACCTTCCTACCTCCTCTCCTCCCTCC	23	<hkxhk>	hh	18	hk	238	kk	0
>ref-122057	TATATATACACACACTCTCCCGCCGTC	9	<lmxll>	ll	114	lm	112		
>ref-122058	ATCACCACCACCCACCCTCCCTCACCA	2	<lmxll>	ll	134	lm	134		
>ref-122058	ATCACCACCACCCACCCTCCCTCACCA	15	<nnxnp>	nn	128	np	138		
>ref-122091	GTGCATGAGACCATTACTCCGTGCATG	3	<nnxnp>	nn	245	np	21		
>ref-122091	GTGCATGAGACCATTACTCCGTGCATG	4	<nnxnp>	nn	246	np	22		
>ref-122091	GTGCATGAGACCATTACTCCGTGCATG	16	<hkxhk>	hh	6	hk	263	kk	0
>ref-122092	TACCCACCAACCTCCCCTCCCTCTCCA	4	<nnxnp>	nn	188	np	73		
>ref-122092	TACCCACCAACCTCCCCTCCCTCTCCA	13	<hkxhk>	hh	0	hk	270	kk	1
>ref-122092	TACCCACCAACCTCCCCTCCCTCTCCA	15	<lmxll>	ll	13	lm	253		
>ref-122092	TACCCACCAACCTCCCCTCCCTCTCCA	16	<nnxnp>	nn	250	np	15		
>ref-122094	AATGCTATTACCCCCTCTCCCCTCACA	24	<lmxll>	ll	131	lm	115		
>ref-122095	GTATGTATAACTTCTCCTCCTCCTCCT	7	<lmxll>	ll	126	lm	119		
>ref-122124	CCGCATGACACCGGGGCTCCCCTTCAG	12	<nnxnp>	nn	260	np	0		
>ref-122137	GGGCCACAAACCATGGCTCCCGCTTTT	4	<hkxhk>	hh	0	hk	103	kk	154
>ref-122137	GGGCCACAAACCATGGCTCCCGCTTTT	5	<hkxhk>	hh	0	hk	103	kk	154
>ref-122137	GGGCCACAAACCATGGCTCCCGCTTTT	8	<hkxhk>	hh	0	hk	273	kk	0
>ref-122137	GGGCCACAAACCATGGCTCCCGCTTTT	21	<nnxnp>	nn	244	np	19		
>ref-122141	GTCTTCTCTACTTTGCCTCCTCCTCCC	21	<nnxnp>	nn	139	np	123		
>ref-122152	GGCACCGCTACACGTCCTCCATCTCGC	23	<lmxll>	ll	136	lm	90		
>ref-122174	CATTACTCCACATTAACTCCACACTAC	21	<nnxnp>	nn	122	np	135		
>ref-122175	TAAACATGCACACTCTCTCCATACGAA	6	<nnxnp>	nn	129	np	123		
>ref-122187	GCCTCTTCCACCCCTCCTCCCTCCATA	24	<lmxll>	ll	124	lm	135		
>ref-122187	GCCTCTTCCACCCCTCCTCCCTCCATA	27	<lmxll>	ll	121	lm	137		
>ref-122226	CCTCCATGGACTATTTCTCCTTCCTTC	14	<hkxhk>	hh	0	hk	272	kk	0
>ref-122230	CCCCGGCATACCTCCCCTCCCTTCCCC	5	<lmxll>	ll	110	lm	110		
>ref-122309	CCCCTTGACACACCACCTCCAGCTCTC	9	<lmxll>	ll	118	lm	123		
>ref-122323	CTTCGACTCACTGACTCTCCTTTCTTA	21	<lmxll>	ll	113	lm	131		
>ref-122323	CTTCGACTCACTGACTCTCCTTTCTTA	23	<nnxnp>	nn	261	np	0		
>ref-122347	TTAAGATATACTCGGTCTCCTTCCACG	12	<hkxhk>	hh	0	hk	273	kk	0

>ref-122347	TTAAGATATACTCGGTCTCCTTCCACG	13	<hkxhk>	hh	5	hk	266	kk	0
>ref-122347	TTAAGATATACTCGGTCTCCTTCCACG	14	<lmxll>	ll	112	lm	146		
>ref-122350	TCCACCCACACCCTCTCTCCACTCCTC	4	<hkxhk>	hh	0	hk	265	kk	0
>ref-122350	TCCACCCACACCCTCTCTCCACTCCTC	6	<hkxhk>	hh	0	hk	265	kk	0
>ref-122361	ACGCCAACCACAAAGCCTCCGCTCAGC	21	<nnxnp>	nn	134	np	129		
>ref-122408	CTTCCCCTTACCTCATCTCCATCACCA	7	<hkxhk>	hh	1	hk	262	kk	1
>ref-122408	CTTCCCCTTACCTCATCTCCATCACCA	12	<hkxhk>	hh	87	hk	162	kk	0
>ref-122408	CTTCCCCTTACCTCATCTCCATCACCA	13	<hkxhk>	hh	1	hk	262	kk	1
>ref-122410	CCATCCCCCACCCCCACTCCCTTTCCC	9	<nnxnp>	nn	189	np	44		
>ref-122410	CCATCCCCCACCCCCACTCCCTTTCCC	12	<lmxll>	ll	42	lm	188		
>ref-122410	CCATCCCCCACCCCCACTCCCTTTCCC	24	<lmxll>	ll	74	lm	157		
>ref-122410	CCATCCCCCACCCCCACTCCCTTTCCC	27	<hkxhk>	hh	49	hk	195	kk	0
>ref-122413	CTAAATAAAACTTCAACTCCAGAGAGA	5	<nnxnp>	nn	155	np	81		
>ref-122419	TACCCCCCCACCGTTCCTCCCTTTCTA	2	<nnxnp>	nn	237	np	1		
>ref-122419	TACCCCCCCACCGTTCCTCCCTTTCTA	3	<nnxnp>	nn	242	np	1		
>ref-122431	ACCATTTTAACTCCGCCTCCTGCAGCT	1	<hkxhk>	hh	0	hk	263	kk	0
>ref-122431	ACCATTTTAACTCCGCCTCCTGCAGCT	26	<hkxhk>	hh	213	hk	43	kk	0
>ref-122432	TCAAAGGTCACCCAGGCTCCCAGGAAC	7	<nnxnp>	nn	271	np	1		
>ref-122462	TGCGCGGCCACTGTTGCTCCGAACTCC	24	<hkxhk>	hh	70	hk	143	kk	51
>ref-122462	TGCGCGGCCACTGTTGCTCCGAACTCC	27	<lmxll>	ll	139	lm	131		
>ref-122472	CTCAATCTCACTCCTCCTCCTCCCTCC	22	<hkxhk>	hh	171	hk	78	kk	7
>ref-122472	CTCAATCTCACTCCTCCTCCTCCCTCC	23	<hkxhk>	hh	171	hk	80	kk	7
>ref-122478	TCCTAACCCACCCTCTCTCCTTCCTTT	3	<hkxhk>	hh	3	hk	265	kk	0
>ref-122478	TCCTAACCCACCCTCTCTCCTTCCTTT	4	<hkxhk>	hh	0	hk	265	kk	3
>ref-122478	TCCTAACCCACCCTCTCTCCTTCCTTT	26	<hkxhk>	hh	0	hk	251	kk	9
>ref-122478	TCCTAACCCACCCTCTCTCCTTCCTTT	27	<hkxhk>	hh	0	hk	251	kk	9
>ref-122484	CACACCCACACACCGTCTCCCACCCAC	1	<nnxnp>	nn	127	np	134		
>ref-122494	TCCCCTCCAACTCCCCCTCCAACCTCC	1	<nnxnp>	nn	140	np	121		
>ref-122494	TCCCCTCCAACTCCCCCTCCAACCTCC	3	<nnxnp>	nn	140	np	119		
>ref-122494	TCCCCTCCAACTCCCCCTCCAACCTCC	12	<lmxll>	ll	12	lm	246		
>ref-122494	TCCCCTCCAACTCCCCCTCCAACCTCC	26	<lmxll>	ll	15	lm	235		
>ref-122513	ATCTACACCACAAACACTCCTACTAAC	14	<hkxhk>	hh	49	hk	140	kk	48
>ref-122517	TTTATATATACATGCACTCCTGGCATC	2	<hkxhk>	hh	2	hk	207	kk	19
>ref-122517	TTTATATATACATGCACTCCTGGCATC	5	<hkxhk>	hh	2	hk	207	kk	19
>ref-122547	CATTATCACACACCCTCTCCCGCCTCT	7	<lmxll>	ll	115	lm	140		
>ref-122547	CATTATCACACACCCTCTCCCGCCTCT	16	<lmxll>	ll	115	lm	140		
>ref-122570	TCTATCTCTACTCCCTCTCCCTGCCTC	2	<nnxnp>	nn	123	np	105		
>ref-122570	TCTATCTCTACTCCCTCTCCCTGCCTC	21	<nnxnp>	nn	126	np	106		

>ref-122575	CTACCTATCACCCTGCCTCCTACCCCT	24	<nnxnp>	nn	121	np	137		
>ref-122616	ATGGGGATAACGCTACCTCCCTTATAG	4	<hkxhk>	hh	0	hk	152	kk	110
>ref-122616	ATGGGGATAACGCTACCTCCCTTATAG	14	<hkxhk>	hh	0	hk	269	kk	2
>ref-122616	ATGGGGATAACGCTACCTCCCTTATAG	15	<hkxhk>	hh	4	hk	268	kk	0
>ref-122616	ATGGGGATAACGCTACCTCCCTTATAG	25	<hkxhk>	hh	0	hk	268	kk	4
>ref-122625	AAAACCCTAACTAAACCTCCGCGACCT	14	<hkxhk>	hh	56	hk	137	kk	60
>ref-122700	CTCCTCCCTACCTCACCTCCATCCTCT	7	<nnxnp>	nn	142	np	120		
>ref-122700	CTCCTCCCTACCTCACCTCCATCCTCT	15	<nnxnp>	nn	142	np	121		
>ref-122704	AGGGACAACACACACTCTCCCGCATGT	4	<lmxll>	ll	116	lm	144		
>ref-122707	GATACTCGTACGTACGCTCCTTTTAGA	4	<nnxnp>	nn	116	np	144		
>ref-122721	TTCTGCGATACCACCGCTCCGAACTTC	21	<nnxnp>	nn	246	np	0		
>ref-122748	GAATCCCGCACACCTTCTCCCTCCCTC	12	<lmxll>	ll	118	lm	143		
>ref-122758	TGTGTGTTTACTTCAACTCCTAAATCT	26	<hkxhk>	hh	60	hk	123	kk	46
>ref-122774	CCACCATGAACCTAAGCTCCAAGCCTC	21	<nnxnp>	nn	130	np	128		
>ref-122801	CCGATACCCACACTTACTCCGATACCC	15	<nnxnp>	nn	126	np	129		
>ref-122814	AATACCCACACTCTCACTCCTTCACCC	12	<nnxnp>	nn	108	np	138		
>ref-122824	AATTTAAATACCCCTCCTCCTCCTCCT	5	<nnxnp>	nn	221	np	0		
>ref-122851	ACCCCTGAAACAGCTCCTCCTACTAAC	1	<lmxll>	ll	122	lm	125		
>ref-122867	AGGTCTCCGACCCTTCCTCCCCCCTCC	26	<lmxll>	ll	133	lm	130		
>ref-122873	TGATAGAGCACCTCACCTCCTCCCCTG	22	<lmxll>	ll	100	lm	143		
>ref-122897	ATCGCCTCGACGTCCTCTCCTTCGGGA	7	<hkxhk>	hh	62	hk	135	kk	68
>ref-122899	TTCGGCGCGACAGCTACTCCCTGGCTG	1	<nnxnp>	nn	271	np	0		
>ref-122921	CCCACTCAAACCTACACTCCCTCTCGA	3	<nnxnp>	nn	116	np	145		
>ref-122938	CCCTTTTCCACTTCTACTCCTCTCTCT	7	<lmxll>	ll	131	lm	105		
>ref-122938	CCCTTTTCCACTTCTACTCCTCTCTCT	15	<lmxll>	ll	130	lm	105		
>ref-122941	CCTGCCTTTACTTCACCTCCACGTCAC	23	<nnxnp>	nn	133	np	132		
>ref-122958	ACCCTCTCTACTCCCCCTCCTTCCCCC	3	<nnxnp>	nn	121	np	133		
>ref-122981	TTCCTCTTTACCTTCCCTCCCATCCTC	4	<hkxhk>	hh	0	hk	273	kk	0
>ref-122981	TTCCTCTTTACCTTCCCTCCCATCCTC	23	<nnxnp>	nn	191	np	67		
>ref-122999	GAAAGCAGAACAAGAGCTCCAAATATT	13	<nnxnp>	nn	106	np	148		
>ref-123001	TTTCCGGTGACCAGCTCTCCATACAAG	12	<lmxll>	ll	123	lm	126		
>ref-123002	TCTCCACCCACCCGTCCTCCCCCATCC	27	<nnxnp>	nn	118	np	137		
>ref-123009	TCCCCACTCACTCATCCTCCACCTTTC	13	<hkxhk>	hh	8	hk	236	kk	9
>ref-123024	TATTTGTCTACCCCTACTCCATTCAAC	2	<hkxhk>	hh	62	hk	126	kk	55
>ref-123024	TATTTGTCTACCCCTACTCCATTCAAC	4	<hkxhk>	hh	55	hk	126	kk	62
>ref-123028	TACAGAGAGACAGATGCTCCCTAATTA	9	<nnxnp>	nn	267	np	0		
>ref-123037	CCGTCGTCTACTCCTACTCCTCCTCAT	26	<nnxnp>	nn	120	np	143		
>ref-123050	CTGTCATCCACCCCGGCTCCCACCCCC	26	<nnxnp>	nn	239	np	1		

>ref-123062	TCTCGTCTAACTTCCTCTCCCTCCTTC	13	<nnxnp>	nn	123	np	146		
>ref-123062	TCTCGTCTAACTTCCTCTCCCTCCTTC	27	<nnxnp>	nn	124	np	144		
>ref-123078	TTTCTCACCACCCTCCCTCCCTTCCTT	1	<nnxnp>	nn	253	np	1		
>ref-123078	TTTCTCACCACCCTCCCTCCCTTCCTT	7	<nnxnp>	nn	233	np	1		
>ref-123078	TTTCTCACCACCCTCCCTCCCTTCCTT	21	<lmxll>	ll	109	lm	130		
>ref-123079	CAGGATCGCACTACCACTCCATGTCTT	26	<nnxnp>	nn	134	np	123		
>ref-123088	CCTTCCATAACCCTCACTCCTTCTTTC	15	<lmxll>	ll	118	lm	103		
>ref-123099	TTCCTCCTCACTTTCCCTCCATTCCTC	6	<lmxll>	ll	252	lm	0		
>ref-123099	TTCCTCCTCACTTTCCCTCCATTCCTC	23	<lmxll>	ll	224	lm	11		
>ref-123100	GTAATGAGAACCCAACCTCCTCGTGTT	12	<lmxll>	ll	103	lm	135		
>ref-123104	CACCTCGGGACTACCACTCCTCCGTGT	3	<hkxhk>	hh	0	hk	271	kk	1
>ref-123104	CACCTCGGGACTACCACTCCTCCGTGT	13	<nnxnp>	nn	149	np	117		
>ref-123104	CACCTCGGGACTACCACTCCTCCGTGT	22	<lmxll>	ll	47	lm	218		
>ref-123104	CACCTCGGGACTACCACTCCTCCGTGT	24	<nnxnp>	nn	249	np	22		
>ref-123104	CACCTCGGGACTACCACTCCTCCGTGT	25	<lmxll>	ll	103	lm	162		
>ref-123104	CACCTCGGGACTACCACTCCTCCGTGT	26	<hkxhk>	hh	0	hk	273	kk	0
>ref-123127	AAAACAAGGACATAAACTCCCCTGCTA	5	<nnxnp>	nn	120	np	129		
>ref-123140	CACTCCCCCACACCCTCTCCCCATCCC	9	<nnxnp>	nn	126	np	134		
>ref-123154	CCCAACATGACACCCTCTCCCCCACCA	5	<hkxhk>	hh	3	hk	251	kk	0
>ref-123154	CCCAACATGACACCCTCTCCCCCACCA	25	<hkxhk>	hh	0	hk	251	kk	3
>ref-123172	TATTTTTTTACATTGTCTCCTCCGTTC	23	<nnxnp>	nn	203	np	34		
>ref-123180	AAAAATCGTACAAGGACTCCCCCCAAG	6	<nnxnp>	nn	107	np	119		
>ref-123180	AAAAATCGTACAAGGACTCCCCCCAAG	13	<nnxnp>	nn	106	np	119		
>ref-123187	TCTCGCCTCACCTCGCCTCCCTAAGCC	1	<hkxhk>	hh	0	hk	270	kk	1
>ref-123196	CCTCCCCTCACCAGCTCTCCTCCCCTC	3	<nnxnp>	nn	272	np	0		
>ref-123210	GCAACAACAACAATAACTCCTTTTCAT	2	<nnxnp>	nn	126	np	120		
>ref-123259	TAGCAAAAGACCACGACTCCCATATAC	13	<lmxll>	ll	112	lm	147		
>ref-123265	TTCGTCTATACTATTCCTCCTTCTCCT	7	<hkxhk>	hh	63	hk	141	kk	56
>ref-123296	TCTTCCCCCACTCTGTCTCCTTGGCTC	22	<lmxll>	ll	111	lm	154		
>ref-123322	TTTCGTACAACCTCCTCTCCACTCCCC	5	<nnxnp>	nn	121	np	132		
>ref-123329	CAGGTCAACACTCAAGCTCCATCAATC	3	<nnxnp>	nn	112	np	147		
>ref-123359	AACTATGGTACTTATTCTCCAGATTTA	24	<lmxll>	ll	109	lm	110		
>ref-123376	GGGAAGTTTACGAACTCTCCTTAGCGT	25	<lmxll>	ll	112	lm	133		
>ref-123417	CCCCAACCGACCCCCACTCCACAGGAG	15	<hkxhk>	hh	55	hk	118	kk	59
>ref-123419	GTGACACCAACTGTGTCTCCATAGGAT	8	<hkxhk>	hh	50	hk	206	kk	0
>ref-123419	GTGACACCAACTGTGTCTCCATAGGAT	21	<hkxhk>	hh	49	hk	206	kk	0
>ref-123435	ACCATCATCACCATACCTCCCTTATCA	15	<nnxnp>	nn	84	np	161		
>ref-123435	ACCATCATCACCATACCTCCCTTATCA	21	<hkxhk>	hh	120	hk	91	kk	37

>ref-123446	CCTCCTTAGACCGCTTCTCCAACTCTC	1	<nnxnp>	nn	259	np	0		
>ref-123446	CCTCCTTAGACCGCTTCTCCAACTCTC	9	<nnxnp>	nn	262	np	0		
>ref-123448	GAAATCATAACCTTTCCTCCTACTGCT	16	<lmxll>	ll	119	lm	134		
>ref-123457	TCTCCCTCAACGTTCTCTCCCTCTCGG	8	<hkxhk>	hh	0	hk	258	kk	2
>ref-123457	TCTCCCTCAACGTTCTCTCCCTCTCGG	27	<lmxll>	ll	5	lm	265		
>ref-123466	CAGTGGCCTACAATGTCTCCAGGGAGA	1	<nnxnp>	nn	19	np	249		
>ref-123466	CAGTGGCCTACAATGTCTCCAGGGAGA	7	<lmxll>	ll	30	lm	234		
>ref-123466	CAGTGGCCTACAATGTCTCCAGGGAGA	8	<lmxll>	ll	30	lm	234		
>ref-123466	CAGTGGCCTACAATGTCTCCAGGGAGA	13	<hkxhk>	hh	38	hk	223	kk	0
>ref-123466	CAGTGGCCTACAATGTCTCCAGGGAGA	21	<nnxnp>	nn	143	np	122		
>ref-123484	CCCTGTTCAACTTCCCCTCCTCCGTTC	13	<nnxnp>	nn	264	np	0		
>ref-123484	CCCTGTTCAACTTCCCCTCCTCCGTTC	26	<nnxnp>	nn	264	np	0		
>ref-123507	GGGAAAGACACCATCCCTCCCCCTAGC	12	<nnxnp>	nn	257	np	0		
>ref-123549	ACCACCACCACTGTGCCTCCTGCTGCT	13	<lmxll>	ll	157	lm	112		
>ref-123556	TTTATCACAACTCTCCCTCCCTCTCTC	9	<nnxnp>	nn	266	np	5		
>ref-123556	TTTATCACAACTCTCCCTCCCTCTCTC	25	<nnxnp>	nn	245	np	10		
>ref-123556	TTTATCACAACTCTCCCTCCCTCTCTC	26	<hkxhk>	hh	7	hk	262	kk	0
>ref-123556	TTTATCACAACTCTCCCTCCCTCTCTC	27	<nnxnp>	nn	263	np	10		
>ref-123558	GTGCATCCTACACGCCCTCCACTGACT	7	<nnxnp>	nn	132	np	137		
>ref-123584	TCCGCCACGACTTCCTCTCCTGAAGTG	8	<lmxll>	ll	137	lm	130		
>ref-123611	TTGCTAACTACTGTTTCTCCTGTCATT	12	<hkxhk>	hh	46	hk	122	kk	58
>ref-123628	CCTGTCCACACTGTAGCTCCCCATCTC	15	<nnxnp>	nn	120	np	145		
>ref-123641	ACAGCTATTACCTCGTCTCCTCCATCA	2	<hkxhk>	hh	54	hk	139	kk	74
>ref-123646	CTGTCTCATACGTGCTCTCCCGTTTCC	12	<nnxnp>	nn	271	np	0		
>ref-123674	CCCTTCCGCACTTACGCTCCCCCTCCC	7	<nnxnp>	nn	121	np	144		
>ref-123676	TCTTTTCTGACTCGGTCTCCTCCTTTC	25	<nnxnp>	nn	87	np	179		
>ref-123686	CTTTCCCATACTTCCCCTCCTCCTTTT	8	<nnxnp>	nn	4	np	263		
>ref-123686	CTTTCCCATACTTCCCCTCCTCCTTTT	27	<nnxnp>	nn	4	np	262		
>ref-123695	TGAGTTAGCACTTAACCTCCCTTTATC	15	<nnxnp>	nn	117	np	127		
>ref-123698	TATCGTATCACTCCGCCTCCCACTCCA	6	<lmxll>	ll	126	lm	115		
>ref-123700	TTACCACCCACCTTTTCTCCTCCTCTT	5	<nnxnp>	nn	78	np	145		
>ref-123700	TTACCACCCACCTTTTCTCCTCCTCTT	23	<nnxnp>	nn	80	np	145		
>ref-123733	TTCTCGGCCACGCTCACTCCAGGGGCA	1	<lmxll>	ll	124	lm	141		
>ref-123735	TATGTGACGACCATTACTCCCTTCAGG	1	<lmxll>	ll	145	lm	110		
>ref-123735	TATGTGACGACCATTACTCCCTTCAGG	3	<nnxnp>	nn	83	np	172		
>ref-123735	TATGTGACGACCATTACTCCCTTCAGG	12	<lmxll>	ll	1	lm	272		
>ref-123735	TATGTGACGACCATTACTCCCTTCAGG	13	<nnxnp>	nn	83	np	173		
>ref-123738	TCGAGAACCACAGACCCTCCGTCGTTA	21	<nnxnp>	nn	131	np	138		

>ref-123760	ACGTGCAACACTTTCCCTCCCTCCGCT	5	<nnxnp>	nn	117	np	147		
>ref-123760	ACGTGCAACACTTTCCCTCCCTCCGCT	8	<nnxnp>	nn	121	np	143		
>ref-123812	CGGGTTATTACCACGCCTCCTCTTCCC	21	<lmxll>	ll	119	lm	146		
>ref-123813	TGATAGAGTACACCCCCTCCCCCATTA	24	<lmxll>	ll	1	lm	247		
>ref-123813	TGATAGAGTACACCCCCTCCCCCATTA	25	<lmxll>	ll	1	lm	246		
>ref-123814	CACACACACACATTGTCTCCACTCACG	26	<lmxll>	ll	122	lm	149		
>ref-123818	TTTCTCTTTACTTCTTCTCCTTTTCTC	8	<lmxll>	ll	157	lm	99		
>ref-123827	CCTCAGCCCACGCCCACTCCCATGCCA	8	<lmxll>	ll	269	lm	0		
>ref-123833	GCATATATAACAGTCACTCCAACGCAA	1	<nnxnp>	nn	135	np	128		
>ref-123848	CCCTTCATCACCTTTCCTCCCTTTCTC	4	<hkxhk>	hh	0	hk	267	kk	5
>ref-123848	CCCTTCATCACCTTTCCTCCCTTTCTC	24	<hkxhk>	hh	0	hk	267	kk	5
>ref-123853	GCCTTCGGGACTCCGGCTCCTCAGAGT	3	<nnxnp>	nn	150	np	118		
>ref-123853	GCCTTCGGGACTCCGGCTCCTCAGAGT	6	<nnxnp>	nn	16	np	250		
>ref-123853	GCCTTCGGGACTCCGGCTCCTCAGAGT	14	<nnxnp>	nn	17	np	249		
>ref-123853	GCCTTCGGGACTCCGGCTCCTCAGAGT	25	<lmxll>	ll	1	lm	272		
>ref-123878	CACCTCAGCACTTTCTCTCCCTCCCAA	6	<nnxnp>	nn	112	np	114		
>ref-123916	CTCCCTTCCACCTCCTCTCCCTTATTT	3	<lmxll>	ll	251	lm	0		
>ref-123916	CTCCCTTCCACCTCCTCTCCCTTATTT	5	<lmxll>	ll	252	lm	0		
>ref-123917	TTTCTCTTAACTCTCTCTCCTCTTCCT	16	<hkxhk>	hh	0	hk	263	kk	6
>ref-123917	TTTCTCTTAACTCTCTCTCCTCTTCCT	23	<hkxhk>	hh	0	hk	264	kk	6
>ref-123931	ATGTAGCAAACTGAGGCTCCACTTGCA	2	<nnxnp>	nn	133	np	127		
>ref-123960	GTGGCTTCCACGTGGGCTCCGCGTGGA	12	<nnxnp>	nn	25	np	245		
>ref-123960	GTGGCTTCCACGTGGGCTCCGCGTGGA	21	<nnxnp>	nn	24	np	245		
>ref-123991	ATCACCCCCACGCTCCCTCCATCACCC	12	<hkxhk>	hh	0	hk	261	kk	1
>ref-123991	ATCACCCCCACGCTCCCTCCATCACCC	14	<hkxhk>	hh	0	hk	270	kk	2
>ref-123996	ACAAACTGCACGGAGACTCCCACAATG	9	<lmxll>	ll	140	lm	124		
>ref-124002	ACAGCTCTTACCCCCCCTCCCTCCCTC	8	<hkxhk>	hh	5	hk	251	kk	1
>ref-124002	ACAGCTCTTACCCCCCCTCCCTCCCTC	9	<hkxhk>	hh	5	hk	251	kk	1
>ref-124002	ACAGCTCTTACCCCCCCTCCCTCCCTC	25	<hkxhk>	hh	0	hk	209	kk	32
>ref-124002	ACAGCTCTTACCCCCCCTCCCTCCCTC	26	<hkxhk>	hh	0	hk	212	kk	31
>ref-124003	CCCCCTCTCACTTCGCCTCCTTCCACT	2	<lmxll>	ll	115	lm	131		
>ref-124016	TCACTGTACACCTTTGCTCCCACGTTG	4	<nnxnp>	nn	129	np	141		
>ref-124033	GTGTGATGCACACAACCTCCCAGACTG	3	<nnxnp>	nn	110	np	149		
>ref-124073	CGGTTTTGTACCTTCGCTCCAAGCCAT	21	<lmxll>	ll	106	lm	158		
>ref-124087	GGTTAGAACACTGGAACTCCGACCCTC	3	<hkxhk>	hh	0	hk	244	kk	22
>ref-124087	GGTTAGAACACTGGAACTCCGACCCTC	6	<nnxnp>	nn	27	np	235		
>ref-124087	GGTTAGAACACTGGAACTCCGACCCTC	8	<hkxhk>	hh	0	hk	272	kk	0
>ref-124087	GGTTAGAACACTGGAACTCCGACCCTC	9	<hkxhk>	hh	105	hk	152	kk	0

>ref-124087	GGTTAGAACACTGGAACTCCGACCCTC	14	<hkxhk>	hh	0	hk	267	kk	4
>ref-124087	GGTTAGAACACTGGAACTCCGACCCTC	26	<hkxhk>	hh	0	hk	244	kk	22
>ref-124087	GGTTAGAACACTGGAACTCCGACCCTC	27	<nnxnp>	nn	144	np	109		
>ref-124088	TCCTCAACCACGCTACCTCCTTCCTTC	15	<nnxnp>	nn	128	np	126		
>ref-124090	CACCTAGCTACTTACCCTCCTACCTAC	7	<hkxhk>	hh	0	hk	265	kk	2
>ref-124090	CACCTAGCTACTTACCCTCCTACCTAC	21	<hkxhk>	hh	0	hk	268	kk	2
>ref-124104	CCTTCCTTCACTGCCCCTCCTTTCTTA	13	<hkxhk>	hh	0	hk	265	kk	1
>ref-124104	CCTTCCTTCACTGCCCCTCCTTTCTTA	25	<hkxhk>	hh	0	hk	265	kk	1
>ref-124121	GGTCATCAAACCTCGGCTCCTTCATTT	16	<lmxll>	ll	149	lm	99		
>ref-124169	TTATTTCCTACCTTCACTCCTGCCTCT	16	<hkxhk>	hh	51	hk	137	kk	49
>ref-124175	GGTTCTGTCACTACGGCTCCTACGTAG	1	<nnxnp>	nn	101	np	163		
>ref-124187	TACTCGACTACTCGACCTCCTCCCCGA	9	<lmxll>	ll	254	lm	0		
>ref-124191	AAAGACATGACCTCTTCTCCCTTACAT	7	<lmxll>	ll	105	lm	160		
>ref-124220	ACACATCCTACTGTTCCTCCTCCCTCT	2	<nnxnp>	nn	128	np	138		
>ref-124251	AAGGGGATGACGCTACCTCCCGTATAG	3	<nnxnp>	nn	126	np	140		
>ref-124252	ATATACATAACCACCCCTCCTTCTACA	3	<hkxhk>	hh	35	hk	142	kk	73
>ref-124255	CACACTCACACGCGCGCTCCACACATG	23	<lmxll>	ll	121	lm	146		
>ref-124264	GGTGCTTAAACCCATTCTCCACAGATG	7	<nnxnp>	nn	127	np	139		
>ref-124268	ATGCCTCATACCCTGCCTCCTAACATT	15	<hkxhk>	hh	57	hk	132	kk	68
>ref-124284	AACGATACTACAGCTGCTCCTCTGTTA	21	<nnxnp>	nn	124	np	143		
>ref-124306	TCTCCGCTCACCTTCCCTCCTTCCTCC	3	<nnxnp>	nn	12	np	258		
>ref-124306	TCTCCGCTCACCTTCCCTCCTTCCTCC	24	<nnxnp>	nn	12	np	259		
>ref-124312	GGCTAGGAGACGGCGTCTCCGCAATTC	15	<lmxll>	ll	100	lm	130		
>ref-124321	ACCTCCCTCACCCTCTCTCCCTGTGTC	2	<nnxnp>	nn	136	np	127		
>ref-124389	CATTCTATAACCCCTCCTCCCCACTTC	2	<lmxll>	ll	254	lm	0		
>ref-124403	TTATCACTCACATTCCCTCCTTCCCTT	9	<lmxll>	ll	112	lm	141		
>ref-124427	TCCTTTTATACGTGCGCTCCCTCGCCA	7	<lmxll>	ll	139	lm	126		
>ref-124427	TCCTTTTATACGTGCGCTCCCTCGCCA	8	<lmxll>	ll	139	lm	126		
>ref-124443	AGGAAAAGGACATAGACTCCATACACA	12	<hkxhk>	hh	0	hk	273	kk	0
>ref-124443	AGGAAAAGGACATAGACTCCATACACA	14	<hkxhk>	hh	0	hk	273	kk	0
>ref-124484	TGGATTCTGACCTAACCTCCCTGGCTA	6	<nnxnp>	nn	107	np	136		
>ref-124484	TGGATTCTGACCTAACCTCCCTGGCTA	22	<hkxhk>	hh	70	hk	115	kk	53
>ref-124489	CCTTATCAAACATTCTCTCCACATGCA	2	<hkxhk>	hh	0	hk	269	kk	2
>ref-124489	CCTTATCAAACATTCTCTCCACATGCA	21	<hkxhk>	hh	0	hk	272	kk	0
>ref-124504	CACACACACACTGACGCTCCCCTCAGC	12	<nnxnp>	nn	241	np	0		
>ref-124514	CCCCTTTCAACTACCTCTCCATCCCTC	2	<nnxnp>	nn	6	np	252		
>ref-124529	CCAAGGAACACAAAGACTCCAAGGAAA	1	<nnxnp>	nn	266	np	0		
>ref-124532	TTCCTTCCTACTTCCCCTCCCTTCCTT	1	<nnxnp>	nn	101	np	152		

>ref-124532	TTCCTTCCTACTTCCCCTCCCTTCCTT	12	<hkxhk>	hh	0	hk	257	kk	5
>ref-124532	TTCCTTCCTACTTCCCCTCCCTTCCTT	22	<hkxhk>	hh	0	hk	257	kk	8
>ref-124539	TAAATACAAACACAATCTCCATTCTCG	21	<nnxnp>	nn	120	np	123		
>ref-124542	CACACGTACACATAACCTCCTTCCCAC	6	<hkxhk>	hh	0	hk	263	kk	6
>ref-124542	CACACGTACACATAACCTCCTTCCCAC	26	<hkxhk>	hh	6	hk	263	kk	0
>ref-124564	GTCTGGCTGACCTCGCCTCCCAGGAGA	1	<nnxnp>	nn	260	np	0		
>ref-124564	GTCTGGCTGACCTCGCCTCCCAGGAGA	23	<lmxll>	ll	115	lm	131		
>ref-124570	TGATCCCCCACCCTTCCTCCTTCTTCT	2	<nnxnp>	nn	128	np	132		
>ref-124570	TGATCCCCCACCCTTCCTCCTTCTTCT	12	<nnxnp>	nn	128	np	134		
>ref-124594	CAACAGGCCACTCTTCCTCCCTCACTC	7	<nnxnp>	nn	123	np	144		
>ref-124614	CGATCGCTTACATAATCTCCTTCGTCG	13	<nnxnp>	nn	114	np	107		
>ref-124617	CCCGAACCTACTGTGGCTCCCAGACTG	12	<nnxnp>	nn	118	np	142		
>ref-124624	CTCGTTCTCACTATTGCTCCGCATCAC	3	<nnxnp>	nn	121	np	144		
>ref-124626	TTTACCCACACCCTCTCTCCACGCACC	13	<nnxnp>	nn	123	np	140		
>ref-124640	TTTACCCTCACTTTCCCTCCCTCTCAC	2	<nnxnp>	nn	232	np	1		
>ref-124640	TTTACCCTCACTTTCCCTCCCTCTCAC	4	<nnxnp>	nn	219	np	6		
>ref-124640	TTTACCCTCACTTTCCCTCCCTCTCAC	14	<nnxnp>	nn	228	np	5		
>ref-124645	CACATATATACATATACTCCTTAAGTT	5	<nnxnp>	nn	118	np	112		
>ref-124649	CCTCTTCCTACCCCTCCTCCTCCAGAT	12	<hkxhk>	hh	61	hk	200	kk	2
>ref-124649	CCTCTTCCTACCCCTCCTCCTCCAGAT	24	<lmxll>	ll	4	lm	265		
>ref-124649	CCTCTTCCTACCCCTCCTCCTCCAGAT	25	<hkxhk>	hh	3	hk	264	kk	0
>ref-124649	CCTCTTCCTACCCCTCCTCCTCCAGAT	26	<hkxhk>	hh	19	hk	248	kk	0
>ref-124656	CTTATACCCACGTGTACTCCTCACTAC	7	<hkxhk>	hh	51	hk	135	kk	70
>ref-124677	TGTTCCTACACCTTACCTCCAGCTGAT	6	<nnxnp>	nn	1	np	226		
>ref-124682	CGTACTTCTACCAATTCTCCAGTTCCT	14	<lmxll>	ll	138	lm	125		
>ref-124703	TCCCCCTCCACTCATCCTCCTATCTTT	9	<lmxll>	ll	121	lm	131		
>ref-124724	ATTCTTTCTACTTCTCCTCCCTTCTTC	5	<nnxnp>	nn	131	np	137		
>ref-124725	ACCCCTCATACCTTCACTCCCATGTCC	7	<hkxhk>	hh	124	hk	133	kk	0
>ref-124728	CCTTTTCCTACTTCCTCTCCCTCTATT	2	<hkxhk>	hh	75	hk	187	kk	0
>ref-124728	CCTTTTCCTACTTCCTCTCCCTCTATT	6	<hkxhk>	hh	0	hk	195	kk	67
>ref-124728	CCTTTTCCTACTTCCTCTCCCTCTATT	8	<nnxnp>	nn	155	np	105		
>ref-124728	CCTTTTCCTACTTCCTCTCCCTCTATT	25	<hkxhk>	hh	0	hk	272	kk	1
>ref-124735	AATTATCCAACCTTCTCTCCCTTTATC	7	<nnxnp>	nn	147	np	113		
>ref-124735	AATTATCCAACCTTCTCTCCCTTTATC	9	<hkxhk>	hh	1	hk	271	kk	0
>ref-124740	TTCCTTCATACCTCTTCTCCTCCTTGT	2	<hkxhk>	hh	1	hk	264	kk	5
>ref-124740	TTCCTTCATACCTCTTCTCCTCCTTGT	14	<hkxhk>	hh	4	hk	265	kk	1
>ref-124750	ACTATCTCCACCCCTTCTCCCCTCCCC	1	<hkxhk>	hh	0	hk	268	kk	0
>ref-124750	ACTATCTCCACCCCTTCTCCCCTCCCC	9	<hkxhk>	hh	0	hk	269	kk	0

>ref-124769	AATTTTCTAACACCTCCTCCTCCTCTT	5	<lmxll>	ll	28	lm	221		
>ref-124769	AATTTTCTAACACCTCCTCCTCCTCTT	16	<lmxll>	ll	28	lm	220		
>ref-124809	CCGAGACCAACACCATCTCCGTCCCCT	3	<nnxnp>	nn	140	np	129		
>ref-124810	CCTCACGCCACTGCCTCTCCCTCGCCG	1	<nnxnp>	nn	272	np	1		
>ref-124810	CCTCACGCCACTGCCTCTCCCTCGCCG	15	<nnxnp>	nn	108	np	162		
>ref-124812	CTTAAGCACACGCCAGCTCCGAGCGTC	22	<nnxnp>	nn	138	np	125		
>ref-124821	GACAGACAGACCCCTTCTCCTCCTTCT	1	<hkxhk>	hh	56	hk	153	kk	52
>ref-124821	GACAGACAGACCCCTTCTCCTCCTTCT	21	<hkxhk>	hh	56	hk	154	kk	52
>ref-124823	AAAACATGAACGGCGACTCCAGAAAAC	4	<hkxhk>	hh	58	hk	178	kk	0
>ref-124823	AAAACATGAACGGCGACTCCAGAAAAC	5	<hkxhk>	hh	91	hk	146	kk	0
>ref-124823	AAAACATGAACGGCGACTCCAGAAAAC	8	<hkxhk>	hh	1	hk	230	kk	0
>ref-124823	AAAACATGAACGGCGACTCCAGAAAAC	9	<lmxll>	ll	81	lm	161		
>ref-124846	AAAATATTCACACGAGCTCCGACAATT	12	<hkxhk>	hh	60	hk	121	kk	44
>ref-124850	GCCTTGATAACATTCCCTCCATTCAAC	1	<nnxnp>	nn	254	np	15		
>ref-124850	GCCTTGATAACATTCCCTCCATTCAAC	4	<nnxnp>	nn	94	np	164		
>ref-124850	GCCTTGATAACATTCCCTCCATTCAAC	8	<lmxll>	ll	201	lm	57		
>ref-124850	GCCTTGATAACATTCCCTCCATTCAAC	15	<lmxll>	ll	198	lm	61		
>ref-124881	TCCTCTCTCACTCCAACTCCCTACTCC	7	<nnxnp>	nn	160	np	78		
>ref-124881	TCCTCTCTCACTCCAACTCCCTACTCC	15	<nnxnp>	nn	153	np	78		
>ref-124905	ACCGACGTGACCTGACCTCCTAGTACA	4	<hkxhk>	hh	0	hk	271	kk	1
>ref-124905	ACCGACGTGACCTGACCTCCTAGTACA	7	<hkxhk>	hh	0	hk	268	kk	1
>ref-124905	ACCGACGTGACCTGACCTCCTAGTACA	21	<lmxll>	ll	10	lm	257		
>ref-124924	ATATACACAACACACACTCCCATAGGC	2	<lmxll>	ll	117	lm	142		
>ref-124926	CGCGCTGGAACGCTCGCTCCACTCGGT	26	<hkxhk>	hh	74	hk	144	kk	51
>ref-124937	GCAATTTCAACAGAGACTCCAAGATCA	21	<hkxhk>	hh	60	hk	128	kk	54
>ref-124943	CTACCTCCCACCCTCCCTCCACCTCAC	3	<nnxnp>	nn	114	np	126		
>ref-124943	CTACCTCCCACCCTCCCTCCACCTCAC	8	<nnxnp>	nn	121	np	117		
>ref-124953	AGATTTTCCACTCTCGCTCCACCCTCT	16	<lmxll>	ll	124	lm	140		
>ref-124963	CTCCCTTTCACCCCTTCTCCTTCGTTA	1	<lmxll>	ll	266	lm	0		
>ref-124979	CTCTCTTTCACTTTCTCTCCGTTATGT	9	<hkxhk>	hh	70	hk	132	kk	40
>ref-125019	TAGAGTTTTACACACACTCCCCGTGGG	1	<hkxhk>	hh	0	hk	267	kk	1
>ref-125019	TAGAGTTTTACACACACTCCCCGTGGG	14	<nnxnp>	nn	248	np	16		
>ref-125019	TAGAGTTTTACACACACTCCCCGTGGG	23	<lmxll>	ll	199	lm	42		
>ref-125031	TTTATTACTACTCCGTCTCCGTCTCCG	8	<lmxll>	ll	124	lm	106		
>ref-125052	CTGCCGTTAACACACCCTCCGTAAATA	14	<lmxll>	ll	108	lm	158		
>ref-125059	CGACCCATCACCATTACTCCATTGCCC	3	<hkxhk>	hh	0	hk	273	kk	0
>ref-125059	CGACCCATCACCATTACTCCATTGCCC	15	<hkxhk>	hh	0	hk	247	kk	16
>ref-125059	CGACCCATCACCATTACTCCATTGCCC	21	<hkxhk>	hh	0	hk	273	kk	0

>ref-125059	CGACCCATCACCATTACTCCATTGCCC	24	<hkxhk>	hh	0	hk	130	kk	133
>ref-125083	GACCGGCCAACTCCAACTCCCGCGCCT	2	<nnxnp>	nn	131	np	127		
>ref-125139	TTAAGGGGGACTCTCTCCCTCTCTT	14	<lmxll>	ll	119	lm	119		
>ref-125152	ACCTTGCACACCTACACTCCTACAAAC	3	<lmxll>	ll	129	lm	129		
>ref-125159	CCACCTCCTACCCTTCCTCCCGCTACC	16	<lmxll>	ll	126	lm	121		
>ref-125169	CGCACGTGCACACGGCCTCCATGACCA	22	<hkxhk>	hh	0	hk	270	kk	3
>ref-125171	CTGTTCCCCACTCTTCCTCCTTGTTCG	16	<hkxhk>	hh	74	hk	130	kk	52
>ref-125196	TCAAGTAGTACAGATCCTCCATAGCAC	3	<lmxll>	ll	157	lm	96		
>ref-125196	TCAAGTAGTACAGATCCTCCATAGCAC	7	<hkxhk>	hh	1	hk	245	kk	13
>ref-125196	TCAAGTAGTACAGATCCTCCATAGCAC	25	<hkxhk>	hh	1	hk	245	kk	13
>ref-125197	TTTCAACGCACTCTGGCTCCCAGCACA	23	<hkxhk>	hh	58	hk	129	kk	54
>ref-125201	CACACCCACACTCACGCTCCCTCGATC	22	<nnxnp>	nn	103	np	162		
>ref-125202	TCATCTGAAACTCACACTCCTTTTGTA	27	<nnxnp>	nn	2	np	237		
>ref-125222	TTCTCCTCTACCCCCTCTCCACATCCT	21	<nnxnp>	nn	133	np	131		
>ref-125229	ACCACGACGACCTATTCTCCCTCCCCT	5	<nnxnp>	nn	137	np	127		
>ref-125233	TTTCCCATAACCCTCACTCCAGCCTCT	22	<nnxnp>	nn	262	np	0		
>ref-125244	TGCACACGCACACACCCTCCCCCCATT	1	<nnxnp>	nn	119	np	111		
>ref-125244	TGCACACGCACACACCCTCCCCCCATT	9	<nnxnp>	nn	122	np	111		
>ref-125255	TCAACGGCCACCGAGGCTCCAACAGCG	16	<hkxhk>	hh	62	hk	134	kk	67
>ref-125271	ATGACACACACACCTCCTCCCAACACA	25	<lmxll>	ll	131	lm	130		
>ref-125279	CCCTGCCCAACCCCACCTCCCTTCCCC	25	<lmxll>	ll	32	lm	188		
>ref-125292	ACTCCTCGAACTCACCCTCCTTCGCTC	13	<lmxll>	ll	131	lm	141		
>ref-125296	CCCTACCCTACCCTCGCTCCGCTCCAC	21	<hkxhk>	hh	2	hk	263	kk	1
>ref-125296	CCCTACCCTACCCTCGCTCCGCTCCAC	26	<hkxhk>	hh	2	hk	261	kk	1
>ref-125331	TCTCCGTCCACTTGCCCTCCGTGTGTT	12	<nnxnp>	nn	130	np	134		
>ref-125331	TCTCCGTCCACTTGCCCTCCGTGTGTT	13	<hkxhk>	hh	0	hk	263	kk	4
>ref-125383	CTGTATATCACCTTCCCTCCCATAACA	16	<nnxnp>	nn	148	np	107		
>ref-125428	TTCCTCTCTACTCCTCCTCCCTACGGT	8	<hkxhk>	hh	53	hk	138	kk	65
>ref-125435	ATCCCCTTCACCCCTCCTCCCCTACTC	26	<lmxll>	ll	178	lm	46		
>ref-125456	GCATACATAACCCTCCCTCCCTCTCTC	5	<nnxnp>	nn	127	np	134		
>ref-125456	GCATACATAACCCTCCCTCCCTCTCTC	8	<nnxnp>	nn	126	np	134		
>ref-125466	CATGCCCCCACTCCCCCTCCTTTCACT	27	<lmxll>	ll	225	lm	1		
>ref-125493	AGCAGGCCCACCGCGGCTCCCTCCACA	16	<nnxnp>	nn	133	np	131		
>ref-125512	CACGCTTCCACCTTTACTCCCTCCACT	1	<nnxnp>	nn	133	np	124		
>ref-125515	ATTTGTTGCACCATAACTCCTTTTCTT	5	<lmxll>	ll	137	lm	108		
>ref-125524	CTACCTTCCACCCTCGCTCCCCAATCT	7	<lmxll>	ll	124	lm	109		
>ref-125524	CTACCTTCCACCCTCGCTCCCCAATCT	14	<lmxll>	ll	111	lm	119		
>ref-125544	AGAAGGGGAACTCTCCCTCCTTCCTCT	26	<nnxnp>	nn	126	np	133		

>ref-125554	ACTTCAACAACTATACCTCCACAACCT	8	<nnxnp>	nn	165	np	93		
>ref-125554	ACTTCAACAACTATACCTCCACAACCT	13	<nnxnp>	nn	64	np	194		
>ref-125554	ACTTCAACAACTATACCTCCACAACCT	22	<lmxll>	ll	169	lm	96		
>ref-125561	ACTTCCTCCACCATCACTCCTTCACCA	1	<lmxll>	ll	183	lm	85		
>ref-125568	TTTCTTTCAACTCGCCCTCCACTTCTT	12	<lmxll>	ll	116	lm	126		
>ref-125568	TTTCTTTCAACTCGCCCTCCACTTCTT	25	<lmxll>	ll	123	lm	119		
>ref-125571	TGCTCTAACACATGTTCTCCGTGTCCG	12	<hkxhk>	hh	59	hk	146	kk	56
>ref-125592	CTCTCTCTGACTAGCTCTCCCTCCTTT	6	<lmxll>	ll	137	lm	119		
>ref-125596	TCTCCTTCAACAAGTCCTCCCCTTATT	4	<lmxll>	ll	135	lm	121		
>ref-125596	TCTCCTTCAACAAGTCCTCCCCTTATT	14	<lmxll>	ll	135	lm	121		
>ref-125600	ACGAGGTACACATGACCTCCTGACCTC	22	<hkxhk>	hh	53	hk	140	kk	65
>ref-125623	CTCTCTTTGACTCTATCTCCCTCTGCA	21	<nnxnp>	nn	102	np	140		
>ref-125653	GCGGAGGCGACGTCAGCTCCTACGGGC	2	<hkxhk>	hh	0	hk	102	kk	169
>ref-125653	GCGGAGGCGACGTCAGCTCCTACGGGC	6	<hkxhk>	hh	0	hk	113	kk	153
>ref-125653	GCGGAGGCGACGTCAGCTCCTACGGGC	24	<lmxll>	ll	151	lm	113		
>ref-125655	ACCTGACAAACGTGACCTCCCACTCGT	8	<lmxll>	ll	142	lm	116		
>ref-125655	ACCTGACAAACGTGACCTCCCACTCGT	16	<lmxll>	ll	136	lm	117		
>ref-125663	GACATCCACACCCACCCTCCGTGCCTC	5	<hkxhk>	hh	0	hk	273	kk	0
>ref-125719	CCCTTCACTACCCTCCCTCCTCTTTCC	8	<nnxnp>	nn	111	np	131		
>ref-125780	CATACCGTCACAACTACTCCGTCACAT	21	<lmxll>	ll	109	lm	132		
>ref-125818	CTGCTTAACACCCCCCCTCCGATAACA	14	<nnxnp>	nn	115	np	145		
>ref-125825	CCTCTCTTTACTATCCCTCCCCTCCTC	2	<lmxll>	ll	99	lm	157		
>ref-125827	CCGGGAAGCACCAAGGCTCCGTTGGTC	1	<nnxnp>	nn	26	np	228		
>ref-125888	TTCTCTGCCACAGCCACTCCCCTCTCC	9	<lmxll>	ll	131	lm	130		
>ref-125899	AGGCCGCCCACGCGCCCTCCAGGTATA	26	<lmxll>	ll	133	lm	139		
>ref-125901	AGCGGAAGCACGTCTCCTCCATTTTAA	7	<lmxll>	ll	136	lm	130		
>ref-125905	CTCTCTTTTACCTATTCTCCTTTATTT	3	<nnxnp>	nn	128	np	91		
>ref-125910	CTATATTTCACTCGCTCTCCCTCTTCC	5	<lmxll>	ll	115	lm	138		
>ref-125911	TTAACACTGACACTATCTCCCTCATTT	1	<nnxnp>	nn	97	np	123		
>ref-125911	TTAACACTGACACTATCTCCCTCATTT	24	<hkxhk>	hh	1	hk	260	kk	2
>ref-125922	AGTAGCATGACTGTCTCTCCCTCTCTC	15	<lmxll>	ll	124	lm	142		
>ref-125922	AGTAGCATGACTGTCTCTCCCTCTCTC	24	<nnxnp>	nn	141	np	126		
>ref-125933	CCACCCAATACAAGGCCTCCATTGACC	13	<nnxnp>	nn	131	np	132		
>ref-125933	CCACCCAATACAAGGCCTCCATTGACC	15	<nnxnp>	nn	130	np	132		
>ref-125938	CTCCCCTCCACTTAGACTCCTTCCCCT	16	<lmxll>	ll	133	lm	132		
>ref-125962	TGCATTTCTACGCCTTCTCCTCCTCCT	13	<hkxhk>	hh	52	hk	147	kk	66
>ref-125971	TCCATCCATACAGTAACTCCTTAATCT	8	<lmxll>	ll	96	lm	127		
>ref-125971	TCCATCCATACAGTAACTCCTTAATCT	25	<lmxll>	ll	96	lm	127		

>ref-126017	CCTAACCTAACCTGGTCTCCTGTGGTA	21	<lmxll>	ll	146	lm	105		
>ref-126022	ACAAACAAAACCAGTTCTCCTCTCGTC	22	<lmxll>	ll	121	lm	141		
>ref-126025	ATTTCCCTTACTTACCCTCCTGTCTAT	1	<lmxll>	ll	20	lm	247		
>ref-126025	ATTTCCCTTACTTACCCTCCTGTCTAT	8	<hkxhk>	hh	1	hk	272	kk	0
>ref-126025	ATTTCCCTTACTTACCCTCCTGTCTAT	26	<hkxhk>	hh	82	hk	160	kk	0
>ref-126029	GAAAGCAACACAGTCACTCCACAACTC	4	<lmxll>	ll	121	lm	142		
>ref-126041	GCTGCCTGAACTGCAGCTCCGTAAGCT	24	<hkxhk>	hh	0	hk	273	kk	0
>ref-126066	CGGCACTTCACCTTCTCTCCAGCACAA	4	<hkxhk>	hh	84	hk	179	kk	0
>ref-126066	CGGCACTTCACCTTCTCTCCAGCACAA	15	<hkxhk>	hh	0	hk	273	kk	0
>ref-126072	CAAAACATAACACCTCCTCCCATGTAC	9	<hkxhk>	hh	38	hk	225	kk	0
>ref-126072	CAAAACATAACACCTCCTCCCATGTAC	12	<hkxhk>	hh	82	hk	178	kk	0
>ref-126072	CAAAACATAACACCTCCTCCCATGTAC	14	<hkxhk>	hh	83	hk	176	kk	0
>ref-126072	CAAAACATAACACCTCCTCCCATGTAC	21	<lmxll>	ll	52	lm	207		
>ref-126075	AGCCTGTAAACTCCTCCTCCCTCTCCA	1	<nnxnp>	nn	250	np	0		
>ref-126075	AGCCTGTAAACTCCTCCTCCCTCTCCA	2	<nnxnp>	nn	251	np	0		
>ref-126121	TATCTTCATACCTATCCTCCTTGTCCC	2	<hkxhk>	hh	64	hk	142	kk	61
>ref-126124	TCTCCCTCCACTCCTCCTCCTCCCTTC	15	<lmxll>	ll	37	lm	220		
>ref-126124	TCTCCCTCCACTCCTCCTCCTCCCTTC	16	<lmxll>	ll	108	lm	147		
>ref-126125	TGTCTCTCTACTTTATCTCCATTCCCC	2	<hkxhk>	hh	1	hk	263	kk	0
>ref-126125	TGTCTCTCTACTTTATCTCCATTCCCC	7	<hkxhk>	hh	0	hk	263	kk	1
>ref-126135	TTTTACCCCACCACCTCTCCCTCGTTT	8	<lmxll>	ll	127	lm	92		
>ref-126150	TGTTAATAGACTGGAGCTCCCACGTGT	24	<nnxnp>	nn	141	np	124		
>ref-126161	GCTCCCCTCACCCCCACTCCTTTCCTC	1	<nnxnp>	nn	5	np	262		
>ref-126175	TTACAGAAAACAAAATCTCCTTTCCGG	26	<nnxnp>	nn	107	np	126		
>ref-126182	CAGGTGGGCACGATTCCTCCTCACTCC	25	<nnxnp>	nn	267	np	0		
>ref-126186	ATAAGTTGGACCAAATCTCCGAGAATC	9	<nnxnp>	nn	222	np	4		
>ref-126203	CTCTCTTCTACCCATCCTCCTCACGAT	9	<hkxhk>	hh	133	hk	132	kk	0
>ref-126225	AGGACCCGCACTGTTGCTCCCGCATAT	7	<lmxll>	ll	122	lm	140		
>ref-126262	CGTGCGCTTACGGACCCTCCGGCCCAC	5	<hkxhk>	hh	4	hk	268	kk	0
>ref-126262	CGTGCGCTTACGGACCCTCCGGCCCAC	27	<hkxhk>	hh	0	hk	271	kk	0
>ref-126269	CATGCATACACACTCACTCCTTCACTC	7	<nnxnp>	nn	145	np	125		
>ref-126269	CATGCATACACACTCACTCCTTCACTC	9	<nnxnp>	nn	120	np	150		
>ref-126291	GACGCCCCGACAATCACTCCCGGGCCA	7	<lmxll>	ll	254	lm	10		
>ref-126291	GACGCCCCGACAATCACTCCCGGGCCA	13	<hkxhk>	hh	0	hk	269	kk	0
>ref-126299	GAATCTGTCACCATCTCTCCCATTTGG	24	<nnxnp>	nn	223	np	0		
>ref-126307	TTACCCCCAACCCCATCTCCTACACCT	24	<hkxhk>	hh	119	hk	132	kk	1
>ref-126309	GGGATGCTAACATGAGCTCCTGCAGGG	22	<lmxll>	ll	121	lm	146		
>ref-126339	ATCCTTGAGACCTCATCTCCCCTCCCT	9	<nnxnp>	nn	266	np	1		

>ref-126352	TGCAAGATGACGAAAGCTCCTTCTCAT	2	<nnxnp>	nn	121	np	137		
>ref-126354	ATGAGCATGACGTCACCTCCCTCACTC	16	<lmxll>	ll	141	lm	131		
>ref-126356	GGAGATTTAACTCTCTCTCCCTCTCTC	15	<lmxll>	ll	124	lm	136		
>ref-126372	CACGAATCAACAATGCCTCCCACTCGG	25	<nnxnp>	nn	115	np	109		
>ref-126386	TTTTCCCCCACATCCTCTCCCTTTGCC	14	<nnxnp>	nn	124	np	119		
>ref-126414	ATCCCTATCACATCCCCTCCCCCTCTT	1	<hkxhk>	hh	1	hk	271	kk	0
>ref-126414	ATCCCTATCACATCCCCTCCCCCTCTT	14	<nnxnp>	nn	272	np	0		
>ref-126477	TTTGAGCTGACGGACCCTCCAGGGCTT	2	<lmxll>	ll	123	lm	147		
>ref-126477	TTTGAGCTGACGGACCCTCCAGGGCTT	9	<lmxll>	ll	123	lm	147		
>ref-126482	TCACTGCACACAGGAACTCCTGACCTC	7	<nnxnp>	nn	125	np	145		
>ref-126487	TCCATCTCTACCTTTACTCCATTTACA	9	<lmxll>	ll	116	lm	143		
>ref-126494	TCAGCCATCACGCCAGCTCCTTCCCCT	1	<nnxnp>	nn	265	np	0		
>ref-126494	TCAGCCATCACGCCAGCTCCTTCCCCT	2	<nnxnp>	nn	265	np	0		
>ref-126505	CAGTGCATGACCGTTGCTCCCTCAGGA	24	<hkxhk>	hh	57	hk	135	kk	70
>ref-126521	GGTAATAAAACTCTCTCTCCCTCTCTC	1	<lmxll>	ll	130	lm	131		
>ref-126521	GGTAATAAAACTCTCTCTCCCTCTCTC	6	<lmxll>	ll	129	lm	131		
>ref-126521	GGTAATAAAACTCTCTCTCCCTCTCTC	7	<hkxhk>	hh	2	hk	236	kk	24
>ref-126521	GGTAATAAAACTCTCTCTCCCTCTCTC	21	<hkxhk>	hh	24	hk	236	kk	2
>ref-126529	CTCATGCTTACACATCCTCCTTCTACT	8	<nnxnp>	nn	122	np	145		
>ref-126547	ACCTGTTACACTGTCTCTCCGACGGAC	3	<lmxll>	ll	138	lm	125		
>ref-126580	CCTCCCTTCACCCTCCCTCCATTCCAT	1	<lmxll>	ll	266	lm	0		
>ref-126593	CCCTGCAGGACCCACCCTCCCTTCCCC	13	<nnxnp>	nn	250	np	0		
>ref-126596	TCCCCCCTCACTCTTACTCCGTTTCTC	25	<lmxll>	ll	119	lm	114		
>ref-126600	CTCTCGCAAACTCCTCCTCCTTTCTCT	6	<hkxhk>	hh	57	hk	144	kk	50
>ref-126615	TCATCTCTCACCCCTCCTCCTTCTCTC	4	<nnxnp>	nn	49	np	210		
>ref-126615	TCATCTCTCACCCCTCCTCCTTCTCTC	22	<hkxhk>	hh	0	hk	247	kk	20
>ref-126615	TCATCTCTCACCCCTCCTCCTTCTCTC	25	<hkxhk>	hh	14	hk	248	kk	0
>ref-126617	CCCCCTCCCACTTCCCCTCCCCCTATT	7	<lmxll>	ll	135	lm	108		
>ref-126617	CCCCCTCCCACTTCCCCTCCCCCTATT	26	<lmxll>	ll	133	lm	111		
>ref-126631	CGGTTCATTACTTTTCCTCCACGCCCT	2	<lmxll>	ll	139	lm	111		
>ref-126637	CCTACTCATACTCACACTCCTCCTCTT	14	<nnxnp>	nn	263	np	3		
>ref-126637	CCTACTCATACTCACACTCCTCCTCTT	16	<nnxnp>	nn	268	np	1		
>ref-126640	CACGCAGCAACTTCCTCTCCCCTCTGG	7	<lmxll>	ll	126	lm	125		
>ref-126650	CACACACAGACTCACCCTCCTCCTCTG	21	<lmxll>	ll	132	lm	129		
>ref-126652	ACTGACTACACTGCACCTCCCACTGCA	4	<nnxnp>	nn	146	np	117		
>ref-126653	CAGCAGCCCACCTCCCCTCCCATCCTC	22	<lmxll>	ll	1	lm	266		
>ref-126655	TCTCTTCTAACTTCCTCTCCGTTCTCC	15	<nnxnp>	nn	142	np	126		
>ref-126660	GTGCTTTGGACGATTCCTCCCTCGTCG	7	<nnxnp>	nn	131	np	136		

>ref-126661	ACCTCCCCAACACCTCCTCCTTCACTT	24	<lmxll>	ll	126	lm	137		
>ref-126661	ACCTCCCCAACACCTCCTCCTTCACTT	26	<lmxll>	ll	123	lm	138		
>ref-126666	CGGAACACAACGGCCTCTCCTCCCCTC	12	<nnxnp>	nn	129	np	138		
>ref-126678	TGTCTGCTGACTCTGTCTCCTCAAGCG	4	<nnxnp>	nn	119	np	109		
>ref-126683	ACTTATCACACGTACGCTCCTTGCATT	16	<nnxnp>	nn	128	np	137		
>ref-126685	GATTATCAAACTCTCTCTCCTTCTCTC	4	<nnxnp>	nn	230	np	0		
>ref-126685	GATTATCAAACTCTCTCTCCTTCTCTC	6	<nnxnp>	nn	227	np	0		
>ref-126694	TCCTTTATTACCCATTCTCCTCCTCCT	2	<nnxnp>	nn	118	np	137		
>ref-126719	AAGCTTCGGACCCTGACTCCGTGGACC	13	<nnxnp>	nn	139	np	129		
>ref-126728	TCAGCATCCACCTGCTCTCCCCCCATG	6	<hkxhk>	hh	56	hk	143	kk	60
>ref-126735	CAGCGACCCACGAAGACTCCTGGTTTT	27	<lmxll>	ll	129	lm	128		
>ref-126745	TCAGCTGGTACTTCACCTCCTGCTGGC	12	<hkxhk>	hh	59	hk	123	kk	58
>ref-126758	TTTAATCCTACAAAGTCTCCGCCGCTG	6	<nnxnp>	nn	123	np	124		
>ref-126779	CCCCCGCCTACACTCCCTCCCCTTCCC	2	<nnxnp>	nn	115	np	127		
>ref-126779	CCCCCGCCTACACTCCCTCCCCTTCCC	23	<nnxnp>	nn	115	np	127		
>ref-126801	TAGATGTGCACAGCTGCTCCGGGCGTG	13	<nnxnp>	nn	144	np	126		
>ref-126805	TCTACTGCTACTGACTCTCCTGCTGAT	1	<hkxhk>	hh	0	hk	273	kk	0
>ref-126805	TCTACTGCTACTGACTCTCCTGCTGAT	4	<hkxhk>	hh	0	hk	273	kk	0
>ref-126807	CACATCCCCACCCACGCTCCTCCTCCC	1	<hkxhk>	hh	0	hk	269	kk	1
>ref-126807	CACATCCCCACCCACGCTCCTCCTCCC	2	<hkxhk>	hh	0	hk	269	kk	1
>ref-126810	TCTCCCTTTACCTCTTCTCCTACTACT	4	<lmxll>	ll	134	lm	107		
>ref-126810	TCTCCCTTTACCTCTTCTCCTACTACT	12	<lmxll>	ll	106	lm	143		
>ref-126810	TCTCCCTTTACCTCTTCTCCTACTACT	24	<lmxll>	ll	134	lm	107		
>ref-126823	CTATCTCACACCGTCACTCCCTTTGCC	24	<lmxll>	ll	127	lm	125		
>ref-126827	GGCTCTATAACGATCGCTCCTTGCCTC	15	<lmxll>	ll	131	lm	138		
>ref-126830	CCCTCATACACTGTTTCTCCGTCTCTC	7	<lmxll>	ll	124	lm	137		
>ref-126839	TCTCCCCAAACCCCTCCTCCTGCCTTC	15	<lmxll>	ll	131	lm	133		
>ref-126862	TTCCTTATGACTCCTCCTCCCCCTCCT	3	<lmxll>	ll	114	lm	132		
>ref-126862	TTCCTTATGACTCCTCCTCCCCCTCCT	7	<nnxnp>	nn	230	np	12		
>ref-126862	TTCCTTATGACTCCTCCTCCCCCTCCT	9	<nnxnp>	nn	241	np	4		
>ref-126866	CGCACACAAACAATCTCTCCATCTCCC	4	<hkxhk>	hh	44	hk	122	kk	69
>ref-126876	AACCCTGCAACTTCACCTCCCCTGTGT	7	<hkxhk>	hh	50	hk	141	kk	67
>ref-126919	CTACGCACCACACATCCTCCTCCTCCT	8	<hkxhk>	hh	53	hk	146	kk	67
>ref-126935	GAACCTGTGACGCCTCCTCCTCCTCTT	3	<lmxll>	ll	131	lm	136		
>ref-126940	CGTGTTTTTACCCCCTCTCCTTGCTAA	9	<nnxnp>	nn	87	np	134		
>ref-126945	ATTATTCCTACATCCTCTCCTTTACTG	24	<nnxnp>	nn	95	np	141		
>ref-126971	GCGCGGTCAACCCTGCCTCCGTGCCTC	6	<lmxll>	ll	262	lm	0		
>ref-126991	CGACCTTCCACCCTTCCTCCACTCGCC	4	<nnxnp>	nn	139	np	125		

>ref-126996	TCTTACTCTACCTTTTCTCCCTCCCTT	5	<hkxhk>	hh	3	hk	267	kk	0
>ref-126996	TCTTACTCTACCTTTTCTCCCTCCCTT	8	<hkxhk>	hh	3	hk	266	kk	0
>ref-126996	TCTTACTCTACCTTTTCTCCCTCCCTT	27	<hkxhk>	hh	0	hk	254	kk	16
>ref-127011	TAAAAACCCACGGTTCCTCCCGACTAA	1	<lmxll>	ll	141	lm	113		
>ref-127016	TGCGAGGAGACTCTCACTCCCTCCTTG	6	<nnxnp>	nn	15	np	255		
>ref-127016	TGCGAGGAGACTCTCACTCCCTCCTTG	7	<nnxnp>	nn	15	np	255		
>ref-127017	ATATATTGCACACACACTCCCACACAC	6	<lmxll>	ll	0	lm	270		
>ref-127017	ATATATTGCACACACACTCCCACACAC	9	<hkxhk>	hh	3	hk	266	kk	0
>ref-127017	ATATATTGCACACACACTCCCACACAC	24	<hkxhk>	hh	12	hk	254	kk	0
>ref-127020	TATGGGCTGACCCACACTCCACATCTC	2	<lmxll>	ll	242	lm	2		
>ref-127033	ACTGCGACAACCTTCACTCCAAGTACC	12	<hkxhk>	hh	7	hk	258	kk	0
>ref-127034	AGGAATTGCACAGTTACTCCATTTCAG	13	<nnxnp>	nn	123	np	114		
>ref-127037	CAGAGTTTCACGAACACTCCCAACACA	1	<hkxhk>	hh	67	hk	137	kk	55
>ref-127057	TCTTCTTGGACACAGCCTCCTTAGCCC	24	<nnxnp>	nn	273	np	0		
>ref-127074	GCCTAAAGCACTCCTCCTCCCATACGT	1	<hkxhk>	hh	0	hk	127	kk	139
>ref-127111	GGTTGATGAACAAATGCTCCAGACATC	2	<hkxhk>	hh	56	hk	148	kk	52
>ref-127118	CTCTCCCCCACGCCCCCTCCTCTTCCA	12	<nnxnp>	nn	114	np	123		
>ref-127128	ATCTTCTCCACCTCTCCTCCTTCCCTC	16	<lmxll>	ll	0	lm	261		
>ref-127128	ATCTTCTCCACCTCTCCTCCTTCCCTC	22	<lmxll>	ll	264	lm	0		
>ref-127128	ATCTTCTCCACCTCTCCTCCTTCCCTC	25	<lmxll>	ll	264	lm	0		
>ref-127209	TCACTCCCTACCCCCACTCCCCGTTCC	3	<lmxll>	ll	102	lm	154		
>ref-127209	TCACTCCCTACCCCCACTCCCCGTTCC	14	<nnxnp>	nn	260	np	7		
>ref-127210	ATCGTCTATACACAAACTCCCGTCTCT	5	<lmxll>	ll	136	lm	121		
>ref-127215	TCTACCTACACATCCTCTCCTCCTCTC	8	<nnxnp>	nn	255	np	0		
>ref-127215	TCTACCTACACATCCTCTCCTCCTCTC	12	<nnxnp>	nn	252	np	0		
>ref-127221	CTTTCCCTTACTTCTTCTCCCTCCTTC	26	<nnxnp>	nn	264	np	2		
>ref-127221	CTTTCCCTTACTTCTTCTCCCTCCTTC	27	<nnxnp>	nn	266	np	0		
>ref-127226	TTAATTAACACTGTGGCTCCAACTTTA	26	<lmxll>	ll	123	lm	127		
>ref-127237	TCTGGACAAACAGCTGCTCCCACCTCT	23	<lmxll>	ll	120	lm	143		
>ref-127249	TTCTTTCTTACTCTTCCTCCTTCTATT	2	<hkxhk>	hh	0	hk	270	kk	1
>ref-127249	TTCTTTCTTACTCTTCCTCCTTCTATT	21	<hkxhk>	hh	0	hk	270	kk	1
>ref-127254	GCTCACGCCACGGCTGCTCCAATTATT	5	<hkxhk>	hh	103	hk	156	kk	0
>ref-127254	GCTCACGCCACGGCTGCTCCAATTATT	9	<lmxll>	ll	116	lm	142		
>ref-127275	GAGTGCCAGACGCTGACTCCGCGACCC	4	<nnxnp>	nn	56	np	207		
>ref-127275	GAGTGCCAGACGCTGACTCCGCGACCC	5	<nnxnp>	nn	227	np	40		
>ref-127275	GAGTGCCAGACGCTGACTCCGCGACCC	9	<hkxhk>	hh	0	hk	251	kk	17
>ref-127275	GAGTGCCAGACGCTGACTCCGCGACCC	13	<lmxll>	ll	8	lm	262		
>ref-127275	GAGTGCCAGACGCTGACTCCGCGACCC	22	<hkxhk>	hh	6	hk	232	kk	0

>ref-127275	GAGTGCCAGACGCTGACTCCGCGACCC	27	<lmxll>	ll	16	lm	256		
>ref-127285	ACCCCCCTCACTCCTCCTCCCTCCCAC	4	<lmxll>	ll	233	lm	21		
>ref-127285	ACCCCCCTCACTCCTCCTCCCTCCCAC	12	<nnxnp>	nn	69	np	171		
>ref-127287	CCCACACTCACGCAAGCTCCCATGTAT	8	<lmxll>	ll	132	lm	124		
>ref-127292	TTTCAATCCACTTTCTCTCCTCTTCCA	5	<lmxll>	ll	114	lm	130		
>ref-127292	TTTCAATCCACTTTCTCTCCTCTTCCA	27	<lmxll>	ll	111	lm	131		
>ref-127323	CTTTTCCTGACATCCCCTCCTTCTATT	3	<nnxnp>	nn	54	np	182		
>ref-127345	GCTATCGCTACGCACGCTCCATTGATC	9	<nnxnp>	nn	265	np	0		
>ref-127362	GGGGTTGTTACACTCGCTCCCGCTCAA	4	<lmxll>	ll	126	lm	118		
>ref-127364	TTTCCAATTACCTTTCCTCCTTTACCT	26	<nnxnp>	nn	259	np	0		
>ref-127364	TTTCCAATTACCTTTCCTCCTTTACCT	27	<nnxnp>	nn	257	np	0		
>ref-127390	AACACGACTACGGTTTCTCCTTATCTC	15	<nnxnp>	nn	132	np	129		
>ref-127410	GAGATTTATACCAACGCTCCTTTGGCA	21	<lmxll>	ll	152	lm	104		
>ref-127416	AAATCACTCACTGAAGCTCCTTTTCCC	1	<hkxhk>	hh	23	hk	226	kk	0
>ref-127416	AAATCACTCACTGAAGCTCCTTTTCCC	16	<hkxhk>	hh	0	hk	210	kk	34
>ref-127421	TCCTCCCACACCGCCCCTCCCTCTCCT	6	<nnxnp>	nn	264	np	0		
>ref-127422	TCTTCAGCTACATTTTCTCCTCCATCT	22	<nnxnp>	nn	118	np	144		
>ref-127422	TCTTCAGCTACATTTTCTCCTCCATCT	27	<nnxnp>	nn	118	np	144		
>ref-127428	TCCCTACCAACCCGACCTCCTTCCTTA	23	<lmxll>	ll	113	lm	157		
>ref-127432	TCAAACCCTACTGGCTCTCCCTCTCCC	27	<lmxll>	ll	260	lm	1		
>ref-127441	CATGTGCAAACTTCCACTCCATCCACC	25	<nnxnp>	nn	130	np	136		
>ref-127503	TCTCCTTCCACTTTCTCTCCGTCTCCT	7	<nnxnp>	nn	134	np	116		
>ref-127562	TAACCCCCTACCCTGACTCCCACCCTT	8	<lmxll>	ll	114	lm	136		
>ref-127566	TACATCCCCACTCTTCCTCCTCTCCCT	9	<nnxnp>	nn	136	np	128		
>ref-127570	ACTACCATTACTGCTACTCCCATGATA	25	<hkxhk>	hh	47	hk	135	kk	49
>ref-127577	AAAGGATAGACTTTCCCTCCCTTTCCC	23	<lmxll>	ll	125	lm	141		
>ref-127592	TTATTGAACACTAACACTCCATAACGG	7	<hkxhk>	hh	6	hk	262	kk	0
>ref-127592	TTATTGAACACTAACACTCCATAACGG	14	<nnxnp>	nn	47	np	197		
>ref-127592	TTATTGAACACTAACACTCCATAACGG	24	<nnxnp>	nn	47	np	197		
>ref-127635	CCCTTTTACACCCTCACTCCCATTCCT	24	<nnxnp>	nn	120	np	114		
>ref-127635	CCCTTTTACACCCTCACTCCCATTCCT	27	<nnxnp>	nn	118	np	114		
>ref-127640	CTCCCTGTCACCCATTCTCCCTTGCCT	24	<nnxnp>	nn	135	np	125		
>ref-127644	CTGTTCCCTACTTCTTCTCCTCTTACC	12	<lmxll>	ll	125	lm	110		
>ref-127651	TGCCTCACCACCCCATCTCCACCCAAA	27	<lmxll>	ll	124	lm	135		
>ref-127652	CAACCTAGCACCTTCTCTCCCTTCCCT	7	<nnxnp>	nn	128	np	135		
>ref-127652	CAACCTAGCACCTTCTCTCCCTTCCCT	25	<nnxnp>	nn	127	np	135		
>ref-127655	AGCCACAGGACAAGGTCTCCTGGAAAT	7	<nnxnp>	nn	265	np	0		
>ref-127670	TTCCTAATCACATCTGCTCCAGGAGAT	2	<nnxnp>	nn	3	np	239		

>ref-127689	AATACCGAAACGCGCTCTCCTCCCAGA	22	<lmxll>	ll	132	lm	107		
>ref-127721	TTACCCCTTACCCTCTCTCCTCACCCC	2	<hkxhk>	hh	0	hk	204	kk	56
>ref-127721	TTACCCCTTACCCTCTCTCCTCACCCC	27	<hkxhk>	hh	0	hk	204	kk	56
>ref-127738	GAAGCGTTCACCTGCTCTCCATAACGC	26	<nnxnp>	nn	269	np	0		
>ref-127738	GAAGCGTTCACCTGCTCTCCATAACGC	27	<nnxnp>	nn	270	np	0		
>ref-127739	ATCTCACAGACGCCGACTCCCAGGCCT	22	<nnxnp>	nn	124	np	138		
>ref-127739	ATCTCACAGACGCCGACTCCCAGGCCT	23	<nnxnp>	nn	125	np	138		
>ref-127753	CACCCCTTTACTCTTTCTCCTATCCCC	4	<lmxll>	ll	140	lm	127		
>ref-127774	ATATGTATAACACACACTCCCACACAT	15	<hkxhk>	hh	59	hk	120	kk	47
>ref-127777	CCCCATCTCACTCTCTCTCCCATCCTC	1	<nnxnp>	nn	269	np	0		
>ref-127777	CCCCATCTCACTCTCTCTCCCATCCTC	4	<nnxnp>	nn	270	np	0		
>ref-127796	ATTTATCTCACCTCTCCTCCTCCTCTT	13	<nnxnp>	nn	99	np	126		
>ref-127796	ATTTATCTCACCTCTCCTCCTCCTCTT	26	<nnxnp>	nn	251	np	1		
>ref-127802	ACCACCCCCACCTATCCTCCACCCCTT	27	<lmxll>	ll	124	lm	132		
>ref-127835	TCTTCAACCACCTCCCCTCCCTCCCTT	25	<lmxll>	ll	135	lm	125		
>ref-127836	TTATGAGGGACACCCCCTCCTTTGTTA	9	<nnxnp>	nn	257	np	2		
>ref-127836	TTATGAGGGACACCCCCTCCTTTGTTA	14	<nnxnp>	nn	163	np	90		
>ref-127836	TTATGAGGGACACCCCCTCCTTTGTTA	16	<lmxll>	ll	5	lm	261		
>ref-127836	TTATGAGGGACACCCCCTCCTTTGTTA	21	<lmxll>	ll	0	lm	272		
>ref-127836	TTATGAGGGACACCCCCTCCTTTGTTA	23	<nnxnp>	nn	265	np	2		
>ref-127836	TTATGAGGGACACCCCCTCCTTTGTTA	24	<hkxhk>	hh	0	hk	118	kk	141
>ref-127844	TCAGATACAACAACTACTCCAGCTAAG	26	<lmxll>	ll	121	lm	134		
>ref-127844	TCAGATACAACAACTACTCCAGCTAAG	27	<lmxll>	ll	120	lm	136		
>ref-127862	TAACTTCGAACTTCATCTCCGTACCCT	24	<nnxnp>	nn	129	np	122		
>ref-127862	TAACTTCGAACTTCATCTCCGTACCCT	26	<nnxnp>	nn	129	np	122		
>ref-127904	AACACACACACACTCTCTCCCTTAACA	3	<lmxll>	ll	120	lm	131		
>ref-127914	TGCCGCAAGACTGGACCTCCTGTGTCC	4	<lmxll>	ll	125	lm	130		
>ref-127922	ACCTTATCCACTCTTCCTCCCTTCTCT	4	<nnxnp>	nn	120	np	148		
>ref-127922	ACCTTATCCACTCTTCCTCCCTTCTCT	15	<nnxnp>	nn	121	np	149		
>ref-127926	TTTTCCCTCACTCCATCTCCCTCCCTT	27	<nnxnp>	nn	216	np	7		
>ref-127933	CATGAGATTACGGGATCTCCTACATCC	23	<hkxhk>	hh	0	hk	154	kk	93
>ref-127951	GCTGAATGCACTGCGACTCCTGCCCAT	4	<lmxll>	ll	118	lm	137		
>ref-127971	TTTTCGTACACTTTCCCTCCCTCTTCC	7	<nnxnp>	nn	99	np	144		
>ref-127971	TTTTCGTACACTTTCCCTCCCTCTTCC	16	<nnxnp>	nn	98	np	144		
>ref-128007	TGTTCCTCCACCCCCACTCCGGCGCCA	2	<hkxhk>	hh	51	hk	124	kk	88
>ref-128011	ATGACTTCTACAGTCTCTCCTCGTTTC	8	<lmxll>	ll	132	lm	125		
>ref-128028	AACGACCCTACAATAGCTCCTTGCTAA	14	<nnxnp>	nn	138	np	128		
>ref-128034	TCCCCTCCAACCTTCCCTCCTCCCGTT	26	<lmxll>	ll	138	lm	121		

>ref-128036	CTTTCTCTTACTCTTTCTCCTATCAGA	27	<lmxll>	ll	128	lm	110		
>ref-128048	CAAACTCCCACTTTTACTCCTGCTCCG	8	<nnxnp>	nn	105	np	116		
>ref-128077	TTCTATCACACACCCTCTCCCTCTCTT	2	<lmxll>	ll	118	lm	129		
>ref-128084	GAACCAACTACCACCACTCCTGAACCA	9	<nnxnp>	nn	109	np	143		
>ref-128104	ATCTTTCTCACCTCCTCTCCTTCATCC	4	<hkxhk>	hh	0	hk	200	kk	59
>ref-128104	ATCTTTCTCACCTCCTCTCCTTCATCC	27	<hkxhk>	hh	0	hk	200	kk	59
>ref-128121	AATCAGTCTACTCAGTCTCCTATGTTT	15	<nnxnp>	nn	124	np	114		
>ref-128152	CCTCCCCCCACTACCCCTCCTCCCACT	9	<nnxnp>	nn	151	np	95		
>ref-128152	CCTCCCCCCACTACCCCTCCTCCCACT	15	<lmxll>	ll	247	lm	9		
>ref-128152	CCTCCCCCCACTACCCCTCCTCCCACT	21	<lmxll>	ll	68	lm	168		
>ref-128154	TTGAGGACAACCTTCGCTCCGACGACA	9	<lmxll>	ll	116	lm	155		
>ref-128161	CCCTATCTCACTCTTCCTCCTCTCTTC	15	<lmxll>	ll	34	lm	217		
>ref-128161	CCCTATCTCACTCTTCCTCCTCTCTTC	23	<lmxll>	ll	18	lm	239		
>ref-128161	CCCTATCTCACTCTTCCTCCTCTCTTC	26	<lmxll>	ll	38	lm	215		
>ref-128192	CATACACACACACATACTCCTAGTATA	1	<hkxhk>	hh	0	hk	273	kk	0
>ref-128192	CATACACACACACATACTCCTAGTATA	21	<hkxhk>	hh	0	hk	273	kk	0
>ref-128207	GCCCCTCCCACTGCTCCTCCAGCGTTC	1	<lmxll>	ll	10	lm	263		
>ref-128207	GCCCCTCCCACTGCTCCTCCAGCGTTC	22	<lmxll>	ll	10	lm	263		
>ref-128229	TCCTCTTAAACTCTACCTCCCTCCCTT	12	<lmxll>	ll	149	lm	102		
>ref-128229	TCCTCTTAAACTCTACCTCCCTCCCTT	23	<lmxll>	ll	148	lm	103		
>ref-128247	AGGCCCAGGACATCGCCTCCTCCTTCA	25	<hkxhk>	hh	0	hk	273	kk	0
>ref-128247	AGGCCCAGGACATCGCCTCCTCCTTCA	27	<hkxhk>	hh	0	hk	273	kk	0
>ref-128264	CTCTCCTGAACCCCCCCTCCTTCCTTC	3	<lmxll>	ll	227	lm	0		
>ref-128265	CTGACCAGCACACTGACTCCAAGCCTG	8	<lmxll>	ll	117	lm	131		
>ref-128270	TATCTCCATACCCGTGCTCCTCTTATG	1	<lmxll>	ll	121	lm	134		
>ref-128278	CTTCCCCACACCTCTTCTCCTCCTGTT	8	<hkxhk>	hh	40	hk	207	kk	0
>ref-128278	CTTCCCCACACCTCTTCTCCTCCTGTT	25	<hkxhk>	hh	39	hk	207	kk	0
>ref-128284	TTTCTACTGACCCCTTCTCCCCTCCCC	6	<nnxnp>	nn	19	np	214		
>ref-128284	TTTCTACTGACCCCTTCTCCCCTCCCC	9	<nnxnp>	nn	19	np	217		
>ref-128322	TGTCTCTCCACCAACCCTCCATTCACA	2	<nnxnp>	nn	120	np	129		
>ref-128322	TGTCTCTCCACCAACCCTCCATTCACA	15	<lmxll>	ll	118	lm	131		
>ref-128332	TAGGTTCTTACAGACCCTCCTTGAAGA	16	<nnxnp>	nn	123	np	140		
>ref-128356	CCCCGCTTTACCTACCCTCCCTTCCAA	24	<lmxll>	ll	119	lm	142		
>ref-128366	AACCCTAATACATGCCCTCCATAACAC	25	<lmxll>	ll	132	lm	133		
>ref-128389	CCTTCTTCAACCATCACTCCTTCTTCT	16	<lmxll>	ll	105	lm	128		
>ref-128396	GGAGGTAATACATACACTCCTTTACTC	6	<lmxll>	ll	116	lm	129		
>ref-128412	CATACACTTACCATGGCTCCATCCTTC	24	<nnxnp>	nn	121	np	137		
>ref-128412	CATACACTTACCATGGCTCCATCCTTC	25	<lmxll>	ll	263	lm	0		

>ref-128422	CTCTCTCCAACCCTTCCTCCCTCCACC	14	<nnxnp>	nn	15	np	230		
>ref-128439	ACCCCCCAAACTTTCCCTCCTTCCCTC	2	<lmxll>	ll	176	lm	75		
>ref-128439	ACCCCCCAAACTTTCCCTCCTTCCCTC	12	<lmxll>	ll	0	lm	260		
>ref-128467	CGAAGACACACTGTTCCTCCTTCTACA	13	<lmxll>	ll	135	lm	132		
>ref-128510	GGTGGTTTAACGTTGCCTCCCTTGATA	21	<nnxnp>	nn	129	np	137		
>ref-128524	CTTTTCCCTACCTTCCCTCCCACATCT	23	<nnxnp>	nn	145	np	104		
>ref-128527	ATCAAGTTAACCTTTGCTCCGTTCCCT	3	<lmxll>	ll	129	lm	138		
>ref-128568	CAATTTCAAACATAAACTCCACAGCGA	7	<hkxhk>	hh	54	hk	124	kk	50
>ref-128572	ATCTTGTAGACTGATCCTCCCAGCTTT	7	<lmxll>	ll	124	lm	115		
>ref-128594	CCTCCCGCGACAGAGCCTCCGCGTCTC	25	<nnxnp>	nn	137	np	136		
>ref-128602	CAATATCTTACACACACTCCCACACTG	7	<nnxnp>	nn	111	np	152		
>ref-128610	CCCTCCGACACCCCCCCTCCCCCAATC	16	<nnxnp>	nn	120	np	114		
>ref-128619	TACTTTGCTACTGTTCCTCCTCCTCCT	1	<hkxhk>	hh	0	hk	268	kk	4
>ref-128619	TACTTTGCTACTGTTCCTCCTCCTCCT	7	<hkxhk>	hh	4	hk	268	kk	0
>ref-128625	CTCTGATCTACATATCCTCCTTCGTCC	5	<hkxhk>	hh	59	hk	148	kk	62
>ref-128630	TCTTTCCCTACTATCACTCCCCTCCCT	7	<lmxll>	ll	115	lm	131		
>ref-128634	CCTGGCCTTACTCCCACTCCCTCCTCC	12	<lmxll>	ll	130	lm	121		
>ref-128646	GGAAACCAAACTTGCTCTCCGGATTAT	24	<hkxhk>	hh	64	hk	124	kk	55
>ref-128683	AATTTTCCTACATTTTCTCCAGTAAAA	4	<hkxhk>	hh	17	hk	219	kk	0
>ref-128684	TGTGTCCCGACCTCTGCTCCCGCCAAG	4	<lmxll>	ll	14	lm	251		
>ref-128684	TGTGTCCCGACCTCTGCTCCCGCCAAG	21	<lmxll>	ll	0	lm	272		
>ref-128690	TCACAATATACATCATCTCCATAAGAT	4	<nnxnp>	nn	143	np	94		
>ref-128696	TTCCTCTCGACCCGCACTCCATTTTAC	6	<nnxnp>	nn	144	np	115		
>ref-128697	GTTTCCTTGACCATGTCTCCTCATTCA	1	<hkxhk>	hh	1	hk	260	kk	3
>ref-128697	GTTTCCTTGACCATGTCTCCTCATTCA	27	<hkxhk>	hh	3	hk	260	kk	1
>ref-128701	TGCTCACTGACAAAGCCTCCTCGAAGT	15	<nnxnp>	nn	73	np	170		
>ref-128701	TGCTCACTGACAAAGCCTCCTCGAAGT	26	<hkxhk>	hh	0	hk	234	kk	25
>ref-128703	CTCCTTATCACTCGTCCTCCTTCTTCC	24	<nnxnp>	nn	107	np	145		
>ref-128712	ACGAGTAGCACGGCACCTCCAAACTAT	14	<lmxll>	ll	115	lm	152		
>ref-128722	CTCTATCCCACTTCCTCTCCTCTTTAT	3	<lmxll>	ll	110	lm	125		
>ref-128726	CAGTTGTTTACAAACCCTCCTGCACCA	1	<hkxhk>	hh	67	hk	195	kk	0
>ref-128726	CAGTTGTTTACAAACCCTCCTGCACCA	24	<hkxhk>	hh	67	hk	194	kk	0
>ref-128731	AAAACACCAACGGCTCCTCCGACGCAG	14	<lmxll>	ll	124	lm	126		
>ref-128731	AAAACACCAACGGCTCCTCCGACGCAG	26	<lmxll>	ll	124	lm	126		
>ref-128766	ATGGCTATCACATGATCTCCCCTCGTA	5	<nnxnp>	nn	131	np	140		
>ref-128766	ATGGCTATCACATGATCTCCCCTCGTA	6	<nnxnp>	nn	131	np	140		
>ref-128791	CACCGCAGTACAACTTCTCCAGCTGTC	24	<lmxll>	ll	133	lm	120		
>ref-128794	CCCTCACCCACTCTCTCTCCTAACACA	5	<nnxnp>	nn	130	np	136		

>ref-128800	TCACAACACACCTGCCCTCCGTCCATA	21	<lmxll>	ll	137	lm	131		
>ref-128804	CATCCATTTACTCACACTCCTCAAGTA	13	<lmxll>	ll	56	lm	205		
>ref-128804	CATCCATTTACTCACACTCCTCAAGTA	15	<lmxll>	ll	1	lm	270		
>ref-128804	CATCCATTTACTCACACTCCTCAAGTA	21	<nnxnp>	nn	37	np	221		
>ref-128804	CATCCATTTACTCACACTCCTCAAGTA	26	<nnxnp>	nn	37	np	221		
>ref-128818	TTTGGTGCAACGTTGCCTCCTTGTCCT	21	<lmxll>	ll	139	lm	134		
>ref-128820	CACCTTTTGACCCATCCTCCAGCAGCT	6	<hkxhk>	hh	1	hk	270	kk	0
>ref-128820	CACCTTTTGACCCATCCTCCAGCAGCT	7	<hkxhk>	hh	1	hk	270	kk	0
>ref-128827	AGAAAGCCAACAGCGACTCCACAACAG	4	<lmxll>	ll	149	lm	118		
>ref-128827	AGAAAGCCAACAGCGACTCCACAACAG	15	<lmxll>	ll	149	lm	118		
>ref-128839	TATTTTCATACTCCTTCTCCTTCCCCT	24	<nnxnp>	nn	250	np	2		
>ref-128853	CCTTCCCTTACTCCTCCTCCTCCCGAC	14	<hkxhk>	hh	6	hk	259	kk	3
>ref-128853	CCTTCCCTTACTCCTCCTCCTCCCGAC	26	<hkxhk>	hh	6	hk	259	kk	3
>ref-128857	GAGCGGGCGACGGGGCCTCCGCTAGAT	5	<lmxll>	ll	113	lm	158		
>ref-128859	TTTCTTTCAACTCTCACTCCTTACTCT	5	<nnxnp>	nn	97	np	137		
>ref-128873	CCTCATTTTACCCAAGCTCCCTCCCTC	25	<lmxll>	ll	149	lm	93		
>ref-128882	CCAAGATGCACTTAACCTCCTTCTAAC	6	<hkxhk>	hh	47	hk	142	kk	68
>ref-128882	CCAAGATGCACTTAACCTCCTTCTAAC	27	<nnxnp>	nn	109	np	149		
>ref-128883	TCCCTCTTCACCTCCTCTCCTTCTCTA	23	<lmxll>	ll	148	lm	95		
>ref-128883	TCCCTCTTCACCTCCTCTCCTTCTCTA	27	<lmxll>	ll	144	lm	101		
>ref-128893	CCCCCTCCGACGCCAGCTCCGCCGCCT	27	<lmxll>	ll	139	lm	113		
>ref-128905	ATCCCCCTCACCCTCCCTCCGCATCTC	7	<nnxnp>	nn	111	np	150		
>ref-128915	GGCTCAGTCACCGCTCCTCCCTTCTTC	22	<hkxhk>	hh	63	hk	146	kk	50
>ref-128920	CGATCACCAACGCTTTCTCCATTGCAG	7	<nnxnp>	nn	122	np	137		
>ref-128920	CGATCACCAACGCTTTCTCCATTGCAG	8	<nnxnp>	nn	122	np	137		
>ref-128922	CCTTCAGCCACTCTGTCTCCAGCGTGG	3	<lmxll>	ll	114	lm	140		
>ref-128930	TAGGAGCACACCTTAGCTCCCTCTGTC	16	<nnxnp>	nn	137	np	116		
>ref-128945	CAATTTATCACTCCGTCTCCTTCTTCT	5	<hkxhk>	hh	2	hk	268	kk	0
>ref-128945	CAATTTATCACTCCGTCTCCTTCTTCT	8	<hkxhk>	hh	2	hk	268	kk	0
>ref-128971	CTTCCTCCCACAGGCTCTCCTCCTTCT	13	<nnxnp>	nn	235	np	7		
>ref-128971	CTTCCTCCCACAGGCTCTCCTCCTTCT	16	<nnxnp>	nn	245	np	4		
>ref-128990	ACACCTATCACGAGACCTCCTTGTCAC	1	<hkxhk>	hh	1	hk	269	kk	0
>ref-128990	ACACCTATCACGAGACCTCCTTGTCAC	7	<hkxhk>	hh	1	hk	261	kk	5
>ref-128993	AATCGCTTAACCCTTACTCCATCTGGA	16	<lmxll>	ll	91	lm	150		
>ref-128993	AATCGCTTAACCCTTACTCCATCTGGA	27	<nnxnp>	nn	261	np	0		
>ref-129014	TCTCATTTTACCCTTTCTCCTTTACCC	1	<nnxnp>	nn	125	np	102		
>ref-129036	TTATTACAAACTTCCTCTCCCGGATTC	15	<lmxll>	ll	113	lm	121		
>ref-129037	TAGCGCCTTACTGCGACTCCCTCCGGT	12	<lmxll>	ll	135	lm	133		

>ref-129050	CCAAGAAGGACCGAAACTCCAACCGGA	3	<nnxnp>	nn	234	np	0		
>ref-129055	TCCACCCACACCATCCCTCCCTCCCTC	3	<nnxnp>	nn	5	np	264		
>ref-129055	TCCACCCACACCATCCCTCCCTCCCTC	4	<nnxnp>	nn	4	np	266		
>ref-129055	TCCACCCACACCATCCCTCCCTCCCTC	7	<lmxll>	ll	196	lm	56		
>ref-129055	TCCACCCACACCATCCCTCCCTCCCTC	23	<nnxnp>	nn	143	np	114		
>ref-129079	ATACCACGAACAAGCCCTCCCATACCA	1	<hkxhk>	hh	0	hk	272	kk	0
>ref-129079	ATACCACGAACAAGCCCTCCCATACCA	25	<hkxhk>	hh	0	hk	247	kk	17
>ref-129091	TACTTATTCACTATCCCTCCTTCCTTT	6	<lmxll>	ll	248	lm	0		
>ref-129092	GCCATGGCAACGCTCACTCCACACTAG	23	<lmxll>	ll	115	lm	135		
>ref-129099	TTTGTTCCTACTCCTCCTCCTTCTTTA	1	<hkxhk>	hh	5	hk	215	kk	31
>ref-129103	ATCTGCCAAACTTTATCTCCGTCTCTG	5	<lmxll>	ll	144	lm	112		
>ref-129104	TAAACCACCACAACCTCTCCCTACCAC	15	<nnxnp>	nn	140	np	101		
>ref-129104	TAAACCACCACAACCTCTCCCTACCAC	25	<nnxnp>	nn	104	np	138		
>ref-129150	TCTCGGCCCACTGTACCTCCCCTCTTT	7	<nnxnp>	nn	129	np	133		
>ref-129156	CCTCCCTCCACTCCTCCTCCCACACAC	12	<nnxnp>	nn	126	np	130		
>ref-129159	TTTGTGCGAACGGCTGCTCCAGGAGTT	5	<lmxll>	ll	4	lm	267		
>ref-129159	TTTGTGCGAACGGCTGCTCCAGGAGTT	13	<lmxll>	ll	5	lm	266		
>ref-129163	GTTGAATCAACGTTCTCTCCATACACC	8	<nnxnp>	nn	247	np	0		
>ref-129163	GTTGAATCAACGTTCTCTCCATACACC	26	<nnxnp>	nn	210	np	32		
>ref-129179	TCTTTTTAAACGTCCCCTCCATTTGAT	24	<nnxnp>	nn	122	np	131		
>ref-129204	CTTCCTTGAACCATCTCTCCTTTACGT	4	<lmxll>	ll	122	lm	149		
>ref-129209	GTTGACATCACTCCCCCTCCCCCGCCT	8	<lmxll>	ll	138	lm	126		
>ref-129209	GTTGACATCACTCCCCCTCCCCCGCCT	13	<lmxll>	ll	138	lm	126		
>ref-129222	CTTTCTTAGACCACTTCTCCCTATCAA	1	<lmxll>	ll	98	lm	143		
>ref-129222	CTTTCTTAGACCACTTCTCCCTATCAA	3	<lmxll>	ll	101	lm	141		
>ref-129229	TATGCAGGCACCGAGCCTCCTCTCATT	15	<nnxnp>	nn	140	np	124		
>ref-129239	AAAAAGAGTACCCAGCCTCCTGCCTTC	27	<lmxll>	ll	113	lm	137		
>ref-129259	GCTGCCCTCACCCCCTCTCCCCTCTCT	1	<lmxll>	ll	2	lm	267		
>ref-129259	GCTGCCCTCACCCCCTCTCCCCTCTCT	13	<nnxnp>	nn	273	np	0		
>ref-129269	CCCTCTAACACACAATCTCCGCTTCTA	2	<lmxll>	ll	123	lm	140		
>ref-129293	TCGTACAAGACCCGATCTCCGTTTTCT	6	<lmxll>	ll	136	lm	117		
>ref-129299	AACATTCCTACTGTTGCTCCCCTGATC	6	<hkxhk>	hh	53	hk	121	kk	55
>ref-129299	AACATTCCTACTGTTGCTCCCCTGATC	24	<hkxhk>	hh	53	hk	121	kk	55
>ref-129305	CCCTGCTGAACCCTCTCTCCGAAGCCC	24	<nnxnp>	nn	157	np	108		
>ref-129322	GGCCCTTGAACCCAACCTCCTTTTCCA	4	<lmxll>	ll	139	lm	118		
>ref-129327	TCTACTCCTACTCTTCCTCCGCCCTTC	23	<lmxll>	ll	118	lm	146		
>ref-129343	TCCCTCAAGACAAACCCTCCTCCTCGC	2	<lmxll>	ll	144	lm	110		
>ref-129344	CACATCACTACACACGCTCCTATAGAC	1	<hkxhk>	hh	0	hk	273	kk	0

>ref-129353	GGACATACTACCTCACCTCCATAGGTG	4	<nnxnp>	nn	125	np	130		
>ref-129353	GGACATACTACCTCACCTCCATAGGTG	8	<hkxhk>	hh	0	hk	201	kk	58
>ref-129353	GGACATACTACCTCACCTCCATAGGTG	9	<hkxhk>	hh	0	hk	254	kk	13
>ref-129353	GGACATACTACCTCACCTCCATAGGTG	14	<lmxll>	ll	31	lm	225		
>ref-129353	GGACATACTACCTCACCTCCATAGGTG	23	<hkxhk>	hh	12	hk	254	kk	0
>ref-129353	GGACATACTACCTCACCTCCATAGGTG	24	<nnxnp>	nn	262	np	7		
>ref-129361	TTCCTCCCCACTTTCCCTCCCCCTTTC	13	<nnxnp>	nn	150	np	81		
>ref-129361	TTCCTCCCCACTTTCCCTCCCCCTTTC	15	<nnxnp>	nn	127	np	106		
>ref-129376	TATCGATACACCCCATCTCCTTTCATC	4	<hkxhk>	hh	65	hk	113	kk	45
>ref-129380	AATCCACCTACTACGCCTCCTCCTTCT	12	<lmxll>	ll	128	lm	135		
>ref-129387	CCTCACTAAACTAACTCTCCCACGATC	7	<hkxhk>	hh	0	hk	262	kk	6
>ref-129399	GAAAAGACAACATTGACTCCCTCTTGA	12	<lmxll>	ll	134	lm	113		
>ref-129399	GAAAAGACAACATTGACTCCCTCTTGA	27	<nnxnp>	nn	263	np	0		
>ref-129411	CACACCCCCACTTCCACTCCCTCCCCA	25	<hkxhk>	hh	90	hk	136	kk	5
>ref-129420	GACAGACAGACACTAACTCCACTAGAT	5	<lmxll>	ll	111	lm	143		
>ref-129471	TTGACGACCACCACGACTCCGCCTCCG	2	<lmxll>	ll	210	lm	13		
>ref-129471	TTGACGACCACCACGACTCCGCCTCCG	3	<lmxll>	ll	225	lm	1		
>ref-129478	AGCAGAGGCACTATATCTCCCTACGGT	26	<nnxnp>	nn	150	np	120		
>ref-129479	TCCTCTGTCACCCTCCCTCCTCCTTTC	7	<nnxnp>	nn	137	np	94		
>ref-129479	TCCTCTGTCACCCTCCCTCCTCCTTTC	16	<nnxnp>	nn	150	np	94		
>ref-129485	GAAGCAGACACATAAGCTCCTTAGATT	13	<nnxnp>	nn	171	np	72		
>ref-129486	GATGGCTCTACAAGTGCTCCGTCACCA	1	<nnxnp>	nn	242	np	23		
>ref-129486	GATGGCTCTACAAGTGCTCCGTCACCA	21	<lmxll>	ll	3	lm	270		
>ref-129486	GATGGCTCTACAAGTGCTCCGTCACCA	22	<lmxll>	ll	3	lm	270		
>ref-129486	GATGGCTCTACAAGTGCTCCGTCACCA	23	<nnxnp>	nn	242	np	23		
>ref-129508	TGCGCACACACTCTAGCTCCGTATCTC	16	<hkxhk>	hh	52	hk	145	kk	53
>ref-129526	TTACGAGGCACTGTGTCTCCTTTGGAC	14	<lmxll>	ll	157	lm	115		
>ref-129528	ACTCTACGTACTCTAACTCCTCTCTCG	14	<lmxll>	ll	118	lm	142		
>ref-129540	CACCCACGCACTCACACTCCACTCACA	8	<hkxhk>	hh	0	hk	132	kk	122
>ref-129540	CACCCACGCACTCACACTCCACTCACA	26	<hkxhk>	hh	0	hk	135	kk	120
>ref-129542	TCAACTCGTACTTACTCTCCTTATGCA	2	<lmxll>	ll	145	lm	103		
>ref-129542	TCAACTCGTACTTACTCTCCTTATGCA	26	<lmxll>	ll	145	lm	104		
>ref-129551	GCCACACTCACATGACCTCCTGGGAGG	23	<lmxll>	ll	138	lm	118		
>ref-129587	CACAAACACACATAACCTCCCCCTCAC	1	<lmxll>	ll	2	lm	264		
>ref-129587	CACAAACACACATAACCTCCCCCTCAC	24	<hkxhk>	hh	1	hk	210	kk	57
>ref-129587	CACAAACACACATAACCTCCCCCTCAC	26	<lmxll>	ll	2	lm	269		
>ref-129589	TTTGCTAAGACTGGAACTCCACTTCAG	3	<nnxnp>	nn	6	np	234		
>ref-129589	TTTGCTAAGACTGGAACTCCACTTCAG	27	<nnxnp>	nn	6	np	234		

>ref-129593	GAGTGTGTGACTTACGCTCCTCTCACA	24	<lmxll>	ll	125	lm	140		
>ref-129599	ACTCAGAACACACCTTCTCCTAGAGTG	25	<nnxnp>	nn	122	np	138		
>ref-129605	CCTCTTCTCACCCCTTCTCCCTCTTTC	2	<hkxhk>	hh	0	hk	272	kk	0
>ref-129605	CCTCTTCTCACCCCTTCTCCCTCTTTC	6	<hkxhk>	hh	0	hk	273	kk	0
>ref-129605	CCTCTTCTCACCCCTTCTCCCTCTTTC	25	<nnxnp>	nn	65	np	191		
>ref-129613	TGGAGGGTCACGCGGCCTCCACGCCTG	23	<lmxll>	ll	132	lm	140		
>ref-129638	CTTCTGCATACCCCCTCTCCTCTCCCC	2	<hkxhk>	hh	15	hk	240	kk	3
>ref-129638	CTTCTGCATACCCCCTCTCCTCTCCCC	8	<hkxhk>	hh	2	hk	13	kk	244
>ref-129639	GCAGGGTCGACTACGACTCCTCCCCCA	9	<nnxnp>	nn	242	np	3		
>ref-129645	AGATCCGGGACAAAAGCTCCTCCCGCC	27	<nnxnp>	nn	122	np	135		
>ref-129663	TCTCTCTACACCCTTTCTCCTATCCCC	8	<lmxll>	ll	84	lm	158		
>ref-129663	TCTCTCTACACCCTTTCTCCTATCCCC	22	<lmxll>	ll	85	lm	157		
>ref-129716	TCCCTTCCTACCTCTACTCCTTGTTTT	6	<hkxhk>	hh	46	hk	133	kk	71
>ref-129716	TCCCTTCCTACCTCTACTCCTTGTTTT	14	<hkxhk>	hh	46	hk	133	kk	71
>ref-129724	TTTTTTCTCACGCCATCTCCCTCTCCC	21	<hkxhk>	hh	45	hk	147	kk	48
>ref-129744	CGCCCGCCCACCGTGCCTCCCGCATCC	9	<lmxll>	ll	131	lm	139		
>ref-129758	ATGTCTTTCACTCTCCCTCCCTTGTTT	4	<lmxll>	ll	67	lm	182		
>ref-129758	ATGTCTTTCACTCTCCCTCCCTTGTTT	22	<lmxll>	ll	67	lm	182		
>ref-129764	AAAAATGCCACGTTAACTCCCCACGAA	1	<nnxnp>	nn	99	np	143		
>ref-129764	AAAAATGCCACGTTAACTCCCCACGAA	2	<nnxnp>	nn	99	np	143		
>ref-129778	CATTCAGACACAGCTGCTCCTCGCACT	22	<nnxnp>	nn	124	np	146		
>ref-129787	CCTCCTCCAACACTAACTCCACCATAA	14	<lmxll>	ll	135	lm	132		
>ref-129806	GATCACGACACATGGACTCCCAAAGAT	9	<hkxhk>	hh	15	hk	234	kk	0
>ref-129806	GATCACGACACATGGACTCCCAAAGAT	16	<lmxll>	ll	156	lm	92		
>ref-129859	AATATCACAACAGCGTCTCCGTCATGT	4	<hkxhk>	hh	0	hk	271	kk	1
>ref-129891	CTATCTCTTACAATCACTCCTCCATTC	3	<hkxhk>	hh	69	hk	132	kk	44
>ref-129891	CTATCTCTTACAATCACTCCTCCATTC	15	<hkxhk>	hh	69	hk	132	kk	44
>ref-129897	TCTGCCCTTACTCTTTCTCCTCTTCTT	24	<nnxnp>	nn	147	np	102		
>ref-129897	TCTGCCCTTACTCTTTCTCCTCTTCTT	25	<nnxnp>	nn	146	np	102		
>ref-129945	TTTCCTGTCACGAATACTCCGCCGTGC	13	<nnxnp>	nn	108	np	151		
>ref-129954	TCTTCTCCCACTCGATCTCCCCACCCC	14	<nnxnp>	nn	122	np	138		
>ref-129995	TACACAACCACCTTTTCTCCTTTCTTC	2	<hkxhk>	hh	12	hk	248	kk	0
>ref-129995	TACACAACCACCTTTTCTCCTTTCTTC	12	<hkxhk>	hh	11	hk	250	kk	0
>ref-130021	CACATACGTACATACACTCCTATGCGT	9	<nnxnp>	nn	124	np	143		
>ref-130021	CACATACGTACATACACTCCTATGCGT	24	<nnxnp>	nn	124	np	143		
>ref-130027	ATTTCATTCACTCCTTCTCCCTCTCCT	25	<nnxnp>	nn	135	np	116		
>ref-130027	ATTTCATTCACTCCTTCTCCCTCTCCT	27	<nnxnp>	nn	131	np	120		
>ref-130085	CGCTTGCTCACGCTCTCTCCCTCTCCC	9	<lmxll>	ll	137	lm	134		

>ref-130121	TTTAATTCAACTGTGCCTCCTTTTCGC	6	<lmxll>	ll	167	lm	70		
>ref-130121	TTTAATTCAACTGTGCCTCCTTTTCGC	12	<nnxnp>	nn	178	np	81		
>ref-130131	TTCCTCTCCACTCTTCCTCCTCCTTGC	22	<nnxnp>	nn	141	np	125		
>ref-130143	GCTCGTGGTACAACCACTCCACCCGTA	1	<nnxnp>	nn	269	np	0		
>ref-130157	GTTTTAGTTACTACAACTCCAAGTCCT	2	<hkxhk>	hh	8	hk	246	kk	1
>ref-130161	ACCGCTTCAACTCCCTCTCCCTCACTC	22	<hkxhk>	hh	0	hk	182	kk	83
>ref-130162	ACCCCATTTACCGCATCTCCTACATCT	6	<nnxnp>	nn	116	np	138		
>ref-130162	ACCCCATTTACCGCATCTCCTACATCT	24	<lmxll>	ll	111	lm	142		
>ref-130166	ATCATATCTACCTTCTCTCCGCCTTTA	9	<lmxll>	ll	126	lm	135		
>ref-130168	GCCCTTGCAACCGCCACTCCGGGACTG	14	<hkxhk>	hh	62	hk	141	kk	45
>ref-130171	TCATCAACCACCCCCCCTCCGCCCTAT	2	<hkxhk>	hh	127	hk	119	kk	0
>ref-130176	TTAGTGATGACATTAGCTCCTGCAAAA	4	<nnxnp>	nn	120	np	120		
>ref-130183	CAATTCCAAACGTGGCCTCCCTTGGCT	3	<hkxhk>	hh	69	hk	126	kk	64
>ref-130195	GAAGCTCGCACTGGCTCTCCCGCGGCG	8	<hkxhk>	hh	48	hk	147	kk	69
>ref-130201	TCAATCACCACCATTCCTCCGTCCCCT	16	<nnxnp>	nn	132	np	130		
>ref-130201	TCAATCACCACCATTCCTCCGTCCCCT	26	<nnxnp>	nn	131	np	133		
>ref-130231	CAGTCACTCACCTTTTCTCCCCATCTC	7	<lmxll>	ll	124	lm	131		
>ref-130231	CAGTCACTCACCTTTTCTCCCCATCTC	22	<nnxnp>	nn	138	np	123		
>ref-130239	CATACACATACGCACACTCCTCTCTCT	1	<hkxhk>	hh	0	hk	273	kk	0
>ref-130239	CATACACATACGCACACTCCTCTCTCT	3	<hkxhk>	hh	0	hk	273	kk	0
>ref-130241	GTGCTCTCCACCCTCCCTCCCGTTCTC	6	<lmxll>	ll	267	lm	0		
>ref-130241	GTGCTCTCCACCCTCCCTCCCGTTCTC	25	<lmxll>	ll	137	lm	122		
>ref-130245	TTTCCTTCAACTTTCTCTCCCCTCACT	5	<lmxll>	ll	134	lm	108		
>ref-130245	TTTCCTTCAACTTTCTCTCCCCTCACT	25	<lmxll>	ll	128	lm	110		
>ref-130247	TGCCTCCAAACATCCCCTCCCTCCTAC	3	<nnxnp>	nn	114	np	153		
>ref-130253	TGACCCCTCACCTGCTCTCCCAGGCTC	22	<hkxhk>	hh	47	hk	139	kk	59
>ref-130253	TGACCCCTCACCTGCTCTCCCAGGCTC	24	<hkxhk>	hh	57	hk	140	kk	53
>ref-130277	AATCTTAACACAGCCTCTCCTTGTCTT	6	<lmxll>	ll	100	lm	149		
>ref-130286	TGAATTCAAACAACAACTCCACCCCAA	15	<nnxnp>	nn	83	np	147		
>ref-130286	TGAATTCAAACAACAACTCCACCCCAA	23	<nnxnp>	nn	83	np	148		
>ref-130297	ATAAGAGCAACAACAACTCCACCGTTA	7	<nnxnp>	nn	125	np	144		
>ref-130323	CACACCCAAACAATCACTCCCTCTCGC	14	<nnxnp>	nn	223	np	0		
>ref-130324	CTCCAGCCAACAACCCCTCCTCTCTTT	26	<hkxhk>	hh	56	hk	132	kk	52
>ref-130325	CCCACTCTAACACTGTCTCCATGTCCT	1	<nnxnp>	nn	106	np	140		
>ref-130325	CCCACTCTAACACTGTCTCCATGTCCT	9	<nnxnp>	nn	111	np	139		
>ref-130346	AGTAGATTTACATTCTCTCCTCTCTCT	8	<hkxhk>	hh	49	hk	131	kk	77
>ref-130346	AGTAGATTTACATTCTCTCCTCTCTCT	21	<nnxnp>	nn	261	np	3		
>ref-130373	TAGCAGGGTACATTATCTCCTTTTCCC	14	<hkxhk>	hh	46	hk	135	kk	57

>ref-130382	TACAAAGCGACTTCAGCTCCGCAGACA	2	<nnxnp>	nn	139	np	124		
>ref-130390	CTCTCCTATACATTATCTCCCTTTTCC	4	<lmxll>	ll	105	lm	143		
>ref-130402	AGAAATCCAACTCGTGCTCCTTGATCA	22	<lmxll>	ll	122	lm	134		
>ref-130416	TTCACCCGCACCCCACCTCCCCCTCTC	7	<lmxll>	ll	117	lm	127		
>ref-130419	CCATCCTCCACTCTACCTCCTCTTCCC	21	<nnxnp>	nn	135	np	135		
>ref-130441	CGGGCGGGGACAACATCTCCGGGTGTC	2	<nnxnp>	nn	269	np	0		
>ref-130444	TTCTTGTTTACATCCCCTCCTCATTTA	2	<lmxll>	ll	110	lm	122		
>ref-130449	GTACTCTGTACGATGACTCCATGGACA	2	<hkxhk>	hh	58	hk	145	kk	60
>ref-130490	TTCTCCTCTACCCTCTCTCCCTACCAC	23	<nnxnp>	nn	256	np	2		
>ref-130490	TTCTCCTCTACCCTCTCTCCCTACCAC	26	<nnxnp>	nn	256	np	0		
>ref-130505	GCCACTACTACACCATCTCCGTTTGCA	22	<hkxhk>	hh	78	hk	137	kk	43
>ref-130510	TGTATATACACATAGGCTCCACCTGCC	15	<nnxnp>	nn	139	np	134		
>ref-130522	TGCACATACACAACCCCTCCCCACACT	4	<nnxnp>	nn	272	np	0		
>ref-130524	TACATTCCTACGTAGCCTCCTACAGTA	21	<lmxll>	ll	138	lm	88		
>ref-130524	TACATTCCTACGTAGCCTCCTACAGTA	27	<lmxll>	ll	138	lm	87		
>ref-130531	TCAACACCTACCCTTCCTCCTCCTTCT	8	<hkxhk>	hh	10	hk	257	kk	0
>ref-130531	TCAACACCTACCCTTCCTCCTCCTTCT	26	<hkxhk>	hh	0	hk	257	kk	10
>ref-130539	TCTAAAAGAACCTGCTCTCCCAAGTCT	4	<nnxnp>	nn	130	np	119		
>ref-130547	TTGCCCCTTACCTTCACTCCTTTAGCA	23	<nnxnp>	nn	268	np	0		
>ref-130549	GGGAGGACTACCCTAACTCCTGACAAA	16	<lmxll>	ll	108	lm	139		
>ref-130549	GGGAGGACTACCCTAACTCCTGACAAA	25	<lmxll>	ll	108	lm	139		
>ref-130588	ACTCACCCCACCTTCCCTCCCTTGCAG	12	<nnxnp>	nn	127	np	143		
>ref-130588	ACTCACCCCACCTTCCCTCCCTTGCAG	15	<nnxnp>	nn	138	np	129		
>ref-130618	TGGCCAGGAACACGCGCTCCTGCTTCT	14	<lmxll>	ll	124	lm	146		
>ref-130652	CACGCACGCACACGAGCTCCCTTAGAT	8	<nnxnp>	nn	137	np	134		
>ref-130652	CACGCACGCACACGAGCTCCCTTAGAT	12	<nnxnp>	nn	138	np	133		
>ref-130664	TTATCCTTCACTTCCTCTCCACCCTTT	2	<lmxll>	ll	126	lm	120		
>ref-130671	CATCACTTGACTCATCCTCCTCGCTTA	1	<nnxnp>	nn	177	np	81		
>ref-130671	CATCACTTGACTCATCCTCCTCGCTTA	15	<hkxhk>	hh	1	hk	270	kk	0
>ref-130671	CATCACTTGACTCATCCTCCTCGCTTA	22	<hkxhk>	hh	2	hk	271	kk	0
>ref-130690	AAGTATAACACGAGAGCTCCTTGAATA	24	<lmxll>	ll	116	lm	141		
>ref-130696	ACACCTCTTACCTTCCCTCCCTCCTCT	6	<hkxhk>	hh	104	hk	143	kk	3
>ref-130719	TGATCAAGCACTCGCTCTCCCTCGCAC	2	<lmxll>	ll	126	lm	141		
>ref-130719	TGATCAAGCACTCGCTCTCCCTCGCAC	23	<nnxnp>	nn	133	np	136		
>ref-130723	TCTTCCCCTACTCGCGCTCCATCCCCC	3	<lmxll>	ll	125	lm	137		
>ref-130728	ACTTTATAAACCTGCTCTCCTCCTTGT	24	<hkxhk>	hh	56	hk	133	kk	55
>ref-130734	TGCAAATAAACCAGGCCTCCACATTCC	5	<nnxnp>	nn	263	np	0		
>ref-130761	CGCCTCCATACGTCCCCTCCCTGGTCT	15	<hkxhk>	hh	65	hk	139	kk	66

>ref-130765	GACTGACTGACTGTCTCTCCAACCGAC	1	<hkxhk>	hh	62	hk	140	kk	49
>ref-130766	ATTCCGCAAACTGACTCTCCCTTTTTC	1	<hkxhk>	hh	79	hk	169	kk	0
>ref-130766	ATTCCGCAAACTGACTCTCCCTTTTTC	5	<nnxnp>	nn	163	np	97		
>ref-130790	CAGTCTTCCACCCTCTCTCCTTCCCGA	23	<hkxhk>	hh	51	hk	131	kk	52
>ref-130801	GGCGCCGCAACAATCTCTCCTACAATC	5	<nnxnp>	nn	272	np	0		
>ref-130802	CCTGTCTATACCTACACTCCTGCCTAT	22	<lmxll>	ll	125	lm	123		
>ref-130852	GAAAAGGAGACTGCTCCTCCTCTTCCT	14	<nnxnp>	nn	260	np	0		
>ref-130852	GAAAAGGAGACTGCTCCTCCTCTTCCT	24	<lmxll>	ll	106	lm	143		
>ref-130863	CATCTCCTGACCCCTTCTCCTGACCCC	2	<nnxnp>	nn	173	np	87		
>ref-130863	CATCTCCTGACCCCTTCTCCTGACCCC	5	<hkxhk>	hh	0	hk	272	kk	1
>ref-130866	TCCCACCCCACCCTCTCTCCCTCCTAT	6	<nnxnp>	nn	112	np	138		
>ref-130871	CATTTATTCACGTGCTCTCCTATTCCC	22	<lmxll>	ll	93	lm	143		
>ref-130876	CTCCCTTATACCCTCCCTCCCTCCGGT	1	<hkxhk>	hh	15	hk	250	kk	0
>ref-130876	CTCCCTTATACCCTCCCTCCCTCCGGT	3	<hkxhk>	hh	2	hk	267	kk	0
>ref-130876	CTCCCTTATACCCTCCCTCCCTCCGGT	25	<hkxhk>	hh	0	hk	249	kk	14
>ref-130879	TCCGCCTTTACCATTTCTCCTTCCCTC	13	<nnxnp>	nn	262	np	0		
>ref-130879	TCCGCCTTTACCATTTCTCCTTCCCTC	16	<nnxnp>	nn	262	np	0		
>ref-130896	CCCTATCCCACTCTTTCTCCTCTTTTA	3	<lmxll>	ll	98	lm	133		
>ref-130896	CCCTATCCCACTCTTTCTCCTCTTTTA	23	<lmxll>	ll	94	lm	134		
>ref-130902	TTGTCGAGTACTGTCTCTCCATCAGTT	1	<hkxhk>	hh	73	hk	113	kk	52
>ref-130902	TTGTCGAGTACTGTCTCTCCATCAGTT	3	<hkxhk>	hh	73	hk	113	kk	52
>ref-130907	AGGAAATTGACTGCCTCTCCAGCTATC	5	<hkxhk>	hh	61	hk	147	kk	55
>ref-130911	TGCCAAGGCACTACATCTCCATTCACA	6	<nnxnp>	nn	141	np	130		
>ref-130911	TGCCAAGGCACTACATCTCCATTCACA	7	<nnxnp>	nn	141	np	130		
>ref-130912	TCCCTTCCCACTCTCCCTCCCTTCACC	26	<hkxhk>	hh	68	hk	197	kk	0
>ref-130943	AGGTTTTCCACGAAAGCTCCAAAGCAT	16	<nnxnp>	nn	131	np	133		
>ref-130950	CTCCTTCCCACGCTTTCTCCCTCTCAA	21	<lmxll>	ll	142	lm	126		
>ref-130970	TATTCCTCTACTTCTCCTCCCCACCCT	14	<lmxll>	ll	116	lm	129		
>ref-130982	CGCTTGGTAACATCTGCTCCGTTGCTA	14	<nnxnp>	nn	266	np	0		
>ref-130983	ATTCTACGGACACCCACTCCATGTTTC	24	<lmxll>	ll	119	lm	133		
>ref-130991	GCCCCCCTCACCCCGTCTCCCTTAGCC	1	<hkxhk>	hh	0	hk	262	kk	6
>ref-130991	GCCCCCCTCACCCCGTCTCCCTTAGCC	2	<hkxhk>	hh	6	hk	261	kk	0
>ref-131008	CTCCCCATCACTTCCCCTCCCTCGTCA	9	<nnxnp>	nn	111	np	138		
>ref-131015	GGTACCTGCACTTGGCCTCCTTTATCG	12	<nnxnp>	nn	120	np	132		
>ref-131065	CACCAATCCACACTTCCTCCTCTCATC	6	<nnxnp>	nn	104	np	152		
>ref-131066	CCACTGTCCACTGTGTCTCCCCCTCTC	22	<nnxnp>	nn	118	np	144		
>ref-131073	TCCCAGCCTACGCGCTCTCCAAGCTCT	8	<nnxnp>	nn	123	np	146		
>ref-131080	GTTAAATCTACGAGGCCTCCCGCCGAA	5	<nnxnp>	nn	271	np	0		

>ref-131080	GTTAAATCTACGAGGCCTCCCGCCGAA	9	<nnxnp>	nn	271	np	0		
>ref-131089	CAGTAAAACACCAGAACTCCTTTTATT	26	<hkxhk>	hh	1	hk	270	kk	0
>ref-131098	GATGGGGGAACCGACCCTCCTTCAGAC	2	<hkxhk>	hh	55	hk	160	kk	50
>ref-131138	CCTTATAACACTCTCCCTCCCTTTAAA	9	<lmxll>	ll	135	lm	110		
>ref-131180	CCGCTCGCGACTCCTCCTCCGCCATCG	9	<nnxnp>	nn	273	np	0		
>ref-131205	CTACCACTGACTTTCCCTCCTTCCGTT	6	<lmxll>	ll	104	lm	125		
>ref-131232	AAATGAATCACAGTGACTCCATCCGAG	1	<nnxnp>	nn	2	np	245		
>ref-131232	AAATGAATCACAGTGACTCCATCCGAG	8	<lmxll>	ll	122	lm	126		
>ref-131232	AAATGAATCACAGTGACTCCATCCGAG	24	<nnxnp>	nn	2	np	245		
>ref-131238	ATTATGATCACCATTGCTCCTGTAATA	27	<nnxnp>	nn	127	np	128		
>ref-131252	GCCCCCAGGACAATAACTCCATCATTC	8	<nnxnp>	nn	128	np	114		
>ref-131258	GTGGGATAAACGGCTACTCCCATAATC	3	<lmxll>	ll	125	lm	125		
>ref-131267	TAACACATCACACACCCTCCTTCCCTC	6	<lmxll>	ll	131	lm	131		
>ref-131276	TTTTTATGCACTCTCTCTCCTTCTCTC	6	<nnxnp>	nn	251	np	1		
>ref-131276	TTTTTATGCACTCTCTCTCCTTCTCTC	23	<lmxll>	ll	130	lm	111		
>ref-131318	CCCACTATCACTCTGTCTCCCTGAATC	2	<nnxnp>	nn	126	np	131		
>ref-131332	ACTGTTTCTACAACTACTCCTTCCACT	16	<nnxnp>	nn	106	np	123		
>ref-131357	ACCTAAGCTACCATACCTCCAACTTAC	7	<lmxll>	ll	140	lm	118		
>ref-131370	CTATTCTGGACTCTCTCTCCCACCCTT	7	<nnxnp>	nn	99	np	157		
>ref-131372	TTATTTCCAACTGAAACTCCTGTTTGG	9	<lmxll>	ll	13	lm	221		
>ref-131408	TTGCCTGATACTTTTCCTCCTTTCACT	3	<lmxll>	ll	77	lm	180		
>ref-131408	TTGCCTGATACTTTTCCTCCTTTCACT	12	<nnxnp>	nn	175	np	80		
>ref-131410	ATACACGACACATAAGCTCCACGTGCC	26	<hkxhk>	hh	72	hk	148	kk	45
>ref-131410	ATACACGACACATAAGCTCCACGTGCC	27	<nnxnp>	nn	138	np	130		
>ref-131423	TGTAAATTTACATATACTCCAAAGCAC	3	<hkxhk>	hh	0	hk	273	kk	0
>ref-131423	TGTAAATTTACATATACTCCAAAGCAC	8	<nnxnp>	nn	242	np	23		
>ref-131431	CTCTCTCCAACACTCTCTCCAACACTT	13	<hkxhk>	hh	2	hk	217	kk	41
>ref-131431	CTCTCTCCAACACTCTCTCCAACACTT	26	<nnxnp>	nn	104	np	151		
>ref-131431	CTCTCTCCAACACTCTCTCCAACACTT	27	<nnxnp>	nn	99	np	152		
>ref-131434	CAAGAGCTCACGTAGCCTCCGTCCAGA	4	<nnxnp>	nn	122	np	144		
>ref-131465	CGAATACAAACAACGCCTCCTGTTTAT	12	<lmxll>	ll	124	lm	132		
>ref-131481	TAAAGTTTTACTATCTCTCCTTTGTGT	2	<nnxnp>	nn	128	np	111		
>ref-131483	CTGGCACACACACACACTCCACTCACT	4	<nnxnp>	nn	269	np	1		
>ref-131497	ACGCCTCCTACCTTCCCTCCCGTCCCT	1	<nnxnp>	nn	10	np	257		
>ref-131512	CCCTTACCCACGTCCCCTCCTGCCCCT	4	<lmxll>	ll	11	lm	248		
>ref-131512	CCCTTACCCACGTCCCCTCCTGCCCCT	12	<lmxll>	ll	11	lm	247		
>ref-131516	TTCCTTCTCACTTACCCTCCCCCCTCT	6	<nnxnp>	nn	129	np	125		
>ref-131516	TTCCTTCTCACTTACCCTCCCCCCTCT	12	<nnxnp>	nn	130	np	125		

>ref-131521	CAGTCACTCACCTTATCTCCTCCTTTC	27	<lmxll>	ll	143	lm	125		
>ref-131528	ATGTTTCCCACCCTCTCTCCTTCACTC	15	<lmxll>	ll	133	lm	125		
>ref-131530	CCTGGCTGCACCCCCTCTCCTACCCCT	4	<nnxnp>	nn	151	np	109		
>ref-131545	CCTTCCCTAACCACTCCTCCACCCCTT	14	<lmxll>	ll	138	lm	116		
>ref-131562	CCTACGCGAACACGTCCTCCAGCTGCT	27	<lmxll>	ll	6	lm	264		
>ref-131592	GCAGCTACTACTGGTGCTCCTGCCACT	3	<nnxnp>	nn	122	np	143		
>ref-131625	CACACCCACACCCCATCTCCACGACCA	27	<nnxnp>	nn	106	np	154		
>ref-131637	CTCGCTCCCACGCTCCCTCCTCCACGT	24	<nnxnp>	nn	144	np	120		
>ref-131647	ATGCAAAATACCTGCACTCCGACTGCT	1	<nnxnp>	nn	12	np	256		
>ref-131647	ATGCAAAATACCTGCACTCCGACTGCT	16	<nnxnp>	nn	9	np	262		
>ref-131665	AGTTGGACTACCTTCTCTCCAGCTTGC	12	<hkxhk>	hh	0	hk	252	kk	7
>ref-131665	AGTTGGACTACCTTCTCTCCAGCTTGC	22	<hkxhk>	hh	0	hk	252	kk	7
>ref-131672	TTACCTAAAACTATCTCTCCATCCATA	2	<nnxnp>	nn	120	np	138		
>ref-131684	CACGTACACACACTTCCTCCCTCTGCA	25	<hkxhk>	hh	38	hk	163	kk	62
>ref-131684	CACGTACACACACTTCCTCCCTCTGCA	26	<lmxll>	ll	92	lm	175		
>ref-131690	ATACCCTTCACTCTGGCTCCCTTCCAT	12	<nnxnp>	nn	134	np	129		
>ref-131690	ATACCCTTCACTCTGGCTCCCTTCCAT	26	<nnxnp>	nn	134	np	129		
>ref-131691	GGTTGTGGTACCGGCGCTCCACGTGAC	14	<lmxll>	ll	127	lm	137		
>ref-131706	TCTTGATAAACTAGTTCTCCCATGTTC	14	<nnxnp>	nn	114	np	144		
>ref-131706	TCTTGATAAACTAGTTCTCCCATGTTC	26	<nnxnp>	nn	116	np	142		
>ref-131720	CTCCTCACAACCCACCCTCCTCACAAC	13	<hkxhk>	hh	2	hk	243	kk	1
>ref-131720	CTCCTCACAACCCACCCTCCTCACAAC	22	<hkxhk>	hh	2	hk	244	kk	1
>ref-131724	AAGGAACGCACGCAACCTCCTTTTTAT	25	<nnxnp>	nn	146	np	118		
>ref-131730	CAGCCACTCACCAACCCTCCACCTCCC	24	<nnxnp>	nn	206	np	48		
>ref-131730	CAGCCACTCACCAACCCTCCACCTCCC	26	<hkxhk>	hh	197	hk	67	kk	0
>ref-131730	CAGCCACTCACCAACCCTCCACCTCCC	27	<nnxnp>	nn	268	np	3		
>ref-131744	CTCGGTCCCACTCCTACTCCTTCCCTC	23	<lmxll>	ll	126	lm	141		
>ref-131756	ATCATCGTCACTGCCACTCCCTTCGTT	12	<lmxll>	ll	133	lm	133		
>ref-131766	TACCTTTCCACCCTCCCTCCCTTTCCC	5	<lmxll>	ll	49	lm	189		
>ref-131766	TACCTTTCCACCCTCCCTCCCTTTCCC	7	<lmxll>	ll	54	lm	182		
>ref-131766	TACCTTTCCACCCTCCCTCCCTTTCCC	26	<nnxnp>	nn	267	np	0		
>ref-131773	ACCACCAACACCATCACTCCTTCTCCG	1	<hkxhk>	hh	60	hk	128	kk	42
>ref-131778	CACACACACACCTTTTCTCCCACCTTC	15	<nnxnp>	nn	267	np	1		
>ref-131778	CACACACACACCTTTTCTCCCACCTTC	23	<hkxhk>	hh	53	hk	145	kk	56
>ref-131778	CACACACACACCTTTTCTCCCACCTTC	25	<nnxnp>	nn	271	np	0		
>ref-131803	GAGATTCGAACGCGCTCTCCGCCACCA	2	<lmxll>	ll	118	lm	150		
>ref-131806	GTTCTGAAGACCTGCCCTCCCTCGGTG	4	<lmxll>	ll	140	lm	81		
>ref-131809	GAGAGGGAAACCCCTGCTCCTGTGTCA	15	<hkxhk>	hh	51	hk	125	kk	87

>ref-131816	CACTCATTCACTTACTCTCCTATTCTC	3	<lmxll>	ll	4	lm	268		
>ref-131816	CACTCATTCACTTACTCTCCTATTCTC	7	<lmxll>	ll	4	lm	268		
>ref-131817	TCCTTATCAACAATCCCTCCCTGCTCT	25	<lmxll>	ll	260	lm	0		
>ref-131822	ACGTGAGGTACGGACACTCCTGTGCAA	23	<lmxll>	ll	127	lm	138		
>ref-131824	CAGGTAGTAACAGCGTCTCCGTGATAT	4	<nnxnp>	nn	113	np	130		
>ref-131825	CACACACACACATTTGCTCCCTCTACG	2	<nnxnp>	nn	1	np	255		
>ref-131826	GATCCGCCTACATCATCTCCTTCCCCC	7	<lmxll>	ll	269	lm	0		
>ref-131827	ATTTAACCTACTAATGCTCCCAGGGCC	3	<nnxnp>	nn	27	np	234		
>ref-131827	ATTTAACCTACTAATGCTCCCAGGGCC	8	<lmxll>	ll	160	lm	99		
>ref-131827	ATTTAACCTACTAATGCTCCCAGGGCC	27	<hkxhk>	hh	0	hk	14	kk	254
>ref-131837	TTATCACACACGTTCACTCCCACACCA	7	<nnxnp>	nn	143	np	126		
>ref-131843	CTACATCTCACGCTTTCTCCTTTACTT	15	<lmxll>	ll	111	lm	138		
>ref-131855	TTCCCCAGGACCAGCTCTCCCTGCACT	9	<hkxhk>	hh	2	hk	266	kk	2
>ref-131855	TTCCCCAGGACCAGCTCTCCCTGCACT	22	<hkxhk>	hh	2	hk	267	kk	2
>ref-131882	ATGTGAGTCACACCATCTCCAGAATCA	8	<hkxhk>	hh	0	hk	273	kk	0
>ref-131882	ATGTGAGTCACACCATCTCCAGAATCA	21	<hkxhk>	hh	9	hk	259	kk	0
>ref-131882	ATGTGAGTCACACCATCTCCAGAATCA	24	<hkxhk>	hh	0	hk	273	kk	0
>ref-131896	TTCGAATCTACAGATTCTCCTACCAGA	12	<hkxhk>	hh	48	hk	128	kk	52
>ref-131916	AGGAGACCTACCTTGTCTCCTAGGCTT	5	<hkxhk>	hh	64	hk	145	kk	53
>ref-131919	TTATCCCCAACCCATTCTCCTGTTCCC	25	<nnxnp>	nn	118	np	147		
>ref-131934	TCTTCCTGCACCCATCCTCCTGTGGGA	2	<nnxnp>	nn	139	np	118		
>ref-131940	CCCCTTCTTACCTCTTCTCCTTCGTTC	27	<lmxll>	ll	133	lm	119		
>ref-131943	CCCCTAGCAACCTCCCCTCCCTCAACA	5	<lmxll>	ll	125	lm	140		
>ref-131943	CCCCTAGCAACCTCCCCTCCCTCAACA	6	<lmxll>	ll	125	lm	140		
>ref-131952	CTCTTCTTTACCCCTTCTCCATCCTCT	3	<nnxnp>	nn	262	np	0		
>ref-131957	AAGCGTTTCACATCGGCTCCCGCACAC	21	<nnxnp>	nn	141	np	131		
>ref-131974	CCTTCCAAAACCACCCCTCCTTCCTAT	25	<nnxnp>	nn	98	np	127		
>ref-131983	CTAGGTTAAACAAGGTCTCCCTACTTC	13	<lmxll>	ll	130	lm	122		
>ref-131991	TGCCTGGACACAGGCTCTCCATCTCAA	5	<nnxnp>	nn	139	np	128		
>ref-132017	CACCACACAACCCCTTCTCCCCTCCCA	5	<nnxnp>	nn	126	np	130		
>ref-132017	CACCACACAACCCCTTCTCCCCTCCCA	15	<nnxnp>	nn	126	np	130		
>ref-132025	CACCTACAGACACACACTCCATCACGC	9	<nnxnp>	nn	143	np	121		
>ref-132025	CACCTACAGACACACACTCCATCACGC	15	<nnxnp>	nn	143	np	121		
>ref-132029	GACAATCCCACTCTCCCTCCTTTCTTC	21	<hkxhk>	hh	0	hk	273	kk	0
>ref-132029	GACAATCCCACTCTCCCTCCTTTCTTC	23	<hkxhk>	hh	0	hk	273	kk	0
>ref-132033	GCATCTTCTACTCATTCTCCTCTTCTT	14	<nnxnp>	nn	129	np	132		
>ref-132033	GCATCTTCTACTCATTCTCCTCTTCTT	23	<hkxhk>	hh	65	hk	137	kk	55
>ref-132037	CCCTTTACGACGCGTCCTCCTCCTTCT	25	<hkxhk>	hh	58	hk	145	kk	60

>ref-132050	GTCTCCCTCACCCTCCCTCCGCTTCCG	4	<nnxnp>	nn	130	np	128		
>ref-132057	TTAACAATAACGCCTTCTCCCAGATCT	3	<hkxhk>	hh	2	hk	271	kk	0
>ref-132057	TTAACAATAACGCCTTCTCCCAGATCT	13	<hkxhk>	hh	10	hk	258	kk	0
>ref-132057	TTAACAATAACGCCTTCTCCCAGATCT	21	<nnxnp>	nn	223	np	35		
>ref-132057	TTAACAATAACGCCTTCTCCCAGATCT	22	<hkxhk>	hh	10	hk	258	kk	0
>ref-132057	TTAACAATAACGCCTTCTCCCAGATCT	23	<nnxnp>	nn	183	np	65		
>ref-132057	TTAACAATAACGCCTTCTCCCAGATCT	24	<hkxhk>	hh	2	hk	271	kk	0
>ref-132071	CCTTCGGCCACCCCTACTCCCCTCCCC	1	<nnxnp>	nn	268	np	2		
>ref-132071	CCTTCGGCCACCCCTACTCCCCTCCCC	6	<nnxnp>	nn	103	np	160		
>ref-132071	CCTTCGGCCACCCCTACTCCCCTCCCC	21	<nnxnp>	nn	164	np	95		
>ref-132073	CTCCTCTTTACTCTTCCTCCCTTTACT	6	<lmxll>	ll	131	lm	123		
>ref-132086	AACTGGAGCACATATCCTCCGCGTGTA	3	<lmxll>	ll	127	lm	137		
>ref-132086	AACTGGAGCACATATCCTCCGCGTGTA	6	<nnxnp>	nn	141	np	125		
>ref-132091	CGCCCTCTCACACGCCCTCCTAGACAT	21	<hkxhk>	hh	62	hk	152	kk	51
>ref-132096	GACACAATAACAAACACTCCAAAGCCA	5	<hkxhk>	hh	68	hk	135	kk	41
>ref-132106	CTCTGCCCTACCTTACCTCCCCTTCCT	5	<lmxll>	ll	139	lm	127		
>ref-132114	TCCCCCCAAACCTCAACTCCAAGGAAG	16	<nnxnp>	nn	241	np	0		
>ref-132119	GATTCGGACACATCACCTCCAAGGAAA	6	<nnxnp>	nn	146	np	111		
>ref-132121	TTGCTCTCCACTCTCACTCCCTCGCGA	9	<nnxnp>	nn	121	np	140		
>ref-132158	TAACACTACACCATGGCTCCACCAAGT	13	<lmxll>	ll	120	lm	110		
>ref-132158	TAACACTACACCATGGCTCCACCAAGT	16	<lmxll>	ll	105	lm	127		
>ref-132161	ACACCTTCCACCTCCCCTCCACCTCCC	15	<lmxll>	ll	119	lm	134		
>ref-132161	ACACCTTCCACCTCCCCTCCACCTCCC	16	<lmxll>	ll	92	lm	131		
>ref-132172	CAGACAAACACGAACGCTCCTTCCTTC	15	<nnxnp>	nn	125	np	147		
>ref-132174	CTTCGCGCGACGGCGACTCCTACTACA	5	<nnxnp>	nn	115	np	144		
>ref-132216	ACGCCATGAACACTACCTCCGTGGACA	1	<hkxhk>	hh	1	hk	272	kk	0
>ref-132216	ACGCCATGAACACTACCTCCGTGGACA	8	<nnxnp>	nn	236	np	33		
>ref-132216	ACGCCATGAACACTACCTCCGTGGACA	15	<hkxhk>	hh	34	hk	239	kk	0
>ref-132224	AAGGGTCGGACTCGTTCTCCCTTTCGC	5	<nnxnp>	nn	157	np	84		
>ref-132224	AAGGGTCGGACTCGTTCTCCCTTTCGC	8	<lmxll>	ll	149	lm	121		
>ref-132224	AAGGGTCGGACTCGTTCTCCCTTTCGC	21	<nnxnp>	nn	157	np	85		
>ref-132224	AAGGGTCGGACTCGTTCTCCCTTTCGC	22	<lmxll>	ll	1	lm	270		
>ref-132242	CCCTCTTCAACACGCCCTCCCTCTCAC	26	<nnxnp>	nn	120	np	149		
>ref-132253	CAATCATAAACAAAGACTCCAAGCATA	4	<hkxhk>	hh	0	hk	272	kk	0
>ref-132263	CCCTTCCAAACGACCCCTCCCCCCTTC	4	<lmxll>	ll	185	lm	75		
>ref-132263	CCCTTCCAAACGACCCCTCCCCCCTTC	6	<lmxll>	ll	187	lm	74		
>ref-132263	CCCTTCCAAACGACCCCTCCCCCCTTC	14	<nnxnp>	nn	149	np	105		
>ref-132263	CCCTTCCAAACGACCCCTCCCCCCTTC	22	<lmxll>	ll	243	lm	18		

>ref-132264	TCACTTTCTACTCTTCCTCCCCTCCTT	16	<hkxhk>	hh	0	hk	169	kk	82
>ref-132276	TTCCTTTCAACGGCGCCTCCGTTTACT	3	<nnxnp>	nn	143	np	127		
>ref-132277	CCCACCCTCACCCCTCCTCCTCACCCT	4	<hkxhk>	hh	230	hk	22	kk	0
>ref-132277	CCCACCCTCACCCCTCCTCCTCACCCT	15	<hkxhk>	hh	0	hk	25	kk	229
>ref-132277	CCCACCCTCACCCCTCCTCCTCACCCT	16	<hkxhk>	hh	3	hk	266	kk	1
>ref-132277	CCCACCCTCACCCCTCCTCCTCACCCT	23	<hkxhk>	hh	3	hk	265	kk	2
>ref-132286	AAAGGCTATACACATCCTCCTCCTTGT	23	<lmxll>	ll	120	lm	119		
>ref-132295	ACACACAGAACGGCAGCTCCTGCATCA	16	<lmxll>	ll	143	lm	125		
>ref-132309	AGGAATTTGACCTTCCCTCCACGCCTC	2	<hkxhk>	hh	0	hk	125	kk	128
>ref-132309	AGGAATTTGACCTTCCCTCCACGCCTC	4	<hkxhk>	hh	1	hk	272	kk	0
>ref-132309	AGGAATTTGACCTTCCCTCCACGCCTC	16	<lmxll>	ll	259	lm	9		
>ref-132345	CCCTTTCCTACTGCCTCTCCATTTCTC	9	<nnxnp>	nn	17	np	217		
>ref-132345	CCCTTTCCTACTGCCTCTCCATTTCTC	13	<nnxnp>	nn	16	np	216		
>ref-132348	CTTCCCTTCACACTTACTCCTCCACTC	27	<nnxnp>	nn	257	np	0		
>ref-132367	GAGAAAGAAACCTCTCCTCCTCCTCGG	2	<hkxhk>	hh	55	hk	155	kk	52
>ref-132418	CTCATACCAACAGCCCCTCCCCCTCCC	2	<lmxll>	ll	138	lm	132		
>ref-132418	CTCATACCAACAGCCCCTCCCCCTCCC	13	<lmxll>	ll	126	lm	142		
>ref-132432	TATTTCTCCACCCTCTCTCCCCCAATT	21	<nnxnp>	nn	127	np	116		
>ref-132432	TATTTCTCCACCCTCTCTCCCCCAATT	25	<nnxnp>	nn	126	np	116		
>ref-132446	CCACCACCCACACCTCCTCCCCCTCCA	3	<hkxhk>	hh	12	hk	253	kk	0
>ref-132446	CCACCACCCACACCTCCTCCCCCTCCA	8	<nnxnp>	nn	223	np	33		
>ref-132446	CCACCACCCACACCTCCTCCCCCTCCA	13	<lmxll>	ll	256	lm	9		
>ref-132450	CACACAAACACATCCCCTCCCCCCATG	1	<hkxhk>	hh	60	hk	149	kk	52
>ref-132455	TCTTAGTCTACCCTCTCTCCCCTCCCT	13	<lmxll>	ll	135	lm	114		
>ref-132463	TGTGCCCTTACTGAGCCTCCCAGAGGT	23	<hkxhk>	hh	60	hk	140	kk	63
>ref-132464	ATATACCGTACAGGATCTCCCCGCCCC	5	<nnxnp>	nn	269	np	0		
>ref-132488	CTATCCCTGACCTTTCCTCCTCCTCCT	27	<lmxll>	ll	123	lm	134		
>ref-132508	TACATTCACACTAGCGCTCCCCGCACA	23	<hkxhk>	hh	2	hk	271	kk	0
>ref-132509	GAATAATGTACTCTCCCTCCCTCTCTC	22	<nnxnp>	nn	139	np	126		
>ref-132541	AGGTTATCTACAATTGCTCCAATTTAC	3	<hkxhk>	hh	44	hk	115	kk	62
>ref-132541	AGGTTATCTACAATTGCTCCAATTTAC	8	<hkxhk>	hh	62	hk	115	kk	44
>ref-132555	CACTTCCGGACCCGATCTCCCTCGACA	25	<nnxnp>	nn	130	np	130		
>ref-132565	CTCTTTGTCACTATTTCTCCGTCTGTC	2	<hkxhk>	hh	0	hk	273	kk	0
>ref-132565	CTCTTTGTCACTATTTCTCCGTCTGTC	23	<hkxhk>	hh	0	hk	272	kk	0
>ref-132603	CTCGGATTCACGCGTCCTCCACCAGCC	15	<hkxhk>	hh	76	hk	138	kk	45
>ref-132649	ACACTTCTCACTGAGCCTCCATCCCAG	2	<nnxnp>	nn	140	np	131		
>ref-132675	GCCCATCCCACCCTGACTCCAACTCTA	21	<nnxnp>	nn	121	np	148		
>ref-132682	GCGGTAGTTACGGCGGCTCCAACGGGG	26	<nnxnp>	nn	144	np	123		

>ref-132693	GTGATCCACACCGCCTCTCCACCCTCT	24	<nnxnp>	nn	114	np	153		
>ref-132717	CAATCGCCAACGCCATCTCCCGGTATC	3	<hkxhk>	hh	65	hk	130	kk	65
>ref-132726	CAGTCTCTCACGGACCCTCCAGCCCTC	23	<nnxnp>	nn	126	np	141		
>ref-132732	CACCCCCCGACGCGACCTCCCCGCCAG	3	<nnxnp>	nn	125	np	124		
>ref-132738	CCTTTCTCTACCCTACCTCCCCCCCTA	24	<lmxll>	ll	134	lm	97		
>ref-132777	CTTGCAGAAACTCTCTCTCCCTGTTAC	21	<lmxll>	ll	157	lm	109		
>ref-132787	CCCTCTCCGACCCGCTCTCCCCTTCTC	14	<nnxnp>	nn	245	np	4		
>ref-132787	CCCTCTCCGACCCGCTCTCCCCTTCTC	26	<nnxnp>	nn	258	np	2		
>ref-132788	ATTCTCCCTACCATCTCTCCCTTCCTA	4	<lmxll>	ll	138	lm	119		
>ref-132822	GCGTGATATACAGAGCCTCCGGGCTAG	7	<lmxll>	ll	114	lm	151		
>ref-132823	TGATCGCGAACATTTTCTCCATTCTTG	12	<lmxll>	ll	125	lm	141		
>ref-132823	TGATCGCGAACATTTTCTCCATTCTTG	13	<lmxll>	ll	125	lm	141		
>ref-132829	AACGAGGTGACAAGAGCTCCTTAATAA	22	<hkxhk>	hh	0	hk	161	kk	86
>ref-132829	AACGAGGTGACAAGAGCTCCTTAATAA	24	<hkxhk>	hh	87	hk	161	kk	0
>ref-132844	AATGTCACTACAGCTGCTCCAGTCAAT	6	<nnxnp>	nn	2	np	270		
>ref-132857	GATCTCCGGACGCGCTCTCCTCTGAGA	27	<hkxhk>	hh	1	hk	272	kk	0
>ref-132866	CCCCCTCTTACGCCCCCTCCTCCCGCT	16	<nnxnp>	nn	109	np	138		
>ref-132904	TAATCGTTTACACTTTCTCCCTTTGCA	14	<lmxll>	ll	128	lm	135		
>ref-132920	CACCCTCTAACCCTTTCTCCTCTCTTT	26	<nnxnp>	nn	137	np	127		
>ref-132925	AACAGCTACACAAACTCTCCTCCTCCG	1	<lmxll>	ll	123	lm	130		
>ref-132931	ATGTAACTTACTGATCCTCCCTAATTA	26	<nnxnp>	nn	226	np	0		
>ref-132941	AAATTAACCACGCCCTCTCCCTAAACA	23	<hkxhk>	hh	71	hk	165	kk	0
>ref-132941	AAATTAACCACGCCCTCTCCCTAAACA	26	<hkxhk>	hh	12	hk	223	kk	1
>ref-132948	AGGAATGGGACAATATCTCCACACACA	2	<lmxll>	ll	127	lm	119		
>ref-132949	TACTATCAGACGGAGCCTCCCTCAGTA	15	<nnxnp>	nn	142	np	130		
>ref-132959	CTCCACCACACTCTGTCTCCTTTTCCT	9	<lmxll>	ll	103	lm	143		
>ref-132960	TCGTTCTCTACCCTTTCTCCTTTTCCA	16	<hkxhk>	hh	52	hk	145	kk	60
>ref-132977	GAGAGAAACACCATTTCTCCATTCATC	12	<nnxnp>	nn	122	np	123		
>ref-132985	TTCCCTACGACGCCCCCTCCCCCCTTC	21	<lmxll>	ll	123	lm	142		
>ref-133004	ATCCTCTCTACCTTCCCTCCCTCTACC	2	<hkxhk>	hh	0	hk	254	kk	4
>ref-133004	ATCCTCTCTACCTTCCCTCCCTCTACC	14	<hkxhk>	hh	0	hk	254	kk	4
>ref-133004	ATCCTCTCTACCTTCCCTCCCTCTACC	15	<lmxll>	ll	151	lm	100		
>ref-133011	CACCCACCCACACCCACTCCCATGCAC	4	<hkxhk>	hh	0	hk	265	kk	0
>ref-133011	CACCCACCCACACCCACTCCCATGCAC	21	<lmxll>	ll	175	lm	84		
>ref-133033	GGCGCCACAACTCTCCCTCCCTCCCTC	5	<nnxnp>	nn	118	np	122		
>ref-133033	GGCGCCACAACTCTCCCTCCCTCCCTC	6	<nnxnp>	nn	68	np	176		
>ref-133033	GGCGCCACAACTCTCCCTCCCTCCCTC	9	<nnxnp>	nn	229	np	27		
>ref-133033	GGCGCCACAACTCTCCCTCCCTCCCTC	24	<lmxll>	ll	135	lm	136		

>ref-133034	GGTCAGTTCACCGACGCTCCTCTTCTG	13	<lmxll>	ll	113	lm	142		
>ref-133040	AATCCATCTACCTCTCCTCCTCTTCTC	1	<hkxhk>	hh	1	hk	272	kk	0
>ref-133040	AATCCATCTACCTCTCCTCCTCTTCTC	5	<hkxhk>	hh	47	hk	212	kk	0
>ref-133040	AATCCATCTACCTCTCCTCCTCTTCTC	21	<hkxhk>	hh	0	hk	272	kk	1
>ref-133042	CTTCCTGAGACCGTCTCTCCCTCTCTT	12	<nnxnp>	nn	132	np	131		
>ref-133062	TCTCGGCCCACTCCCTCTCCCCGCCCA	1	<hkxhk>	hh	0	hk	140	kk	92
>ref-133062	TCTCGGCCCACTCCCTCTCCCCGCCCA	5	<hkxhk>	hh	0	hk	147	kk	107
>ref-133062	TCTCGGCCCACTCCCTCTCCCCGCCCA	16	<lmxll>	ll	232	lm	24		
>ref-133069	AGTATTGGAACCGCCTCTCCCTTTCAT	25	<hkxhk>	hh	62	hk	134	kk	63
>ref-133081	CGATGAGAAACTCGACCTCCGTTCAGT	21	<nnxnp>	nn	130	np	133		
>ref-133088	TTTACCTTTACCCCGCCTCCCCTTTCC	6	<nnxnp>	nn	117	np	112		
>ref-133089	ATCGAGAGAACTTTCCCTCCCCAGTGC	3	<lmxll>	ll	98	lm	149		
>ref-133089	ATCGAGAGAACTTTCCCTCCCCAGTGC	21	<hkxhk>	hh	103	hk	139	kk	0
>ref-133093	TTTCTCTCTACTCTCCCTCCAGCTATA	5	<lmxll>	ll	105	lm	149		
>ref-133128	TCTCTGTTCACCTCCTCTCCCTTTTCT	1	<hkxhk>	hh	106	hk	128	kk	3
>ref-133130	ACCTCCCCTACCTCAGCTCCTGTCTGC	4	<hkxhk>	hh	0	hk	198	kk	68
>ref-133130	ACCTCCCCTACCTCAGCTCCTGTCTGC	26	<hkxhk>	hh	0	hk	199	kk	68
>ref-133177	TTGTTATTTACTAATGCTCCTCTCAAT	23	<hkxhk>	hh	1	hk	114	kk	116
>ref-133180	CGACCGTAAACCGACTCTCCTCTCACC	15	<nnxnp>	nn	130	np	121		
>ref-133183	ACACGGCGTACTATGCCTCCAGTCATG	6	<hkxhk>	hh	51	hk	155	kk	59
>ref-133233	GATAATATAACTCCATCTCCCTTTCTC	2	<hkxhk>	hh	68	hk	140	kk	53
>ref-133237	TCTTCATGCACAATCTCTCCTCGGGAT	12	<lmxll>	ll	132	lm	135		
>ref-133238	CAAGGTAATACACCCCCTCCCTCTCCC	27	<lmxll>	ll	134	lm	131		
>ref-133243	CATATCATCACCATCACTCCCACCCCG	22	<hkxhk>	hh	37	hk	150	kk	69
>ref-133254	GCCACATATACATGGGCTCCCATGCAC	7	<hkxhk>	hh	56	hk	156	kk	49
>ref-133264	TGTTCTGATACCTGCTCTCCACACTCA	25	<lmxll>	ll	126	lm	143		
>ref-133285	GTCTGGCCAACGTCGGCTCCTCCACCA	13	<nnxnp>	nn	219	np	49		
>ref-133310	CACGCCGCTACTTCTGCTCCATCTCAT	7	<nnxnp>	nn	124	np	139		
>ref-133311	CCTCCTTCTACTGCTCCTCCTTGTAAA	23	<hkxhk>	hh	57	hk	147	kk	50
>ref-133341	CATCGACCAACAGGAGCTCCACACAGA	26	<lmxll>	ll	157	lm	95		
>ref-133350	ACTCCCCCTACCATCACTCCCCAGTCC	16	<lmxll>	ll	118	lm	148		
>ref-133379	CCCATTCATACTCTATCTCCTTCACTT	21	<nnxnp>	nn	134	np	130		
>ref-133406	CTACAGTAGACATGATCTCCTTTCGCC	5	<hkxhk>	hh	1	hk	272	kk	0
>ref-133406	CTACAGTAGACATGATCTCCTTTCGCC	6	<nnxnp>	nn	229	np	38		
>ref-133406	CTACAGTAGACATGATCTCCTTTCGCC	9	<hkxhk>	hh	15	hk	252	kk	0
>ref-133406	CTACAGTAGACATGATCTCCTTTCGCC	26	<hkxhk>	hh	0	hk	273	kk	0
>ref-133434	TACCTTGTAACTCCTTCTCCTCCCCTC	9	<nnxnp>	nn	131	np	124		
>ref-133435	TCTCTCTCAACTCTTCCTCCCCTCCAT	5	<lmxll>	ll	21	lm	236		

>ref-133435	TCTCTCTCAACTCTTCCTCCCCTCCAT	12	<lmxll>	ll	21	lm	236		
>ref-133446	GAGAAACCAACTGCGCCTCCTCAAAAT	24	<lmxll>	ll	114	lm	139		
>ref-133460	CTTCCAATTACTGTTACTCCCTTGCTG	22	<nnxnp>	nn	264	np	0		
>ref-133468	TGTGCAGGAACCAGCCCTCCTTCTGTG	14	<lmxll>	ll	131	lm	123		
>ref-133487	TGGAGGGCTACGTACACTCCACACGTT	6	<lmxll>	ll	118	lm	147		
>ref-133500	TTTTCTCTCACCCTTCCTCCTCATTCC	1	<nnxnp>	nn	126	np	124		
>ref-133500	TTTTCTCTCACCCTTCCTCCTCATTCC	4	<nnxnp>	nn	128	np	124		
>ref-133561	CAAATACCCACTCGCTCTCCCTCCCGC	6	<hkxhk>	hh	101	hk	154	kk	0
>ref-133561	CAAATACCCACTCGCTCTCCCTCCCGC	26	<hkxhk>	hh	0	hk	153	kk	98
>ref-133564	TGATCATTCACAATCACTCCACCTCAT	22	<nnxnp>	nn	133	np	116		
>ref-133611	CAGCCACCCACCCTACCTCCTTCTCCT	5	<lmxll>	ll	268	lm	0		
>ref-133618	TTTGCTTTAACACCCTCTCCCTCCTTT	4	<hkxhk>	hh	71	hk	110	kk	45
>ref-133626	TCTATTCTTACCCTCGCTCCTTACCCT	14	<nnxnp>	nn	132	np	102		
>ref-133660	AGCAACAGCACATATACTCCCACAGGA	22	<lmxll>	ll	124	lm	137		
>ref-133676	GCCAGACGAACCCGAACTCCGGAGCTC	26	<nnxnp>	nn	131	np	112		
>ref-133679	CCCTTACGCACCCTTCCTCCACTACAT	27	<nnxnp>	nn	133	np	139		
>ref-133696	TCATCACCCACCACCCCTCCCCCTTCT	5	<nnxnp>	nn	116	np	130		
>ref-133707	CCATCTTACACTTCCTCTCCTCTCTCT	21	<nnxnp>	nn	132	np	129		
>ref-133716	ACTCATGACACTCCCCCTCCTCCCTTC	9	<lmxll>	ll	262	lm	0		
>ref-133728	GGTGACATTACCAAAGCTCCAGCACTG	4	<hkxhk>	hh	0	hk	266	kk	2
>ref-133728	GGTGACATTACCAAAGCTCCAGCACTG	12	<lmxll>	ll	19	lm	245		
>ref-133728	GGTGACATTACCAAAGCTCCAGCACTG	27	<hkxhk>	hh	0	hk	267	kk	2
>ref-133742	ATCGATCCCACCCCCTCTCCTTTTCCA	22	<lmxll>	ll	108	lm	124		
>ref-133742	ATCGATCCCACCCCCTCTCCTTTTCCA	26	<lmxll>	ll	108	lm	124		
>ref-133750	TTTCCCGCCACTCAGCCTCCGCCTCCC	5	<lmxll>	ll	109	lm	153		
>ref-133757	CATTCACACACTCCAGCTCCTCTCTCT	9	<lmxll>	ll	137	lm	129		
>ref-133768	GCCGCTGGAACTCCTCCTCCAGGCGCT	2	<lmxll>	ll	269	lm	2		
>ref-133768	GCCGCTGGAACTCCTCCTCCAGGCGCT	5	<hkxhk>	hh	0	hk	272	kk	0
>ref-133768	GCCGCTGGAACTCCTCCTCCAGGCGCT	23	<nnxnp>	nn	116	np	145		
>ref-133774	CCCCCTCTCACAGCCCCTCCCCATCTC	6	<lmxll>	ll	188	lm	57		
>ref-133774	CCCCCTCTCACAGCCCCTCCCCATCTC	23	<lmxll>	ll	187	lm	58		
>ref-133790	TAGCAGAACACCGCCTCTCCAACCCCC	22	<nnxnp>	nn	15	np	218		
>ref-133790	TAGCAGAACACCGCCTCTCCAACCCCC	23	<hkxhk>	hh	15	hk	224	kk	8
>ref-133790	TAGCAGAACACCGCCTCTCCAACCCCC	26	<nnxnp>	nn	15	np	217		
>ref-133792	ACATTTAGCACCTGTCCTCCTTATCTT	12	<lmxll>	ll	129	lm	128		
>ref-133799	GGATCAAGGACGCTCACTCCAACCCCG	27	<lmxll>	ll	127	lm	125		
>ref-133814	TCCACATTAACGCCGTCTCCTTATTCT	23	<lmxll>	ll	124	lm	109		
>ref-133815	ACAGGTATAACCTTGTCTCCTGCAGAG	15	<lmxll>	ll	119	lm	146		

>ref-133823	CTCCTCTCCACTTCCCCTCCAATCCTT	16	<hkxhk>	hh	1	hk	260	kk	6
>ref-133823	CTCCTCTCCACTTCCCCTCCAATCCTT	24	<hkxhk>	hh	6	hk	260	kk	1
>ref-133843	GTGTATTCTACGAACTCTCCTCCTTTT	5	<hkxhk>	hh	111	hk	126	kk	3
>ref-133874	TACCTCCCTACCTTTCCTCCATCCTTC	15	<nnxnp>	nn	131	np	134		
>ref-133893	ATGTAATTCACTCCTTCTCCCTTGCCT	24	<hkxhk>	hh	49	hk	141	kk	69
>ref-133906	GGAACGAGAACACAGCCTCCTGAATTA	24	<lmxll>	ll	124	lm	139		
>ref-133928	CTCTCTGAAACGGACTCTCCTGACCTA	25	<hkxhk>	hh	0	hk	255	kk	10
>ref-133953	CTAAATCGAACCTTCACTCCCGTAATT	21	<nnxnp>	nn	101	np	132		
>ref-133972	GCCTTTCACACCTAAACTCCACCCTTT	22	<nnxnp>	nn	109	np	139		
>ref-133977	TGCACACACACTAGTTCTCCTTCTGCG	25	<nnxnp>	nn	120	np	146		
>ref-133977	TGCACACACACTAGTTCTCCTTCTGCG	27	<lmxll>	ll	143	lm	128		
>ref-133991	TCTCTACCCACACCGGCTCCCAACTCT	15	<nnxnp>	nn	270	np	0		
>ref-133994	CCTGATGCTACACCACCTCCACTCTCT	8	<nnxnp>	nn	124	np	135		
>ref-134001	CCTCGCTTCACGCCATCTCCATTTGCA	13	<lmxll>	ll	146	lm	122		
>ref-134008	TCCACCCATACCAAGTCTCCATCCACC	9	<lmxll>	ll	117	lm	141		
>ref-134024	GGAGGCTGCACCTCTTCTCCCCCAGAA	3	<lmxll>	ll	136	lm	129		
>ref-134040	TGCGGCCTAACTGGGGCTCCGTTGCGG	25	<nnxnp>	nn	247	np	0		
>ref-134050	TCATATTCAACGAAGGCTCCTATGATG	8	<nnxnp>	nn	129	np	116		
>ref-134069	TCTGCTGACACTGATGCTCCGCGCTGA	26	<nnxnp>	nn	121	np	138		
>ref-134082	TTTGCTACCACACTCCCTCCCCTCCCC	27	<nnxnp>	nn	269	np	0		
>ref-134088	CTTCCTACTACACCTTCTCCAGTGCAT	1	<lmxll>	ll	75	lm	174		
>ref-134088	CTTCCTACTACACCTTCTCCAGTGCAT	23	<hkxhk>	hh	20	hk	185	kk	53
>ref-134095	CTTAAATCCACTTCTTCTCCCATCCTT	5	<lmxll>	ll	128	lm	127		
>ref-134105	AAAAATTCAACCATTCCTCCCTCCCTT	8	<lmxll>	ll	126	lm	102		
>ref-134112	AATTAATCCACGCATGCTCCACTAACG	3	<nnxnp>	nn	127	np	132		
>ref-134125	GGATCAGAAACATGGACTCCAACCAAA	7	<lmxll>	ll	233	lm	23		
>ref-134125	GGATCAGAAACATGGACTCCAACCAAA	12	<hkxhk>	hh	128	hk	120	kk	0
>ref-134125	GGATCAGAAACATGGACTCCAACCAAA	14	<hkxhk>	hh	0	hk	138	kk	100
>ref-134134	GTTCTGTTGACCTTTCCTCCTCCCCAT	22	<lmxll>	ll	140	lm	116		
>ref-134144	TCCGTCTTCACCAATTCTCCTTCTCCT	4	<hkxhk>	hh	0	hk	273	kk	0
>ref-134144	TCCGTCTTCACCAATTCTCCTTCTCCT	14	<hkxhk>	hh	69	hk	200	kk	0
>ref-134144	TCCGTCTTCACCAATTCTCCTTCTCCT	22	<hkxhk>	hh	0	hk	200	kk	68
>ref-134144	TCCGTCTTCACCAATTCTCCTTCTCCT	25	<hkxhk>	hh	0	hk	273	kk	0
>ref-134154	ACCCACGTGACGAGGCCTCCACTTTCT	4	<nnxnp>	nn	252	np	16		
>ref-134159	TATCATTTAACAATTCCTCCTTCAGGC	8	<nnxnp>	nn	121	np	117		
>ref-134161	CTCCTTCATACTTTCCCTCCACCTCCC	5	<lmxll>	ll	124	lm	113		
>ref-134171	TGCAGGATCACACTGGCTCCTGCTCTG	24	<lmxll>	ll	155	lm	114		
>ref-134180	CCTTACGCTACCCTCCCTCCCCCCTCC	25	<nnxnp>	nn	181	np	62		

>ref-134184	CTCACACACACCACTACTCCCTCACAC	27	<lmxll>	ll	113	lm	149		
>ref-134190	ATCTTCCTTACCATCCCTCCTTCCCTT	3	<lmxll>	ll	265	lm	0		
>ref-134192	GCTTCCCACACTTCGTCTCCCTCACAC	7	<lmxll>	ll	119	lm	122		
>ref-134205	CATTTCCTTACCAGCCCTCCCTTCCTC	2	<nnxnp>	nn	120	np	126		
>ref-134205	CATTTCCTTACCAGCCCTCCCTTCCTC	5	<nnxnp>	nn	118	np	126		
>ref-134218	ATTCCCACCACCACCTCTCCCCTTCTT	24	<nnxnp>	nn	119	np	140		
>ref-134219	CCACTCCCTACCATCCCTCCATGCACC	3	<lmxll>	ll	149	lm	97		
>ref-134222	ACGTAACTTACAAAGCCTCCTATCACA	9	<nnxnp>	nn	233	np	0		
>ref-134251	CGCTCCTTTACTCACTCTCCTACTCTT	21	<nnxnp>	nn	142	np	112		
>ref-134263	CCCGCCCACACTCCCTCTCCCAGCCTT	25	<hkxhk>	hh	61	hk	131	kk	57
>ref-134273	CGTATATACACTCGACCTCCCGATGAC	21	<lmxll>	ll	238	lm	0		
>ref-134307	ACTTCCTCCACCCTATCTCCTTCCCCT	3	<hkxhk>	hh	44	hk	203	kk	0
>ref-134307	ACTTCCTCCACCCTATCTCCTTCCCCT	24	<hkxhk>	hh	49	hk	198	kk	0
>ref-134307	ACTTCCTCCACCCTATCTCCTTCCCCT	27	<hkxhk>	hh	0	hk	199	kk	48
>ref-134314	CAAAGAATTACCCATGCTCCACCTACA	22	<nnxnp>	nn	136	np	121		
>ref-134324	TATACATCCACTAGCTCTCCCGACGCA	7	<lmxll>	ll	144	lm	116		
>ref-134360	CAACACAGTACTTTTACTCCAACACAA	8	<hkxhk>	hh	0	hk	253	kk	10
>ref-134360	CAACACAGTACTTTTACTCCAACACAA	14	<hkxhk>	hh	0	hk	246	kk	13
>ref-134360	CAACACAGTACTTTTACTCCAACACAA	27	<hkxhk>	hh	12	hk	245	kk	0
>ref-134375	ACCGAGCCAACCCACTCTCCTAGCCCA	1	<lmxll>	ll	142	lm	119		
>ref-134383	ATTTTAAACACCCTTTCTCCCTTTTAA	7	<hkxhk>	hh	25	hk	225	kk	0
>ref-134383	ATTTTAAACACCCTTTCTCCCTTTTAA	9	<hkxhk>	hh	25	hk	224	kk	0
>ref-134383	ATTTTAAACACCCTTTCTCCCTTTTAA	12	<hkxhk>	hh	0	hk	74	kk	165
>ref-134383	ATTTTAAACACCCTTTCTCCCTTTTAA	13	<hkxhk>	hh	165	hk	75	kk	0
>ref-134383	ATTTTAAACACCCTTTCTCCCTTTTAA	16	<hkxhk>	hh	1	hk	267	kk	3
>ref-134383	ATTTTAAACACCCTTTCTCCCTTTTAA	25	<hkxhk>	hh	1	hk	267	kk	3
>ref-134407	GGGACAGGAACAGGGTCTCCTTGAATT	23	<lmxll>	ll	135	lm	119		
>ref-134413	ATCAACACCACTCCCCCTCCTCCTCCT	22	<nnxnp>	nn	144	np	122		
>ref-134413	ATCAACACCACTCCCCCTCCTCCTCCT	25	<nnxnp>	nn	107	np	150		
>ref-134434	GGCTCCTCCACGGCCCCTCCCCTGCTG	21	<lmxll>	ll	125	lm	148		
>ref-134438	TATCACCCTACCTAGCCTCCACTGATT	2	<nnxnp>	nn	29	np	238		
>ref-134438	TATCACCCTACCTAGCCTCCACTGATT	3	<lmxll>	ll	164	lm	90		
>ref-134438	TATCACCCTACCTAGCCTCCACTGATT	4	<nnxnp>	nn	27	np	239		
>ref-134438	TATCACCCTACCTAGCCTCCACTGATT	15	<hkxhk>	hh	0	hk	270	kk	3
>ref-134438	TATCACCCTACCTAGCCTCCACTGATT	22	<hkxhk>	hh	5	hk	266	kk	0
>ref-134447	TTCCCCCCTACATCATCTCCTCTCTCC	2	<lmxll>	ll	119	lm	131		
>ref-134467	CTGTTACTCACCTAATCTCCTACATAT	8	<lmxll>	ll	121	lm	115		
>ref-134495	CTGCTAATAACCTACACTCCTTCGAAC	9	<lmxll>	ll	72	lm	175		

>ref-134495	CTGCTAATAACCTACACTCCTTCGAAC	16	<nnxnp>	nn	178	np	64		
>ref-134495	CTGCTAATAACCTACACTCCTTCGAAC	22	<lmxll>	ll	71	lm	176		
>ref-134495	CTGCTAATAACCTACACTCCTTCGAAC	24	<nnxnp>	nn	178	np	64		
>ref-134511	GCTACTATAACAGCTACTCCTACTACA	2	<lmxll>	ll	120	lm	128		
>ref-134525	CATCTCCTTACACCCACTCCTTACACC	2	<nnxnp>	nn	207	np	42		
>ref-134525	CATCTCCTTACACCCACTCCTTACACC	4	<hkxhk>	hh	22	hk	231	kk	0
>ref-134525	CATCTCCTTACACCCACTCCTTACACC	5	<hkxhk>	hh	0	hk	236	kk	18
>ref-134525	CATCTCCTTACACCCACTCCTTACACC	6	<lmxll>	ll	100	lm	142		
>ref-134540	TCTTGGCAGACATTTCCTCCATCAGAG	21	<lmxll>	ll	119	lm	126		
>ref-134554	TTCCCCCTCACTATTCCTCCCTCCTCA	6	<nnxnp>	nn	127	np	114		
>ref-134556	CACACTCACACGATCCCTCCATCTGGT	4	<lmxll>	ll	134	lm	135		
>ref-134570	CCCTTACCCACCTATCCTCCCCTCCCT	5	<hkxhk>	hh	51	hk	148	kk	46
>ref-134577	TTGCTTTTTACCTTCTCTCCTCCTTAA	15	<nnxnp>	nn	116	np	125		
>ref-134604	AACACACTAACGCCATCTCCAAAGATC	4	<nnxnp>	nn	128	np	138		
>ref-134613	GGTTGGGATACTTTTCCTCCTTTCAGG	7	<hkxhk>	hh	5	hk	251	kk	2
>ref-134613	GGTTGGGATACTTTTCCTCCTTTCAGG	9	<hkxhk>	hh	5	hk	251	kk	2
>ref-134616	TATATATGCACATTTCCTCCCTCTCCC	27	<lmxll>	ll	125	lm	112		
>ref-134627	TATTGTCACACCTTTCCTCCCTTACAT	7	<hkxhk>	hh	4	hk	237	kk	4
>ref-134627	TATTGTCACACCTTTCCTCCCTTACAT	12	<hkxhk>	hh	3	hk	238	kk	4
>ref-134632	CATTTATCGACCCTCCCTCCTTCTCTC	4	<hkxhk>	hh	69	hk	143	kk	49
>ref-134636	CCCTCCGTCACCTTACCTCCTCCTCAT	3	<nnxnp>	nn	122	np	144		
>ref-134636	CCCTCCGTCACCTTACCTCCTCCTCAT	7	<nnxnp>	nn	122	np	144		
>ref-134660	CTTGCCGCCACGGGTCCTCCGCTGTCT	3	<lmxll>	ll	134	lm	127		
>ref-134660	CTTGCCGCCACGGGTCCTCCGCTGTCT	7	<lmxll>	ll	264	lm	0		
>ref-134665	AAACCCCTCACCCTCTCTCCCTCTCCC	6	<lmxll>	ll	256	lm	3		
>ref-134668	AAGTTAAAGACGGTCACTCCAGCCAGT	22	<nnxnp>	nn	107	np	162		
>ref-134676	AAGCGACTCACACTGACTCCTGTCAAT	13	<nnxnp>	nn	137	np	131		
>ref-134676	AAGCGACTCACACTGACTCCTGTCAAT	21	<hkxhk>	hh	59	hk	141	kk	58
>ref-134682	ATATCTTCCACTTGACCTCCACTACAT	1	<hkxhk>	hh	49	hk	143	kk	62
>ref-134698	CCTTACCCTACCCCTTCTCCCACCTTC	27	<nnxnp>	nn	135	np	113		
>ref-134730	CCATTGCGAACGCGTCCTCCTGACTTT	2	<nnxnp>	nn	261	np	0		
>ref-134739	GGGTATCTCACCTTGCCTCCTCATGTC	2	<nnxnp>	nn	121	np	134		
>ref-134749	ACCCTCTCTACCTTCCCTCCCCTTCCC	12	<nnxnp>	nn	266	np	0		
>ref-134749	ACCCTCTCTACCTTCCCTCCCCTTCCC	15	<nnxnp>	nn	117	np	148		
>ref-134754	ATCGCGGGTACGGCGCCTCCTCTCGCT	5	<nnxnp>	nn	137	np	133		
>ref-134789	ATTTGGTAAACTCCTCCTCCTCCTCCT	3	<lmxll>	ll	118	lm	121		
>ref-134797	GCAACGGCAACCAGACCTCCACATCCT	14	<lmxll>	ll	121	lm	143		
>ref-134817	ATCCCCTACACTGGGCCTCCGTCTCCG	9	<nnxnp>	nn	205	np	60		

>ref-134817	ATCCCCTACACTGGGCCTCCGTCTCCG	27	<nnxnp>	nn	206	np	59		
>ref-134834	GATCCTCCAACAATGTCTCCACTGGAC	9	<hkxhk>	hh	53	hk	157	kk	51
>ref-134837	TGTCCATCCACTCTGTCTCCCAGGGGT	14	<lmxll>	ll	127	lm	134		
>ref-134851	AATATGATCACAATCTCTCCCTGTTAT	6	<nnxnp>	nn	110	np	139		
>ref-134856	TACCCATCTACCCTATCTCCCCTCTTT	9	<nnxnp>	nn	242	np	0		
>ref-134856	TACCCATCTACCCTATCTCCCCTCTTT	13	<nnxnp>	nn	239	np	0		
>ref-134862	GTAGTTGTTACAGTCGCTCCTGTCACC	4	<lmxll>	ll	92	lm	173		
>ref-134862	GTAGTTGTTACAGTCGCTCCTGTCACC	12	<hkxhk>	hh	4	hk	266	kk	0
>ref-134878	CTCCCGATCACCCTCCCTCCCATCTCT	4	<lmxll>	ll	126	lm	134		
>ref-134887	AGGAGGGGCACAGCAGCTCCGGGATAT	7	<nnxnp>	nn	149	np	117		
>ref-134889	TTCTTTTATACCTGTTCTCCTTCGTTC	5	<hkxhk>	hh	1	hk	261	kk	2
>ref-134903	CACTCCTACACCTACACTCCTACACCT	4	<hkxhk>	hh	0	hk	268	kk	3
>ref-134910	TCTGGGAGAACCTCTCCTCCGCCTGGC	14	<lmxll>	ll	149	lm	113		
>ref-134923	CCCAACAGTACTACCTCTCCATACCAT	2	<hkxhk>	hh	11	hk	254	kk	0
>ref-134923	CCCAACAGTACTACCTCTCCATACCAT	27	<hkxhk>	hh	1	hk	269	kk	0
>ref-134929	GAGGCGTCGACGAGGACTCCTCGGCAT	14	<lmxll>	ll	139	lm	128		
>ref-134944	TCATCTCCGACCTGACCTCCTGTGGCT	22	<nnxnp>	nn	142	np	122		
>ref-134948	TACCCACTAACCAGATCTCCTTCCCAT	8	<hkxhk>	hh	56	hk	146	kk	45
>ref-134953	TCTACCCTTACCCTCCCTCCCCTCTCA	13	<lmxll>	ll	129	lm	133		
>ref-134985	CACGCACACACAAGCACTCCCCCAAAC	24	<lmxll>	ll	132	lm	119		
>ref-135006	TCTCCTACAACACCATCTCCAACACCT	7	<nnxnp>	nn	141	np	126		
>ref-135006	TCTCCTACAACACCATCTCCAACACCT	26	<nnxnp>	nn	141	np	126		
>ref-135010	TCTCTCTTTACCGCATCTCCTTTGCAA	12	<hkxhk>	hh	64	hk	139	kk	58
>ref-135011	ACTTCCTCCACACGCCCTCCTCGTCGG	3	<nnxnp>	nn	237	np	0		
>ref-135015	GGGTACACTACCCCCCCTCCTTGGTTC	16	<lmxll>	ll	14	lm	252		
>ref-135015	GGGTACACTACCCCCCCTCCTTGGTTC	21	<lmxll>	ll	14	lm	252		
>ref-135080	TAGGAATGGACTCTCTCTCCCTCTCTC	6	<hkxhk>	hh	1	hk	266	kk	0
>ref-135080	TAGGAATGGACTCTCTCTCCCTCTCTC	13	<hkxhk>	hh	1	hk	266	kk	0
>ref-135080	TAGGAATGGACTCTCTCTCCCTCTCTC	24	<hkxhk>	hh	0	hk	207	kk	58
>ref-135088	TATCGCTTTACCGTTTCTCCCCTTCCT	22	<nnxnp>	nn	109	np	140		
>ref-135088	TATCGCTTTACCGTTTCTCCCCTTCCT	23	<hkxhk>	hh	49	hk	133	kk	67
>ref-135091	CCTCTCCCCACCGTTTCTCCTCTTCCT	7	<nnxnp>	nn	121	np	133		
>ref-135143	TAATCTCTCACTTCTTCTCCCTTGCAA	15	<lmxll>	ll	118	lm	144		
>ref-135148	CCCATTTCAACTCCTTCTCCTTCCCTT	7	<lmxll>	ll	130	lm	121		
>ref-135161	TGTTCATGCACCATCTCTCCCTCGAGA	4	<hkxhk>	hh	0	hk	273	kk	0
>ref-135174	CTACCCATCACCCTCTCTCCCTCCGTT	15	<hkxhk>	hh	0	hk	261	kk	7
>ref-135174	CTACCCATCACCCTCTCTCCCTCCGTT	25	<hkxhk>	hh	0	hk	264	kk	6
>ref-135211	TCCTCCCCCACACACACTCCACGCTTT	22	<nnxnp>	nn	134	np	133		

>ref-135230	CTCAATCACACTCACTCTCCCTCTTTC	14	<nnxnp>	nn	262	np	1		
>ref-135236	TCAGAACAAACGGGGCCTCCAGGAACA	1	<hkxhk>	hh	0	hk	273	kk	0
>ref-135236	TCAGAACAAACGGGGCCTCCAGGAACA	16	<hkxhk>	hh	0	hk	259	kk	13
>ref-135236	TCAGAACAAACGGGGCCTCCAGGAACA	24	<hkxhk>	hh	0	hk	273	kk	0
>ref-135241	CCTCTACCAACTCTTCCTCCCACTCTT	22	<hkxhk>	hh	3	hk	264	kk	0
>ref-135241	CCTCTACCAACTCTTCCTCCCACTCTT	23	<hkxhk>	hh	3	hk	264	kk	0
>ref-135248	CAAACAAACACAAACACTCCTCAAACA	8	<hkxhk>	hh	12	hk	256	kk	0
>ref-135248	CAAACAAACACAAACACTCCTCAAACA	21	<hkxhk>	hh	0	hk	256	kk	12
>ref-135251	CTGCTGCGCACGACCTCTCCCGCTTCT	22	<lmxll>	ll	138	lm	135		
>ref-135294	ATGGAAGTAACCGAAACTCCAACTCCT	4	<hkxhk>	hh	0	hk	168	kk	74
>ref-135294	ATGGAAGTAACCGAAACTCCAACTCCT	14	<lmxll>	ll	248	lm	16		
>ref-135300	ATTTTTTCAACGCATTCTCCTCCCTTT	5	<nnxnp>	nn	117	np	116		
>ref-135303	GTCCTCGCTACACTTCCTCCCCTCTTA	26	<lmxll>	ll	107	lm	164		
>ref-135315	GCCACGTACACAATGTCTCCTGTGTGT	9	<nnxnp>	nn	139	np	128		
>ref-135331	GTGTGTATGACTGTGTCTCCGTCTCTT	21	<hkxhk>	hh	1	hk	268	kk	3
>ref-135331	GTGTGTATGACTGTGTCTCCGTCTCTT	23	<hkxhk>	hh	3	hk	268	kk	1
>ref-135331	GTGTGTATGACTGTGTCTCCGTCTCTT	25	<hkxhk>	hh	9	hk	257	kk	2
>ref-135336	GAGGCGACAACAACATCTCCAGGCTTT	24	<nnxnp>	nn	127	np	97		
>ref-135342	ATCCCCTTCACCCTGCCTCCCCCTCCC	25	<lmxll>	ll	131	lm	137		
>ref-135366	ACCGGCTGAACAGGATCTCCTAGCAGG	6	<lmxll>	ll	79	lm	141		
>ref-135366	ACCGGCTGAACAGGATCTCCTAGCAGG	8	<lmxll>	ll	122	lm	97		
>ref-135366	ACCGGCTGAACAGGATCTCCTAGCAGG	9	<lmxll>	ll	10	lm	243		
>ref-135371	AAAAATGTAACCCTTCCTCCTCCAATT	6	<lmxll>	ll	127	lm	121		
>ref-135398	AAAAAACATACACGCACTCCTGCCGTG	9	<nnxnp>	nn	254	np	0		
>ref-135398	AAAAAACATACACGCACTCCTGCCGTG	16	<lmxll>	ll	113	lm	124		
>ref-135400	CCCCTTCCTACCTTTACTCCCACCTCT	27	<nnxnp>	nn	252	np	1		
>ref-135411	TGCCCCACTACGCGCCCTCCTGTAGAA	25	<nnxnp>	nn	262	np	0		
>ref-135411	TGCCCCACTACGCGCCCTCCTGTAGAA	26	<nnxnp>	nn	262	np	0		
>ref-135431	AGAGAGATTACTTCCCCTCCTCCCCTC	15	<nnxnp>	nn	111	np	135		
>ref-135431	AGAGAGATTACTTCCCCTCCTCCCCTC	26	<nnxnp>	nn	258	np	0		
>ref-135441	CGTTTTTCTACACTTCCTCCCCTCCTT	26	<hkxhk>	hh	0	hk	264	kk	4
>ref-135442	CCCCCTTATACGCCTTCTCCTTTCCCC	1	<nnxnp>	nn	135	np	131		
>ref-135442	CCCCCTTATACGCCTTCTCCTTTCCCC	24	<hkxhk>	hh	57	hk	205	kk	0
>ref-135479	TCATCCCCGACTGCCCCTCCACGCCGG	26	<nnxnp>	nn	107	np	150		
>ref-135486	GACACATGCACGGCATCTCCACATAAA	9	<lmxll>	ll	136	lm	129		
>ref-135490	ACTCCCCCAACACTATCTCCCTCACTT	4	<lmxll>	ll	132	lm	131		
>ref-135499	TTCCTTCCCACACAATCTCCCCACTCT	15	<lmxll>	ll	128	lm	138		
>ref-135504	TGCACCCTAACCCTCGCTCCCTAACAC	27	<lmxll>	ll	137	lm	128		

>ref-135535	CTTCCCTCCACCTTCCCTCCCCGACTC	25	<nnxnp>	nn	91	np	158		
>ref-135554	AAATGAATAACTATCACTCCTCCCCCC	14	<lmxll>	ll	141	lm	113		
>ref-135579	GCCGCTCTAACGAGACCTCCTCCTCCT	4	<nnxnp>	nn	264	np	0		
>ref-135584	TCCTCAGCCACACCTACTCCCATTCAC	14	<nnxnp>	nn	271	np	0		
>ref-135586	TGCCTAATCACTCCCTCTCCTCCCTCA	24	<lmxll>	ll	118	lm	147		
>ref-135592	CCCCTCTTCACCTCTCCTCCATCCTCC	7	<nnxnp>	nn	57	np	204		
>ref-135592	CCCCTCTTCACCTCTCCTCCATCCTCC	23	<nnxnp>	nn	55	np	208		
>ref-135604	CTCTATAATACCCATACTCCTTAGATC	3	<nnxnp>	nn	247	np	0		
>ref-135604	CTCTATAATACCCATACTCCTTAGATC	5	<nnxnp>	nn	246	np	0		
>ref-135653	GAAATGGACACTGAGACTCCCATTAAG	27	<nnxnp>	nn	252	np	0		
>ref-135667	CCCACTGACACCCCTTCTCCGTGTGTC	2	<nnxnp>	nn	114	np	147		
>ref-135667	CCCACTGACACCCCTTCTCCGTGTGTC	16	<hkxhk>	hh	51	hk	144	kk	75
>ref-135669	TTGTCCCTCACTCCTCCTCCTCCCTTT	1	<nnxnp>	nn	8	np	246		
>ref-135669	TTGTCCCTCACTCCTCCTCCTCCCTTT	6	<nnxnp>	nn	8	np	248		
>ref-135672	AGATCCTTGACGAATTCTCCATTTATC	12	<hkxhk>	hh	6	hk	223	kk	3
>ref-135672	AGATCCTTGACGAATTCTCCATTTATC	26	<hkxhk>	hh	3	hk	219	kk	4
>ref-135702	ACTCTTAACACTTCCTCTCCAAAGACT	5	<hkxhk>	hh	8	hk	177	kk	37
>ref-135702	ACTCTTAACACTTCCTCTCCAAAGACT	27	<hkxhk>	hh	8	hk	177	kk	37
>ref-135703	CATGCAAGCACTCTCGCTCCTTCTCTC	15	<lmxll>	ll	135	lm	138		
>ref-135709	TCCGGCCTCACTCATTCTCCAACATTA	14	<hkxhk>	hh	0	hk	261	kk	3
>ref-135709	TCCGGCCTCACTCATTCTCCAACATTA	25	<hkxhk>	hh	3	hk	261	kk	0
>ref-135720	AGCTACTCCACCCCTGCTCCACCACTC	27	<hkxhk>	hh	24	hk	240	kk	0
>ref-135722	GACATGTAAACAACACCTCCCTATCCT	23	<lmxll>	ll	114	lm	136		
>ref-135732	TCTACCACTACACCCCCTCCCTCCTCC	2	<nnxnp>	nn	257	np	1		
>ref-135734	TTCTTTCTCACATTCTCTCCTTTCCCT	5	<lmxll>	ll	145	lm	105		
>ref-135734	TTCTTTCTCACATTCTCTCCTTTCCCT	9	<lmxll>	ll	143	lm	104		
>ref-135790	TATGTACGTACAACATCTCCCTGCTTT	13	<nnxnp>	nn	122	np	136		
>ref-135790	TATGTACGTACAACATCTCCCTGCTTT	26	<nnxnp>	nn	126	np	139		
>ref-135792	ACATTCATGACACTATCTCCACCTCGT	1	<hkxhk>	hh	52	hk	143	kk	67
>ref-135800	GTGATAACCACCTGCGCTCCTTCTATT	15	<lmxll>	ll	130	lm	131		
>ref-135814	CCAAGAGCAACACCGCCTCCAACACCA	2	<lmxll>	ll	136	lm	128		
>ref-135820	GCGAGCGACACGACAGCTCCGGCGATT	6	<lmxll>	ll	125	lm	138		
>ref-135829	CCTCCACCCACTCTCCCTCCCCTCTTC	7	<hkxhk>	hh	0	hk	47	kk	187
>ref-135829	CCTCCACCCACTCTCCCTCCCCTCTTC	8	<nnxnp>	nn	265	np	2		
>ref-135829	CCTCCACCCACTCTCCCTCCCCTCTTC	13	<hkxhk>	hh	0	hk	49	kk	186
>ref-135829	CCTCCACCCACTCTCCCTCCCCTCTTC	27	<lmxll>	ll	268	lm	1		
>ref-135835	TTTGTTAAAACTAAGCCTCCTTGATCA	24	<lmxll>	ll	122	lm	107		
>ref-135840	AATCTTTTGACAGTCCCTCCACGCACA	4	<lmxll>	ll	146	lm	113		

>ref-135843	CGAAGATTAACTCGCTCTCCCCTTCTT	12	<nnxnp>	nn	33	np	221		
>ref-135843	CGAAGATTAACTCGCTCTCCCCTTCTT	22	<hkxhk>	hh	1	hk	76	kk	170
>ref-135851	TTTGCGCCAACCTTTCCTCCCTTCCTT	6	<lmxll>	ll	98	lm	157		
>ref-135874	TTTTCCCCCACTTCTTCTCCTCCTCTG	16	<nnxnp>	nn	116	np	122		
>ref-135878	TTCCCATCCACCCACTCTCCCTCCTCT	2	<lmxll>	ll	224	lm	5		
>ref-135878	TTCCCATCCACCCACTCTCCCTCCTCT	3	<lmxll>	ll	233	lm	3		
>ref-135878	TTCCCATCCACCCACTCTCCCTCCTCT	7	<lmxll>	ll	209	lm	14		
>ref-135878	TTCCCATCCACCCACTCTCCCTCCTCT	12	<lmxll>	ll	235	lm	3		
>ref-135878	TTCCCATCCACCCACTCTCCCTCCTCT	14	<lmxll>	ll	202	lm	24		
>ref-135878	TTCCCATCCACCCACTCTCCCTCCTCT	16	<lmxll>	ll	226	lm	6		
>ref-135878	TTCCCATCCACCCACTCTCCCTCCTCT	21	<lmxll>	ll	230	lm	3		
>ref-135878	TTCCCATCCACCCACTCTCCCTCCTCT	22	<lmxll>	ll	228	lm	4		
>ref-135878	TTCCCATCCACCCACTCTCCCTCCTCT	23	<lmxll>	ll	234	lm	3		
>ref-135878	TTCCCATCCACCCACTCTCCCTCCTCT	25	<lmxll>	ll	208	lm	15		
>ref-135878	TTCCCATCCACCCACTCTCCCTCCTCT	26	<lmxll>	ll	232	lm	3		
>ref-135878	TTCCCATCCACCCACTCTCCCTCCTCT	27	<lmxll>	ll	226	lm	3		
>ref-135884	TGACGAACCACTCTACCTCCTTTTCCC	7	<lmxll>	ll	129	lm	143		
>ref-135884	TGACGAACCACTCTACCTCCTTTTCCC	15	<lmxll>	ll	1	lm	268		
>ref-135884	TGACGAACCACTCTACCTCCTTTTCCC	24	<hkxhk>	hh	0	hk	269	kk	0
>ref-135886	GGCTCCACAACCCCGACTCCATTTTTT	27	<lmxll>	ll	230	lm	0		
>ref-135901	CTTCCTTAGACATCTTCTCCGTTATAA	27	<lmxll>	ll	105	lm	150		
>ref-135912	CCAGCGCCAACAGGTCCTCCACCTTTT	1	<nnxnp>	nn	122	np	135		
>ref-135932	ATCATTATAACACCCCCTCCACCACTA	15	<nnxnp>	nn	126	np	127		
>ref-135932	ATCATTATAACACCCCCTCCACCACTA	26	<nnxnp>	nn	128	np	127		
>ref-135941	TCTCTTCATACTTATCCTCCTCTCCTC	12	<nnxnp>	nn	114	np	133		
>ref-135941	TCTCTTCATACTTATCCTCCTCTCCTC	26	<lmxll>	ll	128	lm	128		
>ref-135950	TCTCCGTTCACAATAACTCCAAGCCCC	12	<nnxnp>	nn	131	np	129		
>ref-135950	TCTCCGTTCACAATAACTCCAAGCCCC	23	<nnxnp>	nn	128	np	134		
>ref-135954	ACTAGACTGACAGTATCTCCTTGTGGA	7	<lmxll>	ll	117	lm	138		
>ref-135984	TCCCTATTTACCTTTGCTCCCCTTTTA	2	<nnxnp>	nn	248	np	0		
>ref-135984	TCCCTATTTACCTTTGCTCCCCTTTTA	5	<nnxnp>	nn	117	np	120		
>ref-136009	GTCCACGTAACGCTTCCTCCACCTCTC	13	<hkxhk>	hh	0	hk	272	kk	0
>ref-136009	GTCCACGTAACGCTTCCTCCACCTCTC	23	<hkxhk>	hh	0	hk	272	kk	0
>ref-136012	CTCCCTCTTACTCATACTCCGTATCTG	21	<lmxll>	ll	115	lm	127		
>ref-136020	TTCCCGTGTACGTACGCTCCCACGTTC	5	<nnxnp>	nn	122	np	147		
>ref-136028	CCGTGGGATACTCCTCCTCCTCCTCCT	5	<lmxll>	ll	132	lm	125		
>ref-136064	TTTCCTCTCACATCCCCTCCCTCCTTA	27	<nnxnp>	nn	5	np	258		
>ref-136068	TCCCTCAAGACTATCGCTCCATCGCAC	1	<hkxhk>	hh	0	hk	263	kk	7

>ref-136068	TCCCTCAAGACTATCGCTCCATCGCAC	15	<hkxhk>	hh	0	hk	273	kk	0
>ref-136068	TCCCTCAAGACTATCGCTCCATCGCAC	24	<lmxll>	ll	124	lm	131		
>ref-136078	CGACAAAGAACTAAGCCTCCCACAGTG	5	<hkxhk>	hh	0	hk	273	kk	0
>ref-136083	TTGTCACTTACGCAGTCTCCCCTCAGT	9	<nnxnp>	nn	273	np	0		
>ref-136093	ACACCTTTTACTCTTTCTCCTTTTTCT	22	<nnxnp>	nn	113	np	130		
>ref-136108	GCTCTTTCCACGTCTTCTCCCCGTCTT	8	<nnxnp>	nn	123	np	141		
>ref-136149	CGAAGAGACACGCTTCCTCCAGCCAAG	26	<nnxnp>	nn	123	np	140		
>ref-136158	CTCCCCTTTACCCCCCCTCCTCTCCTG	1	<lmxll>	ll	14	lm	229		
>ref-136174	AAACCTTCGACGTCGCCTCCGCCTCCC	21	<nnxnp>	nn	140	np	132		
>ref-136182	ATACAAAGTACATACACTCCAAACACA	24	<nnxnp>	nn	132	np	135		
>ref-136187	GTTTTGGCCACCACTCCTCCTACATGG	27	<hkxhk>	hh	47	hk	143	kk	55
>ref-136203	CCCTACTATACCGAATCTCCTTGCATG	12	<nnxnp>	nn	128	np	129		
>ref-136211	GACAGACAGACAGACTCTCCAAGCACA	21	<hkxhk>	hh	5	hk	265	kk	0
>ref-136211	GACAGACAGACAGACTCTCCAAGCACA	25	<hkxhk>	hh	5	hk	265	kk	0
>ref-136218	TCTCTTTCTACAAGTCCTCCTGCTTTT	14	<lmxll>	ll	145	lm	117		
>ref-136235	GCGTGACCTACTTTGTCTCCGCCTTGT	26	<lmxll>	ll	127	lm	138		
>ref-136251	ACAACGTGAACCTAGGCTCCCTCTCTC	12	<nnxnp>	nn	132	np	138		
>ref-136255	AATCGTTTGACAATTACTCCGGTCATT	16	<lmxll>	ll	124	lm	122		
>ref-136297	AAGTAACTAACAGGCGCTCCTTACTTT	16	<lmxll>	ll	91	lm	134		
>ref-136304	AAAGATGTCACAAGGCCTCCATTTCTT	2	<nnxnp>	nn	100	np	125		
>ref-136342	TATAGGCTTACCTGTGCTCCTCGGTAC	22	<nnxnp>	nn	123	np	146		
>ref-136344	CTACGTCCTACATCACCTCCATCTCTT	4	<lmxll>	ll	139	lm	128		
>ref-136350	CTCGCTCCGACGCTATCTCCTGCCTAC	22	<lmxll>	ll	141	lm	127		
>ref-136351	CACACATATACAAACCCTCCCCCCCTC	1	<nnxnp>	nn	147	np	104		
>ref-136356	TAATTCAGGACTTTCCCTCCGTGGAGT	24	<lmxll>	ll	121	lm	138		
>ref-136358	TCCCCGTCCACTTCCTCTCCCCACGTC	8	<lmxll>	ll	139	lm	123		
>ref-136372	CCTGTCCCTACACCCCCTCCCTTATCA	7	<nnxnp>	nn	109	np	149		
>ref-136385	ATCTCCAGCACTTTGTCTCCAACTCCT	1	<hkxhk>	hh	47	hk	196	kk	0
>ref-136385	ATCTCCAGCACTTTGTCTCCAACTCCT	14	<hkxhk>	hh	0	hk	247	kk	8
>ref-136431	ATTGCCTCTACCTCGCCTCCTTGTACA	5	<nnxnp>	nn	129	np	131		
>ref-136435	TCCTCCTCCACCGGTCCTCCTCCACCG	3	<nnxnp>	nn	4	np	263		
>ref-136435	TCCTCCTCCACCGGTCCTCCTCCACCG	23	<nnxnp>	nn	18	np	245		
>ref-136435	TCCTCCTCCACCGGTCCTCCTCCACCG	25	<hkxhk>	hh	1	hk	266	kk	2
>ref-136435	TCCTCCTCCACCGGTCCTCCTCCACCG	27	<hkxhk>	hh	1	hk	160	kk	100
>ref-136438	ACCTCGAGTACAAAGCCTCCTCCACCA	23	<lmxll>	ll	133	lm	132		
>ref-136438	ACCTCGAGTACAAAGCCTCCTCCACCA	26	<lmxll>	ll	131	lm	132		
>ref-136447	TCCCCCAAAACAATCCCTCCTCTCCTC	7	<hkxhk>	hh	1	hk	271	kk	0
>ref-136447	TCCCCCAAAACAATCCCTCCTCTCCTC	13	<hkxhk>	hh	1	hk	271	kk	0

>ref-136488	TACTAACTCACCATCTCTCCCACGCAT	1	<lmxll>	ll	129	lm	135		
>ref-136491	GCAAAAGCTACATCACCTCCTTTTACC	25	<hkxhk>	hh	45	hk	128	kk	51
>ref-136491	GCAAAAGCTACATCACCTCCTTTTACC	26	<hkxhk>	hh	51	hk	128	kk	45
>ref-136524	GATACGCGCACAGCTTCTCCTTTTCCG	15	<lmxll>	ll	128	lm	135		
>ref-136524	GATACGCGCACAGCTTCTCCTTTTCCG	24	<lmxll>	ll	128	lm	135		
>ref-136529	GGCCAATGGACTCCACCTCCGACACTG	16	<lmxll>	ll	139	lm	128		
>ref-136535	ACGCCCACCACACTCCCTCCACGCCCA	7	<nnxnp>	nn	9	np	258		
>ref-136535	ACGCCCACCACACTCCCTCCACGCCCA	27	<hkxhk>	hh	0	hk	273	kk	0
>ref-136549	CCCCTTCCCACCATTCCTCCTCCCTCC	14	<nnxnp>	nn	151	np	95		
>ref-136563	TCACCCATAACACCGTCTCCTAACACT	16	<lmxll>	ll	37	lm	218		
>ref-136563	TCACCCATAACACCGTCTCCTAACACT	27	<lmxll>	ll	37	lm	217		
>ref-136611	ATGAGGTATACTGCATCTCCCTTGCTG	15	<hkxhk>	hh	59	hk	154	kk	43
>ref-136611	ATGAGGTATACTGCATCTCCCTTGCTG	22	<nnxnp>	nn	128	np	132		
>ref-136618	TACTCAAACACATTGACTCCCTTGTAA	15	<hkxhk>	hh	0	hk	137	kk	92
>ref-136649	GAAATAATTACGAGTCCTCCTTCCTCA	6	<nnxnp>	nn	108	np	150		
>ref-136656	TCTTATTCCACTTCCTCTCCTCATCTA	7	<nnxnp>	nn	2	np	268		
>ref-136656	TCTTATTCCACTTCCTCTCCTCATCTA	26	<nnxnp>	nn	2	np	268		
>ref-136658	AAGATCCCAACTCCTCCTCCAGCCCTA	3	<nnxnp>	nn	140	np	120		
>ref-136724	ATGGTTATAACCCGCTCTCCAGCTGCA	3	<hkxhk>	hh	2	hk	270	kk	0
>ref-136724	ATGGTTATAACCCGCTCTCCAGCTGCA	26	<hkxhk>	hh	2	hk	270	kk	0
>ref-136740	AAGTTTGCGACTGATGCTCCGAGTCAT	1	<hkxhk>	hh	0	hk	273	kk	0
>ref-136740	AAGTTTGCGACTGATGCTCCGAGTCAT	25	<lmxll>	ll	159	lm	95		
>ref-136743	ACAGGCACAACTGCATCTCCTTCCCAA	8	<lmxll>	ll	141	lm	104		
>ref-136763	CATCTGTACACTTTGTCTCCTTCTTCC	22	<lmxll>	ll	122	lm	135		
>ref-136765	TAAAAGCCTACCCCCACTCCCCACCCC	12	<lmxll>	ll	99	lm	129		
>ref-136769	CTTACTCACACTTCCTCTCCCTTCTTT	21	<hkxhk>	hh	1	hk	193	kk	75
>ref-136775	ACGATGTTTACTGCCACTCCATTCGAA	2	<hkxhk>	hh	53	hk	128	kk	48
>ref-136782	TTTCATGCAACGCTTCCTCCTGTGTTT	22	<hkxhk>	hh	49	hk	132	kk	64
>ref-136794	TATGCATACACCACATCTCCCTGCAGG	4	<lmxll>	ll	114	lm	134		
>ref-136825	CTCGCCTCCACCCTCTCTCCCTCTGCC	4	<nnxnp>	nn	225	np	11		
>ref-136825	CTCGCCTCCACCCTCTCTCCCTCTGCC	14	<nnxnp>	nn	127	np	130		
>ref-136825	CTCGCCTCCACCCTCTCTCCCTCTGCC	23	<hkxhk>	hh	54	hk	165	kk	34
>ref-136825	CTCGCCTCCACCCTCTCTCCCTCTGCC	25	<nnxnp>	nn	224	np	20		
>ref-136835	TCCTCTCCCACCCGACCTCCTACACCT	14	<lmxll>	ll	145	lm	111		
>ref-136846	CCCTCGCCAACAGCACCTCCTCCCTCG	15	<nnxnp>	nn	227	np	0		
>ref-136859	GGGTGTGTCACCAAAACTCCAGAGCTC	2	<nnxnp>	nn	29	np	232		
>ref-136859	GGGTGTGTCACCAAAACTCCAGAGCTC	22	<lmxll>	ll	0	lm	273		
>ref-136859	GGGTGTGTCACCAAAACTCCAGAGCTC	25	<hkxhk>	hh	3	hk	264	kk	0

>ref-136860	GTGCGTATGACCGTCTCTCCCTGCCCT	13	<lmxll>	ll	124	lm	146		
>ref-136880	ATGCAAGCAACCGCTACTCCAATGCTA	13	<nnxnp>	nn	139	np	128		
>ref-136892	CGCTTCCCCACCCACTCTCCGTCCATT	15	<nnxnp>	nn	129	np	138		
>ref-136903	ACCAATATTACCCCAGCTCCTTTCCCC	21	<hkxhk>	hh	61	hk	120	kk	45
>ref-136912	AACCTGGACACGGACCCTCCCAAGCCC	14	<lmxll>	ll	148	lm	119		
>ref-136918	AAGCCCTTGACCGCATCTCCATCCATA	13	<nnxnp>	nn	135	np	134		
>ref-136923	ACTCAAAACACTGACACTCCACTTTTA	21	<hkxhk>	hh	2	hk	266	kk	0
>ref-136923	ACTCAAAACACTGACACTCCACTTTTA	25	<hkxhk>	hh	0	hk	265	kk	1
>ref-136923	ACTCAAAACACTGACACTCCACTTTTA	27	<hkxhk>	hh	2	hk	266	kk	0
>ref-136930	TTTACCCCCACCCCTCCTCCTCTTCTC	12	<lmxll>	ll	219	lm	10		
>ref-136930	TTTACCCCCACCCCTCCTCCTCTTCTC	27	<lmxll>	ll	208	lm	17		
>ref-136936	TATAGTCAGACTCTGCCTCCACCCCTA	26	<lmxll>	ll	247	lm	0		
>ref-136943	CACTAACCCACAGCCCCTCCTTATCAC	27	<lmxll>	ll	229	lm	3		
>ref-136966	CTCTCCACCACCCTTCCTCCCCTTCTC	5	<lmxll>	ll	168	lm	89		
>ref-136966	CTCTCCACCACCCTTCCTCCCCTTCTC	14	<nnxnp>	nn	9	np	256		
>ref-136973	TCTTTATCTACCCTCTCTCCCCCTCCA	6	<nnxnp>	nn	255	np	2		
>ref-136993	CCACCTCCAACACTTCCTCCTTCCCAT	6	<nnxnp>	nn	123	np	134		
>ref-137029	TCAGACAAGACAATTTCTCCTCGTCCC	4	<hkxhk>	hh	3	hk	264	kk	1
>ref-137029	TCAGACAAGACAATTTCTCCTCGTCCC	22	<hkxhk>	hh	3	hk	264	kk	1
>ref-137031	TTTGTTTTAACCAATGCTCCAGTCTTT	2	<lmxll>	ll	11	lm	249		
>ref-137031	TTTGTTTTAACCAATGCTCCAGTCTTT	5	<lmxll>	ll	11	lm	249		
>ref-137054	ACACTGTATACTCCCTCTCCCTCTCCC	6	<lmxll>	ll	4	lm	266		
>ref-137054	ACACTGTATACTCCCTCTCCCTCTCCC	12	<lmxll>	ll	4	lm	266		
>ref-137063	GACACCGACACCTCATCTCCACCTTCC	22	<nnxnp>	nn	123	np	145		
>ref-137074	CTCCTCCCCACCCCTCCTCCCCCACTT	16	<lmxll>	ll	200	lm	45		
>ref-137074	CTCCTCCCCACCCCTCCTCCCCCACTT	21	<lmxll>	ll	177	lm	60		
>ref-137074	CTCCTCCCCACCCCTCCTCCCCCACTT	26	<lmxll>	ll	30	lm	218		
>ref-137077	ATCCCCCTAACCTCTACTCCTTCACCC	27	<lmxll>	ll	125	lm	139		
>ref-137095	TCACTTAAGACCTGTTCTCCCTCCCTC	5	<nnxnp>	nn	128	np	139		
>ref-137095	TCACTTAAGACCTGTTCTCCCTCCCTC	21	<hkxhk>	hh	55	hk	148	kk	60
>ref-137098	TTGTGCGAGACACTCTCTCCTCGCCGT	23	<hkxhk>	hh	1	hk	272	kk	0
>ref-137101	AACAATTCGACTGACGCTCCCTGAAAT	25	<hkxhk>	hh	47	hk	147	kk	45
>ref-137119	TAGACGCCTACCTATTCTCCCCATTAA	5	<hkxhk>	hh	1	hk	107	kk	111
>ref-137119	TAGACGCCTACCTATTCTCCCCATTAA	6	<lmxll>	ll	25	lm	213		
>ref-137119	TAGACGCCTACCTATTCTCCCCATTAA	27	<lmxll>	ll	216	lm	24		
>ref-137131	CAAGTATCAACAAACGCTCCGTCTCCA	4	<nnxnp>	nn	119	np	144		
>ref-137169	ATTCTTCTAACAGCCTCTCCTTTTCCT	13	<lmxll>	ll	133	lm	114		
>ref-137173	TCTCTCAGTACTTCAGCTCCCCTCCAC	16	<lmxll>	ll	129	lm	128		

>ref-137182	AGTCCCCGCACGCCGCCTCCGGGCCGT	21	<nnxnp>	nn	120	np	140		
>ref-137205	TACCCCCCAACAGCATCTCCGCATCCC	2	<hkxhk>	hh	58	hk	136	kk	70
>ref-137205	TACCCCCCAACAGCATCTCCGCATCCC	5	<nnxnp>	nn	123	np	147		
>ref-137209	CAACCTTCTACCCCCTCTCCCTACCTT	2	<hkxhk>	hh	9	hk	207	kk	29
>ref-137209	CAACCTTCTACCCCCTCTCCCTACCTT	25	<hkxhk>	hh	9	hk	206	kk	29
>ref-137211	CCCCCAACCACACCATCTCCTCCCCTT	5	<nnxnp>	nn	123	np	141		
>ref-137230	TTCTCCTCTACCTTTCCTCCTCCCCAT	5	<lmxll>	ll	22	lm	233		
>ref-137230	TTCTCCTCTACCTTTCCTCCTCCCCAT	27	<lmxll>	ll	22	lm	235		
>ref-137238	TCCTCCTTTACCTGTCCTCCTCCCTCC	7	<hkxhk>	hh	0	hk	259	kk	7
>ref-137238	TCCTCCTTTACCTGTCCTCCTCCCTCC	9	<nnxnp>	nn	11	np	250		
>ref-137238	TCCTCCTTTACCTGTCCTCCTCCCTCC	14	<hkxhk>	hh	3	hk	262	kk	2
>ref-137241	ATCTTTGGAACTCTGTCTCCCTATATT	4	<lmxll>	ll	121	lm	104		
>ref-137259	AAGGGCCTCACTCCCTCTCCACATTCC	12	<nnxnp>	nn	139	np	129		
>ref-137307	CACCGCTAGACCAAGACTCCCACGCCC	7	<nnxnp>	nn	2	np	270		
>ref-137307	CACCGCTAGACCAAGACTCCCACGCCC	16	<hkxhk>	hh	0	hk	273	kk	0
>ref-137308	CTCTCTCTCACTACGTCTCCTTCATTC	13	<lmxll>	ll	139	lm	126		
>ref-137324	GACGAAGGTACTCAGCCTCCTCATGGA	15	<nnxnp>	nn	10	np	262		
>ref-137324	GACGAAGGTACTCAGCCTCCTCATGGA	21	<nnxnp>	nn	10	np	258		
>ref-137324	GACGAAGGTACTCAGCCTCCTCATGGA	22	<nnxnp>	nn	10	np	263		
>ref-137344	AGAGTAACAACTGTTCCTCCAGTGACT	3	<hkxhk>	hh	15	hk	253	kk	0
>ref-137349	CCCTTTCCCACTCGTTCTCCTCTCCCC	4	<lmxll>	ll	124	lm	131		
>ref-137349	CCCTTTCCCACTCGTTCTCCTCTCCCC	6	<lmxll>	ll	123	lm	131		
>ref-137362	CAACGCCTCACTCCCCCTCCCCCATCT	12	<lmxll>	ll	119	lm	151		
>ref-137373	TATACACACACTCATACTCCTTTCTCA	13	<nnxnp>	nn	130	np	129		
>ref-137373	TATACACACACTCATACTCCTTTCTCA	21	<lmxll>	ll	148	lm	110		
>ref-137378	TCCCCCTCCACCCCAGCTCCCTCACTT	22	<lmxll>	ll	122	lm	130		
>ref-137383	TTTACTCCCACCTTCCCTCCTCTCTGT	24	<hkxhk>	hh	2	hk	196	kk	53
>ref-137393	TGCAGAGCGACGGAGGCTCCGACCGGG	7	<hkxhk>	hh	0	hk	271	kk	1
>ref-137393	TGCAGAGCGACGGAGGCTCCGACCGGG	21	<hkxhk>	hh	0	hk	265	kk	7
>ref-137395	CCCCCTCCCACTCTCCCTCCACCAACG	1	<lmxll>	ll	113	lm	121		
>ref-137404	TCTCTTCCCACCTGCTCTCCCTTTTTT	7	<lmxll>	ll	109	lm	121		
>ref-137406	ATAAAACCCACATACGCTCCCCACACA	21	<nnxnp>	nn	258	np	0		
>ref-137406	ATAAAACCCACATACGCTCCCCACACA	23	<hkxhk>	hh	49	hk	132	kk	60
>ref-137412	TTATTCCCCACTTACCCTCCTCTTCCT	5	<lmxll>	ll	128	lm	133		
>ref-137412	TTATTCCCCACTTACCCTCCTCTTCCT	6	<lmxll>	ll	128	lm	133		
>ref-137412	TTATTCCCCACTTACCCTCCTCTTCCT	22	<nnxnp>	nn	123	np	137		
>ref-137431	CTTGTCTCCACGTCTTCTCCTCAGATG	2	<hkxhk>	hh	0	hk	232	kk	34
>ref-137431	CTTGTCTCCACGTCTTCTCCTCAGATG	21	<hkxhk>	hh	0	hk	232	kk	34

>ref-137431	CTTGTCTCCACGTCTTCTCCTCAGATG	24	<nnxnp>	nn	127	np	130			
>ref-137433	CTTCTCCCAACCATCTCTCCCCTTCTC	9	<hkxhk>	hh	1	hk	266	kk	2
>ref-137433	CTTCTCCCAACCATCTCTCCCCTTCTC	16	<hkxhk>	hh	2	hk	266	kk	1
>ref-137458	ACTACCTCGACAATATCTCCTTCTGCC	3	<nnxnp>	nn	102	np	142		
>ref-137459	AAGGACACAACATAACCTCCTGGCACA	4	<hkxhk>	hh	65	hk	145	kk	59
>ref-137463	ACAAAACTAACTTATGCTCCGTTTAAA	2	<hkxhk>	hh	3	hk	258	kk	1
>ref-137463	ACAAAACTAACTTATGCTCCGTTTAAA	3	<hkxhk>	hh	1	hk	259	kk	3
>ref-137478	TTCCCTTATACTCACTCTCCCTAACTT	1	<lmxll>	ll	125	lm	137		
>ref-137478	TTCCCTTATACTCACTCTCCCTAACTT	3	<lmxll>	ll	125	lm	137		
>ref-137514	GTTATTGACACTTCATCTCCTCTTCCT	5	<nnxnp>	nn	113	np	130		
>ref-137525	ATTGTGGAAACGCTTTCTCCACTGTTT	27	<lmxll>	ll	130	lm	134		
>ref-137529	CGCATAAAAACATACACTCCCGCACGA	7	<nnxnp>	nn	110	np	137		
>ref-137529	CGCATAAAAACATACACTCCCGCACGA	13	<nnxnp>	nn	109	np	138		
>ref-137536	AATGGCCACACACCGCCTCCCGACCCT	1	<nnxnp>	nn	271	np	0		
>ref-137536	AATGGCCACACACCGCCTCCCGACCCT	2	<nnxnp>	nn	272	np	0		
>ref-137537	TCGCCTCTTACCTATCCTCCCTCCTCA	1	<nnxnp>	nn	106	np	148		
>ref-137537	TCGCCTCTTACCTATCCTCCCTCCTCA	16	<nnxnp>	nn	106	np	147		
>ref-137538	ATAACTCTCACCTCCCCTCCTCCCTTG	8	<nnxnp>	nn	126	np	129		
>ref-137538	ATAACTCTCACCTCCCCTCCTCCCTTG	14	<lmxll>	ll	123	lm	127		
>ref-137588	CCCCCATCTACTTTCCCTCCCGTCATC	14	<lmxll>	ll	107	lm	127		
>ref-137593	GTCTGCCCCACTTTCCCTCCCACAGGT	22	<nnxnp>	nn	137	np	133		
>ref-137601	ACTGGCCTGACACTTGCTCCTTTAGCA	7	<lmxll>	ll	122	lm	135		
>ref-137605	ACTTCTACAACCGCCTCTCCGACTACC	13	<hkxhk>	hh	0	hk	273	kk	0
>ref-137606	TTGCCTCGCACGGAATCTCCTTGGGAA	12	<lmxll>	ll	129	lm	136		
>ref-137610	CCCTGAGAAACCTGAACTCCAAGCCCT	1	<lmxll>	ll	196	lm	62		
>ref-137610	CCCTGAGAAACCTGAACTCCAAGCCCT	2	<hkxhk>	hh	0	hk	215	kk	26
>ref-137610	CCCTGAGAAACCTGAACTCCAAGCCCT	7	<lmxll>	ll	224	lm	40		
>ref-137623	CAAACATGTACACATCCTCCTCACACC	22	<hkxhk>	hh	115	hk	113	kk	0
>ref-137631	AGCCCAGCGACTCACCCTCCTTGTCAC	15	<hkxhk>	hh	0	hk	267	kk	0
>ref-137631	AGCCCAGCGACTCACCCTCCTTGTCAC	27	<hkxhk>	hh	0	hk	231	kk	31
>ref-137658	TGCTTTCTCACCGTCTCTCCATTCCCC	2	<nnxnp>	nn	146	np	119		
>ref-137658	TGCTTTCTCACCGTCTCTCCATTCCCC	7	<nnxnp>	nn	147	np	119		
>ref-137661	ATGTCAACAACGCTCACTCCAGCCATC	21	<nnxnp>	nn	137	np	129		
>ref-137680	CAAGCTTGCACGCCGTCTCCCCCGCCT	2	<lmxll>	ll	126	lm	138		
>ref-137713	GCTAAGCTAACGCCTCCTCCTCCCGGC	5	<hkxhk>	hh	0	hk	177	kk	80
>ref-137713	GCTAAGCTAACGCCTCCTCCTCCCGGC	8	<hkxhk>	hh	80	hk	177	kk	0
>ref-137714	TACGCGTACACTCTCTCTCCCTTATTA	26	<lmxll>	ll	136	lm	116		
>ref-137723	TATCAAGCCACTTAAACTCCATTCCTG	26	<nnxnp>	nn	112	np	127		

>ref-137724	ATTAAAACTACCATTCCTCCAGCCGGG	24	<nnxnp>	nn	102	np	123		
>ref-137730	TATCAGGCTACTTACCCTCCTAAATAT	4	<nnxnp>	nn	79	np	140		
>ref-137730	TATCAGGCTACTTACCCTCCTAAATAT	12	<nnxnp>	nn	79	np	141		
>ref-137743	GGAGTTGTCACTTCTCCTCCTTGGCCA	12	<lmxll>	ll	124	lm	143		
>ref-137757	GCTAGTATTACCAAGGCTCCTTATACA	22	<nnxnp>	nn	136	np	126		
>ref-137805	CGGACATGAACAGCCTCTCCTACTCGC	23	<nnxnp>	nn	264	np	0		
>ref-137809	TAGCAGATTACCATATCTCCACTTATA	6	<nnxnp>	nn	113	np	116		
>ref-137838	AACTGCCTGACTCTCGCTCCCTGCATC	1	<nnxnp>	nn	120	np	141		
>ref-137838	AACTGCCTGACTCTCGCTCCCTGCATC	2	<nnxnp>	nn	120	np	141		
>ref-137843	AAGGTGCACACAGCCTCTCCAGCTTTC	1	<hkxhk>	hh	73	hk	126	kk	36
>ref-137847	TCCTCTTCAACACCCCCTCCTCCACTC	16	<hkxhk>	hh	9	hk	257	kk	0
>ref-137847	TCCTCTTCAACACCCCCTCCTCCACTC	24	<hkxhk>	hh	8	hk	257	kk	0
>ref-137874	CTCAATATCACTTGGCCTCCTGAATGT	22	<nnxnp>	nn	243	np	0		
>ref-137886	ACGCTTCCCACCTCCTCTCCCTTCTCT	25	<hkxhk>	hh	65	hk	146	kk	59
>ref-137895	TCCCCTTTTACTCCGCCTCCTATTCAC	27	<nnxnp>	nn	131	np	141		
>ref-137902	GGTGCCGACACAGCTCCTCCGGCGTGT	16	<nnxnp>	nn	271	np	0		
>ref-137915	AAAGTCGAGACACGAGCTCCGTGTCCG	21	<nnxnp>	nn	120	np	109		
>ref-137916	CATCATTGCACTTTCCCTCCTTCTTTT	25	<lmxll>	ll	111	lm	140		
>ref-137936	AAGACGGAAACATTTTCTCCCGAGCGG	4	<nnxnp>	nn	108	np	124		
>ref-137938	ATGGCAGCGACTCCTCCTCCTCTCCTT	5	<nnxnp>	nn	18	np	252		
>ref-137938	ATGGCAGCGACTCCTCCTCCTCTCCTT	23	<nnxnp>	nn	18	np	252		
>ref-138009	CCTATCCAGACCCTTTCTCCTCTCTCT	23	<lmxll>	ll	119	lm	145		
>ref-138020	CCATCACAAACACACTCTCCATCCTCA	1	<hkxhk>	hh	46	hk	142	kk	62
>ref-138034	CCACCACCAACAACAACTCCCACAACT	21	<hkxhk>	hh	0	hk	256	kk	6
>ref-138034	CCACCACCAACAACAACTCCCACAACT	26	<hkxhk>	hh	0	hk	256	kk	6
>ref-138042	TAAACATAGACAGCATCTCCCTCTTTG	23	<lmxll>	ll	146	lm	110		
>ref-138043	ATAGACAAAACACTTACTCCCAGGCTG	7	<lmxll>	ll	116	lm	141		
>ref-138060	TGCTCTGAGACTGAAACTCCATTTATT	7	<hkxhk>	hh	0	hk	253	kk	14
>ref-138060	TGCTCTGAGACTGAAACTCCATTTATT	13	<nnxnp>	nn	112	np	151		
>ref-138060	TGCTCTGAGACTGAAACTCCATTTATT	27	<hkxhk>	hh	0	hk	250	kk	15
>ref-138096	CTTTAACCAACGCGTCCTCCCAGTTAT	1	<hkxhk>	hh	20	hk	211	kk	0
>ref-138096	CTTTAACCAACGCGTCCTCCCAGTTAT	2	<hkxhk>	hh	0	hk	76	kk	192
>ref-138096	CTTTAACCAACGCGTCCTCCCAGTTAT	23	<lmxll>	ll	107	lm	151		
>ref-138096	CTTTAACCAACGCGTCCTCCCAGTTAT	27	<lmxll>	ll	234	lm	31		
>ref-138174	CACACGCCCACCTCCACTCCCACACTC	1	<lmxll>	ll	121	lm	149		
>ref-138174	CACACGCCCACCTCCACTCCCACACTC	9	<lmxll>	ll	121	lm	149		
>ref-138181	TCCCTGAGCACAGTCACTCCTGCCCTG	23	<hkxhk>	hh	48	hk	156	kk	65
>ref-138188	ACTGCTCATACATGGACTCCCTGGCAT	9	<nnxnp>	nn	272	np	0		

>ref-138203	GCCCTTAGCACCTCTTCTCCCCTTTAA	21	<nnxnp>	nn	138	np	122		
>ref-138224	CCTCCTGCCACCCCGTCTCCCCCTCTC	5	<lmxll>	ll	125	lm	129		
>ref-138230	GGCAACATTACATATCCTCCTATAGAA	25	<nnxnp>	nn	241	np	0		
>ref-138236	GAAGATCGCACCCCAGCTCCTGTTTGT	13	<lmxll>	ll	121	lm	121		
>ref-138243	ACCTCCTCTACCCCCCCTCCCCCGTCT	24	<nnxnp>	nn	6	np	234		
>ref-138243	ACCTCCTCTACCCCCCCTCCCCCGTCT	25	<hkxhk>	hh	8	hk	233	kk	8
>ref-138255	CTCCCTCACACCTGCTCTCCCTTCACT	12	<nnxnp>	nn	265	np	1		
>ref-138285	ACTTTCGGGACTCCAGCTCCTCAGGGT	5	<nnxnp>	nn	104	np	144		
>ref-138292	ATCTACCTGACTGCTACTCCCTCCCCT	12	<nnxnp>	nn	116	np	111		
>ref-138296	CCGCCATTGACAATTCCTCCCCTGACC	12	<lmxll>	ll	208	lm	57		
>ref-138296	CCGCCATTGACAATTCCTCCCCTGACC	13	<lmxll>	ll	170	lm	89		
>ref-138296	CCGCCATTGACAATTCCTCCCCTGACC	27	<hkxhk>	hh	135	hk	112	kk	0
>ref-138298	CTCCCCTCCACCCCATCTCCATACATT	7	<nnxnp>	nn	255	np	0		
>ref-138298	CTCCCCTCCACCCCATCTCCATACATT	23	<nnxnp>	nn	255	np	0		
>ref-138311	CTCCATTCCACCATAACTCCAACTCTC	12	<hkxhk>	hh	2	hk	268	kk	0
>ref-138311	CTCCATTCCACCATAACTCCAACTCTC	24	<hkxhk>	hh	0	hk	268	kk	2
>ref-138312	TGTAAGTACACACATTCTCCTTTCTTA	22	<hkxhk>	hh	72	hk	135	kk	39
>ref-138328	CAATCCCCTACCTACACTCCTCTCCCT	2	<nnxnp>	nn	122	np	132		
>ref-138350	ATCTTTTACACTCATACTCCCTCACGT	5	<nnxnp>	nn	112	np	125		
>ref-138350	ATCTTTTACACTCATACTCCCTCACGT	15	<nnxnp>	nn	108	np	129		
>ref-138357	ACTTGCAAGACATCTCCTCCAGGACTT	26	<lmxll>	ll	113	lm	138		
>ref-138377	AAGGGGGTCACGTTCACTCCTCGTGAG	21	<lmxll>	ll	10	lm	256		
>ref-138397	TTATTTTTCACTCTCCCTCCCCTTCTC	22	<hkxhk>	hh	25	hk	223	kk	0
>ref-138397	TTATTTTTCACTCTCCCTCCCCTTCTC	27	<hkxhk>	hh	11	hk	248	kk	1
>ref-138404	TCCCCACAGACATTTGCTCCTTCTGGA	5	<nnxnp>	nn	265	np	0		
>ref-138422	CTGAAGCATACTCCACCTCCACGCAAC	9	<nnxnp>	nn	248	np	1		
>ref-138423	AACGGCGACACAGAGGCTCCAGCACTC	5	<nnxnp>	nn	142	np	126		
>ref-138439	TGTACTGAAACTAAATCTCCAATGCAC	25	<hkxhk>	hh	5	hk	253	kk	3
>ref-138439	TGTACTGAAACTAAATCTCCAATGCAC	27	<hkxhk>	hh	3	hk	253	kk	4
>ref-138497	CACTCACTCACACCCCCTCCCCAGCCC	1	<nnxnp>	nn	268	np	1		
>ref-138500	TTCACCATTACAACTTCTCCCGCTTCC	14	<lmxll>	ll	135	lm	121		
>ref-138500	TTCACCATTACAACTTCTCCCGCTTCC	22	<lmxll>	ll	133	lm	121		
>ref-138501	TCCTCCTTCACCTACTCTCCATCATCA	15	<hkxhk>	hh	71	hk	133	kk	61
>ref-138509	CCCTATTTAACACATCCTCCTCCTCCC	2	<lmxll>	ll	134	lm	88		
>ref-138555	TTTGTTTAAACCCTCCCTCCCCCCTCT	13	<lmxll>	ll	234	lm	0		
>ref-138555	TTTGTTTAAACCCTCCCTCCCCCCTCT	16	<lmxll>	ll	229	lm	0		
>ref-138566	CTCTCATCAACAGTGTCTCCCTCAACA	15	<hkxhk>	hh	7	hk	263	kk	0
>ref-138566	CTCTCATCAACAGTGTCTCCCTCAACA	25	<lmxll>	ll	177	lm	83		

>ref-138566	CTCTCATCAACAGTGTCTCCCTCAACA	27	<nnxnp>	nn	186	np	75		
>ref-138586	CTCACACCTACACACACTCCGACACTC	5	<nnxnp>	nn	118	np	147		
>ref-138589	ACCCTGCCTACCTGACCTCCCTATCCT	23	<hkxhk>	hh	56	hk	142	kk	65
>ref-138628	CCCTCCATCACCCTGTCTCCTTCCTCA	3	<hkxhk>	hh	0	hk	59	kk	198
>ref-138640	CTAAACGAAACACACACTCCCACACTC	26	<lmxll>	ll	143	lm	110		
>ref-138662	ACGGACGTAACGCTCTCTCCATGAACG	26	<lmxll>	ll	121	lm	141		
>ref-138680	CGTGTTTTCACCGCGTCTCCGAAAGTG	4	<nnxnp>	nn	263	np	0		
>ref-138680	CGTGTTTTCACCGCGTCTCCGAAAGTG	8	<nnxnp>	nn	263	np	0		
>ref-138681	ACCACCACCACTACCTCTCCTCTTCCT	13	<hkxhk>	hh	239	hk	21	kk	0
>ref-138681	ACCACCACCACTACCTCTCCTCTTCCT	21	<hkxhk>	hh	0	hk	20	kk	239
>ref-138682	CCTGCATGCACGGTGCCTCCGCTATGA	1	<lmxll>	ll	237	lm	1		
>ref-138715	ACCCGTCTAACTTCACCTCCTAACTTC	21	<hkxhk>	hh	51	hk	150	kk	48
>ref-138732	AACAAACAAACAAACTCTCCCCCTCTC	22	<hkxhk>	hh	0	hk	266	kk	3
>ref-138740	GCTGTGCGTACGCAAGCTCCATTCATT	5	<lmxll>	ll	142	lm	129		
>ref-138742	CTCTCTAAAACTCCCTCTCCCTGTATA	21	<nnxnp>	nn	139	np	95		
>ref-138743	TTCTCCTATACCTTGCCTCCCTCTTCT	2	<lmxll>	ll	141	lm	124		
>ref-138772	GTGTACCTTACTGTTCCTCCTTCTTTG	8	<nnxnp>	nn	131	np	128		
>ref-138775	ATCTCCTCCACCCTTCCTCCTCAACCT	9	<nnxnp>	nn	133	np	134		
>ref-138801	GTCATGTGAACCATATCTCCTGCGGTA	14	<nnxnp>	nn	115	np	123		
>ref-138802	CTCCCTCCAACATTTCCTCCCTTTCCC	12	<nnxnp>	nn	264	np	0		
>ref-138809	GGACTTTGCACATCGCCTCCGCTCAAC	14	<lmxll>	ll	134	lm	125		
>ref-138819	GGTTTCAAAACAAACACTCCATCAGGT	22	<hkxhk>	hh	0	hk	270	kk	0
>ref-138819	GGTTTCAAAACAAACACTCCATCAGGT	25	<hkxhk>	hh	0	hk	270	kk	0
>ref-138856	TTGCCTATTACGCGTTCTCCCTCCTTA	23	<lmxll>	ll	121	lm	142		
>ref-138865	CGGAATTCCACGGCAACTCCTCAGCCA	22	<lmxll>	ll	121	lm	146		
>ref-138876	TACATCCACACACCCACTCCTATACTC	26	<lmxll>	ll	121	lm	143		
>ref-138890	GCCCCCTCCACCTATTCTCCCTTCTGT	3	<nnxnp>	nn	126	np	140		
>ref-138898	CACATGTGTACACACACTCCCACACAC	8	<nnxnp>	nn	11	np	257		
>ref-138898	CACATGTGTACACACACTCCCACACAC	24	<nnxnp>	nn	12	np	258		
>ref-138912	TTTCCATTAACCACCACTCCCTTTCTC	8	<lmxll>	ll	130	lm	132		
>ref-138926	ACTCAAAACACCGACACTCCGCTAATA	3	<lmxll>	ll	119	lm	136		
>ref-138926	ACTCAAAACACCGACACTCCGCTAATA	22	<lmxll>	ll	117	lm	129		
>ref-138948	CCACCCACCACCACCTCTCCCTCACCT	4	<hkxhk>	hh	0	hk	257	kk	4
>ref-138948	CCACCCACCACCACCTCTCCCTCACCT	13	<lmxll>	ll	14	lm	240		
>ref-138948	CCACCCACCACCACCTCTCCCTCACCT	27	<lmxll>	ll	15	lm	240		
>ref-138955	CATACCTTCACACACACTCCTACATGC	1	<nnxnp>	nn	271	np	0		
>ref-138956	CGTCACACCACTGTCACTCCGGACTTC	6	<lmxll>	ll	114	lm	153		
>ref-138956	CGTCACACCACTGTCACTCCGGACTTC	21	<nnxnp>	nn	139	np	121		

>ref-138961	AACACTTTCACTATCACTCCTTTTAAT	3	<nnxnp>	nn	121	np	105		
>ref-139002	AACTCTCTCACTTCCACTCCTGTTAGA	23	<nnxnp>	nn	105	np	141		
>ref-139027	GACTCATAAACCAACACTCCATGTACG	1	<nnxnp>	nn	150	np	117		
>ref-139027	GACTCATAAACCAACACTCCATGTACG	3	<nnxnp>	nn	150	np	117		
>ref-139038	ACTGTGTACACAGGCCCTCCTCGTGGA	1	<nnxnp>	nn	134	np	133		
>ref-139083	CCAAACATGACCCAACCTCCTACAGCC	3	<hkxhk>	hh	48	hk	147	kk	61
>ref-139093	TGTGCATCTACACCTCCTCCTTCTCCT	25	<nnxnp>	nn	143	np	116		
>ref-139095	CCATTCTCAACCCTTTCTCCTTTTCTT	3	<lmxll>	ll	0	lm	265		
>ref-139095	CCATTCTCAACCCTTTCTCCTTTTCTT	9	<lmxll>	ll	0	lm	265		
>ref-139095	CCATTCTCAACCCTTTCTCCTTTTCTT	21	<nnxnp>	nn	154	np	106		
>ref-139116	GGTTAGAGCACTTGGACTCCGACCCTC	12	<lmxll>	ll	217	lm	41		
>ref-139116	GGTTAGAGCACTTGGACTCCGACCCTC	13	<hkxhk>	hh	0	hk	273	kk	0
>ref-139116	GGTTAGAGCACTTGGACTCCGACCCTC	15	<lmxll>	ll	220	lm	39		
>ref-139190	CACAGACTTACAGCCCCTCCCTCTTTG	16	<lmxll>	ll	114	lm	138		
>ref-139195	TTGAAGACAACCAAATCTCCATCGTTA	12	<lmxll>	ll	128	lm	131		
>ref-139199	GCGACGTTAACTGGTGCTCCATGTGAA	12	<nnxnp>	nn	273	np	0		
>ref-139201	CGCCGTAACACCCCCTCTCCCGCATTC	26	<nnxnp>	nn	124	np	135		
>ref-139207	CCACGGAAGACCCTCCCTCCGCTCGCA	5	<nnxnp>	nn	122	np	136		
>ref-139223	TCATACTCCACTTCCCCTCCGCTTCCT	2	<lmxll>	ll	131	lm	135		
>ref-139223	TCATACTCCACTTCCCCTCCGCTTCCT	9	<lmxll>	ll	131	lm	135		
>ref-139233	TTGCCTTCTACTTCCCCTCCTCTCCTC	12	<nnxnp>	nn	266	np	0		
>ref-139233	TTGCCTTCTACTTCCCCTCCTCTCCTC	16	<nnxnp>	nn	257	np	2		
>ref-139289	AACACGCTAACACCCCCTCCTCCACAC	13	<nnxnp>	nn	135	np	133		
>ref-139295	ACCACCGCCACGTTAGCTCCGTCTGTC	24	<nnxnp>	nn	141	np	132		
>ref-139298	TTCTCCCTTACTTTATCTCCCCCCTTC	12	<lmxll>	ll	104	lm	141		
>ref-139380	TTTAGAGTGACGTCATCTCCTTCAGAA	15	<lmxll>	ll	123	lm	133		
>ref-139397	GTTATCATCACCTTTCCTCCCCCTTCT	27	<lmxll>	ll	130	lm	128		
>ref-139409	CACCATCCAACACAATCTCCCACACAT	13	<lmxll>	ll	147	lm	115		
>ref-139429	TTCCCTCCTACCCCCTCTCCCGGTCTG	27	<hkxhk>	hh	43	hk	147	kk	73
>ref-139440	CTGGTCATAACATTCCCTCCCTCATTC	4	<hkxhk>	hh	73	hk	140	kk	50
>ref-139475	TTCTGTCCCACCGTTCCTCCTTCCCCT	5	<nnxnp>	nn	133	np	131		
>ref-139480	TGACACTCTACTAATGCTCCACAGGAA	16	<nnxnp>	nn	138	np	131		
>ref-139482	ACACATCAGACCTTTGCTCCTTCTGGT	3	<lmxll>	ll	1	lm	259		
>ref-139489	AATAATACCACCTGCCCTCCCACCTGT	7	<nnxnp>	nn	104	np	131		
>ref-139521	TTTACCAACACCTTCTCTCCTGAACTC	22	<lmxll>	ll	122	lm	131		
>ref-139532	CCTCTTGCTACTTCCCCTCCACCTCCA	1	<lmxll>	ll	130	lm	126		
>ref-139533	TTTTATACCACCTCCTCTCCCTTTCCT	2	<nnxnp>	nn	227	np	17		
>ref-139533	TTTTATACCACCTCCTCTCCCTTTCCT	12	<hkxhk>	hh	0	hk	249	kk	16

>ref-139533	TTTTATACCACCTCCTCTCCCTTTCCT	25	<nnxnp>	nn	260	np	10		
>ref-139535	TTGTACCATACCTTCCCTCCCTTACCT	8	<hkxhk>	hh	54	hk	135	kk	68
>ref-139535	TTGTACCATACCTTCCCTCCCTTACCT	27	<hkxhk>	hh	68	hk	135	kk	54
>ref-139583	TCTTTTCTTACTCCCACTCCTATCATC	4	<hkxhk>	hh	51	hk	139	kk	57
>ref-139596	CACTTATGTACCCCCTCTCCCCCACTT	1	<lmxll>	ll	129	lm	121		
>ref-139599	TGTACACACACACCCACTCCTGTACAC	3	<hkxhk>	hh	1	hk	266	kk	1
>ref-139599	TGTACACACACACCCACTCCTGTACAC	7	<hkxhk>	hh	1	hk	266	kk	1
>ref-139602	CTCCCCCCCACTCCTTCTCCATCTTTT	4	<hkxhk>	hh	0	hk	216	kk	19
>ref-139602	CTCCCCCCCACTCCTTCTCCATCTTTT	9	<hkxhk>	hh	17	hk	218	kk	0
>ref-139604	CTATTGACTACATCTTCTCCTTTTTTT	3	<nnxnp>	nn	118	np	124		
>ref-139611	GTTGTACAAACTCTAGCTCCAAGCATA	6	<hkxhk>	hh	1	hk	269	kk	0
>ref-139611	GTTGTACAAACTCTAGCTCCAAGCATA	14	<lmxll>	ll	136	lm	111		
>ref-139611	GTTGTACAAACTCTAGCTCCAAGCATA	23	<lmxll>	ll	136	lm	111		
>ref-139616	CAACTAACAACCTCCTCTCCACGCATA	22	<lmxll>	ll	146	lm	125		
>ref-139681	CACACAAGCACGTTTTCTCCTCCCATA	27	<nnxnp>	nn	108	np	128		
>ref-139688	TTACCATTGACGGACTCTCCTGATAAG	9	<nnxnp>	nn	237	np	3		
>ref-139689	AGGGGATGAACTACCTCTCCCATCATA	2	<lmxll>	ll	117	lm	122		
>ref-139702	ATCGGTTGGACACATGCTCCTGCCAAT	3	<hkxhk>	hh	6	hk	266	kk	0
>ref-139702	ATCGGTTGGACACATGCTCCTGCCAAT	14	<hkxhk>	hh	10	hk	261	kk	0
>ref-139702	ATCGGTTGGACACATGCTCCTGCCAAT	21	<nnxnp>	nn	227	np	33		
>ref-139702	ATCGGTTGGACACATGCTCCTGCCAAT	23	<nnxnp>	nn	237	np	28		
>ref-139702	ATCGGTTGGACACATGCTCCTGCCAAT	25	<lmxll>	ll	94	lm	165		
>ref-139702	ATCGGTTGGACACATGCTCCTGCCAAT	27	<hkxhk>	hh	0	hk	272	kk	1
>ref-139711	CACTCTCTTACCCACCCTCCTCCTTGC	14	<nnxnp>	nn	236	np	0		
>ref-139749	CCTACGCCCACGCCTTCTCCTTTTCTT	6	<nnxnp>	nn	20	np	251		
>ref-139749	CCTACGCCCACGCCTTCTCCTTTTCTT	21	<nnxnp>	nn	20	np	251		
>ref-139776	TTCCCTTCCACACCTTCTCCTCCCTCC	2	<lmxll>	ll	8	lm	259		
>ref-139776	TTCCCTTCCACACCTTCTCCTCCCTCC	12	<lmxll>	ll	7	lm	258		
>ref-139784	CCCCTTGGCACCATTTCTCCCTCTCCC	2	<hkxhk>	hh	0	hk	269	kk	1
>ref-139825	GGACCAGGAACGACGCCTCCCGGACCA	4	<lmxll>	ll	114	lm	148		
>ref-139832	GGGGCAGCGACCGCGCCTCCTCCCTCC	4	<lmxll>	ll	129	lm	142		
>ref-139857	TCTCTCTCCACCCTTCCTCCTTTATAT	9	<lmxll>	ll	82	lm	155		
>ref-139857	TCTCTCTCCACCCTTCCTCCTTTATAT	23	<lmxll>	ll	134	lm	136		
>ref-139857	TCTCTCTCCACCCTTCCTCCTTTATAT	24	<lmxll>	ll	134	lm	136		
>ref-139857	TCTCTCTCCACCCTTCCTCCTTTATAT	26	<lmxll>	ll	82	lm	153		
>ref-139879	ACGCATCTCACGCCCACTCCTTCACCA	21	<hkxhk>	hh	60	hk	144	kk	64
>ref-139888	TCTCCCGTTACATTCTCTCCCTCTTTA	7	<nnxnp>	nn	118	np	144		
>ref-139888	TCTCCCGTTACATTCTCTCCCTCTTTA	25	<nnxnp>	nn	117	np	144		

>ref-139906	TCCTATATTACCCTGCCTCCTGCATCC	7	<hkxhk>	hh	0	hk	272	kk	0
>ref-139906	TCCTATATTACCCTGCCTCCTGCATCC	12	<hkxhk>	hh	99	hk	155	kk	0
>ref-139908	CCGGATCAAACGCCAACTCCGCCGCAG	3	<lmxll>	ll	121	lm	114		
>ref-139926	ATGCAACAAACTTTACCTCCTCGCTTT	25	<lmxll>	ll	103	lm	154		
>ref-139935	CTGGCCACAACCCCCCCTCCCCTTCTC	9	<lmxll>	ll	136	lm	99		
>ref-139961	TTCTTATCCACCTCTCCTCCCTTCCTT	27	<hkxhk>	hh	3	hk	241	kk	11
>ref-139979	GGAATTAACACCCTTCCTCCTTCCCTC	3	<lmxll>	ll	129	lm	136		
>ref-139979	GGAATTAACACCCTTCCTCCTTCCCTC	21	<lmxll>	ll	120	lm	149		
>ref-139979	GGAATTAACACCCTTCCTCCTTCCCTC	22	<lmxll>	ll	128	lm	136		
>ref-139987	CACACACACACAGATTCTCCCATGACA	12	<lmxll>	ll	134	lm	128		
>ref-139988	GACGTGTAAACAAGATCTCCCGGCCAC	24	<lmxll>	ll	117	lm	132		
>ref-140010	ATCCCCATCACTCCCCCTCCCTCCCCA	4	<nnxnp>	nn	251	np	0		
>ref-140022	CGGTTCAATACTTTACCTCCCACTCTT	5	<hkxhk>	hh	54	hk	131	kk	48
>ref-140061	GATGAATACACTCCCTCTCCCTGTGTT	2	<hkxhk>	hh	52	hk	153	kk	60
>ref-140081	TCTACGACCACGTCTACTCCAGCCTCT	15	<hkxhk>	hh	0	hk	90	kk	159
>ref-140081	TCTACGACCACGTCTACTCCAGCCTCT	16	<hkxhk>	hh	13	hk	255	kk	0
>ref-140081	TCTACGACCACGTCTACTCCAGCCTCT	24	<hkxhk>	hh	14	hk	255	kk	0
>ref-140081	TCTACGACCACGTCTACTCCAGCCTCT	25	<hkxhk>	hh	0	hk	273	kk	0
>ref-140089	TTCTCTTTCACCTTATCTCCTTCTTCC	2	<lmxll>	ll	102	lm	118		
>ref-140096	TTCGTTTCTACTAGTTCTCCTTCGATT	1	<lmxll>	ll	108	lm	138		
>ref-140096	TTCGTTTCTACTAGTTCTCCTTCGATT	2	<lmxll>	ll	108	lm	138		
>ref-140139	GAAAAGGGGACCTCTCCTCCTTCAGCA	6	<nnxnp>	nn	128	np	139		
>ref-140146	ACTCCTTTTACCCTCCCTCCCTCACTC	7	<lmxll>	ll	48	lm	190		
>ref-140158	ACAACCACAACAAAAGCTCCTCCAGTT	26	<nnxnp>	nn	270	np	0		
>ref-140160	CACCCACTCACCCATCCTCCCATCCCT	4	<nnxnp>	nn	7	np	264		
>ref-140160	CACCCACTCACCCATCCTCCCATCCCT	9	<hkxhk>	hh	3	hk	259	kk	4
>ref-140175	CTCTTTTGCACTCCCTCTCCCTCAGGC	25	<nnxnp>	nn	91	np	140		
>ref-140183	AGCTCCCCTACCTACCCTCCCCCCTCA	1	<nnxnp>	nn	125	np	143		
>ref-140183	AGCTCCCCTACCTACCCTCCCCCCTCA	2	<nnxnp>	nn	126	np	142		
>ref-140196	CTCACTCTTACCTTCACTCCCTTGCCT	7	<nnxnp>	nn	116	np	142		
>ref-140198	CCCTAACCCACTCGCGCTCCATCACTC	3	<nnxnp>	nn	115	np	150		
>ref-140198	CCCTAACCCACTCGCGCTCCATCACTC	4	<nnxnp>	nn	115	np	150		
>ref-140208	ACAGCTCGTACGAGAGCTCCTACAACT	8	<hkxhk>	hh	69	hk	150	kk	46
>ref-140209	TCATTGTTCACTAATGCTCCCGTTCAT	22	<hkxhk>	hh	0	hk	271	kk	0
>ref-140215	CGAGTCCCTACCCCCTCTCCTTCTTCT	21	<lmxll>	ll	118	lm	132		
>ref-140221	ATTCTCCTCACTATCCCTCCTCTCTTA	5	<nnxnp>	nn	5	np	255		
>ref-140221	ATTCTCCTCACTATCCCTCCTCTCTTA	25	<lmxll>	ll	263	lm	0		
>ref-140221	ATTCTCCTCACTATCCCTCCTCTCTTA	26	<lmxll>	ll	262	lm	0		

>ref-140221	ATTCTCCTCACTATCCCTCCTCTCTTA	27	<hkxhk>	hh	7	hk	243	kk	3
>ref-140244	TGTTTACAAACTGGAGCTCCAAGGCCA	26	<hkxhk>	hh	66	hk	129	kk	52
>ref-140260	ACTACAACAACAACTGCTCCTCCAACA	3	<nnxnp>	nn	140	np	133		
>ref-140260	ACTACAACAACAACTGCTCCTCCAACA	12	<nnxnp>	nn	140	np	133		
>ref-140260	ACTACAACAACAACTGCTCCTCCAACA	13	<nnxnp>	nn	140	np	133		
>ref-140260	ACTACAACAACAACTGCTCCTCCAACA	21	<nnxnp>	nn	140	np	133		
>ref-140260	ACTACAACAACAACTGCTCCTCCAACA	22	<nnxnp>	nn	140	np	133		
>ref-140260	ACTACAACAACAACTGCTCCTCCAACA	24	<nnxnp>	nn	188	np	74		
>ref-140284	AAACATATAACCGCACCTCCCCGCTAA	6	<lmxll>	ll	142	lm	120		
>ref-140292	CCATCTCTCACTCCCTCTCCCATGCAC	15	<nnxnp>	nn	106	np	122		
>ref-140292	CCATCTCTCACTCCCTCTCCCATGCAC	21	<nnxnp>	nn	108	np	122		
>ref-140300	TTGTTTCATACTATCTCTCCTCCCTAC	6	<lmxll>	ll	119	lm	122		
>ref-140304	CCACCCCTCACCTTTTCTCCTTTCCTT	4	<nnxnp>	nn	233	np	0		
>ref-140304	CCACCCCTCACCTTTTCTCCTTTCCTT	25	<nnxnp>	nn	117	np	103		
>ref-140317	TCTGCTCTAACACTCTCTCCCTCCCTC	2	<lmxll>	ll	265	lm	0		
>ref-140329	CCCACTTCCACTCTCCCTCCTCCTCCA	27	<lmxll>	ll	81	lm	162		
>ref-140350	TTCATTTGCACATCTGCTCCTTCGCAG	3	<hkxhk>	hh	48	hk	119	kk	54
>ref-140364	ACTTAAATGACCCATCCTCCGTAGTCA	16	<nnxnp>	nn	135	np	126		
>ref-140365	CGTAGCAGCACGAGGTCTCCTTCCGCT	27	<hkxhk>	hh	0	hk	239	kk	31
>ref-140413	TGAAATGACACGTTCCCTCCTCCCACA	2	<lmxll>	ll	136	lm	127		
>ref-140443	CTATTTCGTACTCCGTCTCCATCCCTT	15	<hkxhk>	hh	53	hk	141	kk	51
>ref-140449	CCCGCCTTCACCTCCCCTCCCCTTCCT	3	<hkxhk>	hh	0	hk	272	kk	0
>ref-140450	CAGACTCACACCGTCCCTCCCCAACAG	12	<nnxnp>	nn	118	np	132		
>ref-140474	ATTACCTATACACCTCCTCCTCACCCC	5	<hkxhk>	hh	65	hk	138	kk	58
>ref-140492	ACACCTCCCACCGTCCCTCCTCCTCCC	3	<lmxll>	ll	116	lm	125		
>ref-140492	ACACCTCCCACCGTCCCTCCTCCTCCC	24	<lmxll>	ll	123	lm	123		
>ref-140503	CTTACCCACACCTTTCCTCCCTTCTTC	13	<hkxhk>	hh	41	hk	163	kk	54
>ref-140514	ATAGTGATAACGCTCCCTCCCTCCCTC	16	<lmxll>	ll	258	lm	0		
>ref-140524	CACGCCCTCACCCTTTCTCCTTCATCC	25	<nnxnp>	nn	129	np	132		
>ref-140526	TCCATGTCCACTTCCGCTCCAGTCTTT	3	<lmxll>	ll	154	lm	105		
>ref-140526	TCCATGTCCACTTCCGCTCCAGTCTTT	16	<hkxhk>	hh	0	hk	128	kk	136
>ref-140526	TCCATGTCCACTTCCGCTCCAGTCTTT	27	<lmxll>	ll	92	lm	134		
>ref-140541	TCTAGTTGTACAGACCCTCCGCAGCAC	2	<hkxhk>	hh	65	hk	203	kk	1
>ref-140541	TCTAGTTGTACAGACCCTCCGCAGCAC	13	<hkxhk>	hh	3	hk	191	kk	76
>ref-140541	TCTAGTTGTACAGACCCTCCGCAGCAC	15	<lmxll>	ll	23	lm	243		
>ref-140541	TCTAGTTGTACAGACCCTCCGCAGCAC	22	<lmxll>	ll	16	lm	231		
>ref-140548	ACAGATAACACCTTTCCTCCCTGTCCC	15	<lmxll>	ll	121	lm	147		
>ref-140570	CACTCGCTCACTCACACTCCGACTCAC	3	<lmxll>	ll	3	lm	270		

>ref-140570	CACTCGCTCACTCACACTCCGACTCAC	4	<lmxll>	ll	3	lm	270		
>ref-140587	AAGCCATTCACAAGAGCTCCTCGGAGA	24	<nnxnp>	nn	121	np	131		
>ref-140602	GTGTCTCACACTCTCGCTCCCTCCTTC	7	<lmxll>	ll	144	lm	129		
>ref-140603	ACACATCTTACTACGCCTCCCACGGAA	12	<hkxhk>	hh	93	hk	131	kk	36
>ref-140605	CTTCTCTCCACTTTCTCTCCTTACCTC	5	<lmxll>	ll	156	lm	107		
>ref-140608	ATCTTATCAACCTTCTCTCCCCGCCCT	9	<hkxhk>	hh	60	hk	135	kk	48
>ref-140611	ATTCTCTTTACCCTTCCTCCCTTCCCC	4	<nnxnp>	nn	140	np	123		
>ref-140630	ACCTTACATACTAAAGCTCCCGATGTG	27	<nnxnp>	nn	89	np	140		
>ref-140636	TTTATCTCTACCTCTTCTCCTCTCTTC	9	<nnxnp>	nn	226	np	0		
>ref-140645	TCTGACCCCACCCCTTCTCCTTCCTCC	5	<lmxll>	ll	250	lm	3		
>ref-140645	TCTGACCCCACCCCTTCTCCTTCCTCC	7	<lmxll>	ll	248	lm	7		
>ref-140653	ACGCGACGGACCCTTCCTCCCGCCCCG	9	<lmxll>	ll	265	lm	0		
>ref-140669	CTTCTATGCACGGCAACTCCATCTTAG	7	<nnxnp>	nn	117	np	127		
>ref-140679	AAGAGAATTACTTCTCCTCCTTGTCCT	16	<nnxnp>	nn	127	np	141		
>ref-140681	CTTGCGTCTACCATTTCTCCGTATATT	12	<lmxll>	ll	109	lm	148		
>ref-140703	CTTCCATATACGGGGTCTCCGCGGACT	3	<lmxll>	ll	151	lm	116		
>ref-140703	CTTCCATATACGGGGTCTCCGCGGACT	23	<hkxhk>	hh	0	hk	262	kk	6
>ref-140719	CTTGAGGGAACAATCTCTCCGTCTATC	6	<nnxnp>	nn	137	np	122		
>ref-140739	GCCACGCGGACTTAGTCTCCCAATAAA	3	<hkxhk>	hh	32	hk	129	kk	75
>ref-140741	CCTACCTTAACCCCCCCTCCCTTATCC	5	<lmxll>	ll	34	lm	205		
>ref-140741	CCTACCTTAACCCCCCCTCCCTTATCC	9	<nnxnp>	nn	178	np	70		
>ref-140741	CCTACCTTAACCCCCCCTCCCTTATCC	22	<nnxnp>	nn	178	np	69		
>ref-140741	CCTACCTTAACCCCCCCTCCCTTATCC	24	<lmxll>	ll	34	lm	205		
>ref-140759	AGCTTAGATACGGCCTCTCCTGAAAAC	26	<nnxnp>	nn	241	np	0		
>ref-140760	GGTTAATATACAGGAACTCCCTCACTC	9	<nnxnp>	nn	109	np	139		
>ref-140796	CTCTCCTCCACCCTGTCTCCTCACCTC	25	<nnxnp>	nn	121	np	137		
>ref-140800	GCGATGCACACTCCGACTCCGCCTCCC	1	<hkxhk>	hh	233	hk	10	kk	0
>ref-140800	GCGATGCACACTCCGACTCCGCCTCCC	3	<hkxhk>	hh	233	hk	2	kk	0
>ref-140800	GCGATGCACACTCCGACTCCGCCTCCC	16	<lmxll>	ll	109	lm	150		
>ref-140801	TCGACTCTTACTGGGTCTCCGTGGGAT	2	<hkxhk>	hh	0	hk	267	kk	1
>ref-140801	TCGACTCTTACTGGGTCTCCGTGGGAT	5	<hkxhk>	hh	1	hk	267	kk	0
>ref-140827	CTCCGTATTACCCACTCTCCTTGTCTT	27	<hkxhk>	hh	69	hk	131	kk	65
>ref-140832	TCCTCATAAACAAGGACTCCTTACCAG	22	<hkxhk>	hh	62	hk	141	kk	59
>ref-140841	GATTTCATCACTTTTCCTCCTGTCATC	1	<hkxhk>	hh	0	hk	170	kk	76
>ref-140841	GATTTCATCACTTTTCCTCCTGTCATC	25	<hkxhk>	hh	73	hk	173	kk	0
>ref-140848	TACTCAGTCACTCCATCTCCTAATCGG	8	<lmxll>	ll	114	lm	127		
>ref-140850	ATAGAACAAACATTTTCTCCTTTTCCT	16	<lmxll>	ll	100	lm	123		
>ref-140856	TTCCCTTTTACTACCCCTCCCTCTCCC	14	<nnxnp>	nn	229	np	3		

>ref-140885	AGTGATTTTACTCTGCCTCCTCGGTGA	1	<hkxhk>	hh	24	hk	240	kk	0
>ref-140885	AGTGATTTTACTCTGCCTCCTCGGTGA	7	<hkxhk>	hh	0	hk	241	kk	24
>ref-140903	CCTTACTTCACTCTCCCTCCCTCTCAC	4	<nnxnp>	nn	1	np	254		
>ref-140903	CCTTACTTCACTCTCCCTCCCTCTCAC	9	<nnxnp>	nn	35	np	218		
>ref-140903	CCTTACTTCACTCTCCCTCCCTCTCAC	23	<nnxnp>	nn	35	np	218		
>ref-140913	ATGATATTTACTTGATCTCCCTCCTCC	5	<nnxnp>	nn	5	np	267		
>ref-140913	ATGATATTTACTTGATCTCCCTCCTCC	21	<nnxnp>	nn	5	np	267		
>ref-140922	TGATCGTCAACCCATACTCCGTGCGAG	13	<nnxnp>	nn	209	np	55		
>ref-140969	CTGTACTTAACTCAACCTCCCTGTCCT	14	<nnxnp>	nn	135	np	133		
>ref-140969	CTGTACTTAACTCAACCTCCCTGTCCT	23	<nnxnp>	nn	136	np	133		
>ref-140980	CTAACACACACGAACTCTCCCTCGCAA	27	<nnxnp>	nn	119	np	143		
>ref-140987	TACTTCAGAACAAAAACTCCAATCTAC	14	<lmxll>	ll	23	lm	233		
>ref-140987	TACTTCAGAACAAAAACTCCAATCTAC	22	<hkxhk>	hh	174	hk	71	kk	0
>ref-141008	TGCGCATCGACCGTCCCTCCGTGTTCC	1	<hkxhk>	hh	56	hk	152	kk	62
>ref-141020	TCTTTCCTAACTGTACCTCCTCCTCTA	14	<nnxnp>	nn	134	np	137		
>ref-141020	TCTTTCCTAACTGTACCTCCTCCTCTA	16	<nnxnp>	nn	130	np	135		
>ref-141025	GGTGAAAAAACGATGCCTCCCCGGCGG	8	<lmxll>	ll	188	lm	75		
>ref-141025	GGTGAAAAAACGATGCCTCCCCGGCGG	12	<hkxhk>	hh	0	hk	257	kk	10
>ref-141025	GGTGAAAAAACGATGCCTCCCCGGCGG	14	<lmxll>	ll	55	lm	208		
>ref-141025	GGTGAAAAAACGATGCCTCCCCGGCGG	16	<hkxhk>	hh	69	hk	178	kk	0
>ref-141026	CTCCAAGCCACTCAGTCTCCAAGCCAC	4	<hkxhk>	hh	4	hk	267	kk	0
>ref-141026	CTCCAAGCCACTCAGTCTCCAAGCCAC	9	<hkxhk>	hh	0	hk	273	kk	0
>ref-141026	CTCCAAGCCACTCAGTCTCCAAGCCAC	25	<hkxhk>	hh	0	hk	273	kk	0
>ref-141026	CTCCAAGCCACTCAGTCTCCAAGCCAC	26	<hkxhk>	hh	0	hk	273	kk	0
>ref-141040	TACGACTCTACTCTCTCTCCTCTTTTA	6	<lmxll>	ll	268	lm	0		
>ref-141047	TCCGCTCGAACGCTGTCTCCGCGATCA	14	<nnxnp>	nn	153	np	112		
>ref-141056	CTACAACCAACGTCCTCTCCCTATCTC	25	<lmxll>	ll	143	lm	120		
>ref-141056	CTACAACCAACGTCCTCTCCCTATCTC	27	<lmxll>	ll	141	lm	120		
>ref-141100	CTGCTGACCACTTCTTCTCCAGGAAGC	27	<hkxhk>	hh	54	hk	192	kk	0
>ref-141102	TTCTCTCCAACCCTCACTCCCCCCTTT	25	<lmxll>	ll	133	lm	131		
>ref-141102	TTCTCTCCAACCCTCACTCCCCCCTTT	27	<lmxll>	ll	134	lm	131		
>ref-141116	GCACTCATTACAGACTCTCCTCGGTTT	6	<nnxnp>	nn	138	np	125		
>ref-141137	CACCATCACACCCGCACTCCTCCCCCT	27	<nnxnp>	nn	153	np	111		
>ref-141156	CTCTTTTTTACTTACTCTCCTTTTCTC	5	<lmxll>	ll	107	lm	125		
>ref-141156	CTCTTTTTTACTTACTCTCCTTTTCTC	14	<lmxll>	ll	101	lm	130		
>ref-141175	TCAGTCACGACAGCTTCTCCAAGGATG	23	<nnxnp>	nn	8	np	262		
>ref-141207	ATCATTGTAACCATTTCTCCTCTTCTT	3	<nnxnp>	nn	125	np	100		
>ref-141209	CATCTTCACACTCATCCTCCTATCTTT	14	<hkxhk>	hh	68	hk	113	kk	41

>ref-141218	TGTCTTCCGACTGCTCCTCCTCCATGT	23	<hkxhk>	hh	0	hk	272	kk	1
>ref-141229	TGACACAACACCACCTCTCCCTCTCAT	23	<hkxhk>	hh	61	hk	197	kk	1
>ref-141229	TGACACAACACCACCTCTCCCTCTCAT	24	<hkxhk>	hh	1	hk	197	kk	58
>ref-141270	CACCCCCCAACATCACCTCCTCCAACA	7	<lmxll>	ll	194	lm	56		
>ref-141270	CACCCCCCAACATCACCTCCTCCAACA	8	<hkxhk>	hh	140	hk	113	kk	0
>ref-141270	CACCCCCCAACATCACCTCCTCCAACA	14	<hkxhk>	hh	138	hk	112	kk	0
>ref-141272	CGAGTGCAAACCTCTTCTCCCTCTCCC	26	<lmxll>	ll	146	lm	119		
>ref-141281	CACCTCCCCACCCGAGCTCCACGCCGA	26	<lmxll>	ll	219	lm	4		
>ref-141284	ACGAATAGGACGGGATCTCCCTATTAA	21	<lmxll>	ll	117	lm	151		
>ref-141284	ACGAATAGGACGGGATCTCCCTATTAA	23	<lmxll>	ll	117	lm	151		
>ref-141302	GGTTCCGAAACGTTTCCTCCGAAGCTT	13	<nnxnp>	nn	135	np	127		
>ref-141303	CATTTCAGGACGCTAACTCCGCCTATG	4	<nnxnp>	nn	100	np	124		
>ref-141303	CATTTCAGGACGCTAACTCCGCCTATG	22	<nnxnp>	nn	100	np	124		
>ref-141327	TGTCTATCCACCCGTTCTCCTTCCTCC	12	<lmxll>	ll	106	lm	165		
>ref-141342	TATTAGTGTACTGGAGCTCCCAGGCTG	22	<lmxll>	ll	117	lm	120		
>ref-141349	ATGTCTCCTACCTTTTCTCCCTCCTTC	1	<lmxll>	ll	119	lm	114		
>ref-141349	ATGTCTCCTACCTTTTCTCCCTCCTTC	3	<hkxhk>	hh	25	hk	231	kk	0
>ref-141349	ATGTCTCCTACCTTTTCTCCCTCCTTC	6	<nnxnp>	nn	86	np	160		
>ref-141366	GTCTCTCCAACCCTCCCTCCAACTCTC	22	<lmxll>	ll	111	lm	137		
>ref-141407	GGCCCCGTCACTTCGACTCCCGTGAAT	5	<hkxhk>	hh	1	hk	271	kk	0
>ref-141407	GGCCCCGTCACTTCGACTCCCGTGAAT	15	<nnxnp>	nn	273	np	0		
>ref-141433	TCTTTCTTTACTCCCTCTCCTATCCTC	23	<nnxnp>	nn	250	np	0		
>ref-141436	ATGTCATTGACTCCCCCTCCCTCTGTC	4	<lmxll>	ll	147	lm	124		
>ref-141447	GCCTCCGTCACTCGAGCTCCCTCCTTC	2	<nnxnp>	nn	128	np	133		
>ref-141456	AAAGTAAAAACTCCCTCTCCCCACACC	25	<lmxll>	ll	13	lm	238		
>ref-141456	AAAGTAAAAACTCCCTCTCCCCACACC	27	<lmxll>	ll	13	lm	237		
>ref-141472	GCGTCAGAGACTCCGGCTCCGGATTTT	4	<lmxll>	ll	268	lm	1		
>ref-141472	GCGTCAGAGACTCCGGCTCCGGATTTT	6	<hkxhk>	hh	82	hk	155	kk	0
>ref-141472	GCGTCAGAGACTCCGGCTCCGGATTTT	14	<lmxll>	ll	213	lm	40		
>ref-141472	GCGTCAGAGACTCCGGCTCCGGATTTT	16	<nnxnp>	nn	116	np	123		
>ref-141472	GCGTCAGAGACTCCGGCTCCGGATTTT	27	<lmxll>	ll	234	lm	26		
>ref-141496	TTCCCAAATACTGTTCCTCCTCCTCTT	2	<hkxhk>	hh	0	hk	272	kk	0
>ref-141496	TTCCCAAATACTGTTCCTCCTCCTCTT	25	<lmxll>	ll	216	lm	48		
>ref-141496	TTCCCAAATACTGTTCCTCCTCCTCTT	26	<hkxhk>	hh	0	hk	270	kk	3
>ref-141496	TTCCCAAATACTGTTCCTCCTCCTCTT	27	<lmxll>	ll	212	lm	54		
>ref-141522	GACGCGTGGACTAGGGCTCCCATGATC	2	<lmxll>	ll	124	lm	135		
>ref-141522	GACGCGTGGACTAGGGCTCCCATGATC	3	<hkxhk>	hh	12	hk	259	kk	0
>ref-141522	GACGCGTGGACTAGGGCTCCCATGATC	7	<hkxhk>	hh	0	hk	271	kk	1

>ref-141522	GACGCGTGGACTAGGGCTCCCATGATC	15	<hkxhk>	hh	0	hk	271	kk	1
>ref-141522	GACGCGTGGACTAGGGCTCCCATGATC	23	<nnxnp>	nn	273	np	0		
>ref-141522	GACGCGTGGACTAGGGCTCCCATGATC	27	<hkxhk>	hh	0	hk	273	kk	0
>ref-141526	TCCTGACGTACCATCACTCCATGAGGT	2	<hkxhk>	hh	0	hk	272	kk	0
>ref-141530	TGTCATGCAACTCTTCCTCCCGAACAG	13	<nnxnp>	nn	261	np	0		
>ref-141559	CTCTCTTTCACACCCCCTCCTTTCACA	1	<lmxll>	ll	121	lm	112		
>ref-141564	CTTACACCTACCTCCTCTCCTTCCCTC	27	<hkxhk>	hh	132	hk	123	kk	2
>ref-141565	CTGAGCGTGACATTCTCTCCATCTTTA	5	<lmxll>	ll	148	lm	104		
>ref-141577	AGCTGGTCCACATTGGCTCCCACTCAA	4	<lmxll>	ll	135	lm	134		
>ref-141601	AATAGAGGGACATATTCTCCTGGCGAT	14	<hkxhk>	hh	59	hk	153	kk	37
>ref-141603	ACTGCTGTCACTCTTTCTCCCAGTGAT	24	<lmxll>	ll	105	lm	140		
>ref-141617	AATAGGAGGACCGCGCCTCCGTTTCTT	22	<nnxnp>	nn	116	np	133		
>ref-141646	ACTCCGCCAACCTCCCCTCCCCTCGCC	9	<hkxhk>	hh	4	hk	268	kk	0
>ref-141670	TCCCCTCCTACCCACGCTCCTCCAATG	22	<lmxll>	ll	131	lm	139		
>ref-141677	ATCCCCTCAACATGTTCTCCCCTCCCT	2	<lmxll>	ll	118	lm	135		
>ref-141677	ATCCCCTCAACATGTTCTCCCCTCCCT	16	<lmxll>	ll	117	lm	135		
>ref-141688	ATGCTTCAGACTAACCCTCCCCCCTCT	26	<nnxnp>	nn	267	np	0		
>ref-141688	ATGCTTCAGACTAACCCTCCCCCCTCT	27	<nnxnp>	nn	265	np	0		
>ref-141701	CTCACCCACACACACTCTCCTAAATAT	2	<nnxnp>	nn	246	np	0		
>ref-141701	CTCACCCACACACACTCTCCTAAATAT	25	<nnxnp>	nn	250	np	0		
>ref-141704	CCCACCTCTACCCTACCTCCTCTCCTG	13	<lmxll>	ll	103	lm	138		
>ref-141708	TGATCCTAAACTCCGCCTCCTCTTCTC	6	<nnxnp>	nn	115	np	144		
>ref-141716	GAAACGTTTACATACTCTCCCACACAC	2	<nnxnp>	nn	96	np	159		
>ref-141738	CCCTCACCTACACCACCTCCACGCACT	7	<hkxhk>	hh	67	hk	206	kk	0
>ref-141738	CCCTCACCTACACCACCTCCACGCACT	9	<hkxhk>	hh	53	hk	145	kk	67
>ref-141738	CCCTCACCTACACCACCTCCACGCACT	22	<lmxll>	ll	161	lm	103		
>ref-141764	ACCTTCTCTACCTCACCTCCCTCCCTT	15	<nnxnp>	nn	176	np	67		
>ref-141780	ACAGTTGCAACAACAGCTCCTCCAACT	5	<lmxll>	ll	213	lm	45		
>ref-141823	GCCTATACAACCATCCCTCCCATCTCC	23	<hkxhk>	hh	62	hk	146	kk	53
>ref-141856	TGCTATGCCACTCCAGCTCCTTCTATG	2	<hkxhk>	hh	90	hk	116	kk	44
>ref-141876	CTCCTTAACACCCTCTCTCCACTCCCT	16	<hkxhk>	hh	0	hk	265	kk	0
>ref-141909	CATCAATCTACCCCTCCTCCCTCCTTC	8	<nnxnp>	nn	182	np	83		
>ref-141909	CATCAATCTACCCCTCCTCCCTCCTTC	15	<lmxll>	ll	132	lm	120		
>ref-141909	CATCAATCTACCCCTCCTCCCTCCTTC	24	<lmxll>	ll	133	lm	117		
>ref-141935	CCACCCACCACCCACCCTCCTACCCTT	1	<hkxhk>	hh	97	hk	169	kk	0
>ref-141935	CCACCCACCACCCACCCTCCTACCCTT	23	<lmxll>	ll	1	lm	270		
>ref-141935	CCACCCACCACCCACCCTCCTACCCTT	26	<lmxll>	ll	1	lm	270		
>ref-141946	ACCACTCCCACCCCATCTCCTACTCCC	21	<lmxll>	ll	71	lm	161		

>ref-141949	TCGTGATGCACATAGGCTCCTCTGTGA	6	<nnxnp>	nn	123	np	146		
>ref-141953	CACTTTGTTACTTTCTCTCCTTTTGTT	5	<hkxhk>	hh	43	hk	140	kk	63
>ref-141957	CAAATACACACAACCACTCCACACACA	22	<hkxhk>	hh	0	hk	273	kk	0
>ref-141957	CAAATACACACAACCACTCCACACACA	25	<hkxhk>	hh	0	hk	273	kk	0
>ref-142002	ACCAGCCACACCCTTTCTCCCTCCACA	3	<nnxnp>	nn	131	np	132		
>ref-142040	CCTACAACTACGGCATCTCCGGTGACT	22	<hkxhk>	hh	0	hk	273	kk	0
>ref-142043	TTCCCATCTACGCACTCTCCCCCCCTC	3	<lmxll>	ll	122	lm	135		
>ref-142058	AATGTAAACACACCCACTCCAACACGC	5	<hkxhk>	hh	0	hk	272	kk	1
>ref-142058	AATGTAAACACACCCACTCCAACACGC	26	<hkxhk>	hh	0	hk	266	kk	5
>ref-142075	TTCTCCTCCACCTAACCTCCTCTTTCT	2	<hkxhk>	hh	0	hk	9	kk	264
>ref-142075	TTCTCCTCCACCTAACCTCCTCTTTCT	3	<lmxll>	ll	57	lm	199		
>ref-142075	TTCTCCTCCACCTAACCTCCTCTTTCT	6	<hkxhk>	hh	264	hk	9	kk	0
>ref-142075	TTCTCCTCCACCTAACCTCCTCTTTCT	16	<hkxhk>	hh	54	hk	203	kk	0
>ref-142075	TTCTCCTCCACCTAACCTCCTCTTTCT	22	<hkxhk>	hh	0	hk	203	kk	53
>ref-142114	AAATGTCCCACGCTCACTCCTCCCTTT	4	<nnxnp>	nn	116	np	144		
>ref-142140	TACACGCACACTCAACCTCCGTCCTTC	14	<hkxhk>	hh	5	hk	267	kk	0
>ref-142140	TACACGCACACTCAACCTCCGTCCTTC	21	<hkxhk>	hh	0	hk	273	kk	0
>ref-142148	TTACCCATGACACCCCCTCCCCCTCCT	21	<nnxnp>	nn	272	np	0		
>ref-142154	TTATCCCCAACTGCGCCTCCTGGCCAG	9	<nnxnp>	nn	128	np	123		
>ref-142160	CCCATCCTCACTTCTTCTCCCTTTCCT	3	<lmxll>	ll	128	lm	109		
>ref-142160	CCCATCCTCACTTCTTCTCCCTTTCCT	4	<lmxll>	ll	251	lm	0		
>ref-142176	TCTTCTTCTACAGTTACTCCCTCTTTT	21	<lmxll>	ll	128	lm	130		
>ref-142176	TCTTCTTCTACAGTTACTCCCTCTTTT	23	<lmxll>	ll	128	lm	130		
>ref-142180	ACGTGGAGAACCTCCGCTCCAACGCCG	7	<lmxll>	ll	81	lm	174		
>ref-142192	TCTATCCTTACTCCTTCTCCCTCACTT	25	<nnxnp>	nn	8	np	265		
>ref-142209	CCTTCCCAAACATCGCCTCCTCCCTCT	21	<lmxll>	ll	116	lm	138		
>ref-142209	CCTTCCCAAACATCGCCTCCTCCCTCT	26	<lmxll>	ll	115	lm	139		
>ref-142211	AGATTCGACACTGTGGCTCCCTAACGC	12	<lmxll>	ll	125	lm	115		
>ref-142234	TTTTCTCCTACCATCTCTCCCTCCATT	13	<hkxhk>	hh	53	hk	120	kk	53
>ref-142297	GCTGGCAATACTTGCGCTCCGGCAGGC	8	<nnxnp>	nn	121	np	145		
>ref-142306	CTTCTTCCCACATCATCTCCAAGCATC	27	<lmxll>	ll	129	lm	139		
>ref-142332	CTATCCATTACTCCACCTCCCCCTTTC	14	<nnxnp>	nn	109	np	118		
>ref-142332	CTATCCATTACTCCACCTCCCCCTTTC	23	<lmxll>	ll	132	lm	99		
>ref-142333	CCGCCCTCGACCACGCCTCCCTGTGAC	23	<lmxll>	ll	150	lm	119		
>ref-142370	CACACGCTCACTCTCTCTCCTTCATGC	5	<hkxhk>	hh	53	hk	131	kk	52
>ref-142393	CCCATGTCTACTCATGCTCCATCCCCA	6	<nnxnp>	nn	122	np	135		
>ref-142397	AAGGATTAAACATATCCTCCTTAGAAT	5	<nnxnp>	nn	117	np	133		
>ref-142400	TAACTTCAAACACAACCTCCTTATTCA	2	<nnxnp>	nn	93	np	147		

>ref-142416	ATACAAGGGACCTATTCTCCCACACAG	21	<nnxnp>	nn	105	np	139		
>ref-142437	GAGGGAACAACTGCCCCTCCCTGCCCC	1	<lmxll>	ll	134	lm	109		
>ref-142456	GATACAAGAACAGGCTCTCCGAACAAA	15	<nnxnp>	nn	158	np	92		
>ref-142458	TTCCATCTCACTCTCCCTCCATTTCTA	5	<lmxll>	ll	129	lm	127		
>ref-142459	TATAAGCACACAAGCACTCCCTCTCAC	5	<nnxnp>	nn	135	np	134		
>ref-142500	ATTTCCTTTACTCTCTCTCCTCACTTA	27	<lmxll>	ll	125	lm	117		
>ref-142509	CCCAAACCAACTAACCCTCCTCTTCTT	23	<nnxnp>	nn	118	np	129		
>ref-142537	CCAGTCGCTACACCTCCTCCATTGACG	5	<lmxll>	ll	182	lm	76		
>ref-142537	CCAGTCGCTACACCTCCTCCATTGACG	6	<hkxhk>	hh	1	hk	271	kk	0
>ref-142541	CACACGGACACGCACACTCCTCACACG	7	<nnxnp>	nn	123	np	142		
>ref-142589	CCACCCACCACCGCCACTCCTCCTCCT	7	<hkxhk>	hh	5	hk	242	kk	1
>ref-142589	CCACCCACCACCGCCACTCCTCCTCCT	9	<nnxnp>	nn	142	np	96		
>ref-142589	CCACCCACCACCGCCACTCCTCCTCCT	12	<hkxhk>	hh	5	hk	243	kk	1
>ref-142591	GTCCTCATCACGATTACTCCTTCCTGG	14	<nnxnp>	nn	134	np	130		
>ref-142630	CAGCTGCTGACTCTCCCTCCCTGACAT	3	<lmxll>	ll	0	lm	273		
>ref-142633	AGCGGAGCGACTTCCACTCCCCGACCT	23	<nnxnp>	nn	263	np	0		
>ref-142635	CTTACGTTAACTATCACTCCTGTTCAC	6	<hkxhk>	hh	0	hk	10	kk	260
>ref-142635	CTTACGTTAACTATCACTCCTGTTCAC	9	<hkxhk>	hh	259	hk	12	kk	0
>ref-142635	CTTACGTTAACTATCACTCCTGTTCAC	25	<hkxhk>	hh	9	hk	262	kk	0
>ref-142635	CTTACGTTAACTATCACTCCTGTTCAC	26	<lmxll>	ll	7	lm	265		
>ref-142635	CTTACGTTAACTATCACTCCTGTTCAC	27	<hkxhk>	hh	0	hk	273	kk	0
>ref-142655	CTTCCACCAACCTTCGCTCCTTCCACC	25	<nnxnp>	nn	256	np	0		
>ref-142657	CACTTTTCCACCCTCTCTCCTCCCTCT	4	<hkxhk>	hh	0	hk	207	kk	44
>ref-142657	CACTTTTCCACCCTCTCTCCTCCCTCT	25	<hkxhk>	hh	0	hk	207	kk	46
>ref-142676	TTCCTTTCGACCTAAACTCCCTGTGGA	9	<lmxll>	ll	113	lm	133		
>ref-142684	ACTCACTTCACTTCTGCTCCACCAATT	23	<lmxll>	ll	140	lm	127		
>ref-142689	ACAACTTCCACCCTCTCTCCCCGCAAA	9	<lmxll>	ll	121	lm	119		
>ref-142692	ATACTCCCAACCTGACCTCCTACTCCT	22	<nnxnp>	nn	126	np	131		
>ref-142702	AAGAATACGACTCTCCCTCCTCTCTCT	16	<lmxll>	ll	111	lm	142		
>ref-142723	CCTCCGGCCACTGATGCTCCCACGCTA	22	<lmxll>	ll	119	lm	140		
>ref-142731	CATACACACACATCCCCTCCTCCGTTC	14	<nnxnp>	nn	110	np	150		
>ref-142738	GGACCACCGACTCCATCTCCCACCACG	6	<nnxnp>	nn	269	np	0		
>ref-142738	GGACCACCGACTCCATCTCCCACCACG	26	<nnxnp>	nn	271	np	0		
>ref-142740	CACAAACAAACTCACCCTCCATAGTTC	2	<nnxnp>	nn	135	np	123		
>ref-142753	CACACACACACGTTTTCTCCTGAACGA	2	<lmxll>	ll	124	lm	114		
>ref-142801	ACACGAAACACCTGTGCTCCGCTGTTG	4	<nnxnp>	nn	149	np	120		
>ref-142802	TAATTTTGAACTTTGTCTCCCTCTCTG	9	<nnxnp>	nn	257	np	0		
>ref-142857	ATTAGCGCAACAAGTGCTCCGCGGAAG	1	<lmxll>	ll	128	lm	118		

>ref-142860	GAACTAAGGACTGCATCTCCCTGATAG	9	<lmxll>	ll	146	lm	101		
>ref-142864	CGAACCGACACTCTCACTCCACGTCTG	6	<nnxnp>	nn	5	np	257		
>ref-142883	TCCTGTCCAACAGCGCCTCCGCCAATA	1	<lmxll>	ll	30	lm	233		
>ref-142883	TCCTGTCCAACAGCGCCTCCGCCAATA	7	<nnxnp>	nn	153	np	115		
>ref-142883	TCCTGTCCAACAGCGCCTCCGCCAATA	27	<hkxhk>	hh	10	hk	261	kk	0
>ref-142896	TTCCACCCAACCATCCCTCCTTCACTC	24	<lmxll>	ll	126	lm	141		
>ref-142940	CATATCACGACCGCTGCTCCTGGCCTT	27	<nnxnp>	nn	135	np	135		
>ref-142942	CGTATCCCTACTCTCCCTCCCTCTCCC	3	<lmxll>	ll	4	lm	261		
>ref-142942	CGTATCCCTACTCTCCCTCCCTCTCCC	4	<nnxnp>	nn	141	np	111		
>ref-142954	AACGAATACACAAAATCTCCTCTCTCT	9	<lmxll>	ll	139	lm	128		
>ref-142958	CTTTGAAGAACCGACTCTCCCTTCTCT	12	<nnxnp>	nn	137	np	126		
>ref-142959	CAAGGCCCAACCCCCACTCCCGAGTAC	16	<nnxnp>	nn	69	np	157		
>ref-142967	ATCATCACTACATCATCTCCATCATCA	8	<hkxhk>	hh	1	hk	272	kk	0
>ref-142967	ATCATCACTACATCATCTCCATCATCA	9	<hkxhk>	hh	0	hk	268	kk	3
>ref-142967	ATCATCACTACATCATCTCCATCATCA	13	<hkxhk>	hh	0	hk	268	kk	3
>ref-142998	CTTGCATAAACAAGGTCTCCTGTGTAT	14	<lmxll>	ll	148	lm	104		
>ref-143051	AAGCAGCAAACACAGTCTCCGACACAA	21	<hkxhk>	hh	0	hk	81	kk	168
>ref-143051	AAGCAGCAAACACAGTCTCCGACACAA	24	<hkxhk>	hh	168	hk	81	kk	0
>ref-143062	CTTGGTCCTACCTCCTCTCCCTTTCTC	12	<nnxnp>	nn	130	np	136		
>ref-143089	ACCCGCACCACCTCCCCTCCTTCTCAC	22	<hkxhk>	hh	57	hk	157	kk	52
>ref-143089	ACCCGCACCACCTCCCCTCCTTCTCAC	23	<hkxhk>	hh	51	hk	157	kk	57
>ref-143121	CACGTTCTGACGTTCGCTCCAGCGGTC	25	<nnxnp>	nn	137	np	133		
>ref-143123	AAACCAGAAACTTAACCTCCCTAATTT	14	<nnxnp>	nn	136	np	94		
>ref-143123	AAACCAGAAACTTAACCTCCCTAATTT	16	<nnxnp>	nn	136	np	94		
>ref-143125	GTCACACCCACAGGCCCTCCTCTCCTT	15	<nnxnp>	nn	110	np	139		
>ref-143131	CCTCACCACACCCCTCCTCCATCCCTA	25	<hkxhk>	hh	88	hk	171	kk	0
>ref-143134	GAAAGCCCCACTTGACCTCCTCTGCTG	7	<lmxll>	ll	144	lm	121		
>ref-143150	TGTCTAAGGACTGCCGCTCCCAACCTC	26	<hkxhk>	hh	40	hk	131	kk	62
>ref-143205	CTGTCTTCCACTTTTTCTCCTTCTTTC	5	<lmxll>	ll	117	lm	110		
>ref-143209	CCTCCCCTTACCCCTTCTCCCAATCTC	21	<lmxll>	ll	125	lm	117		
>ref-143214	GTCTCCTCAACTCGGCCTCCGTCCCGT	4	<hkxhk>	hh	1	hk	263	kk	0
>ref-143224	CTAACACGCACGTCTTCTCCTTTATCT	2	<hkxhk>	hh	60	hk	131	kk	72
>ref-143236	GCCAGGGCAACGTACCCTCCGCTAAAG	24	<nnxnp>	nn	146	np	102		
>ref-143236	GCCAGGGCAACGTACCCTCCGCTAAAG	25	<nnxnp>	nn	147	np	101		
>ref-143239	ATACAAAAAACATTTCCTCCACAGTGC	21	<nnxnp>	nn	240	np	18		
>ref-143239	ATACAAAAAACATTTCCTCCACAGTGC	24	<nnxnp>	nn	240	np	18		
>ref-143239	ATACAAAAAACATTTCCTCCACAGTGC	27	<lmxll>	ll	231	lm	29		
>ref-143288	CAGGATACGACACTATCTCCTCCCCCT	15	<nnxnp>	nn	130	np	140		

>ref-143298	TTAAACGAGACAAGGGCTCCTTCTCTC	1	<lmxll>	ll	147	lm	123		
>ref-143337	ACCCTCTCCACCAACCCTCCTTCTGAC	14	<nnxnp>	nn	267	np	0		
>ref-143340	ACATCACACACTCCCGCTCCAATCTTC	15	<hkxhk>	hh	55	hk	124	kk	42
>ref-143340	ACATCACACACTCCCGCTCCAATCTTC	25	<lmxll>	ll	94	lm	127		
>ref-143344	TGCCTGACAACAGTTCCTCCGTTATTA	14	<lmxll>	ll	119	lm	151		
>ref-143360	GCATTTTAAACCGGACCTCCGAAGCGT	26	<nnxnp>	nn	119	np	141		
>ref-143360	GCATTTTAAACCGGACCTCCGAAGCGT	27	<nnxnp>	nn	119	np	141		
>ref-143399	TTTACTTTCACTCTCTCTCCATCTCGT	4	<lmxll>	ll	134	lm	110		
>ref-143414	CCTTGCAGCACGAAATCTCCCTCTGTT	7	<hkxhk>	hh	2	hk	270	kk	0
>ref-143432	CGAAGCACAACAGGCCCTCCTGCGCGT	12	<nnxnp>	nn	140	np	120		
>ref-143454	CAGACATGCACTCTCTCTCCAACCCTC	7	<lmxll>	ll	111	lm	158		
>ref-143457	TCCACCTCCACTCTCCCTCCTATCCCC	9	<hkxhk>	hh	1	hk	264	kk	0
>ref-143457	TCCACCTCCACTCTCCCTCCTATCCCC	12	<hkxhk>	hh	0	hk	264	kk	0
>ref-143457	TCCACCTCCACTCTCCCTCCTATCCCC	16	<hkxhk>	hh	2	hk	261	kk	0
>ref-143457	TCCACCTCCACTCTCCCTCCTATCCCC	22	<hkxhk>	hh	1	hk	263	kk	0
>ref-143486	TCTTCCTGGACCCTGTCTCCGCGTCTG	1	<lmxll>	ll	145	lm	123		
>ref-143494	CAGGAACACACGCGCACTCCATCACGT	3	<lmxll>	ll	141	lm	131		
>ref-143514	CCCCTTACGACCCCACCTCCTCTGCCC	1	<nnxnp>	nn	89	np	142		
>ref-143519	TCTTCTCCCACTTCTGCTCCCTATCCT	27	<hkxhk>	hh	46	hk	138	kk	65
>ref-143542	CAGTATAAGACTTCAACTCCTCCTTAA	4	<lmxll>	ll	128	lm	121		
>ref-143558	GAGGCCAGCACCTCAACTCCAACCGCT	4	<nnxnp>	nn	133	np	139		
>ref-143567	CTCTCTTTCACTCTCTCTCCTTGCGCA	25	<nnxnp>	nn	128	np	133		
>ref-143575	CACCTATTCACGGTCGCTCCCCTACAC	15	<nnxnp>	nn	132	np	135		
>ref-143586	GCAAAACATACTGATGCTCCCGTAGCC	16	<nnxnp>	nn	129	np	123		
>ref-143591	TTTCACCCCACTCTCTCTCCCTTTCCC	23	<nnxnp>	nn	231	np	13		
>ref-143591	TTTCACCCCACTCTCTCTCCCTTTCCC	25	<nnxnp>	nn	170	np	61		
>ref-143593	GATCGCGGAACTTTCACTCCATTAGTT	5	<lmxll>	ll	2	lm	237		
>ref-143596	GGATCTAATACCAGAGCTCCAGCACGT	27	<nnxnp>	nn	130	np	133		
>ref-143604	TAGTATCCCACTCTCTCTCCCCTCTTT	8	<hkxhk>	hh	55	hk	212	kk	0
>ref-143609	GCGGAGAGAACTTGAACTCCAGGCTCG	5	<nnxnp>	nn	260	np	0		
>ref-143639	TTCATGAACACCCTCTCTCCTTCTCCT	27	<lmxll>	ll	251	lm	5		
>ref-143683	AATCCCTGCACACTTACTCCCCTCCCC	8	<hkxhk>	hh	1	hk	244	kk	0
>ref-143683	AATCCCTGCACACTTACTCCCCTCCCC	26	<hkxhk>	hh	1	hk	257	kk	7
>ref-143694	TAGGCCGTGACCCTGACTCCTTTAAAT	13	<nnxnp>	nn	129	np	138		
>ref-143744	CACTCTGCCACTCCCTCTCCCATCTCC	14	<lmxll>	ll	155	lm	113		
>ref-143748	TTTCCCTTGACTCCCACTCCATCCCTC	6	<hkxhk>	hh	58	hk	140	kk	58
>ref-143768	ACTCACGCAACCTCGCCTCCAGCGCCT	2	<nnxnp>	nn	271	np	0		
>ref-143776	TTCCCTTCCACTTCATCTCCTCCCCTC	25	<lmxll>	ll	151	lm	114		

>ref-143780	TCCCTTAAGACCCTCTCTCCATCATCT	8	<nnxnp>	nn	94	np	163		
>ref-143800	AGATTATCGACCTGTTCTCCATCACCA	1	<hkxhk>	hh	4	hk	267	kk	1
>ref-143800	AGATTATCGACCTGTTCTCCATCACCA	2	<hkxhk>	hh	1	hk	267	kk	4
>ref-143823	CCTCGACCTACGATTACTCCAACGGAC	2	<lmxll>	ll	76	lm	192		
>ref-143823	CCTCGACCTACGATTACTCCAACGGAC	8	<lmxll>	ll	130	lm	138		
>ref-143858	TAACCACACACACACACTCCAACACAG	7	<lmxll>	ll	122	lm	147		
>ref-143858	TAACCACACACACACACTCCAACACAG	25	<lmxll>	ll	120	lm	149		
>ref-143864	CTCATTCACACTTCATCTCCTCTTCTC	15	<hkxhk>	hh	0	hk	273	kk	0
>ref-143874	TGACTTTAAACTCTCTCTCCCTCATCA	8	<nnxnp>	nn	114	np	146		
>ref-143876	ACGTGCTGTACATGTGCTCCTTCGTTT	9	<lmxll>	ll	130	lm	143		
>ref-143890	CGGGGCGCTACTCGCTCTCCGAGAATC	5	<hkxhk>	hh	2	hk	264	kk	0
>ref-143890	CGGGGCGCTACTCGCTCTCCGAGAATC	6	<lmxll>	ll	144	lm	101		
>ref-143892	CTTAACTTGACTCTTTCTCCATCTATC	25	<hkxhk>	hh	57	hk	117	kk	63
>ref-143925	CTACAGCCAACTTCGTCTCCACAGTTC	12	<nnxnp>	nn	114	np	151		
>ref-143925	CTACAGCCAACTTCGTCTCCACAGTTC	26	<nnxnp>	nn	114	np	151		
>ref-143927	CTGAGTGCCACTCGCTCTCCCTCTCTT	27	<lmxll>	ll	126	lm	144		
>ref-143928	CTGCCTCGAACACGATCTCCCTCCTTC	25	<nnxnp>	nn	63	np	202		
>ref-143930	CACGCACTCACTCATACTCCCTTAGTA	23	<lmxll>	ll	120	lm	145		
>ref-143931	TCAAAGTCAACCAACTCTCCTTCTGAT	24	<lmxll>	ll	98	lm	125		
>ref-143959	ACTCGGGGCACCTGCTCTCCTGCCTTT	4	<lmxll>	ll	151	lm	120		
>ref-143961	ATTGTCAACACTAGCGCTCCATCCGCT	2	<nnxnp>	nn	125	np	137		
>ref-143968	TCCCTCTCGACTAGCACTCCCTCCCTT	2	<hkxhk>	hh	0	hk	273	kk	0
>ref-143968	TCCCTCTCGACTAGCACTCCCTCCCTT	16	<hkxhk>	hh	0	hk	273	kk	0
>ref-143968	TCCCTCTCGACTAGCACTCCCTCCCTT	27	<hkxhk>	hh	0	hk	263	kk	5
>ref-143975	GTTAGACTTACTCTCTCTCCCTACTCT	26	<nnxnp>	nn	125	np	133		
>ref-143976	CTCCGTTTTACGTTATCTCCTCTTTCC	6	<hkxhk>	hh	35	hk	117	kk	69
>ref-143976	CTCCGTTTTACGTTATCTCCTCTTTCC	27	<hkxhk>	hh	37	hk	122	kk	64
>ref-143977	CACGATACAACCCCCCCTCCCCTCTCT	21	<nnxnp>	nn	109	np	117		
>ref-143988	CGTACCATCACTGCTACTCCTCTCTTG	23	<nnxnp>	nn	149	np	120		
>ref-143988	CGTACCATCACTGCTACTCCTCTCTTG	27	<nnxnp>	nn	149	np	120		
>ref-143991	AACATTTGTACCATATCTCCTGATTCT	25	<nnxnp>	nn	104	np	143		
>ref-144001	TTATTATCCACCCTCTCTCCATCCCCA	13	<nnxnp>	nn	140	np	118		
>ref-144049	AGCTACTTGACACGAGCTCCTGACTGC	3	<hkxhk>	hh	21	hk	242	kk	0
>ref-144049	AGCTACTTGACACGAGCTCCTGACTGC	5	<hkxhk>	hh	21	hk	242	kk	0
>ref-144049	AGCTACTTGACACGAGCTCCTGACTGC	24	<hkxhk>	hh	150	hk	96	kk	0
>ref-144056	CGCACATACACATACACTCCCCTCAAA	2	<nnxnp>	nn	269	np	1		
>ref-144056	CGCACATACACATACACTCCCCTCAAA	25	<nnxnp>	nn	270	np	1		
>ref-144064	CATCTTCAGACTTCTTCTCCTCCTCTA	2	<lmxll>	ll	103	lm	135		

>ref-144064	CATCTTCAGACTTCTTCTCCTCCTCTA	5	<lmxll>	ll	103	lm	135		
>ref-144083	CATCCTCATACCCAGCCTCCTCATCTC	15	<nnxnp>	nn	121	np	145		
>ref-144138	CTTTCCGCAACTCTCACTCCTTCTCCT	24	<nnxnp>	nn	93	np	128		
>ref-144146	CACCAGTACACCCCCACTCCTGCCCCC	22	<hkxhk>	hh	34	hk	109	kk	88
>ref-144161	ACTTAACTAACTTTCCCTCCAGATCTA	1	<hkxhk>	hh	1	hk	252	kk	7
>ref-144161	ACTTAACTAACTTTCCCTCCAGATCTA	27	<nnxnp>	nn	47	np	192		
>ref-144164	CCAACCCCCACGCCCCCTCCTCCCTCC	3	<hkxhk>	hh	74	hk	181	kk	1
>ref-144164	CCAACCCCCACGCCCCCTCCTCCCTCC	14	<nnxnp>	nn	16	np	240		
>ref-144173	ATATATTCCACTGTCTCTCCCTCGGCG	9	<nnxnp>	nn	131	np	117		
>ref-144183	TTATGTTGAACATCTTCTCCTAAGATT	8	<nnxnp>	nn	92	np	138		
>ref-144199	TTCCTCTTCACACCCCCTCCCCTCCCT	1	<nnxnp>	nn	67	np	172		
>ref-144199	TTCCTCTTCACACCCCCTCCCCTCCCT	12	<hkxhk>	hh	16	hk	246	kk	0
>ref-144199	TTCCTCTTCACACCCCCTCCCCTCCCT	24	<nnxnp>	nn	240	np	7		
>ref-144199	TTCCTCTTCACACCCCCTCCCCTCCCT	27	<nnxnp>	nn	67	np	171		
>ref-144200	AAGGTATATACAGTTACTCCTAATACC	4	<lmxll>	ll	127	lm	110		
>ref-144211	TAACCCCGGACACACTCTCCCTCCCTC	3	<hkxhk>	hh	117	hk	100	kk	6
>ref-144211	TAACCCCGGACACACTCTCCCTCCCTC	4	<hkxhk>	hh	135	hk	90	kk	5
>ref-144211	TAACCCCGGACACACTCTCCCTCCCTC	5	<hkxhk>	hh	135	hk	90	kk	5
>ref-144211	TAACCCCGGACACACTCTCCCTCCCTC	6	<hkxhk>	hh	5	hk	89	kk	137
>ref-144211	TAACCCCGGACACACTCTCCCTCCCTC	9	<hkxhk>	hh	5	hk	90	kk	137
>ref-144211	TAACCCCGGACACACTCTCCCTCCCTC	12	<hkxhk>	hh	137	hk	90	kk	5
>ref-144211	TAACCCCGGACACACTCTCCCTCCCTC	14	<hkxhk>	hh	134	hk	89	kk	6
>ref-144211	TAACCCCGGACACACTCTCCCTCCCTC	16	<hkxhk>	hh	5	hk	90	kk	137
>ref-144211	TAACCCCGGACACACTCTCCCTCCCTC	24	<hkxhk>	hh	5	hk	90	kk	137
>ref-144211	TAACCCCGGACACACTCTCCCTCCCTC	25	<hkxhk>	hh	5	hk	90	kk	137
>ref-144212	ACAGCCTCAACTCCCTCTCCCCTCTTC	5	<nnxnp>	nn	264	np	0		
>ref-144213	ATACGATATACATTTCCTCCTCATTCA	2	<nnxnp>	nn	254	np	0		
>ref-144213	ATACGATATACATTTCCTCCTCATTCA	21	<nnxnp>	nn	254	np	0		
>ref-144226	TGCCCCCAAACCCCGACTCCCCATCAA	7	<nnxnp>	nn	129	np	125		
>ref-144234	AACTCCACTACAGAAACTCCTGAAGAT	8	<hkxhk>	hh	1	hk	266	kk	0
>ref-144262	TTTACATAGACTTAACCTCCTCTAACT	9	<nnxnp>	nn	110	np	153		
>ref-144274	CCTAATATTACACAGACTCCTCGCTTA	14	<nnxnp>	nn	261	np	0		
>ref-144278	CTCTTTTTAACCCTTCCTCCCCCTTCT	9	<lmxll>	ll	141	lm	104		
>ref-144278	CTCTTTTTAACCCTTCCTCCCCCTTCT	25	<hkxhk>	hh	2	hk	184	kk	62
>ref-144289	CTCCCCCATACCACTACTCCCTCCCTT	8	<nnxnp>	nn	7	np	263		
>ref-144289	CTCCCCCATACCACTACTCCCTCCCTT	13	<nnxnp>	nn	7	np	263		
>ref-144298	CTCTCCTAAACCTAACCTCCCTCCATC	3	<hkxhk>	hh	40	hk	224	kk	0
>ref-144298	CTCTCCTAAACCTAACCTCCCTCCATC	4	<lmxll>	ll	16	lm	244		

>ref-144298	CTCTCCTAAACCTAACCTCCCTCCATC	13	<hkxhk>	hh	0	hk	271	kk	0
>ref-144298	CTCTCCTAAACCTAACCTCCCTCCATC	15	<lmxll>	ll	16	lm	244		
>ref-144298	CTCTCCTAAACCTAACCTCCCTCCATC	16	<nnxnp>	nn	270	np	2		
>ref-144331	CACAGTCCCACTCCCACTCCTAGTCAG	15	<hkxhk>	hh	64	hk	147	kk	47
>ref-144337	CTCTCTCTTACCCTTCCTCCCCCTTAA	12	<nnxnp>	nn	105	np	137		
>ref-144337	CTCTCTCTTACCCTTCCTCCCCCTTAA	15	<nnxnp>	nn	105	np	139		
>ref-144337	CTCTCTCTTACCCTTCCTCCCCCTTAA	16	<nnxnp>	nn	258	np	1		
>ref-144340	CAGCCGCAGACTTTGTCTCCGCCAATG	22	<nnxnp>	nn	135	np	131		
>ref-144360	AACACGCCCACGCCCTCTCCCTCAATC	23	<hkxhk>	hh	90	hk	130	kk	47
>ref-144366	CCGCCCAATACCCCCCCTCCCTTACTT	13	<lmxll>	ll	133	lm	119		
>ref-144392	AAAAGCAAAACTACATCTCCTGTGGCA	6	<lmxll>	ll	105	lm	117		
>ref-144392	AAAAGCAAAACTACATCTCCTGTGGCA	13	<hkxhk>	hh	4	hk	218	kk	0
>ref-144392	AAAAGCAAAACTACATCTCCTGTGGCA	15	<nnxnp>	nn	132	np	97		
>ref-144395	GTCCGCCAAACCTTCGCTCCGGCCGAC	15	<nnxnp>	nn	130	np	137		
>ref-144398	TGGATGAATACAGTCTCTCCTTACAGA	2	<hkxhk>	hh	44	hk	129	kk	61
>ref-144408	ACCCTTCCCACCTTCTCTCCACAGCCT	2	<hkxhk>	hh	62	hk	142	kk	36
>ref-144418	GCATGTGATACCCTCGCTCCCAGGGTT	3	<lmxll>	ll	125	lm	145		
>ref-144421	TTTGGCATAACAACACCTCCAGCCAGA	3	<nnxnp>	nn	259	np	0		
>ref-144439	CGACGCAGAACTCCGACTCCGTTGAAA	13	<nnxnp>	nn	127	np	122		
>ref-144441	ATTGTGCACACACGCCCTCCAGCCATT	13	<hkxhk>	hh	54	hk	151	kk	64
>ref-144450	TCCTATTCAACTCTTCCTCCTTCTCAC	22	<hkxhk>	hh	0	hk	244	kk	21
>ref-144459	TTCTCCCCCACCCCCCCTCCTCTCCTG	6	<lmxll>	ll	234	lm	1		
>ref-144459	TTCTCCCCCACCCCCCCTCCTCTCCTG	23	<lmxll>	ll	228	lm	4		
>ref-144464	CTCCCGTCCACCCTTCCTCCCCACTCT	6	<nnxnp>	nn	2	np	269		
>ref-144464	CTCCCGTCCACCCTTCCTCCCCACTCT	9	<nnxnp>	nn	2	np	268		
>ref-144470	AACACCCTTACCTCCCCTCCTCTTCAT	3	<nnxnp>	nn	262	np	1		
>ref-144470	AACACCCTTACCTCCCCTCCTCTTCAT	7	<nnxnp>	nn	114	np	137		
>ref-144484	TTGACCACGACGGGTGCTCCTCCCGCC	6	<nnxnp>	nn	147	np	123		
>ref-144491	ATGTTGTGCACTCCTACTCCCTCTCCC	15	<lmxll>	ll	108	lm	139		
>ref-144491	ATGTTGTGCACTCCTACTCCCTCTCCC	16	<lmxll>	ll	110	lm	135		
>ref-144494	TCTGCTCCGACTTTGGCTCCAGGTTAC	24	<hkxhk>	hh	53	hk	144	kk	63
>ref-144497	TAAGAGGTTACTCCTCCTCCTCCTCAC	1	<nnxnp>	nn	130	np	130		
>ref-144497	TAAGAGGTTACTCCTCCTCCTCCTCAC	26	<lmxll>	ll	122	lm	142		
>ref-144497	TAAGAGGTTACTCCTCCTCCTCCTCAC	27	<lmxll>	ll	122	lm	142		
>ref-144498	ACGTCTGCCACTGTCCCTCCACCGCCG	14	<hkxhk>	hh	0	hk	273	kk	0
>ref-144499	ATAGAAGTTACCGCTCCTCCACCTTCA	3	<hkxhk>	hh	4	hk	262	kk	0
>ref-144499	ATAGAAGTTACCGCTCCTCCACCTTCA	14	<lmxll>	ll	147	lm	104		
>ref-144505	CATGTTGTAACACTGCCTCCGTGATAT	4	<nnxnp>	nn	142	np	130		

>ref-144515	AAACTTTAAACTCCTTCTCCTCTCTCT	6	<lmxll>	ll	112	lm	125			
>ref-144517	AGACATGGCACTGGCACTCCCTGGCTC	22	<lmxll>	ll	265	lm	1			
>ref-144523	TCGTTTTTCACTTCTCCTCCTTCTCCG	9	<lmxll>	ll	10	lm	249			
>ref-144523	TCGTTTTTCACTTCTCCTCCTTCTCCG	22	<lmxll>	ll	10	lm	249			
>ref-144546	CCCTCTTGTACCATCTCTCCTTTCTTT	3	<hkxhk>	hh	3	hk	263	kk	1	
>ref-144546	CCCTCTTGTACCATCTCTCCTTTCTTT	15	<hkxhk>	hh	3	hk	263	kk	1	
>ref-144576	AGCCTAGGAACCGTGACTCCCTTTGTT	22	<hkxhk>	hh	45	hk	146	kk	57	
>ref-144588	GACCGGGAAACGAAGGCTCCCCGTCAA	12	<nnxnp>	nn	108	np	115			
>ref-144600	CCCTCAACCACCCCCTCTCCCCAAGTA	5	<lmxll>	ll	112	lm	130			
>ref-144614	CCCACTCCCACCTCCACTCCCACCTCT	13	<nnxnp>	nn	3	np	265			
>ref-144652	AATAAGGCCACGCGAGCTCCAGCCGTA	25	<lmxll>	ll	131	lm	133			
>ref-144674	TGACACACGACCCGACCTCCACCTTGC	22	<nnxnp>	nn	124	np	144			
>ref-144678	CTTTCTATAACTTTCTCTCCTCTTTCA	1	<lmxll>	ll	134	lm	110			
>ref-144702	TTCTGTCGTACTCCATCTCCATCGCAG	7	<lmxll>	ll	118	lm	149			
>ref-144707	CAGGATCTCACCGCGACTCCATTCATC	8	<nnxnp>	nn	131	np	133			
>ref-144712	TTCCTTATAACCATTCCTCCCCTCTTC	16	<lmxll>	ll	112	lm	112			
>ref-144712	TTCCTTATAACCATTCCTCCCCTCTTC	26	<lmxll>	ll	112	lm	113			
>ref-144722	CACCCTTCAACCTCTTCTCCCCTCTTT	13	<nnxnp>	nn	33	np	227			
>ref-144722	CACCCTTCAACCTCTTCTCCCCTCTTT	25	<nnxnp>	nn	33	np	227			
>ref-144728	TATATAACTACAGCAGCTCCATTGTGA	22	<nnxnp>	nn	262	np	0			
>ref-144735	TATACATTAACCTCGTCTCCACTGCTT	5	<nnxnp>	nn	123	np	139			
>ref-144752	CCGCACCGCACGTCTTCTCCCTTGCAG	5	<lmxll>	ll	159	lm	113			
>ref-144763	ATTTCCTTGACACACGCTCCCGCCCCT	12	<lmxll>	ll	130	lm	132			
>ref-144767	TTCCCCTCTACCCTTCCTCCAACCCCA	9	<lmxll>	ll	261	lm	0			
>ref-144774	TTCAAAATCACTCAGGCTCCAACCTCG	26	<lmxll>	ll	111	lm	141			
>ref-144819	TTGGTTCTGACCCTCCCTCCTTCCCTC	9	<nnxnp>	nn	263	np	0			
>ref-144822	AGCACAGGAACTTTCGCTCCGTAAGTG	14	<nnxnp>	nn	113	np	143			
>ref-144823	TGCACTGATACCGAAACTCCCACACAG	8	<lmxll>	ll	115	lm	119			
>ref-144825	CTGTACCTCACTTTGACTCCGACGCAT	24	<lmxll>	ll	117	lm	140			
>ref-144831	ACAGGTCAAACAAAGGCTCCTTCCTAC	24	<hkxhk>	hh	147	hk	105	kk	3	
>ref-144838	GGAATTGCCACAACACCTCCGTTATTA	12	<lmxll>	ll	131	lm	115			
>ref-144855	CAAGGTGACACTCTCGCTCCATATCTA	3	<lmxll>	ll	70	lm	187			
>ref-144855	CAAGGTGACACTCTCGCTCCATATCTA	9	<lmxll>	ll	70	lm	186			
>ref-144855	CAAGGTGACACTCTCGCTCCATATCTA	15	<hkxhk>	hh	20	hk	225	kk	0	
>ref-144870	CTCTATCCTACATTCTCTCCCCTCTCC	24	<nnxnp>	nn	116	np	117			
>ref-144883	TTTATCCTCACCTGCCCTCCTTCCACA	6	<nnxnp>	nn	138	np	119			
>ref-144891	TATATTCAGACGGCAGCTCCTCCTGCA	4	<lmxll>	ll	130	lm	125			
>ref-144911	ACCACCACTACGACCACTCCTGAGCCT	1	<hkxhk>	hh	6	hk	263	kk	0	

>ref-144911	ACCACCACTACGACCACTCCTGAGCCT	6	<hkxhk>	hh	0	hk	229	kk	0
>ref-144911	ACCACCACTACGACCACTCCTGAGCCT	12	<hkxhk>	hh	0	hk	273	kk	0
>ref-144911	ACCACCACTACGACCACTCCTGAGCCT	21	<lmxll>	ll	10	lm	256		
>ref-144918	CCCGTTGCCACTTTCTCTCCGCTGGAG	21	<hkxhk>	hh	60	hk	149	kk	58
>ref-144920	GAGAGAAAAACACACTCTCCTCATTCT	23	<nnxnp>	nn	109	np	127		
>ref-144965	AGTCTCTCCACTCCGGCTCCATTTGCA	3	<lmxll>	ll	123	lm	136		
>ref-144978	TCCACGATCACATCAACTCCAACGACA	6	<lmxll>	ll	109	lm	141		
>ref-144978	TCCACGATCACATCAACTCCAACGACA	14	<lmxll>	ll	111	lm	141		
>ref-145019	GTTCCGGTTACTTTCTCTCCCTTTCTC	4	<lmxll>	ll	260	lm	0		
>ref-145049	GCTGCTGCTACTACTACTCCCATTGTT	1	<nnxnp>	nn	264	np	0		
>ref-145050	TGTGTCTGTACAATATCTCCCAAGCGA	1	<nnxnp>	nn	124	np	144		
>ref-145057	TCTCCTCTCACTTCACCTCCCAGTAGA	9	<nnxnp>	nn	140	np	128		
>ref-145078	ACATTCCCTACATCTACTCCTCCCTTC	26	<nnxnp>	nn	127	np	144		
>ref-145091	GACGCTGAAACAACGTCTCCCGCAATC	25	<nnxnp>	nn	121	np	149		
>ref-145099	CTGAGGCGTACCTGTCCTCCGCCGGCC	24	<lmxll>	ll	120	lm	149		
>ref-145125	TATTCTTCTACACTTTCTCCCTTCCAC	9	<lmxll>	ll	92	lm	137		
>ref-145137	CCGAGGATAACACCCCCTCCCTCGCCC	3	<nnxnp>	nn	129	np	120		
>ref-145145	CCTCCCTTCACTCCTTCTCCTTTCACC	6	<nnxnp>	nn	123	np	139		
>ref-145145	CCTCCCTTCACTCCTTCTCCTTTCACC	16	<lmxll>	ll	133	lm	134		
>ref-145145	CCTCCCTTCACTCCTTCTCCTTTCACC	22	<lmxll>	ll	170	lm	85		
>ref-145145	CCTCCCTTCACTCCTTCTCCTTTCACC	25	<lmxll>	ll	93	lm	161		
>ref-145159	ACCCTTCCCACCCAACCTCCTCCTTTT	1	<nnxnp>	nn	158	np	96		
>ref-145159	ACCCTTCCCACCCAACCTCCTCCTTTT	7	<lmxll>	ll	7	lm	261		
>ref-145159	ACCCTTCCCACCCAACCTCCTCCTTTT	25	<lmxll>	ll	7	lm	261		
>ref-145171	TCGTACATAACGCCATCTCCGCATGAA	3	<lmxll>	ll	154	lm	83		
>ref-145171	TCGTACATAACGCCATCTCCGCATGAA	8	<lmxll>	ll	153	lm	82		
>ref-145171	TCGTACATAACGCCATCTCCGCATGAA	13	<nnxnp>	nn	124	np	112		
>ref-145186	CCCGCGCCTACCGTGCCTCCACCAGGA	21	<lmxll>	ll	124	lm	147		
>ref-145214	TGCCGTTCTACCTTTCCTCCCTCATTC	4	<nnxnp>	nn	137	np	132		
>ref-145244	CGCCCACCAACAACAACTCCTTGGCTT	1	<lmxll>	ll	189	lm	77		
>ref-145244	CGCCCACCAACAACAACTCCTTGGCTT	2	<nnxnp>	nn	27	np	241		
>ref-145244	CGCCCACCAACAACAACTCCTTGGCTT	4	<nnxnp>	nn	27	np	241		
>ref-145244	CGCCCACCAACAACAACTCCTTGGCTT	7	<lmxll>	ll	189	lm	77		
>ref-145244	CGCCCACCAACAACAACTCCTTGGCTT	12	<lmxll>	ll	20	lm	248		
>ref-145244	CGCCCACCAACAACAACTCCTTGGCTT	15	<lmxll>	ll	20	lm	247		
>ref-145250	AGACAGTCAACCTTCACTCCAGATCCT	12	<lmxll>	ll	117	lm	128		
>ref-145259	AACGATTACACCGGCACTCCTATTTTG	9	<nnxnp>	nn	153	np	86		
>ref-145274	GTCGTTTACACGGAGTCTCCCAGCTGC	27	<nnxnp>	nn	246	np	1		

>ref-145305	CCAAGCAAGACACCAACTCCTTTCGAC	5	<hkxhk>	hh	0	hk	256	kk	8
>ref-145305	CCAAGCAAGACACCAACTCCTTTCGAC	9	<lmxll>	ll	8	lm	260		
>ref-145305	CCAAGCAAGACACCAACTCCTTTCGAC	24	<hkxhk>	hh	8	hk	256	kk	0
>ref-145325	ACCACAATTACAATACCTCCACAAGAC	22	<nnxnp>	nn	107	np	122		
>ref-145361	ACAGGGAACACCATATCTCCAAGCCAT	26	<hkxhk>	hh	66	hk	150	kk	49
>ref-145372	CCTAATCTCACCCTAACTCCTCTTGCA	14	<nnxnp>	nn	129	np	122		
>ref-145379	CACAAACACACTCTCTCTCCTTCTTAA	7	<nnxnp>	nn	120	np	149		
>ref-145396	CCACCCTCCACCCCCTCTCCACCCCCT	3	<lmxll>	ll	31	lm	189		
>ref-145396	CCACCCTCCACCCCCTCTCCACCCCCT	5	<lmxll>	ll	55	lm	164		
>ref-145397	CTCCCACCTACTTCTCCTCCCTCTTCC	2	<hkxhk>	hh	0	hk	272	kk	1
>ref-145397	CTCCCACCTACTTCTCCTCCCTCTTCC	6	<hkxhk>	hh	0	hk	259	kk	0
>ref-145426	CTCTTGCTCACCCTCTCTCCCTCTTGC	5	<lmxll>	ll	238	lm	16		
>ref-145426	CTCTTGCTCACCCTCTCTCCCTCTTGC	26	<lmxll>	ll	232	lm	5		
>ref-145442	TATGCATATACACTCACTCCCGCACAC	4	<nnxnp>	nn	123	np	121		
>ref-145447	CCCTTCCCCACCAACGCTCCCTAAATA	27	<lmxll>	ll	118	lm	128		
>ref-145454	CGCTGTTGCACTGACTCTCCGACGGAC	12	<lmxll>	ll	122	lm	133		
>ref-145470	TCGTATTCCACACTCTCTCCTTATGCT	15	<lmxll>	ll	110	lm	134		
>ref-145484	CAACAATTAACAATCGCTCCCACAATG	7	<hkxhk>	hh	0	hk	271	kk	0
>ref-145498	CAATCTCTCACTATCTCTCCCCATATT	21	<lmxll>	ll	115	lm	119		
>ref-145536	CACTCAAAAACGTATCCTCCTTTTGCC	25	<lmxll>	ll	147	lm	101		
>ref-145550	ATAGCAACGACGTGTCCTCCCGTCTCT	5	<nnxnp>	nn	270	np	0		
>ref-145559	CCCTCCCTAACTCTCACTCCCTTGTAG	5	<hkxhk>	hh	61	hk	130	kk	52
>ref-145568	CCCTCCCTAACCCTTCCTCCCCTTTAT	9	<lmxll>	ll	124	lm	127		
>ref-145569	TCCCCTCCCACTCCTCCTCCGCTAACT	4	<lmxll>	ll	136	lm	134		
>ref-145599	GGTCAACCGACGCATCCTCCTCCCCTG	2	<lmxll>	ll	139	lm	132		
>ref-145612	TAAATAGTCACATCGTCTCCTCAGTCT	13	<nnxnp>	nn	134	np	117		
>ref-145639	TCTCCCTCCACCTCCTCTCCCGCTTCT	4	<hkxhk>	hh	3	hk	260	kk	5
>ref-145639	TCTCCCTCCACCTCCTCTCCCGCTTCT	24	<hkxhk>	hh	3	hk	259	kk	5
>ref-145648	ATCCATCCCACAATCGCTCCCCGATGT	3	<lmxll>	ll	123	lm	131		
>ref-145675	CCCTACCCTACCATCCCTCCTTTATTT	24	<lmxll>	ll	138	lm	93		
>ref-145699	GCAAGGTCCACGGTTGCTCCCTGAATG	24	<hkxhk>	hh	0	hk	271	kk	1
>ref-145699	GCAAGGTCCACGGTTGCTCCCTGAATG	26	<hkxhk>	hh	0	hk	272	kk	0
>ref-145700	TTCACTGAAACCTTGCCTCCTTTCTCT	24	<nnxnp>	nn	132	np	130		
>ref-145711	CGTCTCCCCACCTTCCCTCCTTCGCTT	4	<nnxnp>	nn	260	np	0		
>ref-145711	CGTCTCCCCACCTTCCCTCCTTCGCTT	7	<lmxll>	ll	263	lm	0		
>ref-145731	CTCTCTGACACTGCCTCTCCATCTCTC	27	<hkxhk>	hh	63	hk	138	kk	56
>ref-145741	CTTTCTCCTACCTTTTCTCCTCCCCTT	4	<lmxll>	ll	139	lm	96		
>ref-145741	CTTTCTCCTACCTTTTCTCCTCCCCTT	9	<lmxll>	ll	134	lm	95		

>ref-145742	TATGAATCCACGGTAGCTCCAGATAAC	21	<lmxll>	ll	108	lm	121		
>ref-145771	CTTCCCTTTACTCCTTCTCCATTGCTC	21	<hkxhk>	hh	5	hk	263	kk	0
>ref-145771	CTTCCCTTTACTCCTTCTCCATTGCTC	24	<hkxhk>	hh	0	hk	263	kk	5
>ref-145775	AGCCTCCCTACGGATACTCCCCTCCTC	21	<lmxll>	ll	109	lm	142		
>ref-145787	CCTCCTCTCACCCCTTCTCCCATCTAT	13	<lmxll>	ll	115	lm	146		
>ref-145789	TATGGACGAACTCTTCCTCCCGTGCAC	2	<hkxhk>	hh	60	hk	139	kk	42
>ref-145797	AAACCCGAAACAAGAGCTCCGTCGGGG	26	<nnxnp>	nn	257	np	0		
>ref-145803	ACCACCACCACCCCTTCTCCCCGCCAC	2	<lmxll>	ll	114	lm	149		
>ref-145846	GGAAGTGGCACCACTCCTCCAGGTGGA	9	<hkxhk>	hh	30	hk	237	kk	0
>ref-145846	GGAAGTGGCACCACTCCTCCAGGTGGA	12	<hkxhk>	hh	0	hk	270	kk	0
>ref-145862	CCCCTCCCAACCCTAACTCCCTTTCCC	24	<nnxnp>	nn	111	np	118		
>ref-145865	ACTCCCCTCACTCTCTCTCCCCACTCC	8	<hkxhk>	hh	0	hk	255	kk	17
>ref-145867	AACTTAAAAACAGGTCCTCCACAGACC	23	<lmxll>	ll	181	lm	70		
>ref-145875	TCCCCACTCACTTTTCCTCCTCCTCCA	1	<lmxll>	ll	53	lm	179		
>ref-145875	TCCCCACTCACTTTTCCTCCTCCTCCA	7	<lmxll>	ll	55	lm	179		
>ref-145880	CCCATTTCCACTTCCTCTCCTTTCTCT	9	<hkxhk>	hh	69	hk	198	kk	0
>ref-145880	CCCATTTCCACTTCCTCTCCTTTCTCT	12	<hkxhk>	hh	0	hk	198	kk	69
>ref-145885	TCTCTGTGTACCCACACTCCAGCTCAA	2	<lmxll>	ll	94	lm	133		
>ref-145892	CCTTCATATACTTTCTCTCCTCCACCT	4	<lmxll>	ll	118	lm	137		
>ref-145910	CGGCCCCCAACACCAACTCCGGAAGAC	5	<lmxll>	ll	133	lm	110		
>ref-145915	ACCGCATCGACTGTTGCTCCTCGTTGT	14	<hkxhk>	hh	60	hk	138	kk	65
>ref-145915	ACCGCATCGACTGTTGCTCCTCGTTGT	15	<hkxhk>	hh	60	hk	138	kk	65
>ref-145922	AAAAGTGGAACATGTTCTCCACAAGCA	4	<hkxhk>	hh	49	hk	129	kk	70
>ref-145938	TCTCTCTGTACTTAGACTCCGTCTCTT	25	<nnxnp>	nn	143	np	119		
>ref-145940	TCCTCCGTTACTTTACCTCCTTTCCCT	9	<nnxnp>	nn	144	np	123		
>ref-145945	CTTGTCCTCACGCACTCTCCCCAACAC	24	<nnxnp>	nn	133	np	125		
>ref-145949	CCTGCCCCTACATCTGCTCCCAATTCT	6	<lmxll>	ll	6	lm	256		
>ref-145949	CCTGCCCCTACATCTGCTCCCAATTCT	12	<lmxll>	ll	6	lm	256		
>ref-145967	ATTATTTTAACATCCTCTCCTTCCCTC	25	<nnxnp>	nn	123	np	141		
>ref-145986	CACCCGACAACATCTTCTCCCCCTCTC	2	<lmxll>	ll	261	lm	0		
>ref-145986	CACCCGACAACATCTTCTCCCCCTCTC	9	<lmxll>	ll	259	lm	1		
>ref-145994	AAAAGGTAGACGCGCACTCCCGCCAAA	22	<lmxll>	ll	134	lm	120		
>ref-145995	GAAGGATATACAGTGACTCCAGATATA	1	<lmxll>	ll	109	lm	115		
>ref-146006	GATTCAAATACATCGTCTCCGAACGAT	14	<nnxnp>	nn	128	np	104		
>ref-146023	AAAGGCCTGACGCTCTCTCCCAGCGAC	8	<lmxll>	ll	138	lm	111		
>ref-146066	TACTTTGTCACTCCTTCTCCTTCCCTC	25	<lmxll>	ll	126	lm	144		
>ref-146066	TACTTTGTCACTCCTTCTCCTTCCCTC	27	<lmxll>	ll	126	lm	144		
>ref-146072	CTGCGTGTCACTTTGTCTCCAGGCATC	22	<nnxnp>	nn	134	np	136		

>ref-146081	CGCCCACGAACGCACACTCCAACAGAC	1	<nnxnp>	nn	114	np	152		
>ref-146081	CGCCCACGAACGCACACTCCAACAGAC	12	<nnxnp>	nn	272	np	0		
>ref-146081	CGCCCACGAACGCACACTCCAACAGAC	21	<nnxnp>	nn	112	np	153		
>ref-146089	TGCAACGCCACCTGCACTCCGGATGCG	26	<nnxnp>	nn	265	np	0		
>ref-146098	ATCTACCCAACTGTCCCTCCCTTCATC	8	<nnxnp>	nn	134	np	123		
>ref-146111	ATCTCCCCCACCCCTTCTCCTTCATAT	5	<lmxll>	ll	68	lm	197		
>ref-146120	ATTCATTCCACTCCCCCTCCCTTCCAC	4	<lmxll>	ll	112	lm	139		
>ref-146122	GTTTCCACAACTCACCCTCCAGAATCA	2	<lmxll>	ll	124	lm	114		
>ref-146123	CCGTTCGCCACTCACTCTCCCTTTCCC	5	<nnxnp>	nn	256	np	0		
>ref-146123	CCGTTCGCCACTCACTCTCCCTTTCCC	14	<nnxnp>	nn	252	np	0		
>ref-146131	CACACAGTCACAGAGACTCCGGAACAC	25	<hkxhk>	hh	48	hk	141	kk	66
>ref-146134	TAAGCCGTTACTTCAGCTCCCGAGTTG	8	<nnxnp>	nn	269	np	0		
>ref-146134	TAAGCCGTTACTTCAGCTCCCGAGTTG	9	<lmxll>	ll	265	lm	1		
>ref-146150	TCTACATCAACTCTTCCTCCCCCCTCA	22	<lmxll>	ll	116	lm	147		
>ref-146150	TCTACATCAACTCTTCCTCCCCCCTCA	24	<lmxll>	ll	138	lm	126		
>ref-146161	ATGGCAGTCACTATTACTCCATGTGCT	15	<hkxhk>	hh	59	hk	128	kk	77
>ref-146163	ACAACAACAACAACAGCTCCCAACAAC	1	<hkxhk>	hh	18	hk	238	kk	0
>ref-146196	CAGCCTCTCACCCTTCCTCCCTCACAT	3	<nnxnp>	nn	129	np	124		
>ref-146198	AATGTGATAACCTCGCCTCCTTTTCGT	22	<lmxll>	ll	119	lm	140		
>ref-146225	ATCCCCCATACCCCCCCTCCTTCCTTG	22	<lmxll>	ll	108	lm	129		
>ref-146230	GTAACTTACACAGTGCCTCCCTTATAA	1	<lmxll>	ll	144	lm	97		
>ref-146234	TTTCTTACAACTCTTCCTCCTTTCCTT	7	<hkxhk>	hh	2	hk	269	kk	0
>ref-146234	TTTCTTACAACTCTTCCTCCTTTCCTT	15	<hkxhk>	hh	0	hk	268	kk	2
>ref-146234	TTTCTTACAACTCTTCCTCCTTTCCTT	26	<nnxnp>	nn	135	np	134		
>ref-146234	TTTCTTACAACTCTTCCTCCTTTCCTT	27	<hkxhk>	hh	0	hk	269	kk	1
>ref-146252	TCCCCTTTTACCTCCTCTCCCTCTTTT	4	<lmxll>	ll	6	lm	259		
>ref-146252	TCCCCTTTTACCTCCTCTCCCTCTTTT	15	<lmxll>	ll	6	lm	259		
>ref-146277	ATTGTTCTTACCCTCTCTCCTCCTCTT	3	<nnxnp>	nn	110	np	143		
>ref-146277	ATTGTTCTTACCCTCTCTCCTCCTCTT	13	<nnxnp>	nn	111	np	143		
>ref-146295	CTAAACATTACTCTCCCTCCCTTCTTC	21	<lmxll>	ll	131	lm	128		
>ref-146296	GGTATACAAACCCTGCCTCCCCTATCA	2	<hkxhk>	hh	8	hk	252	kk	0
>ref-146296	GGTATACAAACCCTGCCTCCCCTATCA	15	<hkxhk>	hh	1	hk	252	kk	7
>ref-146296	GGTATACAAACCCTGCCTCCCCTATCA	24	<lmxll>	ll	131	lm	129		
>ref-146305	TTTTGCCTTACAGAGACTCCTAACGTG	8	<lmxll>	ll	131	lm	108		
>ref-146316	CTCTTTGACACAATTTCTCCTCGTTAC	24	<nnxnp>	nn	119	np	140		
>ref-146340	AGCTGCCCCACCCTCTCTCCCTCCTAC	24	<lmxll>	ll	272	lm	0		
>ref-146342	GCCTCATTCACGCCTCCTCCACCTGGT	22	<hkxhk>	hh	54	hk	160	kk	49
>ref-146343	TTCCTTTCGACTTTCCCTCCCCAGGTA	26	<lmxll>	ll	119	lm	137		

>ref-146377	TCTTTCTGTACATTTTCTCCTTCTATG	13	<lmxll>	ll	121	lm	117		
>ref-146383	AGTGCGTTGACGTCTGCTCCTACTTCG	23	<lmxll>	ll	28	lm	223		
>ref-146389	AATAACCCAACACCACCTCCATTGCCC	27	<lmxll>	ll	127	lm	138		
>ref-146390	GTATCAAGAACTTCCTCTCCCTCTTTC	1	<nnxnp>	nn	133	np	108		
>ref-146392	CTTTCTATAACCCTGCCTCCCTCCTCT	7	<nnxnp>	nn	272	np	0		
>ref-146392	CTTTCTATAACCCTGCCTCCCTCCTCT	15	<nnxnp>	nn	272	np	0		
>ref-146435	GACCGTGATACGTTCTCTCCTATGGTG	22	<lmxll>	ll	133	lm	129		
>ref-146436	CTACACTCCACCCTCGCTCCCCCCTTT	7	<lmxll>	ll	139	lm	117		
>ref-146441	AGCTCACCAACAACTACTCCAGGGGTG	3	<lmxll>	ll	133	lm	128		
>ref-146441	AGCTCACCAACAACTACTCCAGGGGTG	15	<lmxll>	ll	140	lm	125		
>ref-146490	AGCCAAAACACTGTCGCTCCACAGCCC	1	<lmxll>	ll	143	lm	116		
>ref-146490	AGCCAAAACACTGTCGCTCCACAGCCC	26	<lmxll>	ll	143	lm	116		
>ref-146491	CTTTACTCCACCCCTCCTCCATCTTTC	5	<hkxhk>	hh	9	hk	232	kk	4
>ref-146491	CTTTACTCCACCCCTCCTCCATCTTTC	9	<hkxhk>	hh	4	hk	234	kk	9
>ref-146492	TAAAAGCTCACCTTGACTCCACTTTCA	16	<nnxnp>	nn	242	np	0		
>ref-146499	GTTCCATCAACTCTCCCTCCTTCTCCA	6	<lmxll>	ll	134	lm	129		
>ref-146545	CTACCCTTAACTTCCCCTCCTCCACCT	2	<nnxnp>	nn	100	np	141		
>ref-146546	CCCCCCCATACACCTCCTCCTTCCCCA	9	<lmxll>	ll	268	lm	0		
>ref-146553	CTCCTGCTCACTTAGGCTCCTTTTCTT	15	<lmxll>	ll	136	lm	114		
>ref-146562	TATGCAGCAACACCCCCTCCCTCCTCA	22	<lmxll>	ll	7	lm	258		
>ref-146562	TATGCAGCAACACCCCCTCCCTCCTCA	26	<lmxll>	ll	13	lm	257		
>ref-146563	TCAATCGACACATTTCCTCCTTATTTA	7	<hkxhk>	hh	55	hk	126	kk	61
>ref-146577	ATCTCTTCCACCTCCCCTCCCCCCACT	6	<lmxll>	ll	145	lm	97		
>ref-146577	ATCTCTTCCACCTCCCCTCCCCCCACT	14	<lmxll>	ll	146	lm	97		
>ref-146577	ATCTCTTCCACCTCCCCTCCCCCCACT	16	<hkxhk>	hh	91	hk	157	kk	2
>ref-146577	ATCTCTTCCACCTCCCCTCCCCCCACT	22	<hkxhk>	hh	2	hk	157	kk	91
>ref-146634	TCCCCTTATACTCCTCCTCCCTTTTCT	8	<lmxll>	ll	2	lm	271		
>ref-146634	TCCCCTTATACTCCTCCTCCCTTTTCT	23	<lmxll>	ll	56	lm	210		
>ref-146636	CATATGTACACTGCTACTCCATTCCTC	4	<hkxhk>	hh	0	hk	271	kk	0
>ref-146642	ACATGCCTCACATTCGCTCCCTTATTT	27	<nnxnp>	nn	126	np	143		
>ref-146644	CCCCTCCCCACTCCTCCTCCGTTTCCC	6	<nnxnp>	nn	138	np	116		
>ref-146644	CCCCTCCCCACTCCTCCTCCGTTTCCC	15	<nnxnp>	nn	140	np	116		
>ref-146644	CCCCTCCCCACTCCTCCTCCGTTTCCC	23	<nnxnp>	nn	5	np	253		
>ref-146644	CCCCTCCCCACTCCTCCTCCGTTTCCC	27	<nnxnp>	nn	5	np	252		
>ref-146647	ATACCCTAAACCCCGCCTCCAACCCCC	2	<lmxll>	ll	103	lm	142		
>ref-146690	CTCACGCCAACAAAATCTCCCAACAGC	26	<nnxnp>	nn	116	np	108		
>ref-146723	CACTGGACCACACTCACTCCCGCACTA	13	<nnxnp>	nn	151	np	115		
>ref-146742	TCACCCAAAACCCCCTCTCCTCCTCCC	6	<lmxll>	ll	108	lm	113		

>ref-146762	ATACCCGAGACACCCACTCCTTCAGCT	3	<hkxhk>	hh	26	hk	224	kk	3
>ref-146762	ATACCCGAGACACCCACTCCTTCAGCT	5	<nnxnp>	nn	94	np	157		
>ref-146780	TACCCACTGACTGACTCTCCTACTCAC	2	<lmxll>	ll	125	lm	134		
>ref-146795	GAGAGAATTACGAACACTCCTGGTAAT	12	<nnxnp>	nn	116	np	126		
>ref-146795	GAGAGAATTACGAACACTCCTGGTAAT	24	<nnxnp>	nn	257	np	0		
>ref-146807	CCCTTCCCTACCCTTTCTCCTCGTCAC	25	<nnxnp>	nn	148	np	113		
>ref-146807	CCCTTCCCTACCCTTTCTCCTCGTCAC	26	<nnxnp>	nn	148	np	113		
>ref-146813	GGTTTAAAGACCTGGGCTCCACAAACA	3	<hkxhk>	hh	0	hk	258	kk	4
>ref-146836	GCATGGGACACGTGCTCTCCTGTATAC	12	<hkxhk>	hh	0	hk	269	kk	0
>ref-146842	CCCATCTCCACTTTCCCTCCTTCCTTC	2	<lmxll>	ll	247	lm	0		
>ref-146842	CCCATCTCCACTTTCCCTCCTTCCTTC	4	<lmxll>	ll	251	lm	1		
>ref-146843	TTCTACCCAACTCCTACTCCCTCCCCT	1	<lmxll>	ll	58	lm	164		
>ref-146843	TTCTACCCAACTCCTACTCCCTCCCCT	7	<lmxll>	ll	125	lm	122		
>ref-146875	CTGACGTGGACCTCTCCTCCAAGAGCC	16	<hkxhk>	hh	54	hk	133	kk	65
>ref-146897	ACCCCTGTGACCTCTGCTCCGGCCGAG	22	<hkxhk>	hh	0	hk	211	kk	36
>ref-146901	CTTCTTGCCACCCTCCCTCCGAATTCT	26	<hkxhk>	hh	41	hk	129	kk	56
>ref-146910	CATAAAAACACAAAGACTCCGCGCAAA	1	<nnxnp>	nn	147	np	97		
>ref-146912	TACCACTGCACCCTGTCTCCTCCCTCT	6	<nnxnp>	nn	134	np	130		
>ref-146914	CCGACGCCGACTCGTCCTCCTCATCTG	27	<nnxnp>	nn	127	np	140		
>ref-146926	GACCACCAAACCCACACTCCTCCCGCA	7	<lmxll>	ll	138	lm	124		
>ref-146934	CAATCAATAACTTGTTCTCCGGTCGAG	2	<nnxnp>	nn	253	np	0		
>ref-146938	GTCAAAGACACCCCTCCTCCTCCTTCT	25	<hkxhk>	hh	0	hk	271	kk	1
>ref-146960	GCCTCCTCCACACTCTCTCCTCCACAT	21	<lmxll>	ll	123	lm	138		
>ref-146975	GTTTGCTGAACGGAACCTCCCAGGTTT	3	<nnxnp>	nn	138	np	127		
>ref-146990	ATTGGTGCTACTTCATCTCCTTCTTCG	2	<lmxll>	ll	142	lm	111		
>ref-147023	TCGCCTCCCACGCCCCCTCCTAGCCTC	2	<nnxnp>	nn	161	np	92		
>ref-147023	TCGCCTCCCACGCCCCCTCCTAGCCTC	15	<nnxnp>	nn	162	np	92		
>ref-147036	ATCGGCAATACAAACTCTCCAACATAT	5	<hkxhk>	hh	60	hk	154	kk	48
>ref-147036	ATCGGCAATACAAACTCTCCAACATAT	26	<nnxnp>	nn	119	np	150		
>ref-147046	CTAATATGTACAACCTCTCCCCCTCCA	1	<nnxnp>	nn	134	np	129		
>ref-147051	TTCAGTCTAACACTGACTCCCTTTCAA	9	<nnxnp>	nn	132	np	127		
>ref-147072	GTAGATTGAACAGTGGCTCCTGCGTCT	24	<nnxnp>	nn	121	np	150		
>ref-147096	CCTCTTCACACCCTCCCTCCTCAACCA	3	<hkxhk>	hh	0	hk	271	kk	0
>ref-147096	CCTCTTCACACCCTCCCTCCTCAACCA	23	<hkxhk>	hh	0	hk	271	kk	0
>ref-147140	CCCTCCACGACCCTTTCTCCGCGTCCG	22	<lmxll>	ll	126	lm	138		
>ref-147150	CTTCCTCAAACCTTCACTCCTTCTCCC	4	<nnxnp>	nn	268	np	0		
>ref-147156	AGGACATACACACATCCTCCTCCTCCT	26	<lmxll>	ll	135	lm	132		
>ref-147167	CCACATGAAACATTTACTCCAGCGTCA	6	<hkxhk>	hh	58	hk	155	kk	53

>ref-147172	AATATTTGGACAAGAACTCCATATCAT	2	<hkxhk>	hh	19	hk	241	kk	0
>ref-147172	AATATTTGGACAAGAACTCCATATCAT	26	<hkxhk>	hh	8	hk	261	kk	0
>ref-147176	TCTATCCACACCCCTCCTCCTCACCCC	3	<lmxll>	ll	162	lm	92		
>ref-147176	TCTATCCACACCCCTCCTCCTCACCCC	4	<lmxll>	ll	162	lm	92		
>ref-147188	ACTTCAAGCACAAGCTCTCCACTTCGT	12	<lmxll>	ll	259	lm	0		
>ref-147192	CGTATATCCACAAACTCTCCTTCCATG	4	<lmxll>	ll	270	lm	0		
>ref-147226	ATCTTTCTTACCCTTCCTCCCTCCACC	8	<nnxnp>	nn	233	np	0		
>ref-147226	ATCTTTCTTACCCTTCCTCCCTCCACC	9	<lmxll>	ll	234	lm	0		
>ref-147226	ATCTTTCTTACCCTTCCTCCCTCCACC	25	<nnxnp>	nn	221	np	1		
>ref-147226	ATCTTTCTTACCCTTCCTCCCTCCACC	26	<lmxll>	ll	230	lm	3		
>ref-147231	GGCCTTTCTACTTCCCCTCCTCTTCTC	7	<hkxhk>	hh	65	hk	135	kk	65
>ref-147237	AGGACCCTCACTTCGCCTCCAGTCGCC	1	<hkxhk>	hh	0	hk	246	kk	12
>ref-147241	CCCTTTTTAACATTCGCTCCTCACATT	23	<nnxnp>	nn	109	np	153		
>ref-147248	CTTCTTTTCACTTTCTCTCCTCCTCTC	2	<lmxll>	ll	54	lm	202		
>ref-147248	CTTCTTTTCACTTTCTCTCCTCCTCTC	5	<lmxll>	ll	52	lm	202		
>ref-147248	CTTCTTTTCACTTTCTCTCCTCCTCTC	8	<lmxll>	ll	120	lm	140		
>ref-147248	CTTCTTTTCACTTTCTCTCCTCCTCTC	22	<lmxll>	ll	116	lm	140		
>ref-147256	TTGTACAAGACGGGTCCTCCTCGTGTG	26	<lmxll>	ll	133	lm	137		
>ref-147283	CACACAACGACCCTTCCTCCCACGTCC	27	<nnxnp>	nn	125	np	144		
>ref-147296	TCCTCCACCACTTTCTCTCCTTCGGTT	5	<hkxhk>	hh	53	hk	162	kk	40
>ref-147303	CCAACATCTACTGCTACTCCTGCCATC	23	<nnxnp>	nn	225	np	0		
>ref-147305	TTTATTTTCACTCCTTCTCCCTCTCCA	13	<hkxhk>	hh	0	hk	256	kk	5
>ref-147305	TTTATTTTCACTCCTTCTCCCTCTCCA	27	<hkxhk>	hh	0	hk	255	kk	5
>ref-147310	TTTCTCGTCACTTCCGCTCCTCTTCCC	7	<nnxnp>	nn	267	np	0		
>ref-147310	TTTCTCGTCACTTCCGCTCCTCTTCCC	16	<nnxnp>	nn	269	np	0		
>ref-147312	CACGCCATAACATACTCTCCTCCAAAA	15	<lmxll>	ll	114	lm	122		
>ref-147334	TTCGCAACCACTCTCTCTCCTTCTCCC	21	<hkxhk>	hh	54	hk	153	kk	55
>ref-147354	GGTGACAAAACATTATCTCCACAAAGT	4	<lmxll>	ll	112	lm	113		
>ref-147356	GCGGTTTACACAAGGGCTCCCTCTCAT	8	<nnxnp>	nn	126	np	140		
>ref-147356	GCGGTTTACACAAGGGCTCCCTCTCAT	9	<nnxnp>	nn	127	np	140		
>ref-147356	GCGGTTTACACAAGGGCTCCCTCTCAT	26	<hkxhk>	hh	61	hk	137	kk	64
>ref-147368	CTTCTGCCTACCCTTCCTCCTCTCATT	22	<nnxnp>	nn	17	np	250		
>ref-147400	TTCTCTTCCACCCCTCCTCCTCTCTTT	13	<lmxll>	ll	55	lm	184		
>ref-147415	CCCAGTGACACCAAGCCTCCAGCACGA	14	<hkxhk>	hh	65	hk	126	kk	56
>ref-147417	AGTAATCTCACTCGATCTCCTGCTCCT	12	<lmxll>	ll	152	lm	119		
>ref-147457	CCTCACTATACCCCCTCTCCCGTTCCT	21	<nnxnp>	nn	135	np	128		
>ref-147459	AACCTTTACACTCCCCCTCCCTTCCCC	1	<hkxhk>	hh	111	hk	130	kk	0
>ref-147459	AACCTTTACACTCCCCCTCCCTTCCCC	3	<hkxhk>	hh	112	hk	129	kk	0

>ref-147465	CCTCCCTTTACTTTCCCTCCTTTCCTC	5	<nnxnp>	nn	6	np	267		
>ref-147465	CCTCCCTTTACTTTCCCTCCTTTCCTC	14	<nnxnp>	nn	5	np	268		
>ref-147467	TCTTTATCCACACCGTCTCCTCGGGTA	5	<lmxll>	ll	135	lm	132		
>ref-147497	TCTCCCCACACTCTCACTCCCCTTCCC	3	<nnxnp>	nn	181	np	80		
>ref-147497	TCTCCCCACACTCTCACTCCCCTTCCC	26	<nnxnp>	nn	183	np	81		
>ref-147512	TATTTTCTTACCTTGCCTCCCTTTCCC	24	<lmxll>	ll	149	lm	116		
>ref-147512	TATTTTCTTACCTTGCCTCCCTTTCCC	25	<lmxll>	ll	149	lm	116		
>ref-147517	GCGGCTCGAACGTCCTCTCCCTCTCTC	27	<lmxll>	ll	123	lm	144		
>ref-147523	ATTGCCCATACCTCCCCTCCCCTCCTA	6	<lmxll>	ll	247	lm	0		
>ref-147550	TTAACGTCCACTGGTTCTCCCTTTAGA	6	<lmxll>	ll	122	lm	131		
>ref-147621	TCTCGCCTCACTGTGGCTCCCGGCATC	4	<nnxnp>	nn	266	np	0		
>ref-147623	TCCGTGCCCACTTTCCCTCCTTCCCTC	23	<lmxll>	ll	146	lm	122		
>ref-147632	GACACAGCAACAATACCTCCCACTGTC	3	<nnxnp>	nn	134	np	131		
>ref-147651	TTAATATTTACTTCCTCTCCCTCTCCC	5	<lmxll>	ll	61	lm	176		
>ref-147651	TTAATATTTACTTCCTCTCCCTCTCCC	23	<lmxll>	ll	61	lm	177		
>ref-147683	TTTGAGTCAACGCCTCCTCCCCGTCCG	14	<nnxnp>	nn	271	np	0		
>ref-147690	CCTCAGTATACGCGCCCTCCGATAAGT	9	<nnxnp>	nn	268	np	0		
>ref-147699	GTTATCCTTACGTGCGCTCCAAGGGCG	6	<nnxnp>	nn	124	np	145		
>ref-147701	ATCAAGCCAACTGCATCTCCCACAACT	21	<nnxnp>	nn	89	np	151		
>ref-147741	AGCATTATCACGGCAGCTCCGGTCGAC	12	<hkxhk>	hh	56	hk	143	kk	69
>ref-147747	CTCATCCTAACCCCTTCTCCTATCCCA	1	<hkxhk>	hh	64	hk	199	kk	0
>ref-147747	CTCATCCTAACCCCTTCTCCTATCCCA	4	<hkxhk>	hh	0	hk	267	kk	0
>ref-147747	CTCATCCTAACCCCTTCTCCTATCCCA	8	<nnxnp>	nn	74	np	180		
>ref-147757	ACATCATCAACGCCATCTCCACCACAT	25	<lmxll>	ll	134	lm	132		
>ref-147763	CAGACAGACACAATCACTCCAAACCCC	16	<nnxnp>	nn	148	np	123		
>ref-147769	TCCTTCTTCACTTTCCCTCCGTGATTG	15	<lmxll>	ll	122	lm	140		
>ref-147770	CTCCCTCTCACCACCACTCCTCTCCTC	2	<hkxhk>	hh	0	hk	242	kk	25
>ref-147770	CTCCCTCTCACCACCACTCCTCTCCTC	13	<hkxhk>	hh	21	hk	246	kk	0
>ref-147775	CATGCCTTCACATTCCCTCCTCACCTC	25	<nnxnp>	nn	114	np	153		
>ref-147787	GCGTCTCAAACACCTTCTCCTCCGTTC	6	<nnxnp>	nn	264	np	0		
>ref-147797	AACAACTACACGTTTTCTCCCACAATA	4	<nnxnp>	nn	106	np	153		
>ref-147800	TGTTACATAACACTGGCTCCTTCCACT	24	<nnxnp>	nn	136	np	124		
>ref-147811	AGCCAGCATACTCTTTCTCCCTCCACA	26	<lmxll>	ll	132	lm	118		
>ref-147850	GCATCTCCCACCCTCACTCCCTCCCTC	1	<nnxnp>	nn	15	np	232		
>ref-147878	TACACCACCACCAAGTCTCCTCTTTCT	3	<hkxhk>	hh	82	hk	175	kk	0
>ref-147878	TACACCACCACCAAGTCTCCTCTTTCT	9	<hkxhk>	hh	22	hk	242	kk	0
>ref-147878	TACACCACCACCAAGTCTCCTCTTTCT	15	<nnxnp>	nn	139	np	99		
>ref-147910	CCCACTTCCACCTTCCCTCCCAGCTTC	23	<lmxll>	ll	194	lm	51		

>ref-147910	CCCACTTCCACCTTCCCTCCCAGCTTC	26	<lmxll>	ll	203	lm	45		
>ref-147915	ATCGCCATCACCGTTTCTCCTCCGCTT	6	<hkxhk>	hh	122	hk	146	kk	0
>ref-147916	CACTGTACCACGTCGTCTCCCCTCCCC	5	<lmxll>	ll	117	lm	137		
>ref-147916	CACTGTACCACGTCGTCTCCCCTCCCC	13	<hkxhk>	hh	61	hk	135	kk	52
>ref-147924	CGACCCTTCACTTCCCCTCCCCCTTTC	5	<nnxnp>	nn	272	np	0		
>ref-147931	TCTTCCTCCACTTCTACTCCACCCAAT	7	<nnxnp>	nn	119	np	146		
>ref-147931	TCTTCCTCCACTTCTACTCCACCCAAT	25	<lmxll>	ll	119	lm	135		
>ref-147944	CACCCATACACACAAACTCCATTTCGA	25	<lmxll>	ll	140	lm	116		
>ref-147965	ACCTCCACCACCTCCTCTCCTATCACC	2	<hkxhk>	hh	0	hk	273	kk	0
>ref-147965	ACCTCCACCACCTCCTCTCCTATCACC	5	<nnxnp>	nn	142	np	123		
>ref-147965	ACCTCCACCACCTCCTCTCCTATCACC	22	<hkxhk>	hh	0	hk	273	kk	0
>ref-147965	ACCTCCACCACCTCCTCTCCTATCACC	23	<hkxhk>	hh	0	hk	265	kk	5
>ref-147974	TCACGATTCACAGCCGCTCCTCCAGGG	23	<lmxll>	ll	145	lm	125		
>ref-148005	CCACACCACACAACCTCTCCACCACAC	1	<hkxhk>	hh	79	hk	185	kk	0
>ref-148005	CCACACCACACAACCTCTCCACCACAC	2	<hkxhk>	hh	77	hk	184	kk	0
>ref-148005	CCACACCACACAACCTCTCCACCACAC	7	<hkxhk>	hh	0	hk	273	kk	0
>ref-148006	ATTGAATCAACCTGGTCTCCCTCCTCT	3	<hkxhk>	hh	0	hk	195	kk	74
>ref-148006	ATTGAATCAACCTGGTCTCCCTCCTCT	16	<hkxhk>	hh	0	hk	265	kk	4
>ref-148015	TACTCTAACACACACGCTCCCCCGCAC	5	<nnxnp>	nn	96	np	123		
>ref-148048	GGGAGGTATACAATTTCTCCCTCTAAT	5	<hkxhk>	hh	6	hk	222	kk	2
>ref-148065	ATCAGCCTGACATACCCTCCTGCTTGA	1	<lmxll>	ll	96	lm	150		
>ref-148065	ATCAGCCTGACATACCCTCCTGCTTGA	2	<lmxll>	ll	96	lm	150		
>ref-148073	ATCTTTTCCACCCTCCCTCCTCCTTTC	5	<lmxll>	ll	223	lm	3		
>ref-148073	ATCTTTTCCACCCTCCCTCCTCCTTTC	25	<lmxll>	ll	226	lm	3		
>ref-148094	CTGCCACCCACCTTGCCTCCCACCCAC	3	<hkxhk>	hh	6	hk	229	kk	9
>ref-148094	CTGCCACCCACCTTGCCTCCCACCCAC	9	<hkxhk>	hh	0	hk	138	kk	95
>ref-148094	CTGCCACCCACCTTGCCTCCCACCCAC	21	<hkxhk>	hh	11	hk	227	kk	6
>ref-148095	TCAGTTTCGACATCTCCTCCTTCCACC	25	<hkxhk>	hh	55	hk	152	kk	52
>ref-148097	CATACGAACACACACACTCCCGCTTCT	9	<lmxll>	ll	140	lm	127		
>ref-148100	CACCCCCATACCCGCCCTCCTTCTTCC	21	<lmxll>	ll	13	lm	223		
>ref-148105	CAACATCCGACACGAACTCCCTTCCCG	26	<nnxnp>	nn	262	np	1		
>ref-148106	CTCTCTTTCACTCTGTCTCCAGTAGGT	14	<hkxhk>	hh	60	hk	128	kk	57
>ref-148128	ACTTGGCACACAGCAGCTCCTTCGTCA	16	<nnxnp>	nn	126	np	136		
>ref-148168	CTTCTCCCTACTCTTACTCCCTCCCCT	2	<lmxll>	ll	22	lm	228		
>ref-148168	CTTCTCCCTACTCTTACTCCCTCCCCT	12	<nnxnp>	nn	11	np	251		
>ref-148168	CTTCTCCCTACTCTTACTCCCTCCCCT	13	<lmxll>	ll	22	lm	228		
>ref-148168	CTTCTCCCTACTCTTACTCCCTCCCCT	16	<nnxnp>	nn	11	np	252		
>ref-148226	TCCCCTTAAACCCTCCCTCCTTCCTCC	14	<nnxnp>	nn	126	np	130		

>ref-148242	TTCTCGAGGACGGCGACTCCCGGCCT	2	<nnxnp>	nn	115	np	134		
>ref-148253	TTTCATTCCACTTCCCCTCCTTTCTCT	14	<hkxhk>	hh	0	hk	214	kk	37
>ref-148253	TTTCATTCCACTTCCCCTCCTTTCTCT	16	<hkxhk>	hh	0	hk	214	kk	37
>ref-148255	ACGCGGGCGACAGTCACTCCTAATTTA	9	<hkxhk>	hh	0	hk	184	kk	67
>ref-148291	TTGTTTATAACGCCTGCTCCAAAATCA	2	<lmxll>	ll	112	lm	147		
>ref-148316	AGGTGTGAAACGTTCCCTCCGGTCTTG	14	<lmxll>	ll	7	lm	265		
>ref-148316	AGGTGTGAAACGTTCCCTCCGGTCTTG	23	<lmxll>	ll	7	lm	265		
>ref-148321	ATATCTACAACCAAGACTCCAACACAC	1	<nnxnp>	nn	127	np	131		
>ref-148321	ATATCTACAACCAAGACTCCAACACAC	2	<nnxnp>	nn	127	np	131		
>ref-148327	TTGGGTCTGACTCTTCCTCCTCCTATT	5	<nnxnp>	nn	136	np	119		
>ref-148338	CATTGTGAAACAGGTGCTCCAAAGAGG	14	<nnxnp>	nn	253	np	0		
>ref-148384	TATCCCTTTACTCTCACTCCTCACATT	25	<nnxnp>	nn	126	np	140		
>ref-148387	AGGCAATCAACCAGCACTCCCCAGTGT	25	<lmxll>	ll	146	lm	119		
>ref-148404	AATGAACGAACTCGATCTCCCTTTTCC	8	<hkxhk>	hh	53	hk	145	kk	55
>ref-148416	CTTCCTCCAACTTCGCCTCCCCTCTCT	6	<hkxhk>	hh	0	hk	148	kk	104
>ref-148416	CTTCCTCCAACTTCGCCTCCCCTCTCT	15	<hkxhk>	hh	0	hk	153	kk	101
>ref-148461	GACGTACATACACATGCTCCTCTGGAA	1	<lmxll>	ll	123	lm	143		
>ref-148467	CTGTCTCACACATCCTCTCCTTCTCCC	4	<lmxll>	ll	136	lm	135		
>ref-148474	CCTCTCCCTACCCCCTCTCCTGTTCTA	3	<lmxll>	ll	37	lm	194		
>ref-148474	CCTCTCCCTACCCCCTCTCCTGTTCTA	22	<lmxll>	ll	37	lm	195		
>ref-148498	CAAAAACACACACCACCTCCATCGGCT	14	<lmxll>	ll	107	lm	161		
>ref-148507	CACCGGGACACAGTTCCTCCTCCGCTG	13	<hkxhk>	hh	0	hk	273	kk	0
>ref-148543	TCAACACAGACTCGACCTCCCCATCAC	15	<nnxnp>	nn	264	np	0		
>ref-148545	AGAGATTAGACCTTTCCTCCTCCAAGG	1	<lmxll>	ll	140	lm	112		
>ref-148545	AGAGATTAGACCTTTCCTCCTCCAAGG	4	<hkxhk>	hh	1	hk	227	kk	26
>ref-148545	AGAGATTAGACCTTTCCTCCTCCAAGG	22	<hkxhk>	hh	5	hk	252	kk	1
>ref-148546	CTCGCTATCACCCTTCCTCCCTTAATC	3	<hkxhk>	hh	77	hk	183	kk	0
>ref-148546	CTCGCTATCACCCTTCCTCCCTTAATC	4	<hkxhk>	hh	0	hk	184	kk	78
>ref-148546	CTCGCTATCACCCTTCCTCCCTTAATC	24	<hkxhk>	hh	1	hk	232	kk	30
>ref-148547	GAGCTACTTACAGGACCTCCAATGTCT	7	<hkxhk>	hh	0	hk	271	kk	0
>ref-148547	GAGCTACTTACAGGACCTCCAATGTCT	22	<lmxll>	ll	127	lm	144		
>ref-148549	ATCCCCATCACCACCCCTCCCCCTCTC	2	<nnxnp>	nn	217	np	28		
>ref-148549	ATCCCCATCACCACCCCTCCCCCTCTC	13	<nnxnp>	nn	146	np	77		
>ref-148554	TTCATTCTAACCCTCCCTCCTCTCCTG	8	<lmxll>	ll	104	lm	125		
>ref-148559	CGAGGTTTTACTGGCTCTCCGGTTTCA	7	<hkxhk>	hh	0	hk	220	kk	37
>ref-148559	CGAGGTTTTACTGGCTCTCCGGTTTCA	8	<hkxhk>	hh	0	hk	219	kk	38
>ref-148559	CGAGGTTTTACTGGCTCTCCGGTTTCA	16	<hkxhk>	hh	0	hk	273	kk	0
>ref-148559	CGAGGTTTTACTGGCTCTCCGGTTTCA	24	<hkxhk>	hh	0	hk	273	kk	0

>ref-148573	TCCTCCCCTACTCCCGCTCCCCATGCA	8	<lmxll>	ll	127	lm	132		
>ref-148575	TAAAGATTCACTTACGCTCCTAATCTA	27	<hkxhk>	hh	68	hk	129	kk	61
>ref-148593	TGCCATTGGACGACTTCTCCCTGGGTC	14	<nnxnp>	nn	130	np	136		
>ref-148593	TGCCATTGGACGACTTCTCCCTGGGTC	16	<nnxnp>	nn	130	np	136		
>ref-148596	CCCATCTACACTCTCCCTCCTTTCTCC	3	<nnxnp>	nn	233	np	0		
>ref-148596	CCCATCTACACTCTCCCTCCTTTCTCC	8	<nnxnp>	nn	227	np	2		
>ref-148600	AGGAGGAAGACATGGCCTCCGCTCGGC	21	<lmxll>	ll	266	lm	0		
>ref-148601	TTGAGGTTTACATATTCTCCATAGTCT	24	<nnxnp>	nn	263	np	0		
>ref-148611	CTTCTCTGAACCCCCTCTCCTTTCCTC	6	<nnxnp>	nn	237	np	0		
>ref-148617	GCTGCTACTACTACCACTCCTGCCCCT	4	<nnxnp>	nn	133	np	131		
>ref-148646	TATCATAGTACTGCTCCTCCATCAAAT	5	<lmxll>	ll	107	lm	115		
>ref-148664	GAACGCACAACCCGTTCTCCGTCACCC	13	<nnxnp>	nn	133	np	136		
>ref-148680	TCAGTTCTGACACTCCCTCCCGCTCCG	6	<nnxnp>	nn	124	np	133		
>ref-148681	CTCTCTCTCACCCTCTCTCCTTTTAAG	3	<nnxnp>	nn	114	np	129		
>ref-148707	TTTCATAACACTTATTCTCCCTTACAA	1	<nnxnp>	nn	103	np	120		
>ref-148722	GGACCTGAAACAACCACTCCTTCAGCA	2	<hkxhk>	hh	0	hk	272	kk	1
>ref-148722	GGACCTGAAACAACCACTCCTTCAGCA	7	<hkxhk>	hh	0	hk	272	kk	1
>ref-148752	ATAACAATCACCCTTCCTCCTTGACCG	9	<lmxll>	ll	121	lm	134		
>ref-148767	CTCTAGCTTACTCTCTCTCCCTACCCT	5	<lmxll>	ll	13	lm	240		
>ref-148813	CTAAGCTGAACCCCATCTCCGGATTCC	13	<nnxnp>	nn	119	np	124		
>ref-148832	TCTAACACGACCTGCCCTCCCGCAGAT	12	<nnxnp>	nn	123	np	144		
>ref-148833	CCCGAGCCCACACTTTCTCCTCTCGCC	1	<nnxnp>	nn	118	np	142		
>ref-148847	TTTCCTTCGACCTTCTCTCCCCCTTTC	6	<lmxll>	ll	126	lm	106		
>ref-148948	ATGCCTCACACACTCCCTCCAATGGCC	12	<nnxnp>	nn	265	np	0		
>ref-148952	ACCCTTGTGACCGATCCTCCTCCTCCT	1	<hkxhk>	hh	52	hk	135	kk	78
>ref-148952	ACCCTTGTGACCGATCCTCCTCCTCCT	27	<lmxll>	ll	237	lm	5		
>ref-148957	TTAATTTGCACGGAACCTCCCTTGCAA	9	<lmxll>	ll	108	lm	118		
>ref-148958	TGTTGTAACACTACCTCTCCTCACCAC	5	<nnxnp>	nn	120	np	146		
>ref-148967	CCCTTTTCTACTCCCACTCCTCCTACT	1	<hkxhk>	hh	0	hk	271	kk	0
>ref-148967	CCCTTTTCTACTCCCACTCCTCCTACT	9	<hkxhk>	hh	0	hk	271	kk	0
>ref-148967	CCCTTTTCTACTCCCACTCCTCCTACT	24	<nnxnp>	nn	113	np	157		
>ref-148975	CCTCTAACGACCATTCCTCCTCCTCTA	16	<hkxhk>	hh	0	hk	271	kk	1
>ref-148975	CCTCTAACGACCATTCCTCCTCCTCTA	24	<hkxhk>	hh	0	hk	271	kk	1
>ref-148978	TTGTCCATCACTTTCTCTCCCTCTTCT	16	<nnxnp>	nn	123	np	133		
>ref-149008	GAGCCGCTCACTCTGCCTCCTTCCGCC	6	<lmxll>	ll	267	lm	0		
>ref-149038	TTTCTCTCCACCGTCTCTCCTCTTTAC	3	<nnxnp>	nn	113	np	121		
>ref-149074	CCTCCCGTGACTCATCCTCCGCGTCAA	6	<lmxll>	ll	153	lm	112		
>ref-149078	CCCTTCGACACTCCCCCTCCCTGTGTT	26	<lmxll>	ll	92	lm	163		

>ref-149083	AAATCTGCCACGCCCACTCCGAACCGT	2	<hkxhk>	hh	44	hk	121	kk	63
>ref-149083	AAATCTGCCACGCCCACTCCGAACCGT	3	<hkxhk>	hh	44	hk	121	kk	63
>ref-149090	CTGATAATCACTTTATCTCCAACGGCA	1	<nnxnp>	nn	117	np	142		
>ref-149097	GATGTTTAGACTTCGGCTCCAATGCTA	8	<hkxhk>	hh	64	hk	148	kk	58
>ref-149176	TCCTTGTTTACATTTCCTCCTTTTTCT	5	<nnxnp>	nn	153	np	114		
>ref-149176	TCCTTGTTTACATTTCCTCCTTTTTCT	7	<nnxnp>	nn	153	np	114		
>ref-149178	CCTACCGCCACTTCCCCTCCTTCCCTG	7	<nnxnp>	nn	143	np	124		
>ref-149180	TTCCCCTTTACCCATCCTCCCCTTCCA	4	<hkxhk>	hh	53	hk	138	kk	57
>ref-149180	TTCCCCTTTACCCATCCTCCCCTTCCA	5	<lmxll>	ll	116	lm	140		
>ref-149195	AAAACAACAACTACAACTCCTCCCAGT	9	<hkxhk>	hh	48	hk	123	kk	56
>ref-149203	TCTTCTTCTACATCCCCTCCCTTGTTC	2	<hkxhk>	hh	59	hk	142	kk	55
>ref-149207	CCGCTCTCCACCATCTCTCCCTCTCCA	3	<lmxll>	ll	132	lm	126		
>ref-149218	ACCGTTTGTACCTGCCCTCCTCCTATC	14	<nnxnp>	nn	114	np	140		
>ref-149246	CCGCGCCCAACCTTGGCTCCACCTCCT	15	<hkxhk>	hh	0	hk	271	kk	0
>ref-149246	CCGCGCCCAACCTTGGCTCCACCTCCT	16	<hkxhk>	hh	0	hk	271	kk	0
>ref-149267	CTCTTCCCTACACCTCCTCCTCTCCTC	16	<lmxll>	ll	125	lm	140		
>ref-149272	TAAGGTCCCACCCCCCCTCCTCCCACG	27	<nnxnp>	nn	114	np	108		
>ref-149286	CTTATCACCACAGTATCTCCTGGATGT	7	<lmxll>	ll	120	lm	124		
>ref-149302	CAAATAGTAACCGTATCTCCTCATGGT	12	<lmxll>	ll	132	lm	112		
>ref-149359	CTCTATCTTACCCTCTCTCCCCCTTCT	24	<lmxll>	ll	147	lm	104		
>ref-149361	ATCTCCCTTACGTATCCTCCACCCTTT	15	<hkxhk>	hh	63	hk	142	kk	61
>ref-149423	TATAAACACACACACACTCCAACAGAA	4	<nnxnp>	nn	111	np	117		
>ref-149431	CTTAGACATACATATTCTCCATTTGAT	24	<nnxnp>	nn	109	np	117		
>ref-149433	GTCCTCCTCACTGAGGCTCCCCTCAAC	21	<hkxhk>	hh	2	hk	270	kk	0
>ref-149504	GCTGTCACCACTCGCACTCCGCTCTCT	1	<nnxnp>	nn	2	np	268		
>ref-149505	TCCCCTGCCACACAAGCTCCGCGCGGG	22	<lmxll>	ll	135	lm	135		
>ref-149508	GGTTAACTTACCTGCTCTCCATGTGCT	6	<nnxnp>	nn	230	np	5		
>ref-149508	GGTTAACTTACCTGCTCTCCATGTGCT	15	<lmxll>	ll	249	lm	1		
>ref-149508	GGTTAACTTACCTGCTCTCCATGTGCT	26	<lmxll>	ll	67	lm	177		
>ref-149516	ATCTGTCCAACCCCAACTCCCACTCTC	25	<lmxll>	ll	135	lm	124		
>ref-149525	TGAGAAATGACAGTAGCTCCTGACCTG	3	<nnxnp>	nn	124	np	134		
>ref-149544	AGATTTTCCACGGGTTCTCCACTGCAT	1	<hkxhk>	hh	52	hk	131	kk	62
>ref-149562	CCACCTGTCACACTCTCTCCATCCTCC	26	<lmxll>	ll	127	lm	144		
>ref-149575	TTTCCCTCCACTTTCCCTCCCCTAATT	3	<hkxhk>	hh	0	hk	263	kk	4
>ref-149575	TTTCCCTCCACTTTCCCTCCCCTAATT	5	<hkxhk>	hh	25	hk	234	kk	0
>ref-149575	TTTCCCTCCACTTTCCCTCCCCTAATT	9	<hkxhk>	hh	0	hk	263	kk	4
>ref-149575	TTTCCCTCCACTTTCCCTCCCCTAATT	23	<hkxhk>	hh	0	hk	234	kk	25
>ref-149575	TTTCCCTCCACTTTCCCTCCCCTAATT	25	<hkxhk>	hh	59	hk	185	kk	0

>ref-149584	TATTGCACGACACATCCTCCCTTACAC	7	<nnxnp>	nn	119	np	152		
>ref-149586	GTTATTATTACATTTACTCCATTTGTT	9	<nnxnp>	nn	127	np	105		
>ref-149586	GTTATTATTACATTTACTCCATTTGTT	14	<lmxll>	ll	99	lm	130		
>ref-149586	GTTATTATTACATTTACTCCATTTGTT	21	<hkxhk>	hh	2	hk	264	kk	0
>ref-149586	GTTATTATTACATTTACTCCATTTGTT	23	<lmxll>	ll	13	lm	238		
>ref-149589	TTTAGAGTCACCTGCTCTCCTTTCCGC	25	<lmxll>	ll	130	lm	90		
>ref-149589	TTTAGAGTCACCTGCTCTCCTTTCCGC	26	<lmxll>	ll	130	lm	89		
>ref-149598	TTTTCTTTCACCCTCTCTCCCTTCACA	26	<nnxnp>	nn	129	np	113		
>ref-149621	CAGGAACATACAGGCACTCCATCATAC	2	<nnxnp>	nn	129	np	138		
>ref-149633	GCACTTTCTACTGTCACTCCTCACGCT	9	<hkxhk>	hh	58	hk	141	kk	60
>ref-149634	CGCCTCCTTACTCCCCCTCCCTCTCTC	21	<nnxnp>	nn	268	np	1		
>ref-149635	CGCTGCCCGACACCAGCTCCCTCTCCC	5	<lmxll>	ll	136	lm	128		
>ref-149635	CGCTGCCCGACACCAGCTCCCTCTCCC	15	<nnxnp>	nn	124	np	137		
>ref-149647	ATCCTCTATACAAACCCTCCTCTTCCT	8	<hkxhk>	hh	37	hk	146	kk	76
>ref-149652	AATGGCGCCACAATTTCTCCCGCCGCG	4	<lmxll>	ll	135	lm	126		
>ref-149652	AATGGCGCCACAATTTCTCCCGCCGCG	6	<lmxll>	ll	136	lm	125		
>ref-149653	CTCGCCCGCACGGGGCCTCCCTGGGAT	2	<lmxll>	ll	135	lm	119		
>ref-149695	CTTCCCTTTACTTTCCCTCCCCTCACT	3	<nnxnp>	nn	124	np	135		
>ref-149745	CTTATCCTTACTCTCCCTCCCTAACCC	1	<nnxnp>	nn	6	np	266		
>ref-149745	CTTATCCTTACTCTCCCTCCCTAACCC	23	<nnxnp>	nn	6	np	266		
>ref-149760	CCTATTTTCACCACCTCTCCCCCCTCC	2	<lmxll>	ll	64	lm	160		
>ref-149760	CCTATTTTCACCACCTCTCCCCCCTCC	4	<hkxhk>	hh	4	hk	221	kk	5
>ref-149760	CCTATTTTCACCACCTCTCCCCCCTCC	21	<nnxnp>	nn	42	np	179		
>ref-149762	GATAAAAAGACCTAACCTCCCCGGCGG	3	<hkxhk>	hh	0	hk	262	kk	3
>ref-149762	GATAAAAAGACCTAACCTCCCCGGCGG	7	<hkxhk>	hh	8	hk	256	kk	0
>ref-149762	GATAAAAAGACCTAACCTCCCCGGCGG	15	<hkxhk>	hh	0	hk	271	kk	0
>ref-149766	CGGGATGCAACTTCACCTCCTTCTGCA	3	<hkxhk>	hh	63	hk	151	kk	50
>ref-149776	GGACACAGTACAGCAACTCCTCCACAG	8	<lmxll>	ll	132	lm	126		
>ref-149779	GTAAGACGAACCCCCCCTCCCAGCGCC	12	<nnxnp>	nn	272	np	0		
>ref-149790	TCCGTTTCCACTAACCCTCCACCCTTC	13	<nnxnp>	nn	128	np	139		
>ref-149792	GTCTCCCCTACCTCCTCTCCTTTACCT	2	<lmxll>	ll	262	lm	0		
>ref-149825	GTTACTTACACATCTACTCCTACTGTT	7	<hkxhk>	hh	0	hk	273	kk	0
>ref-149825	GTTACTTACACATCTACTCCTACTGTT	9	<hkxhk>	hh	47	hk	223	kk	0
>ref-149827	CCCTCTCAGACTCGATCTCCTGCAGCT	5	<hkxhk>	hh	69	hk	135	kk	48
>ref-149836	CCAACACATACTGATCCTCCTGATGTA	23	<nnxnp>	nn	140	np	123		
>ref-149857	ACTTCTTCCACTCCCACTCCCCACTAA	21	<lmxll>	ll	129	lm	126		
>ref-149861	GCGACGGGGACTTACCCTCCGCCATTT	2	<nnxnp>	nn	119	np	119		
>ref-149862	ATACGCATAACAGCCTCTCCTCCCACG	3	<nnxnp>	nn	132	np	132		

>ref-149873	CAACCTCCAACGGCCCCTCCCCCTCAA	9	<lmxll>	ll	179	lm	76		
>ref-149883	CAAGTCGCAACCTCCTCTCCCATCCTG	9	<hkxhk>	hh	0	hk	269	kk	3
>ref-149888	TTTAACTCAACTACAACTCCACTTCAC	23	<lmxll>	ll	0	lm	266		
>ref-149890	CATCTGCACACCTCTCCTCCTTCTTCT	6	<lmxll>	ll	104	lm	121		
>ref-149890	CATCTGCACACCTCTCCTCCTTCTTCT	15	<lmxll>	ll	105	lm	122		
>ref-149916	GATTTCCCGACCCCTTCTCCTTTGACC	2	<hkxhk>	hh	2	hk	262	kk	2
>ref-149916	GATTTCCCGACCCCTTCTCCTTTGACC	8	<hkxhk>	hh	2	hk	261	kk	2
>ref-149925	CGATTTGCCACATAACCTCCATGGACA	12	<nnxnp>	nn	127	np	126		
>ref-149931	TCACCCTCCACTCCACCTCCTACTGCA	22	<nnxnp>	nn	118	np	148		
>ref-149983	AGGTTAGAAACCTCTTCTCCAGCCTGC	8	<hkxhk>	hh	0	hk	273	kk	0
>ref-150016	GATACATATACACTCTCTCCCCCACAC	1	<lmxll>	ll	105	lm	125		
>ref-150016	GATACATATACACTCTCTCCCCCACAC	15	<lmxll>	ll	105	lm	125		
>ref-150016	GATACATATACACTCTCTCCCCCACAC	23	<nnxnp>	nn	119	np	120		
>ref-150028	CATACATTCACACACTCTCCTGAACAA	6	<nnxnp>	nn	120	np	120		
>ref-150031	TCGTATACCACGTTATCTCCCTTAGCG	27	<nnxnp>	nn	137	np	126		
>ref-150033	CCATCTTCTACCCTCCCTCCGCCCCTT	2	<nnxnp>	nn	266	np	0		
>ref-150042	GCGGTAATGACCTTCACTCCTGACGCG	1	<nnxnp>	nn	7	np	232		
>ref-150042	GCGGTAATGACCTTCACTCCTGACGCG	4	<nnxnp>	nn	7	np	232		
>ref-150052	GGCTAATTAACGCCCCCTCCAAAAAGT	23	<nnxnp>	nn	94	np	127		
>ref-150066	CCCTGCTAGACTTAGGCTCCTCTGAAC	2	<hkxhk>	hh	57	hk	143	kk	47
>ref-150070	CCTTTCTTTACCTACCCTCCTTCTCCT	14	<lmxll>	ll	126	lm	124		
>ref-150070	CCTTTCTTTACCTACCCTCCTTCTCCT	24	<lmxll>	ll	131	lm	122		
>ref-150082	CATTTACCTACCCTAGCTCCCCACATT	5	<lmxll>	ll	135	lm	128		
>ref-150104	CCCCTGTCGACGGCGTCTCCACCTGCA	7	<hkxhk>	hh	60	hk	153	kk	56
>ref-150106	CACGTCCATACAATCCCTCCCTATCCT	3	<hkxhk>	hh	60	hk	136	kk	70
>ref-150106	CACGTCCATACAATCCCTCCCTATCCT	21	<lmxll>	ll	150	lm	117		
>ref-150113	TATCCTTACACTCCCCCTCCCCCTCTC	6	<lmxll>	ll	24	lm	234		
>ref-150127	GACAAGACGACATAGACTCCAGATAAA	22	<nnxnp>	nn	261	np	0		
>ref-150128	CACTTATTCACCGATGCTCCCTCACTC	14	<nnxnp>	nn	132	np	122		
>ref-150136	CTCTCACTCACTTCTTCTCCATCCCCT	6	<nnxnp>	nn	119	np	139		
>ref-150136	CTCTCACTCACTTCTTCTCCATCCCCT	27	<nnxnp>	nn	122	np	138		
>ref-150142	GCACAAGCGACGCGCGCTCCACCTGCT	21	<nnxnp>	nn	143	np	126		
>ref-150142	GCACAAGCGACGCGCGCTCCACCTGCT	23	<nnxnp>	nn	143	np	126		
>ref-150147	AACATACAGACACCCACTCCACAGTTA	6	<lmxll>	ll	158	lm	101		
>ref-150172	TTGTCACACACCTCCCCTCCCTTCCCC	4	<hkxhk>	hh	0	hk	267	kk	0
>ref-150172	TTGTCACACACCTCCCCTCCCTTCCCC	27	<hkxhk>	hh	0	hk	267	kk	0
>ref-150189	TATACCTAGACGGCTCCTCCTCCTCAT	26	<nnxnp>	nn	135	np	134		
>ref-150205	TCTTCCTCCACCTCTCCTCCTCGTTCG	12	<nnxnp>	nn	105	np	148		

>ref-150205	TCTTCCTCCACCTCTCCTCCTCGTTCG	27	<nnxnp>	nn	100	np	148		
>ref-150263	CCACCCAACACTACTCCTCCTTCCCTC	1	<lmxll>	ll	131	lm	132		
>ref-150263	CCACCCAACACTACTCCTCCTTCCCTC	4	<lmxll>	ll	123	lm	139		
>ref-150283	CCGATACTCACTGATCCTCCCTGATAC	7	<hkxhk>	hh	0	hk	271	kk	0
>ref-150283	CCGATACTCACTGATCCTCCCTGATAC	12	<hkxhk>	hh	0	hk	194	kk	73
>ref-150290	ATAAAAGAGACACATGCTCCTATTACC	25	<nnxnp>	nn	200	np	36		
>ref-150318	TCTCCTTCTACCTCTCCTCCCTTCCCC	12	<nnxnp>	nn	206	np	49		
>ref-150318	TCTCCTTCTACCTCTCCTCCCTTCCCC	25	<nnxnp>	nn	206	np	48		
>ref-150344	TCTTTCAACACTTTTCCTCCCTTTCTC	9	<nnxnp>	nn	104	np	126		
>ref-150344	TCTTTCAACACTTTTCCTCCCTTTCTC	13	<nnxnp>	nn	103	np	127		
>ref-150345	CCTCTCTCAACTCCCTCTCCATTCCCT	22	<hkxhk>	hh	0	hk	254	kk	13
>ref-150364	GTTTGTTTCACGCCACCTCCCTCCCCT	23	<lmxll>	ll	116	lm	114		
>ref-150383	TTCTCTCGTACTGCTCCTCCTCCTTTC	12	<lmxll>	ll	134	lm	135		
>ref-150384	CGAACATTGACCAAGGCTCCAGCGGCC	12	<nnxnp>	nn	119	np	151		
>ref-150384	CGAACATTGACCAAGGCTCCAGCGGCC	22	<nnxnp>	nn	120	np	151		
>ref-150393	AATGAAAAGACACGATCTCCAAATTCT	12	<lmxll>	ll	41	lm	196		
>ref-150393	AATGAAAAGACACGATCTCCAAATTCT	14	<lmxll>	ll	26	lm	210		
>ref-150393	AATGAAAAGACACGATCTCCAAATTCT	15	<lmxll>	ll	26	lm	210		
>ref-150409	ACTCCTCCCACACTCTCTCCCACACTC	4	<lmxll>	ll	104	lm	133		
>ref-150409	ACTCCTCCCACACTCTCTCCCACACTC	22	<lmxll>	ll	6	lm	259		
>ref-150409	ACTCCTCCCACACTCTCTCCCACACTC	26	<lmxll>	ll	7	lm	258		
>ref-150426	CGGCGTGTTACTGTTCCTCCGCTAGCC	9	<hkxhk>	hh	54	hk	145	kk	53
>ref-150435	CACGCTTTCACTTCTTCTCCCTGCCCC	15	<nnxnp>	nn	220	np	33		
>ref-150435	CACGCTTTCACTTCTTCTCCCTGCCCC	16	<nnxnp>	nn	206	np	43		
>ref-150437	CTTATTCTTACGTCCCCTCCTCCCCTA	14	<lmxll>	ll	138	lm	124		
>ref-150443	TCTTGCCTAACTTGCGCTCCCAAATCT	21	<lmxll>	ll	117	lm	130		
>ref-150447	GAAGCTCCTACAGGTACTCCCTCTCCA	9	<nnxnp>	nn	232	np	0		
>ref-150460	CTCTCTCTTACTCATTCTCCTTATGTC	2	<nnxnp>	nn	260	np	0		
>ref-150465	CCTGCCGGTACTTTCCCTCCATAATTA	22	<lmxll>	ll	147	lm	83		
>ref-150470	CTTCCACGTACTCCTTCTCCTCTTCTT	21	<lmxll>	ll	146	lm	122		
>ref-150530	CGTATAAGCACCGTAACTCCTTTTCCG	7	<nnxnp>	nn	124	np	138		
>ref-150567	CTGGTCTTAACAACCCCTCCTTCACCA	2	<lmxll>	ll	259	lm	2		
>ref-150567	CTGGTCTTAACAACCCCTCCTTCACCA	4	<lmxll>	ll	260	lm	2		
>ref-150575	TCTGCCCTTACCTAACCTCCCTGCCTA	15	<nnxnp>	nn	138	np	127		
>ref-150576	TGTAATATTACTCATTCTCCTTGCGTT	4	<nnxnp>	nn	126	np	140		
>ref-150588	CCCCGCTCCACCCCATCTCCCATCCCC	4	<nnxnp>	nn	95	np	138		
>ref-150592	TATAACAAGACAGGTGCTCCAGCAAGA	23	<lmxll>	ll	123	lm	123		
>ref-150601	AGAAAAGAAACTTGCCCTCCATGTCCT	21	<hkxhk>	hh	7	hk	261	kk	0

>ref-150601	AGAAAAGAAACTTGCCCTCCATGTCCT	24	<hkxhk>	hh	0	hk	272	kk	0
>ref-150615	TGTTGATAAACCTAGTCTCCGTTAAAT	2	<hkxhk>	hh	0	hk	273	kk	0
>ref-150615	TGTTGATAAACCTAGTCTCCGTTAAAT	7	<lmxll>	ll	170	lm	80		
>ref-150615	TGTTGATAAACCTAGTCTCCGTTAAAT	8	<hkxhk>	hh	0	hk	273	kk	0
>ref-150615	TGTTGATAAACCTAGTCTCCGTTAAAT	21	<hkxhk>	hh	88	hk	157	kk	0
>ref-150615	TGTTGATAAACCTAGTCTCCGTTAAAT	22	<hkxhk>	hh	0	hk	159	kk	88
>ref-150616	CACTCCATCACTCAATCTCCCTTTCTG	14	<lmxll>	ll	118	lm	136		
>ref-150624	CGTGTTTTAACCCCTTCTCCCTCCCCT	23	<nnxnp>	nn	118	np	143		
>ref-150629	CTCCGTGATACAATTTCTCCTTATGTT	4	<lmxll>	ll	110	lm	113		
>ref-150651	CTATGGTAAACAGTGGCTCCGCTCTCG	6	<nnxnp>	nn	143	np	122		
>ref-150651	CTATGGTAAACAGTGGCTCCGCTCTCG	8	<nnxnp>	nn	142	np	122		
>ref-150651	CTATGGTAAACAGTGGCTCCGCTCTCG	13	<nnxnp>	nn	114	np	148		
>ref-150659	CAGACCTACACACCAACTCCTTTAACA	15	<nnxnp>	nn	104	np	129		
>ref-150660	GCAGGTTCTACTCCGCCTCCGCCCTAT	26	<nnxnp>	nn	119	np	141		
>ref-150672	TCCATCGCCACATGTTCTCCATCACTC	12	<hkxhk>	hh	64	hk	136	kk	59
>ref-150674	CAACCACTCACCCTATCTCCCCCACCC	12	<lmxll>	ll	138	lm	130		
>ref-150674	CAACCACTCACCCTATCTCCCCCACCC	14	<nnxnp>	nn	36	np	228		
>ref-150674	CAACCACTCACCCTATCTCCCCCACCC	16	<nnxnp>	nn	32	np	233		
>ref-150674	CAACCACTCACCCTATCTCCCCCACCC	21	<nnxnp>	nn	113	np	146		
>ref-150674	CAACCACTCACCCTATCTCCCCCACCC	25	<lmxll>	ll	155	lm	110		
>ref-150680	AAGTTAACAACGCTATCTCCTCCTCAC	15	<nnxnp>	nn	130	np	136		
>ref-150709	CCTCATCCCACTCTCTCTCCCAACTCT	5	<hkxhk>	hh	7	hk	247	kk	2
>ref-150709	CCTCATCCCACTCTCTCTCCCAACTCT	24	<hkxhk>	hh	8	hk	246	kk	2
>ref-150710	GTCTTCTTTACTTATTCTCCTCGTTTC	12	<nnxnp>	nn	122	np	131		
>ref-150720	TATGATGGGACATCCTCTCCATGCTTC	27	<nnxnp>	nn	109	np	156		
>ref-150744	GCACTCTGGACCCCCCCTCCCCTTCCC	24	<nnxnp>	nn	55	np	191		
>ref-150744	GCACTCTGGACCCCCCCTCCCCTTCCC	25	<nnxnp>	nn	249	np	7		
>ref-150744	GCACTCTGGACCCCCCCTCCCCTTCCC	26	<nnxnp>	nn	115	np	131		
>ref-150755	TTCTGTCTTACTCTTCCTCCTTCCCTT	2	<hkxhk>	hh	0	hk	266	kk	7
>ref-150755	TTCTGTCTTACTCTTCCTCCTTCCCTT	23	<hkxhk>	hh	1	hk	260	kk	7
>ref-150755	TTCTGTCTTACTCTTCCTCCTTCCCTT	26	<hkxhk>	hh	0	hk	266	kk	7
>ref-150755	TTCTGTCTTACTCTTCCTCCTTCCCTT	27	<hkxhk>	hh	2	hk	260	kk	6
>ref-150787	TGGGAACCTACTTCATCTCCCTAGTTC	15	<nnxnp>	nn	113	np	147		
>ref-150805	CACGTTGACACCTTGCCTCCTCTCACC	26	<nnxnp>	nn	123	np	143		
>ref-150806	AAAATCCCCACTCACTCTCCGCTCATC	1	<lmxll>	ll	102	lm	128		
>ref-150807	TACGGCCGTACCTGCCCTCCTGTGTGT	3	<hkxhk>	hh	0	hk	269	kk	2
>ref-150855	TATGACTAGACTCACTCTCCAGGCGTC	3	<lmxll>	ll	123	lm	137		
>ref-150884	CCCCTTACAACCACCTCTCCTCCCCTT	1	<lmxll>	ll	221	lm	16		

>ref-150888	ACAGATAAAACGTCTTCTCCTTCTTCT	8	<nnxnp>	nn	131	np	133		
>ref-150910	ACCATACCAACACCGCCTCCAAGACAC	14	<lmxll>	ll	136	lm	125		
>ref-150914	AACACTCTAACTACATCTCCTGCCTCC	8	<hkxhk>	hh	74	hk	128	kk	62
>ref-150918	TTGGACTAAACAGACTCTCCCTTCATA	13	<nnxnp>	nn	121	np	144		
>ref-150946	CTCCCTTTTACCCTATCTCCCCTCTTC	4	<lmxll>	ll	178	lm	64		
>ref-150946	CTCCCTTTTACCCTATCTCCCCTCTTC	7	<nnxnp>	nn	65	np	199		
>ref-150946	CTCCCTTTTACCCTATCTCCCCTCTTC	15	<nnxnp>	nn	62	np	200		
>ref-150948	CCCTTCCTCACCTCTTCTCCCCTCGCT	6	<nnxnp>	nn	270	np	2		
>ref-150948	CCCTTCCTCACCTCTTCTCCCCTCGCT	7	<nnxnp>	nn	272	np	1		
>ref-150948	CCCTTCCTCACCTCTTCTCCCCTCGCT	16	<hkxhk>	hh	0	hk	268	kk	5
>ref-150951	TGTAATCAAACGTTACCTCCCTAAGAC	1	<hkxhk>	hh	45	hk	131	kk	55
>ref-150951	TGTAATCAAACGTTACCTCCCTAAGAC	4	<hkxhk>	hh	55	hk	131	kk	44
>ref-151000	ATGTAGGGAACATTACCTCCCCTAACC	8	<lmxll>	ll	254	lm	9		
>ref-151004	CAAGACTTCACTTTCACTCCTTCTACA	5	<nnxnp>	nn	117	np	112		
>ref-151017	CTCATCCACACCCTCTCTCCCTTCGTA	3	<lmxll>	ll	131	lm	133		
>ref-151020	ATGCTTGGAACTGATGCTCCTCTCACT	25	<lmxll>	ll	130	lm	137		
>ref-151021	CCCTCCTTCACCCCTTCTCCCCCCTTT	1	<nnxnp>	nn	263	np	0		
>ref-151021	CCCTCCTTCACCCCTTCTCCCCCCTTT	2	<hkxhk>	hh	3	hk	256	kk	2
>ref-151021	CCCTCCTTCACCCCTTCTCCCCCCTTT	3	<nnxnp>	nn	268	np	0		
>ref-151021	CCCTCCTTCACCCCTTCTCCCCCCTTT	4	<hkxhk>	hh	2	hk	253	kk	6
>ref-151023	GAAAGCAAAACGGGTACTCCAGCTTGT	14	<hkxhk>	hh	0	hk	272	kk	0
>ref-151023	GAAAGCAAAACGGGTACTCCAGCTTGT	16	<hkxhk>	hh	1	hk	272	kk	0
>ref-151023	GAAAGCAAAACGGGTACTCCAGCTTGT	22	<hkxhk>	hh	87	hk	172	kk	0
>ref-151023	GAAAGCAAAACGGGTACTCCAGCTTGT	26	<hkxhk>	hh	0	hk	180	kk	88
>ref-151025	CCCAATTTTACCTCTCCTCCTTATCAA	9	<nnxnp>	nn	115	np	110		
>ref-151048	TCTGTTTTTACTCGTCCTCCTCTTTTC	5	<nnxnp>	nn	13	np	242		
>ref-151048	TCTGTTTTTACTCGTCCTCCTCTTTTC	14	<nnxnp>	nn	13	np	242		
>ref-151051	GCCTCCAAGACGGACTCTCCTGCGTAT	3	<nnxnp>	nn	116	np	146		
>ref-151063	GCCCTATCAACTATGCCTCCTGTCATC	2	<nnxnp>	nn	148	np	113		
>ref-151063	GCCCTATCAACTATGCCTCCTGTCATC	4	<nnxnp>	nn	149	np	113		
>ref-151063	GCCCTATCAACTATGCCTCCTGTCATC	27	<nnxnp>	nn	110	np	148		
>ref-151078	CGAGTGAACACCCACGCTCCTTAACAG	22	<lmxll>	ll	136	lm	130		
>ref-151086	TCTCATCCCACTGGTCCTCCCTTTCCC	5	<nnxnp>	nn	146	np	123		
>ref-151093	TCTTCCAACACCTCTTCTCCCCGGTTC	7	<lmxll>	ll	266	lm	0		
>ref-151096	AAATTTCTTACTAACTCTCCGTCGCCA	1	<lmxll>	ll	134	lm	103		
>ref-151104	GCCTCAGAGACGCCTCCTCCCCGCGCC	6	<nnxnp>	nn	114	np	146		
>ref-151118	CTTTGGTACACATGCCCTCCGCCTTCG	21	<lmxll>	ll	137	lm	129		
>ref-151119	GAGATTGTAACTTCGACTCCTGATATG	14	<lmxll>	ll	141	lm	100		

>ref-151121	CTCAACATCACCTACACTCCTTGAAAC	1	<hkxhk>	hh	53	hk	121	kk	65
>ref-151121	CTCAACATCACCTACACTCCTTGAAAC	8	<hkxhk>	hh	65	hk	121	kk	53
>ref-151144	CTTACGCCTACACACCCTCCTACGCTA	16	<lmxll>	ll	131	lm	129		
>ref-151155	CTGTCCATGACCCATCCTCCCTCTTAG	8	<nnxnp>	nn	121	np	145		
>ref-151160	TTTGTCTTAACCCCTTCTCCCCTTCCC	15	<hkxhk>	hh	41	hk	138	kk	56
>ref-151178	ATGTACCTTACCCCTCCTCCTCCTCCT	1	<lmxll>	ll	127	lm	129		
>ref-151193	CCGTATACTACGGCAACTCCCATCTTC	2	<lmxll>	ll	15	lm	248		
>ref-151193	CCGTATACTACGGCAACTCCCATCTTC	7	<hkxhk>	hh	0	hk	259	kk	9
>ref-151201	ATTTTAGCGACTATTTCTCCTGCGTCT	3	<nnxnp>	nn	99	np	127		
>ref-151214	TCTTTCCCAACTCTCCCTCCCTTCCTT	9	<hkxhk>	hh	2	hk	270	kk	0
>ref-151214	TCTTTCCCAACTCTCCCTCCCTTCCTT	13	<hkxhk>	hh	2	hk	270	kk	0
>ref-151214	TCTTTCCCAACTCTCCCTCCCTTCCTT	15	<lmxll>	ll	114	lm	154		
>ref-151221	TCTTCGATGACAGTCCCTCCCCGCTCT	27	<lmxll>	ll	199	lm	37		
>ref-151226	ATCTACCCCACTTTCCCTCCCTTCTCA	3	<lmxll>	ll	263	lm	0		
>ref-151235	CTTCTACTTACTCCTCCTCCTTTTTTC	8	<nnxnp>	nn	270	np	0		
>ref-151245	CCTTCCCCCACCTTTCCTCCCCCCATT	7	<hkxhk>	hh	2	hk	268	kk	0
>ref-151245	CCTTCCCCCACCTTTCCTCCCCCCATT	22	<hkxhk>	hh	2	hk	268	kk	0
>ref-151252	TGCATCCTCACCAACTCTCCTGATATC	4	<hkxhk>	hh	0	hk	260	kk	0
>ref-151252	TGCATCCTCACCAACTCTCCTGATATC	12	<nnxnp>	nn	175	np	79		
>ref-151252	TGCATCCTCACCAACTCTCCTGATATC	14	<hkxhk>	hh	0	hk	260	kk	0
>ref-151252	TGCATCCTCACCAACTCTCCTGATATC	27	<hkxhk>	hh	2	hk	256	kk	0
>ref-151274	TCCTACATCACTCTCTCTCCCTCTTCC	24	<lmxll>	ll	132	lm	131		
>ref-151275	ATTCTTCTCACTCCCTCTCCACTCTCC	7	<lmxll>	ll	16	lm	239		
>ref-151275	ATTCTTCTCACTCCCTCTCCACTCTCC	21	<lmxll>	ll	15	lm	239		
>ref-151279	CTCCCTAAAACTCTCTCTCCTTCCCTC	7	<nnxnp>	nn	219	np	14		
>ref-151279	CTCCCTAAAACTCTCTCTCCTTCCCTC	8	<nnxnp>	nn	230	np	7		
>ref-151279	CTCCCTAAAACTCTCTCTCCTTCCCTC	9	<nnxnp>	nn	230	np	14		
>ref-151292	CAAGCATTAACGACTACTCCTCTCCCC	21	<lmxll>	ll	132	lm	102		
>ref-151292	CAAGCATTAACGACTACTCCTCTCCCC	27	<nnxnp>	nn	101	np	124		
>ref-151299	CCAACACCTACATTATCTCCCATGTAT	1	<nnxnp>	nn	127	np	129		
>ref-151302	AACGACCTGACCCTCGCTCCCCGTCCC	9	<lmxll>	ll	263	lm	0		
>ref-151302	AACGACCTGACCCTCGCTCCCCGTCCC	23	<lmxll>	ll	263	lm	0		
>ref-151319	CTCTCCCCTACCTTCTCTCCCTCAATG	24	<lmxll>	ll	109	lm	149		
>ref-151319	CTCTCCCCTACCTTCTCTCCCTCAATG	27	<lmxll>	ll	110	lm	148		
>ref-151323	GTGCCTAAAACTACATCTCCTACATCT	1	<lmxll>	ll	138	lm	97		
>ref-151339	CTTATCCCTACCTCTCCTCCTCCCTTC	14	<hkxhk>	hh	0	hk	258	kk	3
>ref-151359	CCCAGCAACACCTCCCCTCCCCCACAG	22	<nnxnp>	nn	125	np	134		
>ref-151363	CCTAGCTTAACATTCCCTCCATATCTT	16	<lmxll>	ll	241	lm	1		

>ref-151370	TCGACCACGACAAAGTCTCCCATCACT	5	<hkxhk>	hh	0	hk	273	kk	0
>ref-151371	CCACCGTACACTGTCTCTCCCTCTCCT	27	<lmxll>	ll	130	lm	139		
>ref-151374	CTTCCCTTCACGCCCCCTCCCCCTCCT	6	<lmxll>	ll	255	lm	0		
>ref-151374	CTTCCCTTCACGCCCCCTCCCCCTCCT	21	<lmxll>	ll	232	lm	11		
>ref-151375	GCACGGCGCACGTCTCCTCCTCCTTTC	9	<lmxll>	ll	133	lm	139		
>ref-151405	TCCTCCTCCACCTCCTCTCCCCAAGCA	16	<lmxll>	ll	6	lm	260		
>ref-151405	TCCTCCTCCACCTCCTCTCCCCAAGCA	21	<lmxll>	ll	6	lm	260		
>ref-151421	GAATCGTGCACGTCTTCTCCCCCAGTC	6	<hkxhk>	hh	0	hk	262	kk	3
>ref-151438	CTCCATGCCACGAAGACTCCAAACTTC	21	<nnxnp>	nn	112	np	138		
>ref-151439	CATGTGGGGACACAGCCTCCCCCTATT	3	<hkxhk>	hh	54	hk	138	kk	62
>ref-151444	GTCTCATCTACCCTTCCTCCCTTCTAC	13	<nnxnp>	nn	105	np	165		
>ref-151444	GTCTCATCTACCCTTCCTCCCTTCTAC	16	<nnxnp>	nn	65	np	198		
>ref-151444	GTCTCATCTACCCTTCCTCCCTTCTAC	25	<lmxll>	ll	40	lm	227		
>ref-151444	GTCTCATCTACCCTTCCTCCCTTCTAC	27	<nnxnp>	nn	265	np	6		
>ref-151460	CCCTCTGCCACTCCCCCTCCCCTCAGA	12	<nnxnp>	nn	254	np	1		
>ref-151512	CAGGAGACAACAATCCCTCCCCCACGA	4	<lmxll>	ll	125	lm	95		
>ref-151518	TCCTTAGCCACATCTTCTCCTCTTCCT	7	<lmxll>	ll	138	lm	132		
>ref-151533	ACACTATGCACTATAACTCCCACGGCC	4	<hkxhk>	hh	0	hk	269	kk	0
>ref-151533	ACACTATGCACTATAACTCCCACGGCC	7	<hkxhk>	hh	0	hk	269	kk	0
>ref-151556	GTCGCAGCTACATTCCCTCCATACCAT	9	<nnxnp>	nn	140	np	118		
>ref-151562	CAGTCGACAACACCATCTCCAGTGACC	13	<lmxll>	ll	129	lm	120		
>ref-151604	CCCTCACCCACCCTGCCTCCCCATGCT	1	<nnxnp>	nn	272	np	0		
>ref-151639	CCTCGATCCACATACCCTCCCTTCCCT	24	<nnxnp>	nn	133	np	123		
>ref-151641	CTGCCCTTCACCCCATCTCCTTCTTCC	2	<hkxhk>	hh	0	hk	244	kk	11
>ref-151641	CTGCCCTTCACCCCATCTCCTTCTTCC	7	<hkxhk>	hh	0	hk	244	kk	11
>ref-151655	CCTTCCCTGACCGGCCCTCCGGATTCA	12	<lmxll>	ll	107	lm	143		
>ref-151663	TACCCACCTACCCACCCTCCCAGCCAC	25	<nnxnp>	nn	129	np	140		
>ref-151666	CTCCCTACTACACTCTCTCCCTCCCCG	8	<nnxnp>	nn	109	np	144		
>ref-151675	CTCTTGTCCACGTCTTCTCCTCAGATG	8	<hkxhk>	hh	64	hk	143	kk	48
>ref-151701	CTTCTCCATACTCCCTCTCCCACCCCC	23	<nnxnp>	nn	91	np	129		
>ref-151727	TCTCTTTTGACTGTTTCTCCGTCTGGC	25	<hkxhk>	hh	57	hk	132	kk	49
>ref-151731	TTCCTTCGAACCAGCTCTCCTCTCCCC	3	<lmxll>	ll	166	lm	92		
>ref-151731	TTCCTTCGAACCAGCTCTCCTCTCCCC	22	<hkxhk>	hh	32	hk	235	kk	0
>ref-151732	CTCTCTTCGACTCCGACTCCCTCCTTA	14	<lmxll>	ll	144	lm	105		
>ref-151733	AGCCCGAGTACATTCACTCCCTAAGTA	5	<nnxnp>	nn	113	np	119		
>ref-151739	TACCCCTTGACCTTTTCTCCCTCCCCC	21	<hkxhk>	hh	3	hk	248	kk	0
>ref-151739	TACCCCTTGACCTTTTCTCCCTCCCCC	22	<hkxhk>	hh	0	hk	248	kk	3
>ref-151758	CTCACATGTACGTCCTCTCCCCCAATT	6	<nnxnp>	nn	121	np	143		

>ref-151759	TAGACACTCACTCTCTCTCCCTCTATT	9	<hkxhk>	hh	63	hk	138	kk	56
>ref-151766	CGAGGGAGCACTTTCGCTCCCTGGTGC	1	<hkxhk>	hh	0	hk	265	kk	3
>ref-151766	CGAGGGAGCACTTTCGCTCCCTGGTGC	15	<hkxhk>	hh	4	hk	268	kk	0
>ref-151766	CGAGGGAGCACTTTCGCTCCCTGGTGC	21	<hkxhk>	hh	0	hk	165	kk	99
>ref-151766	CGAGGGAGCACTTTCGCTCCCTGGTGC	24	<hkxhk>	hh	12	hk	257	kk	0
>ref-151774	AACTTGCCTACAACTTCTCCGTCTCAT	15	<lmxll>	ll	148	lm	123		
>ref-151791	ACACCTACCACAACTACTCCTGCTGAA	15	<hkxhk>	hh	66	hk	120	kk	50
>ref-151793	TTCACTCCGACGGCGCCTCCAAACTTA	7	<nnxnp>	nn	146	np	117		
>ref-151798	CTCATCTGGACGTTAACTCCCTTCCTT	12	<nnxnp>	nn	121	np	127		
>ref-151822	ATCATATCTACGTCTTCTCCTTTTCCT	27	<hkxhk>	hh	51	hk	149	kk	56
>ref-151870	GCTCCGTCCACTGTCTCTCCCTCTCTC	1	<nnxnp>	nn	120	np	141		
>ref-151870	GCTCCGTCCACTGTCTCTCCCTCTCTC	6	<nnxnp>	nn	121	np	142		
>ref-151873	AGGTAGACAACCTTGACTCCTTTATTG	2	<hkxhk>	hh	1	hk	266	kk	1
>ref-151902	TCTCCTGTCACTTTCACTCCCTCACTG	7	<hkxhk>	hh	60	hk	146	kk	57
>ref-151907	ACCTTTCCTACTCCTTCTCCACCTTTC	1	<lmxll>	ll	215	lm	20		
>ref-151907	ACCTTTCCTACTCCTTCTCCACCTTTC	2	<lmxll>	ll	260	lm	1		
>ref-151921	ACACCATCGACATTGACTCCTTTGTGG	16	<lmxll>	ll	116	lm	121		
>ref-151929	CTAACTACCACCTCCCCTCCCCCTCCC	8	<lmxll>	ll	8	lm	258		
>ref-151929	CTAACTACCACCTCCCCTCCCCCTCCC	16	<lmxll>	ll	8	lm	258		
>ref-151976	CCACTCCCTACTATCTCTCCCTCTCTC	5	<nnxnp>	nn	247	np	4		
>ref-151976	CCACTCCCTACTATCTCTCCCTCTCTC	9	<nnxnp>	nn	243	np	4		
>ref-151976	CCACTCCCTACTATCTCTCCCTCTCTC	23	<hkxhk>	hh	9	hk	151	kk	77
>ref-152019	TTATTCTCTACTCTGCCTCCTTCCTCT	14	<nnxnp>	nn	130	np	130		
>ref-152020	TTAAGATCTACCGCTTCTCCCCTTCCC	1	<nnxnp>	nn	129	np	138		
>ref-152040	GTCCCTCCCACCGCCCCTCCTTCTTCC	1	<lmxll>	ll	86	lm	141		
>ref-152040	GTCCCTCCCACCGCCCCTCCTTCTTCC	3	<lmxll>	ll	97	lm	137		
>ref-152047	TTTCTCATCACCTTCCCTCCTTCTCCC	8	<hkxhk>	hh	44	hk	207	kk	5
>ref-152047	TTTCTCATCACCTTCCCTCCTTCTCCC	12	<hkxhk>	hh	4	hk	207	kk	45
>ref-152049	GGTGCTGCCACTGTTCCTCCGCGCCCT	5	<hkxhk>	hh	1	hk	268	kk	0
>ref-152049	GGTGCTGCCACTGTTCCTCCGCGCCCT	13	<hkxhk>	hh	1	hk	268	kk	0
>ref-152092	AACAGGACAACCAGAACTCCATGGAAA	9	<nnxnp>	nn	126	np	133		
>ref-152095	ATGCCCTACACATTCACTCCACTCACT	1	<hkxhk>	hh	1	hk	269	kk	0
>ref-152095	ATGCCCTACACATTCACTCCACTCACT	23	<lmxll>	ll	142	lm	123		
>ref-152095	ATGCCCTACACATTCACTCCACTCACT	25	<nnxnp>	nn	25	np	234		
>ref-152096	TTTGCCGATACCCATTCTCCTCCTCCT	6	<lmxll>	ll	7	lm	263		
>ref-152096	TTTGCCGATACCCATTCTCCTCCTCCT	16	<lmxll>	ll	7	lm	263		
>ref-152103	CCTCCCTTTACGGTTCCTCCTCCCTCT	12	<lmxll>	ll	137	lm	113		
>ref-152114	TCCTCATTTACCCTCCCTCCCTCCCTT	27	<nnxnp>	nn	8	np	254		

>ref-152145	CATCTAACAACACATGCTCCTATACAC	8	<hkxhk>	hh	122	hk	102	kk	2
>ref-152147	CGGCCCTCAACAGCCCCTCCTCCCCGG	1	<hkxhk>	hh	0	hk	273	kk	0
>ref-152171	CCACTTCCAACCTCCCCTCCCTTCACT	2	<nnxnp>	nn	108	np	159		
>ref-152192	AGGTCATGCACTCCCCCTCCTCTTCCT	21	<lmxll>	ll	143	lm	125		
>ref-152192	AGGTCATGCACTCCCCCTCCTCTTCCT	27	<lmxll>	ll	143	lm	125		
>ref-152200	AGAGAGCTTACCTTTCCTCCTCCACCG	21	<nnxnp>	nn	267	np	0		
>ref-152201	CATCTGACAACAACCACTCCTTGACAA	3	<nnxnp>	nn	104	np	161		
>ref-152220	CCGTCTTCTACAAGTACTCCAGAGTAT	2	<lmxll>	ll	120	lm	123		
>ref-152228	CCCACCGCCACCTTCCCTCCCTTCCCC	21	<nnxnp>	nn	109	np	124		
>ref-152260	CTCCCCTTCACTCCCTCTCCATCGCTC	1	<nnxnp>	nn	112	np	152		
>ref-152272	TGTTCTGTGACCTCCCCTCCTTTGCCA	16	<nnxnp>	nn	119	np	148		
>ref-152280	TATCCATACACAGGTGCTCCCTTAAGT	12	<lmxll>	ll	140	lm	104		
>ref-152280	TATCCATACACAGGTGCTCCCTTAAGT	13	<hkxhk>	hh	0	hk	269	kk	1
>ref-152280	TATCCATACACAGGTGCTCCCTTAAGT	21	<hkxhk>	hh	1	hk	269	kk	0
>ref-152280	TATCCATACACAGGTGCTCCCTTAAGT	25	<nnxnp>	nn	139	np	110		
>ref-152280	TATCCATACACAGGTGCTCCCTTAAGT	26	<hkxhk>	hh	0	hk	256	kk	0
>ref-152282	AAGTCTTTAACCAATCCTCCCAGTTAT	4	<hkxhk>	hh	0	hk	269	kk	2
>ref-152282	AAGTCTTTAACCAATCCTCCCAGTTAT	5	<hkxhk>	hh	43	hk	217	kk	0
>ref-152282	AAGTCTTTAACCAATCCTCCCAGTTAT	9	<hkxhk>	hh	34	hk	224	kk	0
>ref-152282	AAGTCTTTAACCAATCCTCCCAGTTAT	12	<lmxll>	ll	7	lm	262		
>ref-152282	AAGTCTTTAACCAATCCTCCCAGTTAT	13	<lmxll>	ll	7	lm	262		
>ref-152293	CAGATATAAACACTCTCTCCTACCTGT	23	<hkxhk>	hh	5	hk	220	kk	3
>ref-152296	CATTCCTCAACTCTCTCTCCTTCTTCC	26	<nnxnp>	nn	131	np	132		
>ref-152304	CTAAACGGAACATGTACTCCGAGCAAT	3	<nnxnp>	nn	129	np	134		
>ref-152304	CTAAACGGAACATGTACTCCGAGCAAT	7	<nnxnp>	nn	131	np	131		
>ref-152307	GGCTTGGCTACATCAGCTCCAACATTT	1	<lmxll>	ll	124	lm	130		
>ref-152307	GGCTTGGCTACATCAGCTCCAACATTT	8	<lmxll>	ll	125	lm	125		
>ref-152333	CTTACGAGGACGTCTCCTCCCCTGAAA	16	<lmxll>	ll	3	lm	266		
>ref-152338	TAATAATGCACAATACCTCCCTTGATC	9	<nnxnp>	nn	123	np	124		
>ref-152372	TGGAATACAACCGTAGCTCCTAGATAA	26	<lmxll>	ll	117	lm	121		
>ref-152390	TGATTTTTCACTCAGCCTCCGCTTCGC	16	<lmxll>	ll	144	lm	97		
>ref-152390	TGATTTTTCACTCAGCCTCCGCTTCGC	27	<lmxll>	ll	144	lm	97		
>ref-152393	CCTTCTGCCACCCTCCCTCCCCCCTAT	14	<lmxll>	ll	9	lm	249		
>ref-152393	CCTTCTGCCACCCTCCCTCCCCCCTAT	27	<lmxll>	ll	9	lm	249		
>ref-152412	TTGAATAAGACGATTCCTCCTGCAAAA	21	<lmxll>	ll	114	lm	112		
>ref-152415	CTTCCCTACACCAGCCCTCCAGATCCT	5	<nnxnp>	nn	134	np	126		
>ref-152415	CTTCCCTACACCAGCCCTCCAGATCCT	7	<nnxnp>	nn	134	np	126		
>ref-152427	CACACACACACACAGCCTCCCATGGGA	21	<hkxhk>	hh	62	hk	148	kk	45

>ref-152443	GTTAGGTAAACATTTTCTCCATTTTAC	8	<hkxhk>	hh	62	hk	159	kk	6
>ref-152443	GTTAGGTAAACATTTTCTCCATTTTAC	21	<hkxhk>	hh	62	hk	159	kk	6
>ref-152456	TCCCTCTGTACTCGCTCTCCCTCTGTT	26	<nnxnp>	nn	65	np	169		
>ref-152457	CTCGCCATCACCATTACTCCGCCGTTT	3	<lmxll>	ll	133	lm	125		
>ref-152462	ACTAACACTACGAGGTCTCCGCTCCCT	15	<hkxhk>	hh	61	hk	155	kk	48
>ref-152468	GACACTTCTACGCTGCCTCCGAGAAAA	9	<nnxnp>	nn	254	np	0		
>ref-152475	GCTGGCACCACTCGGGCTCCGACACTC	16	<lmxll>	ll	137	lm	135		
>ref-152475	GCTGGCACCACTCGGGCTCCGACACTC	22	<lmxll>	ll	135	lm	138		
>ref-152486	TCTTCTCCCACTTCTTCTCCTCCGTTC	24	<nnxnp>	nn	241	np	2		
>ref-152518	TCTTCCTCTACTCTCCCTCCGTCCCTC	8	<lmxll>	ll	146	lm	120		
>ref-152520	CTCTTTCTCACTAGTTCTCCTCTTTTA	1	<nnxnp>	nn	156	np	72		
>ref-152520	CTCTTTCTCACTAGTTCTCCTCTTTTA	3	<nnxnp>	nn	156	np	73		
>ref-152523	GGTCTCTCCACCCTTTCTCCCTTCTTC	1	<nnxnp>	nn	133	np	128		
>ref-152523	GGTCTCTCCACCCTTTCTCCCTTCTTC	15	<nnxnp>	nn	132	np	128		
>ref-152530	AGATTTTGAACATTCTCTCCAATTCAT	27	<lmxll>	ll	131	lm	104		
>ref-152539	GACCTAAGCACGACACCTCCCTTACTC	8	<hkxhk>	hh	0	hk	272	kk	0
>ref-152539	GACCTAAGCACGACACCTCCCTTACTC	22	<hkxhk>	hh	0	hk	272	kk	0
>ref-152563	TGTTAGCTTACACAGCCTCCATGTACT	4	<hkxhk>	hh	0	hk	267	kk	2
>ref-152563	TGTTAGCTTACACAGCCTCCATGTACT	24	<lmxll>	ll	129	lm	142		
>ref-152565	GAAGCAGCGACTGATTCTCCGACTGAA	5	<lmxll>	ll	203	lm	62		
>ref-152569	TACACACAAACGTATGCTCCACTTACT	22	<hkxhk>	hh	46	hk	141	kk	46
>ref-152569	TACACACAAACGTATGCTCCACTTACT	26	<hkxhk>	hh	45	hk	140	kk	45
>ref-152585	ATACTGTGCACCGGCTCTCCATTGCTC	12	<nnxnp>	nn	236	np	31		
>ref-152585	ATACTGTGCACCGGCTCTCCATTGCTC	21	<hkxhk>	hh	1	hk	271	kk	0
>ref-152588	GAACAACTAACTGGTTCTCCATCAAGC	6	<hkxhk>	hh	7	hk	254	kk	0
>ref-152641	TGCAAATTCACCCCCTCTCCTTCTCTT	14	<lmxll>	ll	126	lm	127		
>ref-152708	CTCCTACCTACCTTCACTCCATAACAA	4	<lmxll>	ll	131	lm	129		
>ref-152708	CTCCTACCTACCTTCACTCCATAACAA	15	<lmxll>	ll	121	lm	139		
>ref-152710	CTCCCCTGCACTTCCCCTCCTCGTCTA	16	<lmxll>	ll	121	lm	141		
>ref-152710	CTCCCCTGCACTTCCCCTCCTCGTCTA	23	<lmxll>	ll	121	lm	140		
>ref-152734	GTATATTTCACTCTCTCTCCATTTATC	1	<hkxhk>	hh	10	hk	240	kk	2
>ref-152734	GTATATTTCACTCTCTCTCCATTTATC	3	<hkxhk>	hh	10	hk	240	kk	2
>ref-152744	TGTGCGGCAACGCCTCCTCCTCCCCCC	27	<nnxnp>	nn	260	np	0		
>ref-152759	TACACTCCAACCTCCCCTCCCCTCACT	21	<nnxnp>	nn	104	np	147		
>ref-152763	TTCCTAGCCACCCTCACTCCTCCCTCC	1	<lmxll>	ll	2	lm	271		
>ref-152763	TTCCTAGCCACCCTCACTCCTCCCTCC	23	<lmxll>	ll	2	lm	271		
>ref-152773	CCTCATTGTACCTTTCCTCCTTCTCAT	1	<hkxhk>	hh	62	hk	123	kk	59
>ref-152775	ATGTAGACTACGCTCTCTCCTTCACAC	21	<hkxhk>	hh	64	hk	140	kk	67

>ref-152789	GGAGAATTAACTTACGCTCCAGTGTGG	2	<lmxll>	ll	107	lm	132		
>ref-152795	GACTCGAAAACATTGACTCCTATTGAA	13	<hkxhk>	hh	0	hk	168	kk	66
>ref-152810	AGAACTAAAACAAAAACTCCAAGTGAA	1	<lmxll>	ll	26	lm	231		
>ref-152810	AGAACTAAAACAAAAACTCCAAGTGAA	15	<hkxhk>	hh	2	hk	266	kk	0
>ref-152812	CGGAAAGAGACCGTTTCTCCATCAACA	22	<nnxnp>	nn	133	np	123		
>ref-152816	CGCAGTCCGACCGATCCTCCGACCTGA	2	<hkxhk>	hh	2	hk	271	kk	0
>ref-152816	CGCAGTCCGACCGATCCTCCGACCTGA	9	<nnxnp>	nn	273	np	0		
>ref-152816	CGCAGTCCGACCGATCCTCCGACCTGA	14	<hkxhk>	hh	101	hk	138	kk	0
>ref-152816	CGCAGTCCGACCGATCCTCCGACCTGA	15	<hkxhk>	hh	2	hk	271	kk	0
>ref-152816	CGCAGTCCGACCGATCCTCCGACCTGA	24	<lmxll>	ll	168	lm	84		
>ref-152816	CGCAGTCCGACCGATCCTCCGACCTGA	25	<nnxnp>	nn	263	np	6		
>ref-152833	TTCTCACTCACTCATCCTCCATCCACT	23	<nnxnp>	nn	123	np	148		
>ref-152855	TAAATGAGGACGATCCCTCCCCCCTCC	21	<lmxll>	ll	121	lm	136		
>ref-152855	TAAATGAGGACGATCCCTCCCCCCTCC	25	<lmxll>	ll	119	lm	136		
>ref-152859	AATGGCCACACAGGGACTCCCAAAACC	27	<lmxll>	ll	86	lm	140		
>ref-152864	TCTCTTCTTACCCCCCCTCCCCATATC	8	<hkxhk>	hh	0	hk	179	kk	79
>ref-152868	CCTTAAGCAACCCGTTCTCCCATTAGT	15	<nnxnp>	nn	259	np	0		
>ref-152872	TCTCTCTTAACTCTCTCTCCCTTACTG	6	<lmxll>	ll	116	lm	137		
>ref-152897	CTTACTTCCACCATCGCTCCTCCTCAC	27	<nnxnp>	nn	226	np	0		
>ref-152919	GGCCACCCTACGCATCCTCCCGCGGCT	6	<nnxnp>	nn	92	np	162		
>ref-152919	GGCCACCCTACGCATCCTCCCGCGGCT	12	<nnxnp>	nn	93	np	163		
>ref-152919	GGCCACCCTACGCATCCTCCCGCGGCT	24	<lmxll>	ll	0	lm	273		
>ref-152919	GGCCACCCTACGCATCCTCCCGCGGCT	26	<lmxll>	ll	77	lm	193		
>ref-152921	TCGCGCTTCACTCTCACTCCATTTTCT	4	<nnxnp>	nn	255	np	0		
>ref-152933	TGTTAGTAAACCTTCCCTCCCTCCTTC	12	<nnxnp>	nn	145	np	117		
>ref-152952	TCGCTGGTGACCGGAGCTCCGTCCTTG	25	<hkxhk>	hh	40	hk	131	kk	55
>ref-152953	TGTTTATCCACCTTCTCTCCTATCTGT	22	<lmxll>	ll	105	lm	143		
>ref-152957	GTCGATTTCACACACTCTCCTGTTACA	26	<nnxnp>	nn	266	np	0		
>ref-152957	GTCGATTTCACACACTCTCCTGTTACA	27	<nnxnp>	nn	262	np	0		
>ref-152981	CGCTATTCCACCGCACCTCCGTCCCAC	16	<lmxll>	ll	110	lm	158		
>ref-152999	CATAAATGGACGATCTCTCCTTCTCTA	24	<lmxll>	ll	108	lm	141		
>ref-153023	CCCCTCCCAACCTCTCCTCCCGTGTAT	1	<lmxll>	ll	17	lm	246		
>ref-153033	CCCCTTTTTACTCCAACTCCCTCCCCA	9	<lmxll>	ll	120	lm	133		
>ref-153034	CATCTTCCTACTGAACCTCCTCCTATT	13	<nnxnp>	nn	129	np	124		
>ref-153041	GTTATGTGAACCTGCACTCCCATGCAT	3	<nnxnp>	nn	133	np	136		
>ref-153065	TTCACATACACGTAGTCTCCTTTCCCC	1	<lmxll>	ll	140	lm	129		
>ref-153065	TTCACATACACGTAGTCTCCTTTCCCC	2	<lmxll>	ll	140	lm	130		
>ref-153068	TCTCCTTTAACCATTCCTCCTCTATTT	22	<lmxll>	ll	116	lm	142		

>ref-153068	TCTCCTTTAACCATTCCTCCTCTATTT	24	<lmxll>	ll	137	lm	121		
>ref-153075	GGTTATTTGACAGTGTCTCCCTTGCCT	4	<lmxll>	ll	157	lm	111		
>ref-153075	GGTTATTTGACAGTGTCTCCCTTGCCT	7	<hkxhk>	hh	0	hk	229	kk	26
>ref-153075	GGTTATTTGACAGTGTCTCCCTTGCCT	15	<lmxll>	ll	157	lm	111		
>ref-153075	GGTTATTTGACAGTGTCTCCCTTGCCT	16	<hkxhk>	hh	0	hk	272	kk	0
>ref-153082	GTCACCTCCACCTCCTCTCCCCCCTCC	9	<hkxhk>	hh	64	hk	178	kk	16
>ref-153094	ATACATAGTACTCCTCCTCCGAGGATG	15	<lmxll>	ll	123	lm	145		
>ref-153098	ATCTTATCGACTTCACCTCCCTCACCC	15	<hkxhk>	hh	62	hk	113	kk	49
>ref-153099	ACCTACGCAACCCTTTCTCCCTCCCCC	8	<lmxll>	ll	129	lm	132		
>ref-153122	CGCTGTTCTACTCCACCTCCATGTAAT	25	<nnxnp>	nn	116	np	126		
>ref-153126	CCCTCCTCTACCTTGCCTCCTCAGACC	26	<nnxnp>	nn	153	np	109		
>ref-153126	CCCTCCTCTACCTTGCCTCCTCAGACC	27	<nnxnp>	nn	137	np	133		
>ref-153138	TGTTCTCTGACTCTCTCTCCTCTCCCC	27	<nnxnp>	nn	119	np	135		
>ref-153141	TGGCCCCCTACCCGCACTCCCCCACCT	2	<nnxnp>	nn	126	np	136		
>ref-153163	TTTTACATCACTGCTACTCCGACTGCA	13	<lmxll>	ll	140	lm	121		
>ref-153168	TACATACATACATATGCTCCCTCTCCC	13	<nnxnp>	nn	132	np	119		
>ref-153174	TAGGTAATTACCTTCTCTCCTTAAATC	3	<nnxnp>	nn	127	np	106		
>ref-153182	TTCATTCCTACCCTATCTCCTCCCCGG	27	<nnxnp>	nn	120	np	122		
>ref-153206	GACACTCCTACTTATTCTCCTCCTCCT	22	<lmxll>	ll	126	lm	134		
>ref-153233	ATTATTAGTACCCCCTCTCCCTTCCTC	9	<nnxnp>	nn	13	np	248		
>ref-153233	ATTATTAGTACCCCCTCTCCCTTCCTC	15	<nnxnp>	nn	13	np	248		
>ref-153260	GGTATCTGCACATCCTCTCCCGGATAT	4	<nnxnp>	nn	108	np	145		
>ref-153271	ATCAATCTTACACTCACTCCTACCCCA	1	<lmxll>	ll	2	lm	255		
>ref-153271	ATCAATCTTACACTCACTCCTACCCCA	22	<lmxll>	ll	2	lm	255		
>ref-153286	CATCCACCAACTATTCCTCCTCCACTT	9	<hkxhk>	hh	54	hk	193	kk	3
>ref-153289	TCACTGTTTACGAACCCTCCCTTGCCC	2	<nnxnp>	nn	268	np	0		
>ref-153293	CCCTCTTTCACTCTCCCTCCCACTTAT	23	<hkxhk>	hh	0	hk	270	kk	0
>ref-153293	CCCTCTTTCACTCTCCCTCCCACTTAT	25	<hkxhk>	hh	0	hk	270	kk	0
>ref-153295	TTCCATCTCACCTCTTCTCCCCCTCCT	22	<lmxll>	ll	232	lm	2		
>ref-153295	TTCCATCTCACCTCTTCTCCCCCTCCT	23	<lmxll>	ll	235	lm	1		
>ref-153299	CATATGGCAACGTTTACTCCCCCTACA	5	<lmxll>	ll	126	lm	142		
>ref-153305	GCTGATGGAACTCTTGCTCCTCTTGAT	1	<lmxll>	ll	148	lm	121		
>ref-153317	CTGAACACCACCGGCGCTCCTCTTAGC	26	<lmxll>	ll	104	lm	164		
>ref-153318	TCGTCATATACGTCCACTCCCCTCCCT	4	<hkxhk>	hh	60	hk	142	kk	50
>ref-153332	CCCACCCCCACATATCCTCCTAACCTT	8	<lmxll>	ll	2	lm	259		
>ref-153332	CCCACCCCCACATATCCTCCTAACCTT	27	<lmxll>	ll	2	lm	260		
>ref-153358	TCTGTTTCCACCTTTTCTCCTCCGTCT	8	<lmxll>	ll	130	lm	133		
>ref-153378	CAAGTTCGTACACGTCCTCCATGCAAG	27	<nnxnp>	nn	246	np	0		

>ref-153385	TTCCTTTCCACGTTGGCTCCTTGACTC	24	<nnxnp>	nn	116	np	139		
>ref-153395	CTTCACTCCACCGTCTCTCCTACTCTC	5	<lmxll>	ll	127	lm	130		
>ref-153468	GAATGAAAGACAAAGCCTCCACAGCCA	15	<hkxhk>	hh	40	hk	133	kk	62
>ref-153478	GCCCTGAATACCACGACTCCTCTCATA	7	<nnxnp>	nn	115	np	141		
>ref-153478	GCCCTGAATACCACGACTCCTCTCATA	12	<nnxnp>	nn	115	np	141		
>ref-153483	AGGCCCCTCACCCCTACTCCCCTCAAT	23	<lmxll>	ll	133	lm	128		
>ref-153498	CCCTCCCTCACCTTTTCTCCGTTAGCT	24	<nnxnp>	nn	125	np	138		
>ref-153514	GTACTGCCAACACCACCTCCAATGCCA	5	<nnxnp>	nn	122	np	145		
>ref-153521	CCCCCCCACACACAACCTCCTCTTGAC	26	<nnxnp>	nn	248	np	0		
>ref-153535	TCCAAAAACACTTTCACTCCCACACAC	5	<nnxnp>	nn	109	np	144		
>ref-153535	TCCAAAAACACTTTCACTCCCACACAC	25	<nnxnp>	nn	108	np	145		
>ref-153588	AGCCCCCATACCCTCACTCCCCTTGTA	2	<hkxhk>	hh	0	hk	270	kk	2
>ref-153588	AGCCCCCATACCCTCACTCCCCTTGTA	16	<hkxhk>	hh	2	hk	270	kk	0
>ref-153618	CGCCACGACACGCTTTCTCCTTCTCAT	8	<nnxnp>	nn	130	np	132		
>ref-153626	ACTCTTCATACTCCCTCTCCCACGCCT	2	<lmxll>	ll	149	lm	106		
>ref-153626	ACTCTTCATACTCCCTCTCCCACGCCT	6	<lmxll>	ll	26	lm	223		
>ref-153628	ACACCCCTTACTCCCACTCCCTCCCTA	26	<hkxhk>	hh	8	hk	254	kk	0
>ref-153633	CATTTTCCAACTCGCCCTCCTCTCCTC	5	<lmxll>	ll	123	lm	119		
>ref-153645	TGAGATCACACGCACACTCCACATACT	27	<hkxhk>	hh	54	hk	152	kk	56
>ref-153693	TCACCCCTCACCCTCCCTCCCATTCCC	5	<nnxnp>	nn	247	np	0		
>ref-153693	TCACCCCTCACCCTCCCTCCCATTCCC	9	<lmxll>	ll	124	lm	107		
>ref-153700	CTTTCTTTCACTCCCCCTCCTTTCCCT	4	<lmxll>	ll	241	lm	4		
>ref-153700	CTTTCTTTCACTCCCCCTCCTTTCCCT	14	<lmxll>	ll	242	lm	2		
>ref-153702	AGAGAGTAAACCCAGCCTCCCTTTCCC	3	<nnxnp>	nn	139	np	127		
>ref-153705	CTCGTTTTTACTGCTCCTCCCCTCTTC	12	<lmxll>	ll	118	lm	109		
>ref-153717	TGGATTGATACCTTGTCTCCTCTTTCT	27	<lmxll>	ll	126	lm	135		
>ref-153789	GGACGTTTTACTCTCTCTCCCTCGTTC	22	<lmxll>	ll	133	lm	134		
>ref-153806	CCCTGTGAGACTCTCTCTCCCTCTCCG	8	<hkxhk>	hh	0	hk	273	kk	0
>ref-153815	AACCCGCCGACAAAGCCTCCCATTAAC	27	<nnxnp>	nn	271	np	0		
>ref-153818	ACCCATAACACACAAACTCCAACTTCA	14	<hkxhk>	hh	64	hk	141	kk	59
>ref-153836	CGCAGGCGCACTCCAACTCCACCTACG	1	<nnxnp>	nn	258	np	0		
>ref-153856	TGTCCCTCTACTTTTTCTCCTGCAACC	2	<hkxhk>	hh	0	hk	164	kk	65
>ref-153856	TGTCCCTCTACTTTTTCTCCTGCAACC	26	<hkxhk>	hh	66	hk	164	kk	0
>ref-153878	CCACCGTCTACACCGTCTCCACCTTCG	3	<hkxhk>	hh	0	hk	271	kk	0
>ref-153883	ATTTGCATGACGCTCGCTCCATTTCCG	5	<nnxnp>	nn	222	np	0		
>ref-153899	CTCTCTCCCACCCATTCTCCTCTCGTC	14	<lmxll>	ll	122	lm	131		
>ref-153899	CTCTCTCCCACCCATTCTCCTCTCGTC	15	<lmxll>	ll	124	lm	131		
>ref-153901	CCAACACTTACGACTCCTCCCCTCCCT	2	<nnxnp>	nn	110	np	127		

>ref-153914	TATGCTGCTACAACTACTCCTACTACT	4	<nnxnp>	nn	182	np	50		
>ref-153914	TATGCTGCTACAACTACTCCTACTACT	12	<nnxnp>	nn	175	np	54		
>ref-153928	AGTGGCGTGACTACATCTCCACTAGCT	1	<nnxnp>	nn	120	np	141		
>ref-153957	CCTCCCTTTACGACCTCTCCCTCTTCC	9	<lmxll>	ll	136	lm	120		
>ref-153957	CCTCCCTTTACGACCTCTCCCTCTTCC	15	<lmxll>	ll	190	lm	56		
>ref-153957	CCTCCCTTTACGACCTCTCCCTCTTCC	26	<lmxll>	ll	189	lm	56		
>ref-153965	CACCATCATACATTATCTCCCTTTCTT	2	<nnxnp>	nn	109	np	121		
>ref-153971	TAGAATCGAACTTTGGCTCCCTCATTG	1	<hkxhk>	hh	0	hk	254	kk	9
>ref-153971	TAGAATCGAACTTTGGCTCCCTCATTG	9	<hkxhk>	hh	9	hk	254	kk	0
>ref-153979	CACACAAACACGCCTCCTCCCTCCCCC	6	<nnxnp>	nn	147	np	117		
>ref-154002	AGCTGCTCCACTGCTTCTCCTGTTGAC	9	<hkxhk>	hh	0	hk	273	kk	0
>ref-154017	TATGACATCACACATTCTCCCAATCAT	1	<nnxnp>	nn	107	np	144		
>ref-154027	GGCCGCGAAACTGACCCTCCGCTGTCC	12	<hkxhk>	hh	0	hk	216	kk	9
>ref-154027	GGCCGCGAAACTGACCCTCCGCTGTCC	13	<hkxhk>	hh	0	hk	270	kk	1
>ref-154027	GGCCGCGAAACTGACCCTCCGCTGTCC	21	<lmxll>	ll	72	lm	186		
>ref-154027	GGCCGCGAAACTGACCCTCCGCTGTCC	27	<nnxnp>	nn	180	np	75		
>ref-154031	GTCCGTTACACAGTTACTCCAGTCAAC	5	<nnxnp>	nn	245	np	0		
>ref-154031	GTCCGTTACACAGTTACTCCAGTCAAC	27	<lmxll>	ll	112	lm	117		
>ref-154063	CGCATTGGCACAGATTCTCCAGGGGAC	15	<hkxhk>	hh	74	hk	132	kk	40
>ref-154073	TGCCCCGCGACCGGACCTCCGTCGGGA	6	<hkxhk>	hh	0	hk	273	kk	0
>ref-154073	TGCCCCGCGACCGGACCTCCGTCGGGA	14	<hkxhk>	hh	0	hk	273	kk	0
>ref-154094	ATAACAGCTACGTGCGCTCCCTATATT	13	<hkxhk>	hh	116	hk	138	kk	1
>ref-154095	AAGCAGCTAACAAGTACTCCTTACCCT	4	<lmxll>	ll	132	lm	134		
>ref-154098	TGGTACATCACGCTCTCTCCTTCATTC	15	<hkxhk>	hh	54	hk	136	kk	72
>ref-154103	AGTCCGCGGACAGTTCCTCCGAGAAGC	14	<nnxnp>	nn	122	np	126		
>ref-154104	AACAATCCCACTGTACCTCCCATGTCA	8	<nnxnp>	nn	122	np	116		
>ref-154106	GTGGTGGTGACGGCCACTCCTCCACCG	8	<lmxll>	ll	122	lm	146		
>ref-154106	GTGGTGGTGACGGCCACTCCTCCACCG	24	<lmxll>	ll	122	lm	146		
>ref-154113	GCTATTTATACCCTCCCTCCTACTCCC	2	<lmxll>	ll	118	lm	131		
>ref-154130	TCTCTTAGAACCCTTACTCCAGCCCAG	26	<lmxll>	ll	121	lm	139		
>ref-154134	CACACACACACACAACCTCCCCTCGTT	6	<lmxll>	ll	7	lm	265		
>ref-154134	CACACACACACACAACCTCCCCTCGTT	26	<nnxnp>	nn	273	np	0		
>ref-154140	CTCAAATACACAGATTCTCCCCCGACC	3	<lmxll>	ll	16	lm	235		
>ref-154149	ATTTTTTTAACAGCCACTCCTTCCACT	8	<hkxhk>	hh	0	hk	178	kk	63
>ref-154149	ATTTTTTTAACAGCCACTCCTTCCACT	16	<hkxhk>	hh	63	hk	178	kk	0
>ref-154154	GAATGCCGTACATCTGCTCCTCTCATT	4	<lmxll>	ll	112	lm	149		
>ref-154176	ATTATTATCACCAAGCCTCCTCCTTCT	14	<hkxhk>	hh	57	hk	118	kk	50
>ref-154182	TGTCTGCCAACTTTCACTCCCTCTTCA	13	<hkxhk>	hh	0	hk	265	kk	0

>ref-154244	CCGACAAGCACCATTTCTCCCCCACGT	12	<hkxhk>	hh	53	hk	137	kk	38
>ref-154245	TCTCTCCTCACAACTCCTCCTCCCTCT	2	<lmxll>	ll	234	lm	0		
>ref-154257	GGGGATTTCACCTCTTCTCCTCCCCCT	16	<lmxll>	ll	149	lm	97		
>ref-154257	GGGGATTTCACCTCTTCTCCTCCCCCT	27	<lmxll>	ll	147	lm	96		
>ref-154274	CCTAATGGCACCCTAACTCCCACATAA	24	<hkxhk>	hh	6	hk	266	kk	0
>ref-154281	CTCACGCCCACACTCACTCCCGCCGTT	21	<nnxnp>	nn	129	np	144		
>ref-154282	ATTCCTCACACTAAATCTCCTTCGCAG	27	<lmxll>	ll	143	lm	102		
>ref-154306	CTAACCTCAACCCTCTCTCCTCCCCCA	2	<nnxnp>	nn	131	np	113		
>ref-154364	ACCATGCCAACACCTACTCCTTTCTCT	1	<lmxll>	ll	210	lm	23		
>ref-154364	ACCATGCCAACACCTACTCCTTTCTCT	3	<lmxll>	ll	247	lm	8		
>ref-154373	TGTACGCCCACGCCCTCTCCTTCGCCG	22	<hkxhk>	hh	66	hk	151	kk	48
>ref-154375	AGTCCAGCGACAAGTCCTCCCGGGAAA	23	<nnxnp>	nn	109	np	150		
>ref-154376	CGAACACAAACGCGATCTCCTTTGATC	12	<lmxll>	ll	135	lm	123		
>ref-154376	CGAACACAAACGCGATCTCCTTTGATC	24	<lmxll>	ll	137	lm	121		
>ref-154414	CTGCTAGCGACCACCACTCCCTCCTCC	16	<nnxnp>	nn	145	np	123		
>ref-154427	TCACCATCAACATCAACTCCAACATCA	12	<hkxhk>	hh	22	hk	237	kk	0
>ref-154427	TCACCATCAACATCAACTCCAACATCA	26	<hkxhk>	hh	22	hk	237	kk	0
>ref-154432	TGTTCTCCCACTCTTGCTCCCTCTCTC	2	<nnxnp>	nn	259	np	1		
>ref-154432	TGTTCTCCCACTCTTGCTCCCTCTCTC	8	<nnxnp>	nn	193	np	53		
>ref-154447	TCAGCATAAACGAAGTCTCCCTCCCCT	16	<lmxll>	ll	112	lm	122		
>ref-154465	CCAATCTCTACTACAGCTCCAACACCT	4	<lmxll>	ll	227	lm	28		
>ref-154465	CCAATCTCTACTACAGCTCCAACACCT	13	<hkxhk>	hh	0	hk	273	kk	0
>ref-154465	CCAATCTCTACTACAGCTCCAACACCT	22	<hkxhk>	hh	47	hk	217	kk	0
>ref-154475	GCCTCTTCCACGAGTCCTCCACCTCAA	26	<hkxhk>	hh	0	hk	272	kk	0
>ref-154475	GCCTCTTCCACGAGTCCTCCACCTCAA	27	<hkxhk>	hh	0	hk	273	kk	0
>ref-154487	ACGTGGGCAACACTTGCTCCTCACATT	4	<nnxnp>	nn	141	np	130		
>ref-154494	CTATCAACCACTCACTCTCCCCCTTCC	21	<nnxnp>	nn	269	np	0		
>ref-154494	CTATCAACCACTCACTCTCCCCCTTCC	25	<nnxnp>	nn	261	np	0		
>ref-154497	GAACTCTCCACCTCCTCTCCCCTTCCT	25	<nnxnp>	nn	131	np	94		
>ref-154500	TAATCCCTTACGCCCTCTCCTTCTTCT	1	<lmxll>	ll	142	lm	118		
>ref-154500	TAATCCCTTACGCCCTCTCCTTCTTCT	6	<nnxnp>	nn	141	np	118		
>ref-154500	TAATCCCTTACGCCCTCTCCTTCTTCT	25	<lmxll>	ll	110	lm	145		
>ref-154507	GAATGTACAACACATACTCCGTAAACA	3	<nnxnp>	nn	125	np	122		
>ref-154526	TCATTATTCACCATTTCTCCTTCCTCC	1	<lmxll>	ll	120	lm	112		
>ref-154526	TCATTATTCACCATTTCTCCTTCCTCC	2	<lmxll>	ll	117	lm	113		
>ref-154549	AAGAACGTAACCGAATCTCCTCCGCAG	5	<nnxnp>	nn	150	np	121		
>ref-154553	TTATCCCTGACAGGTGCTCCACTCTGG	1	<lmxll>	ll	2	lm	267		
>ref-154553	TTATCCCTGACAGGTGCTCCACTCTGG	4	<lmxll>	ll	2	lm	267		

>ref-154553	TTATCCCTGACAGGTGCTCCACTCTGG	14	<hkxhk>	hh	5	hk	261	kk	1
>ref-154568	GTACTGTCTACCCTCTCTCCCTCCCTC	3	<nnxnp>	nn	117	np	141		
>ref-154591	AGCGGCGTGACCGAGGCTCCGGCAGAC	1	<hkxhk>	hh	0	hk	198	kk	70
>ref-154608	GAATGCAGTACTTTCTCTCCAAGTGAA	2	<nnxnp>	nn	126	np	107		
>ref-154608	GAATGCAGTACTTTCTCTCCAAGTGAA	14	<nnxnp>	nn	125	np	107		
>ref-154611	CTCCTTATAACCTCCACTCCCCATTCC	1	<lmxll>	ll	122	lm	135		
>ref-154611	CTCCTTATAACCTCCACTCCCCATTCC	7	<lmxll>	ll	122	lm	135		
>ref-154620	TTTCATCCTACTTCTCCTCCCTCCCCT	5	<nnxnp>	nn	15	np	247		
>ref-154620	TTTCATCCTACTTCTCCTCCCTCCCCT	6	<nnxnp>	nn	15	np	247		
>ref-154644	ATTCTCTGCACATGCACTCCGTCTCAG	27	<lmxll>	ll	150	lm	104		
>ref-154655	GTATGTGCAACTGTTTCTCCCCATTTC	16	<lmxll>	ll	120	lm	141		
>ref-154660	TCCTCCCACACACACACTCCTCTTCTC	9	<lmxll>	ll	125	lm	128		
>ref-154661	CAACTCGGGACTACCACTCCTGTGTAA	3	<hkxhk>	hh	0	hk	273	kk	0
>ref-154661	CAACTCGGGACTACCACTCCTGTGTAA	8	<hkxhk>	hh	0	hk	273	kk	0
>ref-154664	GAAACCACGACCTTGGCTCCATACTCT	12	<nnxnp>	nn	129	np	125		
>ref-154683	CACGTCTCCACCTTCCCTCCTCTCCCC	14	<lmxll>	ll	134	lm	137		
>ref-154683	CACGTCTCCACCTTCCCTCCTCTCCCC	24	<lmxll>	ll	134	lm	137		
>ref-154689	ATGCCTTACACACGCACTCCTTACGCA	14	<nnxnp>	nn	115	np	153		
>ref-154692	GCAAAGCAAACAGGGCCTCCCGCCCCG	1	<hkxhk>	hh	0	hk	239	kk	10
>ref-154693	GGCCCTCCCACCTTTCCTCCCTCCCTC	12	<hkxhk>	hh	0	hk	10	kk	263
>ref-154693	GGCCCTCCCACCTTTCCTCCCTCCCTC	13	<nnxnp>	nn	268	np	3		
>ref-154706	TTAACTTTAACACACTCTCCTCTTTCT	4	<lmxll>	ll	105	lm	132		
>ref-154727	CCCTTAACCACTCCCTCTCCAGCCGCT	9	<lmxll>	ll	134	lm	133		
>ref-154743	CCCCCCTCTACACGCTCTCCCTTGGAA	1	<nnxnp>	nn	73	np	162		
>ref-154748	GGTCTTTGAACCAAAGCTCCTTCTGAT	23	<lmxll>	ll	144	lm	119		
>ref-154750	GTTATGTCGACAGCGCCTCCTCTGATA	1	<nnxnp>	nn	273	np	0		
>ref-154756	CAGACAAGCACCTCCCCTCCATTCTCC	16	<nnxnp>	nn	125	np	131		
>ref-154784	CCTCCCACCACTTCCTCTCCACCACCC	8	<lmxll>	ll	141	lm	127		
>ref-154802	ACAAACTCCACAATCTCTCCCGACACC	23	<lmxll>	ll	124	lm	139		
>ref-154804	TGCATCATCACCTTACCTCCCTATTAC	27	<lmxll>	ll	129	lm	140		
>ref-154811	CCCCCTTCTACCCATTCTCCTACCCCC	15	<hkxhk>	hh	0	hk	244	kk	14
>ref-154811	CCCCCTTCTACCCATTCTCCTACCCCC	22	<hkxhk>	hh	12	hk	245	kk	0
>ref-154822	TCCTTCTTGACGCCCCCTCCCTCCCTC	24	<nnxnp>	nn	115	np	132		
>ref-154828	TTTCTTCCCACTTTCTCTCCTCTCTCA	3	<hkxhk>	hh	0	hk	265	kk	1
>ref-154828	TTTCTTCCCACTTTCTCTCCTCTCTCA	14	<hkxhk>	hh	0	hk	236	kk	15
>ref-154828	TTTCTTCCCACTTTCTCTCCTCTCTCA	27	<hkxhk>	hh	1	hk	264	kk	1
>ref-154830	TCTTCATCCACTCTCTCTCCATCTTCC	21	<hkxhk>	hh	1	hk	206	kk	34
>ref-154830	TCTTCATCCACTCTCTCTCCATCTTCC	22	<hkxhk>	hh	32	hk	206	kk	1

>ref-154833	CCCCTCTCCACTACCTCTCCCTTTTCG	13	<nnxnp>	nn	104	np	140		
>ref-154846	TGCATCTCCACAGCCTCTCCTTGCCTC	22	<hkxhk>	hh	0	hk	247	kk	23
>ref-154855	CCTCCTTCGACCTCCGCTCCCACAGAA	23	<lmxll>	ll	137	lm	132		
>ref-154861	AGAGAGATCACCCAAACTCCAACACAG	8	<hkxhk>	hh	1	hk	133	kk	127
>ref-154861	AGAGAGATCACCCAAACTCCAACACAG	22	<hkxhk>	hh	50	hk	143	kk	66
>ref-154875	CACGCCCATACATTCGCTCCTACTCTA	6	<lmxll>	ll	124	lm	139		
>ref-154875	CACGCCCATACATTCGCTCCTACTCTA	22	<hkxhk>	hh	57	hk	156	kk	49
>ref-154875	CACGCCCATACATTCGCTCCTACTCTA	24	<hkxhk>	hh	49	hk	156	kk	58
>ref-154879	TTGTCTCCTACATGCTCTCCTGTCGTT	23	<lmxll>	ll	135	lm	133		
>ref-154882	CGTCCTTGAACCCCCTCTCCCTCTCCT	24	<lmxll>	ll	129	lm	135		
>ref-154893	TCCCTCTTCACTCTTGCTCCTTCTTCT	16	<hkxhk>	hh	0	hk	153	kk	104
>ref-154893	TCCCTCTTCACTCTTGCTCCTTCTTCT	23	<hkxhk>	hh	114	hk	144	kk	0
>ref-154909	TCTCCCCCTACCCCTACTCCTTCTCCT	1	<lmxll>	ll	10	lm	258		
>ref-154909	TCTCCCCCTACCCCTACTCCTTCTCCT	6	<hkxhk>	hh	4	hk	268	kk	0
>ref-154909	TCTCCCCCTACCCCTACTCCTTCTCCT	12	<nnxnp>	nn	29	np	242		
>ref-154909	TCTCCCCCTACCCCTACTCCTTCTCCT	16	<hkxhk>	hh	0	hk	273	kk	0
>ref-154909	TCTCCCCCTACCCCTACTCCTTCTCCT	22	<hkxhk>	hh	0	hk	268	kk	2
>ref-154933	GCCTCTTCCACCCTCTCTCCTTCGTTC	22	<nnxnp>	nn	9	np	259		
>ref-154933	GCCTCTTCCACCCTCTCTCCTTCGTTC	24	<nnxnp>	nn	9	np	259		
>ref-154940	GGAGGTGACACGCAAGCTCCGTTTGGA	2	<nnxnp>	nn	126	np	135		
>ref-154945	CCACCTCGCACGTTCCCTCCGCCCTCT	27	<nnxnp>	nn	136	np	135		
>ref-154949	CGCGGCACGACGGCAGCTCCGTGAATA	26	<lmxll>	ll	142	lm	124		
>ref-154958	TCTCTACCTACTCTTTCTCCCTCTTTT	21	<nnxnp>	nn	122	np	124		
>ref-154976	GGAAATGCCACCTCACCTCCATAGGTG	14	<hkxhk>	hh	28	hk	226	kk	0
>ref-154976	GGAAATGCCACCTCACCTCCATAGGTG	22	<hkxhk>	hh	0	hk	266	kk	2
>ref-154994	AGTTCCTCTACCCCCTCTCCTCTCCTC	15	<hkxhk>	hh	0	hk	198	kk	69
>ref-154994	AGTTCCTCTACCCCCTCTCCTCTCCTC	24	<hkxhk>	hh	0	hk	198	kk	69
>ref-154994	AGTTCCTCTACCCCCTCTCCTCTCCTC	25	<nnxnp>	nn	133	np	135		
>ref-155002	CTTTCAATCACTCCCTCTCCCCTTTCC	21	<nnxnp>	nn	99	np	147		
>ref-155017	AACAAATACACACGTACTCCCTCGGTA	8	<lmxll>	ll	115	lm	151		
>ref-155021	CCCTGCACTACCCTCTCTCCCTCCGAG	1	<lmxll>	ll	120	lm	138		
>ref-155026	GTGCCTCGGACGCTCGCTCCTATCAAC	25	<lmxll>	ll	118	lm	142		
>ref-155026	GTGCCTCGGACGCTCGCTCCTATCAAC	27	<lmxll>	ll	118	lm	142		
>ref-155050	TCTGGTTATACCTTCTCTCCCTCCCTC	1	<hkxhk>	hh	52	hk	140	kk	70
>ref-155050	TCTGGTTATACCTTCTCTCCCTCCCTC	27	<nnxnp>	nn	111	np	156		
>ref-155064	TGGGCTCAGACTAGACCTCCAAAGGAA	2	<nnxnp>	nn	143	np	116		
>ref-155101	ATAATGGACACATATTCTCCTCGGCAC	26	<nnxnp>	nn	125	np	100		
>ref-155127	CTTACTTCGACTCTTTCTCCTATTCCT	22	<lmxll>	ll	1	lm	260		

>ref-155127	CTTACTTCGACTCTTTCTCCTATTCCT	25	<lmxll>	ll	1	lm	260		
>ref-155183	TCCTGTCTCACTTTCCCTCCTTATCAG	8	<lmxll>	ll	128	lm	124		
>ref-155185	TAAAAAACGACTTTCGCTCCTTGCCTT	2	<lmxll>	ll	116	lm	116		
>ref-155200	CCCGGAGCCACGTCGCCTCCATCTGCG	5	<nnxnp>	nn	134	np	128		
>ref-155202	TCCAGCCGCACCCGTCCTCCGGGCAGA	15	<hkxhk>	hh	2	hk	261	kk	3
>ref-155202	TCCAGCCGCACCCGTCCTCCGGGCAGA	21	<hkxhk>	hh	1	hk	174	kk	88
>ref-155232	TTTCCCATCACTTTATCTCCCTGTCCC	7	<lmxll>	ll	137	lm	126		
>ref-155232	TTTCCCATCACTTTATCTCCCTGTCCC	22	<nnxnp>	nn	5	np	264		
>ref-155264	ATATCACCGACCCCTCCTCCGTCTGCC	14	<nnxnp>	nn	133	np	114		
>ref-155264	ATATCACCGACCCCTCCTCCGTCTGCC	25	<nnxnp>	nn	127	np	119		
>ref-155271	CCTCAGACAACGCTTTCTCCTCTCTCT	5	<lmxll>	ll	130	lm	126		
>ref-155271	CCTCAGACAACGCTTTCTCCTCTCTCT	12	<lmxll>	ll	130	lm	125		
>ref-155309	TTATACACTACAGAGGCTCCTTGTTCG	6	<nnxnp>	nn	137	np	120		
>ref-155339	CGTCTATCAACACCAACTCCTCCTTTT	2	<lmxll>	ll	158	lm	100		
>ref-155339	CGTCTATCAACACCAACTCCTCCTTTT	23	<nnxnp>	nn	143	np	124		
>ref-155339	CGTCTATCAACACCAACTCCTCCTTTT	25	<nnxnp>	nn	142	np	124		
>ref-155339	CGTCTATCAACACCAACTCCTCCTTTT	26	<lmxll>	ll	158	lm	101		
>ref-155344	AGGCATGGAACATCCTCTCCTTGCATC	1	<nnxnp>	nn	118	np	151		
>ref-155396	TTCATCCTCACTCTCTCTCCTCCTGCC	5	<lmxll>	ll	260	lm	0		
>ref-155396	TTCATCCTCACTCTCTCTCCTCCTGCC	6	<nnxnp>	nn	127	np	121		
>ref-155396	TTCATCCTCACTCTCTCTCCTCCTGCC	16	<nnxnp>	nn	127	np	121		
>ref-155396	TTCATCCTCACTCTCTCTCCTCCTGCC	25	<lmxll>	ll	250	lm	0		
>ref-155410	CAGTCCATTACTACGACTCCTTATCAC	27	<hkxhk>	hh	217	hk	54	kk	0
>ref-155425	ATTAAGCTCACAATCCCTCCTTCAGCT	21	<hkxhk>	hh	0	hk	269	kk	2
>ref-155444	TTAGGGTGTACTCGCTCTCCTTCTCCC	14	<nnxnp>	nn	130	np	121		
>ref-155466	TTCACGAATACGAGTTCTCCTCAGTAT	26	<lmxll>	ll	109	lm	131		
>ref-155477	CAATTTCTTACTACCTCTCCCTTCCTC	9	<lmxll>	ll	117	lm	116		
>ref-155481	GTGCGCGCTACAGTGACTCCACTCCTC	2	<lmxll>	ll	132	lm	136		
>ref-155490	ATTCTCACTACCCCCCCTCCTTCCCTC	22	<lmxll>	ll	113	lm	122		
>ref-155490	ATTCTCACTACCCCCCCTCCTTCCCTC	26	<lmxll>	ll	114	lm	120		
>ref-155507	CAACCACTCACCGCCTCTCCCTCCCTT	13	<lmxll>	ll	138	lm	127		
>ref-155559	AGTATATTAACATCCCCTCCACTTATG	14	<nnxnp>	nn	6	np	224		
>ref-155561	CATATACACACACTGTCTCCATACACA	13	<lmxll>	ll	146	lm	126		
>ref-155566	TTTCCCTTAACCTCCTCTCCGCTTCGA	16	<nnxnp>	nn	115	np	106		
>ref-155571	CACAGGGTAACAGCATCTCCTGTGTTG	15	<hkxhk>	hh	56	hk	149	kk	55
>ref-155576	ACCCAAGCAACCATTCCTCCCTCGGTC	16	<nnxnp>	nn	272	np	0		
>ref-155582	TCCTTTCACACACATCCTCCCTCTCCC	25	<nnxnp>	nn	139	np	128		
>ref-155585	CGTCCCCTCACCTCCCCTCCATGTCAC	9	<nnxnp>	nn	265	np	0		

>ref-155591	CAGAATACCACTTTCACTCCATCCTAA	27	<lmxll>	ll	118	lm	108		
>ref-155626	GATTGGCCCACTTTGGCTCCTCTTGTG	15	<lmxll>	ll	136	lm	135		
>ref-155628	TATTCCCTCACATACTCTCCACCTCCA	22	<lmxll>	ll	129	lm	136		
>ref-155652	TTCCCTCTTACTTCTCCTCCCCATTCC	3	<nnxnp>	nn	125	np	131		
>ref-155653	CTAACCCCAACCCCCACTCCCATACCC	1	<hkxhk>	hh	113	hk	108	kk	0
>ref-155653	CTAACCCCAACCCCCACTCCCATACCC	2	<lmxll>	ll	98	lm	125		
>ref-155653	CTAACCCCAACCCCCACTCCCATACCC	21	<hkxhk>	hh	93	hk	149	kk	0
>ref-155653	CTAACCCCAACCCCCACTCCCATACCC	22	<hkxhk>	hh	2	hk	254	kk	4
>ref-155670	AAGCACATAACTACTTCTCCTCGTTGC	24	<nnxnp>	nn	118	np	114		
>ref-155671	TTCCCCGTCACCCCTCCTCCTCCCCCC	1	<nnxnp>	nn	77	np	147		
>ref-155671	TTCCCCGTCACCCCTCCTCCTCCCCCC	9	<nnxnp>	nn	35	np	209		
>ref-155687	TCCCTTTCCACAATCCCTCCTCCGCCC	8	<lmxll>	ll	118	lm	141		
>ref-155690	AAAGTCTCTACTCACACTCCTGCACAT	15	<nnxnp>	nn	156	np	114		
>ref-155690	AAAGTCTCTACTCACACTCCTGCACAT	16	<nnxnp>	nn	105	np	157		
>ref-155691	AGGTACCTTACATGCCCTCCCCTGCTA	1	<hkxhk>	hh	1	hk	269	kk	2
>ref-155691	AGGTACCTTACATGCCCTCCCCTGCTA	24	<lmxll>	ll	2	lm	271		
>ref-155691	AGGTACCTTACATGCCCTCCCCTGCTA	26	<lmxll>	ll	7	lm	259		
>ref-155696	CTCACTTTCACGCTCGCTCCCTAACCC	21	<lmxll>	ll	109	lm	131		
>ref-155701	GAGGCTGCAACACCCCCTCCCCCCTCT	4	<lmxll>	ll	116	lm	140		
>ref-155703	CCCTGTGATACAGCCTCTCCATTCCCT	6	<hkxhk>	hh	47	hk	150	kk	59
>ref-155706	TGCACTAGAACAGATGCTCCTTCCTGT	8	<hkxhk>	hh	35	hk	130	kk	57
>ref-155723	CCAACAGGAACACCAACTCCAACAGAA	8	<hkxhk>	hh	0	hk	262	kk	2
>ref-155723	CCAACAGGAACACCAACTCCAACAGAA	16	<hkxhk>	hh	2	hk	262	kk	0
>ref-155727	TCTATAAATACAGATCCTCCTTAGCAC	6	<hkxhk>	hh	9	hk	197	kk	18
>ref-155734	AGAGACTTTACCGTATCTCCTCCTCTG	13	<lmxll>	ll	110	lm	121		
>ref-155753	TTACTATACACCATAACTCCACTTACT	6	<nnxnp>	nn	113	np	136		
>ref-155783	TCCTATCCAACTTTCCCTCCGCCTCTC	27	<lmxll>	ll	144	lm	118		
>ref-155787	CCAACCTCCACCACTCCTCCTCTCCTT	3	<nnxnp>	nn	104	np	116		
>ref-155787	CCAACCTCCACCACTCCTCCTCTCCTT	9	<nnxnp>	nn	237	np	1		
>ref-155812	CCATCCAAAACCATCTCTCCCTTTTCT	12	<hkxhk>	hh	52	hk	126	kk	68
>ref-155879	AGCGAATGAACTCCTCCTCCTGATGTT	12	<lmxll>	ll	121	lm	146		
>ref-155890	AACATACACACAACCCCTCCCCTCACT	3	<lmxll>	ll	124	lm	116		
>ref-155895	ATACCCATCACTTGTCCTCCACTGTAC	15	<lmxll>	ll	122	lm	132		
>ref-155899	CTCGCTCACACTTGCCCTCCCAGTCAC	2	<lmxll>	ll	123	lm	125		
>ref-155930	CTCTCTCTCACTCTTTCTCCTAACCCC	15	<hkxhk>	hh	2	hk	258	kk	6
>ref-155930	CTCTCTCTCACTCTTTCTCCTAACCCC	24	<lmxll>	ll	165	lm	91		
>ref-155989	ATGGCGATAACCATAACTCCGTTACAT	5	<lmxll>	ll	139	lm	122		
>ref-155991	AAATACATTACATTGCCTCCACGCACA	23	<hkxhk>	hh	50	hk	140	kk	71

>ref-155992	TGACCCCCAACCCCCCCTCCTCCGCAT	24	<lmxll>	ll	228	lm	0		
>ref-156000	GCCCCCCTCACCCCCTCTCCATCAACG	1	<hkxhk>	hh	1	hk	262	kk	3
>ref-156000	GCCCCCCTCACCCCCTCTCCATCAACG	24	<hkxhk>	hh	3	hk	262	kk	1
>ref-156016	GCACCGCCAACAGTGGCTCCAACACCT	3	<hkxhk>	hh	73	hk	189	kk	0
>ref-156016	GCACCGCCAACAGTGGCTCCAACACCT	6	<hkxhk>	hh	0	hk	272	kk	0
>ref-156016	GCACCGCCAACAGTGGCTCCAACACCT	14	<hkxhk>	hh	0	hk	272	kk	0
>ref-156016	GCACCGCCAACAGTGGCTCCAACACCT	25	<hkxhk>	hh	0	hk	271	kk	1
>ref-156079	AGAGAGAGAACGAATGCTCCATGGGGC	21	<lmxll>	ll	129	lm	124		
>ref-156091	CCTGTCGCAACGCCTACTCCTAGAGCC	1	<lmxll>	ll	119	lm	147		
>ref-156091	CCTGTCGCAACGCCTACTCCTAGAGCC	16	<nnxnp>	nn	96	np	171		
>ref-156118	TATTCCCATACCCTCACTCCCCTTCCT	16	<lmxll>	ll	128	lm	123		
>ref-156142	ATAGTCAGTACATAGTCTCCATGTAGA	4	<hkxhk>	hh	1	hk	255	kk	3
>ref-156173	CTCCTTCCCACTTCATCTCCTGTTTAT	3	<nnxnp>	nn	235	np	1		
>ref-156198	TCCACCCCCACCCCCTCTCCTCCACCC	21	<nnxnp>	nn	9	np	236		
>ref-156198	TCCACCCCCACCCCCTCTCCTCCACCC	22	<nnxnp>	nn	10	np	236		
>ref-156199	ACTCTCACAACCCCTACTCCTTCCTCC	1	<lmxll>	ll	126	lm	130		
>ref-156212	AGCAAATGAACAGCGCCTCCATTCGGT	15	<hkxhk>	hh	78	hk	121	kk	47
>ref-156232	AGCGCTGCCACCTCGCCTCCCGCCCCG	23	<nnxnp>	nn	134	np	120		
>ref-156234	TTCTCTCTCACCTTTCCTCCCTCACGT	6	<nnxnp>	nn	252	np	4		
>ref-156234	TTCTCTCTCACCTTTCCTCCCTCACGT	26	<nnxnp>	nn	247	np	2		
>ref-156242	ATGCCCTCTACCCATCCTCCCTCATTC	21	<hkxhk>	hh	65	hk	149	kk	33
>ref-156243	TGCACCGGAACTGTATCTCCCCAACAG	6	<hkxhk>	hh	0	hk	273	kk	0
>ref-156243	TGCACCGGAACTGTATCTCCCCAACAG	24	<hkxhk>	hh	25	hk	237	kk	0
>ref-156271	CGTCTGTCTACCCTCCCTCCTCGTACC	8	<hkxhk>	hh	51	hk	138	kk	46
>ref-156287	GTCTCGTTCACTGTCTCTCCCTCTGTT	1	<nnxnp>	nn	104	np	163		
>ref-156306	CCTTTATCCACTGTCCCTCCTCCGTCT	24	<nnxnp>	nn	107	np	135		
>ref-156306	CCTTTATCCACTGTCCCTCCTCCGTCT	25	<nnxnp>	nn	108	np	136		
>ref-156309	TCTCCCCATACTTCCTCTCCCATTCCT	8	<hkxhk>	hh	64	hk	177	kk	1
>ref-156309	TCTCCCCATACTTCCTCTCCCATTCCT	21	<hkxhk>	hh	64	hk	178	kk	1
>ref-156315	TCCCCGCCGACCAACGCTCCCTTTCCC	7	<nnxnp>	nn	117	np	153		
>ref-156315	TCCCCGCCGACCAACGCTCCCTTTCCC	23	<lmxll>	ll	148	lm	124		
>ref-156335	CCAAGGCTAACAGAGGCTCCTCCGTTG	15	<nnxnp>	nn	111	np	158		
>ref-156335	CCAAGGCTAACAGAGGCTCCTCCGTTG	16	<nnxnp>	nn	110	np	158		
>ref-156336	TCCTATTCTACTTTCCCTCCGTCCCCC	3	<hkxhk>	hh	1	hk	263	kk	0
>ref-156365	TTCCCTCTGACAGCTGCTCCTCCTCCC	1	<nnxnp>	nn	152	np	112		
>ref-156376	CGCACACGCACTCGCACTCCGATCGCA	6	<hkxhk>	hh	75	hk	193	kk	0
>ref-156376	CGCACACGCACTCGCACTCCGATCGCA	15	<lmxll>	ll	137	lm	133		
>ref-156378	CCACACTCAACCACAGCTCCACCACTC	12	<nnxnp>	nn	120	np	135		

>ref-156381	ACTGGATTAACCTGGTCTCCTAAGGCA	22	<nnxnp>	nn	248	np	0		
>ref-156384	ACAGAATTCACTAACACTCCTCACACA	13	<nnxnp>	nn	158	np	109		
>ref-156403	TTGTTGACGACCTCATCTCCATAGGAT	8	<lmxll>	ll	139	lm	94		
>ref-156403	TTGTTGACGACCTCATCTCCATAGGAT	26	<nnxnp>	nn	254	np	0		
>ref-156410	CTCCCTTCTACATCTTCTCCATCTCTC	27	<lmxll>	ll	263	lm	0		
>ref-156430	CACAGACTCACCAACGCTCCCTCACCA	7	<lmxll>	ll	134	lm	135		
>ref-156436	TCCCCTCCCACCTCGTCTCCTGATGCC	25	<nnxnp>	nn	241	np	8		
>ref-156436	TCCCCTCCCACCTCGTCTCCTGATGCC	27	<nnxnp>	nn	241	np	8		
>ref-156450	ATAAGCCGCACTTGGCCTCCTCTTCTG	23	<nnxnp>	nn	145	np	125		
>ref-156476	TTTTTCTGTACTCTCTCTCCCTCCCTC	4	<hkxhk>	hh	0	hk	193	kk	56
>ref-156476	TTTTTCTGTACTCTCTCTCCCTCCCTC	8	<hkxhk>	hh	56	hk	196	kk	0
>ref-156483	TCATCAGCGACTACTTCTCCTTCTCCT	4	<lmxll>	ll	128	lm	141		
>ref-156484	ATACCGCAAACATTCACTCCAACATAG	27	<nnxnp>	nn	223	np	0		
>ref-156490	ACATTTGACACAGTCCCTCCTCCGCCA	25	<lmxll>	ll	118	lm	139		
>ref-156498	GGCCTATCTACCACCTCTCCCTTCCCC	2	<lmxll>	ll	272	lm	0		
>ref-156498	GGCCTATCTACCACCTCTCCCTTCCCC	12	<nnxnp>	nn	126	np	122		
>ref-156508	GTCACTTTCACTTATCCTCCTTTTCAT	15	<nnxnp>	nn	122	np	137		
>ref-156521	ACCATCTCTACCCTCCCTCCTGCTCCA	1	<lmxll>	ll	133	lm	128		
>ref-156521	ACCATCTCTACCCTCCCTCCTGCTCCA	27	<lmxll>	ll	134	lm	127		
>ref-156529	ATTCTTTACACCATCCCTCCCTTCGTA	9	<lmxll>	ll	104	lm	151		
>ref-156551	CAGCCTGAGACACTTCCTCCTCCATGA	22	<lmxll>	ll	122	lm	127		
>ref-156552	TAGTTCATTACACCCTCTCCTTCTCCC	21	<nnxnp>	nn	111	np	152		
>ref-156552	TAGTTCATTACACCCTCTCCTTCTCCC	27	<nnxnp>	nn	112	np	151		
>ref-156559	GCTTCCGTCACGCTAGCTCCTTCCTCC	6	<lmxll>	ll	124	lm	126		
>ref-156561	CATTCTAGCACATATTCTCCCTTGAAC	7	<lmxll>	ll	119	lm	126		
>ref-156577	AGGCCAATTACTTCCGCTCCCTCCCTT	14	<nnxnp>	nn	116	np	119		
>ref-156585	GGGAAGGCAACGACCTCTCCTCATCCT	26	<nnxnp>	nn	132	np	135		
>ref-156617	TAAGCATCGACCCTCGCTCCTCCGAAG	13	<lmxll>	ll	112	lm	130		
>ref-156622	CACCCTTCCACCCCCCCTCCACCTGGA	1	<nnxnp>	nn	85	np	145		
>ref-156646	TCCATCTCAACCCAACCTCCTCTCTTC	26	<nnxnp>	nn	127	np	137		
>ref-156672	CGGCGGGCAACCATCCCTCCATCCCAC	1	<nnxnp>	nn	130	np	129		
>ref-156676	ACCTCTTTAACGTGCTCTCCCCAGGAA	1	<lmxll>	ll	136	lm	97		
>ref-156680	CTCCCACTCACCTTCCCTCCCTTGTAT	7	<hkxhk>	hh	58	hk	128	kk	72
>ref-156683	AGACGTTGAACTTGTACTCCTAGCTAT	6	<lmxll>	ll	131	lm	123		
>ref-156691	CTTGAAGTTACGCTAGCTCCAGCCATC	14	<lmxll>	ll	135	lm	137		
>ref-156703	ATTACTACCACCCTCCCTCCCTCCTTC	3	<nnxnp>	nn	245	np	2		
>ref-156707	ACTCCACCAACGGGGCCTCCGAAGGAA	12	<hkxhk>	hh	0	hk	271	kk	1
>ref-156707	ACTCCACCAACGGGGCCTCCGAAGGAA	24	<hkxhk>	hh	1	hk	175	kk	72

>ref-156707	ACTCCACCAACGGGGCCTCCGAAGGAA	27	<hkxhk>	hh	72	hk	174	kk	1
>ref-156708	TCCCTCCCCACACCTTCTCCCTACACT	2	<hkxhk>	hh	61	hk	139	kk	56
>ref-156709	ATCGTAGGAACATAAGCTCCGGGGCCC	4	<nnxnp>	nn	144	np	109		
>ref-156727	AGAGGACTGACCGCTCCTCCACTACGT	13	<lmxll>	ll	139	lm	130		
>ref-156730	ATTGTAACCACTCAACCTCCGTCAGGG	7	<nnxnp>	nn	140	np	124		
>ref-156738	TCTCGCAATACCTTTTCTCCATGTACA	26	<nnxnp>	nn	257	np	1		
>ref-156754	ACCTGTATGACCGTTCCTCCTTTTTCA	26	<nnxnp>	nn	139	np	128		
>ref-156754	ACCTGTATGACCGTTCCTCCTTTTTCA	27	<nnxnp>	nn	138	np	128		
>ref-156778	TGTTTAAATACTTGAACTCCTTGCGTC	6	<lmxll>	ll	131	lm	106		
>ref-156795	TATCGCTAAACTAATTCTCCATATAAA	25	<lmxll>	ll	110	lm	128		
>ref-156808	CCCCCCCTTACTCCCTCTCCCCCATCC	2	<hkxhk>	hh	1	hk	194	kk	49
>ref-156808	CCCCCCCTTACTCCCTCTCCCCCATCC	6	<hkxhk>	hh	2	hk	269	kk	0
>ref-156808	CCCCCCCTTACTCCCTCTCCCCCATCC	8	<hkxhk>	hh	0	hk	71	kk	186
>ref-156808	CCCCCCCTTACTCCCTCTCCCCCATCC	12	<lmxll>	ll	198	lm	56		
>ref-156808	CCCCCCCTTACTCCCTCTCCCCCATCC	22	<hkxhk>	hh	2	hk	269	kk	0
>ref-156813	TATTCACTCACTCTCTCTCCTTCTCGT	14	<lmxll>	ll	259	lm	0		
>ref-156828	TTCACCCTCACCCACTCTCCCCTCCCC	3	<lmxll>	ll	267	lm	1		
>ref-156828	TTCACCCTCACCCACTCTCCCCTCCCC	7	<nnxnp>	nn	213	np	41		
>ref-156828	TTCACCCTCACCCACTCTCCCCTCCCC	14	<nnxnp>	nn	26	np	235		
>ref-156828	TTCACCCTCACCCACTCTCCCCTCCCC	15	<lmxll>	ll	68	lm	187		
>ref-156828	TTCACCCTCACCCACTCTCCCCTCCCC	16	<lmxll>	ll	269	lm	2		
>ref-156846	CCCTCTCCTACTCTCGCTCCCACAACC	22	<lmxll>	ll	105	lm	147		
>ref-156847	TCCAACCCTACCCCATCTCCCCATCTT	22	<lmxll>	ll	117	lm	106		
>ref-156880	TCCCATCCTACCATCTCTCCCTCCTAG	4	<lmxll>	ll	211	lm	43		
>ref-156880	TCCCATCCTACCATCTCTCCCTCCTAG	5	<nnxnp>	nn	100	np	137		
>ref-156880	TCCCATCCTACCATCTCTCCCTCCTAG	13	<nnxnp>	nn	100	np	138		
>ref-156880	TCCCATCCTACCATCTCTCCCTCCTAG	25	<lmxll>	ll	258	lm	1		
>ref-156884	CTCTCCCATACTTCCTCTCCTCCATCC	15	<nnxnp>	nn	136	np	112		
>ref-156903	CCCTTACGGACGGATCCTCCAGTTGTC	1	<nnxnp>	nn	263	np	0		
>ref-156903	CCCTTACGGACGGATCCTCCAGTTGTC	3	<nnxnp>	nn	266	np	0		
>ref-156908	AGACACACTACTAACCCTCCTTTTCCC	16	<nnxnp>	nn	117	np	139		
>ref-156913	GTGTGTGTAACTCTTCCTCCCTCTCTC	21	<nnxnp>	nn	270	np	0		
>ref-156913	GTGTGTGTAACTCTTCCTCCCTCTCTC	25	<nnxnp>	nn	270	np	0		
>ref-156932	ATCATCACAACTATCTCTCCCACTACT	12	<hkxhk>	hh	50	hk	135	kk	46
>ref-156979	AGGACACGGACGCAGCCTCCGTGTCCC	27	<lmxll>	ll	263	lm	0		
>ref-156982	TGCTCAGATACTCACTCTCCATCACGA	14	<nnxnp>	nn	119	np	122		
>ref-156985	TTACCCCTAACCTCCACTCCTTCCCTC	25	<nnxnp>	nn	114	np	139		
>ref-157022	TGATGGTAAACAGAATCTCCTAACGGT	12	<hkxhk>	hh	79	hk	157	kk	2

>ref-157022	TGATGGTAAACAGAATCTCCTAACGGT	26	<hkxhk>	hh	2	hk	252	kk	4
>ref-157026	AGATAAATAACTTGACCTCCGTCGTAT	16	<lmxll>	ll	114	lm	142		
>ref-157040	ATGAGTGATACCCTAGCTCCCCCTTCT	15	<hkxhk>	hh	48	hk	160	kk	53
>ref-157044	CAATCCTCCACCATCTCTCCCTCACCA	2	<hkxhk>	hh	59	hk	212	kk	0
>ref-157044	CAATCCTCCACCATCTCTCCCTCACCA	7	<lmxll>	ll	57	lm	190		
>ref-157044	CAATCCTCCACCATCTCTCCCTCACCA	8	<lmxll>	ll	79	lm	184		
>ref-157044	CAATCCTCCACCATCTCTCCCTCACCA	12	<lmxll>	ll	54	lm	196		
>ref-157055	CCTCTTTTCACTCCCTCTCCCCCCTTC	22	<hkxhk>	hh	0	hk	268	kk	2
>ref-157055	CCTCTTTTCACTCCCTCTCCCCCCTTC	23	<hkxhk>	hh	0	hk	269	kk	2
>ref-157063	AACAATAATACTCCATCTCCCTTTCCC	15	<nnxnp>	nn	148	np	107		
>ref-157063	AACAATAATACTCCATCTCCCTTTCCC	23	<nnxnp>	nn	147	np	108		
>ref-157073	TCCTGTAAAACATCCTCTCCGTTGCGT	23	<lmxll>	ll	123	lm	143		
>ref-157092	TCAGCAGTGACGGCTGCTCCTGTAGCA	1	<lmxll>	ll	68	lm	175		
>ref-157092	TCAGCAGTGACGGCTGCTCCTGTAGCA	5	<hkxhk>	hh	2	hk	268	kk	0
>ref-157092	TCAGCAGTGACGGCTGCTCCTGTAGCA	13	<hkxhk>	hh	0	hk	253	kk	8
>ref-157097	CACACATATACACCCACTCCCCATAAA	27	<hkxhk>	hh	0	hk	272	kk	0
>ref-157108	CACATACACACGCGCCCTCCATAAACA	2	<nnxnp>	nn	116	np	126		
>ref-157108	CACATACACACGCGCCCTCCATAAACA	27	<nnxnp>	nn	139	np	109		
>ref-157117	CCTTCCATCACCCCTCCTCCTCTTCCA	3	<hkxhk>	hh	1	hk	263	kk	1
>ref-157117	CCTTCCATCACCCCTCCTCCTCTTCCA	23	<hkxhk>	hh	1	hk	263	kk	1
>ref-157118	CTTACCCTTACCTCTGCTCCCTTTCTT	23	<lmxll>	ll	120	lm	124		
>ref-157159	TTACCCTGCACCTCCCCTCCCTATTCC	27	<nnxnp>	nn	129	np	122		
>ref-157185	ATCACTAACACTCGCTCTCCCTCTCCT	23	<lmxll>	ll	160	lm	111		
>ref-157185	ATCACTAACACTCGCTCTCCCTCTCCT	25	<lmxll>	ll	105	lm	164		
>ref-157186	TAAAACCTGACGCACACTCCCCTGCCT	22	<nnxnp>	nn	127	np	135		
>ref-157190	CTCTGCGCCACGTCGTCTCCTCCCACA	1	<lmxll>	ll	124	lm	145		
>ref-157195	TCCTTCTTCACCTCTTCTCCTTCTCCC	4	<lmxll>	ll	71	lm	197		
>ref-157195	TCCTTCTTCACCTCTTCTCCTTCTCCC	7	<lmxll>	ll	62	lm	201		
>ref-157195	TCCTTCTTCACCTCTTCTCCTTCTCCC	9	<lmxll>	ll	63	lm	200		
>ref-157195	TCCTTCTTCACCTCTTCTCCTTCTCCC	25	<lmxll>	ll	12	lm	254		
>ref-157211	CCTTCCGACACAGACTCTCCTTCCGAC	6	<lmxll>	ll	126	lm	142		
>ref-157211	CCTTCCGACACAGACTCTCCTTCCGAC	25	<lmxll>	ll	127	lm	135		
>ref-157226	ATCGACCTGACCTTACCTCCCCTCAAC	21	<nnxnp>	nn	104	np	149		
>ref-157235	TTCCATCAAACCCTCCCTCCCTCCTCC	3	<lmxll>	ll	256	lm	1		
>ref-157235	TTCCATCAAACCCTCCCTCCCTCCTCC	26	<nnxnp>	nn	250	np	2		
>ref-157237	CAATCTGATACTTTTCCTCCTTCTCTT	2	<nnxnp>	nn	125	np	137		
>ref-157239	GAGATGGAGACTGAGACTCCATGATGG	5	<hkxhk>	hh	0	hk	272	kk	0
>ref-157239	GAGATGGAGACTGAGACTCCATGATGG	14	<lmxll>	ll	127	lm	141		

>ref-157239	GAGATGGAGACTGAGACTCCATGATGG	16	<hkxhk>	hh	31	hk	229	kk	0
>ref-157249	TGTTGTCCGACTGAACCTCCATGCCAC	5	<lmxll>	ll	137	lm	129		
>ref-157282	TTTTCTGTCACTACATCTCCTTCATAA	23	<hkxhk>	hh	75	hk	129	kk	52
>ref-157306	CCACGCGGTACAGCCTCTCCCTCTCGG	26	<lmxll>	ll	262	lm	1		
>ref-157314	CTCTTCCTTACCCCTTCTCCTCTTATC	9	<hkxhk>	hh	55	hk	149	kk	52
>ref-157324	GGAAGATCGACATACTCTCCTTGTAAG	1	<nnxnp>	nn	128	np	114		
>ref-157324	GGAAGATCGACATACTCTCCTTGTAAG	4	<nnxnp>	nn	127	np	114		
>ref-157330	CCCGCCTGTACCCTCCCTCCCCTACCC	8	<hkxhk>	hh	15	hk	216	kk	4
>ref-157330	CCCGCCTGTACCCTCCCTCCCCTACCC	14	<nnxnp>	nn	38	np	187		
>ref-157339	ACTTGTACCACGAGAGCTCCAGCGTTA	16	<nnxnp>	nn	118	np	147		
>ref-157339	ACTTGTACCACGAGAGCTCCAGCGTTA	21	<nnxnp>	nn	116	np	147		
>ref-157350	CAGAAGGGGACCCATTCTCCGCCGCCT	8	<lmxll>	ll	116	lm	152		
>ref-157358	TGCTCACAGACATTTTCTCCTTCATGA	27	<lmxll>	ll	139	lm	129		
>ref-157380	CCTTCCCTCACCCCCTCTCCCCCTTTC	1	<nnxnp>	nn	258	np	7		
>ref-157380	CCTTCCCTCACCCCCTCTCCCCCTTTC	2	<hkxhk>	hh	90	hk	167	kk	0
>ref-157380	CCTTCCCTCACCCCCTCTCCCCCTTTC	3	<lmxll>	ll	1	lm	270		
>ref-157380	CCTTCCCTCACCCCCTCTCCCCCTTTC	4	<hkxhk>	hh	0	hk	240	kk	24
>ref-157380	CCTTCCCTCACCCCCTCTCCCCCTTTC	14	<hkxhk>	hh	0	hk	273	kk	0
>ref-157380	CCTTCCCTCACCCCCTCTCCCCCTTTC	22	<hkxhk>	hh	10	hk	260	kk	0
>ref-157415	AACAGACACACGTTCTCTCCCTCTATT	27	<lmxll>	ll	103	lm	156		
>ref-157420	TGCCATTTAACACAGACTCCATTTCCC	1	<nnxnp>	nn	229	np	0		
>ref-157430	ACGTCTCTTACTTTGCCTCCAAGATGT	2	<lmxll>	ll	122	lm	136		
>ref-157436	TATTCCCCCACTTCCCCTCCCCTCTCC	6	<nnxnp>	nn	218	np	2		
>ref-157436	TATTCCCCCACTTCCCCTCCCCTCTCC	7	<nnxnp>	nn	222	np	3		
>ref-157436	TATTCCCCCACTTCCCCTCCCCTCTCC	9	<nnxnp>	nn	226	np	2		
>ref-157457	TTACAGTTCACAGAAGCTCCAACGCTA	27	<lmxll>	ll	126	lm	141		
>ref-157458	TACACTGGCACACCACCTCCGCAAGCA	14	<lmxll>	ll	133	lm	138		
>ref-157469	TTCTGCTGCACACCACCTCCCACCAAG	22	<nnxnp>	nn	171	np	84		
>ref-157469	TTCTGCTGCACACCACCTCCCACCAAG	27	<nnxnp>	nn	170	np	85		
>ref-157484	AAACTTCAAACTTCACCTCCCCCTCCC	5	<nnxnp>	nn	261	np	3		
>ref-157495	CGATCTTTCACTCTCCCTCCCTCTTTC	3	<hkxhk>	hh	0	hk	213	kk	48
>ref-157495	CGATCTTTCACTCTCCCTCCCTCTTTC	16	<hkxhk>	hh	65	hk	200	kk	0
>ref-157495	CGATCTTTCACTCTCCCTCCCTCTTTC	25	<lmxll>	ll	112	lm	148		
>ref-157504	AAATAAACTACGCCGTCTCCTATCCCT	4	<nnxnp>	nn	106	np	115		
>ref-157512	GCTCTACTGACCCCACCTCCCCCTCTG	1	<nnxnp>	nn	104	np	137		
>ref-157516	ACGATTCCAACTGTATCTCCCTGAAGA	2	<lmxll>	ll	118	lm	129		
>ref-157537	CCTCAAGCCACAGCTCCTCCGTCACAC	26	<nnxnp>	nn	273	np	0		
>ref-157544	TTTAATTTAACCCTCCCTCCCTCTCCC	4	<nnxnp>	nn	139	np	116		

>ref-157544	TTTAATTTAACCCTCCCTCCCTCTCCC	6	<nnxnp>	nn	139	np	116		
>ref-157544	TTTAATTTAACCCTCCCTCCCTCTCCC	21	<hkxhk>	hh	5	hk	235	kk	8
>ref-157576	CTGTTTATAACGCATCCTCCTCCTCCT	26	<hkxhk>	hh	76	hk	128	kk	60
>ref-157576	CTGTTTATAACGCATCCTCCTCCTCCT	27	<hkxhk>	hh	59	hk	130	kk	73
>ref-157579	AGAGGTAGAACACTCACTCCATGAATT	7	<lmxll>	ll	115	lm	127		
>ref-157589	TATTCTTCTACTGCCCCTCCTCCCCCT	24	<nnxnp>	nn	220	np	5		
>ref-157617	ACCAATGCAACAGGTCCTCCTCTCTAT	13	<nnxnp>	nn	125	np	146		
>ref-157624	TAGAAAAACACACACACTCCGTCACTC	26	<nnxnp>	nn	117	np	124		
>ref-157646	AACAACGTAACCTCTCCTCCCTCGAAA	24	<lmxll>	ll	87	lm	142		
>ref-157655	CAAACCCGAACTCGCCCTCCAACTCGC	15	<nnxnp>	nn	130	np	138		
>ref-157659	CTTCGTGTGACTGTTGCTCCATTCCTT	7	<hkxhk>	hh	55	hk	158	kk	50
>ref-157690	CCCTTCTTCACGCTTTCTCCTTTGTCC	5	<nnxnp>	nn	126	np	144		
>ref-157698	CCTTCTTTGACCTTCCCTCCATCCCTC	27	<nnxnp>	nn	130	np	125		
>ref-157701	CAACAGGTGACCGTGGCTCCATACGTG	13	<hkxhk>	hh	61	hk	151	kk	54
>ref-157703	ATAGCCTACACATTATCTCCGCATGAG	21	<nnxnp>	nn	112	np	133		
>ref-157712	GTGACAAACACTCACACTCCTGTCTGT	1	<nnxnp>	nn	138	np	132		
>ref-157713	ATCTCTCCCACTTTCTCTCCTCTTCTA	1	<hkxhk>	hh	1	hk	270	kk	0
>ref-157713	ATCTCTCCCACTTTCTCTCCTCTTCTA	5	<nnxnp>	nn	222	np	27		
>ref-157713	ATCTCTCCCACTTTCTCTCCTCTTCTA	21	<hkxhk>	hh	0	hk	263	kk	4
>ref-157713	ATCTCTCCCACTTTCTCTCCTCTTCTA	22	<hkxhk>	hh	0	hk	263	kk	4
>ref-157713	ATCTCTCCCACTTTCTCTCCTCTTCTA	27	<hkxhk>	hh	29	hk	219	kk	0
>ref-157737	AATGTTGTCACTCCGACTCCCTTCTCC	4	<lmxll>	ll	153	lm	110		
>ref-157740	ACACCTACTACTACTACTCCAACTAGC	9	<nnxnp>	nn	254	np	0		
>ref-157742	CCCTTCTTAACATCCCCTCCTCCCCTC	5	<lmxll>	ll	84	lm	158		
>ref-157749	AGGGGAGGGACGCTGTCTCCTTCTCTC	22	<nnxnp>	nn	106	np	147		
>ref-157759	CTACCTCTCACACCCCCTCCCACCCCC	8	<lmxll>	ll	142	lm	120		
>ref-157759	CTACCTCTCACACCCCCTCCCACCCCC	16	<nnxnp>	nn	7	np	252		
>ref-157760	CCCGCTTCTACTCCCCCTCCTCCATCC	3	<lmxll>	ll	15	lm	249		
>ref-157760	CCCGCTTCTACTCCCCCTCCTCCATCC	4	<hkxhk>	hh	4	hk	269	kk	0
>ref-157760	CCCGCTTCTACTCCCCCTCCTCCATCC	13	<hkxhk>	hh	4	hk	269	kk	0
>ref-157760	CCCGCTTCTACTCCCCCTCCTCCATCC	21	<lmxll>	ll	15	lm	247		
>ref-157774	CCATCTCCTACTCCGCCTCCAGCTGCG	1	<nnxnp>	nn	143	np	122		
>ref-157800	CTTGCTCTAACTCTTCCTCCTCGTTCT	1	<nnxnp>	nn	127	np	135		
>ref-157812	CTGTAAAACACTTTTCCTCCCTGTGAA	3	<lmxll>	ll	126	lm	133		
>ref-157812	CTGTAAAACACTTTTCCTCCCTGTGAA	4	<lmxll>	ll	154	lm	111		
>ref-157812	CTGTAAAACACTTTTCCTCCCTGTGAA	5	<hkxhk>	hh	0	hk	269	kk	0
>ref-157843	TATCTATTTACTGTATCTCCCTTATCC	8	<lmxll>	ll	105	lm	117		
>ref-157847	TCTCCCTTCACCCATTCTCCTCTCCCT	22	<lmxll>	ll	7	lm	255		

>ref-157863	CTGGCCTACACCTGGCCTCCCTGCATC	3	<nnxnp>	nn	115	np	156		
>ref-157884	CAATGAAACACATTCACTCCATACGCA	25	<nnxnp>	nn	129	np	110		
>ref-157903	CATCCACCCACCCACTCTCCTTCTCCT	12	<hkxhk>	hh	1	hk	268	kk	0
>ref-157903	CATCCACCCACCCACTCTCCTTCTCCT	27	<hkxhk>	hh	0	hk	269	kk	1
>ref-157905	CCCTTCGTTACTCCTTCTCCTACCTTT	7	<hkxhk>	hh	0	hk	205	kk	43
>ref-157920	AAAAGACAGACACGCGCTCCTCCTTTA	16	<lmxll>	ll	105	lm	138		
>ref-157928	TCTCTCCTCACGCCCCCTCCCCCCAAT	4	<nnxnp>	nn	268	np	0		
>ref-157928	TCTCTCCTCACGCCCCCTCCCCCCAAT	6	<nnxnp>	nn	117	np	131		
>ref-157934	TTCTGCTAAACCTACCCTCCTCCATTC	15	<nnxnp>	nn	121	np	138		
>ref-157962	GTCCTTAATACTTTATCTCCTTTTGTT	6	<lmxll>	ll	140	lm	101		
>ref-157971	ACTGCCTCTACGTAGCCTCCCTTTCTT	24	<lmxll>	ll	132	lm	137		
>ref-157972	CCCCCCGTCACTCTATCTCCTAATCCC	1	<lmxll>	ll	145	lm	105		
>ref-157981	AGAATGTAAACAAACCCTCCACGCTCT	16	<hkxhk>	hh	0	hk	273	kk	0
>ref-157990	CAAGCGAGCACCCAGCCTCCAACAAAA	27	<nnxnp>	nn	134	np	130		
>ref-157998	TGTGTCCTCACTTGTCCTCCCTTTCCG	16	<nnxnp>	nn	116	np	152		
>ref-158001	TACCAGCACACCATTTCTCCCCACTCT	13	<lmxll>	ll	133	lm	135		
>ref-158001	TACCAGCACACCATTTCTCCCCACTCT	22	<nnxnp>	nn	141	np	129		
>ref-158008	CTCCCTCATACAGCATCTCCCTCTCTC	3	<lmxll>	ll	128	lm	141		
>ref-158045	TACGCATACACTTCACCTCCTTTTATT	5	<lmxll>	ll	249	lm	0		
>ref-158045	TACGCATACACTTCACCTCCTTTTATT	24	<nnxnp>	nn	249	np	0		
>ref-158045	TACGCATACACTTCACCTCCTTTTATT	25	<nnxnp>	nn	248	np	0		
>ref-158077	AGAGAGTAGACCACATCTCCTTTCGTT	2	<lmxll>	ll	120	lm	147		
>ref-158079	ACATAACACACACTTGCTCCCCCCGCC	12	<hkxhk>	hh	2	hk	267	kk	0
>ref-158082	AGCACTGCGACAAATACTCCGGCCATG	21	<lmxll>	ll	128	lm	132		
>ref-158104	TGTCTCTATACTTCCTCTCCCTCCTTT	5	<hkxhk>	hh	55	hk	132	kk	55
>ref-158162	CTAAGTGTCACAGCACCTCCATGTTTG	13	<nnxnp>	nn	133	np	127		
>ref-158163	GAACAGTCGACTAGCTCTCCCAACTCT	4	<hkxhk>	hh	64	hk	121	kk	39
>ref-158180	TTTATCCCTACTTCTGCTCCTCCCACT	3	<hkxhk>	hh	0	hk	273	kk	0
>ref-158180	TTTATCCCTACTTCTGCTCCTCCCACT	4	<nnxnp>	nn	14	np	255		
>ref-158206	CTTATAACAACGTCCCCTCCTTTGCTT	13	<nnxnp>	nn	105	np	122		
>ref-158212	GGAGACACTACATTAGCTCCAATTAGC	1	<hkxhk>	hh	0	hk	242	kk	18
>ref-158212	GGAGACACTACATTAGCTCCAATTAGC	9	<lmxll>	ll	107	lm	153		
>ref-158217	CCTTACCATACAGGGGCTCCACAGGTG	8	<nnxnp>	nn	120	np	126		
>ref-158230	AAGTCAATAACTAGGTCTCCTCCTCCT	26	<hkxhk>	hh	118	hk	123	kk	0
>ref-158230	AAGTCAATAACTAGGTCTCCTCCTCCT	27	<hkxhk>	hh	0	hk	123	kk	116
>ref-158241	TCTAGCTCTACTCCATCTCCTACACAG	12	<lmxll>	ll	138	lm	119		
>ref-158269	AACAATGACACAGTTTCTCCCAGATAA	21	<nnxnp>	nn	132	np	129		
>ref-158280	CACGTCTCCACTCCCTCTCCCTCATCC	21	<nnxnp>	nn	140	np	128		

>ref-158305	CCTCACTTCACATTTTCTCCTCTCTCA	13	<nnxnp>	nn	119	np	130		
>ref-158309	TCTCTCCCAACCCTCCCTCCACCCCCC	2	<nnxnp>	nn	231	np	0		
>ref-158330	CACTCCCCCACCCCCTCTCCAACCCCT	4	<lmxll>	ll	159	lm	76		
>ref-158330	CACTCCCCCACCCCCTCTCCAACCCCT	6	<nnxnp>	nn	269	np	0		
>ref-158346	CAGAGCGTTACAGTGACTCCCAACTAC	3	<hkxhk>	hh	3	hk	216	kk	42
>ref-158346	CAGAGCGTTACAGTGACTCCCAACTAC	16	<hkxhk>	hh	3	hk	215	kk	42
>ref-158349	CCGCCGTAAACAGGGGCTCCTTCGCCT	3	<lmxll>	ll	26	lm	241		
>ref-158349	CCGCCGTAAACAGGGGCTCCTTCGCCT	26	<hkxhk>	hh	159	hk	100	kk	0
>ref-158349	CCGCCGTAAACAGGGGCTCCTTCGCCT	27	<nnxnp>	nn	153	np	104		
>ref-158354	GATGCAACGACCATCTCTCCTGGTCTG	7	<nnxnp>	nn	125	np	140		
>ref-158401	AGACTTAAAACCACATCTCCATCGTAT	25	<hkxhk>	hh	56	hk	140	kk	67
>ref-158407	CCAAGGCCCACATCAACTCCTCGAGAA	2	<lmxll>	ll	145	lm	119		
>ref-158414	ATGGACATTACTTCCTCTCCAAAGCCT	21	<hkxhk>	hh	63	hk	131	kk	52
>ref-158450	CTCACTGCCACTTACACTCCCTTTATC	7	<lmxll>	ll	135	lm	95		
>ref-158452	CTTCAATTCACATTCCCTCCGAACACT	12	<nnxnp>	nn	122	np	134		
>ref-158480	CTCCGAAACACCGAAGCTCCAAAACTC	13	<lmxll>	ll	126	lm	93		
>ref-158489	ATACTACTGACACAGCCTCCATGCCTA	23	<nnxnp>	nn	106	np	114		
>ref-158551	AAGACTACCACTGCAACTCCTGTTGCT	1	<nnxnp>	nn	112	np	140		
>ref-158555	TCGTGGGCCACATCATCTCCTCCTCGA	1	<nnxnp>	nn	264	np	0		
>ref-158555	TCGTGGGCCACATCATCTCCTCCTCGA	6	<nnxnp>	nn	265	np	0		
>ref-158589	AGTGCCTTCACCGGTTCTCCATCAAGT	4	<hkxhk>	hh	0	hk	178	kk	68
>ref-158589	AGTGCCTTCACCGGTTCTCCATCAAGT	21	<hkxhk>	hh	0	hk	250	kk	0
>ref-158592	ACATACTGCACATTCCCTCCTCGTATT	9	<nnxnp>	nn	132	np	123		
>ref-158594	TATTGTCCAACCGCTGCTCCGTCACTC	2	<lmxll>	ll	65	lm	203		
>ref-158594	TATTGTCCAACCGCTGCTCCGTCACTC	12	<lmxll>	ll	64	lm	204		
>ref-158594	TATTGTCCAACCGCTGCTCCGTCACTC	16	<lmxll>	ll	136	lm	130		
>ref-158601	ATCATCCCAACCCCCCCTCCGCAGCTC	1	<nnxnp>	nn	119	np	135		
>ref-158602	GTCACCTATACTTTAACTCCGAATCTG	26	<nnxnp>	nn	250	np	0		
>ref-158613	GTTCCTCTTACATTTTCTCCTTGCCTT	3	<nnxnp>	nn	116	np	153		
>ref-158628	CACATTCCCACATTTGCTCCCATTCCC	9	<hkxhk>	hh	59	hk	205	kk	1
>ref-158628	CACATTCCCACATTTGCTCCCATTCCC	12	<hkxhk>	hh	59	hk	205	kk	1
>ref-158629	CACACACACACGTTGCCTCCTTTTCTT	1	<lmxll>	ll	145	lm	120		
>ref-158629	CACACACACACGTTGCCTCCTTTTCTT	14	<nnxnp>	nn	266	np	0		
>ref-158629	CACACACACACGTTGCCTCCTTTTCTT	15	<nnxnp>	nn	266	np	0		
>ref-158635	TAAGTCCATACACAGTCTCCACTAGTA	13	<lmxll>	ll	122	lm	104		
>ref-158640	CCCTTTCCTACCTCCCCTCCTTACCCA	6	<hkxhk>	hh	9	hk	236	kk	6
>ref-158640	CCCTTTCCTACCTCCCCTCCTTACCCA	27	<hkxhk>	hh	6	hk	236	kk	9
>ref-158642	CCACCTCGCACACCCCCTCCCCTACCT	1	<nnxnp>	nn	135	np	107		

>ref-158642	CCACCTCGCACACCCCCTCCCCTACCT	8	<hkxhk>	hh	29	hk	195	kk	4
>ref-158651	CCTTCCCGCACCTTCTCTCCTTGTCTC	23	<lmxll>	ll	264	lm	0		
>ref-158651	CCTTCCCGCACCTTCTCTCCTTGTCTC	26	<lmxll>	ll	265	lm	0		
>ref-158661	TGTATGGCTACACCCCCTCCCCCACAC	13	<nnxnp>	nn	260	np	0		
>ref-158683	TTTGTTTCCACATTCTCTCCCCTCCCC	8	<hkxhk>	hh	67	hk	137	kk	58
>ref-158685	TCAGGTTATACAAATCCTCCTACTGTC	7	<lmxll>	ll	137	lm	128		
>ref-158688	AGGAGTGTGACAACTTCTCCTGCTCTT	15	<lmxll>	ll	128	lm	142		
>ref-158700	CTCCCCATAACACCACCTCCCCATAAC	14	<hkxhk>	hh	158	hk	107	kk	0
>ref-158710	TGCCCTTGCACTGTGCCTCCTCTCCCA	4	<lmxll>	ll	153	lm	114		
>ref-158772	CCCTTTCACACGCTCCCTCCGCCCCCT	5	<lmxll>	ll	143	lm	123		
>ref-158774	ATTGAACACACCGATGCTCCATATCGG	27	<lmxll>	ll	74	lm	183		
>ref-158776	CTCACATTCACCCTCGCTCCCCCTTCC	1	<nnxnp>	nn	255	np	0		
>ref-158776	CTCACATTCACCCTCGCTCCCCCTTCC	4	<nnxnp>	nn	253	np	0		
>ref-158788	CTACGTCATACCCTATCTCCGACGTCA	1	<lmxll>	ll	141	lm	120		
>ref-158788	CTACGTCATACCCTATCTCCGACGTCA	2	<lmxll>	ll	141	lm	120		
>ref-158808	TGTTCCCTAACCCCTTCTCCTTTCTTC	6	<lmxll>	ll	258	lm	0		
>ref-158821	TCTACCCCTACGTCGCCTCCTTTCGTG	24	<lmxll>	ll	141	lm	119		
>ref-158840	TTCCTTTTTACTCCCTCTCCCTTCACC	6	<lmxll>	ll	109	lm	135		
>ref-158848	TACACCCACACACCCACTCCCCTGCCC	1	<hkxhk>	hh	0	hk	269	kk	0
>ref-158848	TACACCCACACACCCACTCCCCTGCCC	12	<hkxhk>	hh	0	hk	269	kk	0
>ref-158869	TTCACTTAAACCCTTTCTCCTCCTCCT	27	<nnxnp>	nn	122	np	139		
>ref-158872	GGGGTCAATACTCGACCTCCTTCGTCT	1	<lmxll>	ll	133	lm	129		
>ref-158880	ACCTTGTTTACCTTATCTCCCTCCCCT	22	<lmxll>	ll	151	lm	108		
>ref-158881	CCCTTCCACACTCACTCTCCTAACACT	6	<lmxll>	ll	144	lm	89		
>ref-158881	CCCTTCCACACTCACTCTCCTAACACT	12	<nnxnp>	nn	253	np	0		
>ref-158881	CCCTTCCACACTCACTCTCCTAACACT	24	<lmxll>	ll	151	lm	84		
>ref-158899	GCCCACTCTACTCTACCTCCGCCCACT	16	<hkxhk>	hh	1	hk	267	kk	0
>ref-158899	GCCCACTCTACTCTACCTCCGCCCACT	22	<hkxhk>	hh	11	hk	255	kk	1
>ref-158899	GCCCACTCTACTCTACCTCCGCCCACT	27	<hkxhk>	hh	0	hk	267	kk	1
>ref-158914	TTGATGAACACCCCGCCTCCTTTTCCT	2	<nnxnp>	nn	142	np	122		
>ref-158945	CTGCCTCGTACTTCGCCTCCATCGCCC	8	<nnxnp>	nn	149	np	119		
>ref-158947	CCATTATCCACACGTCCTCCTCTTCCC	3	<lmxll>	ll	138	lm	110		
>ref-158947	CCATTATCCACACGTCCTCCTCTTCCC	27	<lmxll>	ll	134	lm	109		
>ref-158962	CTGGACATCACAGAATCTCCATGACTA	22	<nnxnp>	nn	140	np	123		
>ref-158977	TCTTCTACCACGAGTCCTCCTTCGTCA	6	<lmxll>	ll	114	lm	143		
>ref-158985	TTGGCAGTCACGAATGCTCCTCGCTCT	3	<nnxnp>	nn	146	np	125		
>ref-158986	ACACACCGCACCCTCCCTCCTCCACAA	27	<nnxnp>	nn	110	np	157		
>ref-159000	TTTGCCAGAACCCTTTCTCCTCCTTCT	14	<hkxhk>	hh	61	hk	133	kk	53

>ref-159000	TTTGCCAGAACCCTTTCTCCTCCTTCT	15	<hkxhk>	hh	61	hk	133	kk	53
>ref-159001	CATGCACGTACATATACTCCACACGCA	4	<nnxnp>	nn	135	np	133		
>ref-159012	TCATCCTCCACTCTTACTCCCACGCTA	14	<nnxnp>	nn	127	np	128		
>ref-159028	CTCCTCGTCACCGTCTCTCCATCTCTC	7	<hkxhk>	hh	44	hk	149	kk	59
>ref-159061	CAATAGATAACCTCAACTCCCTCTTCT	12	<lmxll>	ll	117	lm	124		
>ref-159069	ACACATATGACTCCCTCTCCCTTGCCA	25	<lmxll>	ll	134	lm	138		
>ref-159085	TCTCCTCCTACCTGCCCTCCTCTACCT	13	<nnxnp>	nn	122	np	134		
>ref-159085	TCTCCTCCTACCTGCCCTCCTCTACCT	24	<nnxnp>	nn	117	np	135		
>ref-159090	CGAGGTTTTACACACACTCCGTCAGTA	21	<nnxnp>	nn	111	np	116		
>ref-159090	CGAGGTTTTACACACACTCCGTCAGTA	22	<nnxnp>	nn	113	np	115		
>ref-159102	GCACCATCTACACGGGCTCCTACCCAG	5	<lmxll>	ll	77	lm	175		
>ref-159102	GCACCATCTACACGGGCTCCTACCCAG	16	<hkxhk>	hh	0	hk	234	kk	23
>ref-159105	CTCCCTCTAACCTTCTCTCCTACTCTC	7	<nnxnp>	nn	143	np	119		
>ref-159105	CTCCCTCTAACCTTCTCTCCTACTCTC	9	<lmxll>	ll	233	lm	22		
>ref-159105	CTCCCTCTAACCTTCTCTCCTACTCTC	22	<lmxll>	ll	221	lm	23		
>ref-159105	CTCCCTCTAACCTTCTCTCCTACTCTC	26	<lmxll>	ll	270	lm	0		
>ref-159119	CTTTTCTCAACACTCGCTCCTCCTCTC	9	<hkxhk>	hh	47	hk	131	kk	50
>ref-159191	CGGGCGTGTACTCTTCCTCCTCTCCCT	26	<nnxnp>	nn	119	np	101		
>ref-159193	ACCCTCCTTACTCTCCCTCCTTATCCT	21	<lmxll>	ll	99	lm	151		
>ref-159193	ACCCTCCTTACTCTCCCTCCTTATCCT	25	<lmxll>	ll	151	lm	116		
>ref-159219	TACTGTCTGACTTTATCTCCCTCATCT	12	<nnxnp>	nn	103	np	142		
>ref-159224	GTTACAAACACCGCCACTCCTGCATGC	25	<nnxnp>	nn	97	np	161		
>ref-159244	TTCCCTTCTACTCACCCTCCCTCTTCT	8	<lmxll>	ll	37	lm	218		
>ref-159244	TTCCCTTCTACTCACCCTCCCTCTTCT	26	<lmxll>	ll	37	lm	216		
>ref-159275	TGGTCGGGAACGTCTTCTCCACACTCG	6	<lmxll>	ll	0	lm	273		
>ref-159275	TGGTCGGGAACGTCTTCTCCACACTCG	9	<lmxll>	ll	0	lm	273		
>ref-159285	AGAGAAATGACAACCTCTCCATGACAG	25	<nnxnp>	nn	139	np	121		
>ref-159297	TCTTCCTACACCCTTTCTCCCTCTTCC	7	<hkxhk>	hh	0	hk	149	kk	96
>ref-159297	TCTTCCTACACCCTTTCTCCCTCTTCC	12	<hkxhk>	hh	93	hk	148	kk	0
>ref-159321	CAATCACAAACGTGGTCTCCCCAAGGT	21	<lmxll>	ll	116	lm	134		
>ref-159325	GCGGTCACTACTCTCTCTCCCTTTTTC	2	<nnxnp>	nn	244	np	1		
>ref-159343	CTCAGGGCAACCGACCCTCCCAGCCTG	21	<lmxll>	ll	123	lm	138		
>ref-159352	AGTCTCTCCACTTTCCCTCCATCTCTT	25	<hkxhk>	hh	63	hk	131	kk	42
>ref-159379	CCTCATACTACCCCTTCTCCTTCCCCT	1	<nnxnp>	nn	269	np	0		
>ref-159379	CCTCATACTACCCCTTCTCCTTCCCCT	7	<lmxll>	ll	151	lm	102		
>ref-159379	CCTCATACTACCCCTTCTCCTTCCCCT	24	<lmxll>	ll	151	lm	104		
>ref-159389	GTGGGGATAACGCTGCCTCCCTTATAA	25	<lmxll>	ll	152	lm	106		
>ref-159392	GACTAACCCACCTACCCTCCTTTCACC	8	<nnxnp>	nn	33	np	210		

>ref-159392	GACTAACCCACCTACCCTCCTTTCACC	21	<hkxhk>	hh	2	hk	261	kk	0
>ref-159419	TGTCATTCAACTCCACCTCCTTTTCAC	8	<nnxnp>	nn	116	np	129		
>ref-159419	TGTCATTCAACTCCACCTCCTTTTCAC	15	<nnxnp>	nn	116	np	129		
>ref-159421	TGCTCTACCACCACGCCTCCCACCACC	5	<lmxll>	ll	148	lm	121		
>ref-159426	CAAACGAGAACATACCCTCCCACACAC	21	<lmxll>	ll	131	lm	132		
>ref-159429	TCTTGTAAAACTTCTCCTCCTCCTCCT	3	<hkxhk>	hh	0	hk	271	kk	1
>ref-159429	TCTTGTAAAACTTCTCCTCCTCCTCCT	8	<hkxhk>	hh	1	hk	270	kk	0
>ref-159429	TCTTGTAAAACTTCTCCTCCTCCTCCT	9	<hkxhk>	hh	0	hk	271	kk	0
>ref-159437	ACCCTCCCCACTTTCCCTCCATATCCC	3	<lmxll>	ll	261	lm	0		
>ref-159471	CGGAGCCTCACAGCTTCTCCAGGCTCT	6	<nnxnp>	nn	130	np	135		
>ref-159481	CACGCTTCAACCCCCCCTCCCCTCGCT	27	<lmxll>	ll	120	lm	131		
>ref-159489	TATCCACGTACTGAGTCTCCAGGCCCG	12	<lmxll>	ll	130	lm	136		
>ref-159493	TTTTCACTTACTTCCTCTCCTTCATTA	27	<nnxnp>	nn	136	np	104		
>ref-159525	GTTAGAGACACCTCCCCTCCCTCCCCT	25	<nnxnp>	nn	142	np	100		
>ref-159525	GTTAGAGACACCTCCCCTCCCTCCCCT	27	<nnxnp>	nn	143	np	97		
>ref-159568	AGATTCAACACTTTATCTCCAGGCGCT	27	<nnxnp>	nn	150	np	107		
>ref-159596	CGCCACGGCACGTCACCTCCGGGGCAC	7	<lmxll>	ll	132	lm	138		
>ref-159605	GCCATGTTCACGAAGGCTCCATCTCAG	9	<nnxnp>	nn	137	np	130		
>ref-159611	CGCTGAGAAACGTGACCTCCTTCCTTT	21	<lmxll>	ll	136	lm	131		
>ref-159620	TAAGACCTCACTGCAGCTCCCCTTTAC	8	<nnxnp>	nn	258	np	0		
>ref-159632	ATTGACTTCACGACTCCTCCTCACCTG	23	<nnxnp>	nn	120	np	136		
>ref-159636	CAACCTGCAACACCTCCTCCAGTGGAT	15	<nnxnp>	nn	31	np	202		
>ref-159637	ATTTTTATTACTCCACCTCCTCTTCTT	1	<hkxhk>	hh	0	hk	260	kk	3
>ref-159637	ATTTTTATTACTCCACCTCCTCTTCTT	5	<hkxhk>	hh	3	hk	260	kk	0
>ref-159643	TCCATTCTTACTTCCTCTCCCTTGCTC	8	<nnxnp>	nn	101	np	159		
>ref-159643	TCCATTCTTACTTCCTCTCCCTTGCTC	12	<nnxnp>	nn	99	np	159		
>ref-159680	TTTCTCCTTACTCCCTCTCCCCCAATA	25	<lmxll>	ll	105	lm	125		
>ref-159696	ATCAAGCCTACCATCCCTCCAACGTCG	25	<nnxnp>	nn	133	np	107		
>ref-159741	CTATGCTCCACTCCCACTCCCTCCCTC	24	<hkxhk>	hh	44	hk	210	kk	0
>ref-159741	CTATGCTCCACTCCCACTCCCTCCCTC	26	<hkxhk>	hh	0	hk	206	kk	44
>ref-159746	TCAGTCAGGACCAACCCTCCCGATCTC	13	<lmxll>	ll	132	lm	129		
>ref-159761	TCCTCCCGAACCTCTCCTCCAATTATC	7	<hkxhk>	hh	59	hk	146	kk	58
>ref-159764	ATTCATTCCACATAATCTCCACACCAC	8	<hkxhk>	hh	66	hk	126	kk	55
>ref-159771	GACAAACGCACAATGACTCCAGGCATA	8	<lmxll>	ll	125	lm	121		
>ref-159773	CCATGCCACACTGGCCCTCCGGTCTCA	5	<lmxll>	ll	122	lm	145		
>ref-159773	CCATGCCACACTGGCCCTCCGGTCTCA	16	<hkxhk>	hh	2	hk	271	kk	0
>ref-159783	GTTCCCTCCACCTTCCCTCCACCTCCG	21	<nnxnp>	nn	91	np	151		
>ref-159783	GTTCCCTCCACCTTCCCTCCACCTCCG	23	<lmxll>	ll	244	lm	10		

>ref-159783	GTTCCCTCCACCTTCCCTCCACCTCCG	24	<lmxll>	ll	242	lm	14		
>ref-159783	GTTCCCTCCACCTTCCCTCCACCTCCG	25	<lmxll>	ll	254	lm	4		
>ref-159799	TCAAAAGGTACATTACCTCCTGCACCC	23	<hkxhk>	hh	51	hk	134	kk	63
>ref-159814	ATTACCCTTACCCCCCCTCCCCTTCCC	7	<lmxll>	ll	133	lm	102		
>ref-159829	TACCTCCCCACCTACCCTCCACCCTCC	3	<nnxnp>	nn	262	np	0		
>ref-159839	CCTTCTTCTACCTTTGCTCCTTATCTC	4	<hkxhk>	hh	1	hk	270	kk	1
>ref-159839	CCTTCTTCTACCTTTGCTCCTTATCTC	16	<hkxhk>	hh	1	hk	270	kk	1
>ref-159839	CCTTCTTCTACCTTTGCTCCTTATCTC	21	<nnxnp>	nn	147	np	117		
>ref-159847	TCCTCCTCAACACTCTCTCCCGTCTCC	22	<lmxll>	ll	157	lm	100		
>ref-159847	TCCTCCTCAACACTCTCTCCCGTCTCC	24	<lmxll>	ll	158	lm	99		
>ref-159856	ACCAGTATCACCACCTCTCCTCCTAAT	26	<nnxnp>	nn	219	np	2		
>ref-159865	CACACACACACGACTGCTCCGATGGTC	24	<lmxll>	ll	0	lm	273		
>ref-159916	CTCACGCCCACTGTCTCTCCCTCATTC	9	<lmxll>	ll	139	lm	127		
>ref-159930	TGCTACTAAACTGCTCCTCCTGCTACC	1	<lmxll>	ll	132	lm	137		
>ref-159943	GTACTCCCGACGCCCACTCCACCATTG	1	<nnxnp>	nn	141	np	126		
>ref-159953	ATCCATTCCACACCCTCTCCACCTATC	24	<hkxhk>	hh	0	hk	272	kk	1
>ref-159953	ATCCATTCCACACCCTCTCCACCTATC	25	<hkxhk>	hh	0	hk	273	kk	0
>ref-159959	CTTTTCCTCACTTATTCTCCTTCCTTT	8	<lmxll>	ll	124	lm	132		
>ref-159959	CTTTTCCTCACTTATTCTCCTTCCTTT	14	<lmxll>	ll	125	lm	132		
>ref-160000	TAGCCTTCTACACTCTCTCCCTCTCTT	8	<hkxhk>	hh	1	hk	272	kk	0
>ref-160000	TAGCCTTCTACACTCTCTCCCTCTCTT	14	<hkxhk>	hh	0	hk	272	kk	1
>ref-160013	CCTGCCATCACCCTTTCTCCTTCCTTT	1	<nnxnp>	nn	105	np	155		
>ref-160013	CCTGCCATCACCCTTTCTCCTTCCTTT	6	<nnxnp>	nn	106	np	155		
>ref-160023	ATCTTTTCCACCCCTTCTCCCTCTCTT	1	<hkxhk>	hh	7	hk	254	kk	1
>ref-160023	ATCTTTTCCACCCCTTCTCCCTCTCTT	24	<hkxhk>	hh	1	hk	254	kk	7
>ref-160028	TAACTCATAACAACAACTCCTTGATAA	26	<lmxll>	ll	134	lm	98		
>ref-160030	GATGCACGTACATTCCCTCCGAGTCAG	14	<lmxll>	ll	143	lm	127		
>ref-160062	TCCCTTCTTACTTATTCTCCTGCAATA	12	<lmxll>	ll	119	lm	134		
>ref-160088	CCCTTTCTTACCTTTTCTCCATTCCAT	2	<nnxnp>	nn	135	np	105		
>ref-160088	CCCTTTCTTACCTTTTCTCCATTCCAT	26	<nnxnp>	nn	133	np	106		
>ref-160101	CTGACTCTGACTTCTACTCCTACTGCT	25	<hkxhk>	hh	0	hk	266	kk	2
>ref-160101	CTGACTCTGACTTCTACTCCTACTGCT	26	<hkxhk>	hh	0	hk	266	kk	2
>ref-160154	TCTCCTTCCACCCTGGCTCCAGCCCTT	25	<nnxnp>	nn	141	np	130		
>ref-160157	CTCTTTCACACCCTGCCTCCTCGGGTT	13	<hkxhk>	hh	45	hk	157	kk	55
>ref-160160	TTTTTCTTCACTCTGTCTCCGTCTGTC	9	<lmxll>	ll	126	lm	138		
>ref-160194	TTTACGGTAACAAATGCTCCAAATCCT	14	<lmxll>	ll	1	lm	272		
>ref-160228	TACCTAATTACCTCATCTCCCCTCCCT	9	<nnxnp>	nn	123	np	134		
>ref-160232	ATGATAAGTACTTTGGCTCCATCCCTT	9	<hkxhk>	hh	52	hk	127	kk	42

>ref-160234	CAAGAAATTACTCTCTCTCCCTCTCTC	5	<lmxll>	ll	142	lm	120		
>ref-160234	CAAGAAATTACTCTCTCTCCCTCTCTC	24	<lmxll>	ll	153	lm	88		
>ref-160234	CAAGAAATTACTCTCTCTCCCTCTCTC	26	<lmxll>	ll	157	lm	85		
>ref-160243	CAACCAAGTACCTAATCTCCTCTCTCT	3	<lmxll>	ll	129	lm	137		
>ref-160253	ATCACTTTTACTCAAGCTCCTCAGTAA	12	<lmxll>	ll	122	lm	119		
>ref-160265	TCCCATTGTACCCCCCCTCCTCCCCCT	4	<hkxhk>	hh	0	hk	244	kk	7
>ref-160265	TCCCATTGTACCCCCCCTCCTCCCCCT	6	<hkxhk>	hh	0	hk	244	kk	7
>ref-160296	CTTGTATGTACTTGCTCTCCTCTTCTG	5	<hkxhk>	hh	55	hk	157	kk	40
>ref-160308	TATTATCTAACTGTTTCTCCTTTATCC	2	<lmxll>	ll	112	lm	112		
>ref-160323	CTCTCACCTACTTCCCCTCCCTCCTCT	1	<nnxnp>	nn	258	np	1		
>ref-160328	GTGTCTTCCACCTTCCCTCCCTCCCAC	26	<nnxnp>	nn	237	np	6		
>ref-160335	ATAATGTTGACTCTATCTCCTCCTCTT	13	<hkxhk>	hh	65	hk	136	kk	39
>ref-160352	CTTGCCTCTACATCTTCTCCAGGTTAA	7	<nnxnp>	nn	129	np	130		
>ref-160363	TTGTTCATCACGAATCCTCCTCAACAA	4	<lmxll>	ll	121	lm	105		
>ref-160363	TTGTTCATCACGAATCCTCCTCAACAA	23	<nnxnp>	nn	108	np	114		
>ref-160366	ACTAAACGCACACATTCTCCACCTCTA	4	<nnxnp>	nn	133	np	131		
>ref-160368	AGTTTTTTTACTTTGTCTCCCTCGTGG	5	<nnxnp>	nn	112	np	128		
>ref-160374	CTTCATCTTACCTTCCCTCCCTTCCCT	6	<lmxll>	ll	132	lm	121		
>ref-160374	CTTCATCTTACCTTCCCTCCCTTCCCT	8	<hkxhk>	hh	0	hk	272	kk	1
>ref-160374	CTTCATCTTACCTTCCCTCCCTTCCCT	14	<hkxhk>	hh	0	hk	237	kk	30
>ref-160399	TTCCCCTCCACTTCCCCTCCTCCCCAC	4	<lmxll>	ll	246	lm	0		
>ref-160399	TTCCCCTCCACTTCCCCTCCTCCCCAC	6	<nnxnp>	nn	245	np	0		
>ref-160399	TTCCCCTCCACTTCCCCTCCTCCCCAC	8	<nnxnp>	nn	70	np	156		
>ref-160399	TTCCCCTCCACTTCCCCTCCTCCCCAC	24	<nnxnp>	nn	90	np	137		
>ref-160401	CTCACTCCCACCCTCTCTCCTTTTCCC	21	<hkxhk>	hh	0	hk	273	kk	0
>ref-160401	CTCACTCCCACCCTCTCTCCTTTTCCC	23	<hkxhk>	hh	0	hk	273	kk	0
>ref-160402	TTATGCTAGACACCTCCTCCATTCAGC	9	<nnxnp>	nn	269	np	0		
>ref-160405	ACGAAAGGGACAGTTTCTCCTGACATG	9	<nnxnp>	nn	104	np	137		
>ref-160412	GGCAAACCCACGAGCTCTCCCTCACAC	26	<nnxnp>	nn	136	np	135		
>ref-160415	TATTTCATCACTTCCTCTCCTATTCTC	25	<hkxhk>	hh	0	hk	273	kk	0
>ref-160415	TATTTCATCACTTCCTCTCCTATTCTC	27	<hkxhk>	hh	231	hk	35	kk	0
>ref-160419	ATCTCGCCCACTCAAACTCCGTCCGTC	15	<hkxhk>	hh	53	hk	144	kk	70
>ref-160444	CAACTCCACACCTCATCTCCTCTTCCC	21	<lmxll>	ll	112	lm	133		
>ref-160454	CGACCTTGGACACCCACTCCTGTTTAC	26	<nnxnp>	nn	131	np	102		
>ref-160474	ACCCCTATCACCTCCTCTCCTTCCTTA	2	<lmxll>	ll	137	lm	128		
>ref-160492	GTGCCTCTCACCTCCTCTCCCCCGTAT	1	<lmxll>	ll	118	lm	151		
>ref-160496	ATTTTGTTTACTACTCCTCCTTCTTCT	25	<hkxhk>	hh	50	hk	135	kk	54
>ref-160503	AAGGCTCTCACTCGAACTCCTACAGTT	1	<lmxll>	ll	133	lm	136		

>ref-160503	AAGGCTCTCACTCGAACTCCTACAGTT	23	<lmxll>	ll	133	lm	136		
>ref-160511	TTAACAATAACGCCTTCTCCCAGGAAT	23	<lmxll>	ll	96	lm	147		
>ref-160520	CAATCCATAACTTTCTCTCCCTCTTAT	7	<hkxhk>	hh	0	hk	272	kk	0
>ref-160520	CAATCCATAACTTTCTCTCCCTCTTAT	8	<hkxhk>	hh	0	hk	272	kk	0
>ref-160520	CAATCCATAACTTTCTCTCCCTCTTAT	9	<nnxnp>	nn	7	np	249		
>ref-160523	CCGCATCCCACCTCTCCTCCCTCCTAC	25	<nnxnp>	nn	134	np	121		
>ref-160523	CCGCATCCCACCTCTCCTCCCTCCTAC	26	<nnxnp>	nn	133	np	122		
>ref-160540	TCCCACACCACTCTTTCTCCTCTCTCC	25	<hkxhk>	hh	75	hk	195	kk	0
>ref-160565	TACCTCTCTACCTCCCCTCCTTTCCCC	23	<hkxhk>	hh	39	hk	147	kk	66
>ref-160584	CTCTTTAACACACCTACTCCTCCCCTA	24	<nnxnp>	nn	135	np	128		
>ref-160599	ATCTGTCTCACTGGCTCTCCCCCATCT	23	<nnxnp>	nn	121	np	134		
>ref-160614	TCTCATTCTACCCCTTCTCCTCCTCTA	12	<hkxhk>	hh	9	hk	263	kk	0
>ref-160614	TCTCATTCTACCCCTTCTCCTCCTCTA	15	<hkxhk>	hh	0	hk	263	kk	9
>ref-160625	CATTCATCTACCGAGCCTCCTCATCTT	4	<nnxnp>	nn	117	np	133		
>ref-160633	CAATTCTTCACTTATTCTCCGTCACTT	6	<nnxnp>	nn	121	np	118		
>ref-160644	CACACACAAACACTTCCTCCACTAACA	2	<hkxhk>	hh	45	hk	143	kk	68
>ref-160644	CACACACAAACACTTCCTCCACTAACA	22	<nnxnp>	nn	148	np	116		
>ref-160648	TCCCCTCTAACTTCCTCTCCGTCCCCT	9	<hkxhk>	hh	59	hk	208	kk	0
>ref-160648	TCCCCTCTAACTTCCTCTCCGTCCCCT	13	<hkxhk>	hh	0	hk	209	kk	58
>ref-160661	TGTCCCCTAACCAGCTCTCCGCGACAG	5	<nnxnp>	nn	268	np	0		
>ref-160665	ACTGGTATCACTACCTCTCCCTTCCCT	5	<hkxhk>	hh	1	hk	132	kk	132
>ref-160694	CTCCCTCCCACCTTTCCTCCATCTGTT	9	<hkxhk>	hh	2	hk	258	kk	2
>ref-160694	CTCCCTCCCACCTTTCCTCCATCTGTT	12	<hkxhk>	hh	2	hk	258	kk	2
>ref-160701	AACCTCGTTACCTTGTCTCCTGCAAGT	23	<lmxll>	ll	131	lm	130		
>ref-160707	CGCGCCTCCACACCTGCTCCAGCCGCG	1	<hkxhk>	hh	63	hk	144	kk	45
>ref-160715	TCCCTCAGGACCCTCCCTCCACCCCTC	27	<nnxnp>	nn	131	np	102		
>ref-160723	TCCAGCTGCACCCGAACTCCTCAAAGG	15	<nnxnp>	nn	122	np	131		
>ref-160786	CATTACTCCACACGACCTCCGCATCCA	1	<nnxnp>	nn	145	np	121		
>ref-160791	ATTTGTATCACCCGCCCTCCCTTACCT	7	<hkxhk>	hh	60	hk	132	kk	55
>ref-160791	ATTTGTATCACCCGCCCTCCCTTACCT	14	<lmxll>	ll	114	lm	140		
>ref-160793	CCCAAATCTACCTTCACTCCTTCCCTC	6	<lmxll>	ll	1	lm	268		
>ref-160793	CCCAAATCTACCTTCACTCCTTCCCTC	22	<lmxll>	ll	1	lm	268		
>ref-160821	GTTTCCCCAACTTTCCCTCCCTTTCTC	2	<lmxll>	ll	4	lm	265		
>ref-160821	GTTTCCCCAACTTTCCCTCCCTTTCTC	23	<lmxll>	ll	4	lm	265		
>ref-160831	CCCTACCCCACAACCTCTCCTCACCAC	1	<lmxll>	ll	0	lm	271		
>ref-160831	CCCTACCCCACAACCTCTCCTCACCAC	26	<lmxll>	ll	0	lm	271		
>ref-160873	TTATTCTCCACACCAACTCCTTATTCA	16	<nnxnp>	nn	152	np	112		
>ref-160874	TCCCCCCCTACGCTTCCTCCCCCTCTC	9	<lmxll>	ll	252	lm	0		

>ref-160883	TCCTTCCCAACATTCTCTCCTTTTCCT	7	<hkxhk>	hh	2	hk	260	kk	0
>ref-160883	TCCTTCCCAACATTCTCTCCTTTTCCT	12	<hkxhk>	hh	2	hk	260	kk	0
>ref-160884	CCTCACTCGACACCACCTCCACTCGAC	2	<hkxhk>	hh	0	hk	273	kk	0
>ref-160884	CCTCACTCGACACCACCTCCACTCGAC	3	<hkxhk>	hh	0	hk	273	kk	0
>ref-160884	CCTCACTCGACACCACCTCCACTCGAC	7	<lmxll>	ll	1	lm	272		
>ref-160903	GCGCCTGCAACGAGACCTCCACGCAAA	21	<nnxnp>	nn	72	np	195		
>ref-160911	AAGTCACAAACCACATCTCCTGAAGAT	6	<lmxll>	ll	141	lm	110		
>ref-160914	TTGTCCATCACTGCGGCTCCCTCGCAG	25	<lmxll>	ll	139	lm	130		
>ref-160915	TTCCTTTCTACCACATCTCCAATCCAC	7	<lmxll>	ll	112	lm	124		
>ref-160918	TCAACATTCACCCTTCCTCCTGCAACA	5	<lmxll>	ll	152	lm	73		
>ref-160918	TCAACATTCACCCTTCCTCCTGCAACA	9	<hkxhk>	hh	7	hk	224	kk	3
>ref-160919	AGTTTTCCCACAAGGACTCCTTCCTAA	1	<lmxll>	ll	45	lm	208		
>ref-160919	AGTTTTCCCACAAGGACTCCTTCCTAA	2	<lmxll>	ll	45	lm	208		
>ref-160932	TACTATTTCACTCGGCCTCCTTAGATT	16	<lmxll>	ll	122	lm	132		
>ref-160938	CAAAGACACACTGACACTCCCCAAGAC	7	<hkxhk>	hh	82	hk	125	kk	52
>ref-160962	CACATACAAACACCCTCTCCCACACCT	22	<nnxnp>	nn	112	np	149		
>ref-160982	ACGGGTATTACTGTTTCTCCTCAAGTA	12	<hkxhk>	hh	0	hk	271	kk	1
>ref-160982	ACGGGTATTACTGTTTCTCCTCAAGTA	21	<hkxhk>	hh	0	hk	271	kk	1
>ref-161020	TCCTCAATTACGTCCCCTCCTTCCGCC	15	<lmxll>	ll	109	lm	131		
>ref-161059	TTCCTCTCCACCCCCCCTCCGCGTGAC	6	<lmxll>	ll	108	lm	131		
>ref-161060	ATCACCCTCACCTATTCTCCTTCCAAT	9	<nnxnp>	nn	111	np	149		
>ref-161065	ATGCCCTCCACTCCCTCTCCCTCACCA	23	<nnxnp>	nn	134	np	130		
>ref-161073	ATTCTTACAACTTTCCCTCCTCTCTTC	9	<nnxnp>	nn	125	np	99		
>ref-161087	CGCCTTCTAACCCCCACTCCCTCTTTA	5	<lmxll>	ll	111	lm	114		
>ref-161088	AAGCCATTCACCCATGCTCCGTAGCCT	5	<lmxll>	ll	139	lm	129		
>ref-161097	TCAACCGGCACAGTATCTCCTATGACA	13	<lmxll>	ll	120	lm	122		
>ref-161097	TCAACCGGCACAGTATCTCCTATGACA	14	<lmxll>	ll	120	lm	122		
>ref-161106	AGCATCGTGACTGTATCTCCCTCATCA	6	<nnxnp>	nn	128	np	141		
>ref-161121	TCCACTGTCACCCATCCTCCTTCACTT	8	<hkxhk>	hh	99	hk	26	kk	99
>ref-161121	TCCACTGTCACCCATCCTCCTTCACTT	12	<nnxnp>	nn	254	np	0		
>ref-161129	TCCTTTCTGACTTCCCCTCCTCCTGCC	5	<lmxll>	ll	111	lm	121		
>ref-161139	TAGCCTTTCACCATTCCTCCTCTCTTT	4	<nnxnp>	nn	128	np	107		
>ref-161139	TAGCCTTTCACCATTCCTCCTCTCTTT	16	<nnxnp>	nn	128	np	107		
>ref-161141	TACCTGGGGACATTGCCTCCAAGCCTC	12	<hkxhk>	hh	50	hk	205	kk	0
>ref-161141	TACCTGGGGACATTGCCTCCAAGCCTC	26	<hkxhk>	hh	0	hk	205	kk	50
>ref-161167	GAAACACATACCGATGCTCCACCAACT	7	<nnxnp>	nn	148	np	119		
>ref-161168	CAGGCCGTTACAGGCACTCCAGTAAAC	21	<hkxhk>	hh	63	hk	150	kk	43
>ref-161186	TTGCCTGTCACCTTTTCTCCCCTTCCC	16	<lmxll>	ll	132	lm	125		

>ref-161188	CATTCTCTTACTGCTCCTCCCCCCTAC	27	<lmxll>	ll	121	lm	131		
>ref-161191	CACATACACACATCCCCTCCCCACGCT	3	<hkxhk>	hh	74	hk	136	kk	48
>ref-161193	TTATTCTTCACTCTCTCTCCCTTCTCT	5	<hkxhk>	hh	25	hk	183	kk	47
>ref-161193	TTATTCTTCACTCTCTCTCCCTTCTCT	16	<lmxll>	ll	135	lm	121		
>ref-161193	TTATTCTTCACTCTCTCTCCCTTCTCT	21	<lmxll>	ll	133	lm	121		
>ref-161226	TTCTCTTCCACGTTCCCTCCATTCGAT	5	<nnxnp>	nn	138	np	132		
>ref-161227	AGCTTGTTGACTTCCTCTCCTCGATTC	15	<lmxll>	ll	128	lm	143		
>ref-161239	TTCCCTCCTACTGACCCTCCTGTCCTT	21	<lmxll>	ll	0	lm	270		
>ref-161257	CCTTCTACTACGCCGCCTCCTACCACT	4	<lmxll>	ll	135	lm	137		
>ref-161314	CACACATGCACGTTAACTCCCTCACTG	2	<lmxll>	ll	126	lm	131		
>ref-161314	CACACATGCACGTTAACTCCCTCACTG	16	<nnxnp>	nn	135	np	123		
>ref-161325	CTCCTTCCAACTCCATCTCCTTCCTTC	1	<lmxll>	ll	1	lm	266		
>ref-161325	CTCCTTCCAACTCCATCTCCTTCCTTC	6	<lmxll>	ll	2	lm	266		
>ref-161371	ACAGCCCCCACCCCCTCTCCCTCCTCC	5	<hkxhk>	hh	6	hk	252	kk	0
>ref-161371	ACAGCCCCCACCCCCTCTCCCTCCTCC	22	<hkxhk>	hh	0	hk	252	kk	5
>ref-161386	CAACACAACACCCCCCCTCCCACACAC	25	<nnxnp>	nn	248	np	0		
>ref-161386	CAACACAACACCCCCCCTCCCACACAC	27	<nnxnp>	nn	249	np	0		
>ref-161399	CCTCCCTTCACATCCCCTCCCTCTTCC	7	<lmxll>	ll	113	lm	124		
>ref-161399	CCTCCCTTCACATCCCCTCCCTCTTCC	12	<lmxll>	ll	89	lm	150		
>ref-161416	CGGAGATTAACCATACCTCCATCATCA	6	<hkxhk>	hh	17	hk	248	kk	0
>ref-161421	CTCTGCCCAACCTCTCCTCCTTCCTCC	3	<hkxhk>	hh	11	hk	248	kk	0
>ref-161421	CTCTGCCCAACCTCTCCTCCTTCCTCC	8	<nnxnp>	nn	139	np	118		
>ref-161429	CTCCTAAGCACGCCCACTCCTAAGCAC	23	<nnxnp>	nn	190	np	41		
>ref-161460	CCCTCCCGCACCCTCACTCCCTATGAT	8	<nnxnp>	nn	126	np	140		
>ref-161461	CTTTCATGCACACACACTCCAACCCCT	25	<nnxnp>	nn	136	np	126		
>ref-161476	GTATAAATCACCCCCTCTCCTCCTCAC	7	<lmxll>	ll	109	lm	136		
>ref-161478	CCAGTCGGGACGTCGTCTCCTCCGGAG	22	<hkxhk>	hh	85	hk	180	kk	0
>ref-161482	TCATCATCAACTTCTTCTCCTTTGGCT	22	<lmxll>	ll	133	lm	134		
>ref-161483	CATAACCCCACCACTTCTCCCTCACCC	4	<nnxnp>	nn	144	np	90		
>ref-161483	CATAACCCCACCACTTCTCCCTCACCC	7	<nnxnp>	nn	145	np	90		
>ref-161483	CATAACCCCACCACTTCTCCCTCACCC	12	<nnxnp>	nn	129	np	121		
>ref-161483	CATAACCCCACCACTTCTCCCTCACCC	14	<nnxnp>	nn	130	np	121		
>ref-161483	CATAACCCCACCACTTCTCCCTCACCC	26	<lmxll>	ll	71	lm	170		
>ref-161490	CCTTCACTCACGTCACCTCCCTCACCT	1	<nnxnp>	nn	203	np	65		
>ref-161490	CCTTCACTCACGTCACCTCCCTCACCT	2	<nnxnp>	nn	205	np	62		
>ref-161493	CATATAAATACATGAACTCCAGCACGC	9	<nnxnp>	nn	231	np	0		
>ref-161519	ACAGGTGTGACCTCATCTCCTACAGTG	25	<hkxhk>	hh	0	hk	197	kk	61
>ref-161519	ACAGGTGTGACCTCATCTCCTACAGTG	27	<hkxhk>	hh	0	hk	196	kk	61

>ref-161540	CTGCCAAGCACCCTCCCTCCCCCTGCC	12	<hkxhk>	hh	0	hk	273	kk	0
>ref-161540	CTGCCAAGCACCCTCCCTCCCCCTGCC	13	<lmxll>	ll	113	lm	139		
>ref-161541	CCAGTTCGGACGACGTCTCCGAACCGC	5	<lmxll>	ll	130	lm	127		
>ref-161541	CCAGTTCGGACGACGTCTCCGAACCGC	8	<hkxhk>	hh	0	hk	121	kk	123
>ref-161541	CCAGTTCGGACGACGTCTCCGAACCGC	15	<nnxnp>	nn	239	np	21		
>ref-161541	CCAGTTCGGACGACGTCTCCGAACCGC	16	<hkxhk>	hh	0	hk	72	kk	176
>ref-161541	CCAGTTCGGACGACGTCTCCGAACCGC	25	<hkxhk>	hh	121	hk	135	kk	0
>ref-161545	GACTGTCTCACCCTCACTCCCACCCCA	2	<lmxll>	ll	20	lm	244		
>ref-161545	GACTGTCTCACCCTCACTCCCACCCCA	12	<lmxll>	ll	3	lm	269		
>ref-161548	CTTCTGCCGACGGCCGCTCCCCGGGAG	21	<nnxnp>	nn	101	np	136		
>ref-161558	TCCTTTCCTACTCCCTCTCCCTTTACA	12	<nnxnp>	nn	135	np	118		
>ref-161569	CAGAGAATAACGGTGTCTCCATACGTG	7	<lmxll>	ll	122	lm	147		
>ref-161569	CAGAGAATAACGGTGTCTCCATACGTG	13	<lmxll>	ll	122	lm	147		
>ref-161573	CTCACGCAGACTCGTCCTCCTGCTCCT	5	<lmxll>	ll	152	lm	120		
>ref-161588	CCATCTACAACTATCCCTCCTCCTCTA	7	<hkxhk>	hh	50	hk	157	kk	46
>ref-161600	CTTTTCGTTACTGTTACTCCCTCTTTC	8	<hkxhk>	hh	55	hk	145	kk	46
>ref-161616	ATCCTATATACATTTGCTCCCACGCTA	7	<nnxnp>	nn	115	np	117		
>ref-161625	ACAACGACTACAGACACTCCACGACTT	26	<nnxnp>	nn	127	np	118		
>ref-161630	TCCTGCCTTACATCCTCTCCTAGACCT	1	<lmxll>	ll	63	lm	196		
>ref-161630	TCCTGCCTTACATCCTCTCCTAGACCT	12	<lmxll>	ll	63	lm	195		
>ref-161644	TCACACTTCACTTCCTCTCCTCCCTTT	3	<lmxll>	ll	187	lm	70		
>ref-161644	TCACACTTCACTTCCTCTCCTCCCTTT	7	<lmxll>	ll	189	lm	69		
>ref-161647	AACTTTATTACCAACGCTCCCTTTACG	5	<lmxll>	ll	103	lm	146		
>ref-161647	AACTTTATTACCAACGCTCCCTTTACG	12	<lmxll>	ll	146	lm	99		
>ref-161708	CCATATCTCACCATAACTCCTTTCCTC	7	<lmxll>	ll	144	lm	124		
>ref-161708	CCATATCTCACCATAACTCCTTTCCTC	24	<nnxnp>	nn	125	np	133		
>ref-161710	TCCTCTTTCACTCCCACTCCCCCCCAC	12	<hkxhk>	hh	1	hk	251	kk	5
>ref-161724	CCCGCCCACACCGGCCCTCCTCCGCCT	24	<nnxnp>	nn	133	np	137		
>ref-161732	TTAAGTGACACTGTGACTCCCTTGGTA	7	<nnxnp>	nn	117	np	145		
>ref-161736	TACAGGTCTACATCTGCTCCAGGAACT	15	<hkxhk>	hh	6	hk	179	kk	68
>ref-161736	TACAGGTCTACATCTGCTCCAGGAACT	22	<hkxhk>	hh	4	hk	237	kk	13
>ref-161819	ATATTATAAACTGATGCTCCATTAACT	2	<hkxhk>	hh	17	hk	237	kk	0
>ref-161819	ATATTATAAACTGATGCTCCATTAACT	12	<hkxhk>	hh	17	hk	237	kk	0
>ref-161835	AAAACACACACACACCCTCCCTCGCCC	1	<nnxnp>	nn	270	np	1		
>ref-161835	AAAACACACACACACCCTCCCTCGCCC	3	<nnxnp>	nn	18	np	247		
>ref-161835	AAAACACACACACACCCTCCCTCGCCC	4	<hkxhk>	hh	6	hk	266	kk	0
>ref-161835	AAAACACACACACACCCTCCCTCGCCC	12	<nnxnp>	nn	272	np	0		
>ref-161835	AAAACACACACACACCCTCCCTCGCCC	14	<nnxnp>	nn	272	np	0		

>ref-161835	AAAACACACACACACCCTCCCTCGCCC	22	<nnxnp>	nn	272	np	0		
>ref-161835	AAAACACACACACACCCTCCCTCGCCC	24	<hkxhk>	hh	1	hk	264	kk	0
>ref-161835	AAAACACACACACACCCTCCCTCGCCC	25	<nnxnp>	nn	272	np	0		
>ref-161835	AAAACACACACACACCCTCCCTCGCCC	27	<nnxnp>	nn	271	np	0		
>ref-161838	AACACAGAAACCCCCTCTCCCTCCGCG	23	<lmxll>	ll	9	lm	220		
>ref-161855	CAGTGGAGAACCATCGCTCCAGCGAGG	13	<hkxhk>	hh	0	hk	273	kk	0
>ref-161912	AATCTTTGCACCTTCCCTCCTTCTCTA	24	<lmxll>	ll	127	lm	128		
>ref-161942	AACGTGGCAACAAGATCTCCTACATGC	26	<nnxnp>	nn	262	np	0		
>ref-161946	CTCGGGTCTACTCACGCTCCTCCTCCA	26	<lmxll>	ll	140	lm	131		
>ref-161977	TGGTAGGAAACTTGTTCTCCCCCTCAC	2	<hkxhk>	hh	0	hk	268	kk	0
>ref-161977	TGGTAGGAAACTTGTTCTCCCCCTCAC	24	<hkxhk>	hh	0	hk	269	kk	1
>ref-161989	CTCCTCCCAACATCCCCTCCCCCCTAC	9	<hkxhk>	hh	4	hk	254	kk	0
>ref-161989	CTCCTCCCAACATCCCCTCCCCCCTAC	15	<hkxhk>	hh	3	hk	244	kk	7
>ref-161991	ATATGGCCGACACCTTCTCCTTCTCCT	26	<nnxnp>	nn	131	np	126		
>ref-161993	GAGCCTCGAACATCACCTCCGCTAATT	6	<lmxll>	ll	140	lm	125		
>ref-161993	GAGCCTCGAACATCACCTCCGCTAATT	22	<lmxll>	ll	130	lm	134		
>ref-162003	CGAACAGTTACACGACCTCCTGCCGTT	26	<lmxll>	ll	142	lm	124		
>ref-162057	CCTCCGCCCACCCTCTCTCCTACCATA	6	<lmxll>	ll	124	lm	121		
>ref-162058	TCTCTCTTCACCCTGTCTCCCTTCTCC	15	<hkxhk>	hh	0	hk	268	kk	2
>ref-162058	TCTCTCTTCACCCTGTCTCCCTTCTCC	21	<hkxhk>	hh	4	hk	268	kk	0
>ref-162063	CCCTCACCCACCTTTCCTCCTTCACAT	3	<lmxll>	ll	126	lm	138		
>ref-162071	TGCTTAGCCACAAACTCTCCACCAGAA	6	<hkxhk>	hh	19	hk	251	kk	0
>ref-162071	TGCTTAGCCACAAACTCTCCACCAGAA	23	<hkxhk>	hh	221	hk	40	kk	0
>ref-162090	TGGACCATAACACTCTCTCCCTTTCCC	5	<hkxhk>	hh	59	hk	143	kk	58
>ref-162090	TGGACCATAACACTCTCTCCCTTTCCC	15	<nnxnp>	nn	126	np	135		
>ref-162118	TCAGAATTAACGGTCCCTCCCGCACGC	7	<nnxnp>	nn	112	np	151		
>ref-162155	TTCATCTCTACTTCTCCTCCTATCTTC	14	<hkxhk>	hh	2	hk	250	kk	5
>ref-162155	TTCATCTCTACTTCTCCTCCTATCTTC	22	<hkxhk>	hh	2	hk	250	kk	5
>ref-162161	CTTACAACCACAACCCCTCCCCCTTAA	16	<nnxnp>	nn	105	np	145		
>ref-162168	CTTCCCTTAACCCTTACTCCTCACCCT	24	<lmxll>	ll	134	lm	113		
>ref-162210	ATACGGGAAACTTAAGCTCCTGTGAGC	27	<nnxnp>	nn	244	np	0		
>ref-162214	TCCCCCCTTACAACCTCTCCTTTACAC	27	<nnxnp>	nn	115	np	140		
>ref-162215	CATACCATCACCACTTCTCCTTCTATT	25	<lmxll>	ll	105	lm	124		
>ref-162233	AAGAAACACACAGAGACTCCGCTTACC	2	<lmxll>	ll	125	lm	140		
>ref-162233	AAGAAACACACAGAGACTCCGCTTACC	23	<lmxll>	ll	125	lm	140		
>ref-162238	CGCGAGAGCACGACATCTCCTCCATCA	5	<nnxnp>	nn	116	np	109		
>ref-162246	TCCTAATACACCCCTCCTCCCTCCCAC	4	<nnxnp>	nn	138	np	88		
>ref-162246	TCCTAATACACCCCTCCTCCCTCCCAC	27	<nnxnp>	nn	87	np	135		

>ref-162256	CTCTCAACTACCCTCCCTCCCTCTTTC	25	<nnxnp>	nn	180	np	65		
>ref-162261	TACTTACTTACCTACTCTCCCTTCCTA	23	<nnxnp>	nn	169	np	80		
>ref-162294	TCTCTTCCCACCTTTCCTCCCTTTCCT	13	<nnxnp>	nn	126	np	110		
>ref-162294	TCTCTTCCCACCTTTCCTCCCTTTCCT	21	<nnxnp>	nn	139	np	102		
>ref-162295	GAATCTCTTACATTCTCTCCTCGGATG	2	<nnxnp>	nn	148	np	113		
>ref-162295	GAATCTCTTACATTCTCTCCTCGGATG	4	<hkxhk>	hh	0	hk	104	kk	149
>ref-162295	GAATCTCTTACATTCTCTCCTCGGATG	9	<nnxnp>	nn	45	np	208		
>ref-162295	GAATCTCTTACATTCTCTCCTCGGATG	14	<hkxhk>	hh	0	hk	104	kk	149
>ref-162295	GAATCTCTTACATTCTCTCCTCGGATG	15	<hkxhk>	hh	0	hk	173	kk	71
>ref-162295	GAATCTCTTACATTCTCTCCTCGGATG	16	<lmxll>	ll	11	lm	251		
>ref-162295	GAATCTCTTACATTCTCTCCTCGGATG	23	<nnxnp>	nn	106	np	139		
>ref-162301	GCTGTGTACACACCCTCTCCCTTACCT	21	<nnxnp>	nn	119	np	141		
>ref-162307	ATCTGGGCCACAGCTCCTCCTCCAGGT	26	<lmxll>	ll	141	lm	129		
>ref-162358	CACCTCCTTACTTCCTCTCCTCACCTC	2	<nnxnp>	nn	122	np	129		
>ref-162364	TCGTCACGTACCCACTCTCCACGCATA	14	<lmxll>	ll	156	lm	116		
>ref-162369	TCTCCCCATACTCCCCCTCCCCCACTC	4	<nnxnp>	nn	146	np	98		
>ref-162369	TCTCCCCATACTCCCCCTCCCCCACTC	24	<nnxnp>	nn	142	np	101		
>ref-162373	CGCTGACTCACCTGTTCTCCCTCGCCA	21	<nnxnp>	nn	122	np	145		
>ref-162406	TGTCCAGGCACTTTTACTCCATCCCCA	9	<nnxnp>	nn	124	np	126		
>ref-162435	AGAGAACCAACCAAACCTCCATTTGCT	4	<lmxll>	ll	122	lm	129		
>ref-162435	AGAGAACCAACCAAACCTCCATTTGCT	9	<nnxnp>	nn	106	np	139		
>ref-162463	CCTTCTCCTACCATCTCTCCGCTTTAT	24	<lmxll>	ll	100	lm	123		
>ref-162465	CCTTATTAAACACCACCTCCTCCTCCT	14	<nnxnp>	nn	116	np	125		
>ref-162477	CTGCTGAGCACTCCACCTCCACGTGAG	8	<hkxhk>	hh	60	hk	145	kk	59
>ref-162484	AATCCCGAAACCTCGACTCCGCCGCTT	22	<hkxhk>	hh	61	hk	145	kk	57
>ref-162498	CATATGACCACCCTCCCTCCCATCCCT	21	<hkxhk>	hh	1	hk	265	kk	3
>ref-162498	CATATGACCACCCTCCCTCCCATCCCT	25	<hkxhk>	hh	1	hk	265	kk	3
>ref-162526	ACATTCGCCACCAAACCTCCTTCCCCG	24	<lmxll>	ll	135	lm	131		
>ref-162527	CCTTTCCCTACCTCATCTCCCCTAGAC	4	<hkxhk>	hh	45	hk	126	kk	65
>ref-162548	TTCACCCCAACCAACCCTCCTGTACAC	6	<lmxll>	ll	130	lm	114		
>ref-162548	TTCACCCCAACCAACCCTCCTGTACAC	21	<hkxhk>	hh	49	hk	136	kk	65
>ref-162568	GGCTGAATAACGGGGACTCCTCGTGTG	7	<lmxll>	ll	135	lm	136		
>ref-162575	AATCTCATAACATTCCCTCCCGTGGCG	24	<lmxll>	ll	112	lm	111		
>ref-162578	AACACGTGCACACCCGCTCCCAGACGC	7	<lmxll>	ll	7	lm	260		
>ref-162578	AACACGTGCACACCCGCTCCCAGACGC	8	<lmxll>	ll	7	lm	260		
>ref-162578	AACACGTGCACACCCGCTCCCAGACGC	26	<hkxhk>	hh	0	hk	202	kk	68
>ref-162585	TCTGAACAAACTTTCTCTCCTTCTGTG	26	<lmxll>	ll	145	lm	113		
>ref-162600	GAATTTAATACCTTTCCTCCGGCCGCC	23	<lmxll>	ll	130	lm	106		

>ref-162611	AATGAGACAACAATAACTCCTTTGAAA	2	<nnxnp>	nn	143	np	125		
>ref-162611	AATGAGACAACAATAACTCCTTTGAAA	8	<lmxll>	ll	148	lm	108		
>ref-162611	AATGAGACAACAATAACTCCTTTGAAA	14	<lmxll>	ll	1	lm	271		
>ref-162611	AATGAGACAACAATAACTCCTTTGAAA	21	<lmxll>	ll	213	lm	41		
>ref-162611	AATGAGACAACAATAACTCCTTTGAAA	26	<lmxll>	ll	86	lm	175		
>ref-162663	GTTAGTGTAACCTGCTCTCCCCGAGAC	27	<hkxhk>	hh	11	hk	255	kk	0
>ref-162677	TGATCGCTCACTCCTCCTCCTTTCGCC	22	<nnxnp>	nn	125	np	131		
>ref-162680	CCTAAGACTACACCCACTCCCTCTCTC	12	<nnxnp>	nn	9	np	257		
>ref-162689	TACACACATACAACTACTCCTTCTCTC	3	<nnxnp>	nn	127	np	143		
>ref-162699	TGCCACCCAACCACGCCTCCTCTTCCA	27	<nnxnp>	nn	129	np	143		
>ref-162700	TGCGCAACAACAGCATCTCCCAAACAT	9	<nnxnp>	nn	131	np	136		
>ref-162702	TCATCATCCACAACCTCTCCATCCTAC	6	<nnxnp>	nn	119	np	151		
>ref-162715	TTGGACATGACCTAGACTCCACTGTGC	13	<lmxll>	ll	116	lm	130		
>ref-162718	GGTCCAGTGACATCCCCTCCGCAACCA	3	<lmxll>	ll	100	lm	132		
>ref-162718	GGTCCAGTGACATCCCCTCCGCAACCA	21	<lmxll>	ll	97	lm	131		
>ref-162749	CTACGTGTTACTCTTGCTCCTTTTATT	3	<hkxhk>	hh	43	hk	154	kk	62
>ref-162753	ACACAATACACTCCCTCTCCTTCTCCT	4	<nnxnp>	nn	132	np	137		
>ref-162753	ACACAATACACTCCCTCTCCTTCTCCT	9	<nnxnp>	nn	130	np	137		
>ref-162754	CTTTCCCTGACCTTTTCTCCTTCGGAT	3	<lmxll>	ll	0	lm	266		
>ref-162754	CTTTCCCTGACCTTTTCTCCTTCGGAT	25	<lmxll>	ll	0	lm	266		
>ref-162790	TCCTTCGCTACGCTTCCTCCACTTTCT	12	<hkxhk>	hh	55	hk	155	kk	42
>ref-162790	TCCTTCGCTACGCTTCCTCCACTTTCT	14	<hkxhk>	hh	55	hk	155	kk	42
>ref-162796	TGCTTGCGCACTTACCCTCCCGCCCTC	24	<lmxll>	ll	265	lm	0		
>ref-162821	TTTGCCCTCACTCTTCCTCCTCGCTCA	6	<lmxll>	ll	116	lm	146		
>ref-162838	ACTGTCTCTACTCCCTCTCCCCCCAGT	25	<nnxnp>	nn	133	np	129		
>ref-162847	GTGGGTGCCACTGGTGCTCCTGAATCT	2	<lmxll>	ll	1	lm	270		
>ref-162847	GTGGGTGCCACTGGTGCTCCTGAATCT	25	<lmxll>	ll	1	lm	270		
>ref-162859	AATACATACACAAACACTCCCATCCAC	13	<nnxnp>	nn	77	np	160		
>ref-162859	AATACATACACAAACACTCCCATCCAC	24	<nnxnp>	nn	78	np	160		
>ref-162861	CATCATCCTACCCCCCCTCCACCTATC	6	<hkxhk>	hh	0	hk	199	kk	52
>ref-162861	CATCATCCTACCCCCCCTCCACCTATC	21	<hkxhk>	hh	52	hk	199	kk	0
>ref-162863	ATCATCTAAACTGGGTCTCCCCGCGCT	6	<lmxll>	ll	144	lm	126		
>ref-162864	TTGTCCCACACTTCTCCTCCCTTATCT	3	<hkxhk>	hh	36	hk	219	kk	2
>ref-162866	TCTTTCATCACCTTCTCTCCGTTCTTC	7	<nnxnp>	nn	5	np	231		
>ref-162885	CTCTACCCAACCGCCTCTCCACCCACT	13	<nnxnp>	nn	270	np	1		
>ref-162885	CTCTACCCAACCGCCTCTCCACCCACT	25	<nnxnp>	nn	272	np	0		
>ref-162895	ACCTCTCCTACTTCTCCTCCTCTCCTA	1	<hkxhk>	hh	1	hk	261	kk	0
>ref-162900	TTTCAATATACAAACACTCCGATGCCT	2	<hkxhk>	hh	0	hk	267	kk	2

>ref-162900	TTTCAATATACAAACACTCCGATGCCT	4	<lmxll>	ll	150	lm	102		
>ref-162900	TTTCAATATACAAACACTCCGATGCCT	8	<lmxll>	ll	0	lm	271		
>ref-162900	TTTCAATATACAAACACTCCGATGCCT	9	<hkxhk>	hh	0	hk	267	kk	2
>ref-162900	TTTCAATATACAAACACTCCGATGCCT	14	<lmxll>	ll	150	lm	101		
>ref-162905	TCCCCTCCTACCTCCCCTCCCTATCCA	14	<lmxll>	ll	265	lm	1		
>ref-162905	TCCCCTCCTACCTCCCCTCCCTATCCA	23	<lmxll>	ll	255	lm	4		
>ref-162913	GTAGGTTACACAGAAACTCCTCGGCAG	24	<nnxnp>	nn	128	np	112		
>ref-162913	GTAGGTTACACAGAAACTCCTCGGCAG	27	<lmxll>	ll	95	lm	139		
>ref-162947	CTCTTTCCCACCCTCTCTCCTCCAACT	21	<nnxnp>	nn	124	np	105		
>ref-162975	AACTAGCTAACTTATTCTCCTTGGTTG	25	<hkxhk>	hh	67	hk	133	kk	64
>ref-162985	GCCCTTCTAACAGCCCCTCCCTGCCTG	15	<lmxll>	ll	142	lm	120		
>ref-163055	GTTTGATTCACTTGACCTCCATCACAA	7	<hkxhk>	hh	0	hk	239	kk	3
>ref-163055	GTTTGATTCACTTGACCTCCATCACAA	9	<hkxhk>	hh	8	hk	261	kk	0
>ref-163055	GTTTGATTCACTTGACCTCCATCACAA	12	<hkxhk>	hh	0	hk	257	kk	9
>ref-163055	GTTTGATTCACTTGACCTCCATCACAA	26	<hkxhk>	hh	9	hk	256	kk	0
>ref-163055	GTTTGATTCACTTGACCTCCATCACAA	27	<hkxhk>	hh	114	hk	137	kk	0
>ref-163062	CGGAGCACCACGGAGGCTCCCCGTCCT	25	<nnxnp>	nn	265	np	0		
>ref-163073	TATATCCACACCTTCACTCCCTTCATC	9	<hkxhk>	hh	2	hk	182	kk	86
>ref-163073	TATATCCACACCTTCACTCCCTTCATC	12	<hkxhk>	hh	0	hk	254	kk	13
>ref-163073	TATATCCACACCTTCACTCCCTTCATC	27	<hkxhk>	hh	0	hk	254	kk	14
>ref-163091	TCAGAATTCACTCTCTCTCCTTCCCTC	1	<hkxhk>	hh	0	hk	261	kk	7
>ref-163091	TCAGAATTCACTCTCTCTCCTTCCCTC	2	<hkxhk>	hh	0	hk	261	kk	7
>ref-163094	AGCCGTCACACTCGCGCTCCGCTGACC	26	<nnxnp>	nn	126	np	136		
>ref-163096	ATTTTATCAACGGATACTCCACTTGTG	25	<nnxnp>	nn	130	np	107		
>ref-163143	CATTTCTATACCATTTCTCCCTCTCCT	13	<hkxhk>	hh	3	hk	266	kk	0
>ref-163143	CATTTCTATACCATTTCTCCCTCTCCT	21	<hkxhk>	hh	0	hk	266	kk	3
>ref-163153	TTTTAAACCACCTTCTCTCCCTCTCCT	9	<nnxnp>	nn	105	np	135		
>ref-163173	ATCTACACAACAGACCCTCCTCATCCA	21	<nnxnp>	nn	124	np	141		
>ref-163229	AGGATCTTCACTTACTCTCCCGTGCGA	2	<lmxll>	ll	115	lm	145		
>ref-163256	GCTTTCACCACCGACGCTCCTCCGCTT	9	<lmxll>	ll	144	lm	112		
>ref-163259	CTTACCCGCACCTACACTCCGCCTACC	2	<lmxll>	ll	123	lm	120		
>ref-163260	ATGCCCGTAACGAGCACTCCGACACAG	12	<hkxhk>	hh	53	hk	160	kk	53
>ref-163270	TTTGTGCATACCCTCTCTCCCTCTCTC	4	<hkxhk>	hh	0	hk	258	kk	7
>ref-163270	TTTGTGCATACCCTCTCTCCCTCTCTC	7	<hkxhk>	hh	7	hk	259	kk	0
>ref-163279	CAGTACCTAACAAACTCTCCCTAAGGA	14	<lmxll>	ll	102	lm	138		
>ref-163297	AATTTATATACATGTTCTCCTACCGCT	7	<hkxhk>	hh	57	hk	143	kk	54
>ref-163300	TCCCCTTCAACCCCTTCTCCACCCCTT	9	<hkxhk>	hh	1	hk	262	kk	0
>ref-163300	TCCCCTTCAACCCCTTCTCCACCCCTT	25	<hkxhk>	hh	1	hk	262	kk	0

>ref-163320	TCAGGTATCACTGAGCCTCCCCCTGGA	14	<nnxnp>	nn	234	np	0		
>ref-163324	ATCGCCTCGACAATTCCTCCTCTTTCA	4	<lmxll>	ll	139	lm	122		
>ref-163334	TCACGTGTGACACTATCTCCAACTCGC	23	<lmxll>	ll	136	lm	117		
>ref-163346	TTACGTCCTACTCCCTCTCCTCTCTCT	12	<lmxll>	ll	144	lm	125		
>ref-163346	TTACGTCCTACTCCCTCTCCTCTCTCT	21	<nnxnp>	nn	131	np	140		
>ref-163351	AAGACAACAACATGCGCTCCTCGTATT	4	<lmxll>	ll	104	lm	164		
>ref-163351	AAGACAACAACATGCGCTCCTCGTATT	27	<hkxhk>	hh	47	hk	125	kk	96
>ref-163366	CCTGGTGAGACTGCACCTCCAGGCTGA	3	<hkxhk>	hh	0	hk	187	kk	53
>ref-163393	GTATCGTGTACTTGCTCTCCCGGAGAA	2	<lmxll>	ll	122	lm	131		
>ref-163398	CACCAAAACACATGGCCTCCCTTTCCA	14	<lmxll>	ll	138	lm	131		
>ref-163410	GTCTCAACAACTCCTTCTCCGTCTCAC	27	<lmxll>	ll	143	lm	106		
>ref-163418	GACGGCAGGACACTCGCTCCTAAACCC	3	<lmxll>	ll	131	lm	132		
>ref-163438	AGGGCGTCCACTTTCTCTCCCCTTCCT	27	<lmxll>	ll	261	lm	0		
>ref-163451	CTCCTCCCGACGCGCCCTCCTCCAGAA	15	<nnxnp>	nn	130	np	134		
>ref-163451	CTCCTCCCGACGCGCCCTCCTCCAGAA	26	<nnxnp>	nn	130	np	134		
>ref-163459	TTCTGCATCACTTTCTCTCCCTTTCTC	1	<hkxhk>	hh	0	hk	273	kk	0
>ref-163459	TTCTGCATCACTTTCTCTCCCTTTCTC	9	<hkxhk>	hh	0	hk	273	kk	0
>ref-163475	ACTTCTCTGACTCCCCCTCCCTGCTCT	1	<hkxhk>	hh	67	hk	156	kk	44
>ref-163480	AATTCCCTCACTCTTTCTCCTTTTTTA	14	<hkxhk>	hh	31	hk	203	kk	1
>ref-163480	AATTCCCTCACTCTTTCTCCTTTTTTA	15	<hkxhk>	hh	31	hk	203	kk	1
>ref-163502	TCCTACCTTACTCTCCCTCCTCACCCC	25	<nnxnp>	nn	133	np	125		
>ref-163506	TTCACTCGTACCCAACCTCCTTCCCCT	21	<nnxnp>	nn	137	np	126		
>ref-163541	CTATGCTTCACCTTCCCTCCCCTTCTC	5	<nnxnp>	nn	118	np	143		
>ref-163542	TCCAGCCGCACGCCTCCTCCTCCGTCT	3	<lmxll>	ll	120	lm	145		
>ref-163544	CCCTCTTAAACCCTCTCTCCCTTCTTC	15	<lmxll>	ll	127	lm	134		
>ref-163565	ATTCTGTTGACTCGCTCTCCCTTTCTT	9	<lmxll>	ll	122	lm	136		
>ref-163582	TATCCCCCTACCCCCGCTCCTTTCCCT	2	<lmxll>	ll	185	lm	72		
>ref-163582	TATCCCCCTACCCCCGCTCCTTTCCCT	13	<nnxnp>	nn	178	np	85		
>ref-163582	TATCCCCCTACCCCCGCTCCTTTCCCT	16	<hkxhk>	hh	0	hk	266	kk	4
>ref-163582	TATCCCCCTACCCCCGCTCCTTTCCCT	24	<nnxnp>	nn	43	np	210		
>ref-163582	TATCCCCCTACCCCCGCTCCTTTCCCT	25	<lmxll>	ll	96	lm	134		
>ref-163585	TACCGATTCACCTATTCTCCCAGACCG	7	<hkxhk>	hh	0	hk	272	kk	1
>ref-163590	CCACCCGTCACCCTCCCTCCTTCCGTC	2	<nnxnp>	nn	117	np	140		
>ref-163590	CCACCCGTCACCCTCCCTCCTTCCGTC	23	<hkxhk>	hh	2	hk	259	kk	0
>ref-163590	CCACCCGTCACCCTCCCTCCTTCCGTC	25	<hkxhk>	hh	1	hk	260	kk	1
>ref-163592	TCACGCGCCACTTACCCTCCACGTTCT	1	<lmxll>	ll	164	lm	82		
>ref-163594	CACAGACACACAAACTCTCCTTTACAC	26	<hkxhk>	hh	69	hk	121	kk	65
>ref-163606	ACCTAGACAACTGATCCTCCCTTTAAA	9	<nnxnp>	nn	134	np	121		

>ref-163611	TCTCCTCCAACTCTGTCTCCATCTCCT	15	<lmxll>	ll	12	lm	216		
>ref-163611	TCTCCTCCAACTCTGTCTCCATCTCCT	27	<lmxll>	ll	12	lm	216		
>ref-163627	CTGTCTTTCACTTTCCCTCCCCCTTTC	1	<nnxnp>	nn	6	np	262		
>ref-163627	CTGTCTTTCACTTTCCCTCCCCCTTTC	22	<nnxnp>	nn	271	np	1		
>ref-163662	CCCTAGAGCACTTCCTCTCCACAGCTG	2	<hkxhk>	hh	0	hk	273	kk	0
>ref-163662	CCCTAGAGCACTTCCTCTCCACAGCTG	24	<lmxll>	ll	38	lm	222		
>ref-163664	ATTCACTGTACGCTGGCTCCAAGCACC	22	<lmxll>	ll	139	lm	126		
>ref-163669	CCCGCTCACACACCATCTCCCGCCCAC	6	<hkxhk>	hh	52	hk	140	kk	59
>ref-163671	TAACGGATGACGTCATCTCCGGTTACC	21	<nnxnp>	nn	129	np	134		
>ref-163687	CATCGACGCACACACACTCCCTCATAC	26	<lmxll>	ll	122	lm	115		
>ref-163689	ATTCTCCCCACCTTCCCTCCCTTTCTG	23	<nnxnp>	nn	104	np	148		
>ref-163695	TGTGCAATAACGCCAGCTCCTTAAATC	25	<lmxll>	ll	266	lm	0		
>ref-163703	CTGAGACTAACCCCCCCTCCTCCGGCT	25	<nnxnp>	nn	131	np	110		
>ref-163715	AGTCTTTCCACAGAGCCTCCTGATAAG	14	<nnxnp>	nn	132	np	118		
>ref-163731	AATGTCTTCACCTTAGCTCCCCAACTA	14	<nnxnp>	nn	122	np	117		
>ref-163747	GGTTGGCACACAAGCACTCCTGTGCGA	15	<nnxnp>	nn	129	np	128		
>ref-163750	TCTTTAAACACCCAACCTCCTCCCTTT	9	<lmxll>	ll	125	lm	124		
>ref-163750	TCTTTAAACACCCAACCTCCTCCCTTT	16	<lmxll>	ll	125	lm	124		
>ref-163757	TGGTTTTGTACTTGTCCTCCAAGAGCT	24	<lmxll>	ll	112	lm	151		
>ref-163817	TCTCTACCCACCCCCTCTCCCCTTCCC	9	<nnxnp>	nn	10	np	257		
>ref-163817	TCTCTACCCACCCCCTCTCCCCTTCCC	15	<nnxnp>	nn	10	np	257		
>ref-163817	TCTCTACCCACCCCCTCTCCCCTTCCC	22	<hkxhk>	hh	0	hk	183	kk	71
>ref-163817	TCTCTACCCACCCCCTCTCCCCTTCCC	24	<hkxhk>	hh	0	hk	183	kk	68
>ref-163833	CCTTCATTGACTCGCCCTCCCTCCCTC	2	<nnxnp>	nn	31	np	228		
>ref-163833	CCTTCATTGACTCGCCCTCCCTCCCTC	16	<nnxnp>	nn	61	np	191		
>ref-163843	TTCAACCTGACACTCCCTCCAGTGACG	9	<nnxnp>	nn	259	np	1		
>ref-163844	TATATGTATACACAGTCTCCTTTCTCT	25	<nnxnp>	nn	117	np	103		
>ref-163870	TAATCACCGACACAGCCTCCTCTTCAC	4	<lmxll>	ll	140	lm	127		
>ref-163885	ATTCTCATTACTCCCACTCCCTCTTTC	1	<nnxnp>	nn	245	np	0		
>ref-163885	ATTCTCATTACTCCCACTCCCTCTTTC	4	<nnxnp>	nn	248	np	0		
>ref-163886	CCTCTTTCTACGTGTCCTCCTCTTCCC	12	<lmxll>	ll	125	lm	128		
>ref-163886	CCTCTTTCTACGTGTCCTCCTCTTCCC	27	<lmxll>	ll	123	lm	128		
>ref-163916	ATGCTAATGACTCAGTCTCCTTCTTCA	1	<nnxnp>	nn	145	np	119		
>ref-163916	ATGCTAATGACTCAGTCTCCTTCTTCA	5	<hkxhk>	hh	3	hk	270	kk	0
>ref-163924	TTCCACCAAACGTTTCCTCCTCCTTCC	22	<hkxhk>	hh	0	hk	271	kk	0
>ref-163926	CCCCCCTCTACCCGCACTCCCTCCTCC	16	<nnxnp>	nn	240	np	11		
>ref-163926	CCCCCCTCTACCCGCACTCCCTCCTCC	22	<nnxnp>	nn	237	np	10		
>ref-163951	TTACTTCACACTCCCTCTCCCTCATCC	23	<hkxhk>	hh	68	hk	124	kk	38

>ref-163954	TCCTACCCCACCTCTCCTCCCTCCCCC	15	<nnxnp>	nn	259	np	3		
>ref-164043	CTTCCCCCAACGTCCCCTCCTGAAGAG	2	<nnxnp>	nn	266	np	0		
>ref-164046	CCTCCCCTCACCCACCCTCCTCTTACT	1	<nnxnp>	nn	234	np	2		
>ref-164106	CACAGACTCACTCACTCTCCAGCGGCA	5	<lmxll>	ll	121	lm	141		
>ref-164109	CCCATTTCTACCATCTCTCCCCTCCCT	14	<nnxnp>	nn	253	np	13		
>ref-164109	CCCATTTCTACCATCTCTCCCCTCCCT	22	<nnxnp>	nn	252	np	13		
>ref-164127	TTTTTGTTGACAGCAACTCCGCAGTCT	2	<nnxnp>	nn	119	np	123		
>ref-164127	TTTTTGTTGACAGCAACTCCGCAGTCT	16	<hkxhk>	hh	61	hk	135	kk	54
>ref-164129	CTCCTCCCCACGCCCACTCCCCTCCTC	13	<hkxhk>	hh	74	hk	165	kk	2
>ref-164129	CTCCTCCCCACGCCCACTCCCCTCCTC	23	<nnxnp>	nn	159	np	84		
>ref-164139	CGCTCAGCCACTCTTGCTCCCAACTCC	5	<lmxll>	ll	116	lm	132		
>ref-164146	CCTTTCCTCACCCTCCCTCCCATTCCC	14	<lmxll>	ll	196	lm	56		
>ref-164146	CCTTTCCTCACCCTCCCTCCCATTCCC	24	<lmxll>	ll	197	lm	56		
>ref-164156	GTGTTTCCCACACGTGCTCCCACCCCT	6	<hkxhk>	hh	1	hk	272	kk	0
>ref-164165	CTCACCCTTACCTCTTCTCCATTCGTC	8	<lmxll>	ll	137	lm	132		
>ref-164175	TGATTCATTACTCATCCTCCTTGATTG	26	<lmxll>	ll	138	lm	126		
>ref-164184	ACACTCAACACCCTAACTCCGGGTTCG	26	<nnxnp>	nn	133	np	127		
>ref-164206	TGTAACCTCACCTAACCTCCCCTTTTC	23	<lmxll>	ll	113	lm	107		
>ref-164214	TTTATCGCTACAGAATCTCCTTTCACA	8	<hkxhk>	hh	55	hk	112	kk	52
>ref-164215	TTTGCTTATACCTTGTCTCCCTCTCCC	5	<hkxhk>	hh	54	hk	152	kk	56
>ref-164234	ACTCTCCCCACACACTCTCCCCCCACA	14	<nnxnp>	nn	51	np	192		
>ref-164234	ACTCTCCCCACACACTCTCCCCCCACA	16	<nnxnp>	nn	50	np	190		
>ref-164258	GTGAGGGGTACATTTTCTCCACCACCA	24	<hkxhk>	hh	99	hk	106	kk	45
>ref-164267	ACATCGATAACGTGATCTCCGCACAGT	6	<nnxnp>	nn	125	np	145		
>ref-164279	CCCTGACTTACCCGTCCTCCCGCTTGT	14	<nnxnp>	nn	269	np	0		
>ref-164290	AGCCAAAATACACGCCCTCCCTCCCCT	1	<nnxnp>	nn	126	np	143		
>ref-164290	AGCCAAAATACACGCCCTCCCTCCCCT	2	<hkxhk>	hh	0	hk	254	kk	13
>ref-164290	AGCCAAAATACACGCCCTCCCTCCCCT	3	<hkxhk>	hh	0	hk	254	kk	13
>ref-164345	GCCCTCTCCACATCTGCTCCCTTCTCT	3	<nnxnp>	nn	126	np	145		
>ref-164371	TCTCTTATCACCTGTTCTCCCTTAGTA	2	<nnxnp>	nn	124	np	139		
>ref-164371	TCTCTTATCACCTGTTCTCCCTTAGTA	6	<hkxhk>	hh	69	hk	131	kk	57
>ref-164379	CTACTGTCAACGCAAGCTCCACGTTGT	7	<hkxhk>	hh	104	hk	81	kk	42
>ref-164404	TCAGTGCGAACCCTCTCTCCTCACAAG	7	<lmxll>	ll	109	lm	132		
>ref-164407	CATACATCTACAAAAGCTCCACAGCCT	1	<nnxnp>	nn	261	np	0		
>ref-164407	CATACATCTACAAAAGCTCCACAGCCT	24	<nnxnp>	nn	109	np	143		
>ref-164412	GCCTCTTTCACTCGTTCTCCACTTCCT	8	<nnxnp>	nn	136	np	129		
>ref-164412	GCCTCTTTCACTCGTTCTCCACTTCCT	26	<lmxll>	ll	132	lm	133		
>ref-164419	TCTCCCATAACTCCCTCTCCCTTCGTT	8	<nnxnp>	nn	87	np	165		

>ref-164420	AAACCTCCTACGTTTACTCCATGAGTG	23	<nnxnp>	nn	125	np	104		
>ref-164420	AAACCTCCTACGTTTACTCCATGAGTG	27	<nnxnp>	nn	124	np	104		
>ref-164464	ACATCAACCACAACCCCTCCTCTCCCC	2	<lmxll>	ll	263	lm	0		
>ref-164467	TTTGCCCCCACCCCTTCTCCTCCACTT	5	<lmxll>	ll	264	lm	0		
>ref-164467	TTTGCCCCCACCCCTTCTCCTCCACTT	6	<lmxll>	ll	264	lm	0		
>ref-164475	CACCGTGGGACTGTACCTCCTGGCGTA	5	<nnxnp>	nn	106	np	139		
>ref-164486	TACTAACCCACACATCCTCCCTGCTTC	7	<nnxnp>	nn	129	np	134		
>ref-164492	AACAGGAACACAACGCCTCCAACTTTC	8	<nnxnp>	nn	114	np	152		
>ref-164492	AACAGGAACACAACGCCTCCAACTTTC	26	<nnxnp>	nn	273	np	0		
>ref-164497	GGTTGCCAAACGCCTCCTCCTTCTCCA	14	<hkxhk>	hh	73	hk	195	kk	0
>ref-164529	ATAACTGCAACACCAGCTCCAACAAAT	2	<lmxll>	ll	126	lm	125		
>ref-164568	CTCTCCCTCACCCCATCTCCTCTTGTT	16	<lmxll>	ll	8	lm	255		
>ref-164568	CTCTCCCTCACCCCATCTCCTCTTGTT	25	<lmxll>	ll	8	lm	255		
>ref-164574	TTCATTTGTACTGTGTCTCCTCGGTCT	5	<hkxhk>	hh	56	hk	139	kk	56
>ref-164609	GGTTATGTGACGCTTCCTCCTTTCCAT	1	<lmxll>	ll	139	lm	121		
>ref-164612	TTTCCCTTAACCCACTCTCCCCCCTTC	9	<lmxll>	ll	154	lm	81		
>ref-164612	TTTCCCTTAACCCACTCTCCCCCCTTC	23	<lmxll>	ll	40	lm	202		
>ref-164612	TTTCCCTTAACCCACTCTCCCCCCTTC	24	<lmxll>	ll	154	lm	81		
>ref-164628	GTCCTTCCTACCTTCTCTCCTCCCCCT	1	<lmxll>	ll	251	lm	3		
>ref-164628	GTCCTTCCTACCTTCTCTCCTCCCCCT	9	<lmxll>	ll	264	lm	1		
>ref-164628	GTCCTTCCTACCTTCTCTCCTCCCCCT	24	<lmxll>	ll	265	lm	1		
>ref-164628	GTCCTTCCTACCTTCTCTCCTCCCCCT	26	<lmxll>	ll	270	lm	0		
>ref-164635	CTCTCTGCCACTTGCTCTCCCTCATCC	23	<hkxhk>	hh	59	hk	156	kk	50
>ref-164682	CCCTCTGACACTCGATCTCCTGATCCT	2	<nnxnp>	nn	254	np	0		
>ref-164691	CTCTCCTTTACCCCCCCTCCCCCCACC	6	<nnxnp>	nn	172	np	48		
>ref-164708	CTATTCCCCACCTCTACTCCCCCTTCC	3	<hkxhk>	hh	10	hk	260	kk	1
>ref-164708	CTATTCCCCACCTCTACTCCCCCTTCC	13	<hkxhk>	hh	1	hk	260	kk	10
>ref-164709	TTCCACATCACTGGCTCTCCTGTCTGC	2	<nnxnp>	nn	123	np	127		
>ref-164718	GTCTGTCTGACTGTCTCTCCCTACCTT	13	<lmxll>	ll	145	lm	121		
>ref-164718	GTCTGTCTGACTGTCTCTCCCTACCTT	25	<lmxll>	ll	145	lm	121		
>ref-164721	AATGAACAAACTAAAACTCCTTATTCA	13	<nnxnp>	nn	254	np	15		
>ref-164736	CCGCCTGTTACCACCACTCCATGTCCT	5	<nnxnp>	nn	143	np	128		
>ref-164765	TCCTCCTCCACTTCCTCTCCGACCTCT	16	<lmxll>	ll	142	lm	125		
>ref-164765	TCCTCCTCCACTTCCTCTCCGACCTCT	22	<nnxnp>	nn	161	np	90		
>ref-164765	TCCTCCTCCACTTCCTCTCCGACCTCT	23	<nnxnp>	nn	152	np	118		
>ref-164766	CCGACAGTCACCCTCCCTCCCTCCTTC	14	<nnxnp>	nn	159	np	65		
>ref-164766	CCGACAGTCACCCTCCCTCCCTCCTTC	26	<nnxnp>	nn	156	np	65		
>ref-164767	TCTGGCATGACCCGAGCTCCCAAACGT	14	<nnxnp>	nn	268	np	0		

>ref-164775	CATCTTCCCACCCCTTCTCCTTTTCCT	13	<lmxll>	ll	129	lm	124		
>ref-164788	TGCCCCTTCACGTTTTCTCCTCTTTCA	6	<hkxhk>	hh	129	hk	139	kk	0
>ref-164788	TGCCCCTTCACGTTTTCTCCTCTTTCA	12	<nnxnp>	nn	130	np	140		
>ref-164788	TGCCCCTTCACGTTTTCTCCTCTTTCA	21	<nnxnp>	nn	130	np	140		
>ref-164802	CTCACACGCACCAGTACTCCCACAACC	8	<lmxll>	ll	4	lm	261		
>ref-164802	CTCACACGCACCAGTACTCCCACAACC	15	<lmxll>	ll	4	lm	261		
>ref-164814	TTGCCATCCACCCTTCCTCCACATCTT	6	<lmxll>	ll	103	lm	162		
>ref-164829	TGCAATGCCACTTTAGCTCCGTTCAAG	5	<lmxll>	ll	125	lm	126		
>ref-164867	CACCTTTCGACGTAATCTCCCTCCTTA	21	<lmxll>	ll	128	lm	131		
>ref-164867	CACCTTTCGACGTAATCTCCCTCCTTA	23	<lmxll>	ll	129	lm	136		
>ref-164869	GTGCCGAAAACCGAACCTCCTGTCCAA	23	<nnxnp>	nn	115	np	151		
>ref-164873	ACATCGAAGACCAAATCTCCATCTCTA	21	<lmxll>	ll	119	lm	130		
>ref-164876	AAAACACGAACAGTATCTCCGTGGATT	12	<hkxhk>	hh	46	hk	130	kk	52
>ref-164886	CTCACCGACACACCCTCTCCTTTCCTC	7	<nnxnp>	nn	99	np	138		
>ref-164886	CTCACCGACACACCCTCTCCTTTCCTC	9	<nnxnp>	nn	99	np	137		
>ref-164899	CTTTCTGACACCCTCCCTCCTTCCCTA	27	<lmxll>	ll	126	lm	128		
>ref-164900	CCTGGATGAACCTCAGCTCCGACCCCT	9	<lmxll>	ll	113	lm	118		
>ref-164900	CCTGGATGAACCTCAGCTCCGACCCCT	26	<nnxnp>	nn	107	np	125		
>ref-164914	GACGCCAATACCCCCTCTCCTTCCCCT	4	<hkxhk>	hh	0	hk	258	kk	1
>ref-164927	TGCCTTTCTACACTTCCTCCGCTCTCT	6	<hkxhk>	hh	66	hk	139	kk	66
>ref-164932	ATGAATGAAACGCCCACTCCCTCGCCC	3	<lmxll>	ll	113	lm	152		
>ref-164996	CACCCTTTAACAGTTTCTCCTTAGATT	22	<lmxll>	ll	58	lm	186		
>ref-165021	CATCACTTCACCCCCTCTCCTCCTTCC	2	<lmxll>	ll	18	lm	231		
>ref-165021	CATCACTTCACCCCCTCTCCTCCTTCC	5	<lmxll>	ll	19	lm	229		
>ref-165055	ATCCAAGATACTTCAACTCCGTCCAGT	14	<nnxnp>	nn	125	np	135		
>ref-165057	CACTCCCCCACTCGAGCTCCCTCACTC	21	<hkxhk>	hh	0	hk	265	kk	1
>ref-165096	ACCTTAACCACCATCCCTCCAAGTTCC	5	<nnxnp>	nn	241	np	0		
>ref-165102	TTCCCTCTCACCTCATCTCCATTTGCC	3	<hkxhk>	hh	63	hk	142	kk	43
>ref-165103	TTTTCCTCTACCTTCTCTCCCCTTTCC	1	<hkxhk>	hh	0	hk	81	kk	164
>ref-165103	TTTTCCTCTACCTTCTCTCCCCTTTCC	2	<lmxll>	ll	29	lm	227		
>ref-165103	TTTTCCTCTACCTTCTCTCCCCTTTCC	3	<lmxll>	ll	83	lm	156		
>ref-165103	TTTTCCTCTACCTTCTCTCCCCTTTCC	9	<hkxhk>	hh	0	hk	80	kk	166
>ref-165103	TTTTCCTCTACCTTCTCTCCCCTTTCC	21	<nnxnp>	nn	9	np	263		
>ref-165103	TTTTCCTCTACCTTCTCTCCCCTTTCC	23	<nnxnp>	nn	8	np	263		
>ref-165106	CTTCTCGGTACATTTACTCCGCGTTTG	25	<lmxll>	ll	134	lm	126		
>ref-165111	CACATAATAACACCACCTCCACAATTG	26	<hkxhk>	hh	38	hk	136	kk	45
>ref-165119	CTTTTCTCTACCCTCCCTCCTTCCCTA	1	<hkxhk>	hh	11	hk	260	kk	0
>ref-165119	CTTTTCTCTACCCTCCCTCCTTCCCTA	14	<hkxhk>	hh	0	hk	260	kk	11

>ref-165151	ACCCCTCCAACATGTCCTCCCTAGTTA	4	<hkxhk>	hh	4	hk	264	kk	0
>ref-165151	ACCCCTCCAACATGTCCTCCCTAGTTA	24	<hkxhk>	hh	4	hk	264	kk	0
>ref-165161	CCCGCTCTCACTCTGTCTCCTCGCCGA	23	<lmxll>	ll	132	lm	88		
>ref-165161	CCCGCTCTCACTCTGTCTCCTCGCCGA	25	<lmxll>	ll	133	lm	89		
>ref-165176	GCGCTTATAACGACGACTCCTTGCCAT	14	<nnxnp>	nn	123	np	145		
>ref-165176	GCGCTTATAACGACGACTCCTTGCCAT	25	<nnxnp>	nn	143	np	129		
>ref-165187	GCACAATTAACTGCTCCTCCCTCCCTC	8	<lmxll>	ll	110	lm	136		
>ref-165212	CAGCGATTGACACCCTCTCCTCCTCCA	13	<lmxll>	ll	132	lm	128		
>ref-165222	CGCTCATACACTTTCCCTCCGTTCCTC	8	<nnxnp>	nn	123	np	136		
>ref-165227	CTCTCAGCCACGTCCTCTCCTTTACAC	8	<lmxll>	ll	133	lm	136		
>ref-165238	ACTCTTCCCACCTTATCTCCAACCCAC	21	<lmxll>	ll	121	lm	105		
>ref-165266	TTTCTCCCTACCCACGCTCCTTTGCAA	13	<nnxnp>	nn	68	np	176		
>ref-165266	TTTCTCCCTACCCACGCTCCTTTGCAA	27	<nnxnp>	nn	184	np	69		
>ref-165278	TACCGCCACACGCATCCTCCGCGTCAC	4	<nnxnp>	nn	131	np	141		
>ref-165296	ATACCAACTACCCCCTCTCCCCTGATA	13	<nnxnp>	nn	6	np	260		
>ref-165296	ATACCAACTACCCCCTCTCCCCTGATA	23	<nnxnp>	nn	64	np	183		
>ref-165355	AGAGTCAGCACAGCTCCTCCGTTGCAT	2	<lmxll>	ll	145	lm	126		
>ref-165367	CTTCCCCTGACTCTCCCTCCTCCCTCT	15	<lmxll>	ll	126	lm	143		
>ref-165372	ACGCTGGGAACCCCCACTCCTTCTCTT	6	<lmxll>	ll	125	lm	144		
>ref-165373	TCTCTCTTCACCTTCCCTCCTCACCCT	23	<nnxnp>	nn	270	np	1		
>ref-165373	TCTCTCTTCACCTTCCCTCCTCACCCT	25	<nnxnp>	nn	273	np	0		
>ref-165375	CACACACACACCTTCCCTCCTTATGCT	2	<nnxnp>	nn	136	np	135		
>ref-165386	ATGTACATTACATGCCCTCCCAGTGTC	14	<nnxnp>	nn	119	np	124		
>ref-165393	ACCGGTGTCACAGATGCTCCTTCTATA	27	<nnxnp>	nn	138	np	131		
>ref-165397	CTCGCACGGACTCCGGCTCCACTACCA	2	<nnxnp>	nn	271	np	0		
>ref-165433	CAACCACCCACGTAACCTCCCTTCCCT	2	<lmxll>	ll	118	lm	135		
>ref-165435	TTGTTCTCTACGTATGCTCCCTGCTTC	27	<nnxnp>	nn	139	np	123		
>ref-165448	CGGGGTCTGACAGCAGCTCCAGGAGGA	8	<hkxhk>	hh	0	hk	194	kk	71
>ref-165451	CCCGCTCCTACTCCATCTCCCTCTCAA	14	<lmxll>	ll	117	lm	111		
>ref-165459	GCTCATTAAACCATCCCTCCCGTAGTT	26	<nnxnp>	nn	121	np	121		
>ref-165465	ACCACATTAACCTTCCCTCCCTACCAC	25	<nnxnp>	nn	107	np	151		
>ref-165476	TAATTGCTCACTTCCCCTCCCTCTCCT	5	<lmxll>	ll	118	lm	122		
>ref-165490	CTCTCTCCGACGGTGCCTCCGCGCCCG	5	<nnxnp>	nn	134	np	136		
>ref-165507	ATTGTGACTACTCAAACTCCTCGAAAC	5	<nnxnp>	nn	250	np	17		
>ref-165507	ATTGTGACTACTCAAACTCCTCGAAAC	15	<hkxhk>	hh	60	hk	186	kk	0
>ref-165507	ATTGTGACTACTCAAACTCCTCGAAAC	25	<hkxhk>	hh	229	hk	36	kk	0
>ref-165507	ATTGTGACTACTCAAACTCCTCGAAAC	27	<hkxhk>	hh	110	hk	130	kk	0
>ref-165509	CGCCATCATACTGTCCCTCCTCCTCCT	25	<nnxnp>	nn	260	np	0		

>ref-165550	CTTTCGTACACTTCATCTCCCTTTCCT	2	<nnxnp>	nn	264	np	0		
>ref-165550	CTTTCGTACACTTCATCTCCCTTTCCT	8	<nnxnp>	nn	263	np	0		
>ref-165557	CTTATTCTAACTTGCTCTCCGGCCGAT	16	<nnxnp>	nn	112	np	133		
>ref-165557	CTTATTCTAACTTGCTCTCCGGCCGAT	24	<nnxnp>	nn	111	np	133		
>ref-165564	TCCTCCCTCACCTCCTCTCCCCCACAG	1	<lmxll>	ll	0	lm	262		
>ref-165564	TCCTCCCTCACCTCCTCTCCCCCACAG	27	<lmxll>	ll	0	lm	263		
>ref-165574	ATACGTAAGACAAACTCTCCGTTCTCA	15	<hkxhk>	hh	50	hk	132	kk	53
>ref-165586	ACCCCCAACACCACCACTCCCAATCTC	24	<hkxhk>	hh	0	hk	5	kk	260
>ref-165586	ACCCCCAACACCACCACTCCCAATCTC	25	<hkxhk>	hh	0	hk	5	kk	261
>ref-165601	AGCCCATTTACTCACCCTCCCCAATCT	23	<hkxhk>	hh	51	hk	203	kk	0
>ref-165601	AGCCCATTTACTCACCCTCCCCAATCT	24	<hkxhk>	hh	50	hk	204	kk	0
>ref-165615	TCCACACCCACCCACTCTCCTACCCAC	15	<lmxll>	ll	6	lm	264		
>ref-165615	TCCACACCCACCCACTCTCCTACCCAC	24	<lmxll>	ll	6	lm	264		
>ref-165630	CCCTTATCCACGCCCACTCCCTCCCCA	25	<hkxhk>	hh	74	hk	123	kk	42
>ref-165630	CCCTTATCCACGCCCACTCCCTCCCCA	27	<hkxhk>	hh	71	hk	123	kk	42
>ref-165647	TGTTTAGTCACGTGACCTCCCGCCCCG	22	<hkxhk>	hh	0	hk	267	kk	5
>ref-165647	TGTTTAGTCACGTGACCTCCCGCCCCG	27	<lmxll>	ll	191	lm	70		
>ref-165649	ACCCAGCCGACCTAATCTCCATCGACA	12	<hkxhk>	hh	56	hk	132	kk	61
>ref-165658	CTCCCCTCTACCCCACCTCCCCCTCAC	3	<hkxhk>	hh	31	hk	220	kk	0
>ref-165658	CTCCCCTCTACCCCACCTCCCCCTCAC	8	<hkxhk>	hh	31	hk	218	kk	0
>ref-165684	TTCTTATCCACATTTCCTCCTCTCCCT	16	<lmxll>	ll	129	lm	128		
>ref-165711	CGCTCCATTACCATTTCTCCAGAAACA	5	<nnxnp>	nn	109	np	125		
>ref-165717	CCCCTCCTGACCCGGACTCCCTCGCGG	5	<hkxhk>	hh	0	hk	228	kk	31
>ref-165717	CCCCTCCTGACCCGGACTCCCTCGCGG	8	<lmxll>	ll	0	lm	268		
>ref-165717	CCCCTCCTGACCCGGACTCCCTCGCGG	24	<lmxll>	ll	0	lm	268		
>ref-165733	CCTGTAGCAACAGCCACTCCGTCAGCA	13	<lmxll>	ll	55	lm	194		
>ref-165733	CCTGTAGCAACAGCCACTCCGTCAGCA	26	<hkxhk>	hh	17	hk	250	kk	0
>ref-165739	GATCTACCTACCCCAACTCCCTGTGTT	14	<hkxhk>	hh	35	hk	142	kk	54
>ref-165782	CTCACCCCCACTCTTCCTCCCCATCCC	12	<hkxhk>	hh	0	hk	265	kk	0
>ref-165782	CTCACCCCCACTCTTCCTCCCCATCCC	14	<hkxhk>	hh	0	hk	265	kk	0
>ref-165789	TTCCTTCCTACCTCACCTCCCCACCCC	13	<lmxll>	ll	118	lm	115		
>ref-165805	ACTGGCACTACACATGCTCCGAGCGAC	22	<lmxll>	ll	129	lm	134		
>ref-165811	AGAGATTCTACTCGTCCTCCACGCTGT	27	<nnxnp>	nn	133	np	137		
>ref-165825	ATGGAAATGACTGAAGCTCCCAATGGA	23	<hkxhk>	hh	65	hk	134	kk	52
>ref-165829	GTTATTCTTACCTGTCCTCCTTTTGTT	7	<hkxhk>	hh	22	hk	213	kk	0
>ref-165829	GTTATTCTTACCTGTCCTCCTTTTGTT	16	<hkxhk>	hh	22	hk	214	kk	0
>ref-165833	GAGCCTAGAACCCAGCCTCCGCCTAGT	15	<hkxhk>	hh	0	hk	195	kk	71
>ref-165842	TCTACCTCTACTTTCCCTCCCTACCTC	9	<lmxll>	ll	263	lm	1		

>ref-165842	TCTACCTCTACTTTCCCTCCCTACCTC	23	<lmxll>	ll	249	lm	3		
>ref-165842	TCTACCTCTACTTTCCCTCCCTACCTC	26	<lmxll>	ll	121	lm	132		
>ref-165875	TCCGCTTTCACTCATTCTCCTCTCCTT	1	<nnxnp>	nn	119	np	141		
>ref-165875	TCCGCTTTCACTCATTCTCCTCTCCTT	2	<nnxnp>	nn	119	np	141		
>ref-165895	CACTTCTCTACTTCCTCTCCTCGTTCC	5	<nnxnp>	nn	259	np	0		
>ref-165895	CACTTCTCTACTTCCTCTCCTCGTTCC	12	<nnxnp>	nn	260	np	0		
>ref-165941	GATGGCATTACCCGTGCTCCATTTCCT	14	<lmxll>	ll	137	lm	123		
>ref-165970	TTTTCCCCCACTTTCTCTCCCTCTCCG	5	<nnxnp>	nn	139	np	91		
>ref-165972	AGAGACCCAACACGACCTCCAGCCATT	3	<hkxhk>	hh	0	hk	273	kk	0
>ref-165986	AATCCCACAACATGGCCTCCGTTTACG	6	<hkxhk>	hh	66	hk	146	kk	57
>ref-165991	CCCTCATCCACCCCTTCTCCTCTCTCT	1	<nnxnp>	nn	264	np	1		
>ref-166031	CCTGGCTGAACAGTCGCTCCCACGTTA	12	<lmxll>	ll	119	lm	149		
>ref-166035	ATATCTGCAACTATCTCTCCATAGATC	24	<hkxhk>	hh	64	hk	117	kk	45
>ref-166050	ACACAGGCAACGCGTCCTCCATCTCAT	12	<hkxhk>	hh	0	hk	271	kk	0
>ref-166092	CAGTATGTCACTTAAACTCCATATGCT	4	<hkxhk>	hh	0	hk	273	kk	0
>ref-166092	CAGTATGTCACTTAAACTCCATATGCT	22	<hkxhk>	hh	0	hk	204	kk	62
>ref-166092	CAGTATGTCACTTAAACTCCATATGCT	24	<lmxll>	ll	157	lm	112		
>ref-166099	CTCTCTCCGACGCCCTCTCCCGTGTCC	25	<lmxll>	ll	154	lm	118		
>ref-166135	CAATCGACTACTCTTTCTCCCCCTCTA	27	<nnxnp>	nn	110	np	147		
>ref-166161	CTTCTAACTACTTCCTCTCCTCCTCTC	13	<hkxhk>	hh	0	hk	271	kk	0
>ref-166161	CTTCTAACTACTTCCTCTCCTCCTCTC	21	<hkxhk>	hh	0	hk	271	kk	0
>ref-166161	CTTCTAACTACTTCCTCTCCTCCTCTC	23	<nnxnp>	nn	137	np	128		
>ref-166168	GAGTTCTCTACGAACACTCCATTCAGA	6	<nnxnp>	nn	143	np	114		
>ref-166172	TCCTTCCCAACCCCTTCTCCATTTCCC	2	<hkxhk>	hh	54	hk	126	kk	40
>ref-166190	GCATCTTCTACTCTTGCTCCTATTCCT	22	<nnxnp>	nn	128	np	118		
>ref-166209	CTCTGTTTTACTTTCTCTCCTCTCGTT	5	<nnxnp>	nn	133	np	131		
>ref-166211	TTCCTAATTACCTCATCTCCCTCTCCC	9	<lmxll>	ll	119	lm	148		
>ref-166231	ATAGCTATCACCAAACCTCCAATCACA	8	<lmxll>	ll	103	lm	166		
>ref-166233	CACCTTCTTACTTTACCTCCCGTGGCG	27	<nnxnp>	nn	240	np	0		
>ref-166263	ATTCAATAAACTATCTCTCCGTCTGCT	3	<nnxnp>	nn	119	np	116		
>ref-166295	CATACTCATACTCAACCTCCAGGACTC	8	<nnxnp>	nn	263	np	0		
>ref-166297	GATGTTAAAACCATTCCTCCTAATTCG	7	<lmxll>	ll	7	lm	263		
>ref-166297	GATGTTAAAACCATTCCTCCTAATTCG	12	<lmxll>	ll	254	lm	3		
>ref-166297	GATGTTAAAACCATTCCTCCTAATTCG	24	<hkxhk>	hh	0	hk	262	kk	3
>ref-166297	GATGTTAAAACCATTCCTCCTAATTCG	26	<lmxll>	ll	254	lm	1		
>ref-166297	GATGTTAAAACCATTCCTCCTAATTCG	27	<hkxhk>	hh	0	hk	272	kk	1
>ref-166327	CGCTATGCAACAACTGCTCCACTTGTG	23	<nnxnp>	nn	268	np	0		
>ref-166346	CCCACCCTCACCCCTCCTCCCTTACAA	4	<nnxnp>	nn	71	np	150		

>ref-166346	CCCACCCTCACCCCTCCTCCCTTACAA	25	<nnxnp>	nn	74	np	149		
>ref-166371	CAAGCGCTGACGGCCTCTCCTCTCTCG	23	<nnxnp>	nn	134	np	119		
>ref-166386	CTCAGAATGACTTAAACTCCCTACCTT	27	<lmxll>	ll	0	lm	270		
>ref-166392	GGTGCTGAAACAATTACTCCCACAAAC	22	<nnxnp>	nn	116	np	140		
>ref-166395	CTGCCTCGTACTCCGCCTCCCCCTTTT	23	<hkxhk>	hh	1	hk	228	kk	0
>ref-166408	TGCTCGTTTACTTCCTCTCCTTGTATC	14	<lmxll>	ll	127	lm	139		
>ref-166408	TGCTCGTTTACTTCCTCTCCTTGTATC	27	<nnxnp>	nn	139	np	132		
>ref-166409	GCCCGTATCACCCTCCCTCCTACTCCT	8	<hkxhk>	hh	62	hk	129	kk	47
>ref-166412	TCCTCTTCCACTGCATCTCCCCCGTTT	15	<hkxhk>	hh	50	hk	150	kk	58
>ref-166414	TCCCTCTCCACCCCCTCTCCTCAGTCC	21	<nnxnp>	nn	130	np	133		
>ref-166414	TCCCTCTCCACCCCCTCTCCTCAGTCC	24	<nnxnp>	nn	129	np	135		
>ref-166430	CCCTCTTCCACTCTTCCTCCACTTCCC	6	<lmxll>	ll	2	lm	268		
>ref-166430	CCCTCTTCCACTCTTCCTCCACTTCCC	9	<lmxll>	ll	190	lm	47		
>ref-166430	CCCTCTTCCACTCTTCCTCCACTTCCC	14	<lmxll>	ll	180	lm	60		
>ref-166430	CCCTCTTCCACTCTTCCTCCACTTCCC	15	<lmxll>	ll	2	lm	268		
>ref-166443	TCTTATCTCACTTCCTCTCCCATCTCC	4	<lmxll>	ll	106	lm	142		
>ref-166456	ATTTCCCTTACATGTTCTCCCATTCCC	23	<nnxnp>	nn	268	np	0		
>ref-166485	CCTTAACCCACCCCCTCTCCTCCATAC	27	<nnxnp>	nn	259	np	0		
>ref-166497	GCTCCTCCCACTCCTCCTCCTAGATGG	1	<hkxhk>	hh	0	hk	4	kk	215
>ref-166497	GCTCCTCCCACTCCTCCTCCTAGATGG	9	<hkxhk>	hh	213	hk	6	kk	0
>ref-166501	GGGAGGACAACTGCCCCTCCCCCCCAA	14	<nnxnp>	nn	127	np	124		
>ref-166503	TCCCGCAAGACGCAACCTCCTCAGCAT	15	<nnxnp>	nn	125	np	142		
>ref-166512	TATATACACACACTGACTCCTTTGGAA	3	<hkxhk>	hh	54	hk	138	kk	66
>ref-166512	TATATACACACACTGACTCCTTTGGAA	5	<lmxll>	ll	121	lm	141		
>ref-166512	TATATACACACACTGACTCCTTTGGAA	7	<hkxhk>	hh	65	hk	138	kk	54
>ref-166538	TTCACACATACTCAAACTCCCACACTC	25	<hkxhk>	hh	68	hk	143	kk	46
>ref-166554	GAAGTCCCCACTGCGACTCCCTCGGCC	24	<nnxnp>	nn	268	np	0		
>ref-166560	GTCTCCCTTACCCGGCCTCCCCCTTAC	27	<hkxhk>	hh	9	hk	260	kk	0
>ref-166564	ATTGAATGTACCAATTCTCCTTCTCTG	4	<hkxhk>	hh	0	hk	272	kk	0
>ref-166564	ATTGAATGTACCAATTCTCCTTCTCTG	6	<hkxhk>	hh	0	hk	272	kk	0
>ref-166628	CAGTTTTGCACCTCGCCTCCCTCTTAA	15	<lmxll>	ll	124	lm	121		
>ref-166630	TCTTACTTCACTATCCCTCCCTCCTCT	7	<hkxhk>	hh	56	hk	140	kk	64
>ref-166664	TTGACTTCGACGTCCTCTCCGCAAGCC	3	<lmxll>	ll	132	lm	106		
>ref-166665	CTACTCCTTACTCCTTCTCCCTTTCCG	3	<nnxnp>	nn	266	np	0		
>ref-166697	CCCGTCGTTACTACAACTCCCTCCGCC	13	<nnxnp>	nn	119	np	134		
>ref-166698	GGACAGGGCACGCCTCCTCCGTCGCCA	3	<lmxll>	ll	122	lm	147		
>ref-166698	GGACAGGGCACGCCTCCTCCGTCGCCA	5	<hkxhk>	hh	74	hk	140	kk	45
>ref-166711	CCCCCAACCACTTTTTCTCCCTCGCCC	23	<nnxnp>	nn	260	np	0		

>ref-166720	CTAATTCCCACTCCTACTCCCTTTCCC	13	<lmxll>	ll	126	lm	113		
>ref-166737	GAAGACCATACTAATCCTCCCGGCATG	23	<nnxnp>	nn	143	np	108		
>ref-166743	AAAAGTTCGACTCCACCTCCATTCCTA	4	<hkxhk>	hh	0	hk	273	kk	0
>ref-166743	AAAAGTTCGACTCCACCTCCATTCCTA	15	<nnxnp>	nn	205	np	54		
>ref-166770	CGGCCGGTGACTCGAACTCCCGAACTC	2	<lmxll>	ll	179	lm	76		
>ref-166770	CGGCCGGTGACTCGAACTCCCGAACTC	15	<lmxll>	ll	7	lm	254		
>ref-166770	CGGCCGGTGACTCGAACTCCCGAACTC	22	<lmxll>	ll	179	lm	76		
>ref-166770	CGGCCGGTGACTCGAACTCCCGAACTC	25	<lmxll>	ll	7	lm	254		
>ref-166772	CTCGTCTTCACATCCTCTCCCTTTCTC	27	<hkxhk>	hh	8	hk	131	kk	96
>ref-166782	CGCTCACTCACCTTTTCTCCTACCCTT	21	<nnxnp>	nn	260	np	0		
>ref-166785	ATCGTCACAACGGTATCTCCCCTTTAC	22	<nnxnp>	nn	130	np	137		
>ref-166785	ATCGTCACAACGGTATCTCCCCTTTAC	25	<nnxnp>	nn	130	np	137		
>ref-166793	GACATTTACACAAACCCTCCCTTGAGA	24	<lmxll>	ll	134	lm	113		
>ref-166799	ATTTTCATAACCTCATCTCCGCCAAGT	21	<nnxnp>	nn	132	np	98		
>ref-166801	AACAACTGAACATTTTCTCCTTCCCTT	22	<nnxnp>	nn	127	np	129		
>ref-166824	CTCCTAAATACATTTCCTCCTTGACAA	9	<nnxnp>	nn	264	np	1		
>ref-166831	CTTCATCACACTATCTCTCCCTATTTC	24	<nnxnp>	nn	256	np	0		
>ref-166831	CTTCATCACACTATCTCTCCCTATTTC	25	<nnxnp>	nn	254	np	0		
>ref-166838	GAACTCTCCACCCCTCCTCCTCTCCCT	23	<hkxhk>	hh	0	hk	255	kk	6
>ref-166838	GAACTCTCCACCCCTCCTCCTCTCCCT	25	<hkxhk>	hh	6	hk	255	kk	0
>ref-166848	ACTTCTACCACCACCACTCCTACTACC	6	<nnxnp>	nn	4	np	222		
>ref-166848	ACTTCTACCACCACCACTCCTACTACC	12	<nnxnp>	nn	4	np	223		
>ref-166883	TCGTCATGCACATCACCTCCATTTTGT	26	<hkxhk>	hh	63	hk	109	kk	48
>ref-166885	TTACATGTTACTTCATCTCCAGATTAA	21	<nnxnp>	nn	101	np	131		
>ref-166892	ACTAGATCTACCAGTCCTCCTGAACAG	3	<nnxnp>	nn	129	np	126		
>ref-166895	TACCTCCCTACCTTTCCTCCTCTCTTC	24	<lmxll>	ll	24	lm	235		
>ref-166895	TACCTCCCTACCTTTCCTCCTCTCTTC	26	<lmxll>	ll	22	lm	237		
>ref-166902	GAAAAAGAAACCTTCTCTCCGGTGGCA	7	<nnxnp>	nn	148	np	110		
>ref-166912	CTCCTATCCACCACCTCTCCACCTCTC	21	<nnxnp>	nn	39	np	206		
>ref-166926	CATGCAGACACACACGCTCCTTCATGC	3	<lmxll>	ll	130	lm	138		
>ref-166928	GTCATTTTTACCCTCCCTCCATTCTTC	21	<lmxll>	ll	105	lm	153		
>ref-166934	TGCGCATATACGTAGTCTCCTTTCTTC	5	<lmxll>	ll	121	lm	122		
>ref-166936	ACCTCTTTCACAACTTCTCCTTTCCCT	15	<lmxll>	ll	128	lm	131		
>ref-166940	TTAATCCCAACATCATCTCCCTCTCAT	24	<lmxll>	ll	106	lm	141		
>ref-166971	CCTACCTCAACGATGCCTCCGTCCTGT	2	<hkxhk>	hh	0	hk	272	kk	0
>ref-166971	CCTACCTCAACGATGCCTCCGTCCTGT	5	<hkxhk>	hh	0	hk	273	kk	0
>ref-166985	CAAGCTCCCACACTCCCTCCTTTCCTT	23	<lmxll>	ll	121	lm	147		
>ref-167029	CCTCCCTTTACTCCATCTCCCCTCTCC	5	<lmxll>	ll	0	lm	266		

>ref-167029	CCTCCCTTTACTCCATCTCCCCTCTCC	15	<lmxll>	ll	0	lm	263		
>ref-167032	TGTATTCCCACACCCACTCCACCCTAT	12	<hkxhk>	hh	73	hk	116	kk	38
>ref-167052	TTACTGATTACTGATTCTCCTCCTTCT	25	<nnxnp>	nn	127	np	139		
>ref-167066	TTCCTCTTTACCATCTCTCCCTTTCTT	12	<nnxnp>	nn	136	np	117		
>ref-167069	GTCTCAGCCACATTCCCTCCCCCTGGT	23	<nnxnp>	nn	112	np	145		
>ref-167073	CCCGCTCCGACAGCGACTCCTCCGGCT	27	<hkxhk>	hh	131	hk	127	kk	3
>ref-167079	GGTCATATCACCAGCCCTCCGCCCCCA	1	<hkxhk>	hh	0	hk	271	kk	1
>ref-167079	GGTCATATCACCAGCCCTCCGCCCCCA	7	<hkxhk>	hh	5	hk	267	kk	0
>ref-167079	GGTCATATCACCAGCCCTCCGCCCCCA	13	<hkxhk>	hh	5	hk	268	kk	0
>ref-167079	GGTCATATCACCAGCCCTCCGCCCCCA	22	<hkxhk>	hh	5	hk	267	kk	0
>ref-167079	GGTCATATCACCAGCCCTCCGCCCCCA	26	<hkxhk>	hh	0	hk	237	kk	33
>ref-167079	GGTCATATCACCAGCCCTCCGCCCCCA	27	<hkxhk>	hh	30	hk	237	kk	0
>ref-167086	ATGCGTGATACTGCAACTCCTCTGCTT	9	<nnxnp>	nn	272	np	0		
>ref-167086	ATGCGTGATACTGCAACTCCTCTGCTT	27	<nnxnp>	nn	133	np	133		
>ref-167139	TACATGATAACACGCGCTCCCATGCAC	1	<nnxnp>	nn	138	np	132		
>ref-167139	TACATGATAACACGCGCTCCCATGCAC	6	<hkxhk>	hh	72	hk	143	kk	55
>ref-167170	TCTTCCATTACCCCAACTCCTCCCCAC	2	<lmxll>	ll	134	lm	115		
>ref-167170	TCTTCCATTACCCCAACTCCTCCCCAC	14	<lmxll>	ll	134	lm	115		
>ref-167176	ACCTAAATCACCGATACTCCATCCACC	5	<nnxnp>	nn	109	np	132		
>ref-167176	ACCTAAATCACCGATACTCCATCCACC	15	<nnxnp>	nn	109	np	132		
>ref-167179	TTCCTCCCTACTCCTTCTCCCTTCTCC	1	<lmxll>	ll	85	lm	172		
>ref-167179	TTCCTCCCTACTCCTTCTCCCTTCTCC	12	<lmxll>	ll	5	lm	264		
>ref-167179	TTCCTCCCTACTCCTTCTCCCTTCTCC	15	<nnxnp>	nn	94	np	160		
>ref-167183	TTCCTCTCCACCTCTCCTCCCTTATTT	8	<nnxnp>	nn	135	np	130		
>ref-167184	CTCCTTTCCACGGCACCTCCTTCTCCT	6	<hkxhk>	hh	57	hk	134	kk	55
>ref-167193	AATGTAGGTACTTTCTCTCCCTTCTTT	21	<lmxll>	ll	144	lm	108		
>ref-167196	CCGGCCTGCACCTAGTCTCCTTCTCTC	27	<lmxll>	ll	136	lm	128		
>ref-167205	TGTATGGATACACGTCCTCCCTCTCTC	1	<hkxhk>	hh	0	hk	268	kk	2
>ref-167205	TGTATGGATACACGTCCTCCCTCTCTC	7	<hkxhk>	hh	0	hk	196	kk	74
>ref-167205	TGTATGGATACACGTCCTCCCTCTCTC	14	<hkxhk>	hh	0	hk	271	kk	0
>ref-167205	TGTATGGATACACGTCCTCCCTCTCTC	27	<hkxhk>	hh	31	hk	234	kk	0
>ref-167221	ACCTTCACCACCTCCTCTCCCTCGCTT	12	<lmxll>	ll	240	lm	26		
>ref-167221	ACCTTCACCACCTCCTCTCCCTCGCTT	23	<hkxhk>	hh	6	hk	260	kk	0
>ref-167221	ACCTTCACCACCTCCTCTCCCTCGCTT	25	<lmxll>	ll	253	lm	16		
>ref-167259	AATGTGAGTACTTGGGCTCCATCATGT	8	<hkxhk>	hh	58	hk	137	kk	63
>ref-167259	AATGTGAGTACTTGGGCTCCATCATGT	12	<lmxll>	ll	130	lm	138		
>ref-167260	TTCACCCTTACTTCTTCTCCTTCTCTC	4	<nnxnp>	nn	259	np	0		
>ref-167260	TTCACCCTTACTTCTTCTCCTTCTCTC	13	<nnxnp>	nn	263	np	0		

>ref-167289	TACTCATTTACTCGTCCTCCTTTTCCT	15	<nnxnp>	nn	118	np	149		
>ref-167289	TACTCATTTACTCGTCCTCCTTTTCCT	16	<nnxnp>	nn	116	np	149		
>ref-167308	TTTCCTCAGACACATACTCCCCATTTG	1	<hkxhk>	hh	0	hk	257	kk	7
>ref-167314	ACAGTTAAGACTCTCACTCCCTAAAAC	13	<nnxnp>	nn	112	np	109		
>ref-167323	CACCTCTCCACTTTTCCTCCTCTTCCT	13	<nnxnp>	nn	55	np	202		
>ref-167323	CACCTCTCCACTTTTCCTCCTCTTCCT	25	<nnxnp>	nn	128	np	127		
>ref-167325	GGATAATAGACGATGCCTCCAAAGTGA	1	<lmxll>	ll	0	lm	255		
>ref-167325	GGATAATAGACGATGCCTCCAAAGTGA	16	<lmxll>	ll	129	lm	108		
>ref-167345	TTTTTTCTTACTGCTCCTCCCCCTCCC	22	<nnxnp>	nn	115	np	112		
>ref-167358	ATCCACGCAACACCGTCTCCAGCTGTG	7	<hkxhk>	hh	0	hk	270	kk	2
>ref-167358	ATCCACGCAACACCGTCTCCAGCTGTG	13	<hkxhk>	hh	2	hk	270	kk	0
>ref-167373	TGACCGCTCACTCTTTCTCCGCACCCT	22	<lmxll>	ll	140	lm	129		
>ref-167379	GTTCCCTTTACTTTACCTCCTTGCTTA	4	<nnxnp>	nn	118	np	134		
>ref-167381	TCCTTCGCCACCTTATCTCCTCTCCTC	9	<nnxnp>	nn	126	np	134		
>ref-167485	CACATACGCACTCACGCTCCCCTCCCC	12	<nnxnp>	nn	120	np	148		
>ref-167485	CACATACGCACTCACGCTCCCCTCCCC	21	<nnxnp>	nn	122	np	147		
>ref-167517	ATGATATACACAGAGGCTCCCTACATT	1	<lmxll>	ll	266	lm	0		
>ref-167600	TGAGAATAAACTGCTCCTCCGTCGCCT	21	<hkxhk>	hh	50	hk	152	kk	63
>ref-167615	TCGATCCCCACCCTCCCTCCTTCCTCC	2	<lmxll>	ll	262	lm	0		
>ref-167615	TCGATCCCCACCCTCCCTCCTTCCTCC	4	<lmxll>	ll	244	lm	7		
>ref-167615	TCGATCCCCACCCTCCCTCCTTCCTCC	21	<lmxll>	ll	250	lm	5		
>ref-167616	ACACTTGCAACCGACTCTCCGGCTCAT	25	<nnxnp>	nn	106	np	160		
>ref-167621	ATTTGAACAACACAGCCTCCCAGCAGC	7	<lmxll>	ll	109	lm	114		
>ref-167642	AGCTGTTTGACTCTCGCTCCACGCACT	23	<nnxnp>	nn	101	np	162		
>ref-167647	CCTCTCTTCACTTCATCTCCTCATACA	9	<nnxnp>	nn	121	np	132		
>ref-167662	TGATGCTGGACTGAGGCTCCTCCTCCA	26	<lmxll>	ll	127	lm	139		
>ref-167685	TACACCAGTACCCTCTCTCCCTCTCTC	25	<lmxll>	ll	126	lm	130		
>ref-167686	TTTACCTACACCCTCCCTCCTCCCTTC	7	<lmxll>	ll	127	lm	130		
>ref-167689	GAGACTTAAACAGACACTCCGACAAAT	6	<lmxll>	ll	12	lm	252		
>ref-167689	GAGACTTAAACAGACACTCCGACAAAT	7	<lmxll>	ll	12	lm	253		
>ref-167702	GGGAATTGAACTCGTACTCCAGTGCTC	3	<lmxll>	ll	39	lm	208		
>ref-167702	GGGAATTGAACTCGTACTCCAGTGCTC	15	<hkxhk>	hh	4	hk	233	kk	0
>ref-167706	ACGAGTATTACTGCGGCTCCCAATACC	27	<lmxll>	ll	136	lm	101		
>ref-167751	ACCTGCCGCACGCCGCCTCCGCTCTGG	14	<nnxnp>	nn	135	np	128		
>ref-167762	CCAAAGGGGACCCTAGCTCCCTAAAGC	25	<lmxll>	ll	132	lm	129		
>ref-167804	TCATGACTCACCTCTCCTCCTCGCTCA	23	<hkxhk>	hh	0	hk	263	kk	6
>ref-167856	CTGCAAGCCACTGTTCCTCCAGTCCTC	25	<hkxhk>	hh	0	hk	273	kk	0
>ref-167865	GGTCTCACAACAATTACTCCAGGTCTG	23	<hkxhk>	hh	0	hk	181	kk	72

>ref-167918	TATATCCTAACACCTTCTCCTTGCATT	9	<nnxnp>	nn	138	np	133		
>ref-167929	TCTTGAATGACTCAGACTCCAAAGCAC	14	<nnxnp>	nn	117	np	139		
>ref-167936	TTCCTCCTTACAGATCCTCCTCCTCAT	8	<nnxnp>	nn	126	np	139		
>ref-167941	CTCTCCATCACCTCATCTCCACCCATT	1	<hkxhk>	hh	76	hk	141	kk	44
>ref-167944	ACTTTTCTTACCCAACCTCCTATACCA	14	<nnxnp>	nn	263	np	0		
>ref-167944	ACTTTTCTTACCCAACCTCCTATACCA	22	<hkxhk>	hh	21	hk	214	kk	0
>ref-167947	CACAGACACACCCCCCCTCCCATCTAC	8	<nnxnp>	nn	119	np	106		
>ref-167964	TCGCCTCCTACCCTCTCTCCAACATCA	22	<nnxnp>	nn	120	np	145		
>ref-167975	CACACAGACACACACACTCCAAATAAC	7	<nnxnp>	nn	174	np	65		
>ref-167975	CACACAGACACACACACTCCAAATAAC	24	<nnxnp>	nn	221	np	1		
>ref-167979	TATCCATTCACTCCCTCTCCTCCCCTC	3	<nnxnp>	nn	136	np	119		
>ref-167979	TATCCATTCACTCCCTCTCCTCCCCTC	6	<lmxll>	ll	135	lm	120		
>ref-167979	TATCCATTCACTCCCTCTCCTCCCCTC	13	<lmxll>	ll	128	lm	130		
>ref-167979	TATCCATTCACTCCCTCTCCTCCCCTC	14	<lmxll>	ll	264	lm	0		
>ref-167980	TGTGTTCCCACAATGACTCCGGTTATA	1	<lmxll>	ll	130	lm	135		
>ref-167983	AACATGCAAACAAACACTCCAAAATAC	7	<hkxhk>	hh	11	hk	241	kk	1
>ref-167995	AACGGTACCACAAGTTCTCCACAATGC	4	<nnxnp>	nn	127	np	119		
>ref-168012	GTTGTGACAACCCTCCCTCCCCCATCT	12	<lmxll>	ll	123	lm	139		
>ref-168030	GAATGTGTAACCATCTCTCCGCTACAC	1	<nnxnp>	nn	118	np	152		
>ref-168030	GAATGTGTAACCATCTCTCCGCTACAC	2	<nnxnp>	nn	118	np	152		
>ref-168032	TTCTTCTCCACTTTCTCTCCCTCGCTC	16	<nnxnp>	nn	228	np	24		
>ref-168032	TTCTTCTCCACTTTCTCTCCCTCGCTC	24	<nnxnp>	nn	213	np	31		
>ref-168052	CACTCACTCACTCGCTCTCCAATGTCC	9	<lmxll>	ll	130	lm	117		
>ref-168065	ATAACACCTACACCTCCTCCTCCTCCC	1	<nnxnp>	nn	263	np	3		
>ref-168071	TCATCTCCCACATCATCTCCATCATCT	14	<lmxll>	ll	24	lm	236		
>ref-168071	TCATCTCCCACATCATCTCCATCATCT	21	<lmxll>	ll	23	lm	236		
>ref-168080	TCCCTCGCCACCCCTCCTCCCCCTTTT	24	<nnxnp>	nn	198	np	35		
>ref-168080	TCCCTCGCCACCCCTCCTCCCCCTTTT	27	<nnxnp>	nn	187	np	38		
>ref-168083	TACTTCTTCACTCTTCCTCCTTTCCGC	1	<hkxhk>	hh	63	hk	134	kk	63
>ref-168083	TACTTCTTCACTCTTCCTCCTTTCCGC	25	<lmxll>	ll	142	lm	120		
>ref-168084	GTAGCTTGCACCGGGCCTCCGTGAATC	13	<hkxhk>	hh	61	hk	141	kk	60
>ref-168108	CTTTCCCACACTCCCACTCCTCCCCAC	26	<lmxll>	ll	108	lm	113		
>ref-168123	TGTACATACACTTCTTCTCCTATCATC	13	<hkxhk>	hh	1	hk	263	kk	5
>ref-168123	TGTACATACACTTCTTCTCCTATCATC	23	<hkxhk>	hh	0	hk	225	kk	32
>ref-168126	CACGCACACACATTTCCTCCCTTCGCG	2	<nnxnp>	nn	130	np	140		
>ref-168126	CACGCACACACATTTCCTCCCTTCGCG	4	<nnxnp>	nn	129	np	140		
>ref-168139	AATCCCACTACCCCCCCTCCTTAGTCT	21	<nnxnp>	nn	135	np	122		
>ref-168140	GCTATTATTACCTCCACTCCTTCCTCC	6	<lmxll>	ll	148	lm	120		

>ref-168173	CTTTCCCTCACCTCTGCTCCCGCATCT	16	<nnxnp>	nn	110	np	138		
>ref-168180	CCCTTGAATACCCTCCCTCCTTTCTCT	13	<lmxll>	ll	126	lm	139		
>ref-168218	CCTCCACCCACACCCCCTCCAACGCCA	9	<hkxhk>	hh	0	hk	263	kk	1
>ref-168218	CCTCCACCCACACCCCCTCCAACGCCA	13	<hkxhk>	hh	27	hk	227	kk	0
>ref-168218	CCTCCACCCACACCCCCTCCAACGCCA	15	<hkxhk>	hh	1	hk	263	kk	0
>ref-168222	CCTTTCTCAACTTTCCCTCCCTCTTCT	14	<nnxnp>	nn	125	np	147		
>ref-168225	CACACACACACTCACCCTCCATCTTGA	15	<hkxhk>	hh	69	hk	138	kk	62
>ref-168276	CTCCTTCTCACTCGCTCTCCCTTCCAA	5	<nnxnp>	nn	127	np	143		
>ref-168284	CACACAGACACATACCCTCCCTGACCA	1	<nnxnp>	nn	143	np	124		
>ref-168286	CTGCCCATCACTCATCCTCCTCGCGAC	22	<nnxnp>	nn	145	np	128		
>ref-168288	GACCCCACTACCTGTGCTCCTCGAGGG	1	<lmxll>	ll	246	lm	6		
>ref-168322	TCCTCTACTACCACTTCTCCATTTCTT	2	<lmxll>	ll	266	lm	0		
>ref-168334	CCTGCTCTTACTTCTGCTCCTTCTGCT	4	<hkxhk>	hh	81	hk	189	kk	0
>ref-168334	CCTGCTCTTACTTCTGCTCCTTCTGCT	22	<hkxhk>	hh	0	hk	189	kk	82
>ref-168339	TCTGACGTCACTGATCCTCCCAGACAG	25	<nnxnp>	nn	121	np	141		
>ref-168341	ATCTTGGAAACGTTTGCTCCTCATAAA	13	<lmxll>	ll	24	lm	229		
>ref-168341	ATCTTGGAAACGTTTGCTCCTCATAAA	16	<lmxll>	ll	144	lm	125		
>ref-168341	ATCTTGGAAACGTTTGCTCCTCATAAA	24	<lmxll>	ll	22	lm	234		
>ref-168344	CTCCTTTCTACGTGTCCTCCTCTTCGT	1	<hkxhk>	hh	55	hk	163	kk	46
>ref-168358	GCCAGGACGACTCCACCTCCGCGGCGG	16	<nnxnp>	nn	263	np	0		
>ref-168377	CCGTTTTTAACCCCCCCTCCACTCCTT	3	<nnxnp>	nn	123	np	127		
>ref-168377	CCGTTTTTAACCCCCCCTCCACTCCTT	12	<nnxnp>	nn	122	np	127		
>ref-168387	CACACACAAACCTGAGCTCCCGTATCC	24	<lmxll>	ll	124	lm	145		
>ref-168412	CATTTTCTAACCATTTCTCCTTTACCC	9	<hkxhk>	hh	47	hk	126	kk	48
>ref-168417	CCATCGAGCACAGGGACTCCAGCAATG	8	<nnxnp>	nn	112	np	132		
>ref-168423	AAGCGTGTTACATAAGCTCCAGGTGTC	9	<nnxnp>	nn	272	np	1		
>ref-168437	CAAGGAGACACTTACCCTCCCTTTAAA	6	<nnxnp>	nn	120	np	142		
>ref-168462	ACCGCATAAACACTCACTCCATTTGGA	5	<lmxll>	ll	144	lm	111		
>ref-168473	CGTTTGGGTACCGAGGCTCCCATGTAA	5	<lmxll>	ll	117	lm	132		
>ref-168479	CTCCTACATACCCACCCTCCCCCATCT	23	<hkxhk>	hh	53	hk	127	kk	47
>ref-168490	CACACACAAACGCGTCCTCCTCCCCTC	22	<nnxnp>	nn	147	np	109		
>ref-168494	GCACCGCCTACCGCCCCTCCCCCTACC	12	<lmxll>	ll	135	lm	131		
>ref-168501	CCCGCACCCACAACACCTCCAGGTCAT	2	<lmxll>	ll	5	lm	268		
>ref-168501	CCCGCACCCACAACACCTCCAGGTCAT	13	<lmxll>	ll	0	lm	270		
>ref-168501	CCCGCACCCACAACACCTCCAGGTCAT	26	<lmxll>	ll	5	lm	268		
>ref-168549	AAATGATTTACGCATACTCCTTTCCCT	6	<nnxnp>	nn	132	np	130		
>ref-168566	CTCCCACTAACTCCCCCTCCATTCCCC	8	<nnxnp>	nn	109	np	113		
>ref-168576	ACGGACGCGACTTCGCCTCCTCCTGCA	27	<lmxll>	ll	137	lm	114		

>ref-168582	TAAGCGACCACAAACTCTCCCCTTCTT	14	<lmxll>	ll	126	lm	135		
>ref-168586	AGCATCCCCACCCCATCTCCTTCATCT	3	<lmxll>	ll	264	lm	3		
>ref-168601	TCTTGTTCCACGCGTGCTCCCTATGCA	14	<lmxll>	ll	134	lm	130		
>ref-168630	TCCTATCCTACTTTCTCTCCTTTCTCT	3	<hkxhk>	hh	2	hk	270	kk	0
>ref-168630	TCCTATCCTACTTTCTCTCCTTTCTCT	13	<hkxhk>	hh	0	hk	270	kk	2
>ref-168663	CTCCTACCCACTTTTCCTCCTCTTCTT	4	<nnxnp>	nn	133	np	130		
>ref-168667	ATTCCCCTTACACCCTCTCCCCTCACT	23	<lmxll>	ll	117	lm	132		
>ref-168670	TCCCTCCATACTCCCTCTCCCCCTCCA	25	<nnxnp>	nn	267	np	0		
>ref-168672	CCTCTTTTAACCCTGCCTCCATCTCCT	15	<hkxhk>	hh	54	hk	138	kk	56
>ref-168703	TATTCCACTACCGCTACTCCTCTGCCA	7	<hkxhk>	hh	62	hk	138	kk	60
>ref-168703	TATTCCACTACCGCTACTCCTCTGCCA	27	<lmxll>	ll	138	lm	128		
>ref-168706	GCCGATGCAACACTCGCTCCTACAGAC	4	<lmxll>	ll	238	lm	0		
>ref-168742	CTATCATCAACAGCATCTCCATCTTCA	4	<nnxnp>	nn	128	np	134		
>ref-168747	AACCTGTCTACCACCACTCCTACCAGT	6	<lmxll>	ll	8	lm	256		
>ref-168747	AACCTGTCTACCACCACTCCTACCAGT	26	<hkxhk>	hh	0	hk	273	kk	0
>ref-168752	GCGGATGCCACAGAGCCTCCGCCTGGG	9	<lmxll>	ll	150	lm	113		
>ref-168754	TGTACTGCCACACCTCCTCCTCCACCA	27	<lmxll>	ll	126	lm	142		
>ref-168755	CCCCCTCTCACCACCCCTCCCCTCCTT	3	<lmxll>	ll	75	lm	177		
>ref-168755	CCCCCTCTCACCACCCCTCCCCTCCTT	8	<lmxll>	ll	31	lm	226		
>ref-168755	CCCCCTCTCACCACCCCTCCCCTCCTT	13	<hkxhk>	hh	1	hk	272	kk	0
>ref-168755	CCCCCTCTCACCACCCCTCCCCTCCTT	25	<hkxhk>	hh	44	hk	204	kk	0
>ref-168761	GTGATCCAAACATCCTCTCCAGTTGCC	6	<lmxll>	ll	124	lm	130		
>ref-168787	CTCCACCTAACCCTGTCTCCTTCCCCC	16	<nnxnp>	nn	115	np	121		
>ref-168795	AACACTATCACCCGACCTCCATACATA	2	<hkxhk>	hh	58	hk	212	kk	0
>ref-168802	TGTCTTCCAACTCTTCCTCCTCCAGCT	6	<nnxnp>	nn	118	np	149		
>ref-168802	TGTCTTCCAACTCTTCCTCCTCCAGCT	7	<nnxnp>	nn	118	np	149		
>ref-168802	TGTCTTCCAACTCTTCCTCCTCCAGCT	25	<lmxll>	ll	133	lm	130		
>ref-168831	TTTCACTTTACCTTCTCTCCCCTTTCC	24	<lmxll>	ll	55	lm	193		
>ref-168837	TCCACTCGCACATCACCTCCTACAAAT	4	<hkxhk>	hh	27	hk	241	kk	0
>ref-168837	TCCACTCGCACATCACCTCCTACAAAT	8	<hkxhk>	hh	0	hk	234	kk	32
>ref-168837	TCCACTCGCACATCACCTCCTACAAAT	21	<hkxhk>	hh	0	hk	94	kk	170
>ref-168837	TCCACTCGCACATCACCTCCTACAAAT	22	<hkxhk>	hh	183	hk	81	kk	0
>ref-168837	TCCACTCGCACATCACCTCCTACAAAT	23	<hkxhk>	hh	141	hk	125	kk	0
>ref-168837	TCCACTCGCACATCACCTCCTACAAAT	24	<hkxhk>	hh	73	hk	188	kk	0
>ref-168837	TCCACTCGCACATCACCTCCTACAAAT	25	<nnxnp>	nn	220	np	47		
>ref-168859	GTTCGTAAGACTATTCCTCCATTTGCC	2	<nnxnp>	nn	130	np	129		
>ref-168876	AACACTAACACTAGCGCTCCCTTTGTG	3	<lmxll>	ll	163	lm	74		
>ref-168880	AGTTCACCTACCTTGGCTCCACCATCA	7	<nnxnp>	nn	59	np	197		

>ref-168880	AGTTCACCTACCTTGGCTCCACCATCA	22	<lmxll>	ll	25	lm	236		
>ref-168880	AGTTCACCTACCTTGGCTCCACCATCA	26	<hkxhk>	hh	8	hk	263	kk	0
>ref-168919	CCGTCAGCAACCCTTCCTCCAACCCCA	7	<lmxll>	ll	131	lm	126		
>ref-168923	TCTTCGTTAACTCTTTCTCCTGCTTCT	13	<hkxhk>	hh	40	hk	200	kk	0
>ref-168923	TCTTCGTTAACTCTTTCTCCTGCTTCT	23	<hkxhk>	hh	0	hk	200	kk	40
>ref-168944	CGATTGATAACTCTGACTCCTCGATTT	1	<nnxnp>	nn	114	np	127		
>ref-168968	CACCTCCCCACACCTCCTCCCTCCCCC	5	<nnxnp>	nn	71	np	182		
>ref-168968	CACCTCCCCACACCTCCTCCCTCCCCC	25	<nnxnp>	nn	76	np	176		
>ref-168980	ATCCCTTCCACCTTTTCTCCGTCTTTC	3	<hkxhk>	hh	34	hk	229	kk	0
>ref-168980	ATCCCTTCCACCTTTTCTCCGTCTTTC	4	<hkxhk>	hh	0	hk	273	kk	0
>ref-168988	ATCATAATTACTTACTCTCCATGATGT	3	<hkxhk>	hh	0	hk	272	kk	0
>ref-168988	ATCATAATTACTTACTCTCCATGATGT	26	<hkxhk>	hh	0	hk	133	kk	109
>ref-168995	TTACCACCTACAGCCCCTCCAAACTCA	25	<nnxnp>	nn	129	np	129		
>ref-169001	TTATGCCTCACCCTTTCTCCCTCGCAA	13	<nnxnp>	nn	265	np	0		
>ref-169002	TGCATTGCAACTTTCACTCCTTGGTTC	1	<lmxll>	ll	141	lm	123		
>ref-169012	CGCCCACACACTCTCTCTCCGCCCATC	27	<nnxnp>	nn	264	np	0		
>ref-169030	CTTTCGCTTACCTCTTCTCCATCCGTC	26	<lmxll>	ll	146	lm	111		
>ref-169034	AACAAAAATACATTCACTCCCAATGCA	23	<nnxnp>	nn	246	np	0		
>ref-169034	AACAAAAATACATTCACTCCCAATGCA	24	<nnxnp>	nn	247	np	0		
>ref-169051	TTAACATTAACATACTCTCCCCATGCA	25	<lmxll>	ll	122	lm	133		
>ref-169051	TTAACATTAACATACTCTCCCCATGCA	26	<lmxll>	ll	122	lm	133		
>ref-169061	TTTCCTCCTACTTTTTCTCCTTTCTCC	3	<nnxnp>	nn	5	np	230		
>ref-169061	TTTCCTCCTACTTTTTCTCCTTTCTCC	15	<nnxnp>	nn	5	np	230		
>ref-169069	TGTATATCTACGCACACTCCCTGAAAC	21	<nnxnp>	nn	1	np	265		
>ref-169072	AGATAAGGTACGTAGACTCCCCTCTTT	16	<hkxhk>	hh	56	hk	145	kk	46
>ref-169079	CTCAATGCAACTCTCTCTCCCTTGCTC	25	<nnxnp>	nn	137	np	129		
>ref-169109	TATAACCTAACCTTCTCTCCAATCTCA	27	<nnxnp>	nn	225	np	0		
>ref-169167	ACCGCCAGTACCACCGCTCCTCGAATG	3	<lmxll>	ll	138	lm	127		
>ref-169169	TCTTTTTTCACTTCTCCTCCTATCCCT	9	<nnxnp>	nn	26	np	217		
>ref-169169	TCTTTTTTCACTTCTCCTCCTATCCCT	12	<nnxnp>	nn	27	np	216		
>ref-169169	TCTTTTTTCACTTCTCCTCCTATCCCT	15	<hkxhk>	hh	6	hk	157	kk	81
>ref-169173	CTGCTCTCTACACCCTCTCCTTTATTC	21	<hkxhk>	hh	62	hk	142	kk	36
>ref-169181	ATCACGCCCACCTTGACTCCTCTCCCC	6	<lmxll>	ll	158	lm	109		
>ref-169194	TCCATCCCTACCCCCGCTCCTCCTTCT	22	<hkxhk>	hh	50	hk	143	kk	46
>ref-169200	ATGTAGGTGACTCTCTCTCCCCTTGCT	26	<lmxll>	ll	136	lm	135		
>ref-169216	CCTATTCGTACCTTCTCTCCTTTTCCT	4	<hkxhk>	hh	1	hk	271	kk	0
>ref-169216	CCTATTCGTACCTTCTCTCCTTTTCCT	15	<hkxhk>	hh	0	hk	271	kk	1
>ref-169216	CCTATTCGTACCTTCTCTCCTTTTCCT	25	<lmxll>	ll	165	lm	96		

>ref-169254	CGTTCGCGAACATTCTCTCCGTCATAT	22	<lmxll>	ll	129	lm	133		
>ref-169262	AGAACAAGGACTTATCCTCCTCCCTTT	7	<nnxnp>	nn	120	np	140		
>ref-169262	AGAACAAGGACTTATCCTCCTCCCTTT	13	<lmxll>	ll	117	lm	146		
>ref-169268	CTGGCTTTTACGACGCCTCCACCGTGC	12	<nnxnp>	nn	128	np	134		
>ref-169296	CCCAGTTTTACCTTATCTCCTCCTCCT	4	<hkxhk>	hh	10	hk	248	kk	0
>ref-169296	CCCAGTTTTACCTTATCTCCTCCTCCT	5	<hkxhk>	hh	0	hk	248	kk	10
>ref-169315	ACGTAGAAGACAAACACTCCTCCTCCC	3	<nnxnp>	nn	223	np	9		
>ref-169323	CTTCTAACCACGTTCTCTCCCTCTTCC	4	<hkxhk>	hh	65	hk	143	kk	48
>ref-169323	CTTCTAACCACGTTCTCTCCCTCTTCC	14	<hkxhk>	hh	47	hk	143	kk	65
>ref-169332	CCCCCATCCACCCACTCTCCAACACTA	7	<hkxhk>	hh	31	hk	127	kk	77
>ref-169332	CCCCCATCCACCCACTCTCCAACACTA	25	<hkxhk>	hh	77	hk	127	kk	31
>ref-169334	ACCGTCAAGACTTTCCCTCCGCCTCCA	27	<nnxnp>	nn	135	np	133		
>ref-169357	CATTTTTCCACCCCTCCTCCTACTCTC	7	<nnxnp>	nn	127	np	106		
>ref-169357	CATTTTTCCACCCCTCCTCCTACTCTC	13	<nnxnp>	nn	50	np	180		
>ref-169357	CATTTTTCCACCCCTCCTCCTACTCTC	22	<nnxnp>	nn	48	np	181		
>ref-169366	GATCACGTTACTCATCCTCCCTTAGAA	1	<nnxnp>	nn	129	np	125		
>ref-169366	GATCACGTTACTCATCCTCCCTTAGAA	22	<lmxll>	ll	123	lm	124		
>ref-169377	CCGTTCTCCACCTCCCCTCCTTCCTAA	13	<hkxhk>	hh	0	hk	226	kk	20
>ref-169377	CCGTTCTCCACCTCCCCTCCTTCCTAA	26	<hkxhk>	hh	20	hk	226	kk	0
>ref-169382	CCTGTTATCACGCTCTCTCCCGTTTCT	4	<nnxnp>	nn	225	np	34		
>ref-169401	TTTCTTCCTACCCTCCCTCCCTTTCCC	24	<nnxnp>	nn	144	np	104		
>ref-169401	TTTCTTCCTACCCTCCCTCCCTTTCCC	26	<nnxnp>	nn	248	np	4		
>ref-169407	CGGGATTCTACATTGTCTCCCTCTCTT	1	<nnxnp>	nn	106	np	139		
>ref-169466	GCTTTCATTACCTCTCCTCCATCTGCT	12	<nnxnp>	nn	124	np	121		
>ref-169466	GCTTTCATTACCTCTCCTCCATCTGCT	13	<nnxnp>	nn	126	np	119		
>ref-169479	CACACACGCACCATCTCTCCCCCTTAT	6	<nnxnp>	nn	135	np	131		
>ref-169485	TTTCTTGCTACCCAGCCTCCATTTCTT	21	<lmxll>	ll	255	lm	0		
>ref-169486	GGTTAGGGAACTGGGCCTCCTTTCCCC	13	<lmxll>	ll	133	lm	133		
>ref-169511	GAGTACGTAACAACCACTCCGGCAAAG	12	<lmxll>	ll	151	lm	110		
>ref-169554	CTCATTAACACCCTCCCTCCAACCCTT	9	<lmxll>	ll	97	lm	145		
>ref-169559	AGAGAGAAGACCGGCACTCCATGGTGG	2	<lmxll>	ll	136	lm	122		
>ref-169571	CCGCGCCGAACGCTGCCTCCTCTTATC	5	<hkxhk>	hh	0	hk	128	kk	134
>ref-169571	CCGCGCCGAACGCTGCCTCCTCTTATC	6	<lmxll>	ll	230	lm	34		
>ref-169586	TAGTGCCTTACCTCCCCTCCCTCCTCC	21	<nnxnp>	nn	115	np	127		
>ref-169588	CCCCCTACTACCACCGCTCCCTCCTCT	9	<hkxhk>	hh	73	hk	136	kk	56
>ref-169610	AAGGGCATCACGGACACTCCGCGGACG	9	<nnxnp>	nn	124	np	143		
>ref-169615	GTTGCTCCAACACCTGCTCCATCAACA	3	<lmxll>	ll	0	lm	271		
>ref-169645	ACCAGACTTACTTTCTCTCCATTCCCC	13	<nnxnp>	nn	155	np	117		

>ref-169645	ACCAGACTTACTTTCTCTCCATTCCCC	21	<hkxhk>	hh	0	hk	273	kk	0
>ref-169657	CCTCCTTCCACCGCCTCTCCACCGTTC	23	<nnxnp>	nn	124	np	130		
>ref-169657	CCTCCTTCCACCGCCTCTCCACCGTTC	24	<nnxnp>	nn	47	np	191		
>ref-169669	GCTAGTCTAACCCTGGCTCCAGGGATA	2	<nnxnp>	nn	128	np	133		
>ref-169699	GGCAGAGTGACTTCGGCTCCGATTTGT	26	<hkxhk>	hh	2	hk	266	kk	0
>ref-169702	TCCACCACAACCCCCACTCCCCCCTTC	2	<nnxnp>	nn	257	np	0		
>ref-169705	CTTGAGGAAACATCTCCTCCTTCAGTT	4	<lmxll>	ll	116	lm	121		
>ref-169725	TTCCCTTATACTTTCCCTCCTTTACTT	25	<lmxll>	ll	33	lm	218		
>ref-169725	TTCCCTTATACTTTCCCTCCTTTACTT	27	<lmxll>	ll	33	lm	219		
>ref-169735	CTGTGTGTGACCCGGGCTCCAGATGGT	21	<nnxnp>	nn	137	np	121		
>ref-169737	TCCCTGACAACAGTAGCTCCAAGATGC	2	<nnxnp>	nn	104	np	150		
>ref-169738	GAACAAACAACAGAAGCTCCTCCTTTG	14	<nnxnp>	nn	134	np	126		
>ref-169754	AATCTACGGACTCATCCTCCCTGCAAG	9	<nnxnp>	nn	238	np	1		
>ref-169790	ACTACTGTGACTGCACCTCCCCGCCCC	9	<lmxll>	ll	112	lm	147		
>ref-169815	TTTGCCTGTACAACCTCTCCTTCATCT	8	<nnxnp>	nn	126	np	127		
>ref-169815	TTTGCCTGTACAACCTCTCCTTCATCT	12	<nnxnp>	nn	128	np	128		
>ref-169819	CCACAATCCACCCAATCTCCTCCACCG	1	<nnxnp>	nn	115	np	147		
>ref-169831	TTCACTGCCACTCTCTCTCCCCATCAT	7	<nnxnp>	nn	129	np	121		
>ref-169855	ATCCACCCAACGGACTCTCCCCACCCA	6	<lmxll>	ll	197	lm	62		
>ref-169867	CCAAGGAGCACGAGATCTCCGTGCCCG	27	<hkxhk>	hh	0	hk	85	kk	181
>ref-169895	AAAAGTTCAACACCATCTCCACAGTGC	13	<lmxll>	ll	0	lm	271		
>ref-169895	AAAAGTTCAACACCATCTCCACAGTGC	27	<lmxll>	ll	0	lm	271		
>ref-169898	CGGTGAAGTACTCCCCCTCCGTGTTTC	15	<lmxll>	ll	117	lm	137		
>ref-169909	CCTTCCAACACCCCTTCTCCCTCCCTC	15	<nnxnp>	nn	175	np	88		
>ref-169909	CCTTCCAACACCCCTTCTCCCTCCCTC	24	<nnxnp>	nn	177	np	85		
>ref-169910	TCTCGCATGACTTCCCCTCCTAATGCT	22	<nnxnp>	nn	121	np	129		
>ref-169922	GTGCTGTCTACTGCCTCTCCCACTCTG	6	<lmxll>	ll	117	lm	145		
>ref-169969	ACCAACCTCACCCCTCCTCCTTTGCCC	9	<lmxll>	ll	138	lm	121		
>ref-169969	ACCAACCTCACCCCTCCTCCTTTGCCC	25	<nnxnp>	nn	115	np	145		
>ref-170022	AGGGCAGCCACTTCCACTCCCATCTAG	3	<nnxnp>	nn	265	np	0		
>ref-170022	AGGGCAGCCACTTCCACTCCCATCTAG	24	<lmxll>	ll	143	lm	112		
>ref-170040	CTTCCTCTCACCTCCCCTCCTTACCCC	12	<nnxnp>	nn	268	np	1		
>ref-170040	CTTCCTCTCACCTCCCCTCCTTACCCC	23	<nnxnp>	nn	69	np	183		
>ref-170052	ATCGACGTCACCTTTCCTCCTACGTCA	13	<hkxhk>	hh	69	hk	136	kk	57
>ref-170067	CCCCCTCCCACCCATCCTCCTTTCCCT	6	<lmxll>	ll	255	lm	0		
>ref-170080	AGTGCGCTCACTCAGTCTCCAGACACG	14	<lmxll>	ll	137	lm	126		
>ref-170156	TCCTAAACTACTCCTTCTCCTCCGTTT	3	<lmxll>	ll	120	lm	105		
>ref-170186	CCTCCACTCACCTCCCCTCCCTCCACC	9	<lmxll>	ll	194	lm	43		

>ref-170186	CCTCCACTCACCTCCCCTCCCTCCACC	13	<lmxll>	ll	179	lm	49		
>ref-170198	ACCAACCCAACCCCTCCTCCTCTCTCT	2	<nnxnp>	nn	267	np	0		
>ref-170211	CTTCTTTTCACTCTCTCTCCCTTTTCT	5	<hkxhk>	hh	0	hk	267	kk	4
>ref-170211	CTTCTTTTCACTCTCTCTCCCTTTTCT	7	<lmxll>	ll	98	lm	145		
>ref-170211	CTTCTTTTCACTCTCTCTCCCTTTTCT	8	<lmxll>	ll	97	lm	146		
>ref-170211	CTTCTTTTCACTCTCTCTCCCTTTTCT	14	<hkxhk>	hh	0	hk	266	kk	4
>ref-170211	CTTCTTTTCACTCTCTCTCCCTTTTCT	21	<hkxhk>	hh	6	hk	252	kk	0
>ref-170211	CTTCTTTTCACTCTCTCTCCCTTTTCT	26	<hkxhk>	hh	0	hk	251	kk	8
>ref-170229	CCGCATTCCACCTTTCCTCCTCCTCTG	12	<lmxll>	ll	133	lm	136		
>ref-170232	CAGCGGCGTACCGAGCCTCCAGCACTT	23	<hkxhk>	hh	64	hk	153	kk	50
>ref-170251	TTGAGGGTCACTTCTACTCCCAGACCT	3	<nnxnp>	nn	31	np	233		
>ref-170251	TTGAGGGTCACTTCTACTCCCAGACCT	7	<nnxnp>	nn	266	np	7		
>ref-170251	TTGAGGGTCACTTCTACTCCCAGACCT	25	<nnxnp>	nn	272	np	0		
>ref-170259	CTTCTCACTACCCACTCTCCTTCCCCC	21	<nnxnp>	nn	131	np	124		
>ref-170278	AATTAGCTTACGTAAACTCCATTTGCT	21	<nnxnp>	nn	223	np	0		
>ref-170300	CCGACCCTCACACCCTCTCCTCTCCGA	9	<hkxhk>	hh	62	hk	126	kk	48
>ref-170305	CCTTGTCCCACTCTCCCTCCTTACCCG	25	<lmxll>	ll	112	lm	125		
>ref-170320	TCTCTCCTCACTTTCTCTCCCCCACAT	7	<lmxll>	ll	265	lm	1		
>ref-170331	TTTCGTCTAACCAACCCTCCTTCCCCT	6	<hkxhk>	hh	49	hk	144	kk	61
>ref-170338	CAAGCACCTACTCAAACTCCACCAACT	4	<nnxnp>	nn	130	np	131		
>ref-170354	CCTCGATGAACCCTCTCTCCCTCTCTG	23	<nnxnp>	nn	241	np	1		
>ref-170363	AGCTTCCAAACCCCGACTCCGAGACAA	6	<hkxhk>	hh	68	hk	120	kk	69
>ref-170363	AGCTTCCAAACCCCGACTCCGAGACAA	16	<hkxhk>	hh	68	hk	121	kk	68
>ref-170373	GTCGCTCCCACCCTGCCTCCCTCTCCC	1	<nnxnp>	nn	258	np	0		
>ref-170373	GTCGCTCCCACCCTGCCTCCCTCTCCC	4	<nnxnp>	nn	248	np	3		
>ref-170373	GTCGCTCCCACCCTGCCTCCCTCTCCC	13	<nnxnp>	nn	263	np	0		
>ref-170373	GTCGCTCCCACCCTGCCTCCCTCTCCC	15	<nnxnp>	nn	252	np	3		
>ref-170378	AATGAGGTCACGATGTCTCCAAGAATA	13	<nnxnp>	nn	103	np	140		
>ref-170405	CGTAGACACACACAACCTCCCTCCCTC	6	<hkxhk>	hh	0	hk	273	kk	0
>ref-170405	CGTAGACACACACAACCTCCCTCCCTC	12	<hkxhk>	hh	0	hk	273	kk	0
>ref-170408	CTGGGTTACACGGCGTCTCCTTGTCAG	6	<nnxnp>	nn	44	np	205		
>ref-170417	AGTGAGCCAACACTGACTCCACGCCTT	7	<nnxnp>	nn	134	np	132		
>ref-170427	GGTGGTATGACGTCAGCTCCATTTTCC	25	<lmxll>	ll	120	lm	141		
>ref-170431	TGCATAGGAACACTTGCTCCTGGTTTC	24	<nnxnp>	nn	124	np	141		
>ref-170434	CGCTCACTCACTCACCCTCCGGACGCT	1	<nnxnp>	nn	270	np	0		
>ref-170460	TACCATCCCACCCCACCTCCCCTACAC	3	<nnxnp>	nn	130	np	115		
>ref-170460	TACCATCCCACCCCACCTCCCCTACAC	13	<lmxll>	ll	98	lm	137		
>ref-170474	CTGCGAAATACTGCTGCTCCACAACCT	5	<nnxnp>	nn	262	np	2		

>ref-170474	CTGCGAAATACTGCTGCTCCACAACCT	24	<nnxnp>	nn	262	np	2		
>ref-170475	CTGCCCATTACCCGCCCTCCCCTTCCC	5	<nnxnp>	nn	85	np	152		
>ref-170475	CTGCCCATTACCCGCCCTCCCCTTCCC	22	<lmxll>	ll	145	lm	123		
>ref-170499	CTCTCCTTTACCTTCCCTCCACCTATC	24	<hkxhk>	hh	54	hk	141	kk	53
>ref-170520	CTCCTTCACACAGTCTCTCCCTCCTTG	5	<lmxll>	ll	136	lm	127		
>ref-170525	CTAGCGTGTACAGTGTCTCCGCTGTTA	21	<nnxnp>	nn	143	np	129		
>ref-170533	CTTCTCATAACATCCTCTCCATGTAAA	1	<hkxhk>	hh	111	hk	141	kk	0
>ref-170533	CTTCTCATAACATCCTCTCCATGTAAA	26	<lmxll>	ll	138	lm	114		
>ref-170541	ATGCTCGACACTTTGTCTCCACACTAT	15	<nnxnp>	nn	144	np	127		
>ref-170565	ACACAACGCACTCTTACTCCTGCCTTC	4	<hkxhk>	hh	55	hk	149	kk	52
>ref-170577	ACAACAATAACAAAAGCTCCACCACCA	14	<hkxhk>	hh	0	hk	269	kk	2
>ref-170577	ACAACAATAACAAAAGCTCCACCACCA	22	<hkxhk>	hh	0	hk	270	kk	2
>ref-170580	GCTCCCTCCACCTTCTCTCCGCCTGCC	26	<nnxnp>	nn	137	np	134		
>ref-170598	GTAAGATGGACGTAGGCTCCCTTGGTG	26	<lmxll>	ll	140	lm	123		
>ref-170615	GTACTTTCTACTCCATCTCCCTCCTTC	24	<lmxll>	ll	121	lm	133		
>ref-170632	CCTCCTTTTACACTTCCTCCTTCTTTC	6	<hkxhk>	hh	51	hk	208	kk	0
>ref-170632	CCTCCTTTTACACTTCCTCCTTCTTTC	8	<hkxhk>	hh	52	hk	207	kk	0
>ref-170632	CCTCCTTTTACACTTCCTCCTTCTTTC	13	<lmxll>	ll	130	lm	127		
>ref-170652	TCAGACGAGACATACCCTCCCGTGGGG	23	<hkxhk>	hh	45	hk	143	kk	69
>ref-170691	ATGTTGGTGACGTTGCCTCCACTCACG	9	<nnxnp>	nn	259	np	1		
>ref-170700	CTAATCCTAACCGTCTCTCCATTCTGT	1	<nnxnp>	nn	264	np	0		
>ref-170785	GGACCCTTCACAGCCCCTCCGAAACCC	2	<nnxnp>	nn	73	np	171		
>ref-170785	GGACCCTTCACAGCCCCTCCGAAACCC	6	<lmxll>	ll	152	lm	104		
>ref-170796	CTTCTTTCTACTGTCTCTCCTCTTCTT	2	<lmxll>	ll	114	lm	113		
>ref-170807	GCTCTACACACTTCCCCTCCCTCCCTT	12	<nnxnp>	nn	124	np	130		
>ref-170823	CACATGTGAACCTTCCCTCCCTTCCCT	23	<lmxll>	ll	116	lm	124		
>ref-170855	CACAAGGCAACAGCCTCTCCAATACAT	8	<lmxll>	ll	147	lm	104		
>ref-170855	CACAAGGCAACAGCCTCTCCAATACAT	25	<lmxll>	ll	95	lm	149		
>ref-170879	TCGACCACTACATACCCTCCAGCCGCT	4	<hkxhk>	hh	0	hk	273	kk	0
>ref-170879	TCGACCACTACATACCCTCCAGCCGCT	27	<hkxhk>	hh	0	hk	273	kk	0
>ref-170918	GCACCTGCCACACCCGCTCCTTCGTGC	1	<nnxnp>	nn	271	np	0		
>ref-170918	GCACCTGCCACACCCGCTCCTTCGTGC	16	<lmxll>	ll	137	lm	129		
>ref-170931	CAGTGCACTACAATAACTCCTGAAACT	15	<lmxll>	ll	105	lm	119		
>ref-170935	TATCCTTCGACCCATCCTCCTCCCCCA	2	<lmxll>	ll	1	lm	269		
>ref-170935	TATCCTTCGACCCATCCTCCTCCCCCA	23	<lmxll>	ll	1	lm	269		
>ref-170972	AAACTCGGGACTGTCTCTCCTCCGTGT	22	<nnxnp>	nn	255	np	0		
>ref-170991	CATGCTTTCACCCCCACTCCCCTTCTC	9	<lmxll>	ll	108	lm	126		
>ref-171009	ATATGCTCGACATCGTCTCCGTAGGTA	16	<nnxnp>	nn	132	np	134		

>ref-171013	TCTATGTCAACCTTCCCTCCCTTTCTT	6	<lmxll>	ll	117	lm	143		
>ref-171026	TATATTATAACCCTCACTCCTGCGTAT	6	<hkxhk>	hh	69	hk	128	kk	41
>ref-171026	TATATTATAACCCTCACTCCTGCGTAT	7	<hkxhk>	hh	41	hk	128	kk	69
>ref-171032	ATCTCGAGGACTACCTCTCCTCCCCAT	26	<hkxhk>	hh	6	hk	250	kk	4
>ref-171033	GCCCCCTCTACCCTCTCTCCCTTTCCC	13	<lmxll>	ll	96	lm	139		
>ref-171051	TGACGTGGCACTCACCCTCCCATGCGG	13	<lmxll>	ll	129	lm	135		
>ref-171067	ACTCCATCAACTCCATCTCCTCCAGCA	9	<hkxhk>	hh	0	hk	273	kk	0
>ref-171070	CATCTGTGTACCAACTCTCCTTGCAAC	15	<hkxhk>	hh	71	hk	143	kk	52
>ref-171072	TCCCTCTCCACTCCTTCTCCATTTCCT	7	<hkxhk>	hh	0	hk	267	kk	4
>ref-171072	TCCCTCTCCACTCCTTCTCCATTTCCT	15	<hkxhk>	hh	0	hk	267	kk	4
>ref-171081	CTCACACTCACATTCCCTCCCTCCTCG	2	<lmxll>	ll	117	lm	142		
>ref-171107	CTCCACCGCACGTCAACTCCCCCACCC	23	<lmxll>	ll	134	lm	117		
>ref-171107	CTCCACCGCACGTCAACTCCCCCACCC	27	<nnxnp>	nn	158	np	94		
>ref-171121	GAAGACAGAACAGATTCTCCTGCAGAT	6	<nnxnp>	nn	114	np	137		
>ref-171146	TCCTATTCCACACTCCCTCCCCCCATT	27	<lmxll>	ll	127	lm	113		
>ref-171150	GGATGACCCACGAGAACTCCACAATTG	12	<nnxnp>	nn	254	np	0		
>ref-171155	CACCTACATACCCATTCTCCCACCTAC	1	<hkxhk>	hh	8	hk	255	kk	0
>ref-171155	CACCTACATACCCATTCTCCCACCTAC	3	<hkxhk>	hh	0	hk	126	kk	119
>ref-171155	CACCTACATACCCATTCTCCCACCTAC	5	<hkxhk>	hh	0	hk	126	kk	119
>ref-171155	CACCTACATACCCATTCTCCCACCTAC	7	<hkxhk>	hh	8	hk	255	kk	0
>ref-171162	TCCCTCGCCACACTCACTCCACCATCT	7	<lmxll>	ll	4	lm	269		
>ref-171162	TCCCTCGCCACACTCACTCCACCATCT	14	<lmxll>	ll	4	lm	269		
>ref-171162	TCCCTCGCCACACTCACTCCACCATCT	27	<nnxnp>	nn	152	np	120		
>ref-171190	TTCCTTGCCACCACCTCTCCTCATGCT	2	<nnxnp>	nn	150	np	117		
>ref-171196	GAATGGTTTACCCCCCCTCCTCCCCCC	9	<nnxnp>	nn	232	np	0		
>ref-171223	TCTCTCTCTACTTGTCCTCCTTCTTCT	2	<nnxnp>	nn	270	np	0		
>ref-171223	TCTCTCTCTACTTGTCCTCCTTCTTCT	12	<nnxnp>	nn	270	np	0		
>ref-171230	AGCACTACCACAACCACTCCTAGCAGC	16	<nnxnp>	nn	112	np	146		
>ref-171271	TAATGATCTACATTATCTCCTTTTCTT	26	<nnxnp>	nn	115	np	123		
>ref-171301	CTCTCCCTTACTCCTGCTCCCCACCGC	21	<hkxhk>	hh	2	hk	256	kk	6
>ref-171301	CTCTCCCTTACTCCTGCTCCCCACCGC	26	<hkxhk>	hh	2	hk	254	kk	6
>ref-171322	CTCAACCCAACTCTTTCTCCAACCCAA	23	<hkxhk>	hh	90	hk	165	kk	0
>ref-171326	CAGATCGAAACAGACACTCCACCGAAT	26	<lmxll>	ll	117	lm	117		
>ref-171337	TCCTCAAGCACACCCTCTCCACCAGGA	25	<lmxll>	ll	121	lm	142		
>ref-171347	ATCCTTCACACTCCCCCTCCCTTCCTC	5	<nnxnp>	nn	253	np	1		
>ref-171347	ATCCTTCACACTCCCCCTCCCTTCCTC	8	<nnxnp>	nn	232	np	4		
>ref-171351	ACCAAGTTAACCTTCTCTCCAATATAT	3	<lmxll>	ll	121	lm	113		
>ref-171361	ATCCCTTTCACCTTATCTCCCTCAGCA	13	<nnxnp>	nn	110	np	160		

>ref-171372	CCTCCTTATACTCGTCCTCCCTCCTCC	8	<lmxll>	ll	15	lm	243		
>ref-171389	GCCTGACGCACAAAGACTCCGTAGCCT	3	<lmxll>	ll	150	lm	116		
>ref-171390	CTGCCGCATACCTGCTCTCCCTCTCTC	9	<lmxll>	ll	267	lm	0		
>ref-171398	CCAACATCTACTTCATCTCCAATCCCA	7	<hkxhk>	hh	1	hk	205	kk	43
>ref-171398	CCAACATCTACTTCATCTCCAATCCCA	22	<hkxhk>	hh	43	hk	205	kk	1
>ref-171425	TAATTCAGCACTCAGTCTCCATTGTCC	8	<lmxll>	ll	122	lm	143		
>ref-171452	TTTTATCTCACAATCCCTCCTCCCTCC	13	<lmxll>	ll	104	lm	115		
>ref-171458	CACGCACGCACACGGCCTCCTACGCAC	8	<lmxll>	ll	142	lm	130		
>ref-171458	CACGCACGCACACGGCCTCCTACGCAC	16	<lmxll>	ll	132	lm	141		
>ref-171469	GCTTTCCTCACTCTCCCTCCCCCATTC	2	<nnxnp>	nn	29	np	196		
>ref-171469	GCTTTCCTCACTCTCCCTCCCCCATTC	24	<nnxnp>	nn	28	np	196		
>ref-171497	CCTTCCTTGACCCTGCCTCCCCTTCCA	4	<nnxnp>	nn	154	np	108		
>ref-171540	CTCTCCTAAACGTTCCCTCCCTCTCCC	23	<lmxll>	ll	149	lm	116		
>ref-171567	CGCTATAGAACTGCCTCTCCCACAGTT	15	<hkxhk>	hh	62	hk	143	kk	53
>ref-171569	ATAATATGCACATGTCCTCCATGCATT	27	<nnxnp>	nn	110	np	125		
>ref-171625	CTTCCCCCTACCTCCTCTCCCTCCTTG	1	<nnxnp>	nn	155	np	103		
>ref-171631	CCTCTCACCACCCCTTCTCCCCCCATC	6	<lmxll>	ll	249	lm	0		
>ref-171656	AGTCCCGCCACCCTCCCTCCTCCAAGA	22	<hkxhk>	hh	74	hk	120	kk	34
>ref-171656	AGTCCCGCCACCCTCCCTCCTCCAAGA	23	<hkxhk>	hh	34	hk	121	kk	73
>ref-171665	GTCCTCCCTACTTTCCCTCCTTTCCTT	1	<lmxll>	ll	106	lm	128		
>ref-171673	TACCCCCTGACCCTCTCTCCCCATCCC	4	<lmxll>	ll	75	lm	177		
>ref-171673	TACCCCCTGACCCTCTCTCCCCATCCC	16	<lmxll>	ll	75	lm	178		
>ref-171682	TATAGCTTGACCTCACCTCCGCTCTCC	9	<nnxnp>	nn	124	np	149		
>ref-171710	TTCTCCTCCACCCTTCCTCCTCAACTA	7	<hkxhk>	hh	1	hk	252	kk	10
>ref-171710	TTCTCCTCCACCCTTCCTCCTCAACTA	21	<hkxhk>	hh	1	hk	252	kk	10
>ref-171713	AGTTATTTAACGTTTCCTCCAACCCCG	7	<lmxll>	ll	123	lm	117		
>ref-171713	AGTTATTTAACGTTTCCTCCAACCCCG	8	<lmxll>	ll	123	lm	117		
>ref-171718	TCTCTTCTAACCCTCCCTCCCCCCTTA	5	<lmxll>	ll	235	lm	0		
>ref-171718	TCTCTTCTAACCCTCCCTCCCCCCTTA	22	<lmxll>	ll	232	lm	0		
>ref-171728	TTACCACTTACCTATCCTCCCCCGGAA	1	<nnxnp>	nn	134	np	132		
>ref-171729	ATATAACTGACTGTCACTCCCTACAGA	5	<nnxnp>	nn	105	np	125		
>ref-171747	AACCTCCTTACGCTTTCTCCGGTCCCT	2	<nnxnp>	nn	129	np	116		
>ref-171758	AATCTTTCTACCCGTTCTCCCTTTCTC	24	<lmxll>	ll	119	lm	131		
>ref-171772	CTCCTTCTTACCCTGTCTCCTTTTCTC	3	<lmxll>	ll	127	lm	111		
>ref-171786	TCAACCACCACCTACTCTCCCCCACCC	23	<lmxll>	ll	140	lm	88		
>ref-171791	CTCTTCCACACTCTCTCTCCGTTCCCC	1	<lmxll>	ll	266	lm	0		
>ref-171791	CTCTTCCACACTCTCTCTCCGTTCCCC	5	<lmxll>	ll	1	lm	263		
>ref-171791	CTCTTCCACACTCTCTCTCCGTTCCCC	21	<lmxll>	ll	1	lm	263		

>ref-171804	CTTGTTCTCACTTAACCTCCGTGTCTC	16	<hkxhk>	hh	55	hk	145	kk	66
>ref-171840	GCCTGTTCTACACCGTCTCCTACGGGA	8	<lmxll>	ll	121	lm	142		
>ref-171853	GGCAGCCGCACCTTTCCTCCTCCTCCT	23	<nnxnp>	nn	133	np	131		
>ref-171854	CCTCCAACAACTTGGCCTCCGCCTTGA	6	<hkxhk>	hh	4	hk	267	kk	0
>ref-171855	ACACACACGACCCAAGCTCCTCCTCCT	8	<lmxll>	ll	139	lm	131		
>ref-171855	ACACACACGACCCAAGCTCCTCCTCCT	25	<hkxhk>	hh	69	hk	139	kk	60
>ref-171874	CGCTCGAGCACAAGATCTCCATCACCC	26	<nnxnp>	nn	252	np	0		
>ref-171874	CGCTCGAGCACAAGATCTCCATCACCC	27	<nnxnp>	nn	250	np	0		
>ref-171923	TCTTCTTCTACTGCTTCTCCTTTATAC	13	<hkxhk>	hh	59	hk	187	kk	1
>ref-171923	TCTTCTTCTACTGCTTCTCCTTTATAC	25	<hkxhk>	hh	1	hk	187	kk	59
>ref-171952	CTCCCTCGCACTCACGCTCCTCCCTTC	24	<nnxnp>	nn	111	np	150		
>ref-171953	GAAGTCGTAACTAACCCTCCTTTATCT	22	<nnxnp>	nn	126	np	142		
>ref-171958	GGCTGGGCTACTTCAACTCCACCCTCA	3	<hkxhk>	hh	0	hk	273	kk	0
>ref-171958	GGCTGGGCTACTTCAACTCCACCCTCA	5	<hkxhk>	hh	0	hk	273	kk	0
>ref-171973	TTTGTTCTTACCGCACCTCCACCTATA	24	<hkxhk>	hh	45	hk	139	kk	58
>ref-172003	GTTTTCCCGACGGCGGCTCCAGCTCCC	2	<nnxnp>	nn	245	np	0		
>ref-172005	GCCTCCGCCACACGCCCTCCTCCTCCT	4	<hkxhk>	hh	0	hk	268	kk	4
>ref-172005	GCCTCCGCCACACGCCCTCCTCCTCCT	6	<hkxhk>	hh	4	hk	268	kk	0
>ref-172024	CAGATGCTAACCTCCTCTCCCAGGGTC	24	<nnxnp>	nn	272	np	0		
>ref-172024	CAGATGCTAACCTCCTCTCCCAGGGTC	27	<nnxnp>	nn	269	np	0		
>ref-172037	ATGCGTGTCACTCTACCTCCCTTTTCT	2	<nnxnp>	nn	138	np	113		
>ref-172037	ATGCGTGTCACTCTACCTCCCTTTTCT	24	<lmxll>	ll	98	lm	138		
>ref-172040	ACTAGTGTTACTCTTCCTCCCAGTGAT	22	<lmxll>	ll	0	lm	273		
>ref-172051	GGACCTACGACTCTCGCTCCCCCGACA	12	<nnxnp>	nn	144	np	123		
>ref-172054	GAATTAGGAACTCGAACTCCTTAATTA	1	<hkxhk>	hh	1	hk	272	kk	0
>ref-172085	CATTCCCCTACCGACCCTCCCCTCTCT	13	<lmxll>	ll	119	lm	138		
>ref-172086	TCTAAGAAAACCAGAGCTCCCTTTCCT	27	<hkxhk>	hh	59	hk	136	kk	45
>ref-172105	ACCTCCTTGACTTTCTCTCCGCCATCG	9	<nnxnp>	nn	113	np	145		
>ref-172125	CCATGCACTACATACTCTCCCAGTGTG	7	<hkxhk>	hh	0	hk	252	kk	3
>ref-172133	AGCCTCTTAACCCTCCCTCCGAGGATG	2	<hkxhk>	hh	0	hk	263	kk	7
>ref-172133	AGCCTCTTAACCCTCCCTCCGAGGATG	21	<hkxhk>	hh	0	hk	85	kk	180
>ref-172133	AGCCTCTTAACCCTCCCTCCGAGGATG	27	<hkxhk>	hh	0	hk	271	kk	2
>ref-172134	CACAAACATACCTTCACTCCACACGCG	23	<lmxll>	ll	129	lm	134		
>ref-172134	CACAAACATACCTTCACTCCACACGCG	27	<lmxll>	ll	126	lm	134		
>ref-172148	TTTAAACTCACTACTTCTCCGTTTTTA	3	<lmxll>	ll	116	lm	114		
>ref-172172	CCTGGCAGGACTGCATCTCCGGGGATA	9	<hkxhk>	hh	0	hk	151	kk	111
>ref-172190	CACACACAAACACACCCTCCTTTCCCT	16	<lmxll>	ll	9	lm	261		
>ref-172190	CACACACAAACACACCCTCCTTTCCCT	25	<hkxhk>	hh	4	hk	267	kk	1

>ref-172190	CACACACAAACACACCCTCCTTTCCCT	27	<lmxll>	ll	5	lm	268		
>ref-172199	ATGGGAACCACCCTCCCTCCTCCCTCC	25	<hkxhk>	hh	52	hk	145	kk	60
>ref-172205	TGCAGACACACCCTTCCTCCCTCCCCC	7	<hkxhk>	hh	0	hk	267	kk	5
>ref-172205	TGCAGACACACCCTTCCTCCCTCCCCC	26	<hkxhk>	hh	5	hk	267	kk	0
>ref-172207	TACTACTCTACTTCCACTCCTACTACT	8	<hkxhk>	hh	1	hk	169	kk	69
>ref-172207	TACTACTCTACTTCCACTCCTACTACT	15	<hkxhk>	hh	66	hk	168	kk	1
>ref-172233	TAACTTCTAACCCTTTCTCCTCATTCT	14	<hkxhk>	hh	88	hk	141	kk	37
>ref-172241	CACCTTCCTACCGTTCCTCCTCCTCAT	12	<lmxll>	ll	117	lm	146		
>ref-172241	CACCTTCCTACCGTTCCTCCTCCTCAT	16	<lmxll>	ll	122	lm	145		
>ref-172268	TCTCTCACGACCCTCTCTCCTCCCCTT	4	<hkxhk>	hh	4	hk	250	kk	1
>ref-172268	TCTCTCACGACCCTCTCTCCTCCCCTT	26	<hkxhk>	hh	1	hk	252	kk	4
>ref-172270	ATACCCAACACGAGTACTCCTTCATTG	27	<nnxnp>	nn	104	np	139		
>ref-172293	CTTTATGCTACTTCATCTCCTCCTTCA	13	<lmxll>	ll	121	lm	133		
>ref-172294	TTTTTGATCACGTCGTCTCCTTGCATG	6	<hkxhk>	hh	82	hk	140	kk	30
>ref-172297	CAATCTGCTACGCTATCTCCTGTTATG	24	<lmxll>	ll	119	lm	131		
>ref-172321	AAGACCCAAACATCTGCTCCAAGGGCT	14	<lmxll>	ll	103	lm	162		
>ref-172375	CCTTCCCCTACAACTCCTCCTTTTCTT	12	<nnxnp>	nn	263	np	0		
>ref-172378	GGTTTTACGACACGAACTCCCTCGCAA	1	<nnxnp>	nn	83	np	180		
>ref-172378	GGTTTTACGACACGAACTCCCTCGCAA	9	<nnxnp>	nn	83	np	180		
>ref-172378	GGTTTTACGACACGAACTCCCTCGCAA	25	<hkxhk>	hh	0	hk	232	kk	25
>ref-172378	GGTTTTACGACACGAACTCCCTCGCAA	27	<hkxhk>	hh	25	hk	230	kk	0
>ref-172379	ACCTCTTTCACTGCCCCTCCTACCTCT	13	<nnxnp>	nn	123	np	131		
>ref-172379	ACCTCTTTCACTGCCCCTCCTACCTCT	25	<nnxnp>	nn	122	np	131		
>ref-172424	AATGACCTGACCTGACCTCCAATCCCC	7	<nnxnp>	nn	147	np	116		
>ref-172428	TTCCCTATCACCGTATCTCCTAAGCTA	6	<lmxll>	ll	131	lm	128		
>ref-172428	TTCCCTATCACCGTATCTCCTAAGCTA	9	<nnxnp>	nn	112	np	140		
>ref-172428	TTCCCTATCACCGTATCTCCTAAGCTA	14	<nnxnp>	nn	112	np	140		
>ref-172434	CCCTCCTGCACTTCCCCTCCTCTTTCC	8	<hkxhk>	hh	10	hk	247	kk	0
>ref-172434	CCCTCCTGCACTTCCCCTCCTCTTTCC	15	<hkxhk>	hh	13	hk	243	kk	0
>ref-172446	GTGTCTAAGACGTTCCCTCCATCTTTC	4	<lmxll>	ll	125	lm	140		
>ref-172498	CCCCTCATAACATACCCTCCCCATGTG	2	<nnxnp>	nn	260	np	0		
>ref-172505	ACCCCTCCCACTCCCACTCCACTCGCC	12	<hkxhk>	hh	0	hk	225	kk	22
>ref-172505	ACCCCTCCCACTCCCACTCCACTCGCC	25	<hkxhk>	hh	0	hk	223	kk	22
>ref-172508	TCCACACGCACACTCTCTCCATCTCAT	27	<hkxhk>	hh	60	hk	140	kk	58
>ref-172511	AATTTATTCACTTTCTCTCCCTTTCTC	4	<lmxll>	ll	105	lm	118		
>ref-172515	CCGTAACTCACTCTTTCTCCCCTTGTC	13	<nnxnp>	nn	122	np	133		
>ref-172515	CCGTAACTCACTCTTTCTCCCCTTGTC	21	<nnxnp>	nn	266	np	0		
>ref-172518	GTCTCTGTTACTATGGCTCCTAATAAT	15	<lmxll>	ll	127	lm	92		

>ref-172528	AAGTGAGACACACCCTCTCCTCACACC	3	<hkxhk>	hh	0	hk	186	kk	77
>ref-172528	AAGTGAGACACACCCTCTCCTCACACC	14	<hkxhk>	hh	29	hk	232	kk	0
>ref-172528	AAGTGAGACACACCCTCTCCTCACACC	27	<lmxll>	ll	69	lm	194		
>ref-172572	GCCTCACCCACTTCCTCTCCCTCCTCT	1	<nnxnp>	nn	127	np	136		
>ref-172572	GCCTCACCCACTTCCTCTCCCTCCTCT	3	<lmxll>	ll	4	lm	253		
>ref-172572	GCCTCACCCACTTCCTCTCCCTCCTCT	24	<lmxll>	ll	4	lm	253		
>ref-172572	GCCTCACCCACTTCCTCTCCCTCCTCT	27	<nnxnp>	nn	133	np	133		
>ref-172589	CTTTCCCTTACTTCACCTCCCTCCCTA	6	<lmxll>	ll	254	lm	0		
>ref-172589	CTTTCCCTTACTTCACCTCCCTCCCTA	14	<lmxll>	ll	255	lm	0		
>ref-172591	GAGCATATTACCGGAGCTCCTCAAGTG	9	<lmxll>	ll	263	lm	0		
>ref-172591	GAGCATATTACCGGAGCTCCTCAAGTG	26	<lmxll>	ll	108	lm	133		
>ref-172597	CACTCGGTGACCAACGCTCCTTCGCCA	1	<lmxll>	ll	129	lm	140		
>ref-172622	TGTCTGGCAACGGCTACTCCTTTCTTT	14	<nnxnp>	nn	136	np	129		
>ref-172654	CTTCTGACTACCCATGCTCCCTTCCAT	4	<nnxnp>	nn	262	np	0		
>ref-172655	CTCTGCGCCACACACCCTCCCTTCCTC	15	<lmxll>	ll	131	lm	135		
>ref-172655	CTCTGCGCCACACACCCTCCCTTCCTC	23	<lmxll>	ll	130	lm	135		
>ref-172656	GCTAAAAGCACCAGGCCTCCTTTTCAA	1	<lmxll>	ll	116	lm	144		
>ref-172656	GCTAAAAGCACCAGGCCTCCTTTTCAA	7	<lmxll>	ll	116	lm	144		
>ref-172678	AAGGAAGTTACATGCTCTCCGTCCACT	27	<nnxnp>	nn	158	np	109		
>ref-172698	TAACCTGTGACGTGAACTCCTTACGTC	27	<nnxnp>	nn	254	np	5		
>ref-172700	ATCAATGGCACAATGCCTCCAGTGGAA	1	<lmxll>	ll	142	lm	122		
>ref-172700	ATCAATGGCACAATGCCTCCAGTGGAA	21	<nnxnp>	nn	126	np	141		
>ref-172704	CATCTTACTACAACCTCTCCCTCCGTC	15	<nnxnp>	nn	125	np	128		
>ref-172719	GCCCACCCTACGCCCACTCCAACACCC	25	<lmxll>	ll	106	lm	158		
>ref-172743	TTTCTTCTAACGCCTCCTCCCTCCCCC	12	<hkxhk>	hh	0	hk	242	kk	28
>ref-172743	TTTCTTCTAACGCCTCCTCCCTCCCCC	25	<hkxhk>	hh	132	hk	132	kk	0
>ref-172743	TTTCTTCTAACGCCTCCTCCCTCCCCC	27	<hkxhk>	hh	132	hk	132	kk	0
>ref-172767	TATCCACATACCGTTTCTCCCTCAATA	12	<nnxnp>	nn	120	np	135		
>ref-172770	CACAGTCACACAGACCCTCCCCCCCAT	7	<nnxnp>	nn	118	np	113		
>ref-172777	AGACCAGGAACTAAGCCTCCAATCCTC	8	<nnxnp>	nn	136	np	133		
>ref-172780	ATCCCATCTACCTCCCCTCCCCCCTAT	6	<nnxnp>	nn	5	np	256		
>ref-172780	ATCCCATCTACCTCCCCTCCCCCCTAT	26	<nnxnp>	nn	5	np	256		
>ref-172784	CGCGTTACCACCTCGTCTCCGAATATG	27	<nnxnp>	nn	114	np	109		
>ref-172799	CCCCCATACACCTCCCCTCCTCTCCTT	7	<nnxnp>	nn	115	np	120		
>ref-172799	CCCCCATACACCTCCCCTCCTCTCCTT	27	<nnxnp>	nn	243	np	0		
>ref-172808	ATTACACCCACCCACCCTCCTACACCC	9	<lmxll>	ll	89	lm	145		
>ref-172813	TTCCCCTCGACTCCCCCTCCCTACCTC	13	<nnxnp>	nn	141	np	127		
>ref-172821	TGTTATATTACGTGAGCTCCTTGTTAG	12	<lmxll>	ll	143	lm	121		

>ref-172824	TTTGTGATCACTTTTACTCCATCAGCA	15	<nnxnp>	nn	94	np	133		
>ref-172853	ATTCACCTTACATTCTCTCCACATTTG	14	<lmxll>	ll	117	lm	128		
>ref-172871	TGCCTTTCGACCTCGCCTCCTGCTCTA	3	<hkxhk>	hh	11	hk	214	kk	20
>ref-172871	TGCCTTTCGACCTCGCCTCCTGCTCTA	4	<hkxhk>	hh	11	hk	214	kk	20
>ref-172871	TGCCTTTCGACCTCGCCTCCTGCTCTA	23	<hkxhk>	hh	0	hk	147	kk	77
>ref-172871	TGCCTTTCGACCTCGCCTCCTGCTCTA	27	<hkxhk>	hh	86	hk	135	kk	0
>ref-172874	TTCCTTTTCACTCTATCTCCCCAATTC	5	<hkxhk>	hh	6	hk	231	kk	14
>ref-172874	TTCCTTTTCACTCTATCTCCCCAATTC	15	<hkxhk>	hh	14	hk	231	kk	6
>ref-172884	CTTCTTGGCACTCTGCCTCCTTACCCC	26	<nnxnp>	nn	235	np	2		
>ref-172884	CTTCTTGGCACTCTGCCTCCTTACCCC	27	<nnxnp>	nn	224	np	8		
>ref-172916	TCCCCCACTACCCACTCTCCCATACCA	27	<lmxll>	ll	126	lm	130		
>ref-172924	CTCTCGTTCACTGCTTCTCCGTTTTCC	25	<nnxnp>	nn	247	np	3		
>ref-172924	CTCTCGTTCACTGCTTCTCCGTTTTCC	27	<nnxnp>	nn	247	np	2		
>ref-172968	TGTCACAAGACACTCCCTCCAGTCGTC	1	<nnxnp>	nn	130	np	138		
>ref-172968	TGTCACAAGACACTCCCTCCAGTCGTC	25	<nnxnp>	nn	123	np	142		
>ref-172970	CATTCCCTAACCCCTTCTCCTCTTCCT	5	<hkxhk>	hh	0	hk	269	kk	0
>ref-172970	CATTCCCTAACCCCTTCTCCTCTTCCT	14	<hkxhk>	hh	0	hk	269	kk	0
>ref-172995	ATAATCAACACTTCATCTCCTGGCTTA	2	<lmxll>	ll	114	lm	141		
>ref-173008	TCGCTGTTCACTATCTCTCCTCCTTCC	7	<nnxnp>	nn	128	np	141		
>ref-173030	ACACACACGACAAGTCCTCCAACATCT	3	<hkxhk>	hh	49	hk	135	kk	81
>ref-173041	TTCCCTTCTACATCACCTCCCTTCCTT	6	<nnxnp>	nn	134	np	137		
>ref-173050	CCCTACACCACCCCTTCTCCCCCACAT	13	<hkxhk>	hh	25	hk	225	kk	0
>ref-173050	CCCTACACCACCCCTTCTCCCCCACAT	27	<hkxhk>	hh	0	hk	225	kk	25
>ref-173093	CATGAGAAAACTCTCTCTCCTCGTCTA	25	<lmxll>	ll	132	lm	117		
>ref-173096	ATATTCTTAACCGACCCTCCACACGCA	14	<lmxll>	ll	148	lm	125		
>ref-173123	CCCCCTTTTACATCCCCTCCTCTCCCC	12	<hkxhk>	hh	2	hk	257	kk	4
>ref-173129	AAAAAGAAAACCAACTCTCCTTTCTTG	6	<hkxhk>	hh	29	hk	211	kk	0
>ref-173129	AAAAAGAAAACCAACTCTCCTTTCTTG	13	<lmxll>	ll	11	lm	242		
>ref-173129	AAAAAGAAAACCAACTCTCCTTTCTTG	21	<lmxll>	ll	10	lm	241		
>ref-173141	TATACTTGTACAAGATCTCCAAATGGT	15	<hkxhk>	hh	1	hk	225	kk	11
>ref-173143	AAAAGGCAGACATGCCCTCCTCACACT	21	<hkxhk>	hh	0	hk	271	kk	2
>ref-173146	CCCTCATCCACCTCCCCTCCCTCTCGC	25	<nnxnp>	nn	35	np	211		
>ref-173146	CCCTCATCCACCTCCCCTCCCTCTCGC	26	<nnxnp>	nn	28	np	214		
>ref-173153	CATACAGACACACATTCTCCTCCAACA	7	<hkxhk>	hh	4	hk	267	kk	0
>ref-173153	CATACAGACACACATTCTCCTCCAACA	15	<nnxnp>	nn	152	np	111		
>ref-173153	CATACAGACACACATTCTCCTCCAACA	22	<hkxhk>	hh	87	hk	178	kk	0
>ref-173155	TTAACATCCACACGCTCTCCCTCTATT	15	<nnxnp>	nn	117	np	135		
>ref-173172	GTAGCTTGGACAATGTCTCCTTTTGAT	6	<hkxhk>	hh	53	hk	134	kk	68

>ref-173190	CTCCTGCCCACTTCCCCTCCCCATCCT	2	<nnxnp>	nn	263	np	0		
>ref-173208	CCCTCCTTAACCTTTTCTCCCTCTCTT	22	<lmxll>	ll	1	lm	268		
>ref-173209	CTCATACCTACCCCCTCTCCACCACCC	2	<nnxnp>	nn	135	np	96		
>ref-173209	CTCATACCTACCCCCTCTCCACCACCC	21	<nnxnp>	nn	131	np	96		
>ref-173214	TTCCCTCTTACTTCCCCTCCTCTCCCC	1	<lmxll>	ll	1	lm	272		
>ref-173214	TTCCCTCTTACTTCCCCTCCTCTCCCC	5	<lmxll>	ll	1	lm	272		
>ref-173214	TTCCCTCTTACTTCCCCTCCTCTCCCC	6	<lmxll>	ll	5	lm	265		
>ref-173214	TTCCCTCTTACTTCCCCTCCTCTCCCC	8	<lmxll>	ll	5	lm	266		
>ref-173225	AAGCTCATTACTATTCCTCCTAACCTC	5	<hkxhk>	hh	65	hk	130	kk	59
>ref-173235	CGCCTACCCACTCACTCTCCACCCACC	23	<hkxhk>	hh	63	hk	189	kk	13
>ref-173241	GTTTCAAGCACAAGCGCTCCTCTCCCC	2	<lmxll>	ll	255	lm	0		
>ref-173253	TTGCGTAAGACAGACTCTCCCCTTGTT	27	<lmxll>	ll	119	lm	129		
>ref-173255	CAGAACACCACGTGGGCTCCAGGTGTC	6	<lmxll>	ll	3	lm	269		
>ref-173255	CAGAACACCACGTGGGCTCCAGGTGTC	12	<lmxll>	ll	3	lm	269		
>ref-173258	GAGCCTTTAACACCAGCTCCATGTATA	23	<lmxll>	ll	150	lm	103		
>ref-173258	GAGCCTTTAACACCAGCTCCATGTATA	26	<lmxll>	ll	150	lm	104		
>ref-173262	GAAACTATCACTGTACCTCCGGGTAAG	16	<hkxhk>	hh	51	hk	208	kk	0
>ref-173262	GAAACTATCACTGTACCTCCGGGTAAG	21	<hkxhk>	hh	0	hk	183	kk	59
>ref-173262	GAAACTATCACTGTACCTCCGGGTAAG	23	<hkxhk>	hh	0	hk	250	kk	14
>ref-173283	TCCCTTCCTACCCCCTCTCCCCCATCA	12	<hkxhk>	hh	0	hk	264	kk	0
>ref-173283	TCCCTTCCTACCCCCTCTCCCCCATCA	21	<nnxnp>	nn	11	np	245		
>ref-173283	TCCCTTCCTACCCCCTCTCCCCCATCA	24	<nnxnp>	nn	49	np	192		
>ref-173283	TCCCTTCCTACCCCCTCTCCCCCATCA	27	<hkxhk>	hh	0	hk	264	kk	0
>ref-173287	CTCCCCTCCACCTGCCCTCCCTCCACC	3	<hkxhk>	hh	69	hk	154	kk	0
>ref-173296	CCCCCACCCACCGTCCCTCCTGCACTC	6	<lmxll>	ll	161	lm	78		
>ref-173296	CCCCCACCCACCGTCCCTCCTGCACTC	22	<lmxll>	ll	161	lm	78		
>ref-173314	ACAGACTGCACAGGGTCTCCTAGTAGA	3	<nnxnp>	nn	76	np	168		
>ref-173314	ACAGACTGCACAGGGTCTCCTAGTAGA	12	<nnxnp>	nn	6	np	262		
>ref-173323	TCCTCTAATACACTTCCTCCATTCTCT	7	<lmxll>	ll	127	lm	104		
>ref-173326	CCTGTCCTCACCTGTCCTCCTATCATC	3	<hkxhk>	hh	69	hk	146	kk	53
>ref-173326	CCTGTCCTCACCTGTCCTCCTATCATC	24	<hkxhk>	hh	55	hk	146	kk	67
>ref-173329	CTCTTTTCCACCTTCCCTCCCTACCTT	7	<lmxll>	ll	56	lm	203		
>ref-173329	CTCTTTTCCACCTTCCCTCCCTACCTT	15	<lmxll>	ll	62	lm	199		
>ref-173329	CTCTTTTCCACCTTCCCTCCCTACCTT	23	<lmxll>	ll	55	lm	181		
>ref-173366	CATCCCCCAACCCCACCTCCACGAGTC	21	<lmxll>	ll	125	lm	114		
>ref-173379	AGATCCTACACATTACCTCCTGAATAG	21	<lmxll>	ll	106	lm	137		
>ref-173446	ACCTGTAGCACTCTCTCTCCCTGTATC	1	<hkxhk>	hh	1	hk	272	kk	0
>ref-173446	ACCTGTAGCACTCTCTCTCCCTGTATC	21	<lmxll>	ll	126	lm	144		

>ref-173454	CCCAGCCACACTCACCCTCCCTCCCTC	5	<lmxll>	ll	260	lm	9		
>ref-173454	CCCAGCCACACTCACCCTCCCTCCCTC	14	<lmxll>	ll	257	lm	9		
>ref-173454	CCCAGCCACACTCACCCTCCCTCCCTC	23	<hkxhk>	hh	4	hk	259	kk	0
>ref-173548	TCTACCAACACACTACCTCCCCCACTA	24	<nnxnp>	nn	149	np	117		
>ref-173554	GACGGACGCACTCGAGCTCCTCCTTGT	1	<lmxll>	ll	98	lm	121		
>ref-173555	TCATTCATCACCAAGGCTCCGTAGCTC	4	<lmxll>	ll	202	lm	67		
>ref-173555	TCATTCATCACCAAGGCTCCGTAGCTC	7	<hkxhk>	hh	51	hk	216	kk	0
>ref-173577	GGGTATGAGACTGGCCCTCCGGTCTCA	3	<lmxll>	ll	182	lm	75		
>ref-173577	GGGTATGAGACTGGCCCTCCGGTCTCA	12	<lmxll>	ll	182	lm	74		
>ref-173581	TCCTCTTCCACGTTCACTCCTTACCTC	25	<nnxnp>	nn	272	np	0		
>ref-173581	TCCTCTTCCACGTTCACTCCTTACCTC	27	<nnxnp>	nn	268	np	0		
>ref-173605	TCCTCCTAAACCTCCTCTCCCTCGTTC	27	<lmxll>	ll	130	lm	127		
>ref-173626	AGGGCAGCTACCTCAACTCCCAACCTC	2	<lmxll>	ll	9	lm	263		
>ref-173626	AGGGCAGCTACCTCAACTCCCAACCTC	12	<hkxhk>	hh	68	hk	188	kk	0
>ref-173626	AGGGCAGCTACCTCAACTCCCAACCTC	25	<nnxnp>	nn	77	np	178		
>ref-173631	CCCGATGGAACAACTGCTCCAGGTAAT	22	<hkxhk>	hh	0	hk	263	kk	4
>ref-173632	TCTCGGGATACGCTTTCTCCGACTTTG	26	<lmxll>	ll	152	lm	113		
>ref-173694	GCATAAATCACACCATCTCCTCCCACC	15	<nnxnp>	nn	110	np	131		
>ref-173704	CAAGCGCACACGTAGCCTCCCGCCGTA	15	<lmxll>	ll	144	lm	126		
>ref-173704	CAAGCGCACACGTAGCCTCCCGCCGTA	27	<nnxnp>	nn	124	np	147		
>ref-173712	ATCCCTACAACCTTCTCTCCCTCTCCC	1	<nnxnp>	nn	94	np	144		
>ref-173717	CTACCCCCTACCTTTACTCCTACCCTT	1	<nnxnp>	nn	237	np	1		
>ref-173730	CTCATCACGACCAACACTCCTAAGGGA	7	<nnxnp>	nn	268	np	0		
>ref-173733	AGCATTCGCACGGCTCCTCCTCAACTC	25	<nnxnp>	nn	140	np	130		
>ref-173755	AAGTGCAGGACTTGAGCTCCCTCTCTG	1	<hkxhk>	hh	0	hk	258	kk	7
>ref-173755	AAGTGCAGGACTTGAGCTCCCTCTCTG	14	<lmxll>	ll	136	lm	126		
>ref-173755	AAGTGCAGGACTTGAGCTCCCTCTCTG	23	<hkxhk>	hh	0	hk	258	kk	7
>ref-173761	CTCTACCACACACTCCCTCCATTTCTC	1	<hkxhk>	hh	84	hk	137	kk	3
>ref-173771	ATCTGACAGACGCTTCCTCCTGTTGCT	12	<hkxhk>	hh	0	hk	271	kk	0
>ref-173771	ATCTGACAGACGCTTCCTCCTGTTGCT	14	<hkxhk>	hh	0	hk	271	kk	0
>ref-173771	ATCTGACAGACGCTTCCTCCTGTTGCT	15	<lmxll>	ll	130	lm	142		
>ref-173778	CCTCCCTATACCCCGTCTCCCCTTTTA	1	<lmxll>	ll	103	lm	128		
>ref-173781	TTAGTCGCCACTACCTCTCCGCGACGG	27	<lmxll>	ll	141	lm	121		
>ref-173785	TCTCCCCTCACTTGTCCTCCTTATCAT	2	<nnxnp>	nn	123	np	131		
>ref-173785	TCTCCCCTCACTTGTCCTCCTTATCAT	24	<nnxnp>	nn	123	np	133		
>ref-173795	CATTTTTCAACCAGTCCTCCTTCTCTC	1	<hkxhk>	hh	8	hk	254	kk	0
>ref-173795	CATTTTTCAACCAGTCCTCCTTCTCTC	3	<hkxhk>	hh	0	hk	254	kk	8
>ref-173822	TTCCTCATCACATCCTCTCCCTCCCTC	3	<hkxhk>	hh	99	hk	150	kk	7

>ref-173822	TTCCTCATCACATCCTCTCCCTCCCTC	5	<lmxll>	ll	129	lm	133		
>ref-173823	CCCACTCCCACCTTCCCTCCACATCCC	21	<lmxll>	ll	9	lm	256		
>ref-173856	GGCGCTGGAACAATCTCTCCCACAATC	4	<lmxll>	ll	11	lm	260		
>ref-173856	GGCGCTGGAACAATCTCTCCCACAATC	23	<hkxhk>	hh	152	hk	102	kk	0
>ref-173856	GGCGCTGGAACAATCTCTCCCACAATC	25	<hkxhk>	hh	86	hk	177	kk	0
>ref-173856	GGCGCTGGAACAATCTCTCCCACAATC	27	<hkxhk>	hh	113	hk	124	kk	0
>ref-173882	ATACGGCTTACGTTTACTCCATGAATG	22	<nnxnp>	nn	137	np	109		
>ref-173913	GTCTGTCCTACGCTCTCTCCTGCCATA	12	<hkxhk>	hh	0	hk	257	kk	12
>ref-173913	GTCTGTCCTACGCTCTCTCCTGCCATA	23	<hkxhk>	hh	13	hk	256	kk	0
>ref-173913	GTCTGTCCTACGCTCTCTCCTGCCATA	25	<lmxll>	ll	149	lm	119		
>ref-173913	GTCTGTCCTACGCTCTCTCCTGCCATA	26	<lmxll>	ll	149	lm	119		
>ref-173931	TCATTAATCACCTTCTCTCCTTCTCTT	25	<lmxll>	ll	132	lm	116		
>ref-173947	TTGGGTGCTACTCGTCCTCCTCTCAGG	6	<lmxll>	ll	102	lm	158		
>ref-173947	TTGGGTGCTACTCGTCCTCCTCTCAGG	8	<lmxll>	ll	102	lm	158		
>ref-173948	TCTCCCTAAACCCCGTCTCCCCTTCTT	12	<lmxll>	ll	108	lm	150		
>ref-173955	TGCTCACTCACTCGTTCTCCTCTCACT	13	<hkxhk>	hh	53	hk	158	kk	51
>ref-173955	TGCTCACTCACTCGTTCTCCTCTCACT	21	<hkxhk>	hh	51	hk	158	kk	53
>ref-173963	ATCGTCCACACTTCCTCTCCAACGGCA	4	<lmxll>	ll	135	lm	127		
>ref-173964	TGTTCCTTCACTATTCCTCCTTCCTCC	2	<lmxll>	ll	4	lm	254		
>ref-173964	TGTTCCTTCACTATTCCTCCTTCCTCC	12	<lmxll>	ll	5	lm	253		
>ref-173970	TTTCTCCGAACTGAATCTCCCCTCTAT	25	<lmxll>	ll	29	lm	196		
>ref-173970	TTTCTCCGAACTGAATCTCCCCTCTAT	26	<lmxll>	ll	86	lm	134		
>ref-173973	CATACTCCCACCTCTTCTCCACTCGCT	1	<lmxll>	ll	117	lm	130		
>ref-173978	TCACGTAACACCTCCCCTCCCCTCGCC	21	<nnxnp>	nn	127	np	138		
>ref-174016	TAGCATTTAACATTCCCTCCTCCTCCT	2	<lmxll>	ll	1	lm	268		
>ref-174016	TAGCATTTAACATTCCCTCCTCCTCCT	21	<lmxll>	ll	1	lm	268		
>ref-174031	TCTCTATCTACCTATGCTCCTCTTTTC	2	<hkxhk>	hh	0	hk	270	kk	0
>ref-174031	TCTCTATCTACCTATGCTCCTCTTTTC	16	<hkxhk>	hh	0	hk	270	kk	0
>ref-174036	ATGGTCGTCACACGCTCTCCATCATCT	14	<nnxnp>	nn	136	np	137		
>ref-174053	TGCTTAACTACCTCATCTCCGTAGACT	14	<lmxll>	ll	133	lm	130		
>ref-174068	ATCTCCATCACCTCATCTCCATCATCT	3	<hkxhk>	hh	26	hk	244	kk	0
>ref-174068	ATCTCCATCACCTCATCTCCATCATCT	14	<hkxhk>	hh	43	hk	221	kk	0
>ref-174068	ATCTCCATCACCTCATCTCCATCATCT	26	<nnxnp>	nn	202	np	62		
>ref-174070	CACACACGTACACAACCTCCCCCCTTA	5	<nnxnp>	nn	125	np	144		
>ref-174076	TCCCTCTCCACCATTCCTCCTTAACCG	21	<lmxll>	ll	134	lm	115		
>ref-174085	ACCATTCTCACTATCCCTCCTTCCTTA	12	<nnxnp>	nn	110	np	127		
>ref-174095	CCTCCATTAACCTCCTCTCCCTCTCCC	12	<lmxll>	ll	11	lm	255		
>ref-174101	TTCCCTCTAACCCCCCCTCCACCTCCC	6	<lmxll>	ll	97	lm	127		

>ref-174101	TTCCCTCTAACCCCCCCTCCACCTCCC	9	<lmxll>	ll	114	lm	115		
>ref-174118	ACCCTTCTCACCCTCACTCCCTCTCCC	2	<lmxll>	ll	245	lm	4		
>ref-174118	ACCCTTCTCACCCTCACTCCCTCTCCC	5	<nnxnp>	nn	250	np	0		
>ref-174118	ACCCTTCTCACCCTCACTCCCTCTCCC	8	<nnxnp>	nn	263	np	0		
>ref-174118	ACCCTTCTCACCCTCACTCCCTCTCCC	12	<lmxll>	ll	254	lm	4		
>ref-174118	ACCCTTCTCACCCTCACTCCCTCTCCC	13	<nnxnp>	nn	109	np	141		
>ref-174118	ACCCTTCTCACCCTCACTCCCTCTCCC	15	<nnxnp>	nn	262	np	0		
>ref-174122	TGAGATGCTACTCGCTCTCCTTTCTGT	9	<nnxnp>	nn	141	np	128		
>ref-174128	CCTCCACACACATACCCTCCTCCGCTT	22	<lmxll>	ll	131	lm	137		
>ref-174142	TAACTCCGCACCGTAACTCCGCACCGT	8	<hkxhk>	hh	1	hk	213	kk	56
>ref-174142	TAACTCCGCACCGTAACTCCGCACCGT	21	<nnxnp>	nn	134	np	131		
>ref-174142	TAACTCCGCACCGTAACTCCGCACCGT	23	<nnxnp>	nn	139	np	122		
>ref-174142	TAACTCCGCACCGTAACTCCGCACCGT	24	<nnxnp>	nn	140	np	122		
>ref-174146	CTCAGATAGACAGATCCTCCCACTCAC	7	<lmxll>	ll	140	lm	126		
>ref-174161	ATGCAAAATACAGCAGCTCCTCCTTGT	13	<nnxnp>	nn	168	np	92		
>ref-174164	CCCTTTCATACTATTCCTCCTTTTCCT	8	<nnxnp>	nn	248	np	0		
>ref-174164	CCCTTTCATACTATTCCTCCTTTTCCT	15	<nnxnp>	nn	252	np	0		
>ref-174169	GTGGTCGTGACAGTGGCTCCGGTGGTC	24	<lmxll>	ll	5	lm	266		
>ref-174181	TAGCTTCCTACCCTTCCTCCTTAATAA	27	<lmxll>	ll	134	lm	117		
>ref-174195	GCCTGCTACACGCTTGCTCCTATTTAC	14	<hkxhk>	hh	0	hk	271	kk	0
>ref-174209	TTGGTCCTCACTCTGTCTCCTCTCCTC	15	<nnxnp>	nn	157	np	107		
>ref-174214	GTTTGTAACACGACTTCTCCTCGCAGC	9	<hkxhk>	hh	49	hk	203	kk	0
>ref-174214	GTTTGTAACACGACTTCTCCTCGCAGC	13	<hkxhk>	hh	1	hk	271	kk	0
>ref-174228	GCTCTAGTTACCGATACTCCTTGACTG	13	<hkxhk>	hh	48	hk	145	kk	71
>ref-174259	TCTCCCCCAACCCACACTCCTTCTTTA	24	<nnxnp>	nn	103	np	123		
>ref-174260	ATTTTTCTAACTCGGTCTCCGTTCGTA	27	<nnxnp>	nn	112	np	138		
>ref-174267	CTCTTCCCTACTTTGACTCCTCACCCC	24	<nnxnp>	nn	112	np	129		
>ref-174269	AAACATCAAACCACATCTCCCAGTGTG	12	<nnxnp>	nn	142	np	112		
>ref-174274	TCCCCTATCACCCTTCCTCCTTCGCCT	9	<nnxnp>	nn	128	np	134		
>ref-174285	ATCACCATCACCCTTACTCCTCCTGAA	1	<hkxhk>	hh	60	hk	123	kk	70
>ref-174294	CCTCCTGTTACTTCACCTCCTTCCTCT	8	<lmxll>	ll	2	lm	261		
>ref-174294	CCTCCTGTTACTTCACCTCCTTCCTCT	27	<lmxll>	ll	5	lm	258		
>ref-174321	AATTCATGTACCCCCGCTCCCTCCACA	6	<nnxnp>	nn	119	np	129		
>ref-174338	CACTCATCCACCCATCCTCCTCCACAT	4	<hkxhk>	hh	0	hk	258	kk	9
>ref-174338	CACTCATCCACCCATCCTCCTCCACAT	24	<hkxhk>	hh	9	hk	259	kk	0
>ref-174362	TAGGTAATTACTCAACCTCCAGGACAC	12	<nnxnp>	nn	266	np	0		
>ref-174378	GTCACTCACACACGCACTCCCTCATTC	25	<lmxll>	ll	122	lm	133		
>ref-174390	TCCCTCATTACGTTCCCTCCCTCTTAC	1	<hkxhk>	hh	0	hk	264	kk	3

>ref-174390	TCCCTCATTACGTTCCCTCCCTCTTAC	27	<nnxnp>	nn	271	np	0		
>ref-174392	CTACTCAACACAAGTCCTCCGTCTGCT	6	<hkxhk>	hh	0	hk	273	kk	0
>ref-174404	GCGTCAGTAACTGCTGCTCCGGACTCG	22	<nnxnp>	nn	271	np	0		
>ref-174412	TCGTTATCCACCTACCCTCCCTTCCTT	14	<lmxll>	ll	123	lm	128		
>ref-174416	TTCCCATCTACGCTTCCTCCATTTTCC	23	<lmxll>	ll	220	lm	0		
>ref-174432	CGCCAGCACACCCCACCTCCACCACCT	5	<nnxnp>	nn	247	np	0		
>ref-174433	CACACAGACACGCACTCTCCCTTAGCT	24	<hkxhk>	hh	60	hk	153	kk	54
>ref-174433	CACACAGACACGCACTCTCCCTTAGCT	25	<lmxll>	ll	140	lm	130		
>ref-174435	CCTCGCTTCACGCTCCCTCCCTCACTC	6	<hkxhk>	hh	61	hk	148	kk	53
>ref-174463	TTCCATCCCACGCCTTCTCCTGTCCTC	24	<hkxhk>	hh	54	hk	149	kk	56
>ref-174464	GCTCCCCCTACTTCCTCTCCTGGCCTT	4	<nnxnp>	nn	121	np	136		
>ref-174464	GCTCCCCCTACTTCCTCTCCTGGCCTT	5	<nnxnp>	nn	126	np	134		
>ref-174487	GATAATCCCACTCTCTCTCCGTGCGCA	6	<lmxll>	ll	131	lm	131		
>ref-174497	TTCATTTCCACCTCCCCTCCTCGTCTT	7	<lmxll>	ll	120	lm	129		
>ref-174497	TTCATTTCCACCTCCCCTCCTCGTCTT	25	<lmxll>	ll	120	lm	129		
>ref-174501	CTTCCTTCTACCGCAACTCCTACTCCC	5	<lmxll>	ll	130	lm	114		
>ref-174501	CTTCCTTCTACCGCAACTCCTACTCCC	24	<lmxll>	ll	130	lm	114		
>ref-174509	ATCTTTATTACTATCTCTCCCTTCCTC	5	<lmxll>	ll	0	lm	263		
>ref-174509	ATCTTTATTACTATCTCTCCCTTCCTC	7	<lmxll>	ll	0	lm	263		
>ref-174528	ACCATCCTCACCATCACTCCTACCAAG	7	<lmxll>	ll	130	lm	125		
>ref-174538	CTCATCTCCACCTCTCCTCCACCCCCT	2	<lmxll>	ll	229	lm	4		
>ref-174538	CTCATCTCCACCTCTCCTCCACCCCCT	3	<lmxll>	ll	223	lm	4		
>ref-174545	CTTCTTTCTACCCTTTCTCCTTGTTCC	9	<lmxll>	ll	126	lm	108		
>ref-174545	CTTCTTTCTACCCTTTCTCCTTGTTCC	23	<lmxll>	ll	124	lm	109		
>ref-174599	ACCTCTCTAACCCCCCCTCCTTCCCTC	2	<nnxnp>	nn	256	np	1		
>ref-174599	ACCTCTCTAACCCCCCCTCCTTCCCTC	22	<lmxll>	ll	4	lm	247		
>ref-174599	ACCTCTCTAACCCCCCCTCCTTCCCTC	27	<lmxll>	ll	4	lm	247		
>ref-174609	CGACTCTGTACAGTGTCTCCTTCAGGT	25	<lmxll>	ll	130	lm	130		
>ref-174657	AACCCCCACACCCTTCCTCCACCCTCA	1	<hkxhk>	hh	4	hk	267	kk	0
>ref-174657	AACCCCCACACCCTTCCTCCACCCTCA	3	<nnxnp>	nn	273	np	0		
>ref-174657	AACCCCCACACCCTTCCTCCACCCTCA	6	<hkxhk>	hh	4	hk	268	kk	0
>ref-174657	AACCCCCACACCCTTCCTCCACCCTCA	7	<hkxhk>	hh	34	hk	229	kk	0
>ref-174694	CTTTCTAATACCATCTCTCCCTCTCTC	1	<nnxnp>	nn	196	np	60		
>ref-174694	CTTTCTAATACCATCTCTCCCTCTCTC	25	<hkxhk>	hh	0	hk	201	kk	69
>ref-174694	CTTTCTAATACCATCTCTCCCTCTCTC	27	<hkxhk>	hh	100	hk	162	kk	0
>ref-174720	TTCTACTCCACCCCCCCTCCCCCCTCC	5	<nnxnp>	nn	21	np	205		
>ref-174720	TTCTACTCCACCCCCCCTCCCCCCTCC	16	<nnxnp>	nn	248	np	0		
>ref-174720	TTCTACTCCACCCCCCCTCCCCCCTCC	22	<nnxnp>	nn	254	np	0		

>ref-174726	ACTCTCTCTACCACTTCTCCTCTCCTC	21	<lmxll>	ll	99	lm	157		
>ref-174726	ACTCTCTCTACCACTTCTCCTCTCCTC	26	<lmxll>	ll	100	lm	159		
>ref-174727	GTCCATCACACCCTACCTCCCTCACCA	26	<nnxnp>	nn	143	np	126		
>ref-174774	TCTTTTCTGACTGTGCCTCCGCCCTTT	15	<nnxnp>	nn	132	np	101		
>ref-174781	ACACATGTAACCGGGTCTCCCTCGAAA	5	<hkxhk>	hh	45	hk	149	kk	68
>ref-174788	TCCTTCCGCACCTCTTCTCCCGCTCCT	7	<lmxll>	ll	116	lm	142		
>ref-174805	TGTAAGCGTACTCTGGCTCCTTATATT	27	<nnxnp>	nn	102	np	133		
>ref-174806	CATTCATTGACTCTCTCTCCTCCCCCC	2	<lmxll>	ll	14	lm	250		
>ref-174806	CATTCATTGACTCTCTCTCCTCCCCCC	4	<lmxll>	ll	14	lm	250		
>ref-174832	CACATACAAACCTGCCCTCCGTTTTTC	6	<nnxnp>	nn	131	np	130		
>ref-174835	TTGAGCTGAACATCGGCTCCATATCCA	15	<nnxnp>	nn	121	np	140		
>ref-174851	TTCTATTGCACGCCTCCTCCTTGCCTC	14	<nnxnp>	nn	135	np	135		
>ref-174868	CTTCCCCTCACTTCATCTCCCTTTATC	5	<lmxll>	ll	264	lm	1		
>ref-174868	CTTCCCCTCACTTCATCTCCCTTTATC	13	<lmxll>	ll	266	lm	0		
>ref-174868	CTTCCCCTCACTTCATCTCCCTTTATC	24	<nnxnp>	nn	96	np	157		
>ref-174873	TCCTTCTTCACAGTCCCTCCTCCTCCG	27	<nnxnp>	nn	114	np	139		
>ref-174885	TCCTCTCCCACCCTCCCTCCTCTTTAG	7	<nnxnp>	nn	117	np	130		
>ref-174889	GCCCATGATACGCACCCTCCCTCACAC	21	<lmxll>	ll	118	lm	147		
>ref-174899	TCTTCGCTTACTCTCTCTCCTTTGCCT	15	<nnxnp>	nn	122	np	142		
>ref-174899	TCTTCGCTTACTCTCTCTCCTTTGCCT	25	<lmxll>	ll	127	lm	140		
>ref-174909	GGATACTGGACATACACTCCAACACAC	12	<lmxll>	ll	107	lm	134		
>ref-174917	ACTTCTGTTACTGTTACTCCTATGTCT	22	<lmxll>	ll	104	lm	154		
>ref-174941	GACCCTTCAACGCTCGCTCCACTTTCT	5	<hkxhk>	hh	0	hk	272	kk	0
>ref-174941	GACCCTTCAACGCTCGCTCCACTTTCT	14	<hkxhk>	hh	0	hk	272	kk	0
>ref-174942	CCCTTACTCACCCCCTCTCCTCTCCCA	16	<lmxll>	ll	16	lm	228		
>ref-174942	CCCTTACTCACCCCCTCTCCTCTCCCA	27	<lmxll>	ll	16	lm	225		
>ref-174944	CCATCTCCAACCTTTACTCCACCATCT	1	<hkxhk>	hh	0	hk	273	kk	0
>ref-174944	CCATCTCCAACCTTTACTCCACCATCT	8	<lmxll>	ll	139	lm	131		
>ref-174944	CCATCTCCAACCTTTACTCCACCATCT	14	<hkxhk>	hh	0	hk	273	kk	0
>ref-174944	CCATCTCCAACCTTTACTCCACCATCT	16	<lmxll>	ll	146	lm	127		
>ref-174944	CCATCTCCAACCTTTACTCCACCATCT	24	<nnxnp>	nn	185	np	77		
>ref-174946	ATCGTTATCACCTCCTCTCCCTCCTCT	1	<nnxnp>	nn	68	np	189		
>ref-174946	ATCGTTATCACCTCCTCTCCCTCCTCT	4	<nnxnp>	nn	69	np	189		
>ref-174946	ATCGTTATCACCTCCTCTCCCTCCTCT	6	<hkxhk>	hh	0	hk	268	kk	4
>ref-174946	ATCGTTATCACCTCCTCTCCCTCCTCT	7	<hkxhk>	hh	4	hk	268	kk	0
>ref-174950	TGAATATCAACATTTTCTCCTCCTTTC	25	<nnxnp>	nn	134	np	95		
>ref-174958	CTGCCATGCACTCTGCCTCCTTCCCTG	8	<hkxhk>	hh	0	hk	273	kk	0
>ref-174958	CTGCCATGCACTCTGCCTCCTTCCCTG	12	<hkxhk>	hh	0	hk	273	kk	0

>ref-174971	CCTCTCGCCACCTGTCCTCCCCTGAAT	9	<nnxnp>	nn	137	np	112		
>ref-174990	GGGGTGACGACCTCGACTCCCTCTCGG	24	<lmxll>	ll	126	lm	124		
>ref-174994	ATTACCTCTACCCTCCCTCCCCTTTCC	5	<lmxll>	ll	129	lm	134		
>ref-175001	TCACTCGCTACCCTTTCTCCTCTTTCT	8	<lmxll>	ll	141	lm	123		
>ref-175008	TTACAATAGACTTTCTCTCCTCCTTAT	14	<hkxhk>	hh	61	hk	166	kk	0
>ref-175018	TTCAGTGGAACTCTTCCTCCTTCCACT	3	<lmxll>	ll	132	lm	125		
>ref-175018	TTCAGTGGAACTCTTCCTCCTTCCACT	25	<lmxll>	ll	264	lm	0		
>ref-175034	TATGCCCACACCGCCCCTCCCTACACC	24	<lmxll>	ll	143	lm	104		
>ref-175034	TATGCCCACACCGCCCCTCCCTACACC	25	<lmxll>	ll	142	lm	104		
>ref-175063	TTTCCACCCACCCCTTCTCCCTCTCCC	1	<hkxhk>	hh	0	hk	273	kk	0
>ref-175063	TTTCCACCCACCCCTTCTCCCTCTCCC	3	<hkxhk>	hh	0	hk	273	kk	0
>ref-175063	TTTCCACCCACCCCTTCTCCCTCTCCC	9	<hkxhk>	hh	140	hk	113	kk	0
>ref-175063	TTTCCACCCACCCCTTCTCCCTCTCCC	13	<lmxll>	ll	30	lm	230		
>ref-175063	TTTCCACCCACCCCTTCTCCCTCTCCC	15	<hkxhk>	hh	0	hk	119	kk	136
>ref-175076	CACACACGCACTTACACTCCTCCCCCA	1	<lmxll>	ll	128	lm	138		
>ref-175111	CTCCTCCCTACCCTCCCTCCAGCCTAT	24	<lmxll>	ll	122	lm	142		
>ref-175121	CGAGAACAGACCCATGCTCCCCAAACA	7	<lmxll>	ll	119	lm	105		
>ref-175130	TCAAATCCCACACCCACTCCACCCCAC	15	<lmxll>	ll	112	lm	120		
>ref-175139	GACACAATCACTCAAACTCCATACGCT	12	<hkxhk>	hh	3	hk	131	kk	120
>ref-175156	CCTCGATGCACTTATTCTCCCCGCATT	27	<nnxnp>	nn	267	np	0		
>ref-175198	GATAAACTAACCGTGGCTCCGGGTGAT	4	<nnxnp>	nn	135	np	129		
>ref-175201	TGAACACACACAAGCACTCCCTCCTCT	15	<lmxll>	ll	135	lm	136		
>ref-175216	CCTCCACTCACTATAGCTCCTGTGTGT	1	<nnxnp>	nn	104	np	140		
>ref-175222	AATCACTCGACTCTATCTCCCCCTTTC	15	<lmxll>	ll	111	lm	122		
>ref-175225	ACCACCATCACCACGGCTCCCTCTCCT	16	<lmxll>	ll	132	lm	131		
>ref-175234	TCCTCCCCTACTCCACCTCCATCTCTA	1	<hkxhk>	hh	16	hk	152	kk	88
>ref-175234	TCCTCCCCTACTCCACCTCCATCTCTA	7	<hkxhk>	hh	88	hk	151	kk	16
>ref-175246	TTTCTCTTCACATTCCCTCCATTTCCC	14	<nnxnp>	nn	108	np	112		
>ref-175246	TTTCTCTTCACATTCCCTCCATTTCCC	25	<nnxnp>	nn	108	np	112		
>ref-175279	ACTCGGACTACTGTACCTCCCTCCTTT	8	<lmxll>	ll	101	lm	132		
>ref-175287	GAGGACTGGACTCCTGCTCCTCCTGTC	24	<nnxnp>	nn	134	np	134		
>ref-175291	ATGGCTCAAACTACAGCTCCAGTCCTG	6	<hkxhk>	hh	61	hk	153	kk	51
>ref-175297	TCCCCCCCTACCTCCCCTCCCTTCTCC	21	<lmxll>	ll	0	lm	240		
>ref-175332	TTTGCCTCTACTCTCCCTCCTCCCCTC	4	<lmxll>	ll	262	lm	0		
>ref-175332	TTTGCCTCTACTCTCCCTCCTCCCCTC	12	<lmxll>	ll	266	lm	0		
>ref-175344	GGTATATTAACATCACCTCCCTCGTTG	1	<lmxll>	ll	137	lm	125		
>ref-175347	GGGCGTCGCACCGTATCTCCCTTCGGC	22	<lmxll>	ll	118	lm	136		
>ref-175350	CTAAACGGTACAATTTCTCCGACATCA	7	<hkxhk>	hh	0	hk	128	kk	93

>ref-175350	CTAAACGGTACAATTTCTCCGACATCA	26	<hkxhk>	hh	94	hk	128	kk	0
>ref-175352	CCTCCACCAACACTCTCTCCTCCAATC	14	<hkxhk>	hh	0	hk	257	kk	14
>ref-175370	TCCATCTTTACTCTCCCTCCCCATCTC	27	<nnxnp>	nn	2	np	248		
>ref-175371	TATAATTACACACACGCTCCCTTTGAA	3	<hkxhk>	hh	0	hk	273	kk	0
>ref-175371	TATAATTACACACACGCTCCCTTTGAA	6	<hkxhk>	hh	0	hk	273	kk	0
>ref-175371	TATAATTACACACACGCTCCCTTTGAA	23	<hkxhk>	hh	0	hk	273	kk	0
>ref-175373	GATTTCCTTACACACACTCCTTCCCTG	9	<lmxll>	ll	114	lm	121		
>ref-175376	CGTGTACCCACTTTGGCTCCCAGGCCC	26	<lmxll>	ll	139	lm	131		
>ref-175391	TAACATTACACTCTCTCTCCCTCCATC	13	<lmxll>	ll	138	lm	125		
>ref-175394	CCCGGACTAACAAAGGCTCCCCCAACA	5	<hkxhk>	hh	58	hk	130	kk	47
>ref-175412	CTTCCTCGAACCTTCTCTCCAGCTGTT	4	<nnxnp>	nn	151	np	118		
>ref-175462	ATTAACTCCACCCTCCCTCCTCCCTCC	1	<nnxnp>	nn	201	np	25		
>ref-175462	ATTAACTCCACCCTCCCTCCTCCCTCC	5	<nnxnp>	nn	244	np	3		
>ref-175462	ATTAACTCCACCCTCCCTCCTCCCTCC	14	<hkxhk>	hh	29	hk	131	kk	66
>ref-175474	ACCCTGGTCACCCTCCCTCCTTCCATT	4	<lmxll>	ll	113	lm	138		
>ref-175487	GTCCCCACTACCCGTGCTCCTCCTGTG	23	<lmxll>	ll	140	lm	119		
>ref-175489	TTATCTGGCACCTCCTCTCCTCTTCTA	2	<nnxnp>	nn	106	np	136		
>ref-175489	TTATCTGGCACCTCCTCTCCTCTTCTA	24	<hkxhk>	hh	42	hk	130	kk	72
>ref-175489	TTATCTGGCACCTCCTCTCCTCTTCTA	25	<hkxhk>	hh	41	hk	130	kk	73
>ref-175489	TTATCTGGCACCTCCTCTCCTCTTCTA	27	<nnxnp>	nn	104	np	136		
>ref-175501	GGTGACGCCACTCACCCTCCCGGCGTC	7	<nnxnp>	nn	267	np	0		
>ref-175504	CACCCACTCACACATCCTCCATCCCTT	27	<hkxhk>	hh	0	hk	267	kk	4
>ref-175521	CAACGAACTACCCTGCCTCCAGAATGT	9	<lmxll>	ll	134	lm	129		
>ref-175524	GCCAACAGGACAGCTCCTCCCAGCAAC	26	<nnxnp>	nn	266	np	0		
>ref-175524	GCCAACAGGACAGCTCCTCCCAGCAAC	27	<nnxnp>	nn	266	np	0		
>ref-175526	GGCGTAAACACACGACCTCCTTGATAA	2	<hkxhk>	hh	2	hk	269	kk	0
>ref-175526	GGCGTAAACACACGACCTCCTTGATAA	12	<hkxhk>	hh	13	hk	253	kk	0
>ref-175549	TCCTTCAGGACTGGCCCTCCGCTGAGC	1	<lmxll>	ll	185	lm	69		
>ref-175549	TCCTTCAGGACTGGCCCTCCGCTGAGC	8	<hkxhk>	hh	0	hk	247	kk	10
>ref-175549	TCCTTCAGGACTGGCCCTCCGCTGAGC	15	<hkxhk>	hh	2	hk	260	kk	0
>ref-175553	CTCTTCCCAACACGTCCTCCGCTGAGT	14	<hkxhk>	hh	54	hk	153	kk	58
>ref-175557	AAAACACTAACATCAACTCCAACACCT	27	<nnxnp>	nn	123	np	125		
>ref-175581	CCCTCTCCTACTCCTCCTCCTCCAAAC	1	<hkxhk>	hh	1	hk	235	kk	16
>ref-175581	CCCTCTCCTACTCCTCCTCCTCCAAAC	3	<hkxhk>	hh	17	hk	233	kk	1
>ref-175588	CTCTCACATACCATCTCTCCTTCATTC	9	<nnxnp>	nn	134	np	125		
>ref-175593	CCTCTCCTCACACCCACTCCTTTCCCT	16	<hkxhk>	hh	4	hk	262	kk	0
>ref-175593	CCTCTCCTCACACCCACTCCTTTCCCT	26	<hkxhk>	hh	4	hk	263	kk	0
>ref-175595	GTTCCACTCACCTTCCCTCCTTCCCTT	5	<lmxll>	ll	264	lm	1		

>ref-175595	GTTCCACTCACCTTCCCTCCTTCCCTT	27	<lmxll>	ll	262	lm	3		
>ref-175605	CACCATCCCACTCTGCCTCCCACCCCA	23	<nnxnp>	nn	137	np	132		
>ref-175606	CTTTCAATTACCTTCCCTCCCCCAGTC	24	<nnxnp>	nn	119	np	139		
>ref-175609	CTCTCGACCACCTTTTCTCCACATGAC	12	<lmxll>	ll	119	lm	125		
>ref-175642	TATTAGCCAACTTACCCTCCACTTTTG	22	<lmxll>	ll	125	lm	117		
>ref-175650	AGGTCAGGGACTCAAGCTCCATTTCTG	21	<hkxhk>	hh	3	hk	268	kk	0
>ref-175662	CCCTTTCGCACGTTCTCTCCTCCTCCC	1	<lmxll>	ll	219	lm	4		
>ref-175662	CCCTTTCGCACGTTCTCTCCTCCTCCC	25	<nnxnp>	nn	140	np	103		
>ref-175665	CAATGAAATACCGTGTCTCCATATGTC	9	<nnxnp>	nn	272	np	0		
>ref-175686	CCACCCCACACCCACCCTCCTACCCCA	4	<nnxnp>	nn	204	np	32		
>ref-175686	CCACCCCACACCCACCCTCCTACCCCA	25	<lmxll>	ll	24	lm	221		
>ref-175702	TCTTCTCTAACTTCGTCTCCTTCTTTA	15	<nnxnp>	nn	143	np	129		
>ref-175704	CCAATCTCTACCTAACCTCCTCCTCAC	2	<nnxnp>	nn	127	np	128		
>ref-175729	CATCCCATTACCTTCCCTCCCTCTTCC	6	<lmxll>	ll	134	lm	130		
>ref-175740	ACTTCGCAGACCCTTGCTCCGCGTGTC	3	<lmxll>	ll	128	lm	139		
>ref-175746	CGTACGCGCACCTTACCTCCTATGCCC	21	<lmxll>	ll	109	lm	128		
>ref-175748	CTCTACCTCACCCCCTCTCCTCACCCC	6	<nnxnp>	nn	261	np	0		
>ref-175763	CATGTTCCTACCTTATCTCCCCCATTA	4	<lmxll>	ll	138	lm	96		
>ref-175777	GGAGGGCGCACCTGGGCTCCCTGCGGG	5	<hkxhk>	hh	0	hk	273	kk	0
>ref-175777	GGAGGGCGCACCTGGGCTCCCTGCGGG	8	<nnxnp>	nn	240	np	29		
>ref-175777	GGAGGGCGCACCTGGGCTCCCTGCGGG	15	<hkxhk>	hh	0	hk	234	kk	32
>ref-175777	GGAGGGCGCACCTGGGCTCCCTGCGGG	25	<lmxll>	ll	146	lm	109		
>ref-175792	AAACCTCCAACGCCCGCTCCGCCCGCT	9	<nnxnp>	nn	104	np	116		
>ref-175799	CTCACCCTCACCCCTCCTCCTTGAACT	6	<hkxhk>	hh	0	hk	262	kk	6
>ref-175799	CTCACCCTCACCCCTCCTCCTTGAACT	25	<hkxhk>	hh	0	hk	261	kk	6
>ref-175822	ATCAAAAGAACTTTAACTCCGCACCAG	7	<lmxll>	ll	116	lm	139		
>ref-175830	CCCTGCTCCACTTTCACTCCACGAACC	4	<nnxnp>	nn	117	np	108		
>ref-175830	CCCTGCTCCACTTTCACTCCACGAACC	5	<nnxnp>	nn	114	np	108		
>ref-175840	TACCTACTTACTTATCCTCCTTCCTAC	3	<hkxhk>	hh	4	hk	205	kk	54
>ref-175840	TACCTACTTACTTATCCTCCTTCCTAC	5	<lmxll>	ll	26	lm	236		
>ref-175840	TACCTACTTACTTATCCTCCTTCCTAC	21	<lmxll>	ll	26	lm	236		
>ref-175883	CACCTACCCACCCTTTCTCCCTCTCTA	9	<nnxnp>	nn	121	np	136		
>ref-175885	CTCCCTGCCACGCTCACTCCCCGAATA	7	<nnxnp>	nn	134	np	136		
>ref-175904	TTCCCCTCAACTTTTACTCCTTCTCCC	13	<hkxhk>	hh	9	hk	254	kk	1
>ref-175904	TTCCCCTCAACTTTTACTCCTTCTCCC	16	<hkxhk>	hh	1	hk	254	kk	9
>ref-175932	AGGCGCATAACGAAGCCTCCATTCATT	24	<lmxll>	ll	126	lm	145		
>ref-175939	GAACATATAACCCATCCTCCCTCAAAT	22	<nnxnp>	nn	129	np	95		
>ref-175955	TCACCCTTAACTCTCTCTCCTCTCTCC	7	<nnxnp>	nn	136	np	137		

>ref-175955	TCACCCTTAACTCTCTCTCCTCTCTCC	22	<hkxhk>	hh	0	hk	129	kk	130
>ref-175955	TCACCCTTAACTCTCTCTCCTCTCTCC	26	<hkxhk>	hh	2	hk	271	kk	0
>ref-175978	CACTCCCGCACTACCTCTCCTACCACC	23	<nnxnp>	nn	159	np	111		
>ref-175979	GTCTCTGGCACGTCGTCTCCCTTATAT	12	<lmxll>	ll	136	lm	124		
>ref-175994	ATTCTCTAAACTTTCTCTCCAATCCCC	8	<hkxhk>	hh	6	hk	253	kk	0
>ref-175994	ATTCTCTAAACTTTCTCTCCAATCCCC	21	<hkxhk>	hh	6	hk	253	kk	0
>ref-176031	GTCACTGTCACTGCCTCTCCCACTCCT	8	<nnxnp>	nn	101	np	143		
>ref-176065	ATCAGGATTACTTCCTCTCCCTTTCTC	5	<lmxll>	ll	131	lm	137		
>ref-176113	CGTCCCCTCACTGTCACTCCTTTGGAG	9	<hkxhk>	hh	12	hk	248	kk	1
>ref-176113	CGTCCCCTCACTGTCACTCCTTTGGAG	16	<hkxhk>	hh	12	hk	248	kk	1
>ref-176143	ATCTTTTATACTCCTTCTCCCTTTCTC	23	<lmxll>	ll	126	lm	125		
>ref-176147	CTCCCTCCCACCGCCTCTCCACTCTCC	7	<hkxhk>	hh	0	hk	256	kk	9
>ref-176147	CTCCCTCCCACCGCCTCTCCACTCTCC	13	<hkxhk>	hh	0	hk	234	kk	5
>ref-176147	CTCCCTCCCACCGCCTCTCCACTCTCC	21	<lmxll>	ll	116	lm	139		
>ref-176165	CTATCTCGTACTTTGTCTCCCACTAAA	3	<hkxhk>	hh	55	hk	130	kk	55
>ref-176188	GGCTGCATTACTAAGCCTCCGCCGAAG	22	<lmxll>	ll	149	lm	120		
>ref-176188	GGCTGCATTACTAAGCCTCCGCCGAAG	23	<lmxll>	ll	149	lm	120		
>ref-176232	ACTTCTATTACTCCTCCTCCAAGTACT	26	<hkxhk>	hh	2	hk	260	kk	3
>ref-176232	ACTTCTATTACTCCTCCTCCAAGTACT	27	<hkxhk>	hh	3	hk	260	kk	2
>ref-176246	CGTTCCTTCACCTATTCTCCGTCGTTC	27	<lmxll>	ll	124	lm	147		
>ref-176263	CGCACACATACACCTCCTCCCACATAC	26	<nnxnp>	nn	123	np	144		
>ref-176269	CTCCCAGTTACTCTTACTCCTTTCTCC	22	<lmxll>	ll	130	lm	132		
>ref-176289	AACCTTCGGACGCATCCTCCACGACAC	3	<lmxll>	ll	127	lm	144		
>ref-176289	AACCTTCGGACGCATCCTCCACGACAC	9	<lmxll>	ll	127	lm	144		
>ref-176300	AACAAACAAACAATAACTCCATATCCA	8	<lmxll>	ll	140	lm	118		
>ref-176308	TTGCTTTGCACGTTTTCTCCTTTTGTT	2	<nnxnp>	nn	124	np	119		
>ref-176322	ACACGTCACACCCAACCTCCACAGCCA	2	<nnxnp>	nn	130	np	141		
>ref-176332	ATTTTTTTCACTACCCCTCCTTCCCCT	16	<nnxnp>	nn	100	np	120		
>ref-176334	CGCCCCACTACCTTCTCTCCCCTTCCT	25	<nnxnp>	nn	135	np	112		
>ref-176340	TCCCCAACCACTCCTTCTCCCCCTCCT	7	<nnxnp>	nn	7	np	260		
>ref-176340	TCCCCAACCACTCCTTCTCCCCCTCCT	27	<nnxnp>	nn	7	np	260		
>ref-176394	CCAGGGGCTACTTCGCCTCCGGGGAAT	2	<hkxhk>	hh	98	hk	156	kk	0
>ref-176394	CCAGGGGCTACTTCGCCTCCGGGGAAT	7	<hkxhk>	hh	0	hk	271	kk	1
>ref-176394	CCAGGGGCTACTTCGCCTCCGGGGAAT	8	<hkxhk>	hh	7	hk	221	kk	0
>ref-176394	CCAGGGGCTACTTCGCCTCCGGGGAAT	15	<hkxhk>	hh	0	hk	269	kk	4
>ref-176435	TCAAAGTGAACGTCGCCTCCGTAATGC	8	<lmxll>	ll	218	lm	1		
>ref-176488	CTGTCGCTGACTATCGCTCCTGTGCCA	1	<nnxnp>	nn	137	np	127		
>ref-176500	CCGGGACTCACGCACACTCCGAGTCAT	23	<nnxnp>	nn	67	np	200		

>ref-176500	CCGGGACTCACGCACACTCCGAGTCAT	24	<lmxll>	ll	125	lm	119		
>ref-176513	GAAGCTGTCACATCTGCTCCACCAATT	1	<nnxnp>	nn	13	np	255		
>ref-176513	GAAGCTGTCACATCTGCTCCACCAATT	7	<lmxll>	ll	62	lm	204		
>ref-176513	GAAGCTGTCACATCTGCTCCACCAATT	14	<nnxnp>	nn	136	np	134		
>ref-176513	GAAGCTGTCACATCTGCTCCACCAATT	25	<nnxnp>	nn	135	np	134		
>ref-176515	TCTCTCTCTACCCTCCCTCCTTTACCG	2	<nnxnp>	nn	258	np	0		
>ref-176529	TCCATACACACATACCCTCCACATTCA	2	<hkxhk>	hh	4	hk	266	kk	0
>ref-176529	TCCATACACACATACCCTCCACATTCA	15	<hkxhk>	hh	4	hk	266	kk	0
>ref-176531	CCGAGCGTGACTCCATCTCCGCGAATG	1	<hkxhk>	hh	70	hk	117	kk	46
>ref-176531	CCGAGCGTGACTCCATCTCCGCGAATG	2	<hkxhk>	hh	46	hk	117	kk	70
>ref-176569	ATAATTCTAACGTCATCTCCGGGTAAT	12	<nnxnp>	nn	152	np	92		
>ref-176606	CCTCCCCTCACCTTCTCTCCTTCGCCC	16	<lmxll>	ll	2	lm	258		
>ref-176606	CCTCCCCTCACCTTCTCTCCTTCGCCC	21	<lmxll>	ll	2	lm	258		
>ref-176608	AACAGGCGTACCTCTGCTCCATGGATA	1	<nnxnp>	nn	112	np	155		
>ref-176608	AACAGGCGTACCTCTGCTCCATGGATA	2	<nnxnp>	nn	113	np	154		
>ref-176611	CTGACTAACACAAGCTCTCCCACCTGT	1	<hkxhk>	hh	1	hk	272	kk	0
>ref-176611	CTGACTAACACAAGCTCTCCCACCTGT	6	<hkxhk>	hh	0	hk	166	kk	94
>ref-176611	CTGACTAACACAAGCTCTCCCACCTGT	9	<hkxhk>	hh	93	hk	166	kk	0
>ref-176611	CTGACTAACACAAGCTCTCCCACCTGT	12	<hkxhk>	hh	0	hk	264	kk	6
>ref-176643	CCCACTCTCACCCCTTCTCCCACCTTC	4	<lmxll>	ll	115	lm	132		
>ref-176643	CCCACTCTCACCCCTTCTCCCACCTTC	25	<lmxll>	ll	120	lm	130		
>ref-176649	TCTCTCTTCACCCTTCCTCCCCTTCTC	5	<lmxll>	ll	6	lm	265		
>ref-176649	TCTCTCTTCACCCTTCCTCCCCTTCTC	21	<lmxll>	ll	6	lm	265		
>ref-176663	TCCCCAGGTACCCCCTCTCCCCCTGCA	8	<nnxnp>	nn	135	np	126		
>ref-176681	TTTACCACCACCGCTTCTCCCACATCC	7	<lmxll>	ll	147	lm	73		
>ref-176682	ATGTAAACAACCCATTCTCCAGTTGCC	25	<nnxnp>	nn	144	np	102		
>ref-176696	CTTACTCTGACCCTCCCTCCTCCCTTA	16	<nnxnp>	nn	99	np	130		
>ref-176716	TCCCTATGCACACTTTCTCCCTTTCTC	8	<hkxhk>	hh	1	hk	271	kk	0
>ref-176716	TCCCTATGCACACTTTCTCCCTTTCTC	13	<hkxhk>	hh	0	hk	271	kk	1
>ref-176717	GCTTGCATCACCTTGTCTCCGCGCCTC	3	<nnxnp>	nn	110	np	151		
>ref-176732	TGCCTATTAACACTTCCTCCTTCTCTC	12	<lmxll>	ll	114	lm	150		
>ref-176733	TGAATGATGACACATTCTCCTTGTATC	23	<lmxll>	ll	131	lm	137		
>ref-176738	GGCATCACGACAGCCGCTCCATCCCAC	13	<lmxll>	ll	121	lm	152		
>ref-176741	CCCACCCGCACAAAACCTCCTCCATTC	2	<lmxll>	ll	123	lm	145		
>ref-176753	ATCTCATATACACTCCCTCCTATTCTT	5	<nnxnp>	nn	131	np	104		
>ref-176760	CACATATACACATGCACTCCTAAGCCA	16	<nnxnp>	nn	110	np	132		
>ref-176772	ACTTTTTCGACGTCGACTCCTCCTTCC	2	<nnxnp>	nn	100	np	134		
>ref-176772	ACTTTTTCGACGTCGACTCCTCCTTCC	5	<nnxnp>	nn	100	np	137		

>ref-176775	CACAATTACACGCGTACTCCTGGTATT	14	<lmxll>	ll	126	lm	129		
>ref-176775	CACAATTACACGCGTACTCCTGGTATT	25	<lmxll>	ll	126	lm	129		
>ref-176776	TGATAATCAACTCGGCCTCCAAATCAG	22	<lmxll>	ll	127	lm	140		
>ref-176776	TGATAATCAACTCGGCCTCCAAATCAG	25	<lmxll>	ll	131	lm	137		
>ref-176804	CCAGGACAGACACCGCCTCCTCGCACT	16	<nnxnp>	nn	122	np	147		
>ref-176805	CATGGAGCCACCACCCCTCCTCCTCCT	7	<nnxnp>	nn	127	np	133		
>ref-176824	GCAGCAGAGACTGCCACTCCAGAATAG	12	<hkxhk>	hh	3	hk	259	kk	3
>ref-176824	GCAGCAGAGACTGCCACTCCAGAATAG	14	<hkxhk>	hh	3	hk	259	kk	3
>ref-176824	GCAGCAGAGACTGCCACTCCAGAATAG	15	<hkxhk>	hh	2	hk	179	kk	79
>ref-176841	CACTCTACCACCCCCTCTCCTCCCCTC	6	<lmxll>	ll	46	lm	219		
>ref-176841	CACTCTACCACCCCCTCTCCTCCCCTC	8	<hkxhk>	hh	133	hk	134	kk	0
>ref-176843	TACCACGACACACGAGCTCCACCTAAT	2	<hkxhk>	hh	0	hk	272	kk	1
>ref-176843	TACCACGACACACGAGCTCCACCTAAT	27	<hkxhk>	hh	1	hk	272	kk	0
>ref-176851	AGGTTGCATACCCCCTCTCCCCCCTAC	24	<nnxnp>	nn	92	np	137		
>ref-176860	CTCTATCCCACCCTACCTCCTCCACCT	14	<nnxnp>	nn	123	np	141		
>ref-176891	TTGACATCCACTATCCCTCCAAACAAT	27	<nnxnp>	nn	96	np	126		
>ref-176893	GACAGAGTAACAATATCTCCCTCATTT	7	<nnxnp>	nn	116	np	133		
>ref-176931	GACAGAGACACACTCCCTCCCCACACA	5	<nnxnp>	nn	113	np	146		
>ref-176931	GACAGAGACACACTCCCTCCCCACACA	12	<nnxnp>	nn	115	np	146		
>ref-176986	TTTTTGCTAACCCCTCCTCCCCCAAAG	24	<lmxll>	ll	110	lm	109		
>ref-177023	CCTCAGCTCACTCTCCCTCCCTCAGCT	2	<hkxhk>	hh	77	hk	179	kk	0
>ref-177027	CTCTTATTAACGTTACCTCCGCCGAGC	2	<nnxnp>	nn	244	np	0		
>ref-177027	CTCTTATTAACGTTACCTCCGCCGAGC	23	<nnxnp>	nn	244	np	0		
>ref-177033	TGCATACATACGACGCCTCCACCTATG	6	<hkxhk>	hh	131	hk	134	kk	1
>ref-177033	TGCATACATACGACGCCTCCACCTATG	23	<nnxnp>	nn	272	np	0		
>ref-177054	TGACCTTGTACACCCCCTCCCCCTCTC	3	<hkxhk>	hh	7	hk	257	kk	0
>ref-177054	TGACCTTGTACACCCCCTCCCCCTCTC	23	<lmxll>	ll	147	lm	116		
>ref-177059	AACCATAACACGGGCTCTCCCACTTTA	2	<nnxnp>	nn	122	np	119		
>ref-177080	TATTTATATACACGACCTCCTCACGCC	5	<lmxll>	ll	132	lm	125		
>ref-177107	TTTCCTCTCACGGCATCTCCTCCTCCT	2	<lmxll>	ll	5	lm	264		
>ref-177107	TTTCCTCTCACGGCATCTCCTCCTCCT	3	<lmxll>	ll	5	lm	264		
>ref-177107	TTTCCTCTCACGGCATCTCCTCCTCCT	26	<hkxhk>	hh	0	hk	270	kk	1
>ref-177111	CTTCCCTCTACTAAGCCTCCTGCCCTC	9	<nnxnp>	nn	119	np	145		
>ref-177127	CTGGCGGCGACGCTGACTCCTTCATTA	1	<lmxll>	ll	146	lm	124		
>ref-177142	GAAAGCATAACACCCACTCCTCTCTTC	16	<lmxll>	ll	120	lm	143		
>ref-177172	ATCTCCCTTACAACCCCTCCCCCTCCC	6	<nnxnp>	nn	141	np	122		
>ref-177173	CATTTACTCACACACCCTCCCACTCCC	16	<lmxll>	ll	15	lm	252		
>ref-177173	CATTTACTCACACACCCTCCCACTCCC	26	<lmxll>	ll	16	lm	251		

>ref-177180	CTCTCTTCCACCTTATCTCCTTCCACC	21	<hkxhk>	hh	1	hk	269	kk	2
>ref-177181	GCTAAGAGCACCCTGTCTCCCTAGTGT	14	<nnxnp>	nn	132	np	136		
>ref-177199	GGGGGATTAACCAAGCCTCCTGACAGA	15	<nnxnp>	nn	127	np	143		
>ref-177202	TAGCGCCATACACTTTCTCCTTTTCAT	25	<nnxnp>	nn	159	np	103		
>ref-177205	CACCCCCACACTGACCCTCCCTCGCCC	4	<hkxhk>	hh	3	hk	264	kk	0
>ref-177205	CACCCCCACACTGACCCTCCCTCGCCC	6	<hkxhk>	hh	3	hk	264	kk	0
>ref-177231	CTCACTTCCACTTCCGCTCCCACTCTT	21	<nnxnp>	nn	271	np	0		
>ref-177231	CTCACTTCCACTTCCGCTCCCACTCTT	23	<nnxnp>	nn	122	np	140		
>ref-177231	CTCACTTCCACTTCCGCTCCCACTCTT	27	<nnxnp>	nn	120	np	139		
>ref-177262	CCTACTGCCACCCCACCTCCAAACAAG	13	<lmxll>	ll	142	lm	120		
>ref-177262	CCTACTGCCACCCCACCTCCAAACAAG	16	<nnxnp>	nn	151	np	115		
>ref-177266	TACGTCGGCACAAGTCCTCCCGAGTTG	15	<nnxnp>	nn	138	np	129		
>ref-177275	GCATGGGGCACTCAGCCTCCCTGAAAT	1	<hkxhk>	hh	2	hk	267	kk	0
>ref-177275	GCATGGGGCACTCAGCCTCCCTGAAAT	22	<hkxhk>	hh	3	hk	266	kk	0
>ref-177297	CACTCAATGACTCACGCTCCACGCTCA	2	<nnxnp>	nn	4	np	267		
>ref-177309	AACAACTTGACACCTGCTCCATCACCA	26	<nnxnp>	nn	132	np	140		
>ref-177332	AACAAACAAACATACACTCCCACCTCT	13	<hkxhk>	hh	0	hk	261	kk	5
>ref-177332	AACAAACAAACATACACTCCCACCTCT	25	<hkxhk>	hh	0	hk	261	kk	5
>ref-177335	CTCTTCTTTACCCTCTCTCCCCTCTCG	2	<lmxll>	ll	125	lm	133		
>ref-177348	CTATTTCTAACCCTTCCTCCTTCACCC	16	<lmxll>	ll	121	lm	135		
>ref-177349	ACAAAGATAACTGCCCCTCCTCATAAT	27	<hkxhk>	hh	57	hk	140	kk	43
>ref-177361	TTCTCCTCCACGCTTCCTCCCCTCTTC	16	<hkxhk>	hh	0	hk	273	kk	0
>ref-177363	ACACTGTACACCCGCCCTCCCTTCCCT	14	<lmxll>	ll	153	lm	106		
>ref-177388	CTAGGAAAGACATTTCCTCCCTGCCCT	4	<nnxnp>	nn	264	np	0		
>ref-177411	CTCTCCCTTACCCTCTCTCCATCTTAT	8	<hkxhk>	hh	0	hk	266	kk	0
>ref-177411	CTCTCCCTTACCCTCTCTCCATCTTAT	25	<hkxhk>	hh	0	hk	266	kk	0
>ref-177425	ATCCCTCTCACCTTAACTCCTCGCTTC	1	<nnxnp>	nn	140	np	115		
>ref-177447	CCGCCACCTACATCTGCTCCTTCTCCA	2	<lmxll>	ll	127	lm	127		
>ref-177447	CCGCCACCTACATCTGCTCCTTCTCCA	3	<lmxll>	ll	120	lm	130		
>ref-177456	AGGATTTATACACATGCTCCCTCTTCT	15	<nnxnp>	nn	128	np	102		
>ref-177457	GATTCTTGCACATTATCTCCCAAGGGA	6	<nnxnp>	nn	127	np	127		
>ref-177457	GATTCTTGCACATTATCTCCCAAGGGA	23	<nnxnp>	nn	133	np	122		
>ref-177460	TTACAGACAACCATCCCTCCTATACTT	27	<hkxhk>	hh	51	hk	141	kk	60
>ref-177461	CACAAACACACTTACCCTCCTTTTCTC	16	<lmxll>	ll	97	lm	154		
>ref-177461	CACAAACACACTTACCCTCCTTTTCTC	22	<lmxll>	ll	95	lm	155		
>ref-177463	GCGAGTGTCACTTCTCCTCCTCCTCCT	4	<lmxll>	ll	102	lm	128		
>ref-177529	CTGCCACTGACGAGAACTCCATCCATC	16	<nnxnp>	nn	110	np	140		
>ref-177538	AGGCCTGGCACAGTGCCTCCTAAGCCT	7	<lmxll>	ll	158	lm	111		

>ref-177584	TGCCACGTCACGAGCACTCCCTTCGCA	21	<nnxnp>	nn	138	np	134		
>ref-177591	ACACAGTCCACCTTACCTCCTGCGTCA	8	<lmxll>	ll	113	lm	152		
>ref-177622	GATAGGCCTACGTTCCCTCCTTTCTCC	23	<nnxnp>	nn	127	np	135		
>ref-177655	GCGATTGAAACCAATGCTCCCCCAATA	9	<nnxnp>	nn	255	np	0		
>ref-177691	ACCTTTGACACCACGACTCCTGATTTC	27	<nnxnp>	nn	261	np	0		
>ref-177718	CATACATAAACTTCCTCTCCCTCTTCC	6	<nnxnp>	nn	29	np	230		
>ref-177718	CATACATAAACTTCCTCTCCCTCTTCC	9	<nnxnp>	nn	29	np	230		
>ref-177727	GCTGCCGTCACCCCTTCTCCCCGCCCC	6	<lmxll>	ll	134	lm	136		
>ref-177739	CACCTCACGACAACTTCTCCACCGTCC	5	<lmxll>	ll	84	lm	143		
>ref-177773	TGACATTCGACTCCTCCTCCTCCTCTT	21	<lmxll>	ll	124	lm	137		
>ref-177791	TTTGAGGACACTGATGCTCCGCCGTGT	3	<hkxhk>	hh	59	hk	151	kk	49
>ref-177811	GAGCCAATTACGAGTACTCCCTGTCTC	12	<lmxll>	ll	55	lm	168		
>ref-177811	GAGCCAATTACGAGTACTCCCTGTCTC	22	<nnxnp>	nn	260	np	10		
>ref-177811	GAGCCAATTACGAGTACTCCCTGTCTC	23	<lmxll>	ll	219	lm	41		
>ref-177811	GAGCCAATTACGAGTACTCCCTGTCTC	27	<lmxll>	ll	63	lm	204		
>ref-177818	TCTCCTTCAACCCCATCTCCCCACCTT	23	<lmxll>	ll	134	lm	131		
>ref-177823	CTCCTTCCTACTTCTCCTCCTCGCTCC	2	<lmxll>	ll	146	lm	123		
>ref-177823	CTCCTTCCTACTTCTCCTCCTCGCTCC	23	<lmxll>	ll	139	lm	124		
>ref-177855	CCCATCTCGACCCCGCCTCCTCCTCTT	25	<nnxnp>	nn	266	np	0		
>ref-177867	CCGCCTCGAACCGGCCCTCCGCCTCGA	21	<lmxll>	ll	172	lm	60		
>ref-177867	CCGCCTCGAACCGGCCCTCCGCCTCGA	22	<lmxll>	ll	173	lm	61		
>ref-177867	CCGCCTCGAACCGGCCCTCCGCCTCGA	25	<nnxnp>	nn	267	np	0		
>ref-177869	TCCCCTTACACTCACTCTCCTTCTCCC	8	<hkxhk>	hh	51	hk	209	kk	0
>ref-177869	TCCCCTTACACTCACTCTCCTTCTCCC	25	<hkxhk>	hh	51	hk	209	kk	0
>ref-177882	CACAACCCAACCACGGCTCCCACCAAA	21	<lmxll>	ll	125	lm	139		
>ref-177898	AGCGGTGCGACGATTACTCCGGGCTTT	5	<hkxhk>	hh	66	hk	153	kk	47
>ref-177906	AGCCTCTCGACCGATCCTCCTTATCTT	2	<nnxnp>	nn	144	np	123		
>ref-177910	CGGCCTTCCACTCCCTCTCCCTTTGAT	15	<nnxnp>	nn	127	np	137		
>ref-177910	CGGCCTTCCACTCCCTCTCCCTTTGAT	21	<lmxll>	ll	137	lm	125		
>ref-177917	TGCGTGCATACACCCCCTCCCCAACTC	3	<lmxll>	ll	154	lm	108		
>ref-177917	TGCGTGCATACACCCCCTCCCCAACTC	22	<nnxnp>	nn	138	np	131		
>ref-177917	TGCGTGCATACACCCCCTCCCCAACTC	26	<lmxll>	ll	155	lm	108		
>ref-177956	GTTACCAACACCATTTCTCCACCACCA	7	<hkxhk>	hh	58	hk	136	kk	51
>ref-177956	GTTACCAACACCATTTCTCCACCACCA	8	<hkxhk>	hh	58	hk	136	kk	50
>ref-177997	CCCTACTCCACTCTCCCTCCCTCACCC	14	<nnxnp>	nn	18	np	242		
>ref-178015	TCCAAAGCCACTCCTCCTCCTCCATCT	15	<nnxnp>	nn	136	np	125		
>ref-178016	TTTGCACACACAATCACTCCCCACATT	25	<nnxnp>	nn	127	np	137		
>ref-178016	TTTGCACACACAATCACTCCCCACATT	26	<nnxnp>	nn	128	np	137		

>ref-178026	CGCCCCTTCACTCATGCTCCACCCCCT	1	<nnxnp>	nn	111	np	154		
>ref-178026	CGCCCCTTCACTCATGCTCCACCCCCT	26	<lmxll>	ll	268	lm	0		
>ref-178026	CGCCCCTTCACTCATGCTCCACCCCCT	27	<lmxll>	ll	267	lm	0		
>ref-178039	CCTCTCTCCACTCCTCCTCCTAGGGTA	3	<hkxhk>	hh	51	hk	132	kk	58
>ref-178076	CTTTCCTGCACTGCATCTCCTTTTCCC	5	<hkxhk>	hh	76	hk	123	kk	41
>ref-178092	AACATACCTACATATCCTCCTTACCGC	25	<lmxll>	ll	136	lm	132		
>ref-178097	GCCCCTCCCACTGTCTCTCCCTCTATT	26	<hkxhk>	hh	71	hk	123	kk	42
>ref-178101	TTTCCATTTACCCCTCCTCCCCTCCTC	22	<lmxll>	ll	230	lm	0		
>ref-178109	ATGCTACATACCTTCCCTCCCTTCTTC	12	<lmxll>	ll	271	lm	0		
>ref-178169	TATAATACGACCTGTGCTCCTATATGG	4	<lmxll>	ll	204	lm	25		
>ref-178169	TATAATACGACCTGTGCTCCTATATGG	25	<nnxnp>	nn	266	np	3		
>ref-178178	GATATTGATACATCCTCTCCATGCTCC	24	<lmxll>	ll	125	lm	130		
>ref-178202	CGCAACAACACCCTCCCTCCTTTCTCT	15	<nnxnp>	nn	120	np	136		
>ref-178202	CGCAACAACACCCTCCCTCCTTTCTCT	23	<nnxnp>	nn	120	np	136		
>ref-178226	AACCTAGATACCTTGACTCCTTTATCG	5	<hkxhk>	hh	0	hk	268	kk	0
>ref-178226	AACCTAGATACCTTGACTCCTTTATCG	26	<hkxhk>	hh	0	hk	268	kk	0
>ref-178235	GCCAGATGTACTGGCCCTCCTAATACC	6	<lmxll>	ll	107	lm	156		
>ref-178237	GAATAGTAAACACGTACTCCTTCCTGT	26	<hkxhk>	hh	62	hk	194	kk	0
>ref-178246	TTCTTTTCTACATTTTCTCCATCTCTC	3	<nnxnp>	nn	118	np	107		
>ref-178246	TTCTTTTCTACATTTTCTCCATCTCTC	13	<nnxnp>	nn	118	np	107		
>ref-178267	CAGGGGTTTACGATCCCTCCGTCACCC	3	<nnxnp>	nn	138	np	132		
>ref-178267	CAGGGGTTTACGATCCCTCCGTCACCC	21	<hkxhk>	hh	67	hk	136	kk	60
>ref-178269	TCCTTCCCCACCTCTTCTCCCACCCCC	2	<hkxhk>	hh	3	hk	268	kk	0
>ref-178269	TCCTTCCCCACCTCTTCTCCCACCCCC	3	<nnxnp>	nn	270	np	0		
>ref-178318	TGGTCTGTGACCATGCCTCCTTTATGG	9	<nnxnp>	nn	258	np	1		
>ref-178319	CTTCCCCTTACTTCCTCTCCTCTTGTC	3	<nnxnp>	nn	75	np	190		
>ref-178319	CTTCCCCTTACTTCCTCTCCTCTTGTC	7	<hkxhk>	hh	111	hk	138	kk	0
>ref-178319	CTTCCCCTTACTTCCTCTCCTCTTGTC	13	<nnxnp>	nn	75	np	190		
>ref-178319	CTTCCCCTTACTTCCTCTCCTCTTGTC	26	<hkxhk>	hh	0	hk	138	kk	110
>ref-178324	ATGAGCATGACTCTTTCTCCTCTTTTC	22	<nnxnp>	nn	110	np	127		
>ref-178327	TTCCCCACCACCTCCCCTCCCTCCATA	22	<lmxll>	ll	127	lm	137		
>ref-178353	AAAGCGCTGACCCTTGCTCCGTCTGCA	16	<lmxll>	ll	126	lm	142		
>ref-178357	AATGGAGTAACTTCGGCTCCGTAGTCA	12	<lmxll>	ll	114	lm	149		
>ref-178362	TTGTCCCTTACCCTCCCTCCCCAGAAC	13	<hkxhk>	hh	47	hk	119	kk	61
>ref-178372	TACAGAGTTACATGCCCTCCTTGCAGC	2	<hkxhk>	hh	3	hk	267	kk	0
>ref-178372	TACAGAGTTACATGCCCTCCTTGCAGC	13	<hkxhk>	hh	0	hk	272	kk	0
>ref-178372	TACAGAGTTACATGCCCTCCTTGCAGC	27	<hkxhk>	hh	3	hk	267	kk	0
>ref-178422	CCCTCTCCTACCATCTCTCCTCCATTA	26	<lmxll>	ll	155	lm	81		

>ref-178422	CCCTCTCCTACCATCTCTCCTCCATTA	27	<lmxll>	ll	155	lm	80		
>ref-178440	CACATGCAAACACTTTCTCCCTCGCCG	13	<lmxll>	ll	125	lm	144		
>ref-178448	CTTTCTTTTACTGCCTCTCCTCTTCCT	15	<nnxnp>	nn	118	np	136		
>ref-178450	GCTTCGTGGACTGCAACTCCACCCTGG	5	<nnxnp>	nn	149	np	117		
>ref-178450	GCTTCGTGGACTGCAACTCCACCCTGG	9	<nnxnp>	nn	12	np	250		
>ref-178450	GCTTCGTGGACTGCAACTCCACCCTGG	23	<nnxnp>	nn	12	np	250		
>ref-178465	CTGACCTCGACCTACACTCCCTCAGTT	4	<lmxll>	ll	118	lm	139		
>ref-178468	AAGGCAATAACTGGTCCTCCTTCAGTC	2	<nnxnp>	nn	130	np	141		
>ref-178471	ACCTCGGTCACTGTCTCTCCCTCCTCT	25	<lmxll>	ll	265	lm	0		
>ref-178472	TCTCTCCTCACGTCTTCTCCTCTCCTC	12	<nnxnp>	nn	247	np	8		
>ref-178472	TCTCTCCTCACGTCTTCTCCTCTCCTC	16	<nnxnp>	nn	264	np	0		
>ref-178472	TCTCTCCTCACGTCTTCTCCTCTCCTC	27	<lmxll>	ll	136	lm	122		
>ref-178476	ACGATCATGACACCTCCTCCCTCCGGT	27	<lmxll>	ll	139	lm	126		
>ref-178478	CTCTCTCTTACACAGTCTCCCTGCCCC	3	<hkxhk>	hh	83	hk	177	kk	0
>ref-178478	CTCTCTCTTACACAGTCTCCCTGCCCC	6	<lmxll>	ll	61	lm	202		
>ref-178510	CTCTTTTATACTCCCTCTCCCCCTCAG	22	<lmxll>	ll	121	lm	105		
>ref-178531	CAGCAACATACACACACTCCAGCATAT	27	<lmxll>	ll	49	lm	196		
>ref-178537	GGGGGACATACTGCCCCTCCTCCATAG	16	<nnxnp>	nn	102	np	129		
>ref-178540	TACCTTCAAACCCCCCCTCCCCTCCCC	25	<lmxll>	ll	67	lm	181		
>ref-178541	ATGCACATTACTCGCTCTCCTATGTTG	24	<nnxnp>	nn	11	np	238		
>ref-178554	ATATCTAAGACTCAGACTCCTTCATCG	6	<hkxhk>	hh	52	hk	153	kk	48
>ref-178566	TTCCTCCAGACAGCTCCTCCTTTCCTC	15	<lmxll>	ll	134	lm	136		
>ref-178566	TTCCTCCAGACAGCTCCTCCTTTCCTC	16	<lmxll>	ll	134	lm	136		
>ref-178578	ATGGTTGGTACTGAAGCTCCCTGGCCT	16	<nnxnp>	nn	114	np	146		
>ref-178585	TTGGTTTCGACATGTCCTCCTTACTAT	3	<hkxhk>	hh	56	hk	139	kk	58
>ref-178587	TTAAGTGTAACAGGAACTCCTTCTCTC	22	<lmxll>	ll	112	lm	152		
>ref-178607	GGGGTGTACACCCATGCTCCCTCAGAA	3	<lmxll>	ll	229	lm	27		
>ref-178607	GGGGTGTACACCCATGCTCCCTCAGAA	15	<lmxll>	ll	230	lm	27		
>ref-178619	CTTCTCCTCACTCTGTCTCCTTCCCTT	15	<nnxnp>	nn	33	np	227		
>ref-178619	CTTCTCCTCACTCTGTCTCCTTCCCTT	24	<nnxnp>	nn	32	np	228		
>ref-178632	AAAAAAGAGACTTGACCTCCGCCTTCA	1	<lmxll>	ll	128	lm	128		
>ref-178639	GTTCTCTCTACTCCCCCTCCTCATCCC	8	<lmxll>	ll	264	lm	0		
>ref-178648	GAGATATACACCATCTCTCCAGCATTT	9	<hkxhk>	hh	46	hk	139	kk	70
>ref-178668	CTTACTCTTACTCCTCCTCCTCCACCC	4	<hkxhk>	hh	0	hk	186	kk	84
>ref-178668	CTTACTCTTACTCCTCCTCCTCCACCC	8	<hkxhk>	hh	0	hk	67	kk	200
>ref-178668	CTTACTCTTACTCCTCCTCCTCCACCC	24	<hkxhk>	hh	0	hk	273	kk	0
>ref-178668	CTTACTCTTACTCCTCCTCCTCCACCC	27	<hkxhk>	hh	0	hk	273	kk	0
>ref-178698	GTGATCCTTACTTCCTCTCCTTCCCGT	26	<nnxnp>	nn	132	np	123		

>ref-178719	ATGATAATAACAACAACTCCTCCTCCT	3	<hkxhk>	hh	29	hk	200	kk	1
>ref-178740	AAAATAAGAACTCGGTCTCCATCTACG	26	<lmxll>	ll	119	lm	124		
>ref-178747	TTGCAGACGACGTTCTCTCCAGTCAAT	27	<nnxnp>	nn	269	np	0		
>ref-178771	TACCCCTTCACCACACCTCCTCCCCCA	27	<lmxll>	ll	198	lm	45		
>ref-178780	TTGTCTTCCACCTTCCCTCCTACTCTT	22	<nnxnp>	nn	229	np	3		
>ref-178780	TTGTCTTCCACCTTCCCTCCTACTCTT	24	<hkxhk>	hh	115	hk	103	kk	2
>ref-178793	GTGAACGTGACGTGTTCTCCTTCGCAG	21	<lmxll>	ll	132	lm	138		
>ref-178806	TCTTTGATCACACCTCCTCCCTTCCTT	6	<hkxhk>	hh	57	hk	119	kk	61
>ref-178807	ACTGCCTTCACTGCGTCTCCCCCATCC	9	<lmxll>	ll	108	lm	161		
>ref-178812	AACAAATCAACCCTTTCTCCACCTCCC	24	<lmxll>	ll	112	lm	146		
>ref-178819	ACATTCGCTACTTACCCTCCCTCTCCT	3	<nnxnp>	nn	0	np	246		
>ref-178823	TATCCACATACCCGCTCTCCCCTTTGT	14	<lmxll>	ll	211	lm	52		
>ref-178823	TATCCACATACCCGCTCTCCCCTTTGT	26	<nnxnp>	nn	60	np	191		
>ref-178823	TATCCACATACCCGCTCTCCCCTTTGT	27	<lmxll>	ll	212	lm	51		
>ref-178836	AATCTTAAGACGCAGCCTCCTTCCTTC	27	<nnxnp>	nn	149	np	109		
>ref-178850	CAGCCCAGAACTGAAACTCCGGCCTGG	1	<lmxll>	ll	121	lm	122		
>ref-178850	CAGCCCAGAACTGAAACTCCGGCCTGG	24	<lmxll>	ll	118	lm	125		
>ref-178884	AACACAGTCACGTTACCTCCCGCAATT	4	<hkxhk>	hh	59	hk	139	kk	62
>ref-178893	CACATACGCACACGCACTCCCCCACAC	5	<lmxll>	ll	6	lm	260		
>ref-178893	CACATACGCACACGCACTCCCCCACAC	8	<hkxhk>	hh	0	hk	19	kk	217
>ref-178893	CACATACGCACACGCACTCCCCCACAC	14	<lmxll>	ll	230	lm	0		
>ref-178893	CACATACGCACACGCACTCCCCCACAC	24	<lmxll>	ll	10	lm	256		
>ref-178900	TCTCTATACACTCTCTCTCCTCCCTTC	4	<lmxll>	ll	267	lm	0		
>ref-178907	CCTGCTCAAACGCCATCTCCTTCAGAG	14	<lmxll>	ll	140	lm	127		
>ref-178921	AACCTCCCTACTTACCCTCCCCCTCCT	15	<hkxhk>	hh	0	hk	272	kk	0
>ref-178941	AGTTACGGTACTACTACTCCACTCCAC	6	<lmxll>	ll	118	lm	125		
>ref-178957	TTCCTTTCCACATTTTCTCCTCTTCAT	2	<nnxnp>	nn	119	np	131		
>ref-178962	CTTCTCCGCACCCTTTCTCCCTCCTCT	4	<hkxhk>	hh	53	hk	198	kk	0
>ref-178962	CTTCTCCGCACCCTTTCTCCCTCCTCT	8	<hkxhk>	hh	0	hk	268	kk	3
>ref-178962	CTTCTCCGCACCCTTTCTCCCTCCTCT	13	<hkxhk>	hh	3	hk	268	kk	0
>ref-178982	TACCTACACACCGACGCTCCCAAACCT	1	<lmxll>	ll	112	lm	158		
>ref-179010	CTCACCTACACATCCCCTCCCCTCCCC	3	<hkxhk>	hh	24	hk	235	kk	0
>ref-179010	CTCACCTACACATCCCCTCCCCTCCCC	12	<hkxhk>	hh	9	hk	227	kk	0
>ref-179021	CACACCCCCACACACTCTCCCCTCCTC	5	<lmxll>	ll	266	lm	0		
>ref-179038	AAAGCACAAACCTCCGCTCCGAATCAC	21	<nnxnp>	nn	124	np	141		
>ref-179042	TCCCCTTCTACTTCCTCTCCTCTCTCA	22	<nnxnp>	nn	130	np	138		
>ref-179050	TGATCACCAACAAGGGCTCCAGCGAGA	23	<hkxhk>	hh	1	hk	272	kk	0
>ref-179050	TGATCACCAACAAGGGCTCCAGCGAGA	25	<nnxnp>	nn	146	np	118		

>ref-179050	TGATCACCAACAAGGGCTCCAGCGAGA	26	<hkxhk>	hh	0	hk	268	kk	4
>ref-179062	GATTTGCCCACTCTCCCTCCTCCTCCT	4	<nnxnp>	nn	270	np	0		
>ref-179062	GATTTGCCCACTCTCCCTCCTCCTCCT	6	<nnxnp>	nn	270	np	0		
>ref-179065	TGTAAAACCACATTCACTCCTGATTGA	12	<nnxnp>	nn	128	np	122		
>ref-179065	TGTAAAACCACATTCACTCCTGATTGA	26	<lmxll>	ll	124	lm	126		
>ref-179072	GCTGGTTCAACCTGCTCTCCCCTCTCA	14	<hkxhk>	hh	1	hk	271	kk	0
>ref-179072	GCTGGTTCAACCTGCTCTCCCCTCTCA	22	<hkxhk>	hh	0	hk	271	kk	1
>ref-179072	GCTGGTTCAACCTGCTCTCCCCTCTCA	25	<lmxll>	ll	3	lm	270		
>ref-179078	GGTGCCCCTACCTGTACTCCTTTGCCT	5	<hkxhk>	hh	41	hk	145	kk	67
>ref-179092	TCTTCTCTGACCCCCTCTCCCATCTTC	25	<nnxnp>	nn	11	np	255		
>ref-179106	CACTCCCCTACCCTTCCTCCATTCCAG	15	<lmxll>	ll	125	lm	115		
>ref-179114	GATGGAGTTACTCTACCTCCCAGTCAT	15	<lmxll>	ll	124	lm	148		
>ref-179124	CCAGAAAAGACACCCCCTCCCTCCACA	8	<nnxnp>	nn	106	np	116		
>ref-179141	GGCCGCCCAACAGGACCTCCAAGCTGC	3	<nnxnp>	nn	127	np	114		
>ref-179144	TCTATCTCTACTTTCCCTCCTCTTCGT	26	<lmxll>	ll	265	lm	0		
>ref-179157	CTAGCCTCTACAAGTACTCCCTTCATC	4	<nnxnp>	nn	125	np	138		
>ref-179158	TGTTGATGGACAGATCCTCCCCAAAGA	13	<lmxll>	ll	125	lm	135		
>ref-179175	CCATGCCCGACTTCGCCTCCATCACGG	16	<hkxhk>	hh	0	hk	270	kk	1
>ref-179193	GTTTCTTTCACACTTTCTCCCCTCCTT	1	<lmxll>	ll	138	lm	87		
>ref-179205	ACATACTTCACATCCTCTCCTCCACTT	8	<nnxnp>	nn	111	np	144		
>ref-179209	ACTCTACTCACACTTACTCCCCTCACA	1	<hkxhk>	hh	115	hk	133	kk	0
>ref-179209	ACTCTACTCACACTTACTCCCCTCACA	6	<hkxhk>	hh	116	hk	127	kk	0
>ref-179237	TGTGCTTGAACTTTAGCTCCTTGAAGC	27	<nnxnp>	nn	224	np	0		
>ref-179262	ATTTCTCAGACCTCTCCTCCCAACTCC	22	<lmxll>	ll	13	lm	246		
>ref-179262	ATTTCTCAGACCTCTCCTCCCAACTCC	24	<lmxll>	ll	13	lm	246		
>ref-179264	CAAGGAGACACACTGCCTCCACGATGT	1	<hkxhk>	hh	0	hk	273	kk	0
>ref-179264	CAAGGAGACACACTGCCTCCACGATGT	15	<nnxnp>	nn	113	np	144		
>ref-179265	AGAAAATAGACTCCCCCTCCTCCTCCT	23	<lmxll>	ll	107	lm	118		
>ref-179269	CATGTAGCAACGATGGCTCCAGCACGA	2	<nnxnp>	nn	129	np	136		
>ref-179326	TATCCTACTACTAGATCTCCATAACTT	2	<nnxnp>	nn	112	np	143		
>ref-179326	TATCCTACTACTAGATCTCCATAACTT	21	<nnxnp>	nn	112	np	143		
>ref-179327	ATGGATCAGACTCTTACTCCAGGGCTC	12	<hkxhk>	hh	73	hk	134	kk	62
>ref-179333	CAAACGAACACTGTCTCTCCCTCATTC	8	<hkxhk>	hh	43	hk	149	kk	59
>ref-179344	CGGGTTTTCACATAACCTCCACCGCTC	27	<nnxnp>	nn	117	np	109		
>ref-179348	GCTGGCGAGACGATTCCTCCAATTCTG	24	<nnxnp>	nn	121	np	109		
>ref-179368	TCCTCCTTCACCCTCCCTCCTGTCCAC	3	<nnxnp>	nn	5	np	264		
>ref-179381	CAATCCGTCACCGTTTCTCCAATTGCA	27	<lmxll>	ll	135	lm	129		
>ref-179406	ACTACAACAACTACCCCTCCTCAATAC	24	<hkxhk>	hh	1	hk	267	kk	0

>ref-179406	ACTACAACAACTACCCCTCCTCAATAC	26	<lmxll>	ll	169	lm	78		
>ref-179406	ACTACAACAACTACCCCTCCTCAATAC	27	<hkxhk>	hh	1	hk	267	kk	0
>ref-179411	GGCGTGAATACAGTGGCTCCCTGTATA	1	<nnxnp>	nn	135	np	132		
>ref-179424	TTCGTTGCGACGTAGACTCCATTTGCA	15	<lmxll>	ll	123	lm	137		
>ref-179434	ATTACGGAGACCCTCTCTCCTCTCTTT	26	<lmxll>	ll	130	lm	132		
>ref-179455	ATCAGCACCACTAATACTCCCTCGTTA	1	<hkxhk>	hh	16	hk	235	kk	0
>ref-179478	CTCCCCCCAACCTCCTCTCCATCCTCT	9	<hkxhk>	hh	14	hk	229	kk	0
>ref-179478	CTCCCCCCAACCTCCTCTCCATCCTCT	22	<hkxhk>	hh	1	hk	265	kk	4
>ref-179478	CTCCCCCCAACCTCCTCTCCATCCTCT	24	<hkxhk>	hh	74	hk	152	kk	0
>ref-179478	CTCCCCCCAACCTCCTCTCCATCCTCT	26	<hkxhk>	hh	24	hk	233	kk	0
>ref-179486	CACTCCCCTACCCAAACTCCACCAAAT	9	<lmxll>	ll	122	lm	109		
>ref-179486	CACTCCCCTACCCAAACTCCACCAAAT	13	<lmxll>	ll	102	lm	136		
>ref-179486	CACTCCCCTACCCAAACTCCACCAAAT	26	<lmxll>	ll	101	lm	135		
>ref-179490	GCCTCCACCACGCCCCCTCCCCCAATA	2	<hkxhk>	hh	0	hk	212	kk	44
>ref-179497	TACACTTCTACATCATCTCCAAGTGAA	27	<nnxnp>	nn	140	np	124		
>ref-179509	TTCTTACTCACAGCTTCTCCAACAAGT	2	<nnxnp>	nn	272	np	0		
>ref-179519	TTATTAATCACCCTTTCTCCTTCTCCT	21	<lmxll>	ll	121	lm	120		
>ref-179519	TTATTAATCACCCTTTCTCCTTCTCCT	24	<lmxll>	ll	122	lm	120		
>ref-179522	TTACTTTTAACTTCTCCTCCCCTTCCT	27	<nnxnp>	nn	124	np	134		
>ref-179526	TCAATATTTACCTCCCCTCCCGCCCTC	22	<nnxnp>	nn	131	np	130		
>ref-179529	GCTTTCCCCACCTCCTCTCCATCTGTC	9	<hkxhk>	hh	56	hk	144	kk	59
>ref-179558	TTCAATTCCACCCTCCCTCCAATCACC	21	<hkxhk>	hh	0	hk	271	kk	0
>ref-179562	CTGCGCAAAACACTGGCTCCCAATCAT	15	<lmxll>	ll	260	lm	8		
>ref-179563	CTCTTACCCACCTCTTCTCCTCCTCTA	16	<nnxnp>	nn	133	np	136		
>ref-179615	AATTAACTAACGTGGACTCCGCTCTCT	6	<nnxnp>	nn	88	np	149		
>ref-179615	AATTAACTAACGTGGACTCCGCTCTCT	27	<nnxnp>	nn	109	np	131		
>ref-179626	CCAAGGCCAACCACGACTCCACGACCA	2	<hkxhk>	hh	6	hk	267	kk	0
>ref-179626	CCAAGGCCAACCACGACTCCACGACCA	9	<nnxnp>	nn	3	np	270		
>ref-179641	GTAAACTTTACCCTAACTCCTTCACTA	25	<lmxll>	ll	120	lm	120		
>ref-179642	CTTGCACTAACCTCACCTCCTTCCCCT	9	<lmxll>	ll	256	lm	1		
>ref-179642	CTTGCACTAACCTCACCTCCTTCCCCT	12	<lmxll>	ll	260	lm	0		
>ref-179653	CAACCTCCAACGGACCCTCCTGCCCTA	9	<nnxnp>	nn	124	np	141		
>ref-179657	ACTCCATCCACAACTTCTCCACTTCTG	4	<lmxll>	ll	123	lm	125		
>ref-179665	CCCCTATCTACCTTCCCTCCTTCCCCT	3	<lmxll>	ll	267	lm	0		
>ref-179673	TTCTCCCTTACCTTCCCTCCCCCCACT	24	<nnxnp>	nn	14	np	247		
>ref-179673	TTCTCCCTTACCTTCCCTCCCCCCACT	25	<nnxnp>	nn	14	np	247		
>ref-179681	AATAATACCACTTGCCCTCCCCACTTG	7	<nnxnp>	nn	115	np	119		
>ref-179681	AATAATACCACTTGCCCTCCCCACTTG	8	<nnxnp>	nn	114	np	121		

>ref-179681	AATAATACCACTTGCCCTCCCCACTTG	26	<hkxhk>	hh	46	hk	129	kk	50
>ref-179709	AAAATCAATACAAACTCTCCACGACCC	4	<lmxll>	ll	90	lm	133		
>ref-179739	TATTCAACAACCATTCCTCCACAAATT	1	<hkxhk>	hh	1	hk	264	kk	2
>ref-179739	TATTCAACAACCATTCCTCCACAAATT	2	<nnxnp>	nn	196	np	49		
>ref-179739	TATTCAACAACCATTCCTCCACAAATT	8	<nnxnp>	nn	177	np	65		
>ref-179739	TATTCAACAACCATTCCTCCACAAATT	25	<lmxll>	ll	30	lm	215		
>ref-179739	TATTCAACAACCATTCCTCCACAAATT	27	<hkxhk>	hh	0	hk	119	kk	118
>ref-179743	TCTTATACAACGTTCACTCCCGCATAC	22	<hkxhk>	hh	52	hk	139	kk	67
>ref-179777	TCTCTCCCCACACCCACTCCCTCTCCT	4	<nnxnp>	nn	265	np	0		
>ref-179777	TCTCTCCCCACACCCACTCCCTCTCCT	16	<nnxnp>	nn	4	np	243		
>ref-179777	TCTCTCCCCACACCCACTCCCTCTCCT	27	<hkxhk>	hh	7	hk	249	kk	2
>ref-179793	ACAATACATACTCTCTCTCCTTTTCTC	9	<hkxhk>	hh	76	hk	120	kk	37
>ref-179793	ACAATACATACTCTCTCTCCTTTTCTC	13	<nnxnp>	nn	139	np	105		
>ref-179808	AAAACTACCACATGTGCTCCAGCGAGA	24	<nnxnp>	nn	120	np	151		
>ref-179811	TCGAGGACAACCCAAGCTCCTCCTTAT	15	<hkxhk>	hh	52	hk	149	kk	59
>ref-179816	GAGGACGCCACCGCGTCTCCATACGAC	13	<nnxnp>	nn	122	np	149		
>ref-179817	GCAAATAAAACCGCATCTCCCACGCCA	21	<lmxll>	ll	125	lm	107		
>ref-179826	GGAGAAAGAACCAATACTCCATGCCTA	15	<hkxhk>	hh	0	hk	270	kk	3
>ref-179826	GGAGAAAGAACCAATACTCCATGCCTA	27	<hkxhk>	hh	3	hk	270	kk	0
>ref-179829	TTGACTCGCACTCGTGCTCCTGTGGGA	23	<nnxnp>	nn	124	np	131		
>ref-179833	TCTCCCTCCACCCCCCCTCCTTTTCAC	23	<nnxnp>	nn	3	np	246		
>ref-179854	GTCTACATGACCCCACCTCCTCCACCT	9	<hkxhk>	hh	0	hk	272	kk	0
>ref-179855	GGAAACCAGACATACTCTCCTCAGTCT	13	<lmxll>	ll	117	lm	139		
>ref-179867	AACACATGAACTTTATCTCCCTCATAA	5	<hkxhk>	hh	0	hk	253	kk	12
>ref-179882	GCCAATACCACCGTCTCTCCTCTCTCG	13	<lmxll>	ll	130	lm	122		
>ref-179884	TCTCTTCTTACTTCCTCTCCCTCTCTT	6	<nnxnp>	nn	135	np	123		
>ref-179884	TCTCTTCTTACTTCCTCTCCCTCTCTT	21	<nnxnp>	nn	137	np	120		
>ref-179920	TAAGGTCCGACGTTCACTCCAAAGAAC	24	<nnxnp>	nn	108	np	142		
>ref-179923	CGACTCACCACCGACTCTCCTCCGACT	21	<hkxhk>	hh	0	hk	272	kk	0
>ref-179923	CGACTCACCACCGACTCTCCTCCGACT	24	<hkxhk>	hh	0	hk	272	kk	0
>ref-179934	TAAACTTCTACCAATGCTCCAGGTTCT	9	<hkxhk>	hh	0	hk	177	kk	79
>ref-179940	AAGTACGTCACTAAGTCTCCTTTCATT	1	<lmxll>	ll	130	lm	132		
>ref-179972	TCATATCCCACCCCCACTCCCATCCCC	27	<lmxll>	ll	115	lm	136		
>ref-179976	TTGCTTTCAACCCCCACTCCATTTCTC	1	<lmxll>	ll	123	lm	128		
>ref-179979	CAGGCATCGACATCTCCTCCACCTGTC	9	<nnxnp>	nn	119	np	148		
>ref-180031	CCCTCAACTACCCTCACTCCCTCATCC	1	<nnxnp>	nn	265	np	0		
>ref-180041	TCTCCCTCGACCTCCTCTCCCCTATCA	25	<nnxnp>	nn	246	np	2		
>ref-180043	CTCCACTGGACGCCAGCTCCACACACA	21	<lmxll>	ll	138	lm	119		

>ref-180054	TGCCTGGCGACTTCTGCTCCTCTCTCT	5	<nnxnp>	nn	113	np	152		
>ref-180113	AAATGCGTTACGGTGCCTCCTTCCAGA	12	<nnxnp>	nn	129	np	108		
>ref-180129	ACTGTCTTTACTTTATCTCCCTCCTCT	8	<hkxhk>	hh	52	hk	144	kk	60
>ref-180133	AATCCCTAAACCGGTTCTCCTTGGCTT	22	<hkxhk>	hh	67	hk	154	kk	45
>ref-180149	CACACATGAACACACTCTCCCCGAGGC	2	<nnxnp>	nn	113	np	123		
>ref-180167	CTCCCTCTCACTCGTTCTCCAACTACA	25	<lmxll>	ll	123	lm	131		
>ref-180181	ATAACCCTCACTCTTTCTCCCTTCTCT	1	<lmxll>	ll	127	lm	123		
>ref-180212	CCATCCCTTACTTTCTCTCCCTTCTCT	8	<hkxhk>	hh	0	hk	261	kk	5
>ref-180212	CCATCCCTTACTTTCTCTCCCTTCTCT	21	<hkxhk>	hh	5	hk	261	kk	0
>ref-180216	CCCCCCTTCACCCCCTCTCCCATCCAT	8	<nnxnp>	nn	112	np	116		
>ref-180231	TATCTTTAAACGGCGTCTCCTCTCCAT	6	<lmxll>	ll	128	lm	133		
>ref-180258	TGTTCTTCAACTTCACCTCCTTCTTCT	16	<lmxll>	ll	146	lm	111		
>ref-180259	CACACACACACTCACACTCCGCAATTA	7	<lmxll>	ll	140	lm	123		
>ref-180259	CACACACACACTCACACTCCGCAATTA	12	<lmxll>	ll	137	lm	125		
>ref-180270	CCCCCTGGCACCTCGTCTCCCCCTGGC	4	<lmxll>	ll	72	lm	190		
>ref-180270	CCCCCTGGCACCTCGTCTCCCCCTGGC	5	<lmxll>	ll	72	lm	189		
>ref-180270	CCCCCTGGCACCTCGTCTCCCCCTGGC	6	<lmxll>	ll	139	lm	123		
>ref-180282	TCCCTCCTTACATCCCCTCCCCCTTCC	24	<lmxll>	ll	53	lm	203		
>ref-180285	TTCGGCAAGACAGGGACTCCACGAGCA	5	<nnxnp>	nn	121	np	132		
>ref-180293	TTCTCACTCACTCCTTCTCCTCACTTC	14	<nnxnp>	nn	7	np	260		
>ref-180293	TTCTCACTCACTCCTTCTCCTCACTTC	15	<nnxnp>	nn	7	np	260		
>ref-180297	CACAGGCTAACCCCTCCTCCCCCCCAC	23	<hkxhk>	hh	67	hk	136	kk	53
>ref-180297	CACAGGCTAACCCCTCCTCCCCCCCAC	24	<hkxhk>	hh	66	hk	137	kk	53
>ref-180314	TGCAGTGAAACCCATACTCCAATATCA	5	<lmxll>	ll	138	lm	110		
>ref-180321	CATCAGGAAACCAGGTCTCCCTCGTCC	23	<nnxnp>	nn	0	np	220		
>ref-180321	CATCAGGAAACCAGGTCTCCCTCGTCC	25	<nnxnp>	nn	0	np	220		
>ref-180329	CTCCTTTCCACTCTCTCTCCGTTTATC	23	<nnxnp>	nn	114	np	131		
>ref-180329	CTCCTTTCCACTCTCTCTCCGTTTATC	27	<nnxnp>	nn	115	np	131		
>ref-180381	CCCAATAGGACACATGCTCCCTGGGTA	16	<nnxnp>	nn	138	np	126		
>ref-180397	CCCTCAATCACTTCACCTCCGTAGATA	7	<hkxhk>	hh	39	hk	112	kk	94
>ref-180405	CTCTGGGGAACACATTCTCCTGTGGCT	5	<nnxnp>	nn	135	np	129		
>ref-180424	CTCCGAGCGACTGCCTCTCCATCCCCC	1	<nnxnp>	nn	270	np	0		
>ref-180429	ACCGAGTGCACTCAGTCTCCCCAAAAA	3	<hkxhk>	hh	5	hk	255	kk	0
>ref-180431	GACCTTTAGACACATCCTCCCCAAATG	9	<nnxnp>	nn	240	np	2		
>ref-180452	TGCCTCCCTACCCACCCTCCTCCCTCC	7	<lmxll>	ll	158	lm	88		
>ref-180457	TACAAATACACTTGCCCTCCCCTCCCC	25	<nnxnp>	nn	229	np	1		
>ref-180457	TACAAATACACTTGCCCTCCCCTCCCC	27	<nnxnp>	nn	228	np	4		
>ref-180549	CTTCCTATTACTTCACCTCCCTTTCCC	6	<lmxll>	ll	124	lm	124		

>ref-180573	CACACCAACACCTCATCTCCAACATAT	8	<lmxll>	ll	242	lm	22		
>ref-180573	CACACCAACACCTCATCTCCAACATAT	26	<lmxll>	ll	1	lm	271		
>ref-180573	CACACCAACACCTCATCTCCAACATAT	27	<nnxnp>	nn	87	np	171		
>ref-180574	GTCATATTGACTGCTGCTCCAGCTCAC	27	<nnxnp>	nn	271	np	0		
>ref-180577	AGCGAAAAAACGGCCACTCCATTGGCT	24	<lmxll>	ll	136	lm	128		
>ref-180593	GCGACAAGAACACAAACTCCACGCGTT	12	<hkxhk>	hh	0	hk	270	kk	0
>ref-180593	GCGACAAGAACACAAACTCCACGCGTT	22	<hkxhk>	hh	1	hk	267	kk	0
>ref-180614	CTCATCACCACACTGACTCCAATGGTC	24	<hkxhk>	hh	47	hk	145	kk	54
>ref-180617	CCACACGCAACAGCAACTCCAGACCCT	6	<hkxhk>	hh	10	hk	260	kk	0
>ref-180617	CCACACGCAACAGCAACTCCAGACCCT	7	<lmxll>	ll	177	lm	88		
>ref-180617	CCACACGCAACAGCAACTCCAGACCCT	8	<hkxhk>	hh	0	hk	192	kk	69
>ref-180617	CCACACGCAACAGCAACTCCAGACCCT	12	<nnxnp>	nn	91	np	165		
>ref-180630	CACACACACACACAATCTCCTAAATTA	16	<hkxhk>	hh	4	hk	265	kk	0
>ref-180630	CACACACACACACAATCTCCTAAATTA	26	<hkxhk>	hh	0	hk	265	kk	4
>ref-180657	CCACATCTAACTGGCTCTCCTCTCCCT	9	<hkxhk>	hh	40	hk	223	kk	0
>ref-180672	TCAGGCGTTACTCCACCTCCGGCACCA	2	<lmxll>	ll	121	lm	142		
>ref-180694	TACTGAACTACTGGATCTCCTGTATGC	1	<hkxhk>	hh	0	hk	223	kk	30
>ref-180694	TACTGAACTACTGGATCTCCTGTATGC	15	<hkxhk>	hh	9	hk	255	kk	0
>ref-180694	TACTGAACTACTGGATCTCCTGTATGC	22	<hkxhk>	hh	0	hk	265	kk	5
>ref-180702	AACTTTGCAACTCGAACTCCCTCCCTC	14	<lmxll>	ll	117	lm	131		
>ref-180704	TGAACTCTGACACTATCTCCCTCTCCC	7	<lmxll>	ll	126	lm	140		
>ref-180717	CCCCCATCTACGATCACTCCAGTACTG	12	<nnxnp>	nn	112	np	151		
>ref-180736	GTTTCCTTTACGTCTTCTCCGCTTTGT	21	<lmxll>	ll	109	lm	147		
>ref-180781	CACATAGACACTCTCCCTCCAGCTTTC	7	<lmxll>	ll	120	lm	114		
>ref-180806	TCCCCTGCAACTCTCCCTCCCCTTCCA	1	<nnxnp>	nn	256	np	0		
>ref-180812	TCTCTCGTCACCCTTTCTCCTTCCCTT	7	<hkxhk>	hh	0	hk	259	kk	11
>ref-180812	TCTCTCGTCACCCTTTCTCCTTCCCTT	12	<hkxhk>	hh	11	hk	258	kk	0
>ref-180818	TCCTAAACCACTCCTACTCCACCCTCT	9	<lmxll>	ll	244	lm	19		
>ref-180818	TCCTAAACCACTCCTACTCCACCCTCT	21	<nnxnp>	nn	73	np	189		
>ref-180818	TCCTAAACCACTCCTACTCCACCCTCT	26	<lmxll>	ll	4	lm	266		
>ref-180821	GGAATGTTAACCTCACCTCCTTTATCT	3	<lmxll>	ll	139	lm	117		
>ref-180832	ATCTTCTTCACTTCTTCTCCGCCCTCT	21	<lmxll>	ll	146	lm	116		
>ref-180863	TTAACCCTTACGCCCCCTCCCTCTTCC	9	<nnxnp>	nn	82	np	145		
>ref-180863	TTAACCCTTACGCCCCCTCCCTCTTCC	22	<hkxhk>	hh	0	hk	254	kk	6
>ref-180864	AACCCATTCACCCGGACTCCAGCCGCA	14	<hkxhk>	hh	93	hk	144	kk	1
>ref-180864	AACCCATTCACCCGGACTCCAGCCGCA	25	<hkxhk>	hh	6	hk	201	kk	36
>ref-180868	GATTGTATGACACGTCCTCCTTGTAGT	4	<nnxnp>	nn	273	np	0		
>ref-180868	GATTGTATGACACGTCCTCCTTGTAGT	21	<hkxhk>	hh	0	hk	188	kk	79

>ref-180882	TCTGTAACTACATATCCTCCCCACTT	9	<hkxhk>	hh	56	hk	127	kk	50
>ref-180887	TCACTTCACACTTGTGCTCCTTGGTCG	9	<lmxll>	ll	128	lm	133		
>ref-180907	ACCAGAGAGACTCGAACTCCCGAACAC	1	<nnxnp>	nn	252	np	12		
>ref-180907	ACCAGAGAGACTCGAACTCCCGAACAC	3	<nnxnp>	nn	202	np	54		
>ref-180907	ACCAGAGAGACTCGAACTCCCGAACAC	21	<hkxhk>	hh	0	hk	272	kk	1
>ref-180907	ACCAGAGAGACTCGAACTCCCGAACAC	26	<hkxhk>	hh	0	hk	272	kk	1
>ref-180932	TCTTCTCCAACTCCTTCTCCTATTCAC	9	<hkxhk>	hh	2	hk	265	kk	1
>ref-180932	TCTTCTCCAACTCCTTCTCCTATTCAC	27	<hkxhk>	hh	2	hk	265	kk	1
>ref-180965	CTAAGCTGAACTCACTCTCCTGAATGT	8	<nnxnp>	nn	235	np	1		
>ref-180976	TCTACTTCTACTTCTGCTCCACGTCAC	22	<lmxll>	ll	143	lm	115		
>ref-180976	TCTACTTCTACTTCTGCTCCACGTCAC	26	<lmxll>	ll	142	lm	115		
>ref-180979	TCTCTCCCCACTTTTCCTCCTTTTCCC	14	<nnxnp>	nn	187	np	70		
>ref-180979	TCTCTCCCCACTTTTCCTCCTTTTCCC	26	<nnxnp>	nn	188	np	69		
>ref-180982	GGGAGAGTGACGGTCCCTCCAAAACCC	12	<hkxhk>	hh	0	hk	161	kk	96
>ref-181027	TCGTCTTGGACAATCCCTCCCTCAGCA	14	<nnxnp>	nn	108	np	145		
>ref-181043	AGCGGGCCGACACAGCCTCCGGCCCTC	14	<nnxnp>	nn	265	np	0		
>ref-181054	GCACAGACAACCCCCCCTCCCTCCTCT	1	<hkxhk>	hh	0	hk	261	kk	5
>ref-181054	GCACAGACAACCCCCCCTCCCTCCTCT	12	<hkxhk>	hh	0	hk	261	kk	5
>ref-181063	TTATCTGAAACTGAAGCTCCGACTCCC	15	<nnxnp>	nn	263	np	0		
>ref-181086	GAGGCTGATACTGTCTCTCCTCGTCGT	3	<lmxll>	ll	141	lm	121		
>ref-181087	TCCATTACAACAAAGGCTCCCTCACAG	3	<lmxll>	ll	115	lm	118		
>ref-181094	ATACGTCCGACATGTCCTCCGTCAGGA	5	<nnxnp>	nn	273	np	0		
>ref-181098	TCTCCACTCACTCTTTCTCCTCTCCCC	4	<lmxll>	ll	255	lm	0		
>ref-181105	GACACCAAAACCCCGCCTCCGTTTGAT	4	<nnxnp>	nn	126	np	135		
>ref-181134	TCTGCCACCACAGTTTCTCCTTCGTAT	26	<lmxll>	ll	119	lm	144		
>ref-181140	AGCCAATAAACCGCATCTCCAAACAGG	9	<hkxhk>	hh	0	hk	273	kk	0
>ref-181140	AGCCAATAAACCGCATCTCCAAACAGG	14	<hkxhk>	hh	0	hk	273	kk	0
>ref-181152	TGACTTCTGACGTCACCTCCTCCTCCA	3	<hkxhk>	hh	63	hk	152	kk	48
>ref-181178	CTCTCTGACACAGCGCCTCCCCGACTG	13	<nnxnp>	nn	128	np	142		
>ref-181209	TAGAAATGCACAATCACTCCTTGGTCT	1	<lmxll>	ll	120	lm	144		
>ref-181210	GTATCCTTTACTTCCTCTCCCTTTCTC	24	<hkxhk>	hh	42	hk	141	kk	75
>ref-181211	TTATACATTACGCATCCTCCGTCTCTC	22	<lmxll>	ll	119	lm	148		
>ref-181211	TTATACATTACGCATCCTCCGTCTCTC	23	<lmxll>	ll	119	lm	148		
>ref-181225	CTATTTCGAACAGTTTCTCCTCTCACA	8	<nnxnp>	nn	125	np	98		
>ref-181283	GGAGGTACAACACGCACTCCCTGTAAT	14	<nnxnp>	nn	127	np	131		
>ref-181285	AGCCTCTGCACGGACCCTCCCATCCCC	21	<lmxll>	ll	138	lm	129		
>ref-181298	CAGTCCATCACATAGGCTCCTCAGAGA	16	<lmxll>	ll	131	lm	124		
>ref-181306	TCTTCTCCCACTCTTTCTCCTTCATCC	6	<nnxnp>	nn	119	np	113		

>ref-181306	TCTTCTCCCACTCTTTCTCCTTCATCC	23	<nnxnp>	nn	256	np	0		
>ref-181306	TCTTCTCCCACTCTTTCTCCTTCATCC	24	<nnxnp>	nn	240	np	0		
>ref-181311	AGGGGACCTACTGCCTCTCCCTCCGAG	25	<lmxll>	ll	138	lm	127		
>ref-181315	CTCACTTGTACTGCCTCTCCTACTTAC	3	<hkxhk>	hh	50	hk	209	kk	0
>ref-181325	TTATATTGAACTGGAGCTCCGCAAGGA	16	<lmxll>	ll	132	lm	134		
>ref-181325	TTATATTGAACTGGAGCTCCGCAAGGA	22	<lmxll>	ll	132	lm	132		
>ref-181327	CCTTTTGTCACAACAGCTCCCTTCCCT	1	<hkxhk>	hh	0	hk	260	kk	5
>ref-181327	CCTTTTGTCACAACAGCTCCCTTCCCT	3	<hkxhk>	hh	0	hk	183	kk	68
>ref-181327	CCTTTTGTCACAACAGCTCCCTTCCCT	7	<hkxhk>	hh	4	hk	264	kk	0
>ref-181330	CTTTCTCTCACACCAGCTCCTCTCTTA	16	<lmxll>	ll	134	lm	86		
>ref-181338	GTCAGCCACACCTGCCCTCCTGGTCTT	26	<nnxnp>	nn	137	np	134		
>ref-181344	GCTTCGACAACCTTACCTCCGCCCTCC	5	<hkxhk>	hh	3	hk	268	kk	0
>ref-181344	GCTTCGACAACCTTACCTCCGCCCTCC	6	<lmxll>	ll	49	lm	214		
>ref-181349	TCTCCCTTTACCTCTCCTCCTGTTCCC	14	<lmxll>	ll	141	lm	113		
>ref-181349	TCTCCCTTTACCTCTCCTCCTGTTCCC	22	<lmxll>	ll	137	lm	114		
>ref-181383	CTCAAAACAACCCCTTCTCCCTCCCCT	3	<nnxnp>	nn	259	np	0		
>ref-181399	CACTTAAACACTAACACTCCCTAGCTG	6	<lmxll>	ll	117	lm	144		
>ref-181403	AAGCGGCTCACCCTTCCTCCTACCTAT	22	<lmxll>	ll	124	lm	132		
>ref-181435	ACTGTACCAACAGCCCCTCCCCTCTCC	9	<hkxhk>	hh	51	hk	158	kk	51
>ref-181435	ACTGTACCAACAGCCCCTCCCCTCTCC	13	<hkxhk>	hh	54	hk	157	kk	50
>ref-181437	CCCCTCTCCACACTTTCTCCCCCTCTT	14	<nnxnp>	nn	10	np	259		
>ref-181451	AGACGGCTGACAACCACTCCGTCTAAG	16	<nnxnp>	nn	103	np	148		
>ref-181461	CCCATGCAAACTACTGCTCCGGTAGTT	5	<hkxhk>	hh	0	hk	193	kk	55
>ref-181461	CCCATGCAAACTACTGCTCCGGTAGTT	9	<hkxhk>	hh	0	hk	273	kk	0
>ref-181461	CCCATGCAAACTACTGCTCCGGTAGTT	21	<hkxhk>	hh	139	hk	111	kk	0
>ref-181461	CCCATGCAAACTACTGCTCCGGTAGTT	23	<hkxhk>	hh	0	hk	273	kk	0
>ref-181462	CTTCTTTCCACTTCCTCTCCTTTCCCT	12	<nnxnp>	nn	137	np	119		
>ref-181462	CTTCTTTCCACTTCCTCTCCTTTCCCT	15	<hkxhk>	hh	1	hk	266	kk	3
>ref-181462	CTTCTTTCCACTTCCTCTCCTTTCCCT	22	<hkxhk>	hh	1	hk	266	kk	3
>ref-181462	CTTCTTTCCACTTCCTCTCCTTTCCCT	24	<nnxnp>	nn	141	np	116		
>ref-181502	TTCCTTCCTACATTCTCTCCTTCCATT	14	<lmxll>	ll	70	lm	201		
>ref-181502	TTCCTTCCTACATTCTCTCCTTCCATT	25	<lmxll>	ll	70	lm	201		
>ref-181506	CACGGAAGCACACTTACTCCCTCACAT	4	<hkxhk>	hh	0	hk	266	kk	5
>ref-181523	TCTCTATCTACCTTTTCTCCTTGTCCT	6	<hkxhk>	hh	1	hk	246	kk	12
>ref-181523	TCTCTATCTACCTTTTCTCCTTGTCCT	13	<hkxhk>	hh	12	hk	246	kk	1
>ref-181526	CTGAGTAGAACGGCGGCTCCATGGTTG	4	<hkxhk>	hh	230	hk	38	kk	0
>ref-181526	CTGAGTAGAACGGCGGCTCCATGGTTG	15	<nnxnp>	nn	152	np	111		
>ref-181526	CTGAGTAGAACGGCGGCTCCATGGTTG	22	<hkxhk>	hh	0	hk	30	kk	241

>ref-181526	CTGAGTAGAACGGCGGCTCCATGGTTG	23	<hkxhk>	hh	0	hk	30	kk	241
>ref-181558	CCTATCGTCACTTTACCTCCTTTCTTC	1	<nnxnp>	nn	220	np	6		
>ref-181558	CCTATCGTCACTTTACCTCCTTTCTTC	6	<nnxnp>	nn	252	np	0		
>ref-181562	CGTAAATATACACACACTCCCTCTGTC	5	<nnxnp>	nn	162	np	94		
>ref-181562	CGTAAATATACACACACTCCCTCTGTC	26	<nnxnp>	nn	162	np	94		
>ref-181564	ATAATTTCAACTCCAACTCCTCCACAT	3	<lmxll>	ll	118	lm	122		
>ref-181580	ACCCTCAATACTCCTCCTCCTCCTACT	22	<nnxnp>	nn	127	np	133		
>ref-181580	ACCCTCAATACTCCTCCTCCTCCTACT	25	<nnxnp>	nn	107	np	142		
>ref-181586	ACCCTCCCAACCTTCCCTCCTTCACCT	4	<lmxll>	ll	262	lm	0		
>ref-181607	TAGGTACCAACATTGTCTCCTGCTGCC	27	<nnxnp>	nn	134	np	132		
>ref-181629	TTCCTGATAACCCTTCCTCCTTCCTTC	7	<nnxnp>	nn	134	np	128		
>ref-181629	TTCCTGATAACCCTTCCTCCTTCCTTC	27	<nnxnp>	nn	136	np	128		
>ref-181631	AAAATCCACACCCTTTCTCCTCTCAAC	14	<nnxnp>	nn	112	np	109		
>ref-181634	TTATCCTCTACTCTCTCTCCCCTTCAC	22	<nnxnp>	nn	115	np	136		
>ref-181643	TTTTATCTCACTCTCTCTCCCTTCTCC	25	<nnxnp>	nn	240	np	6		
>ref-181657	CTAACCCTTACCCCTCCTCCCCCTCCC	2	<nnxnp>	nn	28	np	229		
>ref-181657	CTAACCCTTACCCCTCCTCCCCCTCCC	21	<lmxll>	ll	161	lm	93		
>ref-181658	GGGGAATTTACTACCTCTCCCTCTTGC	1	<nnxnp>	nn	135	np	104		
>ref-181658	GGGGAATTTACTACCTCTCCCTCTTGC	8	<nnxnp>	nn	136	np	104		
>ref-181658	GGGGAATTTACTACCTCTCCCTCTTGC	15	<lmxll>	ll	143	lm	112		
>ref-181680	AATTTACCAACTTTGTCTCCTTGTATT	8	<lmxll>	ll	126	lm	132		
>ref-181695	CTCACTTTCACTAACTCTCCTTCTCCC	4	<lmxll>	ll	264	lm	0		
>ref-181695	CTCACTTTCACTAACTCTCCTTCTCCC	14	<lmxll>	ll	266	lm	0		
>ref-181704	CCCACCCCCACTCTCTCTCCACCCTAT	2	<lmxll>	ll	108	lm	129		
>ref-181704	CCCACCCCCACTCTCTCTCCACCCTAT	24	<nnxnp>	nn	71	np	165		
>ref-181704	CCCACCCCCACTCTCTCTCCACCCTAT	25	<nnxnp>	nn	71	np	166		
>ref-181722	CACGGTTTTACGCGGGCTCCTGCGTGT	6	<nnxnp>	nn	137	np	132		
>ref-181722	CACGGTTTTACGCGGGCTCCTGCGTGT	9	<nnxnp>	nn	137	np	132		
>ref-181728	AGAAGTTCTACTCCCACTCCACCTGGA	5	<hkxhk>	hh	0	hk	228	kk	33
>ref-181728	AGAAGTTCTACTCCCACTCCACCTGGA	14	<hkxhk>	hh	32	hk	229	kk	0
>ref-181737	AGCCCAGCGACTGTCTCTCCGAGTGAT	1	<lmxll>	ll	1	lm	268		
>ref-181746	CTCCCCACTACTCCCCCTCCCCTCCTT	7	<nnxnp>	nn	5	np	253		
>ref-181746	CTCCCCACTACTCCCCCTCCCCTCCTT	21	<nnxnp>	nn	167	np	74		
>ref-181746	CTCCCCACTACTCCCCCTCCCCTCCTT	22	<lmxll>	ll	262	lm	1		
>ref-181746	CTCCCCACTACTCCCCCTCCCCTCCTT	23	<nnxnp>	nn	169	np	75		
>ref-181746	CTCCCCACTACTCCCCCTCCCCTCCTT	26	<lmxll>	ll	264	lm	0		
>ref-181760	ATACGCTAAACCACGTCTCCTAAGCTT	22	<lmxll>	ll	139	lm	115		
>ref-181764	CGGTGTGGCACAGTGCCTCCACATACC	21	<lmxll>	ll	133	lm	137		

>ref-181786	TCTACTGTTACTGTTACTCCTAAGTTT	2	<hkxhk>	hh	3	hk	187	kk	29
>ref-181786	TCTACTGTTACTGTTACTCCTAAGTTT	24	<hkxhk>	hh	3	hk	187	kk	29
>ref-181799	CAGCTGTAGACGTGTCCTCCGGAAAGG	2	<lmxll>	ll	167	lm	82		
>ref-181799	CAGCTGTAGACGTGTCCTCCGGAAAGG	3	<hkxhk>	hh	0	hk	272	kk	0
>ref-181799	CAGCTGTAGACGTGTCCTCCGGAAAGG	14	<hkxhk>	hh	0	hk	263	kk	8
>ref-181800	AACCTTTAAACCTTCTCTCCATCCCTA	6	<lmxll>	ll	102	lm	126		
>ref-181818	CTCGTGCACACCACTTCTCCCCGCCTT	2	<hkxhk>	hh	60	hk	143	kk	57
>ref-181818	CTCGTGCACACCACTTCTCCCCGCCTT	16	<lmxll>	ll	132	lm	136		
>ref-181833	AGTCAAAGGACTTGTTCTCCATGACAC	6	<lmxll>	ll	130	lm	139		
>ref-181858	TCCTCCCCGACACCCTCTCCCCCCTCT	12	<lmxll>	ll	208	lm	28		
>ref-181863	CAACATCATACATTCTCTCCGCCGTAC	21	<lmxll>	ll	132	lm	135		
>ref-181871	AATTCCTAGACGCAATCTCCTGTTAAA	15	<hkxhk>	hh	127	hk	109	kk	0
>ref-181871	AATTCCTAGACGCAATCTCCTGTTAAA	21	<hkxhk>	hh	0	hk	110	kk	128
>ref-181876	TATCCTCTCACTCCAGCTCCTCTTCCT	6	<nnxnp>	nn	131	np	140		
>ref-181878	TTCCTTCAAACCTGACCTCCAACCACC	2	<nnxnp>	nn	160	np	73		
>ref-181878	TTCCTTCAAACCTGACCTCCAACCACC	25	<hkxhk>	hh	87	hk	145	kk	0
>ref-181884	TCCGGCTAAACTGACCCTCCCTAGGAA	15	<hkxhk>	hh	46	hk	136	kk	65
>ref-181884	TCCGGCTAAACTGACCCTCCCTAGGAA	22	<hkxhk>	hh	46	hk	136	kk	65
>ref-181918	AGTCCTCTAACTCACTCTCCCTCTCTC	2	<nnxnp>	nn	255	np	2		
>ref-181918	AGTCCTCTAACTCACTCTCCCTCTCTC	4	<nnxnp>	nn	262	np	0		
>ref-181918	AGTCCTCTAACTCACTCTCCCTCTCTC	9	<hkxhk>	hh	67	hk	146	kk	31
>ref-181956	CTAGGATTTACCTCCGCTCCTCTCGCC	13	<lmxll>	ll	132	lm	135		
>ref-181971	CCCCACCACACTCTTTCTCCTCCTCTT	4	<lmxll>	ll	84	lm	142		
>ref-181976	AACTGAGCAACAAATTCTCCTTTCTGA	6	<hkxhk>	hh	58	hk	123	kk	53
>ref-182006	AACTTCATCACTTTCCCTCCTTTCCCC	3	<lmxll>	ll	228	lm	0		
>ref-182083	GGGCCGGAGACCAGTGCTCCAACCACT	3	<hkxhk>	hh	4	hk	269	kk	0
>ref-182083	GGGCCGGAGACCAGTGCTCCAACCACT	4	<hkxhk>	hh	0	hk	273	kk	0
>ref-182105	GATGGCTGTACTCCTTCTCCTCCACCT	6	<nnxnp>	nn	132	np	139		
>ref-182120	TCCTTCGTCACCCTCTCTCCCACTTCC	6	<nnxnp>	nn	267	np	0		
>ref-182151	AGCTCCAGCACCCTCTCTCCCTTTCCC	16	<nnxnp>	nn	5	np	264		
>ref-182169	TATGCTTATACCTAGACTCCAGCCGAT	5	<nnxnp>	nn	121	np	139		
>ref-182195	CCCTCTACAACTTCCTCTCCCCTTCCT	7	<nnxnp>	nn	118	np	135		
>ref-182196	CATACAGTTACTCTGTCTCCTCCTTTT	6	<hkxhk>	hh	58	hk	152	kk	50
>ref-182223	TCTTTTCCCACCCCCTCTCCTATTCCA	4	<nnxnp>	nn	124	np	104		
>ref-182237	CCGTCTCCCACCGTCTCTCCCTCTCTT	2	<lmxll>	ll	114	lm	150		
>ref-182237	CCGTCTCCCACCGTCTCTCCCTCTCTT	14	<lmxll>	ll	114	lm	150		
>ref-182244	CACGCTCAGACAGACACTCCTCCGATA	1	<nnxnp>	nn	123	np	116		
>ref-182261	TTTCCCCTAACCTCTTCTCCTCCCTCT	16	<nnxnp>	nn	268	np	1		

>ref-182261	TTTCCCCTAACCTCTTCTCCTCCCTCT	25	<nnxnp>	nn	268	np	1		
>ref-182263	CGTGAGCTGACCCGCTCTCCCTACCTC	21	<lmxll>	ll	124	lm	137		
>ref-182264	TGACAGACCACAAGCGCTCCATCTCAA	21	<lmxll>	ll	128	lm	143		
>ref-182264	TGACAGACCACAAGCGCTCCATCTCAA	27	<lmxll>	ll	125	lm	141		
>ref-182277	CGAATTTATACCAACTCTCCTGGCATT	14	<nnxnp>	nn	115	np	140		
>ref-182280	TCCCTCATTACTGATCCTCCACTTTCC	16	<nnxnp>	nn	122	np	140		
>ref-182287	TGCTAGTCTACCCTCTCTCCTCGATCT	15	<lmxll>	ll	110	lm	132		
>ref-182289	TTCCCTCCCACCGTTTCTCCAAGCCCT	2	<lmxll>	ll	170	lm	63		
>ref-182304	TTGATAATAACTGCTACTCCCATATCC	23	<nnxnp>	nn	226	np	0		
>ref-182313	CTCTCTCTGACTGTTGCTCCTCCCCCC	7	<lmxll>	ll	116	lm	150		
>ref-182313	CTCTCTCTGACTGTTGCTCCTCCCCCC	22	<lmxll>	ll	115	lm	151		
>ref-182314	ATTCTAAATACAGATACTCCAATCTCC	2	<nnxnp>	nn	16	np	250		
>ref-182314	ATTCTAAATACAGATACTCCAATCTCC	9	<hkxhk>	hh	0	hk	258	kk	9
>ref-182314	ATTCTAAATACAGATACTCCAATCTCC	12	<hkxhk>	hh	198	hk	55	kk	0
>ref-182314	ATTCTAAATACAGATACTCCAATCTCC	15	<hkxhk>	hh	0	hk	268	kk	4
>ref-182314	ATTCTAAATACAGATACTCCAATCTCC	24	<hkxhk>	hh	0	hk	95	kk	153
>ref-182314	ATTCTAAATACAGATACTCCAATCTCC	27	<hkxhk>	hh	1	hk	271	kk	0
>ref-182317	GAACAACAAACACCCTCTCCCTCCCTA	5	<nnxnp>	nn	113	np	117		
>ref-182358	AAAGACCCAACGAAATCTCCTTCAGAA	16	<hkxhk>	hh	0	hk	256	kk	9
>ref-182371	TTACCTATTACCCATTCTCCCCATCCC	3	<hkxhk>	hh	5	hk	193	kk	42
>ref-182371	TTACCTATTACCCATTCTCCCCATCCC	25	<hkxhk>	hh	5	hk	193	kk	42
>ref-182407	CCTCCTCCAACCCACTCTCCTACTCCT	27	<lmxll>	ll	121	lm	144		
>ref-182435	TCCTCCCATACCCTACCTCCCTCTTCT	25	<lmxll>	ll	148	lm	88		
>ref-182435	TCCTCCCATACCCTACCTCCCTCTTCT	27	<lmxll>	ll	152	lm	89		
>ref-182478	CCTATTTTCACACCTTCTCCTTCTGGA	8	<nnxnp>	nn	115	np	134		
>ref-182515	CTATCTATCACACACTCTCCCTCCTAT	25	<hkxhk>	hh	0	hk	233	kk	13
>ref-182528	AATGTTCCCACGCGAACTCCGATTGAA	6	<lmxll>	ll	138	lm	115		
>ref-182558	CTACAGGAGACAATTGCTCCTGCGTGT	1	<nnxnp>	nn	142	np	123		
>ref-182564	TATCCTACAACTCCCCCTCCTCACTCC	22	<lmxll>	ll	107	lm	114		
>ref-182575	CCCCCCTCTACTCTTTCTCCCCCCTCG	26	<hkxhk>	hh	85	hk	155	kk	0
>ref-182575	CCCCCCTCTACTCTTTCTCCCCCCTCG	27	<nnxnp>	nn	182	np	60		
>ref-182579	ATTAGTCGGACTGCATCTCCCAGTCTC	21	<nnxnp>	nn	133	np	135		
>ref-182588	GCCCTCCTCACTTTTCCTCCTCCTGCT	8	<nnxnp>	nn	133	np	134		
>ref-182605	GTGAAAACTACAGCTGCTCCTGCTATG	9	<lmxll>	ll	118	lm	116		
>ref-182617	CCGCTCACCACGCAAGCTCCCGCCCCC	23	<nnxnp>	nn	126	np	141		
>ref-182621	TTTGTCGTTACCTTATCTCCCTACTCT	4	<lmxll>	ll	129	lm	90		
>ref-182621	TTTGTCGTTACCTTATCTCCCTACTCT	8	<lmxll>	ll	129	lm	90		
>ref-182657	CGCACTTCAACACGCGCTCCCGCTGAG	16	<lmxll>	ll	129	lm	128		

>ref-182658	ACGTGACTAACATTCACTCCACTATTT	15	<nnxnp>	nn	117	np	111		
>ref-182668	TACACCCACACACCAGCTCCCACCCAT	8	<nnxnp>	nn	113	np	143		
>ref-182668	TACACCCACACACCAGCTCCCACCCAT	16	<nnxnp>	nn	114	np	142		
>ref-182678	ATTCAGATAACAATCACTCCTTCTTTC	25	<hkxhk>	hh	57	hk	127	kk	39
>ref-182679	TGAGAAATGACAGCCTCTCCTTTCGTT	9	<hkxhk>	hh	55	hk	141	kk	48
>ref-182679	TGAGAAATGACAGCCTCTCCTTTCGTT	13	<hkxhk>	hh	56	hk	139	kk	48
>ref-182699	ATAATATCCACTGCCTCTCCATTCCTC	7	<nnxnp>	nn	96	np	136		
>ref-182699	ATAATATCCACTGCCTCTCCATTCCTC	8	<nnxnp>	nn	96	np	136		
>ref-182716	AGACGAGTGACCAACTCTCCTAATTCA	14	<nnxnp>	nn	208	np	53		
>ref-182719	CTCCTTTACACCCCTTCTCCCCACCTC	24	<hkxhk>	hh	16	hk	230	kk	0
>ref-182719	CTCCTTTACACCCCTTCTCCCCACCTC	26	<hkxhk>	hh	0	hk	230	kk	16
>ref-182723	CCACCACCCACTCCCCCTCCCTTCCTG	25	<hkxhk>	hh	67	hk	124	kk	44
>ref-182732	TTCCCTTCTACCTATTCTCCCCTATCT	5	<nnxnp>	nn	126	np	125		
>ref-182734	CCTCCCTCAACACGACCTCCCTCCCTC	2	<hkxhk>	hh	134	hk	123	kk	0
>ref-182734	CCTCCCTCAACACGACCTCCCTCCCTC	21	<hkxhk>	hh	1	hk	272	kk	0
>ref-182741	CCTCTCTCTACCTTCCCTCCCTCCTAC	8	<hkxhk>	hh	0	hk	273	kk	0
>ref-182741	CCTCTCTCTACCTTCCCTCCCTCCTAC	14	<hkxhk>	hh	0	hk	247	kk	17
>ref-182741	CCTCTCTCTACCTTCCCTCCCTCCTAC	25	<lmxll>	ll	89	lm	162		
>ref-182749	TACCACTCTACCCACTCTCCTCTCTCG	16	<lmxll>	ll	262	lm	0		
>ref-182778	CACTTGAACACACAACCTCCTTTCTGC	9	<lmxll>	ll	116	lm	105		
>ref-182779	GACAGACTCACTCCAACTCCCTATACT	5	<lmxll>	ll	125	lm	108		
>ref-182797	ACCCCATCTACAGTTGCTCCTCCGTTT	13	<nnxnp>	nn	118	np	114		
>ref-182806	CACAGTGATACTAGCGCTCCTGCCCTG	3	<lmxll>	ll	108	lm	153		
>ref-182823	AAATCCCCAACGATCTCTCCTCCTCCA	8	<hkxhk>	hh	3	hk	259	kk	0
>ref-182838	ATACATCCCACTCATGCTCCCTCGGCA	16	<lmxll>	ll	119	lm	139		
>ref-182845	CCTTCAAATACTTAGGCTCCCATATGT	7	<lmxll>	ll	50	lm	196		
>ref-182845	CCTTCAAATACTTAGGCTCCCATATGT	8	<lmxll>	ll	127	lm	123		
>ref-182845	CCTTCAAATACTTAGGCTCCCATATGT	22	<lmxll>	ll	94	lm	156		
>ref-182845	CCTTCAAATACTTAGGCTCCCATATGT	27	<lmxll>	ll	125	lm	121		
>ref-182848	ACCATCGGTACATCCTCTCCATCACGT	2	<nnxnp>	nn	141	np	132		
>ref-182892	CCCAGGGTTACCACAACTCCAGCATAC	27	<hkxhk>	hh	115	hk	140	kk	0
>ref-182920	TATTAAATCACTCTCTCTCCTCTTCCT	9	<lmxll>	ll	16	lm	249		
>ref-182920	TATTAAATCACTCTCTCTCCTCTTCCT	14	<lmxll>	ll	16	lm	249		
>ref-182923	GATTTGCACACGATCTCTCCTTTTATG	27	<hkxhk>	hh	0	hk	164	kk	88
>ref-182940	GTTTGCTCTACTTCCCCTCCCTCTTCA	24	<nnxnp>	nn	115	np	146		
>ref-182955	CTCTCGTGCACGTGCACTCCTTTCTTT	16	<nnxnp>	nn	270	np	0		
>ref-182968	TTAGAATAAACGGTTCCTCCTCTTGTT	5	<hkxhk>	hh	54	hk	200	kk	1
>ref-182968	TTAGAATAAACGGTTCCTCCTCTTGTT	27	<hkxhk>	hh	1	hk	200	kk	53

>ref-182987	GGCTGGGTGACTGTTGCTCCTGCTTCT	14	<hkxhk>	hh	0	hk	178	kk	75
>ref-182987	GGCTGGGTGACTGTTGCTCCTGCTTCT	27	<hkxhk>	hh	0	hk	188	kk	59
>ref-182997	ACACGTTGCACACCGTCTCCATTTATT	15	<nnxnp>	nn	104	np	139		
>ref-183020	TATCGTGGAACTGCCACTCCTGTGTAA	24	<hkxhk>	hh	0	hk	212	kk	49
>ref-183032	CTACCACTCACCACCACTCCTACCACT	15	<lmxll>	ll	180	lm	71		
>ref-183071	CTTCCGCTGACAACACCTCCTCGGCTG	2	<lmxll>	ll	62	lm	197		
>ref-183071	CTTCCGCTGACAACACCTCCTCGGCTG	6	<hkxhk>	hh	0	hk	235	kk	29
>ref-183071	CTTCCGCTGACAACACCTCCTCGGCTG	16	<hkxhk>	hh	0	hk	273	kk	0
>ref-183081	ACGTGATGGACGAACACTCCCACGAAC	12	<nnxnp>	nn	268	np	0		
>ref-183084	CCTTCCTTCACTCGTTCTCCTATCCCT	14	<nnxnp>	nn	251	np	1		
>ref-183084	CCTTCCTTCACTCGTTCTCCTATCCCT	15	<nnxnp>	nn	257	np	1		
>ref-183089	TTTTAATTCACTCCTTCTCCTCCTCTC	1	<hkxhk>	hh	0	hk	260	kk	1
>ref-183089	TTTTAATTCACTCCTTCTCCTCCTCTC	3	<hkxhk>	hh	1	hk	260	kk	1
>ref-183111	TCACCTTTCACTCTCTCTCCTTCGAGG	3	<nnxnp>	nn	122	np	140		
>ref-183176	TCATCAAGCACTTTCACTCCTCTATAA	2	<lmxll>	ll	145	lm	117		
>ref-183201	CGGCCGAGCACTGGGTCTCCGTCCAAT	23	<lmxll>	ll	124	lm	149		
>ref-183209	TTCCACCTCACTCATTCTCCTCTCCCT	4	<nnxnp>	nn	265	np	0		
>ref-183222	TCATCCCCCACCCCGTCTCCCTCCTCT	25	<hkxhk>	hh	0	hk	105	kk	131
>ref-183237	TTACCCCTAACCCACTCTCCCTCCCTC	14	<hkxhk>	hh	1	hk	222	kk	19
>ref-183244	CCTCTGACTACTCCCTCTCCTCCGTCG	3	<nnxnp>	nn	120	np	140		
>ref-183266	CACAGATACACCTCACCTCCCTTCACA	9	<lmxll>	ll	120	lm	113		
>ref-183285	GCCAAACTCACAGTCCCTCCAGGCATT	16	<hkxhk>	hh	49	hk	141	kk	48
>ref-183312	ACCATTCTCACCATTTCTCCCAATCAT	15	<hkxhk>	hh	0	hk	268	kk	4
>ref-183312	ACCATTCTCACCATTTCTCCCAATCAT	22	<hkxhk>	hh	19	hk	241	kk	0
>ref-183321	TGCACAGACACGCCCGCTCCATTGTCT	8	<lmxll>	ll	140	lm	131		
>ref-183322	CTCCCATCCACTTCACCTCCTCCATCT	22	<hkxhk>	hh	68	hk	144	kk	46
>ref-183351	GGTAATTGTACCATTCCTCCTTACATC	1	<lmxll>	ll	115	lm	122		
>ref-183374	CTCTTCCTCACCCTCTCTCCCCTAACC	24	<nnxnp>	nn	102	np	132		
>ref-183374	CTCTTCCTCACCCTCTCTCCCCTAACC	25	<nnxnp>	nn	101	np	122		
>ref-183377	TGACCTTTGACCTGACCTCCCCCGCTG	25	<nnxnp>	nn	132	np	136		
>ref-183384	CATGGGTTCACCGCCTCTCCTCTCTCT	6	<lmxll>	ll	135	lm	133		
>ref-183388	CCCTCTCCTACTGCCTCTCCTCCCTTC	1	<nnxnp>	nn	8	np	251		
>ref-183388	CCCTCTCCTACTGCCTCTCCTCCCTTC	6	<nnxnp>	nn	8	np	250		
>ref-183394	TGTCACGCCACCTTTTCTCCTTTTCAT	9	<lmxll>	ll	104	lm	119		
>ref-183442	CACCATCCCACCCTTGCTCCTTCTTCA	4	<nnxnp>	nn	120	np	146		
>ref-183442	CACCATCCCACCCTTGCTCCTTCTTCA	6	<nnxnp>	nn	120	np	146		
>ref-183446	TGTTGATTTACATAATCTCCAGGCATA	2	<lmxll>	ll	125	lm	102		
>ref-183464	TCTTCATTTACTCTACCTCCTTTTCCT	12	<hkxhk>	hh	0	hk	247	kk	7

>ref-183464	TCTTCATTTACTCTACCTCCTTTTCCT	14	<hkxhk>	hh	0	hk	244	kk	9
>ref-183464	TCTTCATTTACTCTACCTCCTTTTCCT	26	<lmxll>	ll	137	lm	104		
>ref-183476	CTCTCTCTAACTGCCTCTCCCTGCTTC	2	<lmxll>	ll	145	lm	127		
>ref-183476	CTCTCTCTAACTGCCTCTCCCTGCTTC	15	<hkxhk>	hh	66	hk	150	kk	51
>ref-183488	TCCTCCTCCACTTCTCCTCCACTCTCT	1	<hkxhk>	hh	0	hk	199	kk	52
>ref-183488	TCCTCCTCCACTTCTCCTCCACTCTCT	12	<hkxhk>	hh	0	hk	202	kk	45
>ref-183497	GTTGGGGGAACGGCAACTCCCTATAAG	14	<nnxnp>	nn	237	np	0		
>ref-183526	CTATTCCCCACTCTAACTCCTACTCAT	15	<hkxhk>	hh	57	hk	128	kk	60
>ref-183544	CTCCGTCTCACTGACTCTCCCTCTATT	9	<nnxnp>	nn	117	np	145		
>ref-183554	CTGAATCGAACTCCCGCTCCCGCTCAC	12	<nnxnp>	nn	114	np	144		
>ref-183554	CTGAATCGAACTCCCGCTCCCGCTCAC	15	<nnxnp>	nn	115	np	144		
>ref-183566	ACGAAGATGACTCGGTCTCCTTCGAAG	2	<nnxnp>	nn	120	np	131		
>ref-183566	ACGAAGATGACTCGGTCTCCTTCGAAG	22	<lmxll>	ll	123	lm	131		
>ref-183570	TATTTTATCACTAATGCTCCATATTAC	8	<nnxnp>	nn	164	np	76		
>ref-183570	TATTTTATCACTAATGCTCCATATTAC	9	<lmxll>	ll	168	lm	89		
>ref-183670	GCTATCCTCACTATCCCTCCTTGTTTC	4	<lmxll>	ll	255	lm	0		
>ref-183670	GCTATCCTCACTATCCCTCCTTGTTTC	9	<lmxll>	ll	124	lm	117		
>ref-183698	CCTCTGCACACAGTTACTCCTTTCACA	2	<nnxnp>	nn	132	np	127		
>ref-183705	TTTCCTAACACTTCTTCTCCCTCCACA	5	<nnxnp>	nn	266	np	0		
>ref-183729	ACCTGCCTCACTCTGTCTCCATCTCCA	22	<lmxll>	ll	64	lm	207		
>ref-183735	ACCTACCCTACCCATCCTCCCTTCCCT	14	<hkxhk>	hh	16	hk	250	kk	0
>ref-183735	ACCTACCCTACCCATCCTCCCTTCCCT	15	<hkxhk>	hh	0	hk	249	kk	17
>ref-183748	CATCCGTCTACTTCTCCTCCCTTTCGT	2	<nnxnp>	nn	10	np	262		
>ref-183748	CATCCGTCTACTTCTCCTCCCTTTCGT	21	<nnxnp>	nn	10	np	262		
>ref-183753	CTCGCCTTTACTCTCCCTCCCTCGTTC	7	<nnxnp>	nn	257	np	0		
>ref-183753	CTCGCCTTTACTCTCCCTCCCTCGTTC	24	<nnxnp>	nn	244	np	4		
>ref-183777	TCGCAATATACATTCTCTCCTTATTCA	22	<hkxhk>	hh	55	hk	121	kk	59
>ref-183782	TCTCTTCTTACGCGCACTCCTTTCTCT	14	<hkxhk>	hh	64	hk	147	kk	53
>ref-183784	ATACACCTTACATATGCTCCTATGAAT	23	<nnxnp>	nn	266	np	0		
>ref-183784	ATACACCTTACATATGCTCCTATGAAT	25	<nnxnp>	nn	261	np	0		
>ref-183800	TGCGTTGCCACCGCTCCTCCGCGCCCC	3	<lmxll>	ll	136	lm	136		
>ref-183845	TTATTAATTACAAGATCTCCCAGTGTC	26	<nnxnp>	nn	118	np	129		
>ref-183848	TAGCTTATAACCTTCTCTCCTTCGGCA	26	<nnxnp>	nn	131	np	135		
>ref-183851	CTTAACTGCACTCTTCCTCCTCCTGCT	4	<hkxhk>	hh	0	hk	273	kk	0
>ref-183851	CTTAACTGCACTCTTCCTCCTCCTGCT	25	<hkxhk>	hh	0	hk	273	kk	0
>ref-183854	ACAACATCCACCCTGTCTCCAACGACC	3	<nnxnp>	nn	106	np	152		
>ref-183854	ACAACATCCACCCTGTCTCCAACGACC	27	<nnxnp>	nn	105	np	152		
>ref-183901	ATCGACTGAACGGGTTCTCCTGTGTAT	24	<lmxll>	ll	141	lm	111		

>ref-183901	ATCGACTGAACGGGTTCTCCTGTGTAT	26	<nnxnp>	nn	94	np	152		
>ref-183928	CATTCTCCCACTCTCTCTCCATCCATC	2	<lmxll>	ll	4	lm	266		
>ref-183928	CATTCTCCCACTCTCTCTCCATCCATC	8	<lmxll>	ll	4	lm	266		
>ref-183943	CTACCTTCCACTCTTCCTCCTCCACAC	5	<lmxll>	ll	132	lm	135		
>ref-183944	TTGCTAGTGACTGTTGCTCCTCGGTAA	2	<lmxll>	ll	100	lm	155		
>ref-183953	ATGGGTTACACACTAACTCCCCATCTC	15	<lmxll>	ll	115	lm	144		
>ref-183967	TTCCCCCCAACCTCCCCTCCACTATCT	2	<hkxhk>	hh	46	hk	145	kk	55
>ref-183973	TTACCCGACACTGCTCCTCCTTCCTTC	13	<nnxnp>	nn	142	np	127		
>ref-183974	CCCCCTCTAACCTTAACTCCTCCTCTT	5	<lmxll>	ll	115	lm	132		
>ref-183990	ATAGAAAAAACCTTTCCTCCACGATGC	26	<lmxll>	ll	152	lm	85		
>ref-184060	TTTTCGTCAACCCTCTCTCCACTCCCC	9	<lmxll>	ll	120	lm	134		
>ref-184060	TTTTCGTCAACCCTCTCTCCACTCCCC	12	<lmxll>	ll	120	lm	134		
>ref-184062	TACCGATACACATTTCCTCCATCTCTC	2	<lmxll>	ll	113	lm	148		
>ref-184077	AAAATATCCACCGAATCTCCTCTCAAT	13	<nnxnp>	nn	135	np	114		
>ref-184089	CGGAAGGTCACCCGCCCTCCCTCTTCC	2	<nnxnp>	nn	121	np	140		
>ref-184109	ACAAAACGAACACAGGCTCCCACAGAA	9	<lmxll>	ll	130	lm	137		
>ref-184134	GCTGAATGAACCAAAACTCCATTCATG	12	<lmxll>	ll	119	lm	140		
>ref-184193	CCAAATCTCACTCTCTCTCCCTGTCTG	6	<lmxll>	ll	116	lm	125		
>ref-184194	TATTACGGTACTCGCCCTCCCTGCGTT	24	<lmxll>	ll	111	lm	114		
>ref-184197	GTACGAACTACCCTTTCTCCGTCCCCC	9	<lmxll>	ll	264	lm	1		
>ref-184218	CGCTCAAGGACCCCCCCTCCTCCTCCT	5	<lmxll>	ll	133	lm	120		
>ref-184231	TTCCCTGAAACACTGTCTCCTTCACGC	24	<hkxhk>	hh	47	hk	186	kk	0
>ref-184231	TTCCCTGAAACACTGTCTCCTTCACGC	25	<hkxhk>	hh	2	hk	259	kk	2
>ref-184236	CCCGCCCACACCTTTCCTCCAACTTCA	26	<nnxnp>	nn	107	np	114		
>ref-184244	GCAGTGGGTACTTGGCCTCCTGTCTAG	1	<nnxnp>	nn	268	np	0		
>ref-184248	GGTGATAACACTTACTCTCCACGATAA	27	<nnxnp>	nn	269	np	0		
>ref-184274	ATGTCTCTCACCTGAACTCCTACTCTC	2	<lmxll>	ll	0	lm	271		
>ref-184274	ATGTCTCTCACCTGAACTCCTACTCTC	3	<lmxll>	ll	0	lm	271		
>ref-184282	CCTTCCTCCACGTCTCCTCCTCCCTGT	2	<hkxhk>	hh	0	hk	171	kk	94
>ref-184283	ATGCCTCCGACGTTAACTCCATCCGCG	22	<hkxhk>	hh	2	hk	256	kk	4
>ref-184289	TACAATACCACCCTCCCTCCCTCTCTC	16	<nnxnp>	nn	140	np	117		
>ref-184289	TACAATACCACCCTCCCTCCCTCTCTC	24	<nnxnp>	nn	142	np	117		
>ref-184296	ACCTCAAGTACTCTGACTCCAGCACTT	9	<nnxnp>	nn	266	np	0		
>ref-184313	CTTTCACCCACCTTCCCTCCGCTTCTC	23	<lmxll>	ll	133	lm	126		
>ref-184320	GAATAGGACACCGTGGCTCCATTCATT	24	<lmxll>	ll	140	lm	79		
>ref-184322	AACCGCCACACTCCACCTCCTTCGTGT	22	<lmxll>	ll	272	lm	0		
>ref-184330	ACCTCACTTACCGTTCCTCCAACATGT	5	<nnxnp>	nn	120	np	139		
>ref-184344	ACCAAGAATACACATCCTCCCTTTCTT	21	<nnxnp>	nn	116	np	115		

>ref-184345	AGGCGCAAAACGGCTTCTCCGCCACCG	26	<nnxnp>	nn	270	np	0		
>ref-184346	GCTAGAGTGACGCGAGCTCCTGAGAGT	25	<hkxhk>	hh	56	hk	151	kk	61
>ref-184365	CAGACCATCACCCTCGCTCCCTCACTC	16	<nnxnp>	nn	122	np	134		
>ref-184368	AAAAGAGCAACCCGCCCTCCCCAAGCC	23	<lmxll>	ll	121	lm	143		
>ref-184368	AAAAGAGCAACCCGCCCTCCCCAAGCC	27	<lmxll>	ll	121	lm	143		
>ref-184370	CCACCCTCCACCGACACTCCGCCCCCT	22	<hkxhk>	hh	3	hk	270	kk	0
>ref-184370	CCACCCTCCACCGACACTCCGCCCCCT	27	<hkxhk>	hh	0	hk	270	kk	3
>ref-184372	CTCCTTCTTACTCTCTCTCCTTATTGC	6	<nnxnp>	nn	118	np	134		
>ref-184401	CTGTCCCTCACTCTCTCTCCCCATTTT	6	<nnxnp>	nn	256	np	3		
>ref-184402	CTGCTGTTCACCCTTCCTCCTCCTCCT	5	<hkxhk>	hh	0	hk	268	kk	5
>ref-184402	CTGCTGTTCACCCTTCCTCCTCCTCCT	12	<hkxhk>	hh	0	hk	268	kk	5
>ref-184403	ACCCAGAACACCCTCTCTCCCTCCCCT	23	<lmxll>	ll	110	lm	153		
>ref-184403	ACCCAGAACACCCTCTCTCCCTCCCCT	24	<lmxll>	ll	108	lm	152		
>ref-184408	ATCTTGCAAACACTCACTCCCTTTGAG	8	<lmxll>	ll	113	lm	147		
>ref-184408	ATCTTGCAAACACTCACTCCCTTTGAG	9	<lmxll>	ll	113	lm	147		
>ref-184422	AACATAGCCACGACGCCTCCTAGGAAA	6	<nnxnp>	nn	101	np	156		
>ref-184422	AACATAGCCACGACGCCTCCTAGGAAA	21	<nnxnp>	nn	101	np	156		
>ref-184428	CACTCCACCACGCGTACTCCAGCATCA	4	<nnxnp>	nn	137	np	118		
>ref-184433	AAAGCACTCACAGGCTCTCCCCCTCCA	5	<nnxnp>	nn	132	np	137		
>ref-184459	TTCTCTCTCACCTCACCTCCTTTTGTC	1	<nnxnp>	nn	131	np	133		
>ref-184466	TCCCTCAGCACTCTTCCTCCTTCTACA	16	<lmxll>	ll	87	lm	145		
>ref-184466	TCCCTCAGCACTCTTCCTCCTTCTACA	26	<lmxll>	ll	88	lm	145		
>ref-184485	TAACGCTTTACCCCCCCTCCTCCATCT	21	<nnxnp>	nn	94	np	131		
>ref-184507	GTCCCTCCAACTCTCCCTCCCATGACA	9	<hkxhk>	hh	65	hk	137	kk	62
>ref-184518	AACAGGCACACGCTTCCTCCCTCGCCT	5	<hkxhk>	hh	61	hk	147	kk	49
>ref-184525	CTATCACCAACCACTTCTCCCTTCCTT	5	<hkxhk>	hh	0	hk	268	kk	0
>ref-184525	CTATCACCAACCACTTCTCCCTTCCTT	13	<hkxhk>	hh	0	hk	268	kk	0
>ref-184537	CCTACCCCAACCCTCTCTCCCTTCTAT	24	<lmxll>	ll	124	lm	95		
>ref-184543	GACGGAGGAACACTCACTCCCTCTCTC	8	<nnxnp>	nn	126	np	141		
>ref-184543	GACGGAGGAACACTCACTCCCTCTCTC	16	<nnxnp>	nn	127	np	141		
>ref-184573	AGGATCTCCACCCCCCCTCCTCCCTCC	12	<lmxll>	ll	104	lm	143		
>ref-184573	AGGATCTCCACCCCCCCTCCTCCCTCC	27	<lmxll>	ll	259	lm	0		
>ref-184575	AAGAGGAGAACCAACACTCCCACACCC	26	<nnxnp>	nn	128	np	126		
>ref-184611	ATCCATCTTACCTTAACTCCCTCCCTC	3	<hkxhk>	hh	80	hk	137	kk	40
>ref-184631	ATTATCTTCACTATTTCTCCTCCCATT	26	<lmxll>	ll	111	lm	110		
>ref-184635	TCTTACGGGACTTTCTCTCCTTTTCTC	5	<hkxhk>	hh	77	hk	100	kk	46
>ref-184639	TACTAGACGACTACCACTCCGACCACT	16	<lmxll>	ll	153	lm	115		
>ref-184685	GCCCTTCTCACCCTCTCTCCTTTCCCT	2	<nnxnp>	nn	262	np	0		

>ref-184687	ACCATGGGCACTATTCCTCCTACACAA	6	<nnxnp>	nn	120	np	116		
>ref-184708	TGCCTTTCCACCGATGCTCCTTTAATA	5	<lmxll>	ll	114	lm	127		
>ref-184708	TGCCTTTCCACCGATGCTCCTTTAATA	21	<hkxhk>	hh	48	hk	136	kk	57
>ref-184710	TACTCCCTTACTCCGCCTCCCTTTTCT	14	<lmxll>	ll	119	lm	141		
>ref-184710	TACTCCCTTACTCCGCCTCCCTTTTCT	21	<lmxll>	ll	118	lm	142		
>ref-184719	CTCGGGCCCACACTTTCTCCTCCACCT	1	<hkxhk>	hh	3	hk	262	kk	0
>ref-184719	CTCGGGCCCACACTTTCTCCTCCACCT	23	<hkxhk>	hh	3	hk	262	kk	0
>ref-184741	TCACACTGTACACGACCTCCTTTCCTC	3	<nnxnp>	nn	100	np	144		
>ref-184747	CTGCCACTCACCCTACCTCCCAAGCTC	7	<hkxhk>	hh	0	hk	267	kk	2
>ref-184747	CTGCCACTCACCCTACCTCCCAAGCTC	14	<hkxhk>	hh	0	hk	273	kk	0
>ref-184751	CTTACTTTCACCCTTTCTCCATCCCTA	23	<lmxll>	ll	130	lm	115		
>ref-184772	ACATGGCTGACAGTTCCTCCTCGATCT	5	<nnxnp>	nn	129	np	136		
>ref-184772	ACATGGCTGACAGTTCCTCCTCGATCT	23	<nnxnp>	nn	128	np	137		
>ref-184796	TTACCGATGACTTCCACTCCAATGCCC	12	<lmxll>	ll	130	lm	140		
>ref-184819	TGCTCGCTCACCCTCGCTCCCCGTGCT	26	<lmxll>	ll	127	lm	143		
>ref-184834	GCGCTGGGCACGAACTCTCCAAACCTG	25	<hkxhk>	hh	0	hk	272	kk	0
>ref-184836	CCAACAACAACCCCCTCTCCCCCTCCA	2	<nnxnp>	nn	122	np	127		
>ref-184843	ATCTTATCTACGCCACCTCCTCTAACT	5	<lmxll>	ll	113	lm	126		
>ref-184849	TTCTTCTCCACCTTTCCTCCCCCCCTT	2	<hkxhk>	hh	0	hk	223	kk	34
>ref-184849	TTCTTCTCCACCTTTCCTCCCCCCCTT	22	<lmxll>	ll	146	lm	122		
>ref-184849	TTCTTCTCCACCTTTCCTCCCCCCCTT	27	<hkxhk>	hh	0	hk	222	kk	36
>ref-184852	ATCCCGTCCACCCCTTCTCCTAACCCT	6	<hkxhk>	hh	0	hk	273	kk	0
>ref-184852	ATCCCGTCCACCCCTTCTCCTAACCCT	9	<hkxhk>	hh	0	hk	273	kk	0
>ref-184853	ATTTACCATACACCCCCTCCTTCTACC	6	<nnxnp>	nn	253	np	0		
>ref-184866	TCCGTTTCAACGTCTCCTCCTCTTCTT	1	<lmxll>	ll	138	lm	126		
>ref-184866	TCCGTTTCAACGTCTCCTCCTCTTCTT	24	<lmxll>	ll	138	lm	126		
>ref-184897	ATTGTATGCACCACTACTCCGCCCACA	4	<nnxnp>	nn	125	np	116		
>ref-184897	ATTGTATGCACCACTACTCCGCCCACA	27	<nnxnp>	nn	125	np	115		
>ref-184902	CCGTCAAACACAAGATCTCCAACTTCT	2	<hkxhk>	hh	21	hk	243	kk	0
>ref-184902	CCGTCAAACACAAGATCTCCAACTTCT	8	<hkxhk>	hh	20	hk	243	kk	0
>ref-184949	CATCTTCTTACCTCTTCTCCTCTCCTA	25	<hkxhk>	hh	59	hk	138	kk	58
>ref-184957	GTGTGTGCAACTGGCTCTCCTTCCGTA	3	<lmxll>	ll	132	lm	131		
>ref-184968	TCTTCTCCAACATTTCCTCCCTACGTC	2	<nnxnp>	nn	148	np	114		
>ref-184982	CTCTCCCCTACCTTTTCTCCCTCTTCC	8	<hkxhk>	hh	0	hk	269	kk	2
>ref-184982	CTCTCCCCTACCTTTTCTCCCTCTTCC	21	<hkxhk>	hh	2	hk	269	kk	0
>ref-184982	CTCTCCCCTACCTTTTCTCCCTCTTCC	22	<hkxhk>	hh	0	hk	268	kk	2
>ref-184982	CTCTCCCCTACCTTTTCTCCCTCTTCC	27	<hkxhk>	hh	3	hk	268	kk	0
>ref-185009	TCCTTTATTACTCCATCTCCTTCTCCT	16	<lmxll>	ll	133	lm	126		

>ref-185040	TCATTTTTCACTCTCACTCCCTCTGCC	14	<lmxll>	ll	128	lm	136		
>ref-185078	GCATTTTGTACTTTGCCTCCTGACGGG	22	<nnxnp>	nn	127	np	124		
>ref-185088	CACTCACCCACCTGCCCTCCCTCCCCT	2	<lmxll>	ll	111	lm	118		
>ref-185088	CACTCACCCACCTGCCCTCCCTCCCCT	6	<lmxll>	ll	112	lm	112		
>ref-185094	GGCTCATCGACACGCGCTCCTTTTTTC	2	<nnxnp>	nn	268	np	0		
>ref-185094	GGCTCATCGACACGCGCTCCTTTTTTC	13	<nnxnp>	nn	138	np	123		
>ref-185118	TCACCATCAACTCCACCTCCATCTCCT	1	<hkxhk>	hh	3	hk	251	kk	3
>ref-185118	TCACCATCAACTCCACCTCCATCTCCT	3	<nnxnp>	nn	259	np	1		
>ref-185118	TCACCATCAACTCCACCTCCATCTCCT	24	<hkxhk>	hh	3	hk	250	kk	4
>ref-185118	TCACCATCAACTCCACCTCCATCTCCT	27	<nnxnp>	nn	262	np	1		
>ref-185132	AGTGTATGTACGTCCACTCCGCGCTTC	5	<lmxll>	ll	124	lm	147		
>ref-185164	CTTCTTCTTACTCTCTCTCCTTTCATT	15	<nnxnp>	nn	129	np	118		
>ref-185173	AGTCCGTCCACACGTGCTCCCAAATAG	26	<nnxnp>	nn	96	np	131		
>ref-185200	TCACTCCTTACTTCCTCTCCTTTCTTC	4	<nnxnp>	nn	53	np	207		
>ref-185200	TCACTCCTTACTTCCTCTCCTTTCTTC	22	<hkxhk>	hh	0	hk	258	kk	12
>ref-185214	CCTACCCCCACGCCACCTCCCACCTCC	21	<nnxnp>	nn	145	np	83		
>ref-185214	CCTACCCCCACGCCACCTCCCACCTCC	25	<nnxnp>	nn	144	np	83		
>ref-185242	TGTTATCCCACATTGTCTCCATTCTCT	22	<hkxhk>	hh	54	hk	141	kk	67
>ref-185263	CCTCTTCCTACCTTCTCTCCCGTTAAA	12	<nnxnp>	nn	117	np	134		
>ref-185272	GTCACAGATACCAGAGCTCCAATAGAA	26	<lmxll>	ll	25	lm	214		
>ref-185306	CCTCTAAACACTACTCCTCCTCCTCCT	25	<nnxnp>	nn	148	np	101		
>ref-185319	ATCCGCTCGACCAACGCTCCCACGATA	2	<hkxhk>	hh	0	hk	265	kk	4
>ref-185319	ATCCGCTCGACCAACGCTCCCACGATA	22	<hkxhk>	hh	2	hk	271	kk	0
>ref-185323	TGATGACACACACATGCTCCTGCAACA	5	<lmxll>	ll	138	lm	127		
>ref-185352	TATTCTTATACTTATCCTCCTCATCAT	4	<lmxll>	ll	101	lm	127		
>ref-185368	CCCTAGACCACACCATCTCCTGCCAGA	5	<hkxhk>	hh	50	hk	216	kk	0
>ref-185406	CGACTGTGCACGATTGCTCCCTCTCTC	21	<nnxnp>	nn	270	np	0		
>ref-185432	TCCTCCAACACTCACCCTCCTCCTCCT	2	<hkxhk>	hh	4	hk	267	kk	0
>ref-185432	TCCTCCAACACTCACCCTCCTCCTCCT	27	<hkxhk>	hh	0	hk	267	kk	4
>ref-185438	TTCCTTCACACCCTCACTCCTCCTATC	1	<hkxhk>	hh	0	hk	266	kk	2
>ref-185441	TTTGCACGTACACACGCTCCATATACA	16	<lmxll>	ll	121	lm	137		
>ref-185457	CCACGTGTAACTGTGCCTCCGGCTTGT	9	<lmxll>	ll	129	lm	141		
>ref-185477	TTCTGACTCACTCCAGCTCCATGCATA	14	<hkxhk>	hh	61	hk	143	kk	52
>ref-185480	GAGAGATCAACGTCGTCTCCACACGTG	16	<lmxll>	ll	121	lm	149		
>ref-185491	GTTGCCAAAACCTATCCTCCGTTGGCG	9	<lmxll>	ll	134	lm	130		
>ref-185502	TGTTAAATGACTCTCTCTCCTTCTCTC	14	<lmxll>	ll	133	lm	133		
>ref-185528	GGTCACTAGACTCCTGCTCCTTCTCCT	8	<lmxll>	ll	140	lm	125		
>ref-185528	GGTCACTAGACTCCTGCTCCTTCTCCT	16	<lmxll>	ll	139	lm	125		

>ref-185534	GTCCCAATCACAAATCCTCCAGTATAG	25	<lmxll>	ll	110	lm	122		
>ref-185551	TCCAATACCACTTTCTCTCCCTTCTCC	15	<nnxnp>	nn	124	np	141		
>ref-185553	TCTTCCCCCACCGCCTCTCCTTTTCTC	3	<hkxhk>	hh	48	hk	129	kk	61
>ref-185594	GCTAGGCCTACACTGGCTCCGTCGTCT	21	<nnxnp>	nn	142	np	131		
>ref-185606	AAGAACAAGACACTGGCTCCGCGCTCT	21	<lmxll>	ll	113	lm	154		
>ref-185617	TTCACTTTTACTGACTCTCCATCCCTC	25	<nnxnp>	nn	121	np	134		
>ref-185626	CTCATTCTCACTCCCACTCCTCCCCCT	5	<nnxnp>	nn	241	np	9		
>ref-185626	CTCATTCTCACTCCCACTCCTCCCCCT	12	<hkxhk>	hh	1	hk	252	kk	1
>ref-185626	CTCATTCTCACTCCCACTCCTCCCCCT	16	<hkxhk>	hh	2	hk	253	kk	0
>ref-185626	CTCATTCTCACTCCCACTCCTCCCCCT	22	<nnxnp>	nn	257	np	1		
>ref-185629	AATTACATTACCGTGTCTCCATTCATC	9	<nnxnp>	nn	123	np	126		
>ref-185656	CTCTTCTCCACGTCCTCTCCACTTCTC	12	<hkxhk>	hh	59	hk	134	kk	70
>ref-185673	CAGCCATGCACCTGCTCTCCGTCGAAG	3	<nnxnp>	nn	124	np	118		
>ref-185673	CAGCCATGCACCTGCTCTCCGTCGAAG	9	<lmxll>	ll	252	lm	0		
>ref-185705	ACCCTTGGCACTCCCCCTCCCCCTCCT	2	<hkxhk>	hh	0	hk	242	kk	22
>ref-185705	ACCCTTGGCACTCCCCCTCCCCCTCCT	14	<lmxll>	ll	11	lm	254		
>ref-185705	ACCCTTGGCACTCCCCCTCCCCCTCCT	22	<hkxhk>	hh	24	hk	242	kk	0
>ref-185715	ATCAATGAAACGTCACCTCCCTCCCTC	8	<lmxll>	ll	127	lm	127		
>ref-185715	ATCAATGAAACGTCACCTCCCTCCCTC	14	<lmxll>	ll	132	lm	124		
>ref-185724	TTGCCTTCTACCCTGTCTCCTTCTCCC	21	<nnxnp>	nn	142	np	121		
>ref-185726	AGCCCATCCACCCTAGCTCCAGCTCCA	14	<hkxhk>	hh	0	hk	273	kk	0
>ref-185727	TTCAGTGGCACCTCTTCTCCAGGGGCA	16	<lmxll>	ll	122	lm	130		
>ref-185744	CTTTTTTCGACTCTCCCTCCTTTCTCT	4	<lmxll>	ll	117	lm	116		
>ref-185776	CAGACGCGGACACAGGCTCCATGAAGG	5	<nnxnp>	nn	273	np	0		
>ref-185776	CAGACGCGGACACAGGCTCCATGAAGG	9	<nnxnp>	nn	124	np	140		
>ref-185779	AATCGAACCACTCACTCTCCGCCGCCT	2	<nnxnp>	nn	135	np	130		
>ref-185779	AATCGAACCACTCACTCTCCGCCGCCT	8	<nnxnp>	nn	135	np	130		
>ref-185789	CCACTCCCCACCATCTCTCCTCCCCCA	2	<nnxnp>	nn	96	np	126		
>ref-185789	CCACTCCCCACCATCTCTCCTCCCCCA	13	<hkxhk>	hh	43	hk	135	kk	48
>ref-185801	CTACACCTCACCTTCCCTCCTTCACAC	1	<lmxll>	ll	258	lm	1		
>ref-185801	CTACACCTCACCTTCCCTCCTTCACAC	6	<lmxll>	ll	267	lm	0		
>ref-185842	TCAAGGAGAACTCTAGCTCCAGGGGCT	3	<hkxhk>	hh	1	hk	270	kk	0
>ref-185842	TCAAGGAGAACTCTAGCTCCAGGGGCT	4	<hkxhk>	hh	1	hk	270	kk	0
>ref-185856	AGCCGCACCACACCCCCTCCTCTATCG	13	<lmxll>	ll	104	lm	160		
>ref-185865	GATCCCTGCACTCGCTCTCCCTCGCTG	3	<lmxll>	ll	137	lm	135		
>ref-185877	CCACAACTAACACTACCTCCTCCTCCA	1	<hkxhk>	hh	2	hk	270	kk	0
>ref-185877	CCACAACTAACACTACCTCCTCCTCCA	2	<nnxnp>	nn	190	np	76		
>ref-185897	TTCTCACAAACCCGTTCTCCTTTAACT	13	<lmxll>	ll	124	lm	124		

>ref-185910	CATCACGGAACACACTCTCCCATTCCC	2	<hkxhk>	hh	37	hk	229	kk	0
>ref-185910	CATCACGGAACACACTCTCCCATTCCC	15	<nnxnp>	nn	70	np	200		
>ref-185923	CTCCTTATTACCAATACTCCTTTTTCA	12	<hkxhk>	hh	79	hk	163	kk	0
>ref-185923	CTCCTTATTACCAATACTCCTTTTTCA	21	<lmxll>	ll	204	lm	46		
>ref-185927	GTCCACACCACACCAACTCCACTTCTC	8	<lmxll>	ll	23	lm	242		
>ref-185927	GTCCACACCACACCAACTCCACTTCTC	14	<lmxll>	ll	23	lm	242		
>ref-185927	GTCCACACCACACCAACTCCACTTCTC	21	<lmxll>	ll	10	lm	260		
>ref-185927	GTCCACACCACACCAACTCCACTTCTC	25	<lmxll>	ll	9	lm	260		
>ref-185935	GTTTAATCGACTCATTCTCCCTCGGTA	12	<nnxnp>	nn	138	np	109		
>ref-185959	AACTTTAATACTCCGTCTCCTCTCGTT	7	<nnxnp>	nn	127	np	134		
>ref-185965	CCCAGTCCCACCTTCCCTCCCTCCCTA	27	<lmxll>	ll	8	lm	239		
>ref-185988	TCTAACGCAACACTATCTCCAGCTTTA	5	<hkxhk>	hh	60	hk	122	kk	59
>ref-185991	TACCTTAACACCTCCGCTCCGCCGGTG	6	<hkxhk>	hh	51	hk	161	kk	55
>ref-185998	ACTCTGTACACACTCTCTCCTTCCCAC	1	<nnxnp>	nn	127	np	98		
>ref-186026	ACCCACAAAACCCTGCCTCCATACATA	9	<nnxnp>	nn	118	np	141		
>ref-186026	ACCCACAAAACCCTGCCTCCATACATA	21	<nnxnp>	nn	118	np	141		
>ref-186043	TATCCATAGACCTATACTCCATCCATC	15	<nnxnp>	nn	128	np	122		
>ref-186057	CCCATCAGGACGGAAACTCCTTCTTCC	27	<lmxll>	ll	133	lm	133		
>ref-186112	CCCTTTCCAACTCTCCCTCCTTCCATC	9	<nnxnp>	nn	130	np	114		
>ref-186113	TGTGCGAGTACTGCAGCTCCCCTTAGT	2	<hkxhk>	hh	0	hk	273	kk	0
>ref-186113	TGTGCGAGTACTGCAGCTCCCCTTAGT	26	<hkxhk>	hh	0	hk	273	kk	0
>ref-186116	TGCAACCGAACAATTTCTCCTGCATCA	8	<hkxhk>	hh	43	hk	144	kk	67
>ref-186116	TGCAACCGAACAATTTCTCCTGCATCA	27	<lmxll>	ll	117	lm	139		
>ref-186128	TACGATTGTACGATTTCTCCTCTCCGT	26	<nnxnp>	nn	250	np	0		
>ref-186135	TTCACACAGACAGCAGCTCCAGTTTTT	27	<hkxhk>	hh	56	hk	128	kk	51
>ref-186139	GCAGCTTCCACCCTTCCTCCCTGGGTA	2	<nnxnp>	nn	37	np	227		
>ref-186139	GCAGCTTCCACCCTTCCTCCCTGGGTA	12	<hkxhk>	hh	92	hk	166	kk	0
>ref-186149	GCTGCAGAGACCAACACTCCTCGTCCA	14	<lmxll>	ll	123	lm	150		
>ref-186194	TCCAAGCATACAATCTCTCCTTCTGCT	1	<nnxnp>	nn	20	np	218		
>ref-186215	GAAAGCAGAACAAACCCTCCCAAAATA	16	<lmxll>	ll	118	lm	109		
>ref-186216	TGTTATCTCACTAAAACTCCATTCTCT	4	<lmxll>	ll	110	lm	130		
>ref-186224	TTTCTCTAAACGTAAACTCCATTCTCC	23	<lmxll>	ll	124	lm	120		
>ref-186228	GAGTGGAGAACCTTATCTCCCTCGATG	4	<nnxnp>	nn	137	np	129		
>ref-186230	ACAACCACAACTACCTCTCCACCTACC	1	<nnxnp>	nn	272	np	0		
>ref-186230	ACAACCACAACTACCTCTCCACCTACC	3	<hkxhk>	hh	100	hk	154	kk	0
>ref-186230	ACAACCACAACTACCTCTCCACCTACC	4	<lmxll>	ll	235	lm	31		
>ref-186230	ACAACCACAACTACCTCTCCACCTACC	22	<hkxhk>	hh	1	hk	272	kk	0
>ref-186233	CAGGCAACAACCGTTTCTCCGTGCAAG	13	<hkxhk>	hh	76	hk	142	kk	50

>ref-186233	CAGGCAACAACCGTTTCTCCGTGCAAG	14	<nnxnp>	nn	142	np	126		
>ref-186235	CCTCCGTCAACGCCGCCTCCCGGTCAC	26	<nnxnp>	nn	100	np	152		
>ref-186236	GTATCAGAGACGCGGACTCCAACAGCA	16	<nnxnp>	nn	271	np	0		
>ref-186247	CCTGGCCTCACACTCCCTCCTCCCCAA	26	<lmxll>	ll	267	lm	1		
>ref-186247	CCTGGCCTCACACTCCCTCCTCCCCAA	27	<lmxll>	ll	267	lm	0		
>ref-186249	GATGGAAACACACCTGCTCCTACTAAT	6	<hkxhk>	hh	0	hk	271	kk	0
>ref-186249	GATGGAAACACACCTGCTCCTACTAAT	9	<hkxhk>	hh	34	hk	229	kk	0
>ref-186275	TGCACGCTAACAATTCCTCCCTCAGCG	24	<hkxhk>	hh	50	hk	148	kk	50
>ref-186287	TCCTCCCCGACTGCCTCTCCCACTTCC	2	<nnxnp>	nn	258	np	1		
>ref-186299	CTATATATAACTCCTTCTCCCTCCTTC	1	<nnxnp>	nn	138	np	117		
>ref-186301	GCAAATTATACACACACTCCTTTCCCA	1	<nnxnp>	nn	248	np	0		
>ref-186309	CCTTACCTTACCCGCACTCCCTCCTTT	14	<nnxnp>	nn	123	np	115		
>ref-186309	CCTTACCTTACCCGCACTCCCTCCTTT	25	<nnxnp>	nn	122	np	115		
>ref-186310	CACCTATCCACCTCCCCTCCCACTCAC	21	<hkxhk>	hh	0	hk	245	kk	15
>ref-186310	CACCTATCCACCTCCCCTCCCACTCAC	24	<hkxhk>	hh	0	hk	248	kk	14
>ref-186343	ATACAGCCCACCGTAACTCCATTGTTC	13	<nnxnp>	nn	149	np	113		
>ref-186351	CCCCCTTCTACCCTCCCTCCTTCTTCA	2	<nnxnp>	nn	235	np	0		
>ref-186351	CCCCCTTCTACCCTCCCTCCTTCTTCA	9	<lmxll>	ll	235	lm	1		
>ref-186369	TTTCCAATTACCCTCTCTCCTTCTTTC	1	<hkxhk>	hh	0	hk	270	kk	0
>ref-186369	TTTCCAATTACCCTCTCTCCTTCTTTC	5	<hkxhk>	hh	0	hk	270	kk	0
>ref-186372	TCCCAACCCACTTTCCCTCCCTCCTTA	6	<nnxnp>	nn	190	np	45		
>ref-186372	TCCCAACCCACTTTCCCTCCCTCCTTA	16	<lmxll>	ll	125	lm	127		
>ref-186372	TCCCAACCCACTTTCCCTCCCTCCTTA	27	<nnxnp>	nn	174	np	57		
>ref-186416	CTGGCACCCACCACGCCTCCTAACGCC	7	<lmxll>	ll	139	lm	128		
>ref-186426	TAAATCAACACCGACTCTCCGTTGTTA	1	<hkxhk>	hh	58	hk	149	kk	56
>ref-186432	TGCCAAGGTACGGGGTCTCCTATACCG	8	<hkxhk>	hh	60	hk	148	kk	49
>ref-186443	GATGCCCCAACAGAAGCTCCAACAGAA	4	<hkxhk>	hh	0	hk	249	kk	17
>ref-186443	GATGCCCCAACAGAAGCTCCAACAGAA	12	<hkxhk>	hh	0	hk	273	kk	0
>ref-186461	AAGCCTCTTACGCAATCTCCTTCTGCA	16	<hkxhk>	hh	50	hk	121	kk	69
>ref-186476	AACTTCTGCACTACTTCTCCGAGTACA	24	<nnxnp>	nn	258	np	0		
>ref-186509	CTCATACACACCCCTTCTCCCTTGCTC	1	<nnxnp>	nn	265	np	0		
>ref-186520	ATTCCTCGGACAAGCACTCCGCAACGG	27	<nnxnp>	nn	261	np	0		
>ref-186533	TGGCTTTCGACCTCCTCTCCAAGATTC	4	<lmxll>	ll	59	lm	183		
>ref-186533	TGGCTTTCGACCTCCTCTCCAAGATTC	8	<hkxhk>	hh	27	hk	237	kk	0
>ref-186533	TGGCTTTCGACCTCCTCTCCAAGATTC	15	<hkxhk>	hh	26	hk	237	kk	0
>ref-186533	TGGCTTTCGACCTCCTCTCCAAGATTC	16	<hkxhk>	hh	0	hk	273	kk	0
>ref-186533	TGGCTTTCGACCTCCTCTCCAAGATTC	25	<lmxll>	ll	200	lm	55		
>ref-186533	TGGCTTTCGACCTCCTCTCCAAGATTC	27	<hkxhk>	hh	0	hk	273	kk	0

>ref-186537	TCACCCCCTACTCCCCCTCCCCCCAAG	25	<hkxhk>	hh	231	hk	2	kk	0
>ref-186552	CCAGCTGGTACTCAGACTCCACAGGCT	24	<nnxnp>	nn	270	np	0		
>ref-186552	CCAGCTGGTACTCAGACTCCACAGGCT	26	<hkxhk>	hh	0	hk	242	kk	9
>ref-186579	TTTTGTTTCACTCTCTCTCCCTCCATC	25	<lmxll>	ll	252	lm	2		
>ref-186609	TGAACGATTACATTTGCTCCGTGGAAC	2	<hkxhk>	hh	0	hk	268	kk	0
>ref-186641	TCCTTGTCTACCCCACCTCCCTCTCTT	2	<lmxll>	ll	137	lm	130		
>ref-186644	ATTCCATTTACTCTTCCTCCTCATCAT	27	<hkxhk>	hh	44	hk	128	kk	62
>ref-186649	TCATAAACAACGAATTCTCCAGCATTA	9	<hkxhk>	hh	16	hk	235	kk	0
>ref-186649	TCATAAACAACGAATTCTCCAGCATTA	21	<hkxhk>	hh	9	hk	251	kk	0
>ref-186649	TCATAAACAACGAATTCTCCAGCATTA	25	<nnxnp>	nn	67	np	174		
>ref-186674	CCAGCTCTCACCGTCCCTCCCTCCCAT	6	<nnxnp>	nn	126	np	139		
>ref-186682	TCACGCCTCACCCTTTCTCCCTTCCCC	12	<lmxll>	ll	139	lm	112		
>ref-186708	GAAATAGACACACTGGCTCCCACTCAC	3	<hkxhk>	hh	0	hk	273	kk	0
>ref-186708	GAAATAGACACACTGGCTCCCACTCAC	7	<lmxll>	ll	223	lm	39		
>ref-186708	GAAATAGACACACTGGCTCCCACTCAC	23	<nnxnp>	nn	188	np	70		
>ref-186734	GCCTCTTCTACTTCCTCTCCTTTCCCT	26	<lmxll>	ll	140	lm	113		
>ref-186737	CGCTAAAATACACGTACTCCTACACAT	9	<nnxnp>	nn	130	np	134		
>ref-186742	AGCCATGCTACTCCCCCTCCTCCCCTC	8	<nnxnp>	nn	269	np	0		
>ref-186755	TGTTTGACTACATGAGCTCCATCGCCG	12	<nnxnp>	nn	130	np	135		
>ref-186785	TCCACATCTACGCCGTCTCCATCCACA	6	<hkxhk>	hh	56	hk	217	kk	0
>ref-186814	ATACCTCACACACCCCCTCCCCCCTCT	14	<nnxnp>	nn	124	np	130		
>ref-186816	TTTCTCCTCACTGTCCCTCCCTCACTG	27	<lmxll>	ll	110	lm	132		
>ref-186822	CTGTCTCTAACACCAGCTCCAGACGTA	3	<nnxnp>	nn	121	np	134		
>ref-186829	TCTCCCCCGACGCCCACTCCTTTCCAG	5	<nnxnp>	nn	129	np	129		
>ref-186854	CTCCCTCTCACTCTCACTCCTCATAAT	4	<lmxll>	ll	267	lm	0		
>ref-186854	CTCCCTCTCACTCTCACTCCTCATAAT	5	<lmxll>	ll	268	lm	0		
>ref-186865	TCCTTTCTAACCCTACCTCCCCTTTCA	15	<nnxnp>	nn	127	np	126		
>ref-186870	TCCCCCTCTACTCCCCCTCCACCCTTT	1	<lmxll>	ll	14	lm	245		
>ref-186870	TCCCCCTCTACTCCCCCTCCACCCTTT	2	<lmxll>	ll	51	lm	198		
>ref-186870	TCCCCCTCTACTCCCCCTCCACCCTTT	4	<nnxnp>	nn	147	np	97		
>ref-186870	TCCCCCTCTACTCCCCCTCCACCCTTT	24	<nnxnp>	nn	146	np	96		
>ref-186872	TGATATAAAACCCCTTCTCCTCCGTCT	2	<nnxnp>	nn	101	np	157		
>ref-186881	CACTGGTTTACCCCCTCTCCCTCCCGG	23	<lmxll>	ll	1	lm	263		
>ref-186886	CAAACACACACATACTCTCCCCGACAA	13	<nnxnp>	nn	162	np	99		
>ref-186886	CAAACACACACATACTCTCCCCGACAA	23	<nnxnp>	nn	161	np	99		
>ref-186890	ACCACAGTTACTGACGCTCCCGCACCT	3	<lmxll>	ll	144	lm	111		
>ref-186899	CTTACCCTCACCTCGACTCCGCCCCCT	26	<nnxnp>	nn	273	np	0		
>ref-186899	CTTACCCTCACCTCGACTCCGCCCCCT	27	<nnxnp>	nn	273	np	0		

>ref-186900	AACACCAGTACCCACACTCCCACGTAC	4	<nnxnp>	nn	129	np	136		
>ref-186909	CTCTTTTTGACGCCTCCTCCCTCTCCC	5	<nnxnp>	nn	103	np	134		
>ref-186909	CTCTTTTTGACGCCTCCTCCCTCTCCC	12	<nnxnp>	nn	107	np	133		
>ref-186914	TTCTTTCCTACTTTCCCTCCTATACTT	2	<hkxhk>	hh	28	hk	207	kk	4
>ref-186914	TTCTTTCCTACTTTCCCTCCTATACTT	15	<hkxhk>	hh	3	hk	206	kk	28
>ref-186917	GTCTGCGAGACCAGCGCTCCCTGGGAA	26	<lmxll>	ll	113	lm	111		
>ref-186935	CTTCTTTTCACAATTTCTCCTTCATTG	8	<lmxll>	ll	106	lm	125		
>ref-186935	CTTCTTTTCACAATTTCTCCTTCATTG	26	<nnxnp>	nn	130	np	101		
>ref-186952	TCCTTCCTCACTTCCTCTCCTCTCTTT	24	<hkxhk>	hh	0	hk	271	kk	0
>ref-186952	TCCTTCCTCACTTCCTCTCCTCTCTTT	26	<hkxhk>	hh	0	hk	271	kk	0
>ref-186962	GTGTATCCGACTCAGACTCCGATCCCT	21	<hkxhk>	hh	0	hk	233	kk	25
>ref-186982	GCTGATCCTACTACTGCTCCTTCGCAT	7	<lmxll>	ll	125	lm	118		
>ref-186985	CATATTCCCACTGCTGCTCCTTTTCAT	25	<lmxll>	ll	125	lm	127		
>ref-186999	TCACAACCAACACACCCTCCCACTCAC	15	<hkxhk>	hh	1	hk	202	kk	67
>ref-186999	TCACAACCAACACACCCTCCCACTCAC	21	<hkxhk>	hh	67	hk	202	kk	1
>ref-187006	TTCTTCCTCACCCTCTCTCCACCTTCT	6	<nnxnp>	nn	271	np	0		
>ref-187006	TTCTTCCTCACCCTCTCTCCACCTTCT	8	<nnxnp>	nn	26	np	231		
>ref-187006	TTCTTCCTCACCCTCTCTCCACCTTCT	12	<nnxnp>	nn	125	np	144		
>ref-187006	TTCTTCCTCACCCTCTCTCCACCTTCT	16	<nnxnp>	nn	26	np	231		
>ref-187007	CGAGAGGCGACATACCCTCCTTCTCCT	16	<hkxhk>	hh	0	hk	264	kk	0
>ref-187010	GTTCTGGAGACATAATCTCCATGAGGA	26	<nnxnp>	nn	257	np	0		
>ref-187018	CTATCTCGTACTCTCCCTCCTTCCTCT	22	<hkxhk>	hh	0	hk	270	kk	0
>ref-187022	ACACCATTAACATTCTCTCCACGGCTT	25	<nnxnp>	nn	124	np	139		
>ref-187040	TTGCCCTCCACCTATTCTCCCTCCCTC	14	<nnxnp>	nn	261	np	0		
>ref-187040	TTGCCCTCCACCTATTCTCCCTCCCTC	15	<nnxnp>	nn	262	np	0		
>ref-187072	TCCCCCATCACAACTTCTCCCTCACAA	6	<hkxhk>	hh	0	hk	233	kk	30
>ref-187072	TCCCCCATCACAACTTCTCCCTCACAA	15	<hkxhk>	hh	0	hk	273	kk	0
>ref-187072	TCCCCCATCACAACTTCTCCCTCACAA	16	<hkxhk>	hh	0	hk	219	kk	42
>ref-187080	TGAGGGAGAACCACACCTCCCCCCTCG	27	<lmxll>	ll	128	lm	135		
>ref-187087	CAGTCTGGTACCATTCCTCCTGTTTTG	23	<lmxll>	ll	88	lm	144		
>ref-187111	ACTTTAGATACTGTTCCTCCTTTGTCA	5	<lmxll>	ll	102	lm	125		
>ref-187115	CTCTTTTTAACTCTCTCTCCTCCCCCC	5	<nnxnp>	nn	73	np	161		
>ref-187115	CTCTTTTTAACTCTCTCTCCTCCCCCC	7	<hkxhk>	hh	0	hk	264	kk	1
>ref-187115	CTCTTTTTAACTCTCTCTCCTCCCCCC	9	<nnxnp>	nn	0	np	265		
>ref-187115	CTCTTTTTAACTCTCTCTCCTCCCCCC	22	<nnxnp>	nn	265	np	0		
>ref-187115	CTCTTTTTAACTCTCTCTCCTCCCCCC	27	<lmxll>	ll	106	lm	126		
>ref-187117	ATAGGCGTTACATACTCTCCCTTAGTC	24	<lmxll>	ll	125	lm	126		
>ref-187120	CGAAGAGAGACCCATCCTCCTTCTCCT	22	<nnxnp>	nn	126	np	129		

>ref-187132	CTCATCCCAACAAAGCCTCCTCCCGGT	4	<nnxnp>	nn	144	np	118		
>ref-187132	CTCATCCCAACAAAGCCTCCTCCCGGT	12	<nnxnp>	nn	144	np	118		
>ref-187134	CTCATTCTGACGCCTCCTCCCCCTCCC	3	<lmxll>	ll	12	lm	243		
>ref-187134	CTCATTCTGACGCCTCCTCCCCCTCCC	23	<lmxll>	ll	11	lm	243		
>ref-187142	ATCTTCGAGACGCCCCCTCCTTCCTAG	14	<nnxnp>	nn	133	np	129		
>ref-187169	TTCTAAGCTACTCCTCCTCCCCGAGCA	21	<lmxll>	ll	108	lm	139		
>ref-187186	TCAGAGGTCACGCCCTCTCCCGTGGAT	5	<nnxnp>	nn	265	np	0		
>ref-187200	AAGGAATGCACGCTCACTCCGGCCCTT	25	<lmxll>	ll	144	lm	128		
>ref-187224	CAGAAAGGCACTCCTCCTCCTCTAATA	21	<lmxll>	ll	118	lm	122		
>ref-187224	CAGAAAGGCACTCCTCCTCCTCTAATA	27	<nnxnp>	nn	109	np	127		
>ref-187239	GCCACCCTCACAATTACTCCTTCTCCG	9	<hkxhk>	hh	118	hk	144	kk	0
>ref-187241	CTTGCACTCACTCCGCCTCCCTAATTC	1	<nnxnp>	nn	229	np	38		
>ref-187241	CTTGCACTCACTCCGCCTCCCTAATTC	3	<nnxnp>	nn	182	np	86		
>ref-187241	CTTGCACTCACTCCGCCTCCCTAATTC	26	<hkxhk>	hh	0	hk	270	kk	2
>ref-187244	ACTCCTCACACTCTTGCTCCTATCCTA	2	<nnxnp>	nn	234	np	29		
>ref-187244	ACTCCTCACACTCTTGCTCCTATCCTA	13	<hkxhk>	hh	13	hk	258	kk	0
>ref-187244	ACTCCTCACACTCTTGCTCCTATCCTA	21	<lmxll>	ll	0	lm	273		
>ref-187259	TTCCTACCCACACTCGCTCCCCTCTCC	27	<hkxhk>	hh	48	hk	132	kk	62
>ref-187277	CAGGCGACAACTGCAACTCCTCGCGCC	3	<hkxhk>	hh	0	hk	194	kk	77
>ref-187277	CAGGCGACAACTGCAACTCCTCGCGCC	25	<hkxhk>	hh	0	hk	273	kk	0
>ref-187283	CCTGCACCAACTCTGCCTCCCTACCTC	6	<lmxll>	ll	126	lm	104		
>ref-187316	CTCAGTGTCACCCCGACTCCCGCTGGA	16	<nnxnp>	nn	260	np	0		
>ref-187319	CTAAATAAAACTCTCTCTCCATCCATA	4	<nnxnp>	nn	110	np	124		
>ref-187330	CTAATTGCCACACTGCCTCCATGGCCT	6	<nnxnp>	nn	266	np	0		
>ref-187345	ATGCCCTTAACAATGGCTCCAATAACA	3	<nnxnp>	nn	124	np	136		
>ref-187345	ATGCCCTTAACAATGGCTCCAATAACA	5	<nnxnp>	nn	124	np	136		
>ref-187352	CCAAGAGCAACACATCCTCCAGATATG	9	<hkxhk>	hh	5	hk	264	kk	0
>ref-187352	CCAAGAGCAACACATCCTCCAGATATG	14	<hkxhk>	hh	1	hk	268	kk	0
>ref-187352	CCAAGAGCAACACATCCTCCAGATATG	15	<hkxhk>	hh	0	hk	235	kk	14
>ref-187354	CTGCGCCTCACACCCCCTCCCTCTCCT	5	<lmxll>	ll	260	lm	2		
>ref-187354	CTGCGCCTCACACCCCCTCCCTCTCCT	12	<lmxll>	ll	263	lm	0		
>ref-187354	CTGCGCCTCACACCCCCTCCCTCTCCT	14	<lmxll>	ll	120	lm	138		
>ref-187359	TCTCCCCTCACTTCCCCTCCCTCCTTT	2	<nnxnp>	nn	271	np	0		
>ref-187376	CTCTTCCTGACTTGTCCTCCTCTCTCC	7	<nnxnp>	nn	26	np	239		
>ref-187378	ACTTACACAACGATCACTCCATAACAT	1	<nnxnp>	nn	135	np	122		
>ref-187387	TCCTCACATACTTTTCCTCCTCCCTCT	16	<lmxll>	ll	123	lm	139		
>ref-187414	TCCCTCTCTACCCCTCCTCCCTATCTC	5	<lmxll>	ll	270	lm	3		
>ref-187414	TCCCTCTCTACCCCTCCTCCCTATCTC	6	<hkxhk>	hh	7	hk	264	kk	0

>ref-187414	TCCCTCTCTACCCCTCCTCCCTATCTC	9	<lmxll>	ll	271	lm	2		
>ref-187414	TCCCTCTCTACCCCTCCTCCCTATCTC	15	<hkxhk>	hh	0	hk	268	kk	3
>ref-187414	TCCCTCTCTACCCCTCCTCCCTATCTC	16	<hkxhk>	hh	0	hk	271	kk	0
>ref-187414	TCCCTCTCTACCCCTCCTCCCTATCTC	23	<nnxnp>	nn	17	np	250		
>ref-187426	TGGAACAGTACAGAACCTCCTCCTGTG	1	<nnxnp>	nn	133	np	133		
>ref-187437	TCTAAAAGGACAGCAACTCCCGCAACA	8	<lmxll>	ll	190	lm	74		
>ref-187437	TCTAAAAGGACAGCAACTCCCGCAACA	16	<hkxhk>	hh	251	hk	22	kk	0
>ref-187437	TCTAAAAGGACAGCAACTCCCGCAACA	27	<hkxhk>	hh	262	hk	5	kk	0
>ref-187448	GAGTCTCCAACACAACCTCCCTCAACA	9	<hkxhk>	hh	0	hk	273	kk	0
>ref-187448	GAGTCTCCAACACAACCTCCCTCAACA	14	<hkxhk>	hh	240	hk	31	kk	0
>ref-187448	GAGTCTCCAACACAACCTCCCTCAACA	25	<hkxhk>	hh	0	hk	273	kk	0
>ref-187451	TATTTCATCACTCTTCCTCCCTCTCCT	2	<nnxnp>	nn	131	np	113		
>ref-187451	TATTTCATCACTCTTCCTCCCTCTCCT	26	<nnxnp>	nn	132	np	112		
>ref-187461	TCGCCATTCACTCTCCCTCCTTCCCTC	3	<lmxll>	ll	254	lm	0		
>ref-187461	TCGCCATTCACTCTCCCTCCTTCCCTC	12	<lmxll>	ll	256	lm	1		
>ref-187471	GCACGTACGACCAGTGCTCCTCTCGCA	8	<hkxhk>	hh	51	hk	161	kk	58
>ref-187471	GCACGTACGACCAGTGCTCCTCTCGCA	12	<lmxll>	ll	127	lm	141		
>ref-187482	CACCATCCCACCACCCCTCCTTCCTTC	14	<lmxll>	ll	0	lm	268		
>ref-187482	CACCATCCCACCACCCCTCCTTCCTTC	21	<lmxll>	ll	1	lm	268		
>ref-187486	CCTTGGCTCACCCGGACTCCGGCGTCA	3	<hkxhk>	hh	60	hk	149	kk	54
>ref-187502	ATTCCTTAAACATCCCCTCCTCCTTCT	26	<nnxnp>	nn	131	np	105		
>ref-187513	AAATACGGGACTGTCACTCCGAAGTGT	4	<lmxll>	ll	1	lm	260		
>ref-187513	AAATACGGGACTGTCACTCCGAAGTGT	7	<lmxll>	ll	1	lm	260		
>ref-187517	CTGTTGGCTACACCTCCTCCTCCTCCT	22	<nnxnp>	nn	143	np	125		
>ref-187547	AAAGGAACGACGTATTCTCCCATACTG	7	<nnxnp>	nn	127	np	134		
>ref-187553	CTCCCCCAAACTCTCTCTCCTCCTCCC	4	<lmxll>	ll	197	lm	64		
>ref-187553	CTCCCCCAAACTCTCTCTCCTCCTCCC	6	<lmxll>	ll	23	lm	246		
>ref-187559	CATCTGGGGACTCACACTCCCTCTCTC	8	<hkxhk>	hh	40	hk	145	kk	70
>ref-187583	AGTAGTCTCACTCTCCCTCCTCTCCCC	1	<hkxhk>	hh	3	hk	267	kk	0
>ref-187583	AGTAGTCTCACTCTCCCTCCTCTCCCC	2	<hkxhk>	hh	3	hk	267	kk	0
>ref-187609	TTCTCATTCACTCACTCTCCTCTCCTC	1	<lmxll>	ll	70	lm	154		
>ref-187609	TTCTCATTCACTCACTCTCCTCTCCTC	7	<nnxnp>	nn	49	np	179		
>ref-187609	TTCTCATTCACTCACTCTCCTCTCCTC	14	<lmxll>	ll	88	lm	140		
>ref-187616	CGTTTCTCTACTGTGCCTCCTACCGCT	14	<nnxnp>	nn	116	np	124		
>ref-187640	GCCAATACCACTACCGCTCCCTCCAAC	8	<lmxll>	ll	122	lm	139		
>ref-187645	CTTCACTTCACCTTTTCTCCCCTTCCT	8	<nnxnp>	nn	18	np	251		
>ref-187645	CTTCACTTCACCTTTTCTCCCCTTCCT	15	<nnxnp>	nn	18	np	251		
>ref-187646	CACACATTCACACGCACTCCTGCATAT	24	<hkxhk>	hh	64	hk	125	kk	49

>ref-187665	ACCCGCGCGACGCCCCCTCCTTCAGGT	25	<lmxll>	ll	260	lm	0		
>ref-187665	ACCCGCGCGACGCCCCCTCCTTCAGGT	27	<lmxll>	ll	259	lm	2		
>ref-187675	AGACTTACAACCCAACCTCCCCTTCCC	2	<lmxll>	ll	122	lm	144		
>ref-187675	AGACTTACAACCCAACCTCCCCTTCCC	6	<lmxll>	ll	122	lm	144		
>ref-187680	CACATCTTTACATTCGCTCCTTCCTTT	1	<nnxnp>	nn	139	np	119		
>ref-187682	ACCGACTGCACAGGGTCTCCTAGTAAG	3	<nnxnp>	nn	83	np	181		
>ref-187682	ACCGACTGCACAGGGTCTCCTAGTAAG	8	<nnxnp>	nn	250	np	13		
>ref-187682	ACCGACTGCACAGGGTCTCCTAGTAAG	13	<lmxll>	ll	137	lm	120		
>ref-187688	CACATTCCAACCCAAACTCCTTCCGAG	16	<lmxll>	ll	120	lm	137		
>ref-187694	AGAAACTTCACGCAAACTCCCTGACTC	3	<lmxll>	ll	133	lm	126		
>ref-187698	CCACCCTCCACTAAACCTCCTTCCTCC	5	<nnxnp>	nn	120	np	140		
>ref-187705	TACGCCTCTACTCCCTCTCCCCCACAC	25	<nnxnp>	nn	139	np	109		
>ref-187710	CCTGTTTGGACTGCACCTCCAACCCAT	27	<lmxll>	ll	270	lm	0		
>ref-187721	TGAATCTCAACAGCTTCTCCGTGATCT	21	<lmxll>	ll	148	lm	119		
>ref-187739	CTTCATCCAACTCTTCCTCCCCCATCC	16	<nnxnp>	nn	240	np	0		
>ref-187754	AAACGTTCCACCCCCTCTCCCCCTACC	1	<nnxnp>	nn	246	np	20		
>ref-187805	TCAAACGCCACACCCTCTCCCCCGGCA	1	<hkxhk>	hh	0	hk	273	kk	0
>ref-187806	TCTTTCCTGACTCCTCCTCCTCCTCCT	1	<lmxll>	ll	205	lm	58		
>ref-187806	TCTTTCCTGACTCCTCCTCCTCCTCCT	3	<hkxhk>	hh	0	hk	271	kk	0
>ref-187806	TCTTTCCTGACTCCTCCTCCTCCTCCT	4	<hkxhk>	hh	0	hk	270	kk	3
>ref-187806	TCTTTCCTGACTCCTCCTCCTCCTCCT	6	<lmxll>	ll	273	lm	0		
>ref-187836	CCCTCAAACACCCTGTCTCCTTTTGCT	12	<lmxll>	ll	119	lm	123		
>ref-187836	CCCTCAAACACCCTGTCTCCTTTTGCT	15	<lmxll>	ll	121	lm	124		
>ref-187837	AATCAGCGCACATTATCTCCCCAACTT	25	<lmxll>	ll	135	lm	120		
>ref-187874	CCCTCCCTTACCCTCACTCCTCCTCAG	24	<nnxnp>	nn	137	np	111		
>ref-187944	TTATTTCTGACTCCCTCTCCCTCCTCT	2	<hkxhk>	hh	6	hk	258	kk	1
>ref-187944	TTATTTCTGACTCCCTCTCCCTCCTCT	9	<hkxhk>	hh	6	hk	258	kk	1
>ref-187968	CCCTCACACACCCCACCTCCCCCTCTC	4	<hkxhk>	hh	0	hk	34	kk	212
>ref-188003	CCGCCCTCTACCCCCACTCCCTATATC	2	<lmxll>	ll	120	lm	120		
>ref-188066	TATAAACTAACCATCTCTCCTTTTCTA	9	<lmxll>	ll	113	lm	117		
>ref-188079	TAAAAACTCACACCTACTCCTTGTTAC	8	<hkxhk>	hh	6	hk	228	kk	3
>ref-188112	TGATTACCGACGGCATCTCCAGCATGT	14	<nnxnp>	nn	137	np	130		
>ref-188120	TGTTAAAAAACTTTCTCTCCTCAGATA	8	<nnxnp>	nn	105	np	135		
>ref-188123	TCTAAATATACTCACGCTCCCTACTAT	7	<hkxhk>	hh	2	hk	105	kk	118
>ref-188123	TCTAAATATACTCACGCTCCCTACTAT	27	<nnxnp>	nn	244	np	0		
>ref-188144	TACACTTGTACTAAGTCTCCAATGAGC	12	<hkxhk>	hh	0	hk	33	kk	230
>ref-188144	TACACTTGTACTAAGTCTCCAATGAGC	22	<nnxnp>	nn	256	np	12		
>ref-188144	TACACTTGTACTAAGTCTCCAATGAGC	23	<hkxhk>	hh	0	hk	9	kk	263

>ref-188144	TACACTTGTACTAAGTCTCCAATGAGC	26	<lmxll>	ll	268	lm	4		
>ref-188144	TACACTTGTACTAAGTCTCCAATGAGC	27	<hkxhk>	hh	211	hk	50	kk	0
>ref-188201	CACACACACACAGACGCTCCTCTGCTG	5	<hkxhk>	hh	0	hk	273	kk	0
>ref-188201	CACACACACACAGACGCTCCTCTGCTG	16	<hkxhk>	hh	0	hk	273	kk	0
>ref-188203	AATAACAGTACACGAACTCCCGGAATA	22	<nnxnp>	nn	129	np	102		
>ref-188206	TACCGGGCGACTAGGTCTCCGCCGTCG	15	<nnxnp>	nn	121	np	151		
>ref-188210	ACCTCTTCCACCCTCTCTCCACCACCT	12	<nnxnp>	nn	132	np	138		
>ref-188210	ACCTCTTCCACCCTCTCTCCACCACCT	22	<hkxhk>	hh	7	hk	261	kk	0
>ref-188232	ATTTCCCTCACCCTGTCTCCTTCTCTC	15	<nnxnp>	nn	230	np	31		
>ref-188241	GAGACAGACACACGGACTCCGGCAAAA	3	<nnxnp>	nn	124	np	140		
>ref-188249	GACGTCGCCACTCTGGCTCCAACATTG	22	<nnxnp>	nn	136	np	124		
>ref-188249	GACGTCGCCACTCTGGCTCCAACATTG	23	<lmxll>	ll	159	lm	101		
>ref-188265	AGAGAAAGCACAAAGGCTCCCAAAGCT	8	<hkxhk>	hh	65	hk	131	kk	69
>ref-188267	TCCTTCCTCACCCTCTCTCCTCTTTCT	3	<lmxll>	ll	102	lm	145		
>ref-188267	TCCTTCCTCACCCTCTCTCCTCTTTCT	24	<hkxhk>	hh	0	hk	219	kk	40
>ref-188297	ATTGCAGCCACCCCCTCTCCAACCTTT	9	<lmxll>	ll	135	lm	112		
>ref-188306	TACATCCTTACGTCACCTCCTAAGTTG	26	<hkxhk>	hh	1	hk	97	kk	137
>ref-188312	AACACCACTACCGTCACTCCTACAATG	24	<lmxll>	ll	132	lm	132		
>ref-188315	GCATCAGTCACCGTTTCTCCCATACTT	25	<lmxll>	ll	91	lm	141		
>ref-188360	GCTCGTGTAACAAGTACTCCGTCAGCT	2	<lmxll>	ll	127	lm	122		
>ref-188360	GCTCGTGTAACAAGTACTCCGTCAGCT	5	<nnxnp>	nn	264	np	0		
>ref-188362	GTATATATTACTATCTCTCCTCCTCCC	27	<nnxnp>	nn	239	np	3		
>ref-188392	GCCGTCCGCACCCGCCCTCCGGGCTGA	7	<hkxhk>	hh	1	hk	271	kk	0
>ref-188392	GCCGTCCGCACCCGCCCTCCGGGCTGA	16	<hkxhk>	hh	0	hk	269	kk	2
>ref-188392	GCCGTCCGCACCCGCCCTCCGGGCTGA	22	<hkxhk>	hh	1	hk	272	kk	0
>ref-188392	GCCGTCCGCACCCGCCCTCCGGGCTGA	25	<hkxhk>	hh	0	hk	248	kk	19
>ref-188395	AGCAGTTGGACACGATCTCCGCTTACA	21	<nnxnp>	nn	128	np	142		
>ref-188400	TACCCTTGAACTAACCCTCCCTCCCAC	4	<lmxll>	ll	193	lm	26		
>ref-188400	TACCCTTGAACTAACCCTCCCTCCCAC	5	<lmxll>	ll	193	lm	26		
>ref-188400	TACCCTTGAACTAACCCTCCCTCCCAC	6	<lmxll>	ll	193	lm	26		
>ref-188400	TACCCTTGAACTAACCCTCCCTCCCAC	7	<lmxll>	ll	193	lm	27		
>ref-188400	TACCCTTGAACTAACCCTCCCTCCCAC	12	<lmxll>	ll	193	lm	26		
>ref-188400	TACCCTTGAACTAACCCTCCCTCCCAC	13	<lmxll>	ll	193	lm	26		
>ref-188400	TACCCTTGAACTAACCCTCCCTCCCAC	14	<lmxll>	ll	189	lm	30		
>ref-188400	TACCCTTGAACTAACCCTCCCTCCCAC	24	<lmxll>	ll	193	lm	26		
>ref-188410	CATAAAAACACTACAACTCCGCCGCCA	26	<lmxll>	ll	114	lm	132		
>ref-188435	ACCTCAAGGACGCGGCCTCCTCCCTGC	2	<nnxnp>	nn	116	np	135		
>ref-188437	CACGCTTAGACTCCTCCTCCTCTTCCT	23	<lmxll>	ll	261	lm	0		

>ref-188437	CACGCTTAGACTCCTCCTCCTCTTCCT	26	<lmxll>	ll	267	lm	0		
>ref-188447	TTACCAAGCACCCTCCCTCCCTCAGCT	3	<nnxnp>	nn	135	np	130		
>ref-188468	CCTCTGTAGACTTCTCCTCCAAGCCGG	22	<lmxll>	ll	123	lm	138		
>ref-188507	TCCCCCATCACCTCTCCTCCACCCTCA	2	<lmxll>	ll	269	lm	0		
>ref-188515	CTCACATAAACCGTGTCTCCCACATAA	12	<lmxll>	ll	126	lm	113		
>ref-188530	AGTTATGACACTGCACCTCCATCAGTG	8	<lmxll>	ll	134	lm	136		
>ref-188537	GGTAGTACCACACGCTCTCCACAACAC	2	<lmxll>	ll	137	lm	129		
>ref-188537	GGTAGTACCACACGCTCTCCACAACAC	3	<lmxll>	ll	148	lm	120		
>ref-188537	GGTAGTACCACACGCTCTCCACAACAC	6	<lmxll>	ll	137	lm	130		
>ref-188537	GGTAGTACCACACGCTCTCCACAACAC	21	<hkxhk>	hh	1	hk	262	kk	0
>ref-188543	TTTGTTTCAACTTTTTCTCCCTTTCAT	26	<nnxnp>	nn	116	np	126		
>ref-188559	GGTAACGACACCACCCCTCCCTCCTAT	13	<hkxhk>	hh	5	hk	265	kk	0
>ref-188559	GGTAACGACACCACCCCTCCCTCCTAT	22	<hkxhk>	hh	0	hk	201	kk	50
>ref-188559	GGTAACGACACCACCCCTCCCTCCTAT	23	<hkxhk>	hh	53	hk	197	kk	0
>ref-188571	ATTCTCCCCACCCTCTCTCCCTCCTTC	1	<hkxhk>	hh	238	hk	2	kk	0
>ref-188571	ATTCTCCCCACCCTCTCTCCCTCCTTC	2	<nnxnp>	nn	250	np	6		
>ref-188599	TAAATGCCAACCAAGTCTCCCCCGCAG	2	<hkxhk>	hh	58	hk	116	kk	56
>ref-188600	TTGCATGCAACTTGCACTCCGCCTGCC	7	<nnxnp>	nn	142	np	129		
>ref-188628	AAATACGCGACAACATCTCCGTTTTGG	2	<lmxll>	ll	129	lm	123		
>ref-188628	AAATACGCGACAACATCTCCGTTTTGG	5	<lmxll>	ll	130	lm	123		
>ref-188632	CACATCTCAACATACCCTCCCACATCC	25	<lmxll>	ll	124	lm	122		
>ref-188639	ATTTCCCTCACCTTCTCTCCTCTCTTC	23	<lmxll>	ll	253	lm	4		
>ref-188639	ATTTCCCTCACCTTCTCTCCTCTCTTC	24	<lmxll>	ll	265	lm	0		
>ref-188651	TAATATCACACTGATTCTCCATCTGTG	1	<hkxhk>	hh	62	hk	143	kk	53
>ref-188664	TCTGAATTAACTACATCTCCACTTCCA	25	<lmxll>	ll	92	lm	137		
>ref-188691	CAGCTCCACACCTTCTCTCCCTCTTTC	3	<nnxnp>	nn	138	np	129		
>ref-188708	ACCTCAACAACTTCGCCTCCTCGTTCT	14	<nnxnp>	nn	132	np	134		
>ref-188711	CCACGTCTGACGATCTCTCCTAGCGTT	15	<hkxhk>	hh	0	hk	273	kk	0
>ref-188711	CCACGTCTGACGATCTCTCCTAGCGTT	21	<hkxhk>	hh	0	hk	273	kk	0
>ref-188713	CTAATAAAGACAACTACTCCGCCCACA	3	<lmxll>	ll	0	lm	265		
>ref-188713	CTAATAAAGACAACTACTCCGCCCACA	12	<lmxll>	ll	0	lm	265		
>ref-188722	TCTCTTTCTACGTATCCTCCCCTCTCC	12	<nnxnp>	nn	160	np	101		
>ref-188722	TCTCTTTCTACGTATCCTCCCCTCTCC	22	<nnxnp>	nn	162	np	101		
>ref-188727	TTCCCCATTACCCCTACTCCTTTCCCA	25	<hkxhk>	hh	68	hk	148	kk	45
>ref-188737	TATGGTCTTACGGTATCTCCTAAGCCG	2	<nnxnp>	nn	265	np	0		
>ref-188744	TAGGTGAGCACGTGTTCTCCTTTACAT	27	<hkxhk>	hh	70	hk	146	kk	50
>ref-188762	GCTACTACGACCCTTTCTCCAACTACG	9	<nnxnp>	nn	248	np	1		
>ref-188781	CGAATACCCACACACCCTCCCGACACT	26	<lmxll>	ll	149	lm	118		

>ref-188783	AGGTTAGAAACTTCTTCTCCTGCCATA	12	<hkxhk>	hh	1	hk	266	kk	5
>ref-188783	AGGTTAGAAACTTCTTCTCCTGCCATA	26	<hkxhk>	hh	1	hk	266	kk	5
>ref-188801	CTCTGCCTCACTCCCCCTCCTCAGTTT	9	<lmxll>	ll	123	lm	136		
>ref-188819	CCCAACGAAACTTTCTCTCCTACTATT	22	<lmxll>	ll	91	lm	134		
>ref-188832	GGGACTCGAACCCCAGCTCCCCCGCCT	13	<nnxnp>	nn	127	np	135		
>ref-188842	CCATACCCCACCTCCCCTCCTCCCACC	1	<nnxnp>	nn	213	np	46		
>ref-188842	CCATACCCCACCTCCCCTCCTCCCACC	3	<nnxnp>	nn	57	np	187		
>ref-188842	CCATACCCCACCTCCCCTCCTCCCACC	6	<nnxnp>	nn	187	np	65		
>ref-188842	CCATACCCCACCTCCCCTCCTCCCACC	9	<hkxhk>	hh	2	hk	262	kk	0
>ref-188842	CCATACCCCACCTCCCCTCCTCCCACC	13	<hkxhk>	hh	0	hk	185	kk	52
>ref-188844	CCCTTTTCCACTCCCCCTCCTTTCCTT	2	<hkxhk>	hh	2	hk	265	kk	0
>ref-188844	CCCTTTTCCACTCCCCCTCCTTTCCTT	22	<hkxhk>	hh	0	hk	264	kk	2
>ref-188845	TGTTCCCTCACTTCCCCTCCGACACTC	2	<lmxll>	ll	103	lm	143		
>ref-188847	CCCCCTCAAACCCTACCTCCCCCTCCC	3	<nnxnp>	nn	145	np	96		
>ref-188847	CCCCCTCAAACCCTACCTCCCCCTCCC	9	<nnxnp>	nn	249	np	1		
>ref-188847	CCCCCTCAAACCCTACCTCCCCCTCCC	14	<lmxll>	ll	88	lm	132		
>ref-188853	CTGGCATTGACAGACACTCCAAGAAAC	6	<hkxhk>	hh	32	hk	231	kk	0
>ref-188853	CTGGCATTGACAGACACTCCAAGAAAC	9	<hkxhk>	hh	0	hk	273	kk	0
>ref-188853	CTGGCATTGACAGACACTCCAAGAAAC	16	<hkxhk>	hh	6	hk	267	kk	0
>ref-188872	AAAATCTAAACAAAGTCTCCGATTTCC	6	<hkxhk>	hh	0	hk	147	kk	80
>ref-188872	AAAATCTAAACAAAGTCTCCGATTTCC	14	<hkxhk>	hh	138	hk	90	kk	0
>ref-188872	AAAATCTAAACAAAGTCTCCGATTTCC	16	<lmxll>	ll	161	lm	73		
>ref-188909	CTGCTGAGGACTGGCACTCCCTGGCAT	14	<hkxhk>	hh	57	hk	147	kk	64
>ref-188923	TCCACTTCCACCCCGCCTCCTTCTTTC	1	<hkxhk>	hh	27	hk	186	kk	53
>ref-188923	TCCACTTCCACCCCGCCTCCTTCTTTC	6	<lmxll>	ll	108	lm	154		
>ref-188923	TCCACTTCCACCCCGCCTCCTTCTTTC	21	<lmxll>	ll	109	lm	154		
>ref-188973	GTCCTGTCCACTTCCCCTCCAGGACCC	8	<nnxnp>	nn	105	np	137		
>ref-188975	ACCTCACGCACGTCGCCTCCCGTGACG	1	<nnxnp>	nn	245	np	3		
>ref-188975	ACCTCACGCACGTCGCCTCCCGTGACG	2	<nnxnp>	nn	254	np	0		
>ref-188978	TCACCCTCAACCTTTCCTCCAGCACTC	25	<hkxhk>	hh	3	hk	263	kk	0
>ref-188978	TCACCCTCAACCTTTCCTCCAGCACTC	27	<lmxll>	ll	7	lm	266		
>ref-188986	GCTTTTATTACTCTTCCTCCTTATCTT	7	<hkxhk>	hh	8	hk	220	kk	5
>ref-188986	GCTTTTATTACTCTTCCTCCTTATCTT	23	<hkxhk>	hh	8	hk	219	kk	4
>ref-189020	GAAGCTCAAACAAAATCTCCAAATGCA	25	<nnxnp>	nn	117	np	144		
>ref-189021	CTTGCTAGAACCCCAACTCCTTCCCCA	4	<nnxnp>	nn	130	np	129		
>ref-189041	TACGAGAAGACGCTTCCTCCGCCGCCG	3	<lmxll>	ll	92	lm	163		
>ref-189041	TACGAGAAGACGCTTCCTCCGCCGCCG	21	<hkxhk>	hh	0	hk	233	kk	27
>ref-189139	TGCACCCTCACCCCCCCTCCCCCATCC	5	<nnxnp>	nn	270	np	0		

>ref-189144	CTTCTAAACACATTAACTCCTTAGAGA	5	<nnxnp>	nn	4	np	240		
>ref-189148	GTAACTGTAACTGTTTCTCCACTTCTA	24	<lmxll>	ll	124	lm	118		
>ref-189152	TTCAGAGATACCGGGGCTCCTCGGTCA	1	<lmxll>	ll	114	lm	150		
>ref-189152	TTCAGAGATACCGGGGCTCCTCGGTCA	22	<hkxhk>	hh	57	hk	154	kk	54
>ref-189162	GTGACCTTTACCATGTCTCCGTCCACA	1	<hkxhk>	hh	0	hk	273	kk	0
>ref-189196	CTTTCTTTTACTTTTACTCCACCTTTC	26	<lmxll>	ll	150	lm	97		
>ref-189204	CATCCCCCCACCCATCCTCCACTCCCC	3	<nnxnp>	nn	179	np	77		
>ref-189204	CATCCCCCCACCCATCCTCCACTCCCC	14	<nnxnp>	nn	175	np	78		
>ref-189217	CCACCTGCCACCCTTCCTCCGCTCTCT	3	<lmxll>	ll	265	lm	0		
>ref-189223	TTTCCTCCCACAGCCTCTCCTCGCTTT	24	<nnxnp>	nn	145	np	99		
>ref-189237	CCTTTCTCTACATTCCCTCCTCTACCT	8	<hkxhk>	hh	128	hk	126	kk	0
>ref-189240	CTATCATCTACCTTTTCTCCTATCTTC	16	<lmxll>	ll	248	lm	0		
>ref-189261	CGCCTTTAGACCTCTCCTCCCTCACTG	22	<nnxnp>	nn	272	np	0		
>ref-189263	TCTTTTTTTACGTCCTCTCCTCTTTCT	3	<hkxhk>	hh	54	hk	136	kk	50
>ref-189310	TTCCTCCCCACCTACCCTCCCCCCCTT	6	<hkxhk>	hh	1	hk	221	kk	31
>ref-189310	TTCCTCCCCACCTACCCTCCCCCCCTT	15	<hkxhk>	hh	1	hk	217	kk	32
>ref-189310	TTCCTCCCCACCTACCCTCCCCCCCTT	22	<nnxnp>	nn	253	np	5		
>ref-189310	TTCCTCCCCACCTACCCTCCCCCCCTT	27	<nnxnp>	nn	248	np	5		
>ref-189338	ACTCTCTCCACTTTTCCTCCCCATCTT	26	<lmxll>	ll	131	lm	102		
>ref-189345	CTATCATGAACATTGTCTCCATCATTA	26	<lmxll>	ll	117	lm	140		
>ref-189353	TCCTTTATCACCCGTCCTCCTCTCTCC	24	<nnxnp>	nn	140	np	107		
>ref-189355	CCTGTCACCACTTTTTCTCCTCGCTCC	3	<lmxll>	ll	131	lm	97		
>ref-189355	CCTGTCACCACTTTTTCTCCTCGCTCC	21	<nnxnp>	nn	243	np	0		
>ref-189355	CCTGTCACCACTTTTTCTCCTCGCTCC	27	<nnxnp>	nn	243	np	0		
>ref-189379	TGAAAGTAAACATACACTCCCGTAACA	7	<nnxnp>	nn	121	np	113		
>ref-189423	TGTCCCTTCACACTTCCTCCGTCGCAG	25	<lmxll>	ll	148	lm	119		
>ref-189430	ACAATTATAACATATCCTCCTAATGAT	21	<nnxnp>	nn	105	np	131		
>ref-189469	CATCTTGACACCAGACCTCCAAGACAG	4	<hkxhk>	hh	0	hk	177	kk	74
>ref-189469	CATCTTGACACCAGACCTCCAAGACAG	5	<hkxhk>	hh	0	hk	273	kk	0
>ref-189469	CATCTTGACACCAGACCTCCAAGACAG	8	<hkxhk>	hh	52	hk	204	kk	0
>ref-189469	CATCTTGACACCAGACCTCCAAGACAG	9	<lmxll>	ll	159	lm	93		
>ref-189469	CATCTTGACACCAGACCTCCAAGACAG	24	<lmxll>	ll	15	lm	251		
>ref-189469	CATCTTGACACCAGACCTCCAAGACAG	26	<lmxll>	ll	160	lm	93		
>ref-189479	CCTTTTCCCACATCACCTCCACCACCC	2	<lmxll>	ll	248	lm	0		
>ref-189545	GTCTCTGCCACCTCCTCTCCGTGGCAG	27	<nnxnp>	nn	263	np	0		
>ref-189588	TTATCTTTTACTCGTTCTCCTTCTCCC	3	<nnxnp>	nn	265	np	1		
>ref-189588	TTATCTTTTACTCGTTCTCCTTCTCCC	14	<nnxnp>	nn	264	np	0		
>ref-189605	TGAACACATACACACTCTCCTACTCAC	7	<lmxll>	ll	147	lm	119		

>ref-189606	TCCTGAGCCACTGAGCCTCCTACCTCT	22	<hkxhk>	hh	59	hk	153	kk	56
>ref-189626	TCCCCGTGCACGTCGCCTCCTTCCTAT	15	<lmxll>	ll	132	lm	131		
>ref-189670	CATCATGCCACACACTCTCCTTAACTC	1	<nnxnp>	nn	270	np	0		
>ref-189674	GTAAGCATCACTGAACCTCCTTGTTCT	1	<hkxhk>	hh	40	hk	143	kk	63
>ref-189675	TTATCTCTTACCTTCCCTCCTCCTCCT	4	<hkxhk>	hh	0	hk	267	kk	4
>ref-189675	TTATCTCTTACCTTCCCTCCTCCTCCT	5	<hkxhk>	hh	0	hk	270	kk	1
>ref-189675	TTATCTCTTACCTTCCCTCCTCCTCCT	22	<hkxhk>	hh	2	hk	270	kk	0
>ref-189676	ACCCCTCTCACTGAAGCTCCTCAGTTT	23	<lmxll>	ll	98	lm	137		
>ref-189680	CCCCCCTCCACTGTCTCTCCCAAACAA	6	<lmxll>	ll	118	lm	131		
>ref-189681	CTACCCCACACTCACCCTCCACCCTCC	5	<nnxnp>	nn	268	np	0		
>ref-189692	ACAAGTTTGACTCATCCTCCACGTGTT	5	<nnxnp>	nn	139	np	127		
>ref-189694	TCCTCCTAAACATATGCTCCCCCTGTA	7	<hkxhk>	hh	61	hk	144	kk	55
>ref-189694	TCCTCCTAAACATATGCTCCCCCTGTA	25	<hkxhk>	hh	60	hk	144	kk	55
>ref-189708	TTCTTCCTCACGCTCCCTCCCCTCACT	3	<hkxhk>	hh	3	hk	266	kk	0
>ref-189708	TTCTTCCTCACGCTCCCTCCCCTCACT	25	<hkxhk>	hh	3	hk	266	kk	0
>ref-189718	CCATCATCCACTTTTTCTCCTCGCCCG	8	<lmxll>	ll	272	lm	0		
>ref-189770	TTTCCTTTTACACCTGCTCCTCCTGTT	6	<lmxll>	ll	115	lm	145		
>ref-189781	TCATCTCTCACTCTAGCTCCTTACTTT	3	<nnxnp>	nn	120	np	120		
>ref-189793	GGAAGGGCTACTGTCCCTCCTTCCCAG	3	<hkxhk>	hh	0	hk	273	kk	0
>ref-189793	GGAAGGGCTACTGTCCCTCCTTCCCAG	22	<hkxhk>	hh	0	hk	273	kk	0
>ref-189806	TTTCAGATTACAGTCTCTCCTCCTATA	24	<lmxll>	ll	112	lm	110		
>ref-189825	TTTTATCTCACACAACCTCCTTTTAAC	13	<nnxnp>	nn	261	np	0		
>ref-189837	CGGTAAGGTACGCCTCCTCCTTCGCTA	23	<nnxnp>	nn	137	np	129		
>ref-189843	CAGCATATCACTCTAGCTCCAGAAACA	13	<nnxnp>	nn	97	np	138		
>ref-189853	ATTTATTGCACTCTCCCTCCCTCCCTT	6	<lmxll>	ll	4	lm	263		
>ref-189853	ATTTATTGCACTCTCCCTCCCTCCCTT	16	<lmxll>	ll	4	lm	264		
>ref-189872	CCACCCGTAACGACAACTCCTCTCGCT	26	<lmxll>	ll	1	lm	272		
>ref-189879	TTGCGTCTGACTCCATCTCCTTTCCCC	4	<nnxnp>	nn	263	np	0		
>ref-189883	GGTTTTCGAACAAGCCCTCCCGACTTA	4	<nnxnp>	nn	120	np	131		
>ref-189886	TTATCGTCCACCCCCTCTCCCTCTATT	7	<hkxhk>	hh	0	hk	268	kk	1
>ref-189886	TTATCGTCCACCCCCTCTCCCTCTATT	9	<hkxhk>	hh	0	hk	268	kk	0
>ref-189889	GAGAGACAAACACACCCTCCCATGGTG	7	<lmxll>	ll	127	lm	131		
>ref-189892	TCCACAGCAACCTCCTCTCCCTTCCCT	12	<lmxll>	ll	128	lm	143		
>ref-189901	CCTTTGCGTACACTTCCTCCCTTCAAC	9	<nnxnp>	nn	235	np	0		
>ref-189920	GGACTTCGAACTGATTCTCCTTATACA	25	<lmxll>	ll	133	lm	132		
>ref-189930	CAGGAAGGCACGTCTTCTCCCCAAGTT	1	<lmxll>	ll	134	lm	126		
>ref-189930	CAGGAAGGCACGTCTTCTCCCCAAGTT	9	<hkxhk>	hh	41	hk	152	kk	61
>ref-189930	CAGGAAGGCACGTCTTCTCCCCAAGTT	24	<nnxnp>	nn	97	np	155		

>ref-189935	CCGTCTCCAACACCTCCTCCACCCCTC	6	<nnxnp>	nn	80	np	182		
>ref-189935	CCGTCTCCAACACCTCCTCCACCCCTC	8	<lmxll>	ll	164	lm	103		
>ref-189935	CCGTCTCCAACACCTCCTCCACCCCTC	14	<nnxnp>	nn	161	np	104		
>ref-189935	CCGTCTCCAACACCTCCTCCACCCCTC	25	<lmxll>	ll	58	lm	211		
>ref-189935	CCGTCTCCAACACCTCCTCCACCCCTC	27	<lmxll>	ll	104	lm	161		
>ref-189942	TACCCCTTCACCCCCTCTCCTTGTTTC	1	<nnxnp>	nn	127	np	104		
>ref-189961	TACAATGATACGACCTCTCCAGGCTTC	4	<lmxll>	ll	135	lm	87		
>ref-189977	TATGCATACACTGCTCCTCCTTCCCCA	3	<nnxnp>	nn	174	np	85		
>ref-189989	ATCCTTCTCACCCTCCCTCCTCGCTCT	5	<lmxll>	ll	158	lm	87		
>ref-189989	ATCCTTCTCACCCTCCCTCCTCGCTCT	23	<lmxll>	ll	150	lm	91		
>ref-189994	ATTGCGCCGACATCAACTCCTGCATTT	13	<hkxhk>	hh	58	hk	142	kk	60
>ref-190001	GGTAGGTACACCACACCTCCGTCCAAG	3	<lmxll>	ll	4	lm	265		
>ref-190001	GGTAGGTACACCACACCTCCGTCCAAG	4	<lmxll>	ll	4	lm	265		
>ref-190045	TCCGTTATTACTCTGGCTCCTTACATA	21	<nnxnp>	nn	125	np	141		
>ref-190054	AAAATATATACTAATCCTCCTTCATTA	2	<nnxnp>	nn	207	np	62		
>ref-190070	CTCGCGCACACCCTCTCTCCCTCTTAT	5	<lmxll>	ll	116	lm	131		
>ref-190070	CTCGCGCACACCCTCTCTCCCTCTTAT	8	<nnxnp>	nn	124	np	118		
>ref-190070	CTCGCGCACACCCTCTCTCCCTCTTAT	9	<nnxnp>	nn	124	np	116		
>ref-190070	CTCGCGCACACCCTCTCTCCCTCTTAT	24	<nnxnp>	nn	120	np	132		
>ref-190074	TGTTATTGAACATCCACTCCCCTCCTC	9	<lmxll>	ll	145	lm	118		
>ref-190074	TGTTATTGAACATCCACTCCCCTCCTC	13	<lmxll>	ll	110	lm	147		
>ref-190099	TCCACCAAAACATTTCCTCCTTCCCCT	6	<nnxnp>	nn	126	np	134		
>ref-190100	CCGCCCAACACCGATCCTCCCTCCCGC	7	<nnxnp>	nn	113	np	138		
>ref-190109	TTACTAGGCACACCACCTCCCGCCACA	7	<nnxnp>	nn	115	np	143		
>ref-190116	CTCGAGGAAACGTTTACTCCGAGCTGT	5	<lmxll>	ll	114	lm	138		
>ref-190122	GCAAGGAAAACTCGACCTCCTGGGAAT	13	<lmxll>	ll	131	lm	128		
>ref-190124	TACACATACACATATTCTCCCTCTTTC	2	<lmxll>	ll	135	lm	126		
>ref-190130	GCGCCGCCGACGACGCCTCCGCAGCTT	8	<nnxnp>	nn	271	np	0		
>ref-190134	TCTCCCCTAACTGCTCCTCCTCAGTCC	8	<hkxhk>	hh	59	hk	151	kk	55
>ref-190145	ACCACCAAAACTCTTCCTCCTCCTCCT	6	<hkxhk>	hh	60	hk	195	kk	2
>ref-190145	ACCACCAAAACTCTTCCTCCTCCTCCT	8	<hkxhk>	hh	61	hk	196	kk	2
>ref-190151	CTTACTATCACACTTGCTCCCACACCA	3	<hkxhk>	hh	63	hk	146	kk	47
>ref-190164	AGTTTTCTCACTCTTCCTCCTCTTCCG	25	<lmxll>	ll	144	lm	106		
>ref-190206	TCCTCCATCACAACTTCTCCCTCTCTC	4	<lmxll>	ll	12	lm	246		
>ref-190218	TTCTTTTCCACCCATTCTCCCTCCTCT	27	<hkxhk>	hh	46	hk	116	kk	58
>ref-190224	ACAATGAGGACAAGGTCTCCATCCTGA	9	<hkxhk>	hh	0	hk	177	kk	81
>ref-190224	ACAATGAGGACAAGGTCTCCATCCTGA	12	<hkxhk>	hh	20	hk	245	kk	0
>ref-190224	ACAATGAGGACAAGGTCTCCATCCTGA	13	<hkxhk>	hh	9	hk	254	kk	0

>ref-190224	ACAATGAGGACAAGGTCTCCATCCTGA	21	<hkxhk>	hh	0	hk	273	kk	0
>ref-190230	AGGGTAAAGACGTAGCCTCCTACGTCT	2	<lmxll>	ll	134	lm	134		
>ref-190238	AGAAGCAGAACGTGGGCTCCATGAACG	25	<hkxhk>	hh	67	hk	135	kk	60
>ref-190254	GCATGTTTAACGTTTTCTCCTCTCCCT	9	<nnxnp>	nn	144	np	125		
>ref-190257	CGCATAGACACTGTTTCTCCTGCAGTC	13	<hkxhk>	hh	45	hk	149	kk	72
>ref-190266	AATCTCCTCACTCCTTCTCCTTCTCTA	25	<lmxll>	ll	136	lm	134		
>ref-190291	CTTTCTATCACATTCTCTCCTCATCTC	3	<hkxhk>	hh	2	hk	265	kk	4
>ref-190291	CTTTCTATCACATTCTCTCCTCATCTC	7	<hkxhk>	hh	5	hk	267	kk	0
>ref-190291	CTTTCTATCACATTCTCTCCTCATCTC	12	<hkxhk>	hh	4	hk	265	kk	2
>ref-190291	CTTTCTATCACATTCTCTCCTCATCTC	14	<hkxhk>	hh	0	hk	267	kk	5
>ref-190295	TTCATTCGCACCCTCCCTCCTTCACAT	14	<nnxnp>	nn	131	np	130		
>ref-190295	TTCATTCGCACCCTCCCTCCTTCACAT	15	<nnxnp>	nn	131	np	131		
>ref-190319	CACCCTTTCACACATACTCCCTTATCC	3	<lmxll>	ll	122	lm	127		
>ref-190327	CACACATCCACCCACACTCCCACACCG	21	<nnxnp>	nn	135	np	128		
>ref-190331	CCCCTCTCTACCCGTTCTCCCACACCC	5	<lmxll>	ll	139	lm	84		
>ref-190342	CTCCCCAACACCTCTCCTCCCCCTACA	23	<nnxnp>	nn	130	np	128		
>ref-190356	TCTAGGTGTACCGCTCCTCCTCTCTTC	1	<nnxnp>	nn	113	np	134		
>ref-190361	TCTCCGCTGACTCCCTCTCCTTTCTTC	2	<lmxll>	ll	138	lm	127		
>ref-190362	ATGGCGACGACTACATCTCCGCCGTCT	25	<hkxhk>	hh	49	hk	157	kk	62
>ref-190375	CTCTCCCTTACTTTCCCTCCACTCACA	13	<hkxhk>	hh	47	hk	132	kk	56
>ref-190390	GCCCTCCCTACACCCCCTCCTCCTCCT	6	<lmxll>	ll	22	lm	221		
>ref-190413	CCCACGAGCACTCAGCCTCCCCGTACC	5	<lmxll>	ll	134	lm	131		
>ref-190448	AATGAGATTACTTCAGCTCCTCCAACA	16	<nnxnp>	nn	122	np	125		
>ref-190454	ATCCTCCCCACTCCCTCTCCTAAATTA	3	<lmxll>	ll	111	lm	111		
>ref-190455	ATTTCCTTGACTCTTGCTCCTTCTTAA	26	<lmxll>	ll	111	lm	112		
>ref-190456	TTCCCGAACACCCGTTCTCCCTCCTAT	13	<hkxhk>	hh	51	hk	143	kk	77
>ref-190457	CCTCCTTCTACCCTTTCTCCTTCTCTA	5	<hkxhk>	hh	13	hk	253	kk	0
>ref-190457	CCTCCTTCTACCCTTTCTCCTTCTCTA	7	<hkxhk>	hh	0	hk	231	kk	32
>ref-190457	CCTCCTTCTACCCTTTCTCCTTCTCTA	12	<lmxll>	ll	18	lm	243		
>ref-190457	CCTCCTTCTACCCTTTCTCCTTCTCTA	13	<nnxnp>	nn	221	np	40		
>ref-190457	CCTCCTTCTACCCTTTCTCCTTCTCTA	22	<lmxll>	ll	76	lm	179		
>ref-190457	CCTCCTTCTACCCTTTCTCCTTCTCTA	26	<hkxhk>	hh	0	hk	214	kk	6
>ref-190457	CCTCCTTCTACCCTTTCTCCTTCTCTA	27	<hkxhk>	hh	3	hk	270	kk	0
>ref-190480	CTCTTACACACACTATCTCCTCCTCCT	8	<lmxll>	ll	6	lm	260		
>ref-190480	CTCTTACACACACTATCTCCTCCTCCT	15	<lmxll>	ll	6	lm	260		
>ref-190496	ATGATATCAACAACAACTCCAAAGACA	6	<nnxnp>	nn	131	np	123		
>ref-190496	ATGATATCAACAACAACTCCAAAGACA	26	<nnxnp>	nn	266	np	0		
>ref-190496	ATGATATCAACAACAACTCCAAAGACA	27	<nnxnp>	nn	266	np	0		

>ref-190541	CAGTACTACACACTTTCTCCATCGCTG	12	<lmxll>	ll	135	lm	127		
>ref-190554	CAGCCGGGGACGCAGCCTCCTTGCAAC	2	<lmxll>	ll	138	lm	133		
>ref-190576	TGCCTGTTTACTCTCCCTCCTTCTCTC	6	<nnxnp>	nn	124	np	138		
>ref-190587	CCCCCACTCACTCTCACTCCTCATCAT	13	<nnxnp>	nn	131	np	120		
>ref-190592	TCTCCTCCAACCCCTCCTCCCTCTTCA	25	<hkxhk>	hh	0	hk	178	kk	54
>ref-190596	CTCTTCCTTACTCCCCCTCCTTTCTTC	21	<nnxnp>	nn	120	np	119		
>ref-190596	CTCTTCCTTACTCCCCCTCCTTTCTTC	23	<nnxnp>	nn	247	np	0		
>ref-190606	CCTGCCCCTACCGCCCCTCCTTCCCTG	9	<lmxll>	ll	268	lm	0		
>ref-190645	AGTAATGCAACTTAGTCTCCTCAGTTG	15	<nnxnp>	nn	144	np	121		
>ref-190655	AGACCTGTTACCTGCACTCCTGGAAAA	6	<hkxhk>	hh	57	hk	146	kk	49
>ref-190655	AGACCTGTTACCTGCACTCCTGGAAAA	8	<hkxhk>	hh	57	hk	146	kk	49
>ref-190667	ACTCCCCCTACACCCACTCCCCCTACA	7	<nnxnp>	nn	219	np	39		
>ref-190667	ACTCCCCCTACACCCACTCCCCCTACA	15	<nnxnp>	nn	249	np	3		
>ref-190667	ACTCCCCCTACACCCACTCCCCCTACA	25	<lmxll>	ll	87	lm	178		
>ref-190676	ATACCCTGCACTCACCCTCCTTCCAGG	8	<lmxll>	ll	153	lm	118		
>ref-190684	CCGTCTGCCACTTATTCTCCTCCCTTC	23	<hkxhk>	hh	52	hk	130	kk	47
>ref-190686	TCACAGAGCACTCTCTCTCCTTCTGTT	26	<lmxll>	ll	132	lm	129		
>ref-190689	CTTACCCTTACCCTTACTCCTGCCCAT	26	<lmxll>	ll	112	lm	136		
>ref-190698	TCCCGGCGTACATTCCCTCCCGGATTC	4	<lmxll>	ll	126	lm	94		
>ref-190698	TCCCGGCGTACATTCCCTCCCGGATTC	6	<nnxnp>	nn	110	np	112		
>ref-190722	TGACGTGTTACATTTCCTCCTCCAGGT	27	<lmxll>	ll	13	lm	255		
>ref-190736	AAATAGGTTACACACTCTCCTTCTTTG	4	<nnxnp>	nn	106	np	130		
>ref-190750	TGACATCTTACCAGCTCTCCTTCAGAA	21	<lmxll>	ll	1	lm	266		
>ref-190750	TGACATCTTACCAGCTCTCCTTCAGAA	24	<lmxll>	ll	1	lm	266		
>ref-190794	GACCCCACAACTCTCCCTCCCCCCCTC	21	<nnxnp>	nn	117	np	125		
>ref-190794	GACCCCACAACTCTCCCTCCCCCCCTC	26	<nnxnp>	nn	118	np	125		
>ref-190810	GGTCATATTACGGGATCTCCGGAGAAA	7	<lmxll>	ll	128	lm	115		
>ref-190840	TTTCTCATCACCTTCTCTCCCCTTCAT	7	<hkxhk>	hh	0	hk	270	kk	1
>ref-190848	TCGCTCCCCACCACTTCTCCTTTCCCT	4	<nnxnp>	nn	252	np	0		
>ref-190848	TCGCTCCCCACCACTTCTCCTTTCCCT	12	<lmxll>	ll	110	lm	143		
>ref-190853	ACGTGCCATACATCTTCTCCTTCATGG	22	<hkxhk>	hh	0	hk	273	kk	0
>ref-190853	ACGTGCCATACATCTTCTCCTTCATGG	27	<hkxhk>	hh	0	hk	273	kk	0
>ref-190890	ATAGAGTATACTGAATCTCCGTAGTTA	25	<lmxll>	ll	152	lm	105		
>ref-190912	TCCCTCTTTACCCTCTCTCCAATCCCT	5	<lmxll>	ll	123	lm	125		
>ref-190971	CAAACGGCCACTTAAGCTCCCGTAATG	27	<lmxll>	ll	110	lm	125		
>ref-190980	ACCAAAGGCACCTCCGCTCCTCGCCTC	2	<nnxnp>	nn	119	np	150		
>ref-190992	CCCTAATTAACGGAATCTCCCCGGTAT	25	<nnxnp>	nn	107	np	118		
>ref-191001	TCTGTCTCGACTTGTCCTCCCTTGCTT	25	<nnxnp>	nn	138	np	114		

>ref-191008	CTCCATTCCACTCTTTCTCCTCCTTTT	22	<lmxll>	ll	137	lm	115		
>ref-191010	CGCGCTATTACGCCGGCTCCTACGCGG	14	<nnxnp>	nn	263	np	0		
>ref-191010	CGCGCTATTACGCCGGCTCCTACGCGG	15	<nnxnp>	nn	263	np	0		
>ref-191010	CGCGCTATTACGCCGGCTCCTACGCGG	27	<nnxnp>	nn	263	np	0		
>ref-191025	TTACATACTACCCTACCTCCTCCCTTC	9	<lmxll>	ll	114	lm	144		
>ref-191043	ATATACACAACCTTCTCTCCTTCTTTA	3	<hkxhk>	hh	61	hk	143	kk	51
>ref-191067	ACCTATAAAACTCCTCCTCCTCCTCCA	27	<lmxll>	ll	126	lm	135		
>ref-191084	TTATCTATTACCGTTCCTCCAATGATT	16	<lmxll>	ll	154	lm	95		
>ref-191084	TTATCTATTACCGTTCCTCCAATGATT	24	<lmxll>	ll	154	lm	96		
>ref-191086	TCTTATCCTACTTTTCCTCCCCTTCTT	12	<lmxll>	ll	135	lm	120		
>ref-191096	TGCTGTACGACAACCTCTCCCTCCTCC	13	<nnxnp>	nn	125	np	146		
>ref-191107	TTATTGCCAACACTGTCTCCATTGACA	26	<lmxll>	ll	125	lm	144		
>ref-191112	TCCTTCCCCACCTTACCTCCTCACCCT	13	<nnxnp>	nn	1	np	260		
>ref-191130	CCTCTATACACTGCCTCTCCGCTCCAT	5	<lmxll>	ll	115	lm	144		
>ref-191178	TTGCCCTAGACCATTACTCCGTGACCA	12	<lmxll>	ll	112	lm	117		
>ref-191179	TCTTTTGACACTCTAACTCCAGACACT	26	<lmxll>	ll	120	lm	111		
>ref-191207	CCAGTGCATACACGCACTCCTCAAGTT	25	<nnxnp>	nn	104	np	152		
>ref-191221	CCACCGCTGACTCTTGCTCCTTGTGAA	14	<nnxnp>	nn	128	np	135		
>ref-191221	CCACCGCTGACTCTTGCTCCTTGTGAA	23	<lmxll>	ll	127	lm	129		
>ref-191225	TCCCACTCGACCTCTCCTCCATCTTCT	5	<nnxnp>	nn	115	np	137		
>ref-191234	CAGAAGCAGACGAAGTCTCCCAACGAG	8	<nnxnp>	nn	122	np	143		
>ref-191237	CCACCACTCACAACCTCTCCACTGTCC	5	<nnxnp>	nn	98	np	142		
>ref-191237	CCACCACTCACAACCTCTCCACTGTCC	7	<nnxnp>	nn	46	np	206		
>ref-191237	CCACCACTCACAACCTCTCCACTGTCC	24	<lmxll>	ll	2	lm	271		
>ref-191238	CATCCTCTTACTCATACTCCTCATCAG	23	<nnxnp>	nn	92	np	141		
>ref-191238	CATCCTCTTACTCATACTCCTCATCAG	27	<nnxnp>	nn	90	np	141		
>ref-191251	CCAAGCCATACACTTTCTCCACGTATT	3	<lmxll>	ll	106	lm	147		
>ref-191261	CTGGGCCCTACCTTCCCTCCGGACTGA	2	<hkxhk>	hh	59	hk	152	kk	52
>ref-191309	GGCCCTAGTACGTGTCCTCCGCATCTA	13	<lmxll>	ll	66	lm	201		
>ref-191311	CGCACGCCCACGCTCTCTCCCTCGCTT	27	<lmxll>	ll	133	lm	129		
>ref-191316	GCATATCGGACTTATCCTCCTACTCTC	5	<nnxnp>	nn	139	np	128		
>ref-191327	CACAAATAAACGGTTTCTCCAGTTGCT	3	<nnxnp>	nn	115	np	124		
>ref-191371	TCTTTCATTACCTGCTCTCCCTTGTAT	15	<nnxnp>	nn	154	np	103		
>ref-191375	CCTCCTCACACACCCTCTCCTTACCCT	16	<lmxll>	ll	4	lm	264		
>ref-191404	CGCACACGCACACGACCTCCTGCCCCG	22	<lmxll>	ll	261	lm	0		
>ref-191440	TTTCTTCCAACTGACCCTCCTCCCCTC	14	<nnxnp>	nn	125	np	105		
>ref-191449	AAAAGATGTACCTTCACTCCATGATGA	14	<nnxnp>	nn	86	np	166		
>ref-191449	AAAAGATGTACCTTCACTCCATGATGA	15	<nnxnp>	nn	240	np	22		

>ref-191482	CTTCAACACACACACACTCCCTCATCC	14	<nnxnp>	nn	123	np	143		
>ref-191490	TTGTTTTTAACTTCTCCTCCCCTGCCG	26	<nnxnp>	nn	131	np	132		
>ref-191513	ACTTTCAGTACACAAACTCCGCCACGT	6	<nnxnp>	nn	137	np	133		
>ref-191520	AGCTTCCCTACTCTCTCTCCTTCCCTC	2	<lmxll>	ll	266	lm	0		
>ref-191540	CCGTCCACCACCCTCCCTCCCTCGCTC	23	<nnxnp>	nn	121	np	141		
>ref-191547	ACCAACATAACGCACCCTCCACGAAAG	23	<nnxnp>	nn	119	np	100		
>ref-191548	CAAATTCCTACATTCTCTCCTTGTCTC	22	<nnxnp>	nn	113	np	143		
>ref-191557	GTCGTAGGGACCTCCCCTCCTCTATTT	21	<hkxhk>	hh	64	hk	120	kk	55
>ref-191565	CGCTCCTCAACCCCCACTCCATCTTCT	8	<nnxnp>	nn	138	np	134		
>ref-191586	TCATACAACACACATGCTCCTACACAC	6	<lmxll>	ll	132	lm	137		
>ref-191603	TCTCCTGGCACTTCCCCTCCCTCCCCG	26	<lmxll>	ll	267	lm	0		
>ref-191606	TCCCCCGCTACACTCTCTCCCCTCTTC	21	<nnxnp>	nn	88	np	137		
>ref-191637	CAATACTTTACTCTATCTCCCCGACAA	3	<nnxnp>	nn	261	np	0		
>ref-191657	TATCTCCCTACCTCCACTCCCTTCCCC	16	<nnxnp>	nn	42	np	213		
>ref-191657	TATCTCCCTACCTCCACTCCCTTCCCC	22	<nnxnp>	nn	45	np	210		
>ref-191660	CCTGATCGCACCCCTCCTCCCCCTTCT	25	<lmxll>	ll	146	lm	104		
>ref-191661	TTATTTGTCACGTTTTCTCCTGCAGAA	4	<lmxll>	ll	100	lm	135		
>ref-191661	TTATTTGTCACGTTTTCTCCTGCAGAA	5	<lmxll>	ll	101	lm	135		
>ref-191685	CACCGGATTACGTTGCCTCCCTCTTCC	12	<nnxnp>	nn	119	np	147		
>ref-191722	CTCTCTGCCACCTCCCCTCCCCCACCT	23	<nnxnp>	nn	135	np	135		
>ref-191724	TGACTATCTACCTAGCCTCCTATGCAT	23	<nnxnp>	nn	113	np	143		
>ref-191727	TCCTTCTAAACTTCCTCTCCTCTCCTC	22	<lmxll>	ll	46	lm	215		
>ref-191727	TCCTTCTAAACTTCCTCTCCTCTCCTC	27	<nnxnp>	nn	234	np	30		
>ref-191733	CCTCACCCGACCGACTCTCCCTTTGTG	7	<nnxnp>	nn	135	np	135		
>ref-191734	ACTCGCGAGACCTTCGCTCCAGACCAG	6	<nnxnp>	nn	128	np	140		
>ref-191749	GGGCGCGGCACGCCAGCTCCACCACCA	25	<hkxhk>	hh	0	hk	248	kk	0
>ref-191753	CACGCGCGCACTTACTCTCCCTCTTTG	2	<hkxhk>	hh	54	hk	143	kk	63
>ref-191764	ATCAAAGTCACATGCCCTCCGCTATTA	15	<lmxll>	ll	143	lm	130		
>ref-191776	GACACACACACCATATCTCCCATTGTT	1	<nnxnp>	nn	115	np	153		
>ref-191783	ATCGTGGAAACCACACCTCCTCGAGAG	21	<lmxll>	ll	126	lm	138		
>ref-191787	AACAACAAAACGGTCCCTCCAACATGC	12	<nnxnp>	nn	261	np	0		
>ref-191788	CATTCTCCTACTTCAGCTCCCCCTCCC	3	<hkxhk>	hh	55	hk	142	kk	54
>ref-191807	CGGATCCACACCCTTTCTCCTTCCCTC	22	<lmxll>	ll	133	lm	129		
>ref-191808	CATTCCCTTACCTCCACTCCCTTCCCT	2	<nnxnp>	nn	36	np	214		
>ref-191808	CATTCCCTTACCTCCACTCCCTTCCCT	16	<nnxnp>	nn	35	np	212		
>ref-191828	ACGTAACAAACTGTCACTCCTTATCTG	9	<nnxnp>	nn	126	np	140		
>ref-191862	TCGCCTTTCACTCTCGCTCCCTCCCCT	2	<hkxhk>	hh	0	hk	273	kk	0
>ref-191862	TCGCCTTTCACTCTCGCTCCCTCCCCT	5	<hkxhk>	hh	0	hk	273	kk	0

>ref-191883	AATATAATTACACGAACTCCGTATACA	13	<nnxnp>	nn	95	np	134		
>ref-191884	TCCCTCCTAACCTCTTCTCCCCTTTTC	1	<hkxhk>	hh	43	hk	176	kk	0
>ref-191897	CCCCCTTTCACCCTCACTCCTGCTCAG	4	<lmxll>	ll	115	lm	135		
>ref-191899	ATTTCTTCAACATCCTCTCCTCCCTAA	21	<lmxll>	ll	253	lm	1		
>ref-191914	TGCAGAAGGACTCAAACTCCAGTGCCT	14	<nnxnp>	nn	258	np	0		
>ref-191919	CTATTTAGCACTGGTCCTCCGGGTCAT	1	<hkxhk>	hh	0	hk	270	kk	0
>ref-191919	CTATTTAGCACTGGTCCTCCGGGTCAT	23	<hkxhk>	hh	0	hk	270	kk	0
>ref-191919	CTATTTAGCACTGGTCCTCCGGGTCAT	25	<nnxnp>	nn	165	np	90		
>ref-191929	TGAATGAATACCTATCCTCCAAAAACG	14	<hkxhk>	hh	7	hk	259	kk	0
>ref-191929	TGAATGAATACCTATCCTCCAAAAACG	21	<hkxhk>	hh	124	hk	103	kk	0
>ref-191929	TGAATGAATACCTATCCTCCAAAAACG	25	<nnxnp>	nn	194	np	45		
>ref-191937	TGTCGCTACACGGTTTCTCCTTCTCCT	8	<lmxll>	ll	129	lm	143		
>ref-191952	CTCCCCTTTACATCCTCTCCCTCCCTT	13	<lmxll>	ll	38	lm	200		
>ref-191952	CTCCCCTTTACATCCTCTCCCTCCCTT	24	<lmxll>	ll	140	lm	126		
>ref-191952	CTCCCCTTTACATCCTCTCCCTCCCTT	25	<lmxll>	ll	38	lm	197		
>ref-191981	ACGCTAACAACAGTCCCTCCTTGCCAG	6	<lmxll>	ll	116	lm	144		
>ref-192006	ATTCATCGCACCAATACTCCTTCGTAT	15	<nnxnp>	nn	112	np	127		
>ref-192039	CCCTAGGGCACACGACCTCCATCCTTG	22	<hkxhk>	hh	65	hk	162	kk	39
>ref-192048	TCCTCCCCAACCCCCTCTCCGCAATCA	4	<lmxll>	ll	254	lm	0		
>ref-192077	TCCCACATCACTCACCCTCCTGACGTC	24	<lmxll>	ll	152	lm	114		
>ref-192096	GAGAACCTCACTCTACCTCCACCAATT	27	<lmxll>	ll	134	lm	127		
>ref-192103	AAACAAAGCACTCCTCCTCCTCCTCCC	9	<nnxnp>	nn	137	np	125		
>ref-192103	AAACAAAGCACTCCTCCTCCTCCTCCC	27	<nnxnp>	nn	125	np	131		
>ref-192107	TTATTTGTGACTCGCTCTCCACTTATC	7	<nnxnp>	nn	136	np	129		
>ref-192108	AAGCAGTTAACCATCTCTCCTTGCAGA	12	<hkxhk>	hh	0	hk	273	kk	0
>ref-192108	AAGCAGTTAACCATCTCTCCTTGCAGA	15	<lmxll>	ll	117	lm	137		
>ref-192117	GCAGTGGAAACCGTCGCTCCATCACCT	1	<nnxnp>	nn	141	np	126		
>ref-192125	ATCAGGCATACCATCTCTCCCTGACAC	15	<lmxll>	ll	138	lm	130		
>ref-192125	ATCAGGCATACCATCTCTCCCTGACAC	22	<lmxll>	ll	137	lm	130		
>ref-192129	CTCGGGCTAACTACCTCTCCAATTACG	15	<lmxll>	ll	123	lm	113		
>ref-192141	CCTTACCTCACTCCATCTCCCTTGATT	5	<nnxnp>	nn	127	np	131		
>ref-192141	CCTTACCTCACTCCATCTCCCTTGATT	9	<hkxhk>	hh	66	hk	150	kk	41
>ref-192142	GCTGTTATAACCACCTCTCCGTTCTCT	6	<lmxll>	ll	135	lm	129		
>ref-192161	ACCGACACTACAGCGACTCCAACTAGC	14	<nnxnp>	nn	270	np	1		
>ref-192164	TTAGAATGAACTTCGGCTCCCTTTAAA	14	<lmxll>	ll	112	lm	141		
>ref-192180	CTCTCCCACACTCCCTCTCCTTTCTTT	6	<lmxll>	ll	81	lm	154		
>ref-192180	CTCTCCCACACTCCCTCTCCTTTCTTT	14	<nnxnp>	nn	252	np	0		
>ref-192180	CTCTCCCACACTCCCTCTCCTTTCTTT	16	<nnxnp>	nn	262	np	0		

>ref-192198	TGAACGCGGACTCACGCTCCCATACAC	3	<hkxhk>	hh	7	hk	265	kk	0
>ref-192198	TGAACGCGGACTCACGCTCCCATACAC	7	<hkxhk>	hh	0	hk	273	kk	0
>ref-192224	CCTTCCCCTACACCATCTCCCCAACAT	1	<hkxhk>	hh	3	hk	266	kk	0
>ref-192224	CCTTCCCCTACACCATCTCCCCAACAT	8	<hkxhk>	hh	12	hk	255	kk	0
>ref-192224	CCTTCCCCTACACCATCTCCCCAACAT	13	<hkxhk>	hh	0	hk	254	kk	12
>ref-192225	AAAGGTTGGACAATACCTCCTGTCTGG	21	<lmxll>	ll	126	lm	124		
>ref-192267	CCCTCCCCAACCCCATCTCCCCAACCT	5	<nnxnp>	nn	265	np	0		
>ref-192267	CCCTCCCCAACCCCATCTCCCCAACCT	16	<hkxhk>	hh	71	hk	155	kk	0
>ref-192285	ATTGTGGTGACTGAAGCTCCTATCATT	2	<hkxhk>	hh	14	hk	243	kk	2
>ref-192285	ATTGTGGTGACTGAAGCTCCTATCATT	16	<hkxhk>	hh	14	hk	244	kk	2
>ref-192295	CCTGTTCCTACAGGCCCTCCCCCCTTG	24	<nnxnp>	nn	133	np	120		
>ref-192334	CCGTCTTTAACAACAGCTCCACCGTTA	26	<hkxhk>	hh	42	hk	129	kk	62
>ref-192337	CGCCTACCAACCCTCTCTCCTCTCCAC	24	<nnxnp>	nn	123	np	137		
>ref-192337	CGCCTACCAACCCTCTCTCCTCTCCAC	25	<lmxll>	ll	137	lm	121		
>ref-192341	AAACAATATACATTGTCTCCGTCGGAT	25	<nnxnp>	nn	130	np	139		
>ref-192367	ACATACGATACCGTTGCTCCTCTGAGA	13	<lmxll>	ll	121	lm	131		
>ref-192370	AATACAGACACACGCTCTCCCTCCCTC	4	<hkxhk>	hh	85	hk	127	kk	52
>ref-192370	AATACAGACACACGCTCTCCCTCCCTC	24	<nnxnp>	nn	124	np	143		
>ref-192374	TTCACCTGAACTGCCGCTCCCTTCCCC	2	<lmxll>	ll	114	lm	132		
>ref-192374	TTCACCTGAACTGCCGCTCCCTTCCCC	16	<nnxnp>	nn	125	np	132		
>ref-192379	AAACGTTAAACTTTTTCTCCTTTCTCT	12	<nnxnp>	nn	120	np	136		
>ref-192399	TTCTACCTCACTCTGTCTCCTCTCCTC	2	<lmxll>	ll	115	lm	130		
>ref-192416	GATATTTGGACTATTCCTCCTCAACAG	21	<hkxhk>	hh	44	hk	131	kk	61
>ref-192422	TCCATCTCCACAGGAACTCCAAGCCCC	23	<nnxnp>	nn	136	np	129		
>ref-192422	TCCATCTCCACAGGAACTCCAAGCCCC	25	<nnxnp>	nn	136	np	129		
>ref-192440	TTCTCTATTACTCCCCCTCCGCCCGAG	16	<nnxnp>	nn	135	np	124		
>ref-192440	TTCTCTATTACTCCCCCTCCGCCCGAG	26	<lmxll>	ll	139	lm	124		
>ref-192449	ATTTCTTCCACTTCCCCTCCACTCTCT	1	<lmxll>	ll	17	lm	236		
>ref-192449	ATTTCTTCCACTTCCCCTCCACTCTCT	9	<lmxll>	ll	17	lm	235		
>ref-192449	ATTTCTTCCACTTCCCCTCCACTCTCT	12	<hkxhk>	hh	0	hk	180	kk	61
>ref-192449	ATTTCTTCCACTTCCCCTCCACTCTCT	21	<hkxhk>	hh	61	hk	181	kk	0
>ref-192452	CACTTATAAACGCCCCCTCCTCCTCTT	8	<nnxnp>	nn	105	np	142		
>ref-192464	TGACCAATAACTAATACTCCGATCCCT	6	<nnxnp>	nn	122	np	139		
>ref-192479	TCACGAATCACACGATCTCCAGGGATT	15	<hkxhk>	hh	63	hk	208	kk	0
>ref-192479	TCACGAATCACACGATCTCCAGGGATT	21	<hkxhk>	hh	63	hk	208	kk	0
>ref-192482	ACCATCACCACGATTCCTCCTCTCCAC	3	<nnxnp>	nn	108	np	140		
>ref-192482	ACCATCACCACGATTCCTCCTCTCCAC	14	<nnxnp>	nn	133	np	119		
>ref-192482	ACCATCACCACGATTCCTCCTCTCCAC	24	<lmxll>	ll	121	lm	132		

>ref-192507	AGCAGCCAAACAGTGCCTCCATCCTAC	1	<nnxnp>	nn	136	np	134		
>ref-192508	CCTAGCCCCACCTCCTCTCCTTCCTTA	5	<lmxll>	ll	100	lm	148		
>ref-192508	CCTAGCCCCACCTCCTCTCCTTCCTTA	7	<lmxll>	ll	100	lm	148		
>ref-192515	CCTCCCTCCACCCGGCCTCCATCCCTC	2	<nnxnp>	nn	3	np	235		
>ref-192521	TCCTCCTGGACTTCATCTCCAGGTCTG	12	<nnxnp>	nn	161	np	102		
>ref-192521	TCCTCCTGGACTTCATCTCCAGGTCTG	15	<hkxhk>	hh	2	hk	258	kk	5
>ref-192527	GGAGATCATACGTTCCCTCCAAGTGCT	13	<nnxnp>	nn	126	np	143		
>ref-192532	ATCTCGGTCACTCTCTCTCCCTGTTTC	27	<lmxll>	ll	150	lm	101		
>ref-192535	ACACGCCATACCACCACTCCGCCATCT	6	<lmxll>	ll	133	lm	134		
>ref-192538	CAGGACGAGACTGAAACTCCAGCGGTT	9	<lmxll>	ll	139	lm	133		
>ref-192552	AGCACTTCCACCCCCCCTCCTCCTCCT	3	<nnxnp>	nn	244	np	0		
>ref-192552	AGCACTTCCACCCCCCCTCCTCCTCCT	4	<nnxnp>	nn	216	np	3		
>ref-192552	AGCACTTCCACCCCCCCTCCTCCTCCT	6	<nnxnp>	nn	218	np	9		
>ref-192565	GAAAGCATAACACTCTCTCCAATACCT	1	<lmxll>	ll	155	lm	76		
>ref-192565	GAAAGCATAACACTCTCTCCAATACCT	26	<hkxhk>	hh	83	hk	97	kk	49
>ref-192566	GGGGTTATGACCTCATCTCCTTCGTCT	1	<lmxll>	ll	125	lm	143		
>ref-192574	GTTCTCTTCACCCCTTCTCCTTCTTCT	27	<lmxll>	ll	113	lm	143		
>ref-192581	CATCACTCTACTCATCCTCCTTTCCTG	6	<lmxll>	ll	117	lm	145		
>ref-192590	AATAGGTTAACTTGATCTCCCTCCGTT	25	<hkxhk>	hh	51	hk	146	kk	65
>ref-192608	CATGCACACACTCTCTCTCCTCACATG	12	<nnxnp>	nn	51	np	219		
>ref-192613	TCTGTTCACACCAGTCCTCCCTCTCCC	27	<nnxnp>	nn	123	np	137		
>ref-192633	ACCAACACGACGTCTTCTCCAATGGAC	15	<hkxhk>	hh	63	hk	139	kk	41
>ref-192638	GGGGAATGAACGTCTGCTCCTGCAGGG	27	<nnxnp>	nn	109	np	148		
>ref-192651	GTCTCTCTCACTCTTTCTCCCCACCTT	8	<lmxll>	ll	131	lm	134		
>ref-192657	CTTTGTTTCACTGTAACTCCCCCGCCA	25	<nnxnp>	nn	132	np	131		
>ref-192666	CTTCCTCTTACCTCCACTCCTTATTCC	2	<nnxnp>	nn	128	np	125		
>ref-192671	CACGTACGTACACCCTCTCCCTCATAC	8	<hkxhk>	hh	0	hk	265	kk	0
>ref-192671	CACGTACGTACACCCTCTCCCTCATAC	12	<hkxhk>	hh	1	hk	272	kk	0
>ref-192671	CACGTACGTACACCCTCTCCCTCATAC	23	<hkxhk>	hh	0	hk	273	kk	0
>ref-192673	TCTTCGCCAACCCCATCTCCTTCGTCA	3	<nnxnp>	nn	125	np	143		
>ref-192714	TTAGGGAGTACTGGGCCTCCTGTATGG	24	<nnxnp>	nn	240	np	0		
>ref-192750	CATTTAAAAACCGCCTCTCCAGACGAC	6	<lmxll>	ll	128	lm	118		
>ref-192790	AACACATAAACTTTCTCTCCCAACTCC	4	<hkxhk>	hh	6	hk	124	kk	101
>ref-192823	CCTTTCCTCACTTGCTCTCCCTCTCAT	26	<nnxnp>	nn	135	np	133		
>ref-192846	CACATATATACACACTCTCCTTCCCCA	24	<lmxll>	ll	126	lm	128		
>ref-192850	GTGAAGTTGACTAGCTCTCCTCAGGAC	13	<nnxnp>	nn	115	np	137		
>ref-192858	TCTTCTTCTACGCTCCCTCCTTTCCTC	5	<lmxll>	ll	126	lm	123		
>ref-192862	TTCCCCTCCACACTACCTCCGCCTCGT	2	<nnxnp>	nn	118	np	143		

>ref-192862	TTCCCCTCCACACTACCTCCGCCTCGT	5	<nnxnp>	nn	126	np	139		
>ref-192863	CCTAACTGCACACGCACTCCCTTTTTC	26	<hkxhk>	hh	80	hk	140	kk	39
>ref-192863	CCTAACTGCACACGCACTCCCTTTTTC	27	<hkxhk>	hh	39	hk	140	kk	80
>ref-192883	CCCTCCTTCACTCCTTCTCCCTGGCTT	8	<hkxhk>	hh	2	hk	192	kk	67
>ref-192883	CCCTCCTTCACTCCTTCTCCCTGGCTT	23	<hkxhk>	hh	66	hk	192	kk	2
>ref-192884	ATTCCATCCACTCCCTCTCCACTTACT	1	<hkxhk>	hh	1	hk	264	kk	5
>ref-192884	ATTCCATCCACTCCCTCTCCACTTACT	3	<hkxhk>	hh	5	hk	264	kk	1
>ref-192897	CACTTAATGACTCGCTCTCCCCACATC	16	<lmxll>	ll	126	lm	136		
>ref-192902	TTCCTCCCCACCCCCTCTCCTTTCCTC	8	<nnxnp>	nn	127	np	129		
>ref-192902	TTCCTCCCCACCCCCTCTCCTTTCCTC	14	<lmxll>	ll	88	lm	157		
>ref-192902	TTCCTCCCCACCCCCTCTCCTTTCCTC	21	<lmxll>	ll	23	lm	230		
>ref-192903	GTATGGGTCACTACAACTCCTCCCCAC	1	<hkxhk>	hh	15	hk	249	kk	0
>ref-192931	CCAGAAGCAACACTTACTCCACCAATG	25	<lmxll>	ll	149	lm	96		
>ref-192931	CCAGAAGCAACACTTACTCCACCAATG	26	<lmxll>	ll	149	lm	96		
>ref-192943	CACGCACACACACAATCTCCCCCACAG	6	<nnxnp>	nn	115	np	127		
>ref-192944	GAGGTGAACACGACGCCTCCAGATGAG	15	<lmxll>	ll	125	lm	130		
>ref-192946	CTTCTTCTCACGTCCTCTCCTCTTTGT	23	<nnxnp>	nn	120	np	141		
>ref-192947	CTAAAAGAAACCTAAACTCCCTAAAAC	2	<lmxll>	ll	162	lm	82		
>ref-192947	CTAAAAGAAACCTAAACTCCCTAAAAC	6	<lmxll>	ll	162	lm	80		
>ref-192947	CTAAAAGAAACCTAAACTCCCTAAAAC	23	<hkxhk>	hh	144	hk	87	kk	0
>ref-192950	CTCCTTCCGACCTTGGCTCCCGTAGCA	25	<nnxnp>	nn	118	np	123		
>ref-192955	CATCCCCAGACGCTACCTCCCCGTCCC	1	<lmxll>	ll	12	lm	258		
>ref-192955	CATCCCCAGACGCTACCTCCCCGTCCC	14	<hkxhk>	hh	0	hk	273	kk	0
>ref-192975	CTCTTCCTTACTTCTCCTCCCCTTTTC	9	<hkxhk>	hh	0	hk	271	kk	0
>ref-192975	CTCTTCCTTACTTCTCCTCCCCTTTTC	22	<hkxhk>	hh	80	hk	169	kk	0
>ref-192975	CTCTTCCTTACTTCTCCTCCCCTTTTC	23	<hkxhk>	hh	0	hk	265	kk	2
>ref-192982	CACACACACACGCAACCTCCTCACACT	5	<nnxnp>	nn	140	np	126		
>ref-192997	CTATCACCAACACTCTCTCCATCACCT	2	<lmxll>	ll	140	lm	128		
>ref-193012	CCCTCTACCACCCTCCCTCCCCTTCAT	15	<nnxnp>	nn	167	np	97		
>ref-193033	ATCGGTACAACGTTTTCTCCGAGTCAT	5	<lmxll>	ll	126	lm	132		
>ref-193057	ACGCTCCGAACCGTCACTCCCTCTCAG	12	<nnxnp>	nn	129	np	143		
>ref-193075	TTCTGAGTGACGCTTTCTCCTTCATCC	5	<lmxll>	ll	117	lm	126		
>ref-193090	GTCTGAAAAACGGAGACTCCAACTCAC	5	<nnxnp>	nn	131	np	120		
>ref-193112	CCCATTAAAACCCCTCCTCCCTCCCCT	23	<nnxnp>	nn	142	np	83		
>ref-193126	GGACTTCATACCACTGCTCCATACTCA	3	<hkxhk>	hh	0	hk	273	kk	0
>ref-193126	GGACTTCATACCACTGCTCCATACTCA	6	<hkxhk>	hh	0	hk	272	kk	0
>ref-193126	GGACTTCATACCACTGCTCCATACTCA	15	<hkxhk>	hh	0	hk	268	kk	2
>ref-193162	CCATCCCCCACCACCACTCCCGTCCCT	16	<hkxhk>	hh	13	hk	251	kk	0

>ref-193162	CCATCCCCCACCACCACTCCCGTCCCT	22	<hkxhk>	hh	0	hk	251	kk	13
>ref-193167	CCACTTCGTACCTCCCCTCCTCTCTCT	4	<hkxhk>	hh	2	hk	262	kk	1
>ref-193168	TGTACAGAAACTTGGCCTCCTCGCCAT	1	<hkxhk>	hh	0	hk	271	kk	1
>ref-193168	TGTACAGAAACTTGGCCTCCTCGCCAT	2	<hkxhk>	hh	0	hk	271	kk	1
>ref-193168	TGTACAGAAACTTGGCCTCCTCGCCAT	3	<hkxhk>	hh	0	hk	269	kk	2
>ref-193189	AGGTCACAGACTTTGACTCCACCGATG	12	<nnxnp>	nn	141	np	125		
>ref-193189	AGGTCACAGACTTTGACTCCACCGATG	15	<nnxnp>	nn	144	np	123		
>ref-193190	TCTGTGCGTACCCTGTCTCCCCCTTTA	2	<nnxnp>	nn	125	np	139		
>ref-193197	CGAGGCCGGACCAGCCCTCCGCTTGCT	21	<lmxll>	ll	123	lm	147		
>ref-193228	GGAACAAGCACAGAGGCTCCTGAAACC	26	<hkxhk>	hh	0	hk	273	kk	0
>ref-193230	ACTTCACCCACACCCCCTCCCTCCACC	1	<hkxhk>	hh	20	hk	203	kk	1
>ref-193230	ACTTCACCCACACCCCCTCCCTCCACC	23	<hkxhk>	hh	20	hk	203	kk	1
>ref-193234	GACTTATTTACGTAATCTCCGTTCTTT	27	<nnxnp>	nn	137	np	125		
>ref-193243	TCCTTTACCACCTCGCCTCCTCCTCTT	6	<hkxhk>	hh	1	hk	270	kk	1
>ref-193243	TCCTTTACCACCTCGCCTCCTCCTCTT	12	<hkxhk>	hh	1	hk	270	kk	1
>ref-193250	TCAAGTATCACTTCATCTCCAATTCTT	7	<lmxll>	ll	185	lm	72		
>ref-193255	CTGAAGGCGACCTAATCTCCAGGCTGA	14	<lmxll>	ll	118	lm	139		
>ref-193265	TCCGCGTCCACCCTCCCTCCTTTTCTT	4	<nnxnp>	nn	121	np	110		
>ref-193272	TATGCTGCGACACATTCTCCTTTATAC	24	<lmxll>	ll	100	lm	148		
>ref-193283	TATTGCAACACCCTCTCTCCTTCCCCT	23	<nnxnp>	nn	145	np	120		
>ref-193292	TACCTCAGGACTCCACCTCCATATCAA	5	<nnxnp>	nn	115	np	147		
>ref-193301	CCACTCAACACCTTCCCTCCTTACCCC	13	<nnxnp>	nn	113	np	131		
>ref-193303	GCTCAAGTCACACGTTCTCCTCACAAA	13	<lmxll>	ll	9	lm	252		
>ref-193303	GCTCAAGTCACACGTTCTCCTCACAAA	27	<lmxll>	ll	9	lm	252		
>ref-193327	ATTAATGCTACATAATCTCCCTTCCCA	3	<lmxll>	ll	113	lm	129		
>ref-193349	TTATTTTTCACCCTTTCTCCTTCGTCT	12	<lmxll>	ll	128	lm	119		
>ref-193349	TTATTTTTCACCCTTTCTCCTTCGTCT	13	<hkxhk>	hh	64	hk	118	kk	53
>ref-193352	TTTACATCTACTCTACCTCCCTTTCTC	27	<nnxnp>	nn	118	np	122		
>ref-193366	GAAGTTGCTACAAAAGCTCCTTCTCTC	5	<lmxll>	ll	125	lm	131		
>ref-193366	GAAGTTGCTACAAAAGCTCCTTCTCTC	12	<lmxll>	ll	123	lm	131		
>ref-193370	GCTGTCATCACTCCTCCTCCTCCTCCT	6	<nnxnp>	nn	258	np	2		
>ref-193370	GCTGTCATCACTCCTCCTCCTCCTCCT	23	<nnxnp>	nn	259	np	2		
>ref-193372	GGTTTAATAACATTCTCTCCGAGAACA	15	<hkxhk>	hh	1	hk	272	kk	0
>ref-193383	ACTGTTATTACCCTTTCTCCCTCTCCC	3	<hkxhk>	hh	0	hk	208	kk	63
>ref-193383	ACTGTTATTACCCTTTCTCCCTCTCCC	9	<hkxhk>	hh	1	hk	272	kk	0
>ref-193402	TCCCTTACAACCACAACTCCCCCGCCG	27	<lmxll>	ll	155	lm	111		
>ref-193428	CCTGCTCATACTTGTCCTCCTCTTTTT	22	<nnxnp>	nn	137	np	105		
>ref-193433	TCCCTCCTAACTCATCCTCCTCACTTG	3	<lmxll>	ll	3	lm	263		

>ref-193440	TACCCCCCAACCCCATCTCCCTTCCCT	26	<nnxnp>	nn	105	np	140		
>ref-193457	CCCACACACACTTAAGCTCCCACTCCA	2	<hkxhk>	hh	0	hk	256	kk	14
>ref-193457	CCCACACACACTTAAGCTCCCACTCCA	24	<hkxhk>	hh	0	hk	256	kk	14
>ref-193507	TCTTCATCCACCCTTTCTCCCCTCCCC	3	<nnxnp>	nn	101	np	122		
>ref-193507	TCTTCATCCACCCTTTCTCCCCTCCCC	13	<lmxll>	ll	216	lm	9		
>ref-193507	TCTTCATCCACCCTTTCTCCCCTCCCC	14	<nnxnp>	nn	100	np	123		
>ref-193507	TCTTCATCCACCCTTTCTCCCCTCCCC	24	<lmxll>	ll	216	lm	9		
>ref-193535	TCTGACACCACGTACTCTCCGACCCCA	15	<hkxhk>	hh	0	hk	13	kk	257
>ref-193535	TCTGACACCACGTACTCTCCGACCCCA	21	<hkxhk>	hh	256	hk	14	kk	0
>ref-193535	TCTGACACCACGTACTCTCCGACCCCA	22	<hkxhk>	hh	244	hk	25	kk	0
>ref-193537	ACAAACCACACTGGAGCTCCGTGATGC	23	<nnxnp>	nn	121	np	138		
>ref-193550	GCTTCCTTTACCAGTACTCCCTTGCTG	9	<nnxnp>	nn	268	np	0		
>ref-193562	CACCCCATAACTGACCCTCCCCTAAAG	15	<lmxll>	ll	120	lm	130		
>ref-193589	TTATGTACTACATATGCTCCCTCCCTC	6	<lmxll>	ll	122	lm	140		
>ref-193596	GCATACATTACGCTTTCTCCCTACACT	26	<nnxnp>	nn	109	np	139		
>ref-193599	ATCAATCCTACAGCTACTCCTCTTCAT	7	<lmxll>	ll	122	lm	126		
>ref-193602	CCCACGATGACTTTAGCTCCTGACAAG	27	<nnxnp>	nn	248	np	0		
>ref-193617	ATAAAATTAACACCAGCTCCTCGAGAC	9	<hkxhk>	hh	56	hk	109	kk	61
>ref-193637	GCACTTTCCACTCCTTCTCCCTCTCTC	4	<lmxll>	ll	262	lm	0		
>ref-193637	GCACTTTCCACTCCTTCTCCCTCTCTC	8	<lmxll>	ll	263	lm	0		
>ref-193650	CCTCCCCCTACATACCCTCCCACAGCG	13	<nnxnp>	nn	97	np	125		
>ref-193657	TACCTCTCTACACTCCCTCCTTCACCT	7	<hkxhk>	hh	44	hk	144	kk	72
>ref-193661	CCACAGCCCACGCCCACTCCCCCCTCA	2	<lmxll>	ll	48	lm	218		
>ref-193661	CCACAGCCCACGCCCACTCCCCCCTCA	23	<hkxhk>	hh	0	hk	273	kk	0
>ref-193673	CCCACCTTCACCCCCCCTCCTTCCCTT	26	<nnxnp>	nn	222	np	0		
>ref-193675	GTCATCTCCACTCCATCTCCATCTCTA	1	<hkxhk>	hh	4	hk	263	kk	0
>ref-193675	GTCATCTCCACTCCATCTCCATCTCTA	25	<hkxhk>	hh	3	hk	264	kk	0
>ref-193710	ATCAACAACACACTTCCTCCAATTGAT	21	<lmxll>	ll	104	lm	136		
>ref-193713	CACCTTCCCACTTTCCCTCCTCTCCAC	5	<hkxhk>	hh	38	hk	132	kk	76
>ref-193713	CACCTTCCCACTTTCCCTCCTCTCCAC	22	<hkxhk>	hh	78	hk	131	kk	33
>ref-193728	CTCTTTCCCACTCCTTCTCCCTCACAT	3	<hkxhk>	hh	212	hk	43	kk	0
>ref-193728	CTCTTTCCCACTCCTTCTCCCTCACAT	6	<hkxhk>	hh	1	hk	272	kk	0
>ref-193728	CTCTTTCCCACTCCTTCTCCCTCACAT	15	<hkxhk>	hh	0	hk	47	kk	210
>ref-193728	CTCTTTCCCACTCCTTCTCCCTCACAT	16	<hkxhk>	hh	0	hk	272	kk	0
>ref-193744	TGACAGGCTACCCAGCCTCCTTCAAGC	6	<hkxhk>	hh	0	hk	273	kk	0
>ref-193744	TGACAGGCTACCCAGCCTCCTTCAAGC	15	<hkxhk>	hh	0	hk	273	kk	0
>ref-193744	TGACAGGCTACCCAGCCTCCTTCAAGC	23	<lmxll>	ll	144	lm	124		
>ref-193756	TCTCCTCGTACCCCCTCTCCTCTCTCT	5	<lmxll>	ll	126	lm	131		

>ref-193771	GTGGGAGCGACGGAATCTCCTACGACA	5	<nnxnp>	nn	109	np	154		
>ref-193785	TAGTTTTTACTCCCTCTCCTTCTCCC	3	<lmxll>	ll	60	lm	177		
>ref-193785	TAGTTTTTACTCCCTCTCCTTCTCCC	5	<hkxhk>	hh	6	hk	252	kk	0
>ref-193785	TAGTTTTTACTCCCTCTCCTTCTCCC	16	<nnxnp>	nn	153	np	95		
>ref-193785	TAGTTTTTACTCCCTCTCCTTCTCCC	21	<hkxhk>	hh	6	hk	252	kk	0
>ref-193816	TTCATCGTCACTCTCCCTCCTCCTCTC	15	<hkxhk>	hh	0	hk	273	kk	0
>ref-193816	TTCATCGTCACTCTCCCTCCTCCTCTC	27	<hkxhk>	hh	8	hk	263	kk	0
>ref-193824	CAGTATTCAACAAGCTCTCCTTTGAAG	15	<lmxll>	ll	148	lm	94		
>ref-193838	AGCTACTTTACCTCGCCTCCTTCCCTC	14	<nnxnp>	nn	128	np	138		
>ref-193851	GCGATATGTACCGTGACTCCATGCTTA	9	<nnxnp>	nn	122	np	132		
>ref-193852	CTTTATTTTACCCTCCCTCCATCCATC	5	<nnxnp>	nn	153	np	109		
>ref-193866	GCACTCACAACCCCTTCTCCTTTTGCC	4	<lmxll>	ll	114	lm	105		
>ref-193873	ACCGGAACGACCCCATCTCCCTCTACC	15	<nnxnp>	nn	131	np	92		
>ref-193882	TTCTCACCTACTTTTCCTCCCTCTCCC	6	<nnxnp>	nn	215	np	4		
>ref-193882	TTCTCACCTACTTTTCCTCCCTCTCCC	12	<nnxnp>	nn	239	np	3		
>ref-193895	ATCTCACCTACTCTTTCTCCCCTTTCT	6	<hkxhk>	hh	6	hk	261	kk	0
>ref-193895	ATCTCACCTACTCTTTCTCCCCTTTCT	9	<hkxhk>	hh	0	hk	261	kk	6
>ref-193895	ATCTCACCTACTCTTTCTCCCCTTTCT	24	<hkxhk>	hh	3	hk	263	kk	1
>ref-193899	AACCCGCCCACCTTACCTCCTTCCCTT	2	<lmxll>	ll	2	lm	271		
>ref-193909	TCGTCCTCTACCTTCCCTCCCTACCAT	25	<nnxnp>	nn	147	np	119		
>ref-193925	TCAATATCCACCTTTCCTCCTCGTCTA	2	<nnxnp>	nn	139	np	124		
>ref-193947	TTCCCCTCAACCTGTCCTCCTTCCTCC	6	<hkxhk>	hh	1	hk	272	kk	0
>ref-193947	TTCCCCTCAACCTGTCCTCCTTCCTCC	9	<hkxhk>	hh	1	hk	272	kk	0
>ref-194004	GAGATCAGCACCCGGTCTCCTCCCGCC	7	<lmxll>	ll	120	lm	129		
>ref-194058	TTTCCCCCCACCTTCCCTCCTTCTCTA	13	<lmxll>	ll	241	lm	18		
>ref-194058	TTTCCCCCCACCTTCCCTCCTTCTCTA	25	<lmxll>	ll	241	lm	17		
>ref-194058	TTTCCCCCCACCTTCCCTCCTTCTCTA	26	<nnxnp>	nn	196	np	56		
>ref-194058	TTTCCCCCCACCTTCCCTCCTTCTCTA	27	<nnxnp>	nn	6	np	221		
>ref-194064	AGAGAGGCAACACGACCTCCCTGTATG	1	<nnxnp>	nn	146	np	126		
>ref-194082	CGGCCTTCAACTGCGCCTCCCGAATAC	21	<hkxhk>	hh	45	hk	139	kk	78
>ref-194092	TTCCCCCTAACTCCCTCTCCCACATTT	6	<nnxnp>	nn	103	np	118		
>ref-194130	CCCTGTCCTACTACTTCTCCATCTCTC	8	<nnxnp>	nn	135	np	135		
>ref-194142	CCCTTATTAACCCCCTCTCCTTCTTTC	7	<lmxll>	ll	123	lm	121		
>ref-194172	CCATACAACACAATCACTCCCCTCAAC	24	<nnxnp>	nn	129	np	131		
>ref-194178	CCCCAAAAAACCGCCGCTCCTTTGCAA	22	<nnxnp>	nn	131	np	115		
>ref-194183	ACAGACGCCACTCACACTCCAAGGAAA	5	<hkxhk>	hh	41	hk	145	kk	63
>ref-194187	CCTTAACCCACCTACCCTCCTGCTCTA	9	<lmxll>	ll	127	lm	135		
>ref-194198	AGGTCCCTCACTTCCCCTCCCCTCCTT	3	<nnxnp>	nn	220	np	0		

>ref-194198	AGGTCCCTCACTTCCCCTCCCCTCCTT	21	<nnxnp>	nn	222	np	1		
>ref-194262	CTTTTAACTACCACGCCTCCCTTTAGG	8	<lmxll>	ll	49	lm	193		
>ref-194262	CTTTTAACTACCACGCCTCCCTTTAGG	13	<hkxhk>	hh	63	hk	169	kk	0
>ref-194262	CTTTTAACTACCACGCCTCCCTTTAGG	23	<lmxll>	ll	49	lm	193		
>ref-194262	CTTTTAACTACCACGCCTCCCTTTAGG	27	<hkxhk>	hh	0	hk	171	kk	62
>ref-194268	TTATTCCTAACTCTCTCTCCATCTTTA	1	<nnxnp>	nn	92	np	134		
>ref-194295	TCCTCTCTAACTCCCTCTCCTTGATCT	1	<hkxhk>	hh	54	hk	138	kk	54
>ref-194329	TTCTTGTTCACTCTTTCTCCTGTCATT	4	<lmxll>	ll	116	lm	110		
>ref-194359	TAATCTAGGACGGATGCTCCACAATAA	24	<lmxll>	ll	125	lm	134		
>ref-194360	CATCTCAAAACAGCACCTCCAGCTATA	13	<lmxll>	ll	111	lm	132		
>ref-194391	TCCTCTGCTACAGATTCTCCCTCACTT	21	<lmxll>	ll	128	lm	135		
>ref-194394	GCCTCCTTAACGCAAGCTCCGCCAACC	9	<nnxnp>	nn	107	np	164		
>ref-194419	TGCTTTCTGACACCGCCTCCTGACACC	9	<nnxnp>	nn	273	np	0		
>ref-194422	CATCCATTTACTTCATCTCCCTTCTTC	4	<nnxnp>	nn	128	np	133		
>ref-194438	GAAAGACTGACGCAAGCTCCACAGTTA	5	<lmxll>	ll	125	lm	138		
>ref-194472	CAAACTTCTACCCCCACTCCAATCCCC	1	<lmxll>	ll	111	lm	128		
>ref-194485	ACACCAATCACTGCTGCTCCATGAGGA	5	<hkxhk>	hh	0	hk	273	kk	0
>ref-194485	ACACCAATCACTGCTGCTCCATGAGGA	9	<hkxhk>	hh	0	hk	273	kk	0
>ref-194487	TTCCTAACCACTTCAGCTCCATCGATG	5	<nnxnp>	nn	143	np	122		
>ref-194515	TGATGAACGACAGTCACTCCTCTGCAT	2	<nnxnp>	nn	138	np	129		
>ref-194522	CTCGCTTCCACCTTGTCTCCCACCCTC	23	<hkxhk>	hh	1	hk	270	kk	0
>ref-194524	TACAACCCAACAAGGCCTCCAGGTGGT	3	<lmxll>	ll	127	lm	122		
>ref-194550	TAAGGTTATACCCTCCCTCCTTCCCTC	2	<nnxnp>	nn	124	np	143		
>ref-194556	TCTTCTACCACCTCCTCTCCATCCCTC	5	<lmxll>	ll	262	lm	0		
>ref-194556	TCTTCTACCACCTCCTCTCCATCCCTC	13	<lmxll>	ll	137	lm	119		
>ref-194556	TCTTCTACCACCTCCTCTCCATCCCTC	21	<lmxll>	ll	250	lm	0		
>ref-194556	TCTTCTACCACCTCCTCTCCATCCCTC	26	<lmxll>	ll	134	lm	122		
>ref-194567	CGCTATTGCACGGAAGCTCCTTTCATG	8	<hkxhk>	hh	42	hk	123	kk	72
>ref-194570	CGTGAGCCAACCTAACCTCCTCTCTTT	22	<lmxll>	ll	121	lm	141		
>ref-194586	CCTTCTTCCACCTTTCCTCCTCGTCCG	1	<nnxnp>	nn	241	np	1		
>ref-194590	ACCATCTCTACCCTAGCTCCTTTACAC	24	<nnxnp>	nn	104	np	131		
>ref-194600	CCTCACCTGACCTCGGCTCCGCGTACC	27	<nnxnp>	nn	255	np	0		
>ref-194602	CCGCCCAAGACCATCTCTCCTACGCTT	24	<hkxhk>	hh	0	hk	267	kk	5
>ref-194602	CCGCCCAAGACCATCTCTCCTACGCTT	25	<hkxhk>	hh	0	hk	273	kk	0
>ref-194602	CCGCCCAAGACCATCTCTCCTACGCTT	27	<hkxhk>	hh	0	hk	225	kk	0
>ref-194612	CCCCTGCTTACCCCTTCTCCCCGTCCC	16	<nnxnp>	nn	8	np	235		
>ref-194612	CCCCTGCTTACCCCTTCTCCCCGTCCC	24	<nnxnp>	nn	8	np	235		
>ref-194617	TGCACGCACACAGATACTCCCACATTA	27	<nnxnp>	nn	127	np	141		

>ref-194642	TCTGGGTACACTTCCGCTCCCTCTTCC	3	<nnxnp>	nn	127	np	140		
>ref-194673	TCAGACTTCACAATAACTCCCAGGTAG	3	<lmxll>	ll	16	lm	233		
>ref-194673	TCAGACTTCACAATAACTCCCAGGTAG	22	<lmxll>	ll	16	lm	233		
>ref-194707	CCTCTACTTACCCCTCCTCCTCCTCCC	8	<lmxll>	ll	132	lm	128		
>ref-194707	CCTCTACTTACCCCTCCTCCTCCTCCC	12	<hkxhk>	hh	0	hk	273	kk	0
>ref-194707	CCTCTACTTACCCCTCCTCCTCCTCCC	27	<hkxhk>	hh	0	hk	272	kk	1
>ref-194712	CTGATCATTACCCCTTCTCCCCCTCTC	15	<nnxnp>	nn	139	np	108		
>ref-194733	CCATCTTTTACTCCCTCTCCATCCCTC	4	<lmxll>	ll	115	lm	137		
>ref-194741	CCTATTCCTACGCTAACTCCTTGGTCT	3	<lmxll>	ll	143	lm	124		
>ref-194742	GACTTTCTGACAACTGCTCCACTTTAC	22	<hkxhk>	hh	30	hk	217	kk	0
>ref-194742	GACTTTCTGACAACTGCTCCACTTTAC	24	<lmxll>	ll	28	lm	218		
>ref-194767	CCCCCATAAACCCCATCTCCCCCTCCT	23	<nnxnp>	nn	99	np	123		
>ref-194771	ATTCTCTCCACCCCTTCTCCCCTCTCC	1	<lmxll>	ll	7	lm	257		
>ref-194771	ATTCTCTCCACCCCTTCTCCCCTCTCC	21	<lmxll>	ll	14	lm	249		
>ref-194815	ATACCCGAAACGGATACTCCTCGACAT	27	<nnxnp>	nn	256	np	1		
>ref-194860	TCTTCCTTTACAGGATCTCCAACTGTG	25	<lmxll>	ll	99	lm	132		
>ref-194871	TTGTAGTGTACTTTCACTCCATAGTGT	27	<nnxnp>	nn	235	np	0		
>ref-194886	CACTTTCCCACCCACCCTCCTCCCTTC	1	<nnxnp>	nn	137	np	109		
>ref-194887	TCTCTCCCGACGCCCACTCCTGTTCCC	4	<hkxhk>	hh	225	hk	32	kk	0
>ref-194902	ACCGCTATTACTACAACTCCTACTGAT	4	<lmxll>	ll	116	lm	123		
>ref-194904	ACTACCACAACAACCGCTCCTACAAAC	21	<nnxnp>	nn	116	np	133		
>ref-194932	CGCGCGAGGACCTACTCTCCATGATGA	5	<lmxll>	ll	105	lm	150		
>ref-194932	CGCGCGAGGACCTACTCTCCATGATGA	8	<lmxll>	ll	103	lm	153		
>ref-194939	CCTGCTCCTACTCCTTCTCCTATATCT	22	<nnxnp>	nn	238	np	1		
>ref-194947	AGCTCATATACAGTCTCTCCTACTCTT	22	<lmxll>	ll	128	lm	137		
>ref-194947	AGCTCATATACAGTCTCTCCTACTCTT	26	<hkxhk>	hh	69	hk	141	kk	57
>ref-194972	CCATCCCCCACCTTTTCTCCACCTCCT	2	<nnxnp>	nn	123	np	108		
>ref-194972	CCATCCCCCACCTTTTCTCCACCTCCT	7	<nnxnp>	nn	120	np	111		
>ref-194982	ACTAATACAACCTTCCCTCCAATAGGT	22	<lmxll>	ll	130	lm	126		
>ref-195011	ACTGAAGTGACTAAGACTCCATTAATC	1	<lmxll>	ll	0	lm	273		
>ref-195011	ACTGAAGTGACTAAGACTCCATTAATC	24	<lmxll>	ll	0	lm	273		
>ref-195035	TATTCAAATACATTCTCTCCTTCTCCT	15	<hkxhk>	hh	47	hk	136	kk	56
>ref-195035	TATTCAAATACATTCTCTCCTTCTCCT	27	<hkxhk>	hh	48	hk	135	kk	54
>ref-195044	CGGTCCTAAACTGAATCTCCTCTTCGG	23	<lmxll>	ll	108	lm	118		
>ref-195106	TCACTCAAGACTCTCTCTCCTCCTGTC	4	<nnxnp>	nn	143	np	129		
>ref-195121	CACAGATAAACGCTAACTCCCACTTAC	25	<hkxhk>	hh	0	hk	257	kk	7
>ref-195121	CACAGATAAACGCTAACTCCCACTTAC	26	<lmxll>	ll	151	lm	97		
>ref-195146	TCGTCTATAACCTCCTCTCCTCTCCCT	24	<lmxll>	ll	137	lm	129		

>ref-195155	CACTCGACCACAGCCTCTCCCTTGGTC	1	<lmxll>	ll	124	lm	142		
>ref-195160	GATCTGATAACCTATTCTCCCTGTGTT	1	<nnxnp>	nn	116	np	130		
>ref-195160	GATCTGATAACCTATTCTCCCTGTGTT	3	<nnxnp>	nn	116	np	130		
>ref-195160	GATCTGATAACCTATTCTCCCTGTGTT	9	<lmxll>	ll	121	lm	126		
>ref-195167	CTTTCTCCTACACTTCCTCCTACTTCC	1	<lmxll>	ll	120	lm	120		
>ref-195190	TTTTTACCGACTCCGACTCCAGCAACA	1	<lmxll>	ll	201	lm	54		
>ref-195190	TTTTTACCGACTCCGACTCCAGCAACA	7	<nnxnp>	nn	230	np	24		
>ref-195190	TTTTTACCGACTCCGACTCCAGCAACA	9	<nnxnp>	nn	230	np	24		
>ref-195190	TTTTTACCGACTCCGACTCCAGCAACA	14	<hkxhk>	hh	172	hk	86	kk	0
>ref-195199	CAGGAAAACACAAAGGCTCCAGATACC	16	<lmxll>	ll	92	lm	146		
>ref-195205	ACTCCATCTACTCCCACTCCACCCCCT	16	<nnxnp>	nn	145	np	121		
>ref-195224	TATACCACCACTCTTCCTCCCACTCCT	8	<nnxnp>	nn	260	np	0		
>ref-195224	TATACCACCACTCTTCCTCCCACTCCT	12	<nnxnp>	nn	261	np	0		
>ref-195235	GGCTTAACAACCCCCACTCCCCTGTCC	26	<hkxhk>	hh	54	hk	149	kk	57
>ref-195248	TTTACTCACACTCTTTCTCCCTCTCCT	25	<nnxnp>	nn	136	np	111		
>ref-195264	ACCCTTCCCACTCCCTCTCCTCTCTTC	1	<nnxnp>	nn	61	np	173		
>ref-195264	ACCCTTCCCACTCCCTCTCCTCTCTTC	4	<nnxnp>	nn	259	np	0		
>ref-195264	ACCCTTCCCACTCCCTCTCCTCTCTTC	21	<nnxnp>	nn	68	np	167		
>ref-195266	CCCTTCCCCACCCCATCTCCCCTACAT	5	<hkxhk>	hh	0	hk	162	kk	89
>ref-195266	CCCTTCCCCACCCCATCTCCCCTACAT	8	<hkxhk>	hh	92	hk	160	kk	0
>ref-195275	TCCCCAAGTACACTTCCTCCAACAGCC	1	<lmxll>	ll	115	lm	136		
>ref-195275	TCCCCAAGTACACTTCCTCCAACAGCC	23	<nnxnp>	nn	126	np	115		
>ref-195277	AATACACACACAACACCTCCCTCCCCT	1	<lmxll>	ll	29	lm	218		
>ref-195277	AATACACACACAACACCTCCCTCCCCT	14	<lmxll>	ll	29	lm	218		
>ref-195283	AGAGAGTCGACAGCTGCTCCTGCTACT	6	<lmxll>	ll	117	lm	147		
>ref-195299	CAAGCCAACACTCCTCCTCCACTCTCC	24	<lmxll>	ll	164	lm	106		
>ref-195308	CAGAGAGGCACAGCTTCTCCATCACAG	6	<hkxhk>	hh	65	hk	142	kk	61
>ref-195311	CGAGCCGCCACGAGCTCTCCCACTCTC	27	<nnxnp>	nn	140	np	127		
>ref-195312	CGTGTTTTCACCCCCTCTCCTTCCCTC	12	<hkxhk>	hh	39	hk	134	kk	64
>ref-195316	TTCCTCCACACTTTTCCTCCTTCCCCT	8	<lmxll>	ll	254	lm	0		
>ref-195316	TTCCTCCACACTTTTCCTCCTTCCCCT	15	<lmxll>	ll	260	lm	0		
>ref-195328	CCCATTTTCACTCTTCCTCCTTCTGCT	24	<lmxll>	ll	251	lm	0		
>ref-195347	CTGCCACAGACACTCGCTCCACCTGCT	1	<nnxnp>	nn	273	np	0		
>ref-195370	CTCCACCGTACCATGGCTCCACACCCG	23	<lmxll>	ll	137	lm	127		
>ref-195393	ACTACTCATACTACTACTCCCACATGA	24	<hkxhk>	hh	11	hk	251	kk	0
>ref-195418	TTTATCCCCACCTCATCTCCCTTGTAA	14	<lmxll>	ll	132	lm	116		
>ref-195445	TCACCCGCCACGAGCCCTCCCGCCAAC	26	<lmxll>	ll	115	lm	150		
>ref-195464	GTGGCAGTAACTCACCCTCCATGACAT	21	<nnxnp>	nn	117	np	154		

>ref-195470	TGGCACTGGACGACACCTCCGGGTCTG	26	<lmxll>	ll	127	lm	143		
>ref-195478	ACGACAATCACAAACCCTCCTTCTTAC	5	<lmxll>	ll	144	lm	125		
>ref-195484	CTCCTGACAACAAGTACTCCATTGCCC	5	<lmxll>	ll	145	lm	113		
>ref-195490	CACAAACAAACGCCGCCTCCTCAGCGA	4	<nnxnp>	nn	137	np	117		
>ref-195506	CACTGACACACTAACACTCCCTCCTTC	4	<lmxll>	ll	246	lm	1		
>ref-195506	CACTGACACACTAACACTCCCTCCTTC	5	<lmxll>	ll	246	lm	0		
>ref-195514	TACATACACACAACTCCTCCCAAGGCT	13	<hkxhk>	hh	0	hk	253	kk	6
>ref-195514	TACATACACACAACTCCTCCCAAGGCT	23	<hkxhk>	hh	1	hk	258	kk	4
>ref-195515	CTCACTTGCACTCACACTCCCTCACAT	9	<hkxhk>	hh	101	hk	127	kk	44
>ref-195532	TCTCTCTTAACCCTCCCTCCGTCTATC	9	<nnxnp>	nn	122	np	140		
>ref-195532	TCTCTCTTAACCCTCCCTCCGTCTATC	21	<nnxnp>	nn	118	np	142		
>ref-195563	CCTCTCCCTACCATAACTCCCTCCCCC	16	<nnxnp>	nn	131	np	130		
>ref-195563	CCTCTCCCTACCATAACTCCCTCCCCC	24	<nnxnp>	nn	261	np	1		
>ref-195601	CTGCCTCTCACACCCTCTCCATGGACA	21	<nnxnp>	nn	134	np	132		
>ref-195613	CCCTCCACGACAACCCCTCCACGACAA	27	<nnxnp>	nn	125	np	124		
>ref-195627	AGACCCCTTACCCCTCCTCCTCCCCCC	2	<lmxll>	ll	158	lm	81		
>ref-195648	CTCGCCCTCACGACATCTCCTTCCTTC	5	<lmxll>	ll	146	lm	120		
>ref-195648	CTCGCCCTCACGACATCTCCTTCCTTC	13	<hkxhk>	hh	42	hk	140	kk	74
>ref-195660	TACTTAATGACTTTTTCTCCTTTCCCA	7	<lmxll>	ll	102	lm	125		
>ref-195685	CCTTCTAGTACTCTCTCTCCTTCTCCG	1	<lmxll>	ll	116	lm	144		
>ref-195697	CGAGGGCAGACTGATCCTCCCTTCTTA	27	<nnxnp>	nn	246	np	3		
>ref-195700	CTCGGGACGACTCCGCCTCCAAATAGG	24	<hkxhk>	hh	59	hk	142	kk	51
>ref-195705	CTTCCATCCACTGTCTCTCCTGTCTTC	27	<nnxnp>	nn	147	np	113		
>ref-195732	TCCTCCCCCACCCATTCTCCTCCCCTC	13	<hkxhk>	hh	76	hk	187	kk	0
>ref-195732	TCCTCCCCCACCCATTCTCCTCCCCTC	23	<lmxll>	ll	8	lm	257		
>ref-195739	CGACGACGGACTGCAGCTCCTCAGTGT	22	<hkxhk>	hh	53	hk	152	kk	59
>ref-195739	CGACGACGGACTGCAGCTCCTCAGTGT	23	<hkxhk>	hh	53	hk	154	kk	58
>ref-195753	CACTTTCATACTCATACTCCTCCCTCT	3	<nnxnp>	nn	131	np	128		
>ref-195753	CACTTTCATACTCATACTCCTCCCTCT	6	<nnxnp>	nn	131	np	128		
>ref-195770	CCAATTTTCACATTACCTCCACATCCC	3	<lmxll>	ll	172	lm	71		
>ref-195770	CCAATTTTCACATTACCTCCACATCCC	22	<lmxll>	ll	61	lm	165		
>ref-195770	CCAATTTTCACATTACCTCCACATCCC	25	<lmxll>	ll	61	lm	165		
>ref-195770	CCAATTTTCACATTACCTCCACATCCC	27	<hkxhk>	hh	8	hk	246	kk	1
>ref-195782	GGTCCTAATACCCCCTCTCCTCCCCTC	5	<hkxhk>	hh	1	hk	168	kk	55
>ref-195782	GGTCCTAATACCCCCTCTCCTCCCCTC	7	<hkxhk>	hh	1	hk	168	kk	56
>ref-195788	TATTCTCTTACTACTCCTCCTCTTTCC	5	<nnxnp>	nn	86	np	146		
>ref-195788	TATTCTCTTACTACTCCTCCTCTTTCC	12	<hkxhk>	hh	53	hk	190	kk	1
>ref-195788	TATTCTCTTACTACTCCTCCTCTTTCC	13	<hkxhk>	hh	1	hk	188	kk	54

>ref-195791	CTTCCTGCCACTACTCCTCCTCCCTCC	1	<lmxll>	ll	223	lm	6		
>ref-195791	CTTCCTGCCACTACTCCTCCTCCCTCC	12	<hkxhk>	hh	0	hk	106	kk	114
>ref-195808	AGAAAATATACTAACTCTCCCTCGCCT	12	<lmxll>	ll	125	lm	136		
>ref-195833	CACCTTCCAACGCCCTCTCCCCCTCCT	14	<hkxhk>	hh	1	hk	264	kk	0
>ref-195833	CACCTTCCAACGCCCTCTCCCCCTCCT	22	<hkxhk>	hh	1	hk	264	kk	0
>ref-195883	TCATCCGACACTATAGCTCCCAGATAT	5	<hkxhk>	hh	115	hk	126	kk	0
>ref-195883	TCATCCGACACTATAGCTCCCAGATAT	16	<hkxhk>	hh	0	hk	12	kk	255
>ref-195883	TCATCCGACACTATAGCTCCCAGATAT	26	<lmxll>	ll	127	lm	120		
>ref-195884	TGCCTACTTACCTTCCCTCCTTGCCTA	12	<nnxnp>	nn	27	np	226		
>ref-195884	TGCCTACTTACCTTCCCTCCTTGCCTA	27	<nnxnp>	nn	29	np	224		
>ref-195889	TCTCCACTTACGCCTTCTCCACCTCCT	23	<nnxnp>	nn	145	np	127		
>ref-195914	AAGAAGGCGACCTCAGCTCCTACGGGC	12	<nnxnp>	nn	141	np	108		
>ref-195917	TCTCGCTCCACCTCCCCTCCCCGCTCT	21	<nnxnp>	nn	128	np	131		
>ref-195917	TCTCGCTCCACCTCCCCTCCCCGCTCT	27	<nnxnp>	nn	127	np	131		
>ref-195933	TCACCGCTGACTTCATCTCCGCCAGTG	6	<nnxnp>	nn	121	np	143		
>ref-195941	AAGGGAGTGACAGCAACTCCCTAGTAA	26	<hkxhk>	hh	59	hk	143	kk	56
>ref-195942	TCCTTCCCCACTATCCCTCCTACCCTC	12	<nnxnp>	nn	2	np	262		
>ref-195942	TCCTTCCCCACTATCCCTCCTACCCTC	25	<nnxnp>	nn	2	np	262		
>ref-195943	TAAACCTTAACAGTTTCTCCAGCACTA	9	<hkxhk>	hh	24	hk	233	kk	0
>ref-195954	ATCACCATCACCTGATCTCCCTTCTGG	3	<hkxhk>	hh	41	hk	212	kk	0
>ref-195954	ATCACCATCACCTGATCTCCCTTCTGG	7	<hkxhk>	hh	40	hk	212	kk	0
>ref-195954	ATCACCATCACCTGATCTCCCTTCTGG	12	<hkxhk>	hh	39	hk	212	kk	0
>ref-195954	ATCACCATCACCTGATCTCCCTTCTGG	14	<hkxhk>	hh	0	hk	212	kk	40
>ref-195954	ATCACCATCACCTGATCTCCCTTCTGG	26	<hkxhk>	hh	9	hk	259	kk	0
>ref-195956	AGACGCGAAACACAATCTCCAGGACAA	27	<nnxnp>	nn	266	np	0		
>ref-195965	TACTGGACAACTTACGCTCCCTCTATA	8	<nnxnp>	nn	135	np	130		
>ref-195979	TCCTTCCCTACCTACCCTCCTCCCGCC	16	<lmxll>	ll	121	lm	137		
>ref-195989	AGAAAGGAGACTCTCTCTCCCTCCCTC	6	<lmxll>	ll	224	lm	33		
>ref-195989	AGAAAGGAGACTCTCTCTCCCTCCCTC	21	<nnxnp>	nn	155	np	88		
>ref-195989	AGAAAGGAGACTCTCTCTCCCTCCCTC	26	<lmxll>	ll	225	lm	33		
>ref-196009	ATACTATGCACATATTCTCCTTGGACA	8	<nnxnp>	nn	118	np	144		
>ref-196013	CTACTCCCCACTCTCTCTCCTCGTCTA	15	<lmxll>	ll	99	lm	130		
>ref-196019	GCTTCCCATACAACGGCTCCAAATGGT	9	<nnxnp>	nn	256	np	0		
>ref-196040	TCCCATCCTACCTCCCCTCCCCCTTCC	4	<lmxll>	ll	42	lm	218		
>ref-196098	TTCATTATCACTTTTCCTCCTCCATTT	23	<nnxnp>	nn	154	np	91		
>ref-196122	CGTAAGGCCACATTCTCTCCAGGCAAC	9	<lmxll>	ll	138	lm	127		
>ref-196136	CTCTCGATCACGTTTTCTCCCATCGCG	24	<nnxnp>	nn	104	np	160		
>ref-196141	ACCCTCTAAACCACTCCTCCTACCCCC	13	<lmxll>	ll	53	lm	173		

>ref-196141	ACCCTCTAAACCACTCCTCCTACCCCC	15	<lmxll>	ll	51	lm	174		
>ref-196143	CATATCCCTACCCATCCTCCTTCTCCT	13	<hkxhk>	hh	57	hk	138	kk	54
>ref-196157	TCCTTCTCTACGAGCCCTCCCTCCATC	25	<lmxll>	ll	0	lm	259		
>ref-196173	CAACTGCAAACGATTCCTCCCATGCCC	15	<hkxhk>	hh	56	hk	157	kk	51
>ref-196174	CTACCCTCCACCTCCCCTCCTCCGCTT	1	<lmxll>	ll	260	lm	0		
>ref-196174	CTACCCTCCACCTCCCCTCCTCCGCTT	4	<lmxll>	ll	263	lm	0		
>ref-196189	TCTCCTTTCACTAATCCTCCTCTACCT	24	<nnxnp>	nn	117	np	139		
>ref-196209	CTCCCTTACACCCCCACTCCCCTCCCC	5	<hkxhk>	hh	140	hk	79	kk	0
>ref-196210	ACCCCCCCAACCCCCTCTCCATCGGTA	2	<nnxnp>	nn	102	np	132		
>ref-196240	AGTCGCTCAACTCCGACTCCGACTCGT	1	<hkxhk>	hh	0	hk	269	kk	0
>ref-196240	AGTCGCTCAACTCCGACTCCGACTCGT	4	<hkxhk>	hh	0	hk	269	kk	0
>ref-196286	CCCTGAGTAACTGAGCCTCCTTCCGCA	4	<nnxnp>	nn	140	np	120		
>ref-196286	CCCTGAGTAACTGAGCCTCCTTCCGCA	5	<nnxnp>	nn	140	np	120		
>ref-196316	GTGGCACGCACACCACCTCCGCCGTCT	12	<nnxnp>	nn	264	np	0		
>ref-196326	CATCCTTTCACTTACGCTCCGTGTCAC	5	<nnxnp>	nn	126	np	137		
>ref-196338	CTGCTCCTCACTCTCCCTCCCACTTCC	4	<lmxll>	ll	259	lm	0		
>ref-196353	AATCAGTCCACTGCCTCTCCCAATCTT	1	<lmxll>	ll	3	lm	267		
>ref-196353	AATCAGTCCACTGCCTCTCCCAATCTT	8	<nnxnp>	nn	73	np	183		
>ref-196353	AATCAGTCCACTGCCTCTCCCAATCTT	23	<nnxnp>	nn	73	np	183		
>ref-196359	CAACATTTGACTCATTCTCCTCAACAT	5	<hkxhk>	hh	4	hk	265	kk	0
>ref-196359	CAACATTTGACTCATTCTCCTCAACAT	25	<nnxnp>	nn	161	np	106		
>ref-196363	GAAATATCCACAGACTCTCCTGTTGCC	22	<nnxnp>	nn	120	np	127		
>ref-196366	CATGTACATACGATCTCTCCGCAGAGC	1	<lmxll>	ll	120	lm	114		
>ref-196390	TCTCTCTTTACCTTCCCTCCACTTTCC	2	<nnxnp>	nn	266	np	1		
>ref-196390	TCTCTCTTTACCTTCCCTCCACTTTCC	8	<nnxnp>	nn	266	np	1		
>ref-196390	TCTCTCTTTACCTTCCCTCCACTTTCC	9	<hkxhk>	hh	3	hk	258	kk	4
>ref-196390	TCTCTCTTTACCTTCCCTCCACTTTCC	21	<lmxll>	ll	19	lm	235		
>ref-196390	TCTCTCTTTACCTTCCCTCCACTTTCC	23	<hkxhk>	hh	3	hk	258	kk	4
>ref-196394	TTTTCTGTGACTTCTTCTCCTCCTCCT	22	<lmxll>	ll	127	lm	135		
>ref-196403	TCTGGGCCCACTTTCTCTCCGACTTCC	5	<hkxhk>	hh	53	hk	138	kk	59
>ref-196406	ATCCCCCATACGGTGGCTCCATGGTAG	24	<hkxhk>	hh	68	hk	138	kk	58
>ref-196414	CTCCCCTCCACAGCCCCTCCTTCCCCC	23	<lmxll>	ll	128	lm	132		
>ref-196439	GCCTTGTACACTGGAGCTCCCTTTTCT	15	<lmxll>	ll	129	lm	128		
>ref-196462	AAGTCGCTCACTACAACTCCTCCCCCT	7	<hkxhk>	hh	40	hk	227	kk	0
>ref-196462	AAGTCGCTCACTACAACTCCTCCCCCT	9	<hkxhk>	hh	38	hk	227	kk	0
>ref-196462	AAGTCGCTCACTACAACTCCTCCCCCT	21	<hkxhk>	hh	0	hk	273	kk	0
>ref-196462	AAGTCGCTCACTACAACTCCTCCCCCT	22	<hkxhk>	hh	0	hk	273	kk	0
>ref-196464	CCCTTCTTTACCCTCCCTCCTTGCCTC	21	<hkxhk>	hh	1	hk	261	kk	0

>ref-196464	CCCTTCTTTACCCTCCCTCCTTGCCTC	23	<hkxhk>	hh	2	hk	261	kk	0
>ref-196468	TAAATCTTTACCTGAACTCCGTGACCT	4	<lmxll>	ll	106	lm	113		
>ref-196499	TAAAAAGACACCCTACCTCCGGAGGGC	1	<lmxll>	ll	13	lm	253		
>ref-196499	TAAAAAGACACCCTACCTCCGGAGGGC	9	<nnxnp>	nn	189	np	62		
>ref-196499	TAAAAAGACACCCTACCTCCGGAGGGC	21	<hkxhk>	hh	37	hk	218	kk	0
>ref-196516	GTGACTCGCACCTTTTCTCCTTCTCCT	15	<lmxll>	ll	158	lm	103		
>ref-196561	CTTCCCTCTACCCTCCCTCCCCTCCTA	6	<lmxll>	ll	204	lm	45		
>ref-196561	CTTCCCTCTACCCTCCCTCCCCTCCTA	25	<lmxll>	ll	0	lm	225		
>ref-196578	AAAACGTACACTAACCCTCCTCCACCG	4	<hkxhk>	hh	3	hk	139	kk	108
>ref-196578	AAAACGTACACTAACCCTCCTCCACCG	6	<hkxhk>	hh	107	hk	139	kk	3
>ref-196585	ACCCCCTCAACCATCCCTCCCCCTCAT	2	<nnxnp>	nn	256	np	0		
>ref-196585	ACCCCCTCAACCATCCCTCCCCCTCAT	16	<nnxnp>	nn	115	np	126		
>ref-196589	CTCAGCATTACGGTGACTCCTCTCCTT	21	<lmxll>	ll	11	lm	249		
>ref-196589	CTCAGCATTACGGTGACTCCTCTCCTT	26	<lmxll>	ll	11	lm	249		
>ref-196593	ATTTTCGTGACCGTTTCTCCTTGTTCT	27	<nnxnp>	nn	118	np	108		
>ref-196600	CTTTCTCTAACTTTCCCTCCCTTCCCA	14	<lmxll>	ll	122	lm	126		
>ref-196612	TTCTCCCAGACCGTCTCTCCGTCCATC	12	<nnxnp>	nn	263	np	0		
>ref-196612	TTCTCCCAGACCGTCTCTCCGTCCATC	26	<nnxnp>	nn	263	np	0		
>ref-196612	TTCTCCCAGACCGTCTCTCCGTCCATC	27	<nnxnp>	nn	261	np	0		
>ref-196614	TTCCCTCTCACATTTCCTCCTTTTTCC	3	<hkxhk>	hh	4	hk	255	kk	1
>ref-196614	TTCCCTCTCACATTTCCTCCTTTTTCC	21	<hkxhk>	hh	1	hk	255	kk	4
>ref-196621	AGCCCAAACACCCAACCTCCACATACT	16	<nnxnp>	nn	103	np	145		
>ref-196622	ATTTAATACACCCAAACTCCTTTTATA	12	<hkxhk>	hh	50	hk	132	kk	63
>ref-196623	CCACCATTTACTCTTTCTCCACCATTT	3	<lmxll>	ll	68	lm	179		
>ref-196655	TGACCTCTGACCCTCCCTCCACCTCCT	2	<hkxhk>	hh	0	hk	265	kk	0
>ref-196655	TGACCTCTGACCCTCCCTCCACCTCCT	3	<hkxhk>	hh	0	hk	264	kk	1
>ref-196672	CCTATTCGAACTACATCTCCTTTGTCT	2	<nnxnp>	nn	123	np	113		
>ref-196689	CCCATTCTCACGCCCTCTCCCACCTCT	8	<nnxnp>	nn	262	np	0		
>ref-196689	CCCATTCTCACGCCCTCTCCCACCTCT	22	<nnxnp>	nn	256	np	0		
>ref-196703	CCCAACCCTACCCCCTCTCCCCCCATC	14	<lmxll>	ll	198	lm	65		
>ref-196703	CCCAACCCTACCCCCTCTCCCCCCATC	23	<hkxhk>	hh	20	hk	244	kk	0
>ref-196703	CCCAACCCTACCCCCTCTCCCCCCATC	24	<lmxll>	ll	255	lm	2		
>ref-196731	GAGTAGGAAACTCGATCTCCCTTGTTG	3	<nnxnp>	nn	108	np	144		
>ref-196732	GAGATGGTGACCATCACTCCAGTCTGA	23	<hkxhk>	hh	0	hk	271	kk	2
>ref-196732	GAGATGGTGACCATCACTCCAGTCTGA	25	<hkxhk>	hh	0	hk	271	kk	2
>ref-196740	ATGTGCACCACGTTCACTCCGCGTTCC	6	<nnxnp>	nn	120	np	149		
>ref-196748	CACACGCACACATGTACTCCAACACAT	1	<nnxnp>	nn	127	np	141		
>ref-196753	CCTGTCACGACTTTAGCTCCTCCCACT	4	<nnxnp>	nn	126	np	126		

>ref-196776	ATCCTGTGAACTGACACTCCCGCTGTT	8	<hkxhk>	hh	52	hk	150	kk	54
>ref-196776	ATCCTGTGAACTGACACTCCCGCTGTT	21	<hkxhk>	hh	54	hk	151	kk	51
>ref-196781	TGGCAGACAACCGAAGCTCCGACTAGT	16	<lmxll>	ll	116	lm	149		
>ref-196805	CCCTATCATACATCCTCTCCCTCCACG	13	<lmxll>	ll	115	lm	148		
>ref-196836	ACCACCCCCACTTTCCCTCCCTTCCAC	8	<nnxnp>	nn	128	np	125		
>ref-196841	GAACAGAAAACGGGCGCTCCTCCATTA	14	<nnxnp>	nn	90	np	134		
>ref-196876	CTGCAAGCAACACTACCTCCCTGGTTC	21	<nnxnp>	nn	57	np	197		
>ref-196876	CTGCAAGCAACACTACCTCCCTGGTTC	27	<nnxnp>	nn	58	np	196		
>ref-196915	AACCTCCCCACCAGCTCTCCTTCACAC	24	<lmxll>	ll	114	lm	139		
>ref-196927	GGGGGCACCACTCTCTCTCCTAAAATA	13	<lmxll>	ll	260	lm	9		
>ref-196927	GGGGGCACCACTCTCTCTCCTAAAATA	22	<lmxll>	ll	260	lm	9		
>ref-196947	GAGAGTATGACATTCGCTCCCATTTGC	16	<nnxnp>	nn	251	np	0		
>ref-196957	TCATCCTTCACTCGCCCTCCTCTCCTC	15	<lmxll>	ll	136	lm	122		
>ref-196959	CAAACAGACACACACGCTCCATAGTGT	3	<lmxll>	ll	124	lm	131		
>ref-196959	CAAACAGACACACACGCTCCATAGTGT	22	<nnxnp>	nn	107	np	149		
>ref-196963	TCTTTCCTCACGCTCCCTCCCATCCTG	27	<nnxnp>	nn	138	np	124		
>ref-196970	TCCAACGAGACTCTCTCTCCTTCCCTT	26	<hkxhk>	hh	49	hk	131	kk	81
>ref-196972	ACTCCTCCCACTGTCCCTCCCCTCTCT	13	<lmxll>	ll	163	lm	91		
>ref-196972	ACTCCTCCCACTGTCCCTCCCCTCTCT	27	<lmxll>	ll	165	lm	90		
>ref-196973	TGCTGACTCACACTGACTCCTTCTCCC	23	<nnxnp>	nn	132	np	135		
>ref-196974	GGTTTTACGACAATATCTCCTTTTATG	24	<lmxll>	ll	108	lm	134		
>ref-196993	CCCTTCTTTACCCAAGCTCCCTTGCCT	7	<hkxhk>	hh	0	hk	260	kk	4
>ref-196993	CCCTTCTTTACCCAAGCTCCCTTGCCT	16	<hkxhk>	hh	0	hk	260	kk	4
>ref-196993	CCCTTCTTTACCCAAGCTCCCTTGCCT	24	<hkxhk>	hh	0	hk	270	kk	0
>ref-196999	TTGCCCTCCACTACTCCTCCTCTCTTT	25	<lmxll>	ll	115	lm	145		
>ref-196999	TTGCCCTCCACTACTCCTCCTCTCTTT	26	<lmxll>	ll	111	lm	147		
>ref-197025	TGTTGCACCACCTGGGCTCCCTCTCTT	22	<nnxnp>	nn	124	np	142		
>ref-197025	TGTTGCACCACCTGGGCTCCCTCTCTT	27	<nnxnp>	nn	130	np	135		
>ref-197033	TATTCCTTTACTTCCTCTCCACTTATT	13	<hkxhk>	hh	51	hk	142	kk	40
>ref-197044	CATATTACAACACCACCTCCAAAGCAA	14	<lmxll>	ll	105	lm	142		
>ref-197082	TTCTTCCCCACTTTTCCTCCCTCCCTC	4	<nnxnp>	nn	132	np	114		
>ref-197082	TTCTTCCCCACTTTTCCTCCCTCCCTC	5	<nnxnp>	nn	250	np	5		
>ref-197082	TTCTTCCCCACTTTTCCTCCCTCCCTC	12	<nnxnp>	nn	72	np	180		
>ref-197104	CGGGCTTATACAGCAGCTCCACCTCTC	1	<nnxnp>	nn	141	np	84		
>ref-197104	CGGGCTTATACAGCAGCTCCACCTCTC	5	<nnxnp>	nn	142	np	84		
>ref-197173	TCCTCCTCGACCCTCCCTCCTTCTCGT	9	<nnxnp>	nn	224	np	6		
>ref-197173	TCCTCCTCGACCCTCCCTCCTTCTCGT	26	<nnxnp>	nn	215	np	7		
>ref-197189	CGACATTAGACGTTTCCTCCTCAGCCA	1	<hkxhk>	hh	0	hk	213	kk	48

>ref-197189	CGACATTAGACGTTTCCTCCTCAGCCA	2	<hkxhk>	hh	0	hk	217	kk	34
>ref-197189	CGACATTAGACGTTTCCTCCTCAGCCA	12	<hkxhk>	hh	36	hk	219	kk	0
>ref-197218	CTCCCATTCACACACCCTCCTTCCCCT	16	<hkxhk>	hh	0	hk	201	kk	56
>ref-197218	CTCCCATTCACACACCCTCCTTCCCCT	26	<hkxhk>	hh	58	hk	201	kk	0
>ref-197220	TCCTTTACTACTCTTTCTCCCTGTTCT	12	<lmxll>	ll	60	lm	190		
>ref-197221	CTATTTCTCACTTTCTCTCCGCTGCGC	14	<hkxhk>	hh	59	hk	131	kk	42
>ref-197221	CTATTTCTCACTTTCTCTCCGCTGCGC	16	<nnxnp>	nn	116	np	119		
>ref-197264	AATCTCACTACTCCCACTCCCTCTTCA	8	<lmxll>	ll	8	lm	262		
>ref-197264	AATCTCACTACTCCCACTCCCTCTTCA	25	<lmxll>	ll	8	lm	262		
>ref-197276	TAGTATATCACTGATTCTCCCTTATTA	6	<hkxhk>	hh	0	hk	246	kk	7
>ref-197276	TAGTATATCACTGATTCTCCCTTATTA	26	<hkxhk>	hh	0	hk	246	kk	7
>ref-197288	AAACCAGACACGCCACCTCCAGCCGTC	5	<lmxll>	ll	127	lm	142		
>ref-197288	AAACCAGACACGCCACCTCCAGCCGTC	15	<hkxhk>	hh	64	hk	143	kk	59
>ref-197306	CTCACCCTCACTCTCTCTCCCAATGAA	1	<nnxnp>	nn	133	np	103		
>ref-197320	ACTATTCCGACCTCTACTCCATGCAGA	8	<lmxll>	ll	2	lm	266		
>ref-197320	ACTATTCCGACCTCTACTCCATGCAGA	22	<lmxll>	ll	2	lm	266		
>ref-197339	GGCCCTAGAACCTGGGCTCCTCAAGTC	8	<hkxhk>	hh	89	hk	168	kk	0
>ref-197339	GGCCCTAGAACCTGGGCTCCTCAAGTC	12	<hkxhk>	hh	1	hk	265	kk	0
>ref-197359	ATCTATCTGACTCTATCTCCCTCTTTC	6	<nnxnp>	nn	269	np	1		
>ref-197359	ATCTATCTGACTCTATCTCCCTCTTTC	9	<nnxnp>	nn	234	np	4		
>ref-197359	ATCTATCTGACTCTATCTCCCTCTTTC	25	<nnxnp>	nn	254	np	1		
>ref-197362	GCCCCCTCGACGCGCCCTCCCTCCAAC	15	<nnxnp>	nn	131	np	138		
>ref-197370	TTATTGTCTACTCTGTCTCCATCATCA	9	<lmxll>	ll	128	lm	114		
>ref-197383	TCTCCCCCCACCCCATCTCCTCTCACA	4	<nnxnp>	nn	259	np	0		
>ref-197383	TCTCCCCCCACCCCATCTCCTCTCACA	24	<nnxnp>	nn	261	np	0		
>ref-197384	TCTGCATCCACTTAGACTCCCTTGTGA	14	<hkxhk>	hh	53	hk	129	kk	59
>ref-197384	TCTGCATCCACTTAGACTCCCTTGTGA	15	<hkxhk>	hh	59	hk	129	kk	52
>ref-197400	AGAGCAAAGACCAGTGCTCCCTCTGCC	4	<nnxnp>	nn	135	np	131		
>ref-197406	CTACAATACACCCAAGCTCCAGTTACC	2	<hkxhk>	hh	56	hk	131	kk	68
>ref-197417	TCTACCCCAACATGGCCTCCTCCTCCT	22	<nnxnp>	nn	141	np	124		
>ref-197417	TCTACCCCAACATGGCCTCCTCCTCCT	25	<nnxnp>	nn	141	np	125		
>ref-197431	TCTCCATATACCCACCCTCCCCCTCTC	14	<nnxnp>	nn	123	np	118		
>ref-197441	AGAGATTTAACCTTTCCTCCCCTTCCT	3	<lmxll>	ll	125	lm	123		
>ref-197443	CCCTCCCCCACGTTTTCTCCTCCCCTT	25	<lmxll>	ll	132	lm	135		
>ref-197446	ATTCATCATACATCAGCTCCTCATCAT	9	<nnxnp>	nn	124	np	144		
>ref-197458	ATACACATAACTTTTCCTCCTTCCCCT	13	<nnxnp>	nn	124	np	136		
>ref-197472	CCTGCTGCCACGCTCTCTCCTTTGGTA	9	<nnxnp>	nn	122	np	148		
>ref-197472	CCTGCTGCCACGCTCTCTCCTTTGGTA	22	<lmxll>	ll	121	lm	144		

>ref-197477	CTCCAAGCCACGTCCTCTCCCATAATT	25	<nnxnp>	nn	262	np	1		
>ref-197477	CTCCAAGCCACGTCCTCTCCCATAATT	26	<nnxnp>	nn	262	np	1		
>ref-197487	TCATCTTTCACTTTCTCTCCCCCCTTT	5	<nnxnp>	nn	244	np	0		
>ref-197493	TCCTTCGTAACCTCACCTCCGTCCTGT	16	<lmxll>	ll	106	lm	152		
>ref-197517	TACACACATACAGGCTCTCCCATCCCA	22	<nnxnp>	nn	107	np	159		
>ref-197528	CTAATACTTACAGACTCTCCTGTCAGA	5	<lmxll>	ll	119	lm	112		
>ref-197529	CACTTTCCCACTCTCGCTCCCACTCCC	1	<lmxll>	ll	137	lm	128		
>ref-197546	TTAACAACCACTCGCCCTCCCGAAGAT	16	<lmxll>	ll	114	lm	145		
>ref-197563	CCCTCATTCACTCCATCTCCTGTAATT	2	<nnxnp>	nn	267	np	0		
>ref-197563	CCCTCATTCACTCCATCTCCTGTAATT	22	<hkxhk>	hh	54	hk	143	kk	62
>ref-197564	TCTCTCTCTACCCTTTCTCCTCGTCTT	23	<nnxnp>	nn	266	np	0		
>ref-197586	CTCATACTCACTCACACTCCAGCGACG	1	<lmxll>	ll	105	lm	147		
>ref-197623	ATGGTTTGGACACGAACTCCTCCACCT	23	<nnxnp>	nn	132	np	132		
>ref-197633	TAGCTTATTACTATCCCTCCGTACCCC	8	<lmxll>	ll	116	lm	130		
>ref-197654	TTGCCCAACACCATCTCTCCAACCCTG	5	<hkxhk>	hh	43	hk	116	kk	61
>ref-197679	TACTTTTACACACACACTCCCTGCGCC	21	<lmxll>	ll	135	lm	117		
>ref-197727	TACTGTACAACCAAACCTCCCTGGGTG	25	<nnxnp>	nn	114	np	131		
>ref-197730	GTTGGCCTGACTTCTTCTCCTCTACCC	14	<lmxll>	ll	124	lm	118		
>ref-197734	CTCCCTCTTACTACCACTCCACCTACG	9	<nnxnp>	nn	237	np	0		
>ref-197751	TCAGCGCGTACTGATTCTCCCAATAAA	1	<nnxnp>	nn	263	np	0		
>ref-197754	CATCTGGCAACCTCTTCTCCGTTGACA	23	<lmxll>	ll	123	lm	143		
>ref-197762	TTTCCGTCGACCTTTCCTCCACTCCAT	8	<lmxll>	ll	265	lm	0		
>ref-197773	CCTCCACCCACTCCCACTCCATGACTC	9	<nnxnp>	nn	135	np	127		
>ref-197790	CTGTATGACACTACATCTCCGAAGCCT	1	<nnxnp>	nn	117	np	148		
>ref-197804	ATGCACCTCACCCTCTCTCCACTCTCT	4	<lmxll>	ll	138	lm	132		
>ref-197808	CTTAACCCAACTCTCACTCCATTCCCT	1	<lmxll>	ll	109	lm	158		
>ref-197808	CTTAACCCAACTCTCACTCCATTCCCT	2	<lmxll>	ll	109	lm	158		
>ref-197810	GCCTTCACGACTCTGGCTCCAGCCTCG	16	<nnxnp>	nn	270	np	0		
>ref-197829	AATGCTTCCACCCTGTCTCCCTTTTAA	5	<hkxhk>	hh	49	hk	135	kk	43
>ref-197862	CGCACCCGAACGCCATCTCCCGGCGAT	8	<hkxhk>	hh	51	hk	146	kk	66
>ref-197876	CAGAGGGACACACGCACTCCTAGACAG	27	<lmxll>	ll	135	lm	135		
>ref-197880	CGAAGGTGTACTAACCCTCCATCATCA	1	<nnxnp>	nn	224	np	42		
>ref-197880	CGAAGGTGTACTAACCCTCCATCATCA	15	<hkxhk>	hh	0	hk	273	kk	0
>ref-197885	TACATATACACACGCCCTCCATCACAC	7	<lmxll>	ll	125	lm	142		
>ref-197891	ATTCTCCCAACTGAGCCTCCAAGACGA	8	<lmxll>	ll	127	lm	138		
>ref-197909	TCGAGCCTTACTTCTGCTCCAGAGGAT	24	<hkxhk>	hh	41	hk	143	kk	61
>ref-197915	TCAAGCGTCACTCACCCTCCATACGAC	15	<nnxnp>	nn	125	np	144		
>ref-197954	TCTTCAGCGACCTTGTCTCCTCACCCG	25	<lmxll>	ll	137	lm	132		

>ref-197973	ACTCACACAACTCTTTCTCCCTGTTCA	6	<lmxll>	ll	126	lm	97		
>ref-198012	TCCCTCCTCACTCCACCTCCCCACCCC	15	<lmxll>	ll	15	lm	243		
>ref-198012	TCCCTCCTCACTCCACCTCCCCACCCC	23	<hkxhk>	hh	15	hk	245	kk	0
>ref-198012	TCCCTCCTCACTCCACCTCCCCACCCC	26	<nnxnp>	nn	225	np	23		
>ref-198016	ATCTTTCTTACTCTGCCTCCTTTGCTC	15	<lmxll>	ll	113	lm	154		
>ref-198018	CTCTCCCCCACACTTTCTCCCTTACCC	6	<hkxhk>	hh	3	hk	265	kk	1
>ref-198018	CTCTCCCCCACACTTTCTCCCTTACCC	9	<lmxll>	ll	150	lm	92		
>ref-198018	CTCTCCCCCACACTTTCTCCCTTACCC	12	<lmxll>	ll	230	lm	21		
>ref-198018	CTCTCCCCCACACTTTCTCCCTTACCC	24	<lmxll>	ll	228	lm	24		
>ref-198018	CTCTCCCCCACACTTTCTCCCTTACCC	26	<lmxll>	ll	3	lm	268		
>ref-198018	CTCTCCCCCACACTTTCTCCCTTACCC	27	<hkxhk>	hh	92	hk	155	kk	0
>ref-198021	TATCGTACCACAACCACTCCCTGCATA	5	<nnxnp>	nn	120	np	134		
>ref-198021	TATCGTACCACAACCACTCCCTGCATA	15	<hkxhk>	hh	51	hk	126	kk	69
>ref-198031	CTCCTCGAGACTCCTCCTCCTCGAGAC	2	<hkxhk>	hh	0	hk	272	kk	0
>ref-198031	CTCCTCGAGACTCCTCCTCCTCGAGAC	27	<hkxhk>	hh	33	hk	234	kk	0
>ref-198059	TCGTCCACTACATCCTCTCCCTTTTTC	1	<lmxll>	ll	117	lm	137		
>ref-198079	TAAACGGAAACGGAAACTCCATAGCCG	24	<lmxll>	ll	148	lm	94		
>ref-198083	TTTTTCTCAACGTTCTCTCCCTTTCCT	9	<lmxll>	ll	115	lm	144		
>ref-198088	GGTTATGTTACCTTGTCTCCTCAATCG	25	<nnxnp>	nn	133	np	103		
>ref-198090	GTTTTCTTCACTCTTCCTCCTTCTCCT	21	<lmxll>	ll	117	lm	142		
>ref-198103	AATGGCATGACGCCACCTCCCTGAAAG	22	<nnxnp>	nn	265	np	0		
>ref-198107	ATCATCTACACGTCATCTCCCTTTCCT	1	<hkxhk>	hh	55	hk	150	kk	60
>ref-198107	ATCATCTACACGTCATCTCCCTTTCCT	8	<hkxhk>	hh	55	hk	151	kk	60
>ref-198108	CACTTCCGGACCCACTCTCCCACTCCC	5	<nnxnp>	nn	119	np	130		
>ref-198136	CCCCCCTCCACCATCGCTCCCCCTCTC	7	<nnxnp>	nn	126	np	130		
>ref-198149	TCCCATTGCACCCTGCCTCCTCTTTCC	14	<lmxll>	ll	117	lm	135		
>ref-198160	ACTGCTGCTACTCCCCCTCCACTATTA	14	<lmxll>	ll	94	lm	129		
>ref-198166	CACTCATCTACCCCCGCTCCCACACAC	6	<nnxnp>	nn	132	np	119		
>ref-198175	GTAACCACAACCTTGACTCCTCCCAAC	22	<lmxll>	ll	118	lm	133		
>ref-198180	ACACCTCTCACTCCACCTCCACCTCTA	1	<lmxll>	ll	111	lm	147		
>ref-198180	ACACCTCTCACTCCACCTCCACCTCTA	9	<hkxhk>	hh	0	hk	260	kk	4
>ref-198180	ACACCTCTCACTCCACCTCCACCTCTA	27	<lmxll>	ll	116	lm	145		
>ref-198183	TTTCTCCTCACTTTGCCTCCTCTTCCT	24	<hkxhk>	hh	1	hk	272	kk	0
>ref-198200	CCCCCTGTGACTCTGTCTCCCTTCCCC	1	<nnxnp>	nn	240	np	0		
>ref-198222	AGCTGTTAAACCCTCTCTCCTAATCTG	3	<lmxll>	ll	79	lm	165		
>ref-198222	AGCTGTTAAACCCTCTCTCCTAATCTG	13	<hkxhk>	hh	0	hk	114	kk	106
>ref-198237	TCCGGCTGGACTGAAACTCCCTCAGCT	5	<lmxll>	ll	120	lm	141		
>ref-198242	ACAAAGAGAACGACTTCTCCTTAGAGA	8	<lmxll>	ll	126	lm	136		

>ref-198266	TCCCCCTCAACTTCTTCTCCCTCTTTT	4	<lmxll>	ll	257	lm	0		
>ref-198266	TCCCCCTCAACTTCTTCTCCCTCTTTT	5	<lmxll>	ll	257	lm	0		
>ref-198271	TCCTCCCTCACCCTCACTCCAACAGTA	25	<hkxhk>	hh	39	hk	123	kk	89
>ref-198276	ACTCCTCCGACCTTTCCTCCTTCCCCT	1	<hkxhk>	hh	1	hk	205	kk	60
>ref-198276	ACTCCTCCGACCTTTCCTCCTTCCCCT	6	<hkxhk>	hh	60	hk	204	kk	1
>ref-198276	ACTCCTCCGACCTTTCCTCCTTCCCCT	22	<nnxnp>	nn	140	np	132		
>ref-198276	ACTCCTCCGACCTTTCCTCCTTCCCCT	26	<nnxnp>	nn	139	np	132		
>ref-198297	CACCTGGAAACTTTCTCTCCTTCCCTC	14	<nnxnp>	nn	113	np	134		
>ref-198304	TCTACTGTCACTGCTACTCCTCTGTTT	6	<hkxhk>	hh	3	hk	264	kk	1
>ref-198304	TCTACTGTCACTGCTACTCCTCTGTTT	27	<nnxnp>	nn	128	np	144		
>ref-198317	GATCTGTTCACTCTGTCTCCGCGGTAG	24	<lmxll>	ll	129	lm	141		
>ref-198322	AAAGCCGCTACCCCCTCTCCCCCTTCT	6	<lmxll>	ll	125	lm	122		
>ref-198333	ACACACATAACATCCCCTCCCCCCAGC	23	<nnxnp>	nn	101	np	129		
>ref-198333	ACACACATAACATCCCCTCCCCCCAGC	24	<nnxnp>	nn	101	np	129		
>ref-198342	TAGCTGTCGACTCCTCCTCCCGATCGC	23	<nnxnp>	nn	112	np	138		
>ref-198342	TAGCTGTCGACTCCTCCTCCCGATCGC	26	<nnxnp>	nn	110	np	138		
>ref-198383	TTCTGTCTCACCATCCCTCCCTCCTTC	1	<nnxnp>	nn	2	np	260		
>ref-198383	TTCTGTCTCACCATCCCTCCCTCCTTC	13	<hkxhk>	hh	57	hk	208	kk	1
>ref-198383	TTCTGTCTCACCATCCCTCCCTCCTTC	16	<lmxll>	ll	145	lm	124		
>ref-198394	TTGAAAATGACGCCGTCTCCCTTTTCG	4	<hkxhk>	hh	1	hk	270	kk	0
>ref-198394	TTGAAAATGACGCCGTCTCCCTTTTCG	7	<hkxhk>	hh	2	hk	267	kk	1
>ref-198394	TTGAAAATGACGCCGTCTCCCTTTTCG	16	<lmxll>	ll	231	lm	28		
>ref-198394	TTGAAAATGACGCCGTCTCCCTTTTCG	27	<hkxhk>	hh	0	hk	190	kk	72
>ref-198395	TAATTATACACCTGTGCTCCAAGGAGT	6	<hkxhk>	hh	1	hk	269	kk	0
>ref-198395	TAATTATACACCTGTGCTCCAAGGAGT	7	<hkxhk>	hh	0	hk	268	kk	4
>ref-198395	TAATTATACACCTGTGCTCCAAGGAGT	15	<nnxnp>	nn	216	np	46		
>ref-198395	TAATTATACACCTGTGCTCCAAGGAGT	16	<nnxnp>	nn	157	np	91		
>ref-198395	TAATTATACACCTGTGCTCCAAGGAGT	23	<hkxhk>	hh	0	hk	268	kk	4
>ref-198395	TAATTATACACCTGTGCTCCAAGGAGT	27	<nnxnp>	nn	68	np	176		
>ref-198422	ACAAACAAAACCTCGCCTCCGCCACAG	21	<hkxhk>	hh	55	hk	138	kk	58
>ref-198452	CCACACAAAACCCTCTCTCCATTCCCC	16	<lmxll>	ll	10	lm	257		
>ref-198453	TATTTACTAACCCTTCCTCCGCAACCT	6	<nnxnp>	nn	129	np	139		
>ref-198478	AGCATTTACACGACGTCTCCTGTCTGT	26	<nnxnp>	nn	270	np	0		
>ref-198478	AGCATTTACACGACGTCTCCTGTCTGT	27	<nnxnp>	nn	271	np	0		
>ref-198512	GGGACTCTCACTAGCCCTCCCTCCCTC	12	<lmxll>	ll	122	lm	142		
>ref-198522	CTGAGCTAAACAACATCTCCATCGCTA	23	<lmxll>	ll	143	lm	123		
>ref-198529	AATCTCCCGACGTAATCTCCAAAGTGT	2	<nnxnp>	nn	126	np	133		
>ref-198536	CCTCGACTCACCTACACTCCCCTCGAC	1	<lmxll>	ll	22	lm	235		

>ref-198536	CCTCGACTCACCTACACTCCCCTCGAC	9	<nnxnp>	nn	214	np	47		
>ref-198536	CCTCGACTCACCTACACTCCCCTCGAC	21	<hkxhk>	hh	50	hk	217	kk	0
>ref-198536	CCTCGACTCACCTACACTCCCCTCGAC	24	<hkxhk>	hh	50	hk	219	kk	0
>ref-198537	GTAACATTTACACACTCTCCACACTCT	13	<nnxnp>	nn	120	np	142		
>ref-198545	TCAGTGAAAACCGAAGCTCCTTCCCTG	15	<nnxnp>	nn	123	np	132		
>ref-198545	TCAGTGAAAACCGAAGCTCCTTCCCTG	16	<nnxnp>	nn	122	np	132		
>ref-198545	TCAGTGAAAACCGAAGCTCCTTCCCTG	21	<lmxll>	ll	121	lm	131		
>ref-198558	CTTGTTCAGACTGTAGCTCCGTTCAGT	26	<nnxnp>	nn	130	np	125		
>ref-198575	TATCCTCTAACTTCCTCTCCTGTTCCT	21	<lmxll>	ll	153	lm	100		
>ref-198585	ACAAGCCGCACGCCCCCTCCGCCGTTA	4	<lmxll>	ll	137	lm	133		
>ref-198588	CCCTCGGCCACTTTTCCTCCCGCCTGT	25	<lmxll>	ll	160	lm	109		
>ref-198591	ATACTAGTGACGGCCACTCCTGGAGTT	1	<nnxnp>	nn	244	np	0		
>ref-198591	ATACTAGTGACGGCCACTCCTGGAGTT	15	<nnxnp>	nn	113	np	116		
>ref-198619	CTATTCCCAACCCCTCCTCCCCCATCT	13	<nnxnp>	nn	125	np	98		
>ref-198619	CTATTCCCAACCCCTCCTCCCCCATCT	23	<nnxnp>	nn	127	np	98		
>ref-198639	CACATCCTCACACAGTCTCCTTCGACA	15	<hkxhk>	hh	0	hk	270	kk	1
>ref-198652	CCTCAGATCACCTGGCCTCCTGATAAT	5	<hkxhk>	hh	55	hk	144	kk	44
>ref-198677	AATTCTCCGACCTTCCCTCCCTCCCCC	1	<nnxnp>	nn	259	np	1		
>ref-198677	AATTCTCCGACCTTCCCTCCCTCCCCC	26	<nnxnp>	nn	260	np	1		
>ref-198693	TCGACGCGAACTCTTTCTCCTTTCCCT	12	<lmxll>	ll	230	lm	0		
>ref-198701	CAAATGCACACACGCCCTCCCTCCCCC	25	<nnxnp>	nn	138	np	131		
>ref-198727	GAATGTTATACAATATCTCCTGACAAT	12	<lmxll>	ll	96	lm	151		
>ref-198729	ACTCGTTATACCTTTCCTCCCTTCTTG	8	<lmxll>	ll	118	lm	146		
>ref-198733	CCCGCTCACACGTCCTCTCCCCTGCCA	9	<nnxnp>	nn	129	np	141		
>ref-198734	CCGCACCCTACTACCCCTCCCTCCTTC	5	<lmxll>	ll	101	lm	138		
>ref-198734	CCGCACCCTACTACCCCTCCCTCCTTC	27	<lmxll>	ll	99	lm	140		
>ref-198737	CTACCTTACACTCACGCTCCCTCATCA	13	<lmxll>	ll	134	lm	129		
>ref-198748	CGCCCCCCTACCTCCTCTCCCCCCGGA	3	<lmxll>	ll	143	lm	120		
>ref-198764	CAAGTCCCAACTCGTCCTCCAAGTCCG	13	<nnxnp>	nn	265	np	3		
>ref-198764	CAAGTCCCAACTCGTCCTCCAAGTCCG	14	<nnxnp>	nn	123	np	131		
>ref-198807	GTTGCTGGCACAGCTGCTCCTCATATT	25	<lmxll>	ll	118	lm	133		
>ref-198836	GTCGCTCCCACCTGCACTCCAGTACAC	16	<nnxnp>	nn	267	np	0		
>ref-198841	CCTTCCTTGACCTCTCCTCCTTCTACC	5	<lmxll>	ll	119	lm	137		
>ref-198853	GAGGGGGGTACCTATTCTCCGCACCCG	8	<lmxll>	ll	131	lm	121		
>ref-198868	AGGCCTCCCACCTCCCCTCCCCCCTTC	4	<nnxnp>	nn	222	np	1		
>ref-198868	AGGCCTCCCACCTCCCCTCCCCCCTTC	8	<nnxnp>	nn	220	np	1		
>ref-198882	CCTCACCACACACTCCCTCCCTTACCA	15	<lmxll>	ll	223	lm	3		
>ref-198885	GACTTCCCCACTTCGTCTCCCTTCCCC	13	<nnxnp>	nn	126	np	143		

>ref-198885	GACTTCCCCACTTCGTCTCCCTTCCCC	21	<nnxnp>	nn	139	np	133		
>ref-198887	AGGCTGACAACACCGCCTCCTCCACCT	3	<lmxll>	ll	133	lm	135		
>ref-198894	CATTTTGCTACATATCCTCCACTGGAC	8	<nnxnp>	nn	127	np	135		
>ref-198912	ACGGCCACGACACGATCTCCCCCTTGA	2	<hkxhk>	hh	56	hk	155	kk	53
>ref-198935	TATCTTCCTACCCTCCCTCCTCCCTCC	1	<lmxll>	ll	13	lm	243		
>ref-198935	TATCTTCCTACCCTCCCTCCTCCCTCC	2	<lmxll>	ll	14	lm	240		
>ref-198977	TCCTCCTACACCACCCCTCCTTCCCCT	8	<lmxll>	ll	116	lm	119		
>ref-198977	TCCTCCTACACCACCCCTCCTTCCCCT	24	<lmxll>	ll	137	lm	116		
>ref-198986	CTCGCGCTCACTTCTTCTCCCTCTCGT	5	<lmxll>	ll	263	lm	0		
>ref-198987	TCTCTCAGCACCATCTCTCCCTCTCTC	21	<hkxhk>	hh	0	hk	272	kk	0
>ref-198987	TCTCTCAGCACCATCTCTCCCTCTCTC	24	<hkxhk>	hh	0	hk	272	kk	0
>ref-198993	AATATGAAAACATCATCTCCACCCAGT	16	<nnxnp>	nn	258	np	0		
>ref-199007	CTCCTCATAACTCATACTCCCTCCATC	12	<lmxll>	ll	68	lm	186		
>ref-199007	CTCCTCATAACTCATACTCCCTCCATC	14	<hkxhk>	hh	0	hk	273	kk	0
>ref-199007	CTCCTCATAACTCATACTCCCTCCATC	16	<hkxhk>	hh	21	hk	242	kk	0
>ref-199017	CTAATTGTTACTACACCTCCATTTCCA	16	<nnxnp>	nn	102	np	123		
>ref-199050	ATCTGCTGAACATGGTCTCCAGCTGAT	4	<nnxnp>	nn	148	np	115		
>ref-199053	ACTTTAAAAACTCTCTCTCCCTCTTCC	12	<hkxhk>	hh	2	hk	251	kk	5
>ref-199053	ACTTTAAAAACTCTCTCTCCCTCTTCC	13	<hkxhk>	hh	1	hk	172	kk	74
>ref-199063	TCGGAATCTACTGCACCTCCTCCCATA	13	<lmxll>	ll	129	lm	110		
>ref-199067	CCCTCAACAACACCGCCTCCCCCTCCC	1	<nnxnp>	nn	136	np	117		
>ref-199073	TACATATGAACGGAAACTCCGTAGATA	24	<hkxhk>	hh	60	hk	124	kk	51
>ref-199097	CCCTCATCCACCCCTTCTCCATCGTTC	3	<nnxnp>	nn	126	np	143		
>ref-199097	CCCTCATCCACCCCTTCTCCATCGTTC	16	<nnxnp>	nn	140	np	129		
>ref-199100	TATTAAGGTACTCCCTCTCCCCCTCCC	16	<lmxll>	ll	124	lm	136		
>ref-199108	CAATGTGCAACTACCACTCCAACCAAT	7	<nnxnp>	nn	135	np	129		
>ref-199111	CCTCTCTCTACCACTGCTCCTCCCTCT	2	<lmxll>	ll	237	lm	2		
>ref-199114	TATTCGCTGACACGACCTCCTAGCCTT	9	<nnxnp>	nn	149	np	121		
>ref-199119	TCCTTGTGGACACCCCCTCCCCCTTCT	27	<hkxhk>	hh	51	hk	141	kk	59
>ref-199156	TCCTGTTCCACCTGCTCTCCTGCCCTC	6	<hkxhk>	hh	56	hk	148	kk	60
>ref-199158	TGCCAGTGGACTCTAACTCCTCATATT	25	<nnxnp>	nn	104	np	115		
>ref-199182	ATCTTCACCACCACTTCTCCATCACTC	15	<lmxll>	ll	116	lm	138		
>ref-199184	CACAAATAAACACATTCTCCCTCTCGC	4	<nnxnp>	nn	113	np	150		
>ref-199212	ATTCCTTGCACCTCTCCTCCTCCCTCG	12	<nnxnp>	nn	230	np	0		
>ref-199219	CTCAATTTGACTTTCTCTCCGCCTCTC	26	<hkxhk>	hh	62	hk	131	kk	60
>ref-199256	ACCCTTTCGACATTTCCTCCGTGTCTA	27	<nnxnp>	nn	121	np	126		
>ref-199274	GTGTATTGGACACCCCCTCCTTCCCTT	14	<lmxll>	ll	107	lm	144		
>ref-199279	CACGTGGGCACGCTTGCTCCGCCCTTT	4	<nnxnp>	nn	109	np	156		

>ref-199279	CACGTGGGCACGCTTGCTCCGCCCTTT	7	<nnxnp>	nn	109	np	156		
>ref-199300	CCCTGCTTTACTTTCCCTCCTATTCCT	2	<lmxll>	ll	112	lm	138		
>ref-199331	GTAATTTACACTCACGCTCCCTCGCAG	1	<hkxhk>	hh	0	hk	262	kk	7
>ref-199331	GTAATTTACACTCACGCTCCCTCGCAG	15	<lmxll>	ll	98	lm	153		
>ref-199370	TAGGCAATCACACACGCTCCCACGGCG	21	<lmxll>	ll	157	lm	109		
>ref-199375	TGGGTCTCCACCTCCACTCCACTTCCC	14	<lmxll>	ll	64	lm	184		
>ref-199376	ATTATCCTCACTTTTGCTCCATCATCA	6	<nnxnp>	nn	133	np	125		
>ref-199377	ATGTGGATGACCTTCCCTCCCCATGTG	16	<nnxnp>	nn	9	np	260		
>ref-199377	ATGTGGATGACCTTCCCTCCCCATGTG	21	<nnxnp>	nn	8	np	261		
>ref-199382	CTCACTTTCACACACCCTCCTCCCCTC	4	<nnxnp>	nn	116	np	126		
>ref-199382	CTCACTTTCACACACCCTCCTCCCCTC	26	<nnxnp>	nn	120	np	125		
>ref-199385	ACTTACCCAACACGTCCTCCCTCCCCT	2	<hkxhk>	hh	0	hk	265	kk	4
>ref-199385	ACTTACCCAACACGTCCTCCCTCCCCT	6	<nnxnp>	nn	87	np	174		
>ref-199385	ACTTACCCAACACGTCCTCCCTCCCCT	7	<nnxnp>	nn	142	np	127		
>ref-199421	AAAGGAATCACATCCTCTCCTTCCATC	5	<nnxnp>	nn	265	np	0		
>ref-199421	AAAGGAATCACATCCTCTCCTTCCATC	22	<lmxll>	ll	265	lm	0		
>ref-199422	ACAACATCAACATCGTCTCCACAGTGC	1	<lmxll>	ll	1	lm	267		
>ref-199422	ACAACATCAACATCGTCTCCACAGTGC	15	<lmxll>	ll	1	lm	267		
>ref-199425	GCCTAAGCCACGCCTCCTCCATGTAAG	22	<nnxnp>	nn	137	np	119		
>ref-199436	ATGGCACCAACACCAACTCCAGCCCCT	16	<nnxnp>	nn	271	np	0		
>ref-199448	AGATTTTACACTCATGCTCCTCCTTCT	1	<lmxll>	ll	112	lm	125		
>ref-199452	ATAACCCTTACATGACCTCCCCCCTTC	13	<nnxnp>	nn	272	np	0		
>ref-199463	ATTCCTTGTACCGTTTCTCCCCCTTTC	23	<lmxll>	ll	134	lm	127		
>ref-199490	GTCAGCAATACTTTTCCTCCTAAAATT	5	<hkxhk>	hh	1	hk	213	kk	8
>ref-199490	GTCAGCAATACTTTTCCTCCTAAAATT	7	<hkxhk>	hh	8	hk	213	kk	1
>ref-199499	CATCTTCACACTCTCTCTCCTCTTATA	9	<nnxnp>	nn	116	np	103		
>ref-199505	TCCCTCCTTACCCCCCCTCCCTTCTCG	3	<hkxhk>	hh	0	hk	269	kk	3
>ref-199505	TCCCTCCTTACCCCCCCTCCCTTCTCG	5	<hkxhk>	hh	0	hk	269	kk	3
>ref-199505	TCCCTCCTTACCCCCCCTCCCTTCTCG	12	<hkxhk>	hh	20	hk	246	kk	0
>ref-199505	TCCCTCCTTACCCCCCCTCCCTTCTCG	23	<hkxhk>	hh	0	hk	248	kk	19
>ref-199555	CTCTCACCCACGTTCCCTCCCACGAGA	25	<hkxhk>	hh	1	hk	271	kk	1
>ref-199555	CTCTCACCCACGTTCCCTCCCACGAGA	26	<hkxhk>	hh	1	hk	271	kk	1
>ref-199570	CCATTGTGCACTATTGCTCCCAGGTCA	7	<lmxll>	ll	102	lm	137		
>ref-199576	CATTTGTCTACTCCTTCTCCCTATCCC	9	<lmxll>	ll	121	lm	105		
>ref-199580	CCCTCGCCCACCCTCTCTCCATCCCCC	14	<nnxnp>	nn	254	np	0		
>ref-199580	CCCTCGCCCACCCTCTCTCCATCCCCC	22	<lmxll>	ll	139	lm	122		
>ref-199592	TTTATTTGGACATTAACTCCTTATGGT	8	<hkxhk>	hh	0	hk	116	kk	112
>ref-199612	CAGAGAAGGACCTGACCTCCTCCTCAC	27	<nnxnp>	nn	123	np	145		

>ref-199620	CGTGCTGCTACAAGAACTCCCCCCTCT	7	<lmxll>	ll	136	lm	124		
>ref-199620	CGTGCTGCTACAAGAACTCCCCCCTCT	26	<nnxnp>	nn	11	np	239		
>ref-199665	GTTTTATCAACTATTCCTCCCCAAAGA	2	<hkxhk>	hh	0	hk	203	kk	40
>ref-199665	GTTTTATCAACTATTCCTCCCCAAAGA	7	<nnxnp>	nn	156	np	95		
>ref-199665	GTTTTATCAACTATTCCTCCCCAAAGA	24	<hkxhk>	hh	6	hk	259	kk	0
>ref-199676	GGCTCCTCCACTGTTCCTCCAGAACTA	1	<nnxnp>	nn	142	np	123		
>ref-199677	CAGGACGCGACAGCCTCTCCTCACTCA	24	<nnxnp>	nn	0	np	270		
>ref-199677	CAGGACGCGACAGCCTCTCCTCACTCA	25	<nnxnp>	nn	0	np	270		
>ref-199678	TTCCTCGTTACTTCTTCTCCCTCCTCC	15	<nnxnp>	nn	130	np	135		
>ref-199686	ACAGTTCCAACTAGTACTCCATTGACT	13	<nnxnp>	nn	134	np	118		
>ref-199691	GAGTTACTCACGCCACCTCCGTCACTC	16	<nnxnp>	nn	138	np	100		
>ref-199706	AAGTTTCCAACACATTCTCCAGCTAAG	7	<hkxhk>	hh	3	hk	266	kk	0
>ref-199728	GCTTCCATAACCCTCTCTCCCTCACCT	9	<hkxhk>	hh	5	hk	260	kk	0
>ref-199728	GCTTCCATAACCCTCTCTCCCTCACCT	27	<hkxhk>	hh	0	hk	260	kk	5
>ref-199738	TACAAGGAAACAAAACCTCCAGGGCCC	26	<lmxll>	ll	122	lm	144		
>ref-199739	CCGAATCAGACTCCGCCTCCTCACTCA	1	<nnxnp>	nn	148	np	114		
>ref-199739	CCGAATCAGACTCCGCCTCCTCACTCA	24	<nnxnp>	nn	113	np	153		
>ref-199766	CGTCACCTCACTCGACCTCCTCGCGTC	24	<lmxll>	ll	128	lm	140		
>ref-199775	TCTCTAACAACCCTAACTCCGTCTTTG	21	<lmxll>	ll	124	lm	129		
>ref-199826	TCCTATTCTACTTCATCTCCCTCTCTT	3	<lmxll>	ll	137	lm	125		
>ref-199846	AACTTTCTTACGCCGCCTCCAAGGTCG	13	<lmxll>	ll	149	lm	108		
>ref-199861	TTCCTCACCACGCACCCTCCTGCCCCC	13	<nnxnp>	nn	125	np	135		
>ref-199862	TCCACCTACACTTCCCCTCCACCTCTA	5	<lmxll>	ll	114	lm	139		
>ref-199862	TCCACCTACACTTCCCCTCCACCTCTA	26	<lmxll>	ll	2	lm	253		
>ref-199864	AGCTTGCGAACGTGCGCTCCGTCAGTG	5	<lmxll>	ll	135	lm	138		
>ref-199902	AACAGACAGACAGATACTCCTTATCAA	16	<lmxll>	ll	112	lm	158		
>ref-199926	CCAGACATAACTCGACCTCCTTGAAGA	2	<lmxll>	ll	128	lm	117		
>ref-199933	GGCTGGAACACCTCCCCTCCTTCCCTT	8	<lmxll>	ll	123	lm	129		
>ref-199955	ACATGATTAACCAGCTCTCCTTACTTG	14	<nnxnp>	nn	145	np	121		
>ref-199955	ACATGATTAACCAGCTCTCCTTACTTG	15	<nnxnp>	nn	146	np	121		
>ref-199966	CCGACCCTTACCAACCCTCCCCCTTCC	25	<hkxhk>	hh	44	hk	144	kk	57
>ref-199970	ACACCCACAACACTCACTCCACTGCAC	14	<nnxnp>	nn	120	np	119		
>ref-200003	GGAGATCCTACAGCAACTCCCTGCTGG	4	<hkxhk>	hh	0	hk	269	kk	0
>ref-200037	GACTCTCCCACTTTGTCTCCTATACAT	14	<hkxhk>	hh	50	hk	119	kk	67
>ref-200037	GACTCTCCCACTTTGTCTCCTATACAT	15	<hkxhk>	hh	50	hk	118	kk	68
>ref-200049	TCCCTTTTCACTGTTTCTCCTTCCCTC	3	<nnxnp>	nn	115	np	151		
>ref-200060	CTCATAGCCACTGTGGCTCCAGGTCCA	5	<hkxhk>	hh	0	hk	262	kk	1
>ref-200060	CTCATAGCCACTGTGGCTCCAGGTCCA	15	<hkxhk>	hh	0	hk	262	kk	1

>ref-200081	ACGTGATACACCCCGTCTCCCTCTCCT	26	<hkxhk>	hh	48	hk	142	kk	78
>ref-200098	TTCTCTGTCACGTGTCCTCCTGTTTCC	13	<nnxnp>	nn	143	np	97		
>ref-200111	CTTGTTCCCACCCATCCTCCCCCTTCC	24	<lmxll>	ll	134	lm	105		
>ref-200115	ATTCAGATCACATGACCTCCAACTGGC	1	<hkxhk>	hh	0	hk	267	kk	2
>ref-200116	CACACAAACACACCATCTCCTAACCAC	7	<hkxhk>	hh	70	hk	134	kk	52
>ref-200122	ATCTGTAGTACTCCCCCTCCCCCCTTC	26	<hkxhk>	hh	42	hk	124	kk	57
>ref-200154	CTAGCAAGAACATTCTCTCCTCTCTCT	1	<nnxnp>	nn	133	np	123		
>ref-200173	AACCTATCCACACCTTCTCCCTCATTA	25	<hkxhk>	hh	1	hk	267	kk	0
>ref-200178	AGTCCAACTACCCGCCCTCCCTTCCCT	24	<lmxll>	ll	113	lm	122		
>ref-200184	CGCTGGCTAACTTGGCCTCCGTCCTTG	6	<nnxnp>	nn	121	np	140		
>ref-200194	TTCCATACCACCGCCCCTCCCTTCCCC	1	<lmxll>	ll	32	lm	230		
>ref-200194	TTCCATACCACCGCCCCTCCCTTCCCC	2	<lmxll>	ll	31	lm	228		
>ref-200199	TACTACCCCACTCCCCCTCCCTTACTT	23	<hkxhk>	hh	56	hk	141	kk	53
>ref-200231	CGTCCCCCAACCCTGCCTCCCACGTTA	12	<hkxhk>	hh	66	hk	140	kk	56
>ref-200251	GAAAGTTATACATTATCTCCGTCATTT	6	<nnxnp>	nn	132	np	130		
>ref-200292	CTCACACTTACGCAGCCTCCTAAACAA	5	<nnxnp>	nn	119	np	136		
>ref-200296	AGACCTCGTACCTATTCTCCTTCACAA	14	<hkxhk>	hh	54	hk	122	kk	44
>ref-200298	TGCCTTTACACAGCTCCTCCATCGAAT	26	<nnxnp>	nn	100	np	133		
>ref-200300	CCGTCACTAACGGCATCTCCGCCCCAA	6	<hkxhk>	hh	1	hk	271	kk	0
>ref-200300	CCGTCACTAACGGCATCTCCGCCCCAA	8	<lmxll>	ll	67	lm	198		
>ref-200302	AAGAATCATACACTGACTCCATGAATA	23	<nnxnp>	nn	123	np	134		
>ref-200305	TCAAGATGAACAGCATCTCCAGCTGCA	12	<lmxll>	ll	129	lm	135		
>ref-200336	GAACCGGCAACTCCCCCTCCCTTCCCT	24	<lmxll>	ll	133	lm	131		
>ref-200407	GACGGTCACACAGACTCTCCATCCCAT	8	<hkxhk>	hh	55	hk	153	kk	61
>ref-200407	GACGGTCACACAGACTCTCCATCCCAT	21	<hkxhk>	hh	58	hk	153	kk	58
>ref-200413	TCTAACCACACGCTTTCTCCTCTCCCT	12	<nnxnp>	nn	119	np	150		
>ref-200413	TCTAACCACACGCTTTCTCCTCTCCCT	15	<nnxnp>	nn	119	np	150		
>ref-200414	CAAAACAAAACAAAGACTCCCAGAACT	1	<hkxhk>	hh	120	hk	131	kk	1
>ref-200431	ATATTACCTACTCCCTCTCCCTCCCAC	14	<nnxnp>	nn	19	np	220		
>ref-200431	ATATTACCTACTCCCTCTCCCTCCCAC	26	<nnxnp>	nn	18	np	221		
>ref-200435	ACCCTGAGCACGGACTCTCCGGACATA	1	<lmxll>	ll	98	lm	121		
>ref-200440	TCGATTTTAACGCTCACTCCATAATCT	26	<hkxhk>	hh	64	hk	111	kk	53
>ref-200450	CACTTTCCGACCTCCTCTCCCCCTCCC	5	<nnxnp>	nn	238	np	3		
>ref-200450	CACTTTCCGACCTCCTCTCCCCCTCCC	6	<nnxnp>	nn	249	np	0		
>ref-200450	CACTTTCCGACCTCCTCTCCCCCTCCC	16	<nnxnp>	nn	246	np	0		
>ref-200450	CACTTTCCGACCTCCTCTCCCCCTCCC	22	<nnxnp>	nn	239	np	3		
>ref-200456	AGTAACCAAACGCTGTCTCCTTCTCCC	24	<nnxnp>	nn	140	np	124		
>ref-200467	GGTACCCTAACCCCCCCTCCCCCTCTT	4	<hkxhk>	hh	2	hk	256	kk	1

>ref-200467	GGTACCCTAACCCCCCCTCCCCCTCTT	27	<hkxhk>	hh	1	hk	256	kk	2
>ref-200471	TACTCGTGTACTTCCCCTCCTCGCTCC	5	<lmxll>	ll	133	lm	127		
>ref-200473	AGATCAGTCACTGTAGCTCCTCAAGAT	1	<nnxnp>	nn	103	np	117		
>ref-200475	ATCATCTTTACTCTTCCTCCTTTTCCT	3	<hkxhk>	hh	66	hk	161	kk	0
>ref-200475	ATCATCTTTACTCTTCCTCCTTTTCCT	25	<lmxll>	ll	63	lm	166		
>ref-200475	ATCATCTTTACTCTTCCTCCTTTTCCT	27	<lmxll>	ll	63	lm	167		
>ref-200480	CCCCCCCACACACACACTCCGGCGTCA	21	<hkxhk>	hh	2	hk	212	kk	38
>ref-200480	CCCCCCCACACACACACTCCGGCGTCA	22	<hkxhk>	hh	2	hk	212	kk	39
>ref-200483	CTCGCGCACACACATACTCCTTCTCTA	14	<hkxhk>	hh	60	hk	126	kk	45
>ref-200508	CAGCACAACACAACGTCTCCCTTTCAC	13	<lmxll>	ll	137	lm	134		
>ref-200524	CTCCTCTCCACTCCCACTCCCATTCCC	16	<hkxhk>	hh	18	hk	236	kk	0
>ref-200524	CTCCTCTCCACTCCCACTCCCATTCCC	25	<hkxhk>	hh	21	hk	235	kk	0
>ref-200557	ACCCCATTTACTCCCCCTCCTTTTACC	24	<nnxnp>	nn	11	np	231		
>ref-200564	CATTCTCTTACTTTCCCTCCTTCCCCT	2	<lmxll>	ll	252	lm	1		
>ref-200564	CATTCTCTTACTTTCCCTCCTTCCCCT	21	<lmxll>	ll	257	lm	2		
>ref-200564	CATTCTCTTACTTTCCCTCCTTCCCCT	24	<nnxnp>	nn	257	np	1		
>ref-200573	GTCCTCTTCACTCCCTCTCCTTCTCTA	7	<lmxll>	ll	1	lm	270		
>ref-200573	GTCCTCTTCACTCCCTCTCCTTCTCTA	27	<lmxll>	ll	1	lm	270		
>ref-200616	GTTCTGCCCACCCATCCTCCTCTTACA	3	<nnxnp>	nn	136	np	123		
>ref-200629	TCTTACCTAACTCCTCCTCCCCTTCCT	5	<hkxhk>	hh	8	hk	258	kk	1
>ref-200629	TCTTACCTAACTCCTCCTCCCCTTCCT	14	<hkxhk>	hh	1	hk	258	kk	8
>ref-200632	ACCGCAGTCACCCAGTCTCCCATCACA	8	<nnxnp>	nn	140	np	127		
>ref-200632	ACCGCAGTCACCCAGTCTCCCATCACA	9	<nnxnp>	nn	140	np	127		
>ref-200639	TGCATCTCCACTCCACCTCCTATTGAA	5	<hkxhk>	hh	0	hk	272	kk	1
>ref-200639	TGCATCTCCACTCCACCTCCTATTGAA	22	<hkxhk>	hh	1	hk	272	kk	0
>ref-200639	TGCATCTCCACTCCACCTCCTATTGAA	26	<hkxhk>	hh	3	hk	267	kk	0
>ref-200643	CGGAGCATCACGGCAGCTCCGTGAATA	13	<hkxhk>	hh	51	hk	149	kk	57
>ref-200647	ATTATTTAGACGAGCTCTCCTGTTTTA	8	<nnxnp>	nn	257	np	0		
>ref-200653	TCATCCCCAACTTCATCTCCTTGTGAG	24	<nnxnp>	nn	141	np	105		
>ref-200674	TCGTCCCCCACCATCTCTCCCCACCAT	3	<hkxhk>	hh	2	hk	249	kk	9
>ref-200674	TCGTCCCCCACCATCTCTCCCCACCAT	5	<hkxhk>	hh	2	hk	250	kk	8
>ref-200713	ATACCAATTACACAACCTCCACCACAG	5	<nnxnp>	nn	255	np	0		
>ref-200714	GACCTATAGACCCCTCCTCCCTCTCCC	16	<lmxll>	ll	142	lm	125		
>ref-200717	TCTTTCCTAACCCTAACTCCTTCTTCT	1	<nnxnp>	nn	134	np	120		
>ref-200756	ATTAACGTTACTCGCTCTCCATGTCCA	14	<lmxll>	ll	138	lm	128		
>ref-200760	CCACCCGTAACATACTCTCCGCATGAT	27	<nnxnp>	nn	125	np	126		
>ref-200763	ACACGAGCAACAAACCCTCCCTCTTGC	8	<lmxll>	ll	141	lm	125		
>ref-200807	TCTCTCTCCACTTCCCCTCCCATTCAA	7	<lmxll>	ll	104	lm	146		

>ref-200827	CAGCCATGAACGCCAACTCCTCGCAGT	2	<hkxhk>	hh	0	hk	273	kk	0
>ref-200841	TGTGCAGCTACACGCACTCCTTCGTGT	14	<lmxll>	ll	111	lm	132		
>ref-200880	ATCGTTCTCACTCTCGCTCCCTATCCT	24	<nnxnp>	nn	129	np	141		
>ref-200883	TACCTTTCCACCTTTTCTCCCGTTCTC	1	<hkxhk>	hh	54	hk	138	kk	62
>ref-200883	TACCTTTCCACCTTTTCTCCCGTTCTC	2	<hkxhk>	hh	62	hk	138	kk	55
>ref-200890	TCTCTCTTTACCCCACCTCCACCTTCC	13	<hkxhk>	hh	1	hk	261	kk	4
>ref-200890	TCTCTCTTTACCCCACCTCCACCTTCC	25	<hkxhk>	hh	4	hk	261	kk	1
>ref-200901	GAACCCCTTACTGAACCTCCCGCAACC	1	<hkxhk>	hh	57	hk	132	kk	49
>ref-200902	AAAATCACCACCACCCCTCCACCATCA	3	<nnxnp>	nn	96	np	154		
>ref-200902	AAAATCACCACCACCCCTCCACCATCA	15	<nnxnp>	nn	97	np	154		
>ref-200940	CTCGGCGTGACCACAGCTCCTTTTGCA	12	<nnxnp>	nn	123	np	144		
>ref-200966	TTCTCTCCTACTCTTACTCCCTATTTG	16	<lmxll>	ll	101	lm	141		
>ref-200974	CCTATTGTCACGTCTTCTCCCTTTTTA	2	<nnxnp>	nn	120	np	129		
>ref-200977	CTCTCTCTGACTATGTCTCCAGTTTCT	15	<nnxnp>	nn	113	np	150		
>ref-200988	GAGGCTGCCACTCGCTCTCCTGTATTT	24	<nnxnp>	nn	256	np	0		
>ref-200991	TTTCCCCTCACCCCTCCTCCCTCACAC	7	<nnxnp>	nn	25	np	224		
>ref-200991	TTTCCCCTCACCCCTCCTCCCTCACAC	24	<nnxnp>	nn	24	np	226		
>ref-200994	GTAATTCCAACTCTCCCTCCATCTTGT	15	<nnxnp>	nn	104	np	128		
>ref-200996	TATGCATGCACGCCCACTCCCGAGGCC	26	<nnxnp>	nn	267	np	0		
>ref-201020	TCTCCCCACACCTTGACTCCCACACAC	7	<hkxhk>	hh	0	hk	273	kk	0
>ref-201020	TCTCCCCACACCTTGACTCCCACACAC	22	<hkxhk>	hh	0	hk	273	kk	0
>ref-201028	AACAACAGCACCATTGCTCCAACAACA	8	<hkxhk>	hh	7	hk	263	kk	1
>ref-201028	AACAACAGCACCATTGCTCCAACAACA	9	<hkxhk>	hh	10	hk	261	kk	0
>ref-201033	GATGCGCACACGGGGACTCCTCATTCA	1	<lmxll>	ll	117	lm	134		
>ref-201037	CCTCCAATCACACAAGCTCCTCCCTCC	12	<hkxhk>	hh	68	hk	125	kk	62
>ref-201042	ACAAACCACACACGGTCTCCAACGTTA	24	<hkxhk>	hh	57	hk	138	kk	71
>ref-201044	ACTGCTATCACATCCCCTCCCTGCCCG	6	<hkxhk>	hh	49	hk	148	kk	73
>ref-201045	TATTCTCCTACTTCCGCTCCTATCTAT	27	<lmxll>	ll	128	lm	113		
>ref-201055	AATATAATTACGGTCTCTCCCCCGGGT	4	<nnxnp>	nn	267	np	0		
>ref-201056	CGTACCAGCACGAGTTCTCCGATCTCT	8	<hkxhk>	hh	0	hk	265	kk	2
>ref-201085	TCTCCACTCACTCCTTCTCCTTCTCTC	6	<lmxll>	ll	95	lm	147		
>ref-201085	TCTCCACTCACTCCTTCTCCTTCTCTC	14	<lmxll>	ll	116	lm	140		
>ref-201087	ATATAAGGTACCCTTGCTCCTTCCCCA	24	<hkxhk>	hh	1	hk	264	kk	3
>ref-201097	ACATACATTACACAATCTCCCAGTAAC	14	<nnxnp>	nn	139	np	106		
>ref-201098	TCGCCCTCCACCTTCTCTCCCTTTCTA	6	<lmxll>	ll	130	lm	125		
>ref-201098	TCGCCCTCCACCTTCTCTCCCTTTCTA	9	<lmxll>	ll	136	lm	120		
>ref-201104	AATACAGCCACTATTACTCCTGCTGCT	7	<lmxll>	ll	133	lm	129		
>ref-201128	TCCTCCGCTACCCTTCCTCCGCTACCC	7	<lmxll>	ll	50	lm	177		

>ref-201128	TCCTCCGCTACCCTTCCTCCGCTACCC	21	<lmxll>	ll	67	lm	165		
>ref-201131	TATCTTGGTACTTTTTCTCCCTCACAC	6	<hkxhk>	hh	0	hk	121	kk	118
>ref-201131	TATCTTGGTACTTTTTCTCCCTCACAC	27	<lmxll>	ll	124	lm	109		
>ref-201135	CCCTCCCACACCCACTCTCCTCCAACC	16	<lmxll>	ll	108	lm	131		
>ref-201137	TGCTCCACCACCATGTCTCCCTTCCTT	1	<lmxll>	ll	15	lm	256		
>ref-201137	TGCTCCACCACCATGTCTCCCTTCCTT	3	<nnxnp>	nn	22	np	249		
>ref-201199	CCTCCCTTCACTCTCCCTCCCTGTCCA	3	<hkxhk>	hh	0	hk	265	kk	4
>ref-201203	CGCTCAGGAACAATGCCTCCTGCGATC	4	<nnxnp>	nn	106	np	159		
>ref-201203	CGCTCAGGAACAATGCCTCCTGCGATC	16	<nnxnp>	nn	110	np	154		
>ref-201207	GTTCCGATTACCCTTACTCCCTAATTC	15	<hkxhk>	hh	48	hk	126	kk	64
>ref-201229	CCATCCCTCACTTCCCCTCCTCCTCTC	16	<nnxnp>	nn	258	np	2		
>ref-201229	CCATCCCTCACTTCCCCTCCTCCTCTC	22	<nnxnp>	nn	261	np	2		
>ref-201239	AAGCTATGAACCCCCCCTCCCATGCTT	26	<nnxnp>	nn	132	np	138		
>ref-201251	TCCATCCCCACGTTATCTCCCTCCCTT	3	<hkxhk>	hh	79	hk	189	kk	1
>ref-201276	CGGAGCAACACAGACCCTCCTCGTTTG	13	<hkxhk>	hh	52	hk	137	kk	50
>ref-201276	CGGAGCAACACAGACCCTCCTCGTTTG	26	<hkxhk>	hh	50	hk	137	kk	52
>ref-201294	GCACTTAACACCCCCTCTCCTCTGTTG	1	<hkxhk>	hh	0	hk	243	kk	20
>ref-201294	GCACTTAACACCCCCTCTCCTCTGTTG	3	<hkxhk>	hh	0	hk	272	kk	0
>ref-201294	GCACTTAACACCCCCTCTCCTCTGTTG	4	<hkxhk>	hh	0	hk	58	kk	200
>ref-201294	GCACTTAACACCCCCTCTCCTCTGTTG	12	<lmxll>	ll	79	lm	189		
>ref-201310	GTTTTGCCAACTGAATCTCCTAACGCT	2	<lmxll>	ll	127	lm	142		
>ref-201376	CCTTCCTTCACCCCTTCTCCTAAACCC	26	<lmxll>	ll	242	lm	0		
>ref-201390	CCTCTACCCACACCCCCTCCTCTACCC	2	<hkxhk>	hh	0	hk	225	kk	34
>ref-201390	CCTCTACCCACACCCCCTCCTCTACCC	4	<hkxhk>	hh	44	hk	205	kk	0
>ref-201390	CCTCTACCCACACCCCCTCCTCTACCC	14	<nnxnp>	nn	208	np	52		
>ref-201399	TCCGGACGCACCCTCTCTCCGCCCTTG	15	<nnxnp>	nn	130	np	141		
>ref-201433	TGGTCTTCCACTTACACTCCAGCCGAA	5	<hkxhk>	hh	1	hk	272	kk	0
>ref-201433	TGGTCTTCCACTTACACTCCAGCCGAA	24	<hkxhk>	hh	29	hk	232	kk	0
>ref-201445	GCACTGATCACCTTTTCTCCTGTCACG	1	<lmxll>	ll	122	lm	136		
>ref-201446	TACAAATCCACTACCACTCCTTTCTCA	16	<hkxhk>	hh	6	hk	260	kk	0
>ref-201446	TACAAATCCACTACCACTCCTTTCTCA	23	<hkxhk>	hh	0	hk	260	kk	6
>ref-201448	TTGAGATCAACATAAGCTCCAAGAATA	21	<hkxhk>	hh	1	hk	270	kk	0
>ref-201448	TTGAGATCAACATAAGCTCCAAGAATA	24	<hkxhk>	hh	1	hk	270	kk	0
>ref-201450	CTCATTCCCACTCCATCTCCATCATCA	1	<nnxnp>	nn	136	np	127		
>ref-201454	CCCTCACCTACGCTCGCTCCCGGCCTC	23	<nnxnp>	nn	264	np	0		
>ref-201456	GAAGACATTACACCACCTCCCCAGGAA	6	<lmxll>	ll	115	lm	104		
>ref-201476	CCCTCCGTCACCCCCCCTCCTCTTCAC	1	<lmxll>	ll	7	lm	236		
>ref-201476	CCCTCCGTCACCCCCCCTCCTCTTCAC	2	<lmxll>	ll	256	lm	0		

>ref-201476	CCCTCCGTCACCCCCCCTCCTCTTCAC	5	<lmxll>	ll	256	lm	0		
>ref-201476	CCCTCCGTCACCCCCCCTCCTCTTCAC	6	<lmxll>	ll	254	lm	0		
>ref-201476	CCCTCCGTCACCCCCCCTCCTCTTCAC	27	<lmxll>	ll	15	lm	227		
>ref-201491	TATAAACGCACAATCTCTCCTCGGACA	1	<hkxhk>	hh	0	hk	272	kk	1
>ref-201491	TATAAACGCACAATCTCTCCTCGGACA	8	<hkxhk>	hh	0	hk	257	kk	10
>ref-201491	TATAAACGCACAATCTCTCCTCGGACA	13	<nnxnp>	nn	134	np	117		
>ref-201491	TATAAACGCACAATCTCTCCTCGGACA	26	<hkxhk>	hh	11	hk	261	kk	0
>ref-201500	ACCCACCCCACTCCCTCTCCCCTCATA	6	<lmxll>	ll	188	lm	68		
>ref-201500	ACCCACCCCACTCCCTCTCCCCTCATA	14	<nnxnp>	nn	68	np	180		
>ref-201520	TTCTGCTGCACTCTTCCTCCAGAGAGC	13	<lmxll>	ll	140	lm	120		
>ref-201526	CACGTATAAACAAACACTCCTTCACCC	4	<hkxhk>	hh	69	hk	141	kk	32
>ref-201562	TGACCGCTCACATGTGCTCCTTAGTAG	9	<hkxhk>	hh	20	hk	238	kk	0
>ref-201562	TGACCGCTCACATGTGCTCCTTAGTAG	21	<hkxhk>	hh	0	hk	235	kk	23
>ref-201579	TGGATTAACACTCCTCCTCCTCCTCCT	1	<nnxnp>	nn	247	np	1		
>ref-201579	TGGATTAACACTCCTCCTCCTCCTCCT	2	<nnxnp>	nn	231	np	0		
>ref-201579	TGGATTAACACTCCTCCTCCTCCTCCT	3	<nnxnp>	nn	237	np	4		
>ref-201579	TGGATTAACACTCCTCCTCCTCCTCCT	7	<nnxnp>	nn	247	np	0		
>ref-201581	CAACACATAACTCATTCTCCCTCTGCT	2	<lmxll>	ll	140	lm	114		
>ref-201587	CGTGCTGCTACAGGTACTCCCCTCCAG	12	<hkxhk>	hh	0	hk	272	kk	0
>ref-201587	CGTGCTGCTACAGGTACTCCCCTCCAG	14	<hkxhk>	hh	0	hk	272	kk	0
>ref-201587	CGTGCTGCTACAGGTACTCCCCTCCAG	22	<hkxhk>	hh	29	hk	233	kk	0
>ref-201587	CGTGCTGCTACAGGTACTCCCCTCCAG	25	<hkxhk>	hh	0	hk	233	kk	29
>ref-201599	TGCTCACGTACACAGCCTCCATGTACT	3	<hkxhk>	hh	65	hk	150	kk	56
>ref-201622	CAAAAGACCACGCCCACTCCCATCCTC	26	<nnxnp>	nn	270	np	0		
>ref-201633	CATGTCAATACTCACGCTCCCTCCCCT	15	<lmxll>	ll	248	lm	0		
>ref-201678	AACCTCACTACCTTCTCTCCGGCTCTT	26	<nnxnp>	nn	273	np	0		
>ref-201688	GAACAGCGAACTTTCTCTCCAGTTATT	21	<lmxll>	ll	99	lm	138		
>ref-201700	CCTGCCCTCACCCCCACTCCACTAAAT	15	<lmxll>	ll	125	lm	117		
>ref-201754	TTAAGTGTGACTCACTCTCCTCTCCTT	26	<nnxnp>	nn	263	np	0		
>ref-201768	GAAAACATAACCCCCGCTCCGCTCATA	24	<hkxhk>	hh	57	hk	120	kk	61
>ref-201771	CACATTTCCACAATTCCTCCTTTAAGG	4	<nnxnp>	nn	118	np	118		
>ref-201777	CCTATCCCCACTCCCACTCCCCCCACC	16	<hkxhk>	hh	54	hk	186	kk	0
>ref-201777	CCTATCCCCACTCCCACTCCCCCCACC	24	<hkxhk>	hh	61	hk	169	kk	0
>ref-201779	CCCTCCCCTACCCTGCCTCCTTATCCT	15	<hkxhk>	hh	1	hk	269	kk	0
>ref-201779	CCCTCCCCTACCCTGCCTCCTTATCCT	21	<hkxhk>	hh	0	hk	269	kk	1
>ref-201785	GAACAGTCGACACCAGCTCCTCTTCTA	9	<hkxhk>	hh	8	hk	250	kk	0
>ref-201785	GAACAGTCGACACCAGCTCCTCTTCTA	13	<hkxhk>	hh	0	hk	268	kk	0
>ref-201785	GAACAGTCGACACCAGCTCCTCTTCTA	26	<lmxll>	ll	53	lm	195		

>ref-201786	TCTCAGTCAACGCAGCCTCCCATCATA	3	<nnxnp>	nn	124	np	146		
>ref-201799	TTGAGTCGCACTTCAGCTCCCTTTCAT	9	<lmxll>	ll	111	lm	152		
>ref-201810	CTCGCGCCTACTCACCCTCCCCTCCTC	8	<nnxnp>	nn	242	np	0		
>ref-201812	GCGTGACCAACGGCGCCTCCAACGGCC	9	<lmxll>	ll	133	lm	129		
>ref-201817	ACTTAACAAACTTGATCTCCCAATACT	6	<nnxnp>	nn	117	np	109		
>ref-201818	CAGTGCTACACTATTCCTCCCAACATA	16	<hkxhk>	hh	0	hk	269	kk	1
>ref-201818	CAGTGCTACACTATTCCTCCCAACATA	21	<hkxhk>	hh	1	hk	269	kk	0
>ref-201818	CAGTGCTACACTATTCCTCCCAACATA	25	<hkxhk>	hh	2	hk	270	kk	0
>ref-201820	TCCTTCCTAACTCTTCCTCCCCTTTCT	12	<lmxll>	ll	122	lm	141		
>ref-201852	TCAACCCTCACCTTTCCTCCCTCTCCC	5	<nnxnp>	nn	264	np	0		
>ref-201852	TCAACCCTCACCTTTCCTCCCTCTCCC	12	<lmxll>	ll	264	lm	0		
>ref-201856	TCCTTGCCTACTCTCTCTCCCTTTTCT	16	<nnxnp>	nn	118	np	119		
>ref-201866	CTTCTTCTCACTCCTTCTCCTCACCCT	2	<nnxnp>	nn	108	np	140		
>ref-201866	CTTCTTCTCACTCCTTCTCCTCACCCT	23	<nnxnp>	nn	97	np	145		
>ref-201870	ATCACTATCACTCTGTCTCCATATGGA	21	<lmxll>	ll	130	lm	118		
>ref-201872	AATACTGCTACCCCTTCTCCTACTAGT	4	<lmxll>	ll	117	lm	133		
>ref-201872	AATACTGCTACCCCTTCTCCTACTAGT	21	<nnxnp>	nn	103	np	143		
>ref-201879	ATTAATAACACTTTCTCTCCACCTAAC	8	<lmxll>	ll	114	lm	125		
>ref-201883	AACAAACACACACAAACTCCTAAACAA	9	<hkxhk>	hh	0	hk	169	kk	94
>ref-201883	AACAAACACACACAAACTCCTAAACAA	27	<hkxhk>	hh	94	hk	169	kk	0
>ref-201888	CAACAGAAAACGTGCTCTCCAACAGTG	6	<hkxhk>	hh	49	hk	145	kk	57
>ref-201910	CAAACACAAACGCATGCTCCCAAACAA	3	<lmxll>	ll	269	lm	0		
>ref-201919	CGTCCGAGCACCTCTTCTCCGCCAGAG	8	<lmxll>	ll	132	lm	137		
>ref-201926	AAGGCAATAACAGATCCTCCTTTTGCT	25	<lmxll>	ll	160	lm	110		
>ref-201936	CGACAGGCAACCGAAGCTCCGCCTGCT	3	<hkxhk>	hh	65	hk	137	kk	59
>ref-201954	CTCACCCTCACCCTCCCTCCCAACGTA	21	<lmxll>	ll	107	lm	132		
>ref-201985	CTCAACACCACACTACCTCCCTCCCTC	27	<lmxll>	ll	125	lm	137		
>ref-201996	TTGTTACGCACCATCCCTCCCCTCCCC	4	<lmxll>	ll	125	lm	135		
>ref-201998	TATGTCCCCACACCTTCTCCTCCTTTC	1	<lmxll>	ll	228	lm	6		
>ref-201998	TATGTCCCCACACCTTCTCCTCCTTTC	2	<lmxll>	ll	239	lm	1		
>ref-202008	TCCTTCCCAACCCTCCCTCCACTCCCA	13	<lmxll>	ll	104	lm	148		
>ref-202010	GCTAGACGTACGTCCACTCCTAACTTT	7	<nnxnp>	nn	107	np	128		
>ref-202053	CGAGGAGCCACTTTATCTCCATTTGAT	9	<hkxhk>	hh	66	hk	141	kk	51
>ref-202071	TTCCTTCCTACCGTTCCTCCTTCTTTC	2	<lmxll>	ll	108	lm	130		
>ref-202097	TGAGATGACACGTTCTCTCCTCTGTGA	12	<nnxnp>	nn	96	np	145		
>ref-202109	AAGCACCTCACACTCTCTCCCTCCCTC	12	<hkxhk>	hh	1	hk	271	kk	0
>ref-202124	GCTCTCGCTACTGCTCCTCCTCCTCCT	13	<lmxll>	ll	244	lm	10		
>ref-202131	ACCACGCTCACGCAGCCTCCCCCTCAG	13	<nnxnp>	nn	123	np	141		

>ref-202134	ACACACACAACCACCCCTCCCCCCTCC	16	<hkxhk>	hh	1	hk	272	kk	0
>ref-202162	CTCTGCCTTACTCTTTCTCCTCTCTCC	3	<hkxhk>	hh	3	hk	270	kk	0
>ref-202162	CTCTGCCTTACTCTTTCTCCTCTCTCC	13	<hkxhk>	hh	3	hk	270	kk	0
>ref-202181	CCCCAATATACCCCTCCTCCCTCTTCC	5	<nnxnp>	nn	162	np	89		
>ref-202181	CCCCAATATACCCCTCCTCCCTCTTCC	24	<nnxnp>	nn	160	np	89		
>ref-202223	CAACGGCAAACGAATGCTCCAGGGAGG	2	<hkxhk>	hh	0	hk	273	kk	0
>ref-202223	CAACGGCAAACGAATGCTCCAGGGAGG	14	<hkxhk>	hh	0	hk	273	kk	0
>ref-202223	CAACGGCAAACGAATGCTCCAGGGAGG	22	<nnxnp>	nn	146	np	125		
>ref-202239	CCCACCTTAACCATGTCTCCTTCCCCA	3	<hkxhk>	hh	41	hk	164	kk	53
>ref-202239	CCCACCTTAACCATGTCTCCTTCCCCA	12	<hkxhk>	hh	52	hk	165	kk	42
>ref-202240	AACACTTTCACACAGGCTCCCTCCCTG	2	<nnxnp>	nn	128	np	91		
>ref-202268	ATGAAAAAAACAGTCGCTCCTTCACTT	22	<hkxhk>	hh	68	hk	134	kk	63
>ref-202282	ATCCCAGGGACGCCTCCTCCCTCGGGT	5	<nnxnp>	nn	128	np	136		
>ref-202286	CTGCCTTTCACTCAGGCTCCTCTTCCC	15	<nnxnp>	nn	144	np	127		
>ref-202306	CCCCAGCCCACCTTCACTCCTTCAGCG	16	<nnxnp>	nn	128	np	144		
>ref-202310	CAGGGTCGCACAGCACCTCCAGGAGCT	13	<nnxnp>	nn	111	np	155		
>ref-202329	TTCACTGGAACTTTATCTCCACATTAC	1	<nnxnp>	nn	122	np	125		
>ref-202329	TTCACTGGAACTTTATCTCCACATTAC	21	<lmxll>	ll	140	lm	118		
>ref-202334	AGAGACCGCACAAAGCCTCCTCTTCTC	12	<nnxnp>	nn	112	np	152		
>ref-202334	AGAGACCGCACAAAGCCTCCTCTTCTC	15	<nnxnp>	nn	147	np	119		
>ref-202379	CCTGAATCTACCCACTCTCCTCACTAT	13	<nnxnp>	nn	128	np	121		
>ref-202388	AAAGGAAGGACAAAAGCTCCATCTCCT	24	<nnxnp>	nn	111	np	148		
>ref-202401	CCTCCCACCACCTTCGCTCCCTCCCCC	14	<nnxnp>	nn	122	np	147		
>ref-202415	AGTGACGTTACTGTTACTCCTTTTTTG	5	<lmxll>	ll	123	lm	130		
>ref-202430	TCCCTCCTTACAGCCTCTCCCCTATCC	14	<lmxll>	ll	171	lm	79		
>ref-202431	CGTTGCGGTACAGTTCCTCCACACTCC	14	<hkxhk>	hh	57	hk	134	kk	63
>ref-202442	GAGACCCTGACGCTCGCTCCCTTTCCA	1	<lmxll>	ll	136	lm	130		
>ref-202442	GAGACCCTGACGCTCGCTCCCTTTCCA	2	<lmxll>	ll	136	lm	130		
>ref-202447	TGTACACACACATATACTCCCTCCCCG	22	<nnxnp>	nn	266	np	0		
>ref-202450	ACAGAAAACACTCACCCTCCCCTCTTA	13	<lmxll>	ll	126	lm	139		
>ref-202461	CATCTTTCTACCTTTTCTCCGCCGTTC	27	<lmxll>	ll	121	lm	138		
>ref-202475	CCCCACAAGACCCTTTCTCCCGGCTTA	3	<nnxnp>	nn	136	np	129		
>ref-202490	TCAACGAATACTGTTGCTCCTGCCGGC	23	<nnxnp>	nn	128	np	122		
>ref-202492	CTGCCTTGCACGTGGGCTCCTATCCTG	15	<lmxll>	ll	137	lm	134		
>ref-202517	CTCTTTGCTACTCCTGCTCCCCCTCCT	7	<nnxnp>	nn	7	np	256		
>ref-202524	AACACTGCCACACTATCTCCCTCCTAC	8	<nnxnp>	nn	125	np	141		
>ref-202524	AACACTGCCACACTATCTCCCTCCTAC	24	<lmxll>	ll	130	lm	138		
>ref-202524	AACACTGCCACACTATCTCCCTCCTAC	25	<hkxhk>	hh	52	hk	150	kk	58

>ref-202532	CTCTAGTTCACCTGCCCTCCGTCTGAG	14	<lmxll>	ll	135	lm	131		
>ref-202537	CGCACGCACACCCACACTCCTATCTAT	13	<lmxll>	ll	124	lm	145		
>ref-202551	CTGTGGTCGACTGTCTCTCCTGGCTTC	9	<lmxll>	ll	9	lm	263		
>ref-202574	TCTCGTAGGACTCCCTCTCCTTCTTCT	23	<hkxhk>	hh	65	hk	134	kk	50
>ref-202580	CCATTACCCACCTCTTCTCCTCATCTC	1	<lmxll>	ll	146	lm	97		
>ref-202580	CCATTACCCACCTCTTCTCCTCATCTC	22	<lmxll>	ll	108	lm	137		
>ref-202580	CCATTACCCACCTCTTCTCCTCATCTC	25	<lmxll>	ll	146	lm	97		
>ref-202590	TGAGTCCCAACTATAGCTCCCCCTTGT	25	<hkxhk>	hh	0	hk	124	kk	109
>ref-202600	CATACATACACCGAAACTCCATGATAC	7	<hkxhk>	hh	0	hk	262	kk	2
>ref-202600	CATACATACACCGAAACTCCATGATAC	9	<hkxhk>	hh	0	hk	139	kk	96
>ref-202606	GCAACAACTACTCTCTCTCCTAATGGG	4	<lmxll>	ll	135	lm	126		
>ref-202612	TTCTCAGCTACCCACCCTCCCTCCTTC	8	<hkxhk>	hh	40	hk	179	kk	0
>ref-202614	CTGAATAGAACCTGCACTCCTACTCCG	3	<hkxhk>	hh	1	hk	259	kk	1
>ref-202614	CTGAATAGAACCTGCACTCCTACTCCG	14	<hkxhk>	hh	2	hk	256	kk	1
>ref-202615	CATCATTACACTCTCTCTCCCTCCCTC	1	<hkxhk>	hh	0	hk	271	kk	1
>ref-202615	CATCATTACACTCTCTCTCCCTCCCTC	2	<hkxhk>	hh	1	hk	271	kk	0
>ref-202615	CATCATTACACTCTCTCTCCCTCCCTC	16	<hkxhk>	hh	0	hk	273	kk	0
>ref-202615	CATCATTACACTCTCTCTCCCTCCCTC	24	<hkxhk>	hh	0	hk	273	kk	0
>ref-202616	CACACACATACACCGACTCCCAACTAC	7	<nnxnp>	nn	133	np	114		
>ref-202645	CGAGGCCTCACTTTCTCTCCGGCGTTG	4	<nnxnp>	nn	161	np	107		
>ref-202645	CGAGGCCTCACTTTCTCTCCGGCGTTG	7	<nnxnp>	nn	148	np	112		
>ref-202664	AGAGCACCGACTGCGGCTCCCTCAGCA	8	<lmxll>	ll	146	lm	85		
>ref-202668	TTTTATCAAACCTTCCCTCCTTCCCTC	5	<nnxnp>	nn	228	np	1		
>ref-202670	GTGCCAGAAACAGATACTCCACCCAAT	22	<nnxnp>	nn	134	np	138		
>ref-202671	TACATGATGACGCACGCTCCACTCTGC	24	<lmxll>	ll	111	lm	151		
>ref-202704	TGTAGCAGTACACTGTCTCCTCCGTTT	8	<lmxll>	ll	109	lm	112		
>ref-202711	AGGACCCAGACAGGTGCTCCTCCTCCA	8	<lmxll>	ll	159	lm	110		
>ref-202718	TACCATTTAACTAACCCTCCTCCTCCC	25	<nnxnp>	nn	110	np	135		
>ref-202737	TCGCTGGCAACCTCCTCTCCCCTCCTC	2	<lmxll>	ll	140	lm	124		
>ref-202762	GCGTGCATTACGAGAGCTCCCTGTACA	13	<lmxll>	ll	133	lm	134		
>ref-202764	TATTCTCTAACTTTTTCTCCTCTTCTC	13	<lmxll>	ll	130	lm	112		
>ref-202773	GAAGAATTTACCTGATCTCCGCGCCTG	22	<lmxll>	ll	122	lm	139		
>ref-202785	CCCCTCCTCACTCTCTCTCCATGTCTT	23	<nnxnp>	nn	142	np	121		
>ref-202818	GTATAGTTGACCCCCACTCCTTAAGCA	23	<lmxll>	ll	126	lm	132		
>ref-202837	TACACACAAACACACGCTCCCACGTGC	5	<lmxll>	ll	129	lm	136		
>ref-202845	GGTACATTTACTCTTACTCCTTGTCCC	24	<hkxhk>	hh	45	hk	130	kk	64
>ref-202849	GCATCCTTCACCCCTCCTCCTTCTCCT	1	<nnxnp>	nn	133	np	122		
>ref-202849	GCATCCTTCACCCCTCCTCCTTCTCCT	6	<nnxnp>	nn	146	np	116		

>ref-202852	ACCTTCCCTACCCCTTCTCCTTTTTCC	2	<lmxll>	ll	266	lm	0		
>ref-202852	ACCTTCCCTACCCCTTCTCCTTTTTCC	22	<nnxnp>	nn	264	np	0		
>ref-202862	CCCCATCTGACACGTTCTCCCCATCTG	5	<hkxhk>	hh	0	hk	248	kk	2
>ref-202870	CTTCCCCTTACAGCTCCTCCCTCCCTC	14	<nnxnp>	nn	136	np	104		
>ref-202896	CGCATTCGAACGAGTCCTCCAACCCCA	21	<nnxnp>	nn	116	np	143		
>ref-202896	CGCATTCGAACGAGTCCTCCAACCCCA	22	<nnxnp>	nn	116	np	143		
>ref-202898	TCATCCTTCACCCCTACTCCCTCCCTC	3	<lmxll>	ll	9	lm	245		
>ref-202898	TCATCCTTCACCCCTACTCCCTCCCTC	16	<lmxll>	ll	9	lm	245		
>ref-202899	TCCGTTTGGACGCGACCTCCTCTCGGG	25	<lmxll>	ll	127	lm	143		
>ref-202918	TTGCTCTCTACTCTCCCTCCCTCTCAT	15	<nnxnp>	nn	109	np	122		
>ref-202920	TTTGGCATTACTAACTCTCCACGCAGC	9	<nnxnp>	nn	271	np	0		
>ref-202939	ACTCTACACACCTGGCCTCCCTTACCT	24	<lmxll>	ll	131	lm	136		
>ref-202954	CACTCTTCCACCCTCCCTCCCTTTTCT	2	<nnxnp>	nn	125	np	111		
>ref-202954	CACTCTTCCACCCTCCCTCCCTTTTCT	26	<nnxnp>	nn	138	np	111		
>ref-202959	TCGCTGCTGACAGGGACTCCTGGAAGC	2	<hkxhk>	hh	0	hk	273	kk	0
>ref-202959	TCGCTGCTGACAGGGACTCCTGGAAGC	5	<lmxll>	ll	36	lm	226		
>ref-202959	TCGCTGCTGACAGGGACTCCTGGAAGC	6	<hkxhk>	hh	0	hk	244	kk	20
>ref-202959	TCGCTGCTGACAGGGACTCCTGGAAGC	21	<hkxhk>	hh	0	hk	246	kk	0
>ref-202959	TCGCTGCTGACAGGGACTCCTGGAAGC	23	<hkxhk>	hh	0	hk	214	kk	40
>ref-202959	TCGCTGCTGACAGGGACTCCTGGAAGC	26	<lmxll>	ll	130	lm	124		
>ref-202964	TTTACTCTCACCCTCCCTCCATCTTCC	16	<hkxhk>	hh	2	hk	252	kk	7
>ref-202964	TTTACTCTCACCCTCCCTCCATCTTCC	25	<hkxhk>	hh	2	hk	254	kk	6
>ref-202966	TACTACTTAACAGCCCCTCCCCCCCTC	25	<nnxnp>	nn	138	np	118		
>ref-202966	TACTACTTAACAGCCCCTCCCCCCCTC	26	<nnxnp>	nn	137	np	120		
>ref-202973	CTACCTTCTACCACCTCTCCCTTCCCC	6	<hkxhk>	hh	1	hk	223	kk	41
>ref-202973	CTACCTTCTACCACCTCTCCCTTCCCC	13	<hkxhk>	hh	76	hk	178	kk	0
>ref-202975	CGCCCCTGTACCCTCCCTCCACGCCCA	15	<nnxnp>	nn	99	np	137		
>ref-202999	TCCCATTCCACTTAGCCTCCGGGCTTG	26	<nnxnp>	nn	129	np	137		
>ref-203025	CATTAACAGACTAACACTCCTGAGAAT	1	<lmxll>	ll	119	lm	131		
>ref-203028	ATACGATGGACGCAACCTCCCGTCAAA	3	<lmxll>	ll	120	lm	138		
>ref-203028	ATACGATGGACGCAACCTCCCGTCAAA	26	<lmxll>	ll	122	lm	137		
>ref-203058	TCATGGCTCACTTCCACTCCAGCACCA	25	<lmxll>	ll	119	lm	124		
>ref-203060	TAAATTTGGACGAAGGCTCCGCGGGGA	26	<lmxll>	ll	122	lm	141		
>ref-203078	CTTCCCCTCACTTCTCCTCCCCATCAA	1	<nnxnp>	nn	264	np	1		
>ref-203115	TTGTTATTCACCGGTCCTCCTCAATTA	1	<lmxll>	ll	114	lm	144		
>ref-203129	GACACAGCCACTTCTACTCCCATCCCT	16	<nnxnp>	nn	273	np	0		
>ref-203153	TCATGTGAAACCAATGCTCCTCCATTT	14	<nnxnp>	nn	30	np	230		
>ref-203153	TCATGTGAAACCAATGCTCCTCCATTT	15	<nnxnp>	nn	31	np	230		

>ref-203158	GGGGTCCCTACATCCTCTCCGCGTCCT	14	<lmxll>	ll	138	lm	130		
>ref-203172	TCAAATCAAACCTAAACTCCACAAGCA	7	<nnxnp>	nn	120	np	118		
>ref-203172	TCAAATCAAACCTAAACTCCACAAGCA	23	<lmxll>	ll	114	lm	122		
>ref-203172	TCAAATCAAACCTAAACTCCACAAGCA	24	<lmxll>	ll	114	lm	122		
>ref-203173	TTTCTTACTACCCTTCCTCCTTTCCCT	8	<lmxll>	ll	115	lm	135		
>ref-203175	CTCACAGTGACTCATACTCCGTTGTAC	14	<nnxnp>	nn	127	np	138		
>ref-203182	ATCGTCTTCACCCTCTCTCCTTTCCCT	27	<lmxll>	ll	237	lm	4		
>ref-203187	CTCCCTCCCACCTGGTCTCCTTTTCCC	16	<nnxnp>	nn	4	np	253		
>ref-203212	CCTATTCCCACTCTCCCTCCAGCTCCC	8	<lmxll>	ll	129	lm	135		
>ref-203264	TCTGCATGCACTCCTTCTCCTTCTCCT	8	<nnxnp>	nn	50	np	212		
>ref-203264	TCTGCATGCACTCCTTCTCCTTCTCCT	22	<lmxll>	ll	119	lm	140		
>ref-203264	TCTGCATGCACTCCTTCTCCTTCTCCT	23	<nnxnp>	nn	52	np	212		
>ref-203269	CCCTCCCCTACACCCTCTCCTTCCCCA	5	<nnxnp>	nn	260	np	3		
>ref-203269	CCCTCCCCTACACCCTCTCCTTCCCCA	9	<hkxhk>	hh	0	hk	258	kk	2
>ref-203269	CCCTCCCCTACACCCTCTCCTTCCCCA	12	<nnxnp>	nn	230	np	10		
>ref-203269	CCCTCCCCTACACCCTCTCCTTCCCCA	15	<hkxhk>	hh	54	hk	184	kk	0
>ref-203269	CCCTCCCCTACACCCTCTCCTTCCCCA	16	<hkxhk>	hh	0	hk	259	kk	1
>ref-203269	CCCTCCCCTACACCCTCTCCTTCCCCA	21	<hkxhk>	hh	0	hk	185	kk	54
>ref-203283	TGCACCGGAACTGTTTCTCCCCAGCAA	6	<lmxll>	ll	180	lm	81		
>ref-203283	TGCACCGGAACTGTTTCTCCCCAGCAA	13	<nnxnp>	nn	134	np	132		
>ref-203287	AGAGGGATAACTATACCTCCAACAGGT	12	<lmxll>	ll	140	lm	123		
>ref-203298	TGCACACAAACACGAACTCCACAAAAT	21	<lmxll>	ll	141	lm	109		
>ref-203320	TTCCCTCTTACGTCCTCTCCTTTACTC	1	<lmxll>	ll	256	lm	0		
>ref-203320	TTCCCTCTTACGTCCTCTCCTTTACTC	4	<nnxnp>	nn	106	np	131		
>ref-203320	TTCCCTCTTACGTCCTCTCCTTTACTC	23	<lmxll>	ll	115	lm	117		
>ref-203342	CCCATCGTCACCGTCCCTCCCTAGCCT	8	<nnxnp>	nn	119	np	151		
>ref-203352	TATCTGAGAACTGAACCTCCACGTCAT	8	<hkxhk>	hh	52	hk	126	kk	62
>ref-203352	TATCTGAGAACTGAACCTCCACGTCAT	15	<lmxll>	ll	140	lm	106		
>ref-203372	CTCTTTCTCACCTCCGCTCCTCTGTCT	16	<nnxnp>	nn	250	np	0		
>ref-203372	CTCTTTCTCACCTCCGCTCCTCTGTCT	24	<nnxnp>	nn	250	np	0		
>ref-203390	ACCATCACAACCCTCACTCCTGTTGTG	25	<nnxnp>	nn	271	np	0		
>ref-203391	TTCTTTCGCACCTTCCCTCCTTCGTTC	1	<lmxll>	ll	123	lm	142		
>ref-203391	TTCTTTCGCACCTTCCCTCCTTCGTTC	3	<lmxll>	ll	123	lm	142		
>ref-203395	CTACTGCCAACTTGCTCTCCAATTCCC	14	<lmxll>	ll	108	lm	111		
>ref-203431	CTGACCACAACTCTCCCTCCTTTCTTC	23	<lmxll>	ll	128	lm	133		
>ref-203454	ACTCTCAGGACTTCATCTCCTTCATAT	7	<nnxnp>	nn	122	np	131		
>ref-203463	CCCACCCACACTGACCCTCCTCCTCCT	9	<lmxll>	ll	130	lm	127		
>ref-203469	CCTTTCAGCACCCTCCCTCCGTCCGTC	21	<lmxll>	ll	46	lm	200		

>ref-203469	CCTTTCAGCACCCTCCCTCCGTCCGTC	25	<lmxll>	ll	44	lm	200		
>ref-203471	ACACCCTCTACCCCCTCTCCCCCACCT	5	<nnxnp>	nn	13	np	250		
>ref-203471	ACACCCTCTACCCCCTCTCCCCCACCT	16	<nnxnp>	nn	13	np	249		
>ref-203511	GGCCTTATCACCCTCCCTCCGCACTTA	7	<hkxhk>	hh	0	hk	273	kk	0
>ref-203511	GGCCTTATCACCCTCCCTCCGCACTTA	21	<hkxhk>	hh	0	hk	273	kk	0
>ref-203515	AGCAAACAAACAAATCCTCCTCCCCTA	15	<nnxnp>	nn	132	np	123		
>ref-203535	CATATATATACGCTCCCTCCCATGTTA	3	<lmxll>	ll	102	lm	121		
>ref-203540	CTCGTTACCACCCTTCCTCCATTCTAT	7	<lmxll>	ll	109	lm	131		
>ref-203561	ACGACACCGACCGGGCCTCCGTACCTT	27	<nnxnp>	nn	118	np	139		
>ref-203592	TATGAGCCTACATTACCTCCTGGTCTG	16	<lmxll>	ll	100	lm	120		
>ref-203595	CCTTTGCCCACCGGCTCTCCCTCCCCT	1	<nnxnp>	nn	261	np	1		
>ref-203597	GATACGGAGACACCTGCTCCCAAGGTC	2	<lmxll>	ll	23	lm	239		
>ref-203613	TGGATGGGTACACCTGCTCCTAGTCAG	7	<nnxnp>	nn	152	np	118		
>ref-203628	CCCCCACTAACGTATCCTCCACCTCCT	25	<lmxll>	ll	120	lm	145		
>ref-203639	CATCCCTTCACCATCTCTCCCCTCCTC	16	<lmxll>	ll	0	lm	265		
>ref-203639	CATCCCTTCACCATCTCTCCCCTCCTC	25	<lmxll>	ll	0	lm	265		
>ref-203658	TTACTGGCGACTGCAACTCCTTCTAAC	22	<nnxnp>	nn	121	np	146		
>ref-203667	CTTGGACACACTCCTCCTCCCACAGTC	1	<nnxnp>	nn	159	np	107		
>ref-203667	CTTGGACACACTCCTCCTCCCACAGTC	2	<nnxnp>	nn	145	np	119		
>ref-203674	CCATCCACTACAACCACTCCACCGACC	2	<lmxll>	ll	144	lm	116		
>ref-203674	CCATCCACTACAACCACTCCACCGACC	27	<lmxll>	ll	118	lm	123		
>ref-203696	AAAGTTCAGACGTCATCTCCACTCGAG	2	<nnxnp>	nn	140	np	125		
>ref-203696	AAAGTTCAGACGTCATCTCCACTCGAG	8	<nnxnp>	nn	137	np	128		
>ref-203724	CTCCTCCTCACTGGTTCTCCTTTCATC	3	<nnxnp>	nn	118	np	136		
>ref-203730	GTCACAATAACTCACACTCCTGAAGTG	15	<nnxnp>	nn	107	np	128		
>ref-203739	CTCTTTCTAACCCTCCCTCCCCCATTC	14	<lmxll>	ll	145	lm	94		
>ref-203739	CTCTTTCTAACCCTCCCTCCCCCATTC	21	<lmxll>	ll	149	lm	93		
>ref-203758	ATTTGTTTTACATATACTCCTTGGGTA	25	<nnxnp>	nn	99	np	138		
>ref-203774	ATCTCTGGAACGGCGTCTCCTGTGAGT	7	<nnxnp>	nn	122	np	145		
>ref-203781	CTCCCTTACACTCTTACTCCCTTGCTT	23	<nnxnp>	nn	102	np	150		
>ref-203781	CTCCCTTACACTCTTACTCCCTTGCTT	27	<nnxnp>	nn	100	np	150		
>ref-203783	GAATAGTGGACACGCACTCCTCAAAAA	15	<nnxnp>	nn	260	np	0		
>ref-203790	GTTTCCAACACTCCTCCTCCTCTCCAT	26	<lmxll>	ll	103	lm	157		
>ref-203790	GTTTCCAACACTCCTCCTCCTCTCCAT	27	<lmxll>	ll	105	lm	155		
>ref-203806	TATCTCCTTACCTTCCCTCCCGTTGCA	21	<lmxll>	ll	125	lm	125		
>ref-203806	TATCTCCTTACCTTCCCTCCCGTTGCA	25	<lmxll>	ll	125	lm	125		
>ref-203807	CTTTTCATCACTATTTCTCCTCTCTCT	16	<lmxll>	ll	265	lm	0		
>ref-203807	CTTTTCATCACTATTTCTCCTCTCTCT	21	<lmxll>	ll	264	lm	0		

>ref-203811	TACCCATCCACACGCACTCCTTCTCTT	1	<nnxnp>	nn	121	np	144		
>ref-203816	CACAGGGTTACCAACTCTCCCGACGGA	7	<hkxhk>	hh	0	hk	265	kk	6
>ref-203816	CACAGGGTTACCAACTCTCCCGACGGA	16	<hkxhk>	hh	0	hk	265	kk	6
>ref-203822	CCTACCTTTACTTCTTCTCCACGTTTA	6	<hkxhk>	hh	171	hk	93	kk	0
>ref-203822	CCTACCTTTACTTCTTCTCCACGTTTA	22	<hkxhk>	hh	136	hk	127	kk	0
>ref-203822	CCTACCTTTACTTCTTCTCCACGTTTA	24	<hkxhk>	hh	0	hk	272	kk	0
>ref-203853	TCCTCCCTCACTATCTCTCCATGTCCC	24	<lmxll>	ll	259	lm	1		
>ref-203869	ATCTCTCCCACCTTTCCTCCCTCTCCA	1	<nnxnp>	nn	59	np	173		
>ref-203869	ATCTCTCCCACCTTTCCTCCCTCTCCA	23	<nnxnp>	nn	265	np	1		
>ref-203869	ATCTCTCCCACCTTTCCTCCCTCTCCA	27	<nnxnp>	nn	52	np	172		
>ref-203885	TCACACCGAACACTTCCTCCACATGCC	8	<lmxll>	ll	159	lm	98		
>ref-203906	TATATACACACACCACCTCCTCATCAC	9	<lmxll>	ll	121	lm	112		
>ref-203920	ATGCCCTTTACCGATGCTCCCCTGTTT	26	<hkxhk>	hh	52	hk	144	kk	72
>ref-203922	CCCCTCACCACCTGTTCTCCTTTCCTT	22	<lmxll>	ll	116	lm	137		
>ref-203936	CCGCGTTTCACCCCCTCTCCCCTCTCC	12	<lmxll>	ll	117	lm	151		
>ref-203936	CCGCGTTTCACCCCCTCTCCCCTCTCC	21	<nnxnp>	nn	257	np	11		
>ref-203957	TCTTTCTTCACCTTCTCTCCTCTTGTG	25	<lmxll>	ll	17	lm	248		
>ref-203963	CGGCCGTCTACCCTTTCTCCCTCTATC	3	<hkxhk>	hh	58	hk	147	kk	52
>ref-203966	TGCTCCTCGACAGCTCCTCCTCGACAG	2	<hkxhk>	hh	0	hk	268	kk	2
>ref-203966	TGCTCCTCGACAGCTCCTCCTCGACAG	8	<lmxll>	ll	145	lm	112		
>ref-203966	TGCTCCTCGACAGCTCCTCCTCGACAG	9	<hkxhk>	hh	0	hk	265	kk	4
>ref-204004	CCCGACGCTACCGTGTCTCCCCTGGTT	4	<lmxll>	ll	137	lm	122		
>ref-204007	AGTGGCTTTACCCATACTCCCAGTCGT	5	<nnxnp>	nn	271	np	0		
>ref-204031	CTCTTCCTTACTTACCCTCCCTTCCTC	27	<hkxhk>	hh	50	hk	168	kk	27
>ref-204052	GCCTCCACCACTTTCCCTCCTTCCATT	14	<nnxnp>	nn	127	np	122		
>ref-204056	TCCCCCTGCACTACCCCTCCTGCTCTC	26	<nnxnp>	nn	158	np	96		
>ref-204058	TCTTCTGCTACCTTCTCTCCTTCTACT	6	<hkxhk>	hh	0	hk	271	kk	0
>ref-204059	TCATTACTCACTAATTCTCCGTGATGC	9	<lmxll>	ll	124	lm	108		
>ref-204064	ATCATAAAAACAGCCTCTCCAACAACA	1	<nnxnp>	nn	119	np	104		
>ref-204070	CAAATGCATACACCCTCTCCAACACAA	23	<nnxnp>	nn	100	np	120		
>ref-204140	CCAACTATCACAACCACTCCCTCCACC	8	<nnxnp>	nn	109	np	135		
>ref-204142	CAGCGCCCAACATAGCCTCCAATCTGC	25	<nnxnp>	nn	239	np	0		
>ref-204142	CAGCGCCCAACATAGCCTCCAATCTGC	27	<nnxnp>	nn	235	np	0		
>ref-204189	ATCTCCCTTACCCCCCCTCCCATCTCC	13	<lmxll>	ll	253	lm	0		
>ref-204189	ATCTCCCTTACCCCCCCTCCCATCTCC	14	<lmxll>	ll	258	lm	1		
>ref-204189	ATCTCCCTTACCCCCCCTCCCATCTCC	22	<lmxll>	ll	9	lm	239		
>ref-204199	TTTAACCTCACCTCACCTCCTCGCGCT	22	<hkxhk>	hh	0	hk	271	kk	0
>ref-204205	CTACCCCTCACCCTCTCTCCACTTTTC	4	<nnxnp>	nn	248	np	0		

>ref-204256	AGACAAAGAACGAAGCCTCCATCATGA	12	<lmxll>	ll	118	lm	112			
>ref-204261	TCATCCTTCACATCATCTCCGCTCTCG	25	<nnxnp>	nn	123	np	146			
>ref-204271	ATCGTCATTACCACCACTCCCTCCCCT	24	<lmxll>	ll	208	lm	33			
>ref-204271	ATCGTCATTACCACCACTCCCTCCCCT	27	<lmxll>	ll	206	lm	33			
>ref-204325	CTCACGCCCACTGCCCCTCCCCTCCCC	23	<nnxnp>	nn	269	np	3			
>ref-204325	CTCACGCCCACTGCCCCTCCCCTCCCC	26	<nnxnp>	nn	269	np	3			
>ref-204336	AGCACACTCACATTAGCTCCTTTTATC	7	<nnxnp>	nn	119	np	142			
>ref-204382	CCACGCCCTACCAGTGCTCCTTCTGCG	5	<nnxnp>	nn	258	np	0			
>ref-204393	AGAGACTACACTGAAACTCCTACTTAA	4	<nnxnp>	nn	118	np	112			
>ref-204393	AGAGACTACACTGAAACTCCTACTTAA	7	<nnxnp>	nn	118	np	112			
>ref-204412	CCCTCCTCCACTTTATCTCCTCCAGTA	15	<nnxnp>	nn	18	np	248			
>ref-204439	TTTACTTTCACTCTGACTCCGTATCTG	25	<nnxnp>	nn	121	np	111			
>ref-204446	TATCTCCCAACTCCGTCTCCTCCATCT	2	<hkxhk>	hh	57	hk	153		kk	50
>ref-204447	GTGTGTTCCACCTCGCCTCCTCCGTCT	4	<nnxnp>	nn	134	np	134			
>ref-204457	CCCCTGCCCACCCAAGCTCCAATATGT	22	<hkxhk>	hh	28	hk	205		kk	0
>ref-204468	TCCCCCTACACACAGCCTCCCTCCATA	12	<nnxnp>	nn	116	np	132			
>ref-204474	CAATGCCATACGTCTTCTCCATGCCCC	5	<nnxnp>	nn	140	np	129			
>ref-204496	CCCTTCTCTACTCTCACTCCCTTGCAT	25	<nnxnp>	nn	28	np	228			
>ref-204496	CCCTTCTCTACTCTCACTCCCTTGCAT	26	<nnxnp>	nn	29	np	228			
>ref-204519	CCTACCAATACACAACCTCCTGGATTC	27	<lmxll>	ll	138	lm	88			
>ref-204530	ATCATGGCGACTCACGCTCCTTATTAT	1	<hkxhk>	hh	59	hk	141		kk	62
>ref-204530	ATCATGGCGACTCACGCTCCTTATTAT	3	<hkxhk>	hh	60	hk	140		kk	62
>ref-204533	CGCTTTCTCACTCATCCTCCTTATCTC	16	<lmxll>	ll	133	lm	127			
>ref-204544	ACTCCCCTAACTCCCCCTCCCTTCTCT	25	<hkxhk>	hh	0	hk	245		kk	3
>ref-204544	ACTCCCCTAACTCCCCCTCCCTTCTCT	26	<lmxll>	ll	272	lm	1			
>ref-204544	ACTCCCCTAACTCCCCCTCCCTTCTCT	27	<hkxhk>	hh	1	hk	269		kk	0
>ref-204585	ACCAAGCTCACTCGCTCTCCTCCTTTT	24	<nnxnp>	nn	116	np	152			
>ref-204587	TCGCCCTCGACTCTTCCTCCTCGTCCT	26	<nnxnp>	nn	132	np	141			
>ref-204626	CTGGCTTCTACTTCCTCTCCCCTTCCC	2	<nnxnp>	nn	257	np	0			
>ref-204655	TCAACACTGACATCATCTCCATTATCA	7	<lmxll>	ll	124	lm	114			
>ref-204655	TCAACACTGACATCATCTCCATTATCA	26	<nnxnp>	nn	251	np	0			
>ref-204660	CTCTCTATCACCCACACTCCTCTCTTT	5	<lmxll>	ll	124	lm	132			
>ref-204674	TGAATCATCACCTCGTCTCCTTGTCAT	12	<lmxll>	ll	117	lm	136			
>ref-204677	TGCTCAGCCACACAAGCTCCTTATGGT	27	<lmxll>	ll	129	lm	131			
>ref-204723	GAAGCAACCACCTGTGCTCCTCTGCCA	2	<lmxll>	ll	140	lm	131			
>ref-204785	ACGACGATGACACCTACTCCACAAGAT	2	<lmxll>	ll	123	lm	137			
>ref-204805	ACATTATAAACTGCGCCTCCCTCGGAC	12	<nnxnp>	nn	124	np	136			
>ref-204808	CTTCACCTCACCCTCACTCCACCTTCC	5	<nnxnp>	nn	272	np	0			

>ref-204808	CTTCACCTCACCCTCACTCCACCTTCC	16	<nnxnp>	nn	272	np	0		
>ref-204810	ATTTGACATACGGGGCCTCCGCAAGCA	6	<nnxnp>	nn	123	np	135		
>ref-204810	ATTTGACATACGGGGCCTCCGCAAGCA	7	<lmxll>	ll	135	lm	128		
>ref-204826	AAGACAAGCACGGCGTCTCCTTTAGAT	16	<nnxnp>	nn	123	np	134		
>ref-204832	CCCATATCTACGTCACCTCCACCCCCA	22	<nnxnp>	nn	119	np	124		
>ref-204834	CATTTACACACCGACCCTCCAGCAGGA	27	<hkxhk>	hh	58	hk	151	kk	50
>ref-204847	CACACACACACTGAAGCTCCAAATTCC	8	<lmxll>	ll	116	lm	127		
>ref-204861	GCAGGAAGTACCTCGCCTCCTTGGTGT	9	<nnxnp>	nn	263	np	0		
>ref-204867	TCTGAAAGAACGTCTCCTCCATCTTTT	6	<hkxhk>	hh	0	hk	273	kk	0
>ref-204867	TCTGAAAGAACGTCTCCTCCATCTTTT	12	<hkxhk>	hh	0	hk	251	kk	10
>ref-204867	TCTGAAAGAACGTCTCCTCCATCTTTT	14	<hkxhk>	hh	9	hk	255	kk	0
>ref-204870	GCTTGTGTTACAGACGCTCCAATGACC	2	<lmxll>	ll	125	lm	144		
>ref-204934	CCTCTTTCCACCTTGTCTCCCCCTTTT	2	<lmxll>	ll	123	lm	97		
>ref-204934	CCTCTTTCCACCTTGTCTCCCCCTTTT	23	<lmxll>	ll	123	lm	98		
>ref-204937	CTATATCAGACACAGCCTCCCTCTCCC	4	<nnxnp>	nn	130	np	119		
>ref-204945	CGAGAGAGCACGACAACTCCCGGGCGT	15	<lmxll>	ll	13	lm	253		
>ref-204945	CGAGAGAGCACGACAACTCCCGGGCGT	24	<lmxll>	ll	152	lm	117		
>ref-204954	CACACACACACACATTCTCCAAATGAC	24	<lmxll>	ll	122	lm	146		
>ref-204961	AGTAACGCAACGTCCTCTCCCGTCACT	13	<lmxll>	ll	140	lm	128		
>ref-204973	TCTGATGTTACTGCTGCTCCTCCTCCT	16	<lmxll>	ll	227	lm	0		
>ref-204979	TCACACCTCACACTACCTCCTTCACAC	1	<lmxll>	ll	143	lm	114		
>ref-204979	TCACACCTCACACTACCTCCTTCACAC	12	<nnxnp>	nn	114	np	147		
>ref-204984	CTCCCCACCACCTTCTCTCCCCCATCC	14	<lmxll>	ll	124	lm	136		
>ref-205022	TGTTCCCTCACTTCGCCTCCTTTGTTA	16	<nnxnp>	nn	120	np	137		
>ref-205041	GCCTTACTCACTCCATCTCCTTTTTAT	1	<nnxnp>	nn	125	np	127		
>ref-205066	TACACAAACACATATTCTCCAACGCAC	1	<nnxnp>	nn	139	np	129		
>ref-205080	CTTCTTTCTACTTCTTCTCCCCTTCTT	2	<hkxhk>	hh	0	hk	182	kk	72
>ref-205080	CTTCTTTCTACTTCTTCTCCCCTTCTT	9	<lmxll>	ll	115	lm	120		
>ref-205080	CTTCTTTCTACTTCTTCTCCCCTTCTT	21	<nnxnp>	nn	147	np	91		
>ref-205080	CTTCTTTCTACTTCTTCTCCCCTTCTT	22	<hkxhk>	hh	72	hk	182	kk	0
>ref-205080	CTTCTTTCTACTTCTTCTCCCCTTCTT	25	<lmxll>	ll	114	lm	121		
>ref-205120	CTACTTCCTACCTTTGCTCCTTCTCCA	8	<nnxnp>	nn	17	np	251		
>ref-205120	CTACTTCCTACCTTTGCTCCTTCTCCA	13	<nnxnp>	nn	10	np	261		
>ref-205121	GACCTAAGGACCTTGCCTCCTTACACC	24	<lmxll>	ll	126	lm	132		
>ref-205139	GGACCTTTCACTCTGTCTCCTACCGTC	22	<nnxnp>	nn	144	np	121		
>ref-205146	CCCACCCATACACACCCTCCGTCTATA	7	<lmxll>	ll	128	lm	128		
>ref-205153	CATCCTGCAACCACCTCTCCTCCCTCA	16	<nnxnp>	nn	112	np	137		
>ref-205162	CCTTCTCTTACCCTATCTCCATCACTC	2	<hkxhk>	hh	54	hk	133	kk	44

>ref-205173	TCACATTCTACTTTTCCTCCCTGCTGT	27	<nnxnp>	nn	111	np	124		
>ref-205176	CAACCTCCCACTCGGCCTCCCACTCGT	21	<nnxnp>	nn	271	np	0		
>ref-205176	CAACCTCCCACTCGGCCTCCCACTCGT	25	<lmxll>	ll	265	lm	2		
>ref-205202	CACATGTACACATTGCCTCCGTCTTTT	1	<lmxll>	ll	117	lm	155		
>ref-205224	AGAAACTAAACAAATTCTCCCTTGCCG	7	<nnxnp>	nn	106	np	122		
>ref-205230	CCTACATAAACCTTTCCTCCCGCTGTT	21	<nnxnp>	nn	128	np	119		
>ref-205241	CTGACCATCACGAGCTCTCCGACCTCG	24	<hkxhk>	hh	101	hk	164	kk	0
>ref-205241	CTGACCATCACGAGCTCTCCGACCTCG	27	<nnxnp>	nn	1	np	271		
>ref-205249	TAATCCCCCACCCTCTCTCCTACCCCC	2	<lmxll>	ll	24	lm	216		
>ref-205249	TAATCCCCCACCCTCTCTCCTACCCCC	26	<lmxll>	ll	27	lm	215		
>ref-205265	GTCCGCTAGACACACTCTCCAATTCTC	12	<hkxhk>	hh	64	hk	201	kk	0
>ref-205271	ATGTCTCTCACCTCCTCTCCTTTCTCC	5	<nnxnp>	nn	263	np	0		
>ref-205281	CCCCCATACACTTTCTCTCCTGCAGTC	7	<lmxll>	ll	127	lm	108		
>ref-205281	CCCCCATACACTTTCTCTCCTGCAGTC	13	<lmxll>	ll	128	lm	108		
>ref-205290	CCCCTCTATACCCCTCCTCCATCCCCC	3	<lmxll>	ll	127	lm	119		
>ref-205290	CCCCTCTATACCCCTCCTCCATCCCCC	23	<hkxhk>	hh	71	hk	178	kk	0
>ref-205290	CCCCTCTATACCCCTCCTCCATCCCCC	25	<hkxhk>	hh	0	hk	213	kk	39
>ref-205305	ACTCTCTCTACCCCCTCTCCCTCAGAA	1	<nnxnp>	nn	247	np	6		
>ref-205305	ACTCTCTCTACCCCCTCTCCCTCAGAA	27	<nnxnp>	nn	257	np	2		
>ref-205327	TCCCCTCTCACTCCCCCTCCATTCACT	3	<nnxnp>	nn	260	np	1		
>ref-205327	TCCCCTCTCACTCCCCCTCCATTCACT	6	<lmxll>	ll	124	lm	124		
>ref-205332	TGTTTATCTACCTATCCTCCTATGTAT	21	<hkxhk>	hh	54	hk	128	kk	47
>ref-205343	CTCACATCCACACTAACTCCCACTCCC	27	<lmxll>	ll	128	lm	127		
>ref-205354	CGAAAAAAGACCAAACCTCCTACTCCA	5	<hkxhk>	hh	31	hk	124	kk	81
>ref-205355	CTCAGACTGACCCCACCTCCACCCCAC	6	<hkxhk>	hh	51	hk	161	kk	55
>ref-205359	CCGCTACCTACTTGCGCTCCCCTCCCC	3	<lmxll>	ll	10	lm	259		
>ref-205388	CCACATTTCACCCATCCTCCTTCCCCA	5	<nnxnp>	nn	144	np	114		
>ref-205388	CCACATTTCACCCATCCTCCTTCCCCA	6	<nnxnp>	nn	144	np	114		
>ref-205402	GTGACACCGACGACCTCTCCCGAGGAC	6	<lmxll>	ll	125	lm	108		
>ref-205422	TGCTTACGTACTTCACCTCCTCTTCTT	21	<lmxll>	ll	121	lm	144		
>ref-205435	CGTGGTGTCACTCACCCTCCCCGAGTG	23	<lmxll>	ll	132	lm	129		
>ref-205439	TACTTGTTCACTCATCCTCCTTCAGCT	13	<hkxhk>	hh	37	hk	153	kk	73
>ref-205464	CCTGCATATACTCTATCTCCATCCCTT	13	<nnxnp>	nn	122	np	106		
>ref-205476	CTACCTCTCACTTTCTCTCCCTGCTAA	2	<nnxnp>	nn	120	np	137		
>ref-205496	CTTCTAGACACAGTGTCTCCGTTGAAA	7	<nnxnp>	nn	146	np	107		
>ref-205497	GCTTCCCGCACAACGCCTCCGAGTACT	8	<lmxll>	ll	5	lm	267		
>ref-205497	GCTTCCCGCACAACGCCTCCGAGTACT	23	<lmxll>	ll	4	lm	268		
>ref-205503	GGAAGCGAGACCCTTCCTCCTCTGGCA	5	<hkxhk>	hh	0	hk	272	kk	0

>ref-205515	TGGTGAAAGACACACTCTCCTCCCATC	4	<nnxnp>	nn	16	np	251		
>ref-205515	TGGTGAAAGACACACTCTCCTCCCATC	7	<hkxhk>	hh	0	hk	272	kk	0
>ref-205515	TGGTGAAAGACACACTCTCCTCCCATC	12	<nnxnp>	nn	16	np	251		
>ref-205515	TGGTGAAAGACACACTCTCCTCCCATC	15	<hkxhk>	hh	0	hk	272	kk	0
>ref-205521	TCATCTTCCACTTCCTCTCCTCACTTC	3	<lmxll>	ll	121	lm	146		
>ref-205521	TCATCTTCCACTTCCTCTCCTCACTTC	4	<lmxll>	ll	121	lm	146		
>ref-205544	ACCCAGCATACTGTCGCTCCGTCACCC	5	<nnxnp>	nn	272	np	0		
>ref-205554	CCCACACATACATATCCTCCTCTATCA	5	<nnxnp>	nn	136	np	122		
>ref-205557	ATAAATACTACGTGACCTCCGAGGCAC	25	<hkxhk>	hh	124	hk	132	kk	0
>ref-205601	TTCCCTTTCACCCATCCTCCCACACCC	6	<lmxll>	ll	3	lm	223		
>ref-205601	TTCCCTTTCACCCATCCTCCCACACCC	24	<lmxll>	ll	3	lm	222		
>ref-205609	AGATTGTAGACCCTTCCTCCTCTAGCT	4	<lmxll>	ll	115	lm	137		
>ref-205631	ACTGCACAAACCTTAACTCCCACAAAA	16	<nnxnp>	nn	126	np	123		
>ref-205633	AACTTTGCCACATTCCCTCCACTGATG	4	<lmxll>	ll	115	lm	135		
>ref-205653	CCGACACAGACCTGCCCTCCTTGACTC	5	<hkxhk>	hh	0	hk	273	kk	0
>ref-205669	ATTAAATCCACACAGACTCCCTCGTCC	25	<nnxnp>	nn	136	np	129		
>ref-205672	AACCTCTTCACCCTCCCTCCACCCCCT	6	<lmxll>	ll	266	lm	0		
>ref-205683	CCGGTACTTACCCCTCCTCCTCACTCG	2	<hkxhk>	hh	16	hk	217	kk	1
>ref-205683	CCGGTACTTACCCCTCCTCCTCACTCG	5	<hkxhk>	hh	1	hk	217	kk	16
>ref-205692	GTGTTTACGACTGTGACTCCGTAATTT	27	<hkxhk>	hh	52	hk	123	kk	45
>ref-205697	GTGAATTCTACAAGTCCTCCAACTTTT	3	<lmxll>	ll	135	lm	122		
>ref-205697	GTGAATTCTACAAGTCCTCCAACTTTT	9	<lmxll>	ll	187	lm	55		
>ref-205702	CCGTCGCTAACTCCTCCTCCTCCTGCT	3	<nnxnp>	nn	259	np	0		
>ref-205702	CCGTCGCTAACTCCTCCTCCTCCTGCT	6	<nnxnp>	nn	260	np	0		
>ref-205702	CCGTCGCTAACTCCTCCTCCTCCTGCT	25	<nnxnp>	nn	121	np	124		
>ref-205702	CCGTCGCTAACTCCTCCTCCTCCTGCT	27	<nnxnp>	nn	127	np	121		
>ref-205725	TGCCTCCTTACCCCTCCTCCTCTTCCC	2	<lmxll>	ll	3	lm	254		
>ref-205725	TGCCTCCTTACCCCTCCTCCTCTTCCC	12	<lmxll>	ll	131	lm	128		
>ref-205802	CCTCTCAGAACCCCGCCTCCCTTCCTT	25	<lmxll>	ll	120	lm	147		
>ref-205822	TACCCTTCCACTCCACCTCCCCTTCTC	7	<lmxll>	ll	146	lm	104		
>ref-205822	TACCCTTCCACTCCACCTCCCCTTCTC	8	<lmxll>	ll	148	lm	104		
>ref-205822	TACCCTTCCACTCCACCTCCCCTTCTC	25	<nnxnp>	nn	130	np	111		
>ref-205824	TTATTCCTCACGTCCTCTCCTTCCCAT	5	<nnxnp>	nn	137	np	132		
>ref-205824	TTATTCCTCACGTCCTCTCCTTCCCAT	12	<lmxll>	ll	132	lm	135		
>ref-205848	TCCGGCACGACCTCCTCTCCCCCGCTA	6	<lmxll>	ll	120	lm	142		
>ref-205880	CCCTCCCTCACGCCCCCTCCATCTGCT	4	<nnxnp>	nn	110	np	141		
>ref-205881	CTCACCCCCACCTTCCCTCCTCCCTCA	15	<lmxll>	ll	159	lm	89		
>ref-205881	CTCACCCCCACCTTCCCTCCTCCCTCA	24	<nnxnp>	nn	41	np	205		

>ref-205886	TCTATCACCACCATCCCTCCCCATCTC	1	<nnxnp>	nn	104	np	146		
>ref-205894	CCTTTATCAACTGCCCCTCCTTCAGAA	8	<hkxhk>	hh	2	hk	266	kk	0
>ref-205894	CCTTTATCAACTGCCCCTCCTTCAGAA	16	<lmxll>	ll	95	lm	148		
>ref-205906	GGACCCGACACCCTTCCTCCTTCAGTC	15	<nnxnp>	nn	135	np	126		
>ref-205907	CCCTTCCCCACCCTTCCTCCCTCTGTA	13	<hkxhk>	hh	2	hk	265	kk	0
>ref-205907	CCCTTCCCCACCCTTCCTCCCTCTGTA	25	<hkxhk>	hh	2	hk	265	kk	0
>ref-205908	TCGACTATAACGCTCCCTCCTTCCGAA	15	<lmxll>	ll	119	lm	143		
>ref-205920	TCCTTGGCAACAGCTCCTCCTGGCTGT	27	<hkxhk>	hh	37	hk	115	kk	71
>ref-205942	CTCTCACCCACTCTCTCTCCTCGATTC	9	<lmxll>	ll	111	lm	150		
>ref-205942	CTCTCACCCACTCTCTCTCCTCGATTC	16	<nnxnp>	nn	186	np	73		
>ref-205942	CTCTCACCCACTCTCTCTCCTCGATTC	23	<nnxnp>	nn	177	np	74		
>ref-205943	ACTGATACCACATGGCCTCCCTCCCTG	2	<nnxnp>	nn	141	np	129		
>ref-205943	ACTGATACCACATGGCCTCCCTCCCTG	8	<nnxnp>	nn	140	np	129		
>ref-205943	ACTGATACCACATGGCCTCCCTCCCTG	9	<lmxll>	ll	139	lm	133		
>ref-205953	ATTACGTACACTAGCTCTCCTCAGTTA	8	<nnxnp>	nn	113	np	126		
>ref-205953	ATTACGTACACTAGCTCTCCTCAGTTA	14	<nnxnp>	nn	131	np	116		
>ref-205976	CCATCTCTTACTCTGCCTCCTTCCTCC	26	<hkxhk>	hh	50	hk	217	kk	0
>ref-205976	CCATCTCTTACTCTGCCTCCTTCCTCC	27	<lmxll>	ll	130	lm	132		
>ref-205985	ATGGTACATACTATGCCTCCTTATGTA	27	<lmxll>	ll	129	lm	135		
>ref-205988	AATCATGTCACCCACTCTCCCGTACAC	3	<hkxhk>	hh	67	hk	124	kk	40
>ref-205988	AATCATGTCACCCACTCTCCCGTACAC	24	<hkxhk>	hh	38	hk	125	kk	69
>ref-206033	AAGCCTTCGACCAAGGCTCCATCTTCT	13	<lmxll>	ll	118	lm	144		
>ref-206035	ATCAGGAAGACACCTCCTCCTGCTCCT	6	<nnxnp>	nn	119	np	153		
>ref-206055	AGCCCTAGGACGACACCTCCTTTGTGG	6	<hkxhk>	hh	0	hk	272	kk	0
>ref-206055	AGCCCTAGGACGACACCTCCTTTGTGG	8	<hkxhk>	hh	0	hk	272	kk	0
>ref-206081	ATCCCACTCACTCACCCTCCGCATTAC	2	<nnxnp>	nn	3	np	266		
>ref-206096	AATACTGCAACATACGCTCCACATGGT	22	<lmxll>	ll	126	lm	144		
>ref-206108	CTCCAACCTACCACCACTCCCTCCTTT	14	<lmxll>	ll	127	lm	135		
>ref-206139	ACTGTGCGAACACGTCCTCCATTGTCT	4	<hkxhk>	hh	0	hk	269	kk	1
>ref-206146	TCGAGGTGGACGTGCGCTCCAGGAAAT	27	<nnxnp>	nn	113	np	148		
>ref-206152	TAAAGCGATACCTGAGCTCCAGTCCCT	1	<nnxnp>	nn	119	np	150		
>ref-206182	TTTTATTGTACAGTGCCTCCCTGGTGT	13	<nnxnp>	nn	124	np	115		
>ref-206189	TCTATCTCGACCAGCTCTCCGTGGGCG	27	<nnxnp>	nn	270	np	0		
>ref-206192	TTTTCTTTCACTTACACTCCGTTCCTT	7	<lmxll>	ll	123	lm	127		
>ref-206200	GATATTCACACTGGACCTCCTTTGTGT	4	<nnxnp>	nn	103	np	131		
>ref-206213	ATGAATCTAACACTCGCTCCACTCCTT	25	<lmxll>	ll	132	lm	127		
>ref-206261	TCCTCCTCCACTGAGACTCCGCCCCCA	13	<hkxhk>	hh	70	hk	134	kk	48
>ref-206261	TCCTCCTCCACTGAGACTCCGCCCCCA	24	<lmxll>	ll	115	lm	145		

>ref-206264	TCTCTTCATACACTCTCTCCCTCCCTC	4	<lmxll>	ll	8	lm	262		
>ref-206264	TCTCTTCATACACTCTCTCCCTCCCTC	8	<lmxll>	ll	8	lm	260		
>ref-206264	TCTCTTCATACACTCTCTCCCTCCCTC	21	<lmxll>	ll	28	lm	237		
>ref-206264	TCTCTTCATACACTCTCTCCCTCCCTC	24	<lmxll>	ll	26	lm	239		
>ref-206280	CACACATGTACATGCACTCCCATACAC	3	<hkxhk>	hh	0	hk	273	kk	0
>ref-206280	CACACATGTACATGCACTCCCATACAC	8	<hkxhk>	hh	0	hk	271	kk	1
>ref-206280	CACACATGTACATGCACTCCCATACAC	9	<hkxhk>	hh	0	hk	273	kk	0
>ref-206289	TATTCCCTCACTGTGCCTCCGCCTTTG	5	<hkxhk>	hh	70	hk	145	kk	46
>ref-206289	TATTCCCTCACTGTGCCTCCGCCTTTG	13	<hkxhk>	hh	46	hk	145	kk	70
>ref-206294	CCCCTCTCCACTATTCCTCCCTCCTCA	26	<lmxll>	ll	110	lm	117		
>ref-206325	AGGGTTCACACACCCCCTCCCCTCCTC	24	<lmxll>	ll	138	lm	130		
>ref-206341	GTCCTTCCGACTCCGTCTCCACACCCC	5	<nnxnp>	nn	172	np	90		
>ref-206341	GTCCTTCCGACTCCGTCTCCACACCCC	9	<hkxhk>	hh	0	hk	273	kk	0
>ref-206341	GTCCTTCCGACTCCGTCTCCACACCCC	16	<hkxhk>	hh	0	hk	255	kk	14
>ref-206341	GTCCTTCCGACTCCGTCTCCACACCCC	26	<hkxhk>	hh	0	hk	255	kk	14
>ref-206341	GTCCTTCCGACTCCGTCTCCACACCCC	27	<hkxhk>	hh	84	hk	183	kk	0
>ref-206355	CACCCTTCCACCTTCGCTCCTCACTCT	2	<hkxhk>	hh	63	hk	141	kk	58
>ref-206394	CCGTCGCCAACGTCTTCTCCTTCGCCA	5	<lmxll>	ll	126	lm	137		
>ref-206399	TTTCCCTCCACTTGCCCTCCACCTTAT	5	<hkxhk>	hh	62	hk	142	kk	49
>ref-206428	TACATTTACACCCCTACTCCGTAAACC	21	<lmxll>	ll	108	lm	112		
>ref-206457	CCCTTCCCCACCTTCTCTCCTCCTGTC	22	<hkxhk>	hh	1	hk	270	kk	0
>ref-206457	CCCTTCCCCACCTTCTCTCCTCCTGTC	25	<hkxhk>	hh	1	hk	270	kk	0
>ref-206478	CCTCATCACACTCTTTCTCCTTGTCTC	5	<nnxnp>	nn	255	np	0		
>ref-206478	CCTCATCACACTCTTTCTCCTTGTCTC	8	<nnxnp>	nn	255	np	0		
>ref-206491	ACACATTGCACTGATTCTCCCTTCAAC	4	<lmxll>	ll	115	lm	124		
>ref-206492	GTCTCTCCCACTCCTTCTCCCCTCCCC	3	<lmxll>	ll	240	lm	0		
>ref-206505	CACCCACCAACATCTTCTCCCCGCCCA	9	<lmxll>	ll	136	lm	130		
>ref-206528	TCATTAATCACTGATGCTCCGCCGTCA	25	<lmxll>	ll	141	lm	120		
>ref-206529	TTGAAAGGTACCAATTCTCCATCGGTT	3	<lmxll>	ll	145	lm	112		
>ref-206529	TTGAAAGGTACCAATTCTCCATCGGTT	6	<hkxhk>	hh	179	hk	80	kk	0
>ref-206548	AATCACTAAACTGCACCTCCTTGTAAA	12	<nnxnp>	nn	107	np	145		
>ref-206555	CTGCTGCTGACGTCTCCTCCCTCCCTT	8	<lmxll>	ll	137	lm	132		
>ref-206561	ATTCCACTCACATTTCCTCCCTTCCCT	6	<hkxhk>	hh	39	hk	133	kk	54
>ref-206561	ATTCCACTCACATTTCCTCCCTTCCCT	7	<hkxhk>	hh	54	hk	131	kk	39
>ref-206561	ATTCCACTCACATTTCCTCCCTTCCCT	24	<nnxnp>	nn	118	np	107		
>ref-206584	ATTTTGAAAACCGTCCCTCCGCGCGTT	25	<lmxll>	ll	117	lm	117		
>ref-206613	CTCTCTTACACCCCCCCTCCCCTCTCC	5	<nnxnp>	nn	162	np	91		
>ref-206613	CTCTCTTACACCCCCCCTCCCCTCTCC	6	<nnxnp>	nn	162	np	91		

>ref-206615	TCTCTAGCCACGTCCCCTCCTCATCCC	24	<lmxll>	ll	125	lm	140		
>ref-206635	CAACCATCTACCCACTCTCCCGCACAC	23	<hkxhk>	hh	84	hk	176	kk	0
>ref-206639	CTGCAGGTTACCCACACTCCCGCTGTT	3	<nnxnp>	nn	117	np	145		
>ref-206683	AAAACAACAACGCTTTCTCCCTTGACT	12	<nnxnp>	nn	118	np	102		
>ref-206683	AAAACAACAACGCTTTCTCCCTTGACT	25	<nnxnp>	nn	125	np	100		
>ref-206696	GGAATGTGTACAGTAGCTCCCTCGCCA	16	<nnxnp>	nn	141	np	127		
>ref-206698	TTCTTTTCTACATTCACTCCTCCTTTG	6	<nnxnp>	nn	132	np	121		
>ref-206709	CCTACTTCCACTTTTTCTCCTTCTCTC	8	<nnxnp>	nn	136	np	124		
>ref-206711	CCACATCCTACCCTTCCTCCTACGTTT	16	<nnxnp>	nn	96	np	168		
>ref-206759	TCAAAGTTCACTTATCCTCCGTGCTAA	9	<nnxnp>	nn	122	np	127		
>ref-206759	TCAAAGTTCACTTATCCTCCGTGCTAA	16	<hkxhk>	hh	58	hk	127	kk	60
>ref-206791	CATCCCTCCACCTCCTCTCCACTAATT	23	<lmxll>	ll	115	lm	144		
>ref-206808	TATACAACCACAGACACTCCGATCGCT	14	<hkxhk>	hh	0	hk	273	kk	0
>ref-206812	ATACACATAACAATATCTCCATACATA	2	<nnxnp>	nn	125	np	134		
>ref-206830	CCAACGCGGACAACATCTCCTATGGGT	6	<lmxll>	ll	145	lm	124		
>ref-206832	TCGTTCACCACTCATGCTCCTCAGCAT	5	<nnxnp>	nn	109	np	160		
>ref-206838	CATATATACACAACTGCTCCTCAGTGG	22	<nnxnp>	nn	241	np	23		
>ref-206845	AGATCCACAACAATTCCTCCACCGTTT	22	<nnxnp>	nn	101	np	130		
>ref-206856	TTTTATAGTACCTACTCTCCCTCTTCT	4	<lmxll>	ll	118	lm	114		
>ref-206856	TTTTATAGTACCTACTCTCCCTCTTCT	12	<lmxll>	ll	104	lm	125		
>ref-206856	TTTTATAGTACCTACTCTCCCTCTTCT	25	<lmxll>	ll	120	lm	114		
>ref-206891	TCTCTTTTCACTAGACCTCCAACCACT	16	<hkxhk>	hh	62	hk	128	kk	52
>ref-206912	TACACATATACATTCTCTCCCCCCTCT	16	<hkxhk>	hh	0	hk	272	kk	0
>ref-206912	TACACATATACATTCTCTCCCCCCTCT	26	<hkxhk>	hh	79	hk	184	kk	0
>ref-206916	TTTTCCTCGACCCTCACTCCTCGTCAG	9	<lmxll>	ll	256	lm	1		
>ref-206921	CTCTCCACCACCTTTCCTCCCCCTCCC	2	<lmxll>	ll	132	lm	106		
>ref-206921	CTCTCCACCACCTTTCCTCCCCCTCCC	3	<nnxnp>	nn	165	np	66		
>ref-206921	CTCTCCACCACCTTTCCTCCCCCTCCC	6	<lmxll>	ll	192	lm	37		
>ref-206921	CTCTCCACCACCTTTCCTCCCCCTCCC	21	<lmxll>	ll	139	lm	100		
>ref-206921	CTCTCCACCACCTTTCCTCCCCCTCCC	23	<lmxll>	ll	249	lm	5		
>ref-206925	TTTTCACTGACAAAAACTCCCTGCGTT	25	<lmxll>	ll	131	lm	100		
>ref-206926	CCCCTTCTTACCCTTCCTCCTCTTCTA	3	<nnxnp>	nn	144	np	121		
>ref-206926	CCCCTTCTTACCCTTCCTCCTCTTCTA	12	<lmxll>	ll	34	lm	223		
>ref-206926	CCCCTTCTTACCCTTCCTCCTCTTCTA	16	<lmxll>	ll	36	lm	219		
>ref-206932	CTCTATCGTACCTATCCTCCTCCCCTC	22	<nnxnp>	nn	115	np	144		
>ref-206933	TTCTTCCCCACCTACCCTCCTATCCTC	14	<hkxhk>	hh	10	hk	189	kk	40
>ref-206933	TTCTTCCCCACCTACCCTCCTATCCTC	27	<hkxhk>	hh	10	hk	193	kk	36
>ref-206953	GATTACTTGACCTTGGCTCCCCTCCTG	6	<lmxll>	ll	21	lm	240		

>ref-206953	GATTACTTGACCTTGGCTCCCCTCCTG	22	<hkxhk>	hh	4	hk	267	kk	0
>ref-206957	TCCCTACTTACTTTACCTCCTTCCTTC	27	<lmxll>	ll	130	lm	114		
>ref-206959	TCTTCCCTCACCTCATCTCCTTCTTCT	2	<lmxll>	ll	261	lm	0		
>ref-206959	TCTTCCCTCACCTCATCTCCTTCTTCT	6	<nnxnp>	nn	261	np	0		
>ref-206959	TCTTCCCTCACCTCATCTCCTTCTTCT	7	<nnxnp>	nn	255	np	2		
>ref-206967	AGACTCCTCACTCCTCCTCCTGCCCCA	26	<lmxll>	ll	135	lm	134		
>ref-206974	TCTTCTGGTACTGAACCTCCATAGAAT	21	<hkxhk>	hh	6	hk	262	kk	0
>ref-207002	CGAACGACAACACCCGCTCCGGCTTGC	8	<hkxhk>	hh	73	hk	142	kk	39
>ref-207028	CTGTTTATGACTCTTACTCCAGATAAC	9	<nnxnp>	nn	238	np	0		
>ref-207038	CTCATCCCTACTTCCCCTCCTTTCCTC	22	<hkxhk>	hh	0	hk	184	kk	68
>ref-207038	CTCATCCCTACTTCCCCTCCTTTCCTC	23	<hkxhk>	hh	0	hk	184	kk	62
>ref-207041	TTCAAATGAACGCCGTCTCCCAGTCAC	12	<nnxnp>	nn	113	np	138		
>ref-207044	GCAATTGCTACTGAAGCTCCGCTGCCT	26	<nnxnp>	nn	128	np	125		
>ref-207056	ATAACCTTTACGGATGCTCCTGACCAA	15	<lmxll>	ll	115	lm	122		
>ref-207060	GTCGCTCTCACTTCCCCTCCCCTCTAA	21	<nnxnp>	nn	127	np	130		
>ref-207072	TTCCTCCATACTTCCCCTCCCCCCATC	6	<hkxhk>	hh	0	hk	258	kk	7
>ref-207072	TTCCTCCATACTTCCCCTCCCCCCATC	8	<hkxhk>	hh	0	hk	258	kk	7
>ref-207077	TCTCCCTTTACTCGTTCTCCTTTTGCC	5	<nnxnp>	nn	141	np	108		
>ref-207091	GGAGAGTCAACTCTGGCTCCTTCTCTG	27	<nnxnp>	nn	99	np	136		
>ref-207094	GAGTAAGGCACTTCAGCTCCCAGGCAC	21	<lmxll>	ll	137	lm	121		
>ref-207105	TAAAAGCAGACGCTGCCTCCGAGCGCG	25	<nnxnp>	nn	257	np	1		
>ref-207105	TAAAAGCAGACGCTGCCTCCGAGCGCG	27	<nnxnp>	nn	255	np	1		
>ref-207173	TCCGTATCTACCTCCCCTCCCCCTCTC	2	<hkxhk>	hh	45	hk	152	kk	55
>ref-207173	TCCGTATCTACCTCCCCTCCCCCTCTC	15	<hkxhk>	hh	46	hk	153	kk	55
>ref-207175	TCCACTTCCACCTCTTCTCCTTCTTCA	5	<lmxll>	ll	267	lm	0		
>ref-207188	TTGCATCTAACATTATCTCCATCTCTC	14	<hkxhk>	hh	68	hk	132	kk	57
>ref-207198	GTGCCTCACACTCGCCCTCCGACGGAC	7	<lmxll>	ll	84	lm	151		
>ref-207198	GTGCCTCACACTCGCCCTCCGACGGAC	26	<lmxll>	ll	99	lm	136		
>ref-207218	TCTGGTGCTACGCTTTCTCCATCTTTA	22	<nnxnp>	nn	124	np	129		
>ref-207220	ATAACCCCCACCCTTTCTCCTCCTCCT	8	<lmxll>	ll	140	lm	126		
>ref-207220	ATAACCCCCACCCTTTCTCCTCCTCCT	14	<hkxhk>	hh	7	hk	257	kk	1
>ref-207220	ATAACCCCCACCCTTTCTCCTCCTCCT	15	<hkxhk>	hh	0	hk	267	kk	3
>ref-207220	ATAACCCCCACCCTTTCTCCTCCTCCT	26	<lmxll>	ll	156	lm	101		
>ref-207231	CACACACACACTGACACTCCTTTCTTC	13	<lmxll>	ll	9	lm	259		
>ref-207231	CACACACACACTGACACTCCTTTCTTC	15	<lmxll>	ll	9	lm	259		
>ref-207241	CACTCCTTGACGCCCCCTCCCCTCCCC	4	<nnxnp>	nn	107	np	139		
>ref-207262	TTCCCTGCAACACTCCCTCCCTCACTT	7	<lmxll>	ll	1	lm	271		
>ref-207262	TTCCCTGCAACACTCCCTCCCTCACTT	24	<lmxll>	ll	1	lm	271		

>ref-207264	CAGTGCATCACCCTGACTCCAAGTCCT	4	<hkxhk>	hh	0	hk	271	kk	0
>ref-207264	CAGTGCATCACCCTGACTCCAAGTCCT	7	<hkxhk>	hh	1	hk	270	kk	0
>ref-207264	CAGTGCATCACCCTGACTCCAAGTCCT	13	<hkxhk>	hh	0	hk	271	kk	0
>ref-207264	CAGTGCATCACCCTGACTCCAAGTCCT	15	<hkxhk>	hh	125	hk	132	kk	0
>ref-207264	CAGTGCATCACCCTGACTCCAAGTCCT	22	<hkxhk>	hh	1	hk	270	kk	0
>ref-207326	CAGATTCAAACTTGGCCTCCCTTTATG	6	<lmxll>	ll	6	lm	266		
>ref-207326	CAGATTCAAACTTGGCCTCCCTTTATG	13	<hkxhk>	hh	0	hk	265	kk	6
>ref-207326	CAGATTCAAACTTGGCCTCCCTTTATG	23	<hkxhk>	hh	0	hk	265	kk	6
>ref-207326	CAGATTCAAACTTGGCCTCCCTTTATG	26	<hkxhk>	hh	0	hk	273	kk	0
>ref-207326	CAGATTCAAACTTGGCCTCCCTTTATG	27	<hkxhk>	hh	0	hk	254	kk	13
>ref-207341	CTTCCTCTCACTGCCTCTCCTCCTTTC	2	<hkxhk>	hh	0	hk	273	kk	0
>ref-207341	CTTCCTCTCACTGCCTCTCCTCCTTTC	4	<hkxhk>	hh	0	hk	224	kk	39
>ref-207341	CTTCCTCTCACTGCCTCTCCTCCTTTC	25	<hkxhk>	hh	0	hk	272	kk	0
>ref-207376	CCCAAGCACACTTCACCTCCCTCCCTT	12	<nnxnp>	nn	108	np	112		
>ref-207400	GATCTACCAACAACTACTCCCTTGGGG	3	<hkxhk>	hh	1	hk	233	kk	14
>ref-207400	GATCTACCAACAACTACTCCCTTGGGG	27	<hkxhk>	hh	1	hk	233	kk	15
>ref-207406	TCTCTCCTTACCCACTCTCCCCATCTT	3	<hkxhk>	hh	49	hk	153	kk	59
>ref-207407	CTCCAGTCCACTCTTGCTCCTCCAGCG	3	<hkxhk>	hh	65	hk	142	kk	54
>ref-207414	CAAGAGTGAACTGGGACTCCAAGCTGA	3	<lmxll>	ll	1	lm	260		
>ref-207429	CCTCTCTGCACATACCCTCCTTCTTTC	22	<nnxnp>	nn	148	np	121		
>ref-207442	AATTTAAGTACCTCCTCTCCACGACCT	8	<nnxnp>	nn	117	np	106		
>ref-207447	AGCACTATTACAAGTCCTCCCTTACCA	6	<nnxnp>	nn	140	np	126		
>ref-207471	ACTACACCTACCCCCTCTCCCCTCTCC	13	<nnxnp>	nn	140	np	120		
>ref-207490	GTCACGAGTACAGGTCCTCCACGATCT	21	<hkxhk>	hh	70	hk	146	kk	46
>ref-207498	CCTTCTTCCACCTTTTCTCCTTGGTTA	6	<nnxnp>	nn	111	np	142		
>ref-207510	CCTATCCTCACCTTCCCTCCCTGCCAG	4	<lmxll>	ll	119	lm	108		
>ref-207510	CCTATCCTCACCTTCCCTCCCTGCCAG	25	<lmxll>	ll	119	lm	107		
>ref-207538	CCCTCCTCCACCATAACTCCGTCCTAT	13	<lmxll>	ll	132	lm	130		
>ref-207548	TCTTCTCTCACTTCTTCTCCTCTCGAT	12	<hkxhk>	hh	0	hk	271	kk	2
>ref-207548	TCTTCTCTCACTTCTTCTCCTCTCGAT	26	<hkxhk>	hh	2	hk	271	kk	0
>ref-207564	TTCCTTCTTACCTCTTCTCCTCCCCCC	5	<lmxll>	ll	5	lm	238		
>ref-207564	TTCCTTCTTACCTCTTCTCCTCCCCCC	12	<lmxll>	ll	5	lm	238		
>ref-207577	CCCATCAGCACCCCCTCTCCCATACCC	3	<hkxhk>	hh	0	hk	74	kk	183
>ref-207577	CCCATCAGCACCCCCTCTCCCATACCC	21	<lmxll>	ll	51	lm	202		
>ref-207579	CCCTCATCCACCCCCGCTCCTCGACCC	2	<nnxnp>	nn	124	np	121		
>ref-207630	CACACACACACCAACGCTCCTGAACAA	9	<lmxll>	ll	146	lm	124		
>ref-207640	CTGCGTTTAACTTTTCCTCCAAACCCC	1	<hkxhk>	hh	47	hk	138	kk	48
>ref-207640	CTGCGTTTAACTTTTCCTCCAAACCCC	21	<lmxll>	ll	132	lm	113		

>ref-207662	CCCTATTGCACGCCCACTCCCTCTTCC	8	<hkxhk>	hh	75	hk	136	kk	45
>ref-207664	CAAGCAAACACCATGGCTCCCTGTAAT	26	<lmxll>	ll	132	lm	129		
>ref-207665	CGACGTCTTACTCTTCCTCCTCTTTTC	8	<nnxnp>	nn	110	np	122		
>ref-207666	AAAACTGTAACTCGCTCTCCAAAACCG	1	<nnxnp>	nn	114	np	135		
>ref-207666	AAAACTGTAACTCGCTCTCCAAAACCG	2	<nnxnp>	nn	120	np	118		
>ref-207682	CGTGTATATACAGAAGCTCCTCAGTCT	1	<lmxll>	ll	118	lm	102		
>ref-207684	CCTCTCTCTACCTCCTCTCCTTCAGTA	27	<hkxhk>	hh	51	hk	135	kk	61
>ref-207711	CTCCTCTTCACCCTTTCTCCCTTCCAT	24	<nnxnp>	nn	162	np	84		
>ref-207711	CTCCTCTTCACCCTTTCTCCCTTCCAT	26	<nnxnp>	nn	140	np	114		
>ref-207748	GTCATTCTTACCACTCCTCCTTCCTTC	3	<nnxnp>	nn	118	np	133		
>ref-207748	GTCATTCTTACCACTCCTCCTTCCTTC	9	<nnxnp>	nn	262	np	1		
>ref-207760	TATCACGAAACACTCTCTCCCTCTCTC	21	<nnxnp>	nn	100	np	154		
>ref-207761	ACCCTCGTTACTACCACTCCTACCACC	2	<hkxhk>	hh	18	hk	247	kk	0
>ref-207761	ACCCTCGTTACTACCACTCCTACCACC	5	<hkxhk>	hh	0	hk	247	kk	18
>ref-207761	ACCCTCGTTACTACCACTCCTACCACC	8	<lmxll>	ll	49	lm	204		
>ref-207761	ACCCTCGTTACTACCACTCCTACCACC	22	<hkxhk>	hh	5	hk	263	kk	0
>ref-207761	ACCCTCGTTACTACCACTCCTACCACC	26	<lmxll>	ll	72	lm	177		
>ref-207761	ACCCTCGTTACTACCACTCCTACCACC	27	<hkxhk>	hh	5	hk	263	kk	0
>ref-207768	TCTTCCTCTACTCTTTCTCCGTCTTCC	24	<lmxll>	ll	117	lm	135		
>ref-207768	TCTTCCTCTACTCTTTCTCCGTCTTCC	25	<lmxll>	ll	117	lm	134		
>ref-207769	ATCTTTCACACGCCCTCTCCTTCCTCA	4	<nnxnp>	nn	119	np	151		
>ref-207770	CCTCCTGATACCTCACCTCCTGCCCCT	16	<lmxll>	ll	126	lm	134		
>ref-207782	CTCACAACCACTGGAGCTCCGTCCACT	24	<lmxll>	ll	161	lm	110		
>ref-207810	TTTGCTCTCACACCCTCTCCTGCACGT	25	<hkxhk>	hh	46	hk	139	kk	67
>ref-207839	ACAACCCTCACTCACCCTCCCAACAAT	6	<lmxll>	ll	131	lm	98		
>ref-207839	ACAACCCTCACTCACCCTCCCAACAAT	12	<lmxll>	ll	131	lm	98		
>ref-207849	TGCCCGACCACTCTCACTCCCCCACTT	24	<hkxhk>	hh	55	hk	149	kk	54
>ref-207853	TCCTCTTCTACACTAACTCCATTCCCT	21	<lmxll>	ll	122	lm	132		
>ref-207870	CCTTCCTCTACCTCCTCTCCCCCCCAT	9	<lmxll>	ll	262	lm	0		
>ref-207930	CCCACAACTACCCCCTCTCCCTCCTAT	26	<hkxhk>	hh	0	hk	273	kk	0
>ref-207936	ATTTATCTCACGACTTCTCCAGGCTCT	7	<lmxll>	ll	123	lm	118		
>ref-207949	CACACACCCACACCCCCTCCCTACCCC	1	<lmxll>	ll	26	lm	225		
>ref-207949	CACACACCCACACCCCCTCCCTACCCC	8	<hkxhk>	hh	2	hk	268	kk	0
>ref-207949	CACACACCCACACCCCCTCCCTACCCC	13	<hkxhk>	hh	0	hk	273	kk	0
>ref-207951	TTTAAGTCCACCCTCTCTCCCCTGTCC	15	<nnxnp>	nn	128	np	116		
>ref-207956	CCTCCAACAACCTTTGCTCCTCTCCCT	6	<hkxhk>	hh	72	hk	124	kk	63
>ref-207979	CGCATCTTCACCGCATCTCCACTGTAT	3	<lmxll>	ll	107	lm	145		
>ref-207979	CGCATCTTCACCGCATCTCCACTGTAT	13	<hkxhk>	hh	45	hk	155	kk	54

>ref-207982	CACGTACCAACACATCCTCCATGCACA	26	<nnxnp>	nn	135	np	130		
>ref-207983	TACAATCACACTCTCCCTCCACGCATC	1	<lmxll>	ll	194	lm	64		
>ref-207983	TACAATCACACTCTCCCTCCACGCATC	3	<nnxnp>	nn	131	np	129		
>ref-207983	TACAATCACACTCTCCCTCCACGCATC	5	<hkxhk>	hh	3	hk	270	kk	0
>ref-207983	TACAATCACACTCTCCCTCCACGCATC	7	<hkxhk>	hh	3	hk	270	kk	0
>ref-207983	TACAATCACACTCTCCCTCCACGCATC	13	<lmxll>	ll	192	lm	67		
>ref-207983	TACAATCACACTCTCCCTCCACGCATC	24	<hkxhk>	hh	86	hk	177	kk	0
>ref-207983	TACAATCACACTCTCCCTCCACGCATC	25	<hkxhk>	hh	1	hk	271	kk	0
>ref-207984	CCCACTTCCACCCCCTCTCCTCCCTAA	4	<lmxll>	ll	8	lm	245		
>ref-207984	CCCACTTCCACCCCCTCTCCTCCCTAA	12	<lmxll>	ll	151	lm	109		
>ref-207984	CCCACTTCCACCCCCTCTCCTCCCTAA	26	<lmxll>	ll	8	lm	245		
>ref-207994	CCCCCGTCAACCTTTCCTCCTTCCTTC	23	<lmxll>	ll	147	lm	123		
>ref-208008	GCCGGCTAAACTATTCCTCCTTCTACC	1	<hkxhk>	hh	63	hk	127	kk	57
>ref-208012	TCTCTCTCGACGCTGTCTCCACCAACC	2	<lmxll>	ll	42	lm	219		
>ref-208012	TCTCTCTCGACGCTGTCTCCACCAACC	6	<hkxhk>	hh	0	hk	273	kk	0
>ref-208028	TGCAGGCCCACAATCCCTCCCTTGAAA	16	<nnxnp>	nn	126	np	132		
>ref-208035	TGTTCACAGACTCTGGCTCCTCCCCCT	6	<lmxll>	ll	136	lm	129		
>ref-208035	TGTTCACAGACTCTGGCTCCTCCCCCT	9	<lmxll>	ll	136	lm	129		
>ref-208048	CACATTCTCACTATTTCTCCGTCTGCC	4	<hkxhk>	hh	56	hk	139	kk	46
>ref-208085	ACTGCTATCACAGTACCTCCTCTCTCC	27	<nnxnp>	nn	146	np	125		
>ref-208088	CCGTTCACCACGTGACCTCCACAAGCA	13	<nnxnp>	nn	127	np	93		
>ref-208088	CCGTTCACCACGTGACCTCCACAAGCA	24	<nnxnp>	nn	127	np	93		
>ref-208105	ATAGTGTGAACCAGTCCTCCTGCTGAG	4	<lmxll>	ll	1	lm	268		
>ref-208105	ATAGTGTGAACCAGTCCTCCTGCTGAG	5	<lmxll>	ll	1	lm	268		
>ref-208116	CATACACGCACACATTCTCCTACTCAC	25	<nnxnp>	nn	120	np	133		
>ref-208117	ATATCAGTTACACTCTCTCCATTACTC	14	<lmxll>	ll	132	lm	131		
>ref-208119	ACTTCTCTTACCACTACTCCATCTTCC	5	<lmxll>	ll	133	lm	118		
>ref-208147	TACAAACTTACTATTTCTCCTCTGCAG	26	<nnxnp>	nn	268	np	0		
>ref-208175	TTCACCCCCACCCCCTCTCCTTAGCTT	5	<nnxnp>	nn	122	np	117		
>ref-208189	AGCTCCCCGACCGCCGCTCCTTCCGAG	12	<lmxll>	ll	111	lm	145		
>ref-208194	CCCAGTCGCACCTCTCCTCCTGCCTTC	3	<hkxhk>	hh	138	hk	114	kk	0
>ref-208195	CTCTTTCCTACAATTACTCCTTCCTCT	6	<nnxnp>	nn	114	np	139		
>ref-208215	CATCCCCTCACTTCTCCTCCTCCCCTC	12	<lmxll>	ll	264	lm	3		
>ref-208215	CATCCCCTCACTTCTCCTCCTCCCCTC	14	<lmxll>	ll	15	lm	242		
>ref-208215	CATCCCCTCACTTCTCCTCCTCCCCTC	16	<lmxll>	ll	268	lm	1		
>ref-208215	CATCCCCTCACTTCTCCTCCTCCCCTC	26	<lmxll>	ll	110	lm	130		
>ref-208227	AGATAGCACACCTTCACTCCACGCACC	1	<nnxnp>	nn	135	np	138		
>ref-208227	AGATAGCACACCTTCACTCCACGCACC	26	<nnxnp>	nn	132	np	141		

>ref-208233	CTGCATAATACTGTCGCTCCACTCGAG	27	<nnxnp>	nn	272	np	0		
>ref-208240	TCCGCCCGTACCGGACCTCCGCAGAGT	14	<nnxnp>	nn	128	np	120		
>ref-208243	CCAACCTTCACTCATTCTCCCTCCCTG	1	<lmxll>	ll	128	lm	132		
>ref-208276	ATGCTCATTACAGTACCTCCATCACTT	1	<lmxll>	ll	136	lm	114		
>ref-208276	ATGCTCATTACAGTACCTCCATCACTT	16	<lmxll>	ll	110	lm	138		
>ref-208297	CCTGTTGAAACGCAATCTCCAACAGCG	26	<lmxll>	ll	120	lm	138		
>ref-208345	GTGATTATGACTAGGCCTCCATCATTA	6	<lmxll>	ll	96	lm	143		
>ref-208345	GTGATTATGACTAGGCCTCCATCATTA	7	<lmxll>	ll	96	lm	143		
>ref-208349	CGTATCCTCACCTTTACTCCCTCACCC	6	<lmxll>	ll	141	lm	105		
>ref-208350	GATTTGATCACGTTGACTCCCCATGTT	23	<lmxll>	ll	134	lm	130		
>ref-208352	CTAACCCTTACTTCATCTCCTTCTCTC	2	<lmxll>	ll	136	lm	129		
>ref-208354	CCGATGACAACACTCCCTCCGCTCAGC	3	<hkxhk>	hh	0	hk	269	kk	3
>ref-208354	CCGATGACAACACTCCCTCCGCTCAGC	12	<hkxhk>	hh	1	hk	271	kk	0
>ref-208354	CCGATGACAACACTCCCTCCGCTCAGC	14	<hkxhk>	hh	0	hk	269	kk	3
>ref-208354	CCGATGACAACACTCCCTCCGCTCAGC	23	<hkxhk>	hh	0	hk	271	kk	1
>ref-208359	ATCCCCACCACCAGCGCTCCCCGTCCT	22	<hkxhk>	hh	56	hk	147	kk	57
>ref-208401	ATCACTCAGACCCGACCTCCTTCCTCC	14	<lmxll>	ll	120	lm	129		
>ref-208402	TGCTCCCACACATCCTCTCCCTCTCCT	23	<nnxnp>	nn	130	np	135		
>ref-208407	TTAAGGGAAACCAAGGCTCCATCTCCC	25	<lmxll>	ll	135	lm	130		
>ref-208410	CCTTTGCATACCTTCTCTCCGTCTTAT	13	<lmxll>	ll	111	lm	117		
>ref-208410	CCTTTGCATACCTTCTCTCCGTCTTAT	26	<lmxll>	ll	111	lm	117		
>ref-208434	TCTTCTTCAACTAACGCTCCAGGATGC	3	<nnxnp>	nn	130	np	99		
>ref-208454	TCCTCCCTGACGCCCCCTCCACTCTTT	12	<hkxhk>	hh	3	hk	266	kk	0
>ref-208454	TCCTCCCTGACGCCCCCTCCACTCTTT	21	<hkxhk>	hh	3	hk	266	kk	0
>ref-208471	GAGATTATTACATACCCTCCCACCTCA	5	<nnxnp>	nn	114	np	122		
>ref-208471	GAGATTATTACATACCCTCCCACCTCA	6	<lmxll>	ll	126	lm	119		
>ref-208515	CTCGCGCTTACGTCTCCTCCTTGTGTA	2	<lmxll>	ll	137	lm	131		
>ref-208522	CCTGTTTTCACGAAAGCTCCCACCTCT	23	<hkxhk>	hh	0	hk	272	kk	1
>ref-208522	CCTGTTTTCACGAAAGCTCCCACCTCT	26	<hkxhk>	hh	18	hk	236	kk	1
>ref-208537	TCATTACCCACACTTCCTCCGTCTCTC	22	<nnxnp>	nn	134	np	129		
>ref-208575	CAACCCCACACCTGTTCTCCGTGATGA	9	<nnxnp>	nn	134	np	130		
>ref-208600	CGGCAGCTGACAGAGTCTCCTGCTTAA	1	<hkxhk>	hh	258	hk	11	kk	0
>ref-208600	CGGCAGCTGACAGAGTCTCCTGCTTAA	6	<hkxhk>	hh	0	hk	9	kk	256
>ref-208600	CGGCAGCTGACAGAGTCTCCTGCTTAA	26	<lmxll>	ll	175	lm	85		
>ref-208629	TCCTCTGTTACTGCGTCTCCCACTTCT	8	<lmxll>	ll	137	lm	134		
>ref-208629	TCCTCTGTTACTGCGTCTCCCACTTCT	24	<lmxll>	ll	137	lm	134		
>ref-208703	GACCTTTCAACTTTCCCTCCCCGAGAA	23	<nnxnp>	nn	120	np	134		
>ref-208717	CTTATCCTAACCTTTTCTCCTCCGTCT	4	<hkxhk>	hh	56	hk	151	kk	50

>ref-208722	CCCTCTTTCACCCCTTCTCCCAACTAT	16	<lmxll>	ll	249	lm	0		
>ref-208727	ACCTATACAACAGAGTCTCCCCCTGAA	2	<lmxll>	ll	125	lm	136		
>ref-208744	TAAATATACACACATACTCCGCCACGC	3	<lmxll>	ll	117	lm	119		
>ref-208744	TAAATATACACACATACTCCGCCACGC	26	<lmxll>	ll	112	lm	129		
>ref-208745	TTCTCTCTTACTCCTCCTCCTTATCAT	3	<hkxhk>	hh	16	hk	247	kk	0
>ref-208745	TTCTCTCTTACTCCTCCTCCTTATCAT	13	<hkxhk>	hh	11	hk	252	kk	0
>ref-208745	TTCTCTCTTACTCCTCCTCCTTATCAT	24	<hkxhk>	hh	0	hk	252	kk	11
>ref-208745	TTCTCTCTTACTCCTCCTCCTTATCAT	25	<hkxhk>	hh	17	hk	245	kk	0
>ref-208789	ATGTGACCGACTCCTCCTCCTTAATCT	1	<nnxnp>	nn	121	np	140		
>ref-208799	TTGTTACATACAGATGCTCCACATGTG	1	<lmxll>	ll	19	lm	252		
>ref-208799	TTGTTACATACAGATGCTCCACATGTG	2	<lmxll>	ll	19	lm	252		
>ref-208799	TTGTTACATACAGATGCTCCACATGTG	22	<hkxhk>	hh	1	hk	272	kk	0
>ref-208799	TTGTTACATACAGATGCTCCACATGTG	23	<hkxhk>	hh	1	hk	272	kk	0
>ref-208828	TTAATCCCTACCCTTACTCCTTCCCTT	15	<nnxnp>	nn	152	np	111		
>ref-208828	TTAATCCCTACCCTTACTCCTTCCCTT	22	<hkxhk>	hh	1	hk	257	kk	7
>ref-208828	TTAATCCCTACCCTTACTCCTTCCCTT	26	<hkxhk>	hh	1	hk	257	kk	7
>ref-208837	TTCACCCTCACGTCTCCTCCCAGGAAC	6	<hkxhk>	hh	33	hk	221	kk	0
>ref-208837	TTCACCCTCACGTCTCCTCCCAGGAAC	7	<hkxhk>	hh	0	hk	272	kk	1
>ref-208837	TTCACCCTCACGTCTCCTCCCAGGAAC	23	<hkxhk>	hh	1	hk	272	kk	0
>ref-208839	CCACCAGTGACTCCATCTCCCTCACAA	5	<lmxll>	ll	115	lm	105		
>ref-208847	CTTCACCCGACGTTTCCTCCATCCCCC	7	<lmxll>	ll	122	lm	143		
>ref-208858	AACTCAGCAACTCCTCCTCCTCCTCCT	3	<nnxnp>	nn	231	np	1		
>ref-208858	AACTCAGCAACTCCTCCTCCTCCTCCT	5	<nnxnp>	nn	262	np	0		
>ref-208858	AACTCAGCAACTCCTCCTCCTCCTCCT	6	<nnxnp>	nn	259	np	1		
>ref-208858	AACTCAGCAACTCCTCCTCCTCCTCCT	9	<nnxnp>	nn	248	np	4		
>ref-208868	GCCACTGACACCTGAGCTCCTGCATCT	23	<lmxll>	ll	10	lm	261		
>ref-208868	GCCACTGACACCTGAGCTCCTGCATCT	24	<lmxll>	ll	10	lm	261		
>ref-208872	GCTATTCGGACAGTCACTCCGTCCTTG	25	<lmxll>	ll	141	lm	124		
>ref-208875	ATGTGAGTAACAGGACCTCCGCCTCCC	25	<nnxnp>	nn	130	np	129		
>ref-208884	AAAGAATACACTCTCCCTCCCTCAACA	27	<nnxnp>	nn	128	np	127		
>ref-208895	CCCCTTAGCACCGTTCCTCCCCCCCTT	25	<nnxnp>	nn	122	np	128		
>ref-208895	CCCCTTAGCACCGTTCCTCCCCCCCTT	27	<nnxnp>	nn	121	np	128		
>ref-208910	TCATCCCCAACAATCTCTCCCCTTCTC	12	<nnxnp>	nn	156	np	93		
>ref-208910	TCATCCCCAACAATCTCTCCCCTTCTC	13	<nnxnp>	nn	154	np	93		
>ref-208927	CCCTTTTCAACCACGTCTCCTTTATTC	6	<lmxll>	ll	115	lm	134		
>ref-208932	AAGGCTGCGACTTTGCCTCCATCATTG	8	<lmxll>	ll	130	lm	126		
>ref-208962	ACCTTCCTAACTGCGTCTCCTCCTTCT	1	<lmxll>	ll	137	lm	89		
>ref-208985	CCCCTTCTCACTTCCTCTCCTCCTAAC	14	<hkxhk>	hh	44	hk	163	kk	60

>ref-208985	CCCCTTCTCACTTCCTCTCCTCCTAAC	15	<hkxhk>	hh	60	hk	163	kk	44
>ref-209018	ATTTTGATCACACTCTCTCCTCTCTCT	7	<lmxll>	ll	116	lm	141		
>ref-209018	ATTTTGATCACACTCTCTCCTCTCTCT	9	<lmxll>	ll	116	lm	141		
>ref-209034	CCCGCGCCCACCTCCTCTCCCCAACCC	2	<nnxnp>	nn	255	np	0		
>ref-209034	CCCGCGCCCACCTCCTCTCCCCAACCC	4	<lmxll>	ll	102	lm	141		
>ref-209037	CCCTCCAACACCCACCCTCCGACACCC	3	<nnxnp>	nn	216	np	43		
>ref-209037	CCCTCCAACACCCACCCTCCGACACCC	7	<hkxhk>	hh	16	hk	245	kk	0
>ref-209037	CCCTCCAACACCCACCCTCCGACACCC	21	<hkxhk>	hh	0	hk	272	kk	0
>ref-209047	ACATCACCGACACTCTCTCCAGCCTAC	26	<lmxll>	ll	111	lm	149		
>ref-209054	TATCTTCCTACACTCCCTCCTCCTCCT	12	<nnxnp>	nn	162	np	83		
>ref-209061	TCACCCCCTACCACTCCTCCCTCCACT	4	<lmxll>	ll	269	lm	0		
>ref-209075	GTCTCCTCCACCCTCTCTCCCTGTCTC	9	<hkxhk>	hh	3	hk	269	kk	0
>ref-209075	GTCTCCTCCACCCTCTCTCCCTGTCTC	23	<hkxhk>	hh	0	hk	269	kk	2
>ref-209081	CTGCTTGCTACCGCTGCTCCTGCTTCC	8	<lmxll>	ll	127	lm	144		
>ref-209089	AATTAATATACTAAATCTCCCTTGACC	5	<lmxll>	ll	5	lm	262		
>ref-209089	AATTAATATACTAAATCTCCCTTGACC	25	<hkxhk>	hh	24	hk	242	kk	0
>ref-209098	GTCATGCTTACACAATCTCCTCGTGCA	5	<nnxnp>	nn	124	np	146		
>ref-209123	CTCTATTTCACTCCCCCTCCCCCCATC	4	<lmxll>	ll	14	lm	241		
>ref-209123	CTCTATTTCACTCCCCCTCCCCCCATC	12	<hkxhk>	hh	1	hk	142	kk	97
>ref-209150	ACATCAACAACTTCGCCTCCGCCTCAA	9	<lmxll>	ll	153	lm	108		
>ref-209185	ATTCAAGCCACTCACTCTCCACGACGA	22	<nnxnp>	nn	122	np	138		
>ref-209185	ATTCAAGCCACTCACTCTCCACGACGA	23	<nnxnp>	nn	122	np	138		
>ref-209195	TTCACTTTGACTTCTTCTCCTTCTCTT	3	<hkxhk>	hh	5	hk	263	kk	0
>ref-209195	TTCACTTTGACTTCTTCTCCTTCTCTT	25	<hkxhk>	hh	5	hk	263	kk	0
>ref-209202	CTACCTTATACCTTTCCTCCCCCTTTA	7	<hkxhk>	hh	56	hk	124	kk	52
>ref-209247	CTTCTGTCTACTTTCCCTCCCTCCTTC	2	<nnxnp>	nn	146	np	112		
>ref-209247	CTTCTGTCTACTTTCCCTCCCTCCTTC	9	<hkxhk>	hh	1	hk	264	kk	1
>ref-209247	CTTCTGTCTACTTTCCCTCCCTCCTTC	24	<hkxhk>	hh	1	hk	264	kk	1
>ref-209248	AACTCTTTTACATTCCCTCCTTACTCA	2	<nnxnp>	nn	116	np	134		
>ref-209253	CGGATTCACACACATTCTCCATTCTCC	2	<hkxhk>	hh	62	hk	147	kk	47
>ref-209261	TATCCGTGTACTCTATCTCCCTCTCTT	2	<hkxhk>	hh	46	hk	120	kk	60
>ref-209290	TCACGAATGACTCACTCTCCTTTTCTG	27	<nnxnp>	nn	111	np	117		
>ref-209296	CCCTAACCCACCCTCACTCCCACCTTT	1	<lmxll>	ll	221	lm	13		
>ref-209296	CCCTAACCCACCCTCACTCCCACCTTT	4	<nnxnp>	nn	117	np	137		
>ref-209300	ATTACTGCTACTTAAACTCCTCATCTA	22	<hkxhk>	hh	0	hk	228	kk	15
>ref-209315	CTCTCCCCCACCCTATCTCCTCCCCCT	1	<nnxnp>	nn	261	np	0		
>ref-209315	CTCTCCCCCACCCTATCTCCTCCCCCT	2	<lmxll>	ll	202	lm	30		
>ref-209315	CTCTCCCCCACCCTATCTCCTCCCCCT	13	<lmxll>	ll	0	lm	259		

>ref-209323	CCCTCCCCTACCCTCTCTCCTCATAAT	9	<lmxll>	ll	119	lm	129		
>ref-209336	TCTGCTTTGACAACCTCTCCTTCCTCA	12	<hkxhk>	hh	0	hk	258	kk	0
>ref-209338	ATCTCCACAACTACATCTCCACATTCA	14	<nnxnp>	nn	137	np	120		
>ref-209339	AGATCTTACACAGTCGCTCCCGCACAG	27	<nnxnp>	nn	269	np	0		
>ref-209347	TTCCCACCTACAATCCCTCCTCTTCAT	22	<nnxnp>	nn	120	np	126		
>ref-209348	ACACTTTGTACACTTCCTCCATTTCTC	21	<nnxnp>	nn	85	np	151		
>ref-209352	TTTCTCTACACTCGCCCTCCTTTCTTC	21	<lmxll>	ll	126	lm	133		
>ref-209377	CCCTTCTTCACTTTATCTCCGCTCTTT	9	<nnxnp>	nn	114	np	141		
>ref-209377	CCCTTCTTCACTTTATCTCCGCTCTTT	12	<lmxll>	ll	120	lm	133		
>ref-209377	CCCTTCTTCACTTTATCTCCGCTCTTT	21	<hkxhk>	hh	42	hk	153	kk	57
>ref-209392	GACTCGGTGACCTCTCCTCCTCCTCCT	9	<hkxhk>	hh	0	hk	272	kk	0
>ref-209392	GACTCGGTGACCTCTCCTCCTCCTCCT	12	<hkxhk>	hh	0	hk	272	kk	0
>ref-209415	CCTTCCCCTACACTCCCTCCCATCTTC	9	<nnxnp>	nn	123	np	124		
>ref-209442	TAACCCTCCACCTTCCCTCCCTGCCTC	3	<hkxhk>	hh	3	hk	253	kk	7
>ref-209442	TAACCCTCCACCTTCCCTCCCTGCCTC	14	<hkxhk>	hh	7	hk	255	kk	3
>ref-209443	CCCGTATCCACTCGCCCTCCTTTGTGT	3	<lmxll>	ll	132	lm	136		
>ref-209472	CCATCGTCCACCAATTCTCCTCCTCCT	26	<nnxnp>	nn	148	np	106		
>ref-209485	CGACTCGGAACTTCGCCTCCTGATGGA	27	<hkxhk>	hh	64	hk	150	kk	46
>ref-209516	CGCTTCCACACCGCGCCTCCAATCGTA	3	<hkxhk>	hh	0	hk	271	kk	1
>ref-209516	CGCTTCCACACCGCGCCTCCAATCGTA	12	<lmxll>	ll	5	lm	268		
>ref-209537	CGATTTGAGACTGCCACTCCATACTCC	1	<hkxhk>	hh	0	hk	273	kk	0
>ref-209537	CGATTTGAGACTGCCACTCCATACTCC	2	<hkxhk>	hh	3	hk	268	kk	0
>ref-209537	CGATTTGAGACTGCCACTCCATACTCC	12	<hkxhk>	hh	0	hk	268	kk	3
>ref-209543	GGGGTCTACACCTGTCCTCCTGCGAGA	1	<nnxnp>	nn	140	np	129		
>ref-209543	GGGGTCTACACCTGTCCTCCTGCGAGA	23	<nnxnp>	nn	130	np	140		
>ref-209559	TATGCTTCCACTTTCTCTCCTCGCTTA	4	<nnxnp>	nn	122	np	108		
>ref-209568	CATTAATCTACCCTCCCTCCCTCTCCT	13	<hkxhk>	hh	3	hk	265	kk	0
>ref-209568	CATTAATCTACCCTCCCTCCCTCTCCT	21	<hkxhk>	hh	0	hk	265	kk	3
>ref-209581	ACATGTTTCACGTTCTCTCCAAGGGCC	21	<hkxhk>	hh	67	hk	155	kk	43
>ref-209583	ACCAAAGTAACCCTTTCTCCAACCAAC	6	<lmxll>	ll	116	lm	139		
>ref-209591	TTGACTTTTACACGCCCTCCCTCCCTT	24	<lmxll>	ll	231	lm	0		
>ref-209618	CTAAGTCACACTCACGCTCCAGTCCTC	6	<nnxnp>	nn	143	np	114		
>ref-209618	CTAAGTCACACTCACGCTCCAGTCCTC	15	<nnxnp>	nn	141	np	115		
>ref-209634	CACACACACACGCACTCTCCTTTTCGT	12	<lmxll>	ll	187	lm	78		
>ref-209634	CACACACACACGCACTCTCCTTTTCGT	21	<lmxll>	ll	185	lm	81		
>ref-209634	CACACACACACGCACTCTCCTTTTCGT	25	<lmxll>	ll	192	lm	71		
>ref-209634	CACACACACACGCACTCTCCTTTTCGT	26	<lmxll>	ll	190	lm	73		
>ref-209675	GACCCATTCACCTCCCCTCCTTCACCC	6	<hkxhk>	hh	1	hk	272	kk	0

>ref-209675	GACCCATTCACCTCCCCTCCTTCACCC	21	<hkxhk>	hh	0	hk	272	kk	1
>ref-209686	CGTGACAGTACAATCCCTCCCACCTGA	1	<lmxll>	ll	115	lm	118		
>ref-209693	GATCCTCGAACTAAACCTCCTTCACTC	22	<hkxhk>	hh	0	hk	263	kk	4
>ref-209707	CTCCTATCTACTCCCTCTCCTTCCTAC	3	<hkxhk>	hh	13	hk	252	kk	0
>ref-209707	CTCCTATCTACTCCCTCTCCTTCCTAC	21	<hkxhk>	hh	13	hk	252	kk	0
>ref-209733	TCAGACACGACATCCTCTCCGCATGAC	5	<nnxnp>	nn	133	np	135		
>ref-209740	CGACAAAACACCCAGACTCCATCCCAT	1	<hkxhk>	hh	1	hk	270	kk	1
>ref-209740	CGACAAAACACCCAGACTCCATCCCAT	22	<hkxhk>	hh	1	hk	270	kk	1
>ref-209748	TTAGCCTCGACTTATGCTCCCGATATT	8	<lmxll>	ll	20	lm	231		
>ref-209748	TTAGCCTCGACTTATGCTCCCGATATT	12	<hkxhk>	hh	0	hk	232	kk	11
>ref-209748	TTAGCCTCGACTTATGCTCCCGATATT	16	<hkxhk>	hh	0	hk	232	kk	11
>ref-209758	GTCTAGGCCACTTTTCCTCCTGCTGAT	23	<nnxnp>	nn	141	np	123		
>ref-209767	GCATGCGCCACCTCTGCTCCTGGACAG	9	<nnxnp>	nn	126	np	144		
>ref-209767	GCATGCGCCACCTCTGCTCCTGGACAG	25	<lmxll>	ll	134	lm	137		
>ref-209769	AAAGACCCCACCTTCCCTCCTTCAGTA	4	<hkxhk>	hh	43	hk	151	kk	61
>ref-209769	AAAGACCCCACCTTCCCTCCTTCAGTA	8	<lmxll>	ll	126	lm	137		
>ref-209776	GATAATACAACGGCATCTCCTCCTTCA	25	<nnxnp>	nn	139	np	122		
>ref-209779	CTTCCTCCCACGAACCCTCCTCCCCCG	27	<nnxnp>	nn	98	np	138		
>ref-209796	TCGTGATCGACACGCTCTCCGCACGTC	8	<nnxnp>	nn	133	np	135		
>ref-209810	ATCCCTACGACTGCTGCTCCACGATCT	13	<nnxnp>	nn	224	np	0		
>ref-209821	TGTTTCGCCACCCCCTCTCCTCTCCTC	13	<lmxll>	ll	126	lm	137		
>ref-209827	CTACCACCCACTTTCACTCCCCTGCTG	3	<lmxll>	ll	120	lm	134		
>ref-209828	CTTCTCCCGACATCATCTCCATCTCCT	3	<hkxhk>	hh	67	hk	148	kk	39
>ref-209838	TACACAGGTACACCCACTCCATCTTGA	6	<nnxnp>	nn	216	np	40		
>ref-209838	TACACAGGTACACCCACTCCATCTTGA	9	<hkxhk>	hh	0	hk	253	kk	9
>ref-209838	TACACAGGTACACCCACTCCATCTTGA	13	<lmxll>	ll	178	lm	56		
>ref-209838	TACACAGGTACACCCACTCCATCTTGA	24	<lmxll>	ll	199	lm	56		
>ref-209838	TACACAGGTACACCCACTCCATCTTGA	25	<nnxnp>	nn	266	np	5		
>ref-209850	CTGTACGGTACACGACCTCCTGTCTTT	5	<hkxhk>	hh	0	hk	273	kk	0
>ref-209850	CTGTACGGTACACGACCTCCTGTCTTT	16	<hkxhk>	hh	0	hk	273	kk	0
>ref-209851	TCCAGGGTCACGTGATCTCCCGCCTCA	9	<nnxnp>	nn	146	np	115		
>ref-209883	CTTTCACCAACCCTTTCTCCCTCTTTC	21	<lmxll>	ll	116	lm	134		
>ref-209890	TACACACACACAGCCCCTCCCCACTCC	1	<nnxnp>	nn	113	np	150		
>ref-209902	CTATATTTCACCCTCTCTCCTCCATCA	2	<lmxll>	ll	263	lm	0		
>ref-209913	CTCCACGTGACCCGTCCTCCGCTCCCC	14	<nnxnp>	nn	55	np	203		
>ref-209925	CCGCTCCCTACACCTCCTCCACCCTTC	3	<nnxnp>	nn	123	np	143		
>ref-209950	GTGACAGTGACATTCCCTCCATGATGT	5	<hkxhk>	hh	115	hk	130	kk	1
>ref-209974	GTTCTTCTCACCGTCCCTCCTCCCCCC	12	<nnxnp>	nn	266	np	0		

>ref-209990	CTTCCTTTTACCTTTCCTCCTCTTCTA	23	<nnxnp>	nn	254	np	0		
>ref-210008	CAACCCGCCACCACACCTCCCTTACCA	7	<lmxll>	ll	126	lm	133		
>ref-210045	TTCCTCCCCACTCACTCTCCCTCCCCC	8	<lmxll>	ll	98	lm	147		
>ref-210067	CTCTCTCTTACTTCTTCTCCCACACTT	2	<lmxll>	ll	105	lm	119		
>ref-210088	TTCACAACCACAGCAACTCCGTTCCTC	7	<hkxhk>	hh	66	hk	201	kk	0
>ref-210088	TTCACAACCACAGCAACTCCGTTCCTC	13	<hkxhk>	hh	0	hk	201	kk	67
>ref-210094	ATCCCCCCAACACTAACTCCTCCCACA	1	<lmxll>	ll	120	lm	106		
>ref-210094	ATCCCCCCAACACTAACTCCTCCCACA	3	<lmxll>	ll	123	lm	104		
>ref-210100	TCACACTTCACCCTATCTCCATTCATC	25	<lmxll>	ll	128	lm	124		
>ref-210107	AAAACACCCACCTCCTCTCCCCTCCAT	24	<nnxnp>	nn	119	np	128		
>ref-210112	GTAAATACCACACGACCTCCTGCGGCG	8	<lmxll>	ll	120	lm	145		
>ref-210133	AACCCATTTACCTCAACTCCATCTTTC	27	<nnxnp>	nn	127	np	132		
>ref-210141	CAAGCAACAACCACAACTCCCGTGATC	5	<lmxll>	ll	1	lm	251		
>ref-210182	TCCCTAGTAACTATCCCTCCATTTCCT	27	<nnxnp>	nn	245	np	0		
>ref-210216	TCCTTTCTCACTTCCCCTCCCTCCCTC	1	<hkxhk>	hh	0	hk	268	kk	1
>ref-210216	TCCTTTCTCACTTCCCCTCCCTCCCTC	2	<hkxhk>	hh	49	hk	218	kk	0
>ref-210216	TCCTTTCTCACTTCCCCTCCCTCCCTC	5	<hkxhk>	hh	0	hk	272	kk	1
>ref-210216	TCCTTTCTCACTTCCCCTCCCTCCCTC	16	<lmxll>	ll	80	lm	173		
>ref-210216	TCCTTTCTCACTTCCCCTCCCTCCCTC	23	<lmxll>	ll	194	lm	61		
>ref-210216	TCCTTTCTCACTTCCCCTCCCTCCCTC	24	<lmxll>	ll	45	lm	220		
>ref-210216	TCCTTTCTCACTTCCCCTCCCTCCCTC	25	<lmxll>	ll	47	lm	217		
>ref-210231	CCGTCCCATACTCTCCCTCCCTCCCCT	1	<nnxnp>	nn	137	np	128		
>ref-210240	TTGCTTCTCACATCCTCTCCCAATATT	27	<lmxll>	ll	124	lm	118		
>ref-210254	CTCATCCTGACTCATTCTCCCACTTAC	23	<nnxnp>	nn	137	np	99		
>ref-210254	CTCATCCTGACTCATTCTCCCACTTAC	27	<lmxll>	ll	132	lm	112		
>ref-210257	ATCTCCCCCACCTCTCCTCCTGTCTCC	27	<nnxnp>	nn	105	np	151		
>ref-210258	TATTGCACAACGCTAACTCCGTACTTA	8	<lmxll>	ll	0	lm	268		
>ref-210271	GTTCTGGCGACGCTTCCTCCATCTATG	25	<lmxll>	ll	123	lm	135		
>ref-210279	GTTTTCCTAACTATTTCTCCTCCCCTT	23	<nnxnp>	nn	261	np	0		
>ref-210294	CGCAAAGCCACACGGACTCCCACCGGG	5	<lmxll>	ll	97	lm	135		
>ref-210301	ACCACTACCACCACCACTCCCACCAAT	3	<lmxll>	ll	142	lm	108		
>ref-210326	ACTCATTGTACCATCCCTCCAACGCAT	3	<hkxhk>	hh	0	hk	256	kk	10
>ref-210326	ACTCATTGTACCATCCCTCCAACGCAT	4	<nnxnp>	nn	187	np	77		
>ref-210330	GAAGTCTACACATGAACTCCTGTCCAT	1	<lmxll>	ll	137	lm	122		
>ref-210346	GTACTCTAAACAGCATCTCCAAATGCC	27	<nnxnp>	nn	144	np	118		
>ref-210366	CTCTTTGCTACTCATCCTCCTTCTGTT	12	<nnxnp>	nn	125	np	126		
>ref-210380	CTGGATCTCACTTTGCCTCCCCTTATA	8	<lmxll>	ll	147	lm	113		
>ref-210380	CTGGATCTCACTTTGCCTCCCCTTATA	13	<hkxhk>	hh	0	hk	119	kk	142

>ref-210395	AACACACAAACTTATCCTCCCTCGCGA	3	<lmxll>	ll	120	lm	117		
>ref-210410	CTCCTTACAACCCTCCCTCCCTTCCTT	9	<hkxhk>	hh	15	hk	212	kk	3
>ref-210410	CTCCTTACAACCCTCCCTCCCTTCCTT	26	<nnxnp>	nn	188	np	47		
>ref-210410	CTCCTTACAACCCTCCCTCCCTTCCTT	27	<lmxll>	ll	236	lm	5		
>ref-210413	CACGGAATAACTCATTCTCCATTCATC	26	<lmxll>	ll	133	lm	131		
>ref-210415	CCCACAAGAACGAAGACTCCTGCTTCT	26	<nnxnp>	nn	159	np	102		
>ref-210426	TTCCTTTCTACCATTTCTCCTCTCCTT	7	<lmxll>	ll	28	lm	221		
>ref-210426	TTCCTTTCTACCATTTCTCCTCTCCTT	21	<lmxll>	ll	28	lm	221		
>ref-210445	AATTTAATCACGTACACTCCATGCTAC	6	<nnxnp>	nn	235	np	0		
>ref-210468	CACTATGACACTCTCTCTCCAATCGTC	8	<hkxhk>	hh	3	hk	269	kk	0
>ref-210468	CACTATGACACTCTCTCTCCAATCGTC	26	<lmxll>	ll	195	lm	68		
>ref-210470	CTTATCGATACTCTTCCTCCTTCTCCT	24	<lmxll>	ll	120	lm	123		
>ref-210481	TTCTGCCTCACTCTCTCTCCGTCTCAG	1	<nnxnp>	nn	139	np	129		
>ref-210481	TTCTGCCTCACTCTCTCTCCGTCTCAG	3	<nnxnp>	nn	139	np	129		
>ref-210483	TCAAAGGGCACTGTGTCTCCTAGAGAA	7	<lmxll>	ll	108	lm	147		
>ref-210492	CTACCCCCCACAACACCTCCCCATCTG	5	<nnxnp>	nn	97	np	142		
>ref-210492	CTACCCCCCACAACACCTCCCCATCTG	14	<nnxnp>	nn	96	np	142		
>ref-210526	AACTCCGGCACATTCCCTCCCTCCTCT	5	<nnxnp>	nn	128	np	129		
>ref-210530	CTCGCTCGTACCCTCTCTCCGTCTCTA	3	<nnxnp>	nn	140	np	125		
>ref-210530	CTCGCTCGTACCCTCTCTCCGTCTCTA	27	<nnxnp>	nn	135	np	125		
>ref-210550	AACTCCATCACTCCTCCTCCAAACCTC	1	<nnxnp>	nn	253	np	0		
>ref-210551	CTTGCTCCTACAATGTCTCCCCTCTTC	7	<lmxll>	ll	116	lm	122		
>ref-210560	TGTTAATGGACTGAAGCTCCATCGCGT	1	<lmxll>	ll	268	lm	0		
>ref-210597	TCGTTCCTAACCCAATCTCCAGGGTAC	2	<hkxhk>	hh	0	hk	266	kk	2
>ref-210597	TCGTTCCTAACCCAATCTCCAGGGTAC	4	<hkxhk>	hh	2	hk	266	kk	0
>ref-210604	CGTACCATCACTGCTACTCCATGAGGT	1	<hkxhk>	hh	1	hk	272	kk	0
>ref-210604	CGTACCATCACTGCTACTCCATGAGGT	2	<hkxhk>	hh	0	hk	266	kk	6
>ref-210604	CGTACCATCACTGCTACTCCATGAGGT	4	<lmxll>	ll	117	lm	139		
>ref-210604	CGTACCATCACTGCTACTCCATGAGGT	16	<hkxhk>	hh	0	hk	273	kk	0
>ref-210619	CCCATCACCACCATCCCTCCTAGAACC	21	<lmxll>	ll	130	lm	129		
>ref-210623	CTCCTTCCTACTTCACCTCCTTACCTC	15	<nnxnp>	nn	74	np	178		
>ref-210623	CTCCTTCCTACTTCACCTCCTTACCTC	23	<nnxnp>	nn	73	np	178		
>ref-210643	CCAGTCATCACACCCTCTCCCTCCCTC	15	<hkxhk>	hh	66	hk	152	kk	45
>ref-210707	TCCTTCCTCACCCTCACTCCTTCCTCT	1	<hkxhk>	hh	8	hk	261	kk	0
>ref-210707	TCCTTCCTCACCCTCACTCCTTCCTCT	24	<nnxnp>	nn	155	np	109		
>ref-210707	TCCTTCCTCACCCTCACTCCTTCCTCT	27	<hkxhk>	hh	29	hk	239	kk	0
>ref-210718	AACGTAAATACTGGAGCTCCATTCTTA	16	<lmxll>	ll	83	lm	136		
>ref-210718	AACGTAAATACTGGAGCTCCATTCTTA	25	<lmxll>	ll	90	lm	132		

>ref-210722	GAAAATAACACACGTCCTCCTCCCCTC	15	<nnxnp>	nn	116	np	127		
>ref-210730	ATTCCATATACCTTTTCTCCATGCGTT	25	<lmxll>	ll	113	lm	126		
>ref-210775	TCCGTTGCGACTCTCCCTCCTCCCCTC	2	<hkxhk>	hh	52	hk	153	kk	61
>ref-210777	TGGACGCCCACCACACCTCCACGAGCA	4	<hkxhk>	hh	64	hk	207	kk	0
>ref-210777	TGGACGCCCACCACACCTCCACGAGCA	15	<nnxnp>	nn	153	np	116		
>ref-210777	TGGACGCCCACCACACCTCCACGAGCA	27	<hkxhk>	hh	0	hk	273	kk	0
>ref-210780	CTCTCTCACACACTCTCTCCCCCGACC	8	<hkxhk>	hh	1	hk	268	kk	0
>ref-210780	CTCTCTCACACACTCTCTCCCCCGACC	21	<hkxhk>	hh	0	hk	268	kk	1
>ref-210786	GCGTCGGCAACTCGGCCTCCATCATGT	15	<lmxll>	ll	153	lm	118		
>ref-210787	CAGATAAATACACTCACTCCCCGTCCC	23	<lmxll>	ll	157	lm	104		
>ref-210834	CCATCATCAACCCCGCCTCCTCCCCTT	5	<lmxll>	ll	138	lm	105		
>ref-210834	CCATCATCAACCCCGCCTCCTCCCCTT	14	<nnxnp>	nn	255	np	0		
>ref-210845	CTAGGGCCTACTGAGTCTCCTTGCCGG	8	<hkxhk>	hh	5	hk	264	kk	0
>ref-210845	CTAGGGCCTACTGAGTCTCCTTGCCGG	16	<hkxhk>	hh	0	hk	264	kk	5
>ref-210846	CACGCCTCCACCTTGCCTCCATCCTTC	9	<nnxnp>	nn	136	np	134		
>ref-210846	CACGCCTCCACCTTGCCTCCATCCTTC	27	<nnxnp>	nn	130	np	140		
>ref-210853	GAGAGGGGTACAGCTACTCCTACCCAG	6	<nnxnp>	nn	108	np	137		
>ref-210855	GTAACACTCACGGATCCTCCTCCTCCT	1	<lmxll>	ll	115	lm	144		
>ref-210888	CACTGCATGACTCTCACTCCGGCGTCG	5	<nnxnp>	nn	273	np	0		
>ref-210890	ATCAGGAGGACTCGACCTCCGAAATAA	7	<nnxnp>	nn	119	np	134		
>ref-210902	GTCTCACCCACACTTTCTCCGGCGGCC	9	<nnxnp>	nn	140	np	114		
>ref-210908	CCTCCCTTCACCCTCCCTCCACCTCAT	12	<lmxll>	ll	10	lm	260		
>ref-210924	GGCGGGCCAACGTCCCCTCCGCGCGCC	8	<nnxnp>	nn	129	np	142		
>ref-210938	TCAATGACCACAGACTCTCCTTGCTCC	6	<nnxnp>	nn	126	np	127		
>ref-210949	CTCTTTCCTACATTTTCTCCATTCTCT	1	<hkxhk>	hh	15	hk	252	kk	0
>ref-210949	CTCTTTCCTACATTTTCTCCATTCTCT	21	<hkxhk>	hh	15	hk	252	kk	0
>ref-210952	GCAACATTTACAGCTCCTCCCGCTGCG	9	<hkxhk>	hh	2	hk	262	kk	1
>ref-210956	GGGCCTAATACCTATCCTCCGCTGGTT	16	<nnxnp>	nn	127	np	142		
>ref-210978	CGTTTTCCCACCCTCACTCCCCCTTCT	8	<lmxll>	ll	117	lm	129		
>ref-210986	CACCCCTCCACCTTCCCTCCTATCACC	3	<lmxll>	ll	257	lm	0		
>ref-210986	CACCCCTCCACCTTCCCTCCTATCACC	16	<nnxnp>	nn	127	np	107		
>ref-210998	CTTACGTCTACTGACTCTCCACACATA	15	<hkxhk>	hh	48	hk	137	kk	67
>ref-210998	CTTACGTCTACTGACTCTCCACACATA	26	<hkxhk>	hh	67	hk	138	kk	48
>ref-211032	CCGCTACTGACCCATTCTCCATCGGTC	13	<lmxll>	ll	128	lm	137		
>ref-211042	CAGAACTCTACAGGGTCTCCTTATACC	15	<nnxnp>	nn	138	np	120		
>ref-211063	TCCCAGGCCACCTCCCCTCCACCCCCT	7	<nnxnp>	nn	126	np	124		
>ref-211072	GTGAAGACAACACGCGCTCCTACGATG	7	<nnxnp>	nn	131	np	134		
>ref-211072	GTGAAGACAACACGCGCTCCTACGATG	16	<hkxhk>	hh	0	hk	271	kk	0

>ref-211072	GTGAAGACAACACGCGCTCCTACGATG	25	<lmxll>	ll	150	lm	117		
>ref-211073	ATGTACTTAACTGCATCTCCGCCTCAC	22	<nnxnp>	nn	139	np	118		
>ref-211083	GCCAGGTACACTTGCTCTCCGAGTCAG	1	<nnxnp>	nn	108	np	150		
>ref-211112	GACCCCTTTACTTCCCCTCCTCCTCCT	9	<nnxnp>	nn	145	np	119		
>ref-211114	CTCGCTCAGACTGCTCCTCCGTCTGCT	9	<nnxnp>	nn	129	np	141		
>ref-211134	CCAAAAACGACATTCACTCCGTTATCC	24	<hkxhk>	hh	0	hk	265	kk	1
>ref-211196	ATAACAGGAACATTTCCTCCACATCCA	8	<lmxll>	ll	109	lm	153		
>ref-211196	ATAACAGGAACATTTCCTCCACATCCA	24	<lmxll>	ll	135	lm	125		
>ref-211221	CTGAATGTTACTAGCCCTCCCCGTGAA	5	<lmxll>	ll	110	lm	137		
>ref-211225	CTTTTAAGGACTAAAACTCCACATTTA	5	<hkxhk>	hh	0	hk	270	kk	1
>ref-211225	CTTTTAAGGACTAAAACTCCACATTTA	8	<lmxll>	ll	117	lm	123		
>ref-211225	CTTTTAAGGACTAAAACTCCACATTTA	9	<lmxll>	ll	115	lm	118		
>ref-211225	CTTTTAAGGACTAAAACTCCACATTTA	26	<lmxll>	ll	13	lm	246		
>ref-211226	AGAGACGCGACCCGAGCTCCTTCTCGG	2	<lmxll>	ll	143	lm	123		
>ref-211231	TCAAGCCTTACCCTCCCTCCTTACTTC	24	<nnxnp>	nn	175	np	70		
>ref-211246	TCTTCCCCAACACCCCCTCCTCCCTCC	8	<nnxnp>	nn	128	np	123		
>ref-211295	ATCACCCACACGCCCCCTCCCCTCCCC	7	<nnxnp>	nn	202	np	59		
>ref-211295	ATCACCCACACGCCCCCTCCCCTCCCC	22	<nnxnp>	nn	91	np	160		
>ref-211295	ATCACCCACACGCCCCCTCCCCTCCCC	25	<nnxnp>	nn	78	np	177		
>ref-211295	ATCACCCACACGCCCCCTCCCCTCCCC	27	<nnxnp>	nn	256	np	7		
>ref-211296	CCCTTCATCACCCTACCTCCCTCCCCT	1	<nnxnp>	nn	138	np	114		
>ref-211296	CCCTTCATCACCCTACCTCCCTCCCCT	5	<nnxnp>	nn	138	np	114		
>ref-211304	TGAAAGATAACAGATGCTCCGTAGTCA	1	<lmxll>	ll	123	lm	107		
>ref-211320	CGTGGCGAAACCGACACTCCACTATCG	14	<nnxnp>	nn	141	np	116		
>ref-211332	GTAAAACGAACATAAACTCCACGCAAC	7	<lmxll>	ll	124	lm	125		
>ref-211346	CTCTCTTTTACTCCGTCTCCCTCTGTC	3	<hkxhk>	hh	0	hk	273	kk	0
>ref-211346	CTCTCTTTTACTCCGTCTCCCTCTGTC	15	<hkxhk>	hh	0	hk	273	kk	0
>ref-211398	ACTGCCTTTACTTCCCCTCCCAAAGGC	26	<nnxnp>	nn	100	np	144		
>ref-211400	CACACATACACATTCACTCCCCTGTCC	6	<lmxll>	ll	69	lm	195		
>ref-211400	CACACATACACATTCACTCCCCTGTCC	14	<lmxll>	ll	30	lm	238		
>ref-211400	CACACATACACATTCACTCCCCTGTCC	24	<lmxll>	ll	69	lm	195		
>ref-211414	TCCCCCCTCACTATCCCTCCTCCCTCA	13	<hkxhk>	hh	0	hk	262	kk	0
>ref-211414	TCCCCCCTCACTATCCCTCCTCCCTCA	21	<lmxll>	ll	90	lm	148		
>ref-211414	TCCCCCCTCACTATCCCTCCTCCCTCA	27	<hkxhk>	hh	1	hk	262	kk	0
>ref-211415	AATCCACTTACTACCCCTCCATCTCCC	12	<hkxhk>	hh	0	hk	210	kk	18
>ref-211425	TCGCCTCGTACGACGACTCCTCGAAGA	15	<nnxnp>	nn	142	np	122		
>ref-211430	ATCATCATCACCAACACTCCGATCACT	2	<hkxhk>	hh	0	hk	273	kk	0
>ref-211447	ACTATCACTACGCGTTCTCCTTCTAAA	12	<lmxll>	ll	144	lm	123		

>ref-211475	TCTCTACACACATGTTCTCCTCCCTCC	1	<lmxll>	ll	136	lm	119		
>ref-211485	ATCCTTACCACGGTCACTCCTACGTCC	8	<lmxll>	ll	132	lm	136		
>ref-211491	TGCCCGATGACGGTGTCTCCTGTGATG	5	<hkxhk>	hh	0	hk	272	kk	0
>ref-211491	TGCCCGATGACGGTGTCTCCTGTGATG	7	<hkxhk>	hh	0	hk	272	kk	0
>ref-211501	TTCCCCAAGACTGTCCCTCCTACCCAG	6	<lmxll>	ll	136	lm	135		
>ref-211505	ACACCTACGACGCATCCTCCTCCTCCC	7	<nnxnp>	nn	125	np	134		
>ref-211542	ATCTCTCTTACCCACACTCCTCGCAAC	12	<nnxnp>	nn	137	np	127		
>ref-211544	AGTCCCTCCACCCTGTCTCCTCCTCCC	26	<nnxnp>	nn	264	np	0		
>ref-211544	AGTCCCTCCACCCTGTCTCCTCCTCCC	27	<nnxnp>	nn	264	np	0		
>ref-211550	CATTCTCCTACCTTCTCTCCCTTGCCT	5	<lmxll>	ll	135	lm	128		
>ref-211553	TGCCATACGACTTACTCTCCCGCTGAG	25	<nnxnp>	nn	126	np	138		
>ref-211555	CAGCCTCTCACTCTATCTCCACCCTTC	3	<hkxhk>	hh	1	hk	271	kk	0
>ref-211584	TTCTTCTCTACTCTTTCTCCTTTCATT	22	<hkxhk>	hh	0	hk	272	kk	1
>ref-211584	TTCTTCTCTACTCTTTCTCCTTTCATT	23	<hkxhk>	hh	0	hk	214	kk	57
>ref-211584	TTCTTCTCTACTCTTTCTCCTTTCATT	24	<hkxhk>	hh	2	hk	269	kk	0
>ref-211589	CCCTAGGTGACTTCCGCTCCTCTCCAG	3	<lmxll>	ll	169	lm	96		
>ref-211589	CCCTAGGTGACTTCCGCTCCTCTCCAG	6	<lmxll>	ll	168	lm	91		
>ref-211589	CCCTAGGTGACTTCCGCTCCTCTCCAG	7	<hkxhk>	hh	0	hk	270	kk	3
[truncated: 819,975 more chars]
